# Refined Multiscale Fuzzy Entropy to Analyse Post-Exercise Cardiovascular Response in Older Adults With Orthostatic Intolerance

**DOI:** 10.3390/e20110860

**Published:** 2018-11-08

**Authors:** Marcos Hortelano, Richard B. Reilly, Francisco Castells, Raquel Cervigón

**Affiliations:** 1 Escuela Politécnica, UCLM Camino del Pozuelo sn, 16071 Cuenca, Spain; 2 School of Engineering, Trinity College, The University of Dublin, Dublin 2 D02 PN40, Ireland; 3 School of Medicine, Trinity College, The University of Dublin, Dublin 2 D02 PN40, Ireland; 4 Trinity Centre for Bioengineering, Trinity College, The University of Dublin, Dublin 2 D02 PN40, Ireland; 5 Instituto ITACA, Universitat Politècnica de València, 46022 Valencia, Spain

**Keywords:** refined composite multiscale fuzzy entropy, hear rate, blood pressure, aging, exercise, orthostatic intolerance

## Abstract

Orthostatic intolerance syndrome occurs when the autonomic nervous system is incapacitated and fails to respond to the demands associated with the upright position. Assessing this syndrome among the elderly population is important in order to prevent falls. However, this problem is still challenging. The goal of this work was to determine the relationship between orthostatic intolerance (OI) and the cardiovascular response to exercise from the analysis of heart rate and blood pressure. More specifically, the behavior of these cardiovascular variables was evaluated in terms of refined composite multiscale fuzzy entropy (RCMFE), measured at different scales. The dataset was composed by 65 older subjects, 44.6% (*n* = 29) were OI symptomatic and 55.4% (*n* = 36) were not. Insignificant differences were found in age and gender between symptomatic and asymptomatic OI participants. When heart rate was evaluated, higher differences between groups were observed during the recovery period immediately after exercise. With respect to the blood pressure and other hemodynamic parameters, most significant results were obtained in the post-exercise stage. In any case, the symptomatic OI group exhibited higher irregularity in the measured parameters, as higher RCMFE levels in all time scales were obtained. This information could be very helpful for a better understanding of cardiovascular instability, as well as to recognize risk factors for falls and impairment of functional status.

## 1. Introduction

Orthostatic intolerance (OI) is characterized by the development of symptoms such as lightheadedness, sweatiness, nausea, cold, clamminess, dizziness, fatigue, lethargy, palpitations, and blurred vision prior to the fall during standing. Previous studies suggest that OI may be associated with significant morbidity and mortality [1,2,3]. With the progressive increase of life expectancy, the prevalence of this pathology is likely to increase accordingly and, consequently, the risk of falls as well. Thus, improved OI assessment is a challenge of clinical importance.

This syndrome is a consequence of a complex multifactorial pathophysiology that involves cardiovascular instability with an unusual autonomic nervous system response or alterations in cerebral blood flow [4,5,6]. Among several causes, it is most commonly caused by orthostatic hypotension (OH) and these orthostatic symptoms are caused by cerebral hypofunction [7]. OH is defined as a decrease in systolic blood pressure (SBP) of greater than 20 mmHg or decrease in dyastolic blood pressure (DBP) of greater than 10 mmHg upon standing or head-up tilt, designed to detect OH. This is a non-invasive diagnostic test that could provoke fainting or syncope and involves placing a patient on a table with a foot-support, then tilting the table upward while their blood pressure and pulse are being monitored.

Previous works have already proposed linear methods to associate cardiovascular responses and orthostatic stress [8,9]. In this study, we propose to apply non-linear methods to analyze blood pressure (BP) and heart rate (HR) response. HR presents a high degree of variability [10] as it is modulated by multiple physiologic mechanisms that operate over a wide range of time scales [11]. There are several main types of measures to compute the complexity of a system or signal, such as diverse entropy definitions, fractal dimensions or fuzzy logic techniques, among others. Nevertheless, classical entropy and physiologic complexity concepts do not have a straightforward correspondence, as other aspects such as signal pre-processing [12] or multiple time scales inherent in physiologic systems [13,14,15,16] may have a great influence on the measurements. To deal with these problems, refined composite multiscale fuzzy entropy (RCMFE) analysis has been developed to quantify the complexity of a time series over multiple time scales, and has arised as a promising method for biomedical signal analysis [17]. Indeed, this is a meaningful approach for the assessment of biological systems since these are also multiscale in essence. This approach focuses on identifying physiological dynamic over multiple temporal scales to quantify the information of a disease, aging or the impact of the exercise on individual time scales that may evaluate their behavior. Moreover, this method is also characterised by the accuracy of entropy estimation, independence of the data length, and computational efficiency [18].

The aim of this study is to provide a more accurate measure of cardiovascular parameters to aid the assessment of OI and its association with falls in a cohort ofelderly people. For this, RCMFE is computed from the signals recorded during a 6 min walk distance (6MWD) test.

## 2. Materials

The dataset included a total of 65 participants, aged over 70 years old (70.11 ± 5.85, 65% females), 29 (44.6%) being symptomatic of OI and 36 (55.4%) asymptomatic. There were no significant differences in either age, weight, height, body mass index or gender between symptomatic and asymptomatic participants. (Table 1).

All participants were evaluated in the Technology Research for Independent Living Clinic at St James hospital in Dublin [19], according to the protocol approved by the Local Research Ethics Committee, including informed consent to participate in the study. Each participant got a minimum score of 23 in a mini-mental state examination, which is the threshold in insanity detection in the Irish framework [20]. None of the participants suffered from Parkinson, diabetes mellitus, acute chronic renal failure, deficiency in B12 vitamin or a pacemaker. Likewise, the participants were not asked to stop taking their medicines before the examination. The measurements were carried out using the Finapres method (Finger Arterial Pressure).

## 3. Methods

The participants carried out the Active Stand Protocol, following an orthostatic test: going from supine position to bipedalism. Data was collected using the FINOMETER PRO Hemodynamic beat to beat (FINAPRES, NY, USA) a non-invasive beat to beat BP monitor. This device uses an oscillometric pressure cuff in the upper part of the arm to get an individual calibration of the reconstruction of the pressure signal of the fingers at a branchial level. The hydrostatic head correction system was used during the whole study to balance out the movements of the hand with respect to the heart level. Finometer PRO that incorporates the patented Modelflow technology providing a reliable non-invasive method for the assessment of hemodynamic parameters such as cardiac output or pulse rate (variability). The Modelflow cardiac output has been validated against thermodilution cardiac output in patients undergoing coronary artery bypass surgery [21].

**Figure 1:**
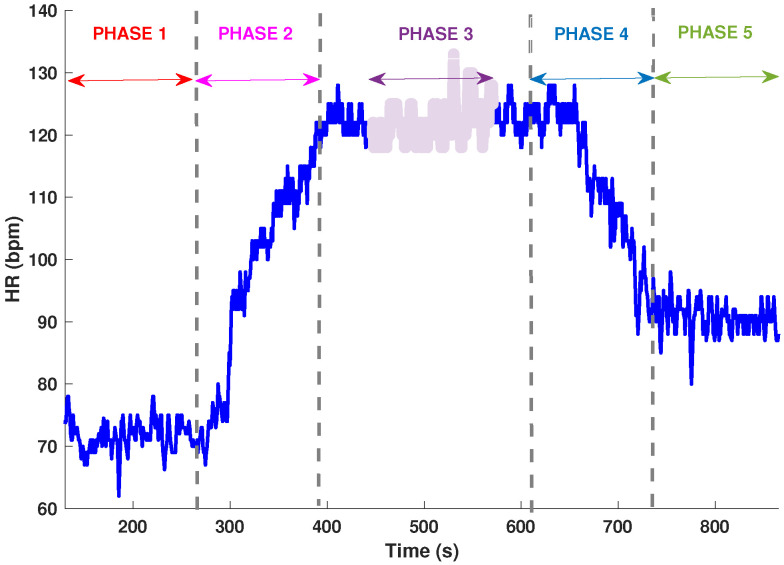
Heart rate (HR) measure belong to an asymptomatic orthostatic intolerance (OI) participant during 6 min walk distance (6MWD) test. The five phases analyzed are showed, all of the with the same data length, it is highlighted during the *Phase 3*.

Before standing up, the participants rested in supine position for at least 10 min. Their BP was then monitored for 3 min while they were standing. The 6MWD test consists of measuring cardiovascular response of the subjects for six min while they walk along a flat, straight corridor at their own pace [22]. The 6MWD test is divided into clearly five identified and differentiated phases: a first phase of rest with a duration of 3 min, *Phase 1 (pre-exercise)*. The walking period includes two phases: *Phase 2* and *Phase 3* with a duration of 6 min. During the *Phase 2* participants begin the walk *(starting of exercise)* and during the *Phase 3* the participants are doing exercise *(active phase)*. The rest period includes two phases: *Phase 4* and *Phase 5* with a duration of 3 min. The *Phase 4* is the time interval after the exercise *(recovery phase)* and the *Phase 5* is the post-exercise at rest *(post-exercise)*. Consequently, a total of five phases are available for our study. The number of intervals extracted in each of the five phases once the signal was stabilised was always the same (C=180 samples), in order to avoid biased results due to segment length differences (Figure 1). Moreover, the evolution exhibited in phases 2 and 4 can be modelled by the change in the mean over time. Once this has been modelled, detrending is applied by removing the main trend from the time series.

The following beat by beat measurements were recorded and analyzed:

- SBP (mmHg): the maximum blood pressure (systole).
- DBP (mmHg): as the minimum value of pressure (diastole).
- MBP (mmHg): the mean arterial blood pressure during the cardiac cycle.
- HR (bpm): heart rate or cardiac frequency, derived from the pulse interval.
- CO (lpm): blood volume expelled by each ventricle per minute.
- TPR (mmHg/lpm): Total peripheral resistance, the overall resistance to blood flow through the systemic blood vessels.

### 3.1. Fuzzy Entropy Method

This method to measure entropy was proposed to overcome up to a certain extent the limitations of sample entropy (SampEn) in terms of consistency and dependency to data length. Although fuzzy entropy (FuzEn) requires a higher computational capacity, due to its improved performance and robustness it is being considered as an appropriate method in several applications.

For a *N*-length time series *y*, embedding dimension *m*, power *n* and tolerance *r*, the FuzEn is computed as [17,23]:

(1) $$FuzEn\left(y,m,n,r\right)=-ln\left(\frac{\phi^{m+1}\left(y,n,r\right)}{\phi^{m}\left(y,n,r\right)}\right),$$

where $\phi^{m}\left(y,n,r\right)$ is the fuzzy function, defined as:

(2) $$\phi^{m}\left(y,n,r\right)=\frac{1}{N-m}\sum_{t_{1}}^{N-m}\frac{1}{N-m-1}\sum_{t_{2}=1,t_{1}\neq t_{2}}^{N-m}exp\left(\frac{-{\left(d_{t_{1}t_{2}}\right)}^{n}}{r}\right)$$

and $d_{t_{1}t_{2}}$ is the distance between two zero-mean embedded sequences.

### 3.2. Coarse-Graining Process for a Multiscale Approach

Multiscale entropy (MSE) was defined in order to account for the complexity of signals with long-term correlations structures, and has been successfully employed to quantify the complexity of biomedical time series. Recent developments have tried to alleviate the problem of undefined MSE values for short signals [17]. The concept of fuzzy entropy was proposed in 2009 [24] and it corresponds to a more accurate entropy definition than sample entropy (SE). Due to this fact, it owns stronger relative consistence and less dependence on data length [15]. A fuzzy entropy (FuzEn) algorithm has been proposed to overcome the short time series problem, which was described in previous studies [23,25,26]. In addition, this method has also been applied to analyze non-stationary signals [25].

To compute the MSE, a coarse-graining process is applied by averaging the elements periodically spaced by τ samples [27]. Later on, Costa introduced the generalized multiscale entropy by using the variance in the coarse-graining process [28].

After this process, either the multiscale entropy (MSE_*μ*_/MSE_*σ*_) or the multiscale fuzzy entropy (MFE_*μ*_/MFE_*σ*_) can be obtained by computing the SampEn or the FuzEn from the coarse-grain series, respectively. In this notation, the sub-index μ and σ stands for the way the coarse-grain is obtained (by averaging or using the variance, respectively).

Despite the advantages of multiscale entropies, the application of the coarse-graining procedure leads to the following drawbacks: firstly, their dependency on the asymmetry of the samples within the time series and, secondly, their increased variability at high scale factors, due to a reduced number of elements in the coarse-grain series.

### 3.3. Refined Composite Multiscale Fuzzy Entropy Method

The refined composite algorithm was proposed to overcome the limitations of multiscale entropies [29]. Basically, this algorithm outputs τ time series after the coarse-graining process τ time series instead of only one, by considering all possible data shifts. Next, the corresponding probability or fuzzy functions according to definition of SampEn or FuzEn, respectively, are computed for each time series and averaged. Finally, the natural logarithm is applied to obtain RCMSE or RCMFE [17].

Taking into account the properties of our datasets, the data length and the superiority of FuzEn over SE, and RCMSE over MSE, we employed RCMFE in this study, with m=2,n=2, r=0.15 and scales from 1 upto 5. The configuration of the RCMFE with these parameters was based on and supported by the results derived from previous works [17,23,30,31].

### 3.4. Statistical Analysis

The normal distribution of the variables was firstly validated by the Shapiro-Wilk test. If so, a Student’s unpaired t -test will be carried out to check the difference between parameters derived from different groups. Otherwise, a non-parametric test will be employed instead. Moreover, the general linear model repeated measures ANOVA including phase as a within-subjects factor and group and gender as between-subjects factor has been applied. With this statistical analysis, we were able to test whether RCMFE values of measured parameters were different across both groups. After Bonferroni correction, in order to address the problem of multiple comparisons, a *p*-value < 0.05 (two-tailed) was considered statistically significant. All variables will be presented as mean and standard deviation.

## 4. Results

### 4.1. Parameters Analysis

**Figure 2:**
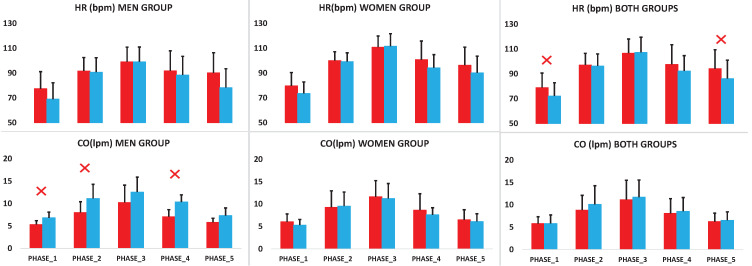
Mean and standard deviation for cardiac output (CO) and HR in the male (**left**), female (**middle**) and both gender (**right**) symptomatic OI participants (red) and asymptomatic OI participants (blue) (X symbol *p* < 0.05).

To assess the effect of exercise in OI symptomatic participants, we used the 6WMD test. In Figure 1 we indicated the five phases of the 6WMD test that were used in this study. We tested the different parameters measures along phases in both groups, moreover only the parameters represented in Figure 2, HR and CO, showed any statistical significant differences between both groups. In HR we could find differences during *Phase 1* (*p* = 0.019) with 72.09 ± 10.72 vs. 78.87 ± 11.77 bpm in asymptomatic and symptomatic OI participants, respectively, and in *Phase 5* with 86.10 ± 14.92 vs. 94.11 ± 15.25 bpm (*p* = 0.022) in asymptomatic and symptomatic OI participants, respectively. Nevertheless, no evidence was found for gender differences. Moreover, only male participants showed differences in CO during *Phase 1*, *Phase 2* and *Phase 4*, with lower values in the pathology participants group. For instance these values during *Phase 1* were from 6.82 ± 2.53 lpm in the asymptomatic OI participants to 5.33 ± 0.85 lpm in the symptomatic OI participants (*p* = 0.034). Furthermore, special emphasis should be given to the *(post-exercise)* stage, *Phase 5*, where only HR showed statistical significant differences between both groups, with an increase of this parameter in the symptomatic OI patients compared with the asymptomatic OI participants (Table 2).

### 4.2. Refined Composite Multiscale Fuzzy Entropy Results

**Figure 3:**
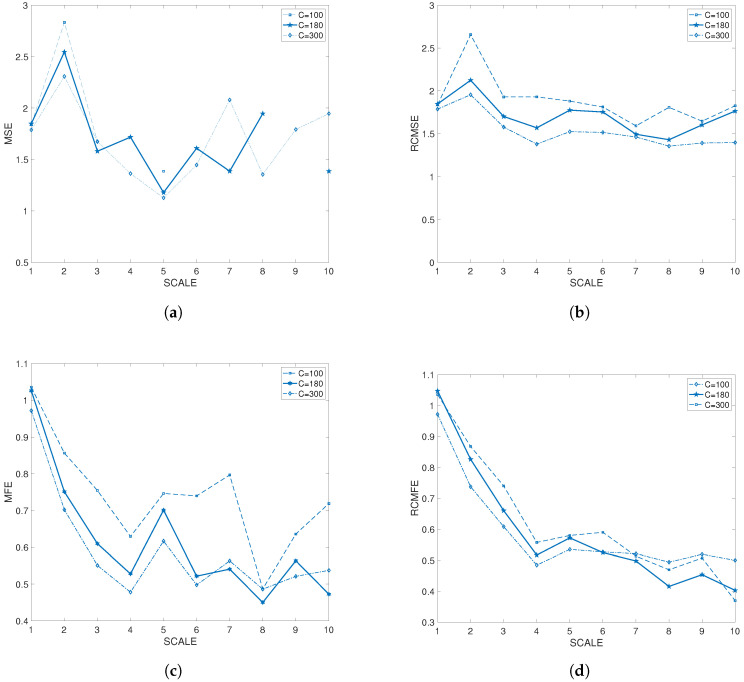
Multiscale entropy (MSE), refined composite multiscale entropy (RCMSE), multiscale fuzzy entropy (MFE) and refined composite multiscale fuzzy entropy (RCMFE) at different time scales as a function of data length 100, 180 and 300 computed.(**a**) MSE in time scales 1-10; (**b**) RCMSE in time scales 1-10; (**c**) MFE in time scales 1-10; (**d**) RCMFE in time scales 1-10.

RCMFE was applied to evaluate regularity along different phases in both groups. The improvement and the applicability to short time series was demonstrated in comparison to other measures in previous studies [17], where in terms of reliability for short signals of length *C* equal to 100 only RCMFE lead reliable results. In addition, to check the suitability of this measure in our signal, MSE, RCMSE, MFE and RCMFE values for HR signal length of 100, 180 and 300 were compared (Figure 3). The results show that values for length of 100 are still undefined at some scale factors for some estimators, for instance, the crosscorrelation coefficient for MSE between length of 180 and 300 was lower than 0.5, however for RCMSE was 0.8652 and for RCMFE the correlation was larger (0.9926), showing the stronger the relationship between the variables. These results show the suitability of small C value for RCMFE estimation.

**Figure 4:**
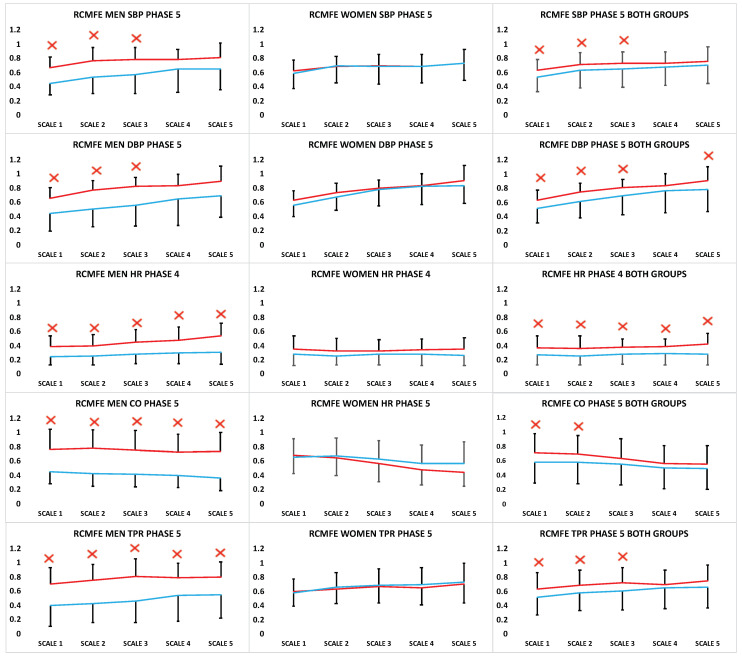
RCMFE analysis from systolic blood pressure (SBP), dyastolic blood pressure (DBP), heart rate (HR) and total peripheral resistance (TPR) in the (**left**), females (**middle**) and both gender (**right**) symptomatic OI (red) and asymptomatic OI participants (blue) along the time scales 1, 2, 3, 4 and 5 (X symbol = *p* < 0.05).

Moreover, in order to analyze the data different time scales were chosen to verify if different trends were observed. Accordingly, RCMFE was applied to all the parameters along the different phases and a reduction of RCMFE in the asymptomatic OI compared with the symptomatic OI participants in the post-exercise phases was observed (Figure 4).

Although several differences were observed along all the phases, the higher statistical significant differences between both groups were found in the *(post-exercise)* stage, where regularity of SBP, DBP, CO and TPR showed a clear increase in irregularity in the OI participants during the rest stage (*Phase 5*), with higher differences in the male participants. (Figure 4). The same trend in these parameters was observed in the female group, although these differences did not reach statistical significance on any temporal scale. Moreover, HR RCMFE parameter showed a significant reduction along all the chosen scales in asymptomatic OI group in *Phase 4* (Figure 4). Specifically, the male participants with symptomatic OI showed higher irregularity at all scales compared with the asymptomatic OI group. In addition, in the female group the same trend was observed, nevertheless, differences did not reach the statistical signification Figure 4).

## 5. Discussion

In this study, we analyzed cardiopostural interactions in order to diagnose orthostatic intolerance in older adults. In order to study this syndrome, the 6MWD test was applied. This test has been proven to be a good reflection of the activities in daily life, and is considered a protocol of simple effort and easy to do.

Firstly, we analyzed the parameters extracted from the recordings in both groups. Only HR and CO measurements showed statistical significant differences between symptomatic and asymptomatic OI groups. Nevertheless, the rest of the measures did not show statistical significant differences between both groups. Results showed an increase of HR in the symptomatic OI participants, with statistical significant decreased of CO in the symptomatic OI male participants. These findings are consistent with previous research and syncope guidelines, where OH results from the normal delay of arterial baroreflex detection and response to gravitational blood volume redistribution. Thereafter, venous return decreases central blood volume and CO by 20% despite baroreflex mediates vasoconstriction and increases cardiac contractility and HR [32]. Conversely, these significant differences were not observed in the female group.

In addition, we have proposed a non linear approach, as a refined technique with respect to previous studies. As previously mentioned, entropy had already been used by some researchers to assess the complexity of cardiovascular time series. For instance, [33] showed that different entropy indices can be helpful to monitor sympathovagal balance, evidencing that increasing of the tilt table inclination suppose a decrease of complexity. Moreover, previous works have used a multiscale approach to study these signals. For instance, Turianikova et al. showed MSE of heart rate and blood pressure signals were able to follow the changes in autonomic balance caused by postural change from the supine to the standing position [34]. In addition, a previous work highlights that response to mental stress shows a reduction in time asymmetry that reflects a shift in sympathovagal balance [35]. This result is consistent with a previous study that time irreversibility in hemodynamic parameters during the 6MWD that showed a similar trend during exercise stress [36].

In this paper, RCMFE is applied, which was recently proposed as a multiscale approach to short-term cardiovascular variability at different temporal scales [17,18]. Indeed, one of the most important restrictions of MSE is the length of the time series [15,37,38], which has been also overcome with other recent approaches [39]. Results showed a higher HR irregularity in OI participants at all the temporal scales in both groups during the transfer from walking to standing. However, only the male group showed statistical significant differences on all the scales. These results are also consistent with previous studies where cardiac Autonomous Nervous System (ANS) is impaired in frail elderly people, as indicated by a reduction in the complexity of HR dynamics, reduced HR variability, reduced HR changes in response to daily activities and is likely to be a consequence of a reduced vagal activity. Nevertheless, in this case the reduction is lower in symptomatic OI patients if we compared them with asymptomatic OI participants [40,41].

The hemodynamic and cardiovascular parameters of SBP, DBP, CO and TPR also showed very interesting results. These findings were focused on *Phase 5*, during the rest after the exercise stage, which showed a greater irregularity at all temporal scales in the symptomatic OI male participants (Figure 4). Regarding these results, some studies showed that there was an increase of TPR in patients with OH, suggesting that alpha-adrenergic sympathetic hyperactivity is the predominant pathophysiologic mechanism of OH. Moreover, a previous report suggested that a possible mechanism of OH may be an excessive peripheral vasoconstriction due to a sympathetic hyperactivity as an over compensation from an excessive pooling in the leg during the upright position [42].

In addition, a more pronounced irregularity decrease observed in the female group compared to the male group can be due by the fact that women have a more active parasympathetic system. This behavior could be regarded as if women compensated less effectively the drop of BP in response to positional change. These reports suggested that gender specific differences were found in either nerve activity or blood flow response, which may be responsible for females reduced orthostatic tolerance [43]. Owing to this fact, these studies concluded that lower orthostatic tolerance in women is associated with decreased cardiac filling rather than reduced responsiveness of vascular resistance during orthostatic challenges. In summary, women had significantly lower capacity to regulate blood pressure and maintain orthostatic function compared with men [9,44,45,46]. Nevertheless, the responsible mechanisms have not been identified, but the results of the present study indicate gender differences in autonomic mechanisms that affect in orthostatic tolerance.

These results could be helpful in focusing therapies and interventions to analyze cardiopostural and pot-exercise responses with a very easy procedure for patients. Attention should also be drawn to entropy in different time scales to detect changes in HR, SBP, DBP, TPR and CO after the orthostatic test in older subjects. Entropy-based measures might provide useful indicators of pathological changes in cardiac activity after exercise. These results could be very useful and easily put into practice, due to the simplicity of the register. Based on this knowledge, the current study investigated the feasibility of shortening the time for data acquisition in differentiating among the symptomatic and asymptomatic OI aged populations using a novel method of computation. To conclude, this study highlights the important information cardiac parameters regularity changes provide after exercise in symptomatic OI participants, emphasizing the importance of this analysis to asses this syndrome.

## Figures and Tables

**Table 1:** Participants characteristics.

		Women	Men	Total
Symptomatic	Participants	23	13	36
	Age	69.65 ± 5.29	71.77 ± 6.47	70.42 ± 5.74
	Weight	72.39 ± 14.32	84.23 ± 12.81	76.67 ± 14.78
	Height	163.96 ± 7.57	173.85 ± 5.88	167.53 ± 8.43
	Body mass index	26.77 ± 5.36	26.09 ± 4.01	26.86 ± 5.44
Asymptomatic	Participants	19	10	29
	Age	70.63 ± 5.34	68.00 ± 7.21	69.72 ± 6.05
	Weight	70.89 ± 10.57	81.60 ± 10.78	74.59 ± 11.66
	Height	163.84 ± 7.42	176.00 ± 2.94	168.03 ± 8.53
	Body mass index	26.09 ± 4.01	25.72 ± 3.97	25.96 ± 3.93

**Table 2:** The mean and standard deviation for systolic blood pressure (SBP), dyastolic blood pressure (DBP), heart rate (HR), cardiac output (CO) and total peripheral resistance (TPR) parameters in the *Phase 5*, in male, female and both gender groups were represented.

Phase 5	Men			Women			Both Gender		
	Symptomatic OI	Asymptomatic OI	*p*	Symptomatic OI	Asymptomatic OI	*p*	Symptomatic OI	Asymptomatic OI	*p*
SBP (mmHg)	134.97 ± 21.80	140.47 ± 20.43	>0.05	127.37 ± 25.72	130.54 ± 18.67	>0.05	130.11 ± 24.34	133.96 ± 19.53	>0.05
DBP (mmHg)	70.18 ± 13.83	66.33 ± 11.37	>0.05	62.78 ± 10.56	62.70 ± 8.41	>0.05	65.45 ± 12.19	63.95 ± 9.50	>0.05
HR (bpm)	90.17 ± 16.18	78.32 ± 15.19	>0.05	96.34 ± 14.59	90.20 ± 13.40	>0.05	94.11 ± 15.25	86.10 ± 14.92	0.022
CO (lpm)	5.80 ± 0.91	7.31 ± 2.03	>0.05	6.47 ± 2.24	6.12 ± 1.71	>0.05	6.23 ± 1.88	6.53 ± 1.88	>0.05
TPR (mmHg/lpm)	17.67 ± 6.05	15.94 ± 5.71	>0.05	16.35 ± 5.44	16.86 ± 3.86	>0.05	16.22 ± 5.62	16.54 ± 4.50	>0.05

## Supplementary Materials

Supplementary File 1

## Abbreviations

The following abbreviations are used in this manuscript:

BP	Blood Pressure
DBP	Dyastolic Blood Pressure
CO	Cardiac Output
FE	Fuzzy Entropy
HR	Heart Rate
MBP	Mean Arterial Blood Pressure
MFE	Multiscale Fuzzy Entropy
MSE	Multiscale entropy
OI	Orthostatic Intolerance
OH	Orthostatic Hypotension
RCMSE	Refined composite multiscale entropy
RCMFE	Refined composite multiscale fuzzy entropy
SBP	Systolic Blood Pressure
SE	Sample Entropy
TPR	Total Peripheral Resistance
