# Supplementary material for: Refined Multiscale Fuzzy Entropy to Analyse Post-Exercise Cardiovascular Response in Older Adults With Orthostatic Intolerance
Source: Entropy (Basel). 2018 Nov 8;20(11):860. doi: 10.3390/e20110860 (PMC7512426; doi:10.3390/e20110860)
Supplement: Supplementary File 1 [file entropy-20-00860-s001.zip › entropy-353357-supplementary.pdf]

Explore SBP
GENDER

Case Processing Summary

	GENDER	Cases					
		Valid		Missing		Total	
		N	Percent	N	Percent	N	Percent
RCMFE_PHASE1_SCALE_1_SBP	WOMEN	42	100,0%	0	0,0%	42	100,0%
	MEN	23	100,0%	0	0,0%	23	100,0%
RCMFE_PHASE1_SCALE_2_SBP	WOMEN	42	100,0%	0	0,0%	42	100,0%
	MEN	23	100,0%	0	0,0%	23	100,0%
RCMFE_PHASE1_SCALE_3_SBP	WOMEN	42	100,0%	0	0,0%	42	100,0%
	MEN	23	100,0%	0	0,0%	23	100,0%
RCMFE_PHASE1_SCALE_4_SBP	WOMEN	42	100,0%	0	0,0%	42	100,0%
	MEN	23	100,0%	0	0,0%	23	100,0%
RCMFE_PHASE1_SCALE_5_SBP	WOMEN	42	100,0%	0	0,0%	42	100,0%
	MEN	23	100,0%	0	0,0%	23	100,0%
RCMFE_PHASE2_SCALE_1_SBP	WOMEN	42	100,0%	0	0,0%	42	100,0%
	MEN	23	100,0%	0	0,0%	23	100,0%
RCMFE_PHASE2_SCALE_2_SBP	WOMEN	42	100,0%	0	0,0%	42	100,0%
	MEN	23	100,0%	0	0,0%	23	100,0%
RCMFE_PHASE2_SCALE_3_SBP	WOMEN	42	100,0%	0	0,0%	42	100,0%
	MEN	23	100,0%	0	0,0%	23	100,0%
RCMFE_PHASE2_SCALE_4_SBP	WOMEN	42	100,0%	0	0,0%	42	100,0%
	MEN	23	100,0%	0	0,0%	23	100,0%
RCMFE_PHASE2_SCALE_5_SBP	WOMEN	42	100,0%	0	0,0%	42	100,0%
	MEN	23	100,0%	0	0,0%	23	100,0%
RCMFE_PHASE3_SCALE_1_SBP	WOMEN	42	100,0%	0	0,0%	42	100,0%
	MEN	23	100,0%	0	0,0%	23	100,0%
RCMFE_PHASE3_SCALE_2_SBP	WOMEN	42	100,0%	0	0,0%	42	100,0%
	MEN	23	100,0%	0	0,0%	23	100,0%
RCMFE_PHASE3_SCALE_3_SBP	WOMEN	42	100,0%	0	0,0%	42	100,0%
	MEN	23	100,0%	0	0,0%	23	100,0%
RCMFE_PHASE3_SCALE_4_SBP	WOMEN	42	100,0%	0	0,0%	42	100,0%
	MEN	23	100,0%	0	0,0%	23	100,0%
RCMFE_PHASE3_SCALE_5_SBP	WOMEN	42	100,0%	0	0,0%	42	100,0%
	MEN	23	100,0%	0	0,0%	23	100,0%
RCMFE_PHASE4_SCALE_1_SBP	WOMEN	42	100,0%	0	0,0%	42	100,0%
	MEN	23	100,0%	0	0,0%	23	100,0%
RCMFE_PHASE4_SCALE_2_SBP	WOMEN	42	100,0%	0	0,0%	42	100,0%
	MEN	23	100,0%	0	0,0%	23	100,0%
RCMFE_PHASE4_SCALE_3	WOMEN	42	100,0%	0	0,0%	42	100,0%
	MEN	23	100,0%	0	0,0%	23	100,0%
RCMFE_PHASE4_SCALE_4_SBP	WOMEN	42	100,0%	0	0,0%	42	100,0%
	MEN	23	100,0%	0	0,0%	23	100,0%
RCMFE_PHASE4_SCALE_5_SBP	WOMEN	42	100,0%	0	0,0%	42	100,0%
	MEN	23	100,0%	0	0,0%	23	100,0%
RCMFE_PHASE5_SCALE_1_SBP	WOMEN	42	100,0%	0	0,0%	42	100,0%

Case Processing Summary

	GENDER	Cases					
		Valid		Missing		Total	
		N	Percent	N	Percent	N	Percent
RCMFE_PHASE5_SCALE_1_ SBP	MEN	23	100,0%	0	0,0%	23	100,0%
RCMFE_PHASE5_SCALE_2_	WOMEN	42	100,0%	0	0,0%	42	100,0%
SBP	MEN	23	100,0%	0	0,0%	23	100,0%
RCMFE_PHASE5_SCALE_3_	WOMEN	42	100,0%	0	0,0%	42	100,0%
SBP	MEN	23	100,0%	0	0,0%	23	100,0%
RCMFE_PHASE5_SCALE_4_	WOMEN	42	100,0%	0	0,0%	42	100,0%
SBP	MEN	23	100,0%	0	0,0%	23	100,0%
RCMFE_PHASE5_SCALE_5_	WOMEN	42	100,0%	0	0,0%	42	100,0%
SBP	MEN	23	100,0%	0	0,0%	23	100,0%

Tests of Normality

	GENDER	Kolmogorov-Smirnov[a]			Shapiro-Wilk		
		Statistic	df	Sig.	Statistic	df	Sig.
RCMFE_PHASE1_SCALE_1_	WOMEN	,086	42	,200*	,978	42	,602
SBP	MEN	,115	23	,200*	,971	23	,718
RCMFE_PHASE1_SCALE_2_	WOMEN	,085	42	,200*	,972	42	,372
SBP	MEN	,122	23	,200*	,964	23	,540
RCMFE_PHASE1_SCALE_3_	WOMEN	,112	42	,200*	,960	42	,148
SBP	MEN	,081	23	,200*	,973	23	,767
RCMFE_PHASE1_SCALE_4_	WOMEN	,127	42	,088	,948	42	,056
SBP	MEN	,120	23	,200*	,954	23	,357
RCMFE_PHASE1_SCALE_5_	WOMEN	,084	42	,200*	,988	42	,942
SBP	MEN	,094	23	,200*	,974	23	,790
RCMFE_PHASE2_SCALE_1_	WOMEN	,059	42	,200*	,982	42	,725
SBP	MEN	,123	23	,200*	,984	23	,957
RCMFE_PHASE2_SCALE_2_	WOMEN	,085	42	,200*	,988	42	,924
SBP	MEN	,129	23	,200*	,974	23	,775
RCMFE_PHASE2_SCALE_3_	WOMEN	,092	42	,200*	,988	42	,926
SBP	MEN	,190	23	,031	,929	23	,103
RCMFE_PHASE2_SCALE_4_	WOMEN	,080	42	,200*	,977	42	,533
SBP	MEN	,234	23	,022	,876	23	,068
RCMFE_PHASE2_SCALE_5_	WOMEN	,097	42	,200*	,972	42	,396
SBP	MEN	,186	23	,038	,901	23	,056
RCMFE_PHASE3_SCALE_1_	WOMEN	,095	42	,200*	,967	42	,258
SBP	MEN	,135	23	,200*	,935	23	,143
RCMFE_PHASE3_SCALE_2_	WOMEN	,132	42	,062	,966	42	,243
SBP	MEN	,099	23	,200*	,988	23	,993
RCMFE_PHASE3_SCALE_3_	WOMEN	,079	42	,200*	,981	42	,683
SBP	MEN	,126	23	,200*	,963	23	,527
RCMFE_PHASE3_SCALE_4_	WOMEN	,087	42	,200*	,973	42	,403
SBP	MEN	,133	23	,200*	,952	23	,317
RCMFE_PHASE3_SCALE_5_	WOMEN	,062	42	,200*	,986	42	,871

SBP	MEN	,081	23	,200*	,967	23	,628
RCMFE_PHASE4_SCALE_1_	WOMEN	,082	42	,200*	,964	42	,205
SBP	MEN	,199	23	,018	,929	23	,102
RCMFE_PHASE4_SCALE_2_	WOMEN	,113	42	,200*	,941	42	,051
SBP	MEN	,156	23	,151	,949	23	,277
RCMFE_PHASE4_SCALE_3	WOMEN	,109	42	,200*	,937	42	,053
	MEN	,160	23	,131	,944	23	,214
RCMFE_PHASE4_SCALE_4_	WOMEN	,132	42	,063	,957	42	,114
SBP	MEN	,171	23	,079	,930	23	,107
RCMFE_PHASE4_SCALE_5_	WOMEN	,109	42	,200*	,952	42	,076
SBP	MEN	,133	23	,200*	,928	23	,101
RCMFE_PHASE5_SCALE_1_	WOMEN	,065	42	,200*	,977	42	,548
SBP	MEN	,151	23	,185	,967	23	,627

Tests of Normality

	GENDER	Kolmogorov-Smirnov[a]			Shapiro-Wilk		
		Statistic	df	Sig.	Statistic	df	Sig.
RCMFE_PHASE5_SCALE_2_	WOMEN	,081	42	,200*	,976	42	,522
SBP	MEN	,120	23	,200*	,969	23	,672
RCMFE_PHASE5_SCALE_3_	WOMEN	,087	42	,200*	,965	42	,223
SBP	MEN	,103	23	,200*	,959	23	,445
RCMFE_PHASE5_SCALE_4_	WOMEN	,065	42	,200*	,975	42	,466
SBP	MEN	,123	23	,200*	,949	23	,273
RCMFE_PHASE5_SCALE_5_	WOMEN	,095	42	,200	,970	42	,323
SBP	MEN	,088	23	,200*	,977	23	,848

*. This is a lower bound of the true significance.

a. Lilliefors Significance Correction

RCMFE_PHASE1_SCALE_1_SBP

Normal Q-Q Plots

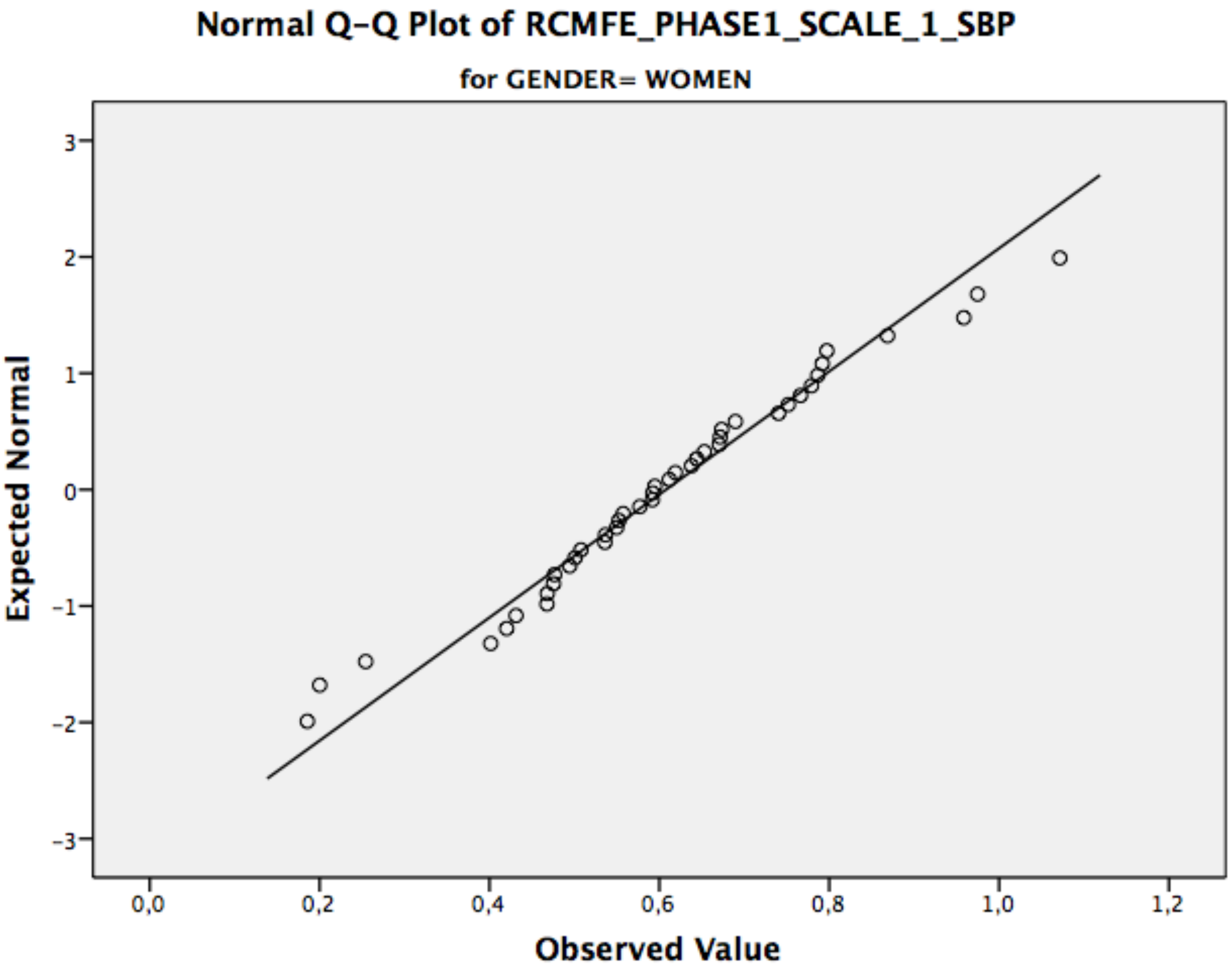

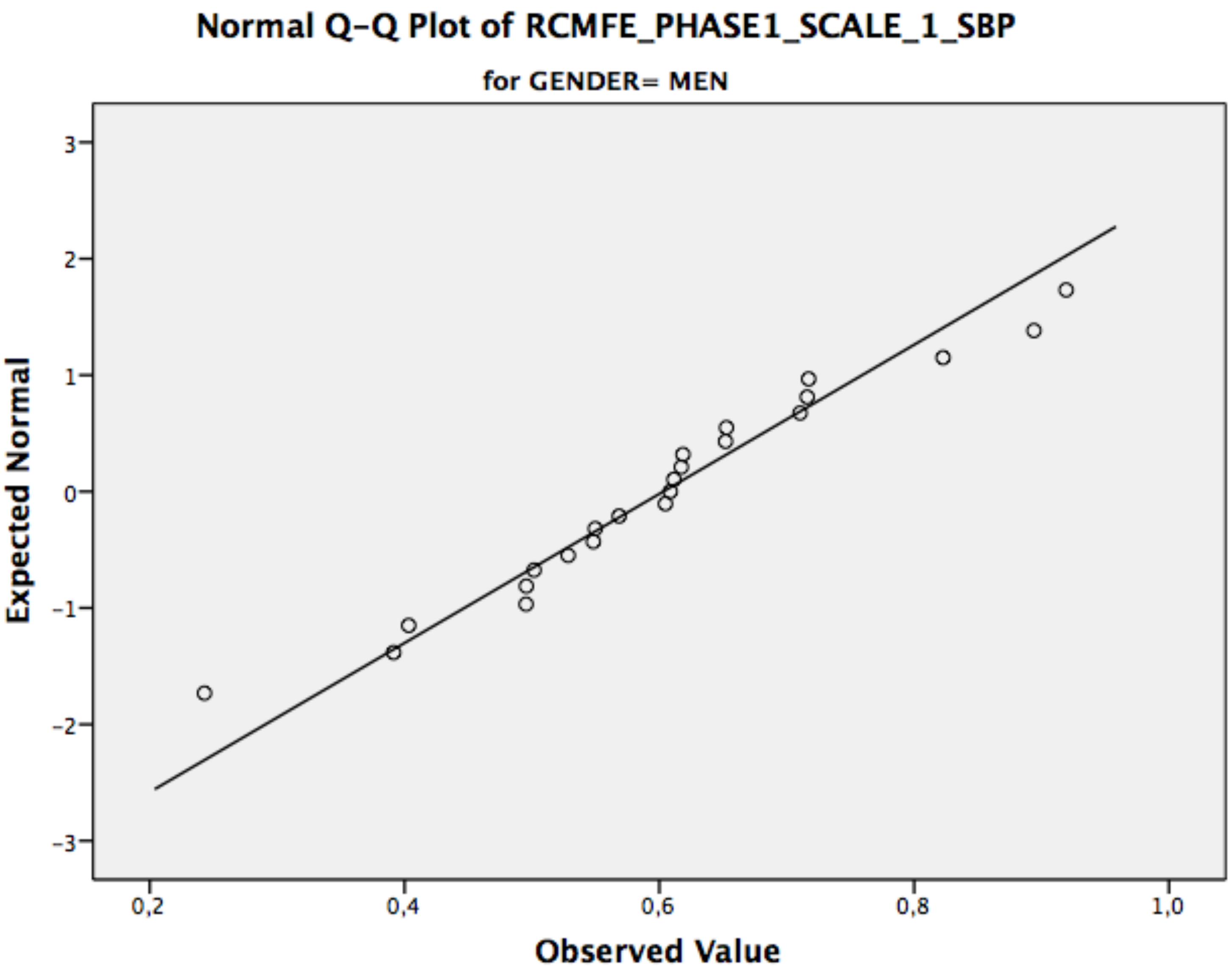
Normal Q-Q Plot of RCMFE_PHASE1_SCALE_1_SBP
for GENDER= MEN
Expected Normal
Observed Value
3
2
1
0
-1
-2
-3
0,2
0,4
0,6
0,8
1,0

RCMFE_PHASE1_SCALE_2_SBP

Normal Q-Q Plots

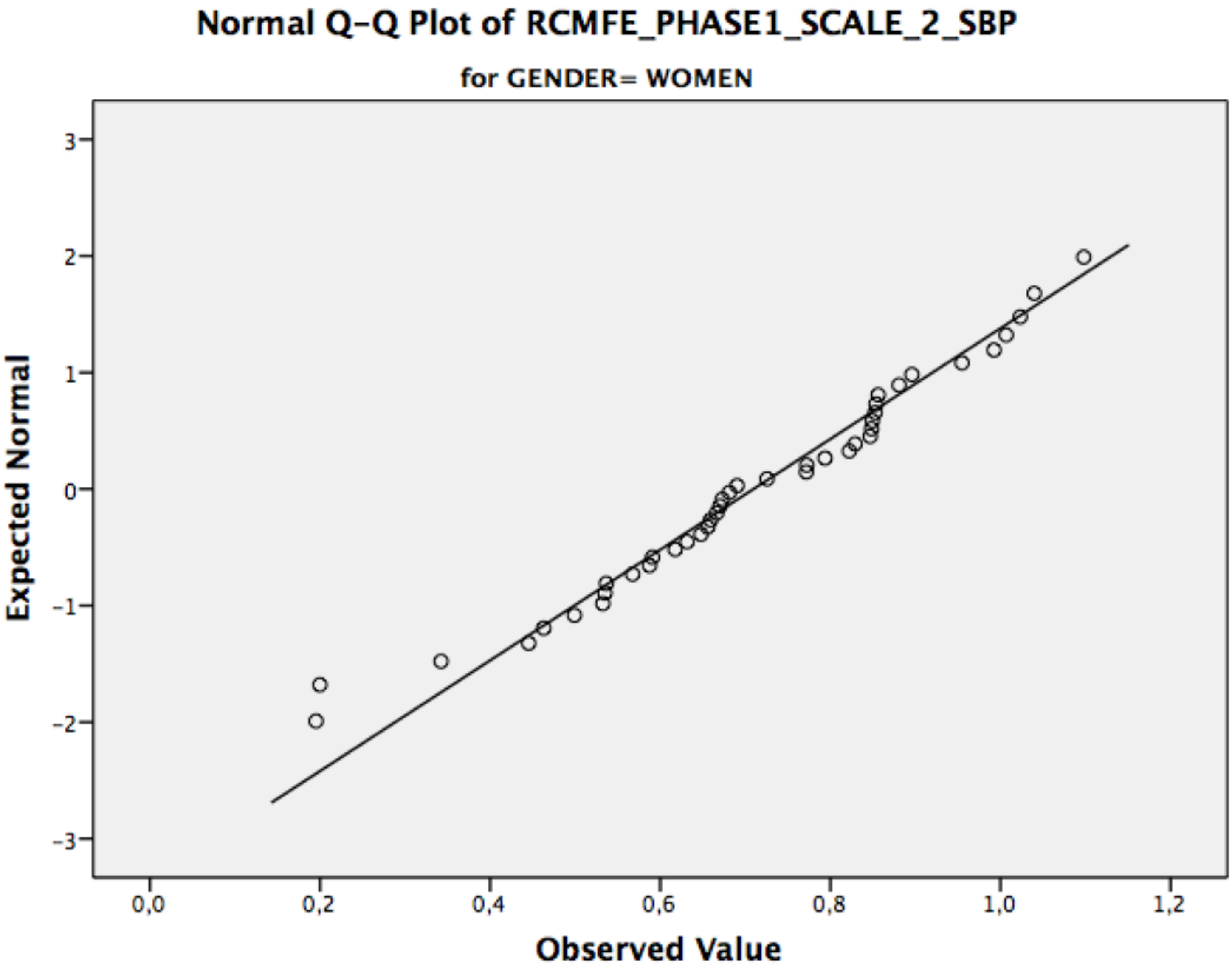

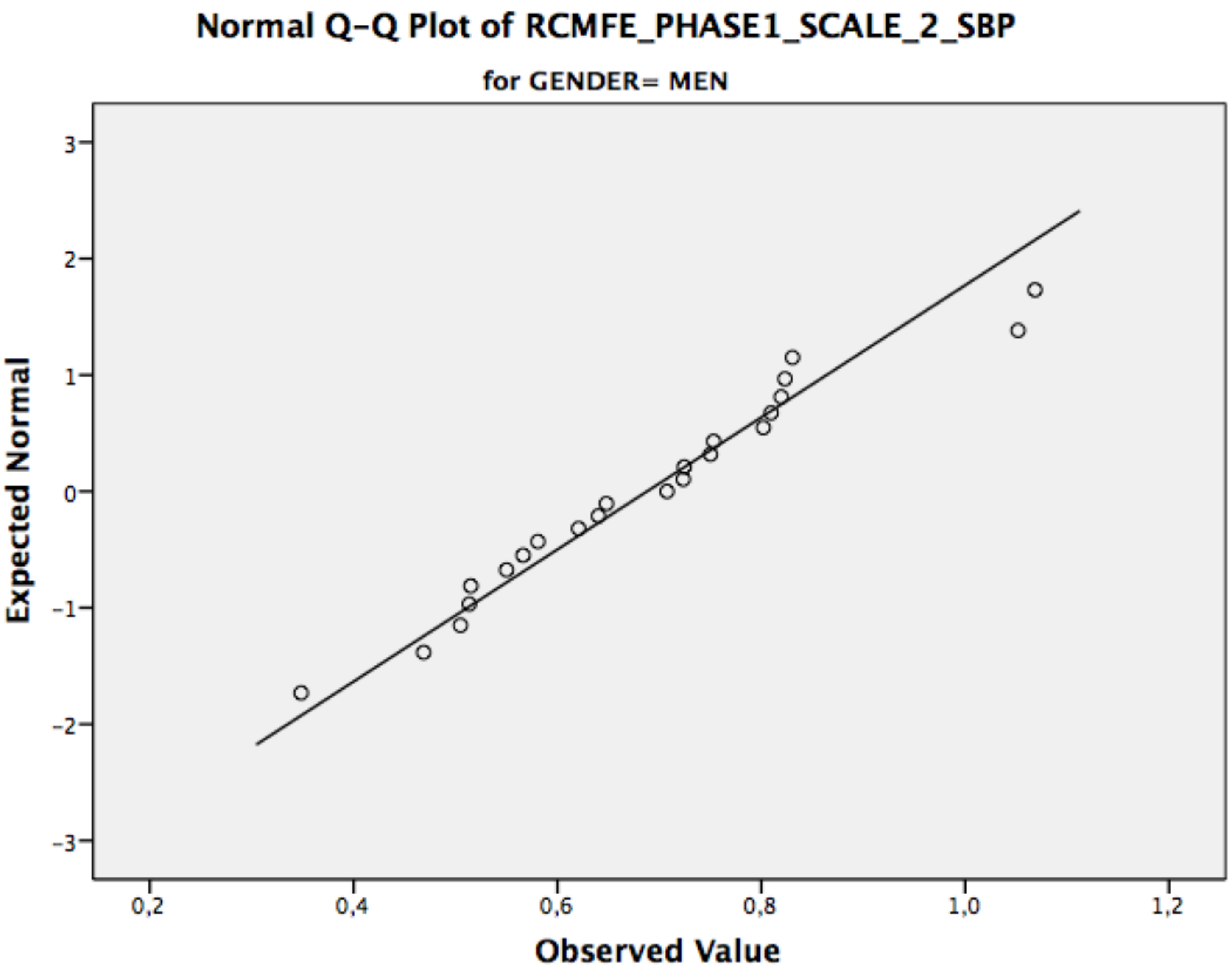
Normal Q-Q Plot of RCMFE_PHASE1_SCALE_2_SBP
for GENDER= MEN
Expected Normal
Observed Value
3
2
1
0
-1
-2
-3
0,2
0,4
0,6
0,8
1,0
1,2

RCMFE_PHASE1_SCALE_3_SBP

Normal Q-Q Plots

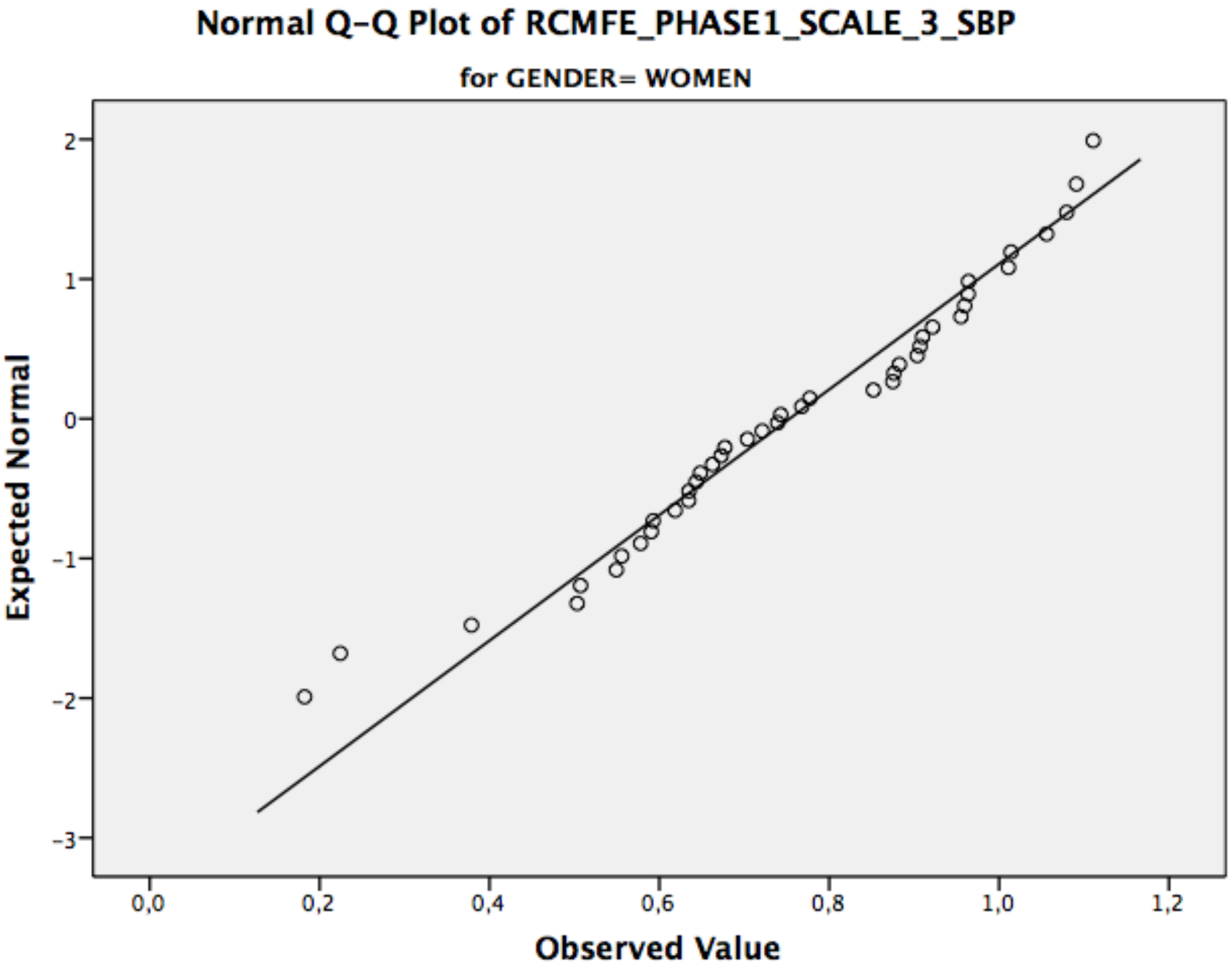

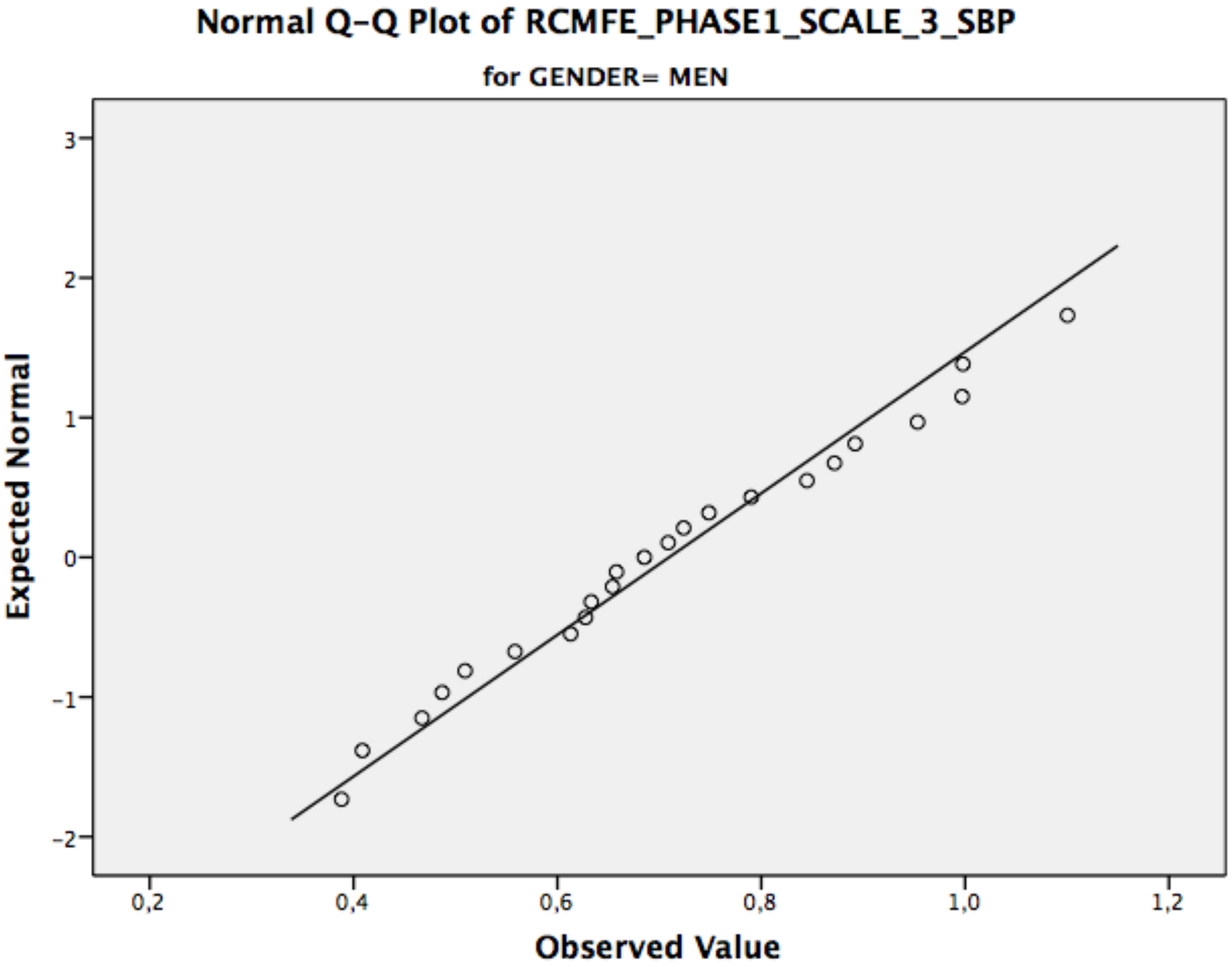
Normal Q-Q Plot of RCMFE_PHASE1_SCALE_3_SBP
for GENDER= MEN
Expected Normal
3
2
1
0
-1
-2
0,2
0,4
0,6
0,8
1,0
1,2
Observed Value

RCMFE_PHASE1_SCALE_4_SBP

Normal Q-Q Plots

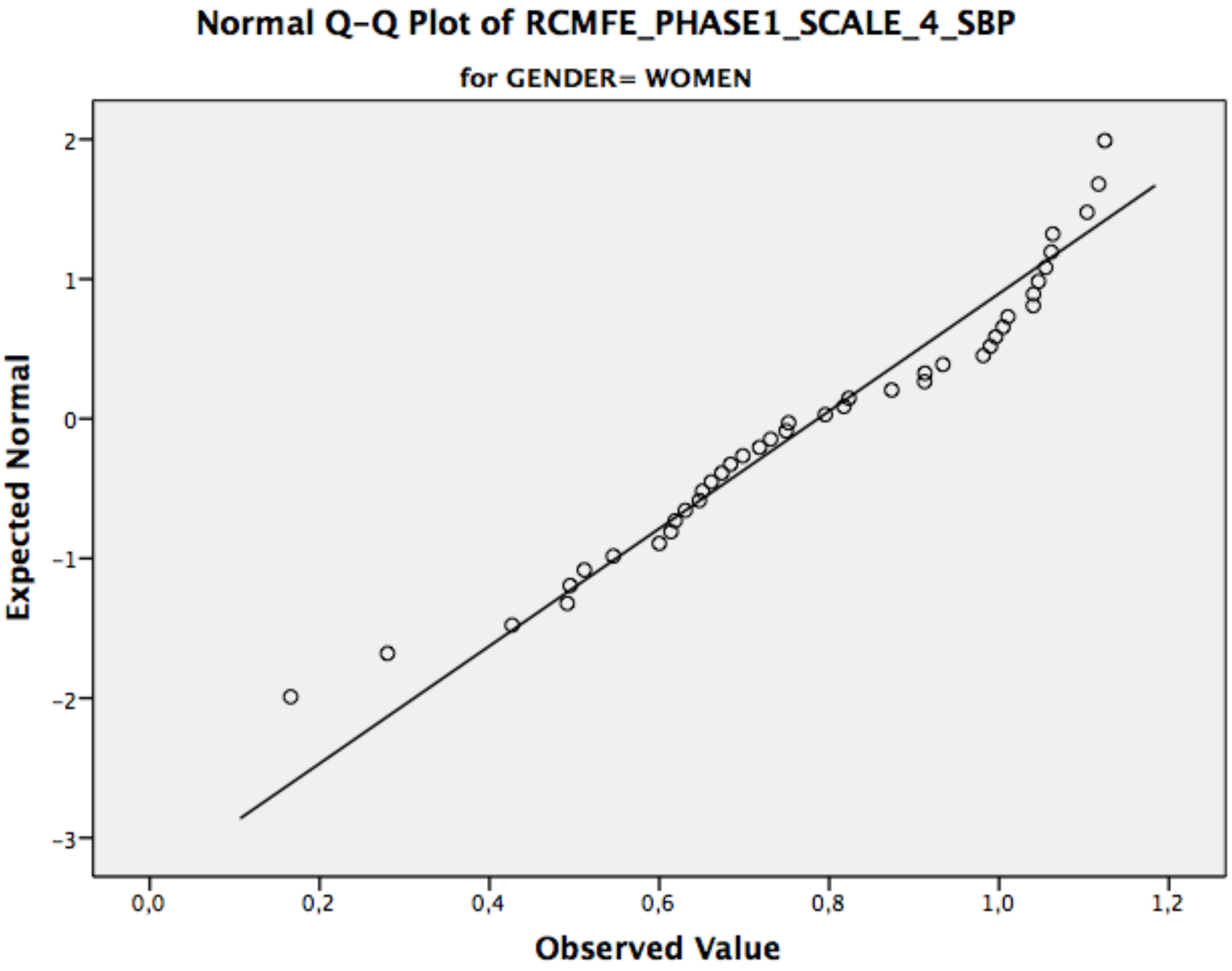

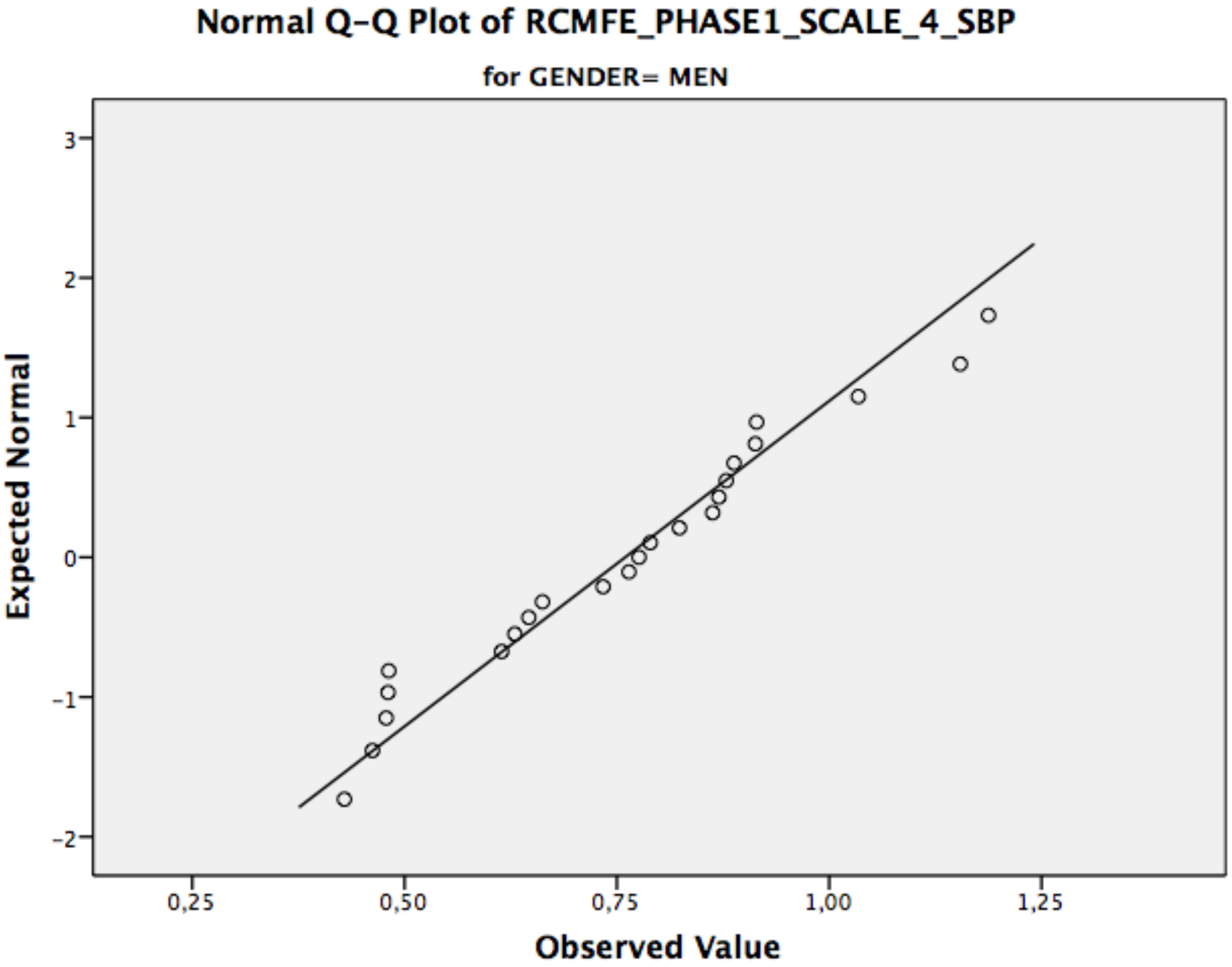

Normal Q-Q Plot of RCMFE_PHASE1_SCALE_4_SBP
for GENDER= MEN
Expected Normal
3
2
1
0
-1
-2
0,25
0,50
0,75
1,00
1,25
Observed Value

RCMFE_PHASE1_SCALE_5_SBP

Normal Q-Q Plots

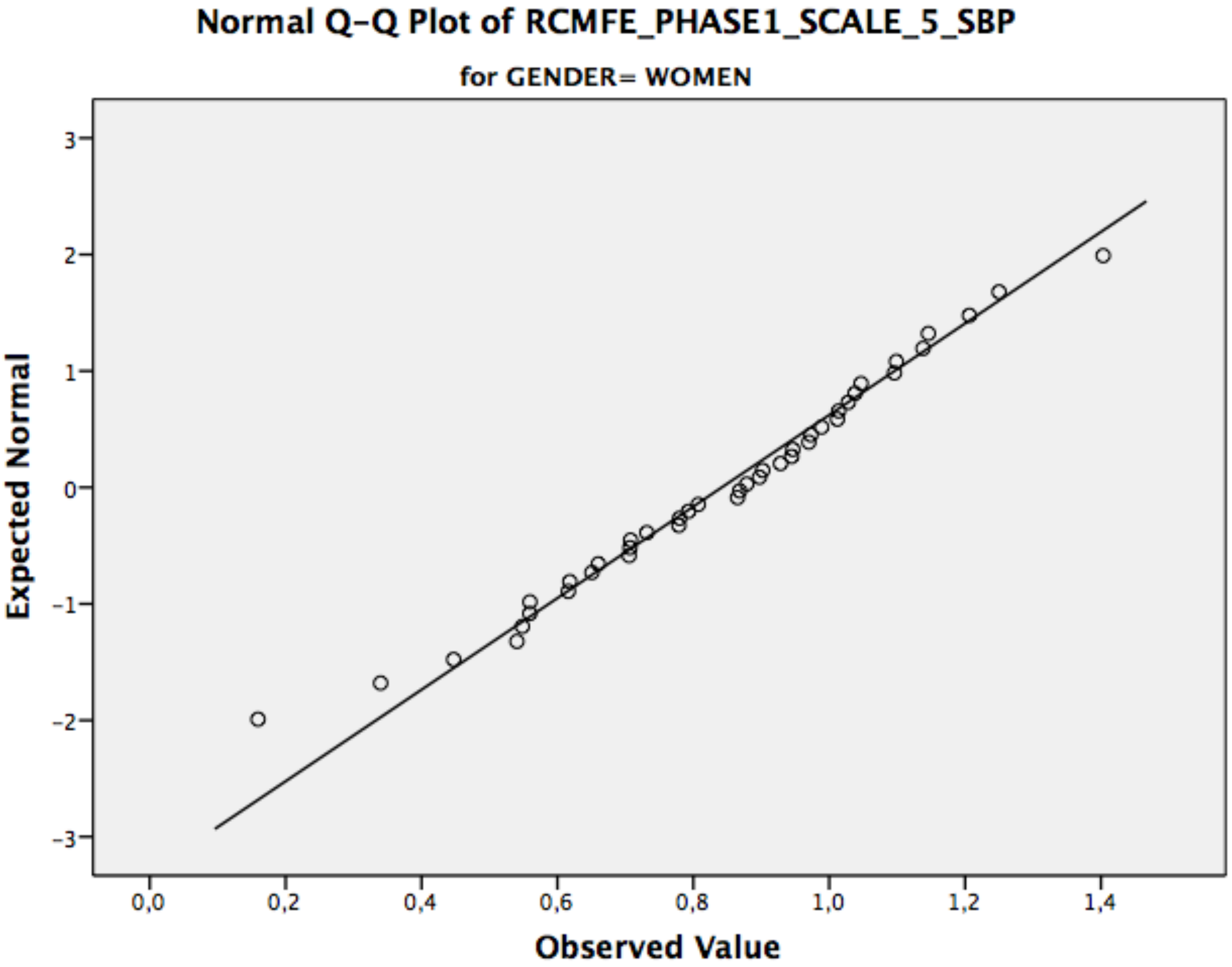

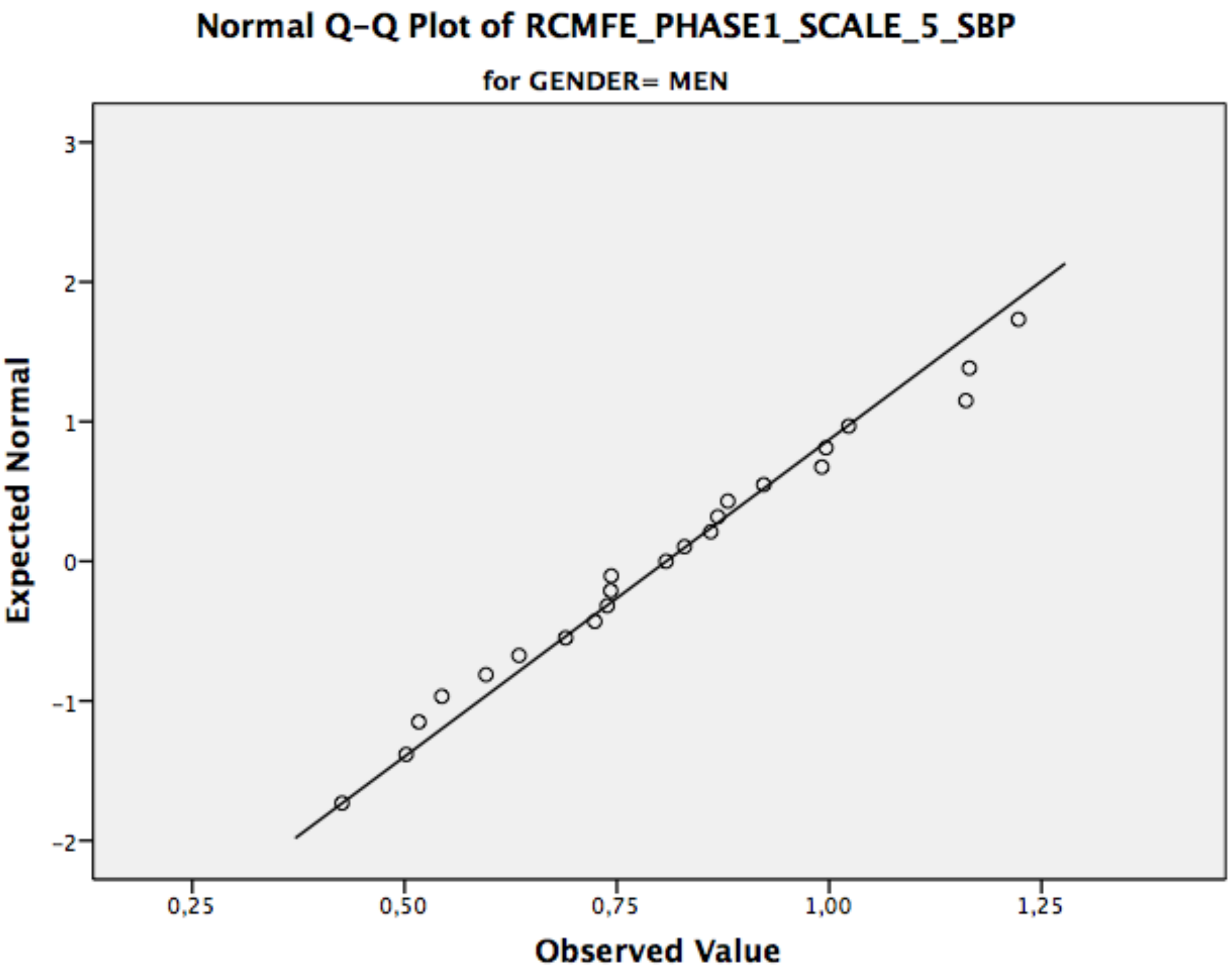
Normal Q-Q Plot of RCMFE_PHASE1_SCALE_5_SBP
for GENDER= MEN
Expected Normal
3
2
1
0
-1
-2
0,25
0,50
0,75
1,00
1,25
Observed Value

RCMFE_PHASE2_SCALE_1_SBP

Normal Q-Q Plots

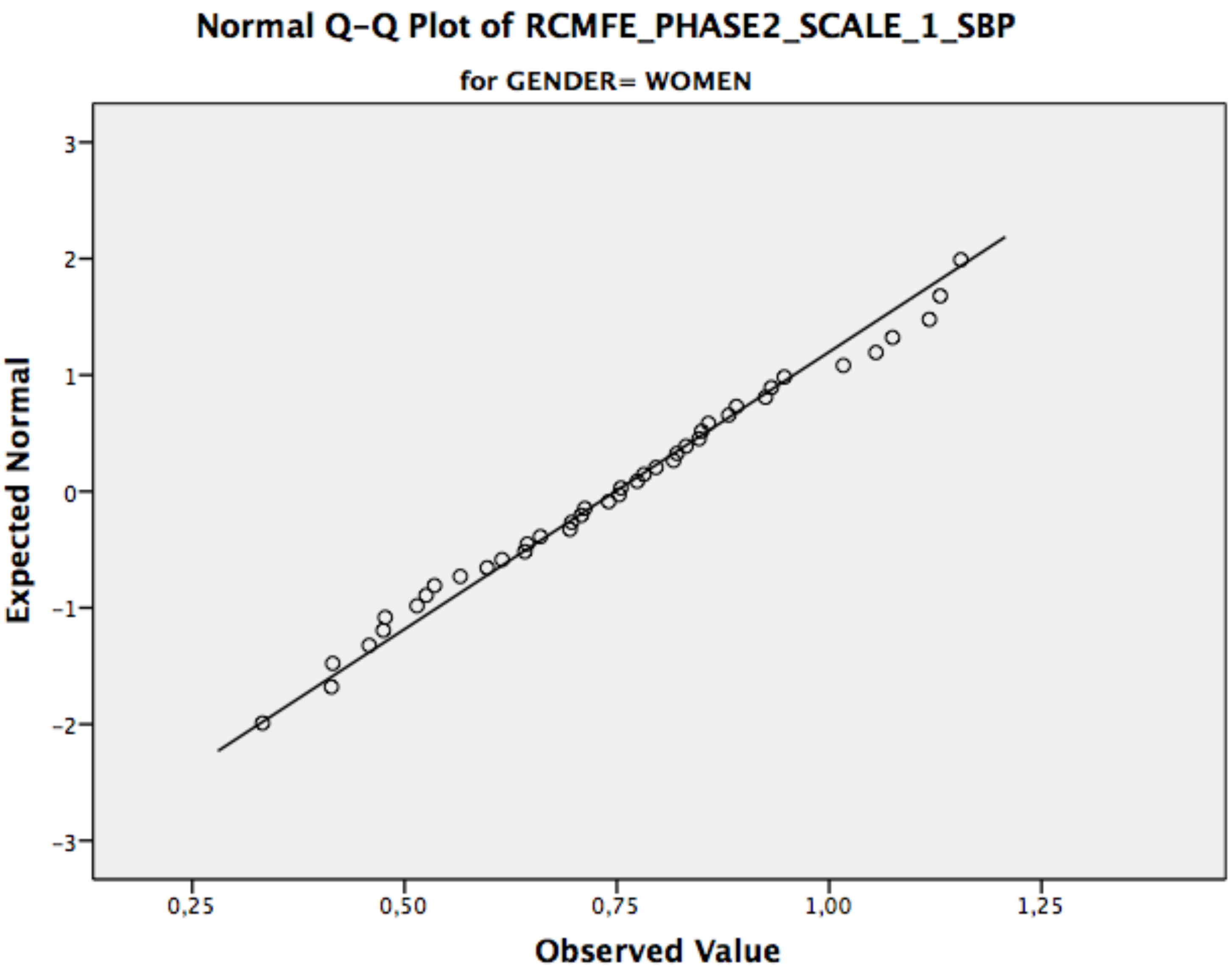

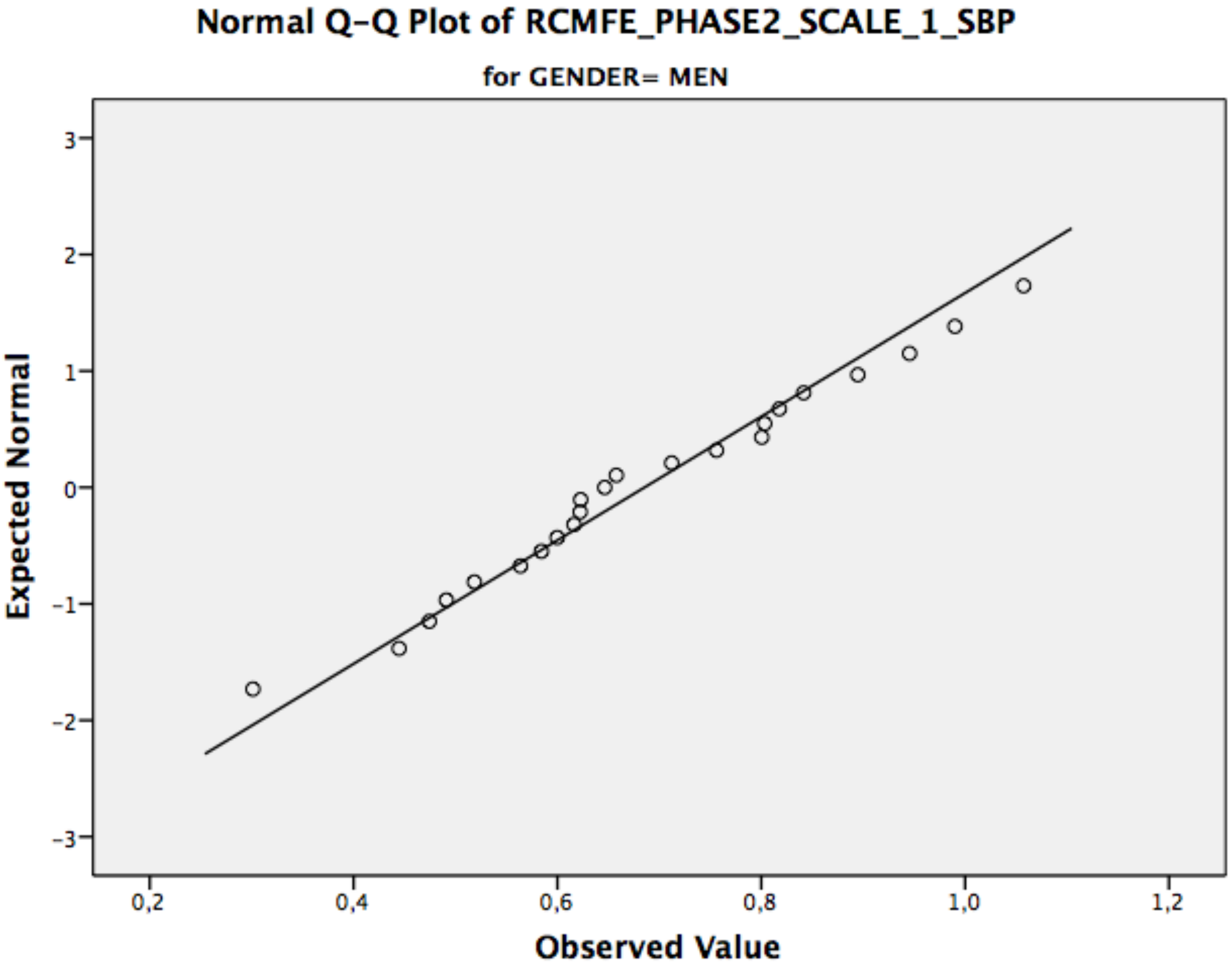
Normal Q-Q Plot of RCMFE_PHASE2_SCALE_1_SBP
for GENDER= MEN
Expected Normal
Observed Value
3
2
1
0
-1
-2
-3
0,2
0,4
0,6
0,8
1,0
1,2

RCMFE_PHASE2_SCALE_2_SBP

Normal Q-Q Plots

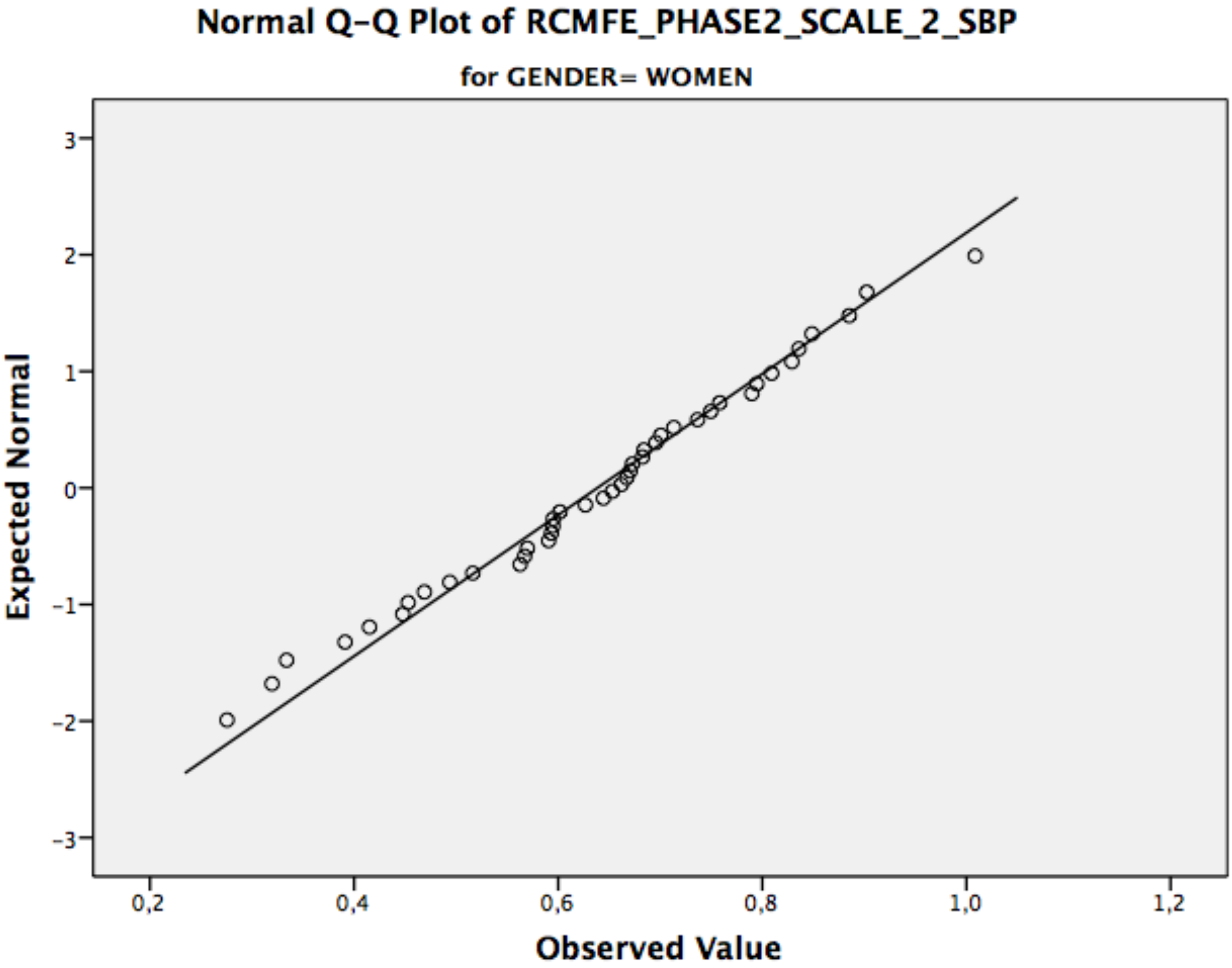

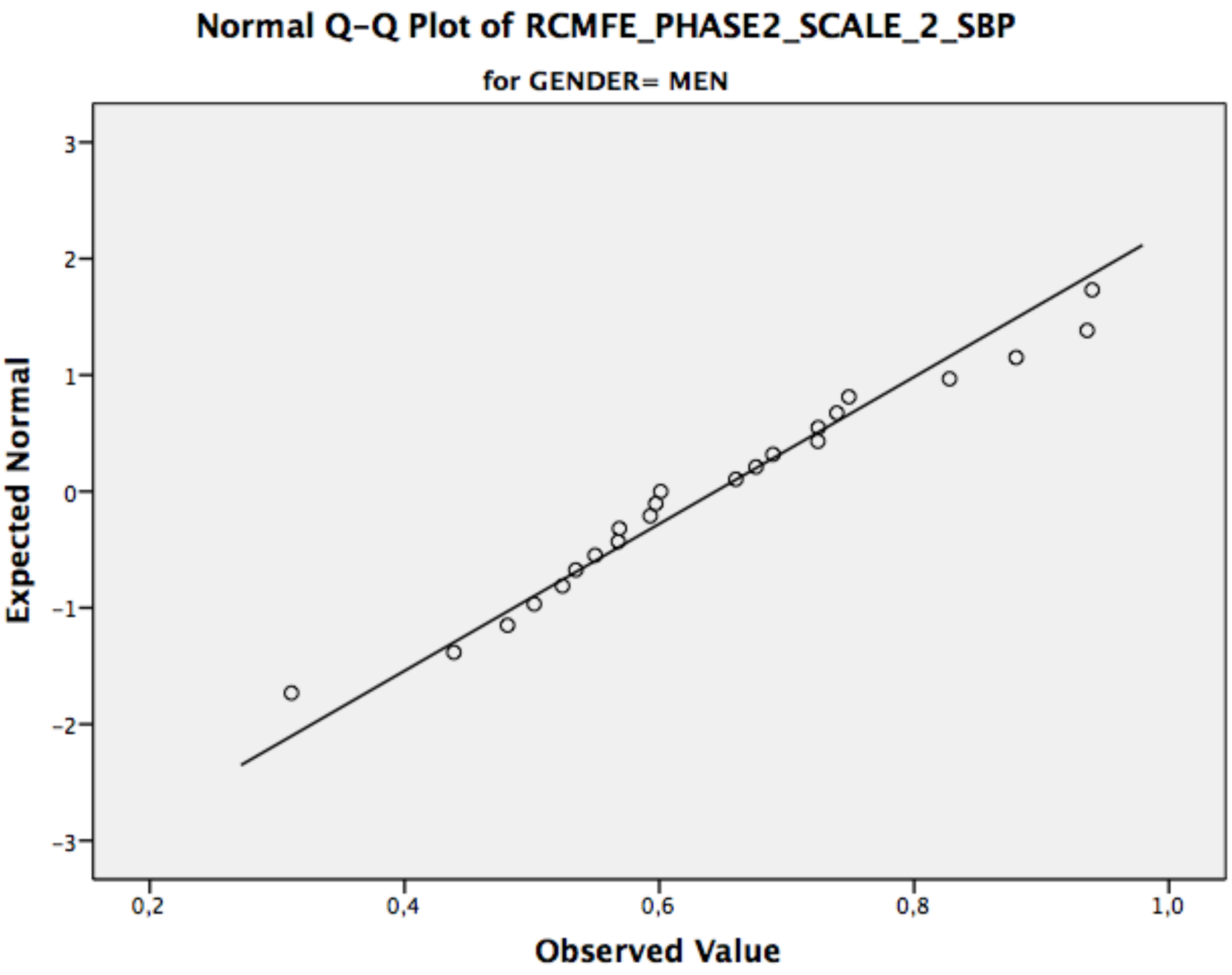

Normal Q-Q Plot of RCMFE_PHASE2_SCALE_2_SBP
for GENDER= MEN
Expected Normal
Observed Value
3
2
1
0
-1
-2
-3
0,2
0,4
0,6
0,8
1,0

RCMFE_PHASE2_SCALE_3_SBP

Normal Q-Q Plots

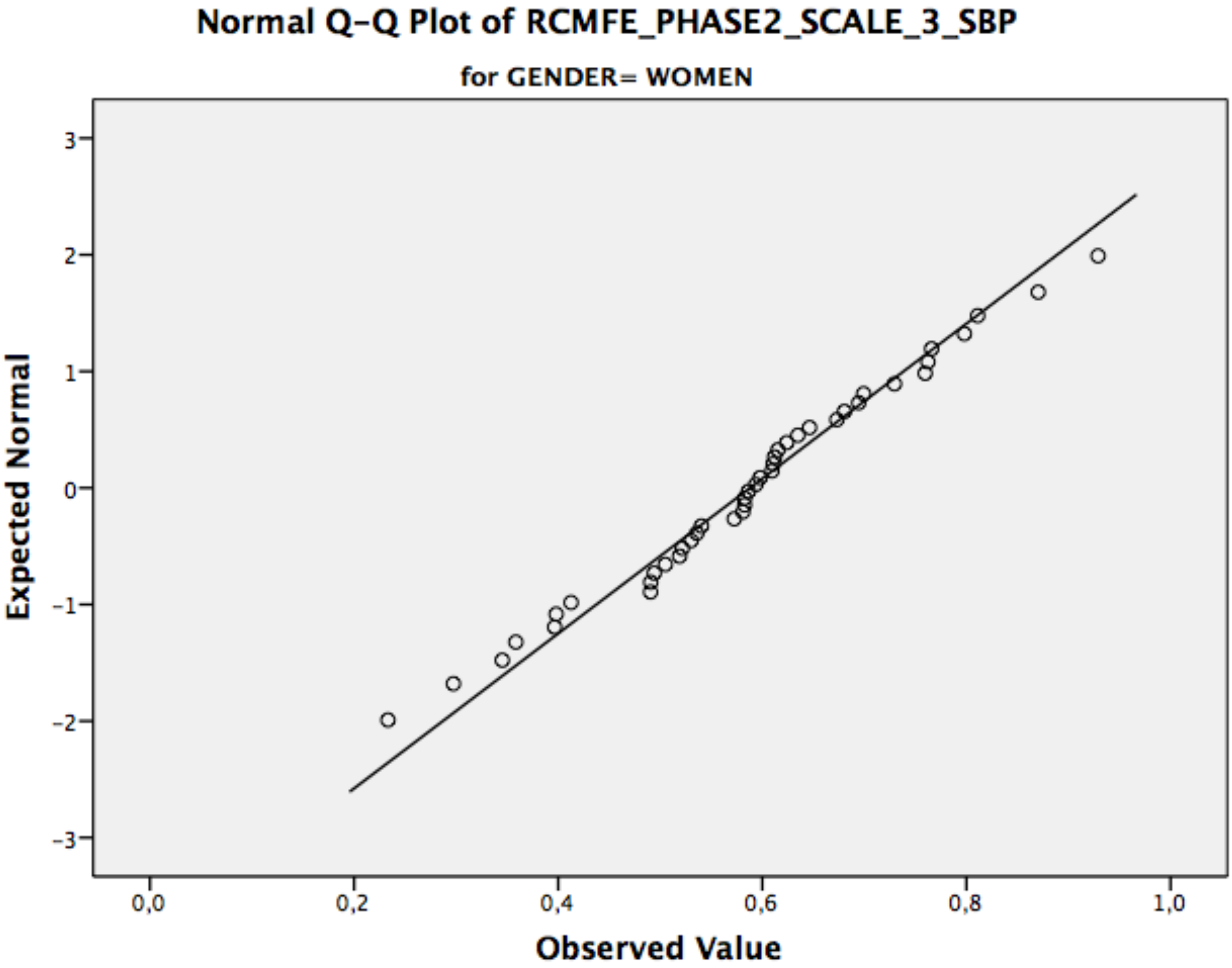

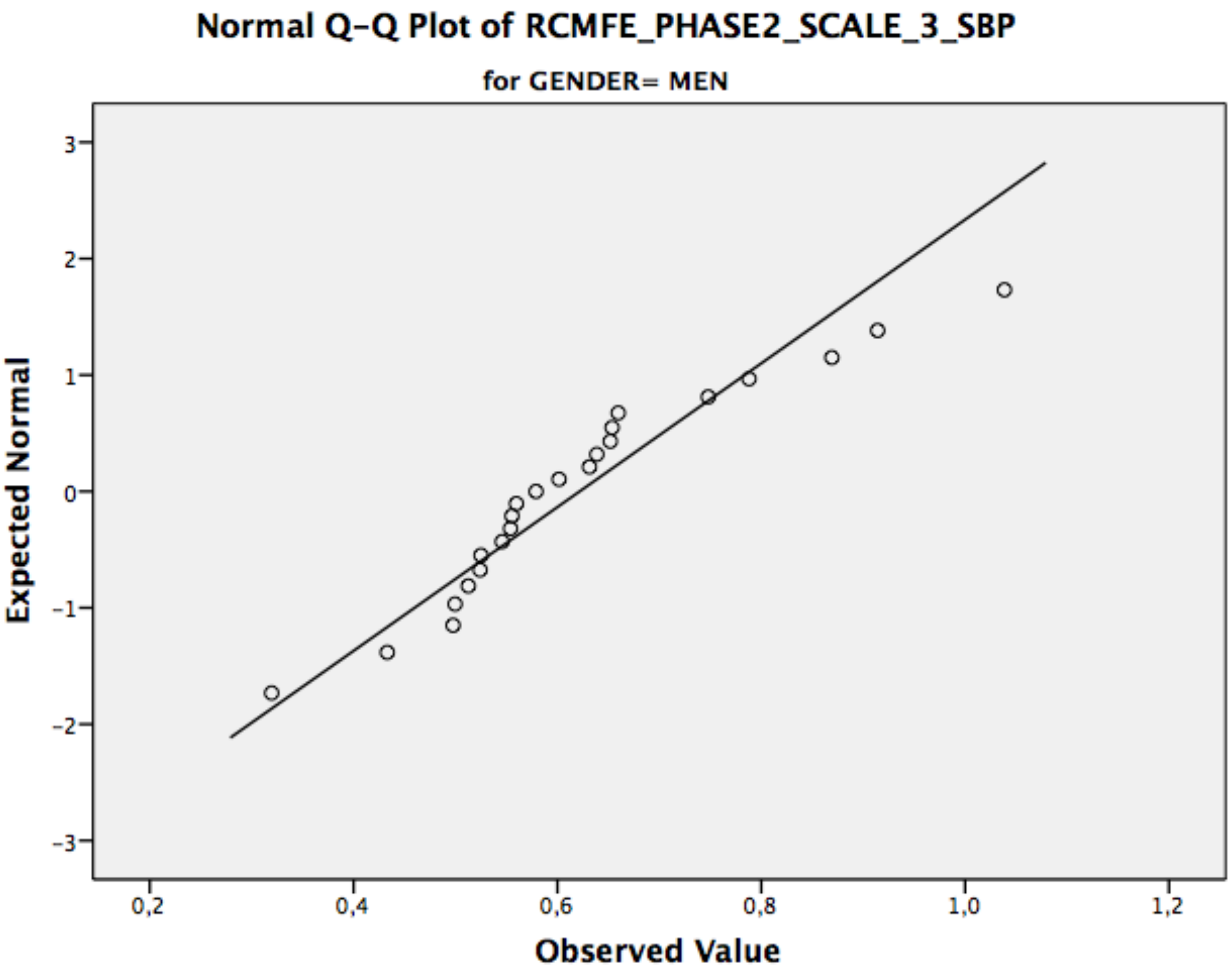

Normal Q-Q Plot of RCMFE_PHASE2_SCALE_3_SBP
for GENDER= MEN
Expected Normal
3
2
1
0
-1
-2
-3
0,2
0,4
0,6
0,8
1,0
1,2
Observed Value

RCMFE_PHASE2_SCALE_4_SBP

Normal Q-Q Plots

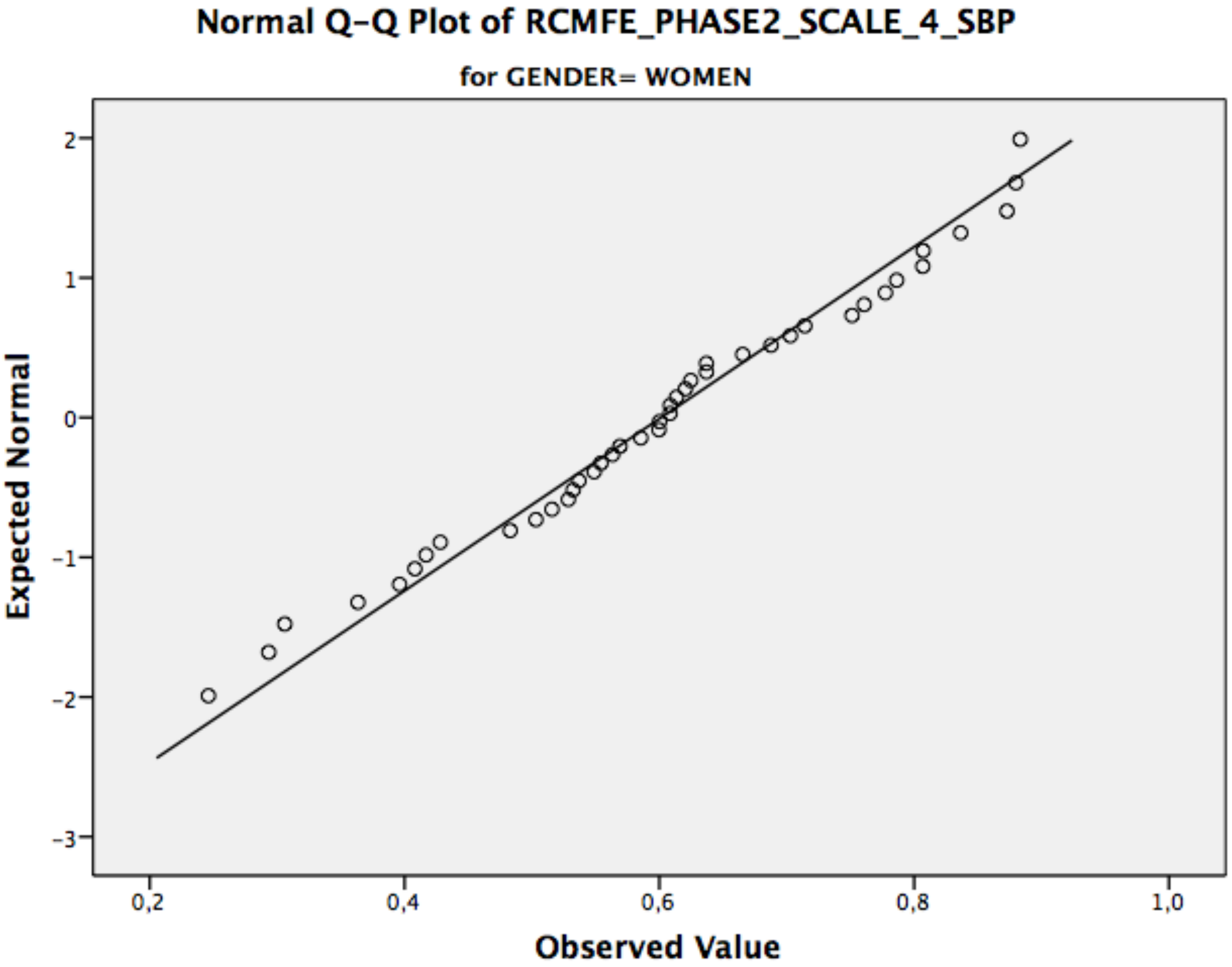

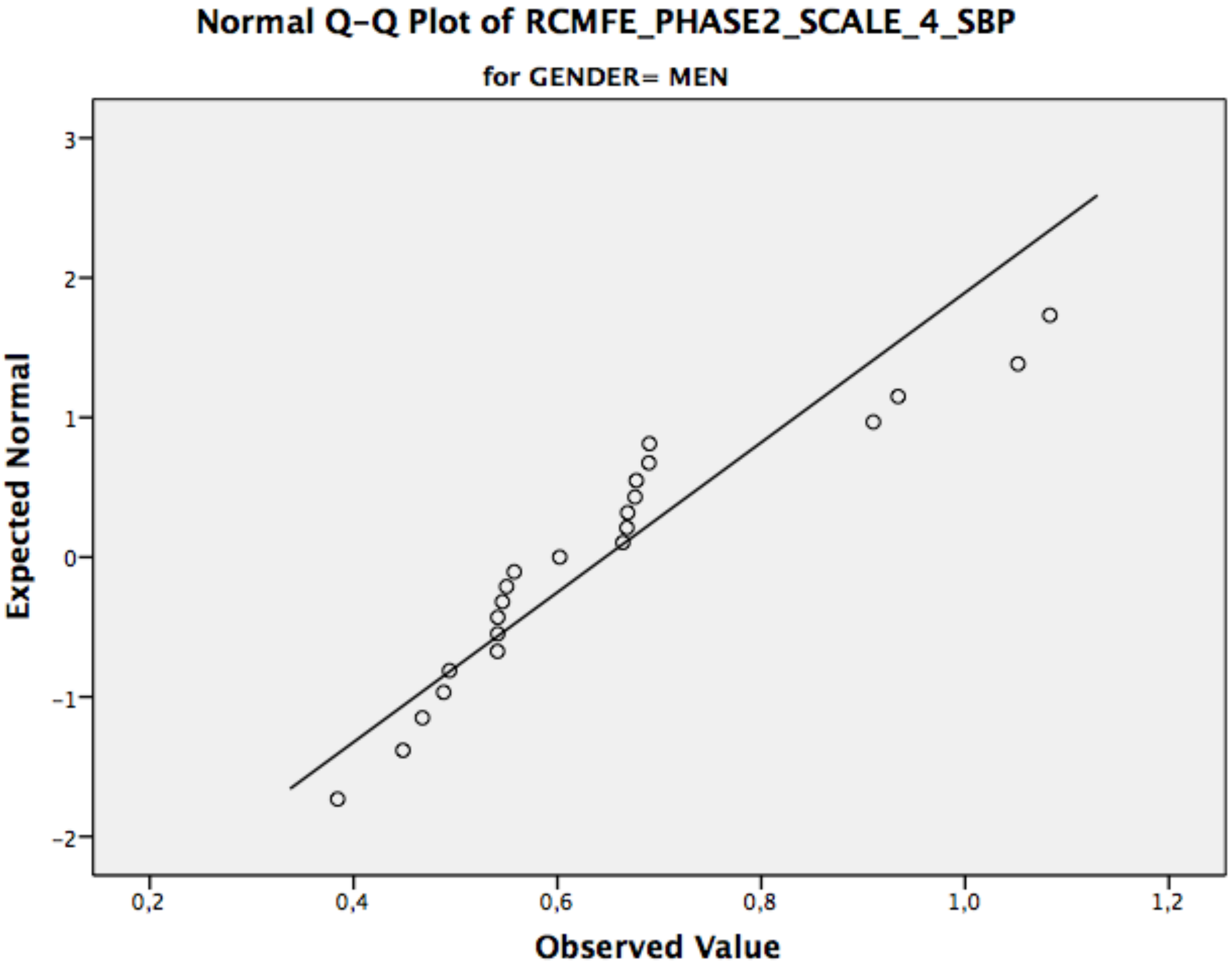
Normal Q-Q Plot of RCMFE_PHASE2_SCALE_4_SBP
for GENDER= MEN
Expected Normal
3
2
1
0
-1
-2
0,2
0,4
0,6
0,8
1,0
1,2
Observed Value

RCMFE_PHASE2_SCALE_5_SBP

Normal Q-Q Plots

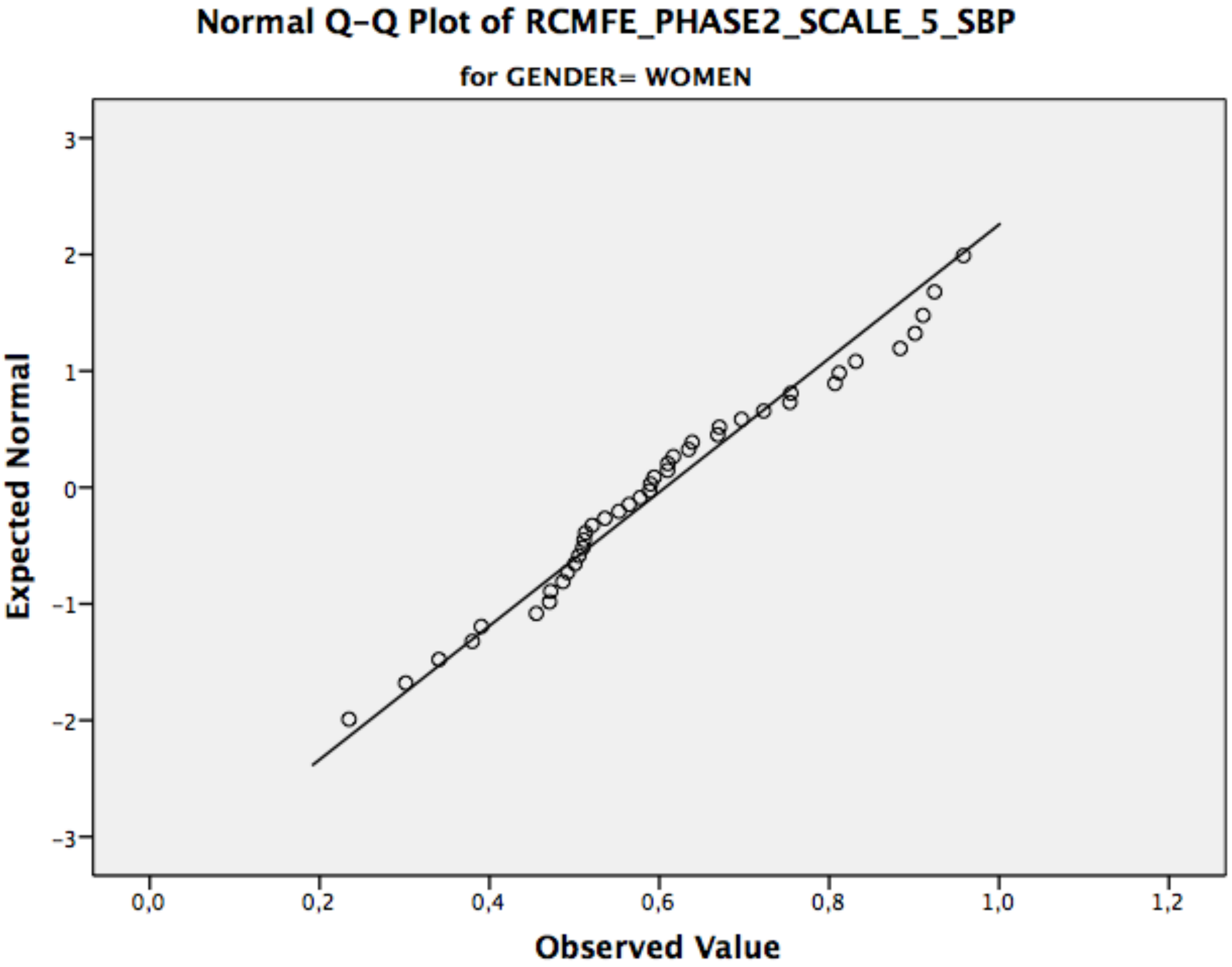

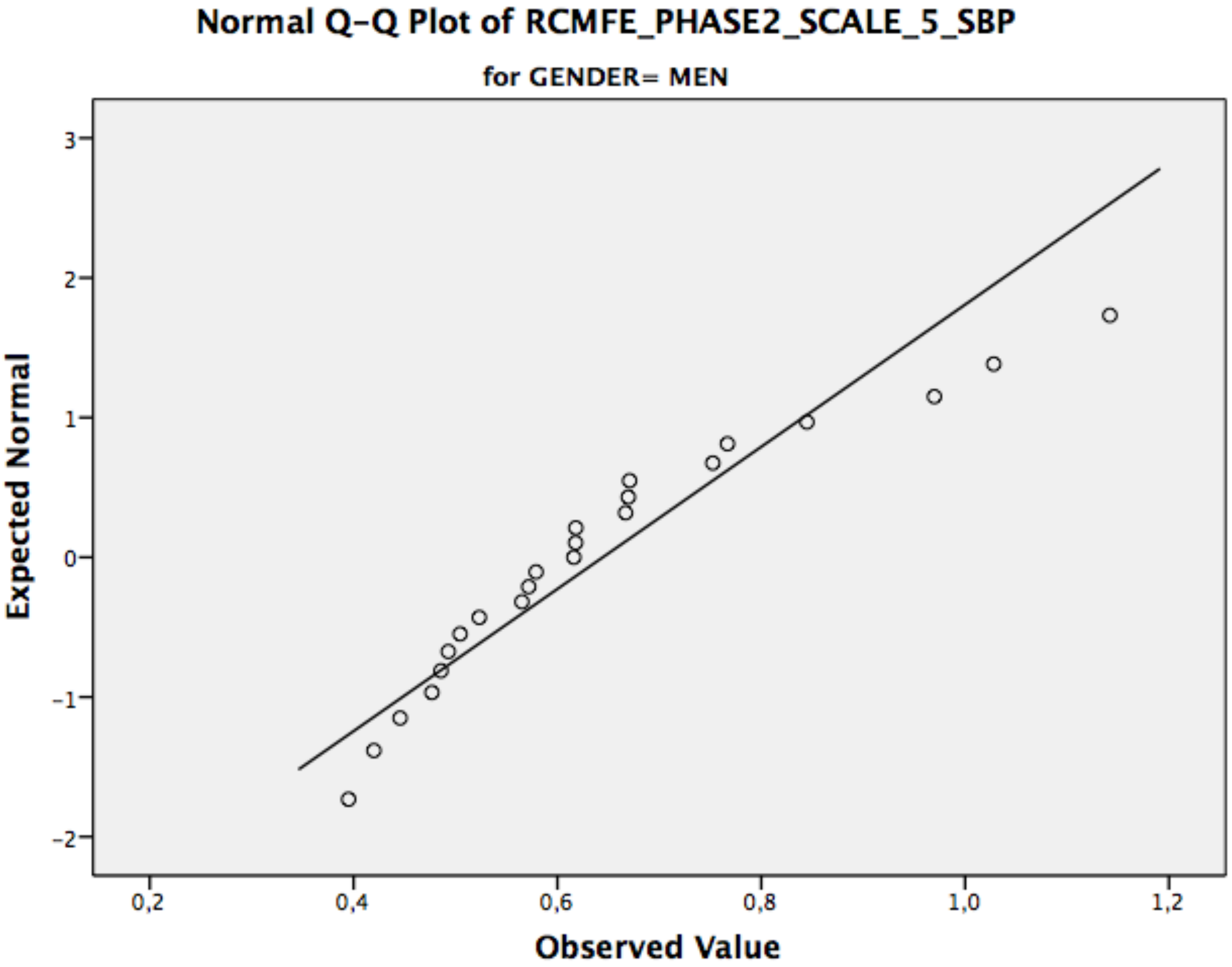
Normal Q-Q Plot of RCMFE_PHASE2_SCALE_5_SBP
for GENDER= MEN
Expected Normal
Observed Value
3
2
1
0
-1
-2
0,2
0,4
0,6
0,8
1,0
1,2

RCMFE_PHASE3_SCALE_1_SBP

Normal Q-Q Plots

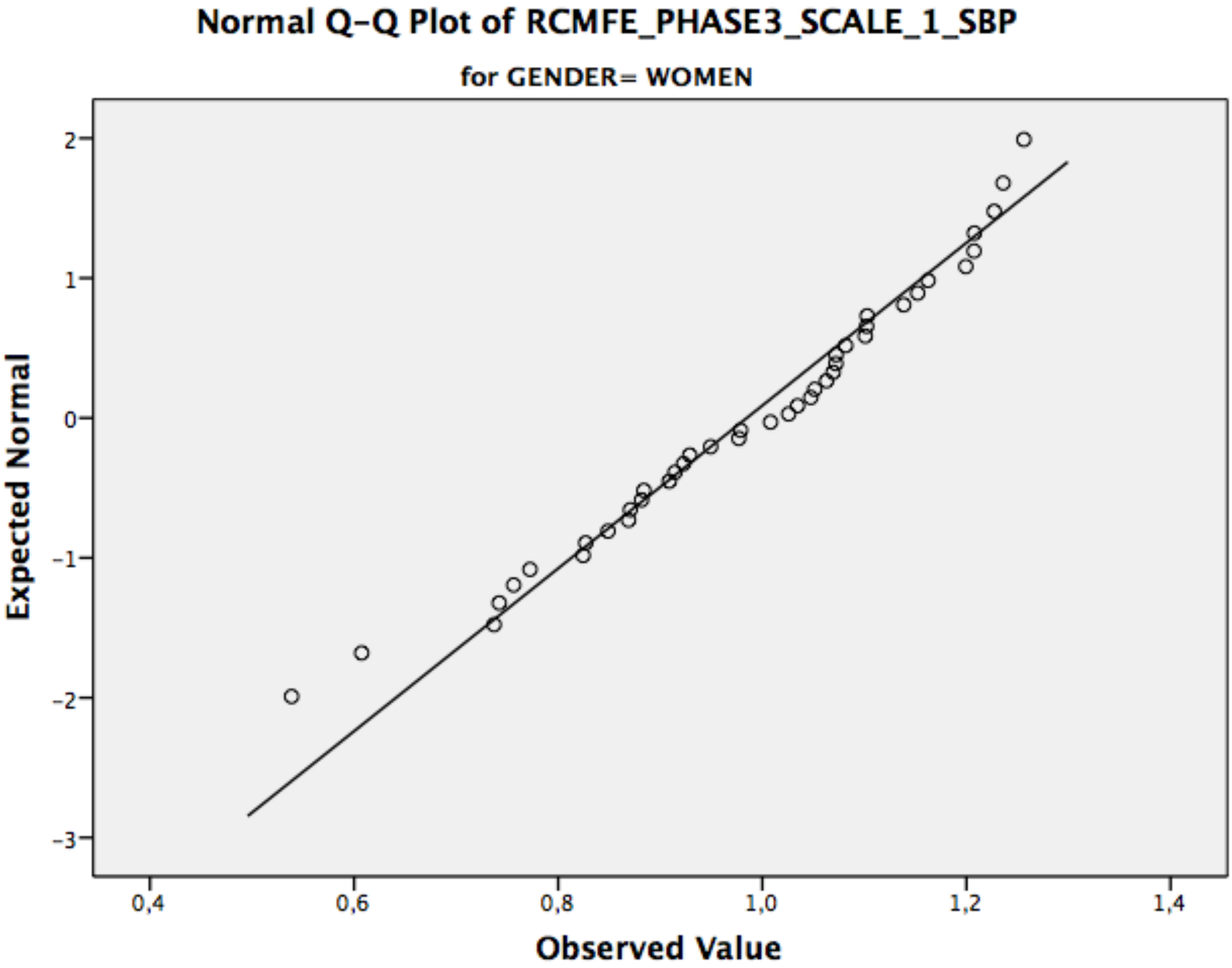

Normal Q-Q Plot of RCMFE_PHASE3_SCALE_1_SBP

for GENDER= MEN

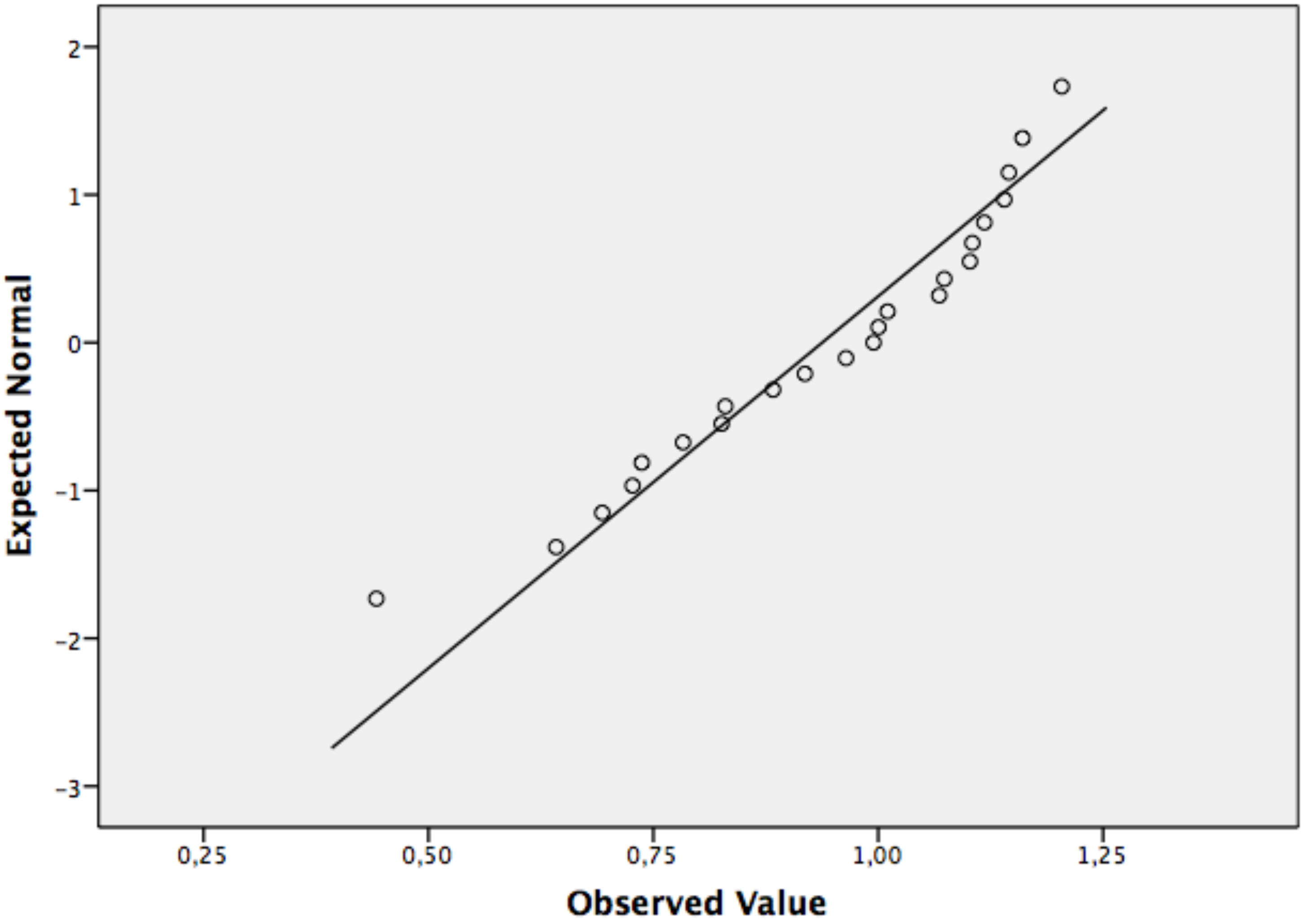

RCMFE_PHASE3_SCALE_2_SBP

Normal Q-Q Plots

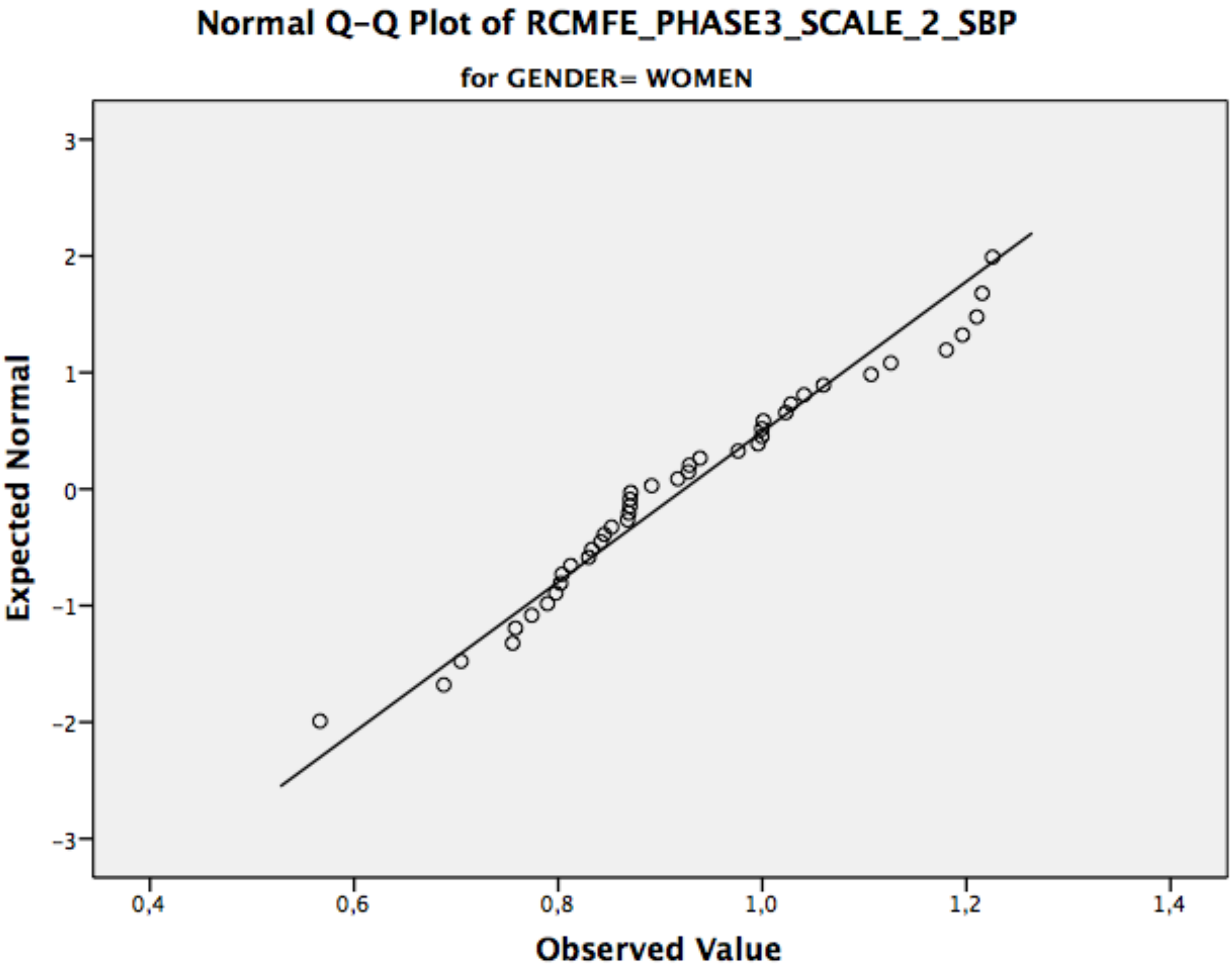

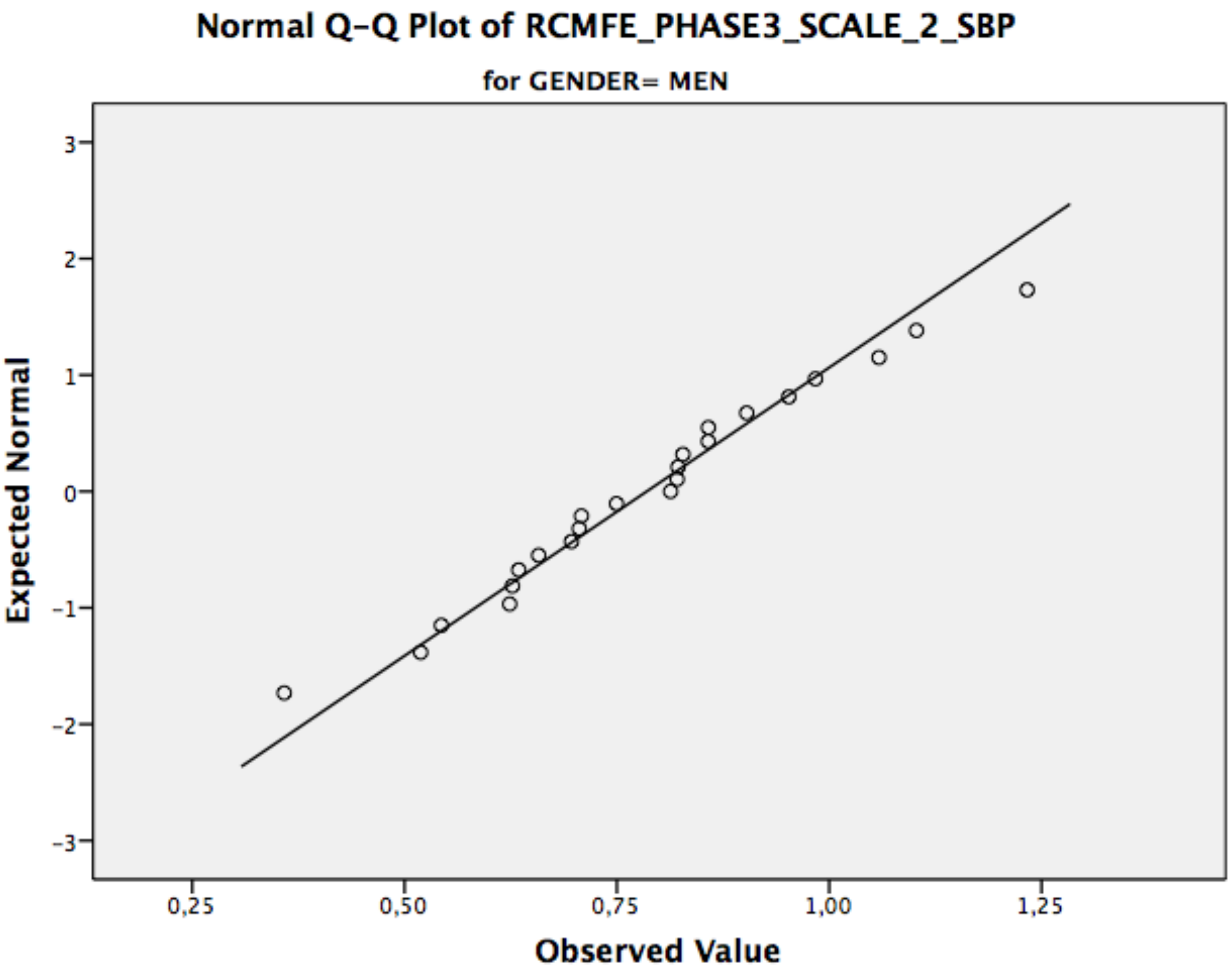

Normal Q-Q Plot of RCMFE_PHASE3_SCALE_2_SBP
for GENDER= MEN
Expected Normal
Observed Value
3
2
1
0
-1
-2
-3
0,25
0,50
0,75
1,00
1,25

RCMFE_PHASE3_SCALE_3_SBP

Normal Q-Q Plots

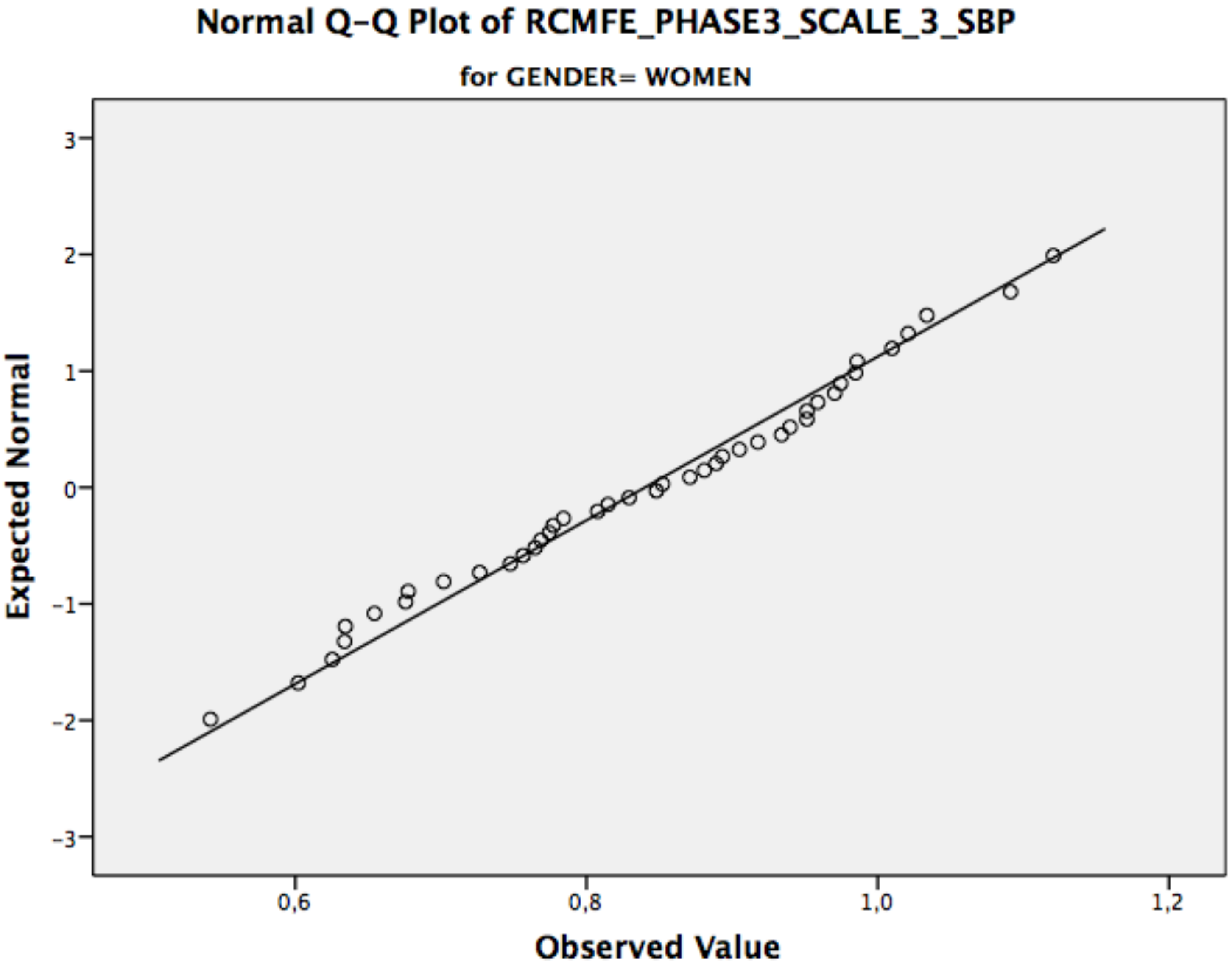

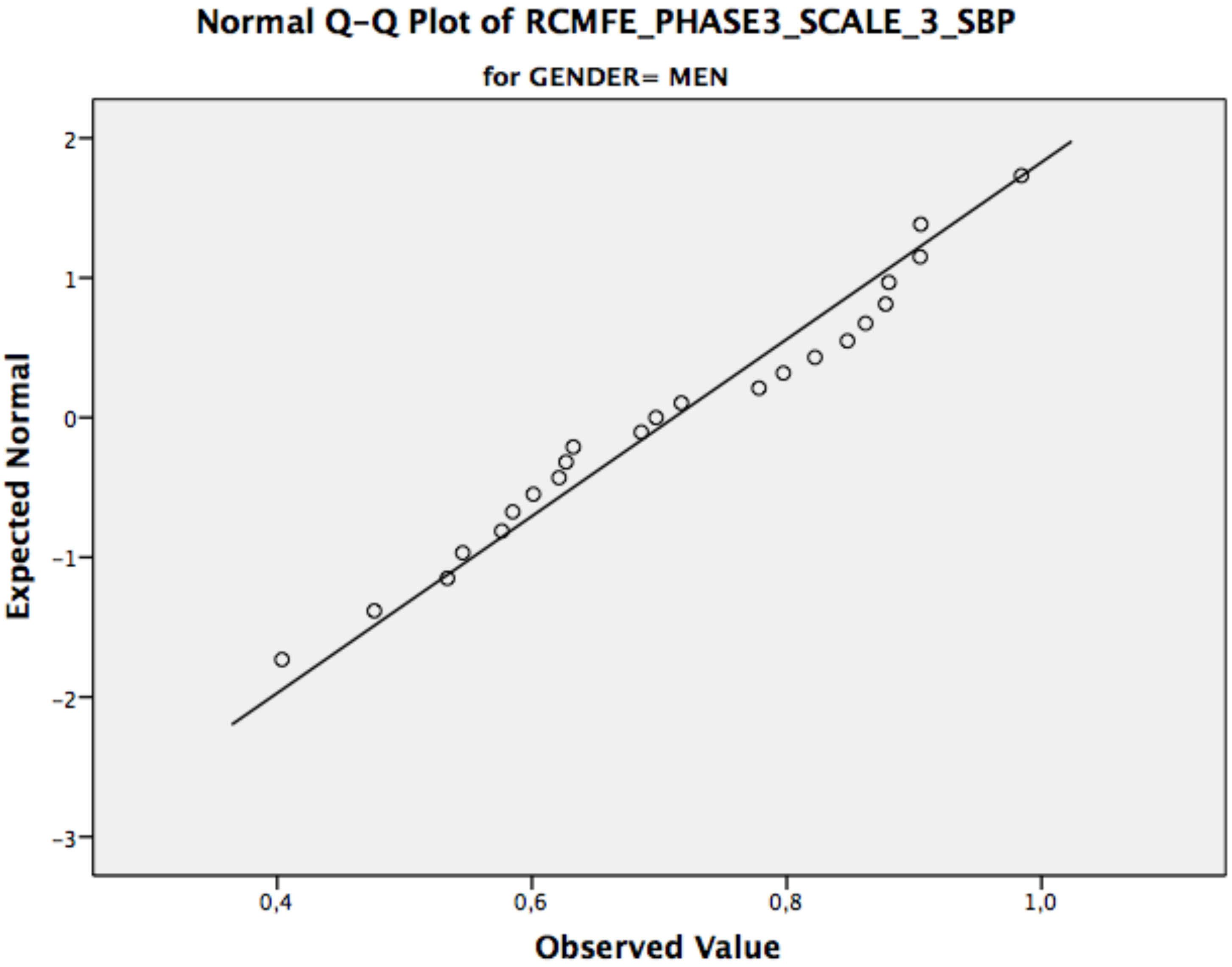

Normal Q-Q Plot of RCMFE_PHASE3_SCALE_3_SBP
for GENDER= MEN
Expected Normal
2
1
0
-1
-2
-3
0,4
0,6
0,8
1,0
Observed Value

RCMFE_PHASE3_SCALE_4_SBP

Normal Q-Q Plots

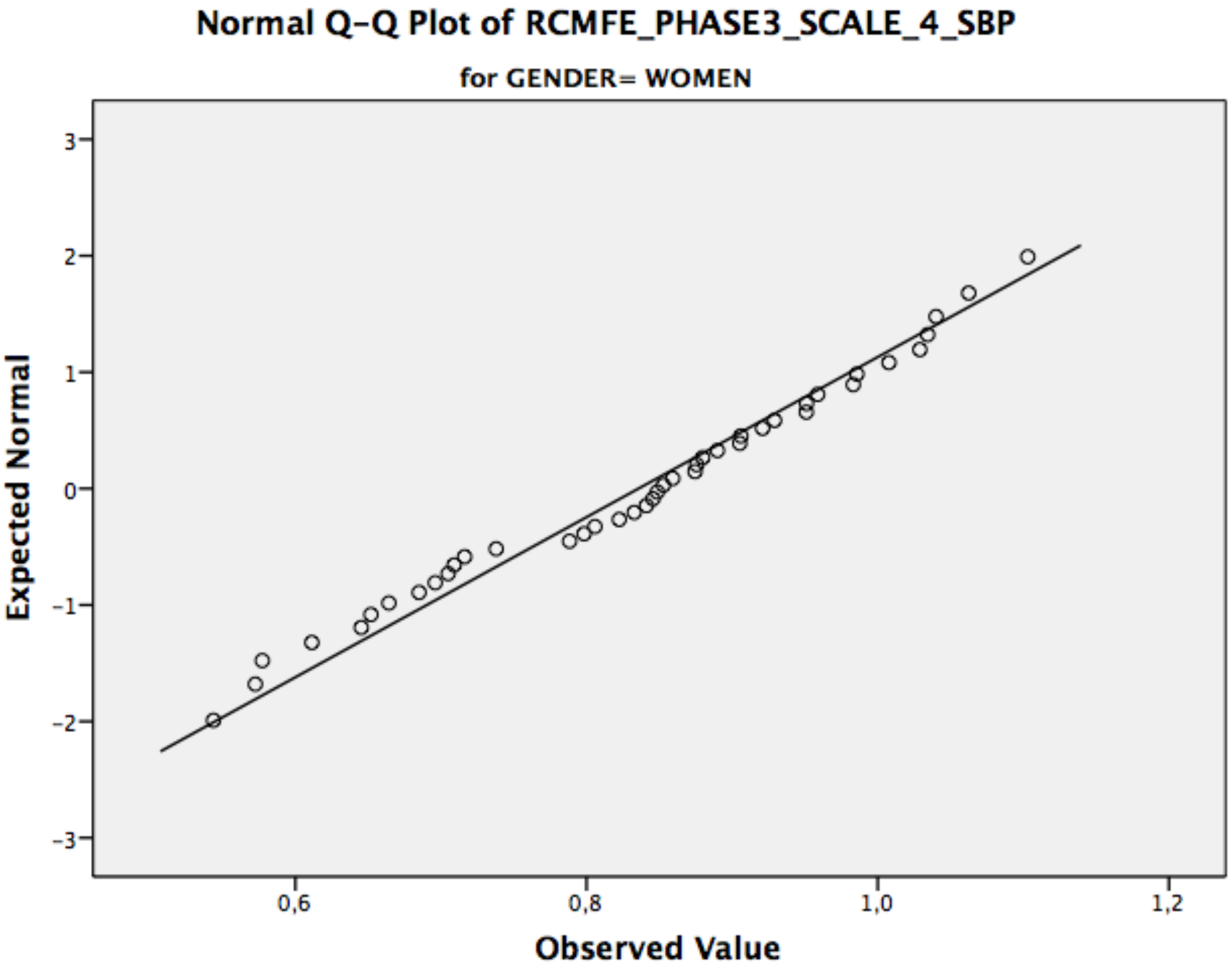

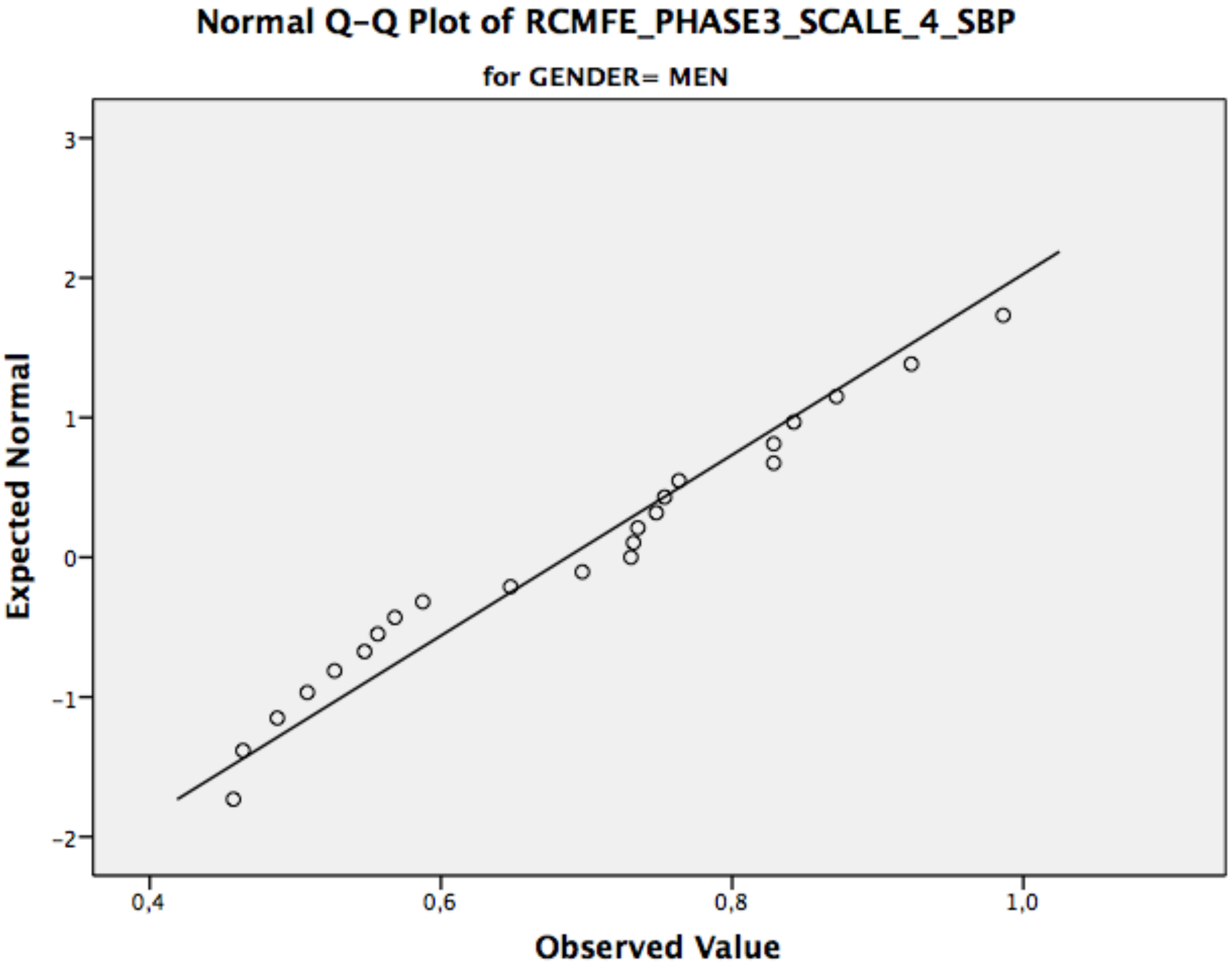

Normal Q-Q Plot of RCMFE_PHASE3_SCALE_4_SBP
for GENDER= MEN
Expected Normal
Observed Value
3
2
1
0
-1
-2
0,4
0,6
0,8
1,0

Normal Q-Q Plots

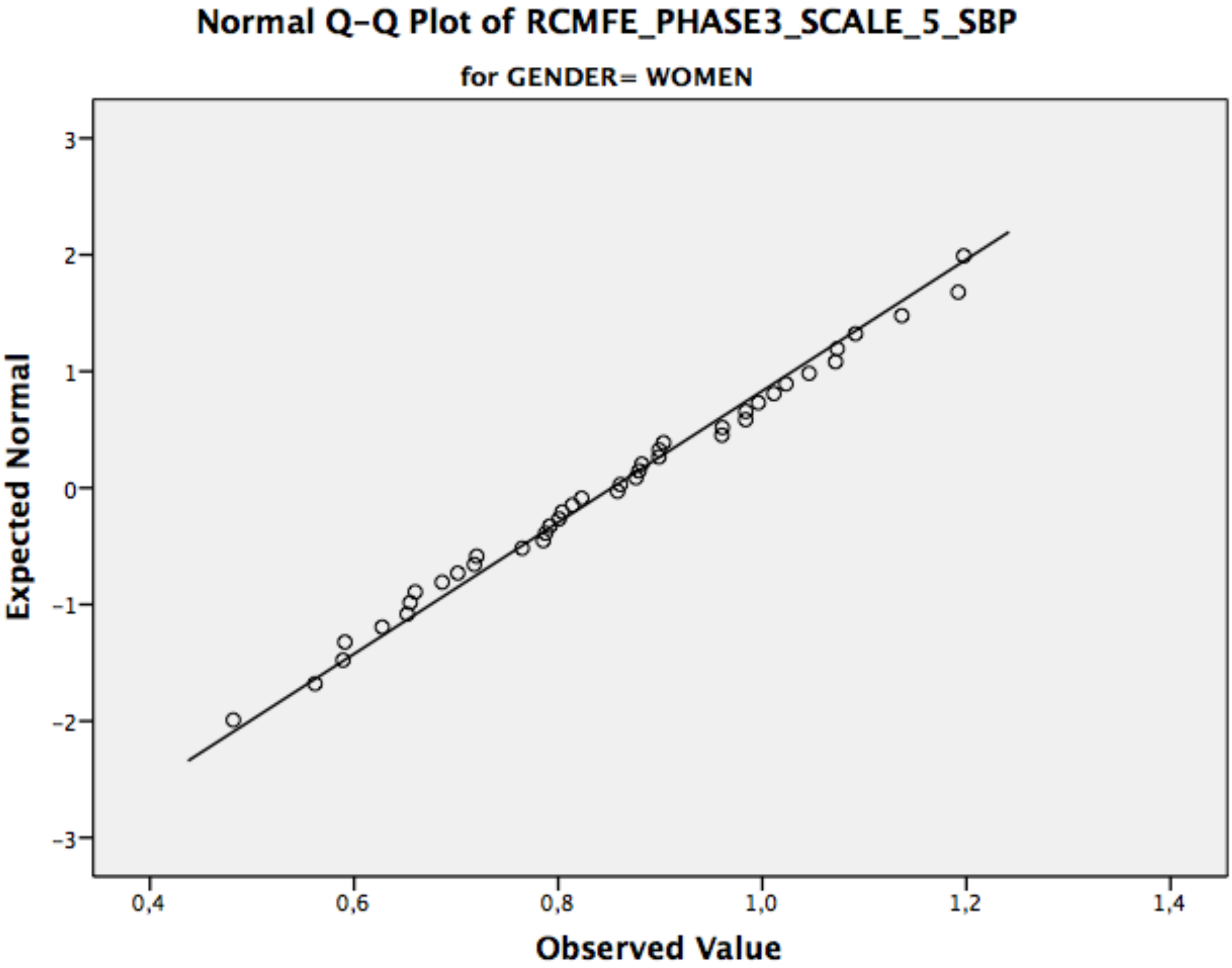

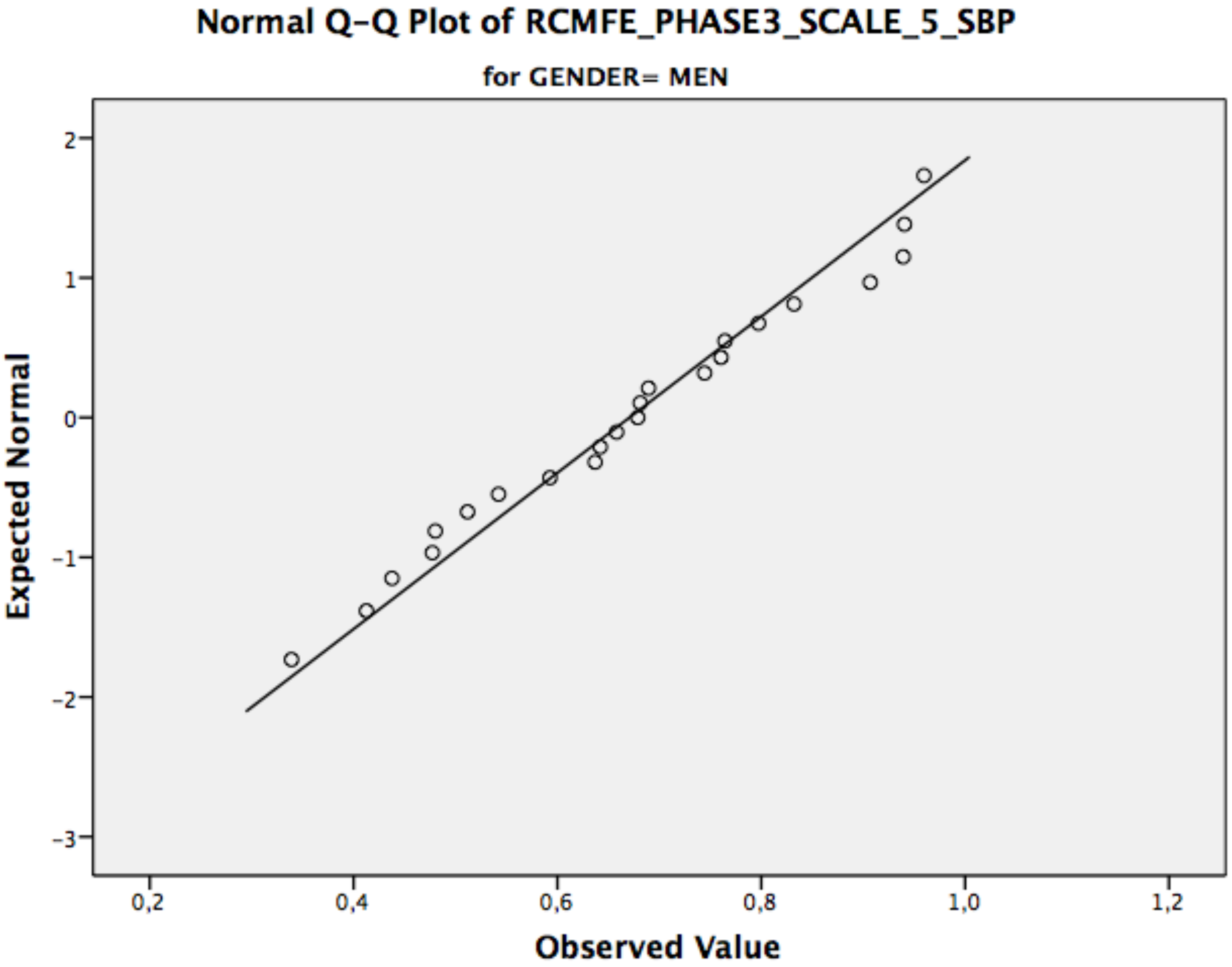

Normal Q-Q Plot of RCMFE_PHASE3_SCALE_5_SBP
for GENDER= MEN
Expected Normal
Observed Value
2
1
0
-1
-2
-3
0,2
0,4
0,6
0,8
1,0
1,2

RCMFE_PHASE4_SCALE_1_SBP

Normal Q-Q Plots

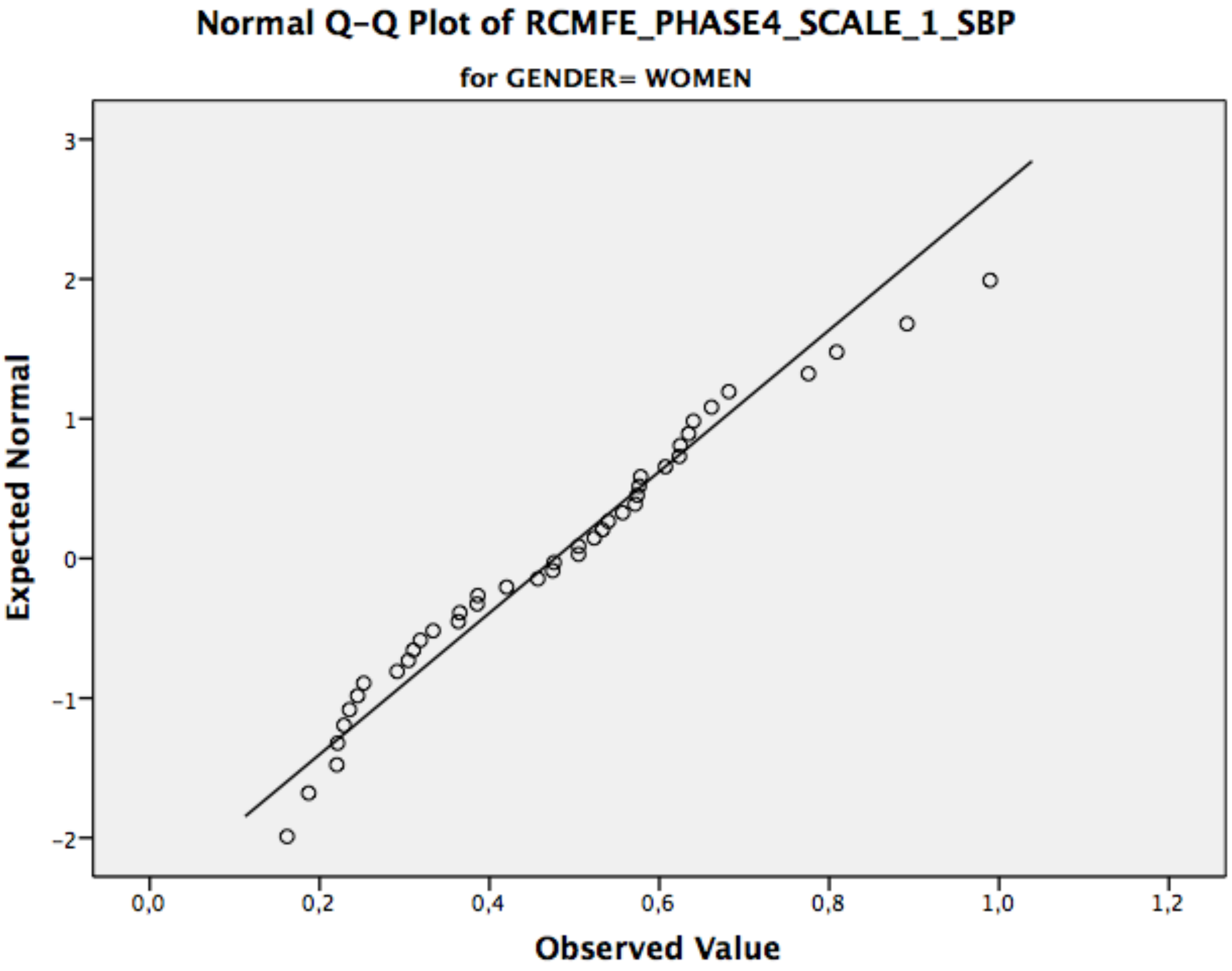

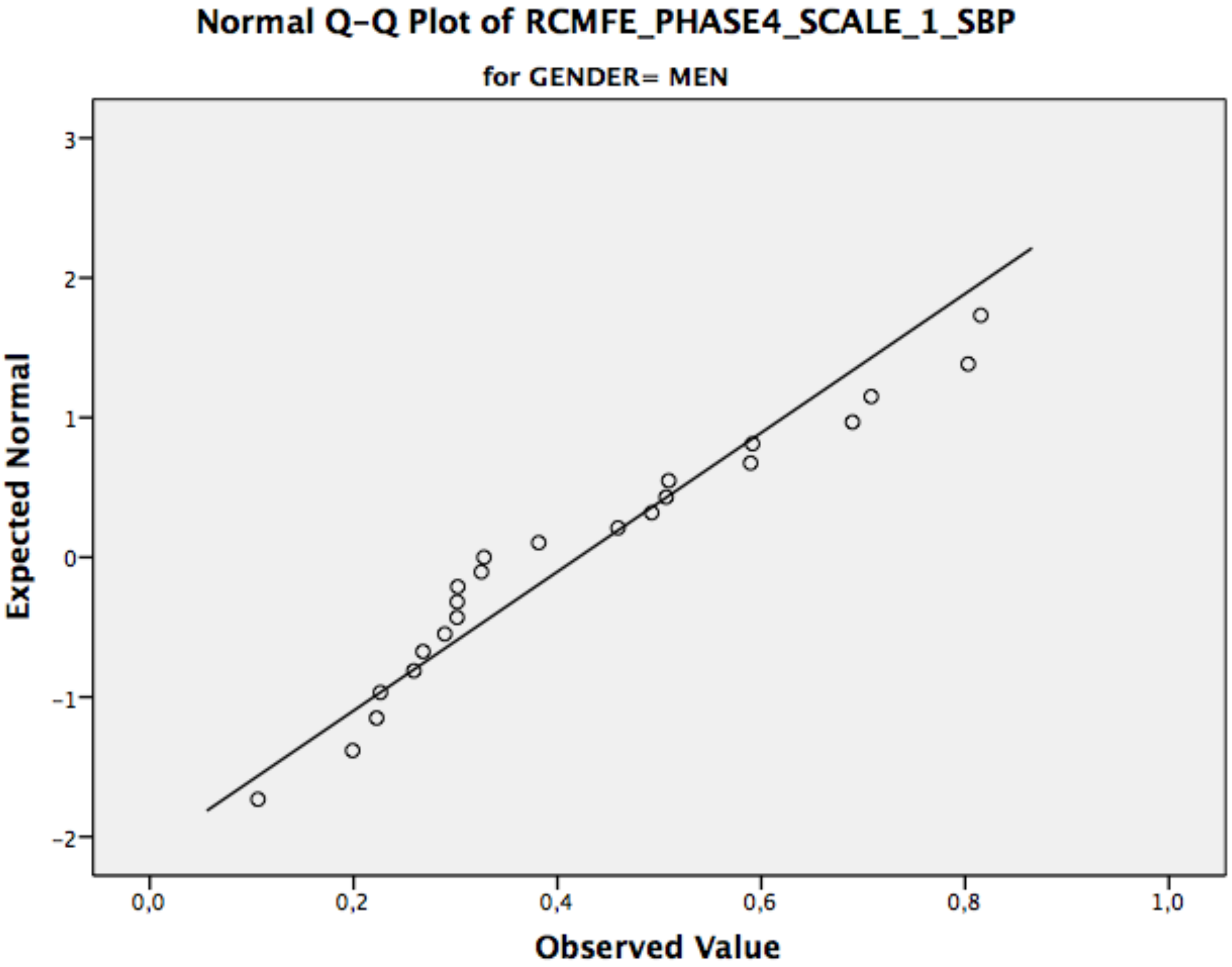

Normal Q-Q Plot of RCMFE_PHASE4_SCALE_1_SBP
for GENDER= MEN
Expected Normal
Observed Value
3
2
1
0
-1
-2
0,0
0,2
0,4
0,6
0,8
1,0

RCMFE_PHASE4_SCALE_2_SBP

Normal Q-Q Plots

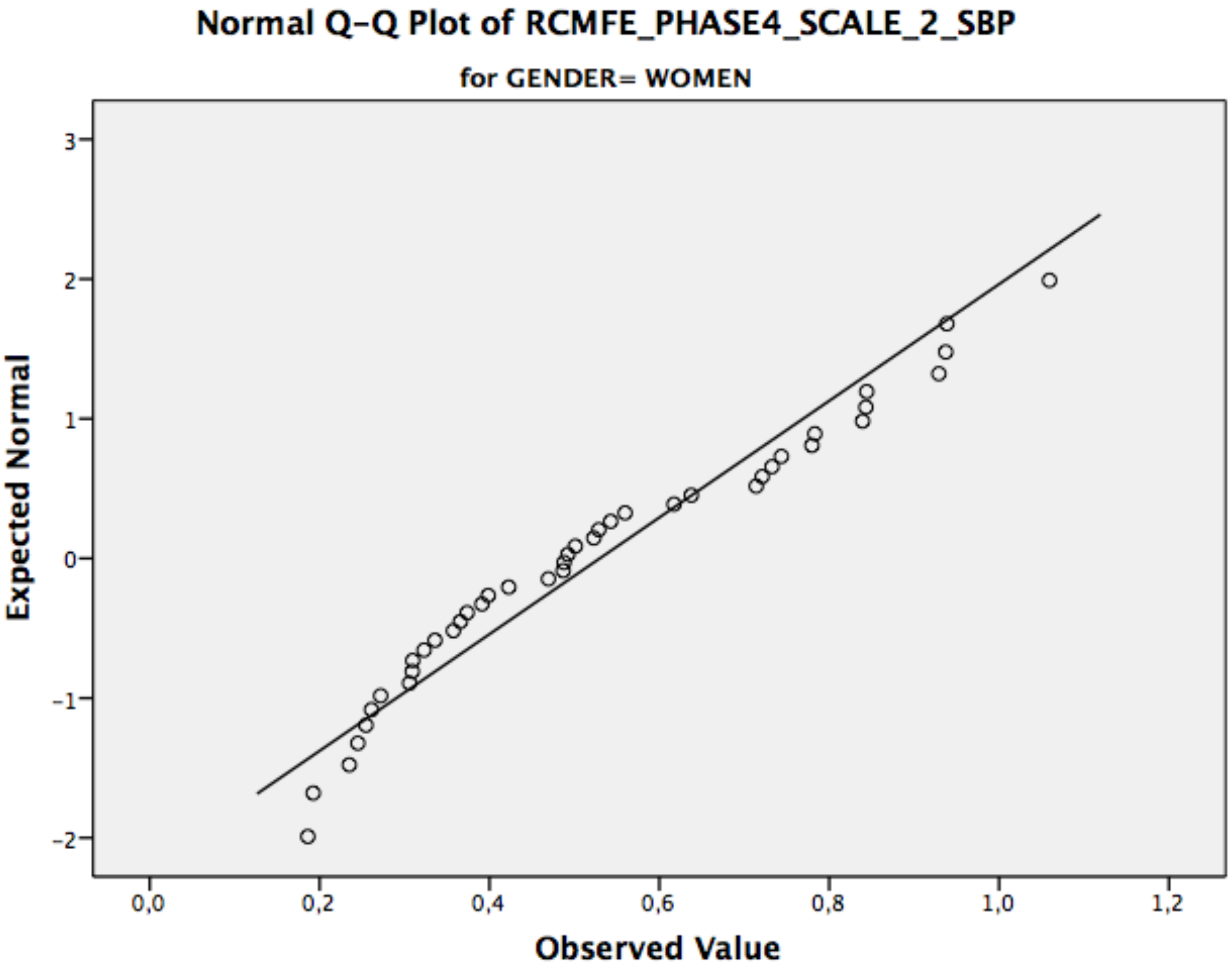

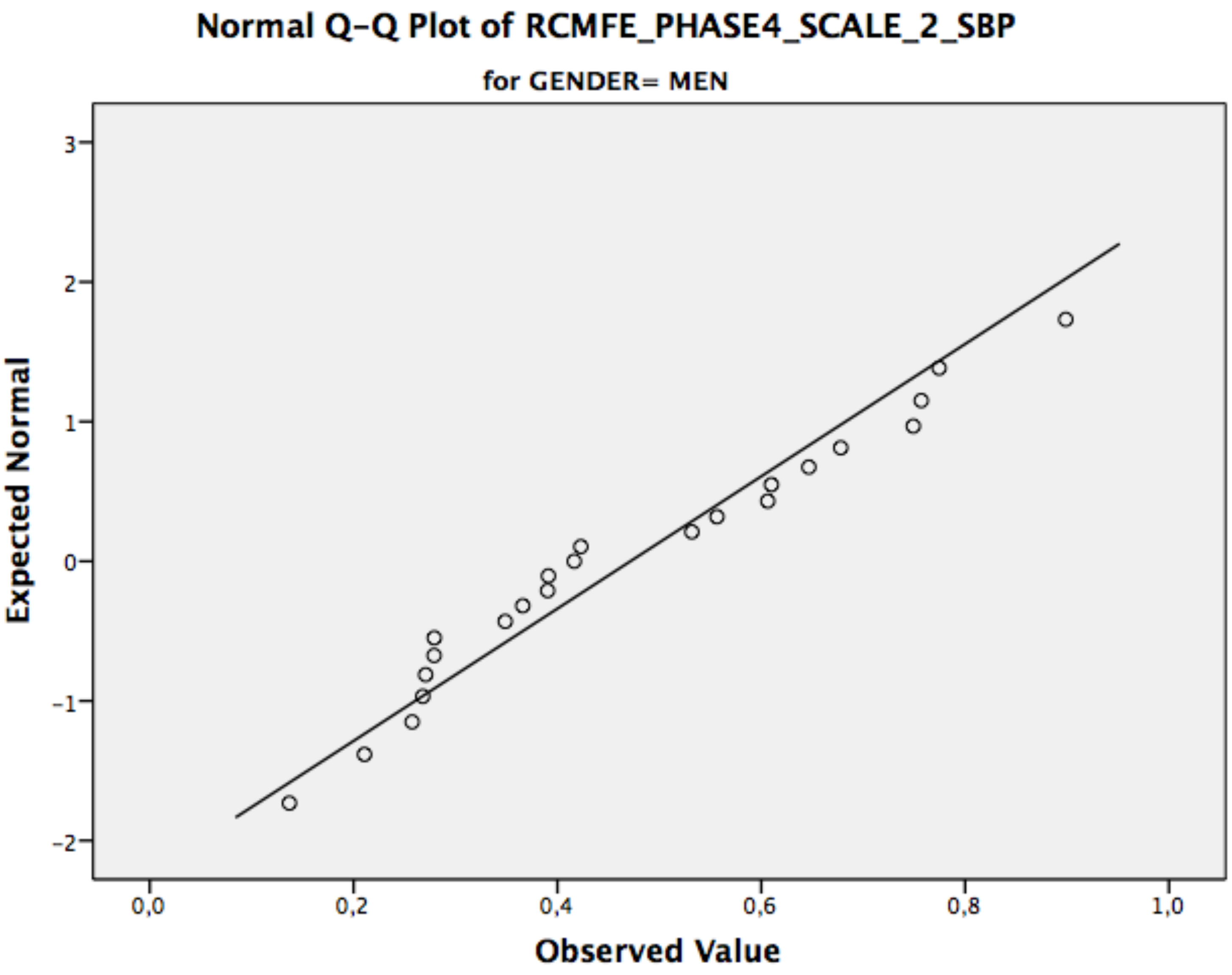
Normal Q-Q Plot of RCMFE_PHASE4_SCALE_2_SBP
for GENDER= MEN
Expected Normal
Observed Value
3
2
1
0
-1
-2
0,0
0,2
0,4
0,6
0,8
1,0

RCMFE_PHASE4_SCALE_3_SBP

Normal Q-Q Plots

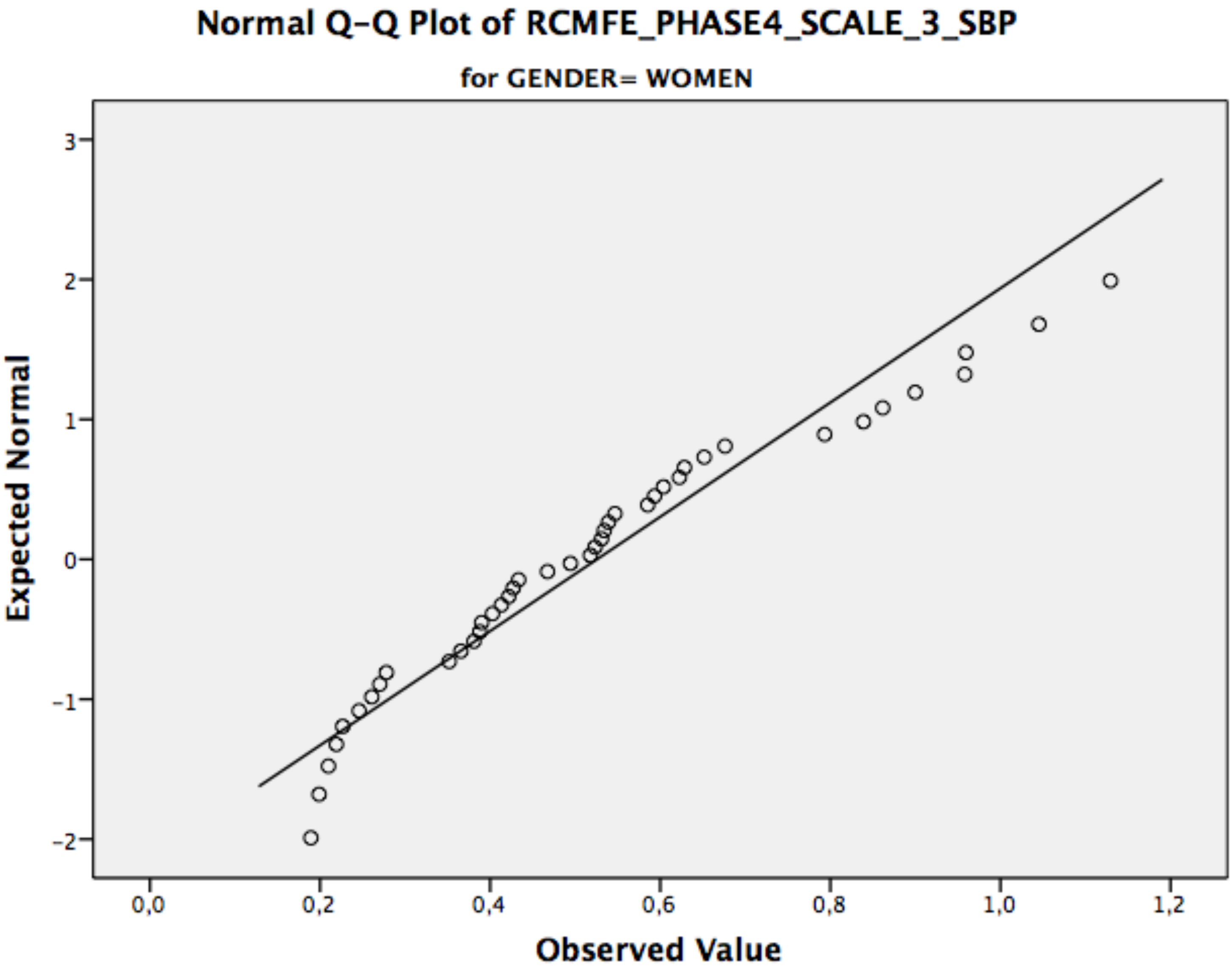

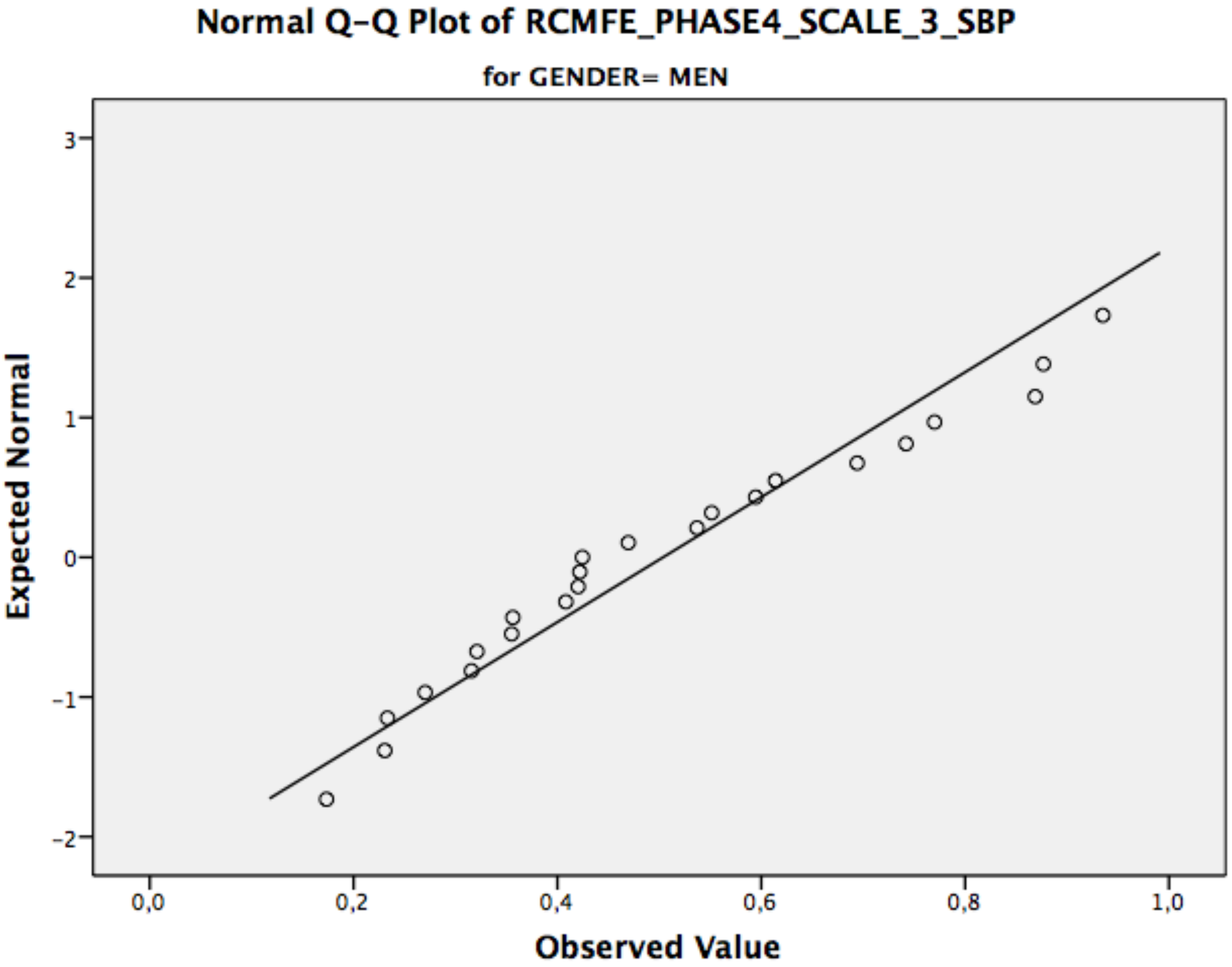

Normal Q-Q Plot of RCMFE_PHASE4_SCALE_3_SBP
for GENDER= MEN
Expected Normal
Observed Value
3
2
1
0
−1
−2
0,0
0,2
0,4
0,6
0,8
1,0

RCMFE_PHASE4_SCALE_4_SBP

Normal Q-Q Plots

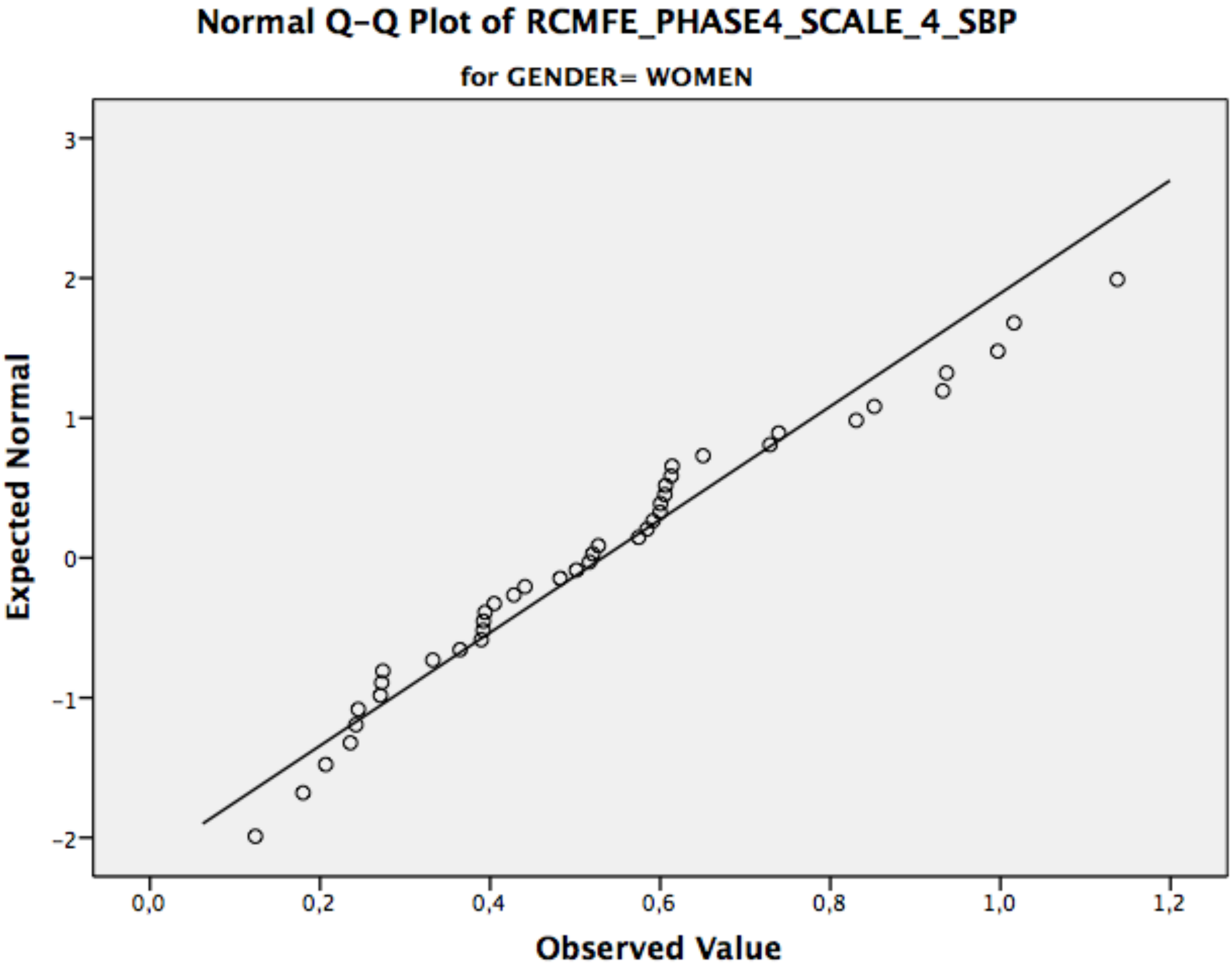

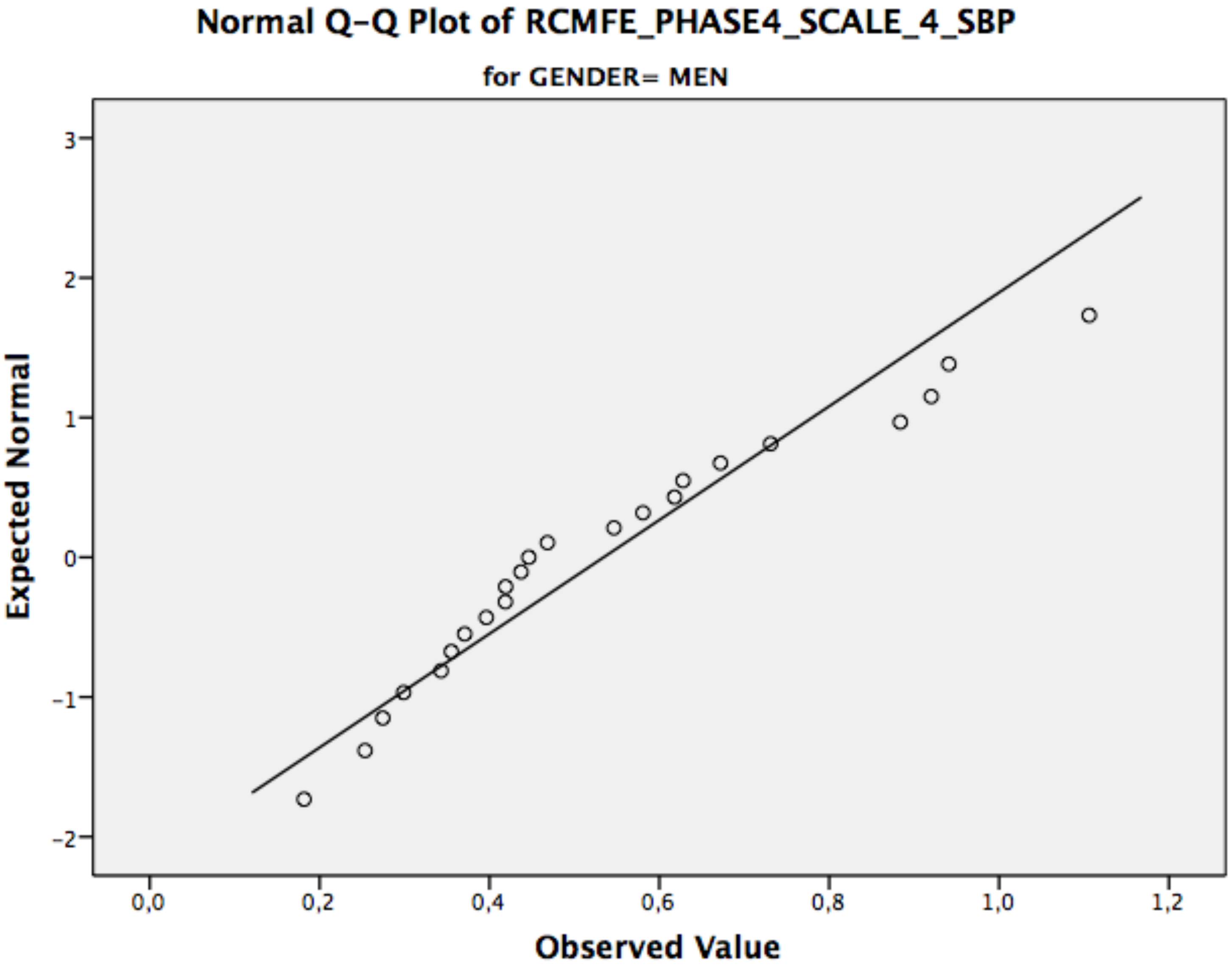
Normal Q-Q Plot of RCMFE_PHASE4_SCALE_4_SBP
for GENDER= MEN
Expected Normal
Observed Value
3
2
1
0
-1
-2
0,0
0,2
0,4
0,6
0,8
1,0
1,2

RCMFE_PHASE4_SCALE_5_SBP

Normal Q-Q Plots

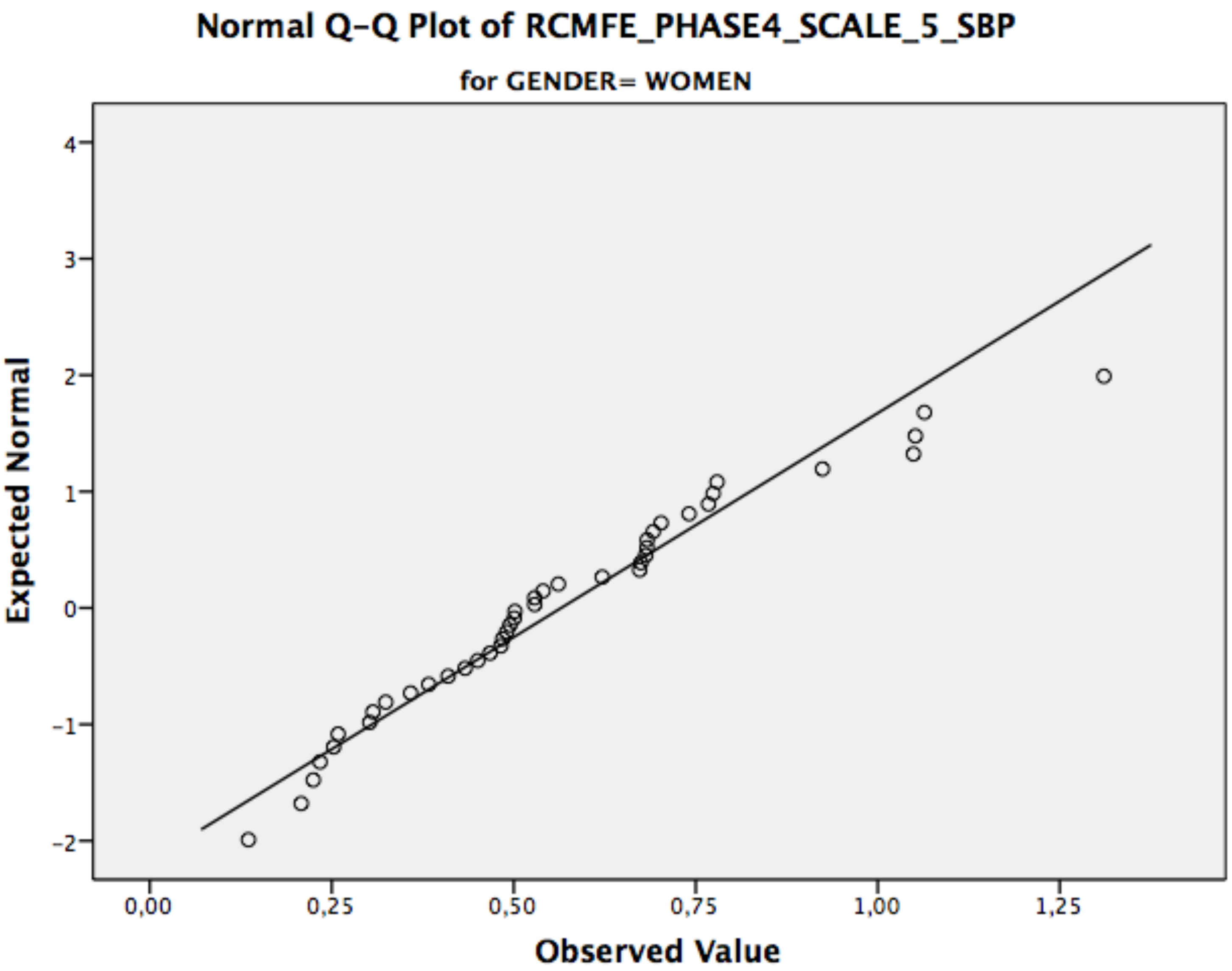

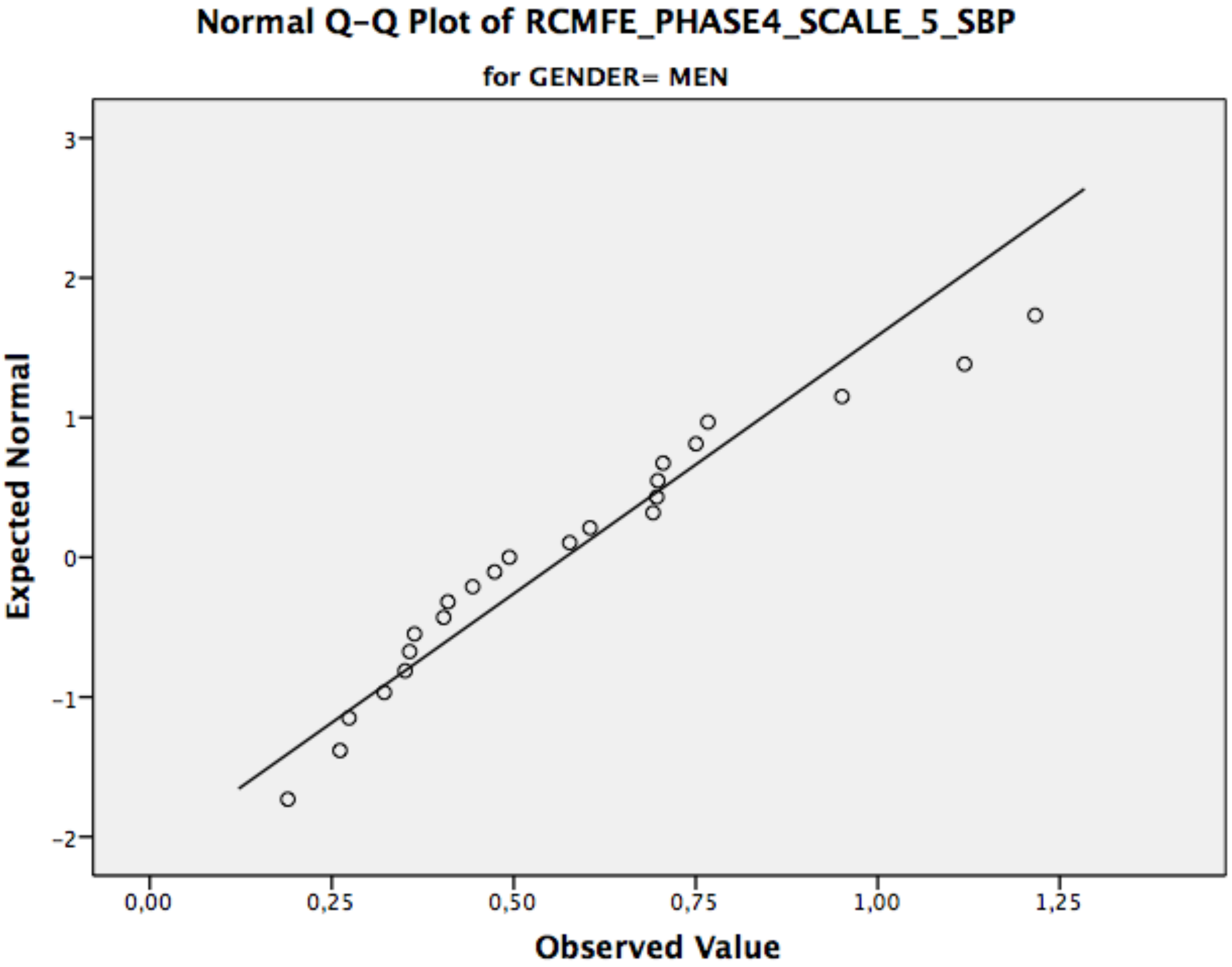
Normal Q-Q Plot of RCMFE_PHASE4_SCALE_5_SBP
for GENDER= MEN
Expected Normal
3
2
1
0
-1
-2
0,00
0,25
0,50
0,75
1,00
1,25
Observed Value

RCMFE_PHASE5_SCALE_1_SBP

Normal Q-Q Plots

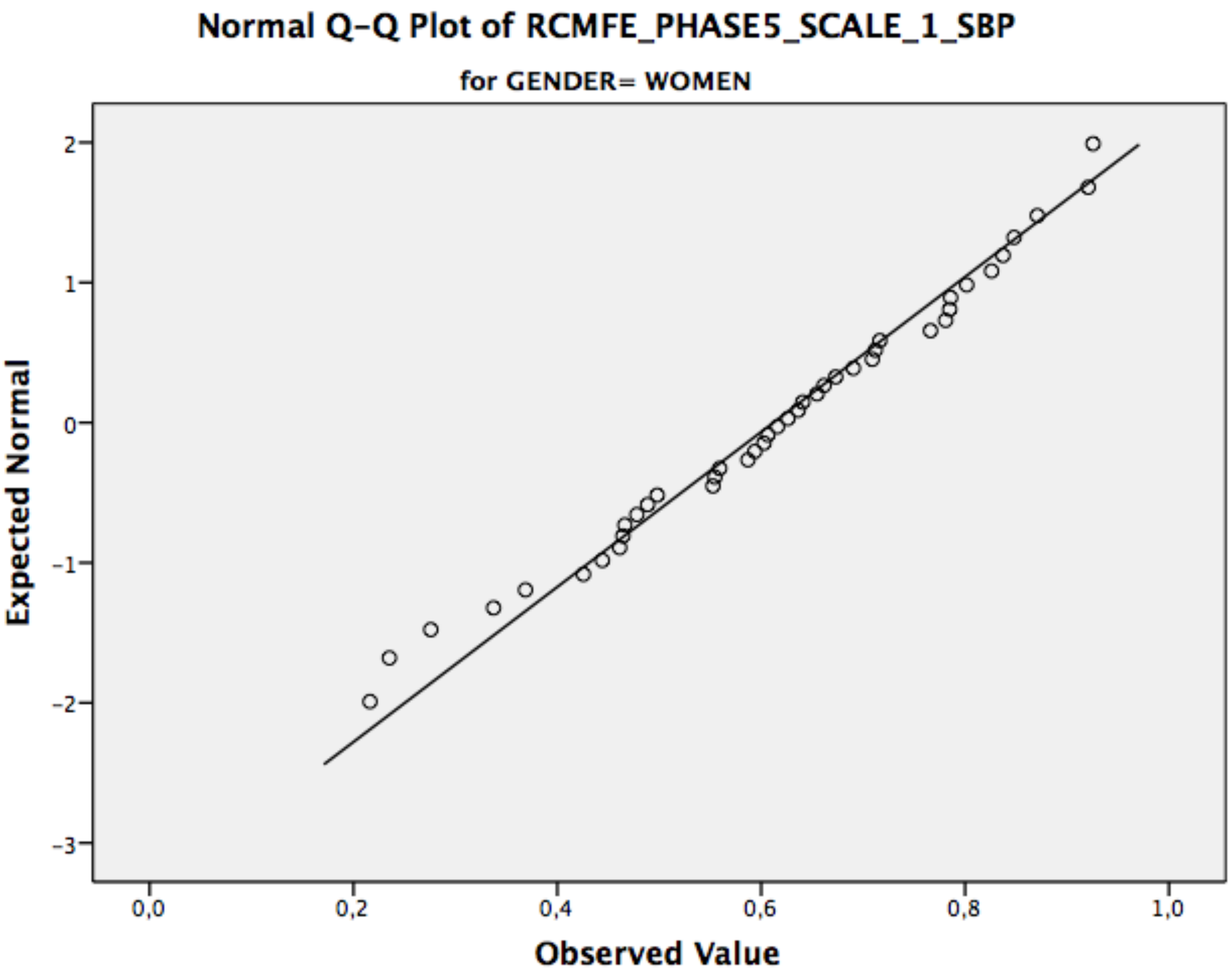

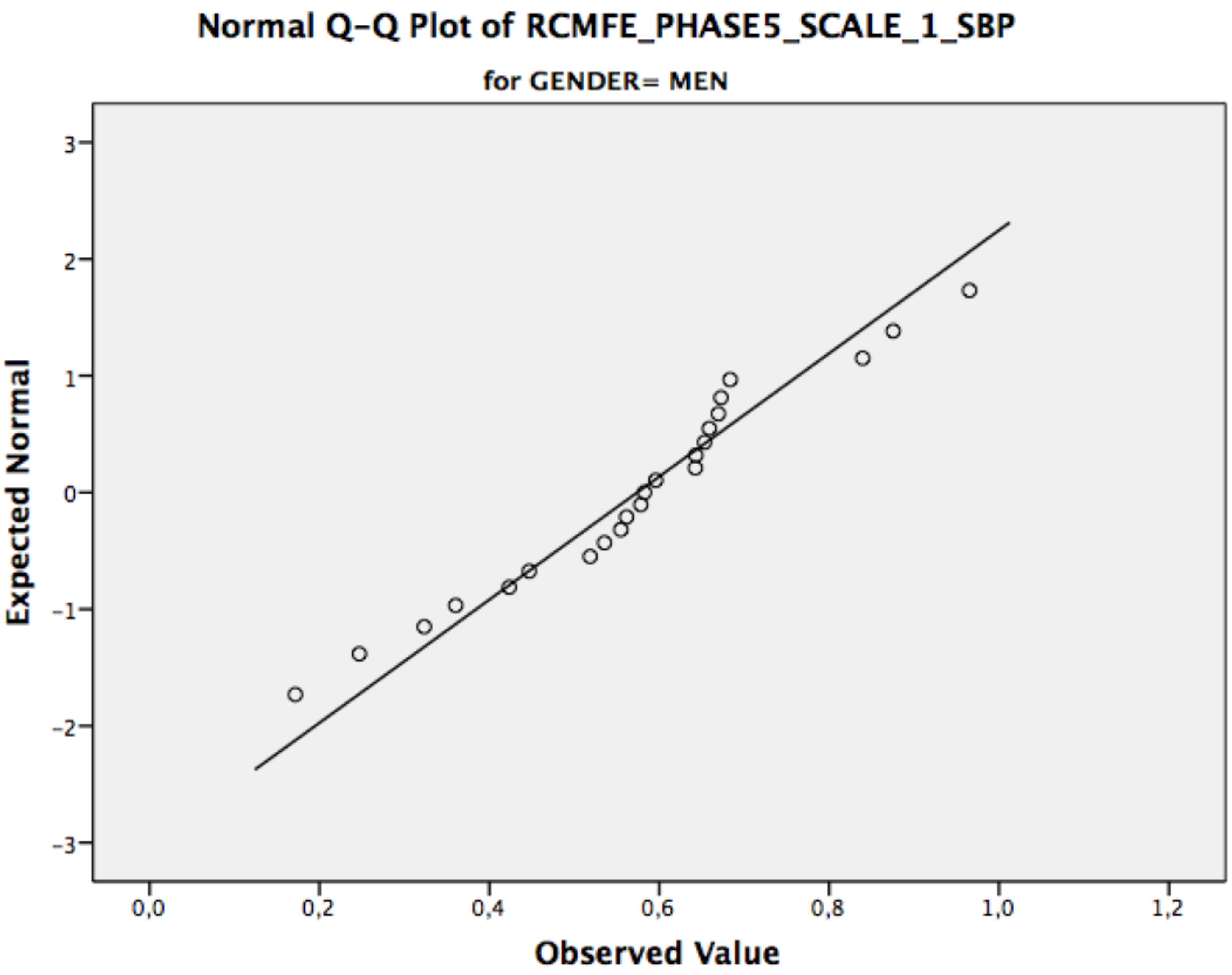
Normal Q-Q Plot of RCMFE_PHASE5_SCALE_1_SBP
for GENDER= MEN
Expected Normal
3
2
1
0
−1
−2
−3
0,0
0,2
0,4
0,6
0,8
1,0
1,2
Observed Value

RCMFE_PHASE5_SCALE_2_SBP

Normal Q-Q Plots

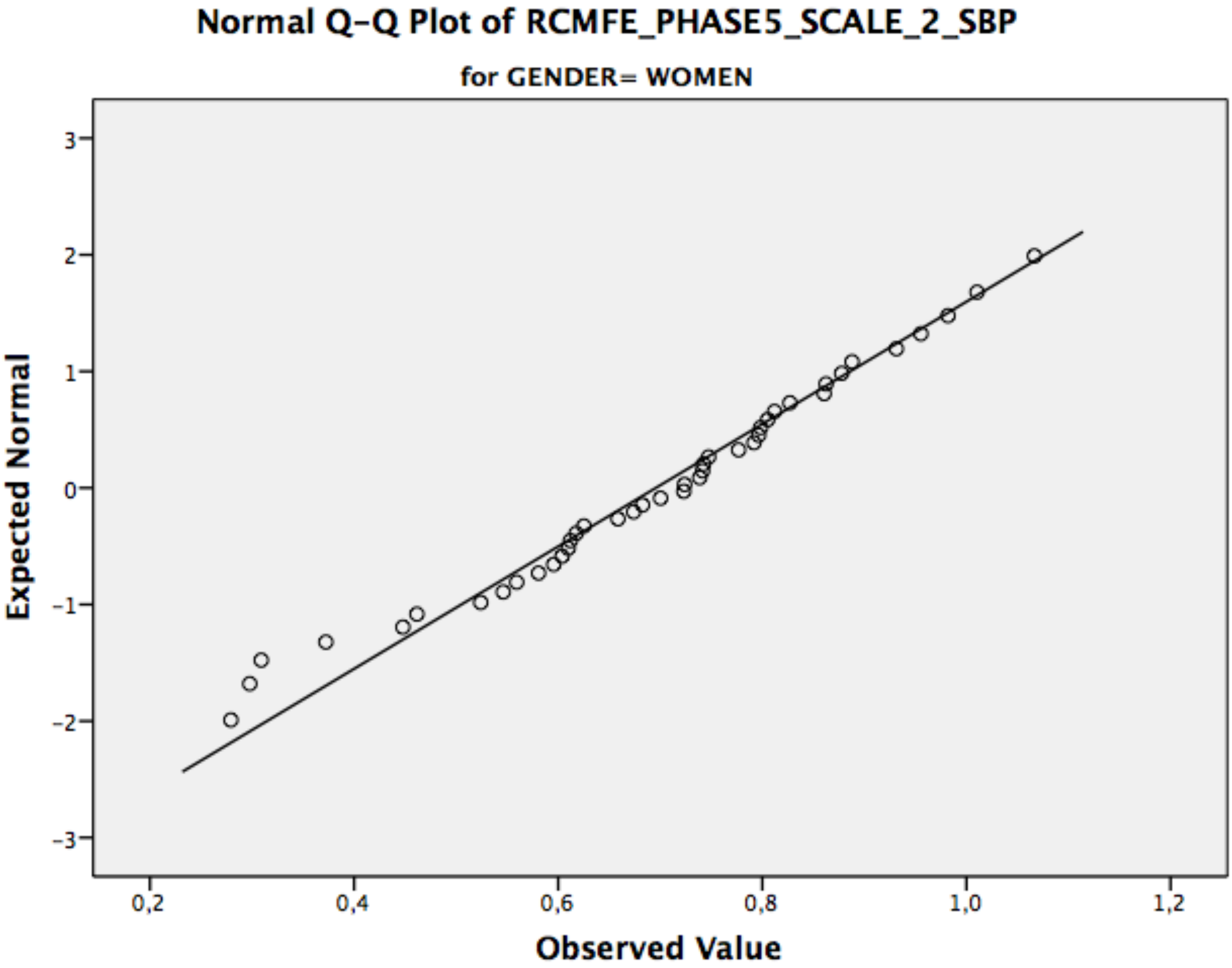

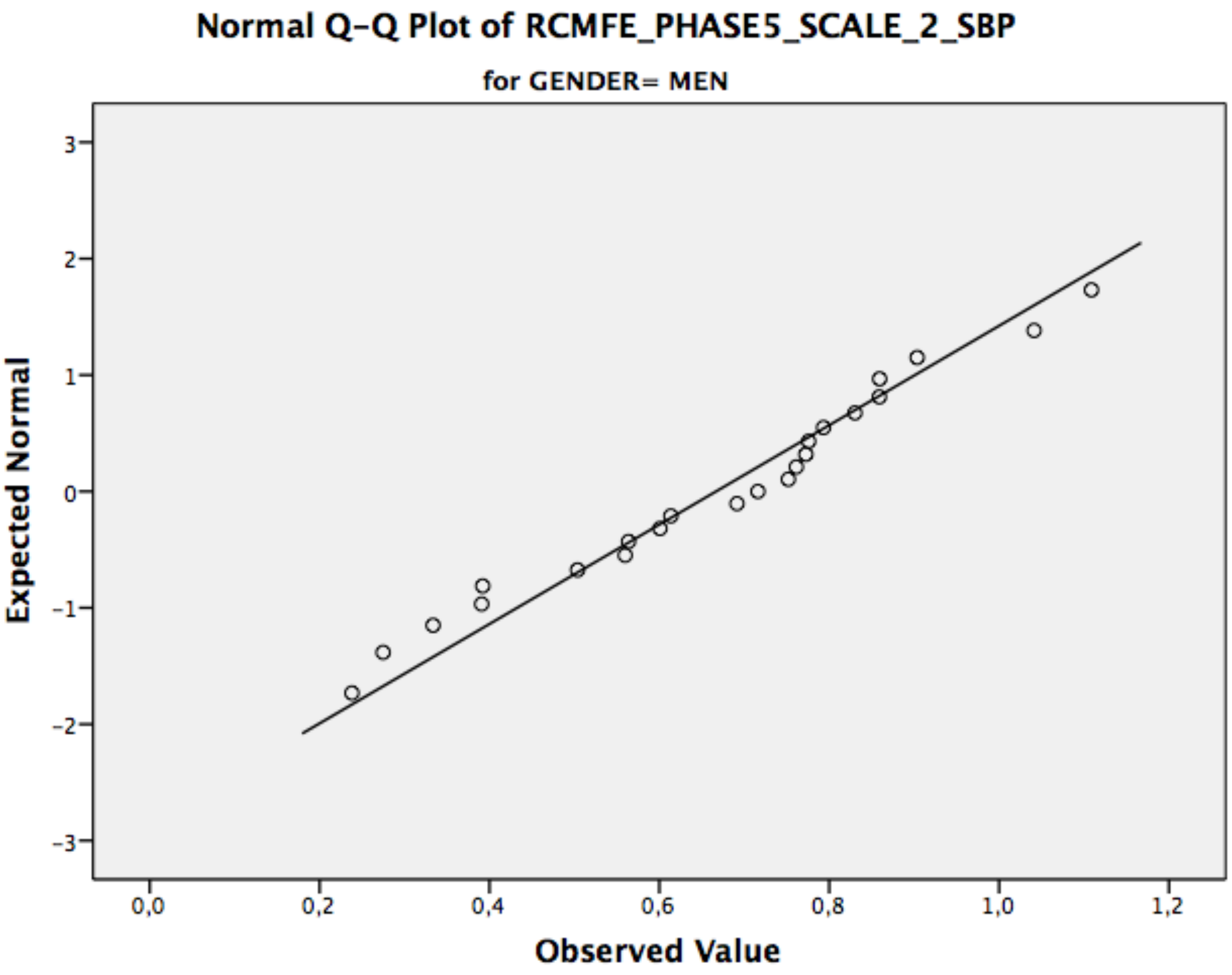
Normal Q-Q Plot of RCMFE_PHASE5_SCALE_2_SBP
for GENDER= MEN
Expected Normal
Observed Value
3
2
1
0
−1
−2
−3
0,0
0,2
0,4
0,6
0,8
1,0
1,2

RCMFE_PHASE5_SCALE_3_SBP

Normal Q-Q Plots

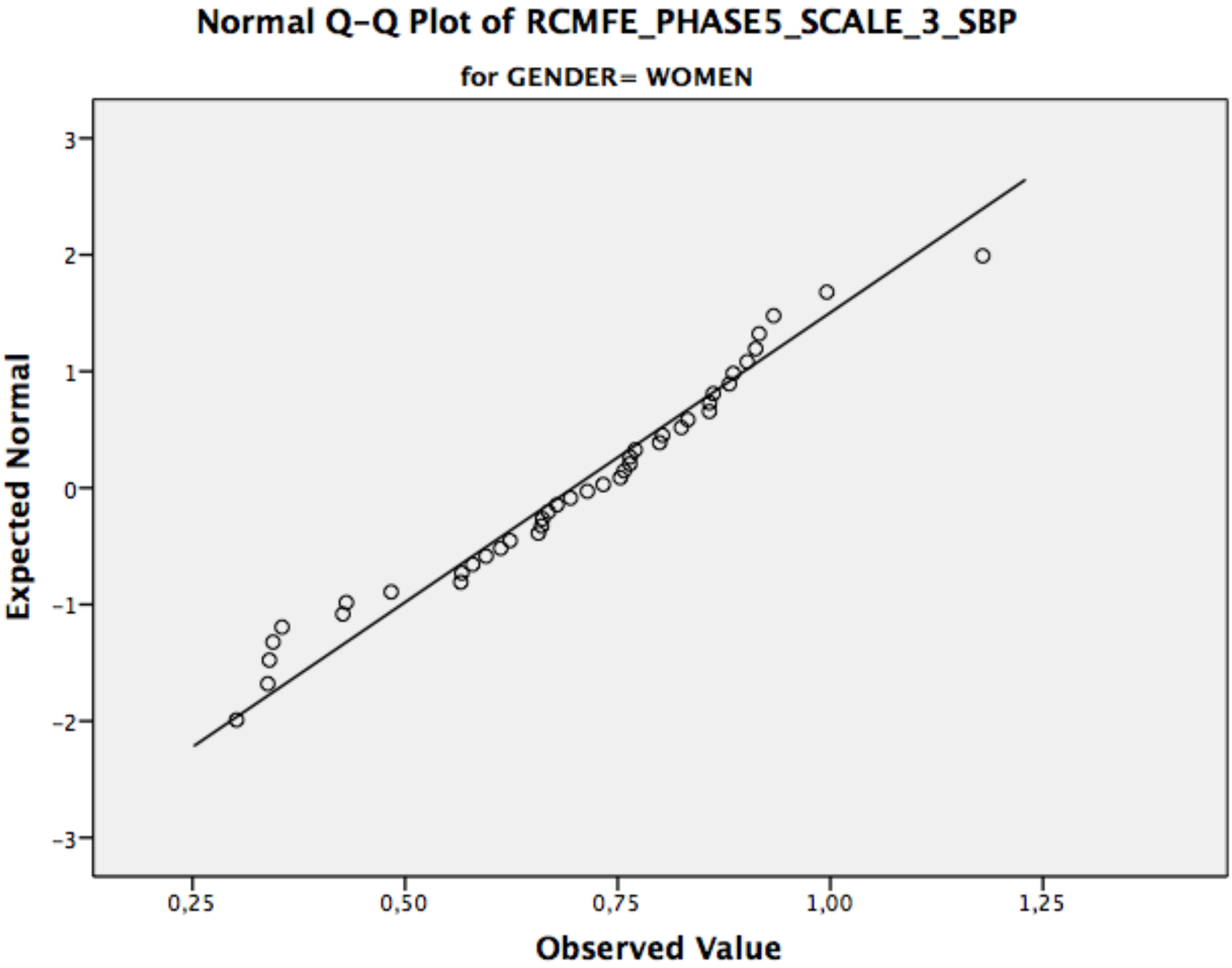

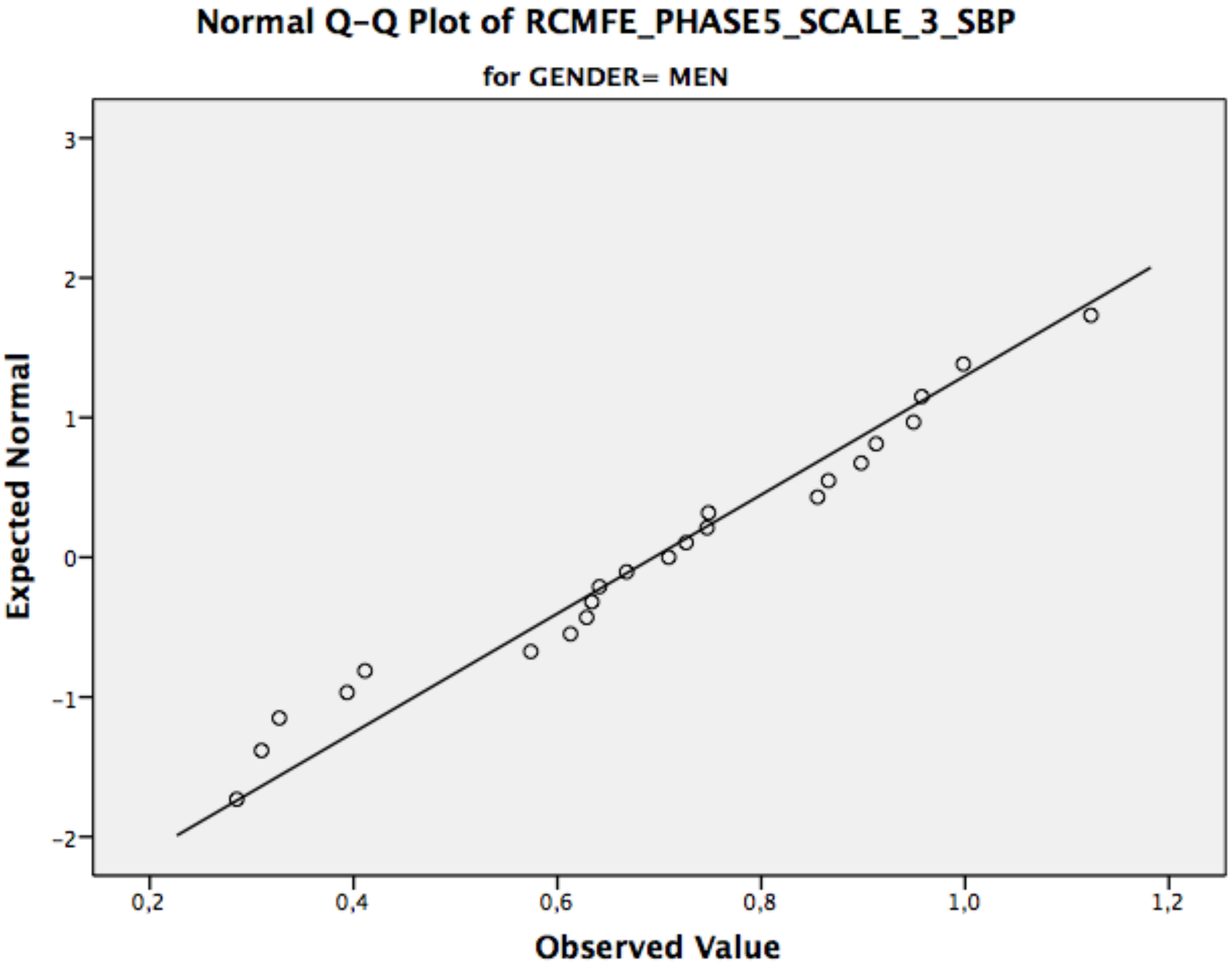
Normal Q-Q Plot of RCMFE_PHASE5_SCALE_3_SBP
for GENDER= MEN
Expected Normal
3
2
1
0
-1
-2
0,2
0,4
0,6
0,8
1,0
1,2
Observed Value

RCMFE_PHASE5_SCALE_4_SBP

Normal Q-Q Plots

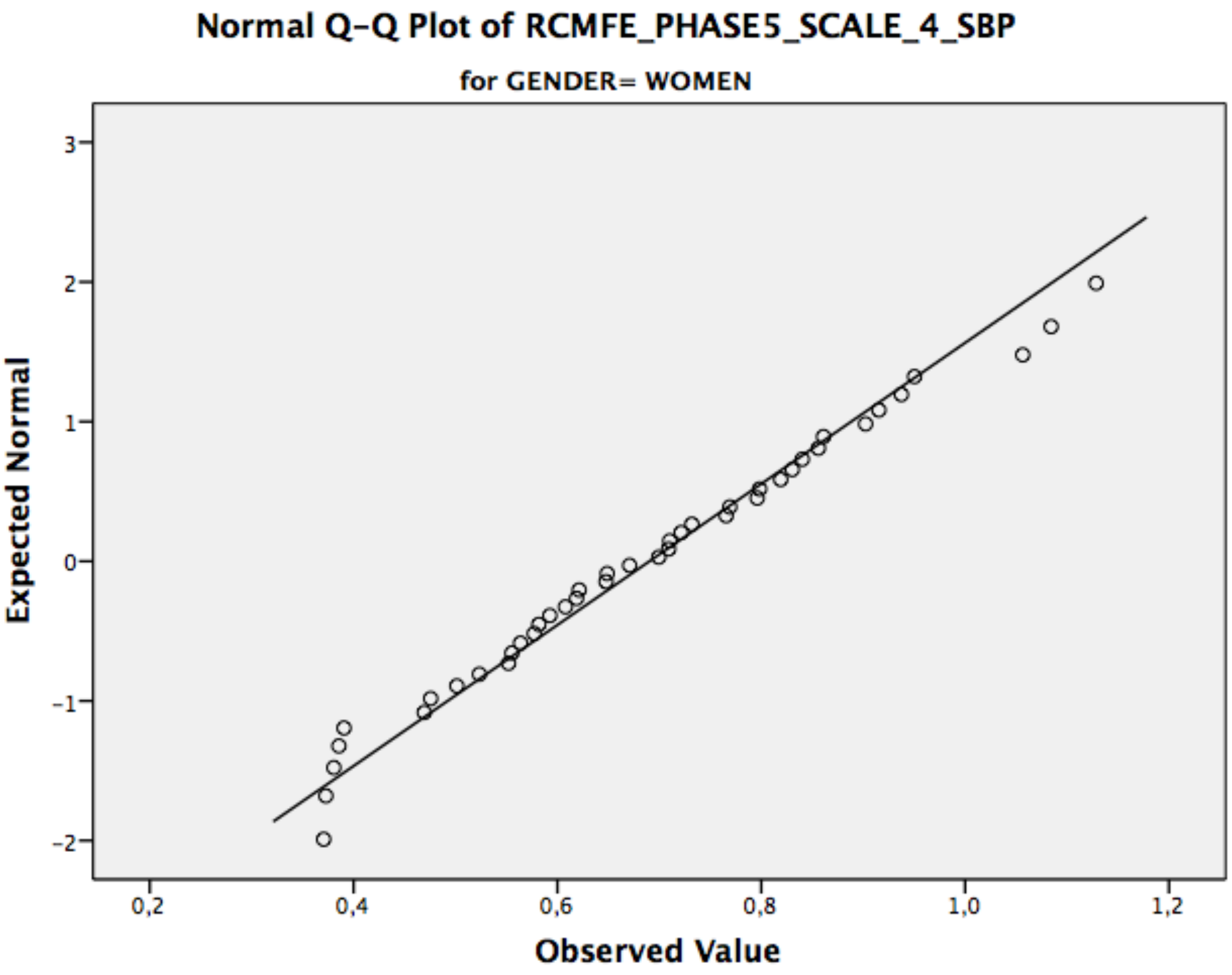

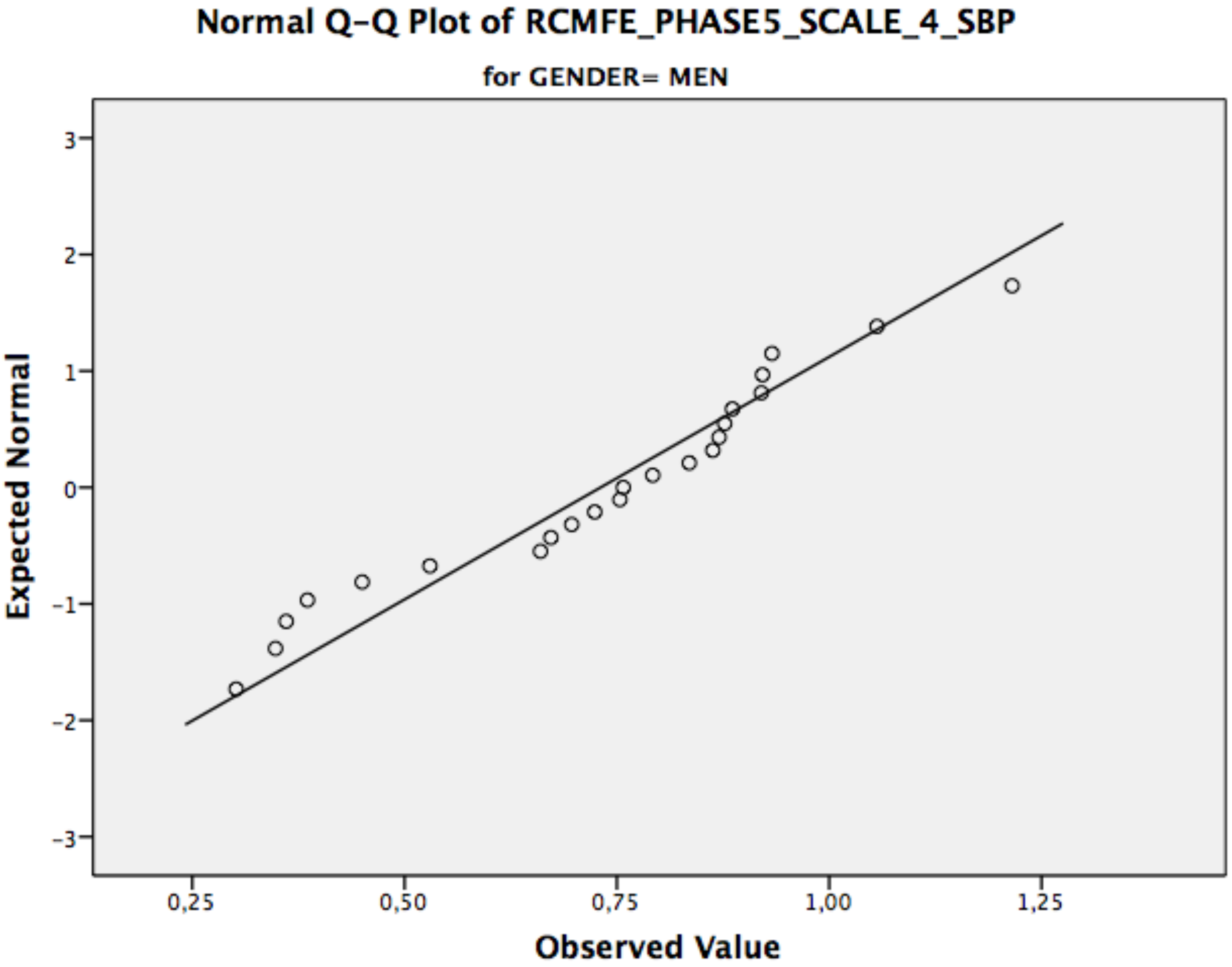

Normal Q-Q Plot of RCMFE_PHASE5_SCALE_4_SBP
for GENDER= MEN
Expected Normal
Observed Value
3
2
1
0
-1
-2
-3
0,25
0,50
0,75
1,00
1,25

RCMFE_PHASE5_SCALE_5_SBP

Normal Q-Q Plots

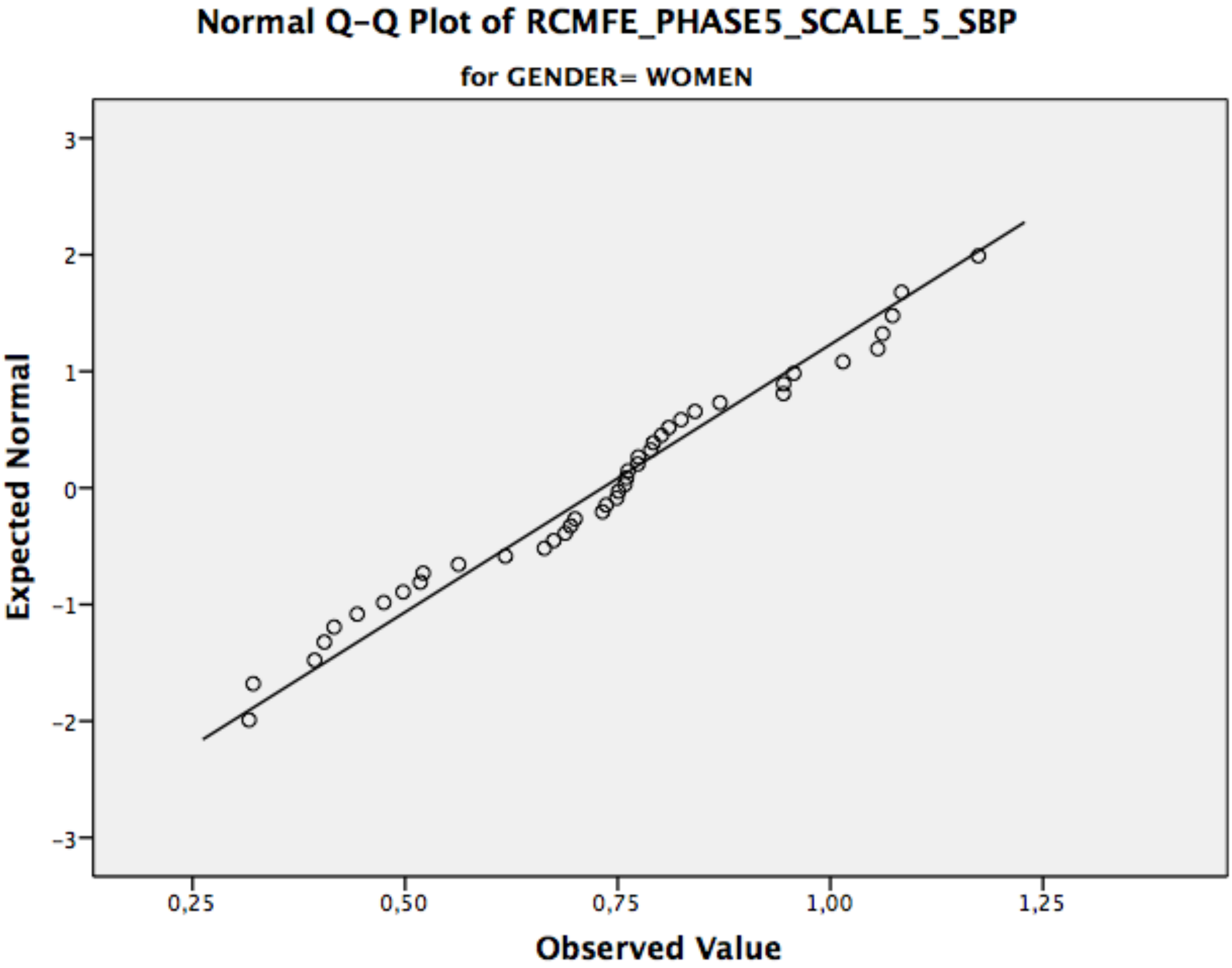

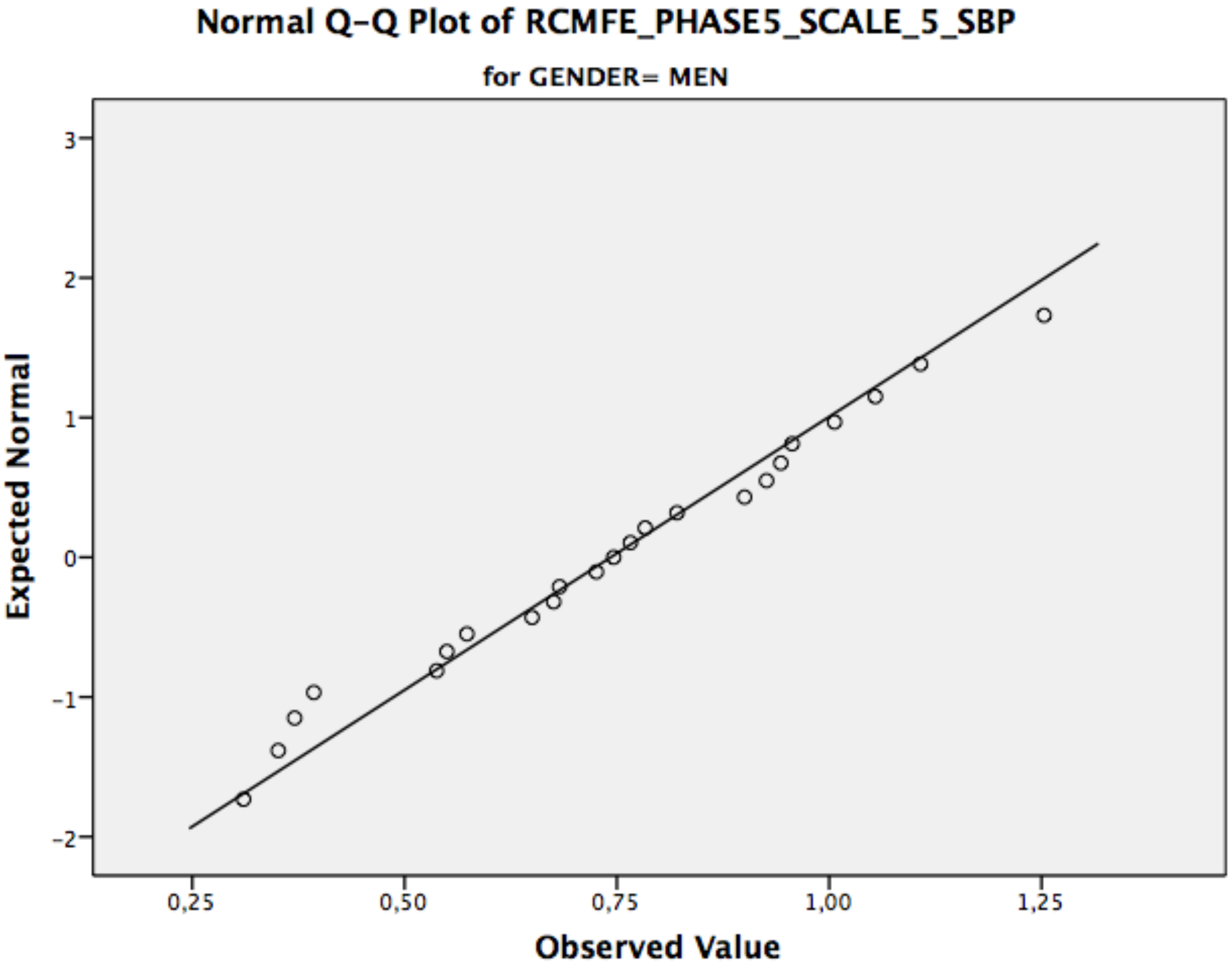

Normal Q-Q Plot of RCMFE_PHASE5_SCALE_5_SBP
for GENDER= MEN
Expected Normal
Observed Value
3
2
1
0
-1
-2
0,25
0,50
0,75
1,00
1,25

Explore SBP

PATHOLOGY

Case Processing Summary

	PATHOLOGY	Cases					
		Valid		Missing		Total	
		N	Percent	N	Percent	N	Percent
RCMFE_PHASE1_SCALE_1_SBP	NO-OI	29	100,0%	0	0,0%	29	100,0%
	OI	36	100,0%	0	0,0%	36	100,0%
RCMFE_PHASE1_SCALE_2_SBP	NO-OI	29	100,0%	0	0,0%	29	100,0%
	OI	36	100,0%	0	0,0%	36	100,0%
RCMFE_PHASE1_SCALE_3_SBP	NO-OI	29	100,0%	0	0,0%	29	100,0%
	OI	36	100,0%	0	0,0%	36	100,0%
RCMFE_PHASE1_SCALE_4_SBP	NO-OI	29	100,0%	0	0,0%	29	100,0%
	OI	36	100,0%	0	0,0%	36	100,0%
RCMFE_PHASE1_SCALE_5_SBP	NO-OI	29	100,0%	0	0,0%	29	100,0%
	OI	36	100,0%	0	0,0%	36	100,0%
RCMFE_PHASE2_SCALE_1_SBP	NO-OI	29	100,0%	0	0,0%	29	100,0%
	OI	36	100,0%	0	0,0%	36	100,0%
RCMFE_PHASE2_SCALE_2_SBP	NO-OI	29	100,0%	0	0,0%	29	100,0%
	OI	36	100,0%	0	0,0%	36	100,0%
RCMFE_PHASE2_SCALE_3_SBP	NO-OI	29	100,0%	0	0,0%	29	100,0%
	OI	36	100,0%	0	0,0%	36	100,0%
RCMFE_PHASE2_SCALE_4_SBP	NO-OI	29	100,0%	0	0,0%	29	100,0%
	OI	36	100,0%	0	0,0%	36	100,0%
RCMFE_PHASE2_SCALE_5_SBP	NO-OI	29	100,0%	0	0,0%	29	100,0%
	OI	36	100,0%	0	0,0%	36	100,0%
RCMFE_PHASE3_SCALE_1_SBP	NO-OI	29	100,0%	0	0,0%	29	100,0%
	OI	36	100,0%	0	0,0%	36	100,0%
RCMFE_PHASE3_SCALE_2_SBP	NO-OI	29	100,0%	0	0,0%	29	100,0%
	OI	36	100,0%	0	0,0%	36	100,0%
RCMFE_PHASE3_SCALE_3_SBP	NO-OI	29	100,0%	0	0,0%	29	100,0%
	OI	36	100,0%	0	0,0%	36	100,0%
RCMFE_PHASE3_SCALE_4_SBP	NO-OI	29	100,0%	0	0,0%	29	100,0%
	OI	36	100,0%	0	0,0%	36	100,0%
RCMFE_PHASE3_SCALE_5_SBP	NO-OI	29	100,0%	0	0,0%	29	100,0%
	OI	36	100,0%	0	0,0%	36	100,0%
RCMFE_PHASE4_SCALE_1_SBP	NO-OI	29	100,0%	0	0,0%	29	100,0%
	OI	36	100,0%	0	0,0%	36	100,0%
RCMFE_PHASE4_SCALE_2_SBP	NO-OI	29	100,0%	0	0,0%	29	100,0%
	OI	36	100,0%	0	0,0%	36	100,0%
RCMFE_PHASE4_SCALE_3	NO-OI	29	100,0%	0	0,0%	29	100,0%
	OI	36	100,0%	0	0,0%	36	100,0%
RCMFE_PHASE4_SCALE_4_SBP	NO-OI	29	100,0%	0	0,0%	29	100,0%
	OI	36	100,0%	0	0,0%	36	100,0%
RCMFE_PHASE4_SCALE_5	NO-OI	29	100,0%	0	0,0%	29	100,0%

_SBP	OI	36	100,0%	0	0,0%	36	100,0%
RCMFE_PHASE5_SCALE_1 _SBP	NO-OI	29	100,0%	0	0,0%	29	100,0%

Case Processing Summary

	PATHOLOGY	Cases					
		Valid		Missing		Total	
		N	Percent	N	Percent	N	Percent
RCMFE_PHASE5_SCALE_1 _SBP	OI	36	100,0%	0	0,0%	36	100,0%
RCMFE_PHASE5_SCALE_2	NO-OI	29	100,0%	0	0,0%	29	100,0%
_SBP	OI	36	100,0%	0	0,0%	36	100,0%
RCMFE_PHASE5_SCALE_3	NO-OI	29	100,0%	0	0,0%	29	100,0%
_SBP	OI	36	100,0%	0	0,0%	36	100,0%
RCMFE_PHASE5_SCALE_4	NO-OI	29	100,0%	0	0,0%	29	100,0%
_SBP	OI	36	100,0%	0	0,0%	36	100,0%
RCMFE_PHASE5_SCALE_5	NO-OI	29	100,0%	0	0,0%	29	100,0%
_SBP	OI	36	100,0%	0	0,0%	36	100,0%

Tests of Normality

	PATHOLOGY	Kolmogorov-Smirnov[a]			Shapiro-Wilk		
		Statistic	df	Sig.	Statistic	df	Sig.
RCMFE_PHASE1_SCALE_1	NO-OI	,149	29	,100	,953	29	,214
_SBP	OI	,095	36	,200*	,974	36	,557
RCMFE_PHASE1_SCALE_2	NO-OI	,064	29	,200*	,990	29	,994
_SBP	OI	,079	36	,200*	,974	36	,538
RCMFE_PHASE1_SCALE_3	NO-OI	,135	29	,189	,965	29	,437
_SBP	OI	,071	36	,200*	,974	36	,541
RCMFE_PHASE1_SCALE_4	NO-OI	,148	29	,106	,948	29	,162
_SBP	OI	,099	36	,200*	,974	36	,546
RCMFE_PHASE1_SCALE_5	NO-OI	,086	29	,200*	,984	29	,917
_SBP	OI	,076	36	,200*	,986	36	,923
RCMFE_PHASE2_SCALE_1	NO-OI	,085	29	,200*	,961	29	,341
_SBP	OI	,081	36	,200*	,981	36	,791
RCMFE_PHASE2_SCALE_2	NO-OI	,126	29	,200*	,973	29	,635
_SBP	OI	,071	36	,200*	,982	36	,812
RCMFE_PHASE2_SCALE_3	NO-OI	,127	29	,200*	,975	29	,705
_SBP	OI	,106	36	,200*	,962	36	,256
RCMFE_PHASE2_SCALE_4	NO-OI	,103	29	,200*	,977	29	,762
_SBP	OI	,098	36	,200*	,962	36	,253
RCMFE_PHASE2_SCALE_5	NO-OI	,151	29	,089	,945	29	,134
_SBP	OI	,113	36	,200*	,962	36	,254
RCMFE_PHASE3_SCALE_1	NO-OI	,090	29	,200*	,962	29	,363
_SBP	OI	,112	36	,200*	,951	36	,115
RCMFE_PHASE3_SCALE_2	NO-OI	,082	29	,200*	,980	29	,845
_SBP	OI	,135	36	,093	,980	36	,760
RCMFE_PHASE3_SCALE_3	NO-OI	,143	29	,132	,953	29	,218

_SBP	OI	,125	36	,167	,967	36	,359
RCMFE_PHASE3_SCALE_4	NO-OI	,173	29	,027	,953	29	,220
_SBP	OI	,061	36	,200*	,977	36	,632
RCMFE_PHASE3_SCALE_5	NO-OI	,117	29	,200*	,966	29	,468
_SBP	OI	,125	36	,166	,968	36	,371
RCMFE_PHASE4_SCALE_1	NO-OI	,109	29	,200*	,973	29	,639
_SBP	OI	,130	36	,128	,941	36	,056
RCMFE_PHASE4_SCALE_2	NO-OI	,145	29	,122	,951	29	,198
_SBP	OI	,155	36	,029	,936	36	,058
RCMFE_PHASE4_SCALE_3	NO-OI	,159	29	,058	,928	29	,048
	OI	,116	36	,200*	,947	36	,084
RCMFE_PHASE4_SCALE_4	NO-OI	,164	29	,044	,949	29	,175
_SBP	OI	,129	36	,137	,949	36	,097
RCMFE_PHASE4_SCALE_5	NO-OI	,155	29	,073	,937	29	,085
_SBP	OI	,112	36	,200*	,956	36	,167
RCMFE_PHASE5_SCALE_1	NO-OI	,085	29	,200*	,970	29	,553
_SBP	OI	,104	36	,200*	,973	36	,507

Tests of Normality

	PATHOLOGY	Kolmogorov-Smirnov[a]			Shapiro-Wilk		
		Statistic	df	Sig.	Statistic	df	Sig.
RCMFE_PHASE5_SCALE_2	NO-OI	,179	29	,019	,921	29	,052
_SBP	OI	,088	36	,200*	,973	36	,505
RCMFE_PHASE5_SCALE_3	NO-OI	,150	29	,094*	,920	29	,031
_SBP	OI	,080	36	,200*	,992	36	,994
RCMFE_PHASE5_SCALE_4	NO-OI	,140	29	,153	,940	29	,102
_SBP	OI	,069	36	,200*	,990	36	,978
RCMFE_PHASE5_SCALE_5	NO-OI	,127	29	,200	,916	29	,025
_SBP	OI	,160	36	,020*	,965	36	,308

*. This is a lower bound of the true significance.

a. Lilliefors Significance Correction

RCMFE_PHASE1_SCALE_1_SBP

Normal Q-Q Plots

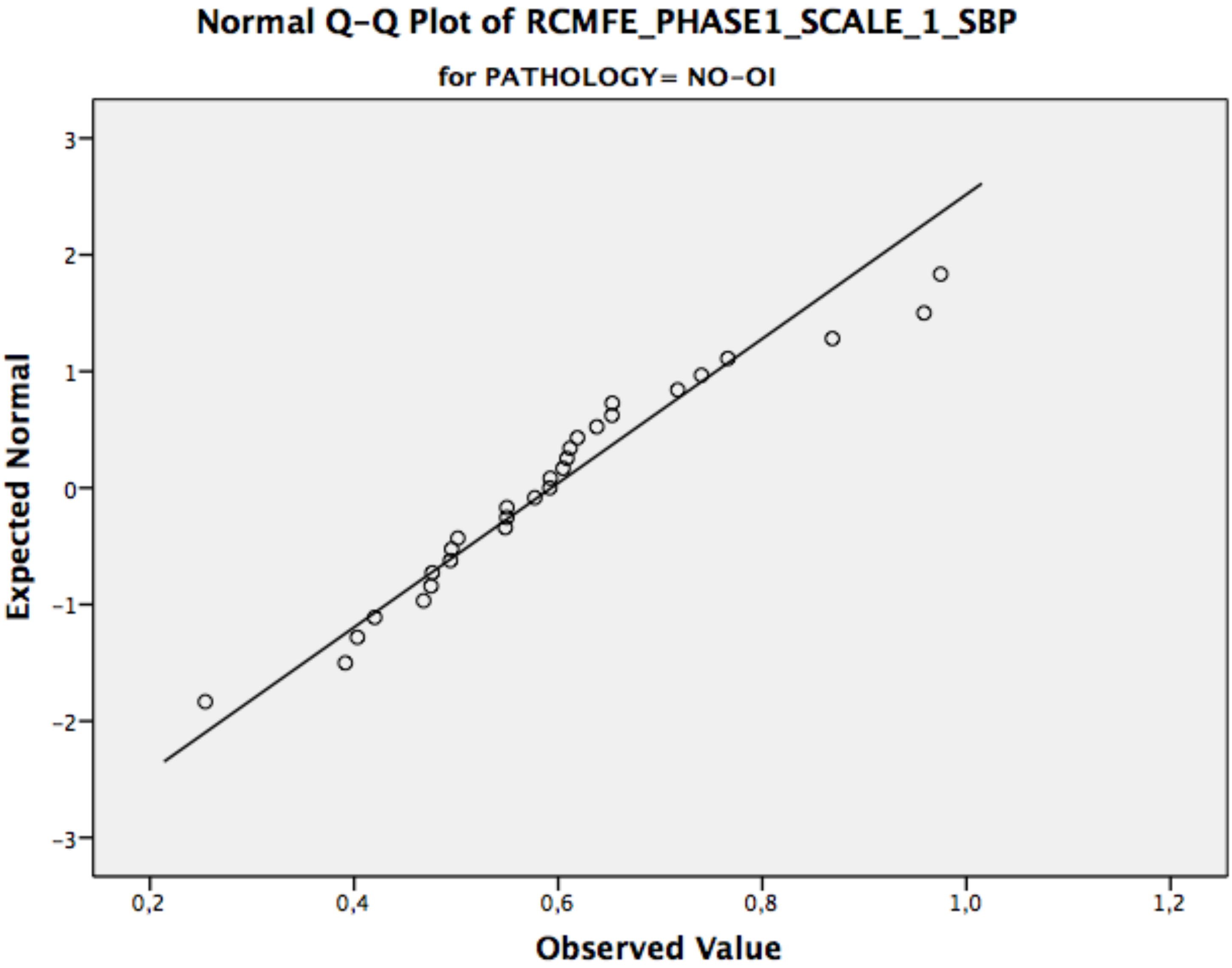

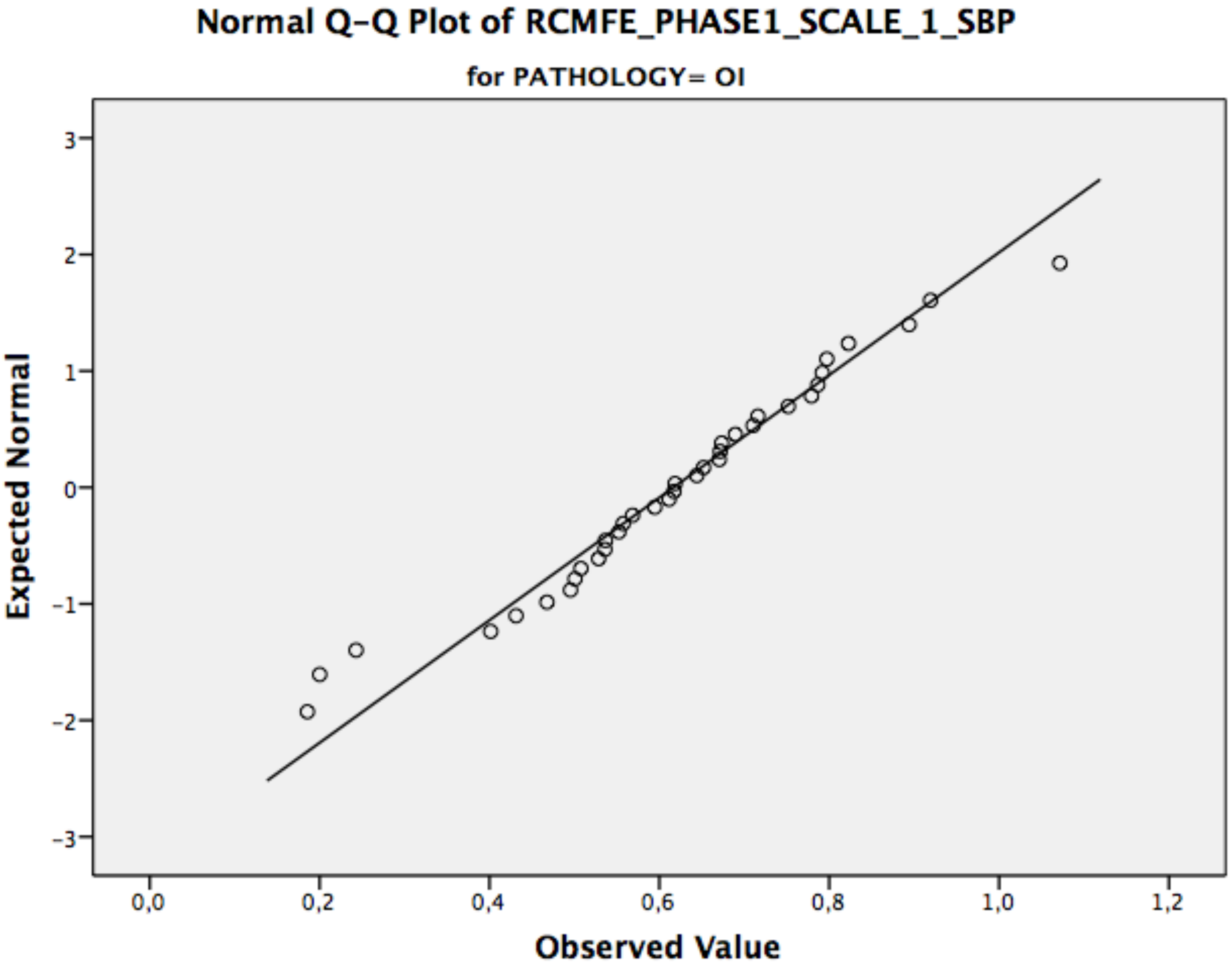
Normal Q-Q Plot of RCMFE_PHASE1_SCALE_1_SBP
for PATHOLOGY= OI
Expected Normal
3
2
1
0
−1
−2
−3
0,0
0,2
0,4
0,6
0,8
1,0
1,2
Observed Value

RCMFE_PHASE1_SCALE_2_SBP

Normal Q-Q Plots

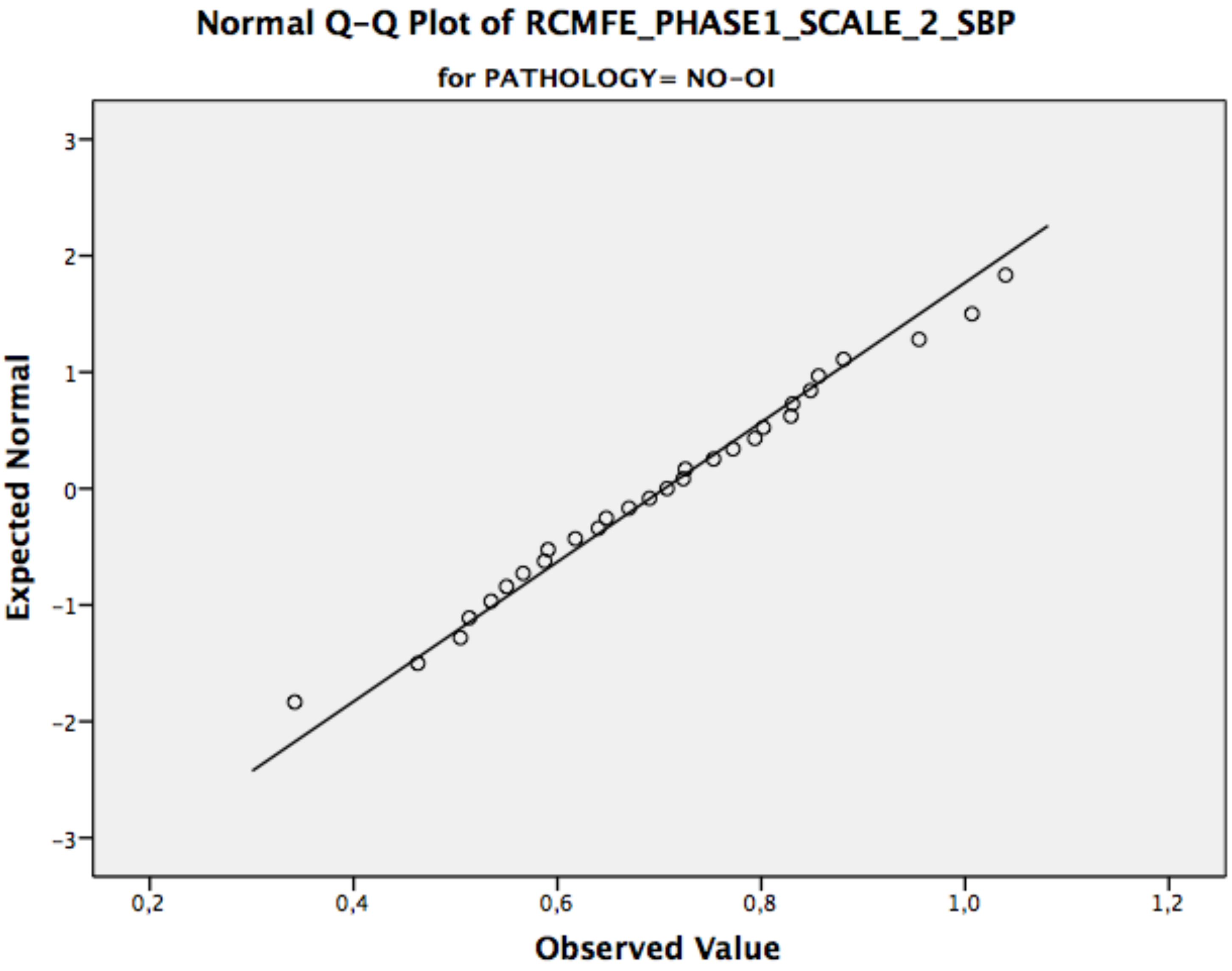

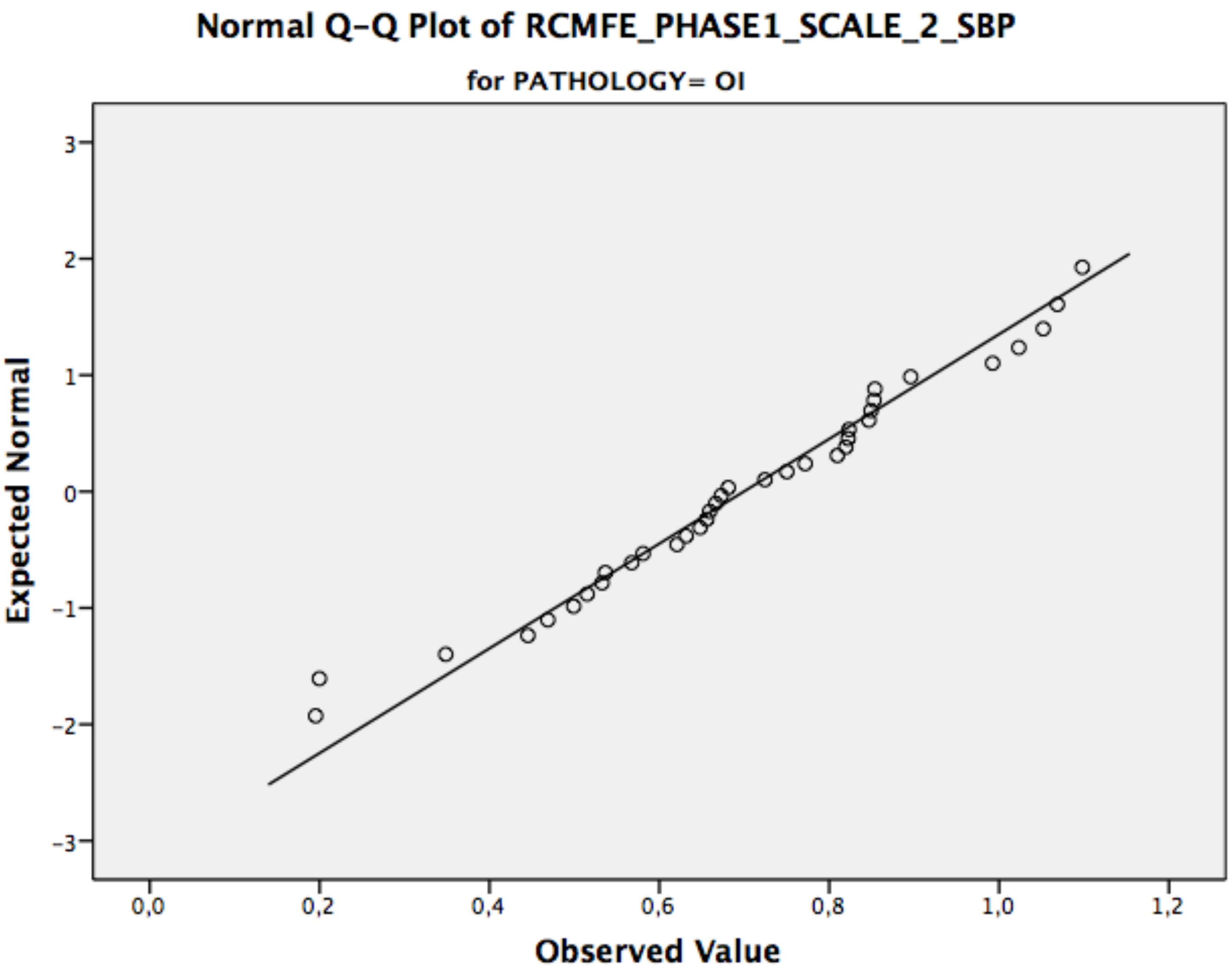
Normal Q-Q Plot of RCMFE_PHASE1_SCALE_2_SBP
for PATHOLOGY= OI
Expected Normal
Observed Value
3
2
1
0
−1
−2
−3
0,0
0,2
0,4
0,6
0,8
1,0
1,2

RCMFE_PHASE1_SCALE_3_SBP

Normal Q-Q Plots

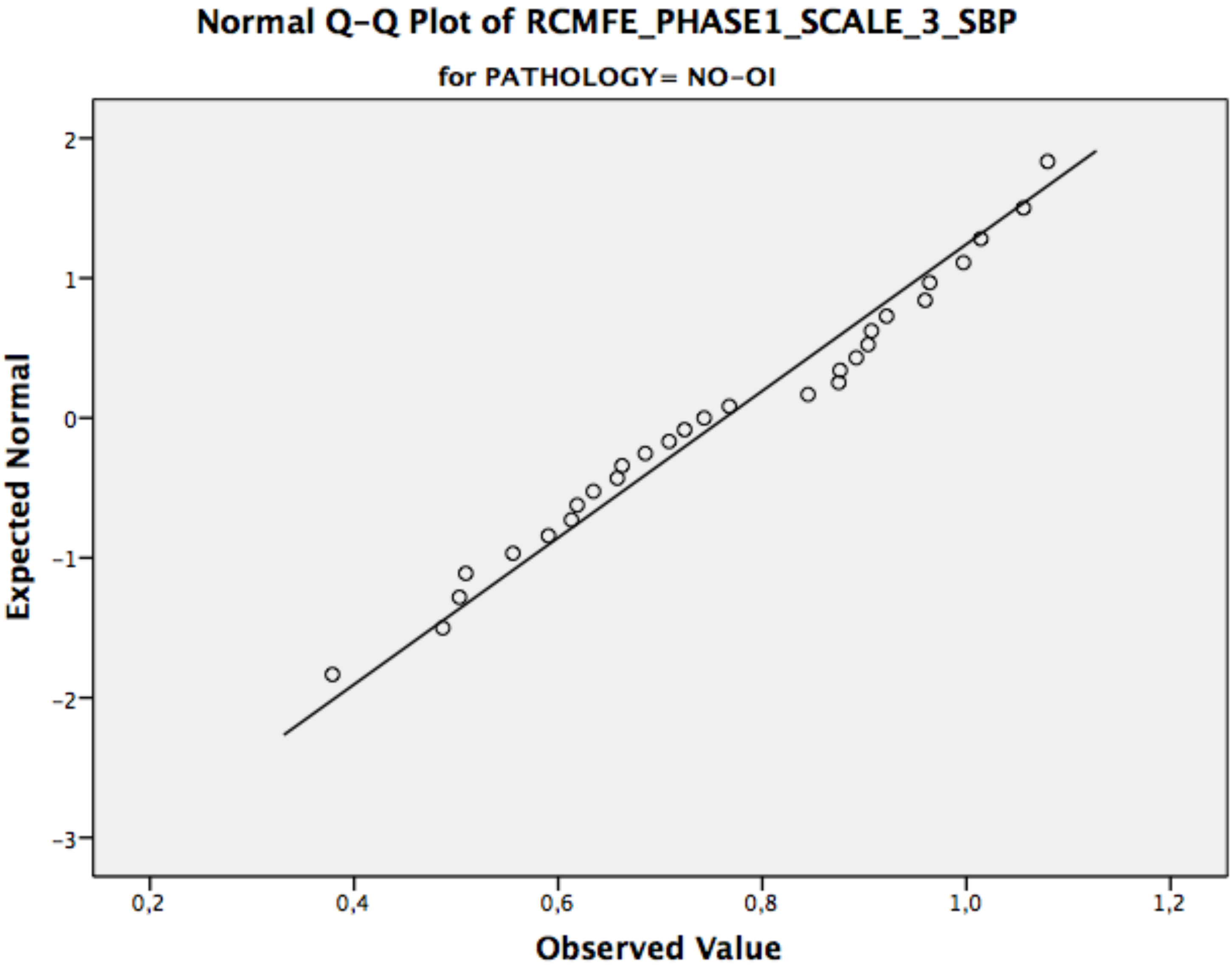

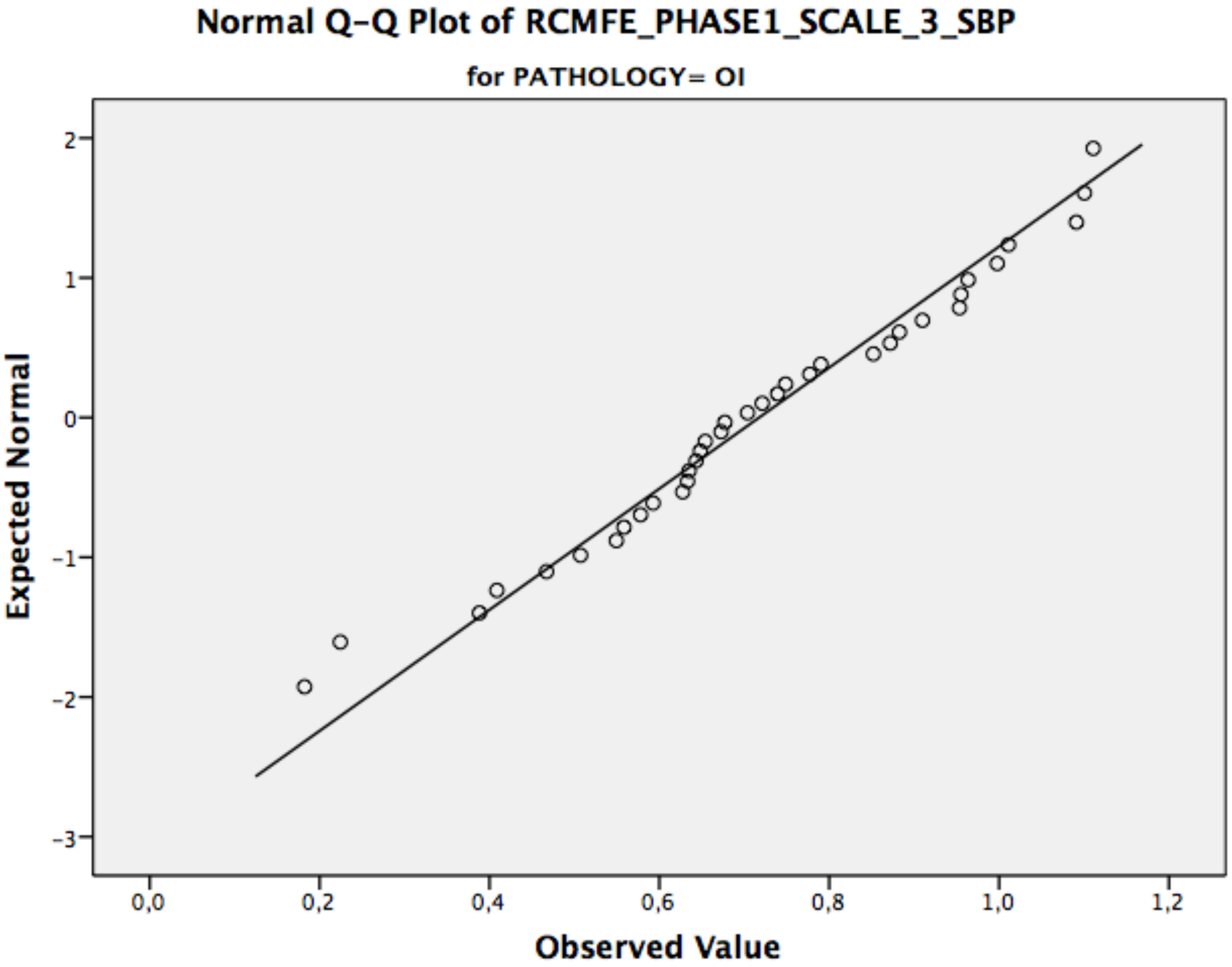
Normal Q-Q Plot of RCMFE_PHASE1_SCALE_3_SBP
for PATHOLOGY= OI
Expected Normal
Observed Value
2
1
0
-1
-2
-3
0,0
0,2
0,4
0,6
0,8
1,0
1,2

RCMFE_PHASE1_SCALE_4_SBP

Normal Q-Q Plots

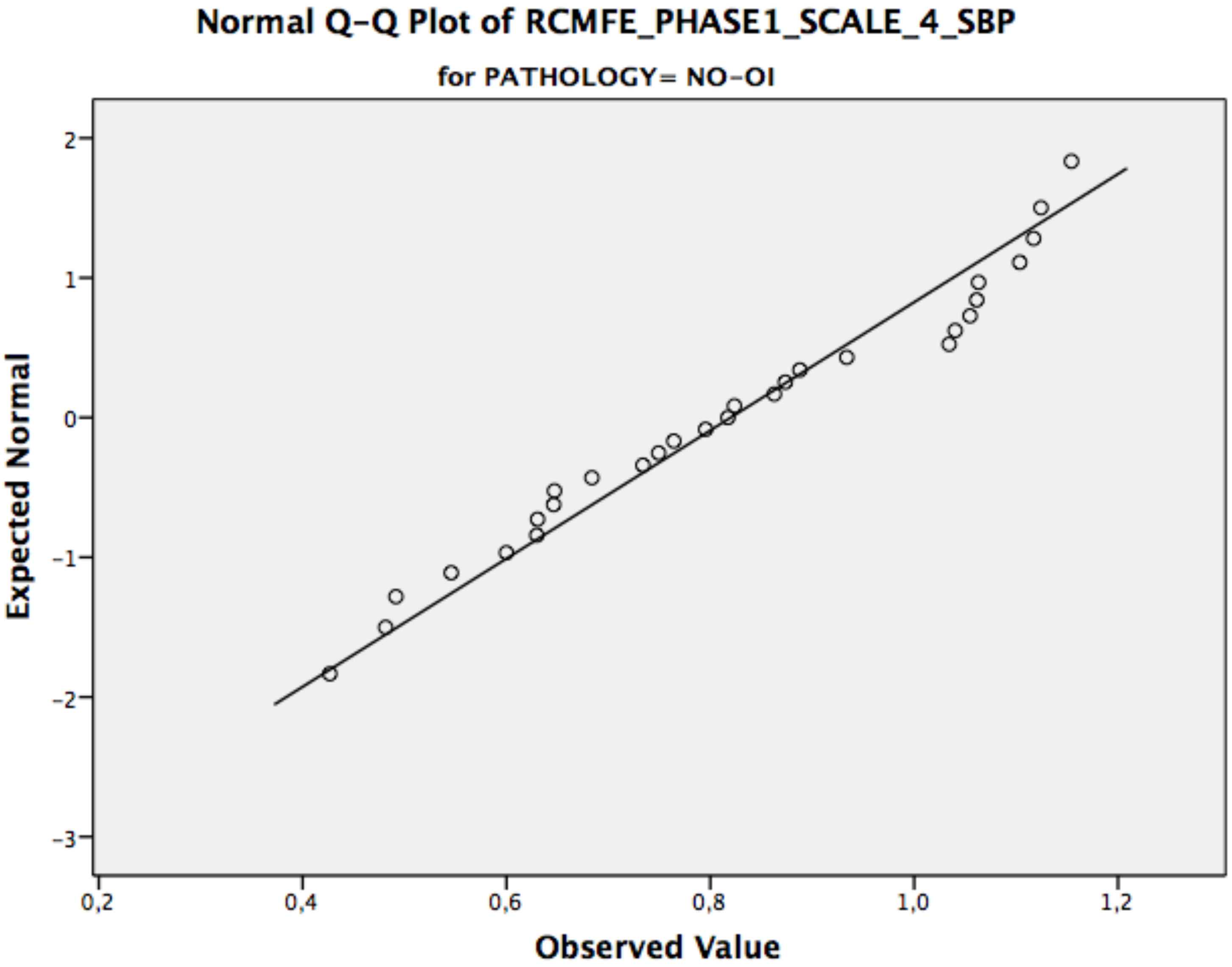

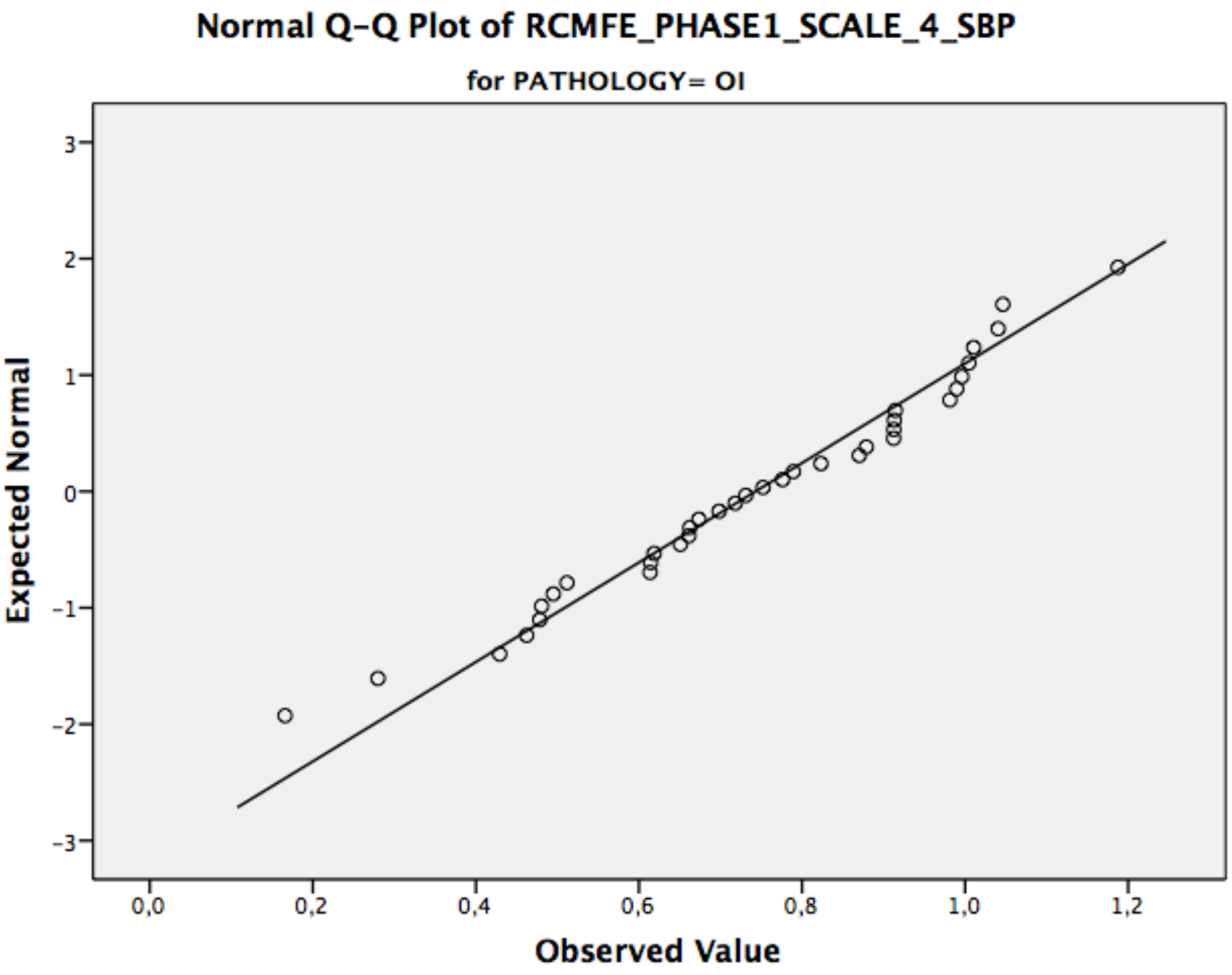
Normal Q-Q Plot of RCMFE_PHASE1_SCALE_4_SBP
for PATHOLOGY= OI
Expected Normal
Observed Value
3
2
1
0
-1
-2
-3
0,0
0,2
0,4
0,6
0,8
1,0
1,2

RCMFE_PHASE1_SCALE_5_SBP

Normal Q-Q Plots

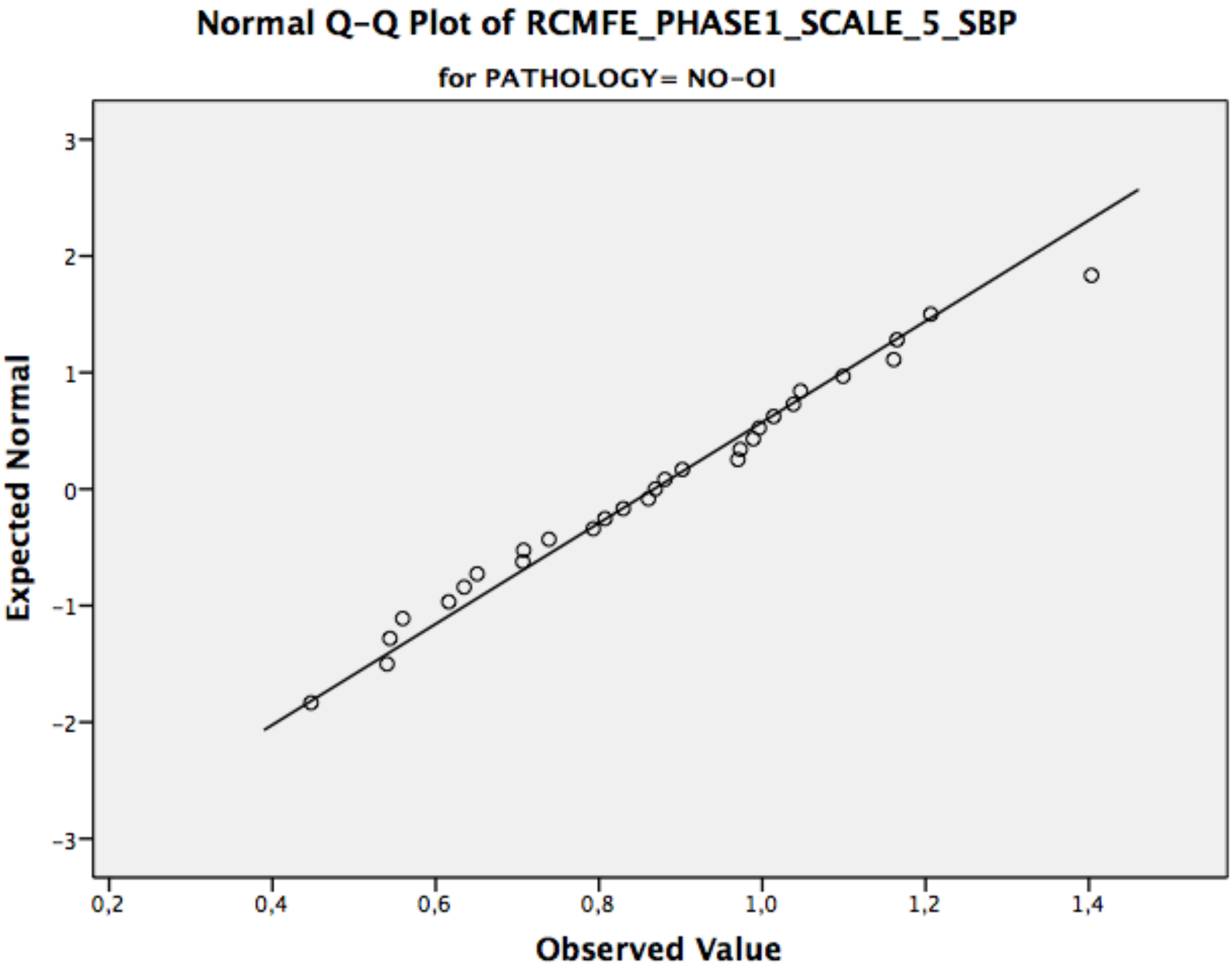

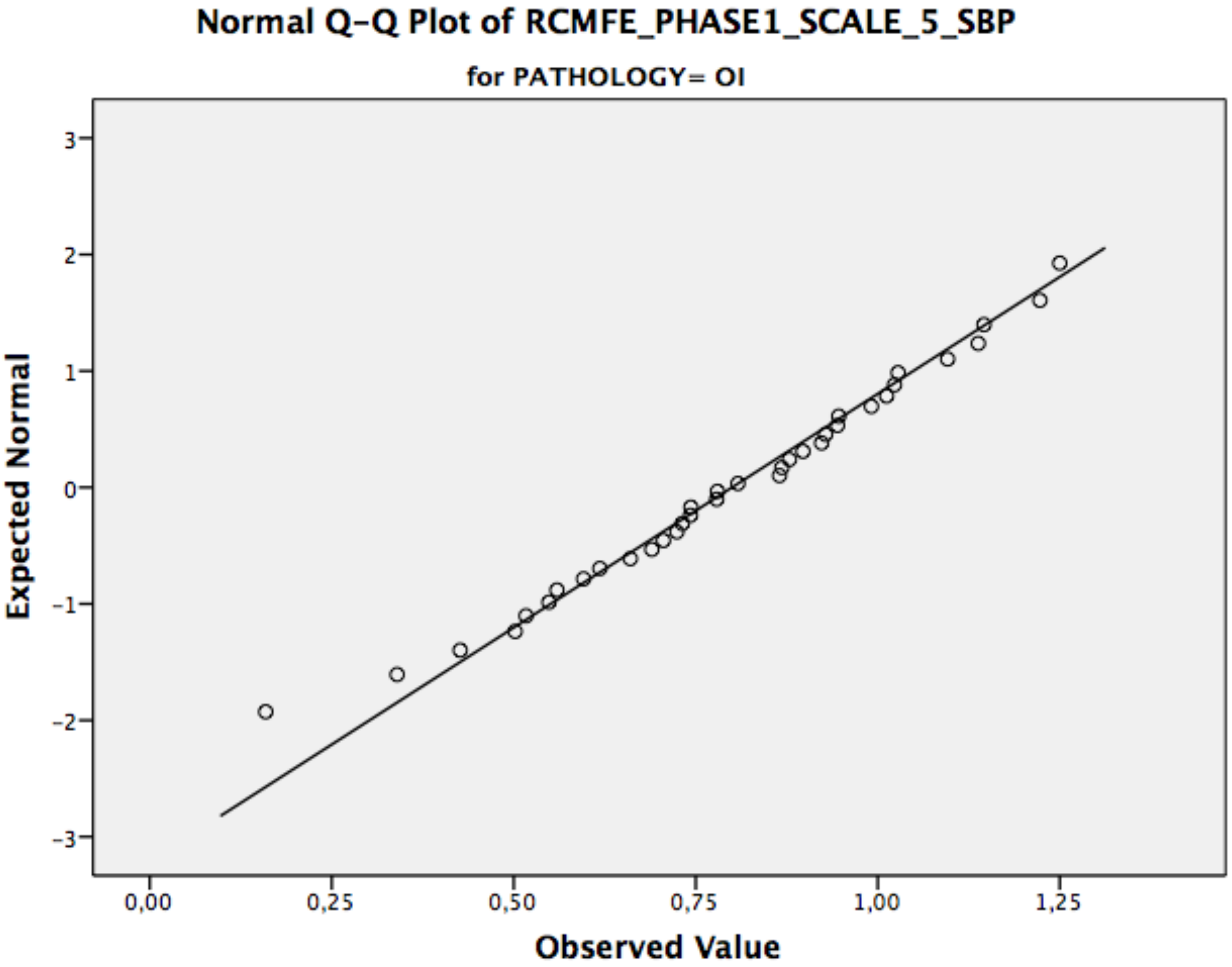
Normal Q-Q Plot of RCMFE_PHASE1_SCALE_5_SBP
for PATHOLOGY= OI
Expected Normal
Observed Value
3
2
1
0
-1
-2
-3
0,00
0,25
0,50
0,75
1,00
1,25

RCMFE_PHASE2_SCALE_1_SBP

Normal Q-Q Plots

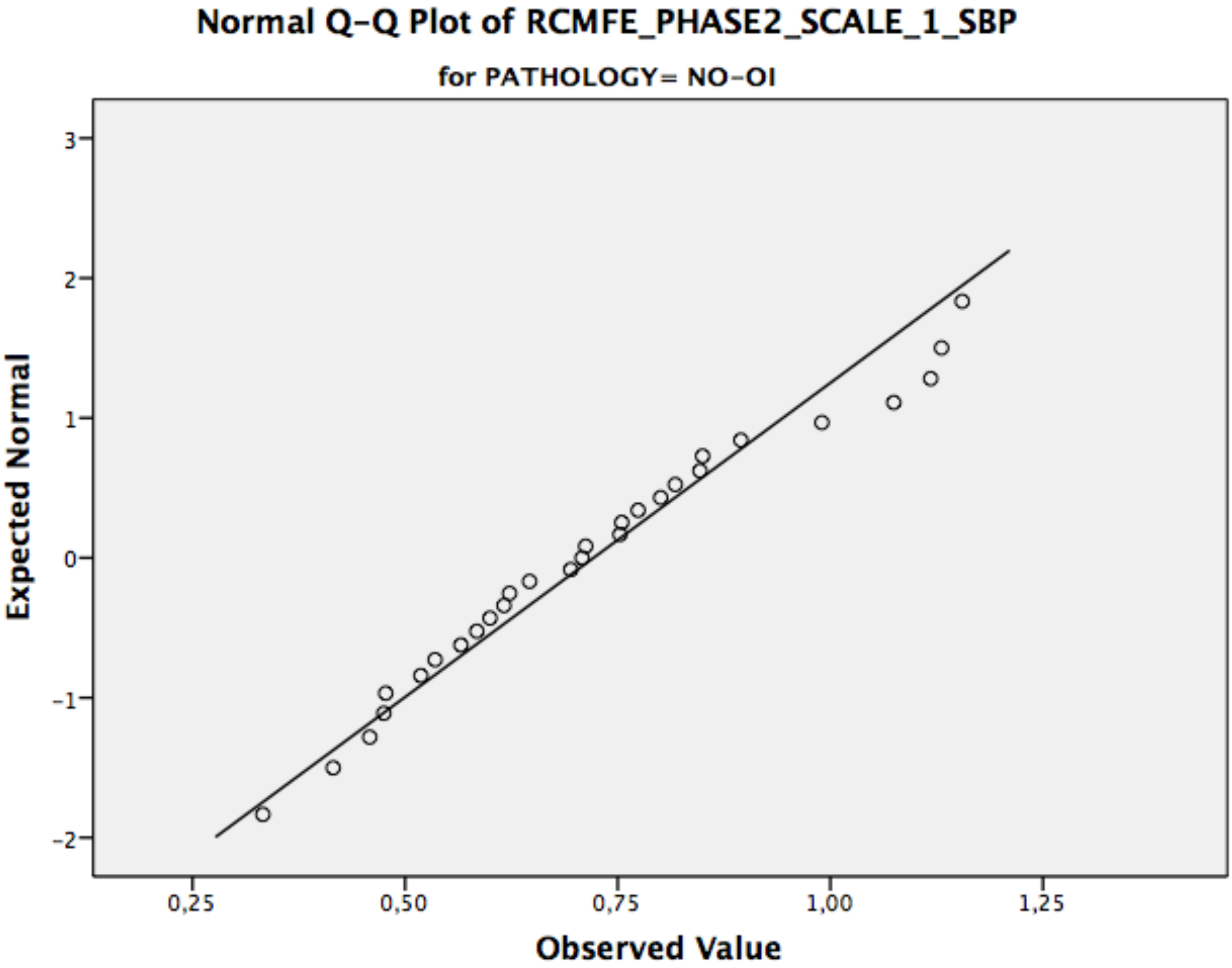

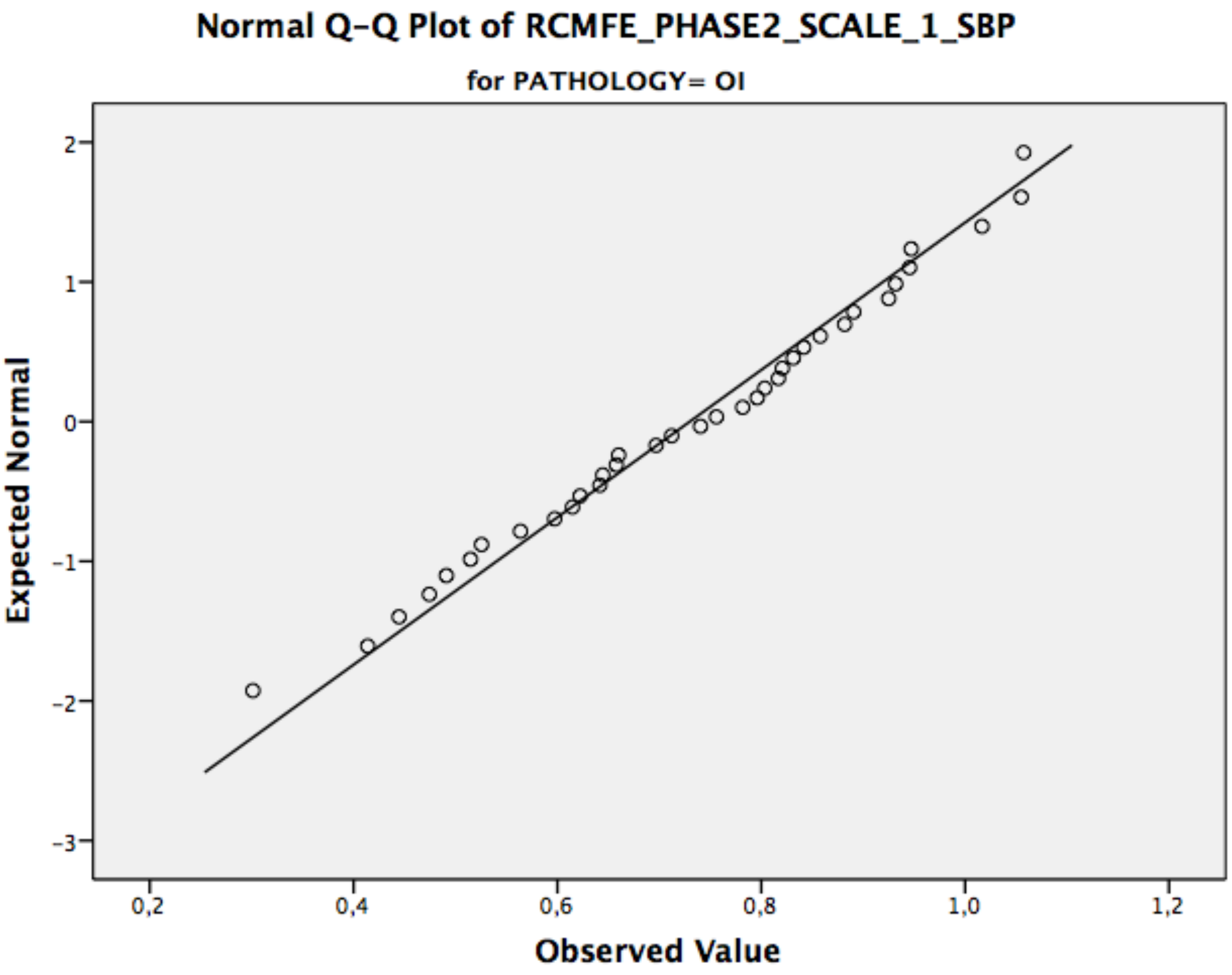
Normal Q-Q Plot of RCMFE_PHASE2_SCALE_1_SBP
for PATHOLOGY= OI
Expected Normal
Observed Value
2
1
0
-1
-2
-3
0,2
0,4
0,6
0,8
1,0
1,2

RCMFE_PHASE2_SCALE_2_SBP

Normal Q-Q Plots

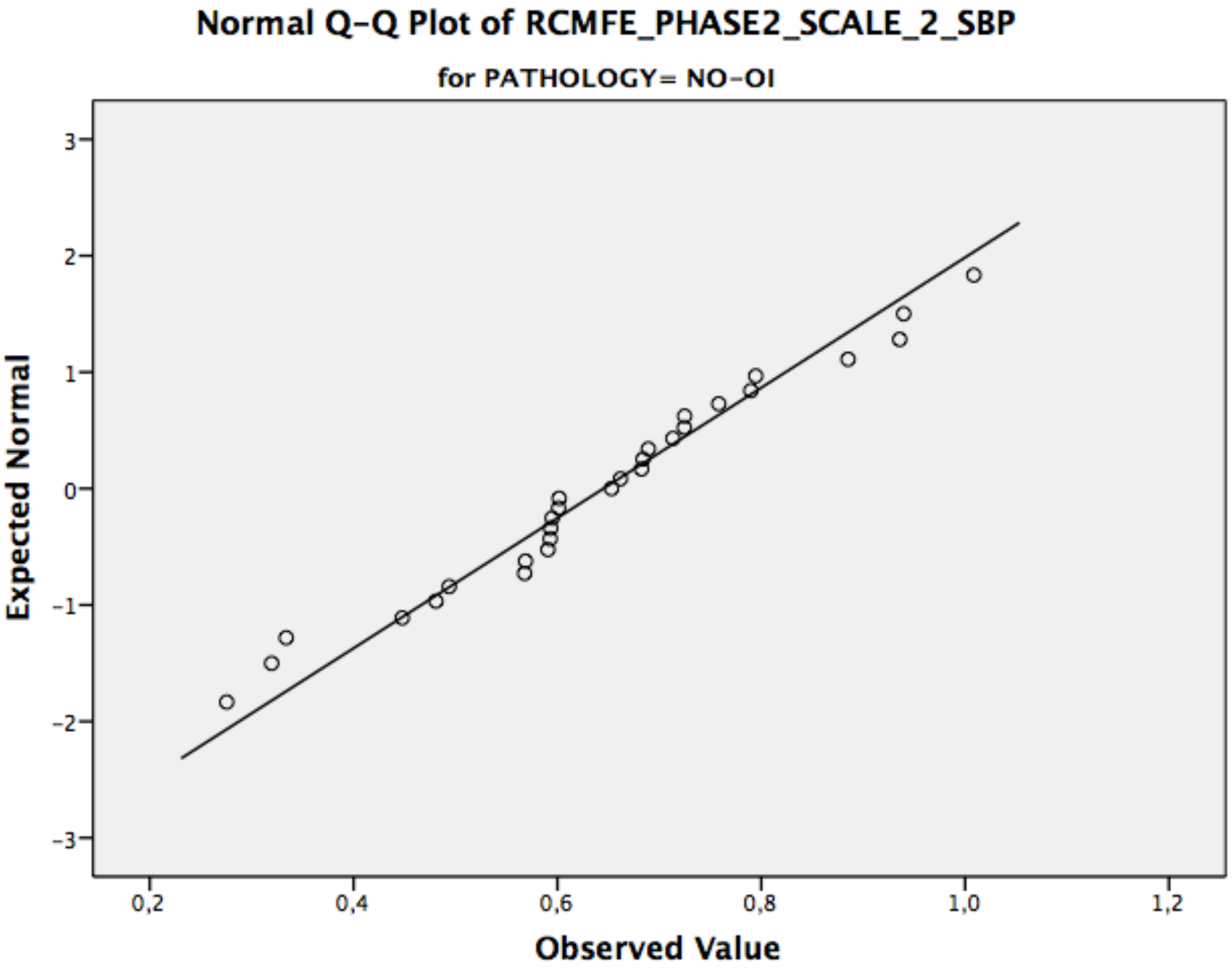

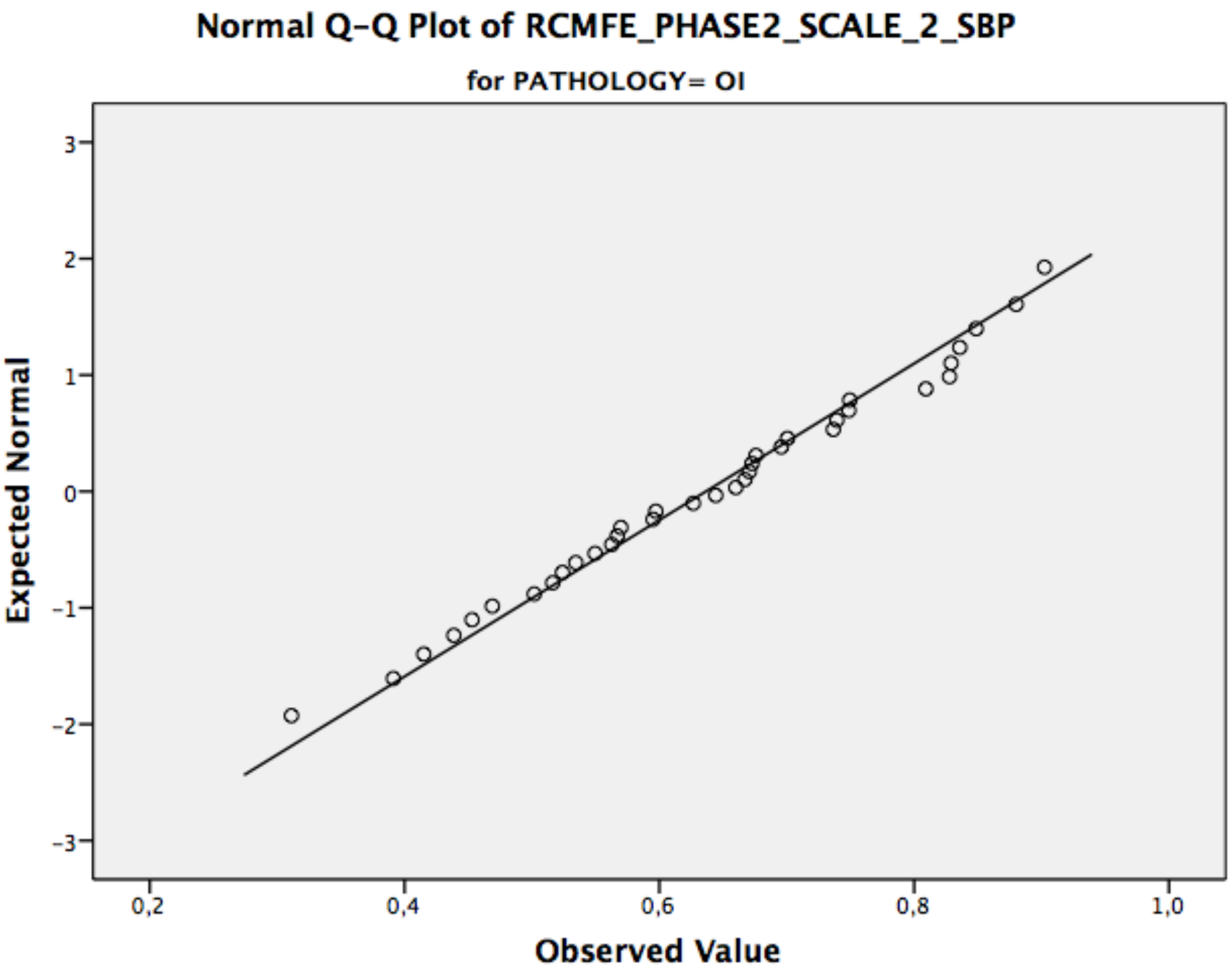

Normal Q-Q Plot of RCMFE_PHASE2_SCALE_2_SBP
for PATHOLOGY= OI
Expected Normal
Observed Value
3
2
1
0
-1
-2
-3
0,2
0,4
0,6
0,8
1,0

RCMFE_PHASE2_SCALE_3_SBP

Normal Q-Q Plots

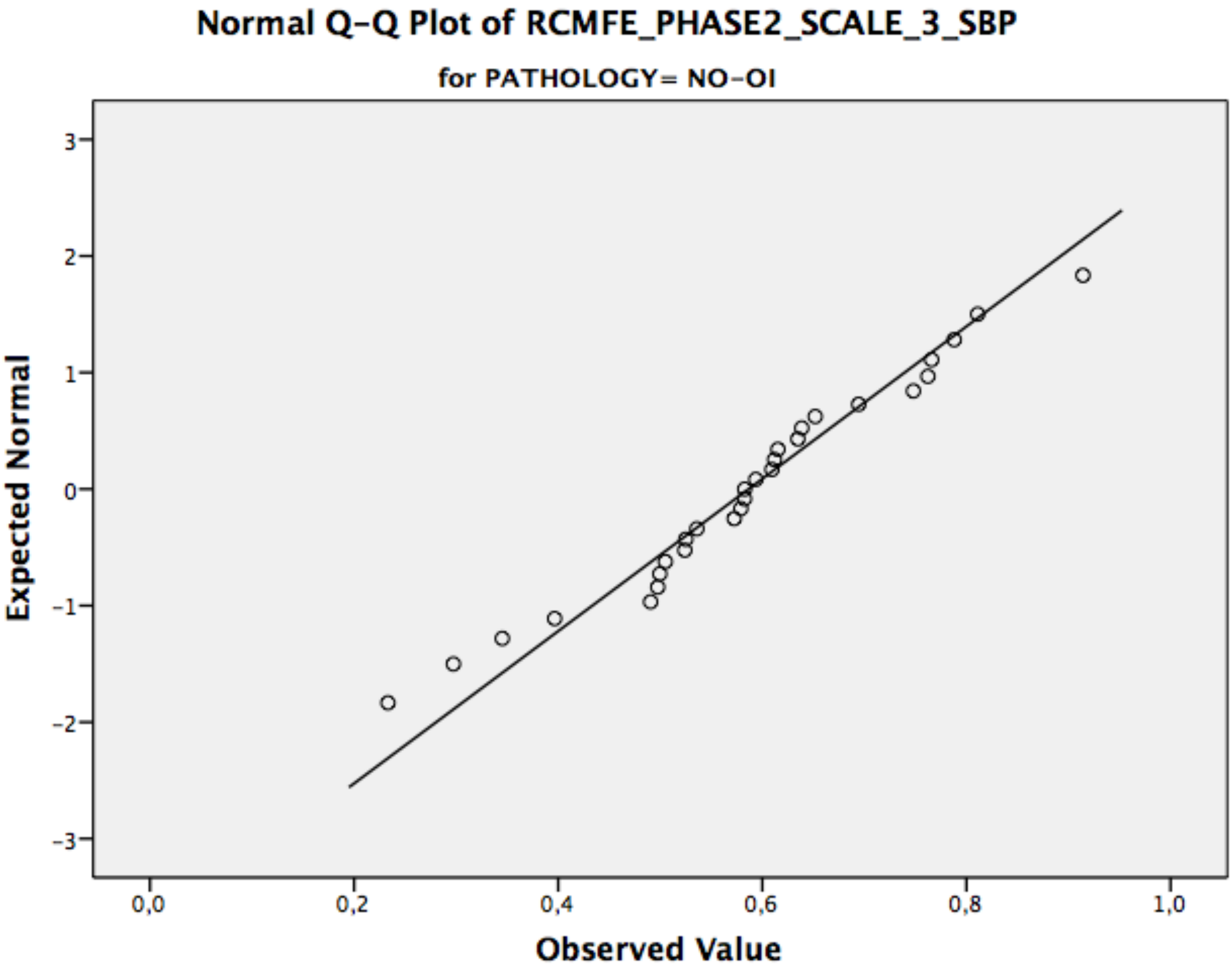

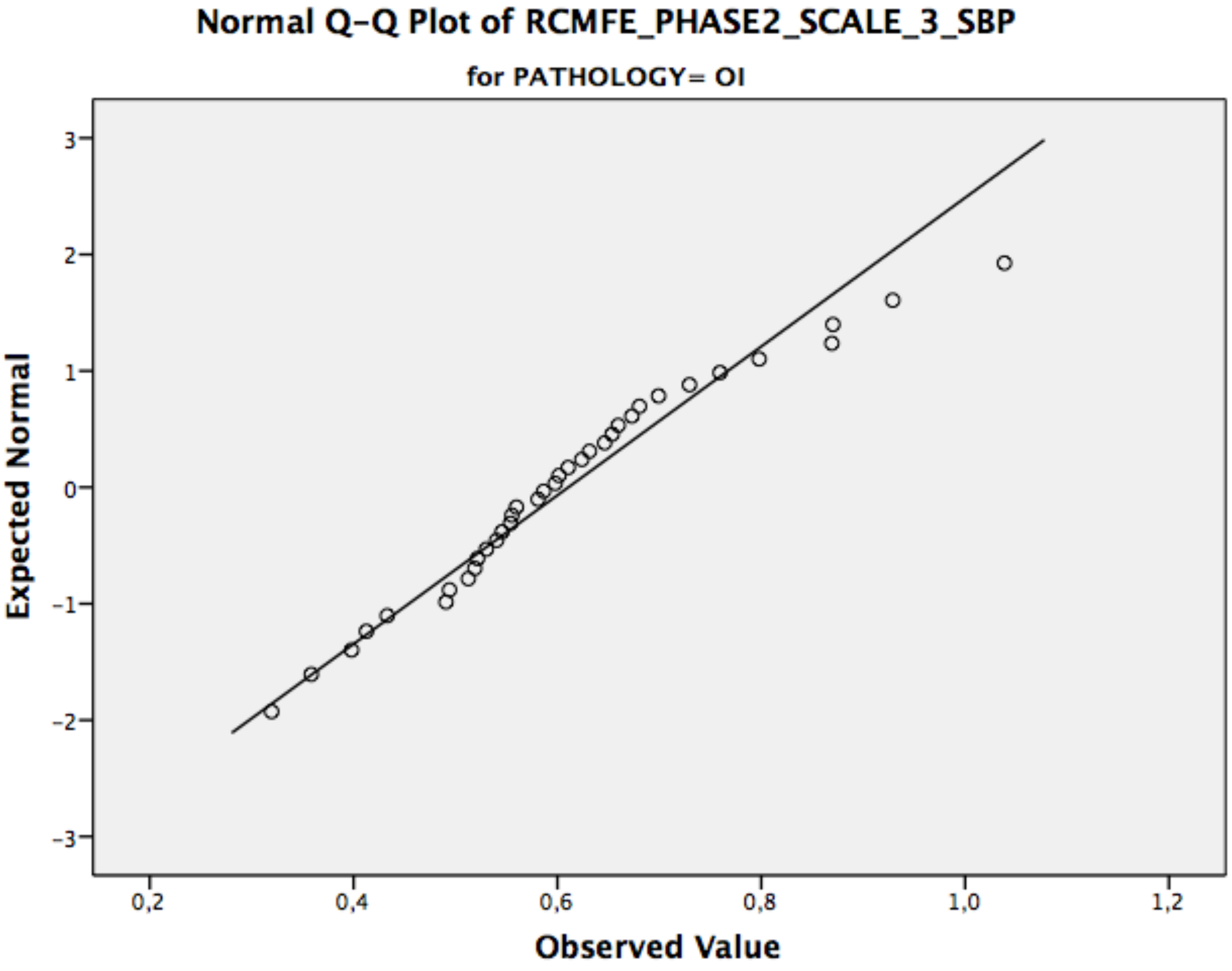
Normal Q-Q Plot of RCMFE_PHASE2_SCALE_3_SBP
for PATHOLOGY= OI
Expected Normal
3
2
1
0
-1
-2
-3
0,2
0,4
0,6
0,8
1,0
1,2
Observed Value

RCMFE_PHASE2_SCALE_4_SBP

Normal Q-Q Plots

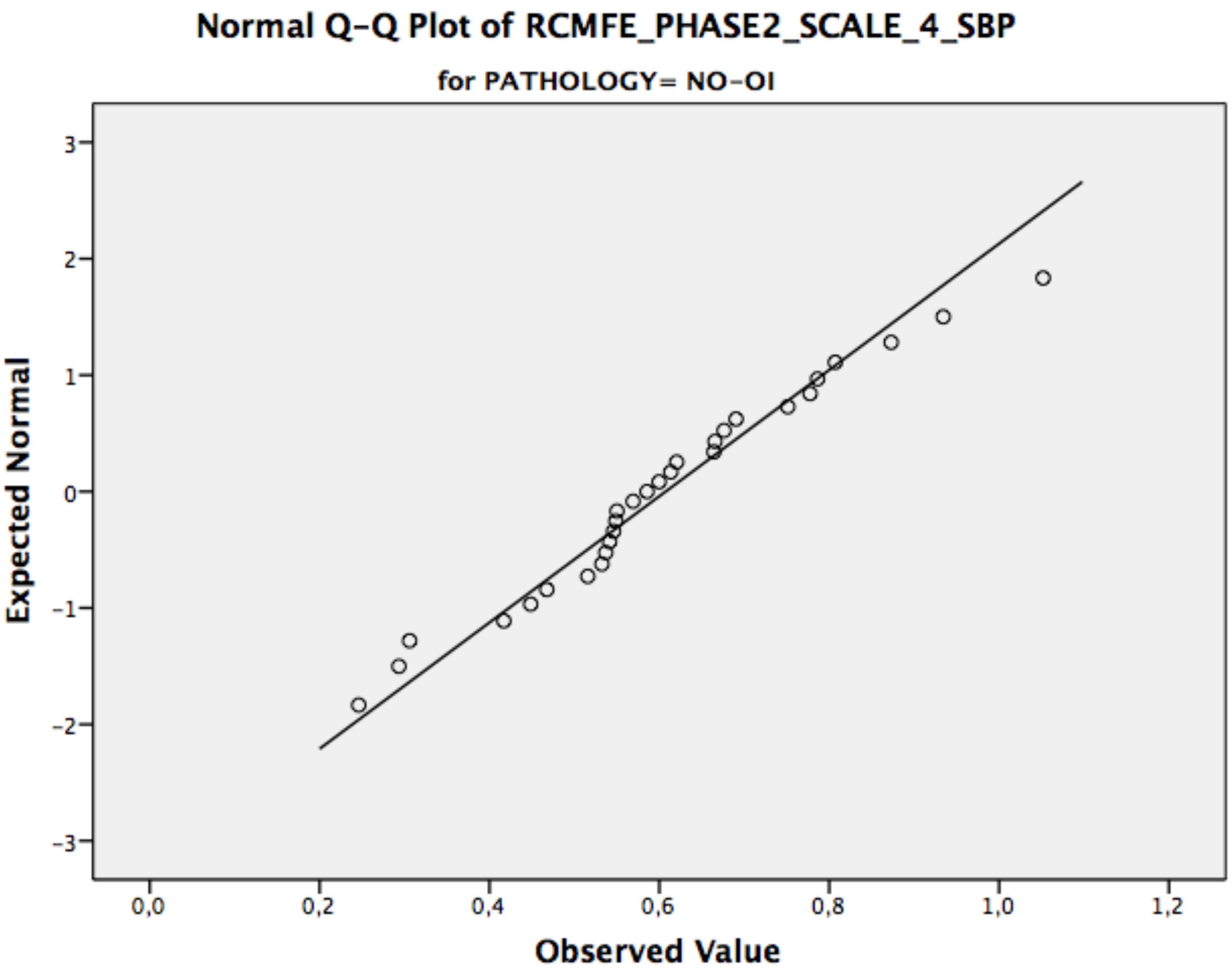

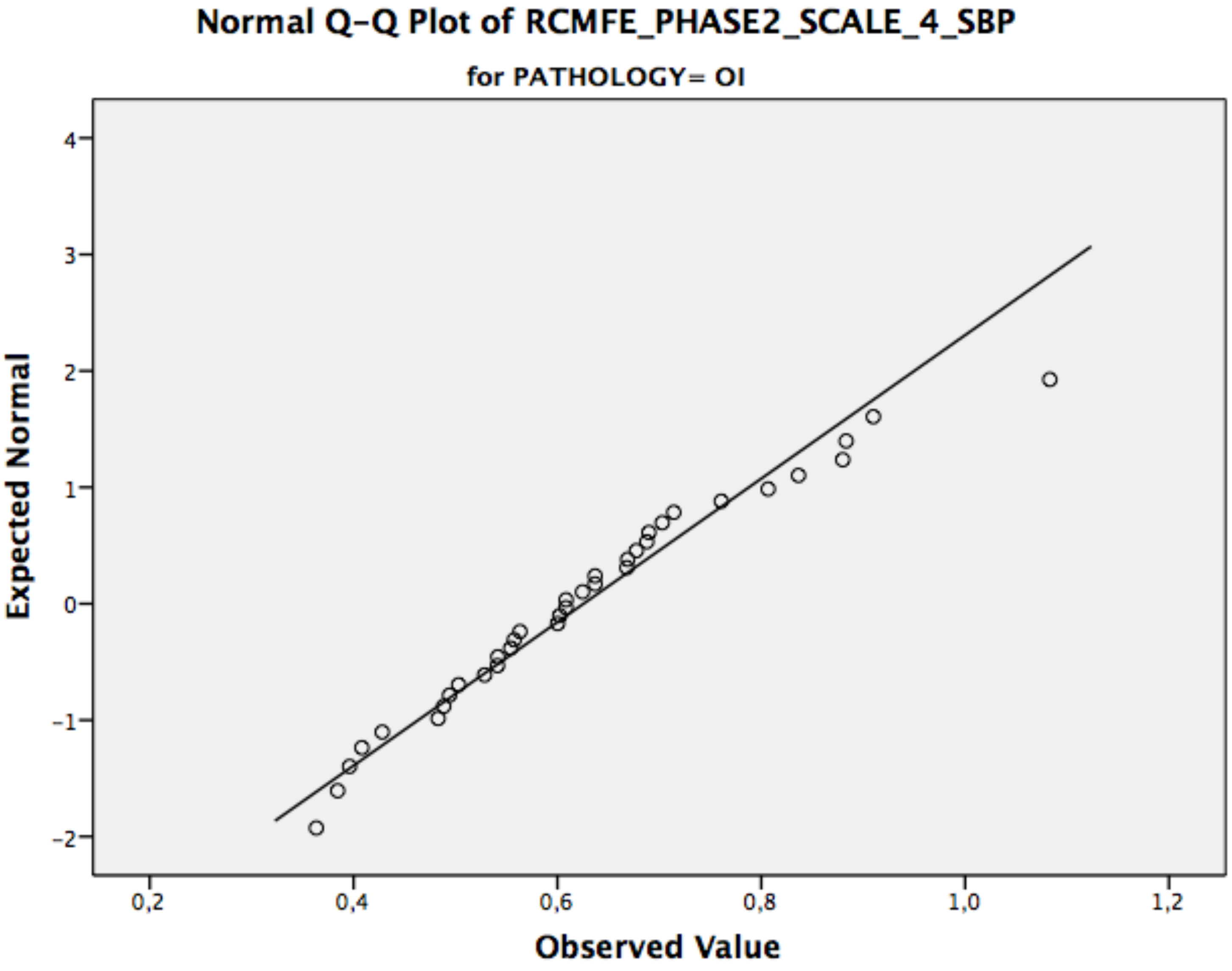
Normal Q-Q Plot of RCMFE_PHASE2_SCALE_4_SBP
for PATHOLOGY= OI
Expected Normal
Observed Value
4
3
2
1
0
-1
-2
0,2
0,4
0,6
0,8
1,0
1,2

RCMFE_PHASE2_SCALE_5_SBP

Normal Q-Q Plots

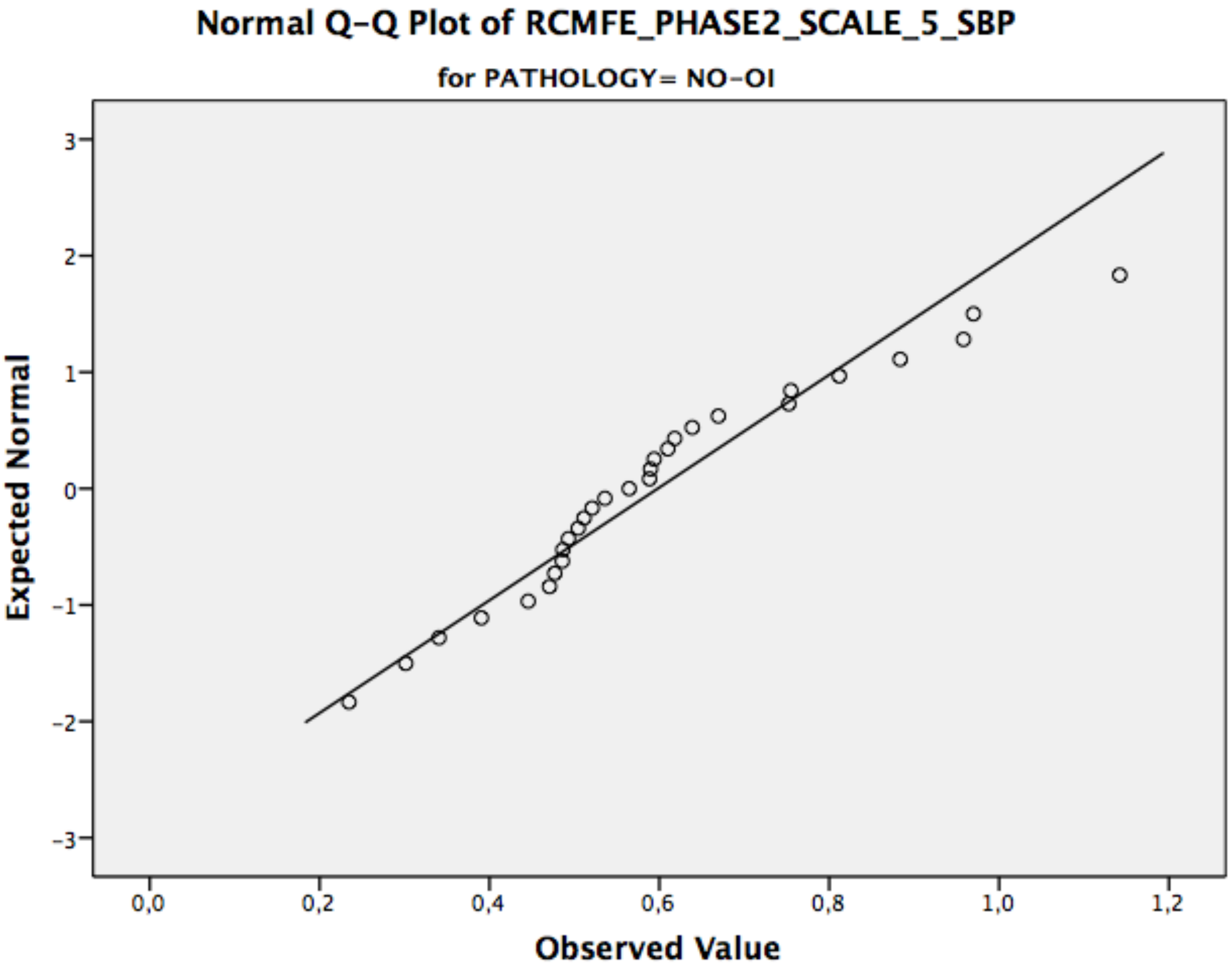

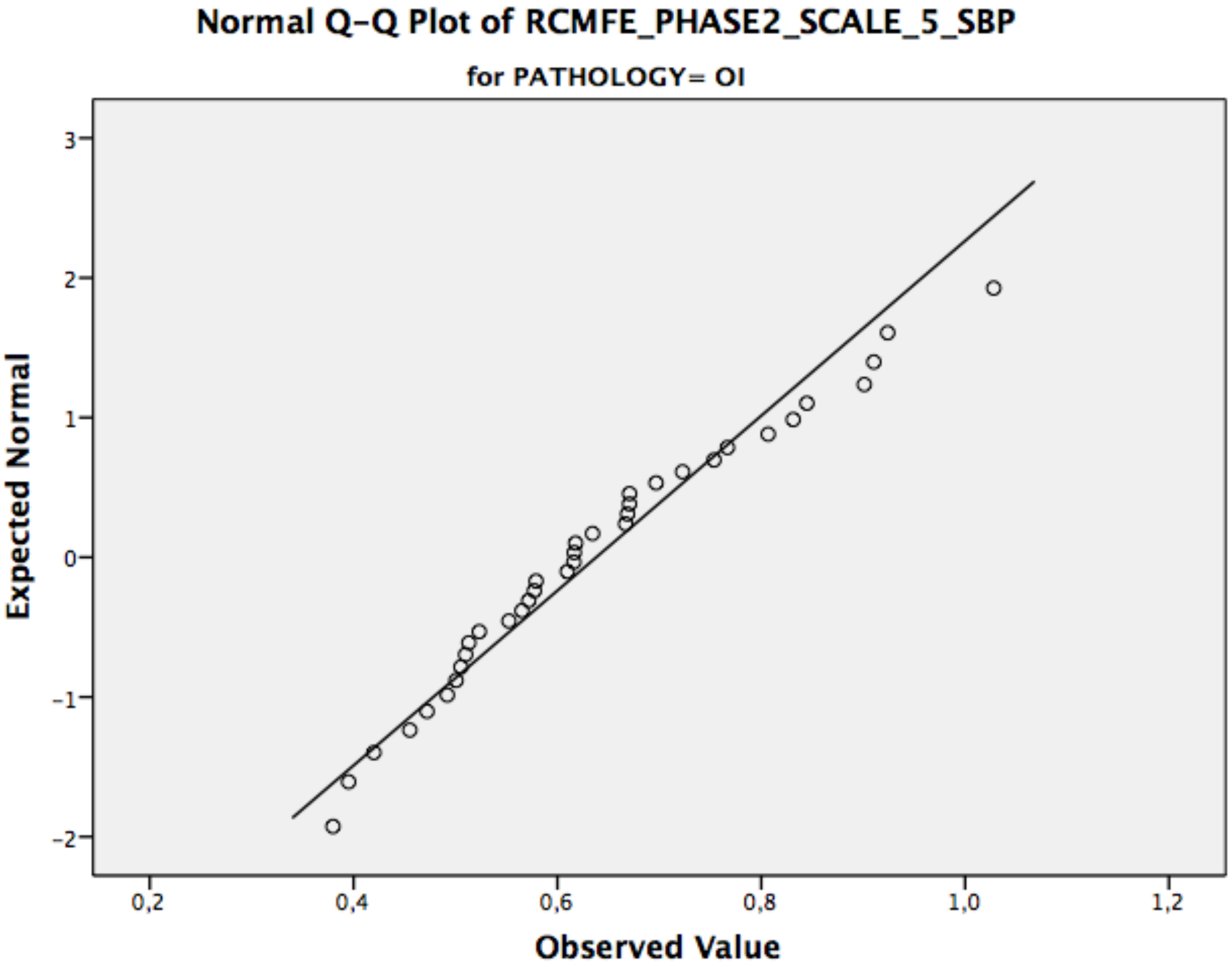

Normal Q-Q Plot of RCMFE_PHASE2_SCALE_5_SBP
for PATHOLOGY= OI
Expected Normal
Observed Value
3
2
1
0
-1
-2
0,2
0,4
0,6
0,8
1,0
1,2

RCMFE_PHASE3_SCALE_1_SBP

Normal Q-Q Plots

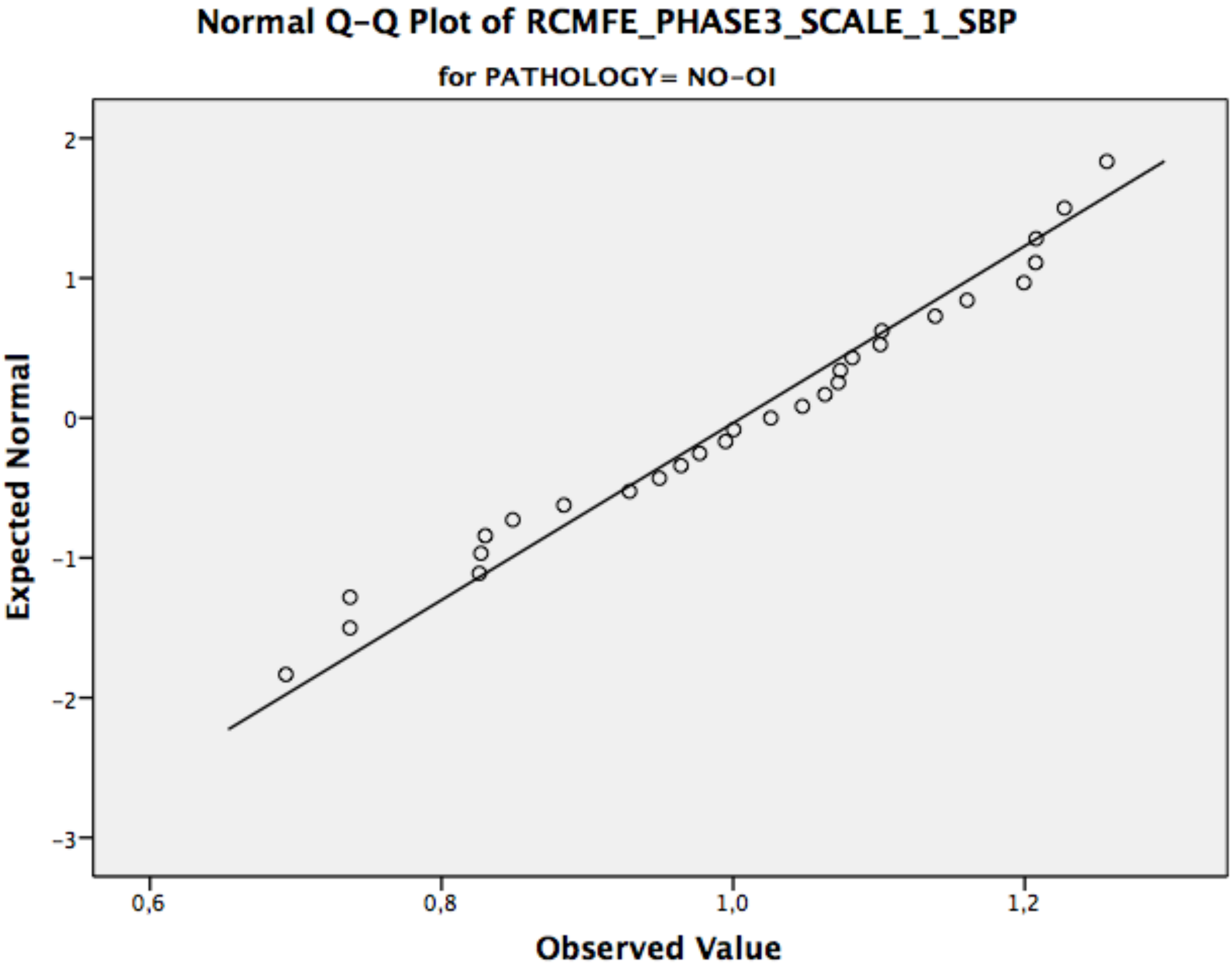

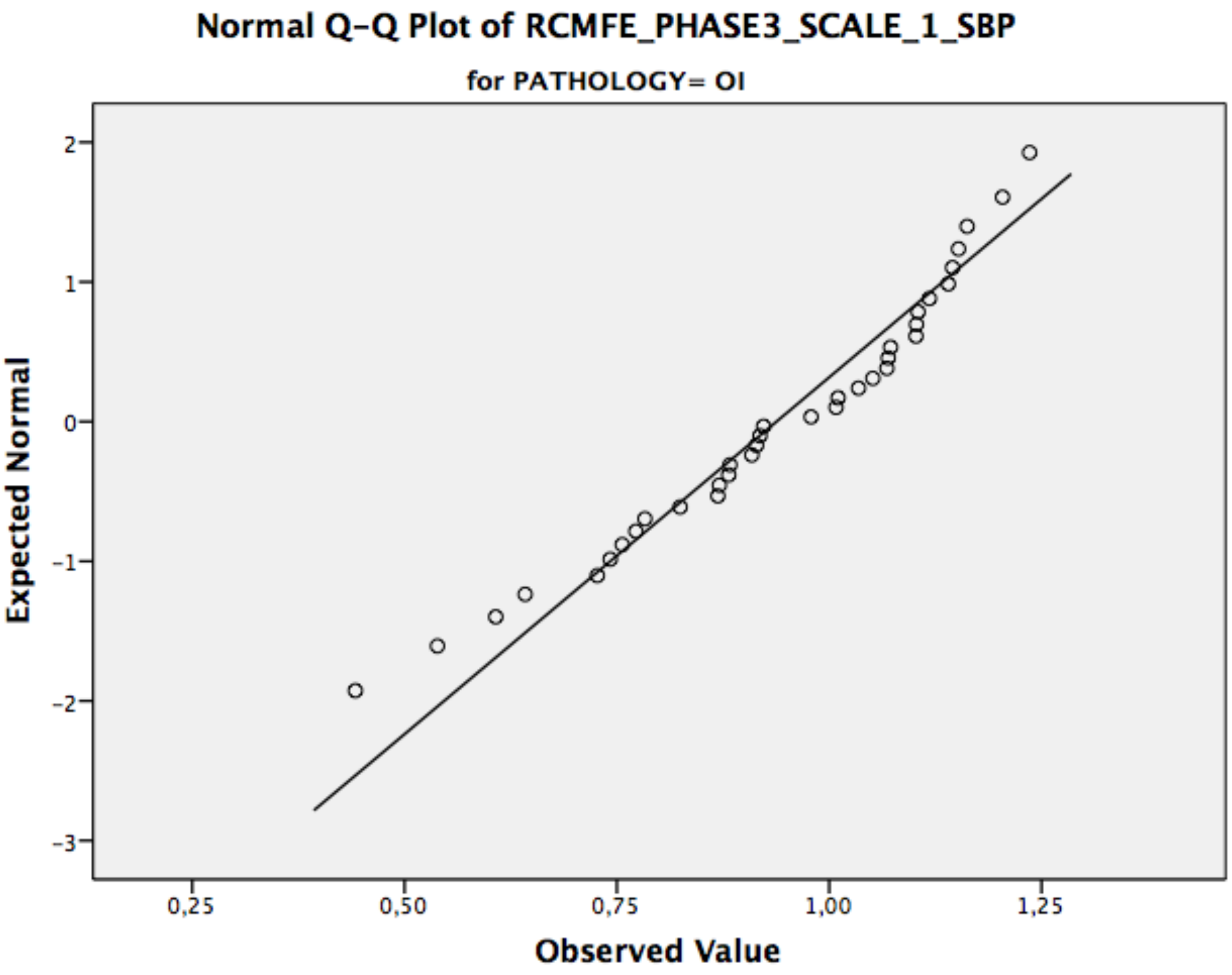

Normal Q-Q Plot of RCMFE_PHASE3_SCALE_1_SBP
for PATHOLOGY= OI
Expected Normal
2
1
0
−1
−2
−3
0,25
0,50
0,75
1,00
1,25
Observed Value

RCMFE_PHASE3_SCALE_2_SBP

Normal Q-Q Plots

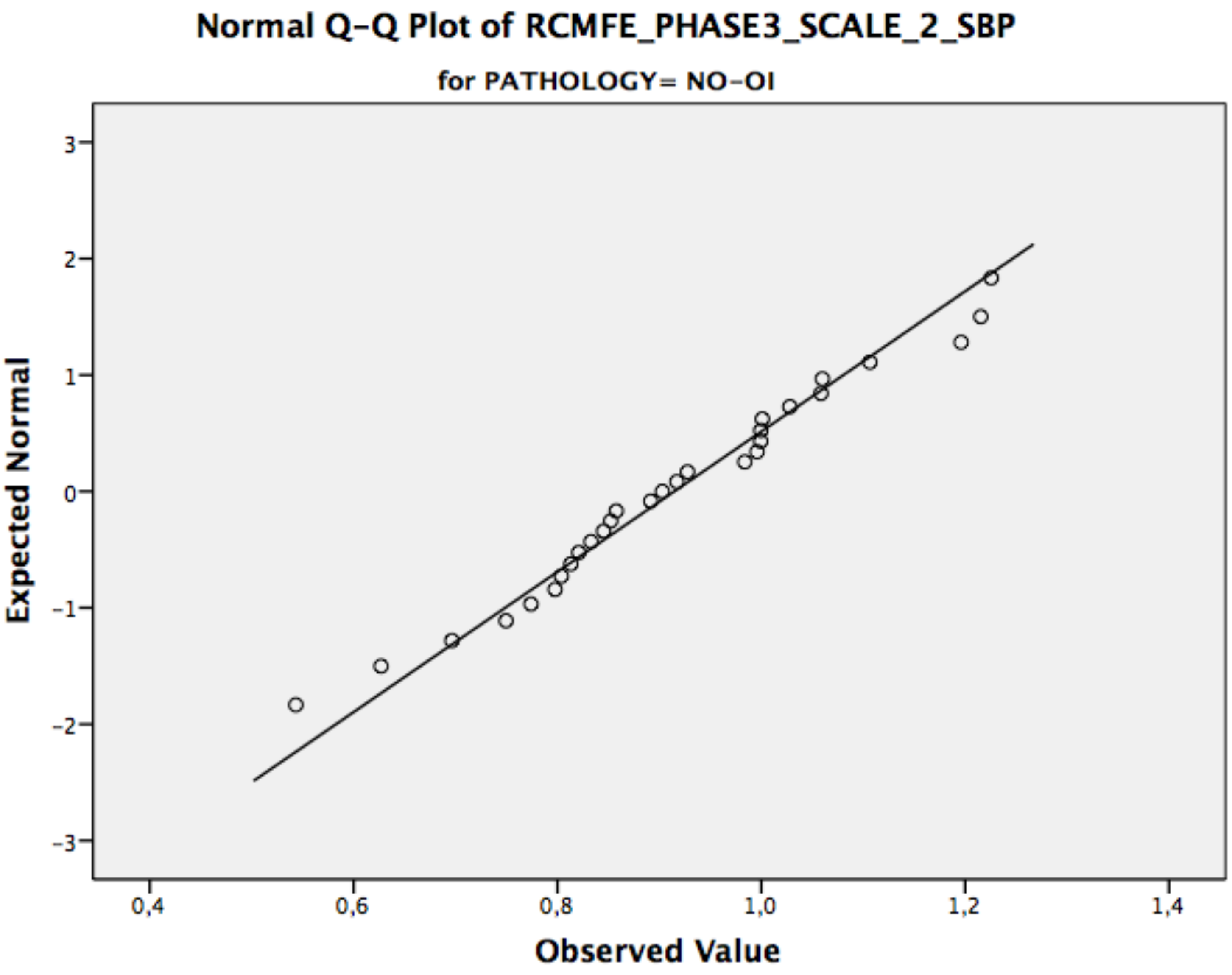

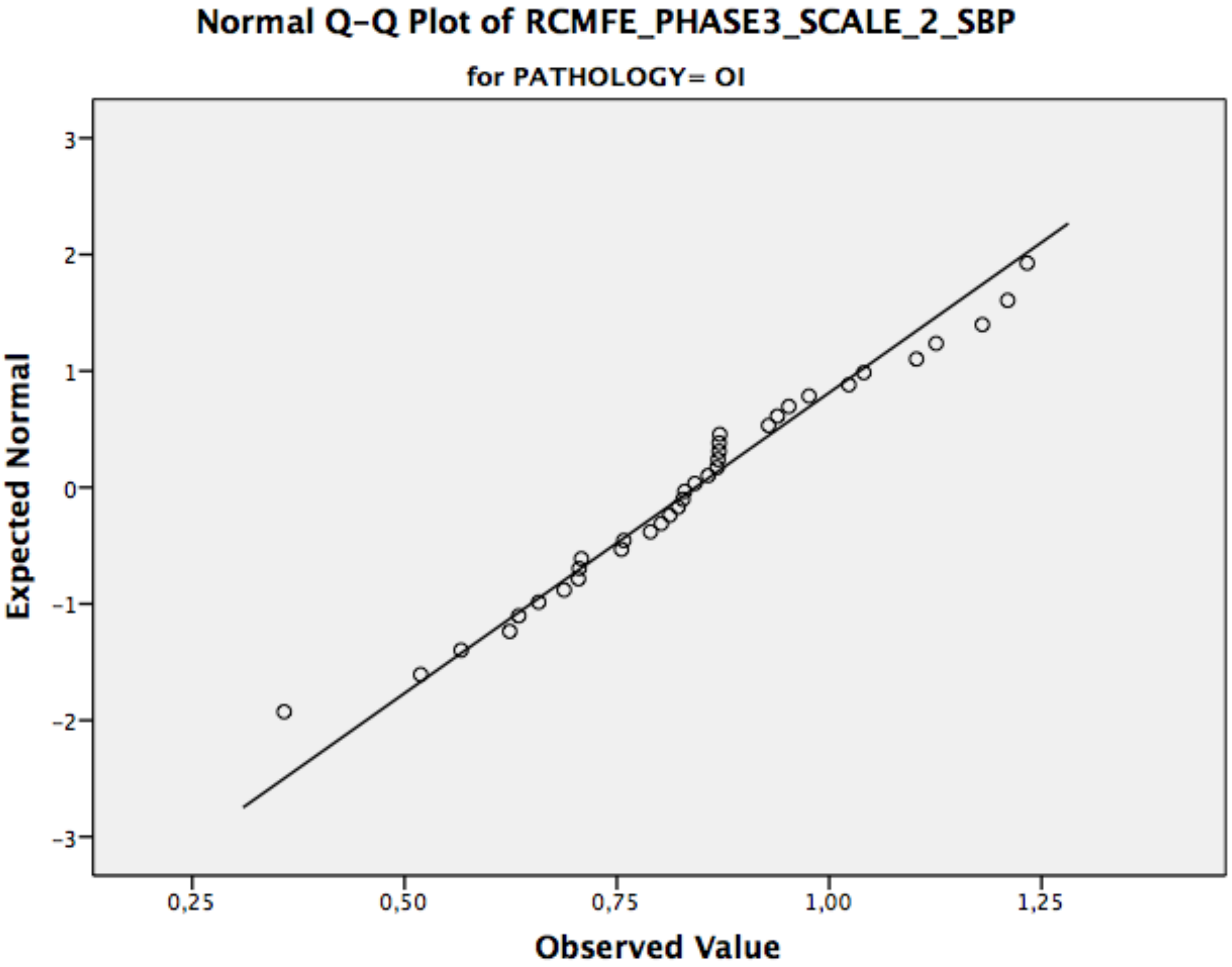

Normal Q-Q Plot of RCMFE_PHASE3_SCALE_2_SBP
for PATHOLOGY= OI
Expected Normal
Observed Value
3
2
1
0
-1
-2
-3
0,25
0,50
0,75
1,00
1,25

RCMFE_PHASE3_SCALE_3_SBP

Normal Q-Q Plots

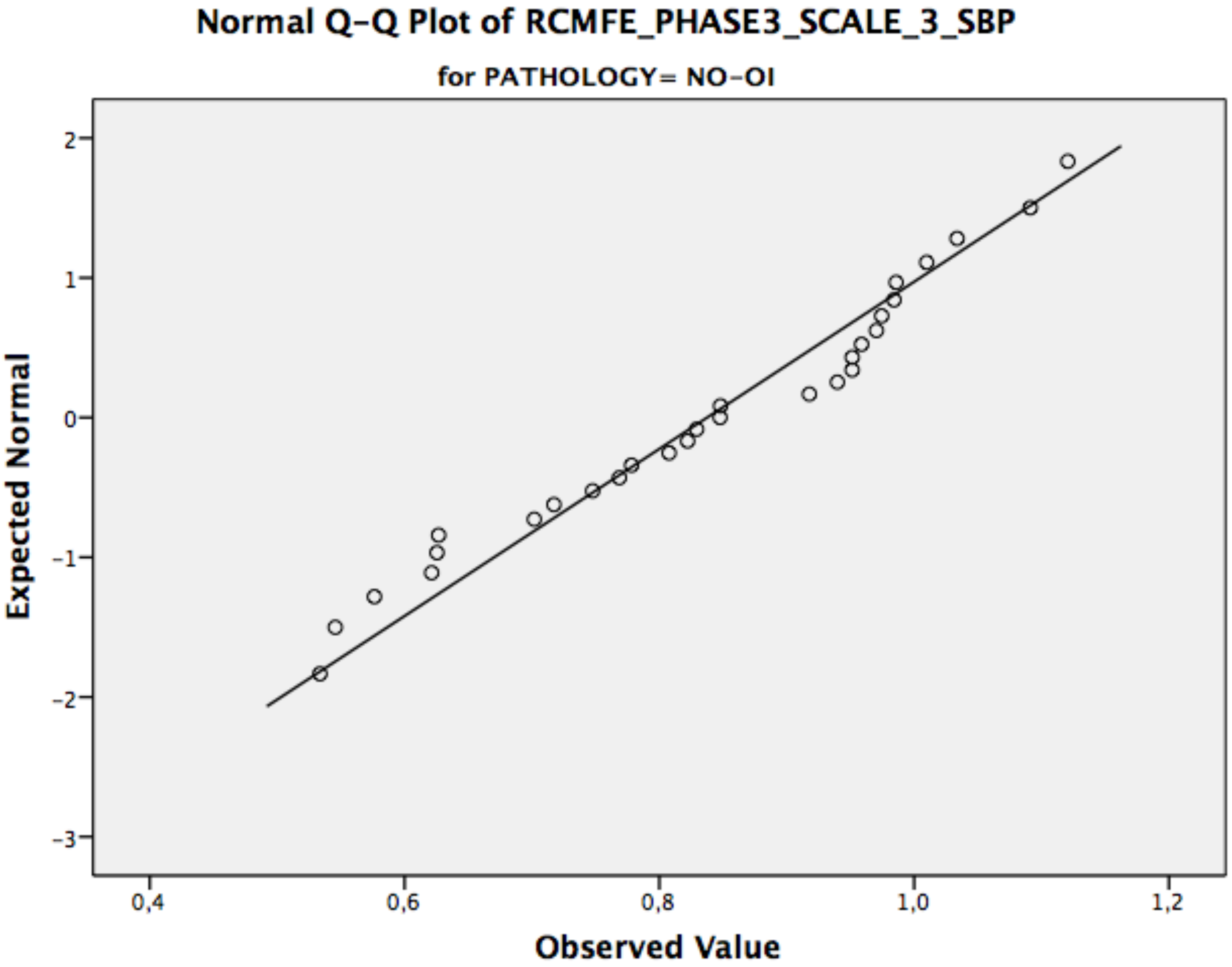

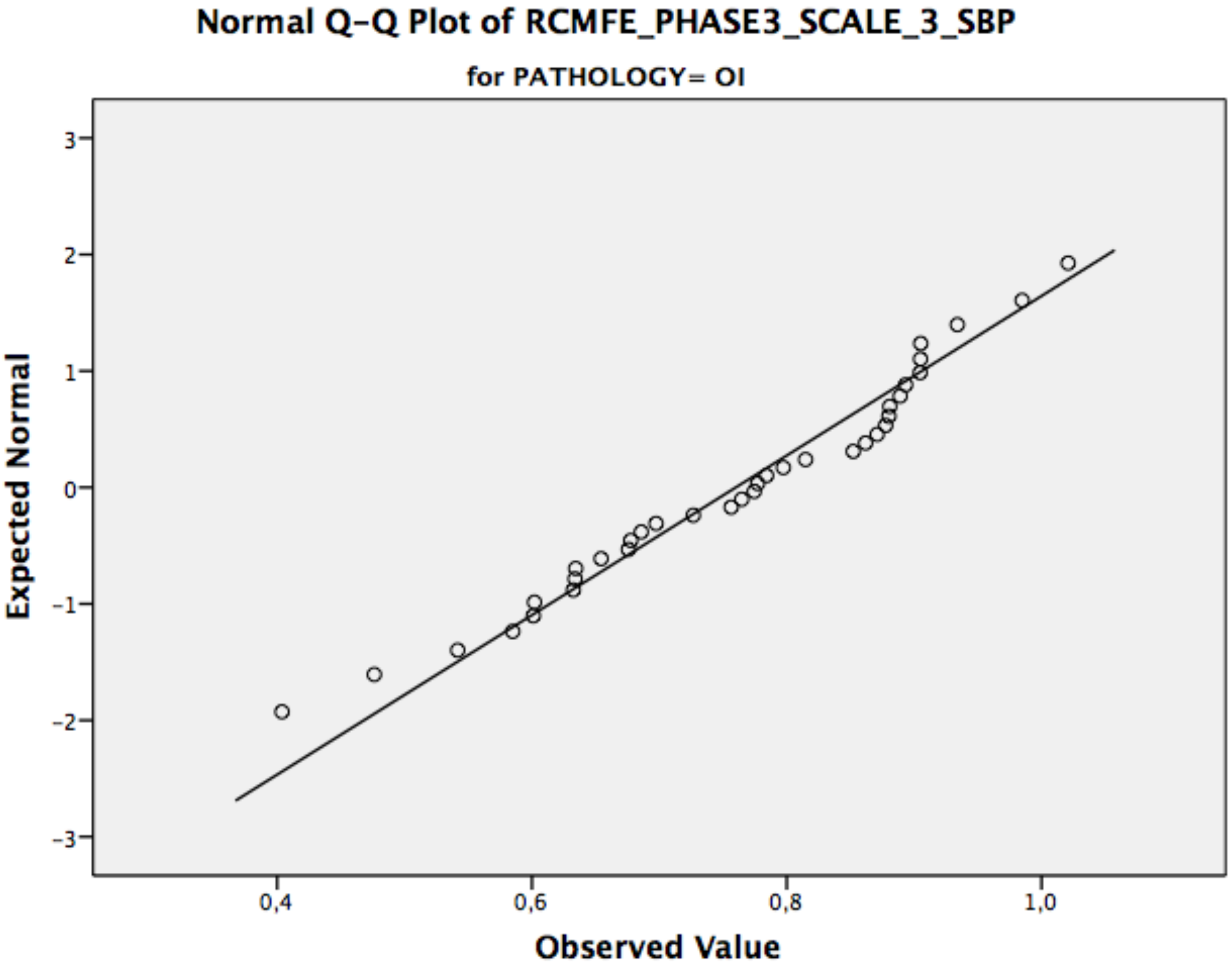
Normal Q-Q Plot of RCMFE_PHASE3_SCALE_3_SBP
for PATHOLOGY= OI
Expected Normal
Observed Value
3
2
1
0
-1
-2
-3
0,4
0,6
0,8
1,0

RCMFE_PHASE3_SCALE_4_SBP

Normal Q-Q Plots

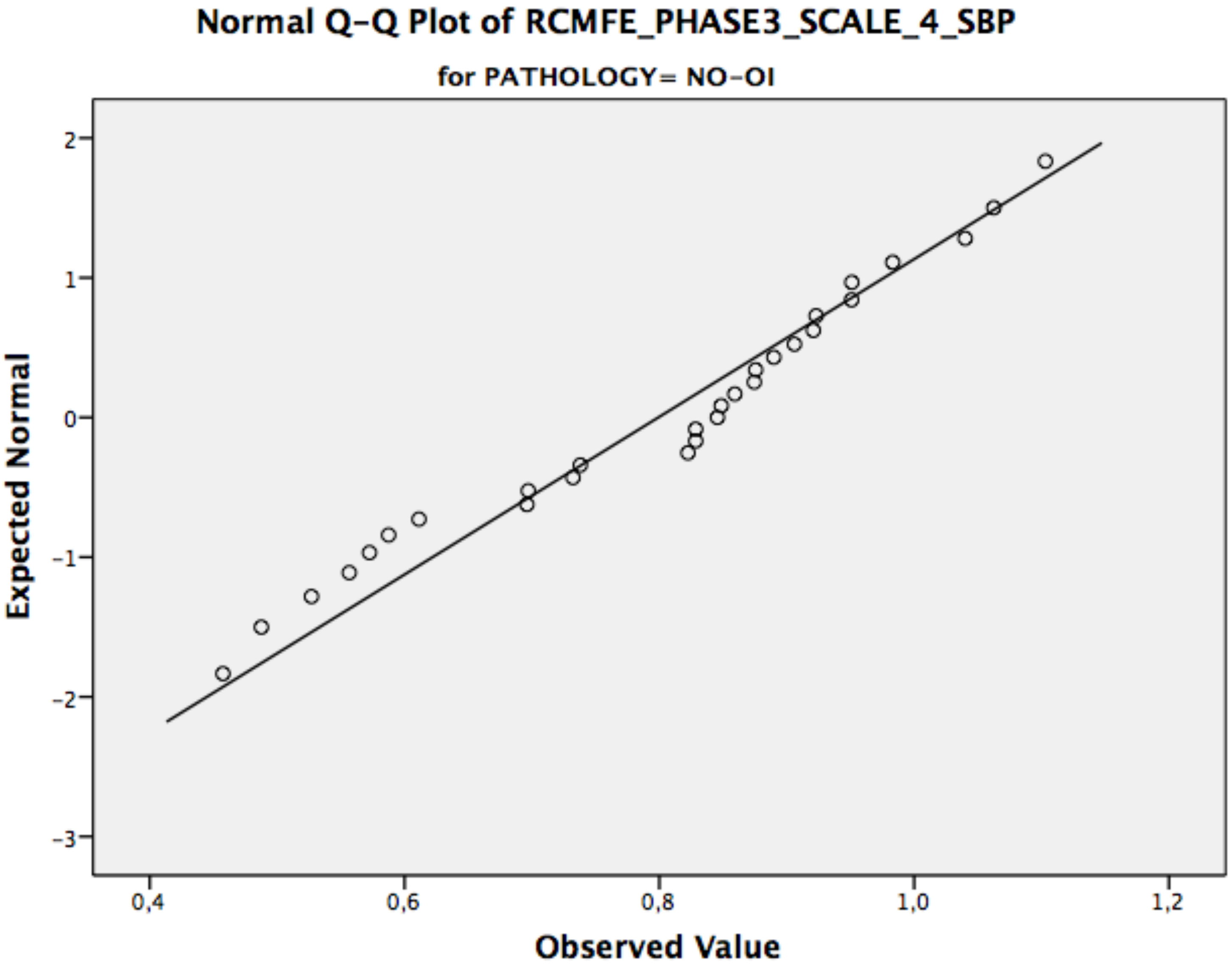

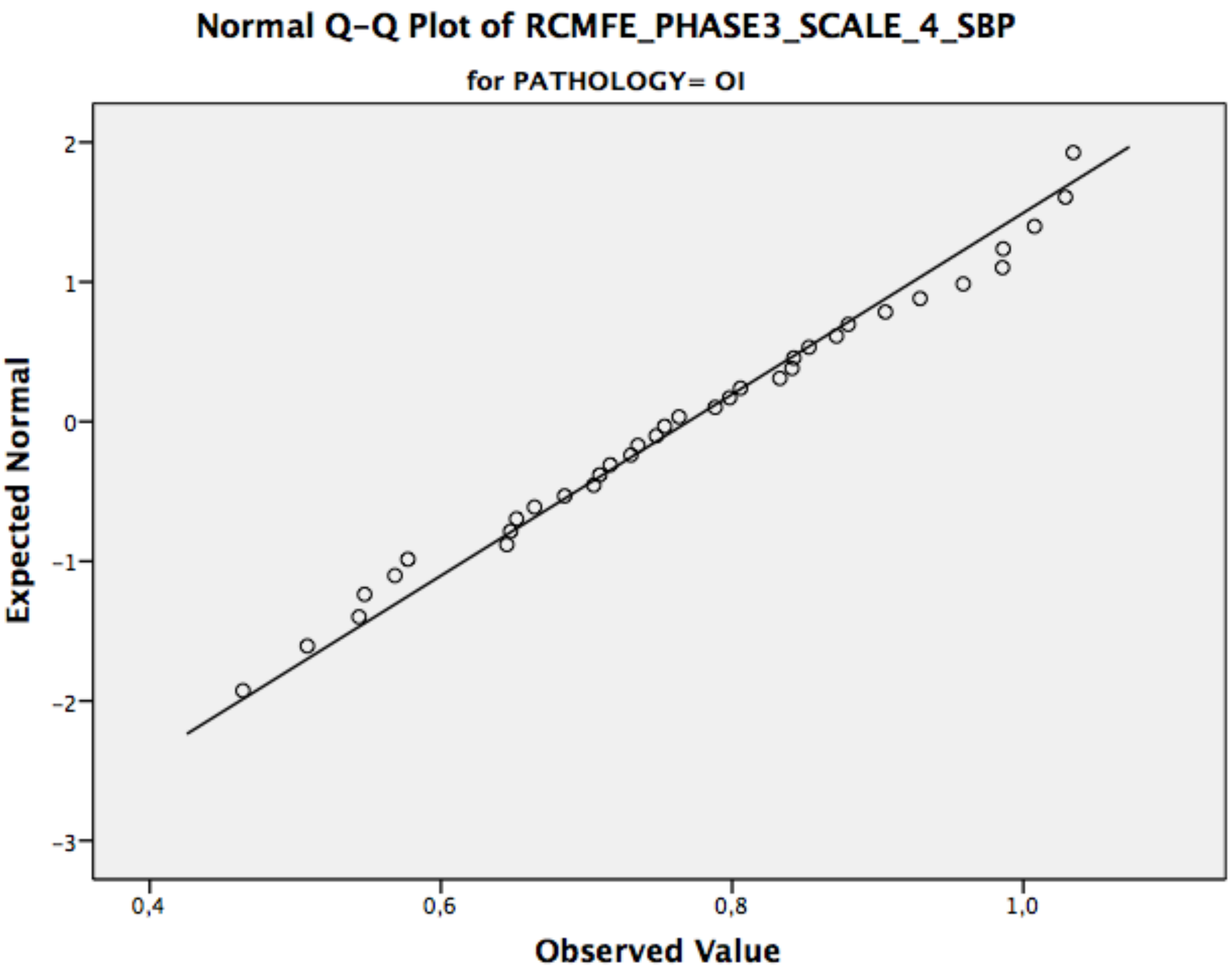

Normal Q-Q Plot of RCMFE_PHASE3_SCALE_4_SBP
for PATHOLOGY= OI
Expected Normal
Observed Value
2
1
0
−1
−2
−3
0,4
0,6
0,8
1,0

RCMFE_PHASE3_SCALE_5_SBP

Normal Q-Q Plots

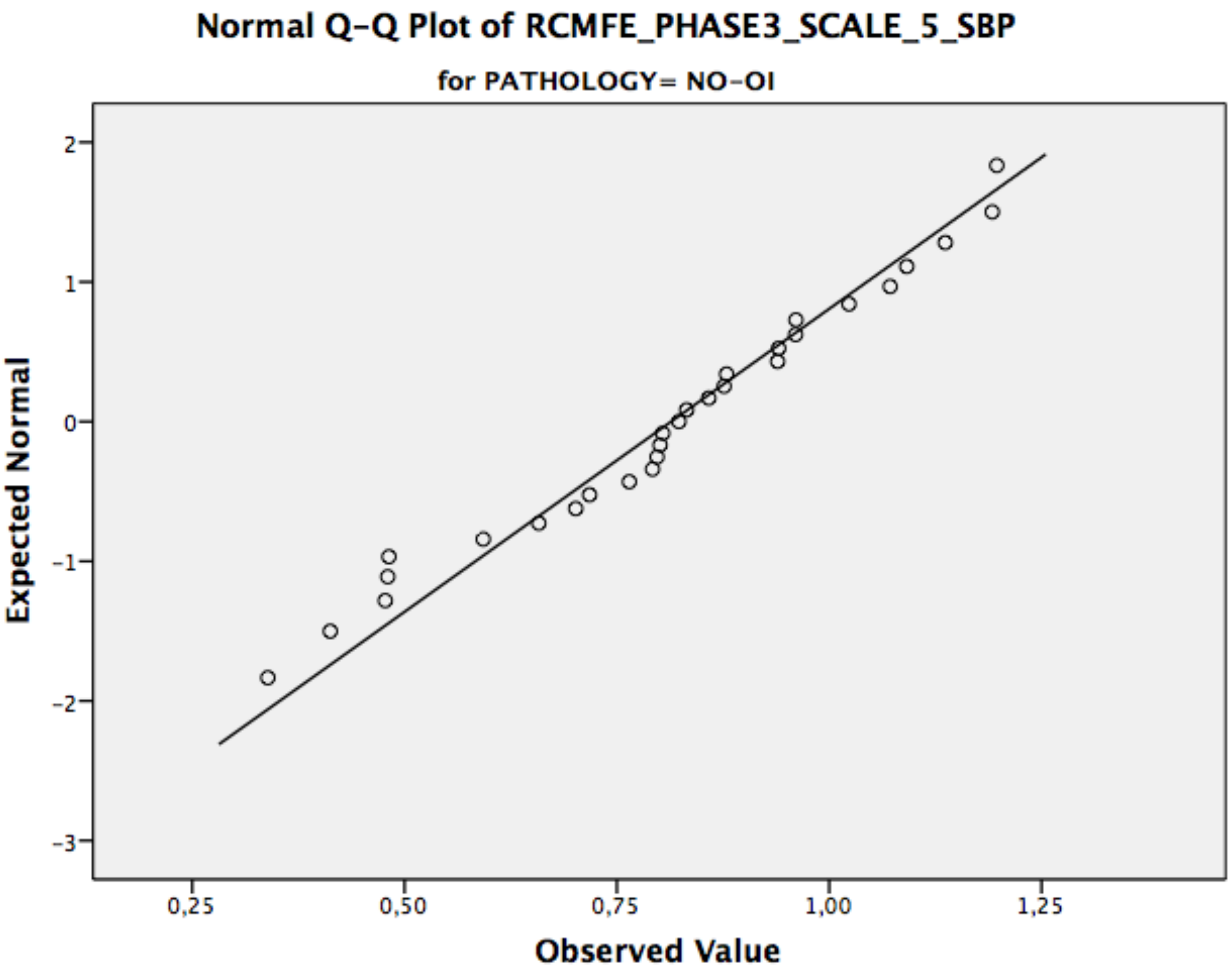

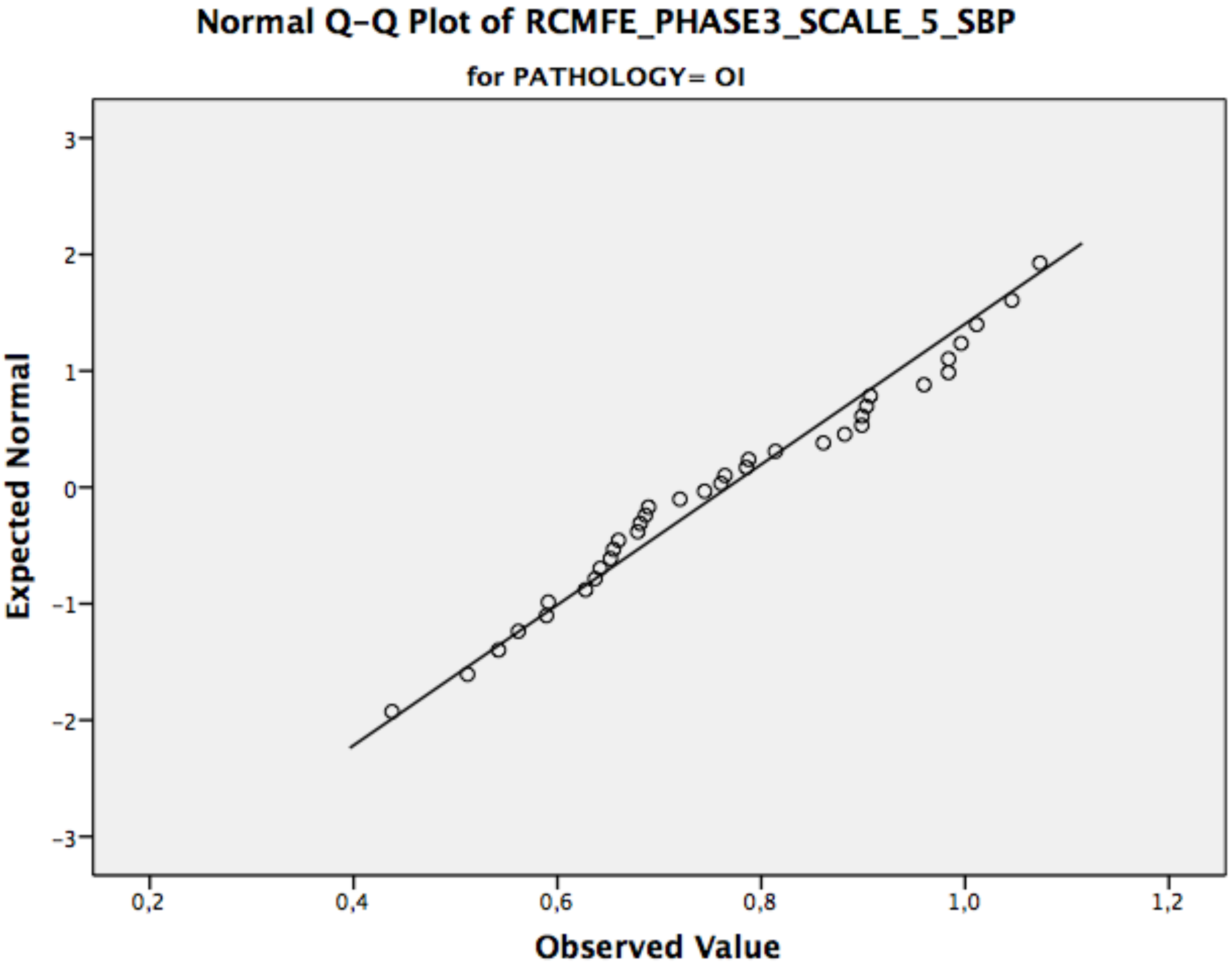
Normal Q-Q Plot of RCMFE_PHASE3_SCALE_5_SBP
for PATHOLOGY= OI
Expected Normal
3
2
1
0
−1
−2
−3
0,2
0,4
0,6
0,8
1,0
1,2
Observed Value

RCMFE_PHASE4_SCALE_1_SBP

Normal Q-Q Plots

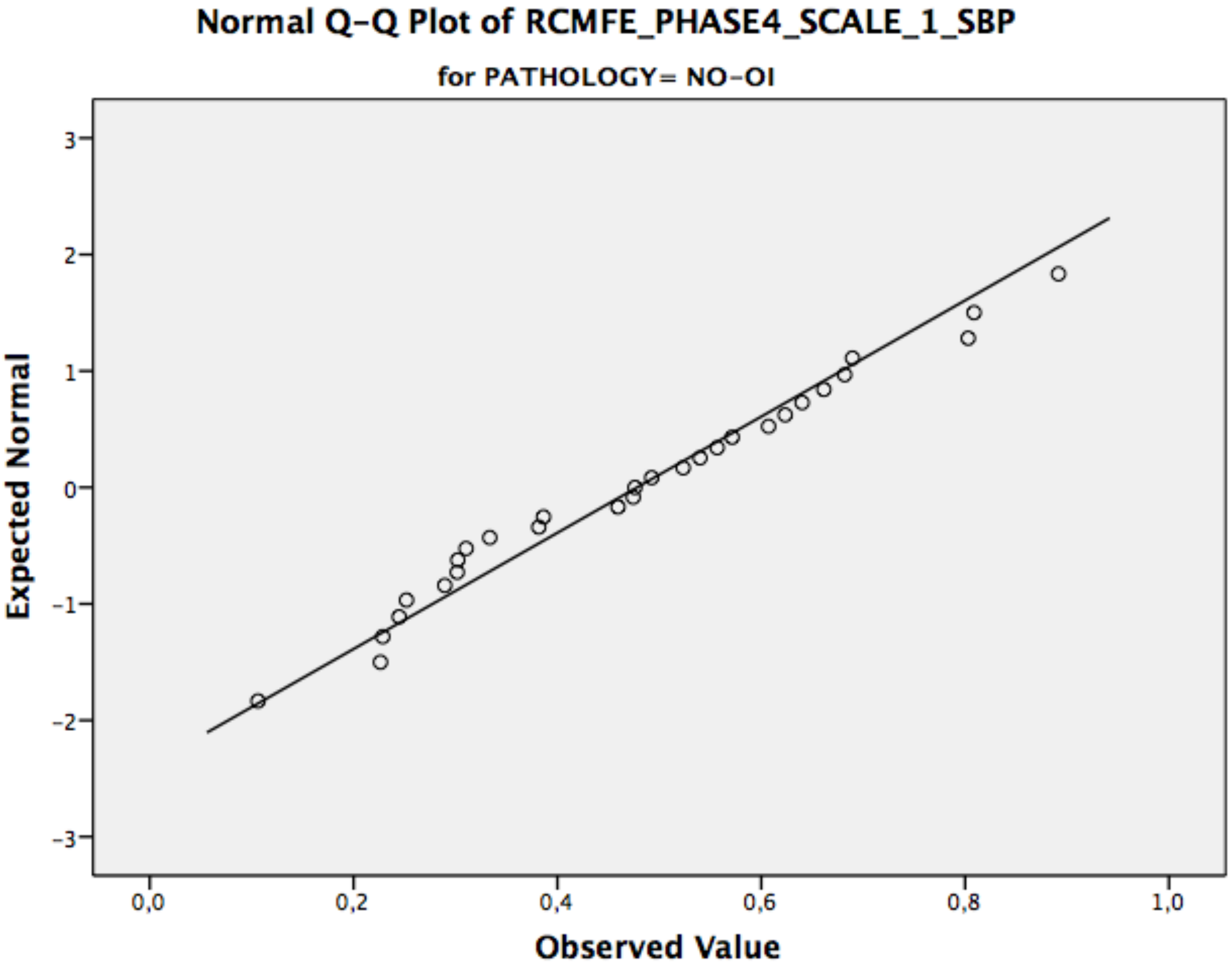

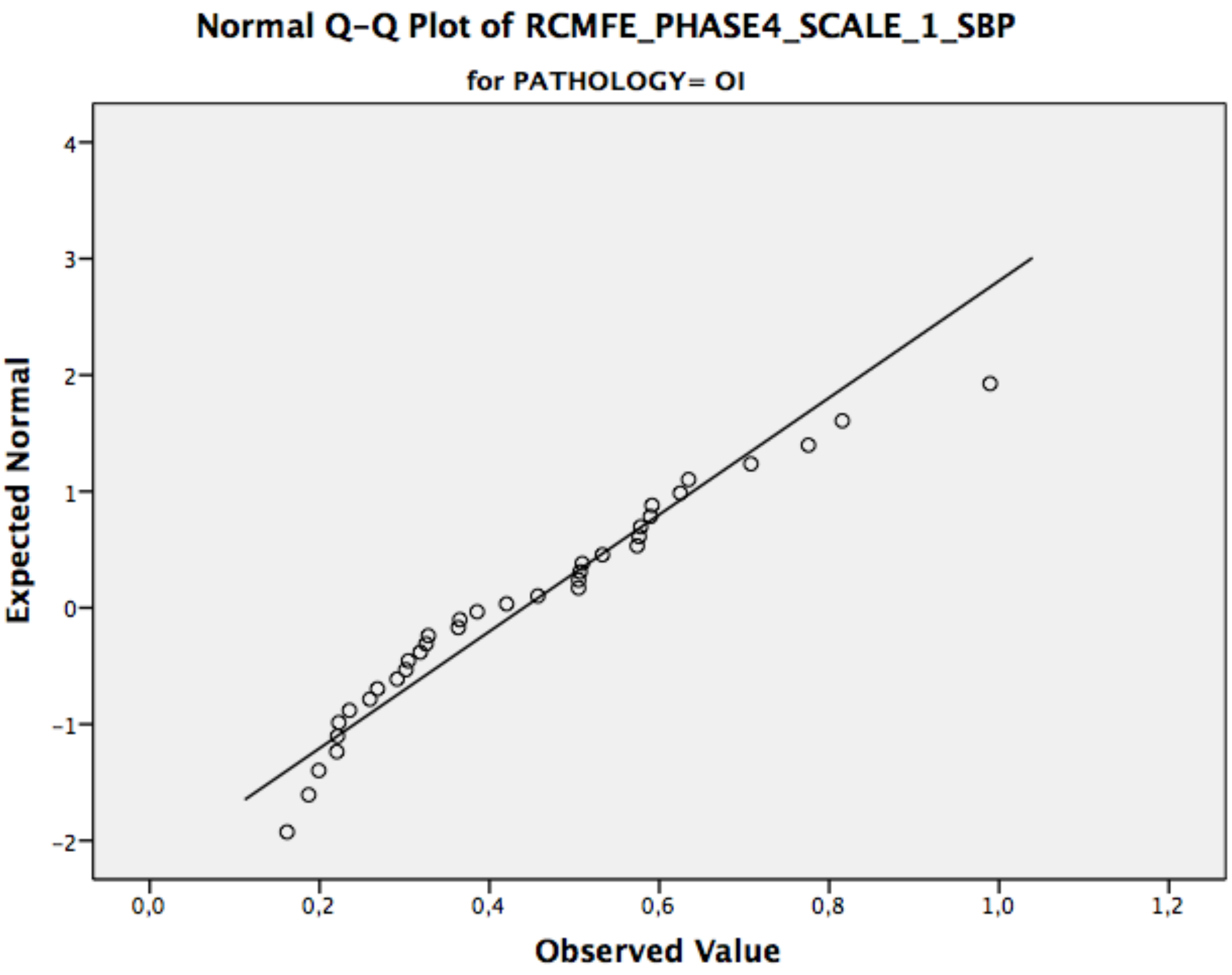
Normal Q-Q Plot of RCMFE_PHASE4_SCALE_1_SBP
for PATHOLOGY= OI
Expected Normal
4
3
2
1
0
-1
-2
0,0
0,2
0,4
0,6
0,8
1,0
1,2
Observed Value

RCMFE_PHASE4_SCALE_2_SBP

Normal Q-Q Plots

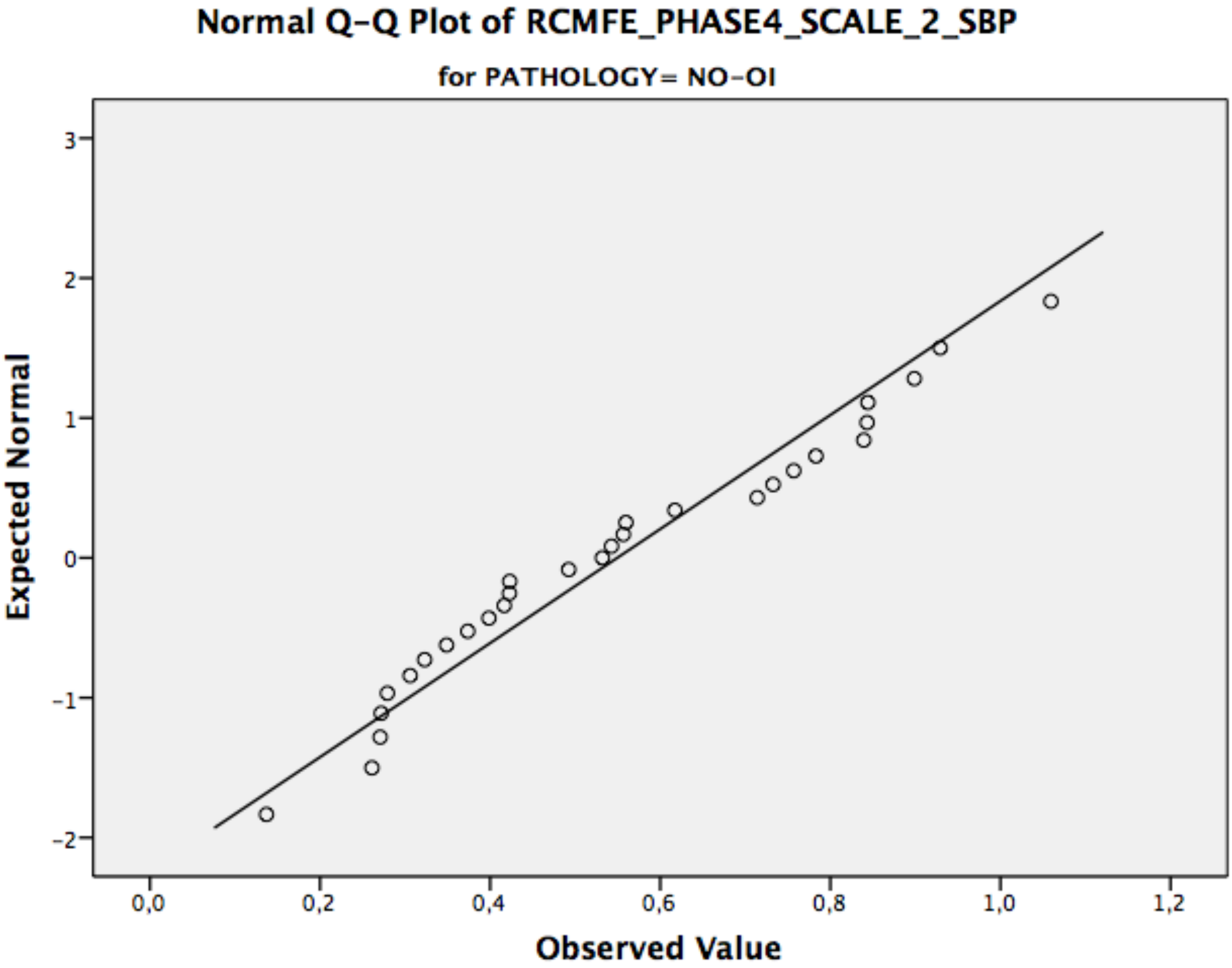

Normal Q-Q Plot of RCMFE_PHASE4_SCALE_2_SBP

for PATHOLOGY= OI

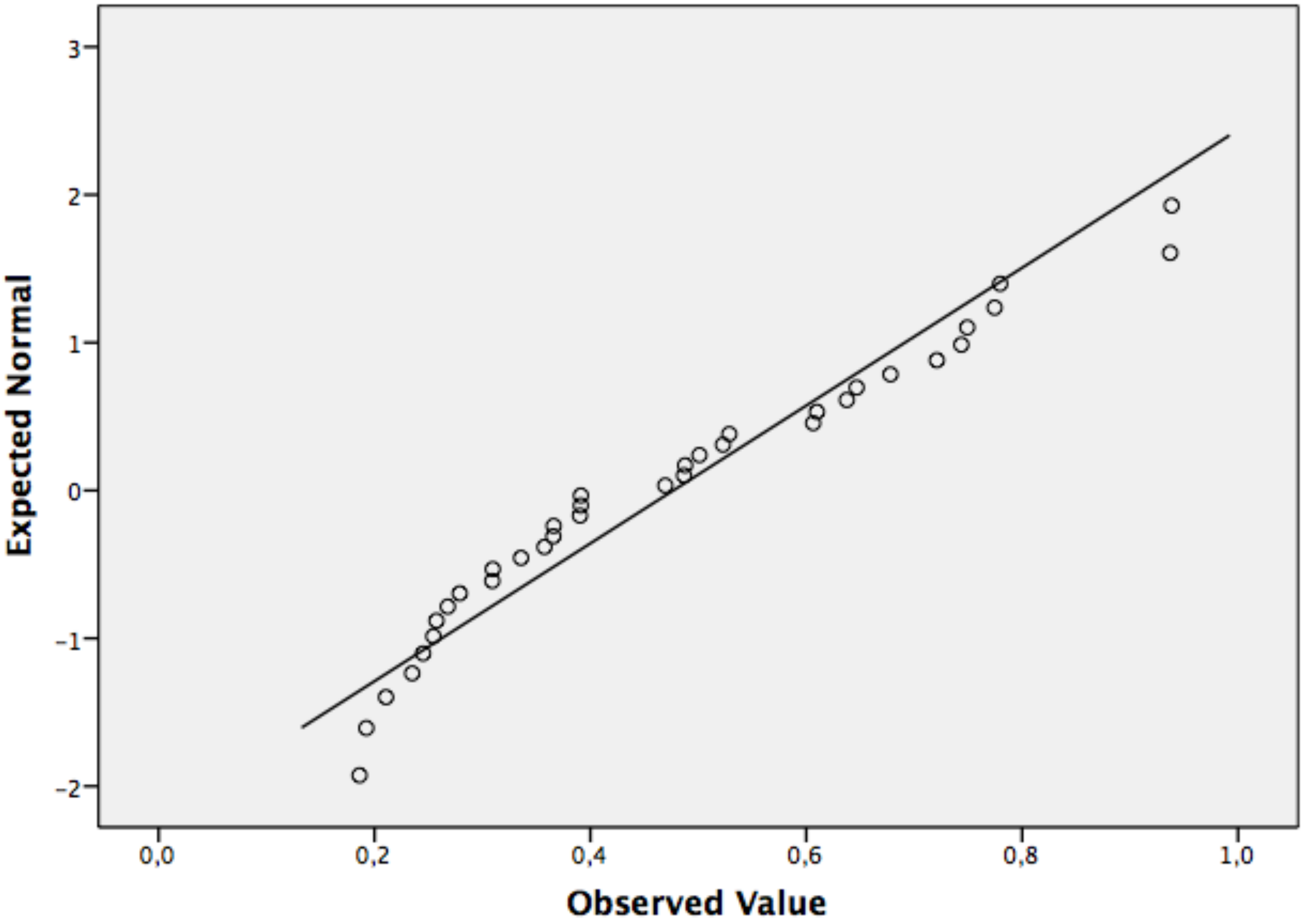

RCMFE_PHASE4_SCALE_3_SBP

Normal Q-Q Plots

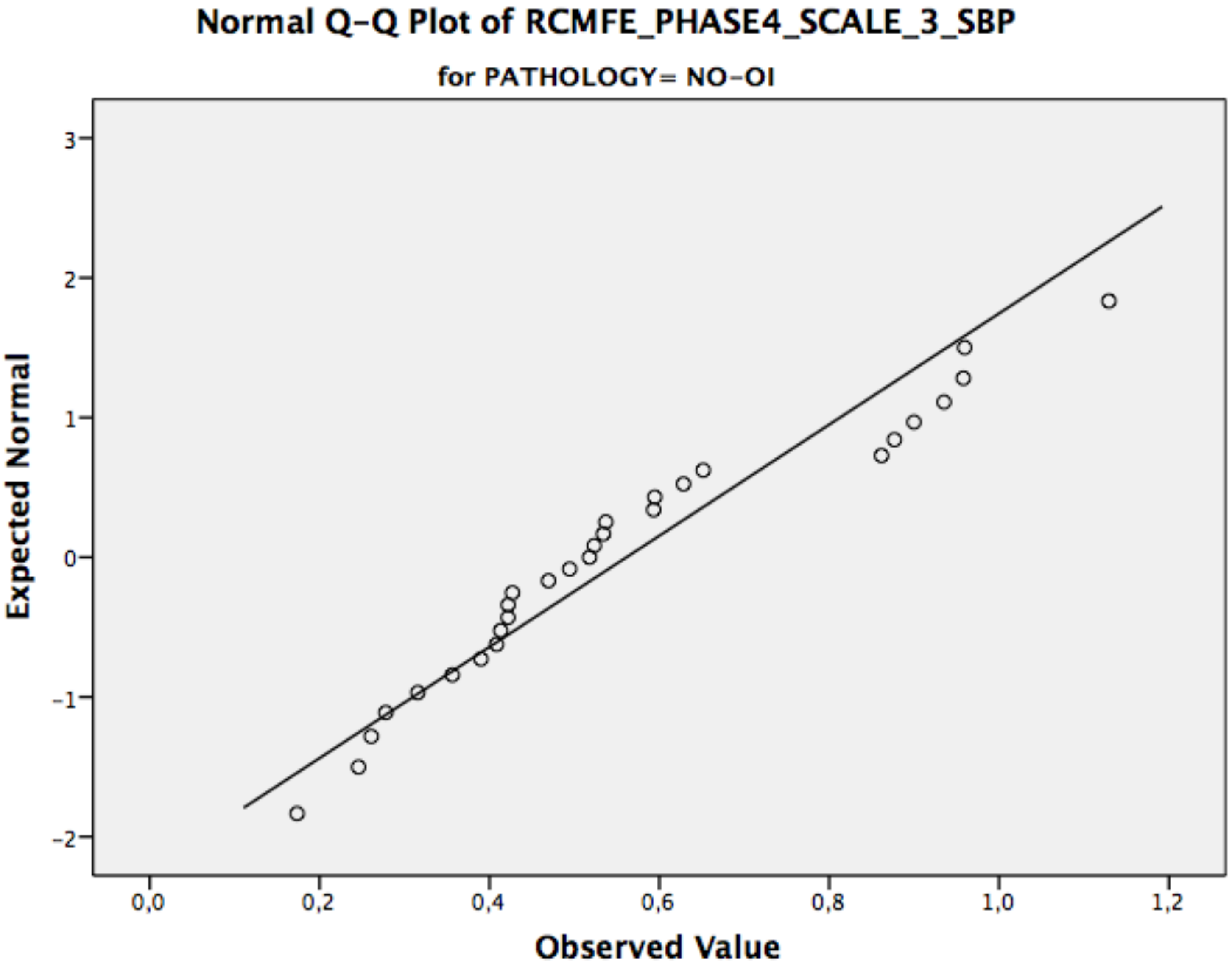

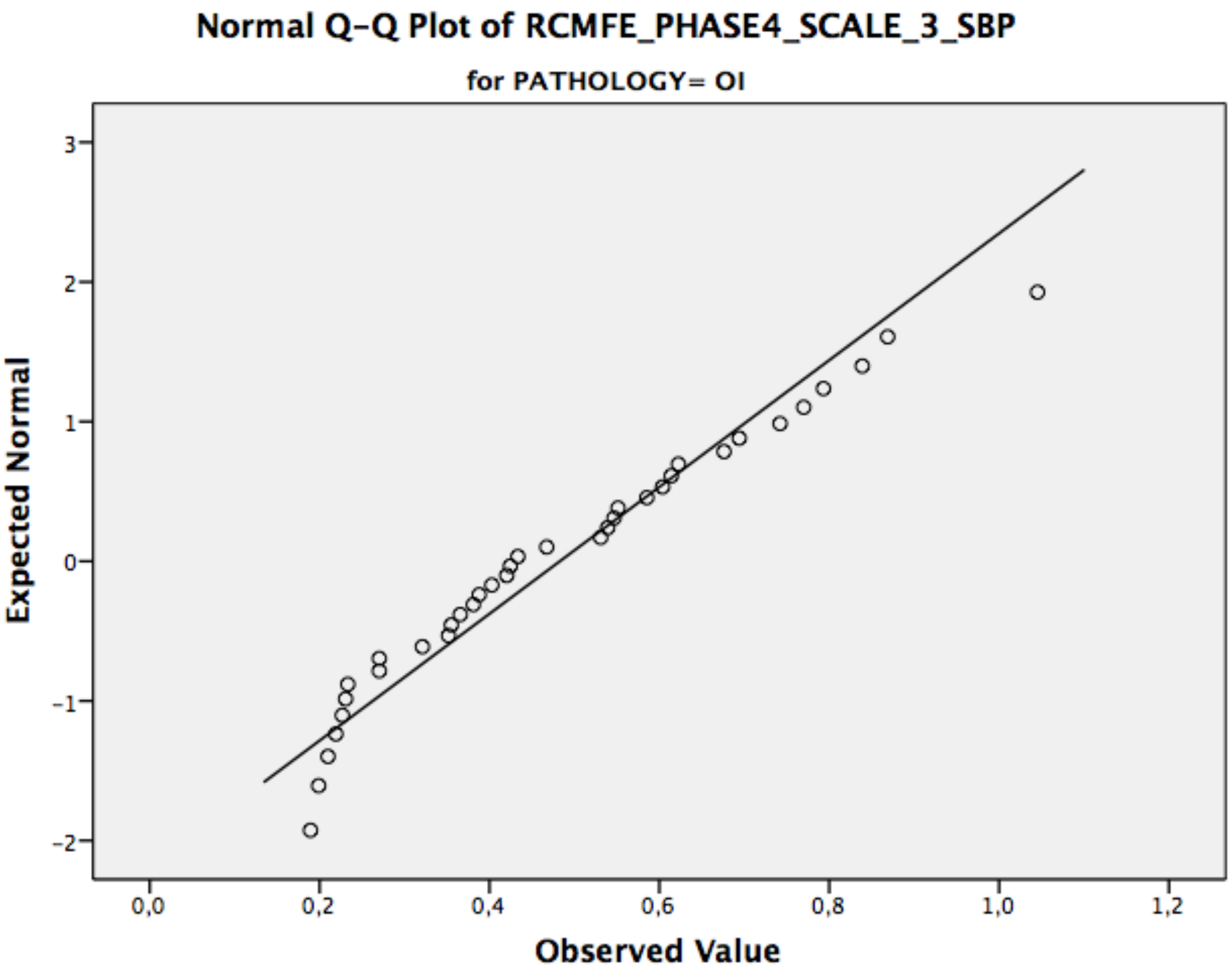

Normal Q-Q Plot of RCMFE_PHASE4_SCALE_3_SBP
for PATHOLOGY= OI
Expected Normal
3
2
1
0
−1
−2
0,0
0,2
0,4
0,6
0,8
1,0
1,2
Observed Value

RCMFE_PHASE4_SCALE_4_SBP

Normal Q-Q Plots

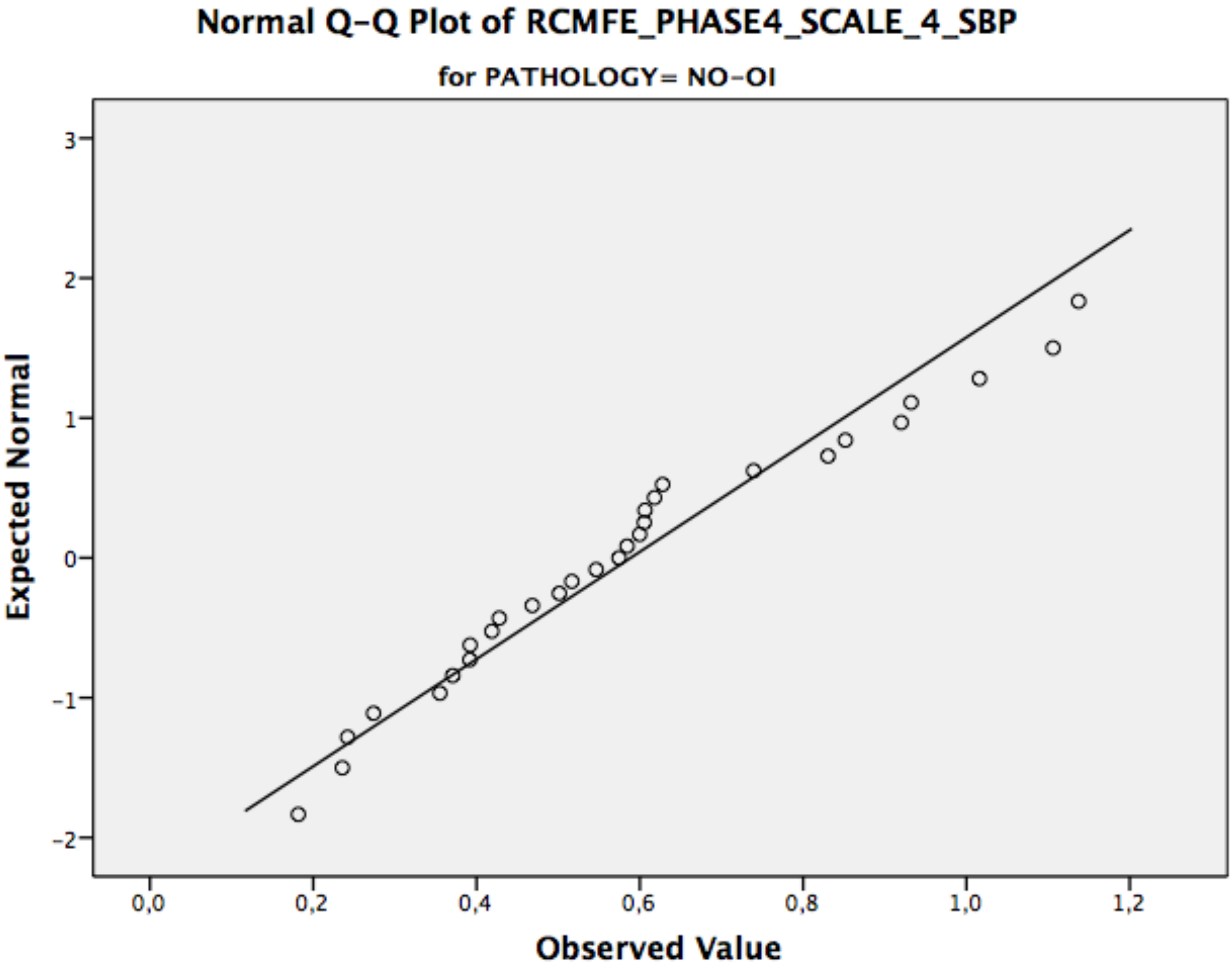

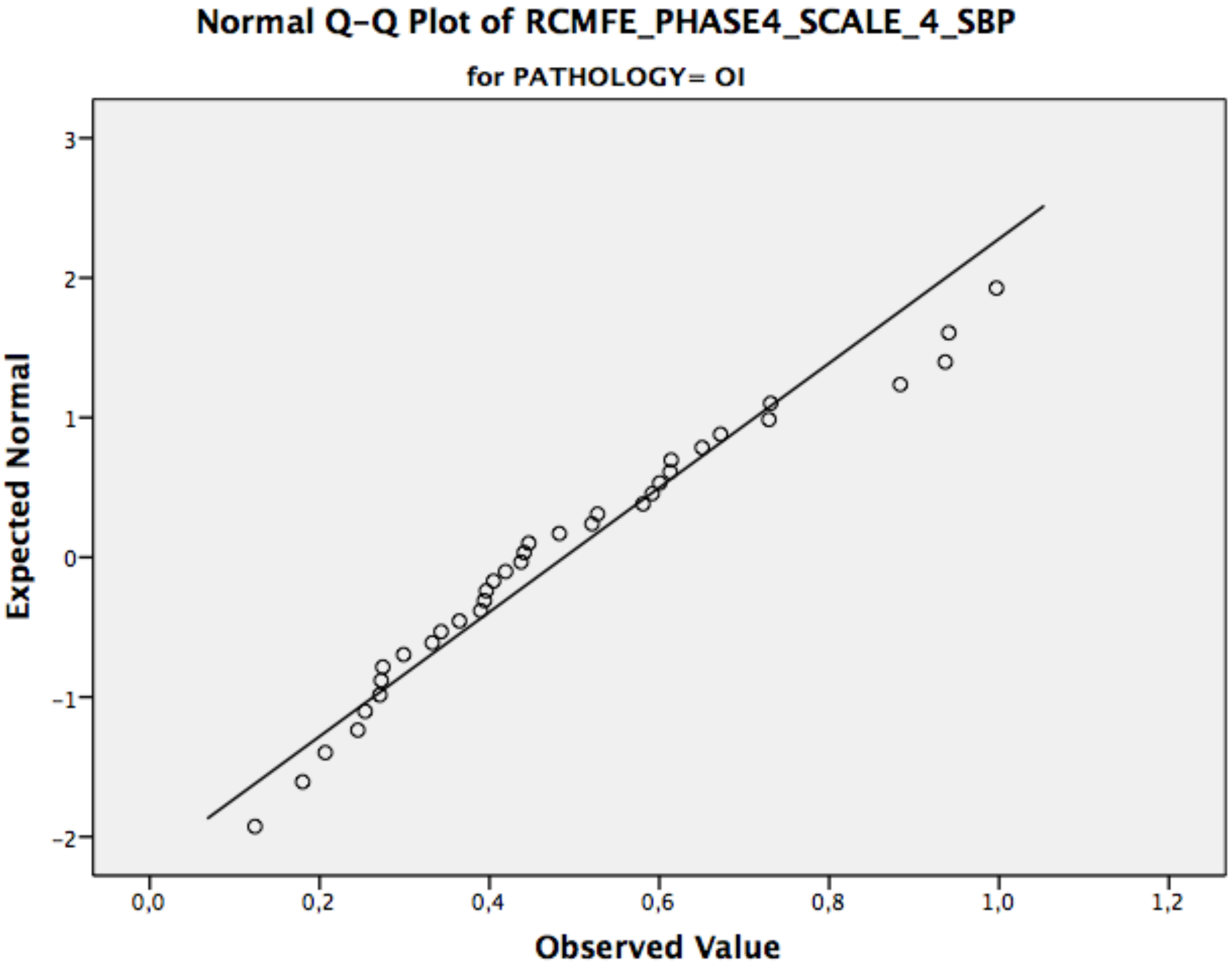

Normal Q-Q Plot of RCMFE_PHASE4_SCALE_4_SBP
for PATHOLOGY= OI
Expected Normal
3
2
1
0
−1
−2
0,0
0,2
0,4
0,6
0,8
1,0
1,2
Observed Value

RCMFE_PHASE4_SCALE_5_SBP

Normal Q-Q Plots

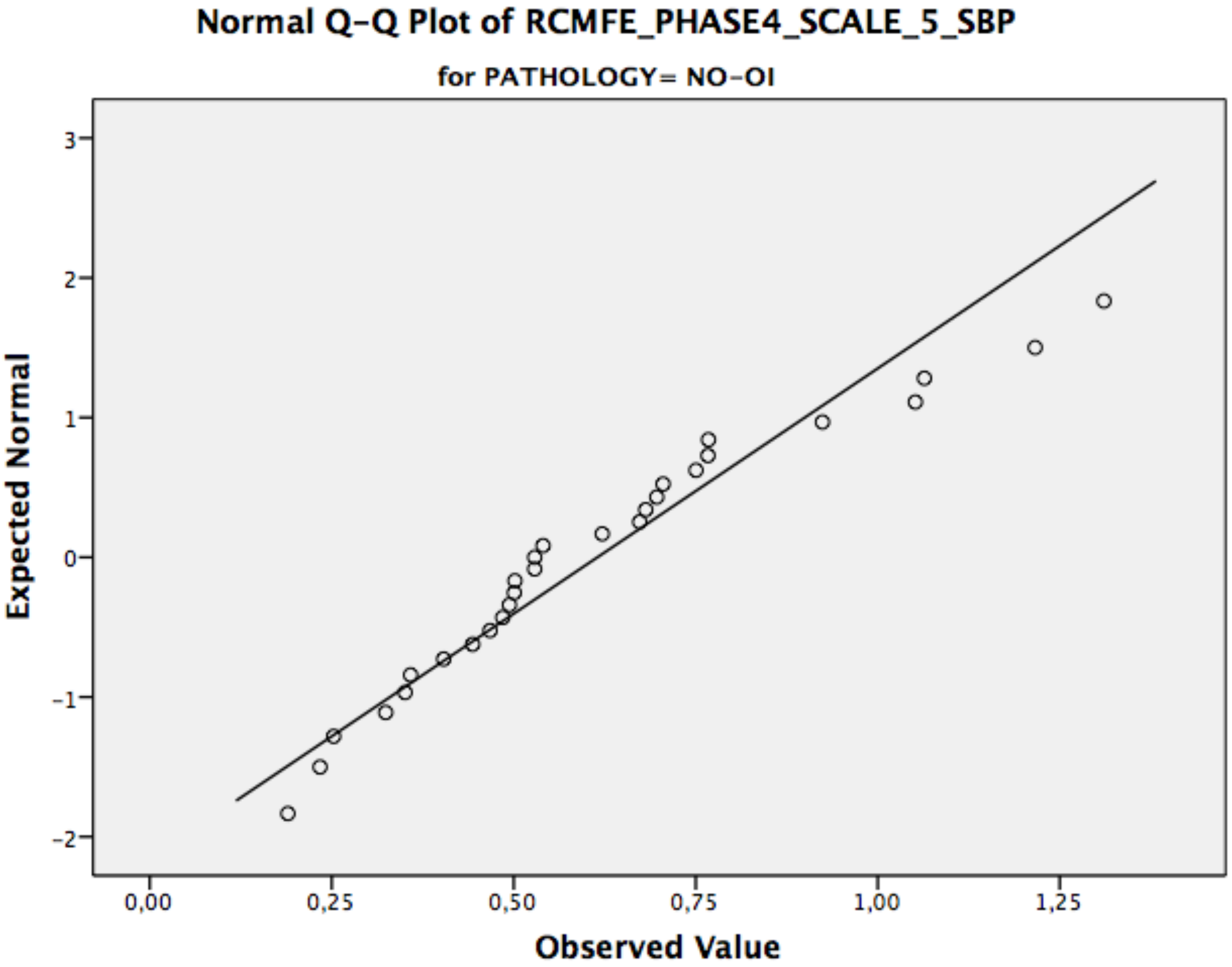

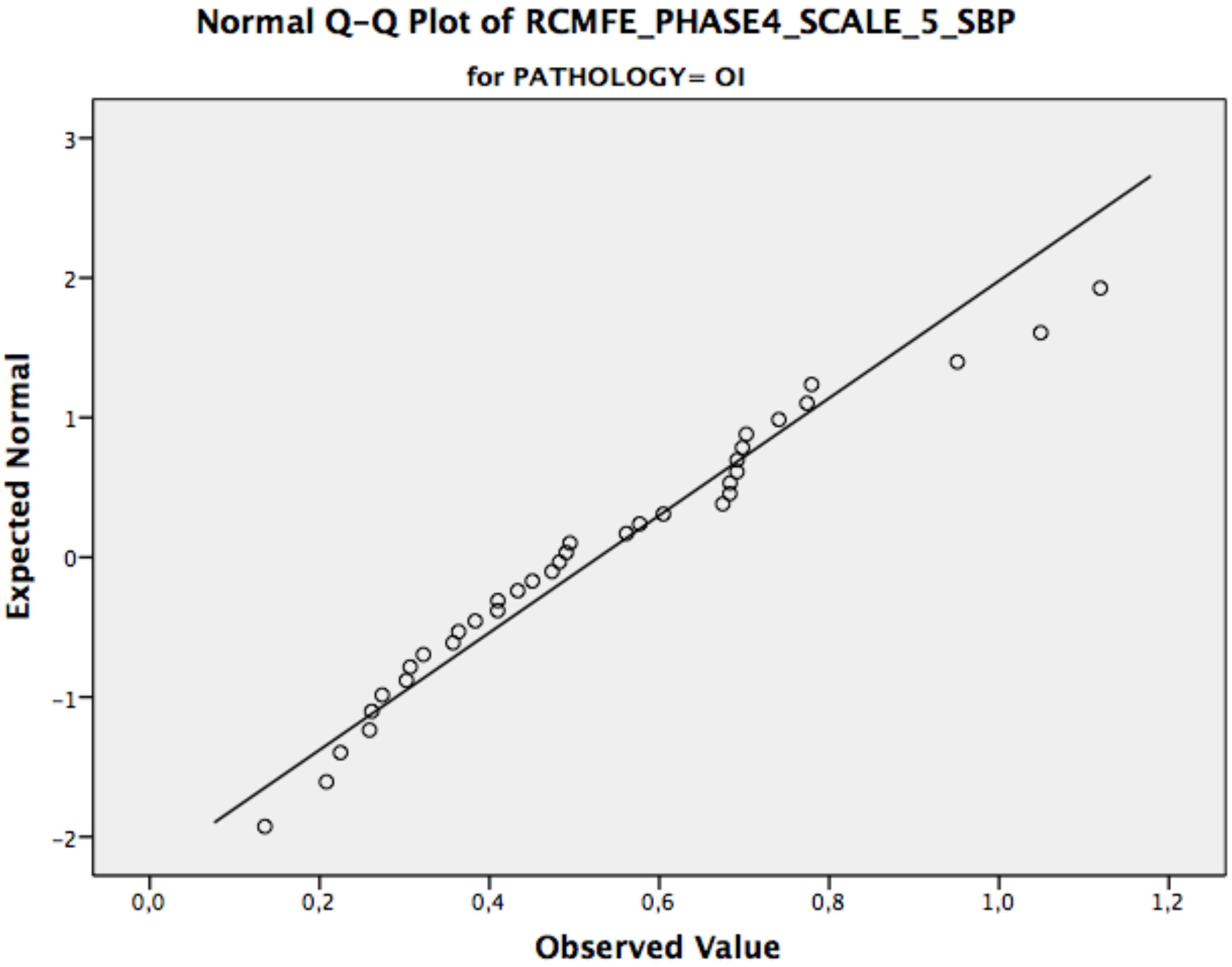

Normal Q-Q Plot of RCMFE_PHASE4_SCALE_5_SBP
for PATHOLOGY= OI
Expected Normal
Observed Value
3
2
1
0
-1
-2
0,0
0,2
0,4
0,6
0,8
1,0
1,2

RCMFE_PHASE5_SCALE_1_SBP

Normal Q-Q Plots

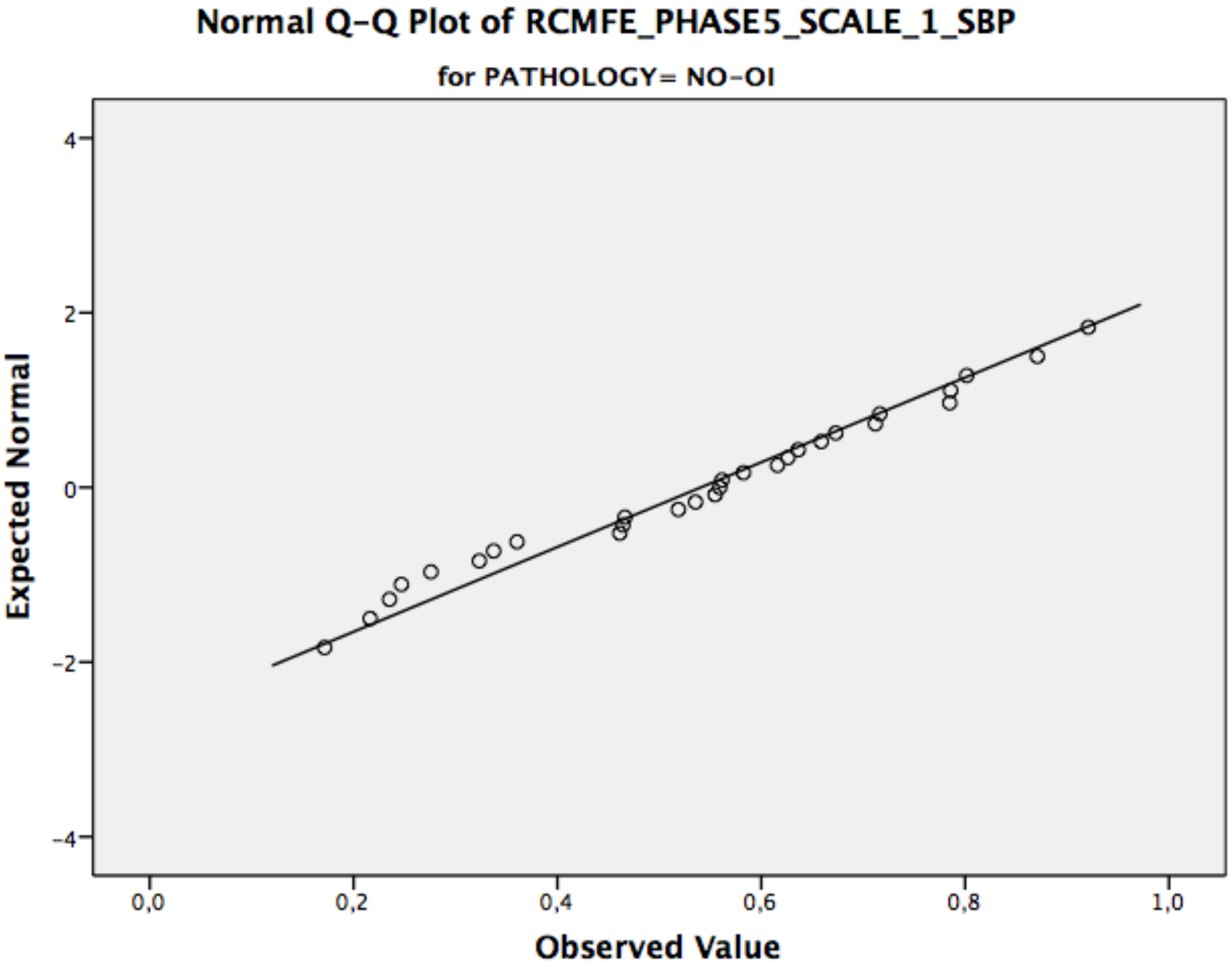

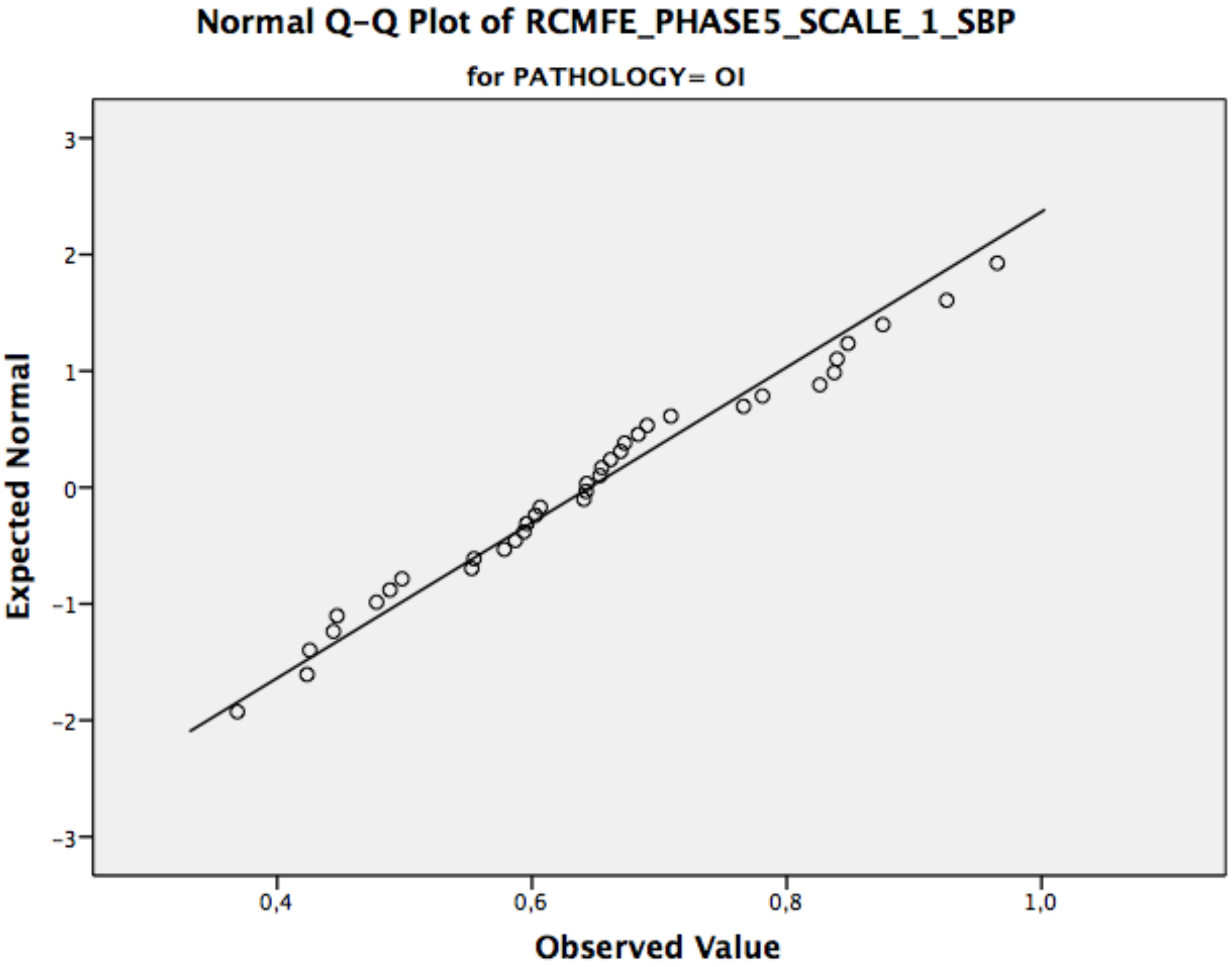
Normal Q-Q Plot of RCMFE_PHASE5_SCALE_1_SBP
for PATHOLOGY= OI
Expected Normal
Observed Value
3
2
1
0
-1
-2
-3
0,4
0,6
0,8
1,0

RCMFE_PHASE5_SCALE_2_SBP

Normal Q-Q Plots

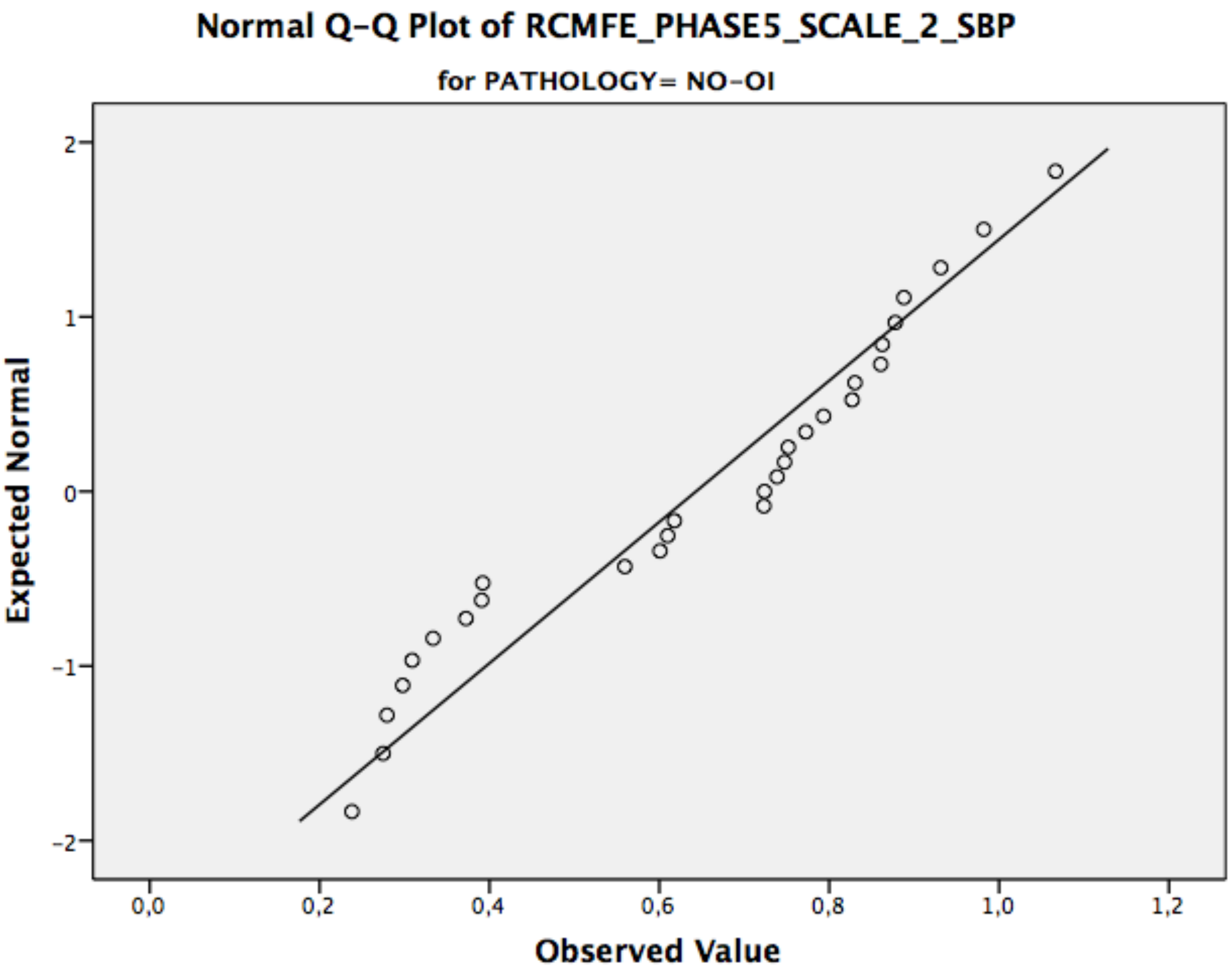

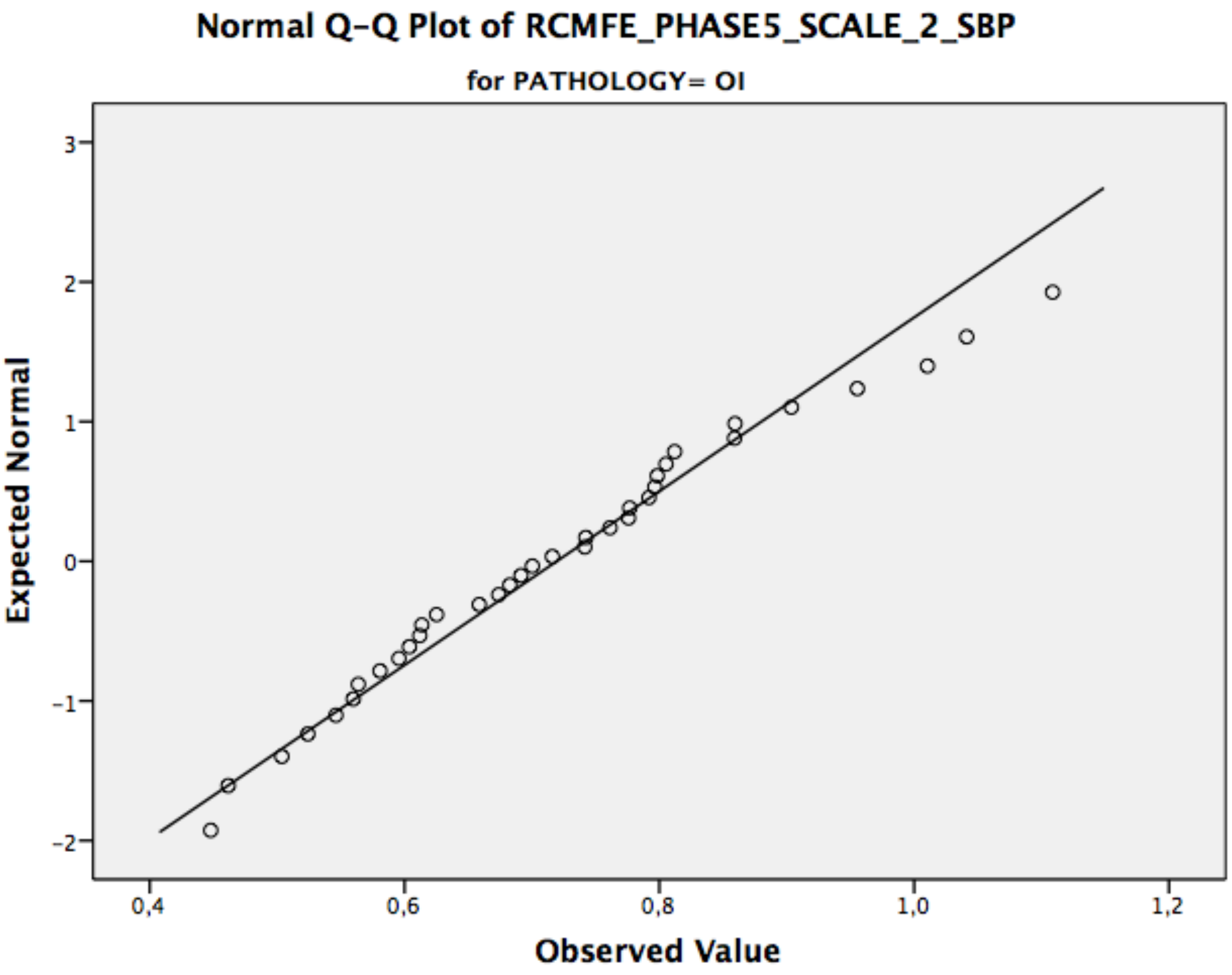

Normal Q-Q Plot of RCMFE_PHASE5_SCALE_2_SBP
for PATHOLOGY= OI
Expected Normal
Observed Value
3
2
1
0
−1
−2
0,4
0,6
0,8
1,0
1,2

RCMFE_PHASE5_SCALE_3_SBP

Normal Q-Q Plots

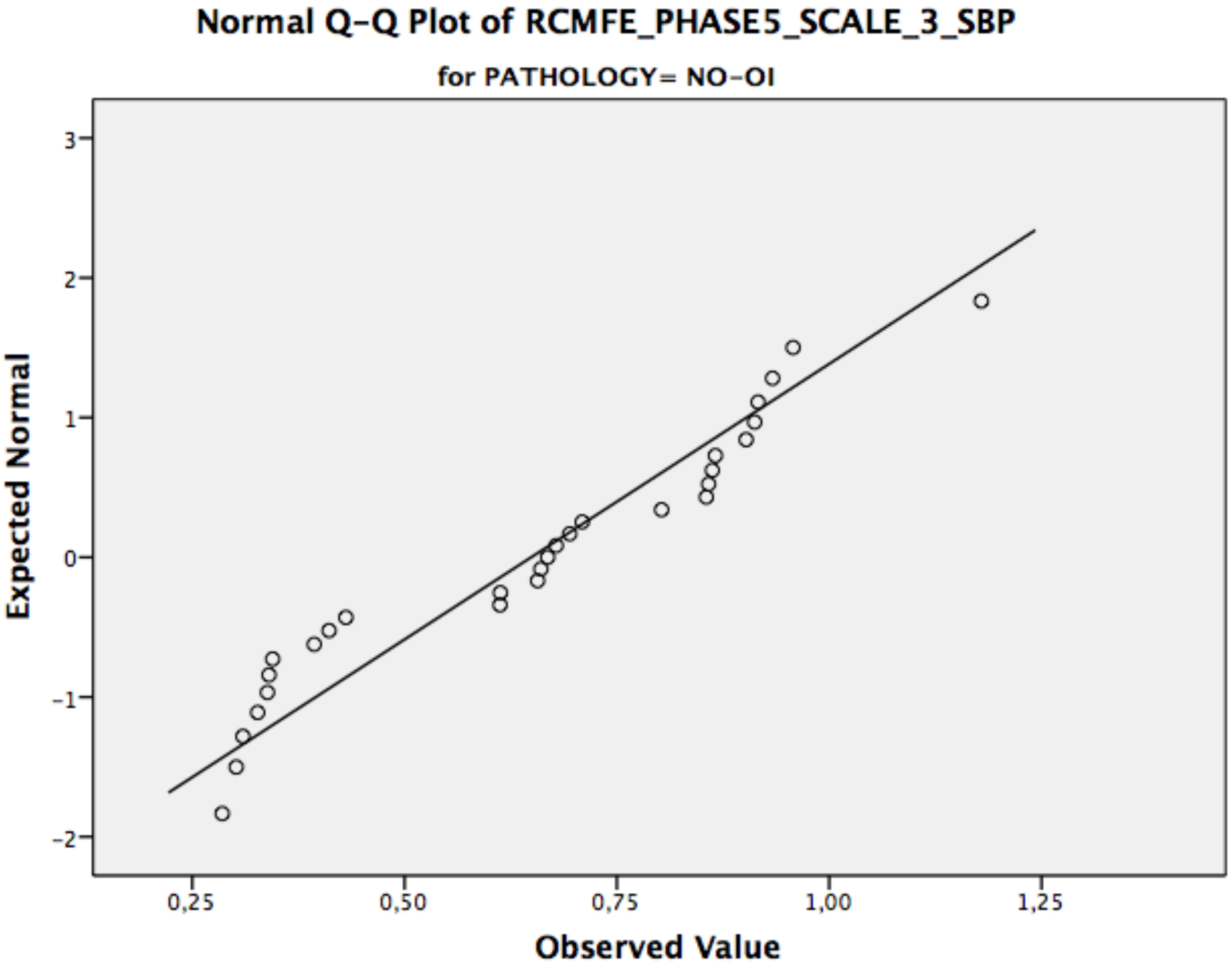

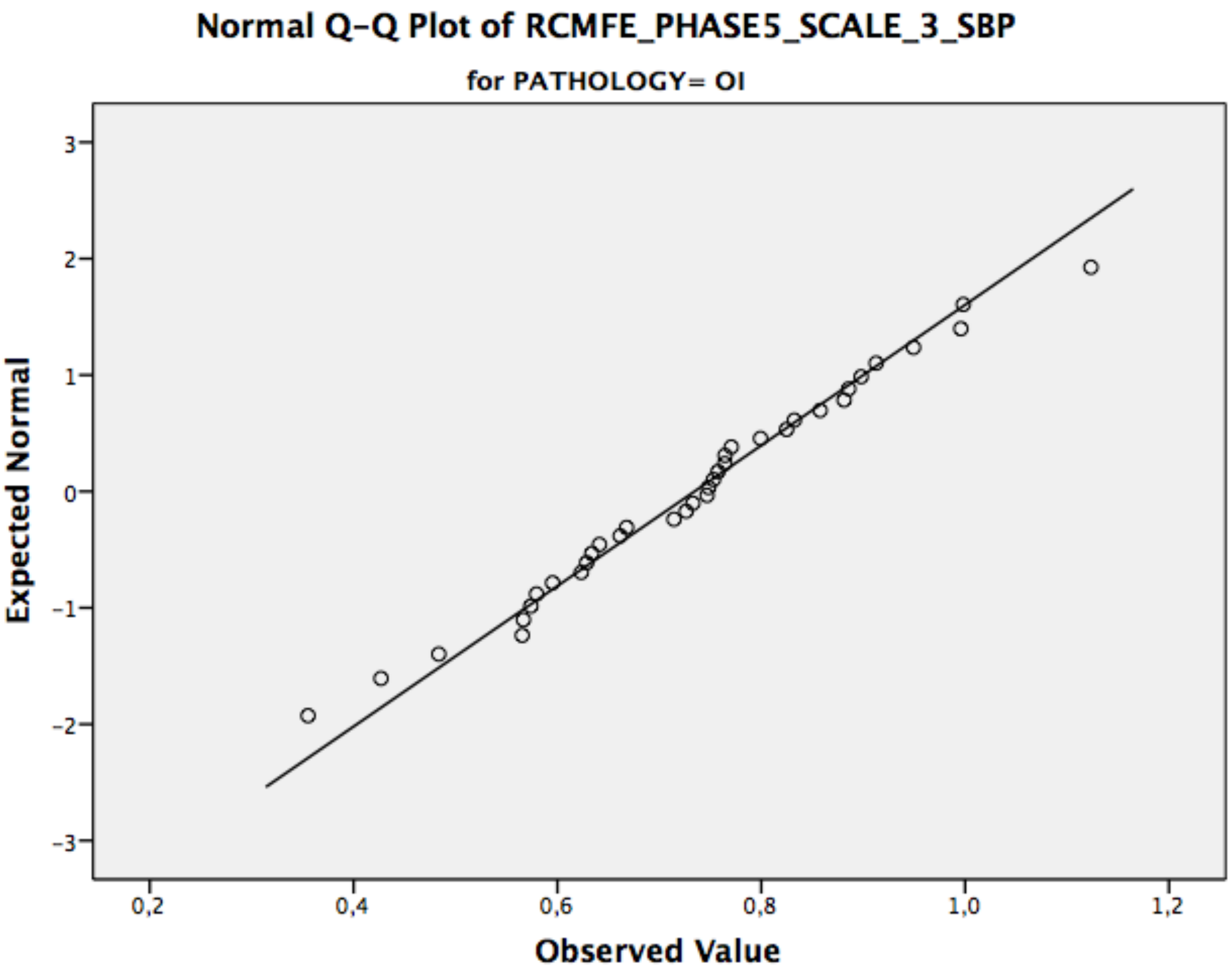
Normal Q-Q Plot of RCMFE_PHASE5_SCALE_3_SBP
for PATHOLOGY= OI
Expected Normal
3
2
1
0
-1
-2
-3
0,2
0,4
0,6
0,8
1,0
1,2
Observed Value

RCMFE_PHASE5_SCALE_4_SBP

Normal Q-Q Plots

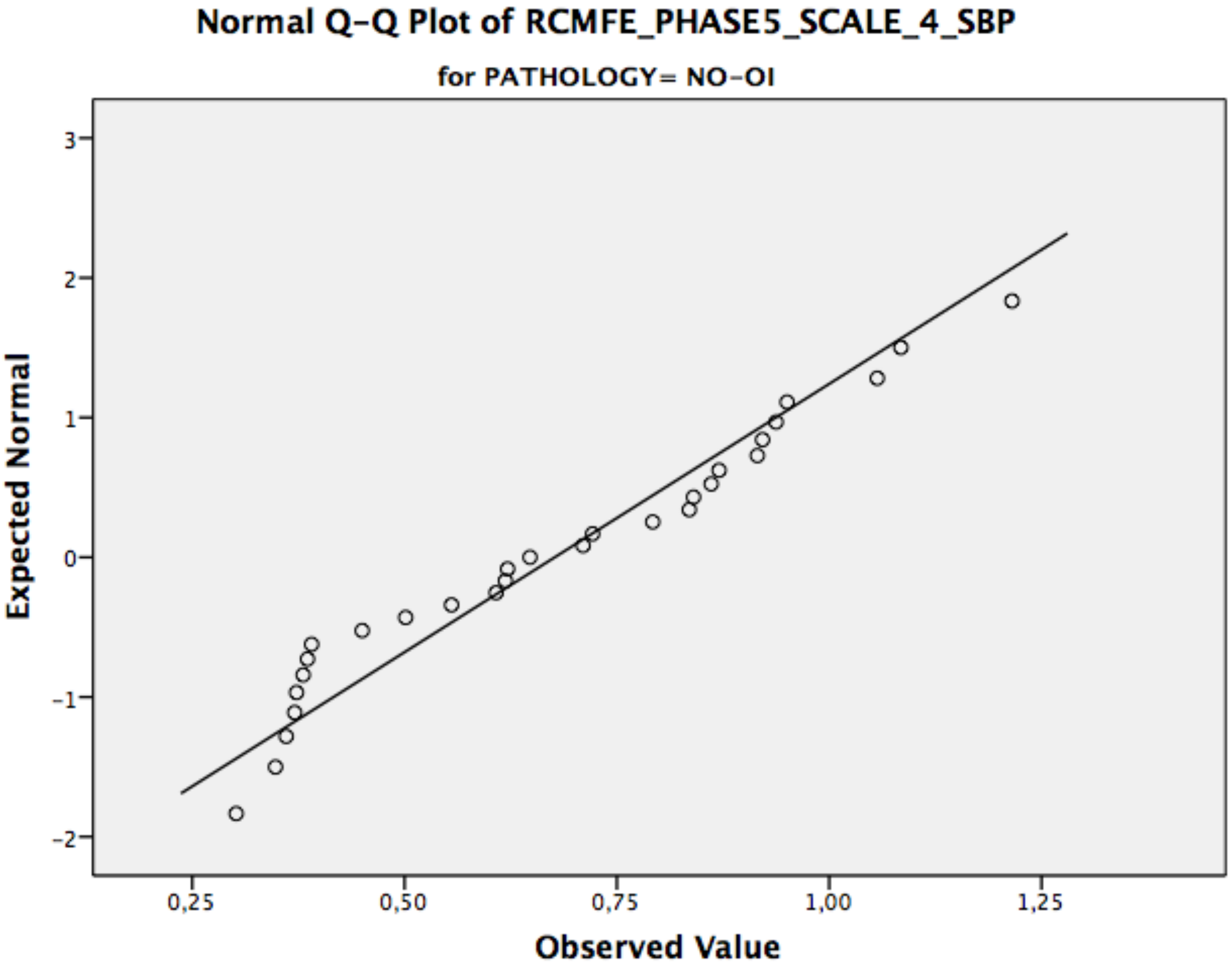

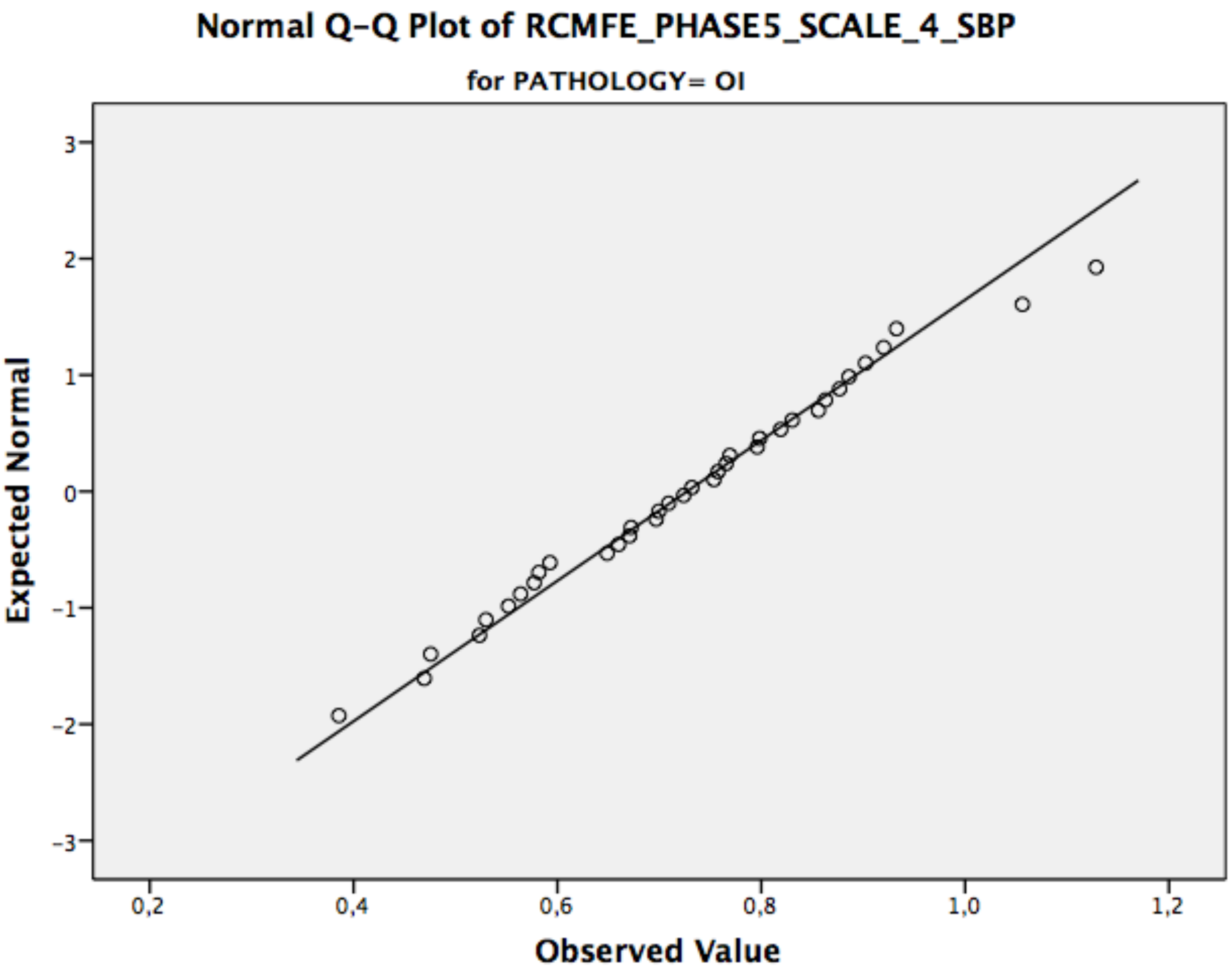
Normal Q-Q Plot of RCMFE_PHASE5_SCALE_4_SBP
for PATHOLOGY= OI
Expected Normal
3
2
1
0
-1
-2
-3
0,2
0,4
0,6
0,8
1,0
1,2
Observed Value

RCMFE_PHASE5_SCALE_5_SBP

Normal Q-Q Plots

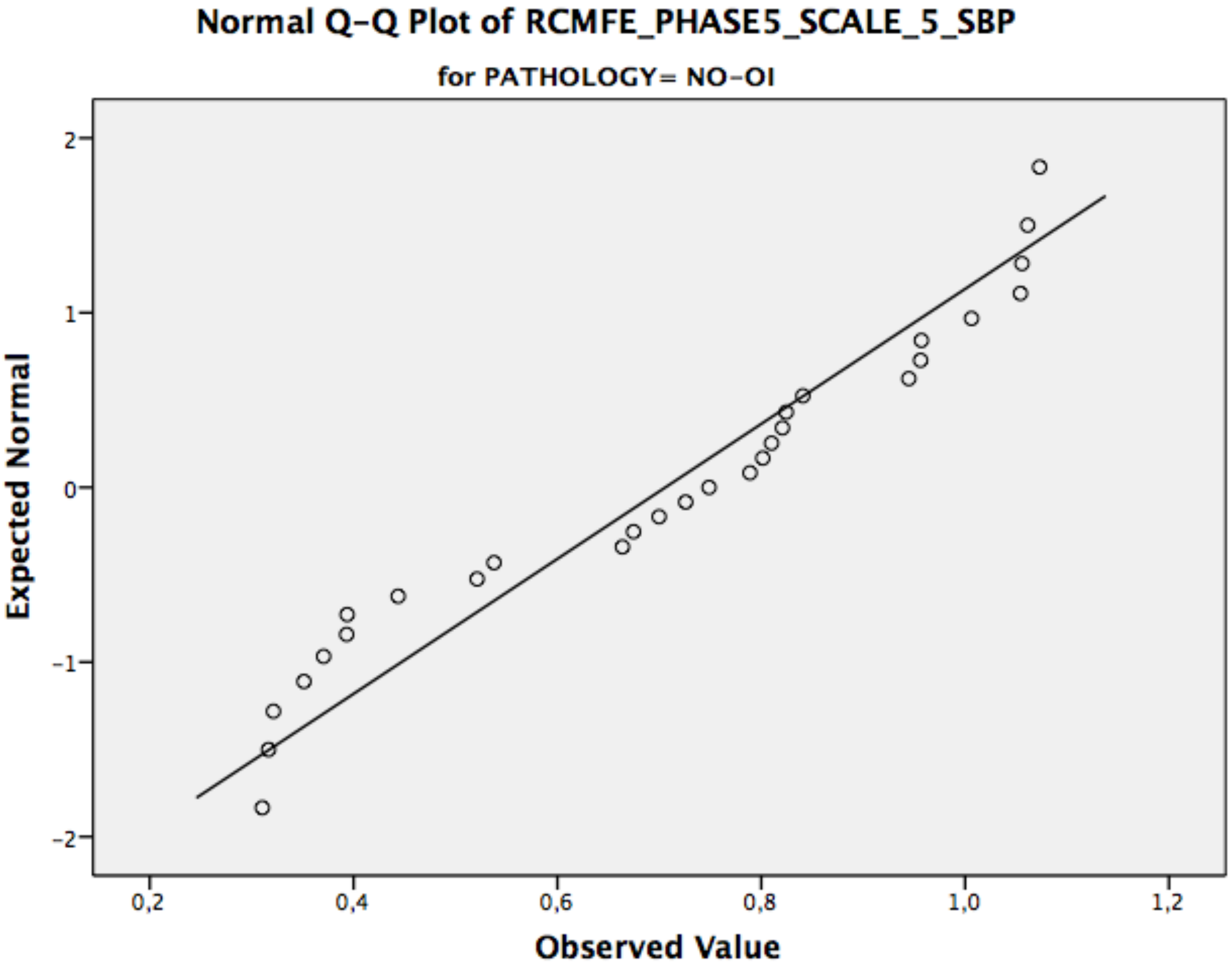

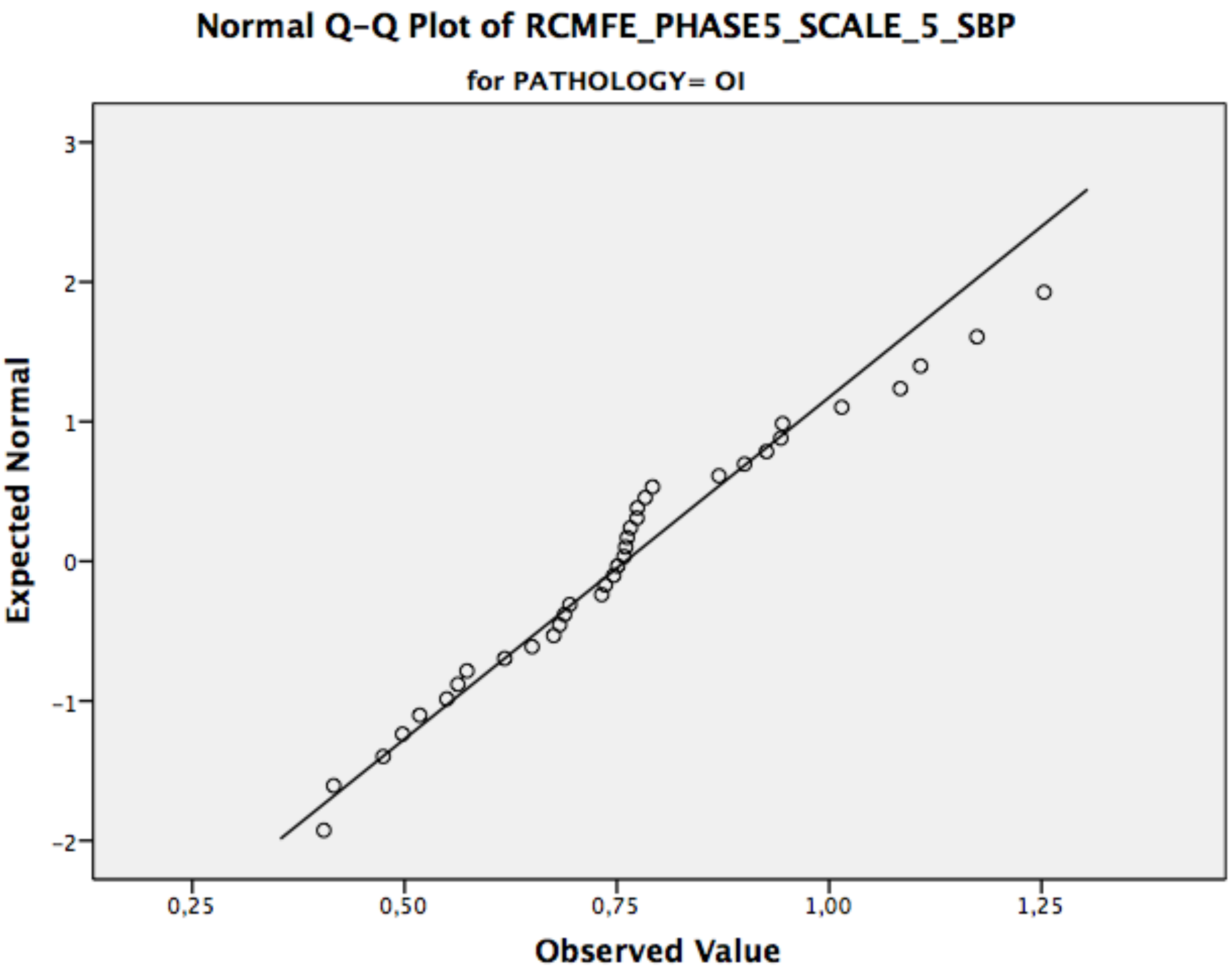

Normal Q-Q Plot of RCMFE_PHASE5_SCALE_5_SBP
for PATHOLOGY= OI
Expected Normal
Observed Value
3
2
1
0
-1
-2
0,25
0,50
0,75
1,00
1,25

Explore DBP

GENDER

Case Processing Summary

	GENDER	Cases					
		Valid		Missing		Total	
		N	Percent	N	Percent	N	Percent
RCMFE_PHASE1_SCALE_1_DBP	WOMEN	42	100,0%	0	0,0%	42	100,0%
	MEN	23	100,0%	0	0,0%	23	100,0%
RCMFE_PHASE1_SCALE_2_DBP	WOMEN	42	100,0%	0	0,0%	42	100,0%
	MEN	23	100,0%	0	0,0%	23	100,0%
RCMFE_PHASE1_SCALE_3_DBP	WOMEN	42	100,0%	0	0,0%	42	100,0%
	MEN	23	100,0%	0	0,0%	23	100,0%
RCMFE_PHASE1_SCALE_4_DBP	WOMEN	42	100,0%	0	0,0%	42	100,0%
	MEN	23	100,0%	0	0,0%	23	100,0%
RCMFE_PHASE1_SCALE_5_DBP	WOMEN	42	100,0%	0	0,0%	42	100,0%
	MEN	23	100,0%	0	0,0%	23	100,0%
RCMFE_PHASE2_SCALE_1_DBP	WOMEN	42	100,0%	0	0,0%	42	100,0%
	MEN	23	100,0%	0	0,0%	23	100,0%
RCMFE_PHASE2_SCALE_2_DBP	WOMEN	42	100,0%	0	0,0%	42	100,0%
	MEN	23	100,0%	0	0,0%	23	100,0%
RCMFE_PHASE2_SCALE_3_DBP	WOMEN	42	100,0%	0	0,0%	42	100,0%
	MEN	23	100,0%	0	0,0%	23	100,0%
RCMFE_PHASE2_SCALE_4_DBP	WOMEN	42	100,0%	0	0,0%	42	100,0%
	MEN	23	100,0%	0	0,0%	23	100,0%
RCMFE_PHASE2_SCALE_5_DBP	WOMEN	42	100,0%	0	0,0%	42	100,0%
	MEN	23	100,0%	0	0,0%	23	100,0%
RCMFE_PHASE3_SCALE_1_DBP	WOMEN	42	100,0%	0	0,0%	42	100,0%
	MEN	23	100,0%	0	0,0%	23	100,0%
RCMFE_PHASE3_SCALE_2_DBP	WOMEN	42	100,0%	0	0,0%	42	100,0%
	MEN	23	100,0%	0	0,0%	23	100,0%
RCMFE_PHASE3_SCALE_3_DBP	WOMEN	42	100,0%	0	0,0%	42	100,0%
	MEN	23	100,0%	0	0,0%	23	100,0%
RCMFE_PHASE3_SCALE_4_DBP	WOMEN	42	100,0%	0	0,0%	42	100,0%
	MEN	23	100,0%	0	0,0%	23	100,0%
RCMFE_PHASE3_SCALE_5_DBP	WOMEN	42	100,0%	0	0,0%	42	100,0%
	MEN	23	100,0%	0	0,0%	23	100,0%
RCMFE_PHASE4_SCALE_1_DBP	WOMEN	42	100,0%	0	0,0%	42	100,0%
	MEN	23	100,0%	0	0,0%	23	100,0%
RCMFE_PHASE4_SCALE_2_DBP	WOMEN	42	100,0%	0	0,0%	42	100,0%
	MEN	23	100,0%	0	0,0%	23	100,0%
RCMFE_PHASE4_SCALE_3	WOMEN	42	100,0%	0	0,0%	42	100,0%
	MEN	23	100,0%	0	0,0%	23	100,0%
RCMFE_PHASE4_SCALE_4_DBP	WOMEN	42	100,0%	0	0,0%	42	100,0%
	MEN	23	100,0%	0	0,0%	23	100,0%
RCMFE_PHASE4_SCALE_5_DBP	WOMEN	42	100,0%	0	0,0%	42	100,0%
	MEN	23	100,0%	0	0,0%	23	100,0%
RCMFE_PHASE5_SCALE_1_DBP	WOMEN	42	100,0%	0	0,0%	42	100,0%

Case Processing Summary

	GENDER	Cases					
		Valid		Missing		Total	
		N	Percent	N	Percent	N	Percent
RCMFE_PHASE5_SCALE_1_DBP	MEN	23	100,0%	0	0,0%	23	100,0%
RCMFE_PHASE5_SCALE_2_DBP	WOMEN	42	100,0%	0	0,0%	42	100,0%
	MEN	23	100,0%	0	0,0%	23	100,0%
RCMFE_PHASE5_SCALE_3_DBP	WOMEN	42	100,0%	0	0,0%	42	100,0%
	MEN	23	100,0%	0	0,0%	23	100,0%
RCMFE_PHASE5_SCALE_4_DBP	WOMEN	42	100,0%	0	0,0%	42	100,0%
	MEN	23	100,0%	0	0,0%	23	100,0%
RCMFE_PHASE5_SCALE_5_DBP	WOMEN	42	100,0%	0	0,0%	42	100,0%
	MEN	23	100,0%	0	0,0%	23	100,0%

Tests of Normality

	GENDER	Kolmogorov-Smirnov[a]			Shapiro-Wilk		
		Statistic	df	Sig.	Statistic	df	Sig.
RCMFE_PHASE1_SCALE_1_DBP	WOMEN	,092	42	,200*	,969	42	,301
	MEN	,158	23	,141	,961	23	,484
RCMFE_PHASE1_SCALE_2_DBP	WOMEN	,099	42	,200*	,978	42	,578
	MEN	,168	23	,093	,945	23	,227
RCMFE_PHASE1_SCALE_3_DBP	WOMEN	,114	42	,197	,969	42	,295
	MEN	,129	23	,200*	,952	23	,315
RCMFE_PHASE1_SCALE_4_DBP	WOMEN	,092	42	,200*	,952	42	,077
	MEN	,145	23	,200*	,956	23	,382
RCMFE_PHASE1_SCALE_5_DBP	WOMEN	,106	42	,200*	,944	42	,051
	MEN	,069	23	,200*	,984	23	,963
RCMFE_PHASE2_SCALE_1_DBP	WOMEN	,104	42	,200*	,970	42	,336
	MEN	,093	23	,200*	,967	23	,624
RCMFE_PHASE2_SCALE_2_DBP	WOMEN	,117	42	,170	,957	42	,117
	MEN	,115	23	,200*	,955	23	,368
RCMFE_PHASE2_SCALE_3_DBP	WOMEN	,092	42	,200*	,980	42	,657
	MEN	,103	23	,200*	,973	23	,767
RCMFE_PHASE2_SCALE_4_DBP	WOMEN	,079	42	,200*	,975	42	,467
	MEN	,071	23	,200*	,974	23	,789
RCMFE_PHASE2_SCALE_5_DBP	WOMEN	,068	42	,200*	,987	42	,912
	MEN	,088	23	,200*	,990	23	,996
RCMFE_PHASE3_SCALE_1_DBP	WOMEN	,124	42	,103	,957	42	,115
	MEN	,107	23	,200*	,972	23	,733
RCMFE_PHASE3_SCALE_2_DBP	WOMEN	,090	42	,200*	,949	42	,059
	MEN	,157	23	,146	,956	23	,388
RCMFE_PHASE3_SCALE_3_DBP	WOMEN	,088	42	,200*	,977	42	,566
	MEN	,075	23	,200*	,979	23	,894
RCMFE_PHASE3_SCALE_4_DBP	WOMEN	,066	42	,200*	,990	42	,964
	MEN	,080	23	,200*	,987	23	,988
RCMFE_PHASE3_SCALE_5_	WOMEN	,072	42	,200*	,967	42	,271

DBP	MEN	,149	23	,200*	,955	23	,377
RCMFE_PHASE4_SCALE_1_	WOMEN	,170	42	,034	,905	42	,052
DBP	MEN	,123	23	,200*	,963	23	,520
RCMFE_PHASE4_SCALE_2_	WOMEN	,109	42	,200*	,916	42	,005
DBP	MEN	,134	23	,200*	,970	23	,685
RCMFE_PHASE4_SCALE_3	WOMEN	,125	42	,096	,935	42	,020
	MEN	,159	23	,135	,956	23	,393
RCMFE_PHASE4_SCALE_4_	WOMEN	,111	42	,200*	,976	42	,519
DBP	MEN	,108	23	,200*	,969	23	,666
RCMFE_PHASE4_SCALE_5_	WOMEN	,077	42	,200*	,975	42	,479
DBP	MEN	,108	23	,200*	,973	23	,769
RCMFE_PHASE5_SCALE_1_	WOMEN	,078	42	,200*	,978	42	,571
DBP	MEN	,105	23	,200*	,981	23	,929

Tests of Normality

	GENDER	Kolmogorov-Smirnov[a]			Shapiro-Wilk		
		Statistic	df	Sig.	Statistic	df	Sig.
RCMFE_PHASE5_SCALE_2_	WOMEN	,119	42	,142*	,924	42	,008
DBP	MEN	,125	23	,200	,922	23	,074
RCMFE_PHASE5_SCALE_3_	WOMEN	,149	42	,020*	,936	42	,021
DBP	MEN	,164	23	,111	,932	23	,121
RCMFE_PHASE5_SCALE_4_	WOMEN	,098	42	,200	,974	42	,440
DBP	MEN	,080	23	,200*	,983	23	,950
RCMFE_PHASE5_SCALE_5_	WOMEN	,092	42	,200*	,977	42	,542
DBP	MEN	,104	23	,200*	,986	23	,977

*. This is a lower bound of the true significance.

a. Lilliefors Significance Correction

RCMFE_PHASE1_SCALE_1_DBP

Normal Q-Q Plots

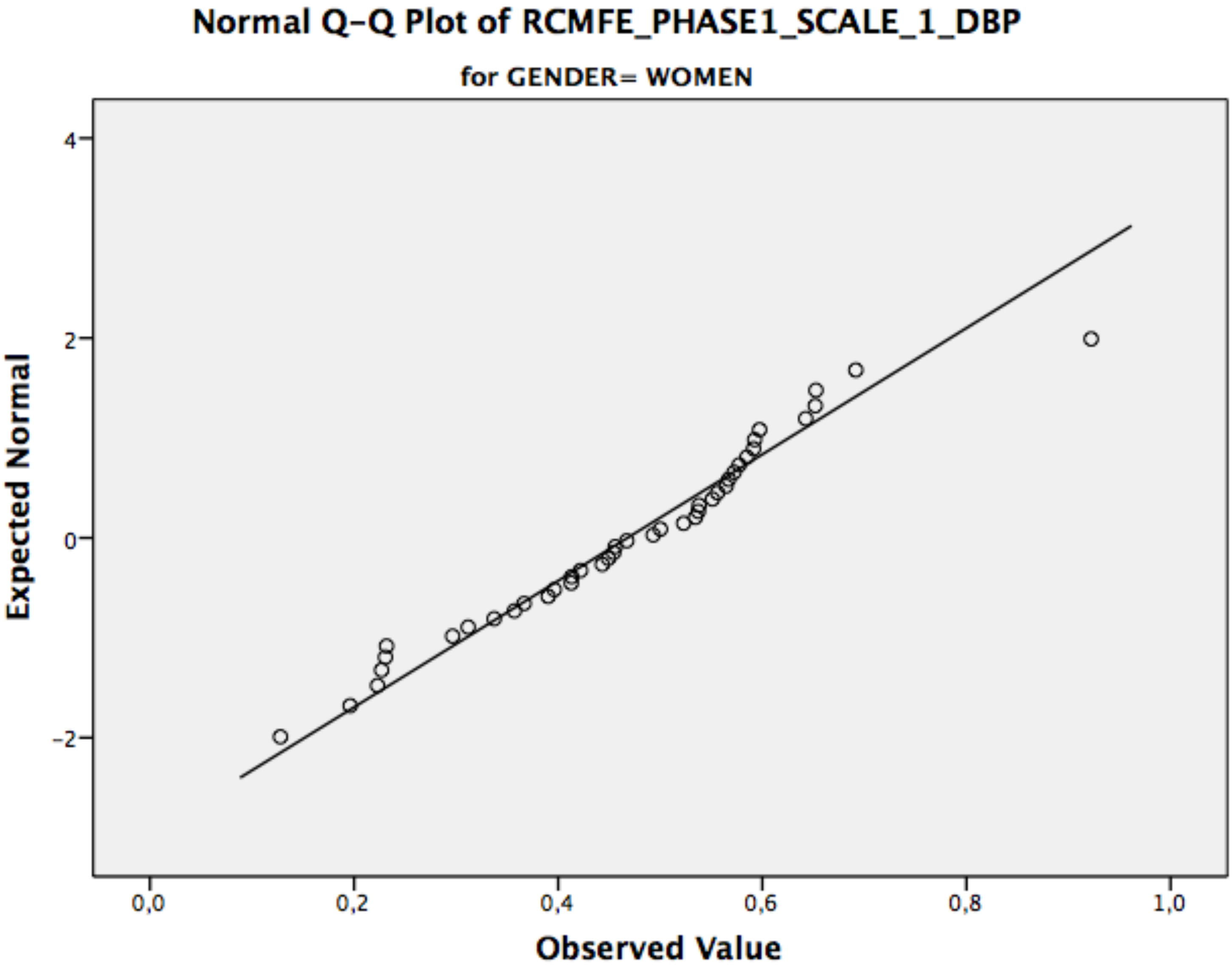

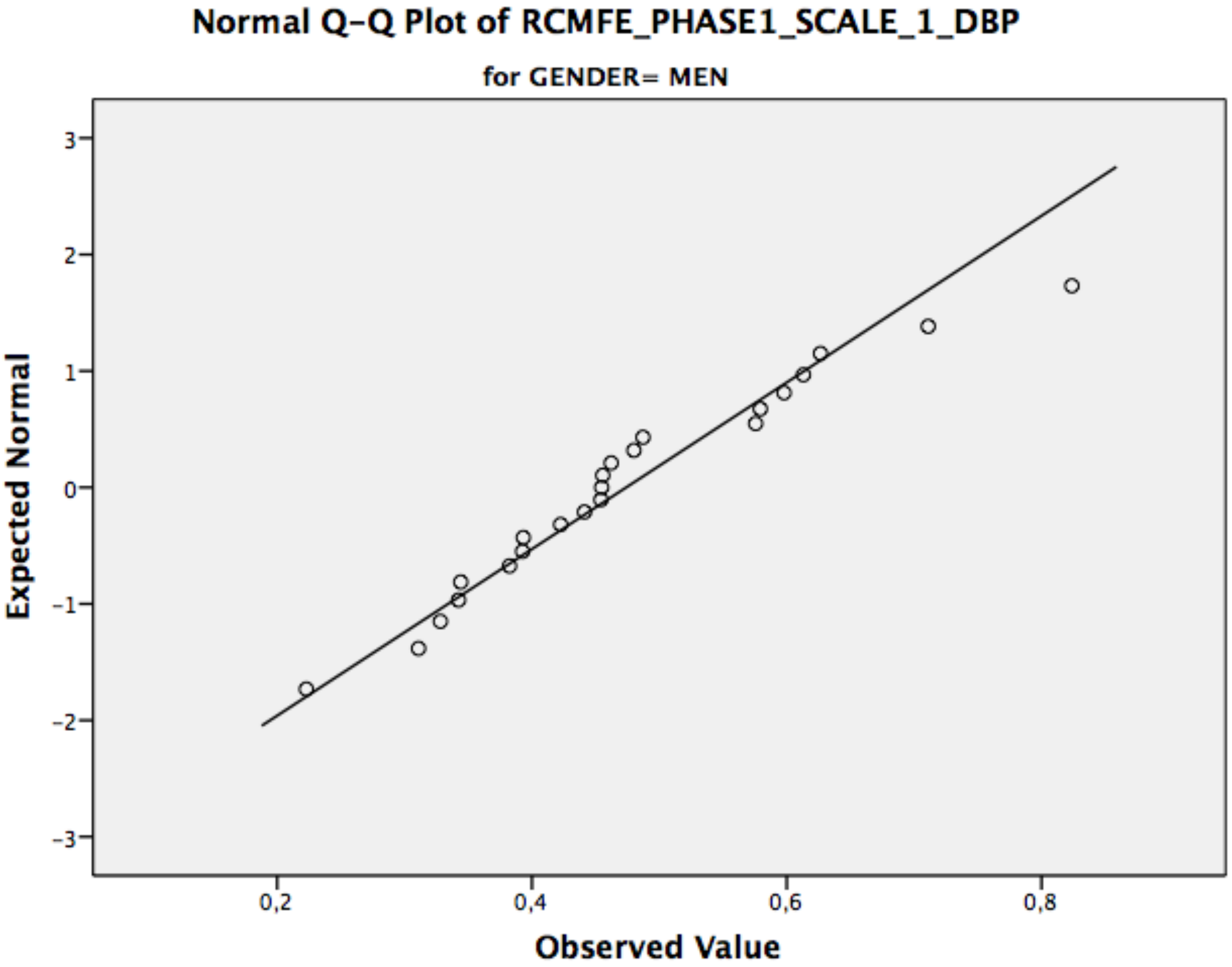
Normal Q-Q Plot of RCMFE_PHASE1_SCALE_1_DBP
for GENDER= MEN
Expected Normal
3
2
1
0
-1
-2
-3
0,2
0,4
0,6
0,8
Observed Value

RCMFE_PHASE1_SCALE_2_DBP

Normal Q-Q Plots

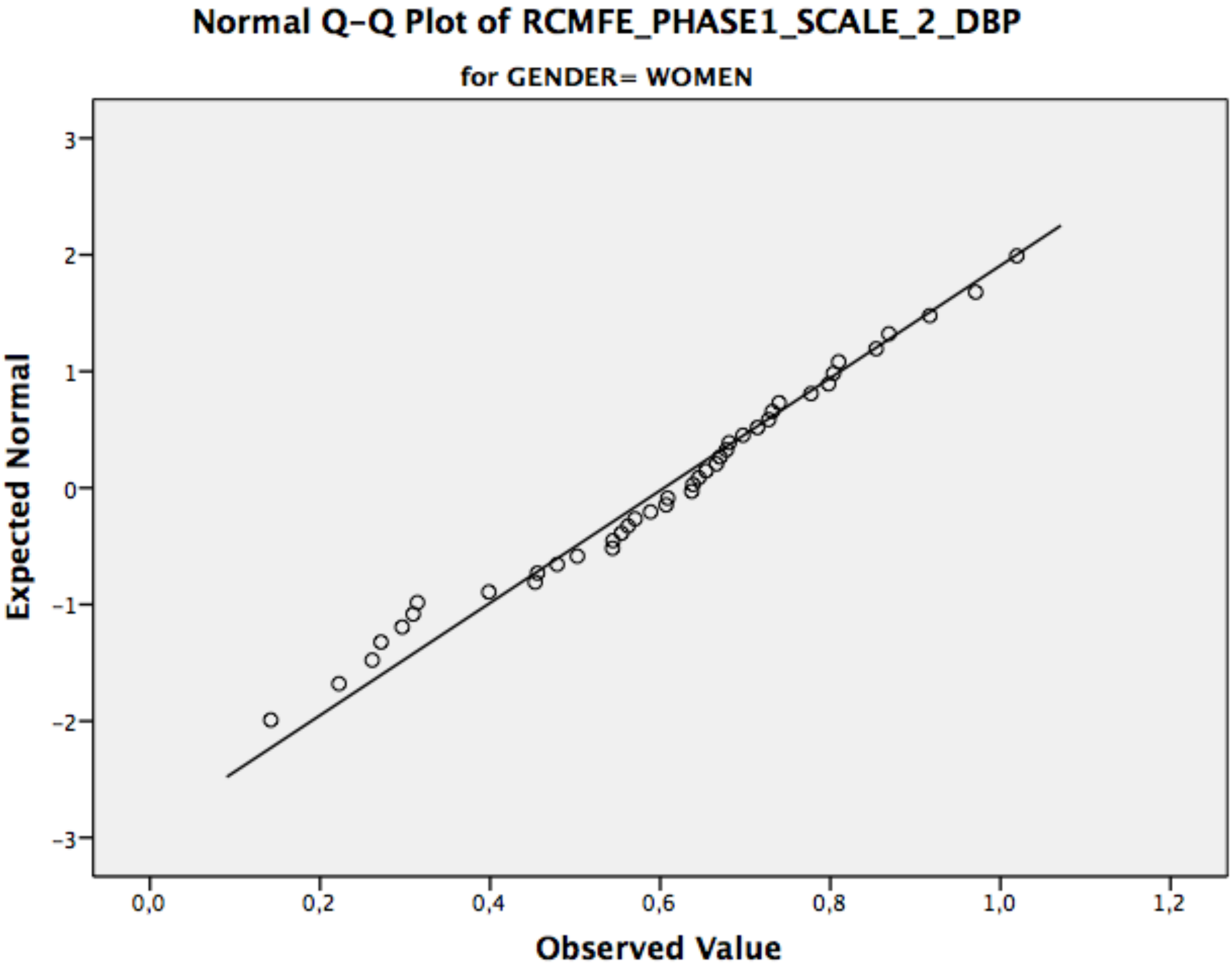

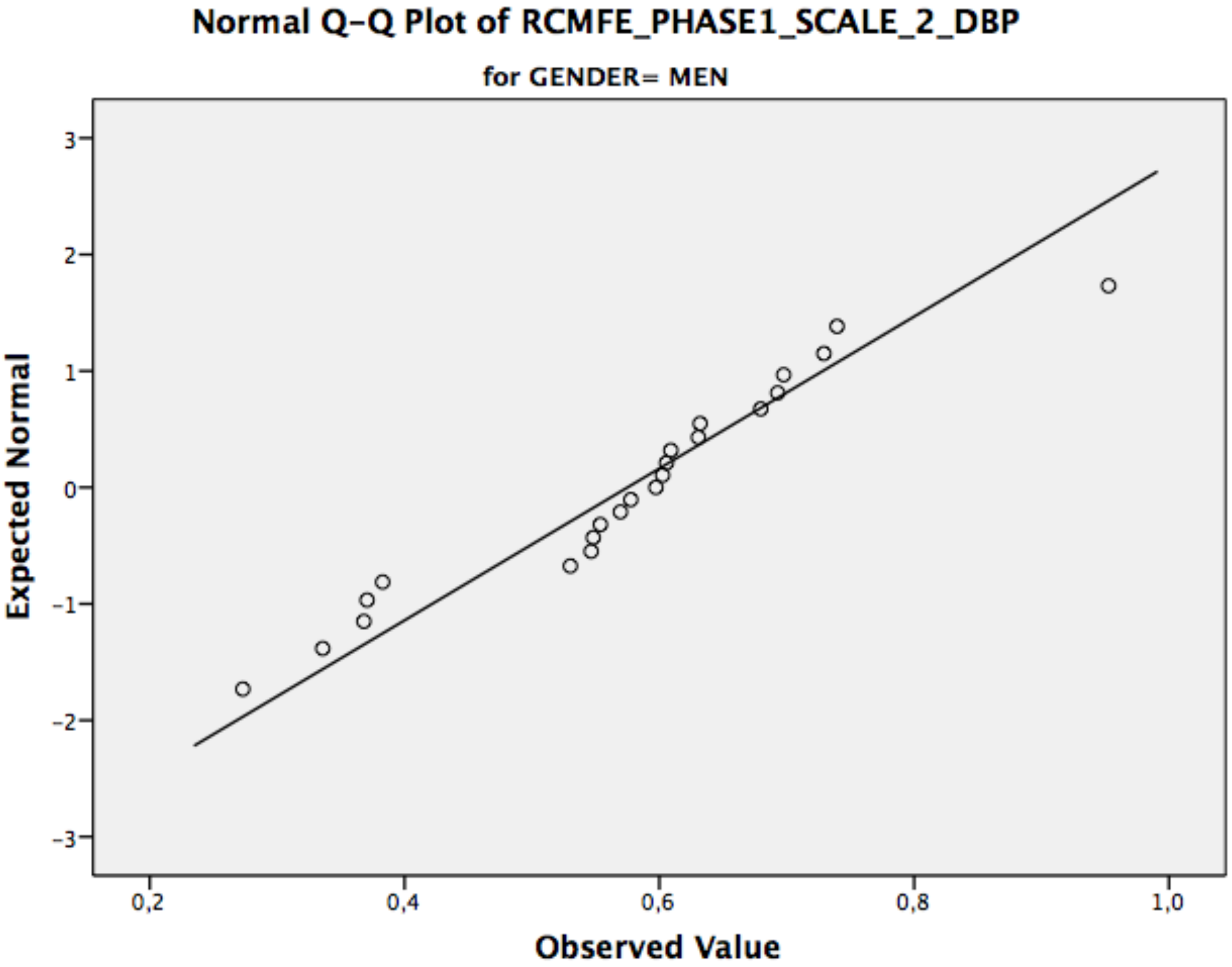
Normal Q-Q Plot of RCMFE_PHASE1_SCALE_2_DBP
for GENDER= MEN
Expected Normal
3
2
1
0
-1
-2
-3
0,2
0,4
0,6
0,8
1,0
Observed Value

Normal Q-Q Plots

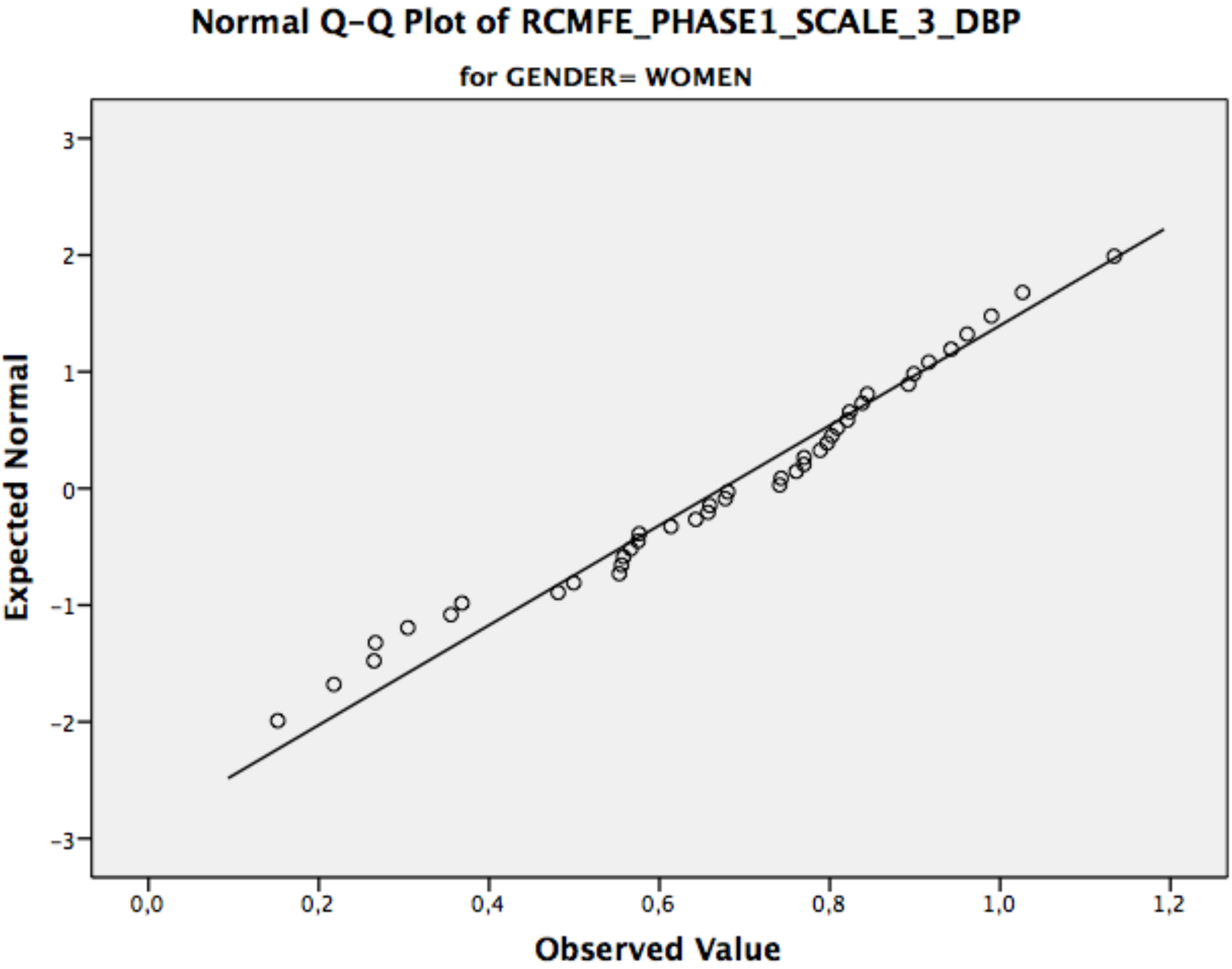

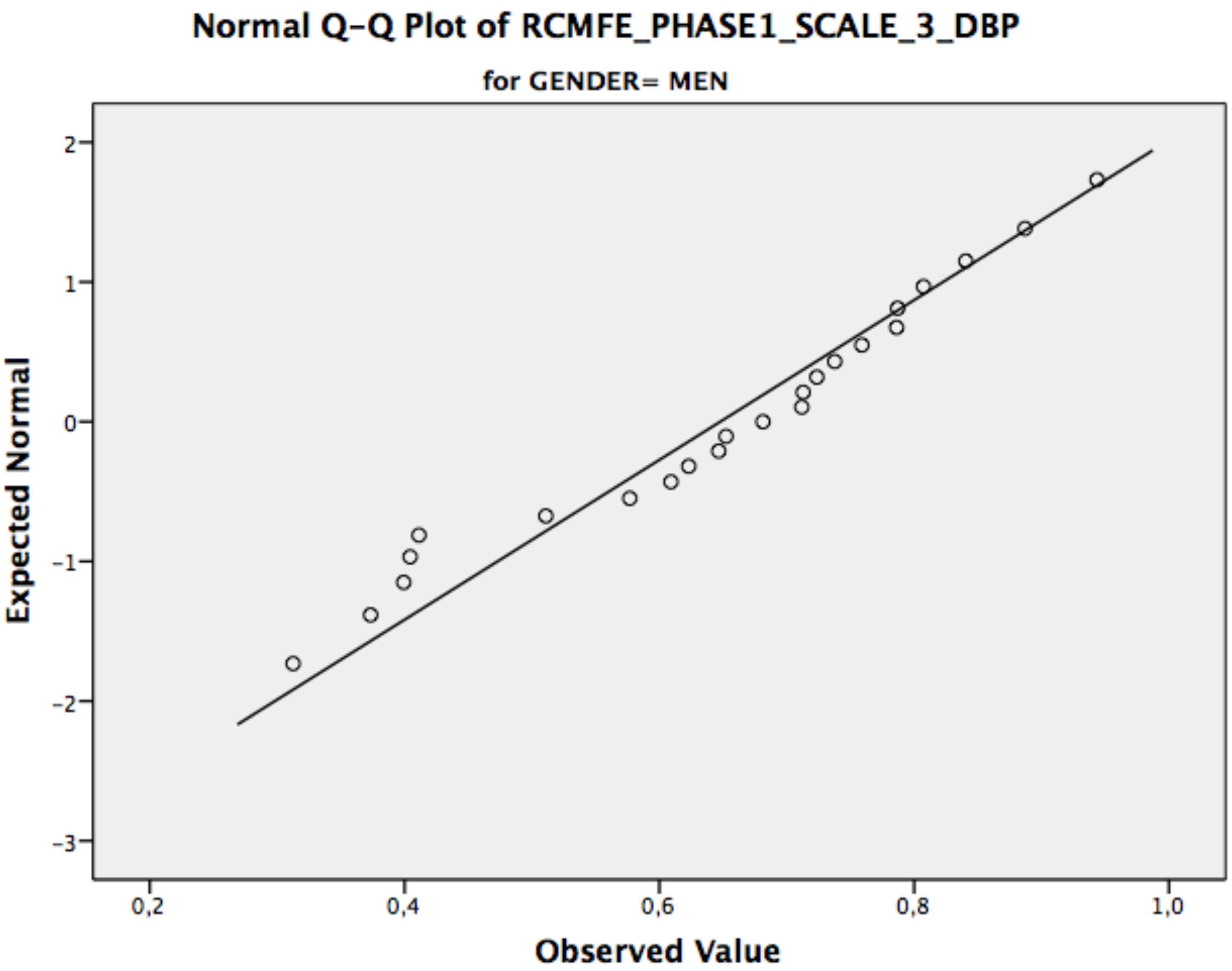
Normal Q-Q Plot of RCMFE_PHASE1_SCALE_3_DBP
for GENDER= MEN
Expected Normal
2
1
0
-1
-2
-3
0,2
0,4
0,6
0,8
1,0
Observed Value

RCMFE_PHASE1_SCALE_4_DBP

Normal Q-Q Plots

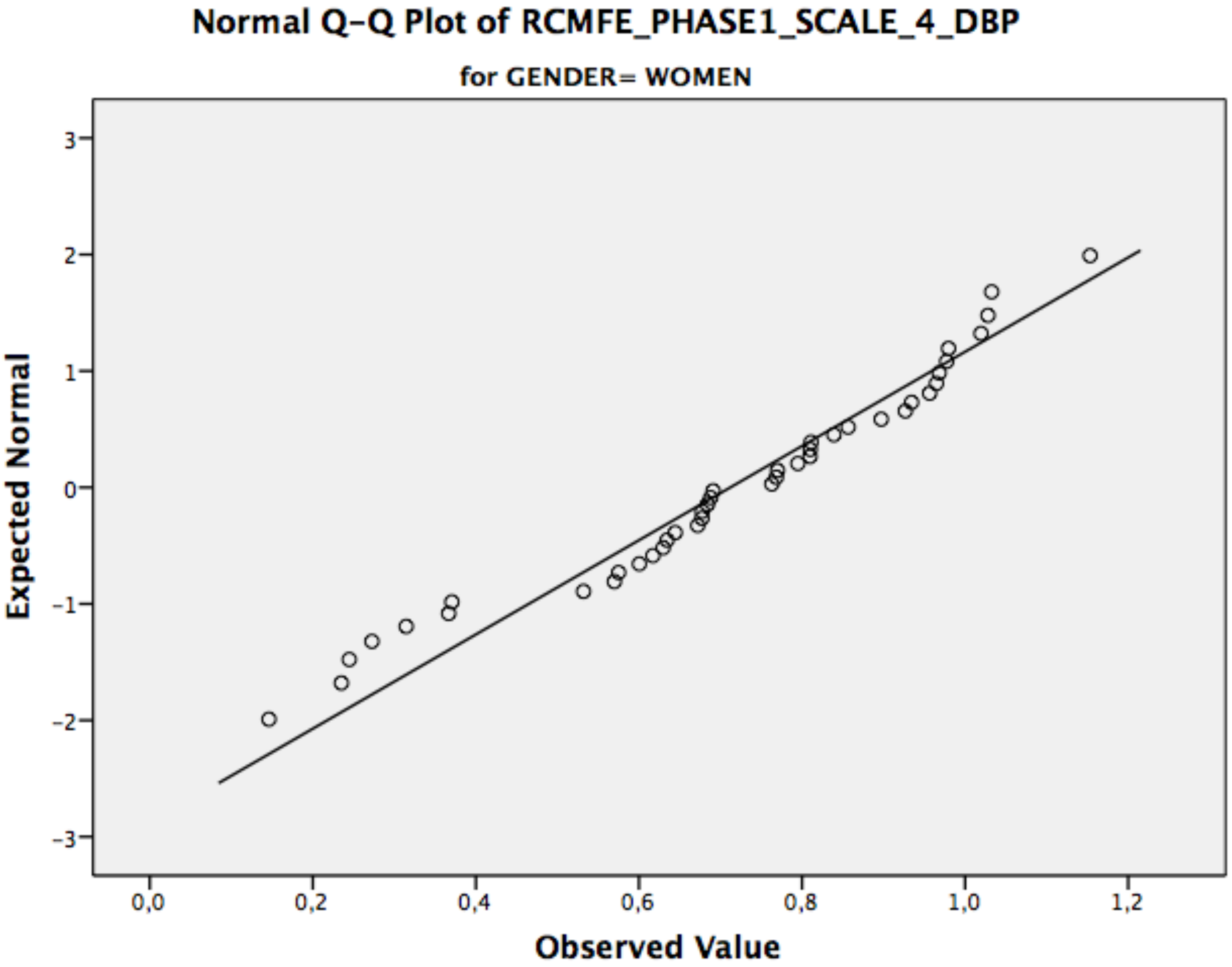

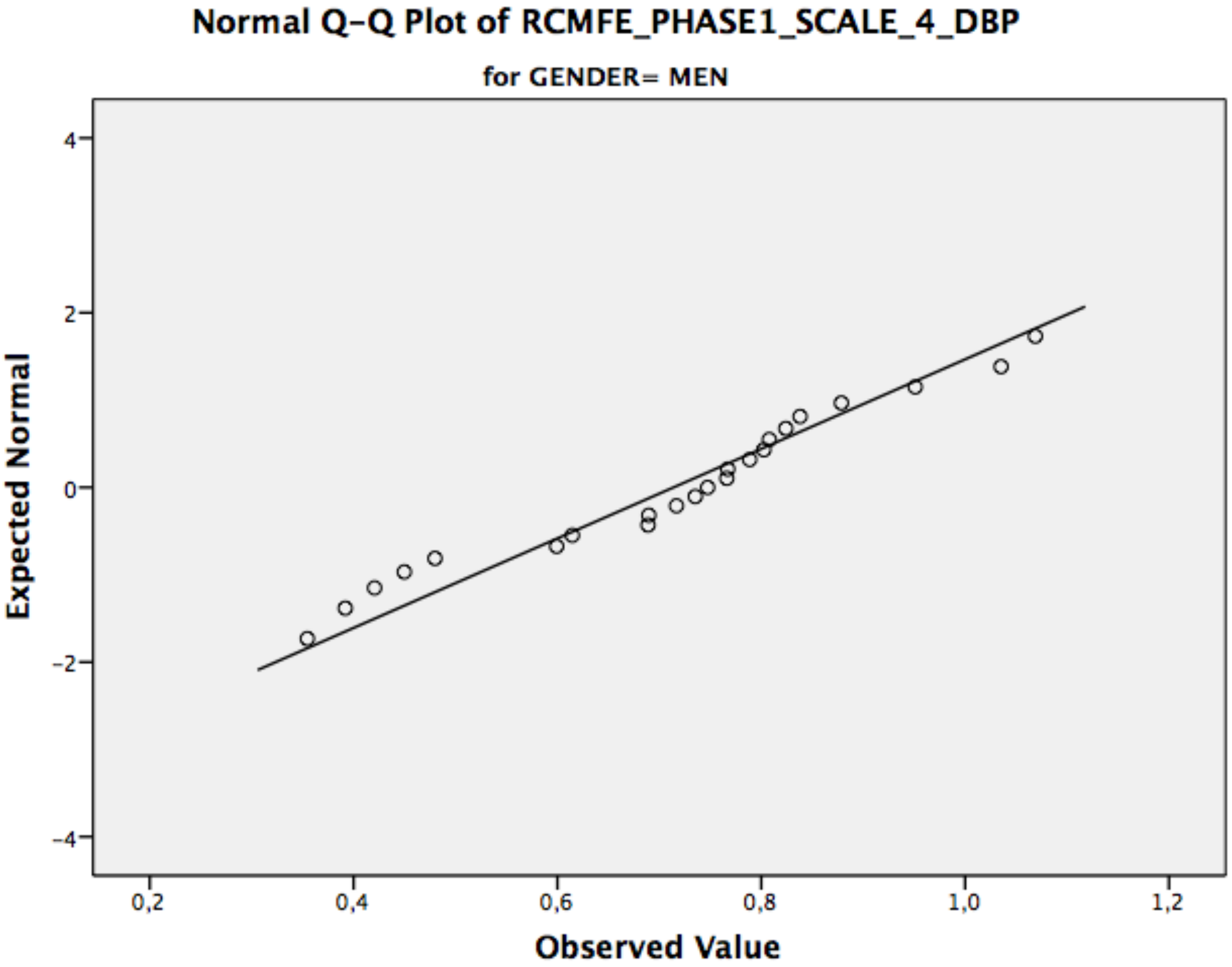

Normal Q-Q Plot of RCMFE_PHASE1_SCALE_4_DBP
for GENDER= MEN
Expected Normal
4
2
0
-2
-4
0,2
0,4
0,6
0,8
1,0
1,2
Observed Value

RCMFE_PHASE1_SCALE_5_DBP

Normal Q-Q Plots

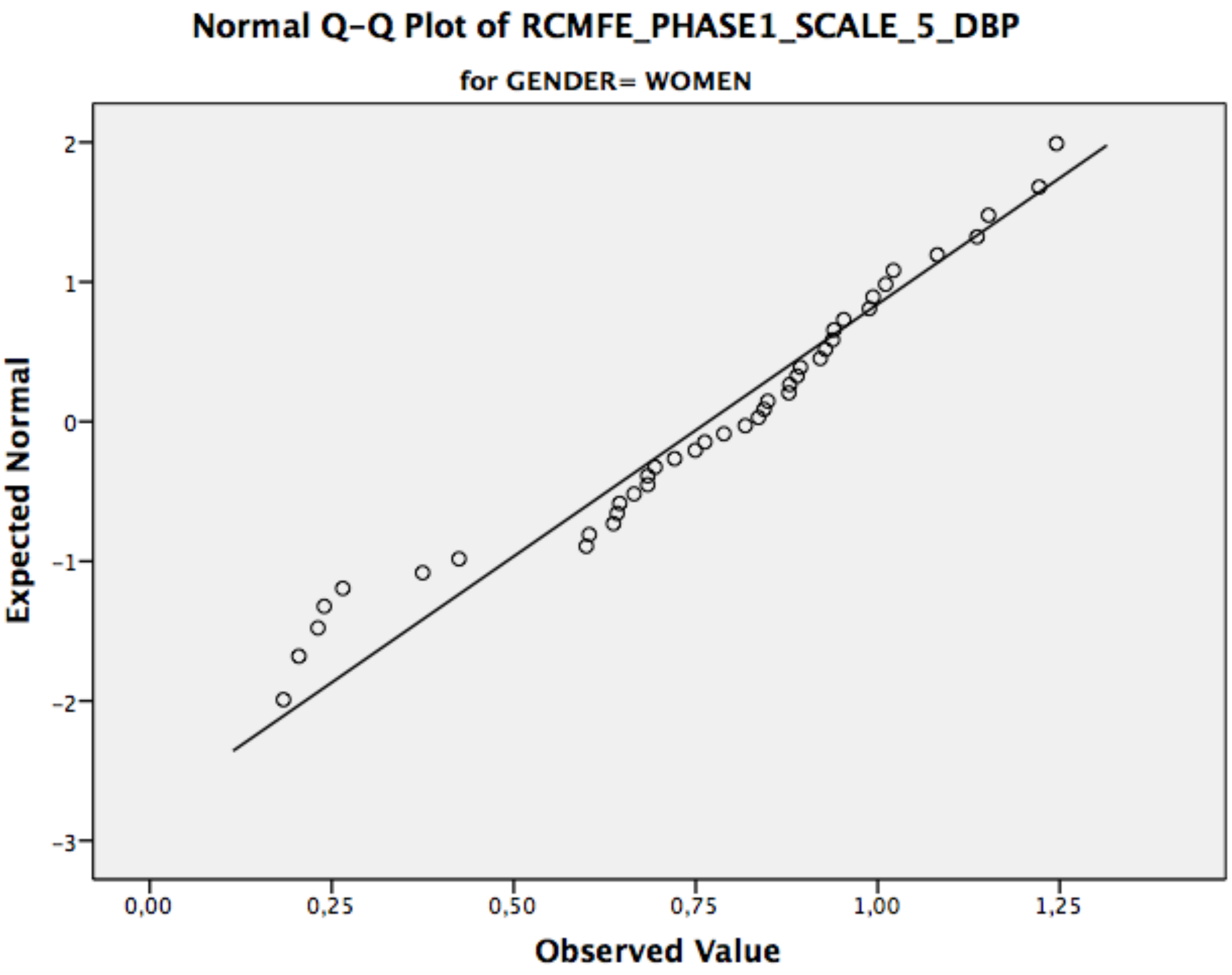

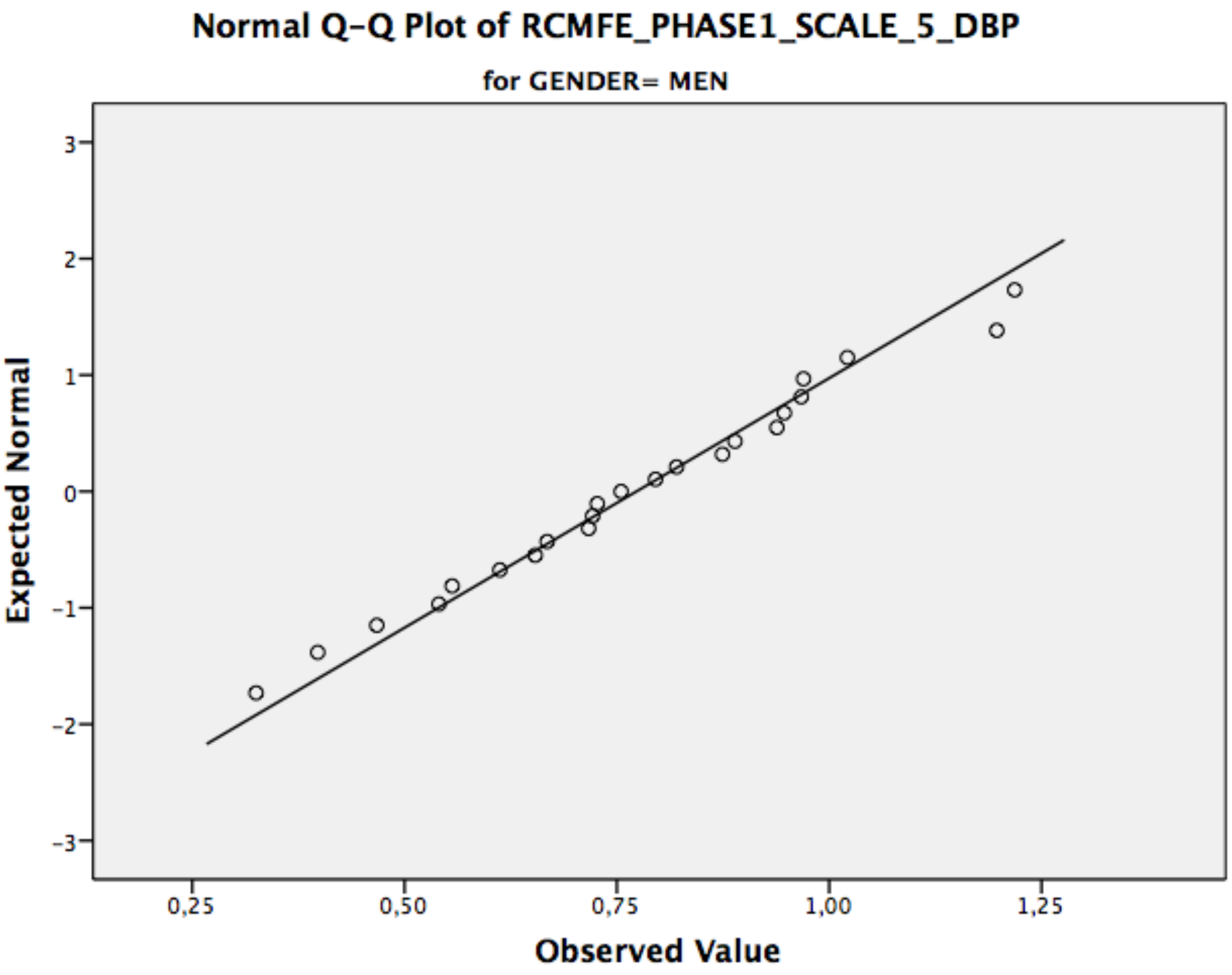

Normal Q-Q Plot of RCMFE_PHASE1_SCALE_5_DBP
for GENDER= MEN
Expected Normal
3
2
1
0
−1
−2
−3
0,25
0,50
0,75
1,00
1,25
Observed Value

RCMFE_PHASE2_SCALE_1_DBP

Normal Q-Q Plots

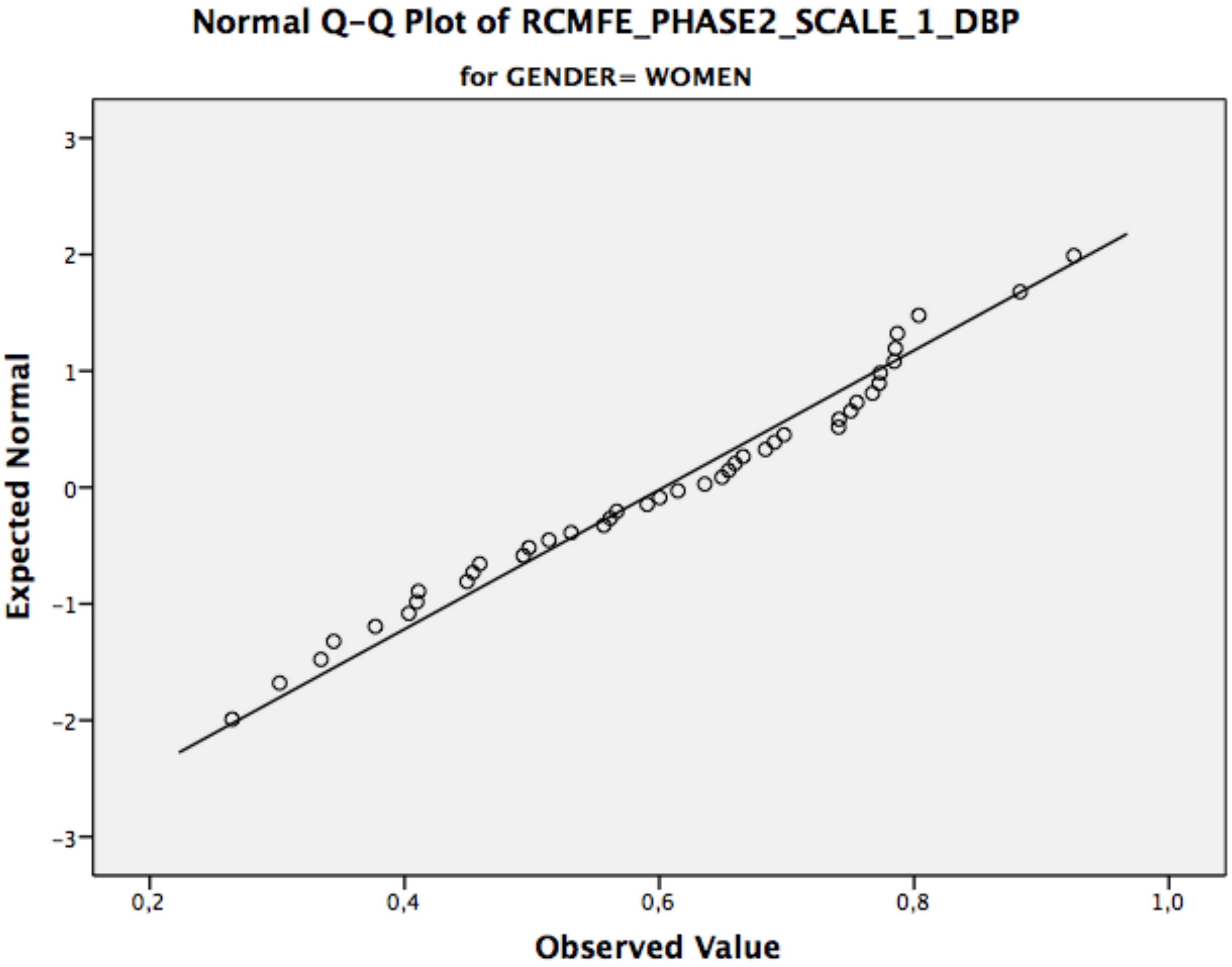

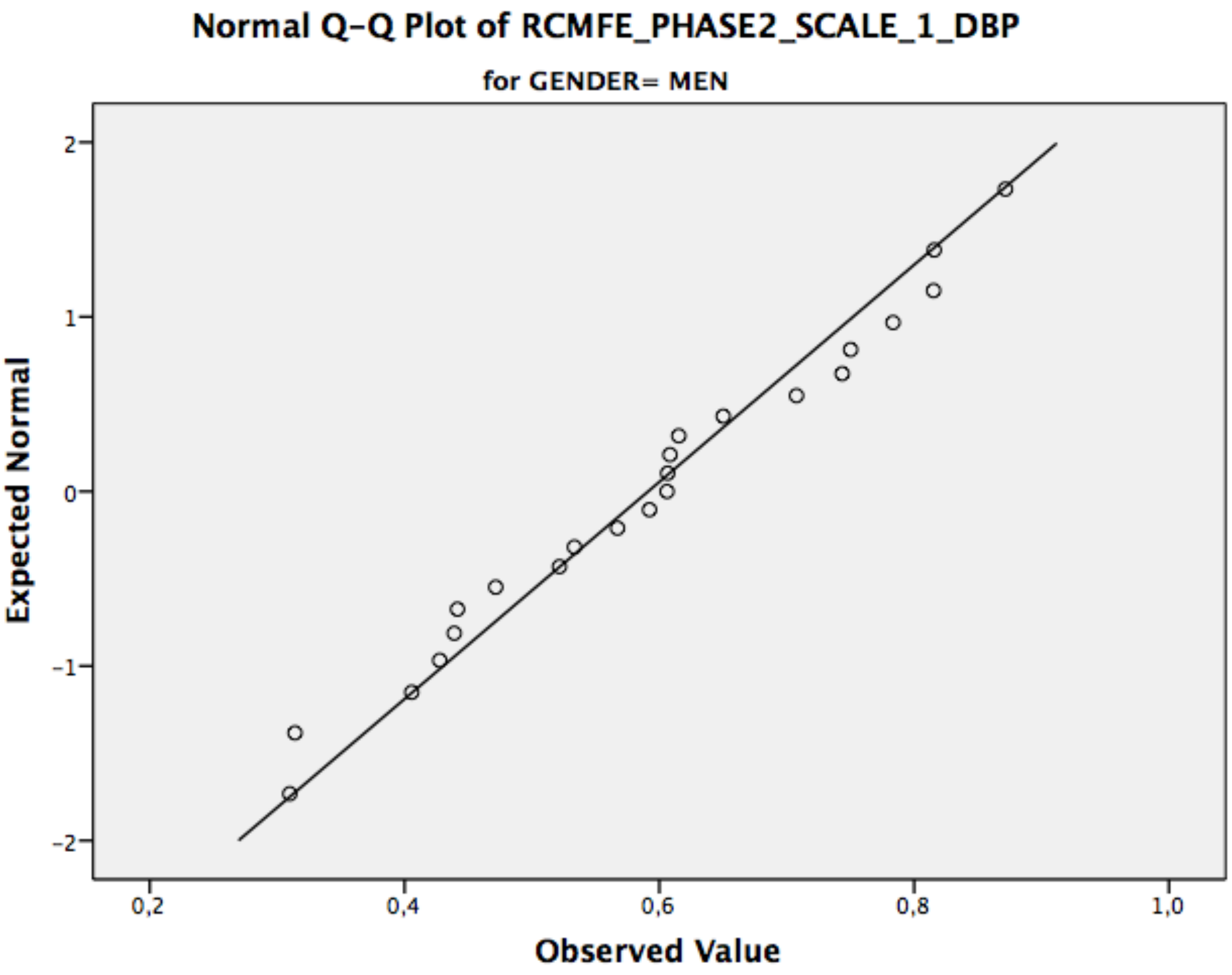
Normal Q-Q Plot of RCMFE_PHASE2_SCALE_1_DBP
for GENDER= MEN
Expected Normal
2
1
0
-1
-2
0,2
0,4
0,6
0,8
1,0
Observed Value

RCMFE_PHASE2_SCALE_2_DBP

Normal Q-Q Plots

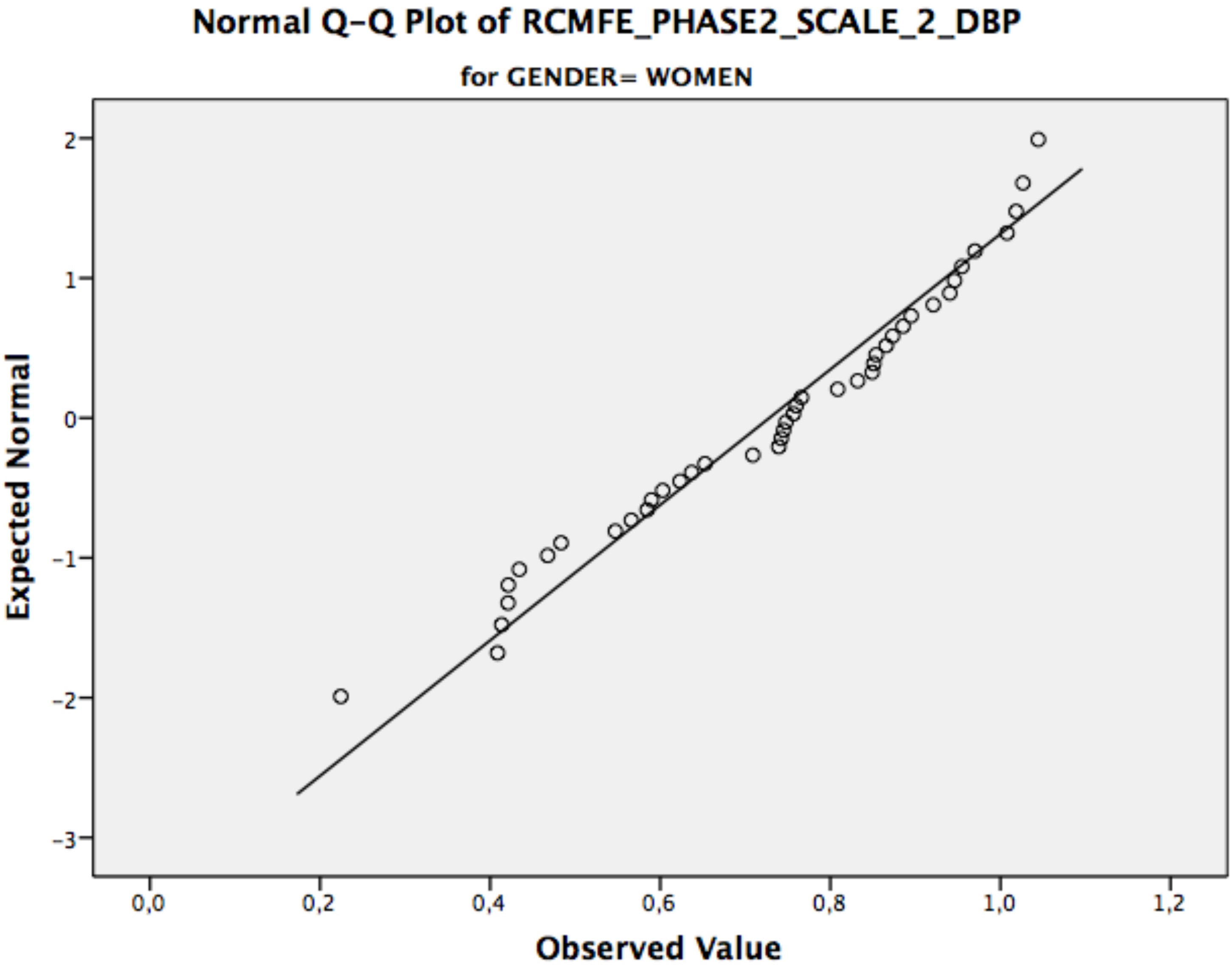

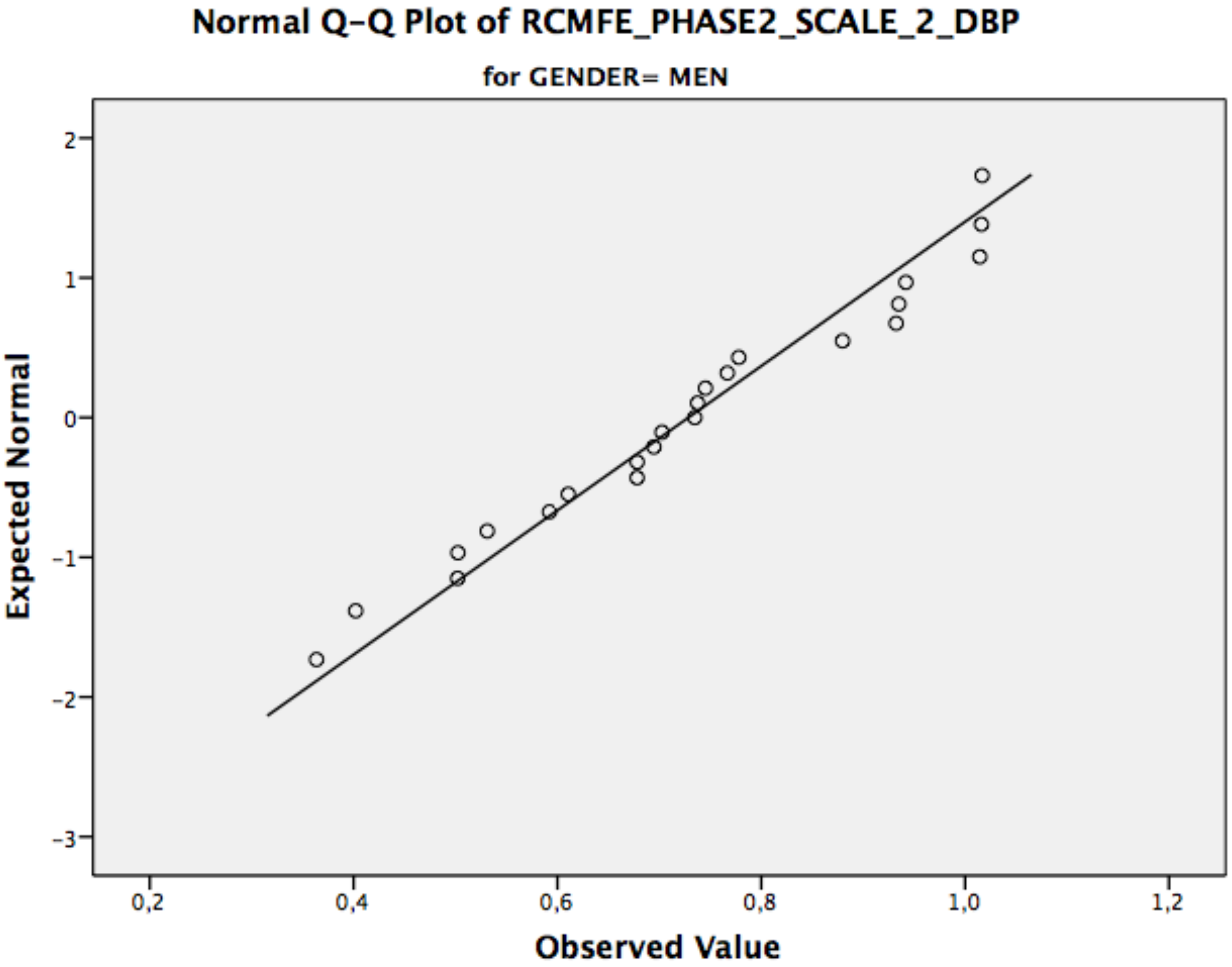

Normal Q-Q Plot of RCMFE_PHASE2_SCALE_2_DBP
for GENDER= MEN
Expected Normal
2
1
0
-1
-2
-3
0,2
0,4
0,6
0,8
1,0
1,2
Observed Value

RCMFE_PHASE2_SCALE_3_DBP

Normal Q-Q Plots

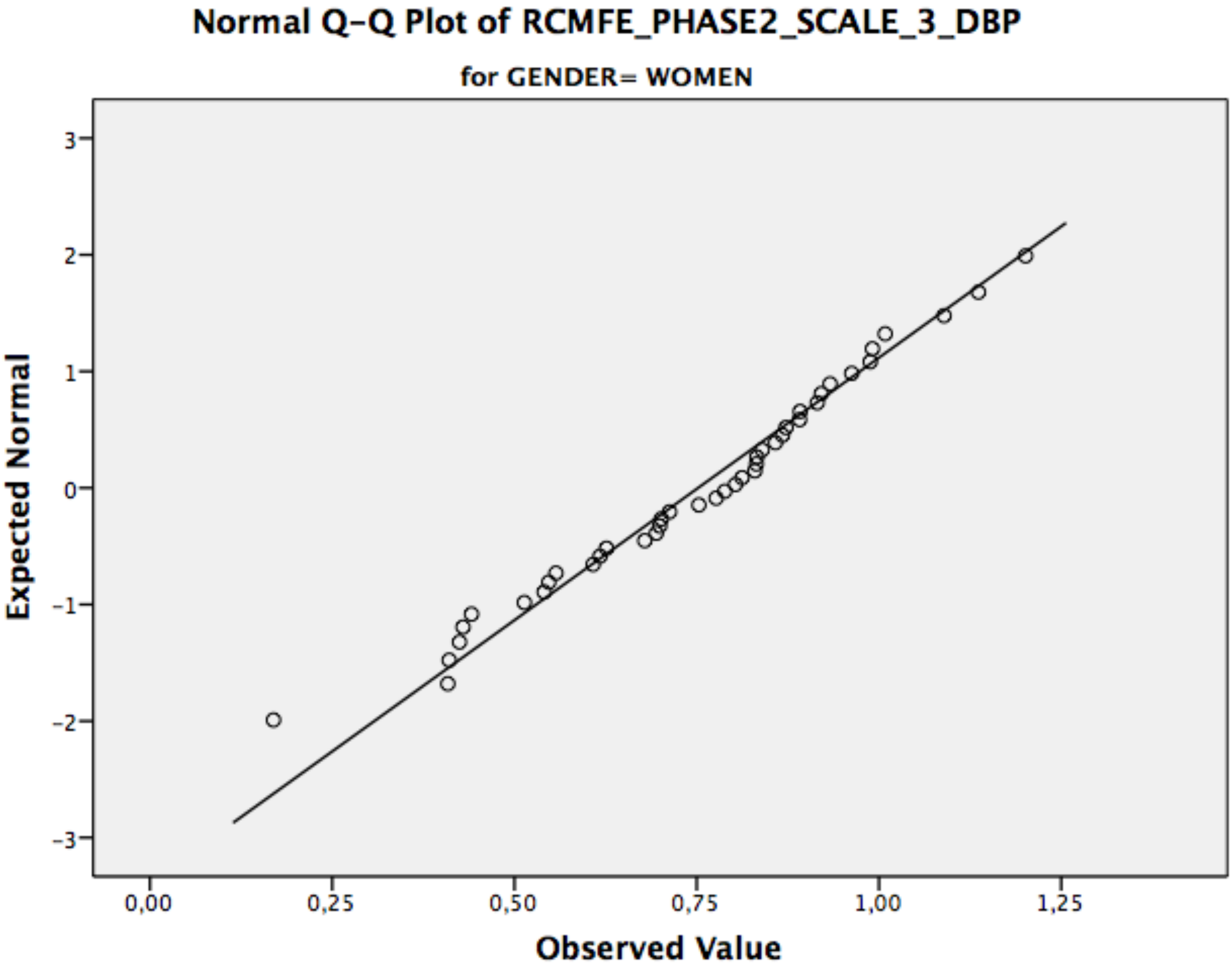

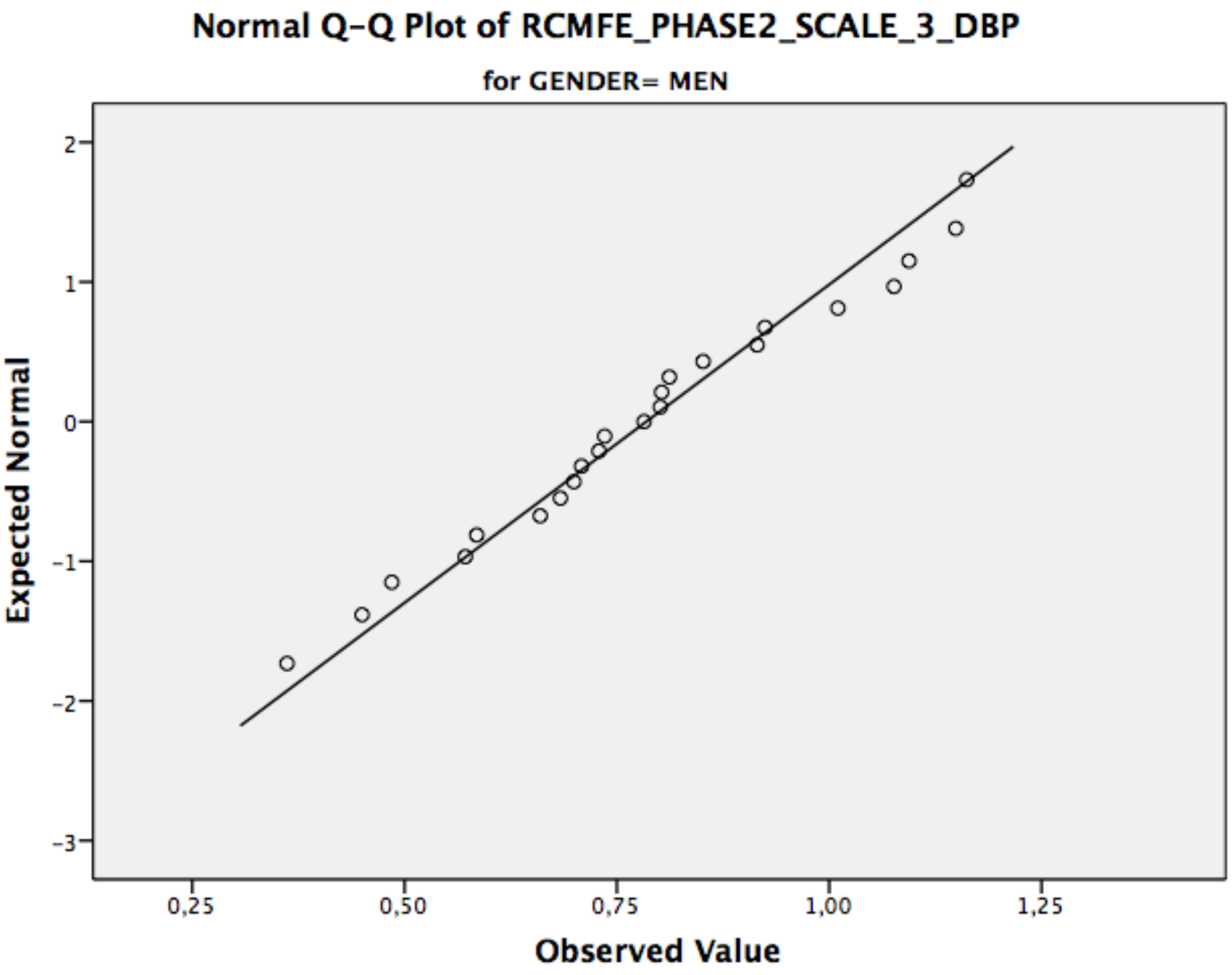
Normal Q-Q Plot of RCMFE_PHASE2_SCALE_3_DBP
for GENDER= MEN
Expected Normal
Observed Value
2
1
0
-1
-2
-3
0,25
0,50
0,75
1,00
1,25

RCMFE_PHASE2_SCALE_4_DBP

Normal Q-Q Plots

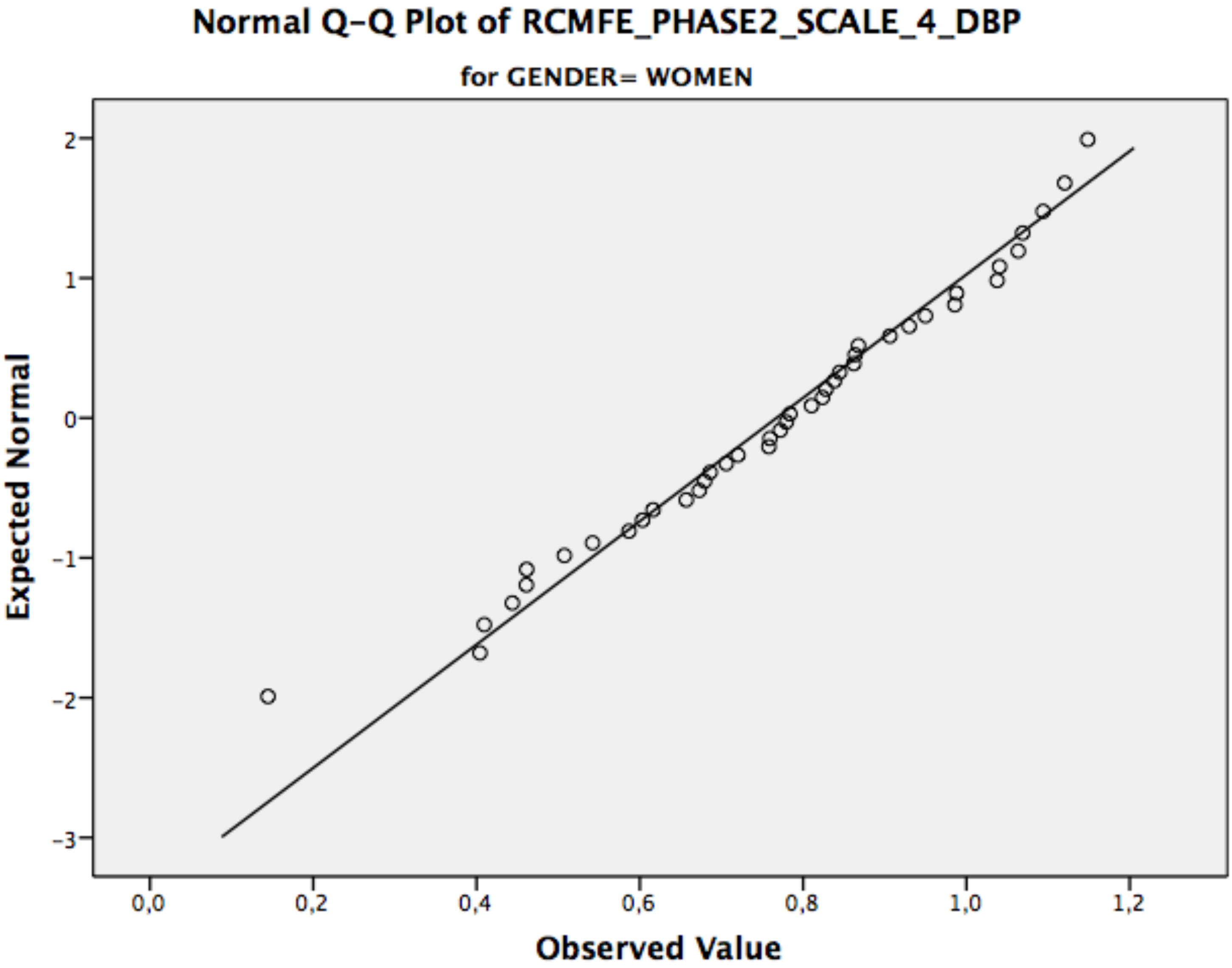

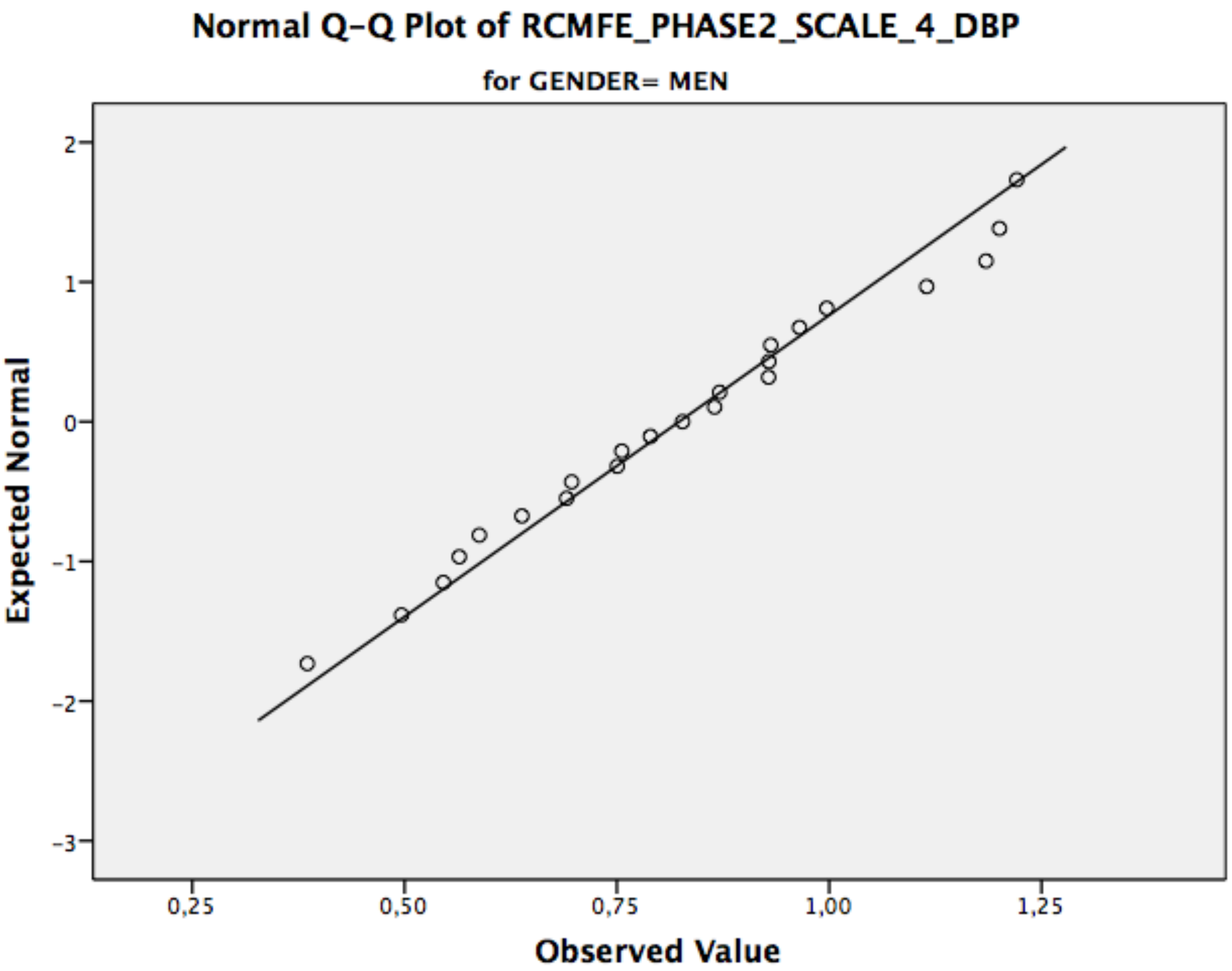

Normal Q-Q Plot of RCMFE_PHASE2_SCALE_4_DBP
for GENDER= MEN
Expected Normal
Observed Value
2
1
0
-1
-2
-3
0,25
0,50
0,75
1,00
1,25

RCMFE_PHASE2_SCALE_5_DBP

Normal Q-Q Plots

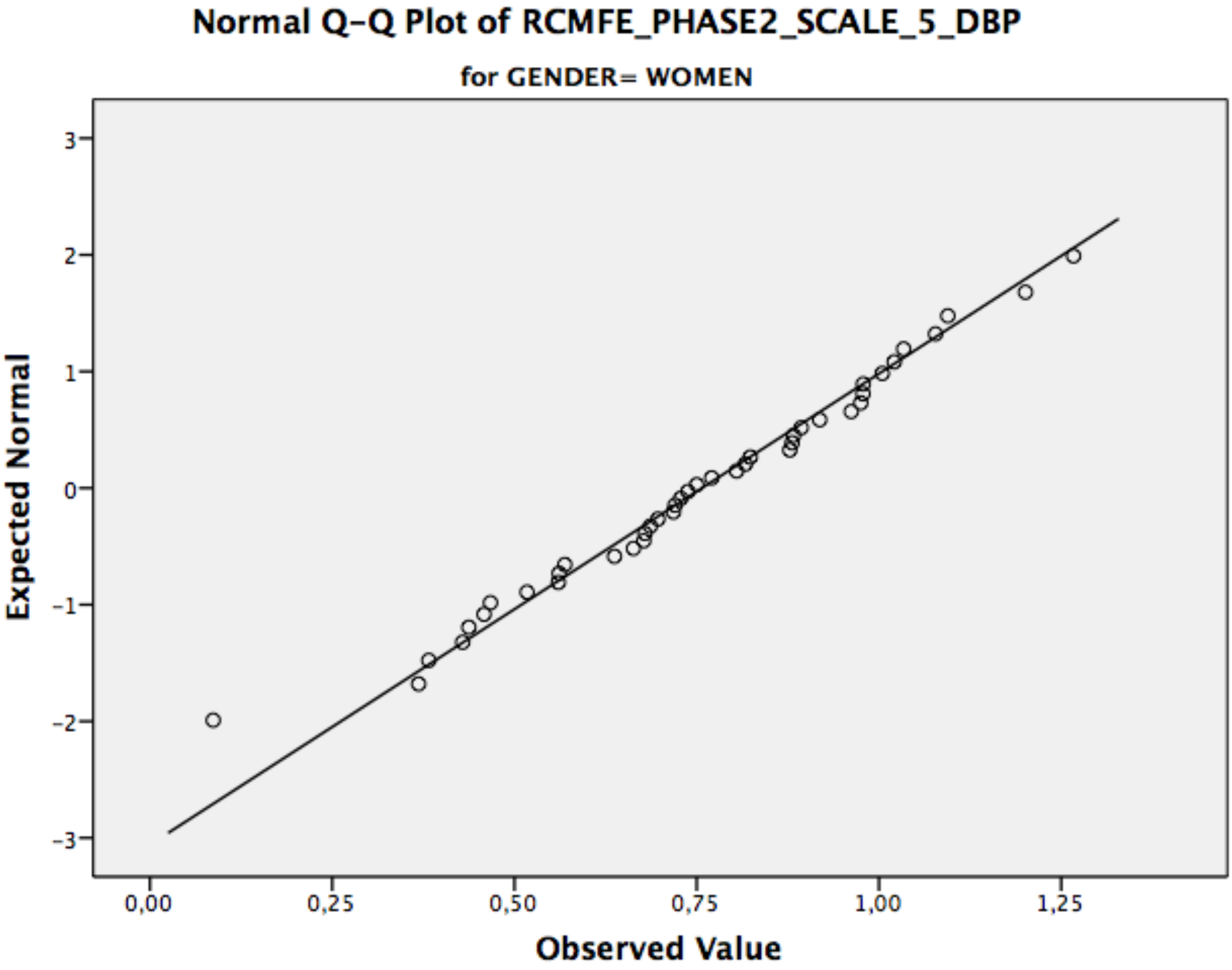

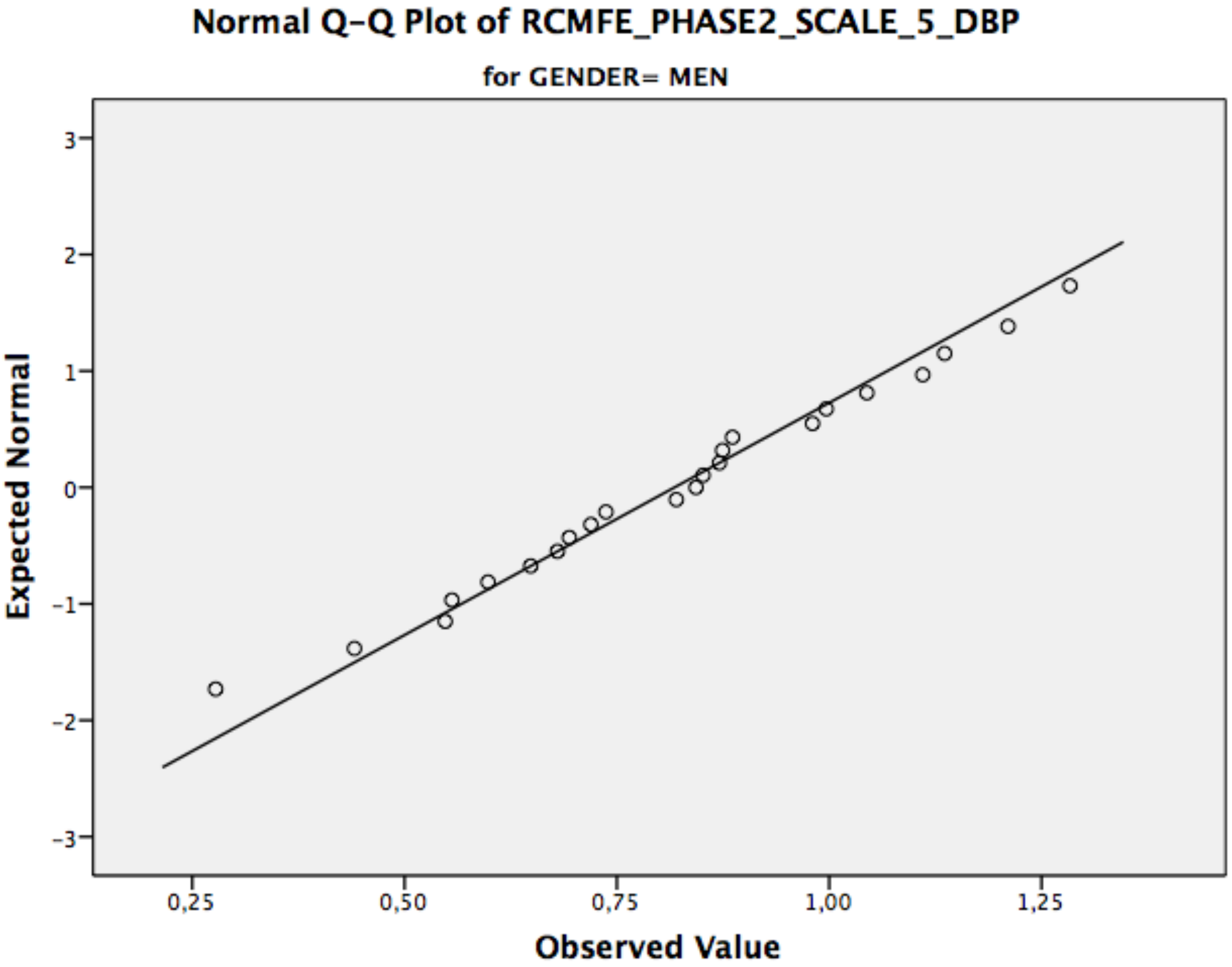
Normal Q-Q Plot of RCMFE_PHASE2_SCALE_5_DBP
for GENDER= MEN
Expected Normal
3
2
1
0
-1
-2
-3
0,25
0,50
0,75
1,00
1,25
Observed Value

RCMFE_PHASE3_SCALE_1_DBP

Normal Q-Q Plots

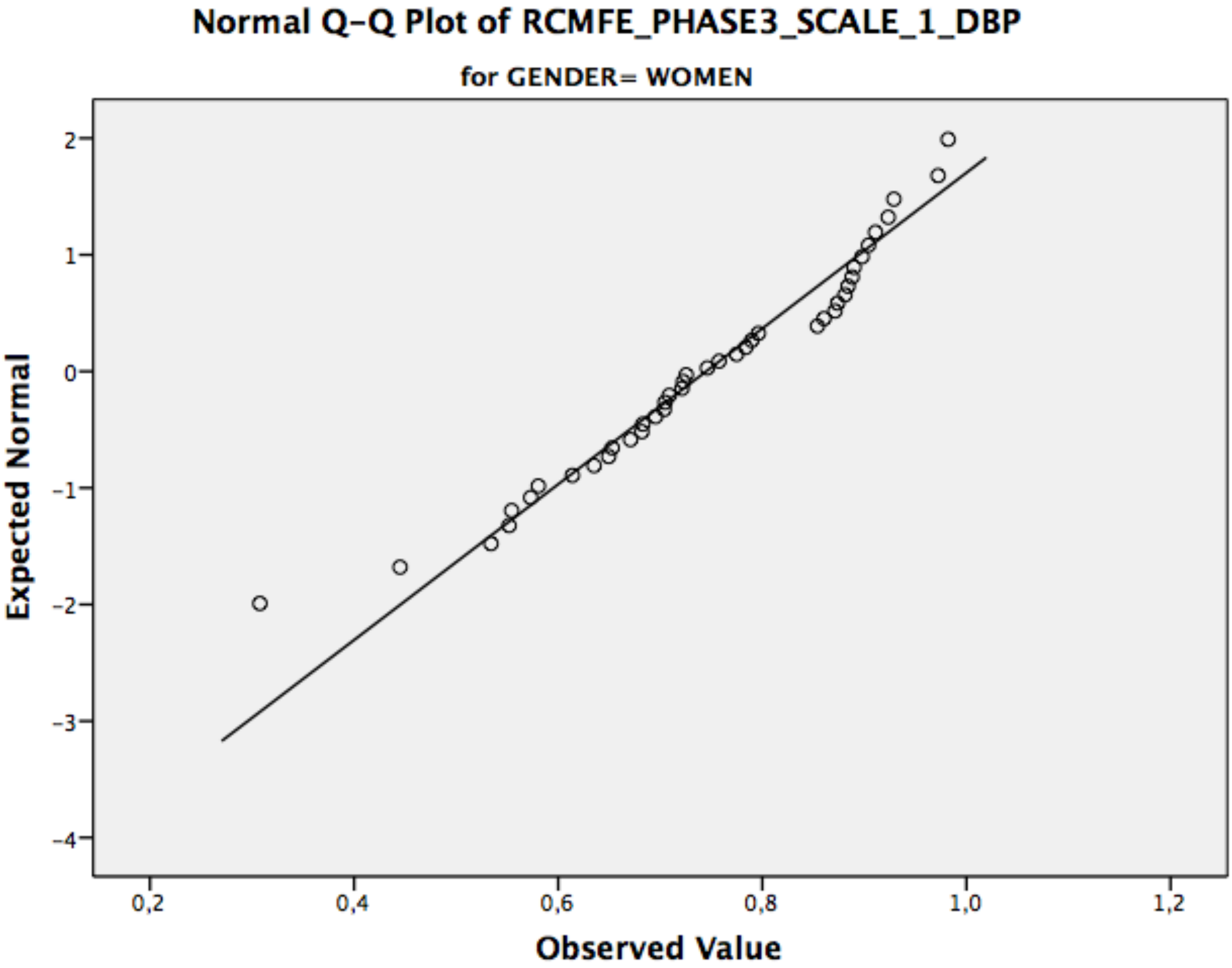

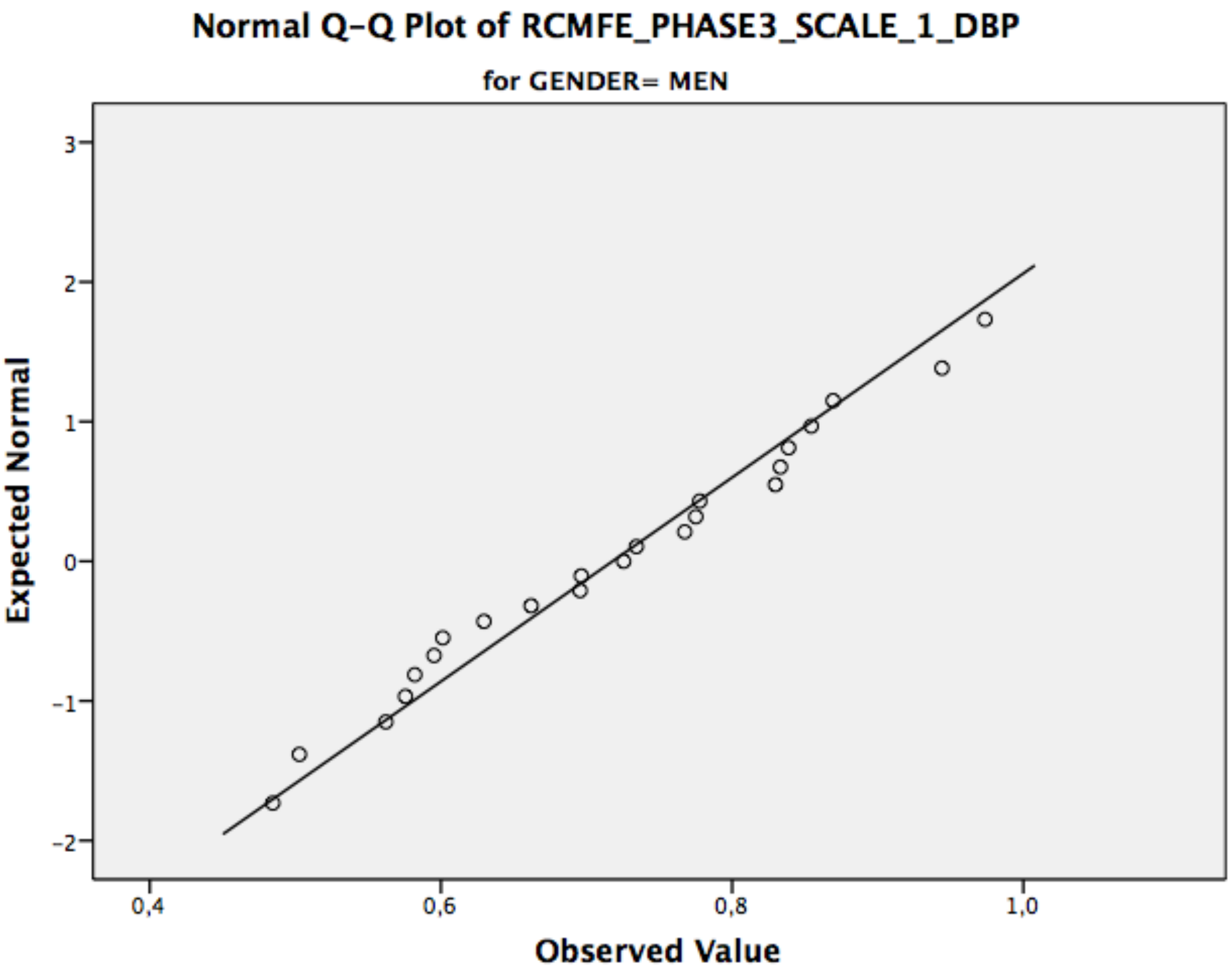
Normal Q-Q Plot of RCMFE_PHASE3_SCALE_1_DBP
for GENDER= MEN
Expected Normal
Observed Value
3
2
1
0
-1
-2
0,4
0,6
0,8
1,0

RCMFE_PHASE3_SCALE_2_DBP

Normal Q-Q Plots

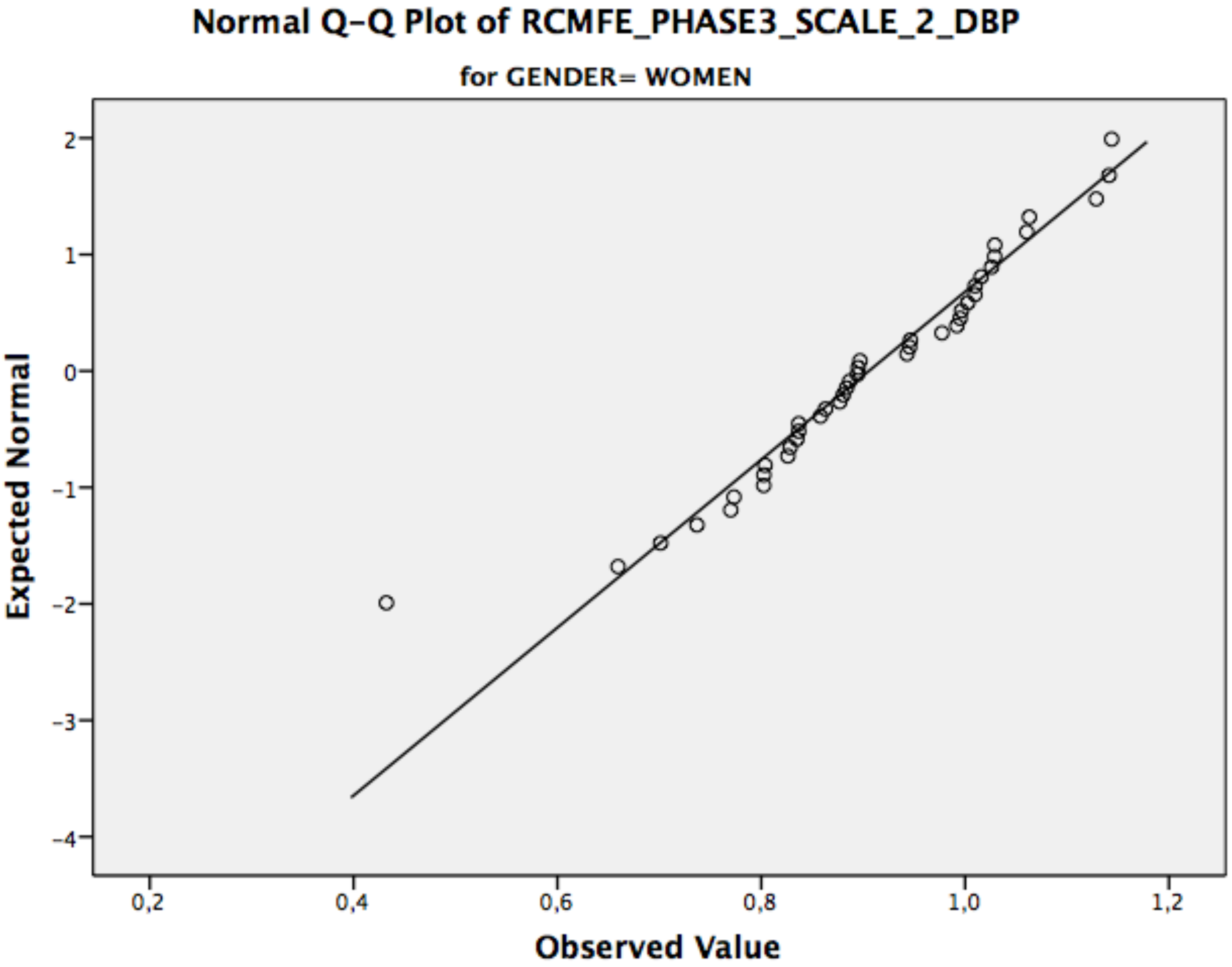

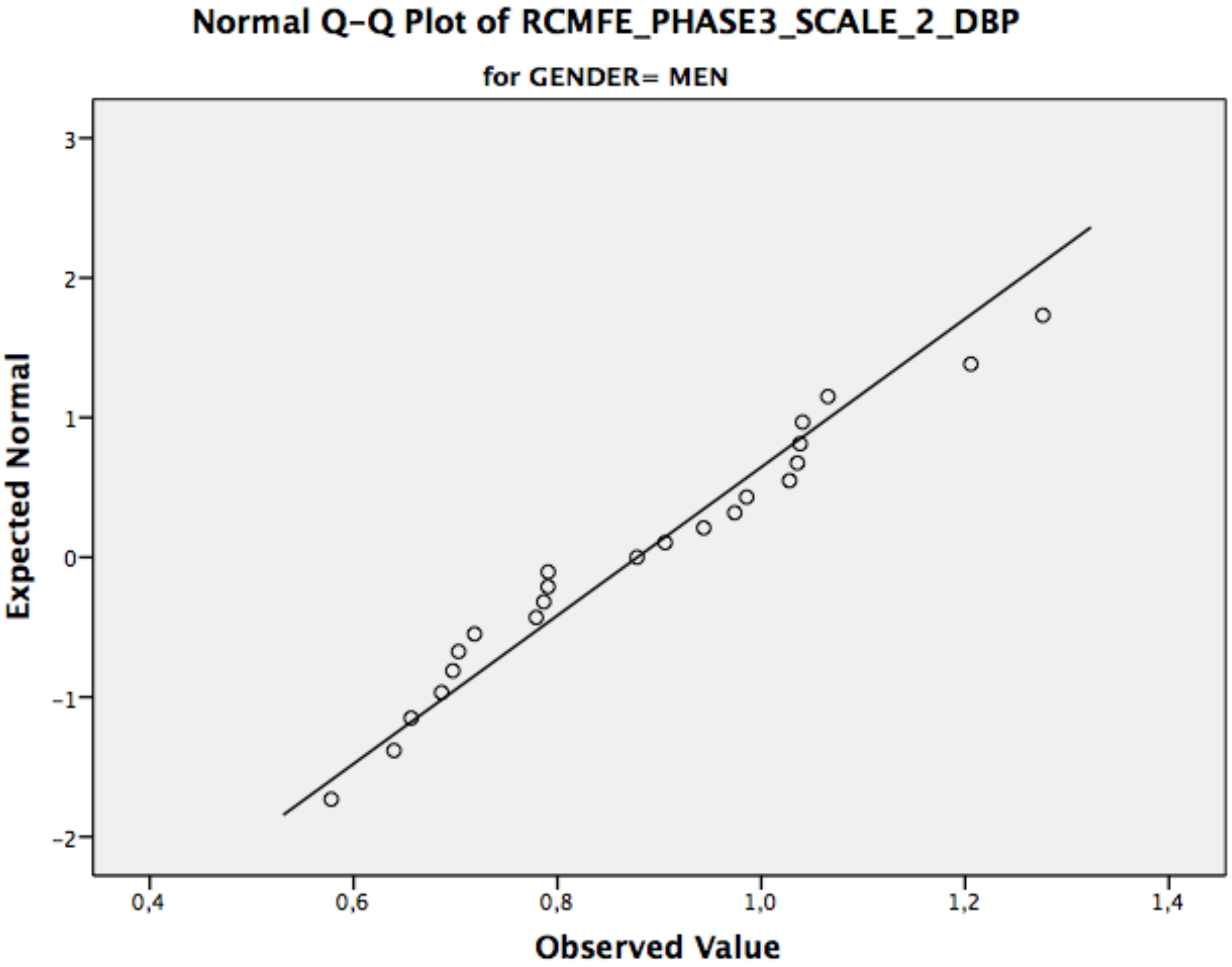

Normal Q-Q Plot of RCMFE_PHASE3_SCALE_2_DBP
for GENDER= MEN
Expected Normal
Observed Value
3
2
1
0
-1
-2
0,4
0,6
0,8
1,0
1,2
1,4

RCMFE_PHASE3_SCALE_3_DBP

Normal Q-Q Plots

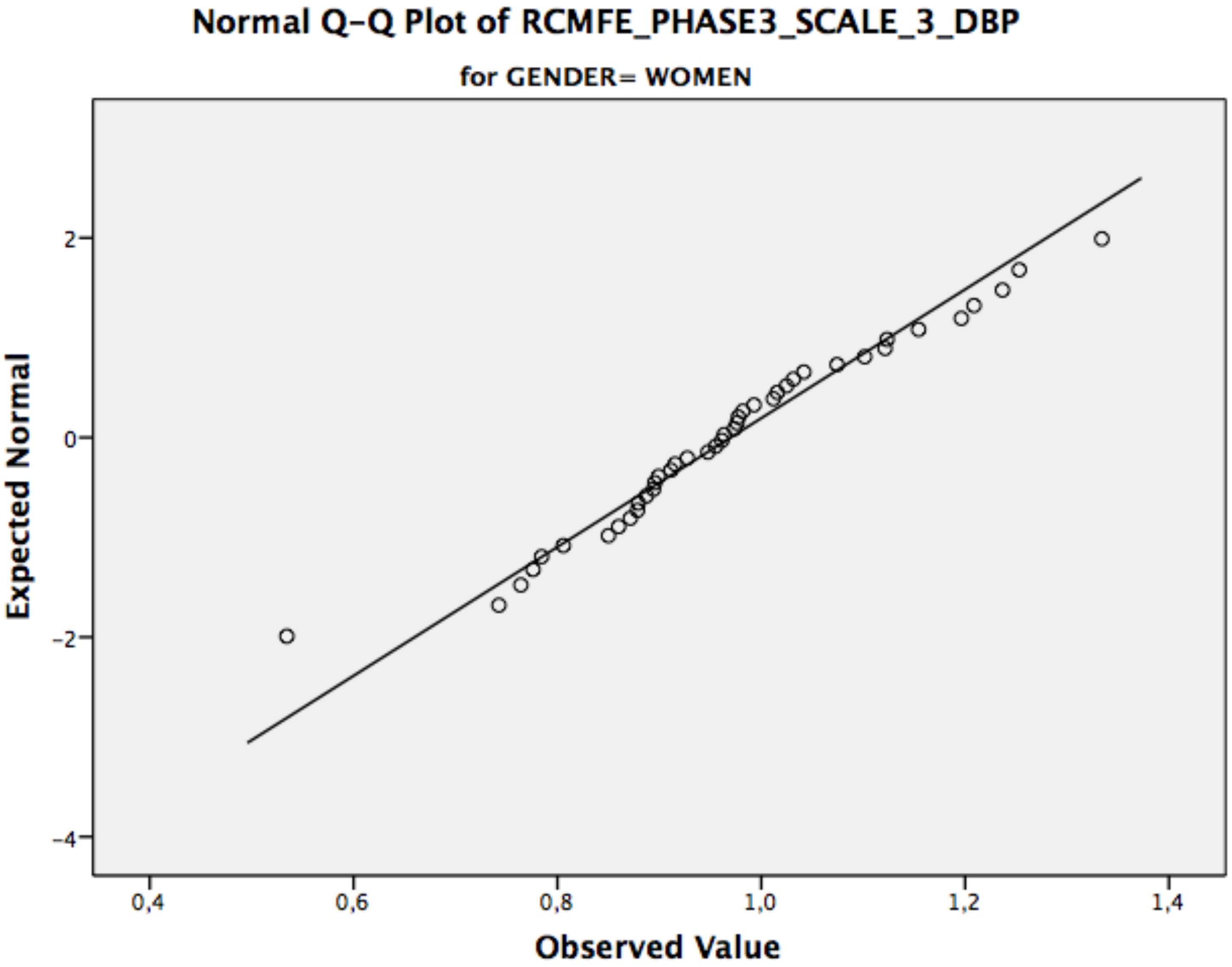

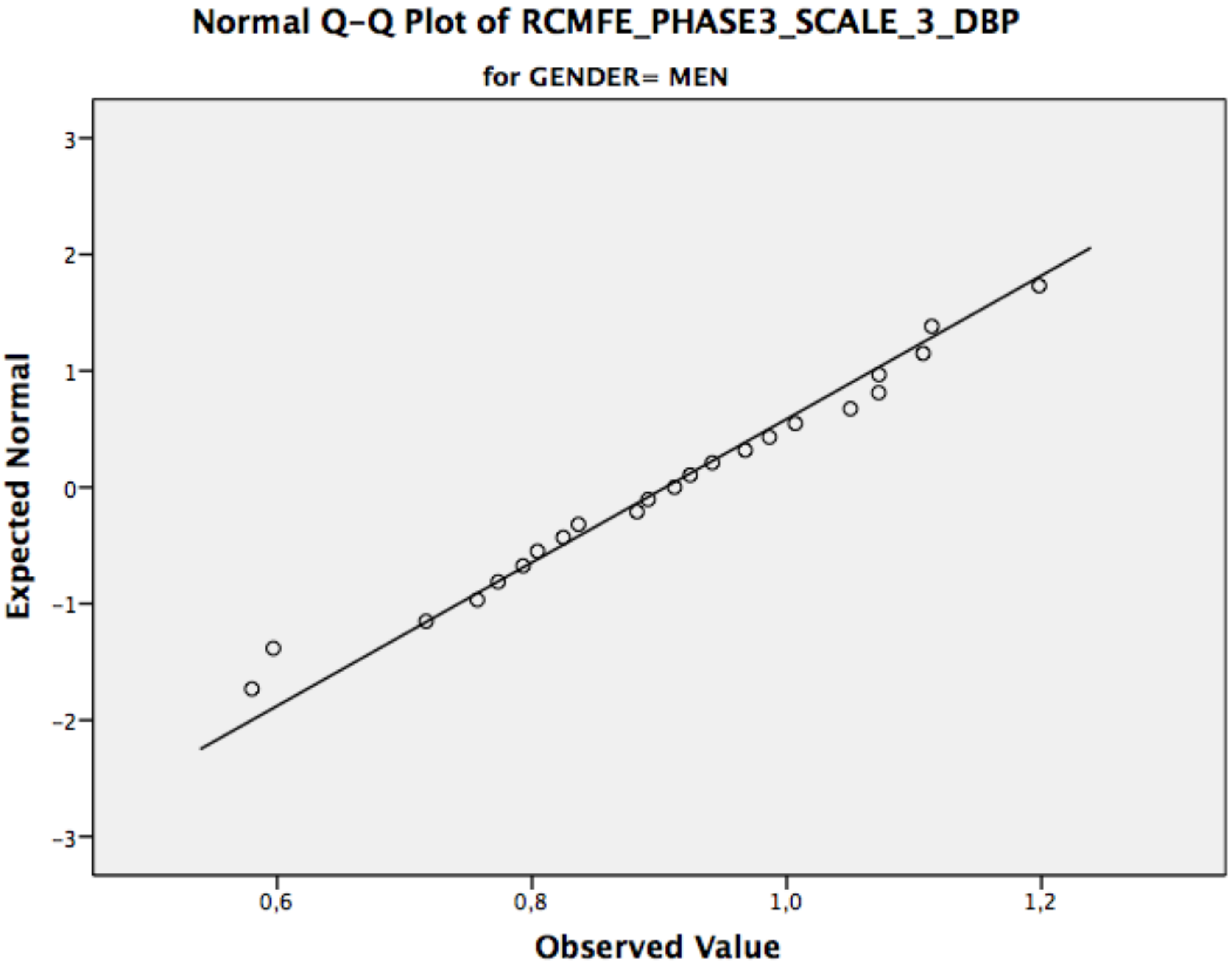

Normal Q-Q Plot of RCMFE_PHASE3_SCALE_3_DBP
for GENDER= MEN
Expected Normal
Observed Value
3
2
1
0
-1
-2
-3
0,6
0,8
1,0
1,2

RCMFE_PHASE3_SCALE_4_DBP

Normal Q-Q Plots

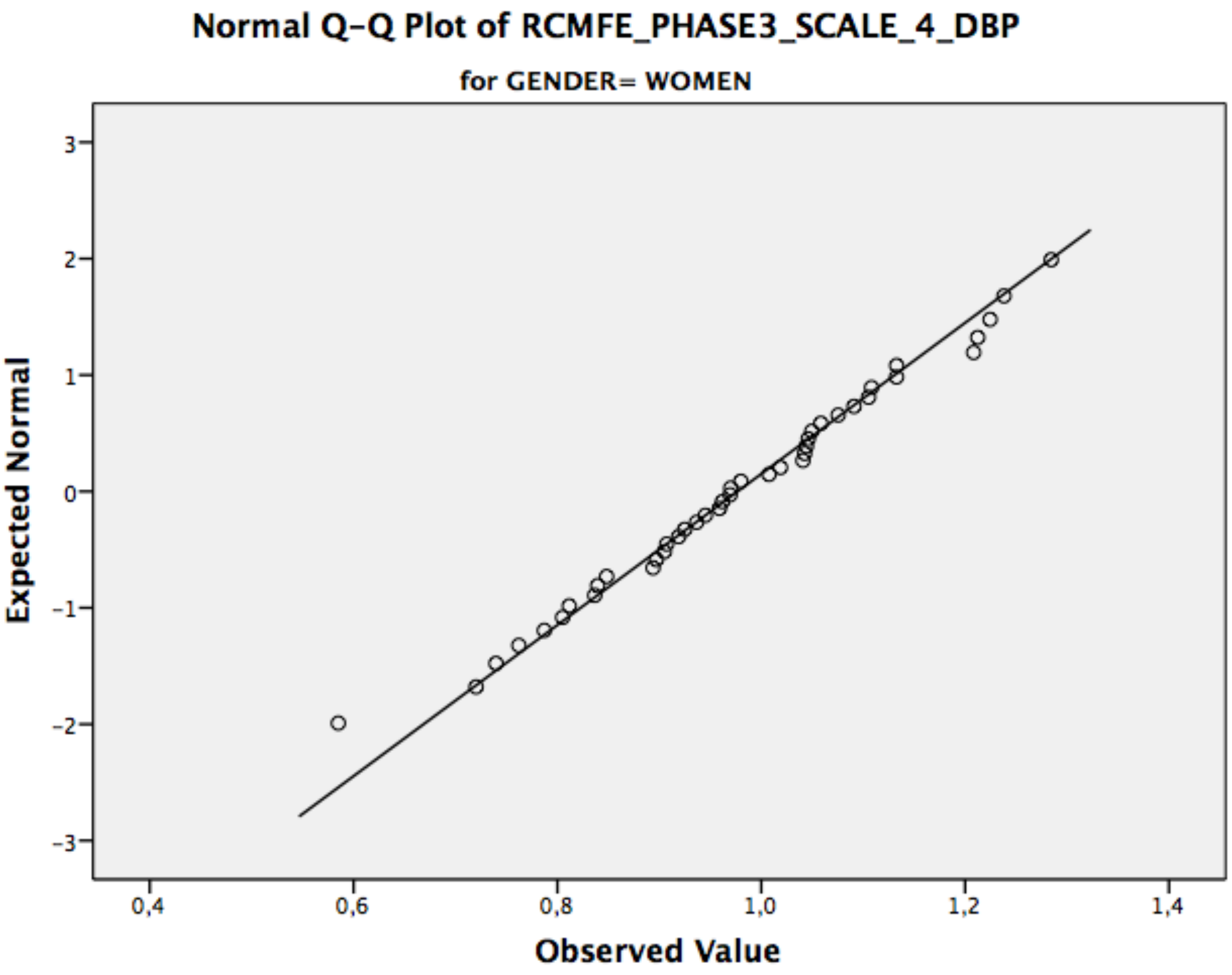

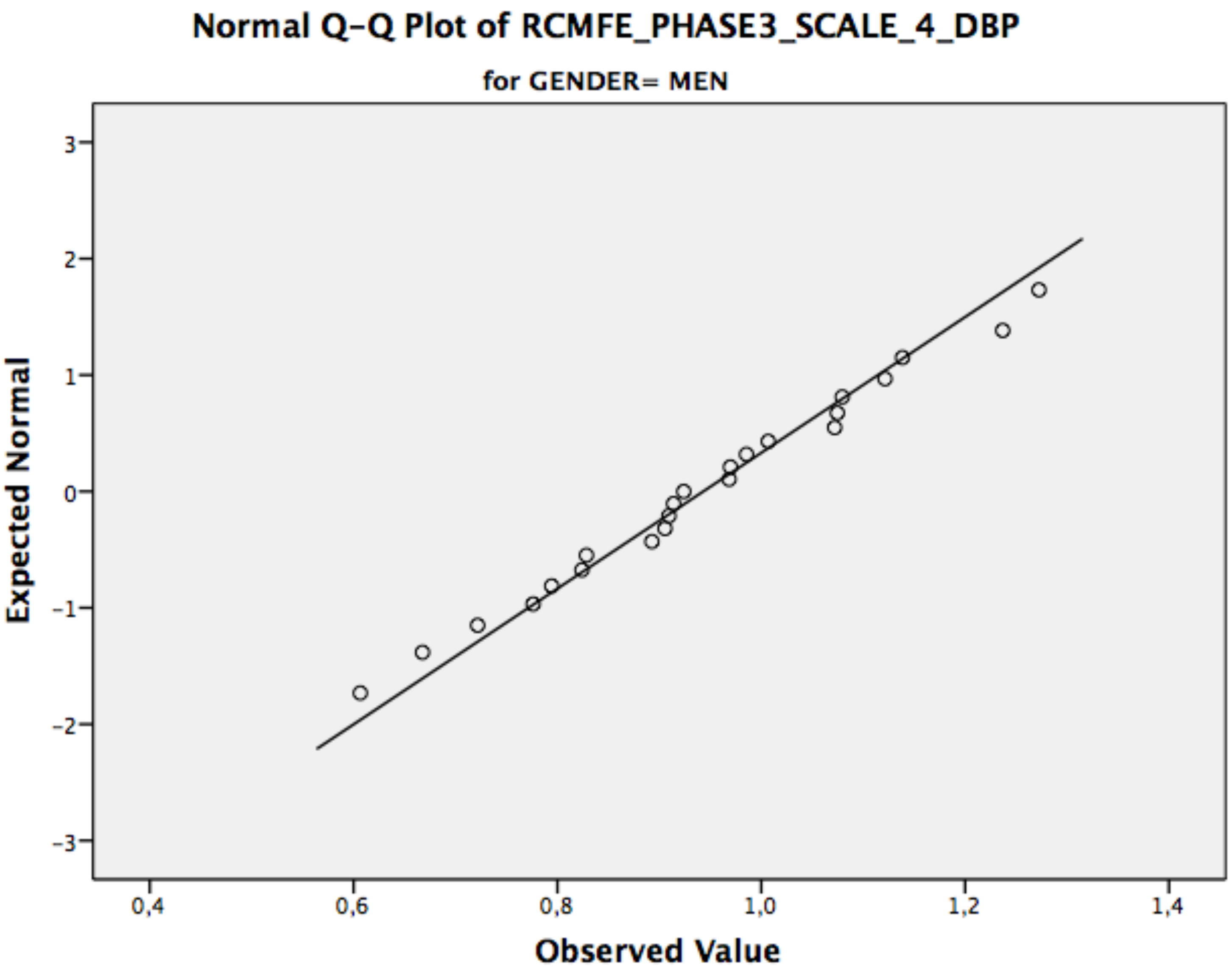

Normal Q-Q Plot of RCMFE_PHASE3_SCALE_4_DBP
for GENDER= MEN
Expected Normal
Observed Value
3
2
1
0
-1
-2
-3
0,4
0,6
0,8
1,0
1,2
1,4

RCMFE_PHASE3_SCALE_5_DBP

Normal Q-Q Plots

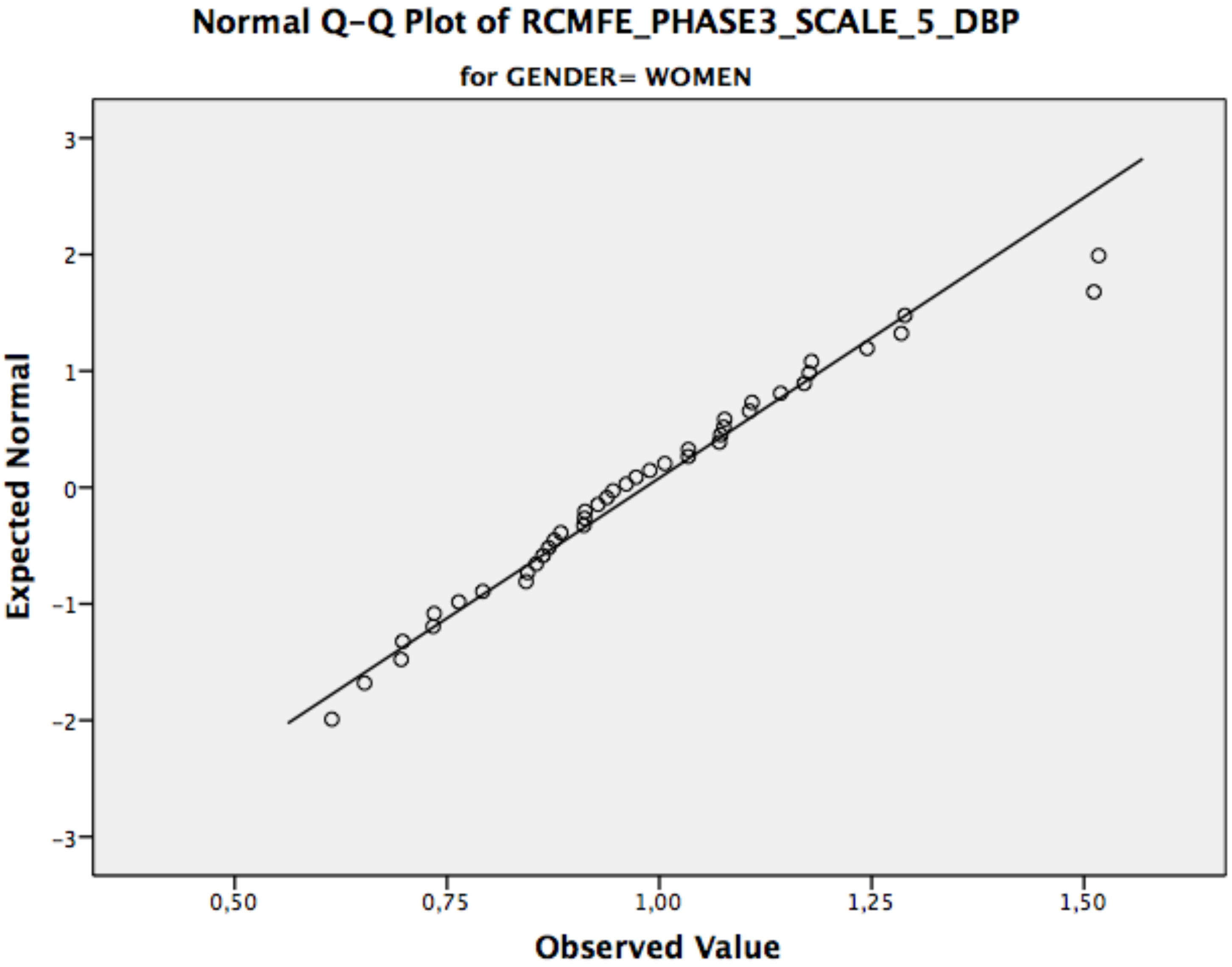

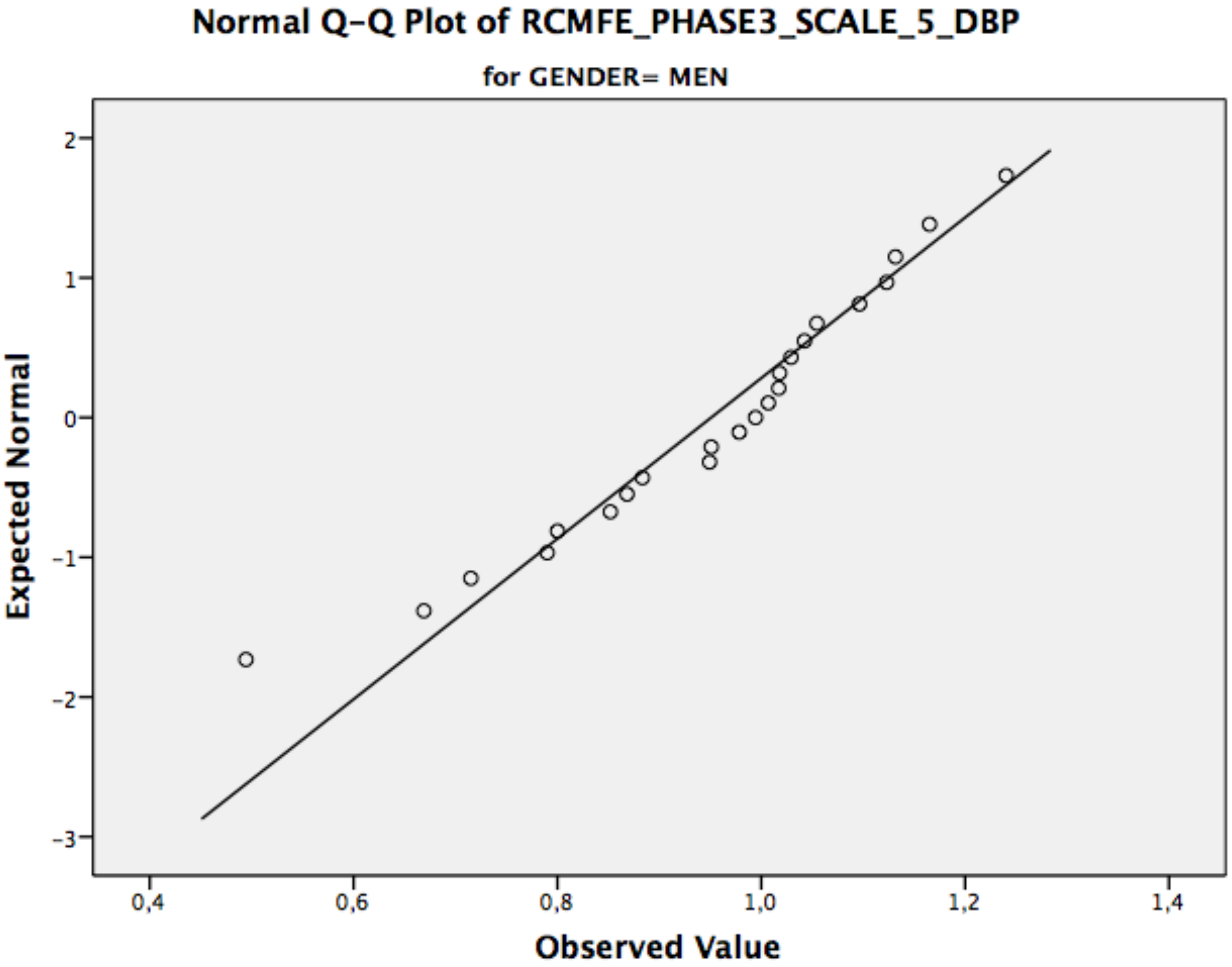
Normal Q-Q Plot of RCMFE_PHASE3_SCALE_5_DBP
for GENDER= MEN
Expected Normal
2
1
0
-1
-2
-3
0,4
0,6
0,8
1,0
1,2
1,4
Observed Value

RCMFE_PHASE4_SCALE_1_DBP

Normal Q-Q Plots

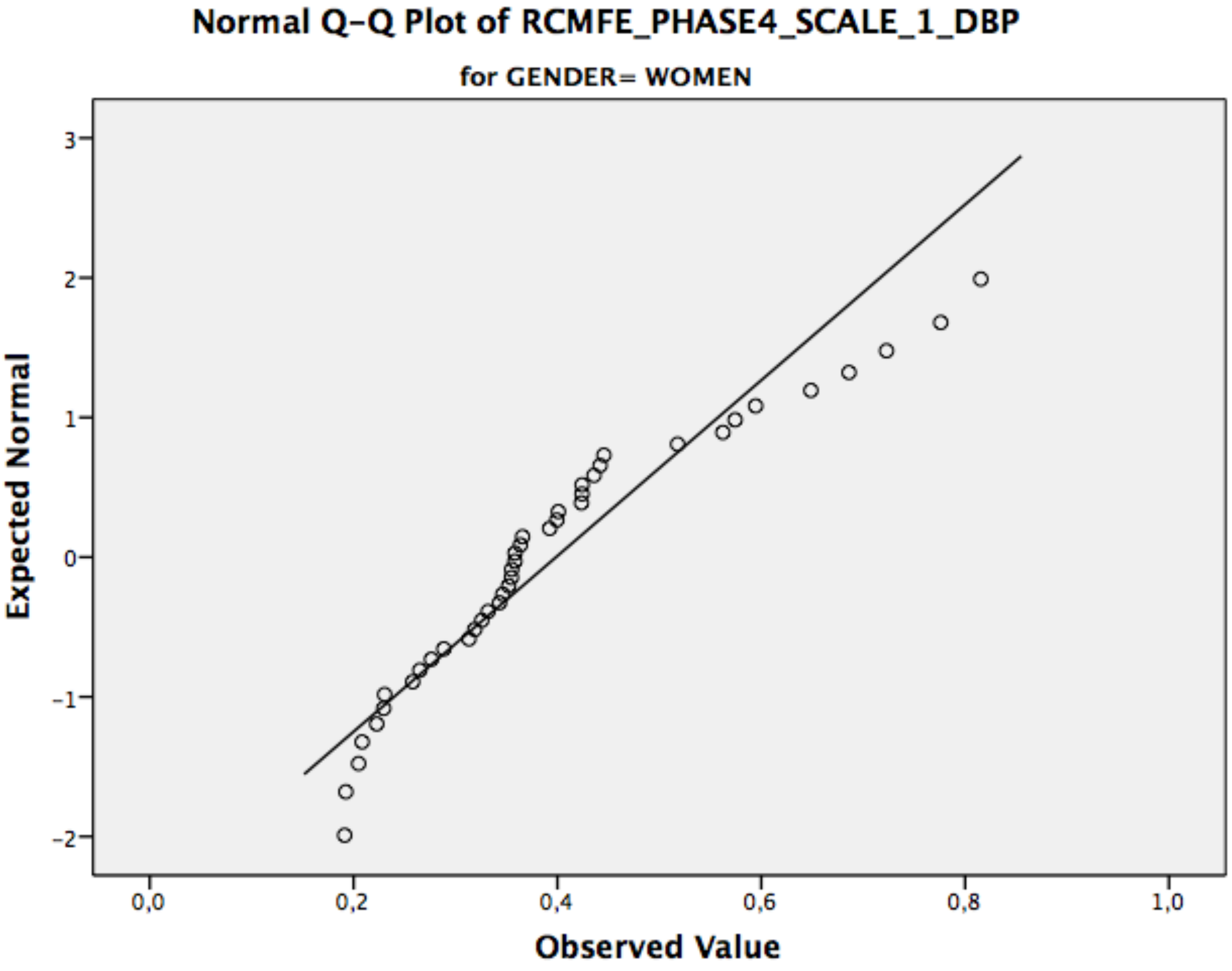

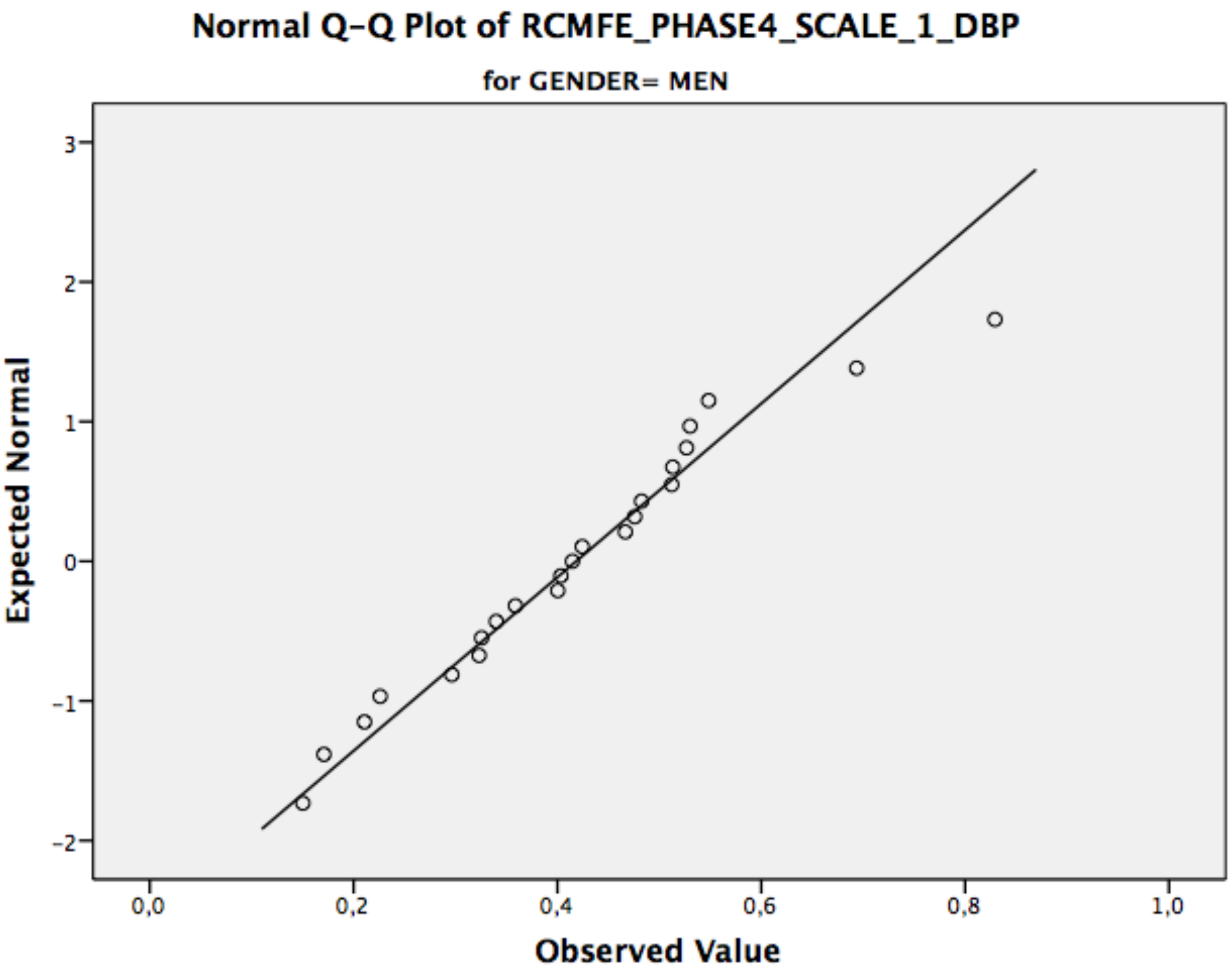
Normal Q-Q Plot of RCMFE_PHASE4_SCALE_1_DBP
for GENDER= MEN
Expected Normal
Observed Value
3
2
1
0
-1
-2
0,0
0,2
0,4
0,6
0,8
1,0

RCMFE_PHASE4_SCALE_2_DBP

Normal Q-Q Plots

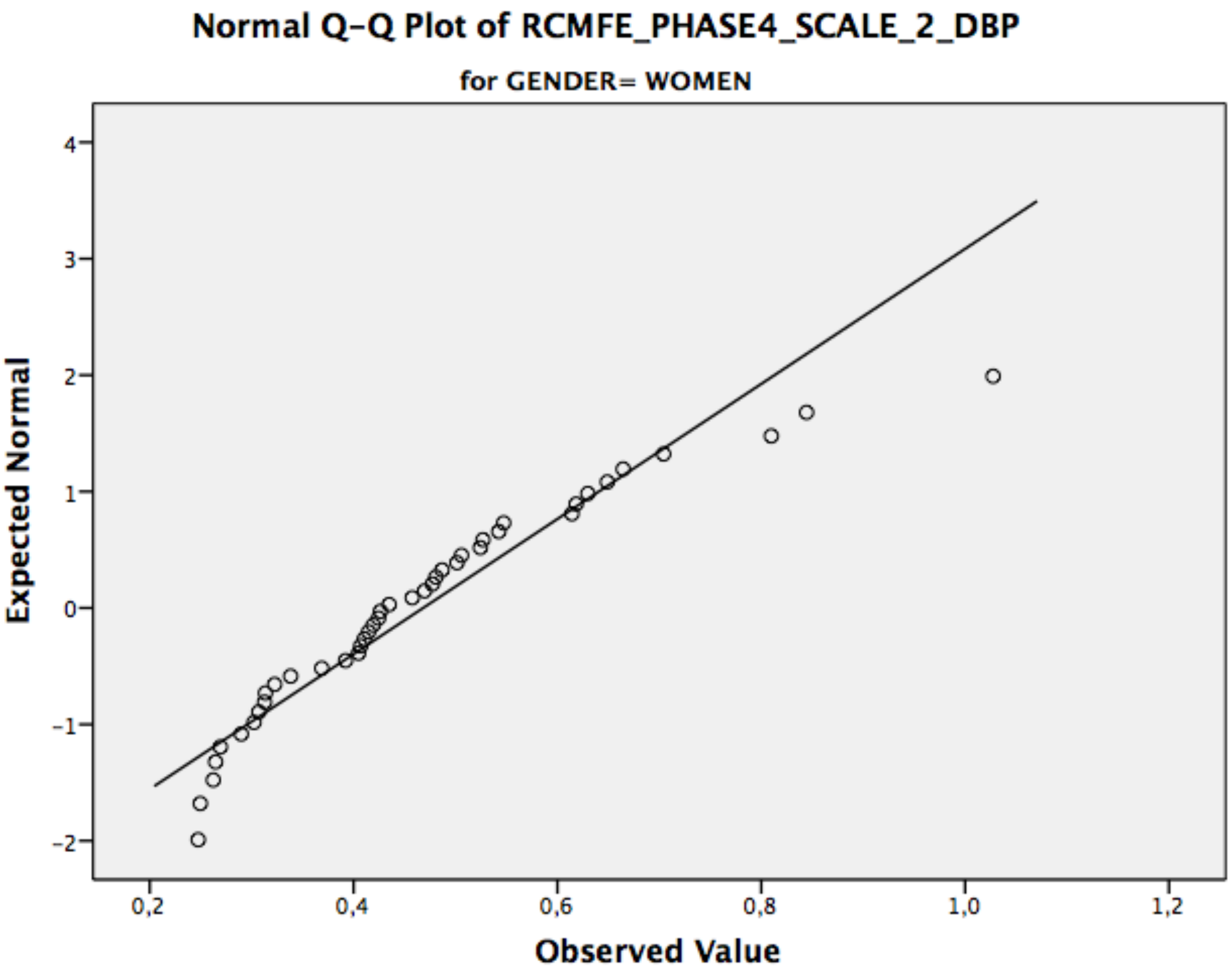

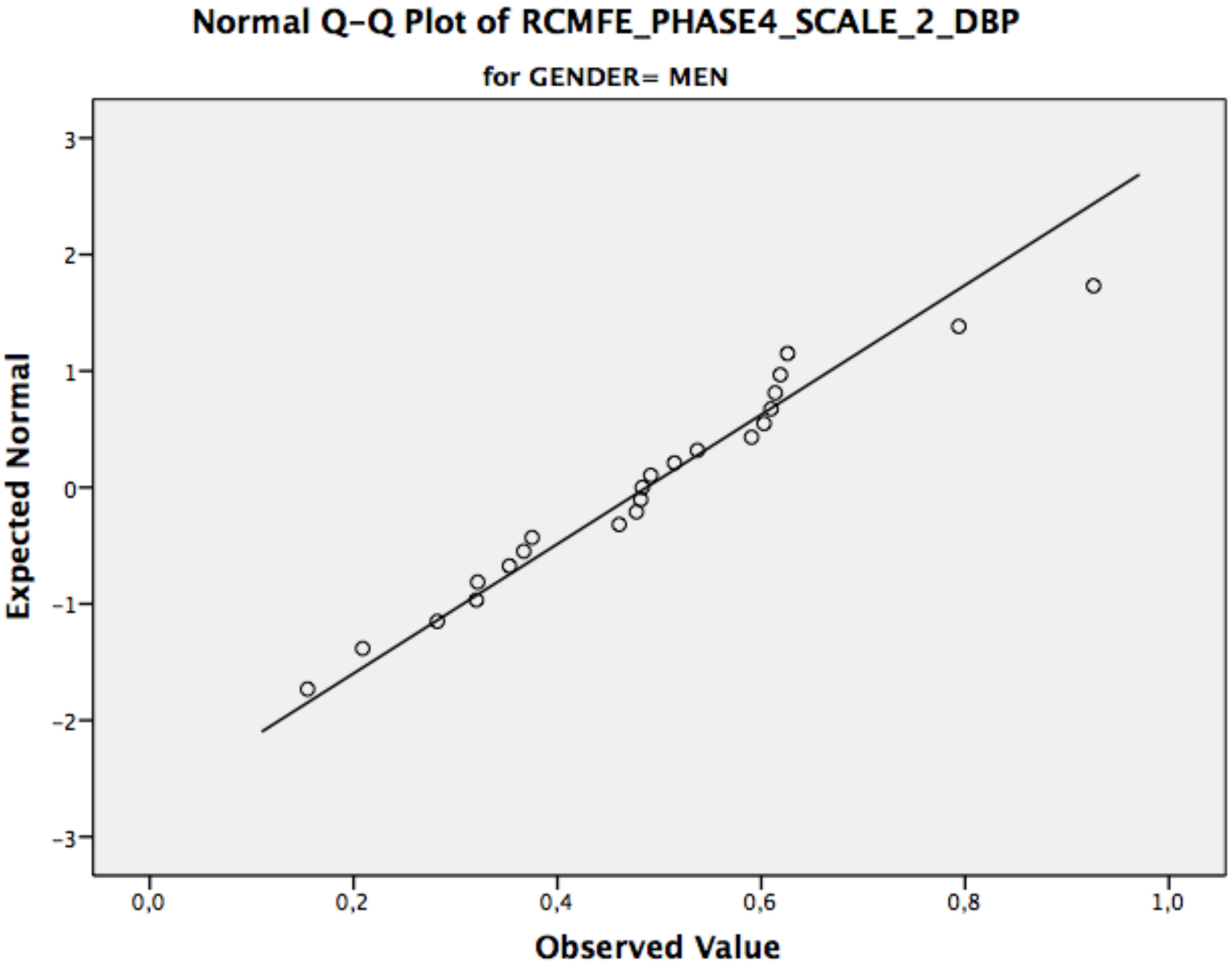
Normal Q-Q Plot of RCMFE_PHASE4_SCALE_2_DBP
for GENDER= MEN
Expected Normal
Observed Value
3
2
1
0
-1
-2
-3
0,0
0,2
0,4
0,6
0,8
1,0

RCMFE_PHASE4_SCALE_3_DBP

Normal Q-Q Plots

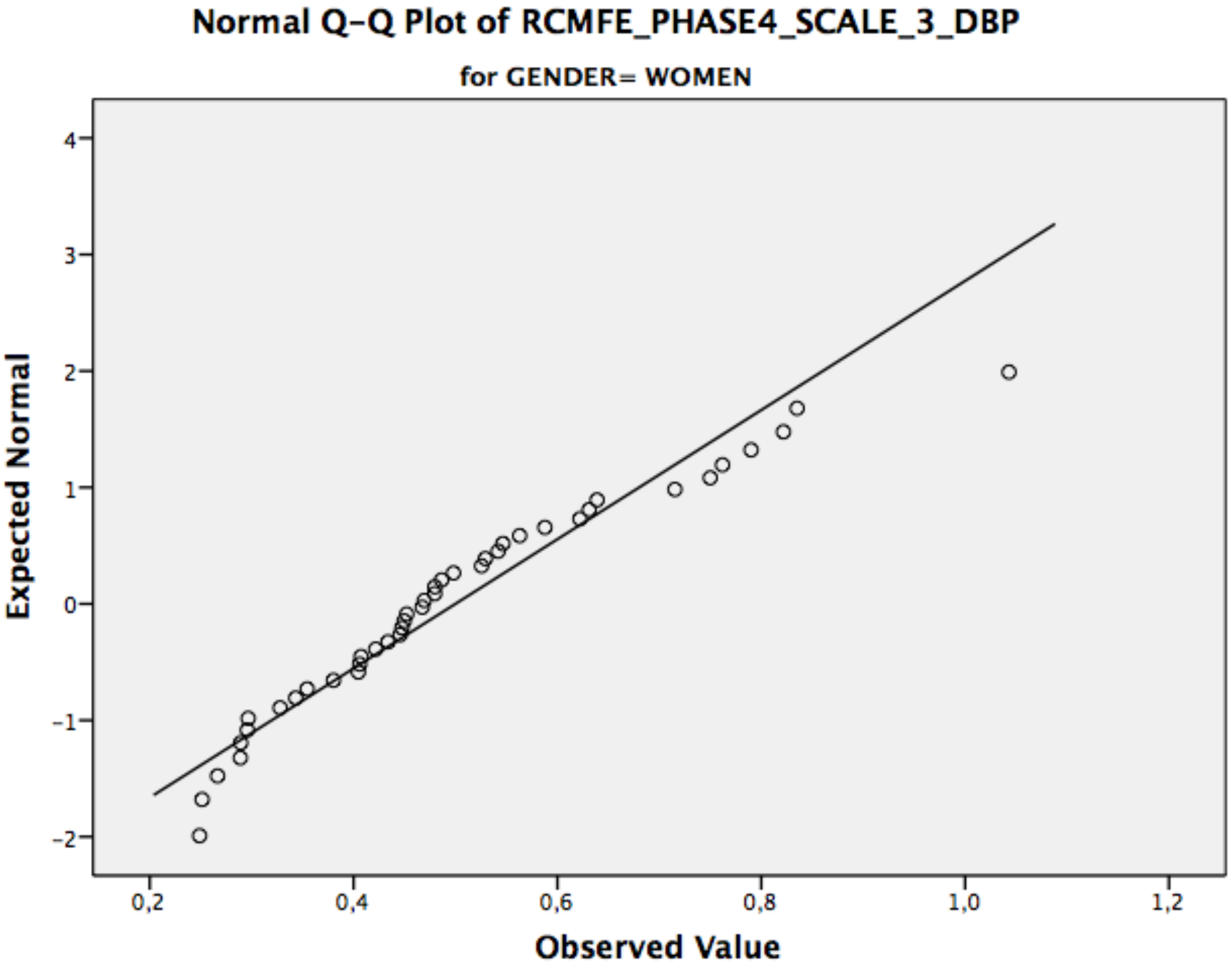

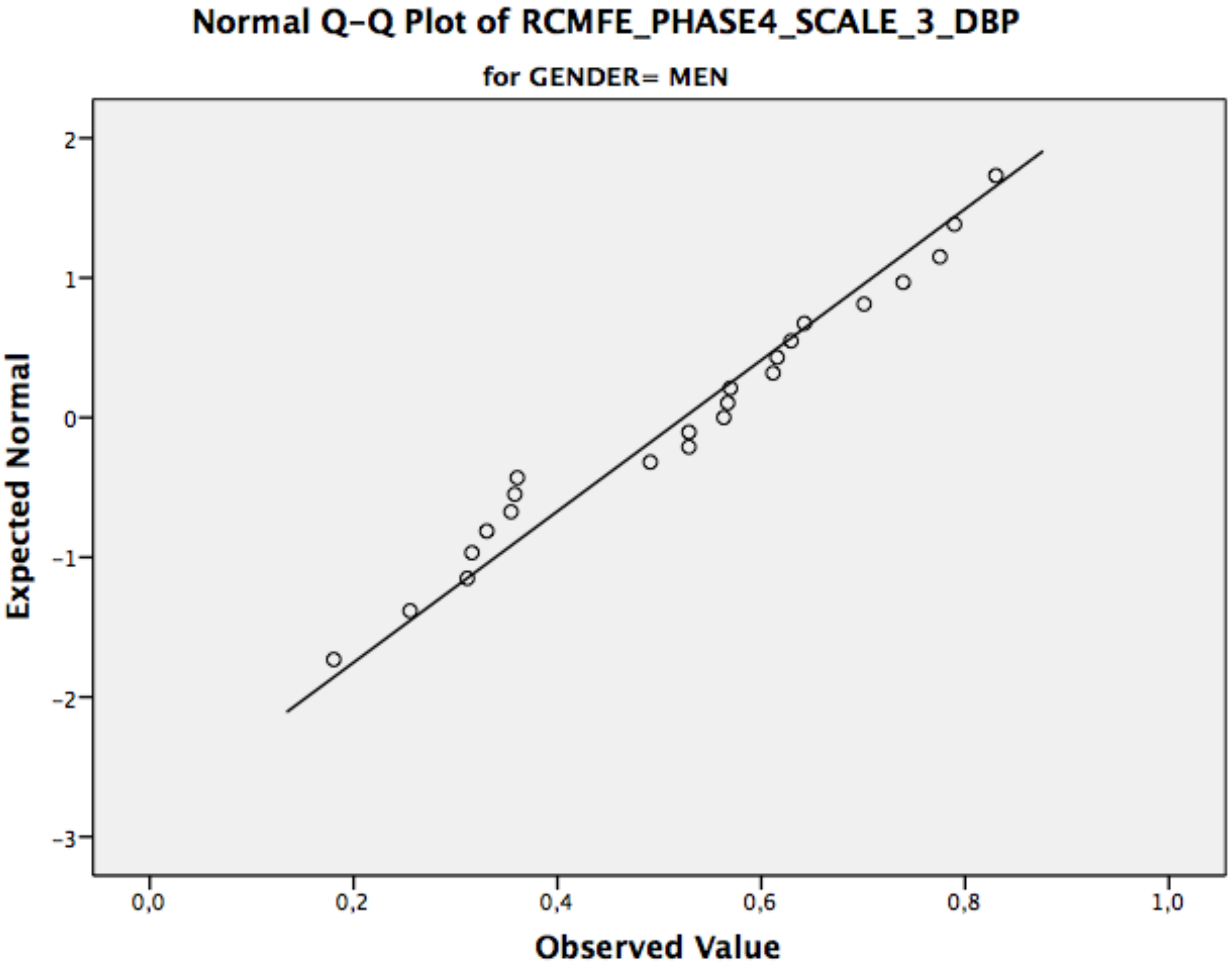

Normal Q-Q Plot of RCMFE_PHASE4_SCALE_3_DBP
for GENDER= MEN
Expected Normal
2
1
0
-1
-2
-3
0,0
0,2
0,4
0,6
0,8
1,0
Observed Value

RCMFE_PHASE4_SCALE_4_DBP

Normal Q-Q Plots

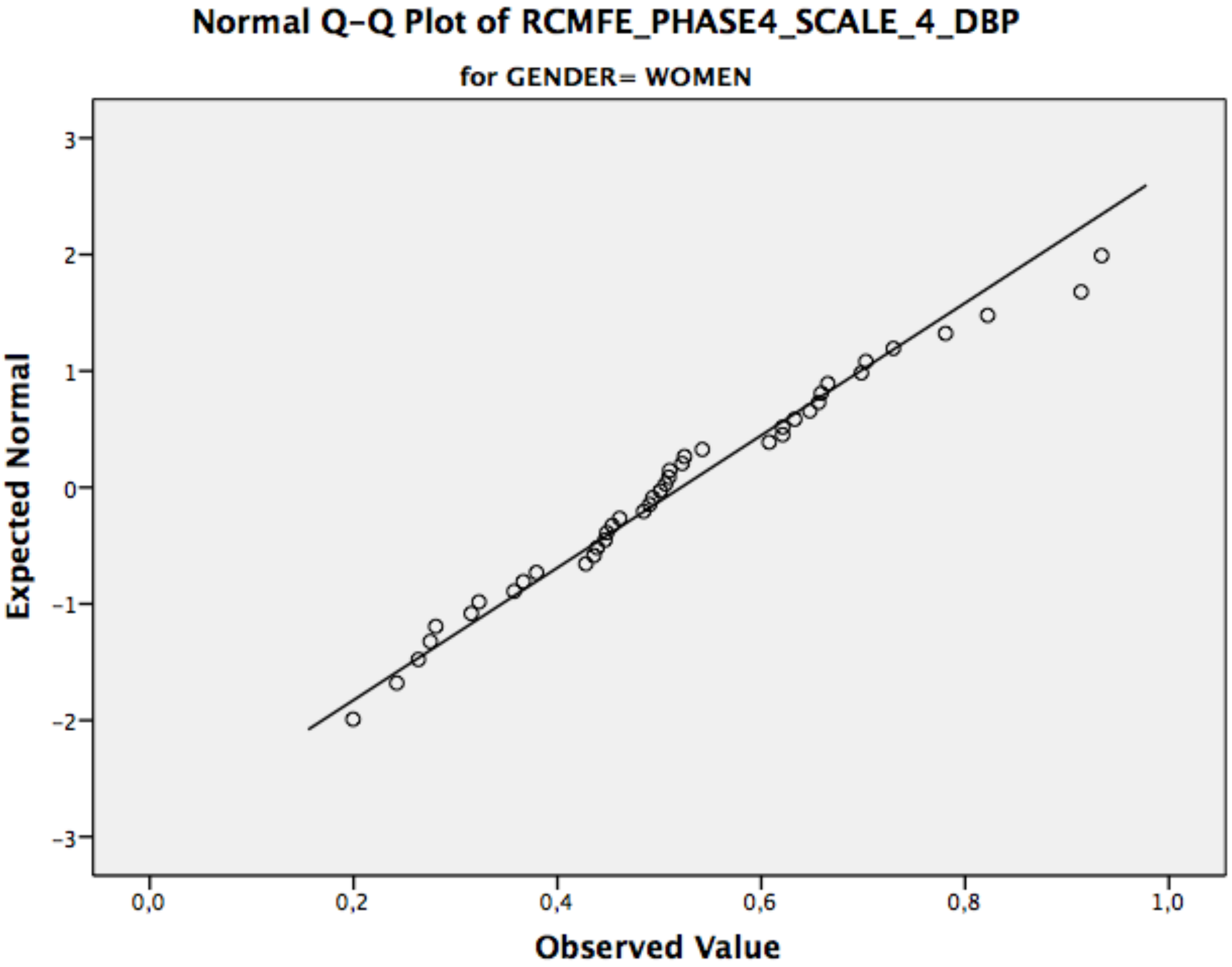

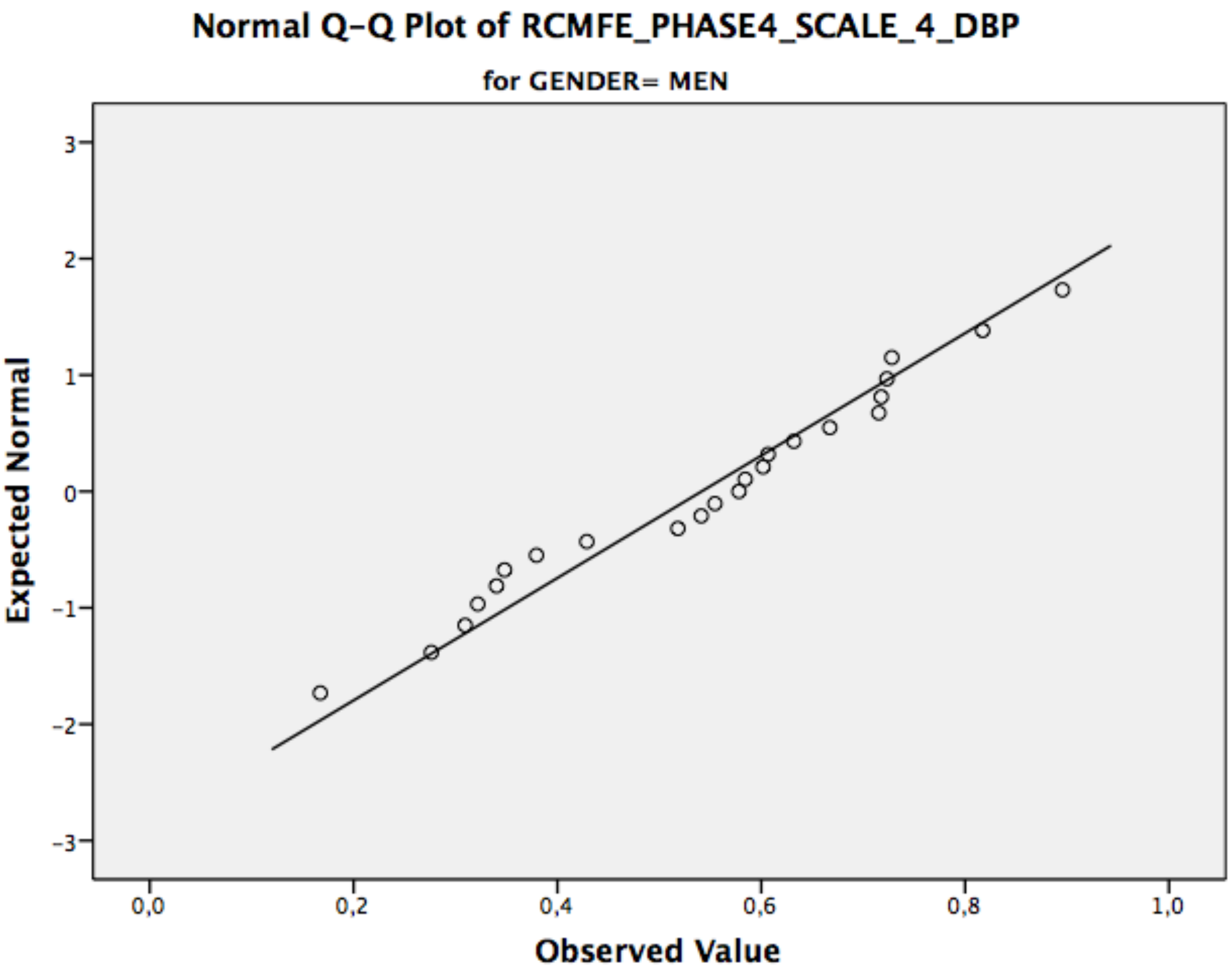
Normal Q-Q Plot of RCMFE_PHASE4_SCALE_4_DBP
for GENDER= MEN
Expected Normal
Observed Value
3
2
1
0
−1
−2
−3
0,0
0,2
0,4
0,6
0,8
1,0

RCMFE_PHASE4_SCALE_5_DBP

Normal Q-Q Plots

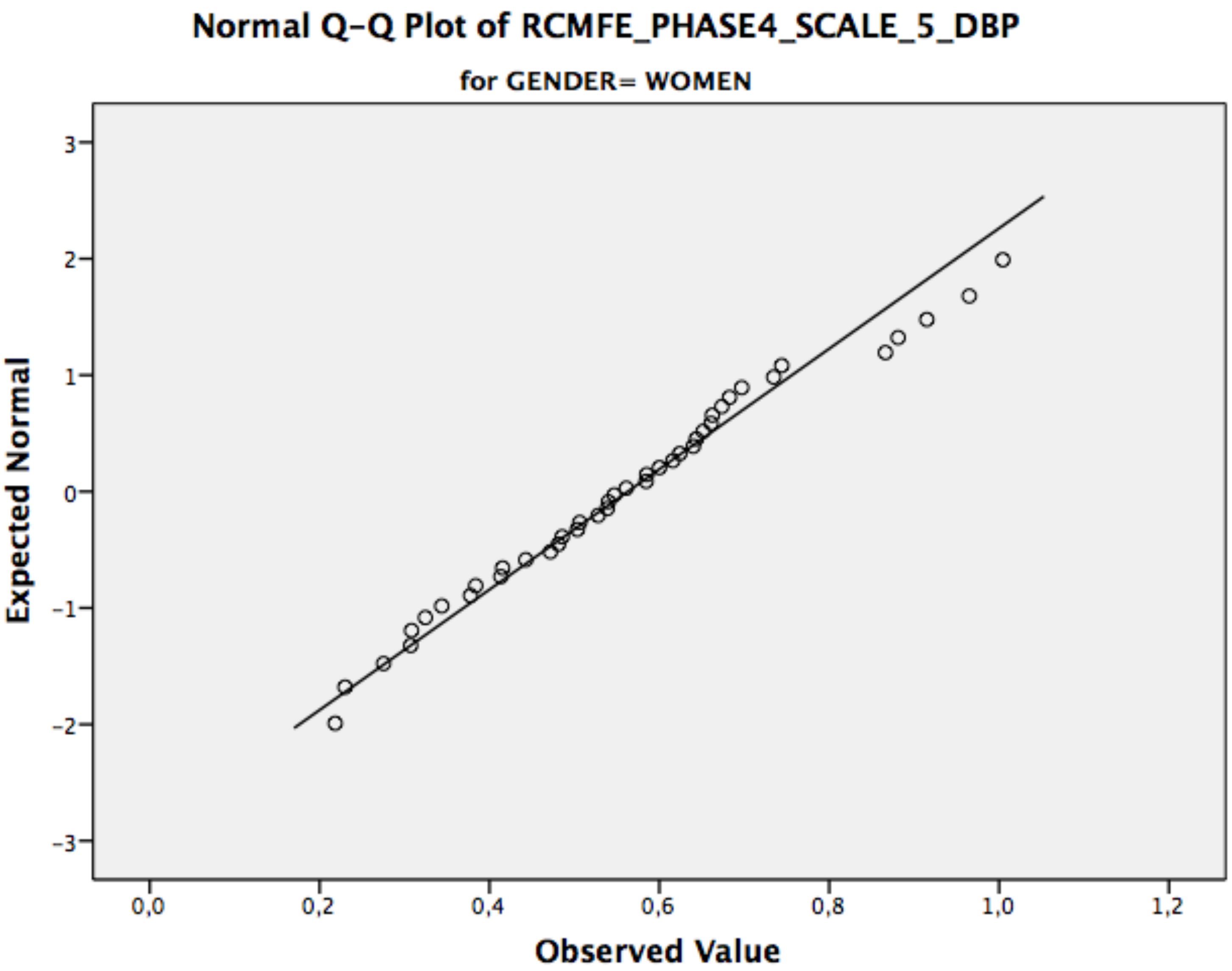

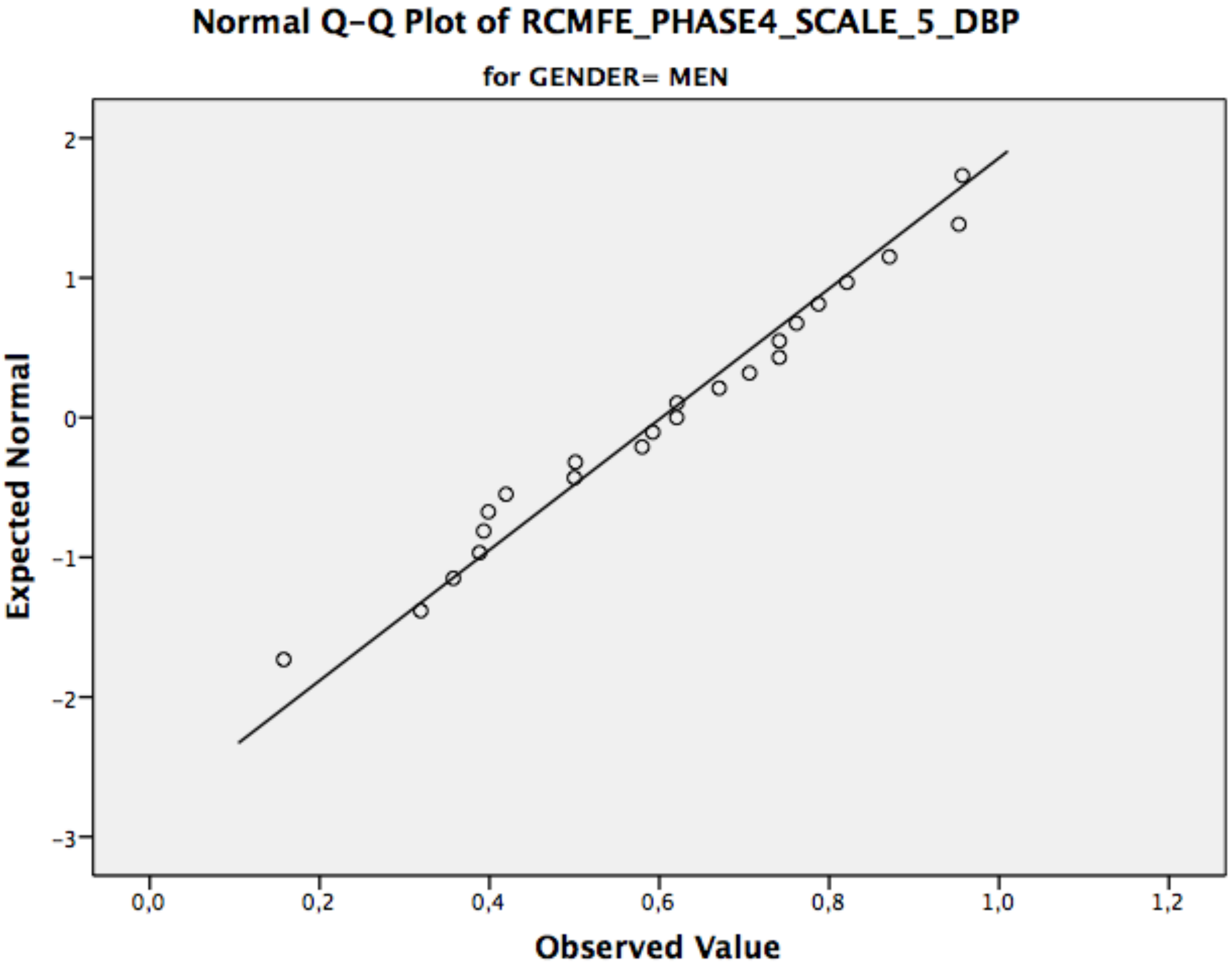

Normal Q-Q Plot of RCMFE_PHASE4_SCALE_5_DBP
for GENDER= MEN
Expected Normal
Observed Value
2
1
0
-1
-2
-3
0,0
0,2
0,4
0,6
0,8
1,0
1,2

RCMFE_PHASE5_SCALE_1_DBP

Normal Q-Q Plots

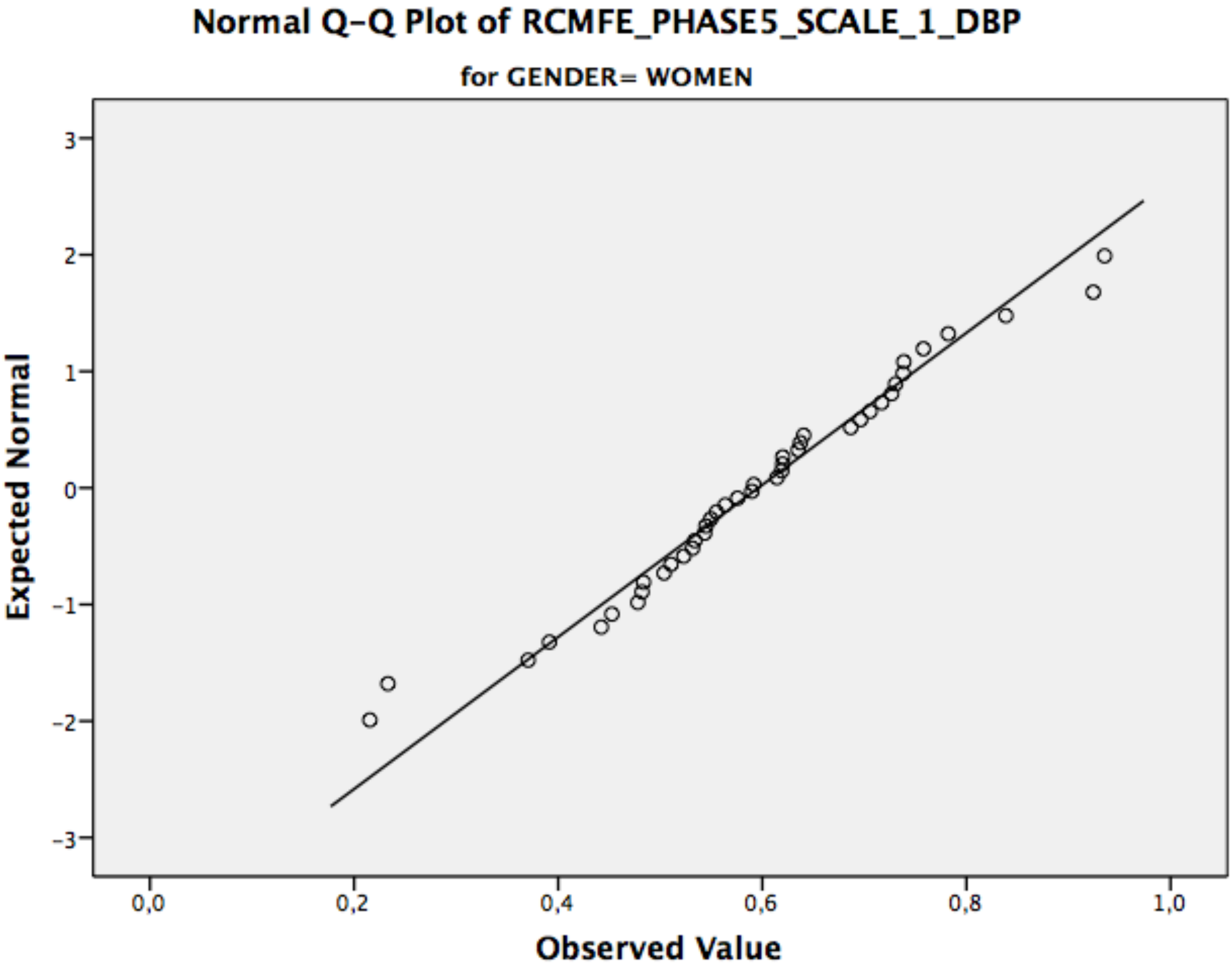

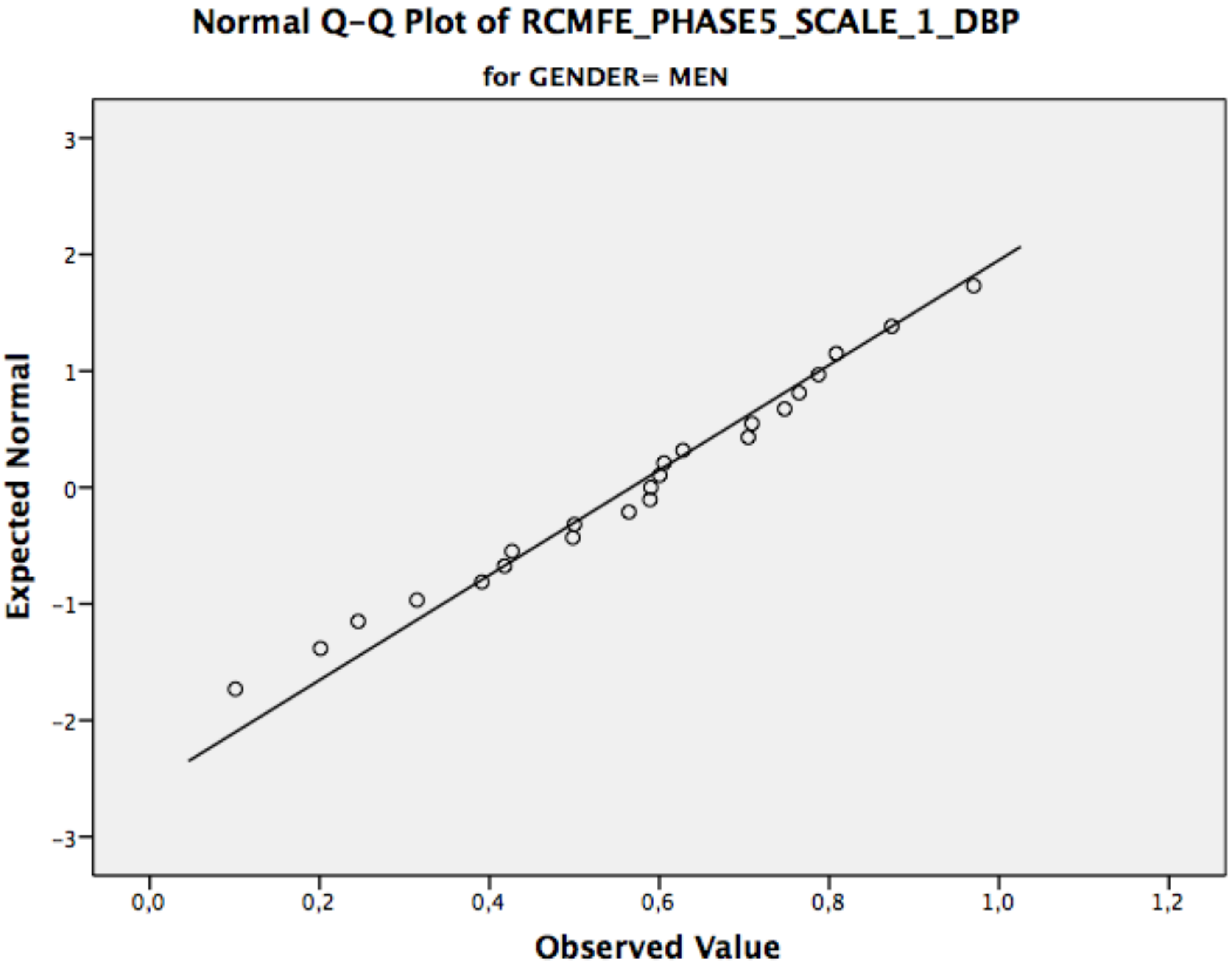

Normal Q-Q Plot of RCMFE_PHASE5_SCALE_1_DBP
for GENDER= MEN
Expected Normal
3
2
1
0
-1
-2
-3
0,0
0,2
0,4
0,6
0,8
1,0
1,2
Observed Value

RCMFE_PHASE5_SCALE_2_DBP

Normal Q-Q Plots

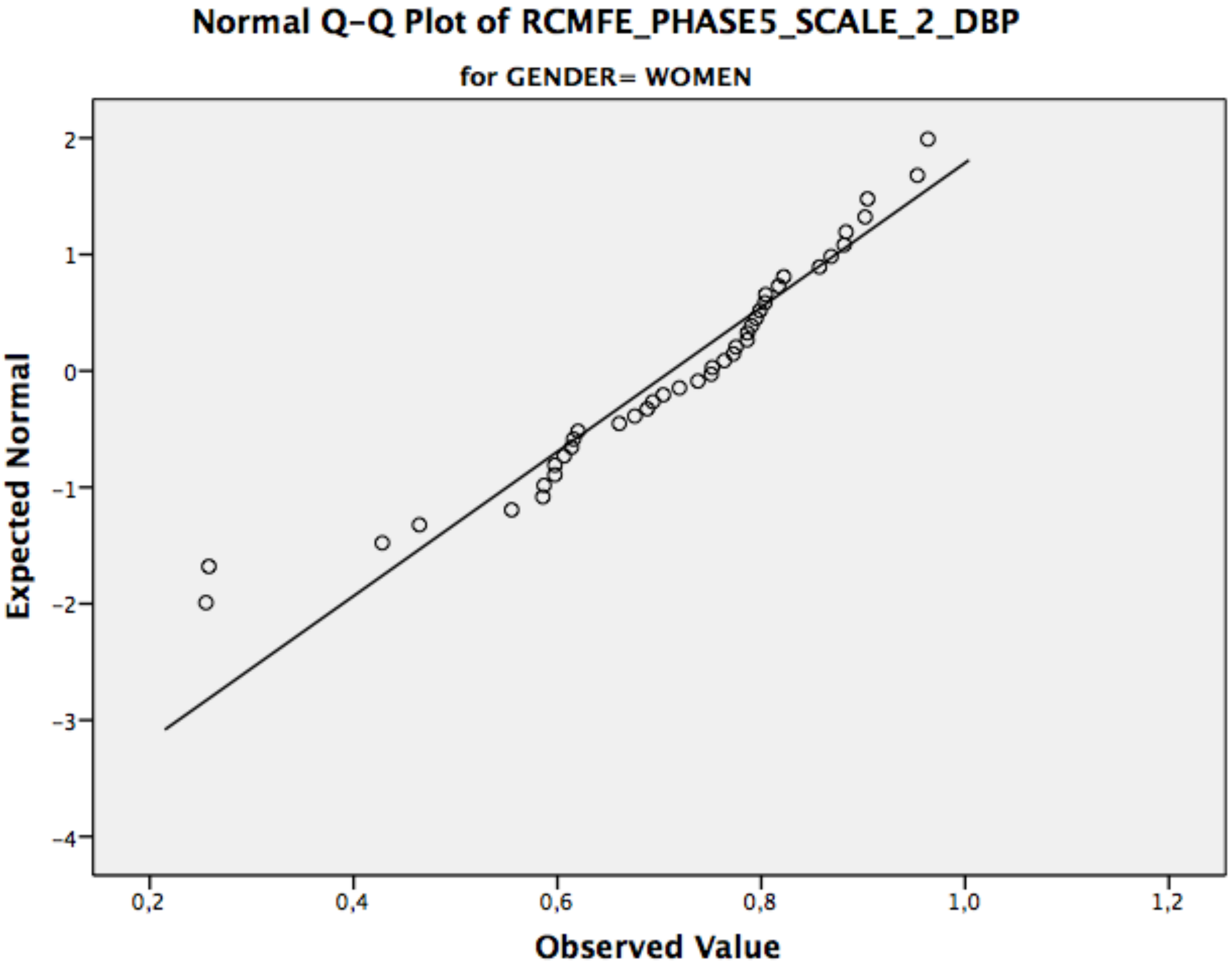

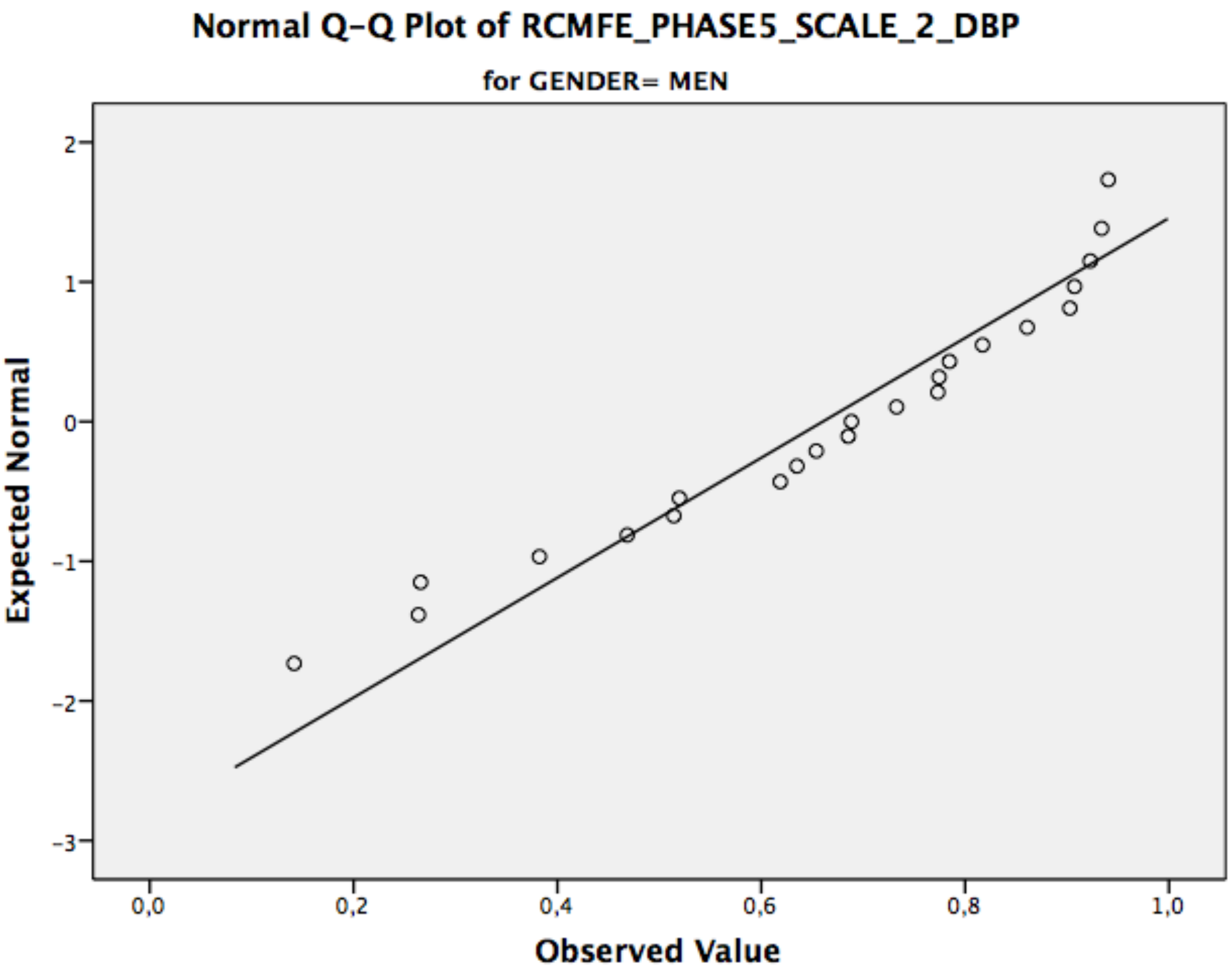
Normal Q-Q Plot of RCMFE_PHASE5_SCALE_2_DBP
for GENDER= MEN
Expected Normal
Observed Value
2
1
0
-1
-2
-3
0,0
0,2
0,4
0,6
0,8
1,0

RCMFE_PHASE5_SCALE_3_DBP

Normal Q-Q Plots

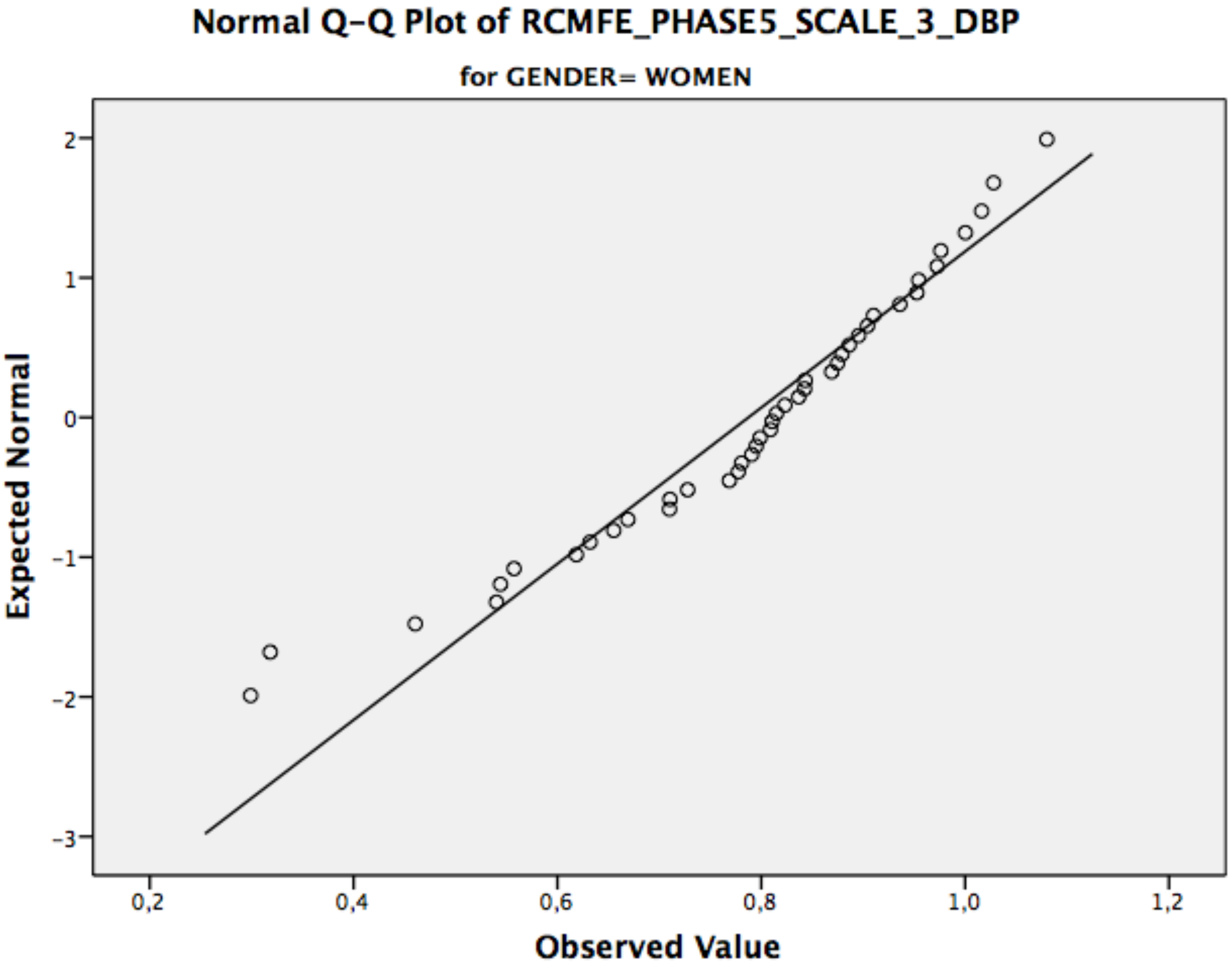

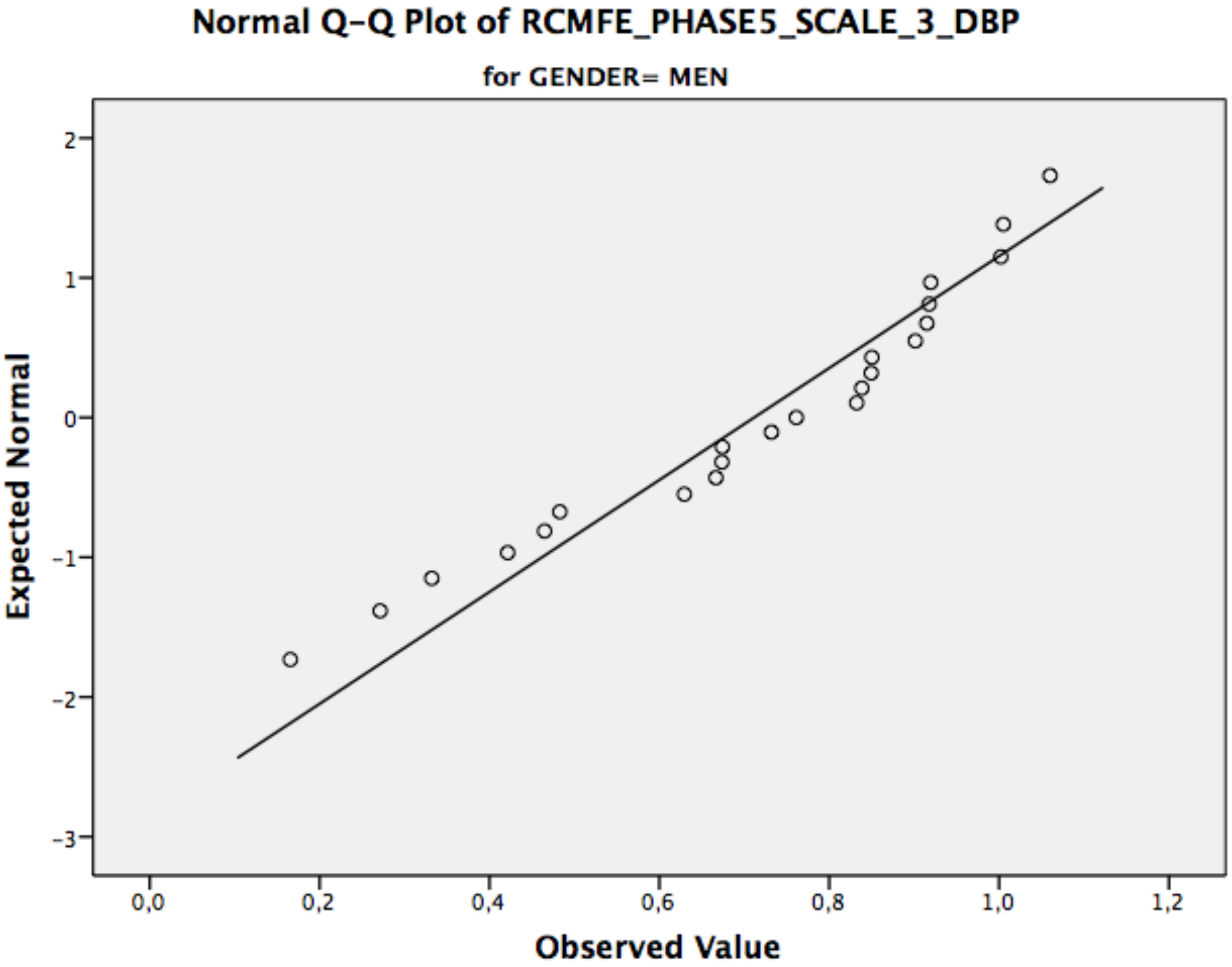

Normal Q-Q Plot of RCMFE_PHASE5_SCALE_3_DBP
for GENDER= MEN
Expected Normal
Observed Value
2
1
0
-1
-2
-3
0,0
0,2
0,4
0,6
0,8
1,0
1,2

RCMFE_PHASE5_SCALE_4_DBP

Normal Q-Q Plots

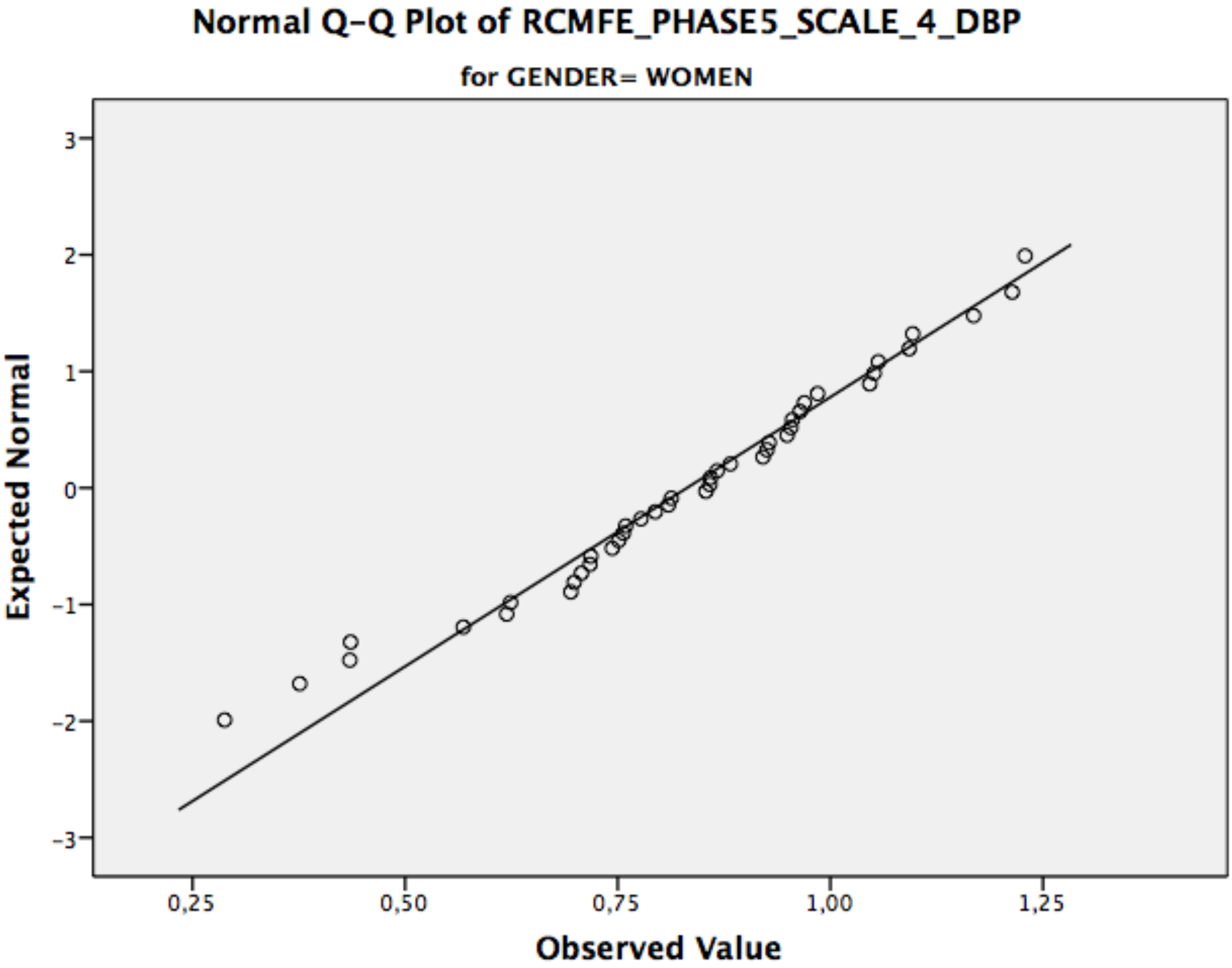

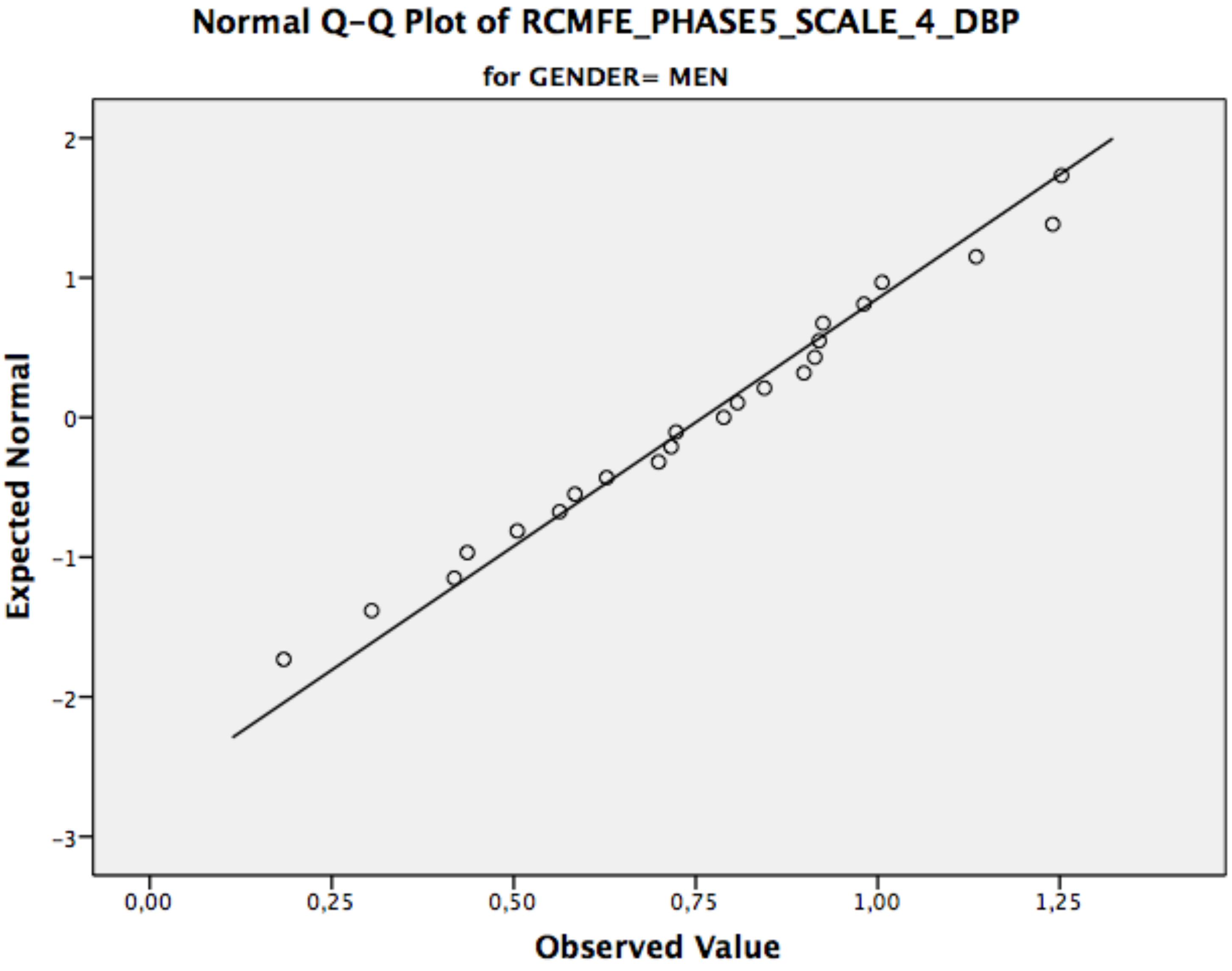
Normal Q-Q Plot of RCMFE_PHASE5_SCALE_4_DBP
for GENDER= MEN
Expected Normal
Observed Value
2
1
0
-1
-2
-3
0,00
0,25
0,50
0,75
1,00
1,25

RCMFE_PHASE5_SCALE_5_DBP

Normal Q-Q Plots

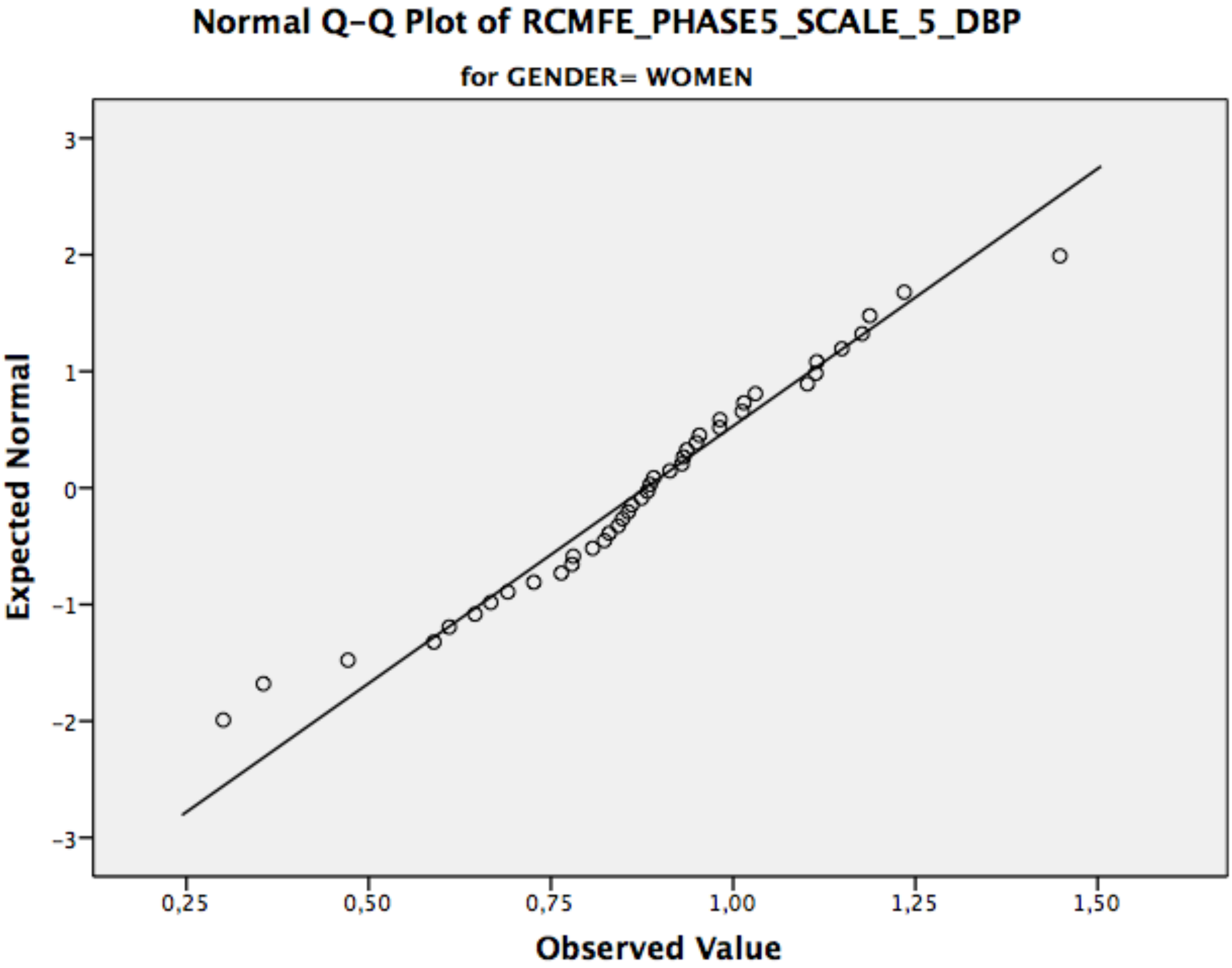

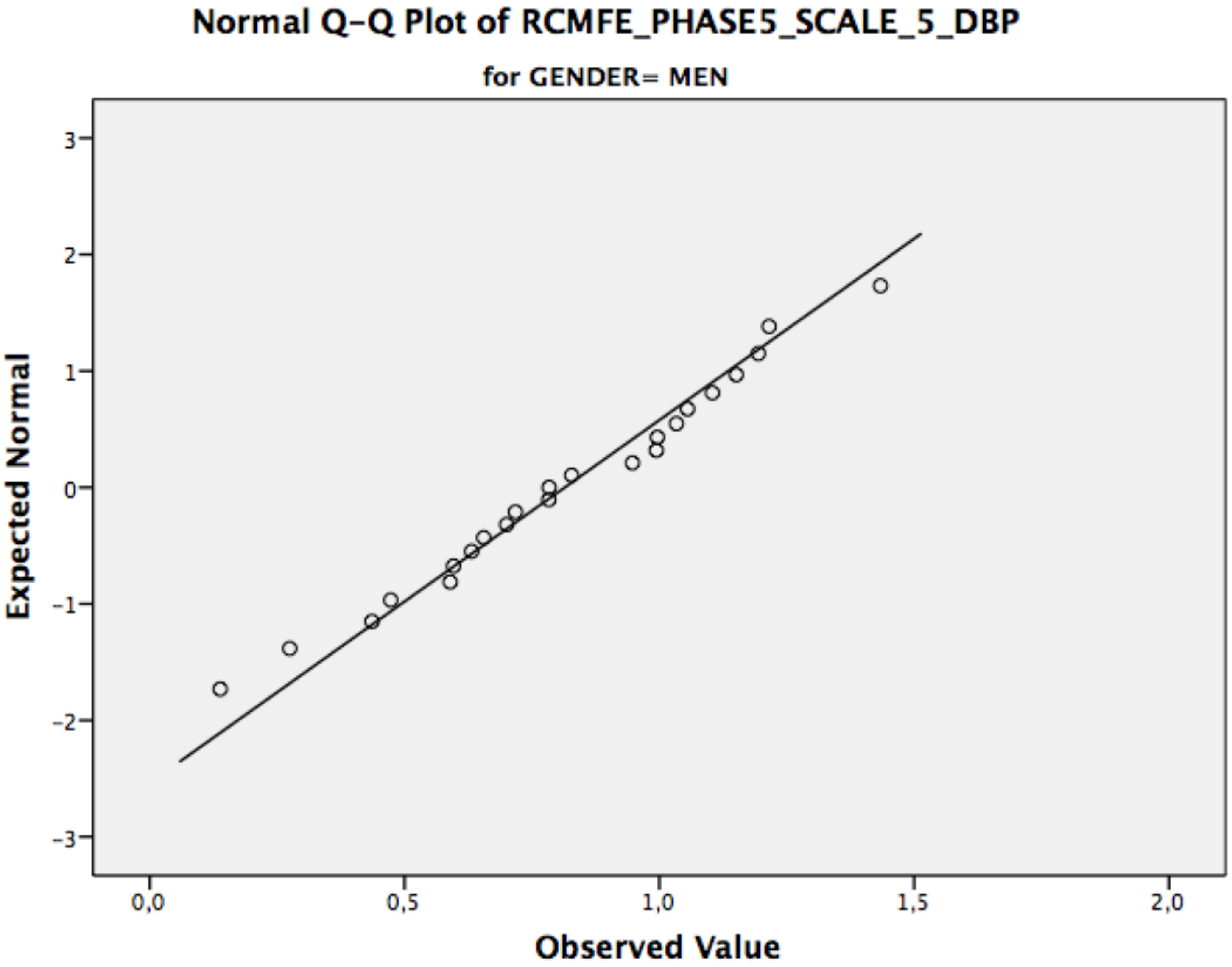
Normal Q-Q Plot of RCMFE_PHASE5_SCALE_5_DBP
for GENDER= MEN
Expected Normal
Observed Value
3
2
1
0
-1
-2
-3
0,0
0,5
1,0
1,5
2,0

Explore DBP

PATHOLOGY

Case Processing Summary

	PATHOLOGY	Cases					
		Valid		Missing		Total	
		N	Percent	N	Percent	N	Percent
RCMFE_PHASE1_SCALE_1_DBP	NO-OI	29	100,0%	0	0,0%	29	100,0%
	OI	36	100,0%	0	0,0%	36	100,0%
RCMFE_PHASE1_SCALE_2_DBP	NO-OI	29	100,0%	0	0,0%	29	100,0%
	OI	36	100,0%	0	0,0%	36	100,0%
RCMFE_PHASE1_SCALE_3_DBP	NO-OI	29	100,0%	0	0,0%	29	100,0%
	OI	36	100,0%	0	0,0%	36	100,0%
RCMFE_PHASE1_SCALE_4_DBP	NO-OI	29	100,0%	0	0,0%	29	100,0%
	OI	36	100,0%	0	0,0%	36	100,0%
RCMFE_PHASE1_SCALE_5_DBP	NO-OI	29	100,0%	0	0,0%	29	100,0%
	OI	36	100,0%	0	0,0%	36	100,0%
RCMFE_PHASE2_SCALE_1_DBP	NO-OI	29	100,0%	0	0,0%	29	100,0%
	OI	36	100,0%	0	0,0%	36	100,0%
RCMFE_PHASE2_SCALE_2_DBP	NO-OI	29	100,0%	0	0,0%	29	100,0%
	OI	36	100,0%	0	0,0%	36	100,0%
RCMFE_PHASE2_SCALE_3_DBP	NO-OI	29	100,0%	0	0,0%	29	100,0%
	OI	36	100,0%	0	0,0%	36	100,0%
RCMFE_PHASE2_SCALE_4_DBP	NO-OI	29	100,0%	0	0,0%	29	100,0%
	OI	36	100,0%	0	0,0%	36	100,0%
RCMFE_PHASE2_SCALE_5_DBP	NO-OI	29	100,0%	0	0,0%	29	100,0%
	OI	36	100,0%	0	0,0%	36	100,0%
RCMFE_PHASE3_SCALE_1_DBP	NO-OI	29	100,0%	0	0,0%	29	100,0%
	OI	36	100,0%	0	0,0%	36	100,0%
RCMFE_PHASE3_SCALE_2_DBP	NO-OI	29	100,0%	0	0,0%	29	100,0%
	OI	36	100,0%	0	0,0%	36	100,0%
RCMFE_PHASE3_SCALE_3_DBP	NO-OI	29	100,0%	0	0,0%	29	100,0%
	OI	36	100,0%	0	0,0%	36	100,0%
RCMFE_PHASE3_SCALE_4_DBP	NO-OI	29	100,0%	0	0,0%	29	100,0%
	OI	36	100,0%	0	0,0%	36	100,0%
RCMFE_PHASE3_SCALE_5_DBP	NO-OI	29	100,0%	0	0,0%	29	100,0%
	OI	36	100,0%	0	0,0%	36	100,0%
RCMFE_PHASE4_SCALE_1_DBP	NO-OI	29	100,0%	0	0,0%	29	100,0%
	OI	36	100,0%	0	0,0%	36	100,0%
RCMFE_PHASE4_SCALE_2_DBP	NO-OI	29	100,0%	0	0,0%	29	100,0%
	OI	36	100,0%	0	0,0%	36	100,0%
RCMFE_PHASE4_SCALE_3	NO-OI	29	100,0%	0	0,0%	29	100,0%
	OI	36	100,0%	0	0,0%	36	100,0%
RCMFE_PHASE4_SCALE_4_DBP	NO-OI	29	100,0%	0	0,0%	29	100,0%
	OI	36	100,0%	0	0,0%	36	100,0%
RCMFE_PHASE4_SCALE_5_DBP	NO-OI	29	100,0%	0	0,0%	29	100,0%
	OI	36	100,0%	0	0,0%	36	100,0%
RCMFE_PHASE5_SCALE_1_DBP	NO-OI	29	100,0%	0	0,0%	29	100,0%

Case Processing Summary

	PATHOLOGY	Cases					
		Valid		Missing		Total	
		N	Percent	N	Percent	N	Percent
RCMFE_PHASE5_SCALE_1_DBP	OI	36	100,0%	0	0,0%	36	100,0%
RCMFE_PHASE5_SCALE_2_DBP	NO-OI	29	100,0%	0	0,0%	29	100,0%
	OI	36	100,0%	0	0,0%	36	100,0%
RCMFE_PHASE5_SCALE_3_DBP	NO-OI	29	100,0%	0	0,0%	29	100,0%
	OI	36	100,0%	0	0,0%	36	100,0%
RCMFE_PHASE5_SCALE_4_DBP	NO-OI	29	100,0%	0	0,0%	29	100,0%
	OI	36	100,0%	0	0,0%	36	100,0%
RCMFE_PHASE5_SCALE_5_DBP	NO-OI	29	100,0%	0	0,0%	29	100,0%
	OI	36	100,0%	0	0,0%	36	100,0%

Tests of Normality

	PATHOLOGY	Kolmogorov-Smirnov[a]			Shapiro-Wilk		
		Statistic	df	Sig.	Statistic	df	Sig.
RCMFE_PHASE1_SCALE_1_DBP	NO-OI	,143	29	,136	,945	29	,137
	OI	,072	36	,200*	,987	36	,945
RCMFE_PHASE1_SCALE_2_DBP	NO-OI	,123	29	,200*	,967	29	,477
	OI	,115	36	,200*	,974	36	,557
RCMFE_PHASE1_SCALE_3_DBP	NO-OI	,144	29	,128	,949	29	,173
	OI	,132	36	,115	,979	36	,717
RCMFE_PHASE1_SCALE_4_DBP	NO-OI	,125	29	,200*	,955	29	,249
	OI	,130	36	,132	,948	36	,088
RCMFE_PHASE1_SCALE_5_DBP	NO-OI	,100	29	,200*	,971	29	,588
	OI	,130	36	,126	,951	36	,113
RCMFE_PHASE2_SCALE_1_DBP	NO-OI	,137	29	,171	,961	29	,339
	OI	,096	36	,200*	,972	36	,477
RCMFE_PHASE2_SCALE_2_DBP	NO-OI	,124	29	,200*	,937	29	,085
	OI	,098	36	,200*	,965	36	,306
RCMFE_PHASE2_SCALE_3_DBP	NO-OI	,113	29	,200*	,965	29	,431
	OI	,068	36	,200*	,983	36	,855
RCMFE_PHASE2_SCALE_4_DBP	NO-OI	,123	29	,200*	,965	29	,433
	OI	,087	36	,200*	,976	36	,595
RCMFE_PHASE2_SCALE_5_DBP	NO-OI	,132	29	,200*	,951	29	,191
	OI	,083	36	,200*	,981	36	,781
RCMFE_PHASE3_SCALE_1_DBP	NO-OI	,104	29	,200*	,971	29	,599
	OI	,156	36	,028	,952	36	,118
RCMFE_PHASE3_SCALE_2_DBP	NO-OI	,159	29	,060	,952	29	,207
	OI	,073	36	,200*	,986	36	,914
RCMFE_PHASE3_SCALE_3_DBP	NO-OI	,090	29	,200*	,973	29	,636
	OI	,087	36	,200*	,978	36	,681
RCMFE_PHASE3_SCALE_4_DBP	NO-OI	,076	29	,200*	,978	29	,794
	OI	,072	36	,200*	,976	36	,620
RCMFE_PHASE3_SCALE_5	NO-OI	,096	29	,200*	,957	29	,280

_DBP	OI	,088	36	,200*	,984	36	,865
RCMFE_PHASE4_SCALE_1	NO-OI	,155	29	,072	,913	29	,021
_DBP	OI	,135	36	,097	,961	36	,230
RCMFE_PHASE4_SCALE_2	NO-OI	,136	29	,183	,933	29	,066
_DBP	OI	,071	36	,200*	,986	36	,921
RCMFE_PHASE4_SCALE_3	NO-OI	,108	29	,200*	,958	29	,299
	OI	,073	36	,200*	,981	36	,766
RCMFE_PHASE4_SCALE_4	NO-OI	,077	29	,200*	,978	29	,787
_DBP	OI	,103	36	,200*	,976	36	,608
RCMFE_PHASE4_SCALE_5	NO-OI	,134	29	,197	,949	29	,173
_DBP	OI	,112	36	,200*	,978	36	,694
RCMFE_PHASE5_SCALE_1	NO-OI	,114	29	,200*	,973	29	,634
_DBP	OI	,085	36	,200*	,969	36	,406

Tests of Normality

	PATHOLOGY	Kolmogorov-Smirnov[a]			Shapiro-Wilk		
		Statistic	df	Sig.	Statistic	df	Sig.
RCMFE_PHASE5_SCALE_2	NO-OI	,144	29	,126	,922	29	,035
_DBP	OI	,122	36	,197*	,971	36	,467
RCMFE_PHASE5_SCALE_3	NO-OI	,150	29	,093*	,933	29	,064
_DBP	OI	,107	36	,200*	,968	36	,378
RCMFE_PHASE5_SCALE_4	NO-OI	,129	29	,200	,955	29	,250
_DBP	OI	,096	36	,200	,988	36	,956
RCMFE_PHASE5_SCALE_5	NO-OI	,121	29	,200*	,970	29	,550
_DBP	OI	,097	36	,200	,968	36	,377

*. This is a lower bound of the true significance.

a. Lilliefors Significance Correction

RCMFE_PHASE1_SCALE_1_DBP

Normal Q-Q Plots

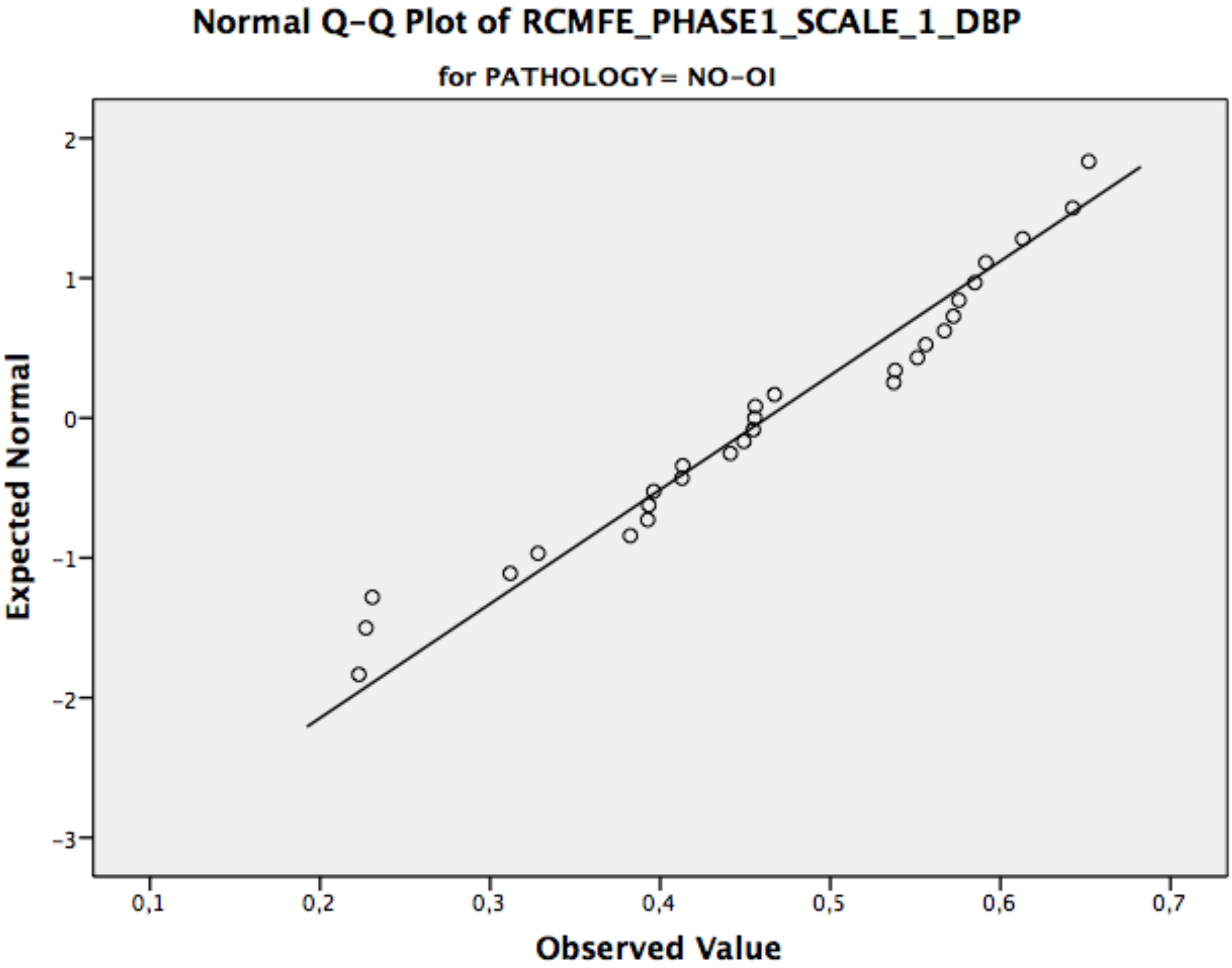

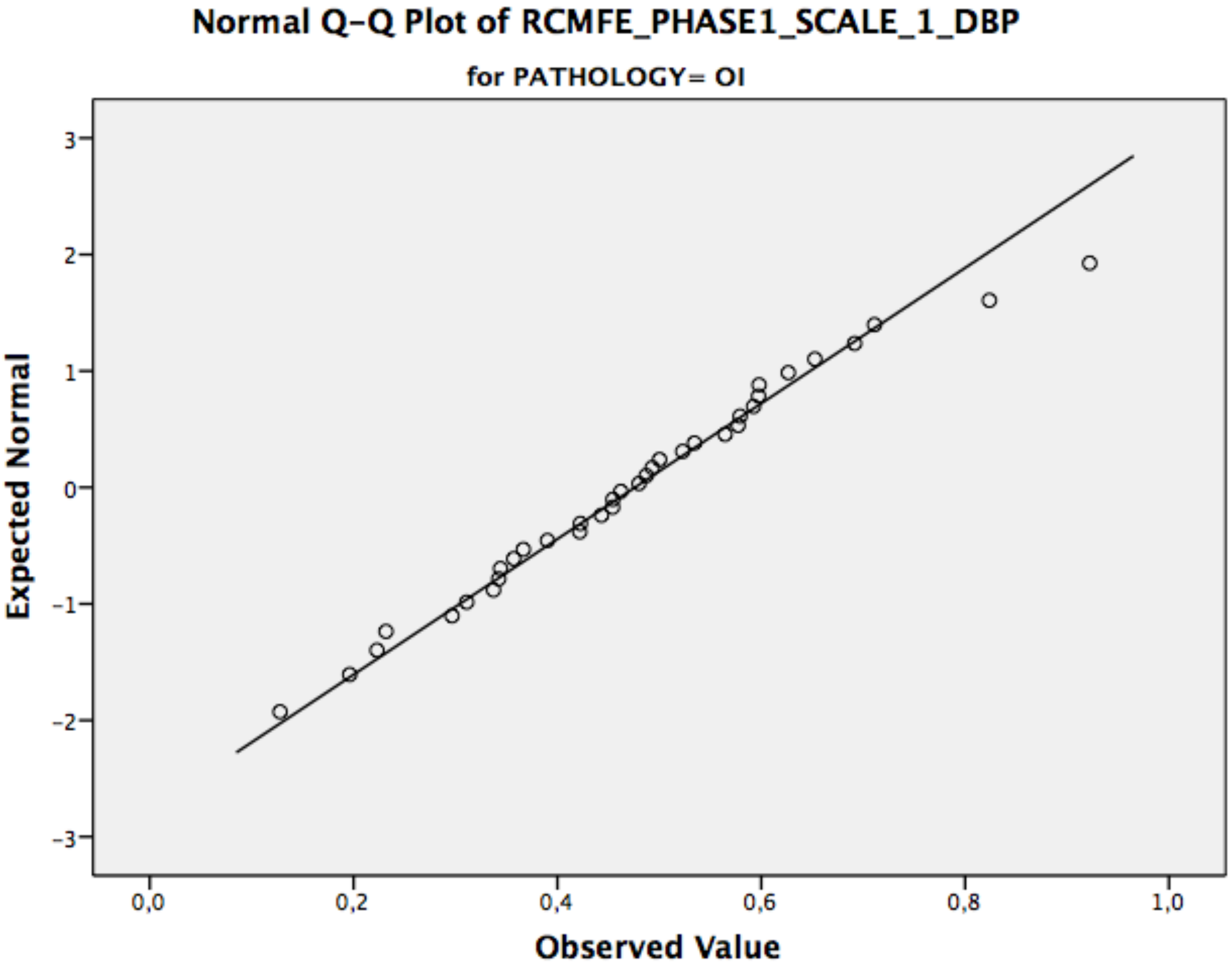
Normal Q-Q Plot of RCMFE_PHASE1_SCALE_1_DBP
for PATHOLOGY= OI
Expected Normal
3
2
1
0
-1
-2
-3
0,0
0,2
0,4
0,6
0,8
1,0
Observed Value

RCMFE_PHASE1_SCALE_2_DBP

Normal Q-Q Plots

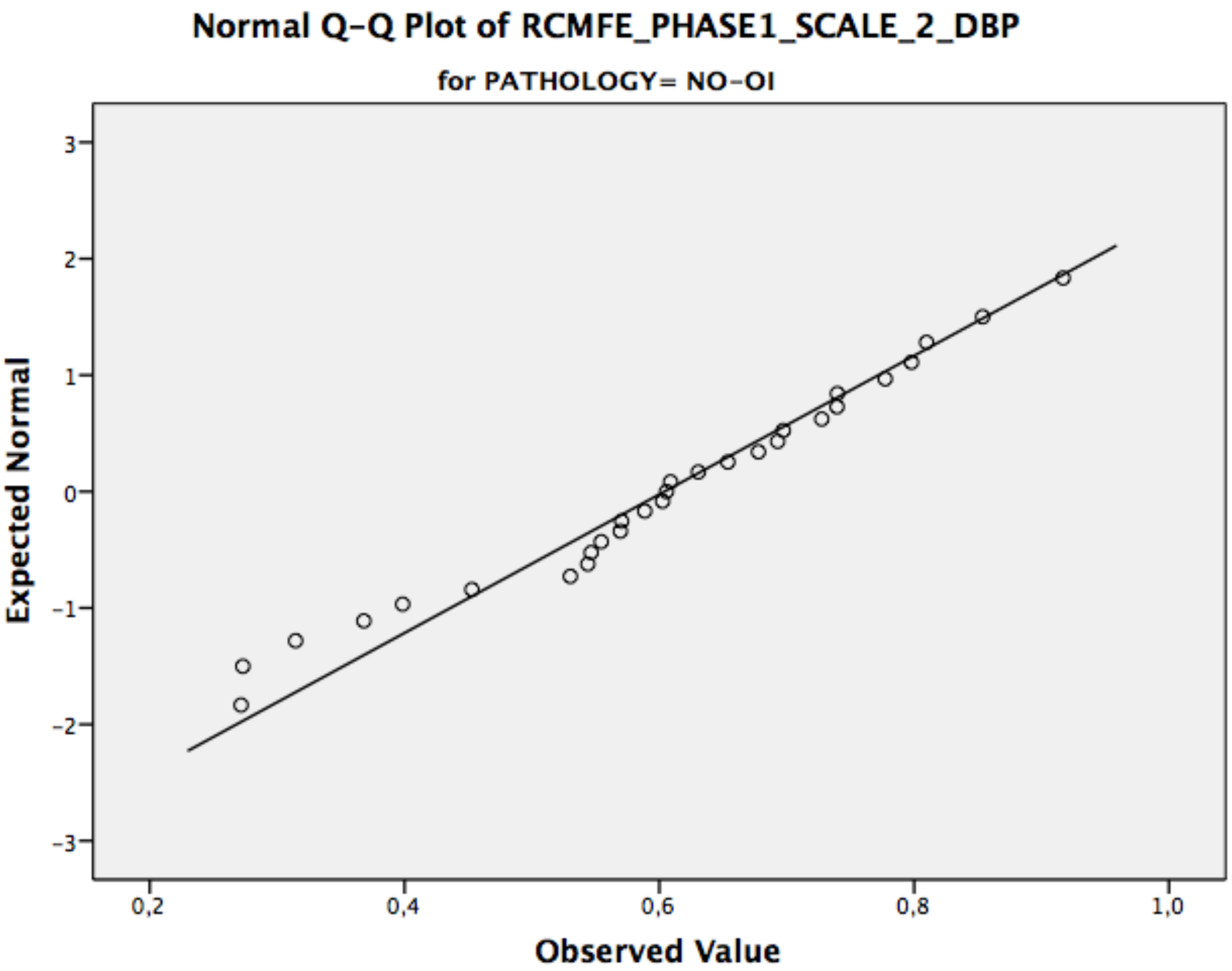

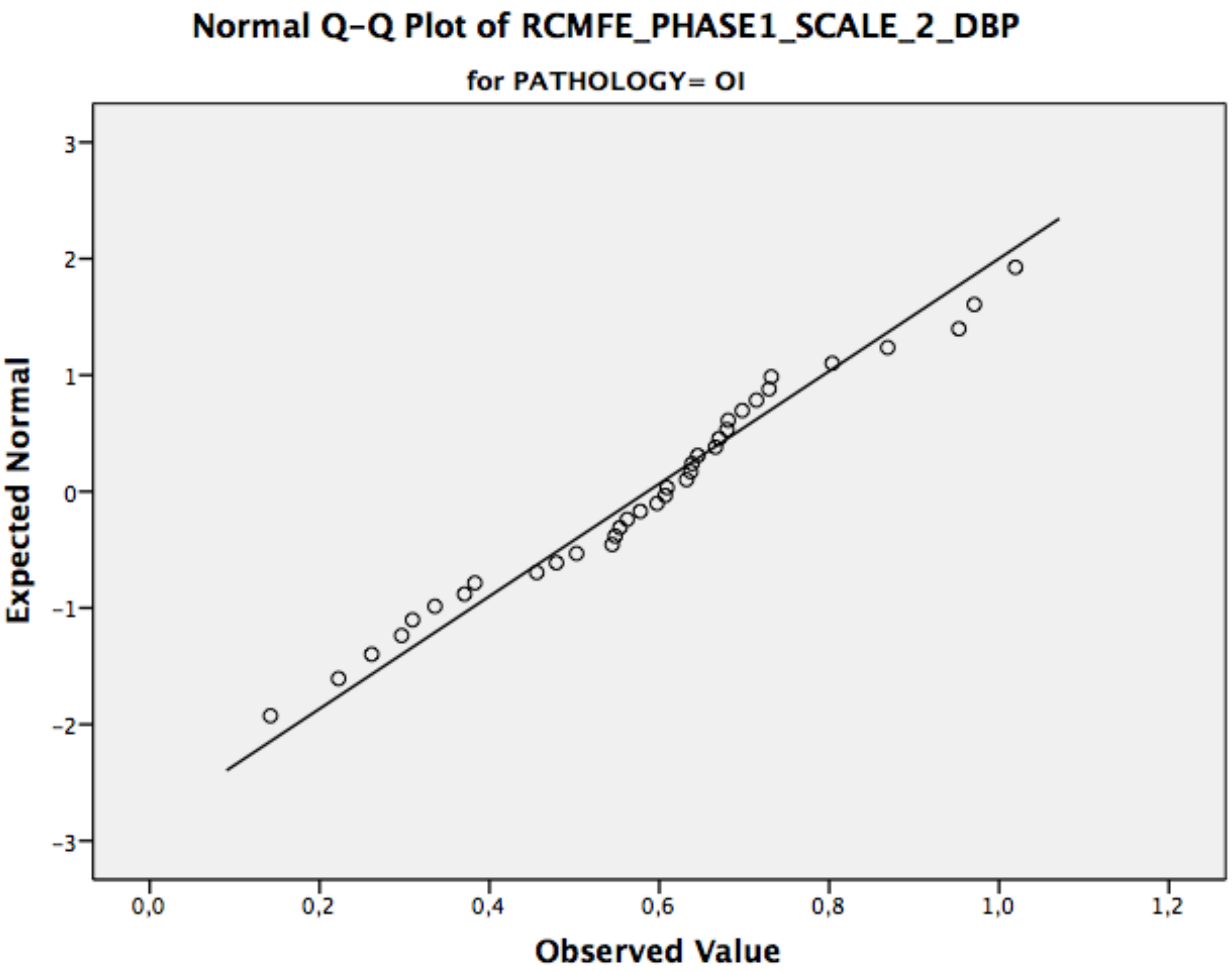

Normal Q-Q Plot of RCMFE_PHASE1_SCALE_2_DBP
for PATHOLOGY= OI
Expected Normal
Observed Value
3
2
1
0
-1
-2
-3
0,0
0,2
0,4
0,6
0,8
1,0
1,2

RCMFE_PHASE1_SCALE_3_DBP

Normal Q-Q Plots

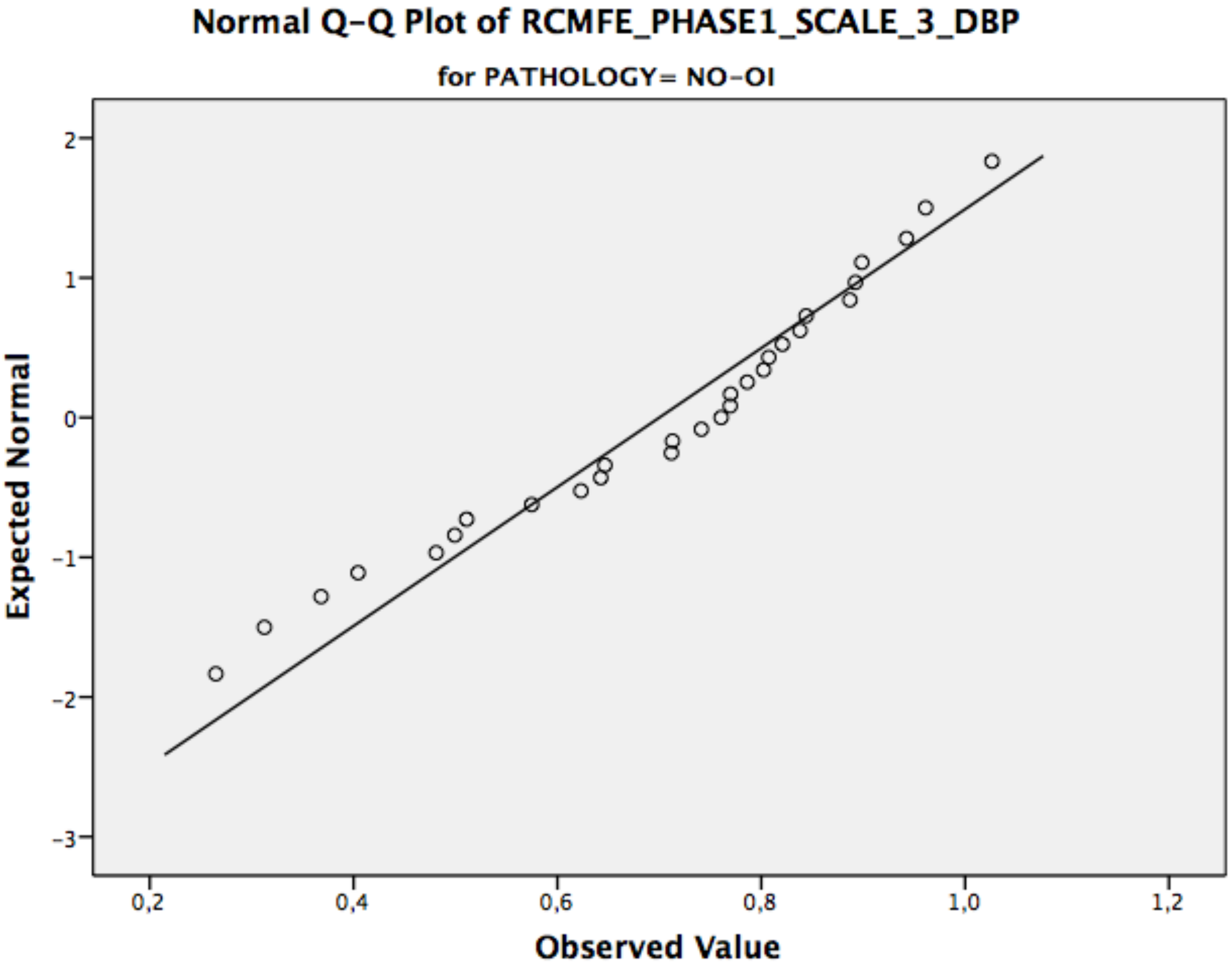

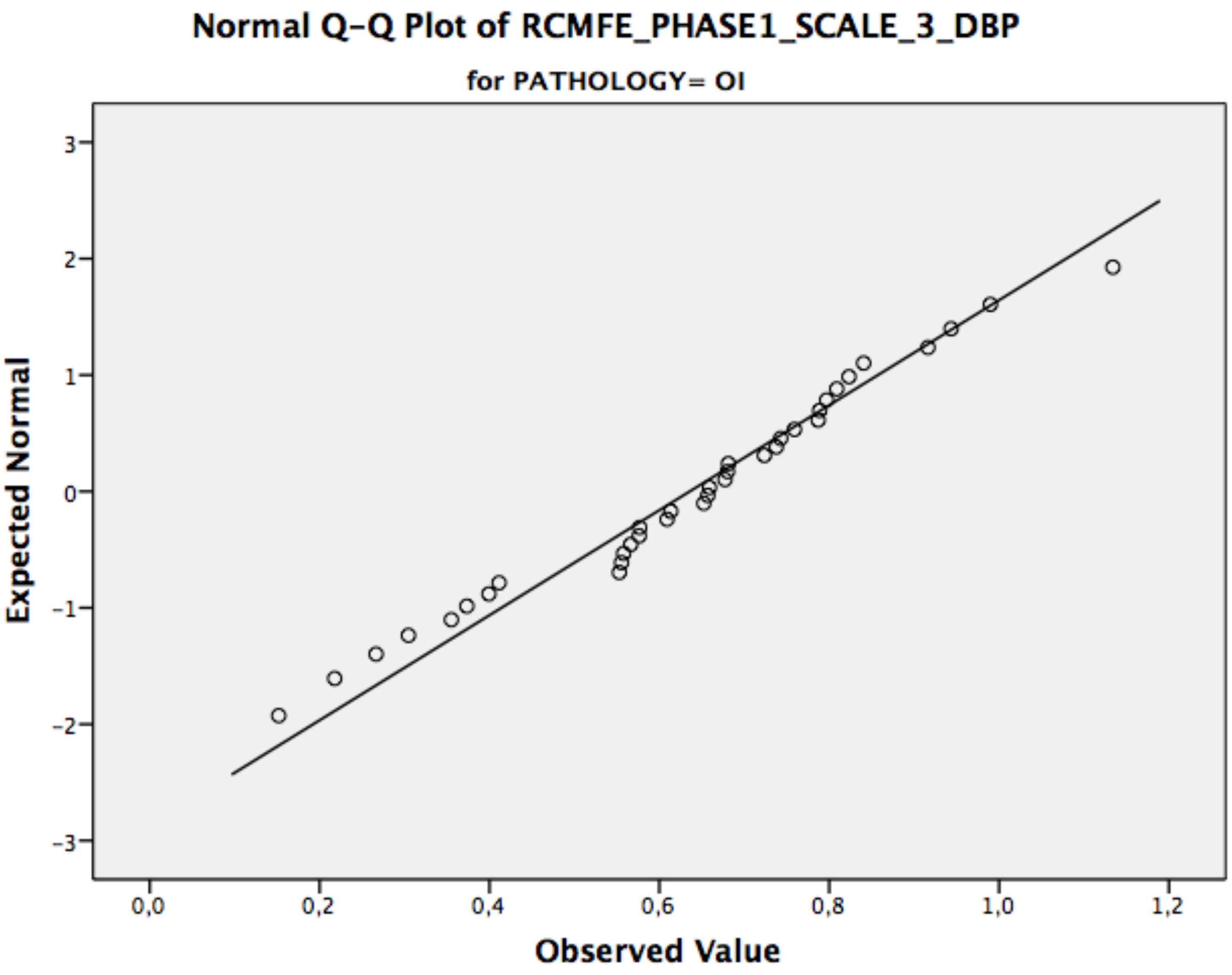

Normal Q-Q Plot of RCMFE_PHASE1_SCALE_3_DBP
for PATHOLOGY= OI
Expected Normal
Observed Value
3
2
1
0
-1
-2
-3
0,0
0,2
0,4
0,6
0,8
1,0
1,2

RCMFE_PHASE1_SCALE_4_DBP

Normal Q-Q Plots

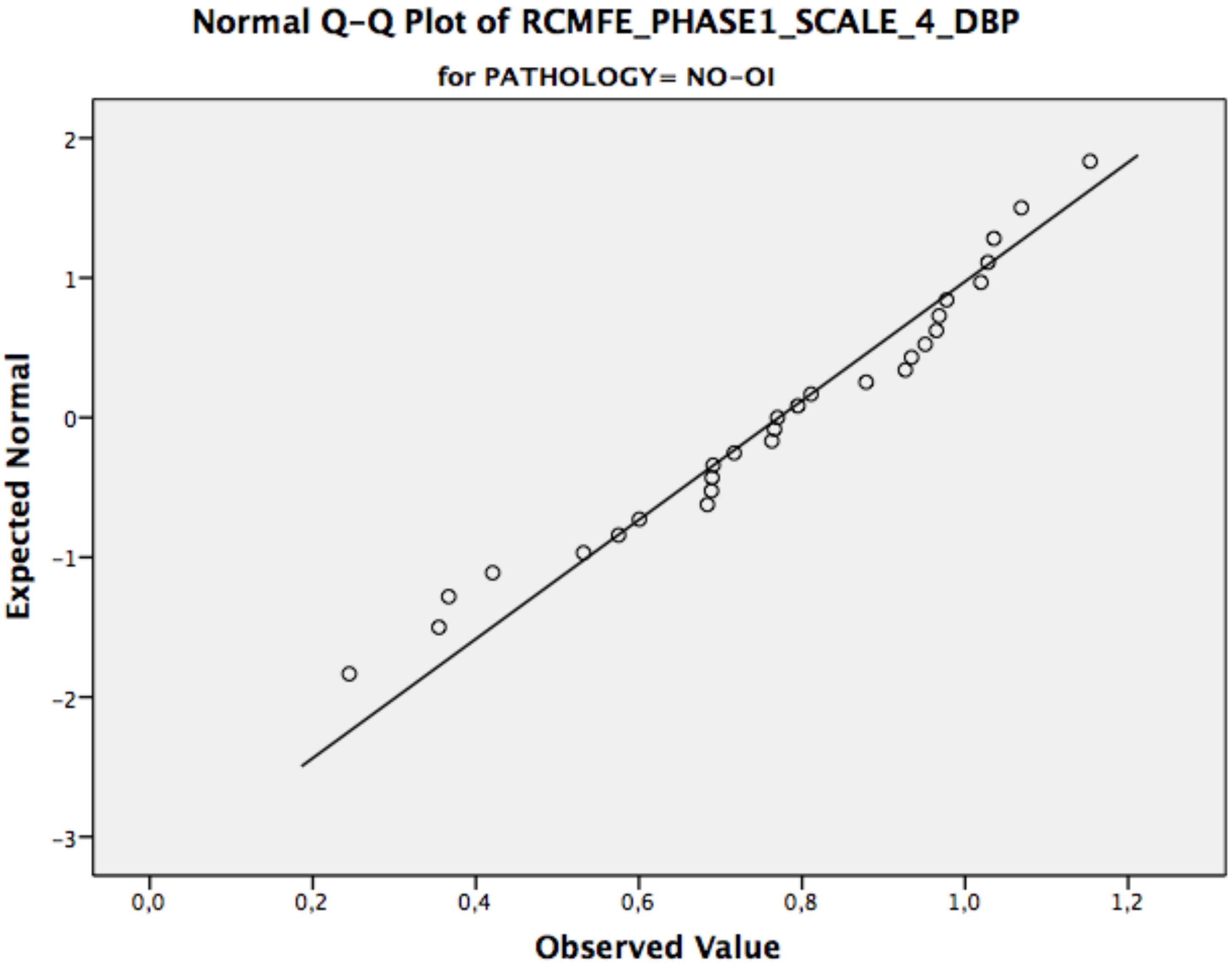

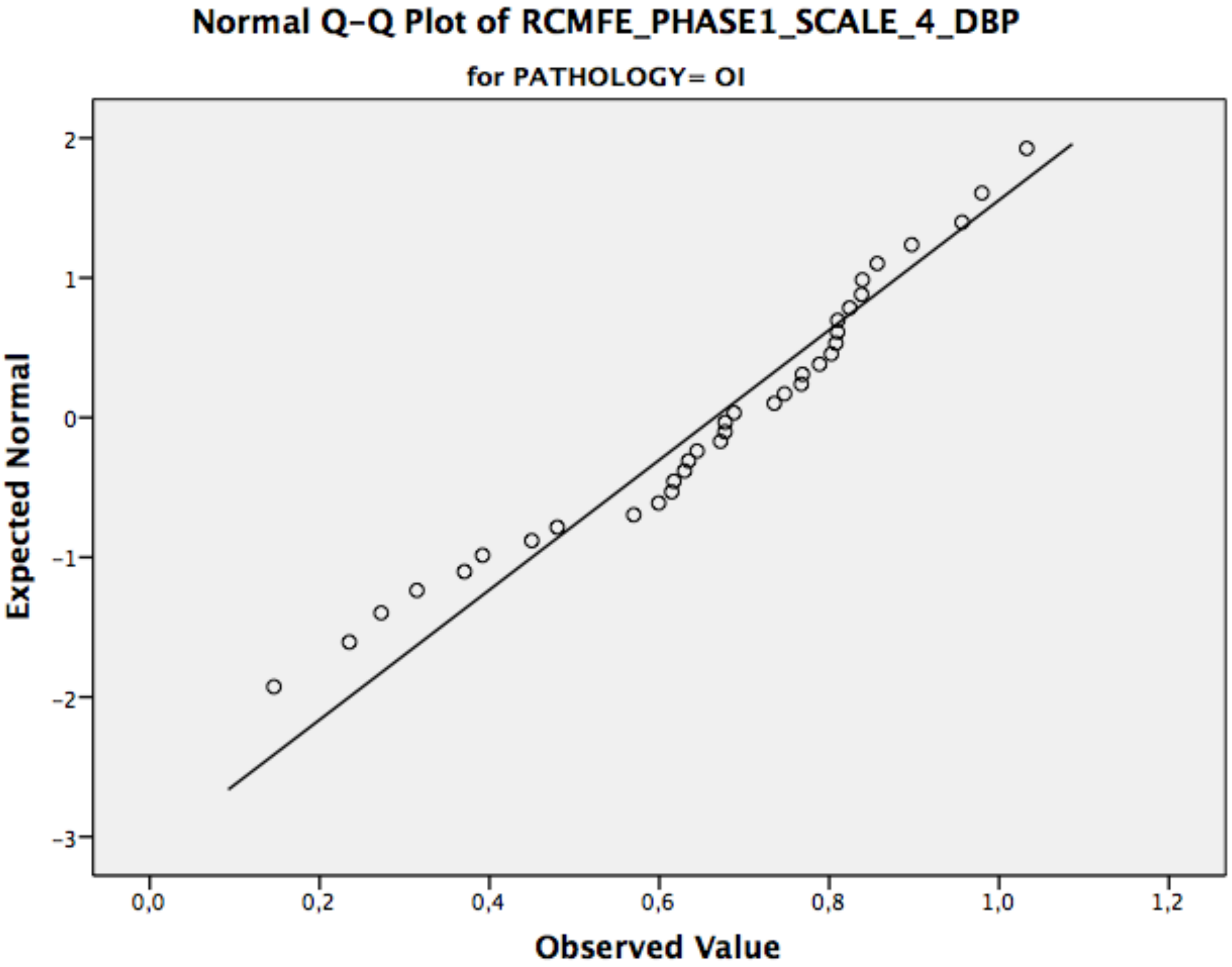
Normal Q-Q Plot of RCMFE_PHASE1_SCALE_4_DBP
for PATHOLOGY= OI
Expected Normal
2
1
0
-1
-2
-3
0,0
0,2
0,4
0,6
0,8
1,0
1,2
Observed Value

RCMFE_PHASE1_SCALE_5_DBP

Normal Q-Q Plots

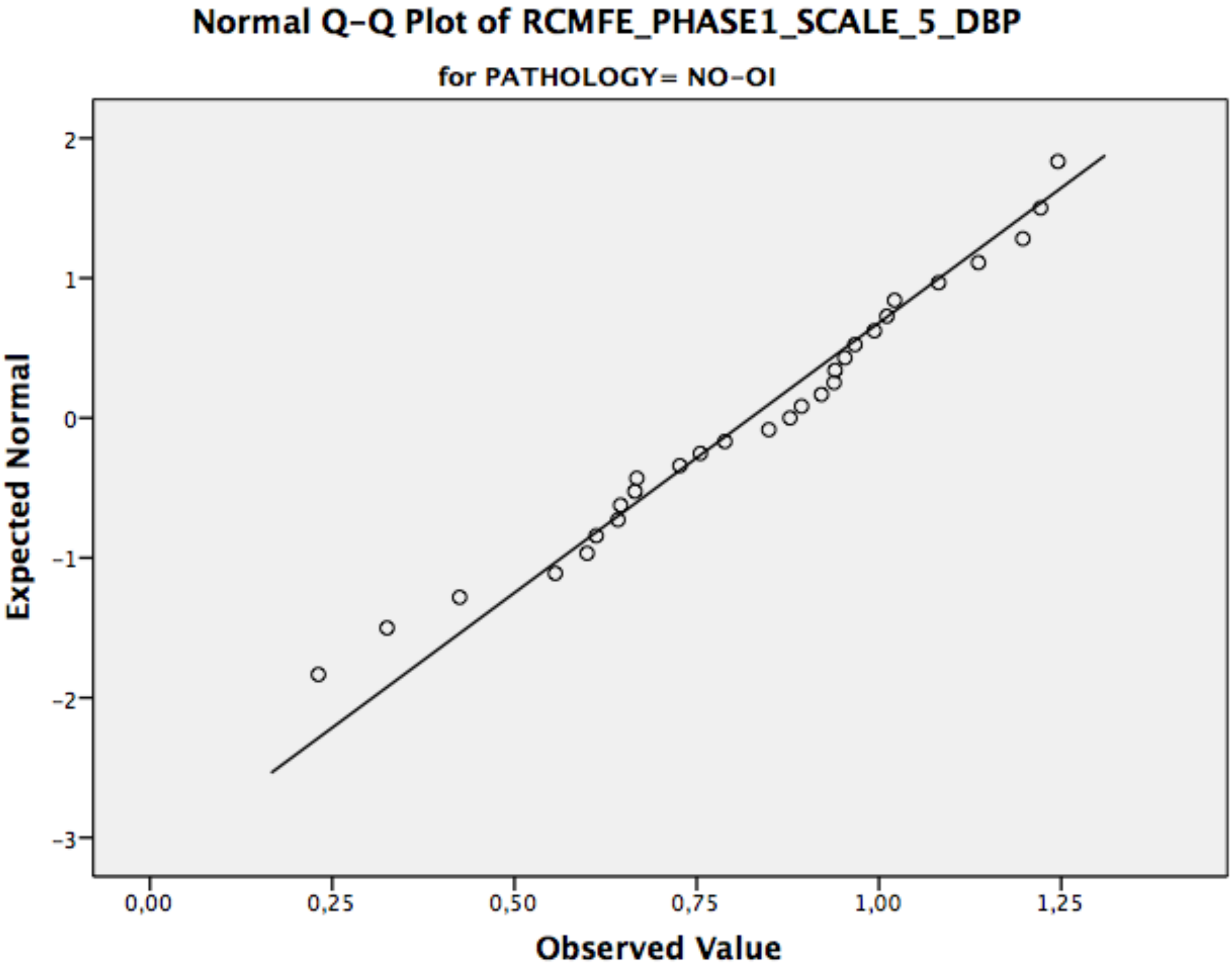

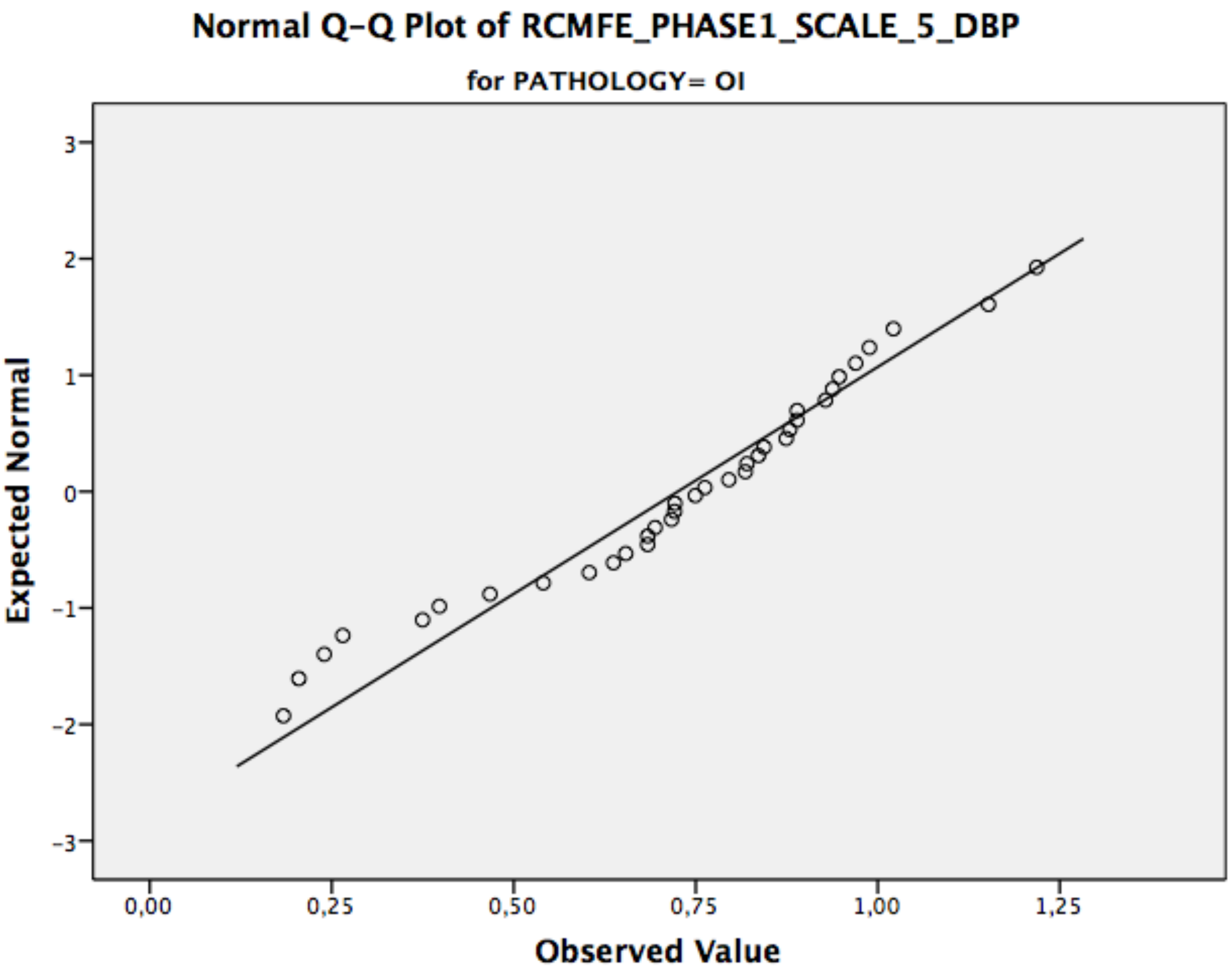
Normal Q-Q Plot of RCMFE_PHASE1_SCALE_5_DBP
for PATHOLOGY= OI
Expected Normal
3
2
1
0
-1
-2
-3
0,00
0,25
0,50
0,75
1,00
1,25
Observed Value

RCMFE_PHASE2_SCALE_1_DBP

Normal Q-Q Plots

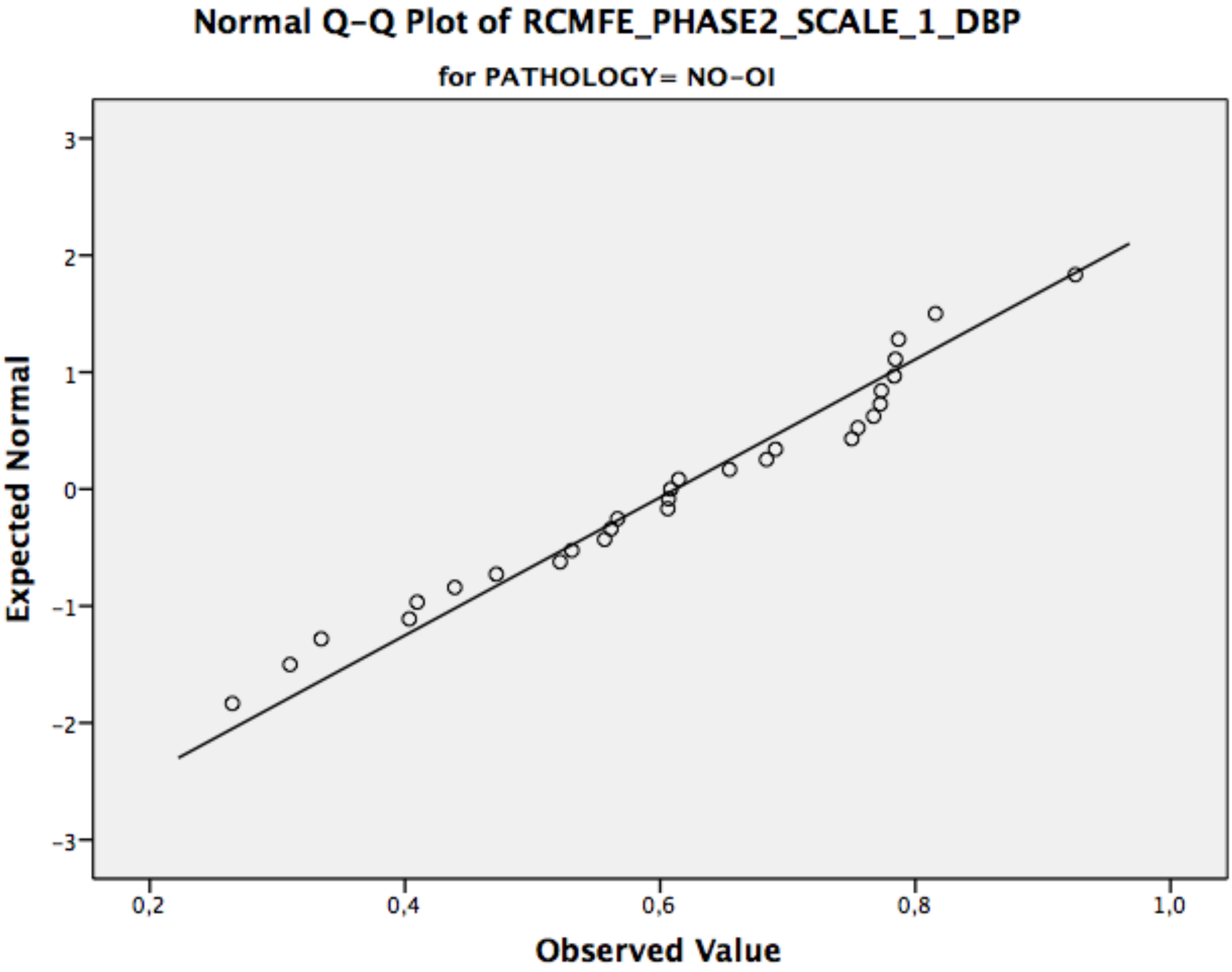

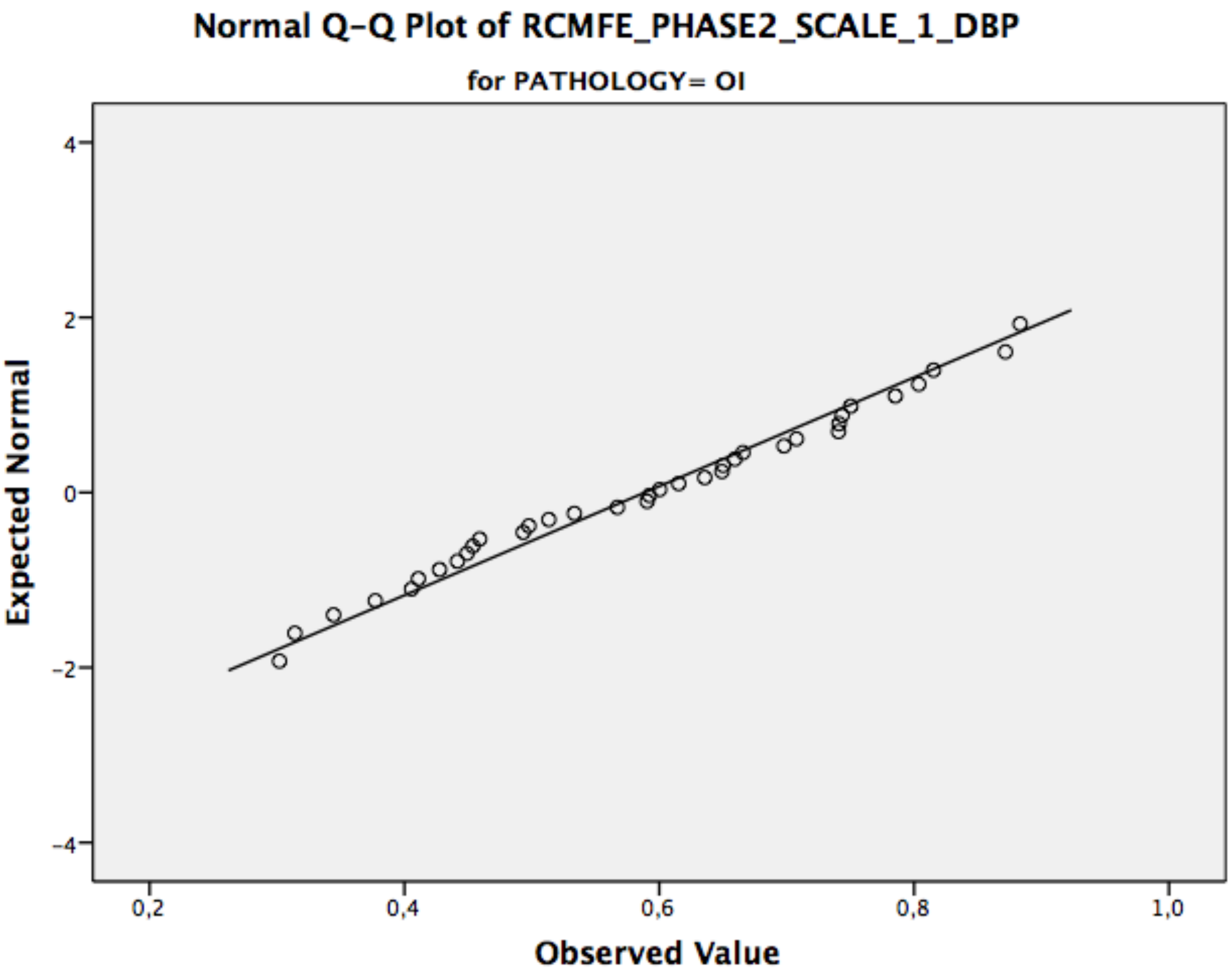
Normal Q-Q Plot of RCMFE_PHASE2_SCALE_1_DBP
for PATHOLOGY= OI
Expected Normal
Observed Value
4
2
0
-2
-4
0,2
0,4
0,6
0,8
1,0

RCMFE_PHASE2_SCALE_2_DBP

Normal Q-Q Plots

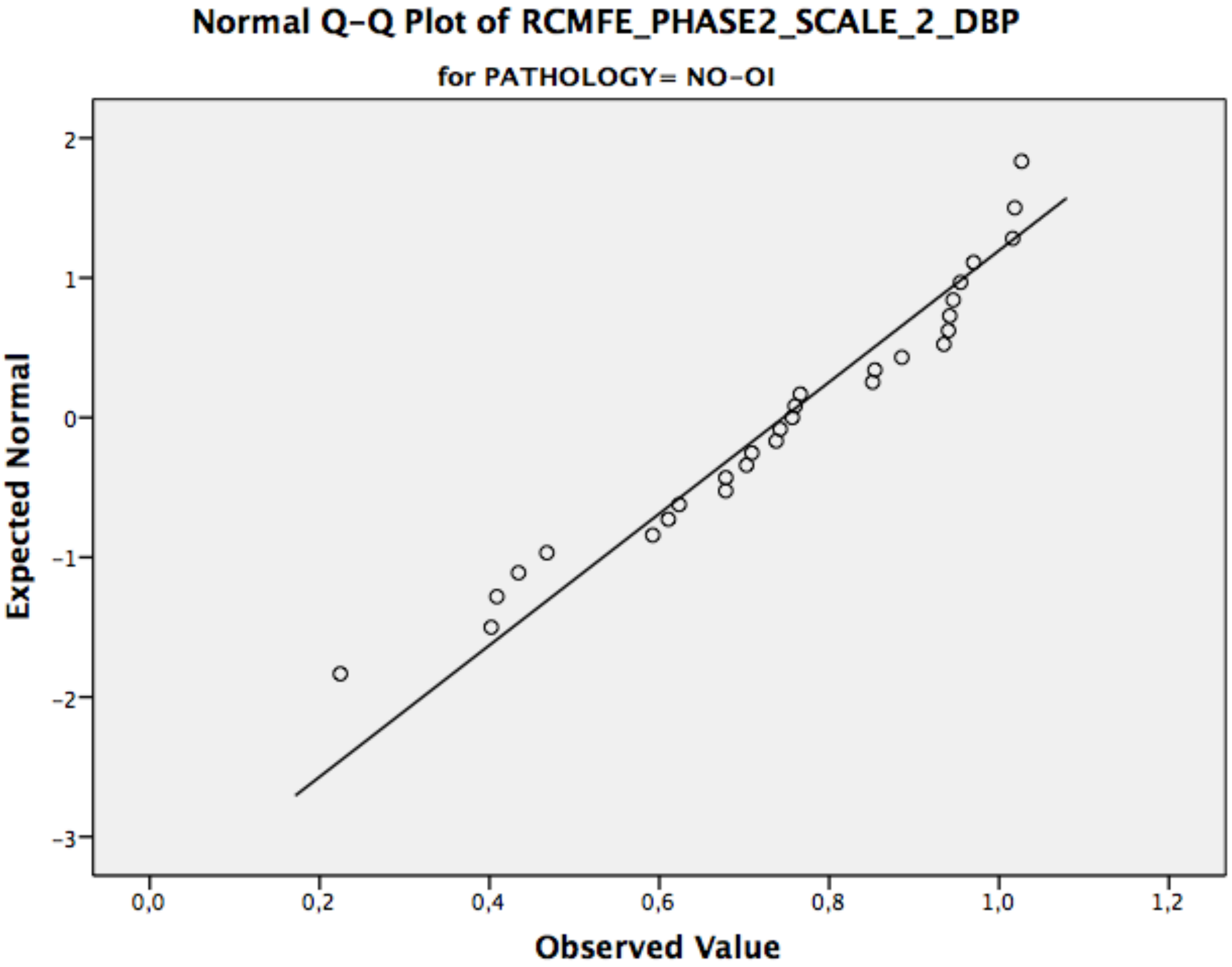

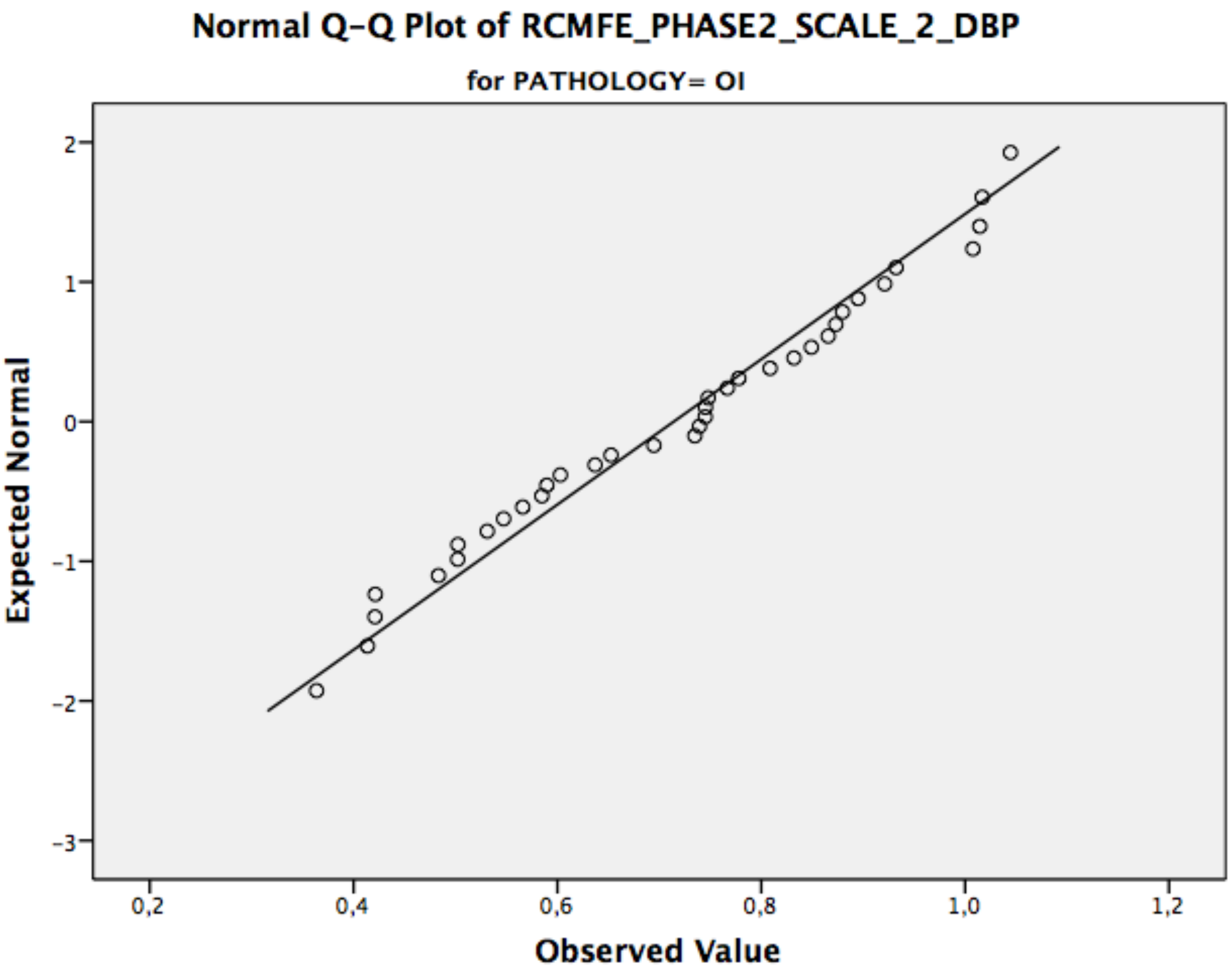
Normal Q-Q Plot of RCMFE_PHASE2_SCALE_2_DBP
for PATHOLOGY= OI
Expected Normal
Observed Value
2
1
0
-1
-2
-3
0,2
0,4
0,6
0,8
1,0
1,2

RCMFE_PHASE2_SCALE_3_DBP

Normal Q-Q Plots

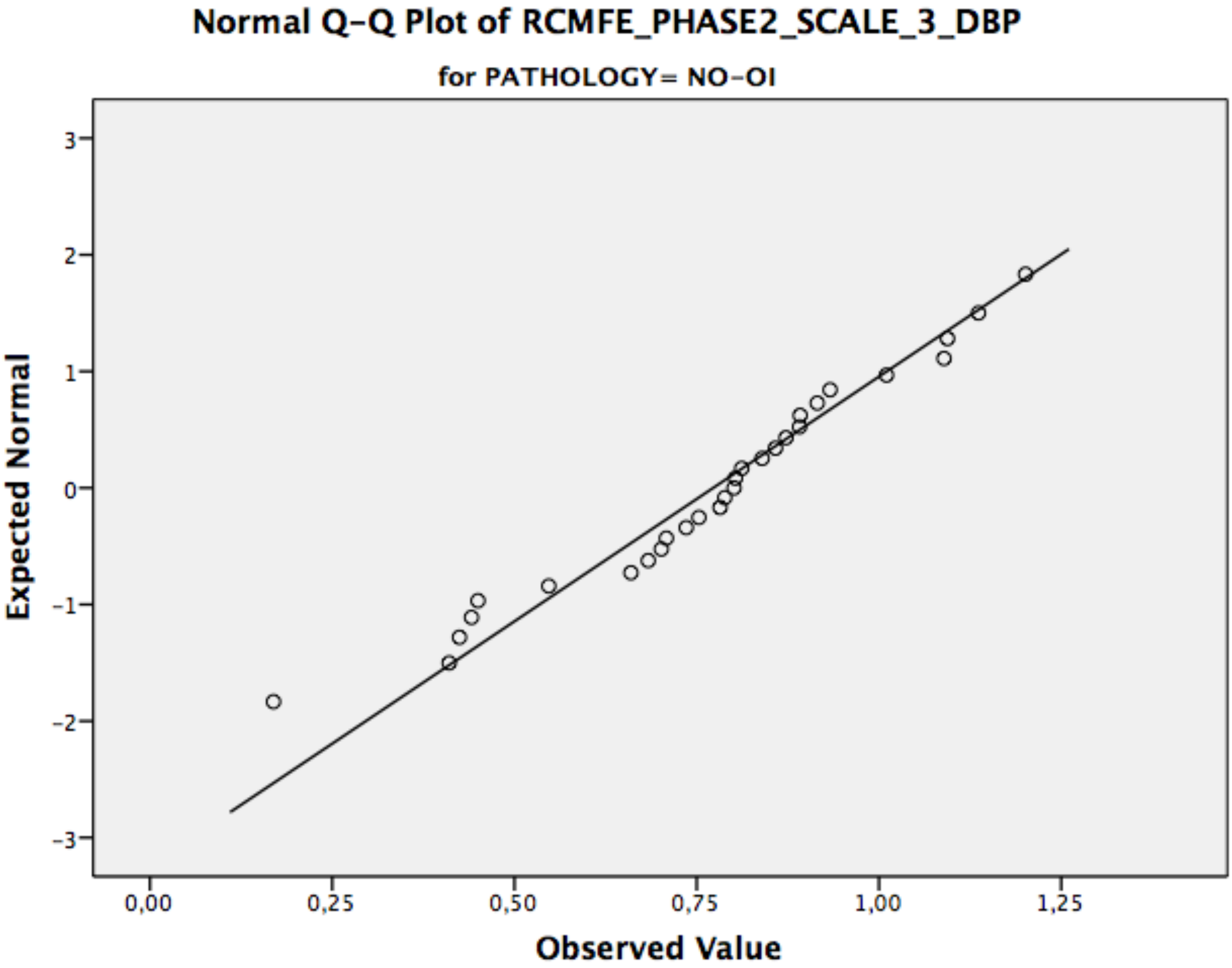

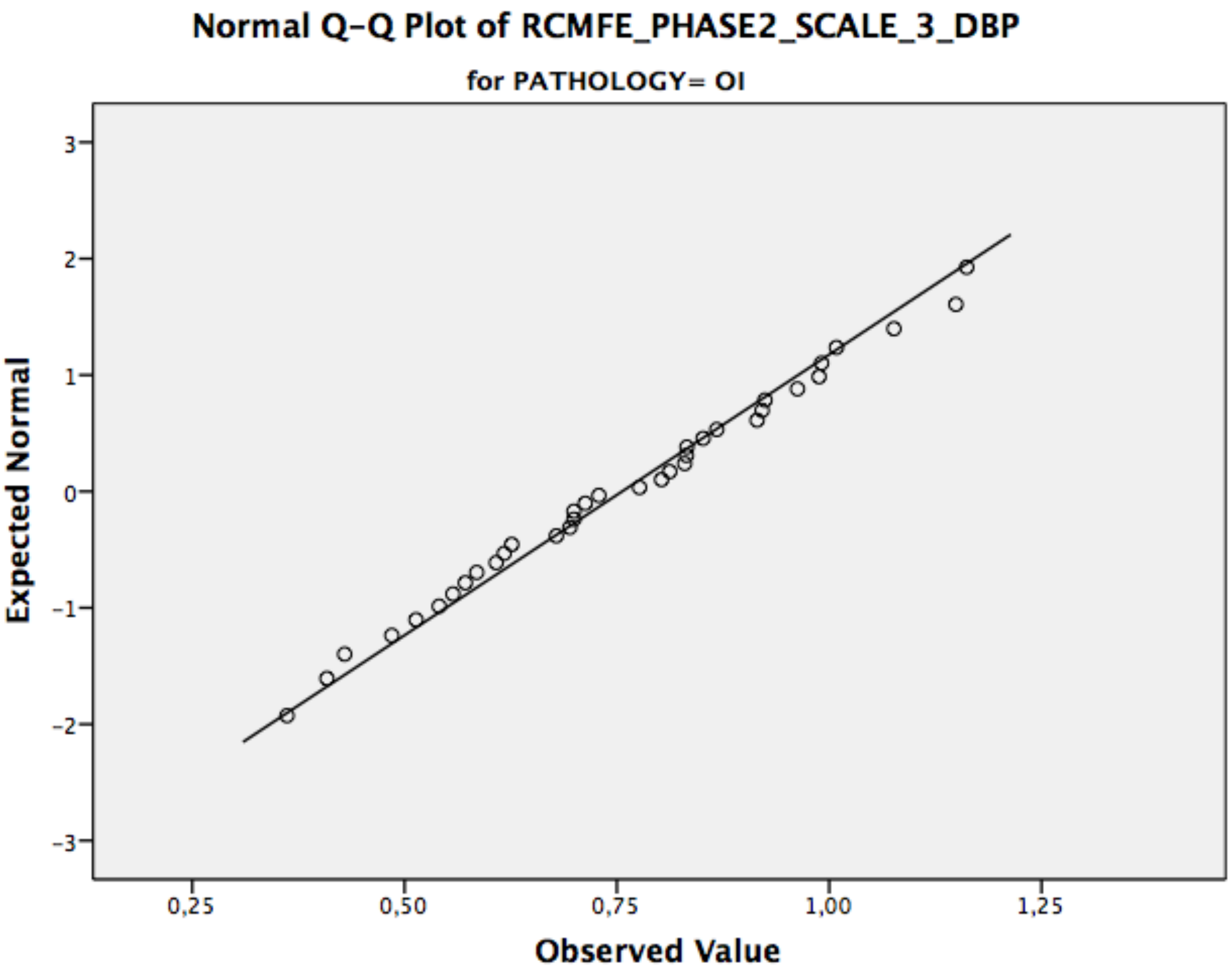
Normal Q-Q Plot of RCMFE_PHASE2_SCALE_3_DBP
for PATHOLOGY= OI
Expected Normal
3
2
1
0
-1
-2
-3
0,25
0,50
0,75
1,00
1,25
Observed Value

RCMFE_PHASE2_SCALE_4_DBP

Normal Q-Q Plots

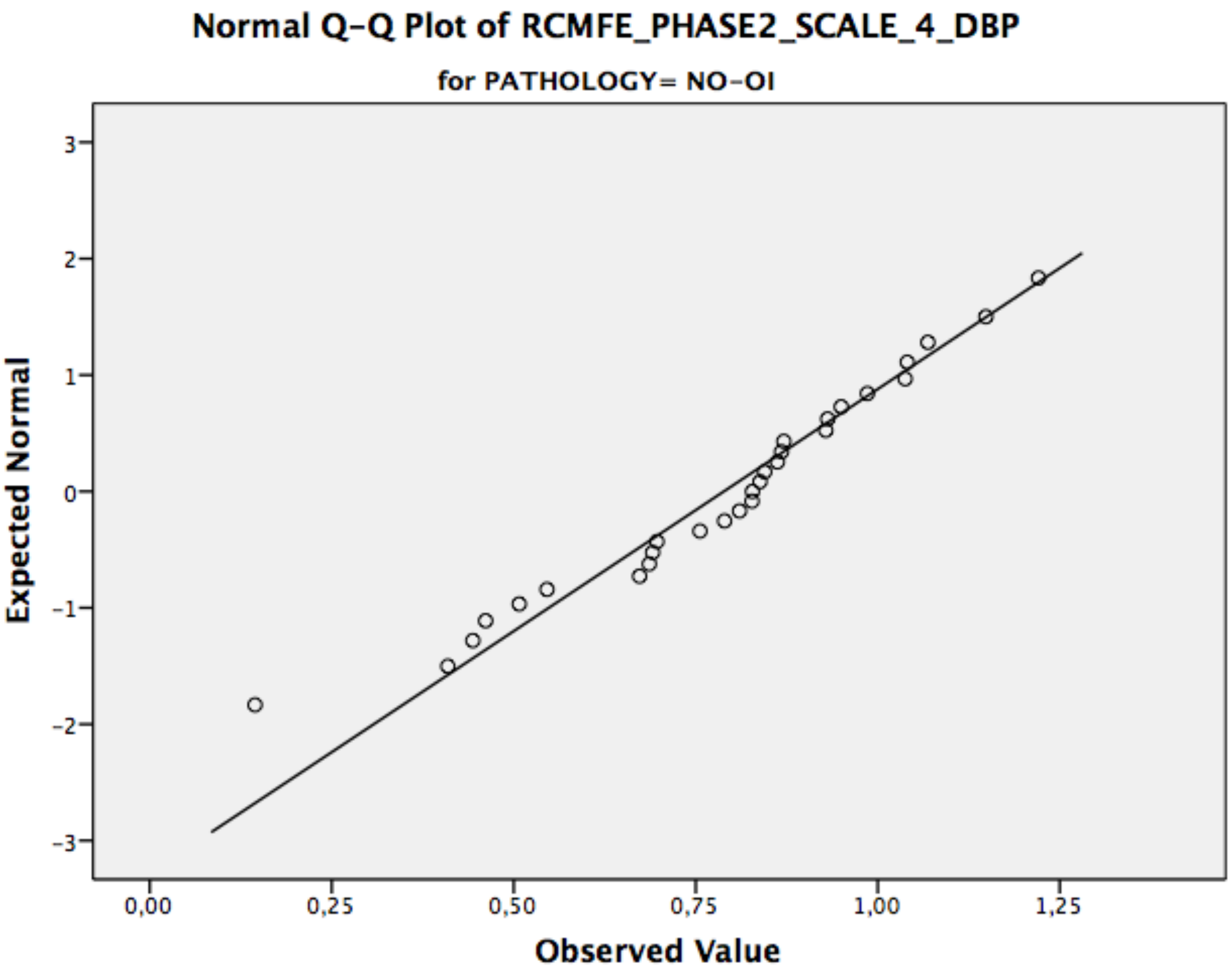

Normal Q-Q Plot of RCMFE_PHASE2_SCALE_4_DBP

for PATHOLOGY= OI

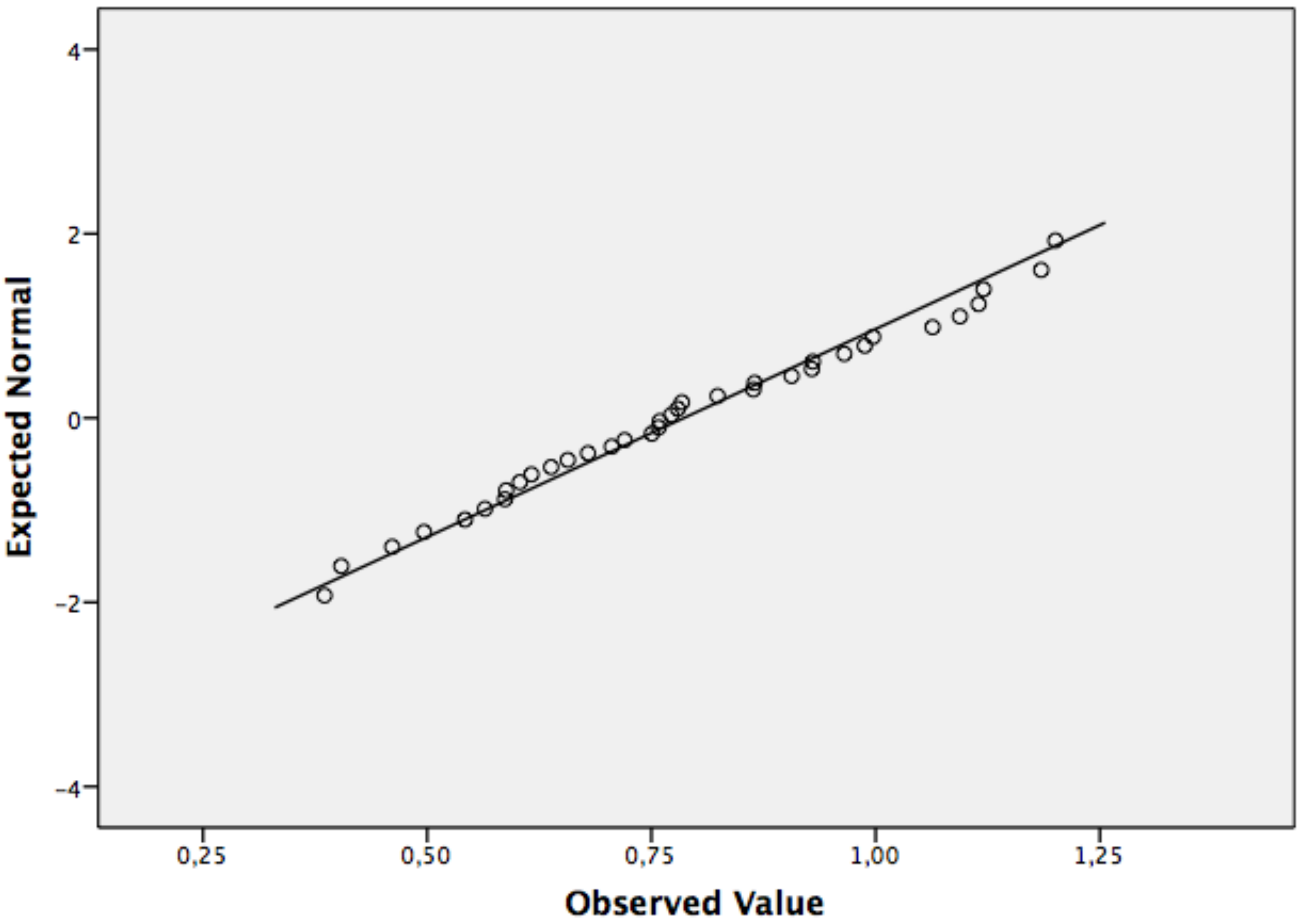

RCMFE_PHASE2_SCALE_5_DBP

Normal Q-Q Plots

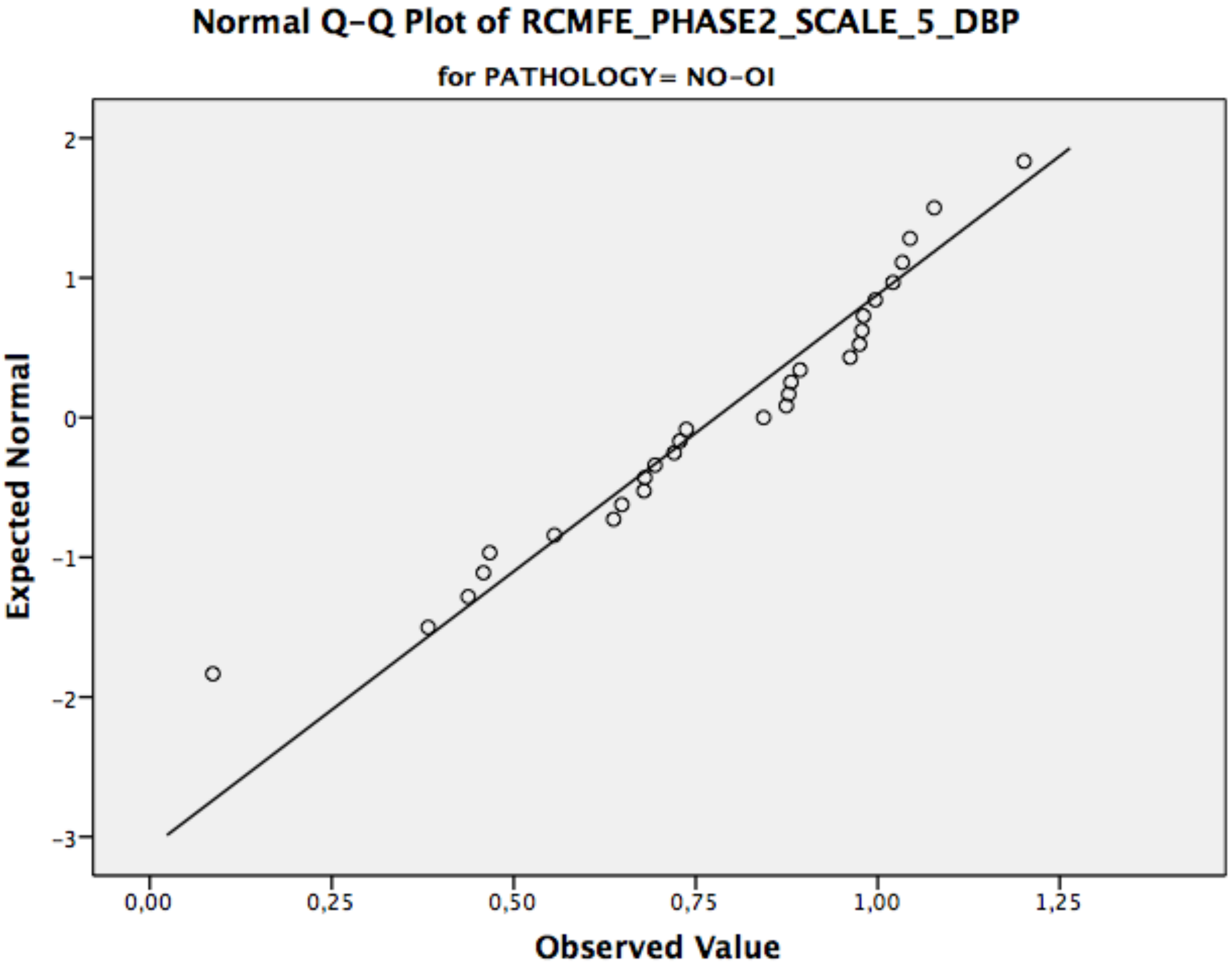

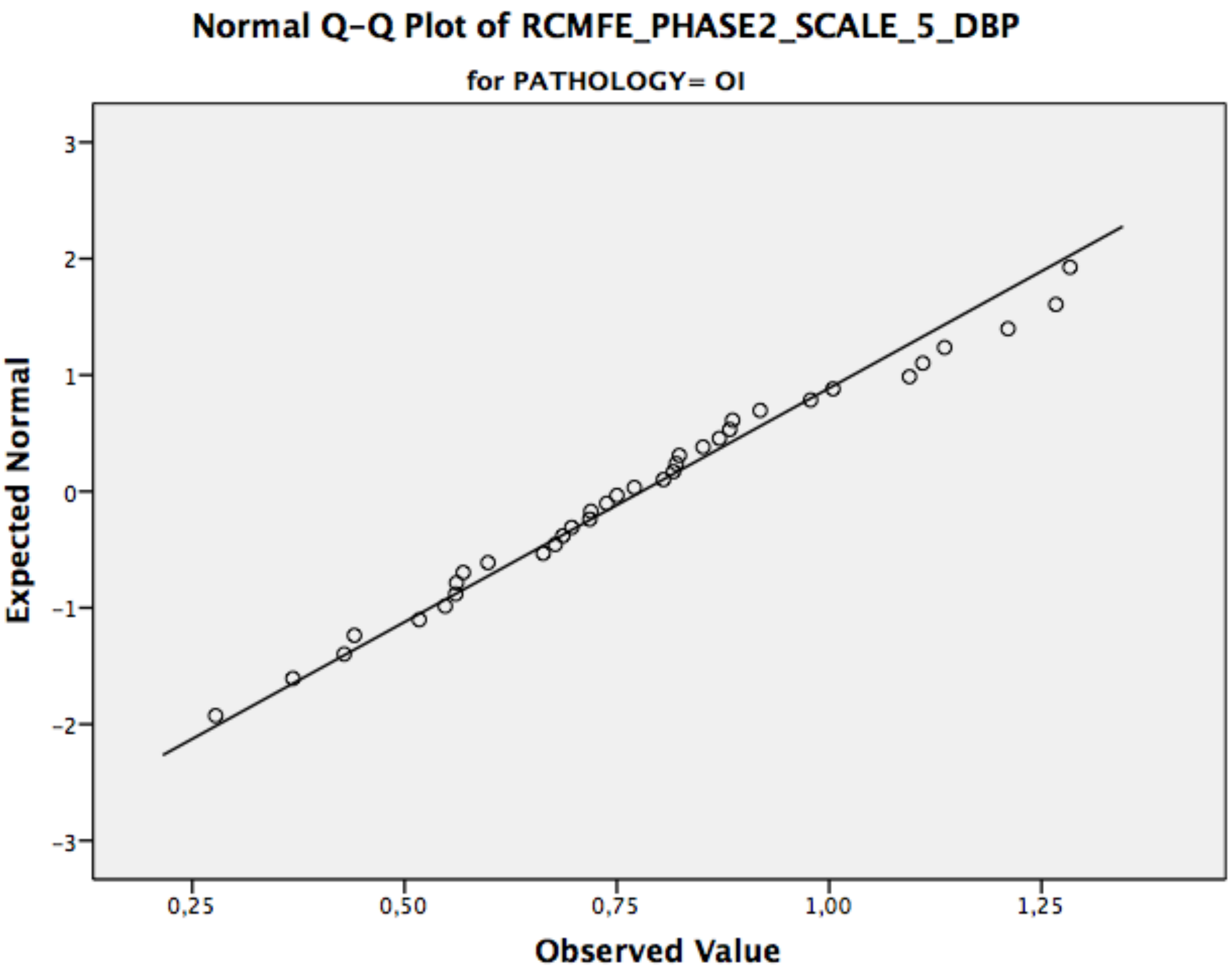
Normal Q-Q Plot of RCMFE_PHASE2_SCALE_5_DBP
for PATHOLOGY= OI
Expected Normal
3
2
1
0
-1
-2
-3
0,25
0,50
0,75
1,00
1,25
Observed Value

RCMFE_PHASE3_SCALE_1_DBP

Normal Q-Q Plots

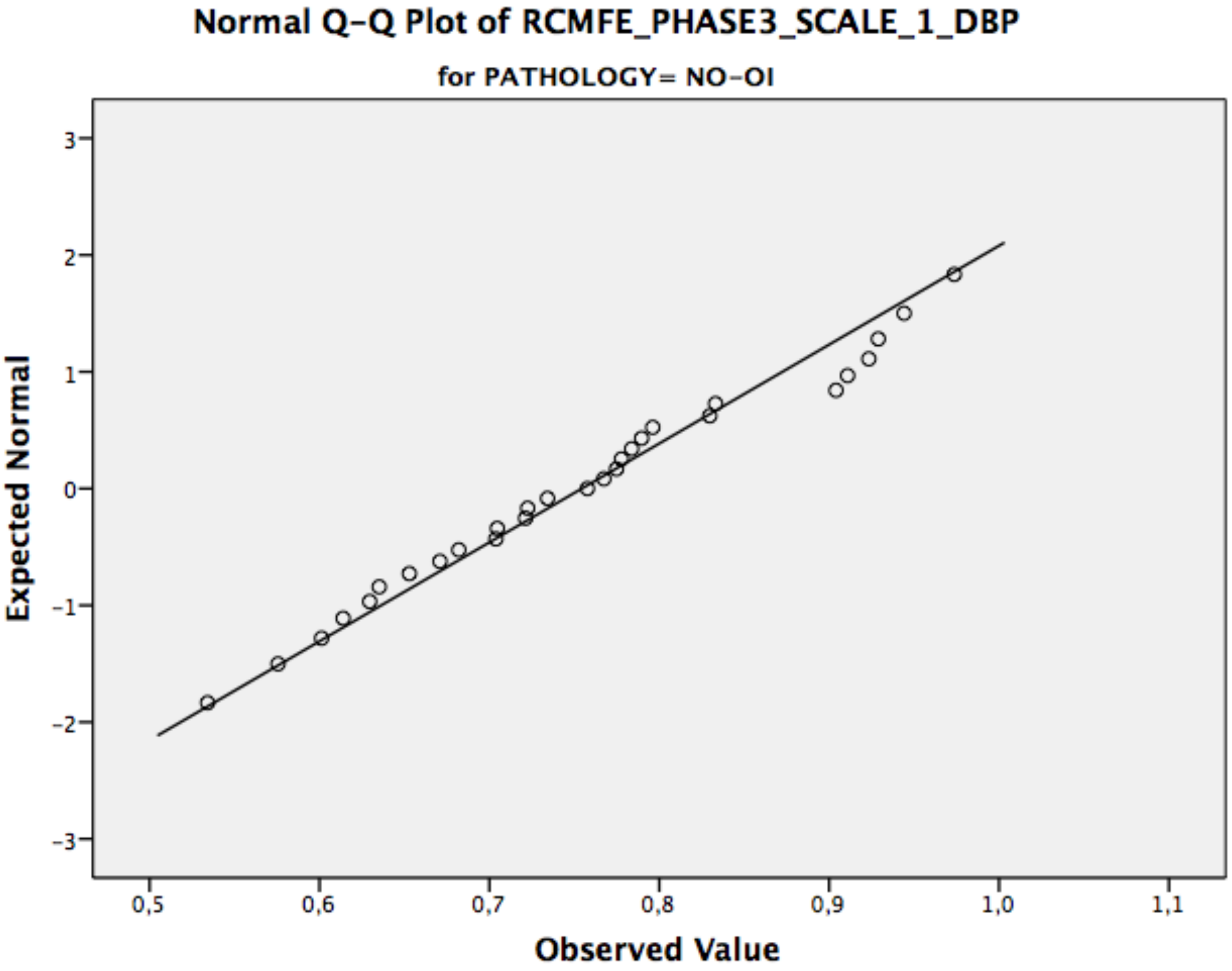

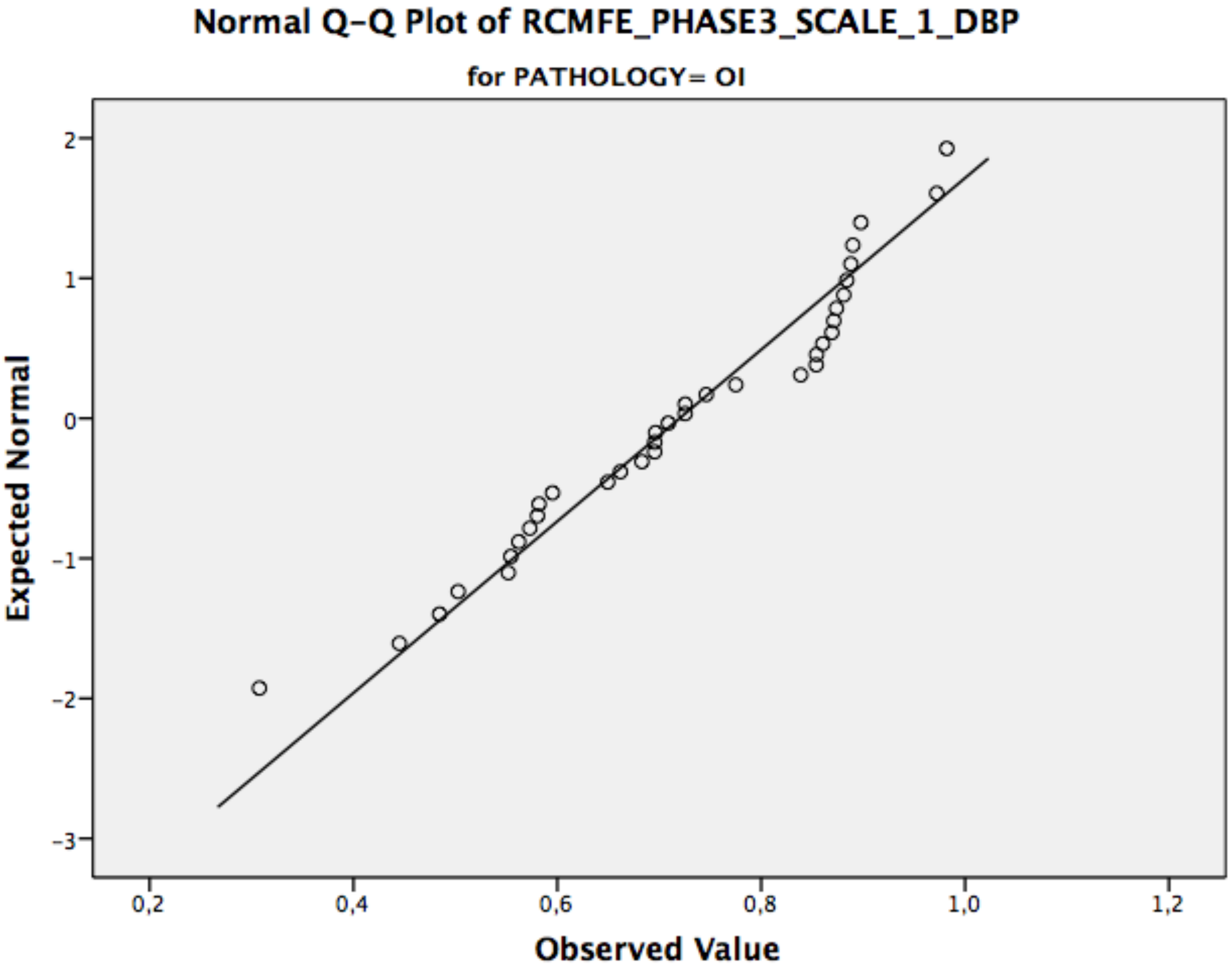
Normal Q-Q Plot of RCMFE_PHASE3_SCALE_1_DBP
for PATHOLOGY= OI
Expected Normal
Observed Value
2
1
0
-1
-2
-3
0,2
0,4
0,6
0,8
1,0
1,2

RCMFE_PHASE3_SCALE_2_DBP

Normal Q-Q Plots

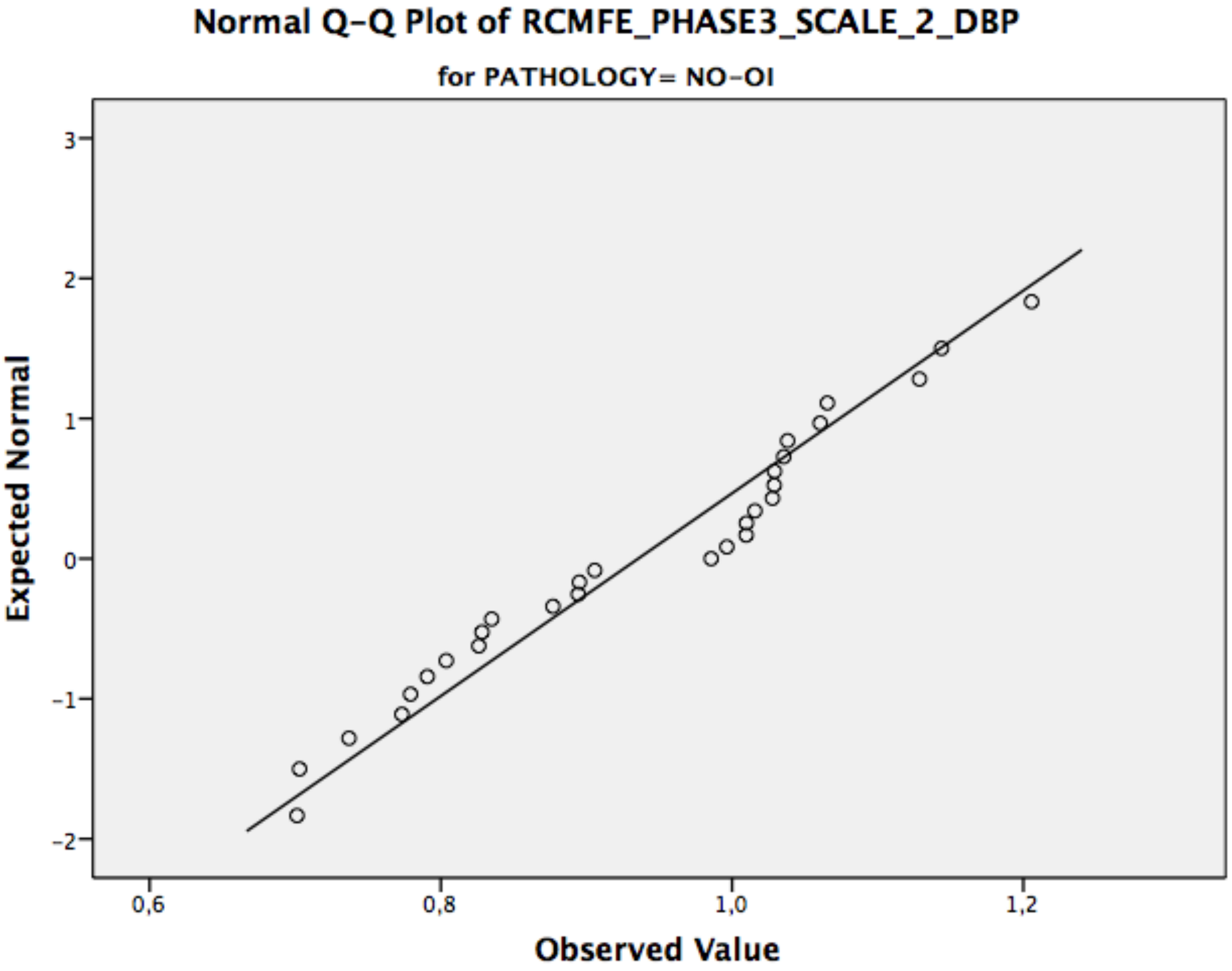

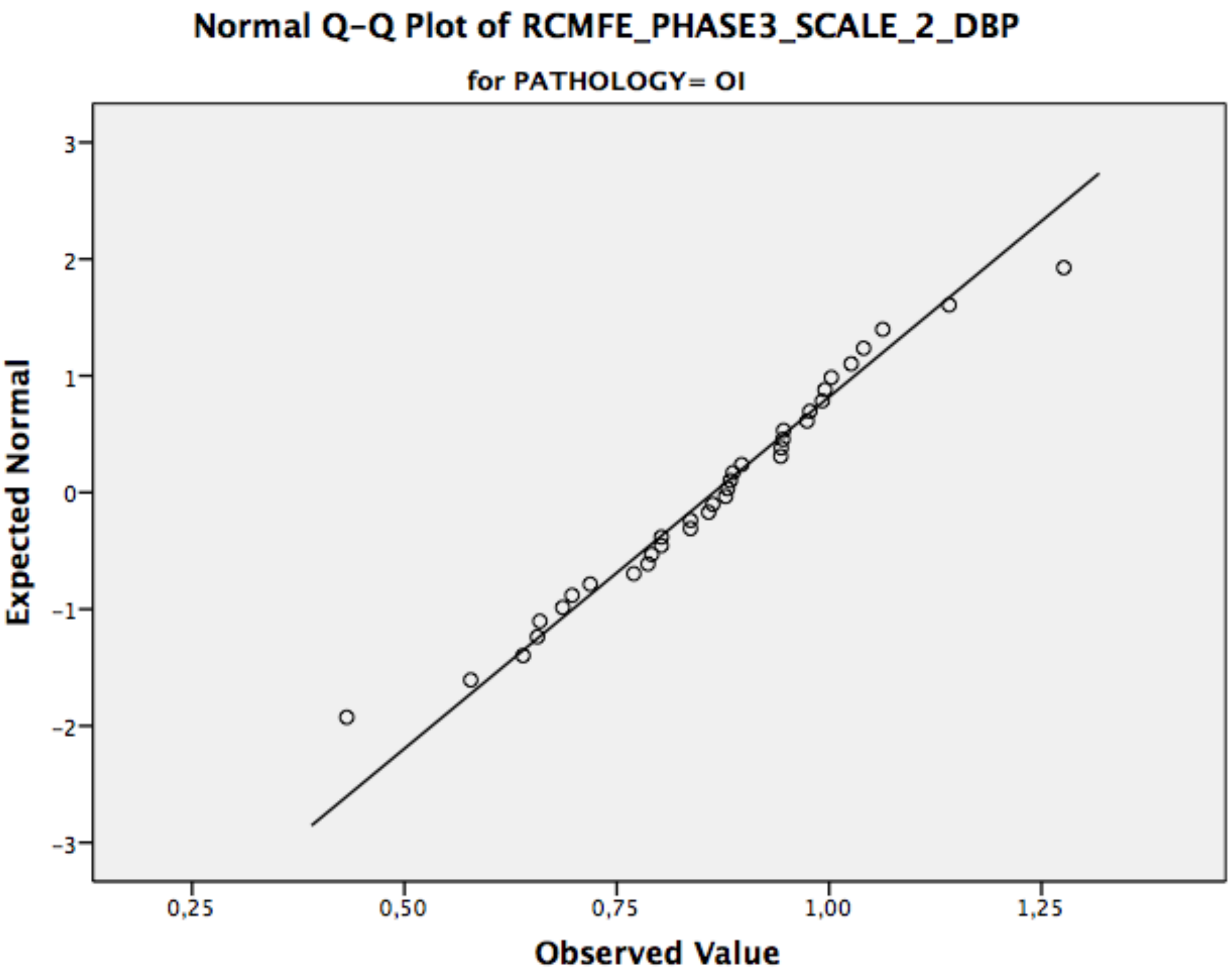
Normal Q-Q Plot of RCMFE_PHASE3_SCALE_2_DBP
for PATHOLOGY= OI
Expected Normal
Observed Value
3
2
1
0
-1
-2
-3
0,25
0,50
0,75
1,00
1,25

RCMFE_PHASE3_SCALE_3_DBP

Normal Q-Q Plots

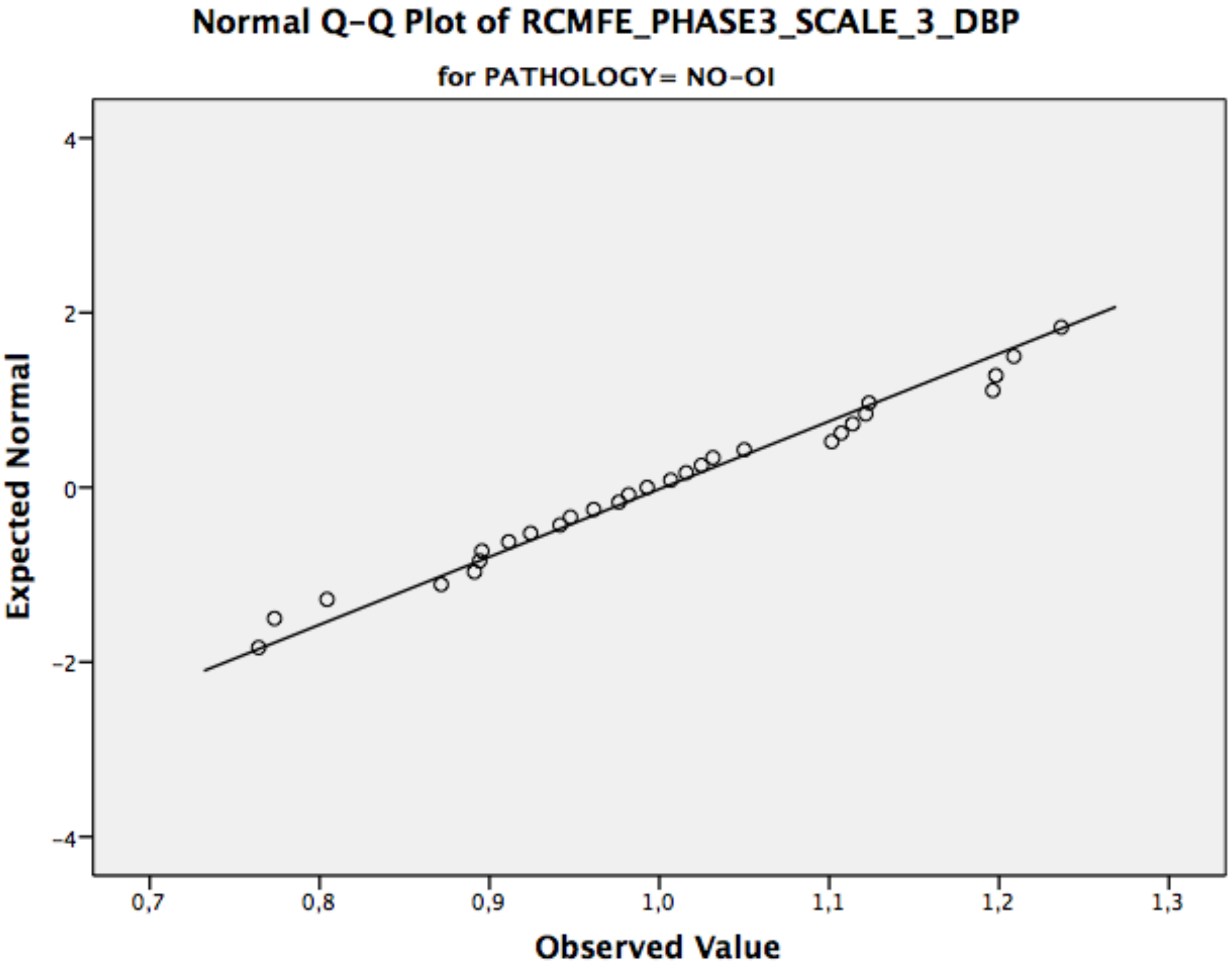

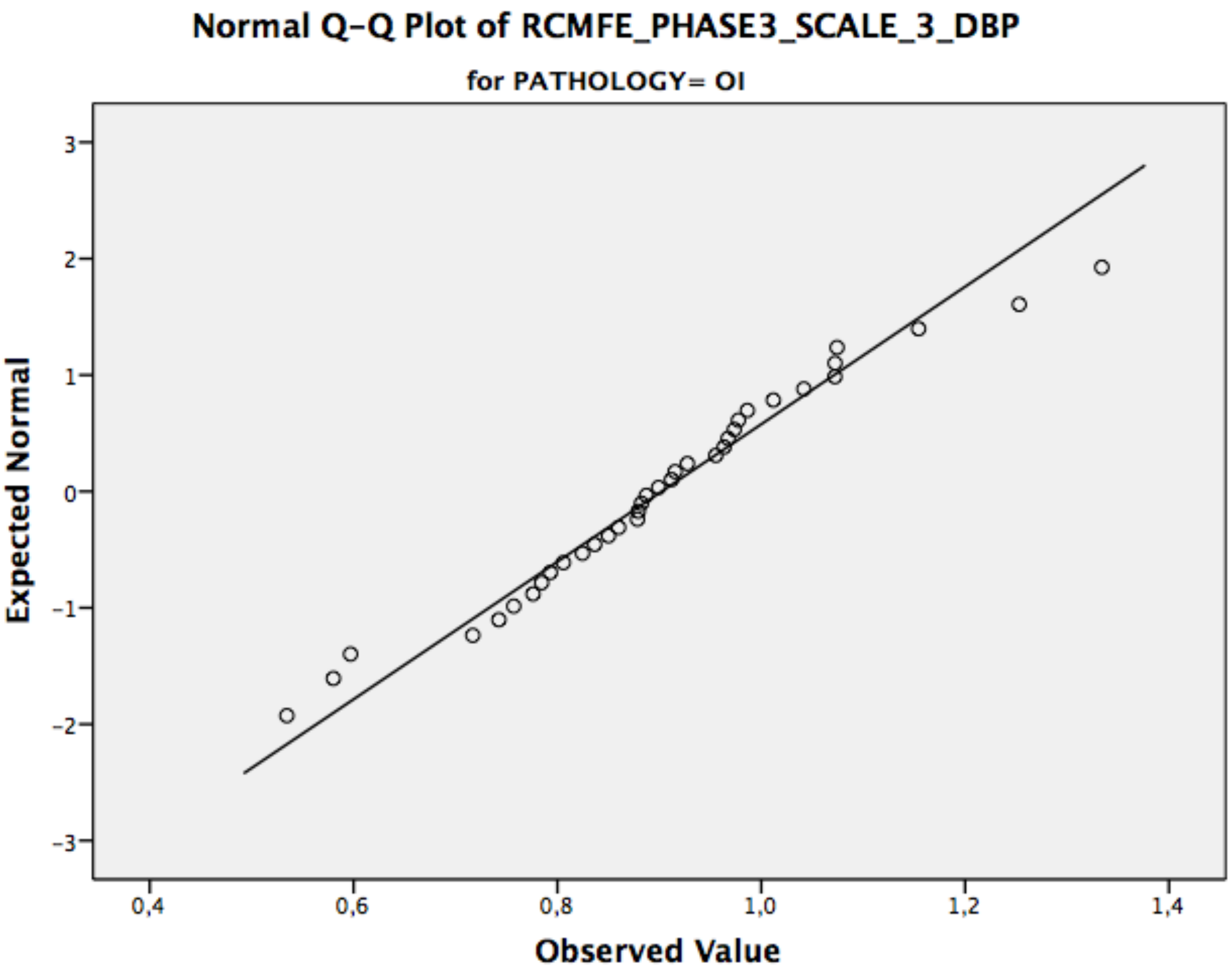

Normal Q-Q Plot of RCMFE_PHASE3_SCALE_3_DBP
for PATHOLOGY= OI
Expected Normal
Observed Value
3
2
1
0
-1
-2
-3
0,4
0,6
0,8
1,0
1,2
1,4

RCMFE_PHASE3_SCALE_4_DBP

Normal Q-Q Plots

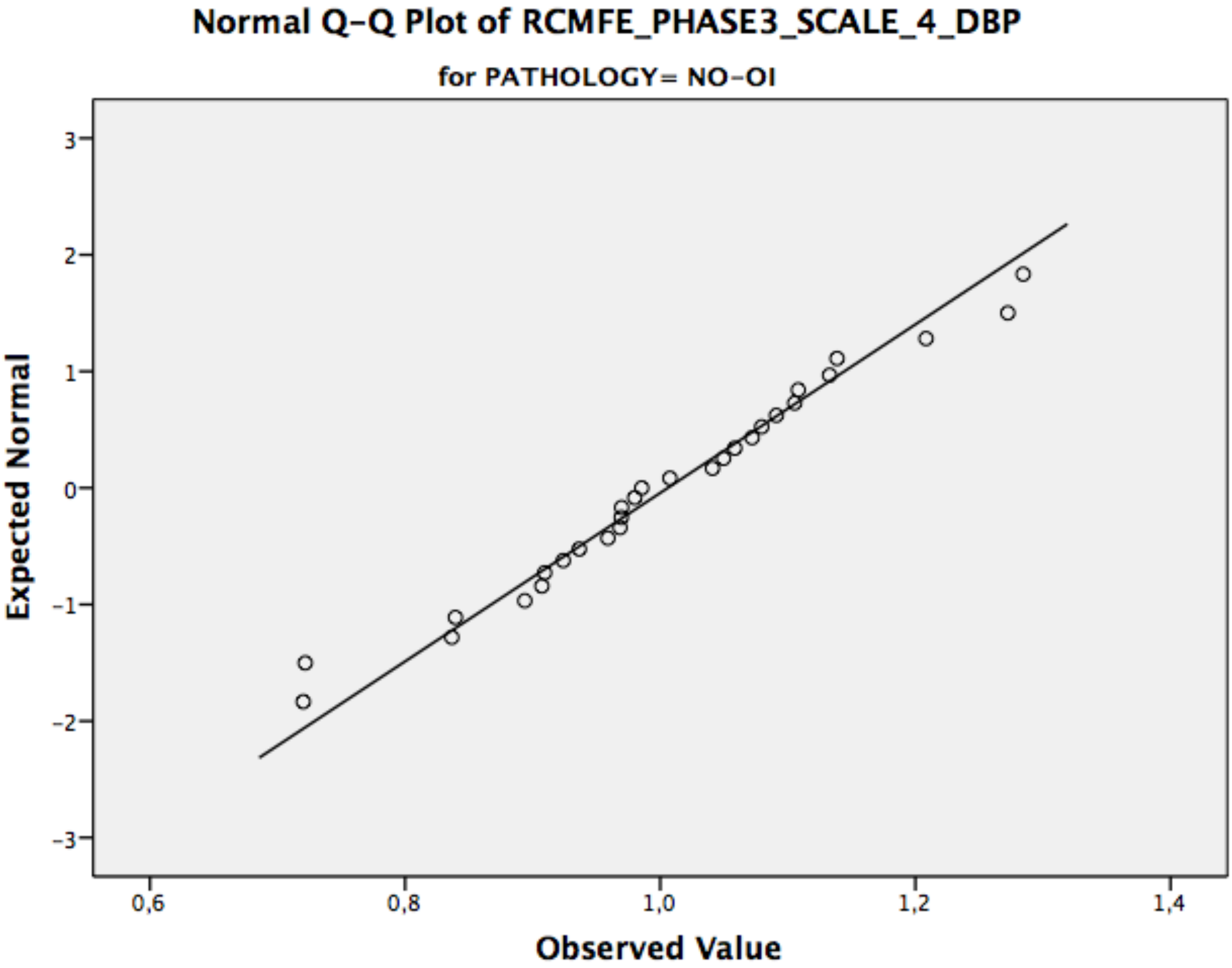

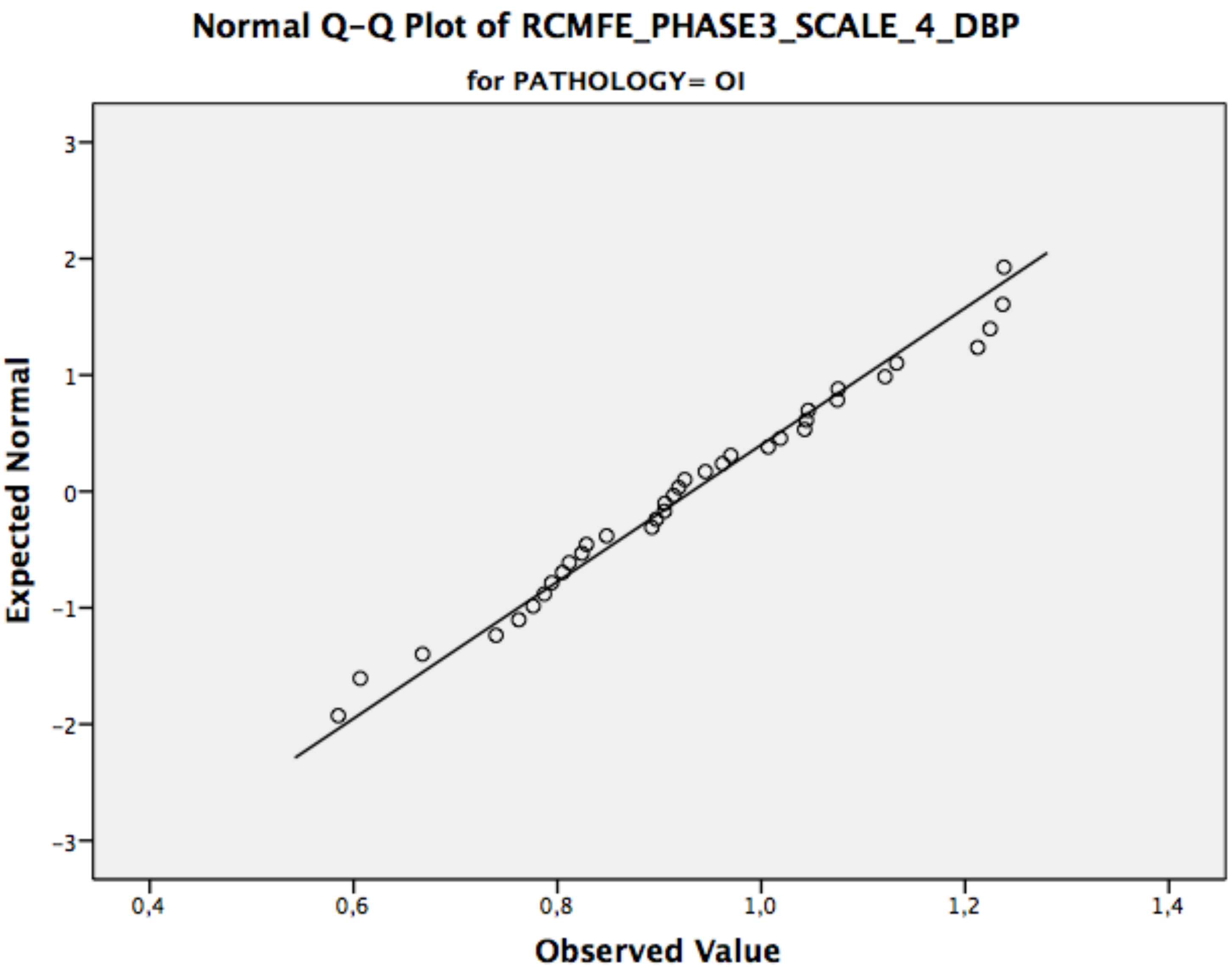
Normal Q-Q Plot of RCMFE_PHASE3_SCALE_4_DBP
for PATHOLOGY= OI
Expected Normal
Observed Value
3
2
1
0
-1
-2
-3
0,4
0,6
0,8
1,0
1,2
1,4

RCMFE_PHASE3_SCALE_5_DBP

Normal Q-Q Plots

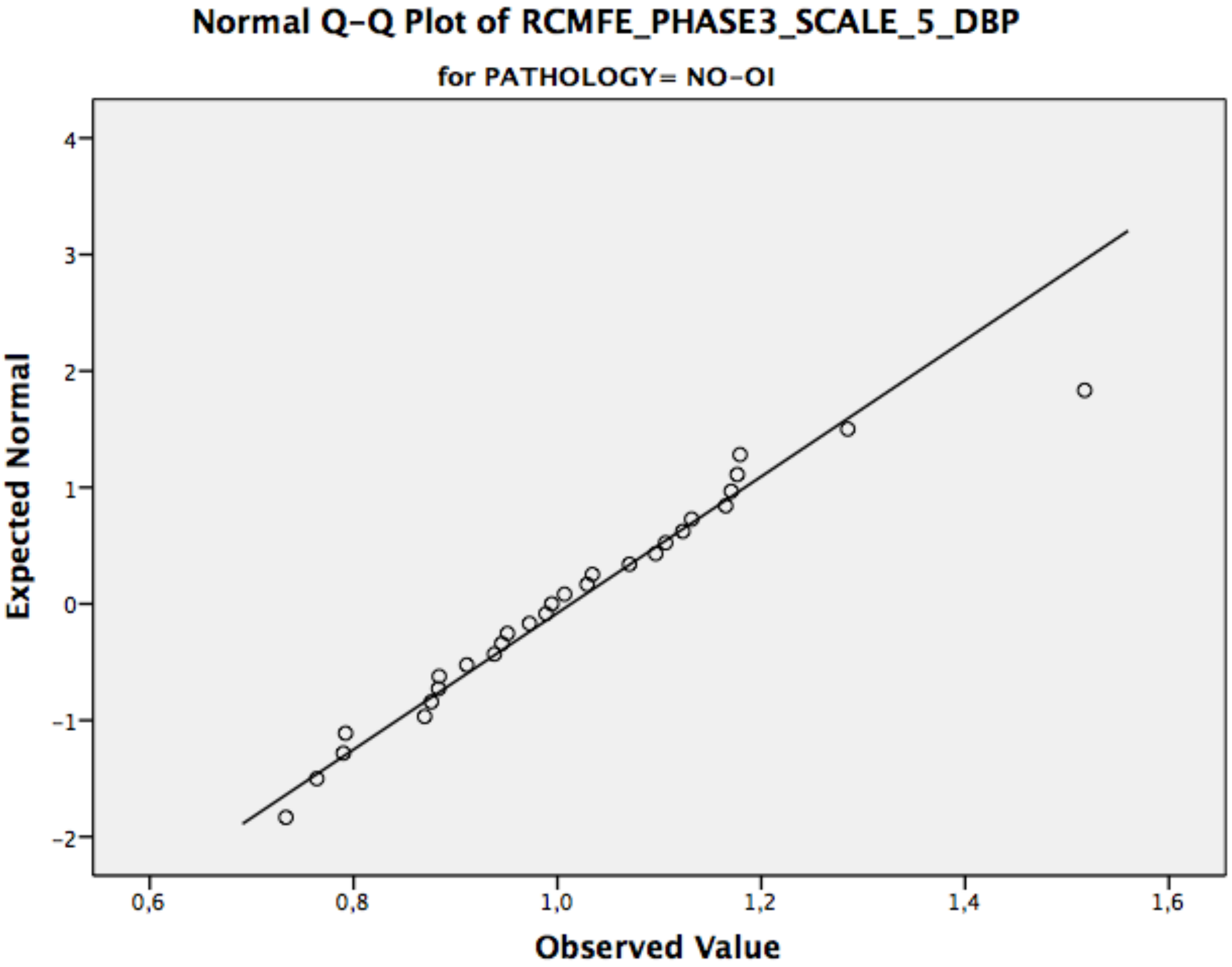

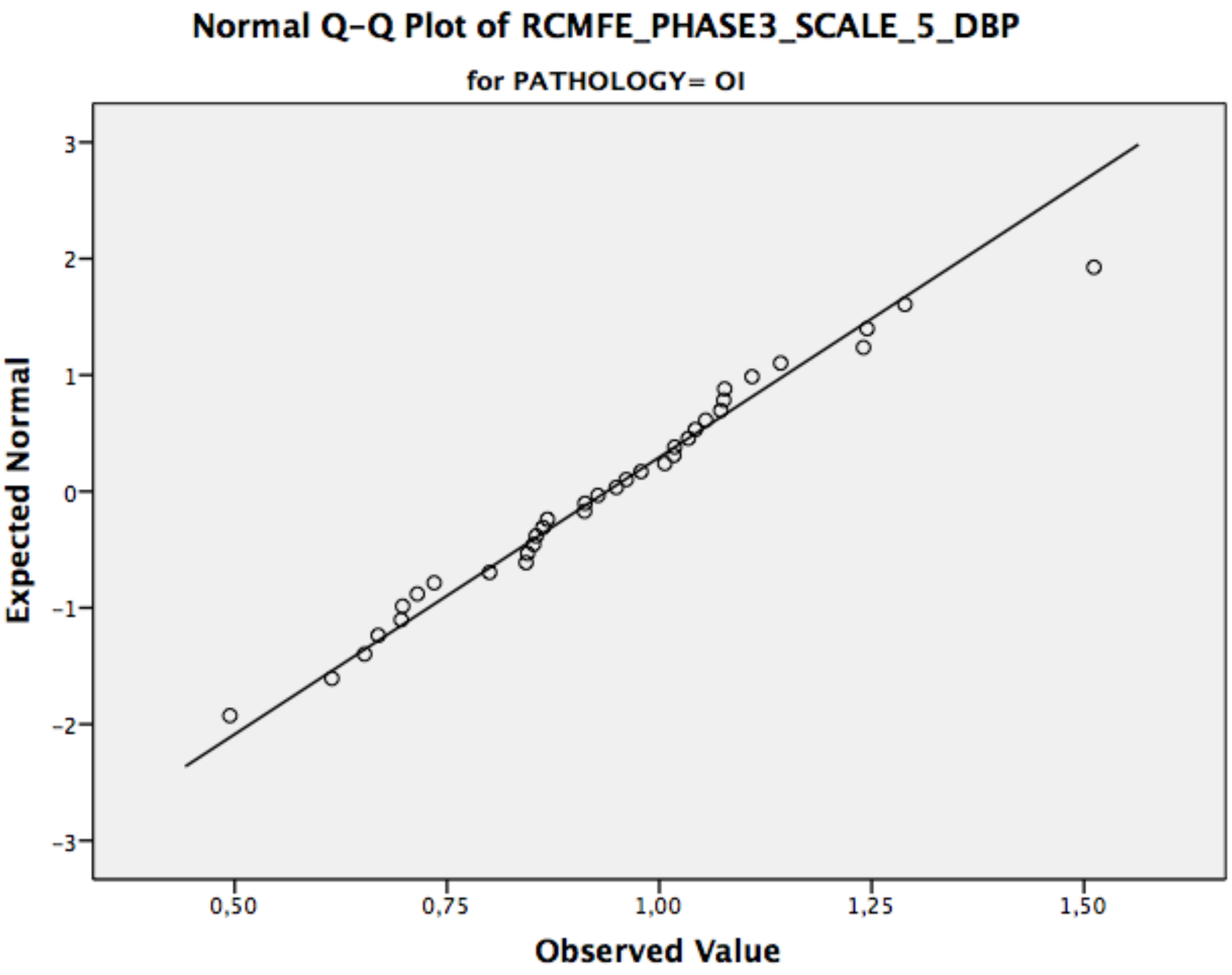
Normal Q-Q Plot of RCMFE_PHASE3_SCALE_5_DBP
for PATHOLOGY= OI
Expected Normal
3
2
1
0
-1
-2
-3
0,50
0,75
1,00
1,25
1,50
Observed Value

RCMFE_PHASE4_SCALE_1_DBP

Normal Q-Q Plots

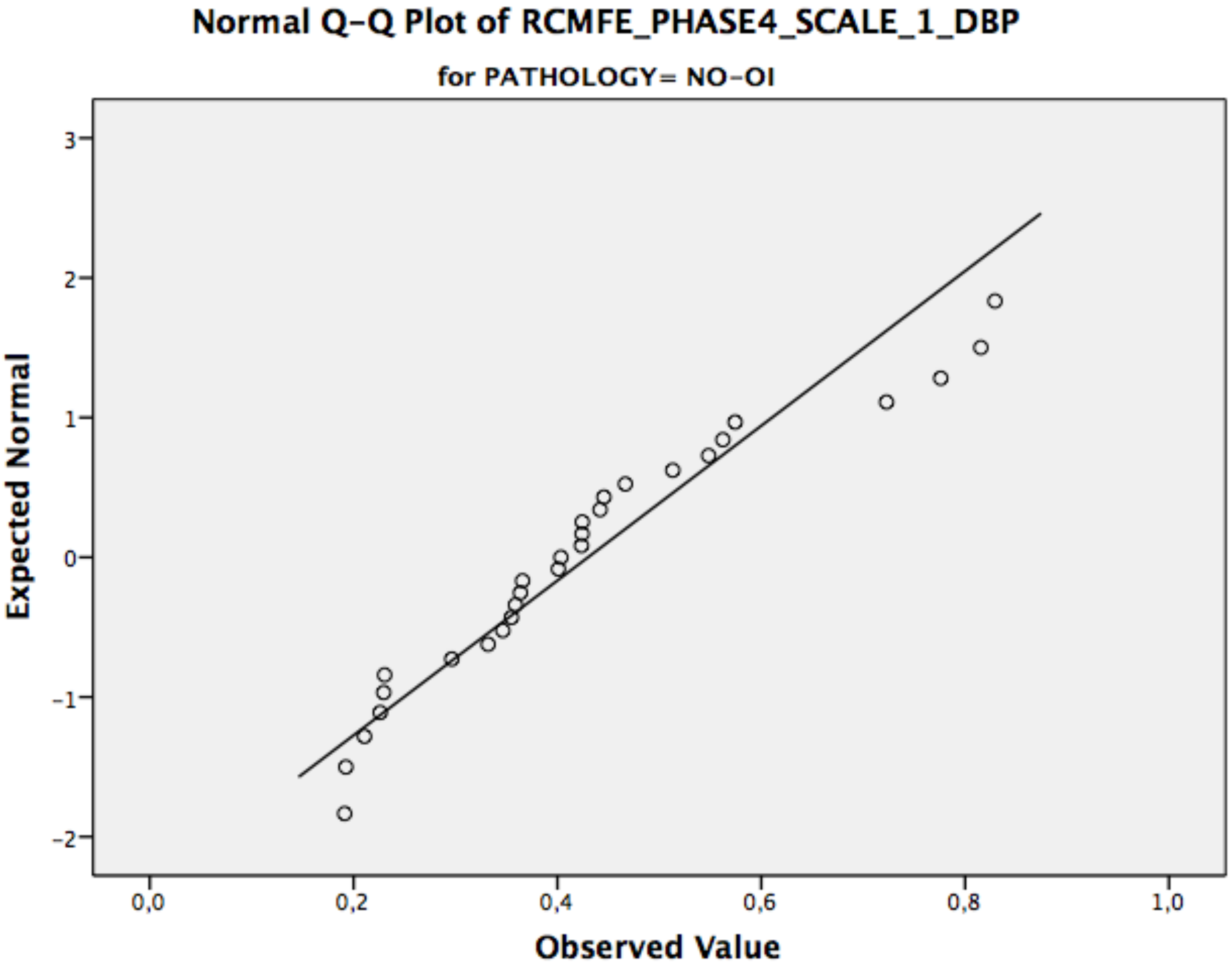

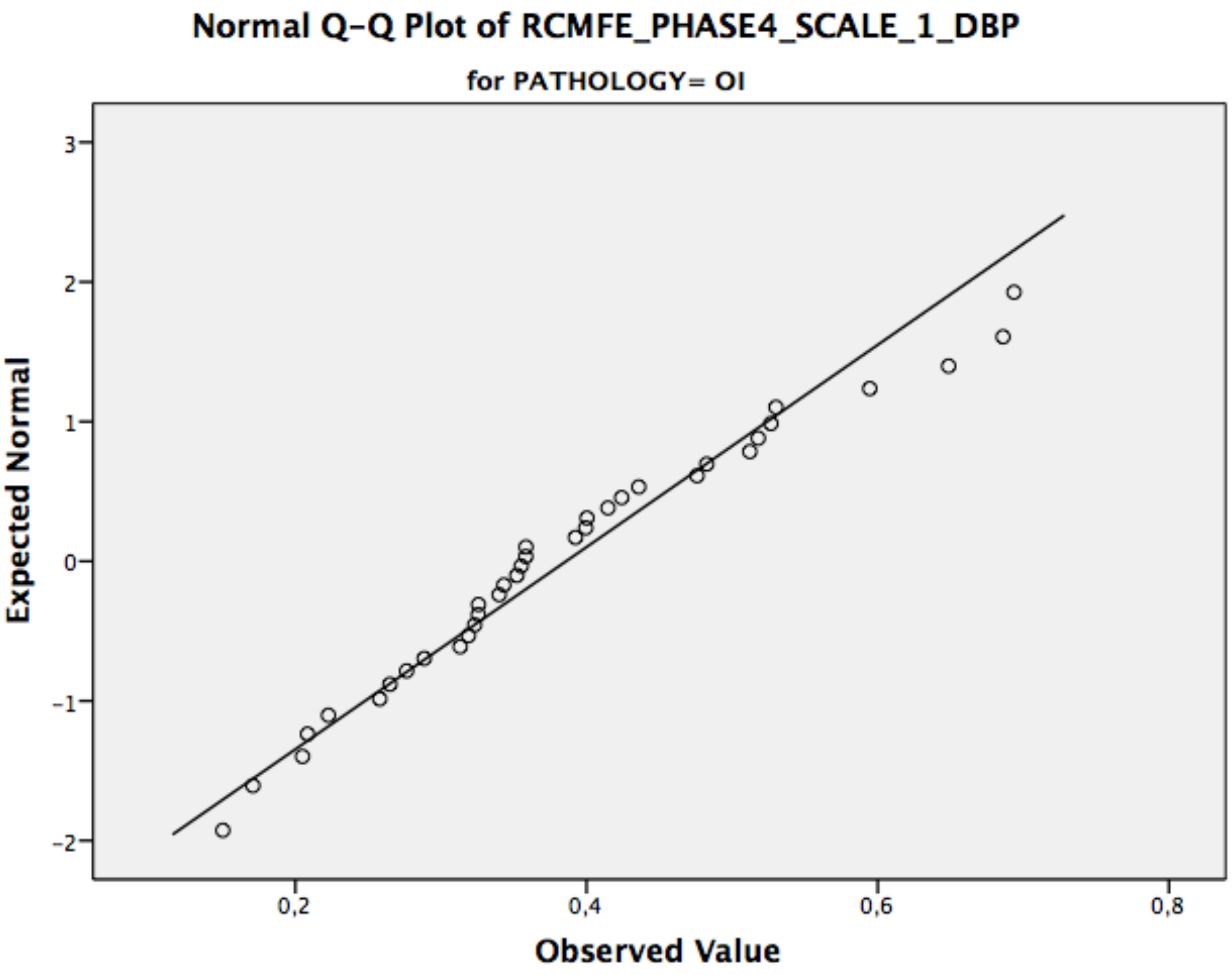
Normal Q-Q Plot of RCMFE_PHASE4_SCALE_1_DBP
for PATHOLOGY= OI
Expected Normal
3
2
1
0
−1
−2
0,2
0,4
0,6
0,8
Observed Value

RCMFE_PHASE4_SCALE_2_DBP

Normal Q-Q Plots

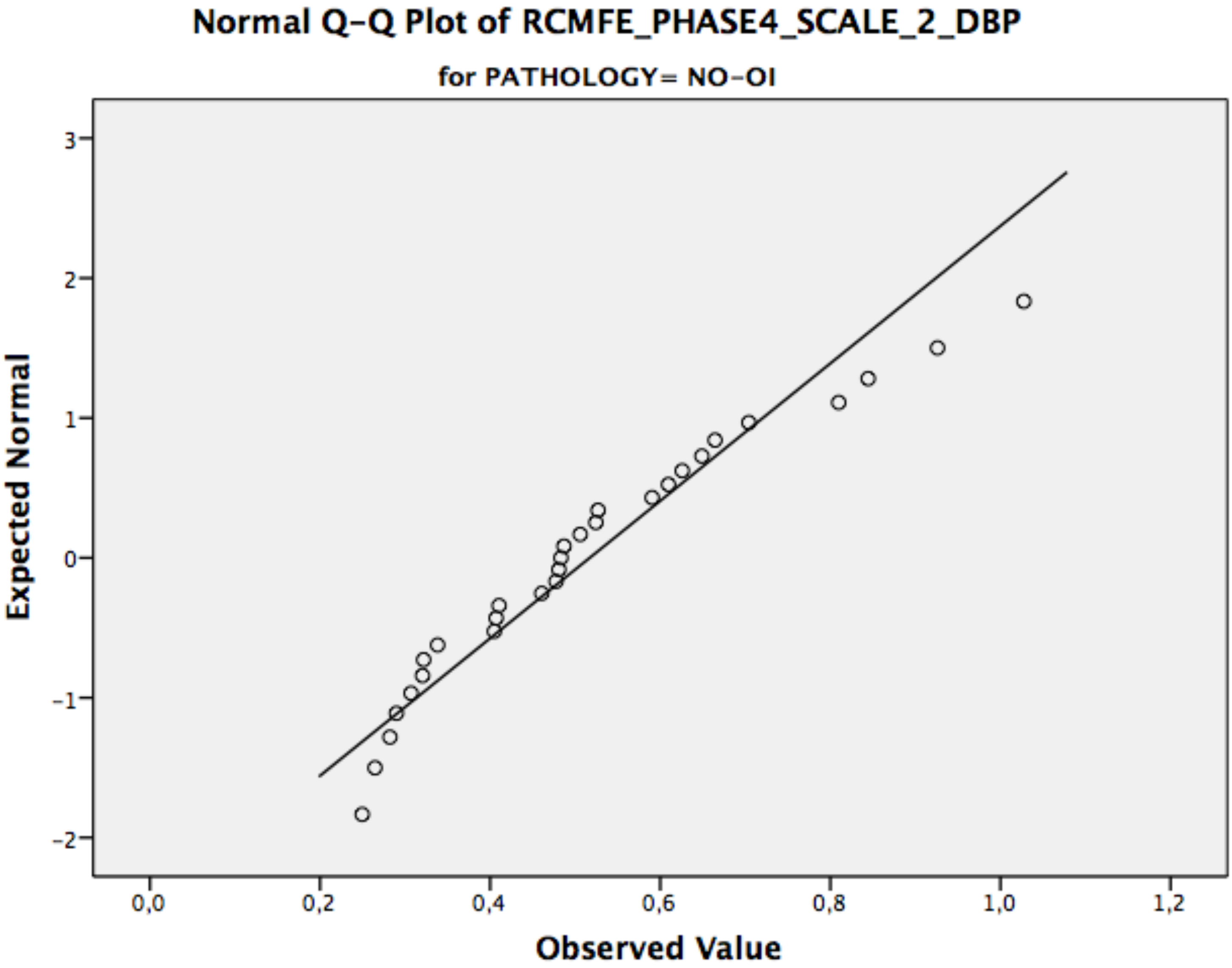

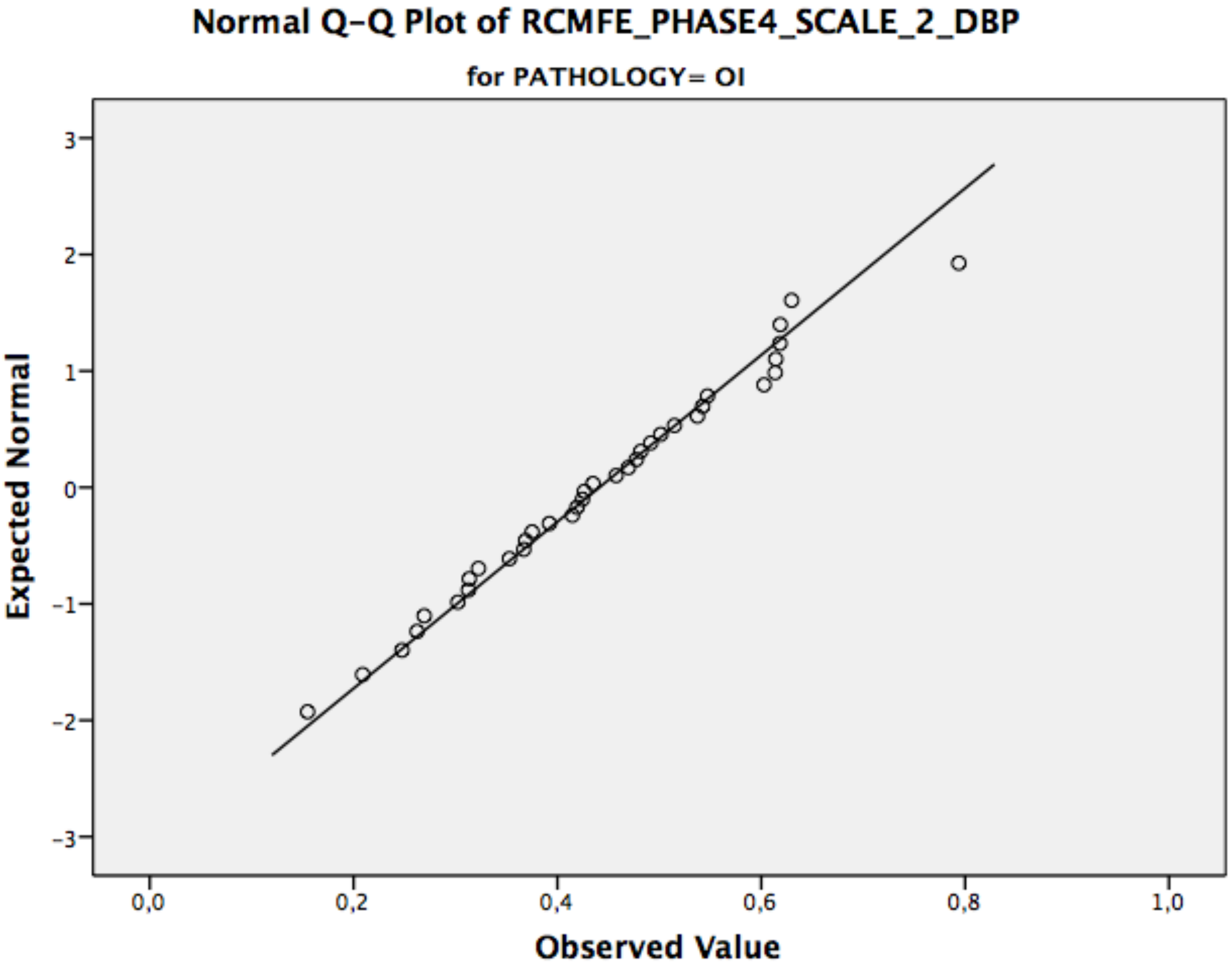
Normal Q-Q Plot of RCMFE_PHASE4_SCALE_2_DBP
for PATHOLOGY= OI
Expected Normal
3
2
1
0
-1
-2
-3
0,0
0,2
0,4
0,6
0,8
1,0
Observed Value

RCMFE_PHASE4_SCALE_3_DBP

Normal Q-Q Plots

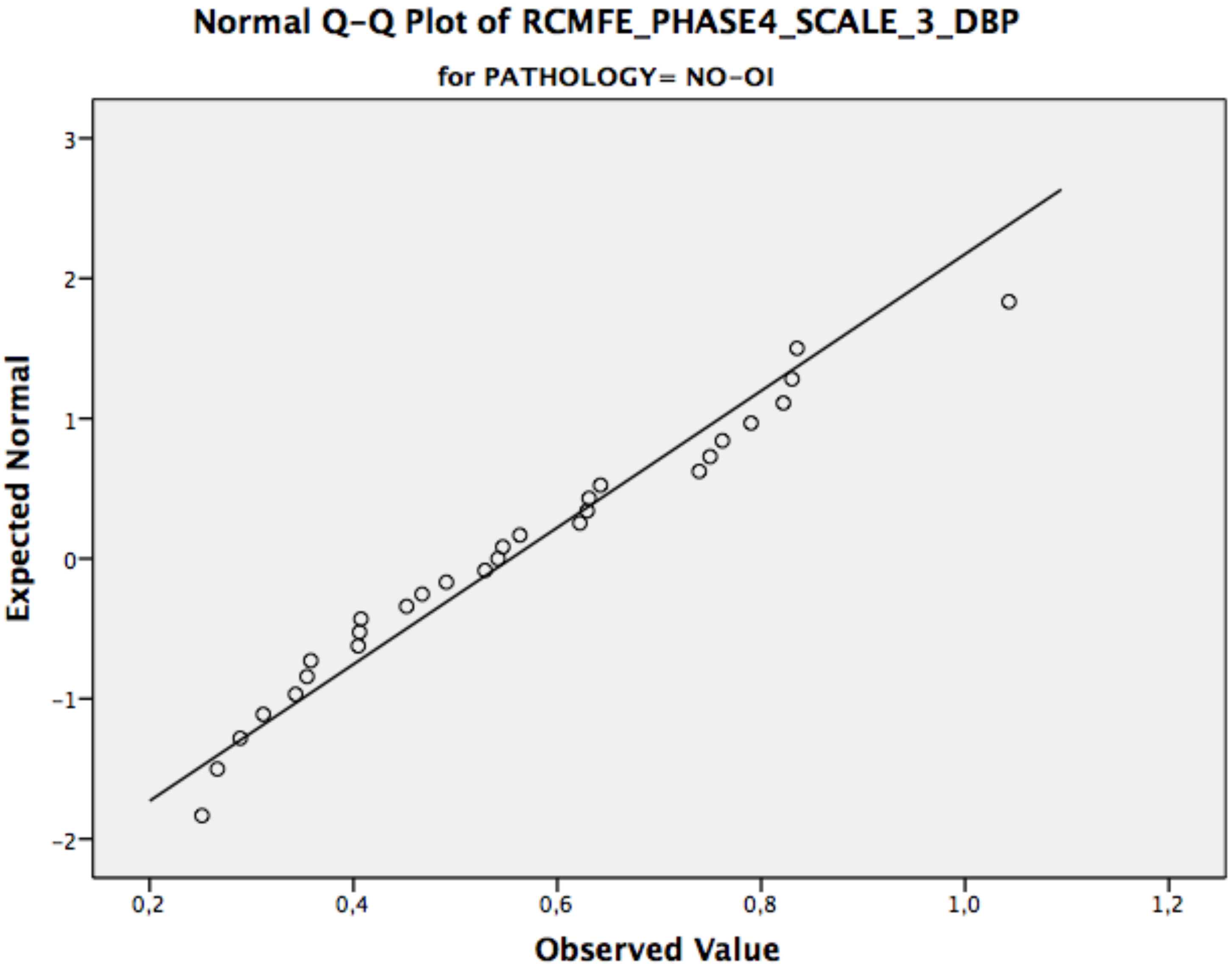

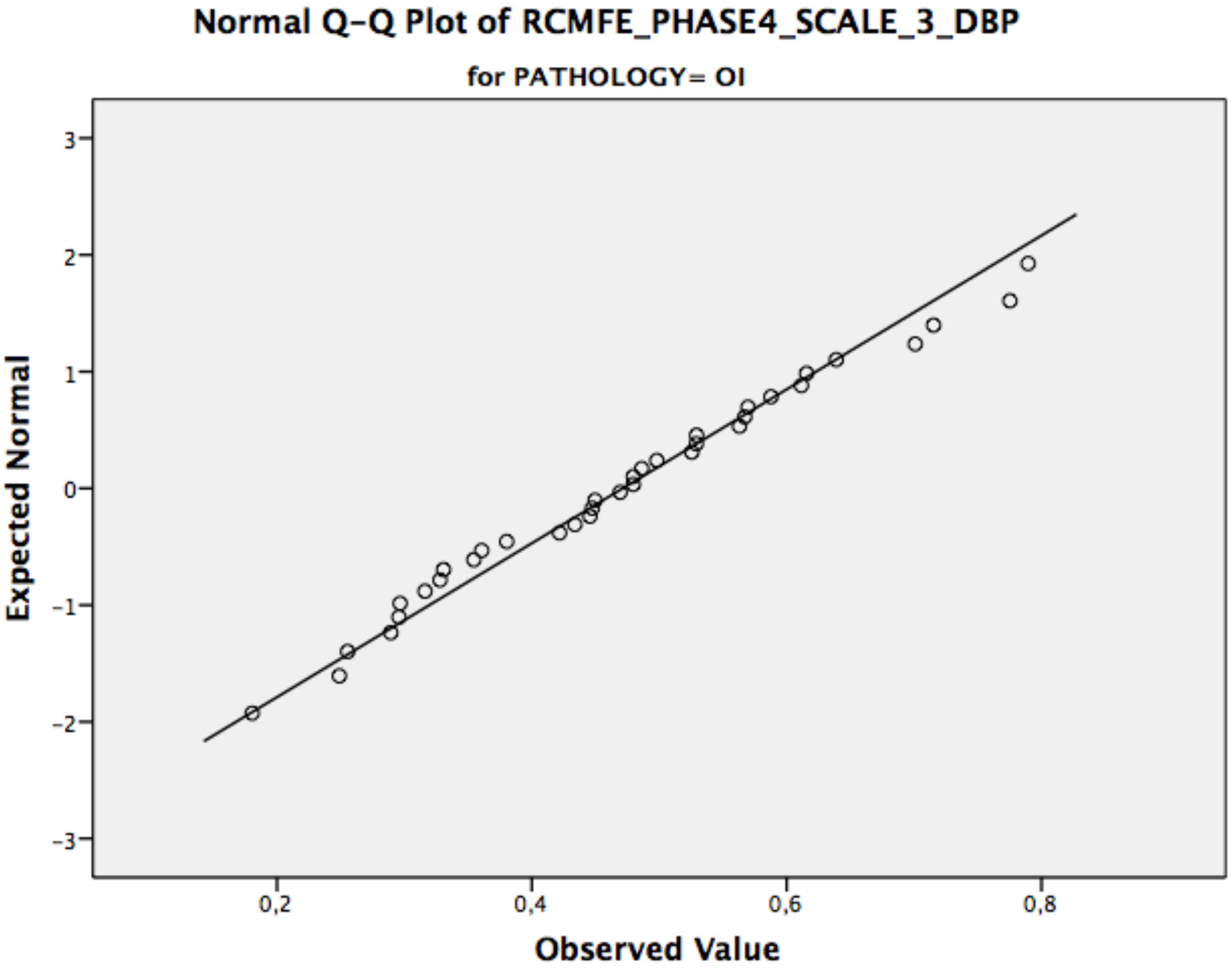
Normal Q-Q Plot of RCMFE_PHASE4_SCALE_3_DBP
for PATHOLOGY= OI
Expected Normal
3
2
1
0
−1
−2
−3
0,2
0,4
0,6
0,8
Observed Value

RCMFE_PHASE4_SCALE_4_DBP

Normal Q-Q Plots

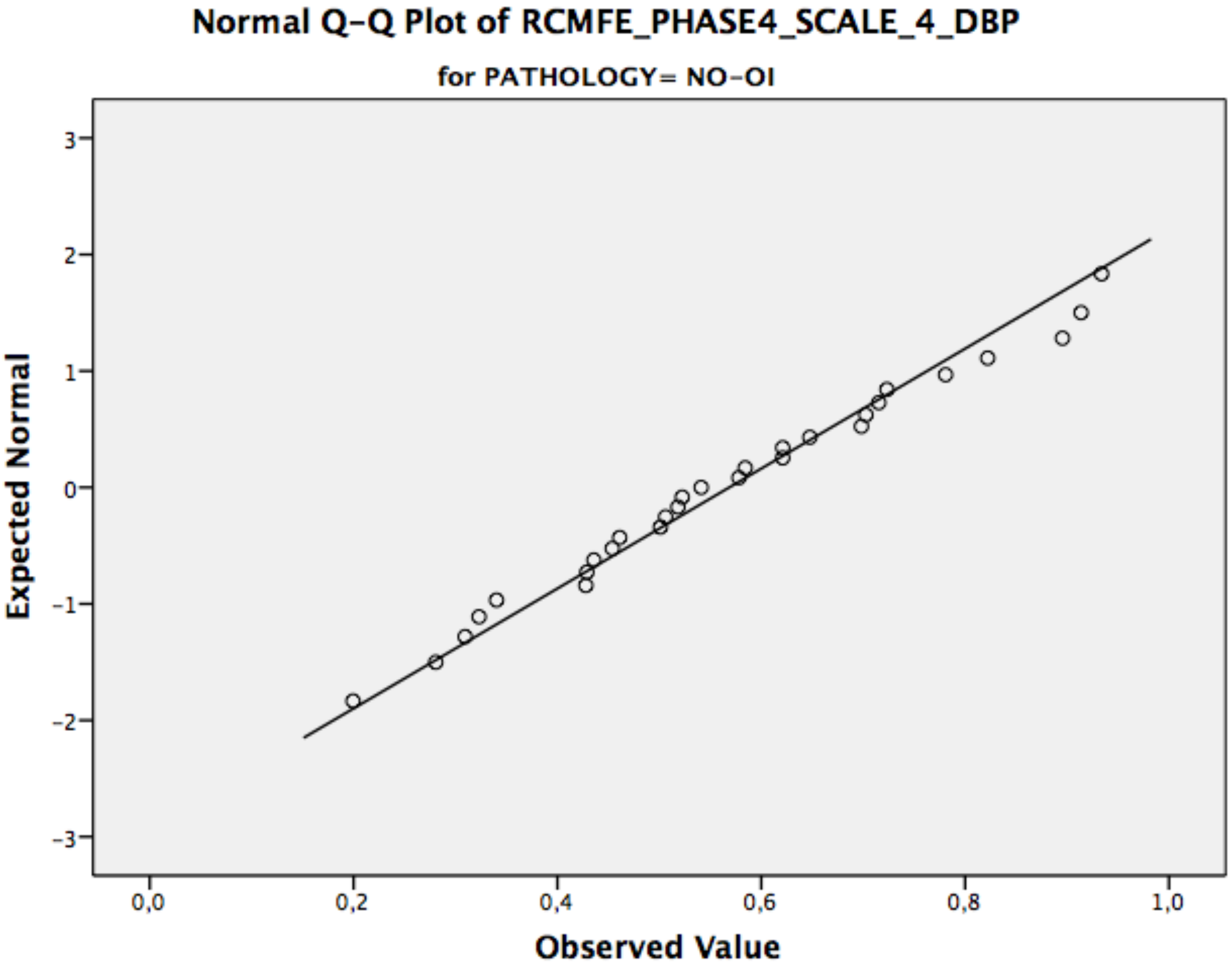

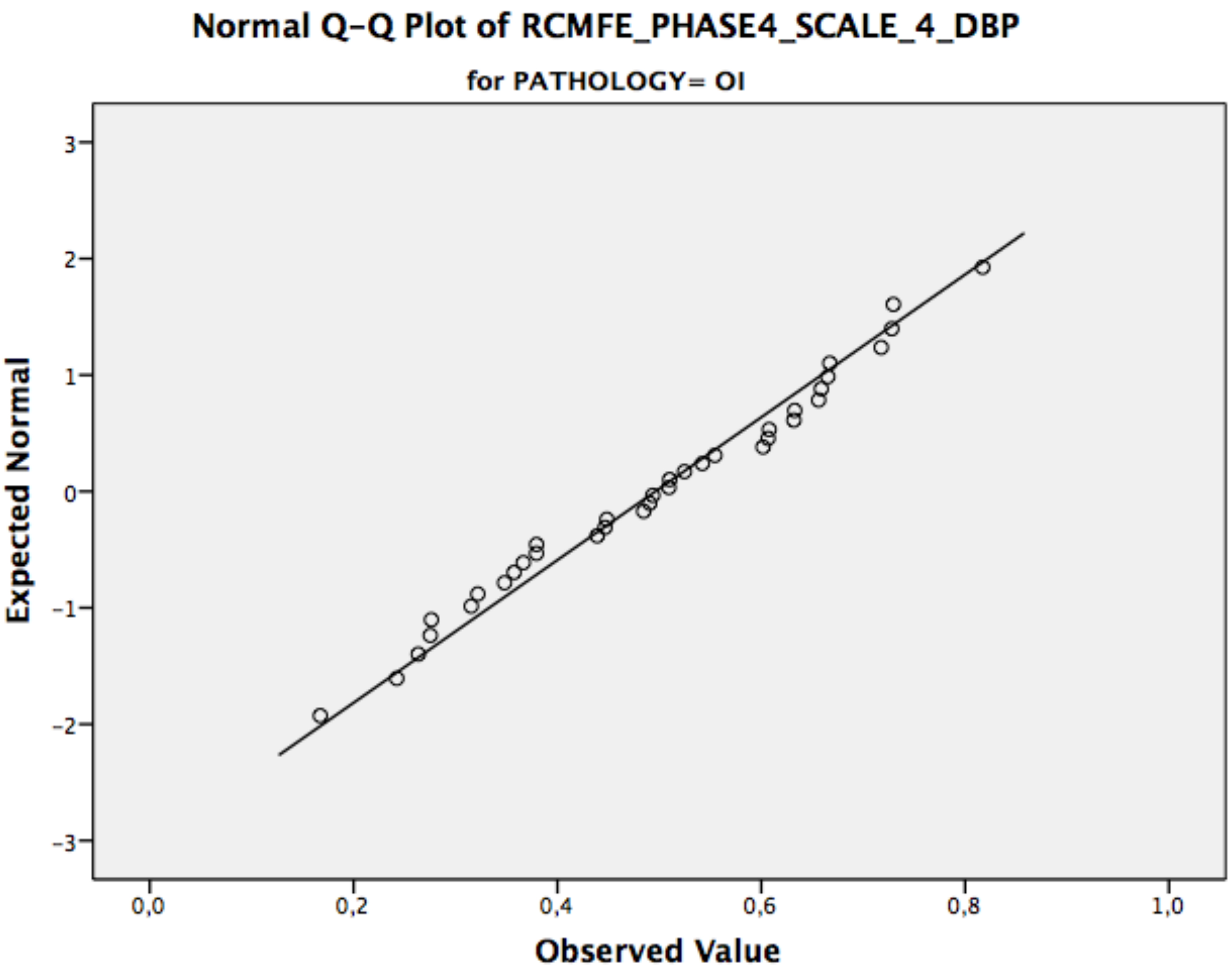
Normal Q-Q Plot of RCMFE_PHASE4_SCALE_4_DBP
for PATHOLOGY= OI
Expected Normal
Observed Value
3
2
1
0
-1
-2
-3
0,0
0,2
0,4
0,6
0,8
1,0

RCMFE_PHASE4_SCALE_5_DBP

Normal Q-Q Plots

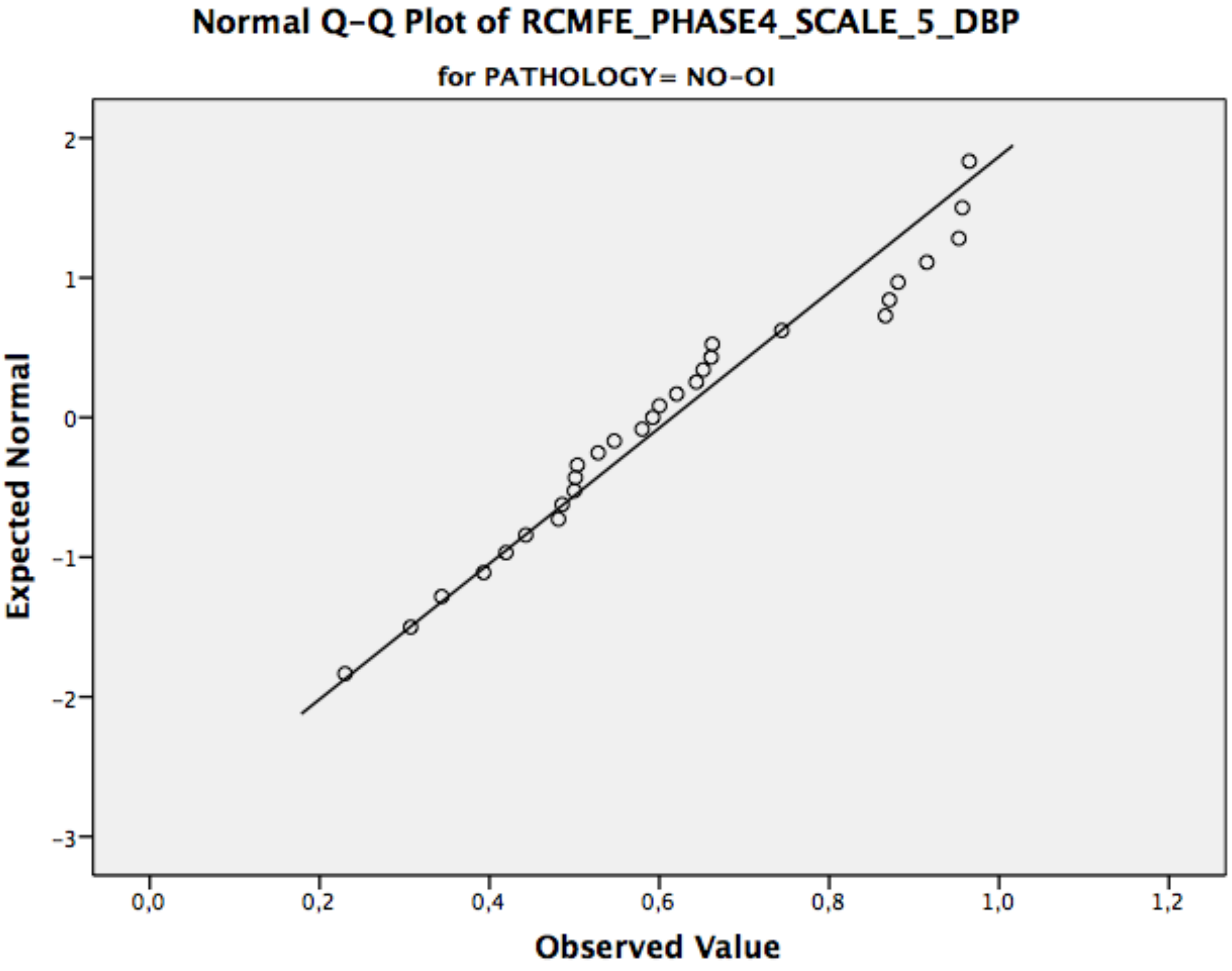

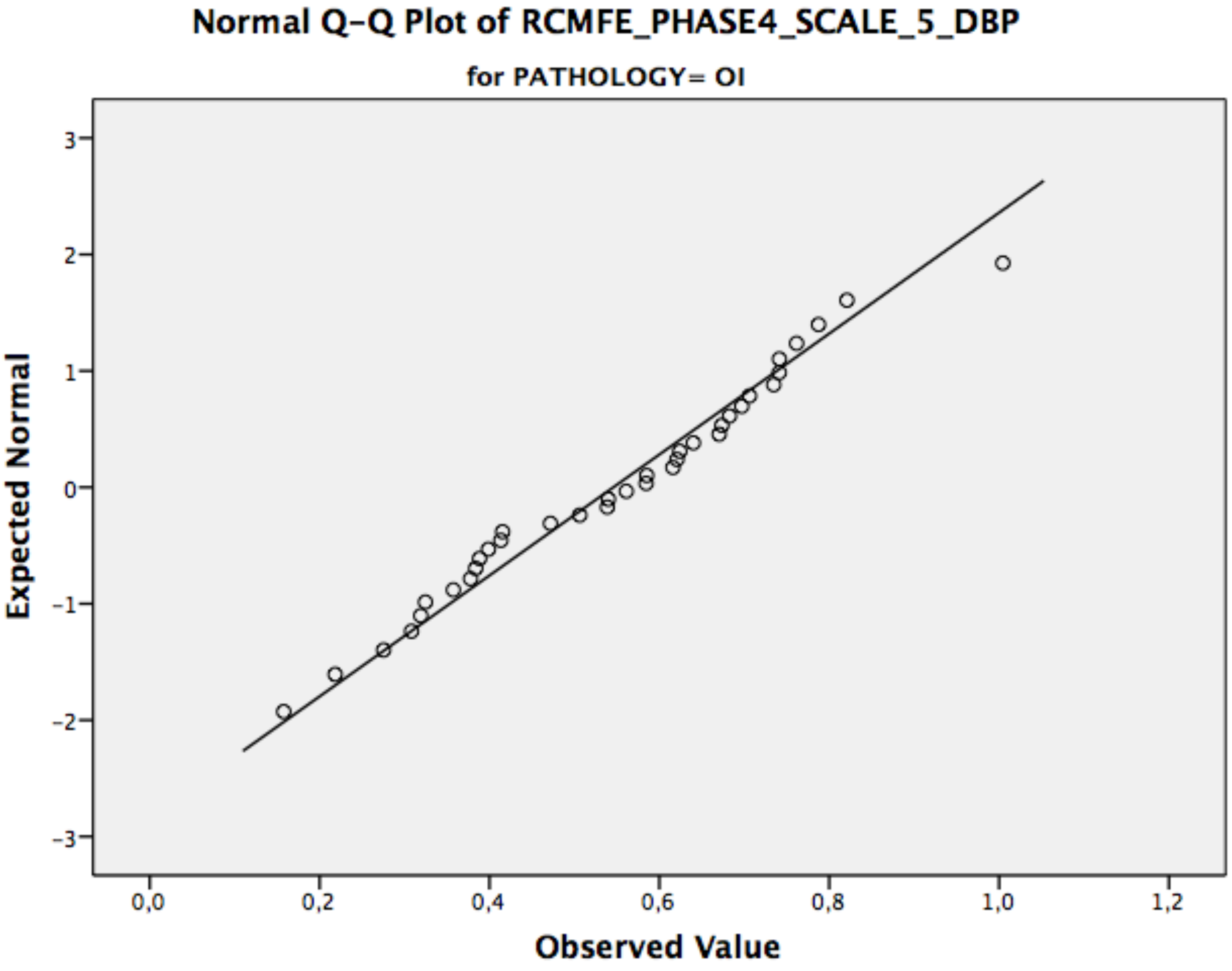

Normal Q-Q Plot of RCMFE_PHASE4_SCALE_5_DBP
for PATHOLOGY= OI
Expected Normal
Observed Value
3
2
1
0
-1
-2
-3
0,0
0,2
0,4
0,6
0,8
1,0
1,2

RCMFE_PHASE5_SCALE_1_DBP

Normal Q-Q Plots

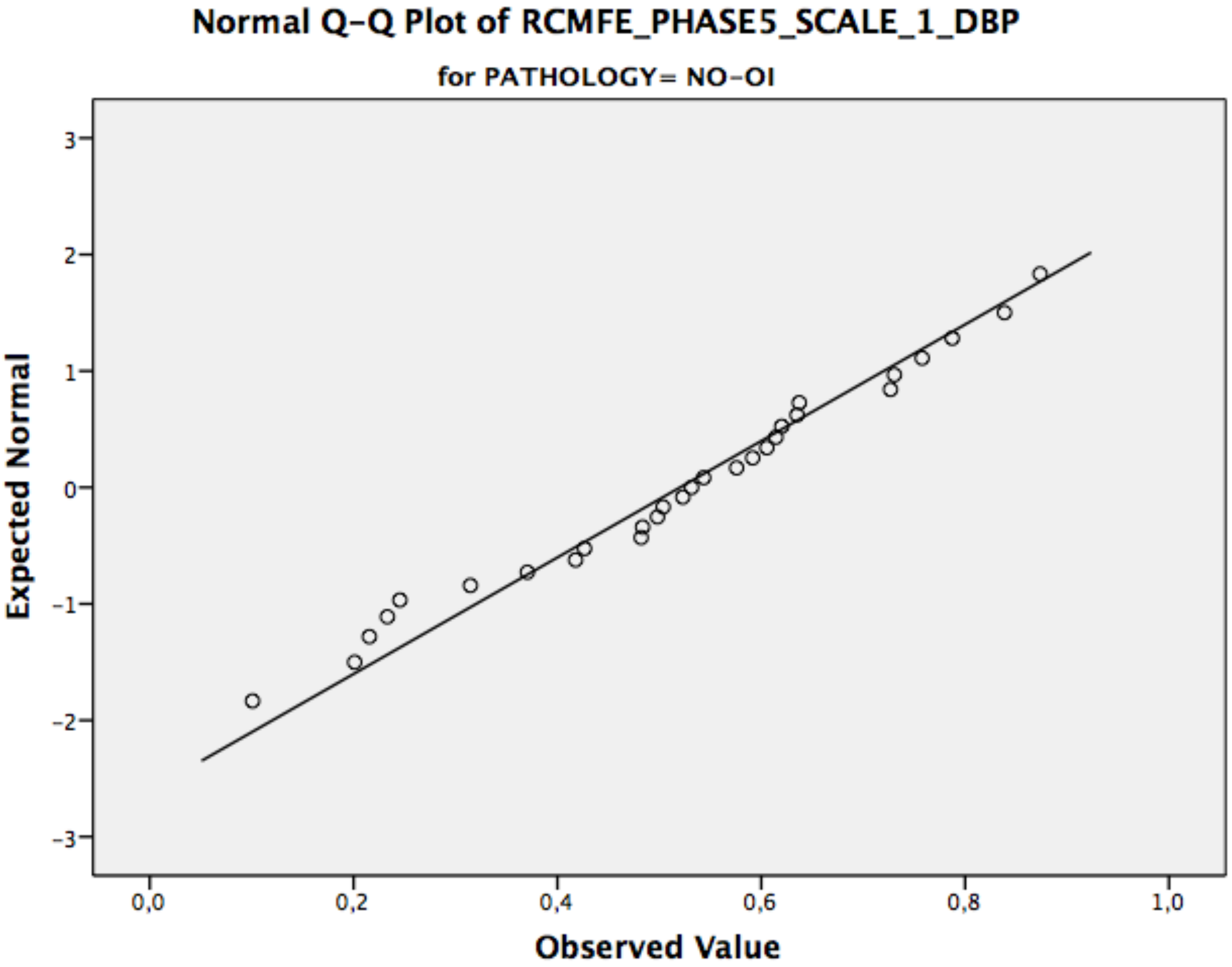

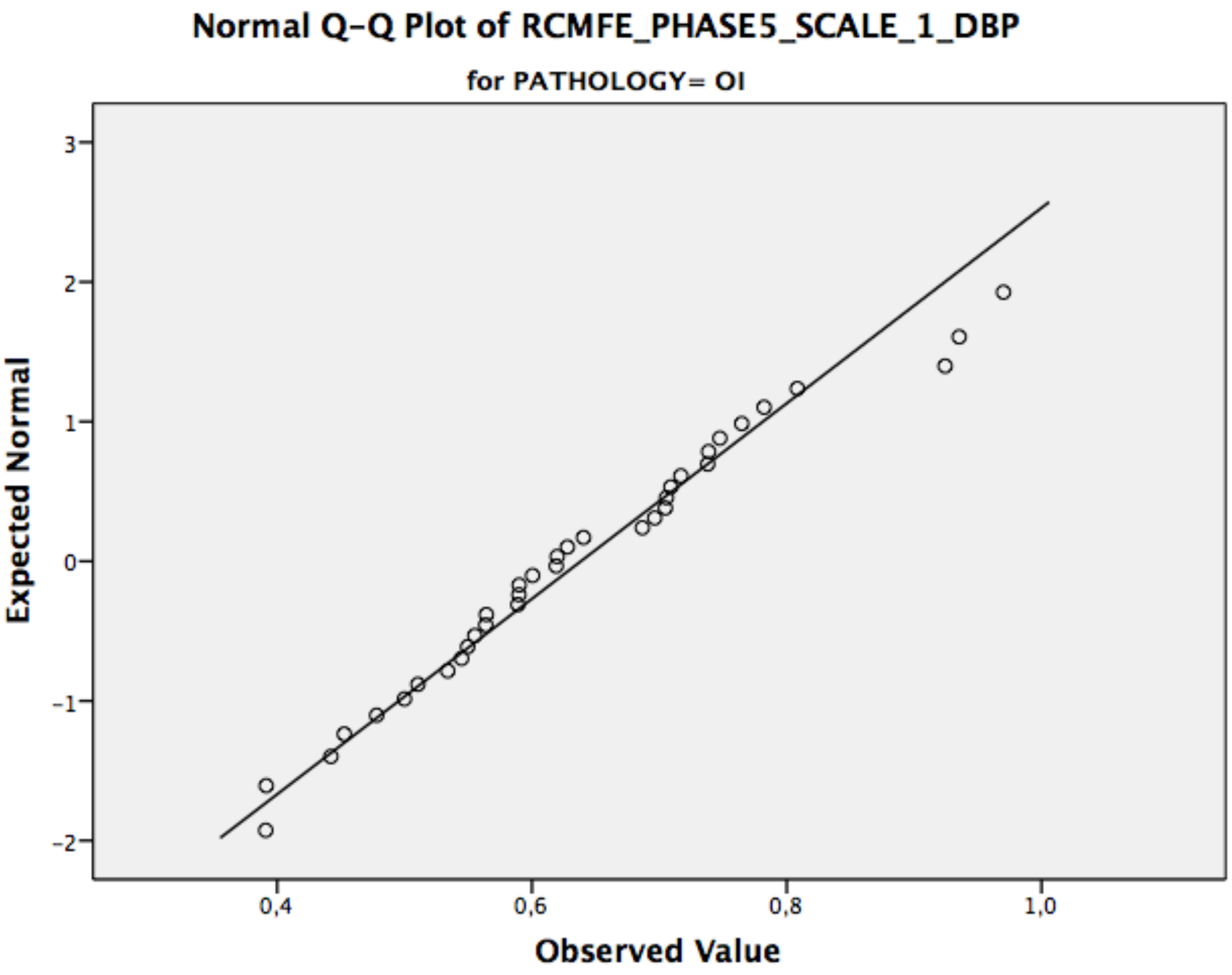
Normal Q-Q Plot of RCMFE_PHASE5_SCALE_1_DBP
for PATHOLOGY= OI
Expected Normal
3
2
1
0
−1
−2
0,4
0,6
0,8
1,0
Observed Value

RCMFE_PHASE5_SCALE_2_DBP

Normal Q-Q Plots

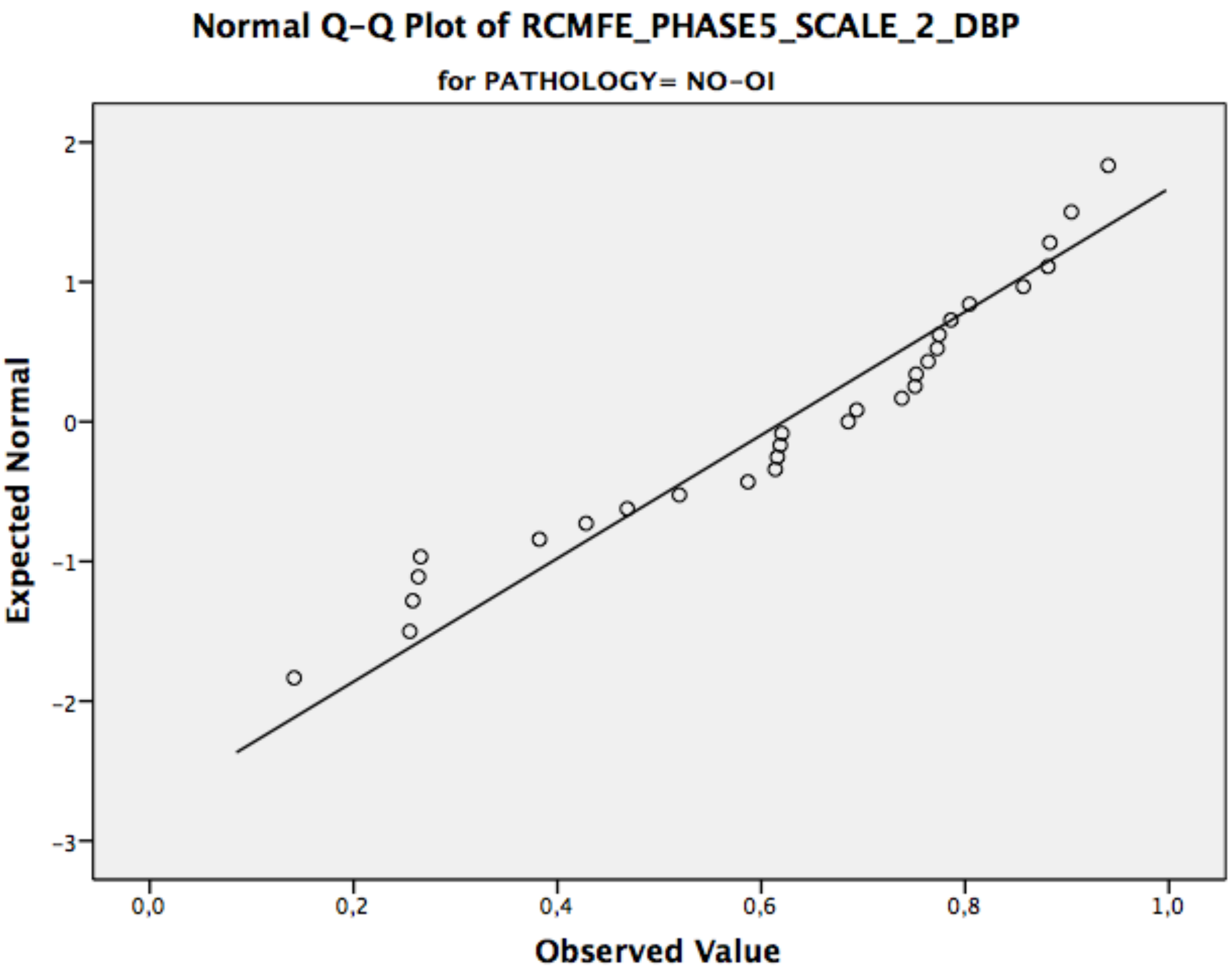

Normal Q-Q Plot of RCMFE_PHASE5_SCALE_2_DBP

for PATHOLOGY= OI

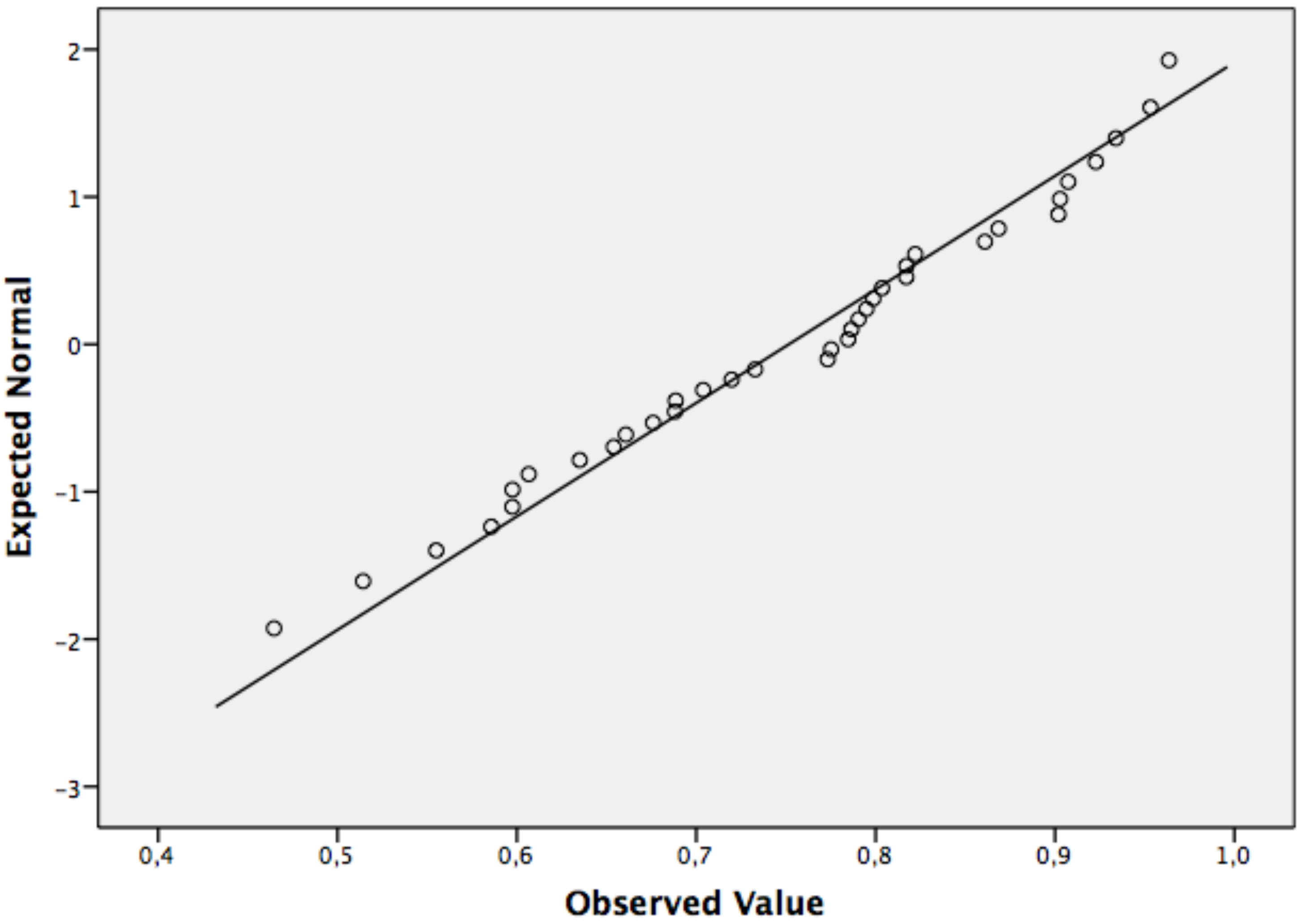

RCMFE_PHASE5_SCALE_3_DBP

Normal Q-Q Plots

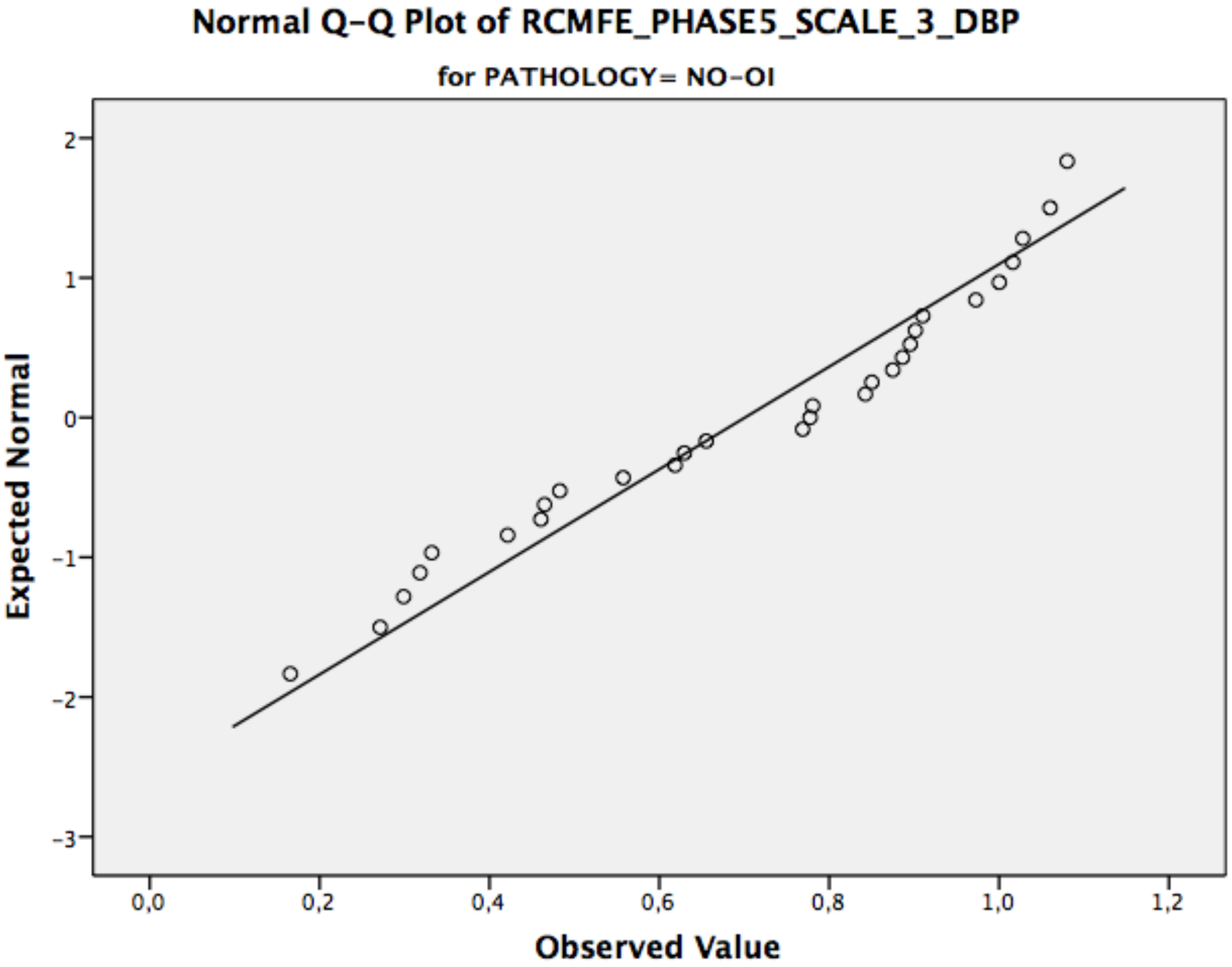

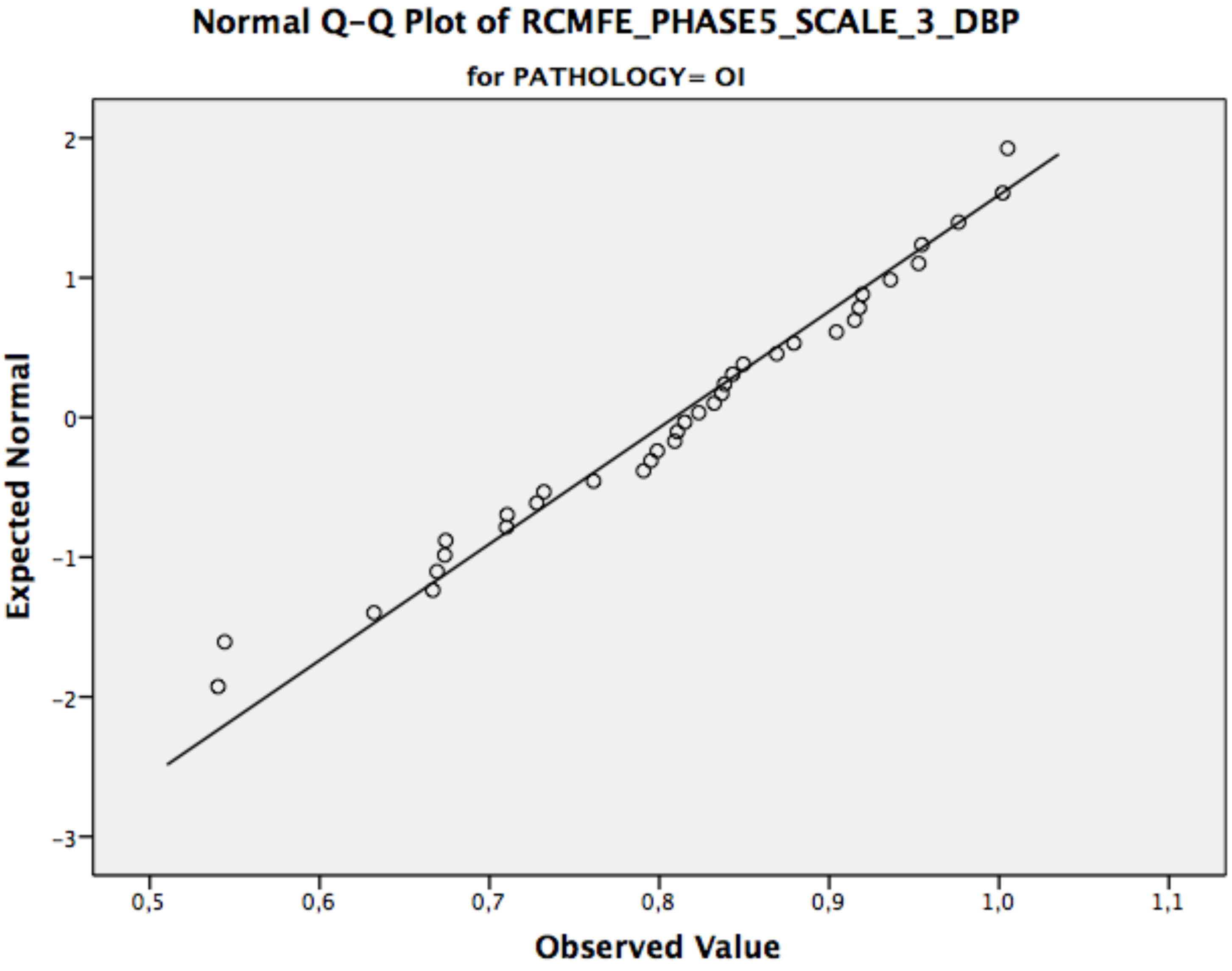
Normal Q-Q Plot of RCMFE_PHASE5_SCALE_3_DBP
for PATHOLOGY= OI
Expected Normal
Observed Value
2
1
0
-1
-2
-3
0,5
0,6
0,7
0,8
0,9
1,0
1,1

RCMFE_PHASE5_SCALE_4_DBP

Normal Q-Q Plots

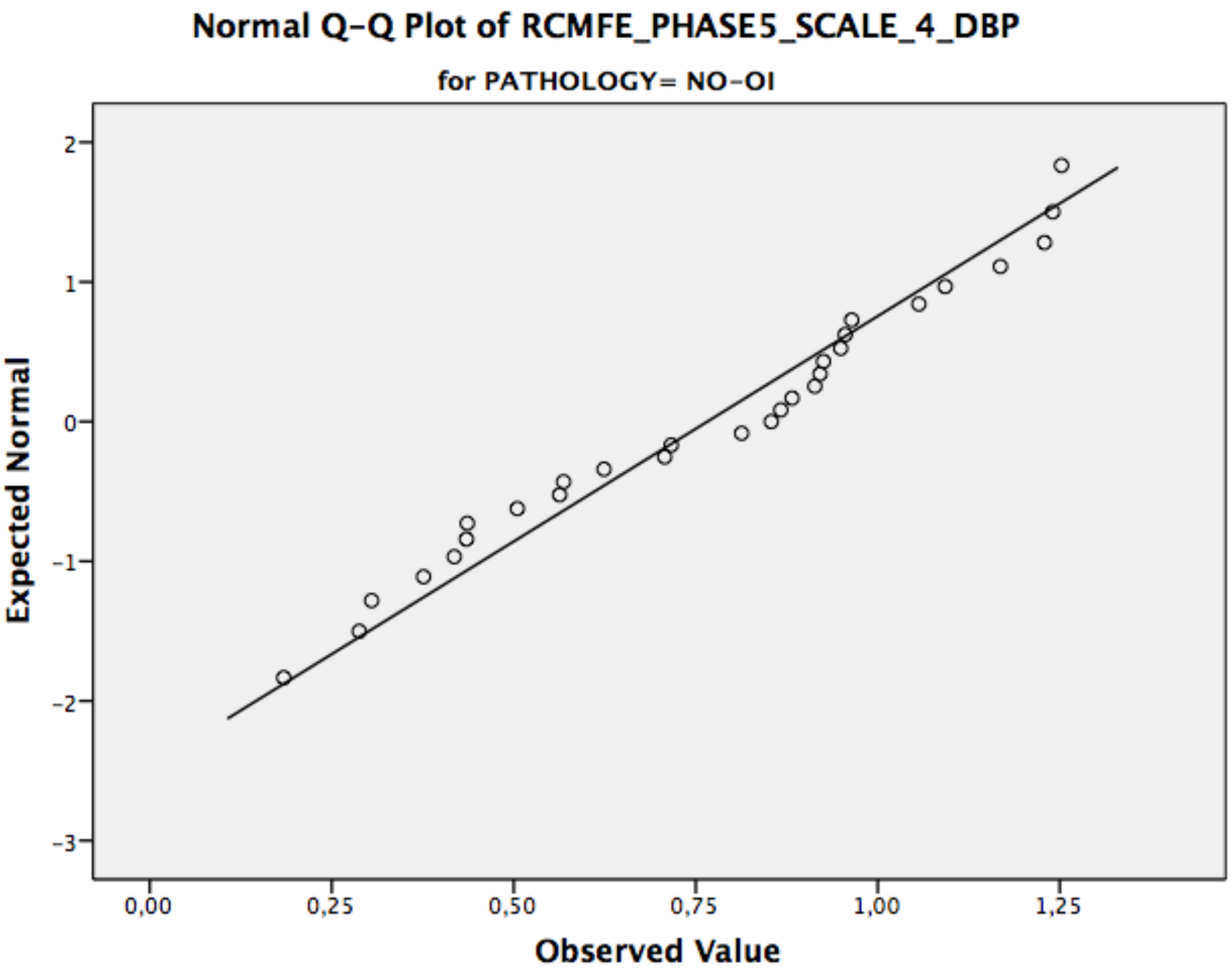

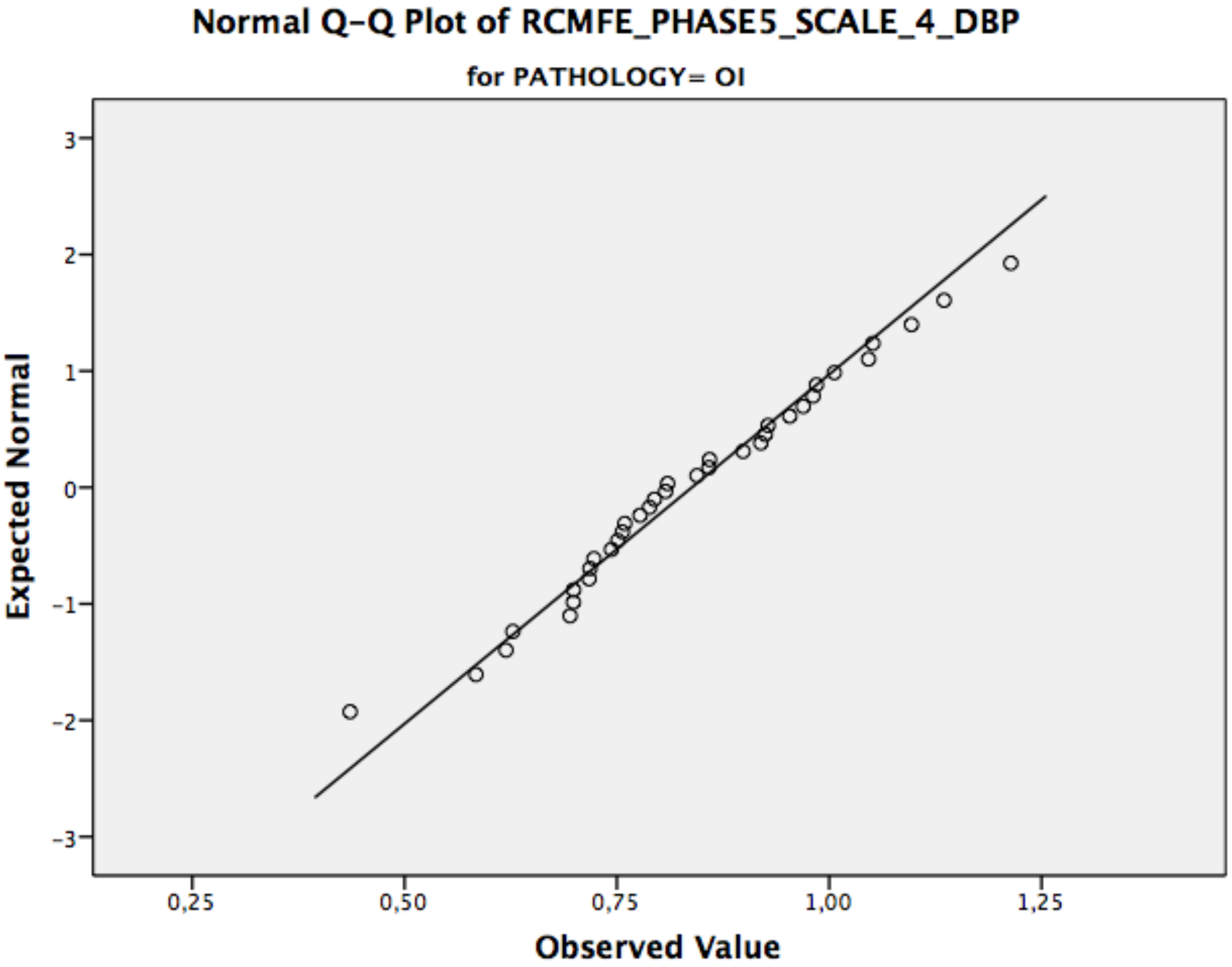

Normal Q-Q Plot of RCMFE_PHASE5_SCALE_4_DBP
for PATHOLOGY= OI
Expected Normal
3
2
1
0
-1
-2
-3
0,25
0,50
0,75
1,00
1,25
Observed Value

RCMFE_PHASE5_SCALE_5_DBP

Normal Q-Q Plots

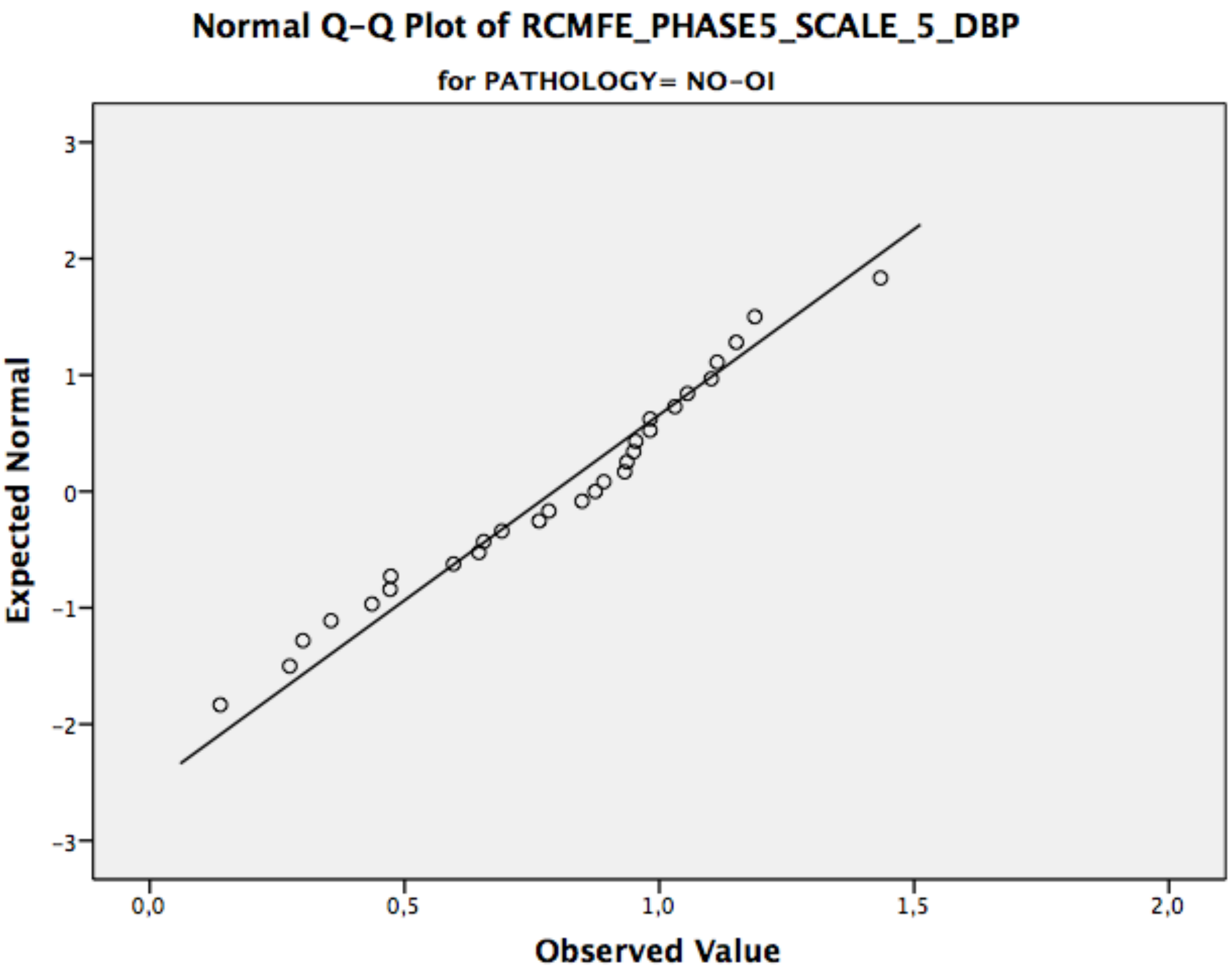

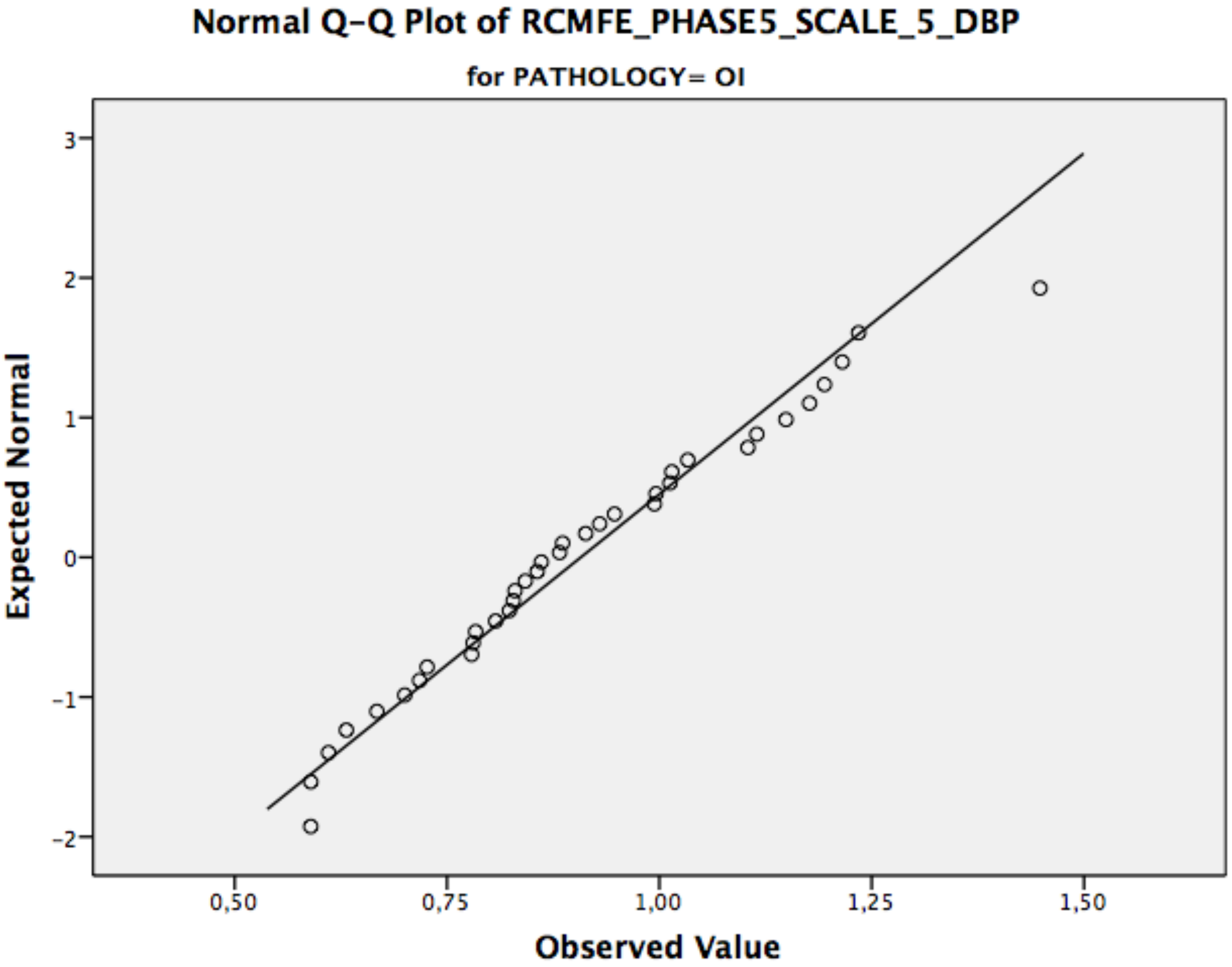

Normal Q-Q Plot of RCMFE_PHASE5_SCALE_5_DBP
for PATHOLOGY= OI
Expected Normal
Observed Value
3
2
1
0
-1
-2
0,50
0,75
1,00
1,25
1,50

Explore TPR

GENDER

Case Processing Summary

	GENDER	Cases					
		Valid		Missing		Total	
		N	Percent	N	Percent	N	Percent
RCMFE_PHASE1_SCALE_1_TPR	WOMEN	42	100,0%	0	0,0%	42	100,0%
	MEN	23	100,0%	0	0,0%	23	100,0%
RCMFE_PHASE1_SCALE_2_TPR	WOMEN	42	100,0%	0	0,0%	42	100,0%
	MEN	23	100,0%	0	0,0%	23	100,0%
RCMFE_PHASE1_SCALE_3_TPR	WOMEN	42	100,0%	0	0,0%	42	100,0%
	MEN	23	100,0%	0	0,0%	23	100,0%
RCMFE_PHASE1_SCALE_4_TPR	WOMEN	42	100,0%	0	0,0%	42	100,0%
	MEN	23	100,0%	0	0,0%	23	100,0%
RCMFE_PHASE1_SCALE_5_TPR	WOMEN	42	100,0%	0	0,0%	42	100,0%
	MEN	23	100,0%	0	0,0%	23	100,0%
RCMFE_PHASE2_SCALE_1_TPR	WOMEN	42	100,0%	0	0,0%	42	100,0%
	MEN	23	100,0%	0	0,0%	23	100,0%
RCMFE_PHASE2_SCALE_2_TPR	WOMEN	42	100,0%	0	0,0%	42	100,0%
	MEN	23	100,0%	0	0,0%	23	100,0%
RCMFE_PHASE2_SCALE_3_TPR	WOMEN	42	100,0%	0	0,0%	42	100,0%
	MEN	23	100,0%	0	0,0%	23	100,0%
RCMFE_PHASE2_SCALE_4_TPR	WOMEN	42	100,0%	0	0,0%	42	100,0%
	MEN	23	100,0%	0	0,0%	23	100,0%
RCMFE_PHASE2_SCALE_5_TPR	WOMEN	42	100,0%	0	0,0%	42	100,0%
	MEN	23	100,0%	0	0,0%	23	100,0%
RCMFE_PHASE3_SCALE_1_TPR	WOMEN	42	100,0%	0	0,0%	42	100,0%
	MEN	23	100,0%	0	0,0%	23	100,0%
RCMFE_PHASE3_SCALE_2_TPR	WOMEN	42	100,0%	0	0,0%	42	100,0%
	MEN	23	100,0%	0	0,0%	23	100,0%
RCMFE_PHASE3_SCALE_3_TPR	WOMEN	42	100,0%	0	0,0%	42	100,0%
	MEN	23	100,0%	0	0,0%	23	100,0%
RCMFE_PHASE3_SCALE_4_TPR	WOMEN	42	100,0%	0	0,0%	42	100,0%
	MEN	23	100,0%	0	0,0%	23	100,0%
RCMFE_PHASE3_SCALE_5_TPR	WOMEN	42	100,0%	0	0,0%	42	100,0%
	MEN	23	100,0%	0	0,0%	23	100,0%
RCMFE_PHASE4_SCALE_1_TPR	WOMEN	42	100,0%	0	0,0%	42	100,0%
	MEN	23	100,0%	0	0,0%	23	100,0%
RCMFE_PHASE4_SCALE_2_TPR	WOMEN	42	100,0%	0	0,0%	42	100,0%
	MEN	23	100,0%	0	0,0%	23	100,0%
RCMFE_PHASE4_SCALE_3	WOMEN	42	100,0%	0	0,0%	42	100,0%
	MEN	23	100,0%	0	0,0%	23	100,0%
RCMFE_PHASE4_SCALE_4_TPR	WOMEN	42	100,0%	0	0,0%	42	100,0%
	MEN	23	100,0%	0	0,0%	23	100,0%
RCMFE_PHASE4_SCALE_5_TPR	WOMEN	42	100,0%	0	0,0%	42	100,0%
	MEN	23	100,0%	0	0,0%	23	100,0%
RCMFE_PHASE5_SCALE_1_TPR	WOMEN	42	100,0%	0	0,0%	42	100,0%

Case Processing Summary

	GENDER	Cases					
		Valid		Missing		Total	
		N	Percent	N	Percent	N	Percent
RCMFE_PHASE5_SCALE_1_ TPR	MEN	23	100,0%	0	0,0%	23	100,0%
RCMFE_PHASE5_SCALE_2_	WOMEN	42	100,0%	0	0,0%	42	100,0%
TPR	MEN	23	100,0%	0	0,0%	23	100,0%
RCMFE_PHASE5_SCALE_3_	WOMEN	42	100,0%	0	0,0%	42	100,0%
TPR	MEN	23	100,0%	0	0,0%	23	100,0%
RCMFE_PHASE5_SCALE_4_	WOMEN	42	100,0%	0	0,0%	42	100,0%
TPR	MEN	23	100,0%	0	0,0%	23	100,0%
RCMFE_PHASE5_SCALE_5_	WOMEN	42	100,0%	0	0,0%	42	100,0%
TPR	MEN	23	100,0%	0	0,0%	23	100,0%

Tests of Normality

	GENDER	Kolmogorov-Smirnov[a]			Shapiro-Wilk		
		Statistic	df	Sig.	Statistic	df	Sig.
RCMFE_PHASE1_SCALE_1_	WOMEN	,080	42	,200[*]	,973	42	,423
TPR	MEN	,184	23	,053	,893	23	,018
RCMFE_PHASE1_SCALE_2_	WOMEN	,080	42	,200[*]	,978	42	,600
TPR	MEN	,140	23	,200[*]	,955	23	,362
RCMFE_PHASE1_SCALE_3_	WOMEN	,089	42	,200[*]	,984	42	,799
TPR	MEN	,153	23	,172	,955	23	,378
RCMFE_PHASE1_SCALE_4_	WOMEN	,094	42	,200[*]	,964	42	,208
TPR	MEN	,133	23	,200[*]	,967	23	,609
RCMFE_PHASE1_SCALE_5_	WOMEN	,064	42	,200[*]	,991	42	,986
TPR	MEN	,133	23	,200[*]	,969	23	,668
RCMFE_PHASE2_SCALE_1_	WOMEN	,090	42	,200[*]	,969	42	,299
TPR	MEN	,140	23	,200[*]	,930	23	,111
RCMFE_PHASE2_SCALE_2_	WOMEN	,071	42	,200[*]	,969	42	,302
TPR	MEN	,119	23	,200[*]	,947	23	,255
RCMFE_PHASE2_SCALE_3_	WOMEN	,136	42	,049	,957	42	,116
TPR	MEN	,143	23	,200[*]	,936	23	,151
RCMFE_PHASE2_SCALE_4_	WOMEN	,126	42	,093	,953	42	,086
TPR	MEN	,159	23	,139	,923	23	,079
RCMFE_PHASE2_SCALE_5_	WOMEN	,091	42	,200[*]	,936	42	,021
TPR	MEN	,142	23	,200[*]	,919	23	,064
RCMFE_PHASE3_SCALE_1_	WOMEN	,105	42	,200[*]	,959	42	,139
TPR	MEN	,156	23	,151	,950	23	,290
RCMFE_PHASE3_SCALE_2_	WOMEN	,067	42	,200[*]	,970	42	,321
TPR	MEN	,132	23	,200[*]	,956	23	,385
RCMFE_PHASE3_SCALE_3_	WOMEN	,094	42	,200[*]	,972	42	,370
TPR	MEN	,166	23	,100	,956	23	,393
RCMFE_PHASE3_SCALE_4_	WOMEN	,082	42	,200[*]	,981	42	,692
TPR	MEN	,178	23	,056	,958	23	,432
RCMFE_PHASE3_SCALE_5_	WOMEN	,068	42	,200[*]	,981	42	,681

TPR	MEN	,106	23	,200*	,962	23	,498
RCMFE_PHASE4_SCALE_1_	WOMEN	,242	42	,080	,716	42	,090
TPR	MEN	,118	23	,200*	,973	23	,757
RCMFE_PHASE4_SCALE_2_	WOMEN	,219	42	,088	,777	42	,090
TPR	MEN	,098	23	,200*	,928	23	,099
RCMFE_PHASE4_SCALE_3	WOMEN	,236	42	,080	,795	42	,050
	MEN	,120	23	,200*	,934	23	,133
RCMFE_PHASE4_SCALE_4_	WOMEN	,197	42	,045	,879	42	,055
TPR	MEN	,165	23	,103	,916	23	,055
RCMFE_PHASE4_SCALE_5_	WOMEN	,224	42	,030	,823	42	,070
TPR	MEN	,156	23	,155	,898	23	,023
RCMFE_PHASE5_SCALE_1_	WOMEN	,119	42	,147	,965	42	,218
TPR	MEN	,101	23	,200*	,968	23	,647

Tests of Normality

	GENDER	Kolmogorov-Smirnov[a]			Shapiro-Wilk		
		Statistic	df	Sig.	Statistic	df	Sig.
RCMFE_PHASE5_SCALE_2_	WOMEN	,063	42	,200*	,974	42	,440
TPR	MEN	,113	23	,200	,937	23	,154
RCMFE_PHASE5_SCALE_3_	WOMEN	,061	42	,200*	,982	42	,735
TPR	MEN	,118	23	,200*	,939	23	,175
RCMFE_PHASE5_SCALE_4_	WOMEN	,120	42	,135*	,975	42	,472
TPR	MEN	,129	23	,200	,928	23	,098
RCMFE_PHASE5_SCALE_5_	WOMEN	,102	42	,200*	,961	42	,155
TPR	MEN	,146	23	,200*	,906	23	,053

*. This is a lower bound of the true significance.

a. Lilliefors Significance Correction

RCMFE_PHASE1_SCALE_1_TPR

Normal Q-Q Plots

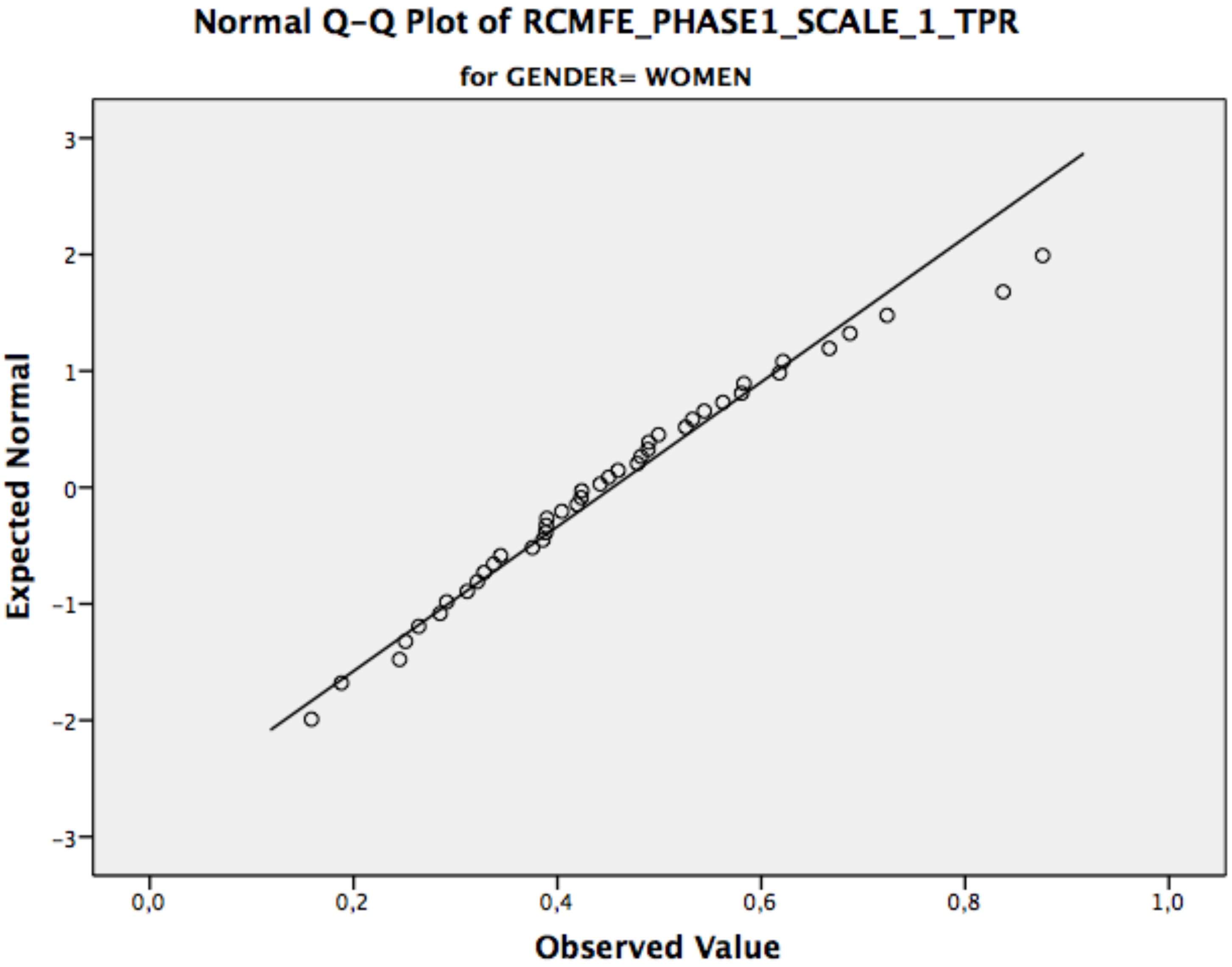

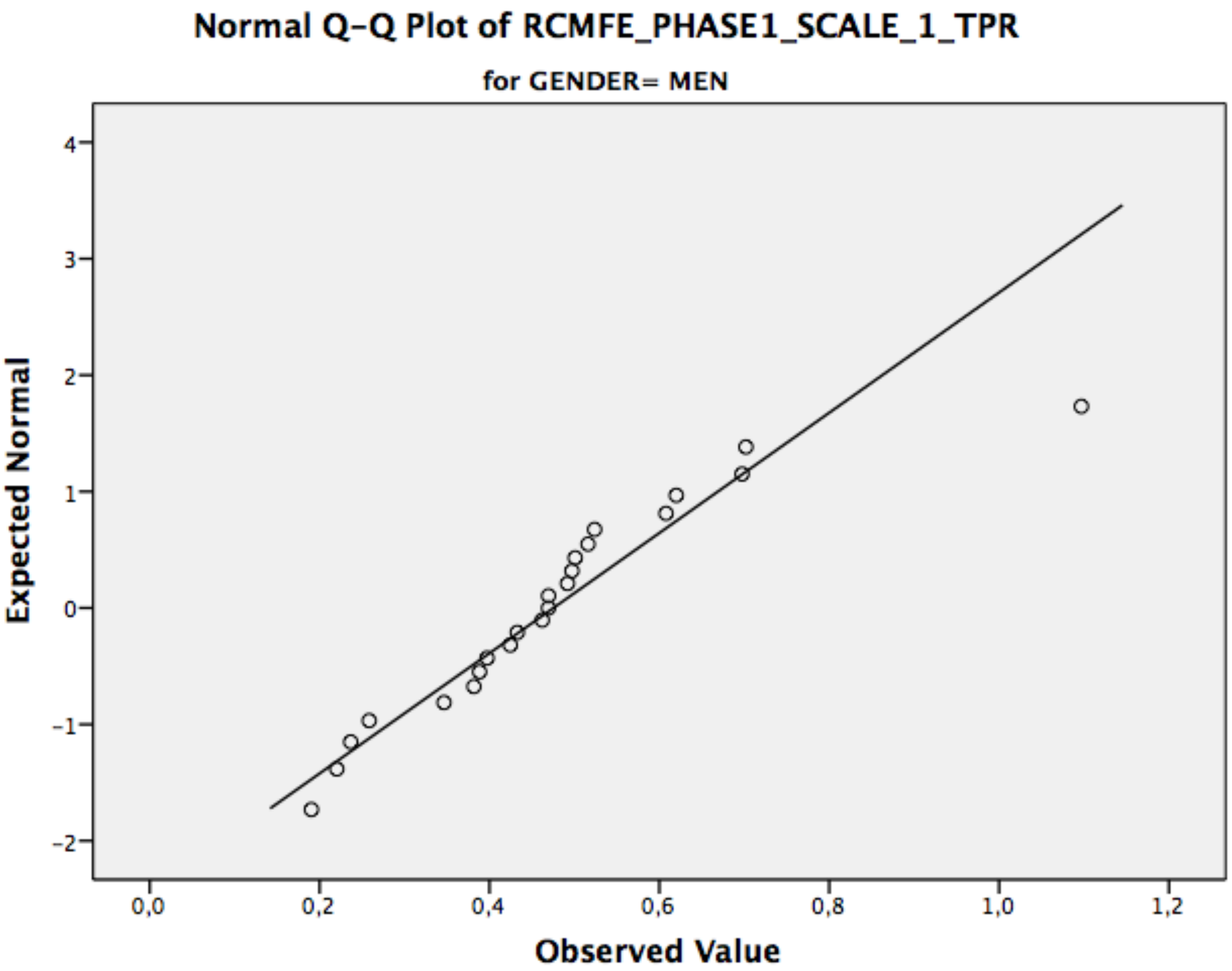
Normal Q-Q Plot of RCMFE_PHASE1_SCALE_1_TPR
for GENDER= MEN
Expected Normal
Observed Value
4
3
2
1
0
−1
−2
0,0
0,2
0,4
0,6
0,8
1,0
1,2

RCMFE_PHASE1_SCALE_2_TPR

Normal Q-Q Plots

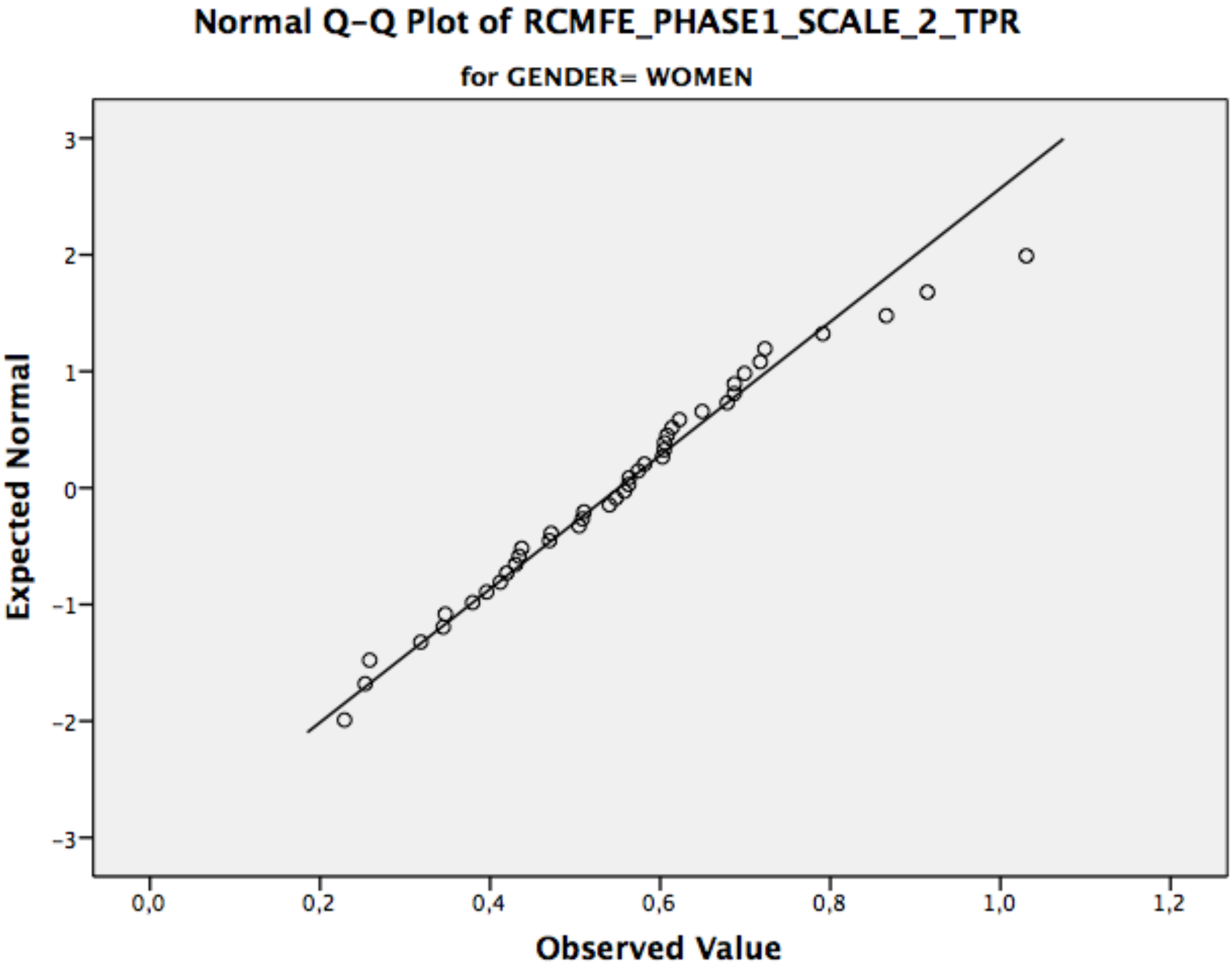

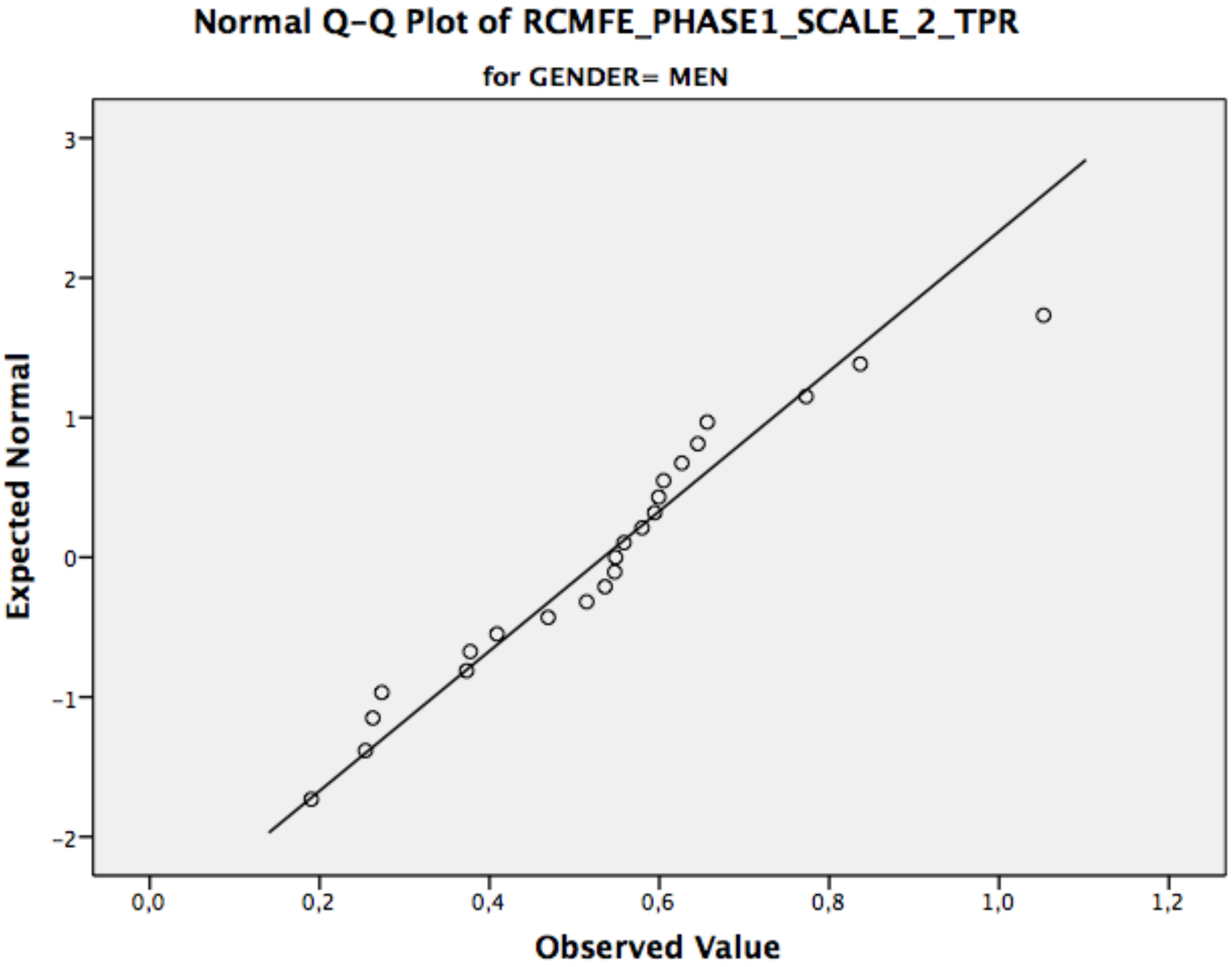
Normal Q-Q Plot of RCMFE_PHASE1_SCALE_2_TPR
for GENDER= MEN
Expected Normal
3
2
1
0
-1
-2
0,0
0,2
0,4
0,6
0,8
1,0
1,2
Observed Value

RCMFE_PHASE1_SCALE_3_TPR

Normal Q-Q Plots

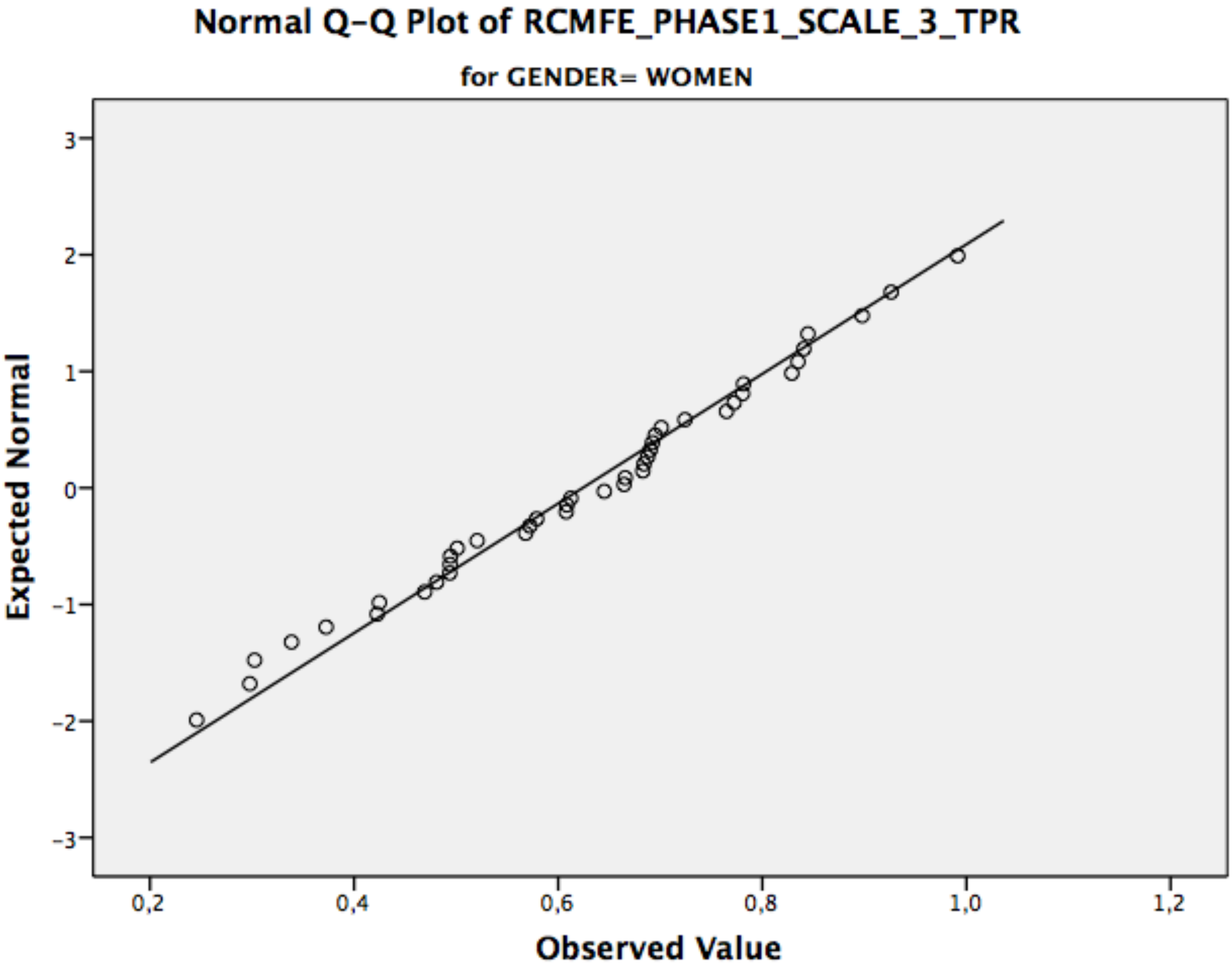

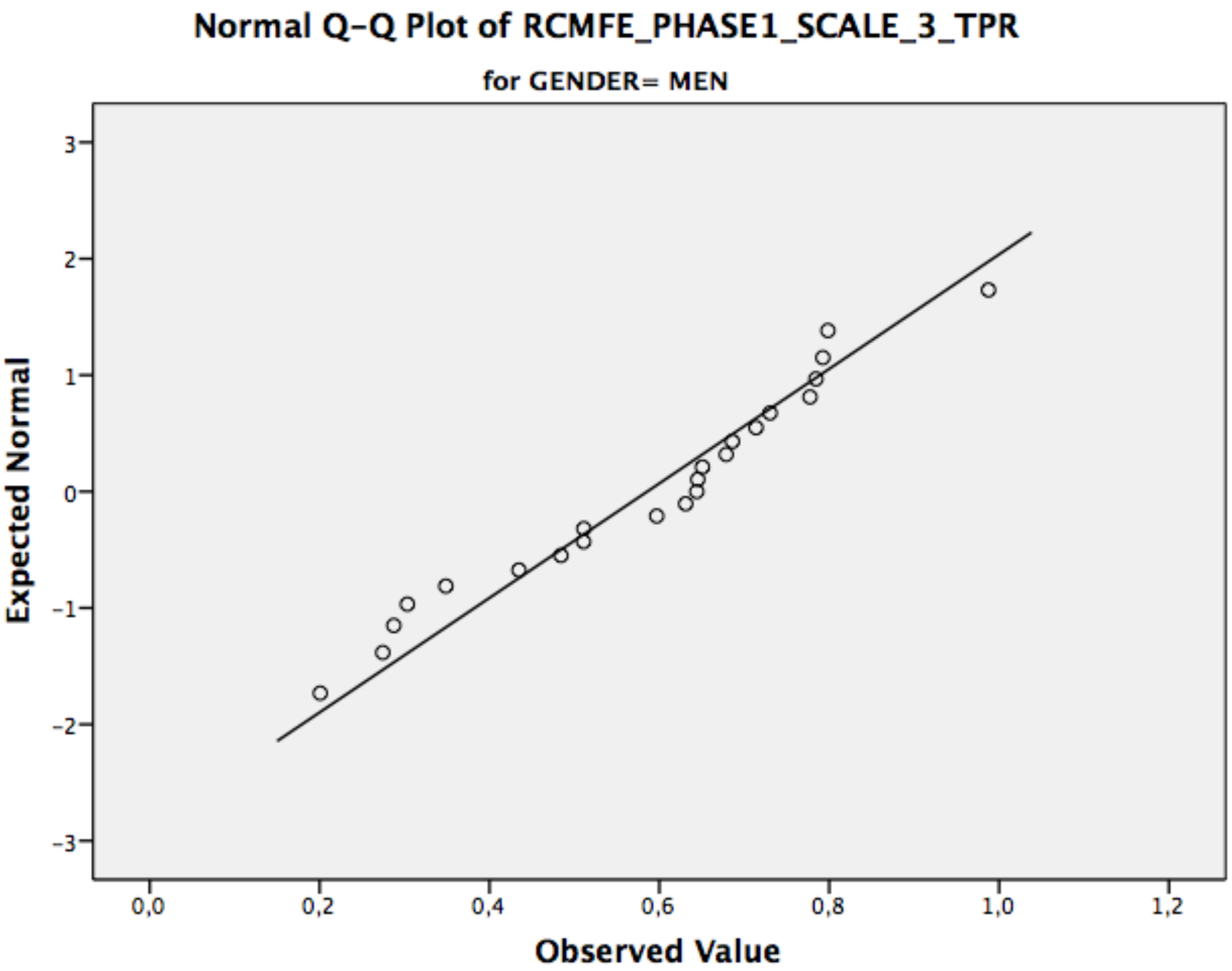

Normal Q-Q Plot of RCMFE_PHASE1_SCALE_3_TPR
for GENDER= MEN
Expected Normal
3
2
1
0
-1
-2
-3
0,0
0,2
0,4
0,6
0,8
1,0
1,2
Observed Value

RCMFE_PHASE1_SCALE_4_TPR

Normal Q-Q Plots

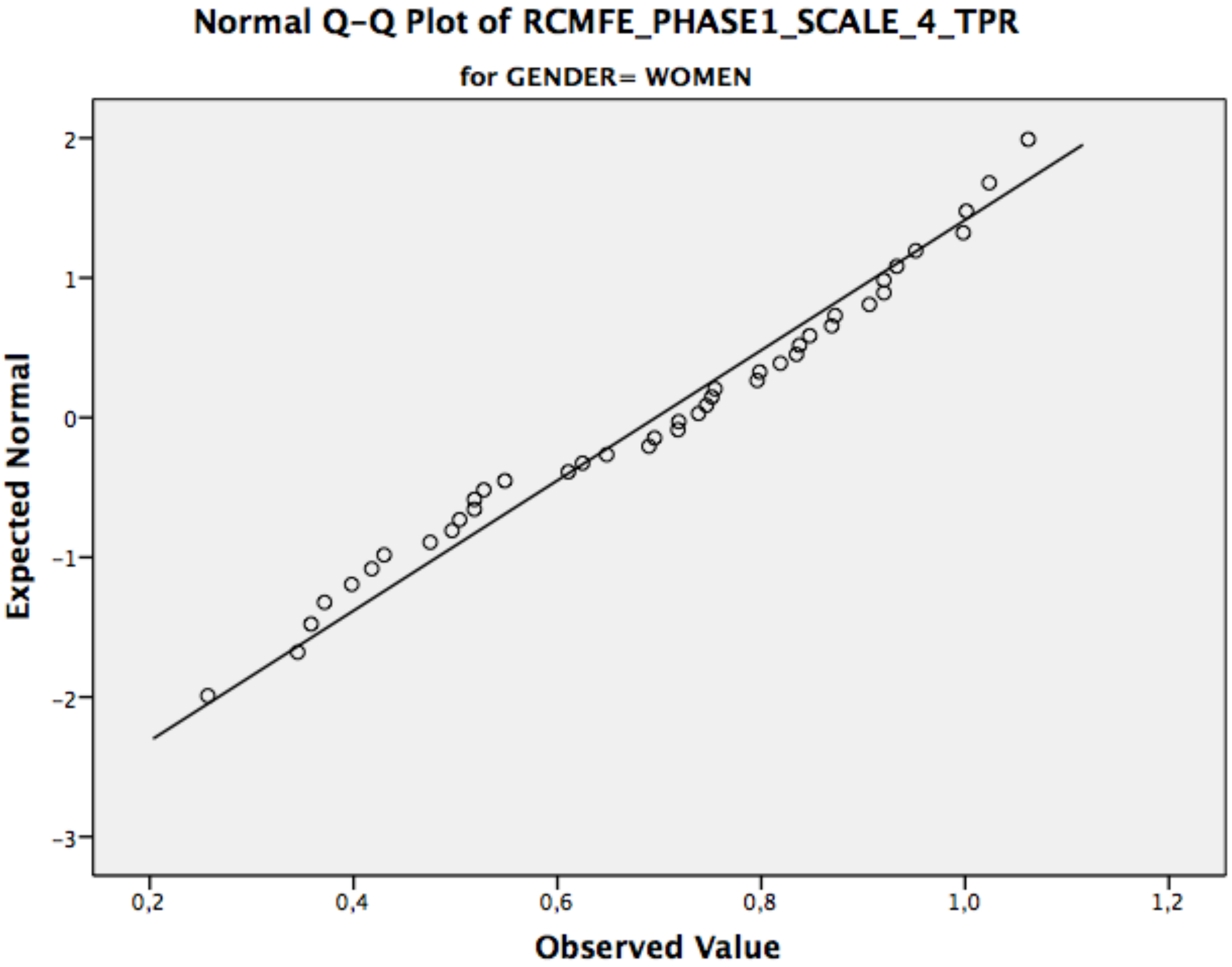

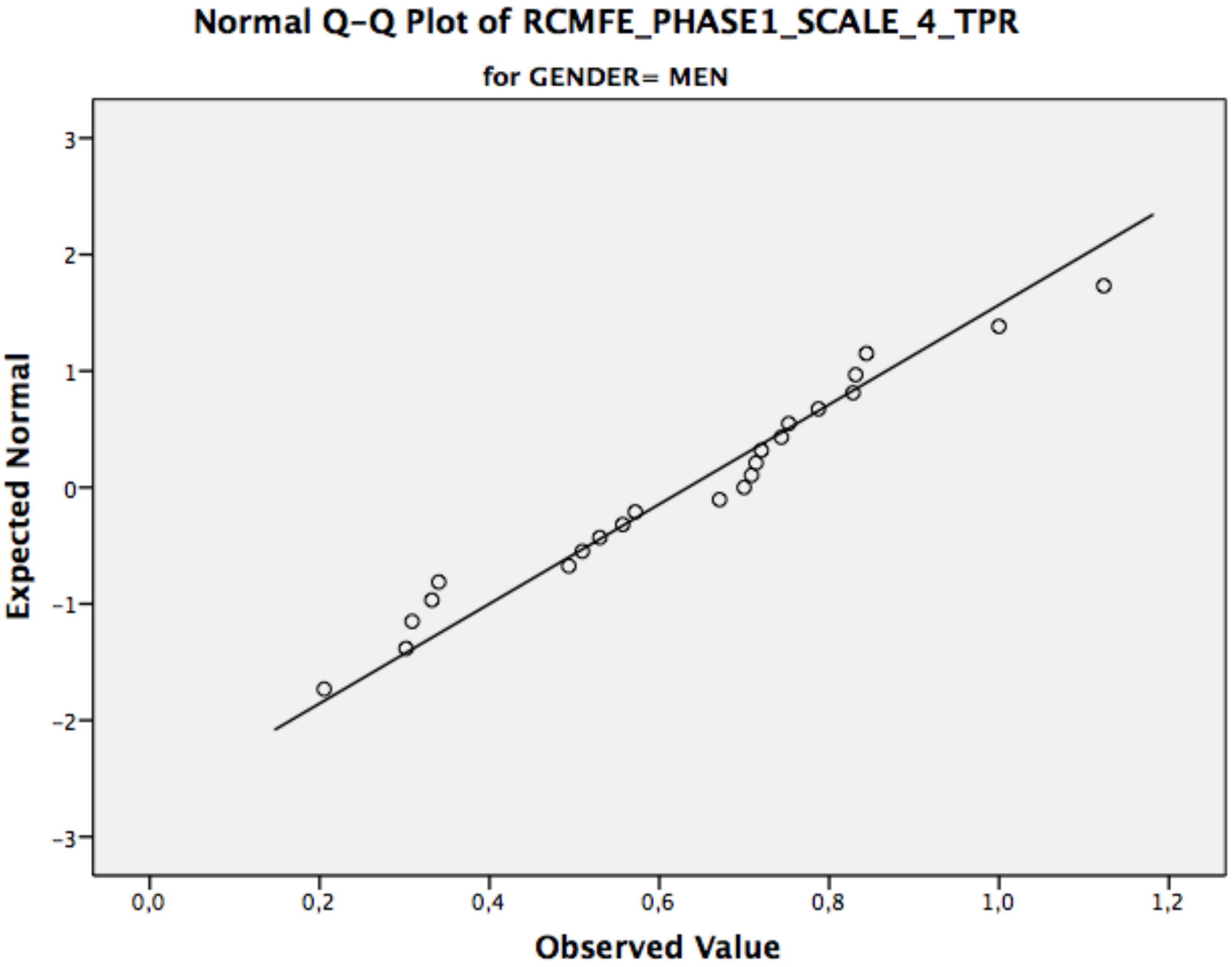

Normal Q-Q Plot of RCMFE_PHASE1_SCALE_4_TPR
for GENDER= MEN
Expected Normal
Observed Value
3
2
1
0
-1
-2
-3
0,0
0,2
0,4
0,6
0,8
1,0
1,2

RCMFE_PHASE1_SCALE_5_TPR

Normal Q-Q Plots

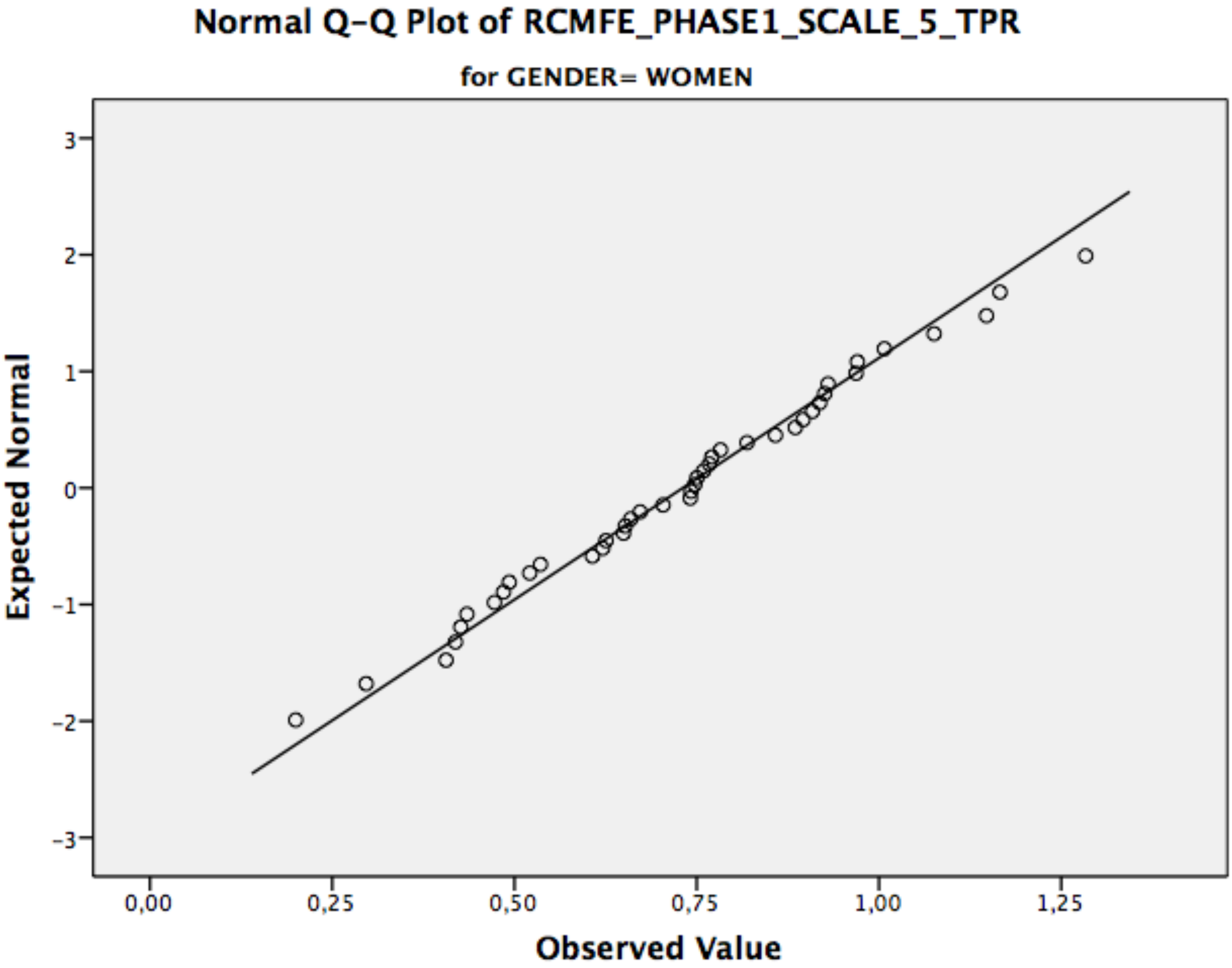

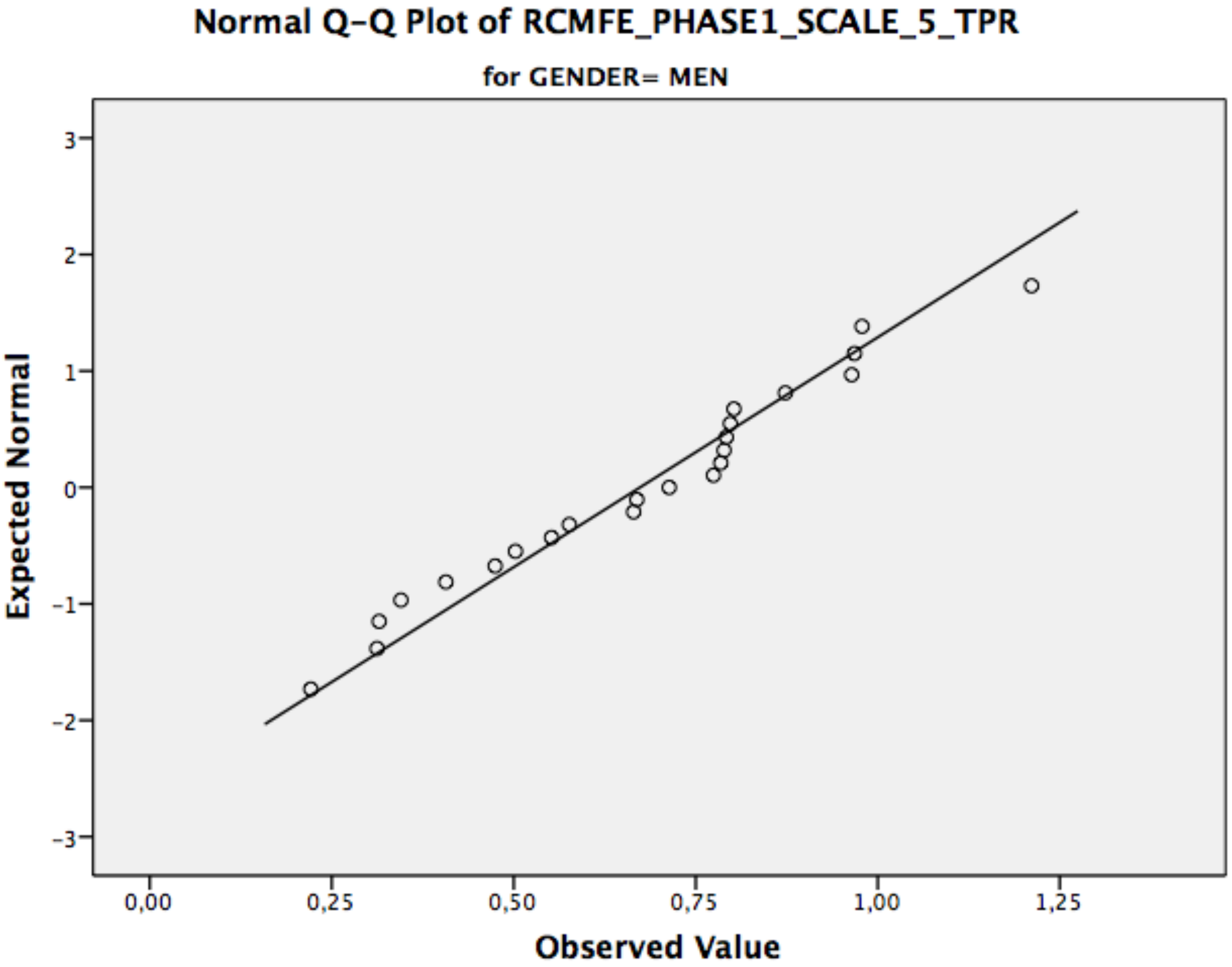

Normal Q-Q Plot of RCMFE_PHASE1_SCALE_5_TPR
for GENDER= MEN
Expected Normal
Observed Value
3
2
1
0
−1
−2
−3
0,00
0,25
0,50
0,75
1,00
1,25

RCMFE_PHASE2_SCALE_1_TPR

Normal Q-Q Plots

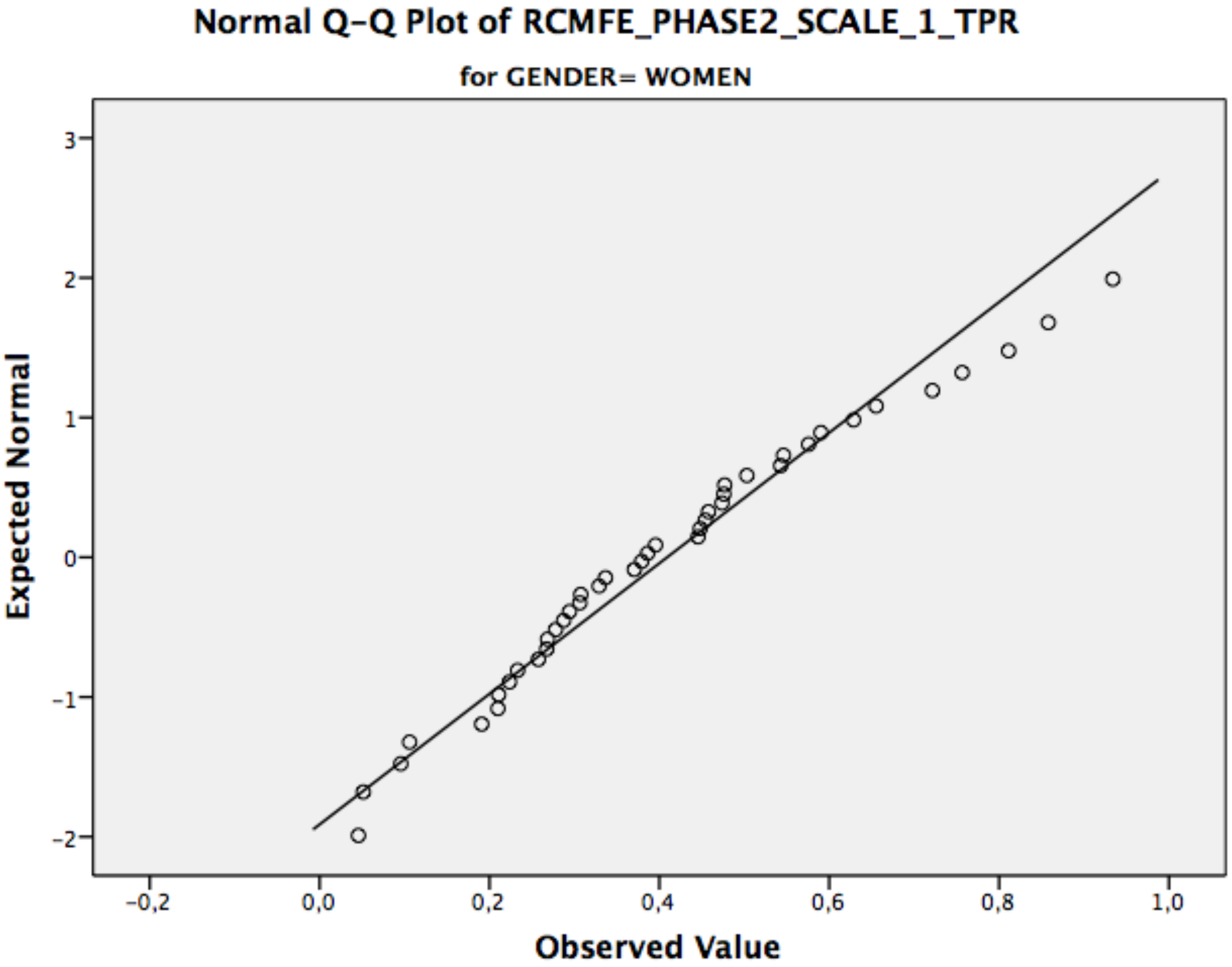

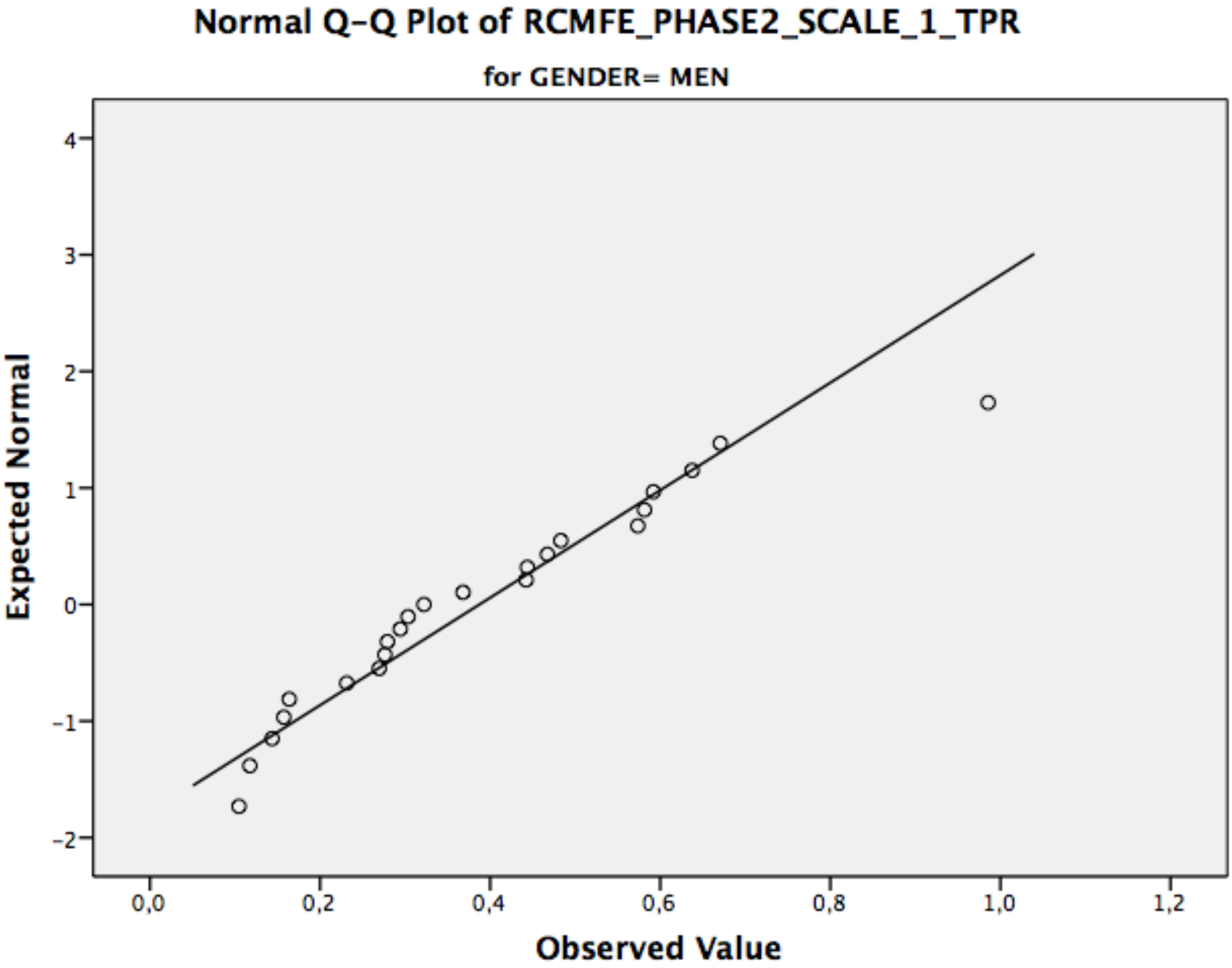

Normal Q-Q Plot of RCMFE_PHASE2_SCALE_1_TPR
for GENDER= MEN
Expected Normal
Observed Value
4
3
2
1
0
-1
-2
0,0
0,2
0,4
0,6
0,8
1,0
1,2

RCMFE_PHASE2_SCALE_2_TPR

Normal Q-Q Plots

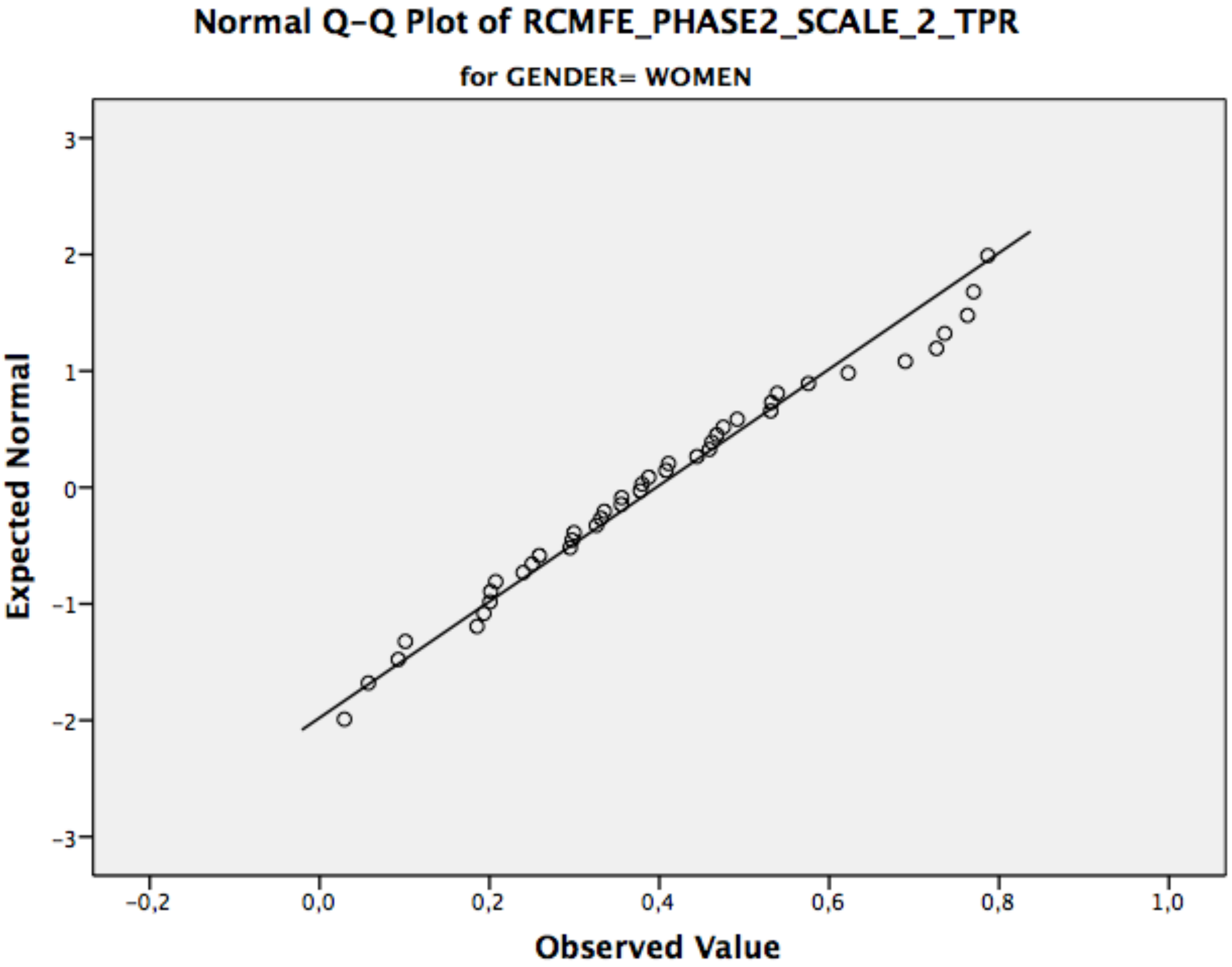

Normal Q-Q Plot of RCMFE_PHASE2_SCALE_2_TPR

for GENDER= MEN

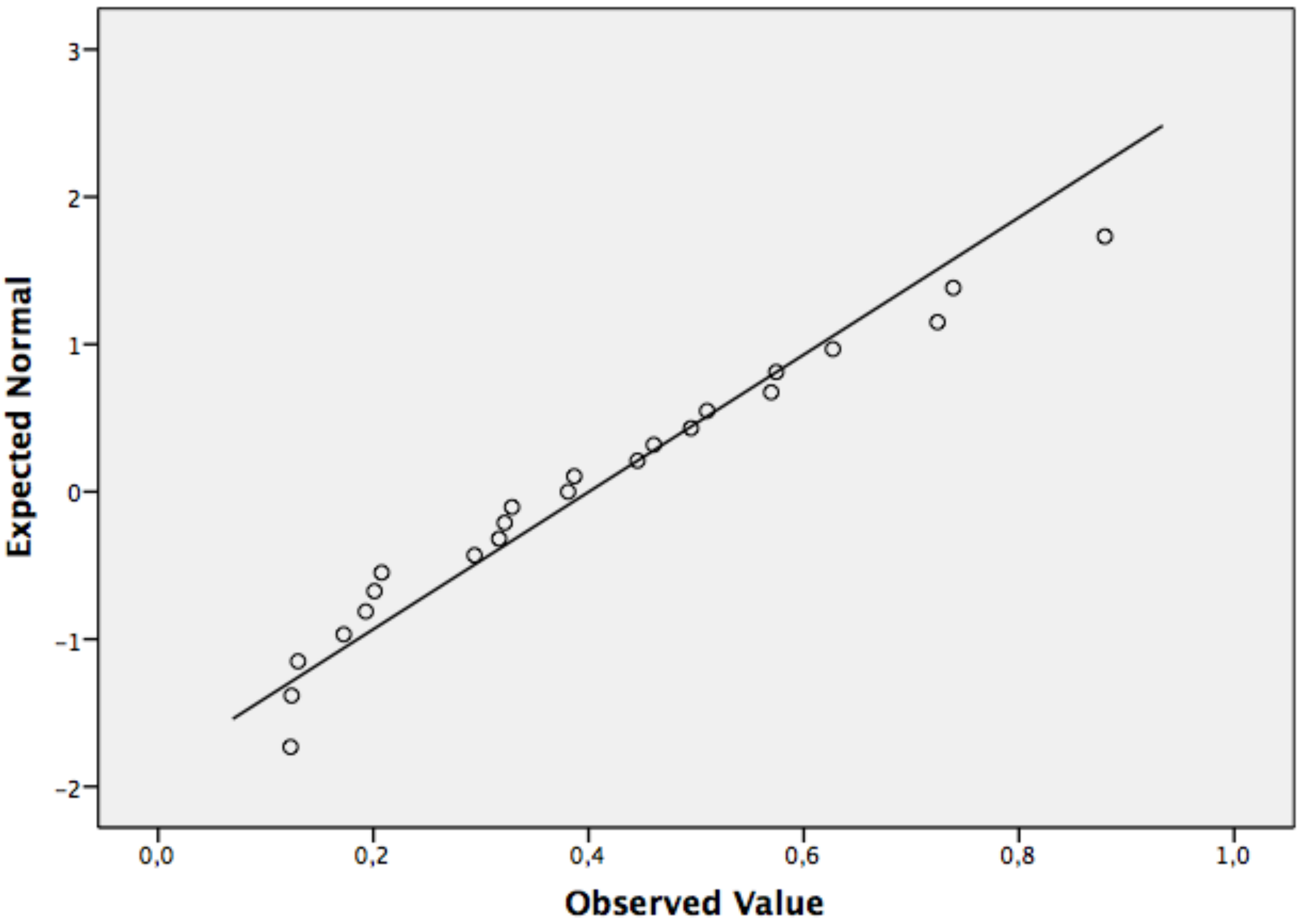

RCMFE_PHASE2_SCALE_3_TPR

Normal Q-Q Plots

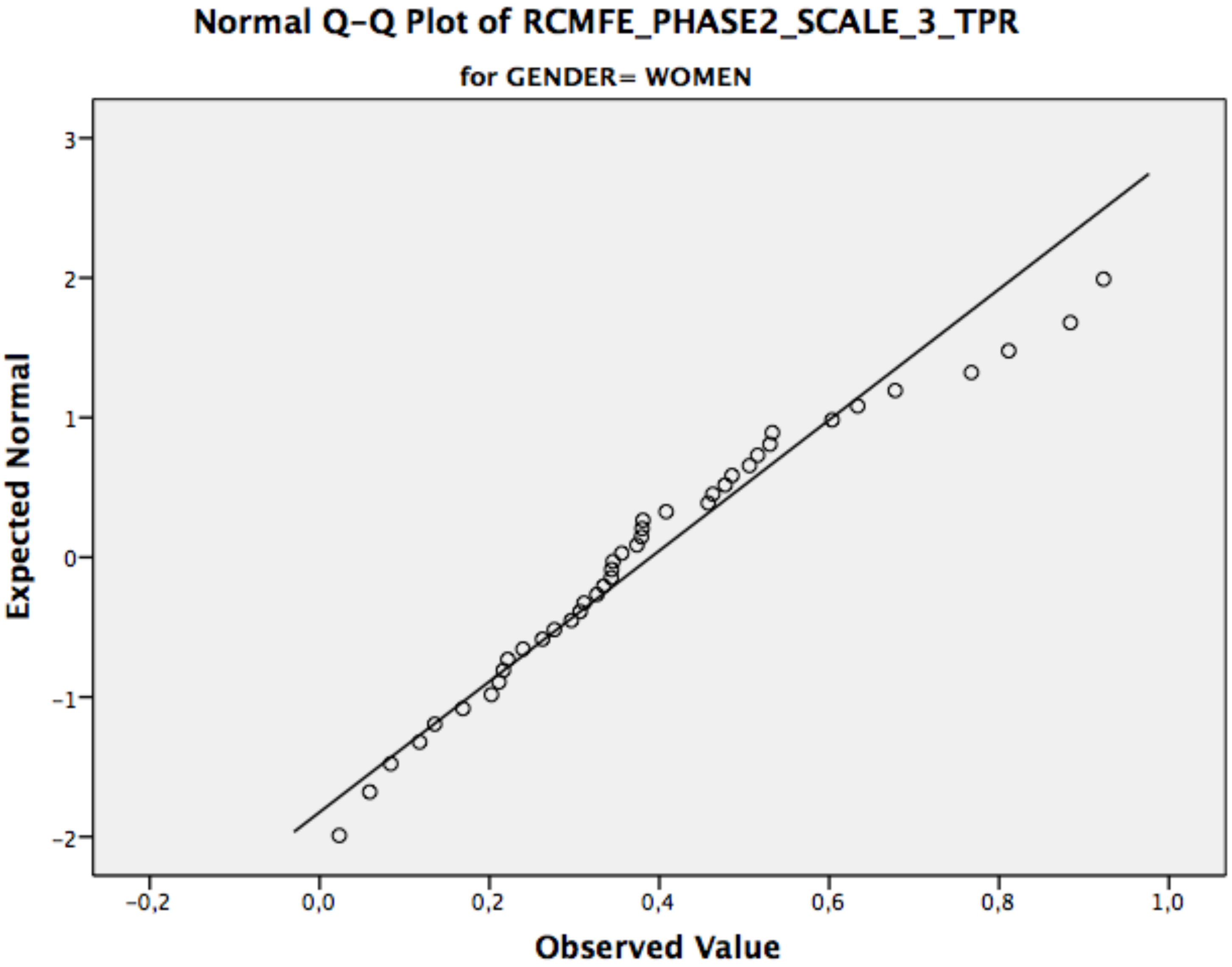

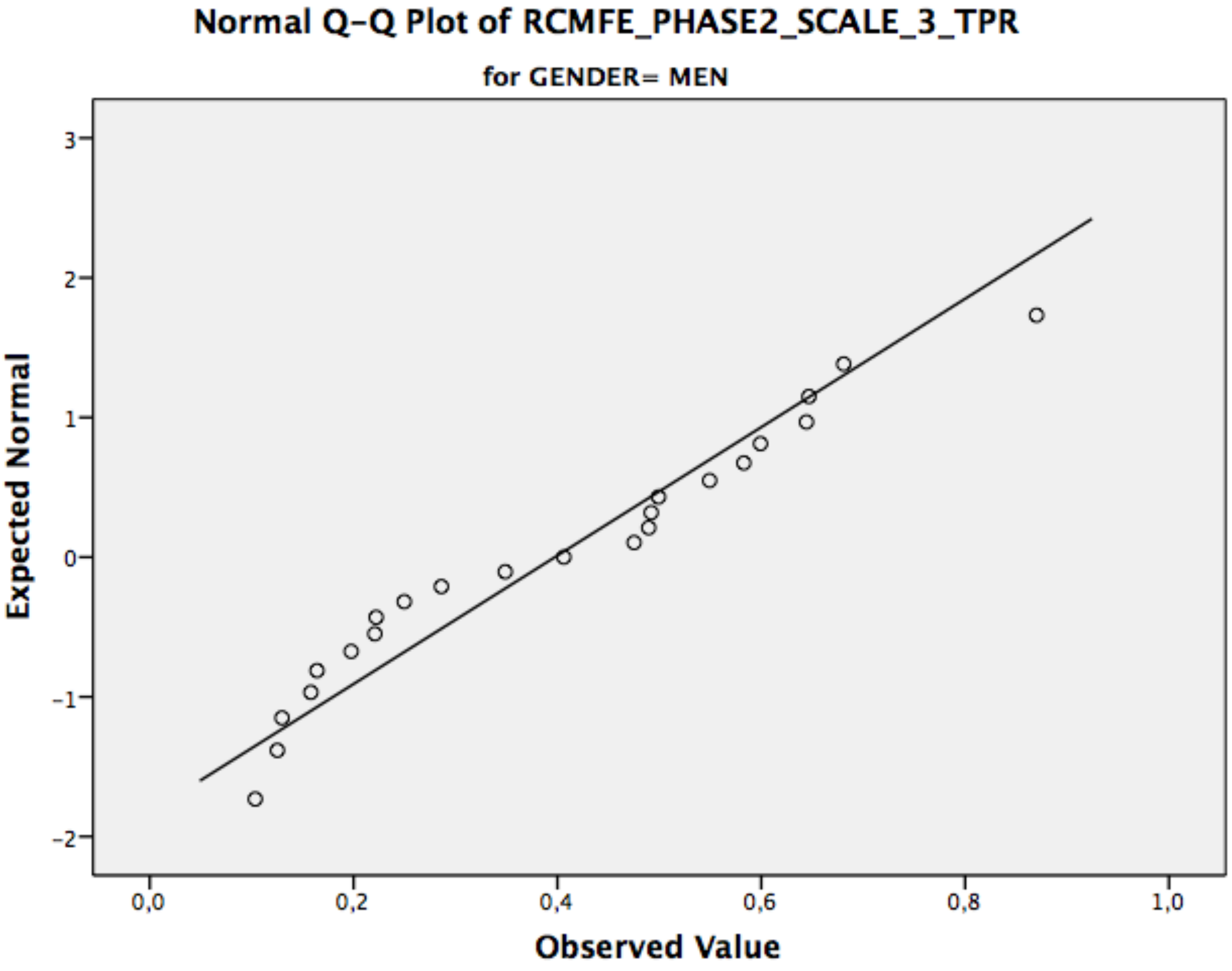

Normal Q-Q Plot of RCMFE_PHASE2_SCALE_3_TPR
for GENDER= MEN
Expected Normal
3
2
1
0
-1
-2
0,0
0,2
0,4
0,6
0,8
1,0
Observed Value

Normal Q-Q Plots

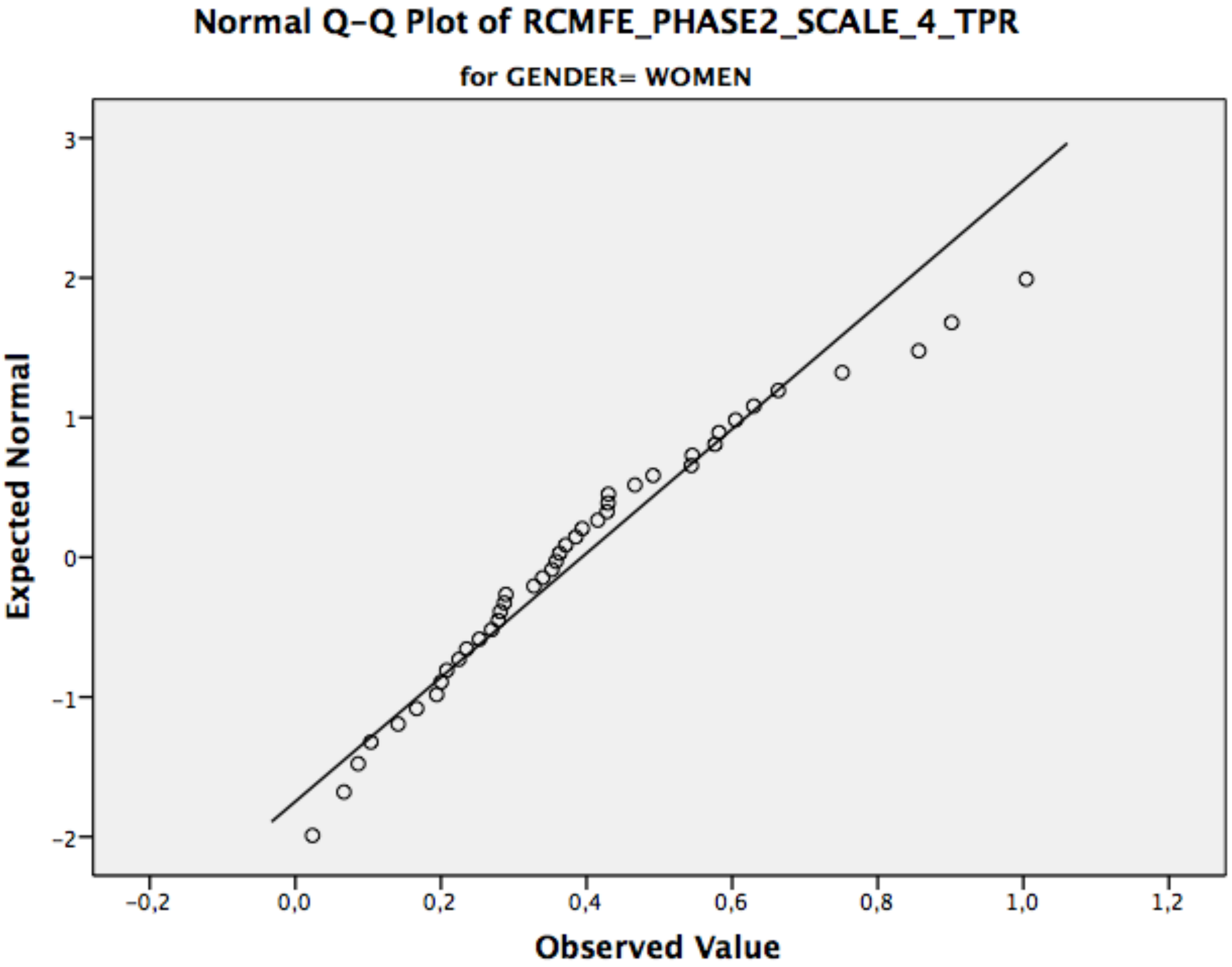

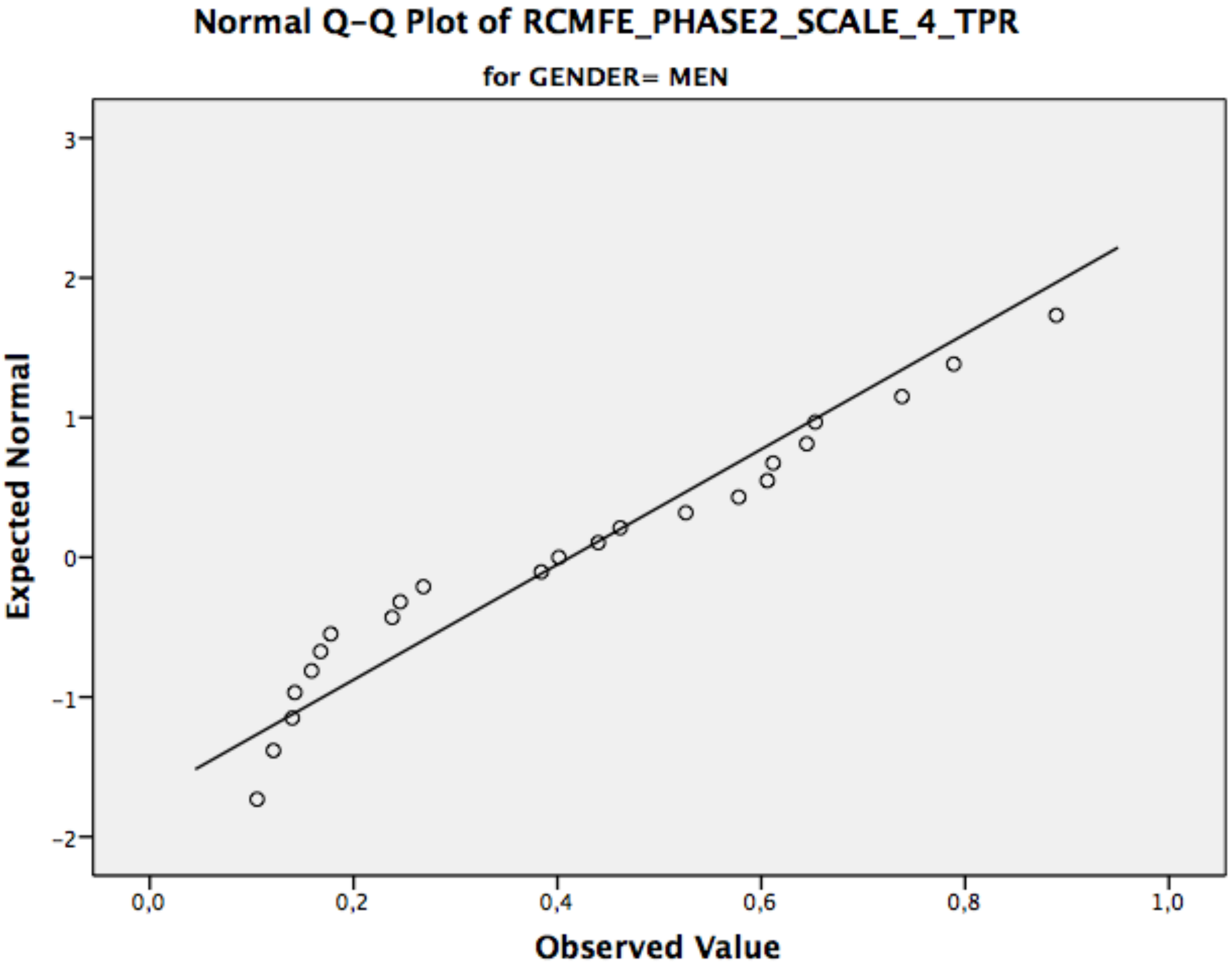
Normal Q-Q Plot of RCMFE_PHASE2_SCALE_4_TPR
for GENDER= MEN
Expected Normal
Observed Value
3
2
1
0
−1
−2
0,0
0,2
0,4
0,6
0,8
1,0

RCMFE_PHASE2_SCALE_5_TPR

Normal Q-Q Plots

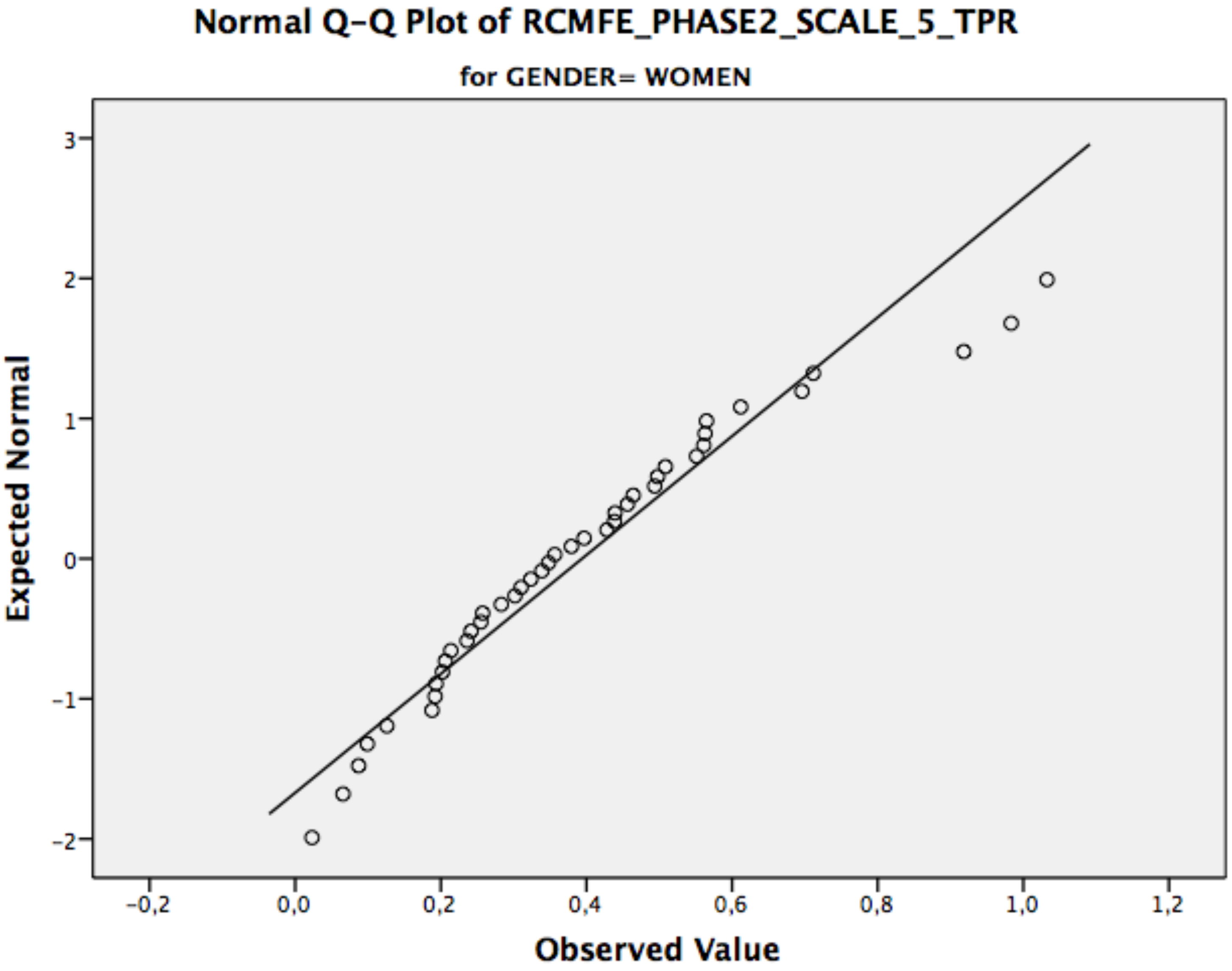

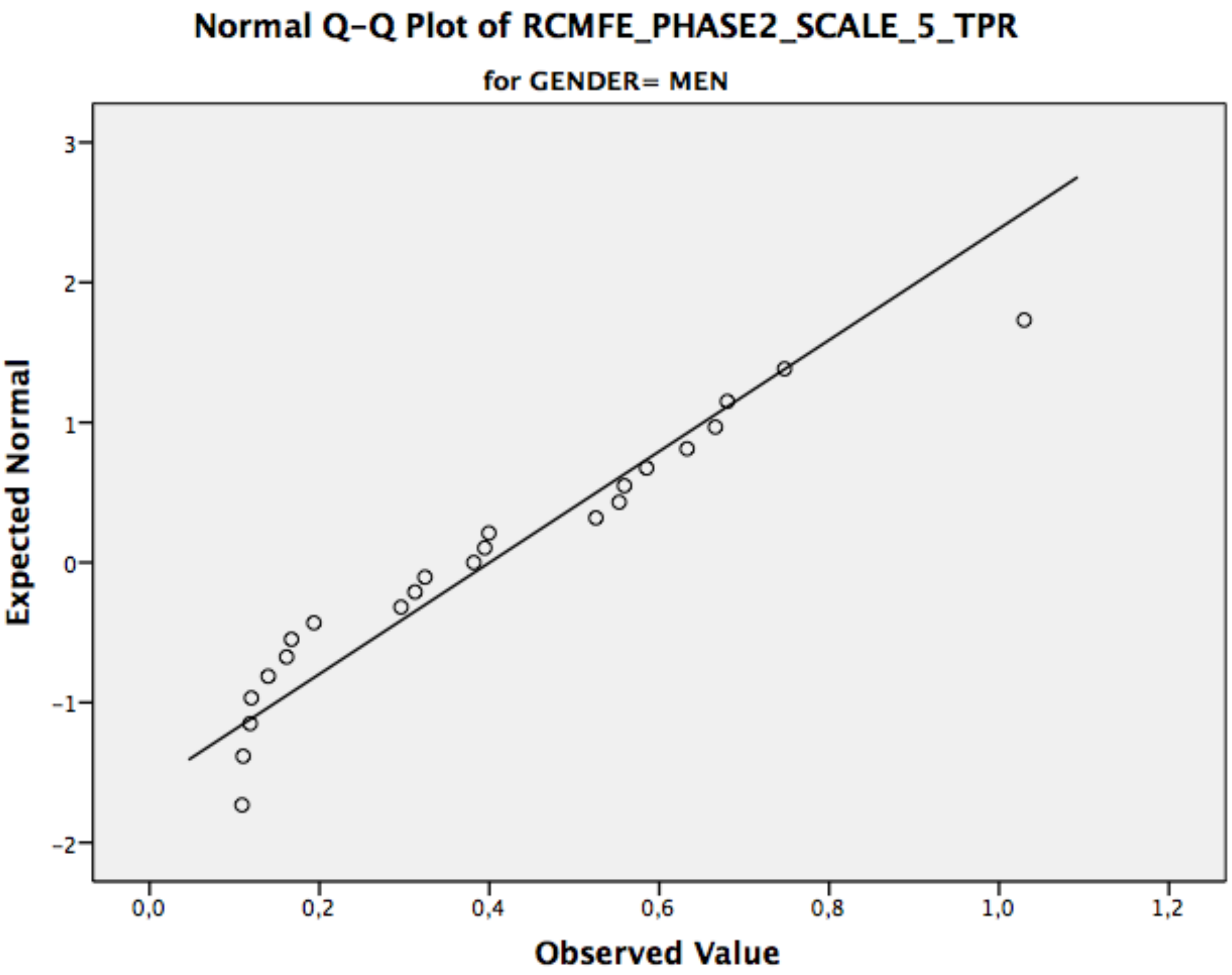

Normal Q-Q Plot of RCMFE_PHASE2_SCALE_5_TPR
for GENDER= MEN
Expected Normal
Observed Value
3
2
1
0
-1
-2
0,0
0,2
0,4
0,6
0,8
1,0
1,2

Normal Q-Q Plots

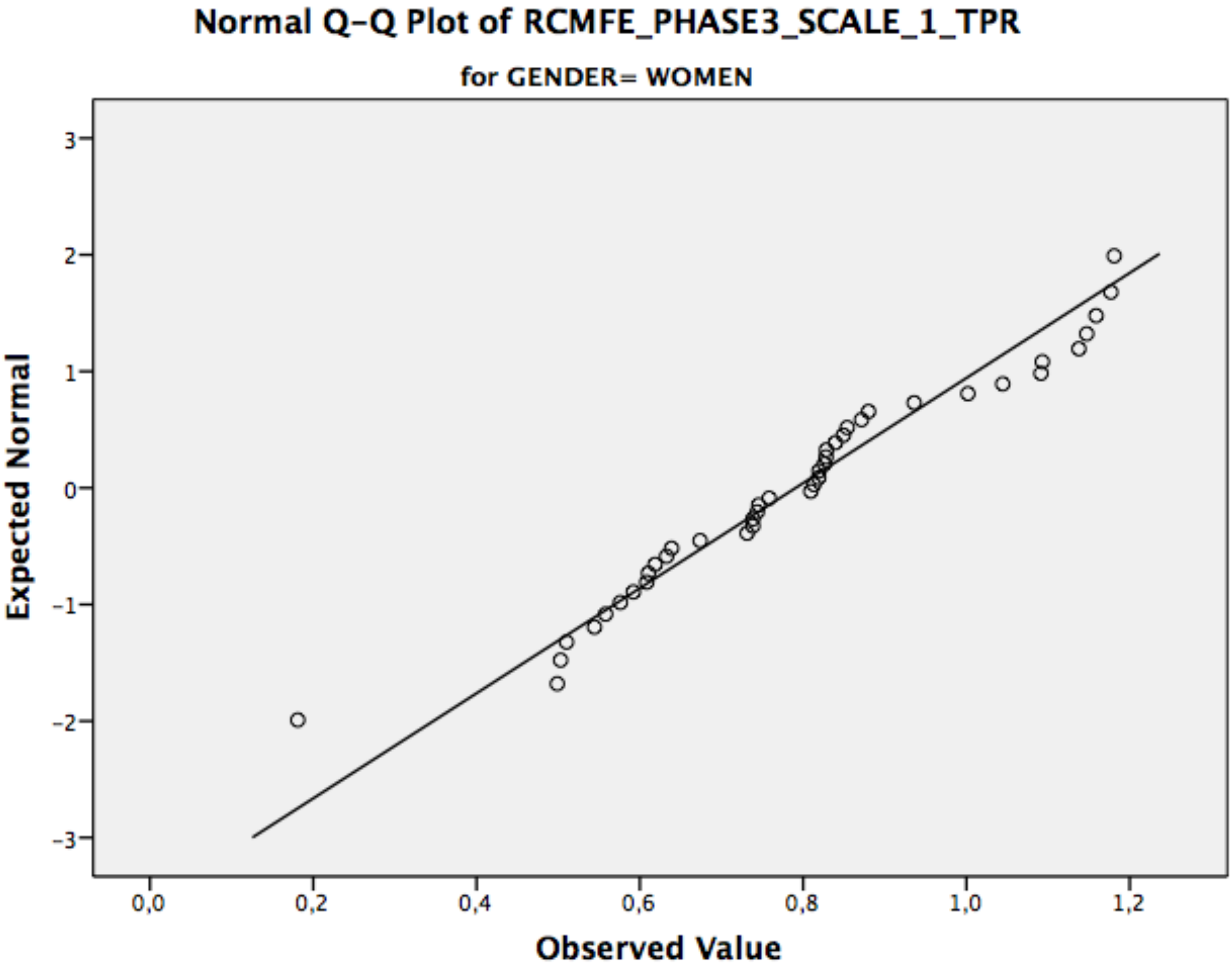

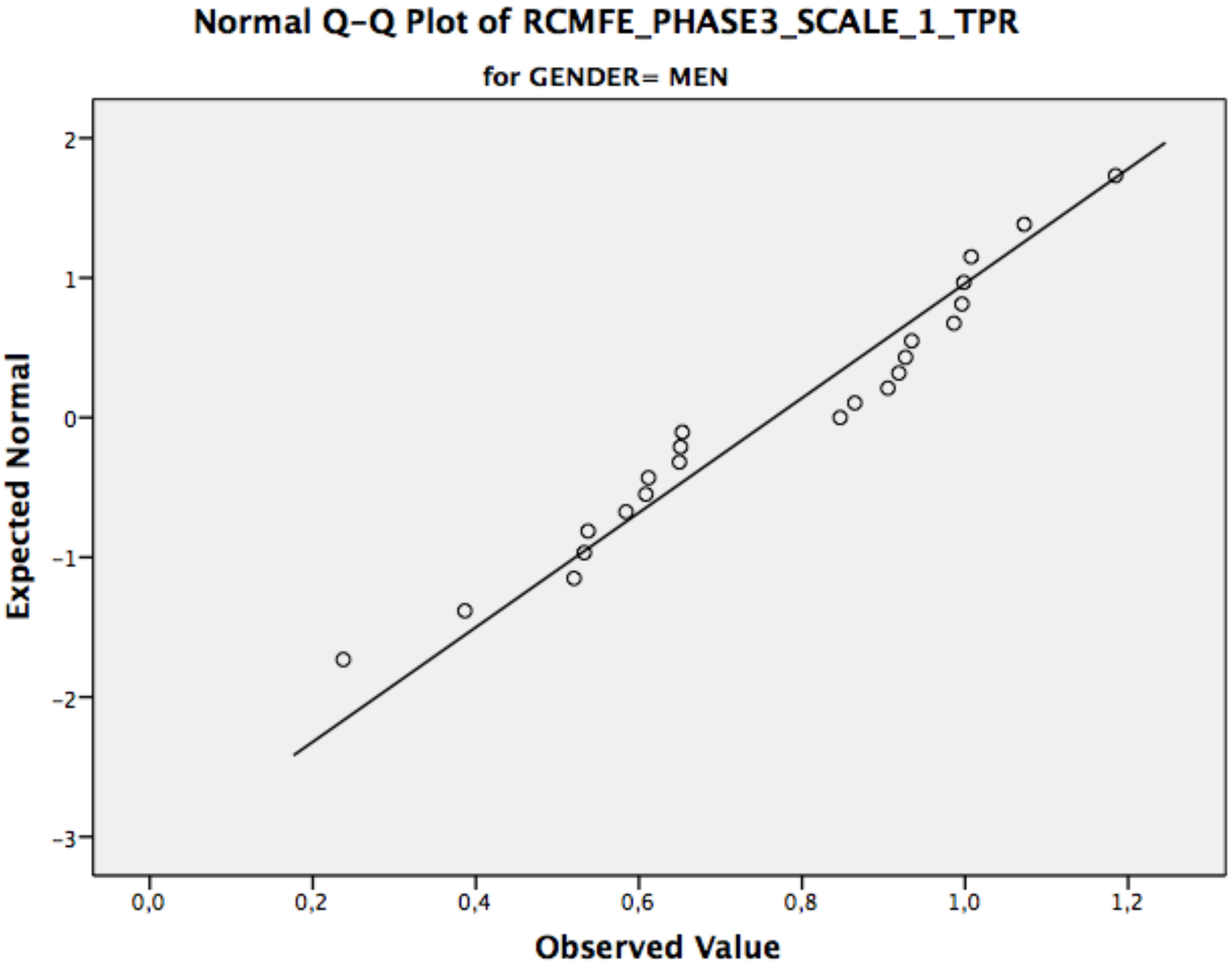

Normal Q-Q Plot of RCMFE_PHASE3_SCALE_1_TPR
for GENDER= MEN
Expected Normal
Observed Value
2
1
0
-1
-2
-3
0,0
0,2
0,4
0,6
0,8
1,0
1,2

RCMFE_PHASE3_SCALE_2_TPR

Normal Q-Q Plots

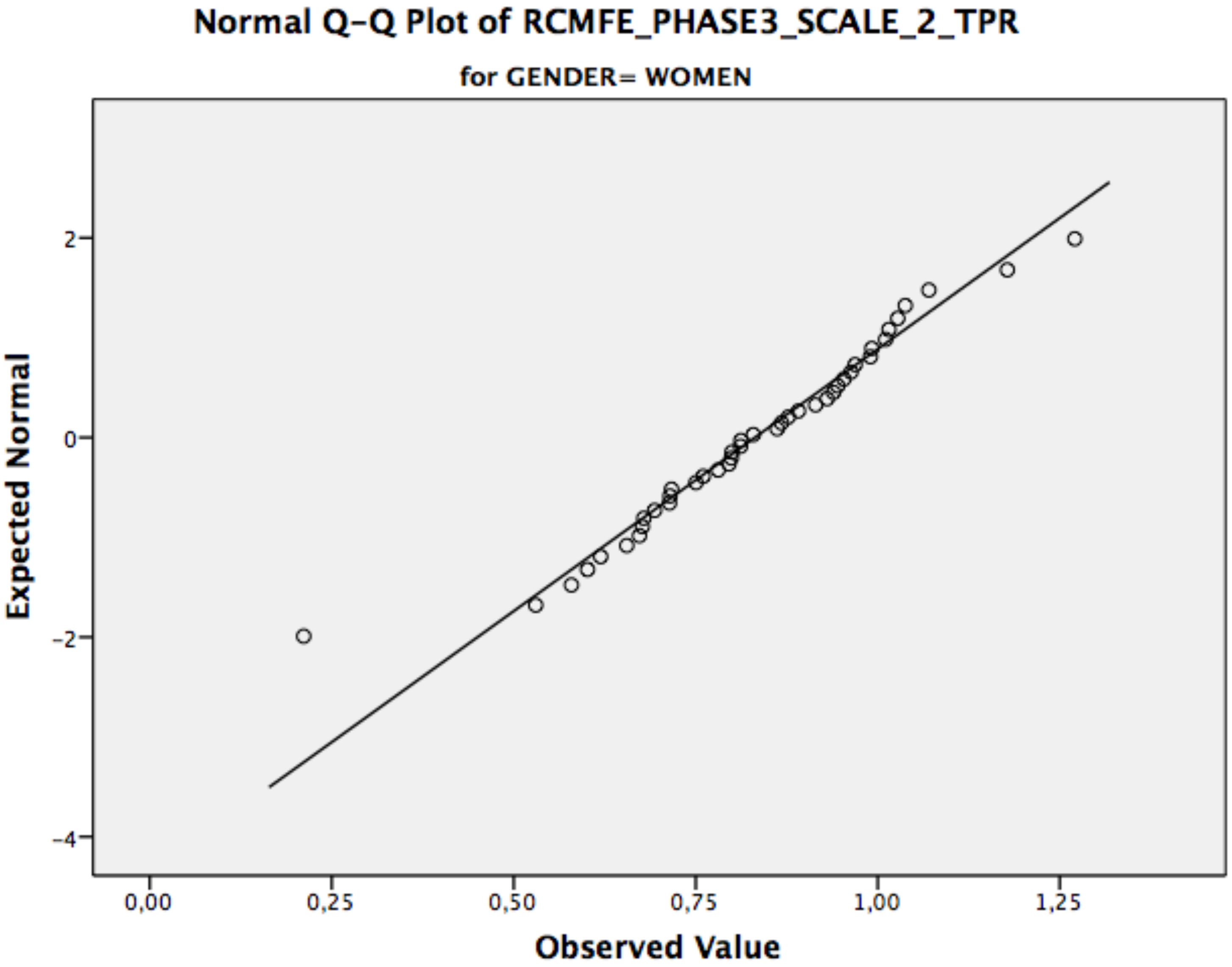

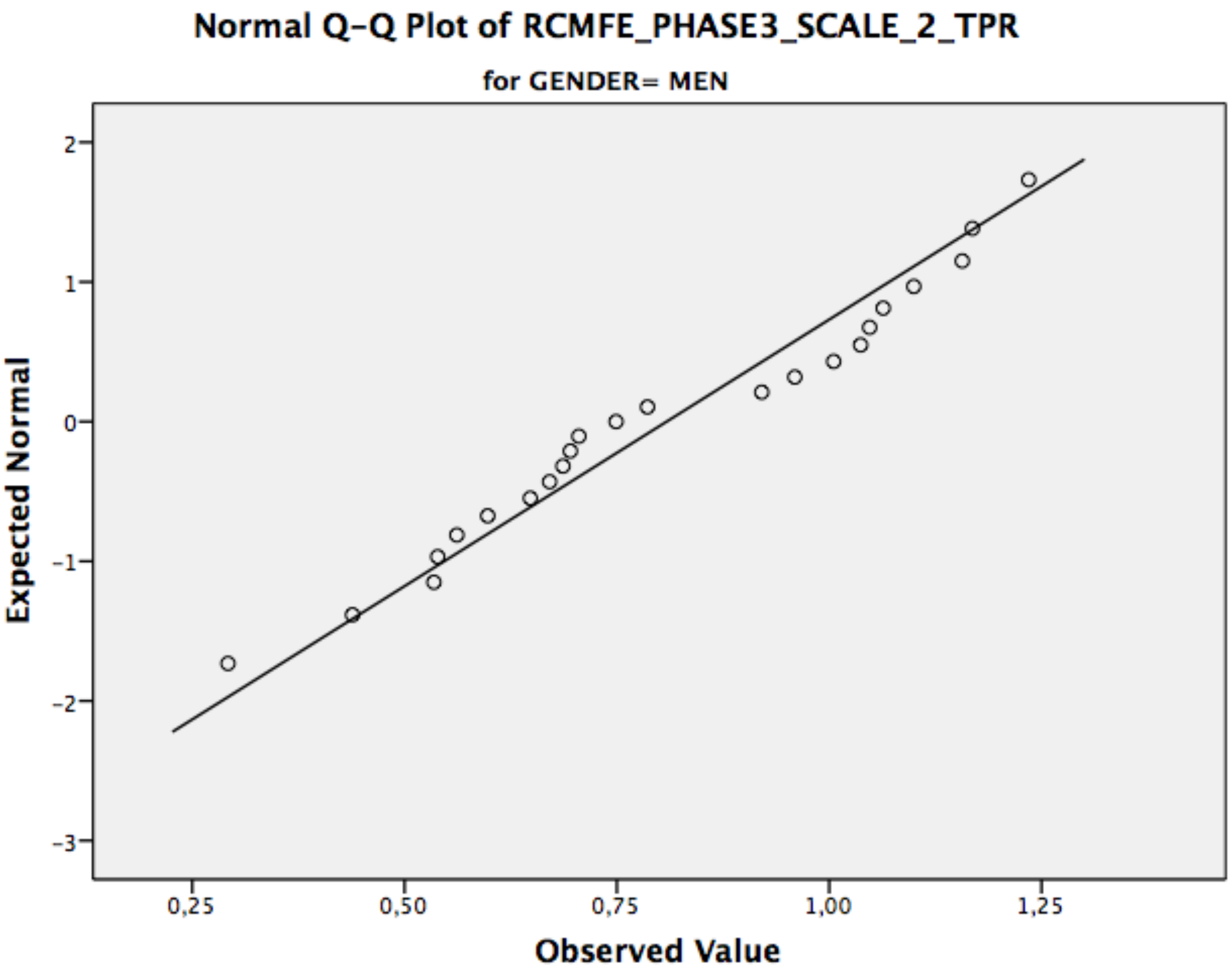

Normal Q-Q Plot of RCMFE_PHASE3_SCALE_2_TPR
for GENDER= MEN
Expected Normal
2
1
0
-1
-2
-3
0,25
0,50
0,75
1,00
1,25
Observed Value

RCMFE_PHASE3_SCALE_3_TPR

Normal Q-Q Plots

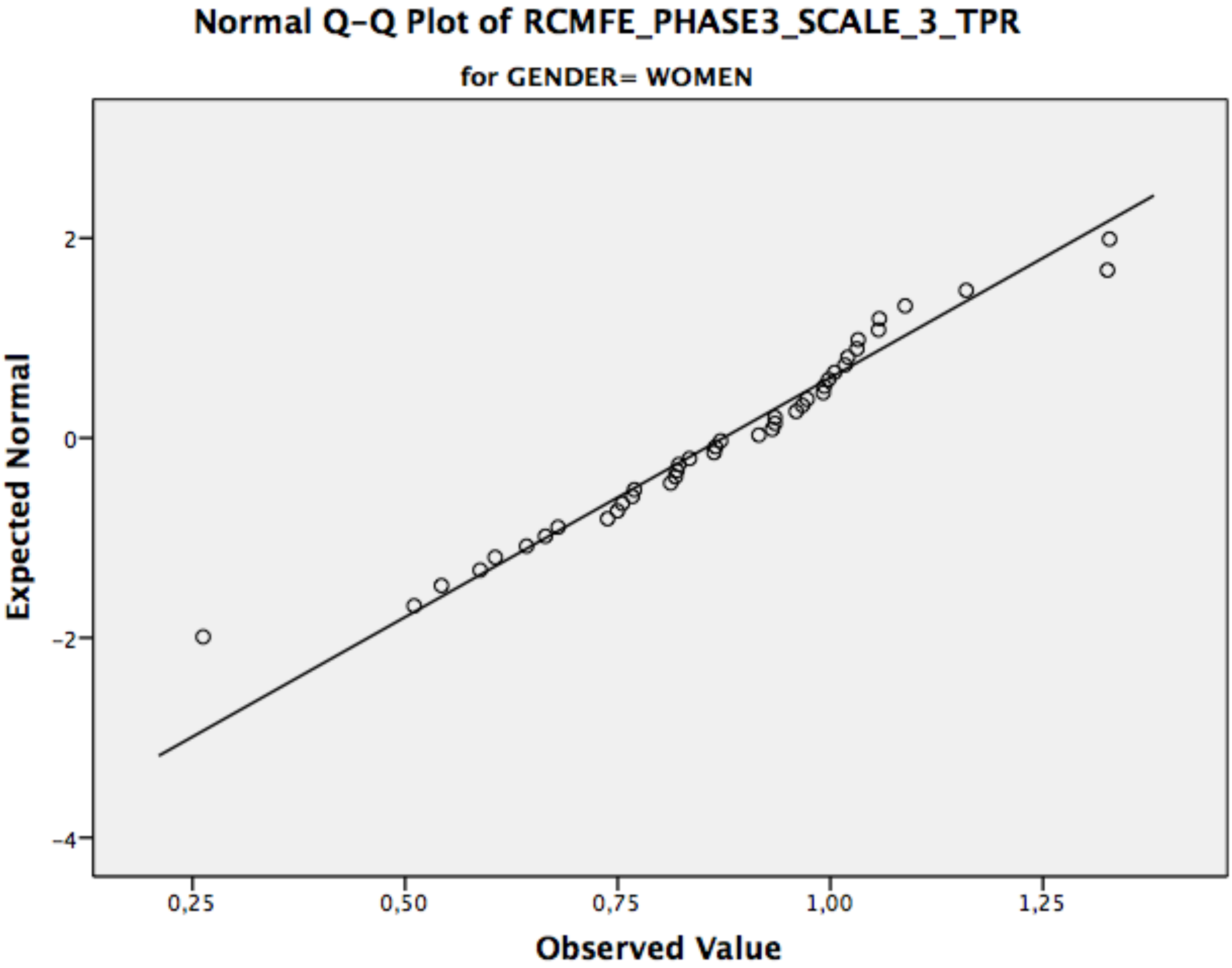

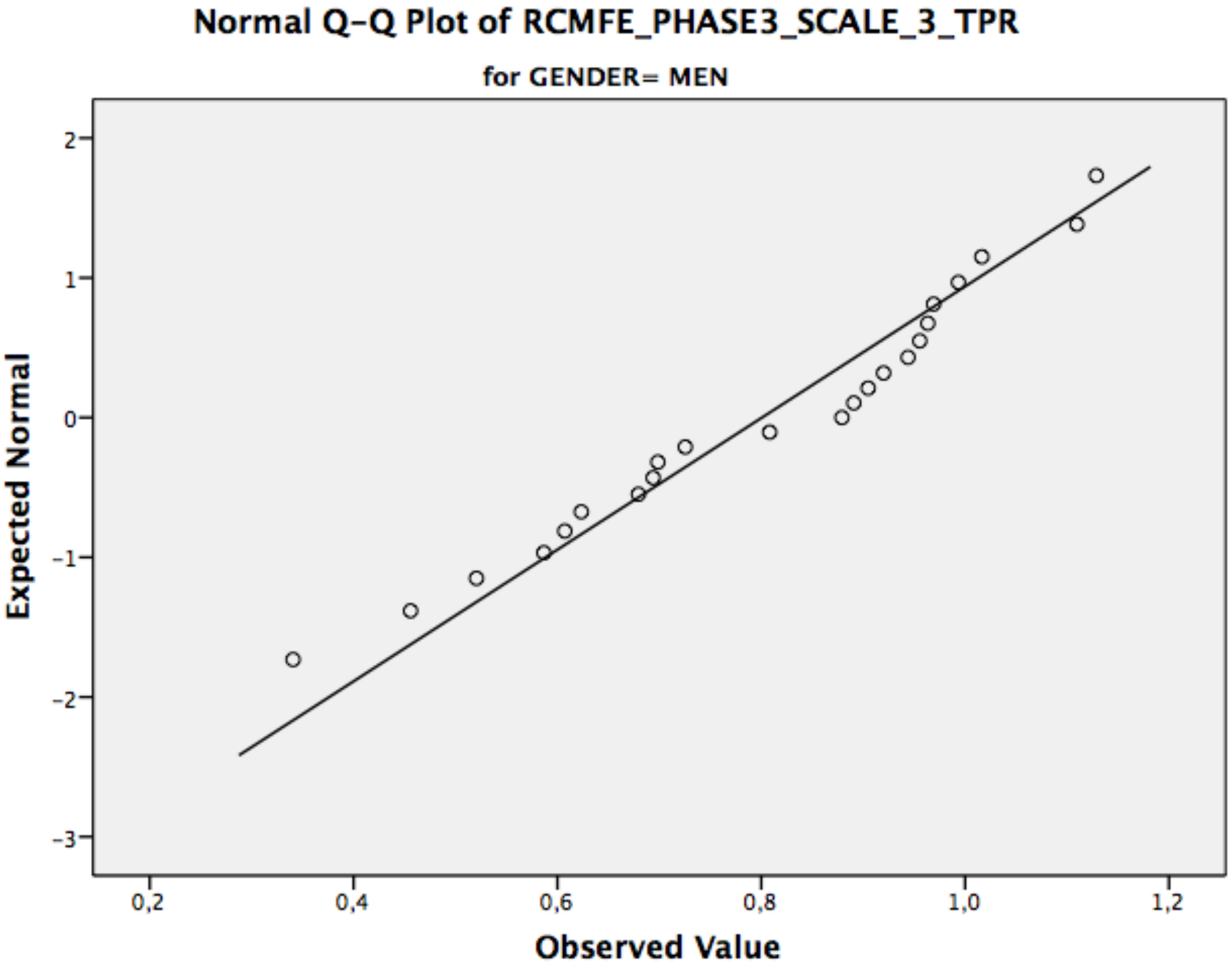
Normal Q-Q Plot of RCMFE_PHASE3_SCALE_3_TPR
for GENDER= MEN
Expected Normal
Observed Value
2
1
0
-1
-2
-3
0,2
0,4
0,6
0,8
1,0
1,2

RCMFE_PHASE3_SCALE_4_TPR

Normal Q-Q Plots

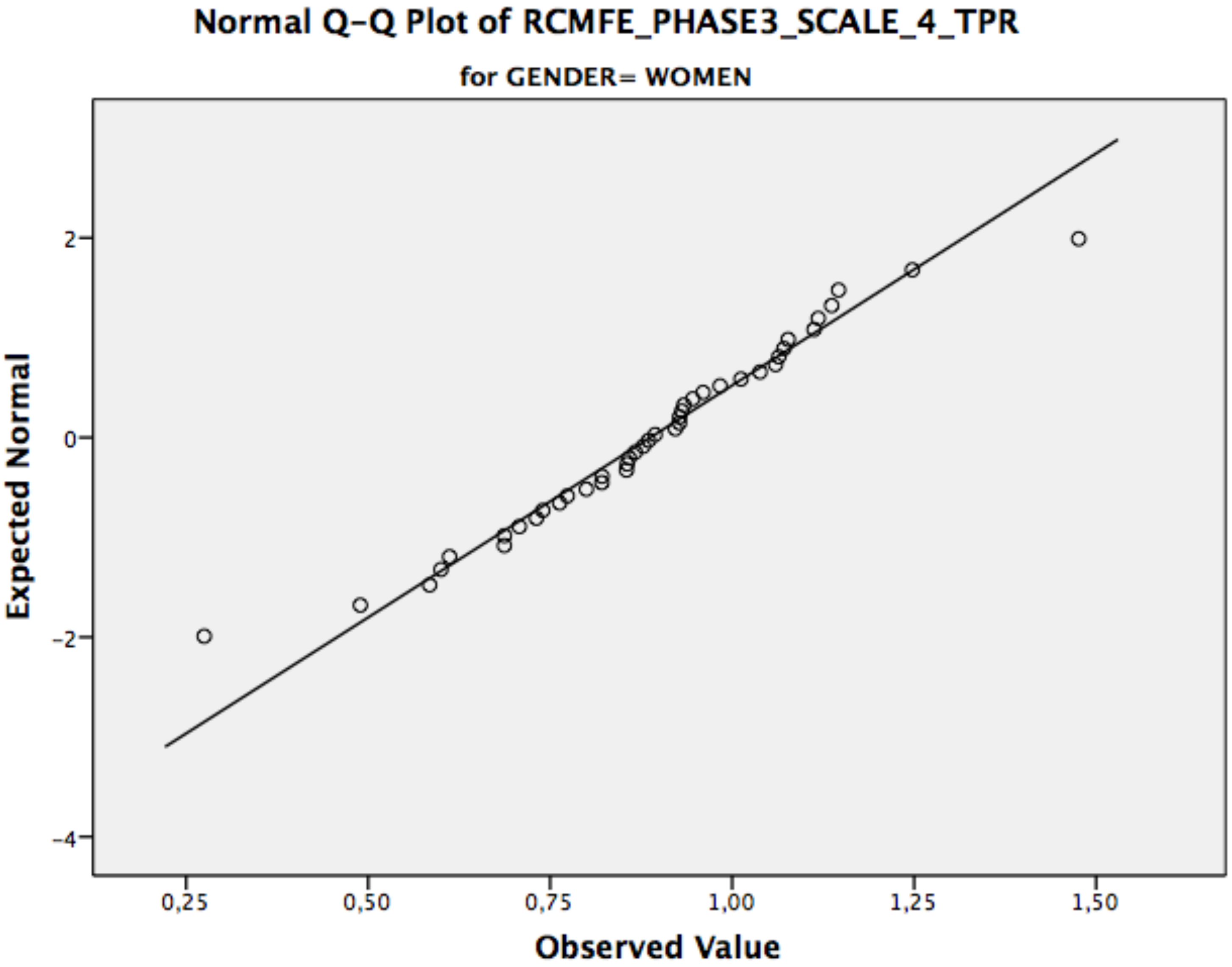

Normal Q-Q Plot of RCMFE_PHASE3_SCALE_4_TPR

for GENDER= MEN

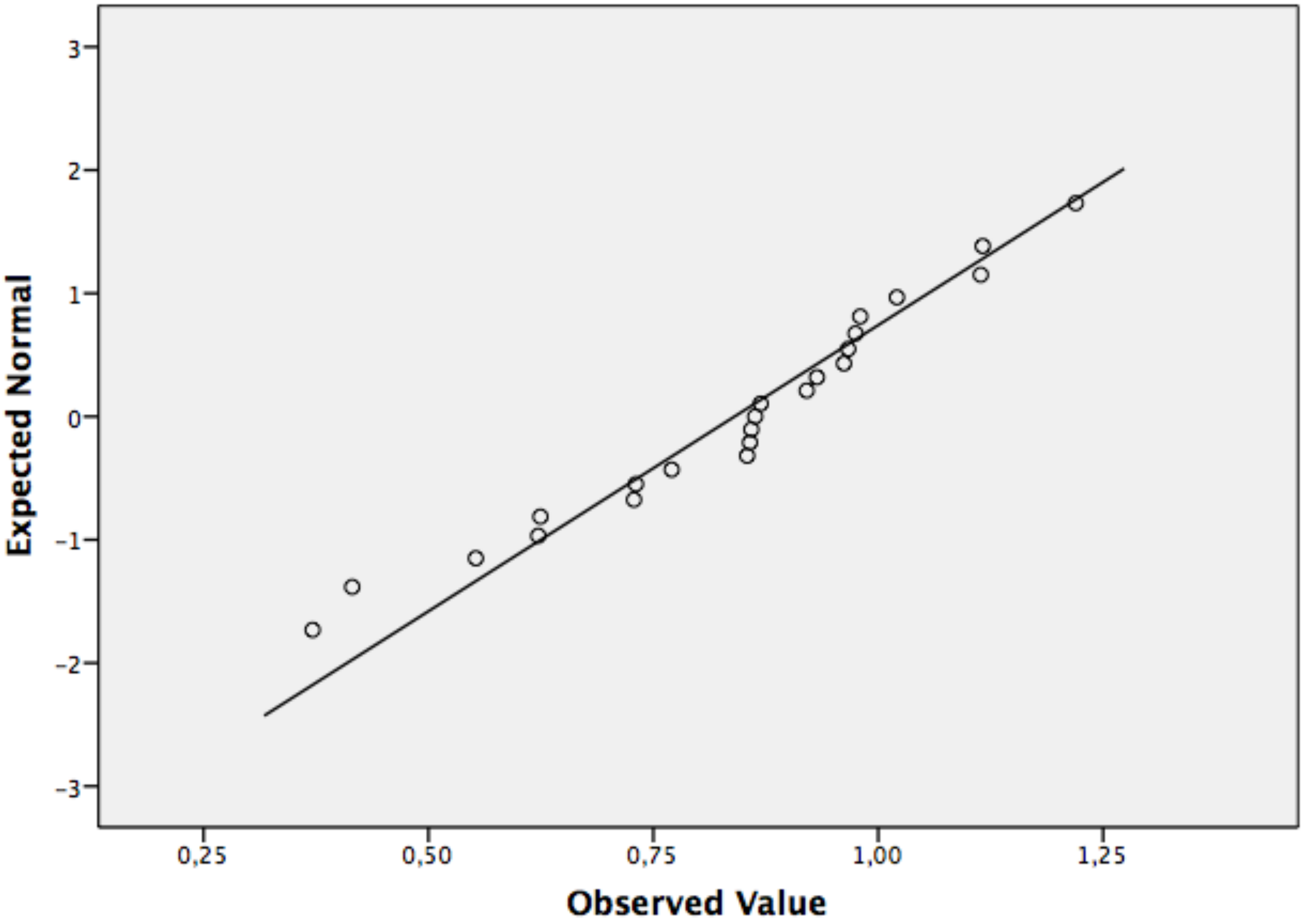

RCMFE_PHASE3_SCALE_5_TPR

Normal Q-Q Plots

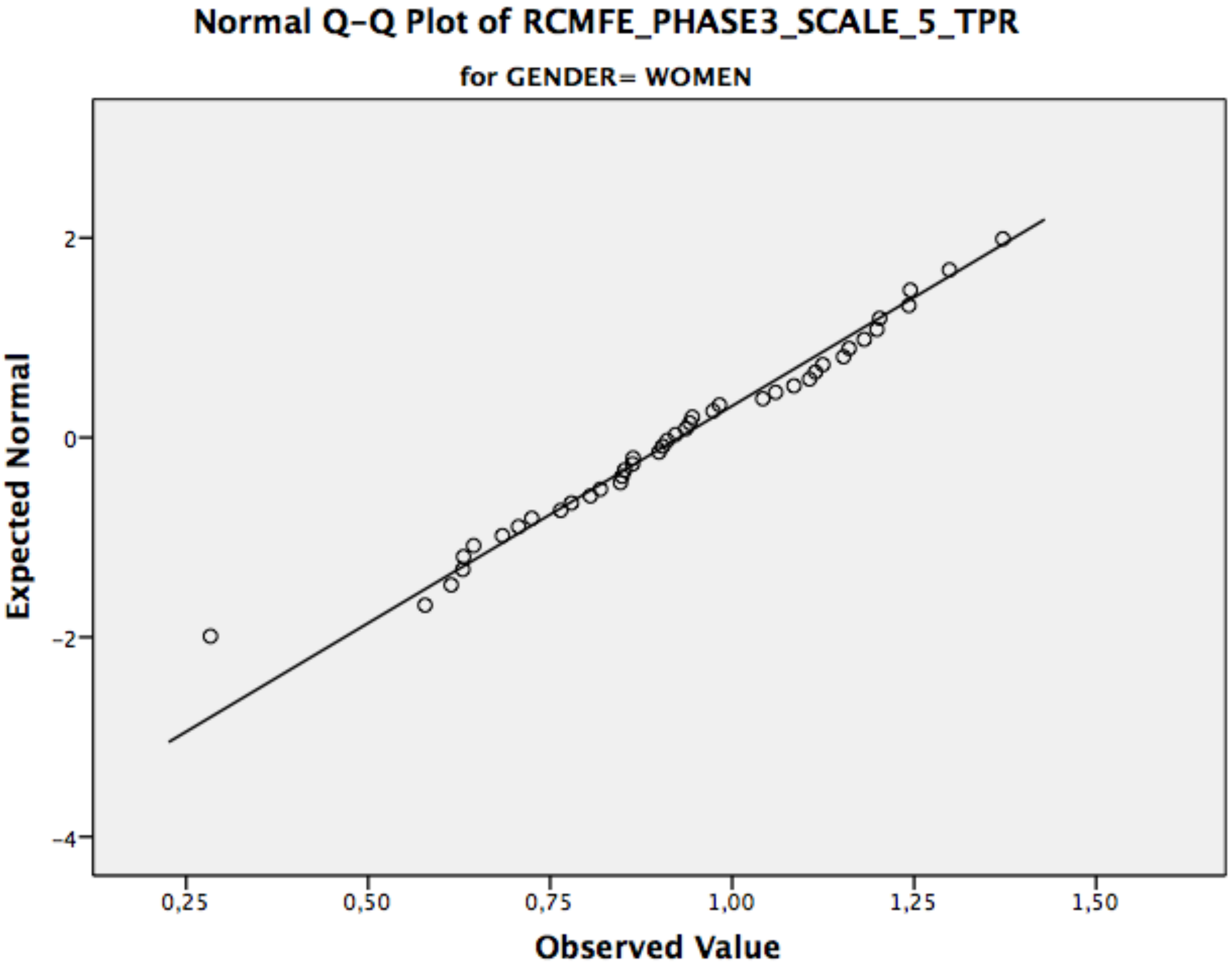

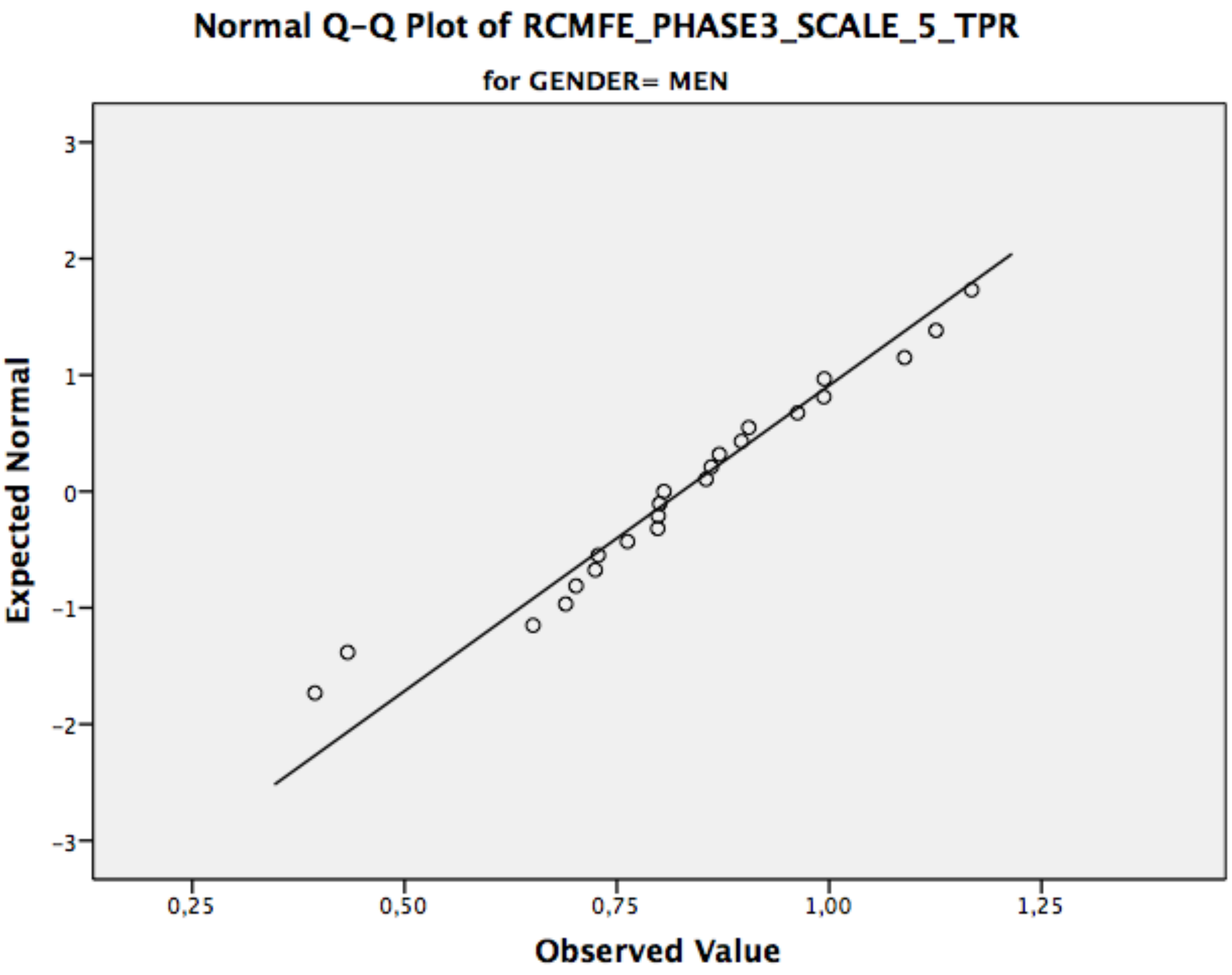

Normal Q-Q Plot of RCMFE_PHASE3_SCALE_5_TPR
for GENDER= MEN
Expected Normal
Observed Value
3
2
1
0
-1
-2
-3
0,25
0,50
0,75
1,00
1,25

RCMFE_PHASE4_SCALE_1_TPR

Normal Q-Q Plots

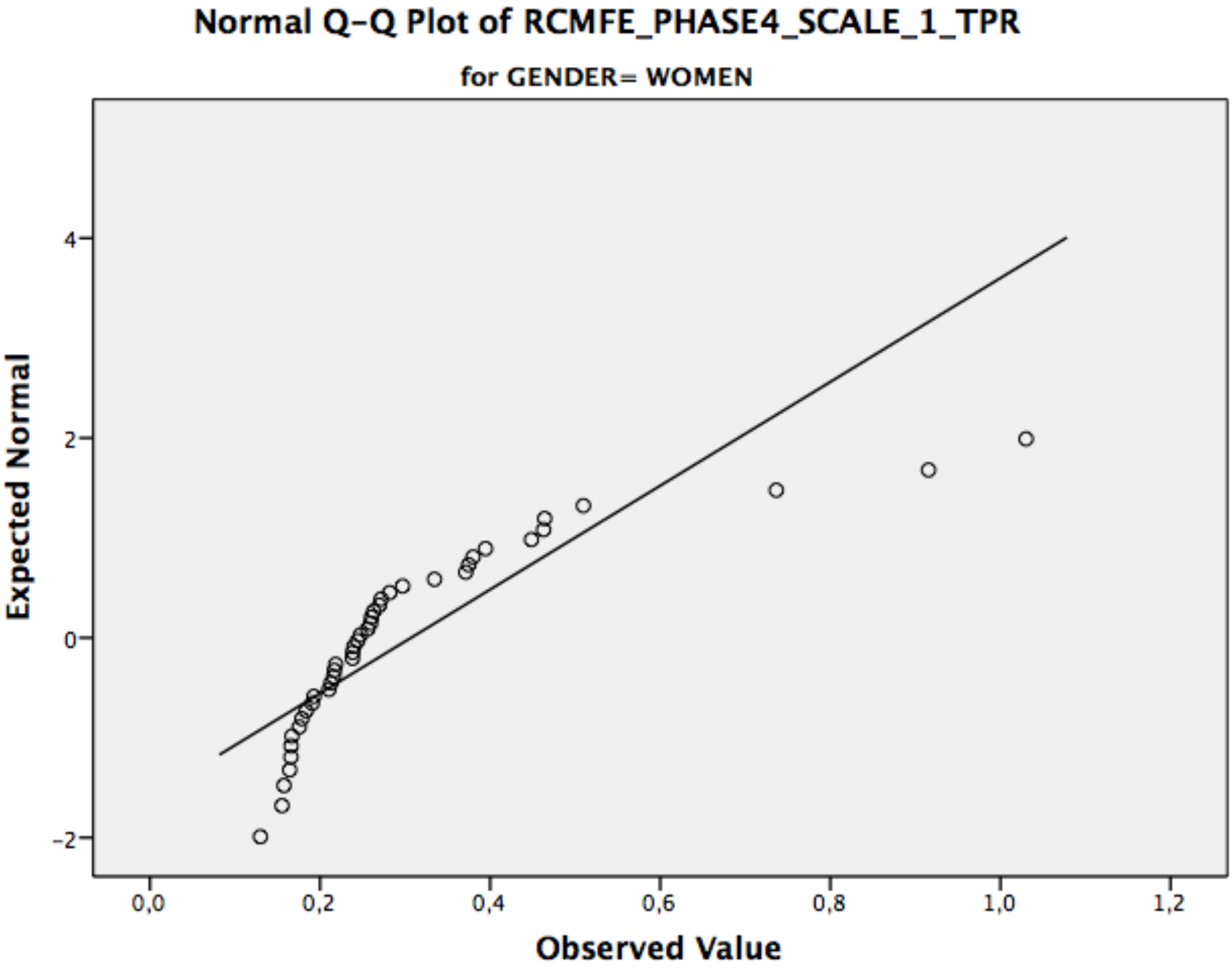

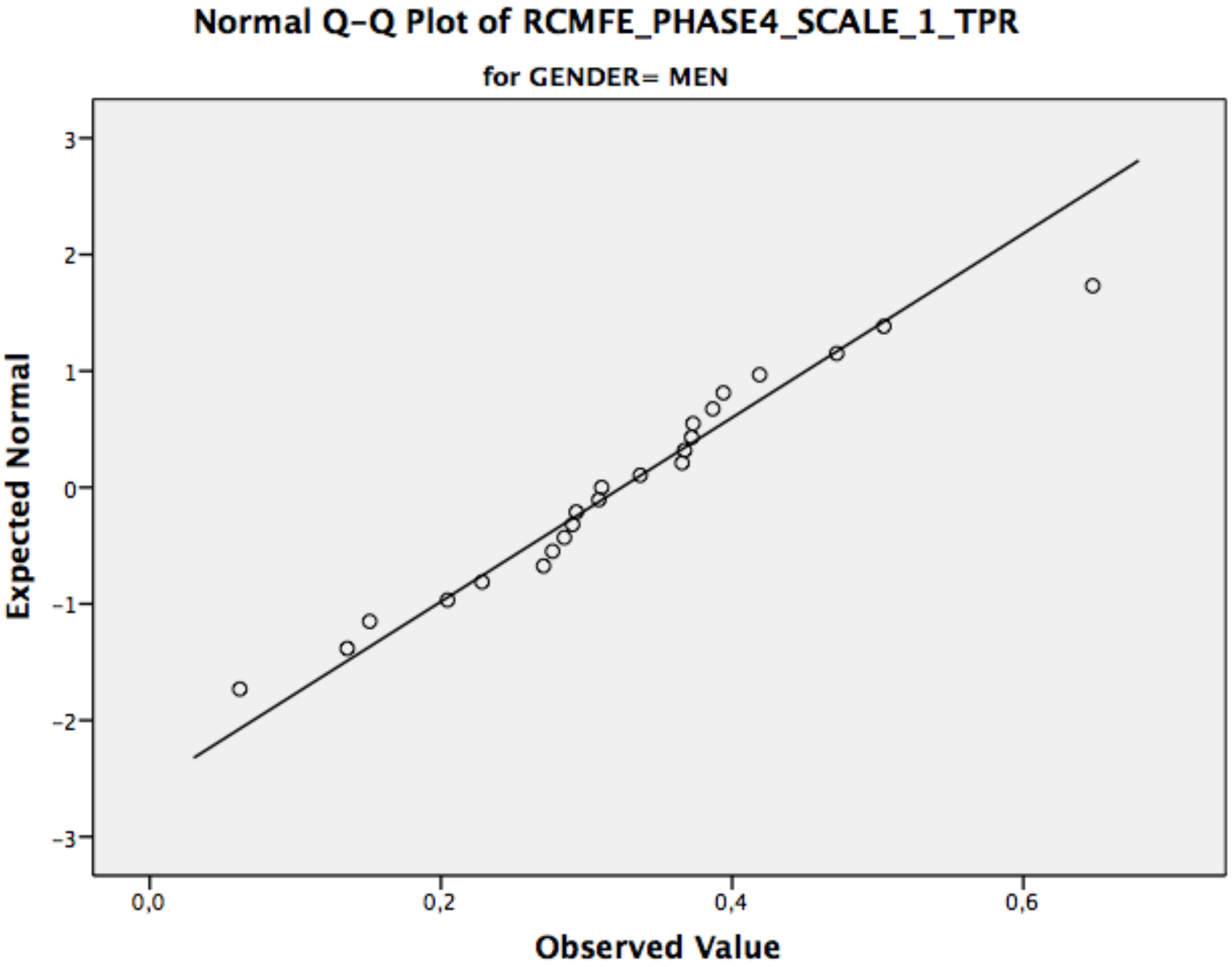
Normal Q-Q Plot of RCMFE_PHASE4_SCALE_1_TPR
for GENDER= MEN
Expected Normal
Observed Value
3
2
1
0
-1
-2
-3
0,0
0,2
0,4
0,6

RCMFE_PHASE4_SCALE_2_TPR

Normal Q-Q Plots

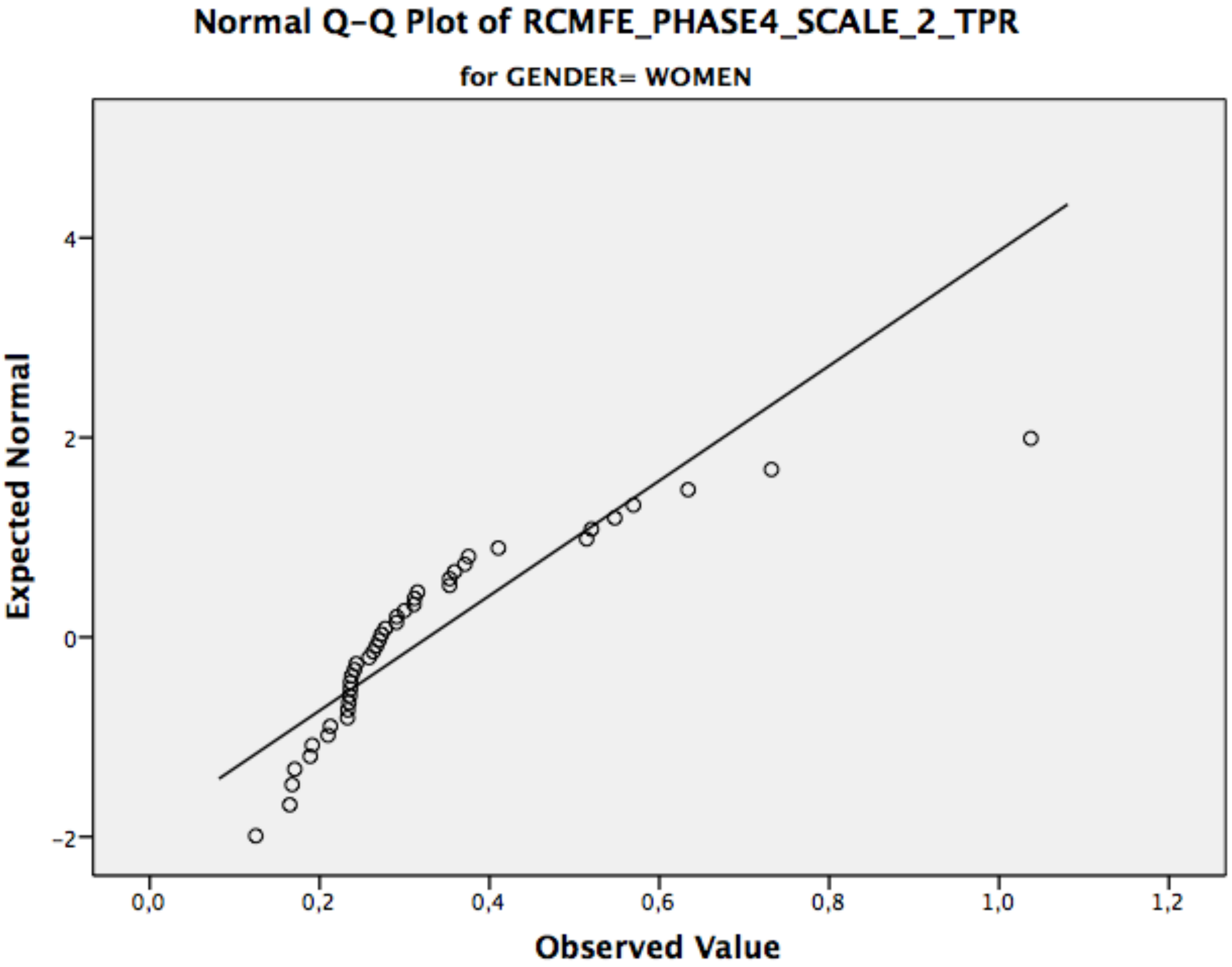

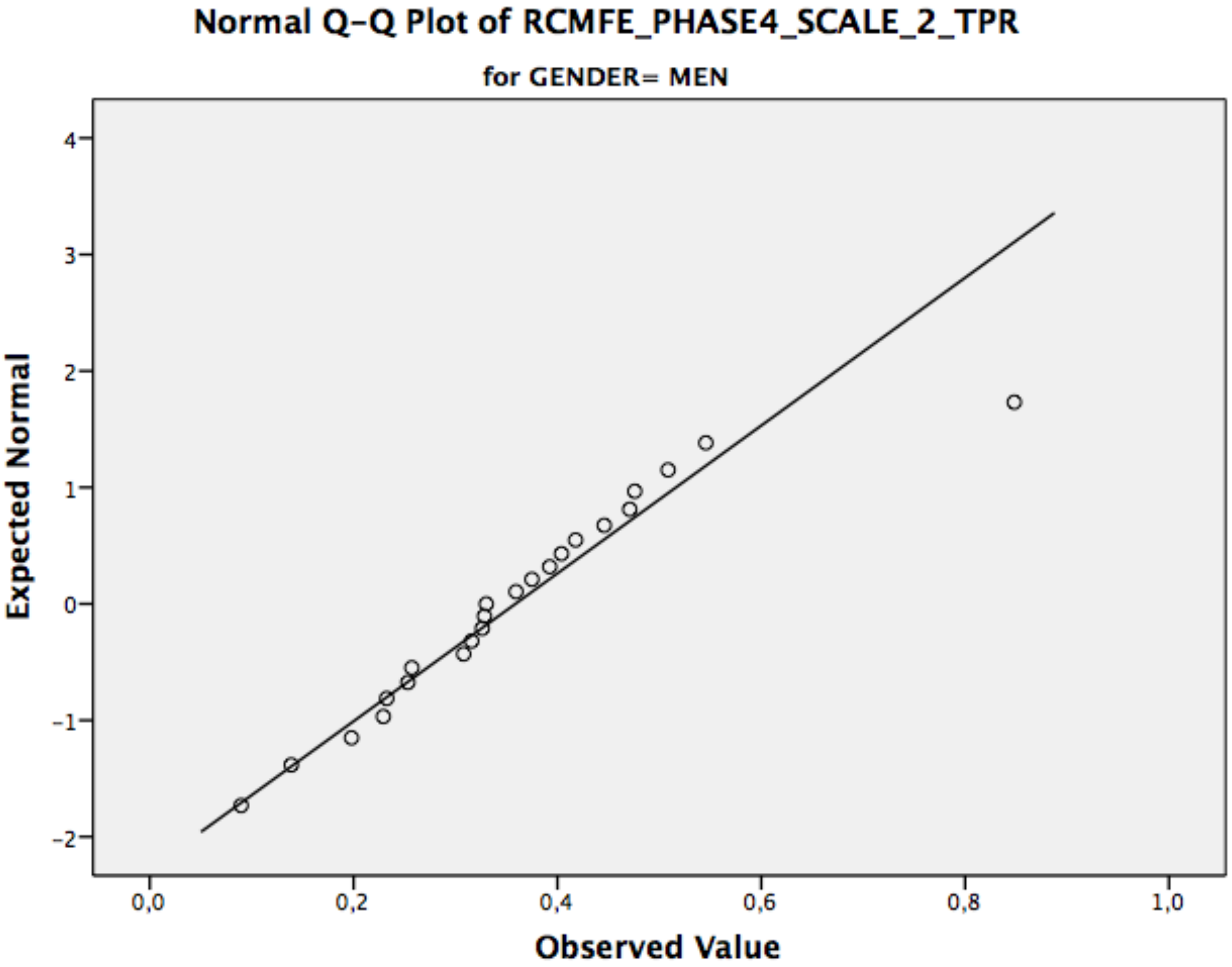
Normal Q-Q Plot of RCMFE_PHASE4_SCALE_2_TPR
for GENDER= MEN
Expected Normal
Observed Value
4
3
2
1
0
-1
-2
0,0
0,2
0,4
0,6
0,8
1,0

RCMFE_PHASE4_SCALE_3_TPR

Normal Q-Q Plots

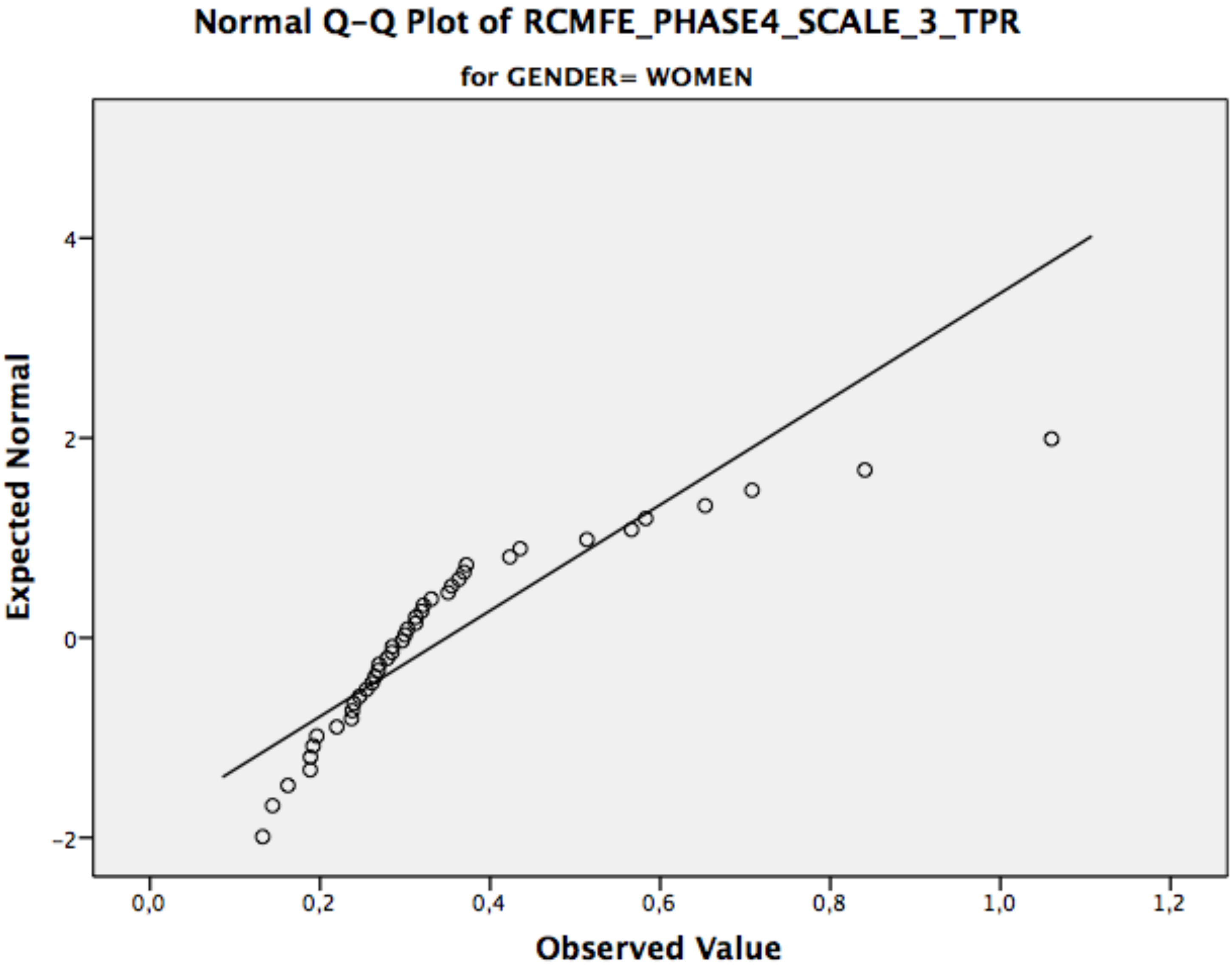

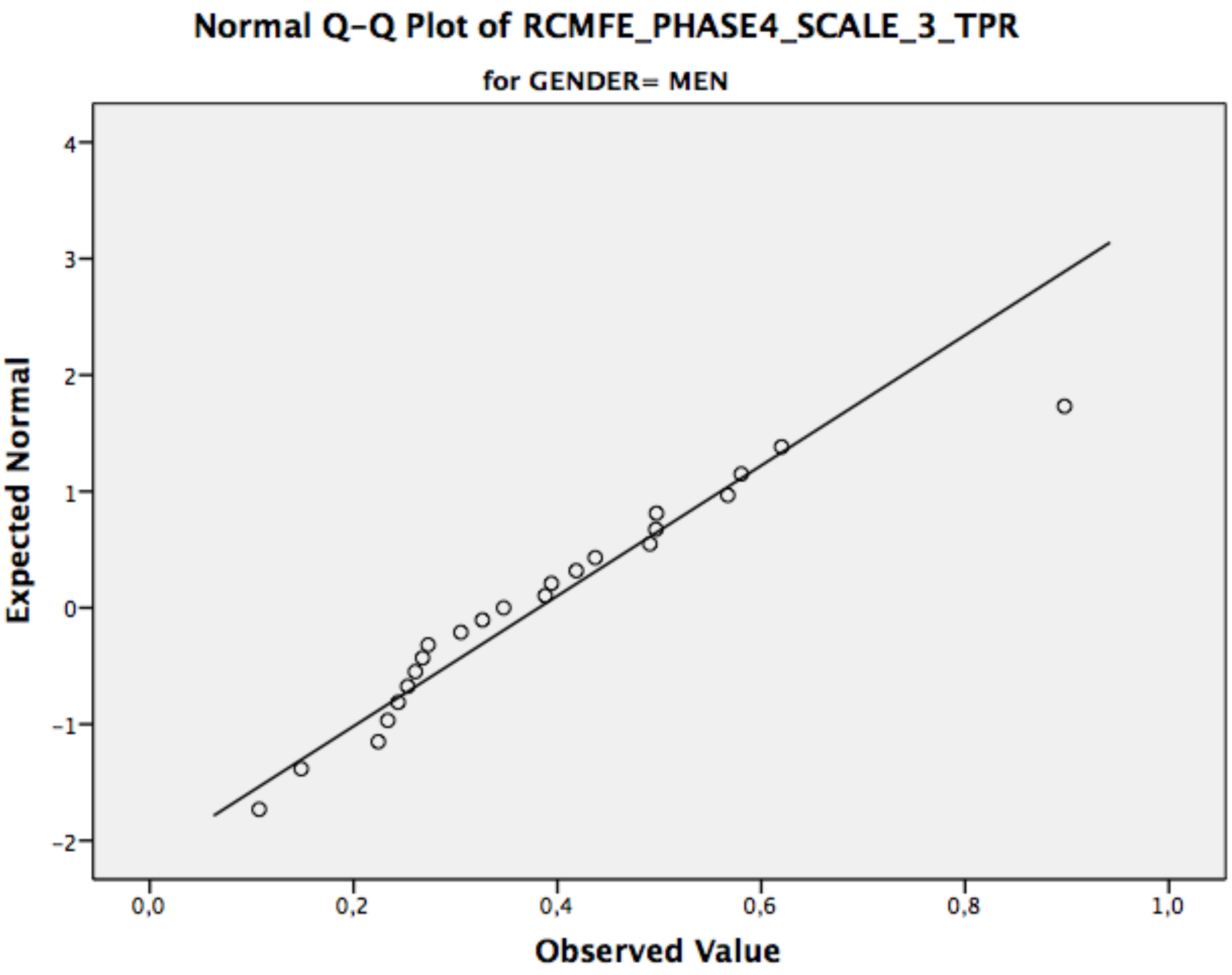

Normal Q-Q Plot of RCMFE_PHASE4_SCALE_3_TPR
for GENDER= MEN
Expected Normal
4
3
2
1
0
-1
-2
0,0
0,2
0,4
0,6
0,8
1,0
Observed Value

RCMFE_PHASE4_SCALE_4_TPR

Normal Q-Q Plots

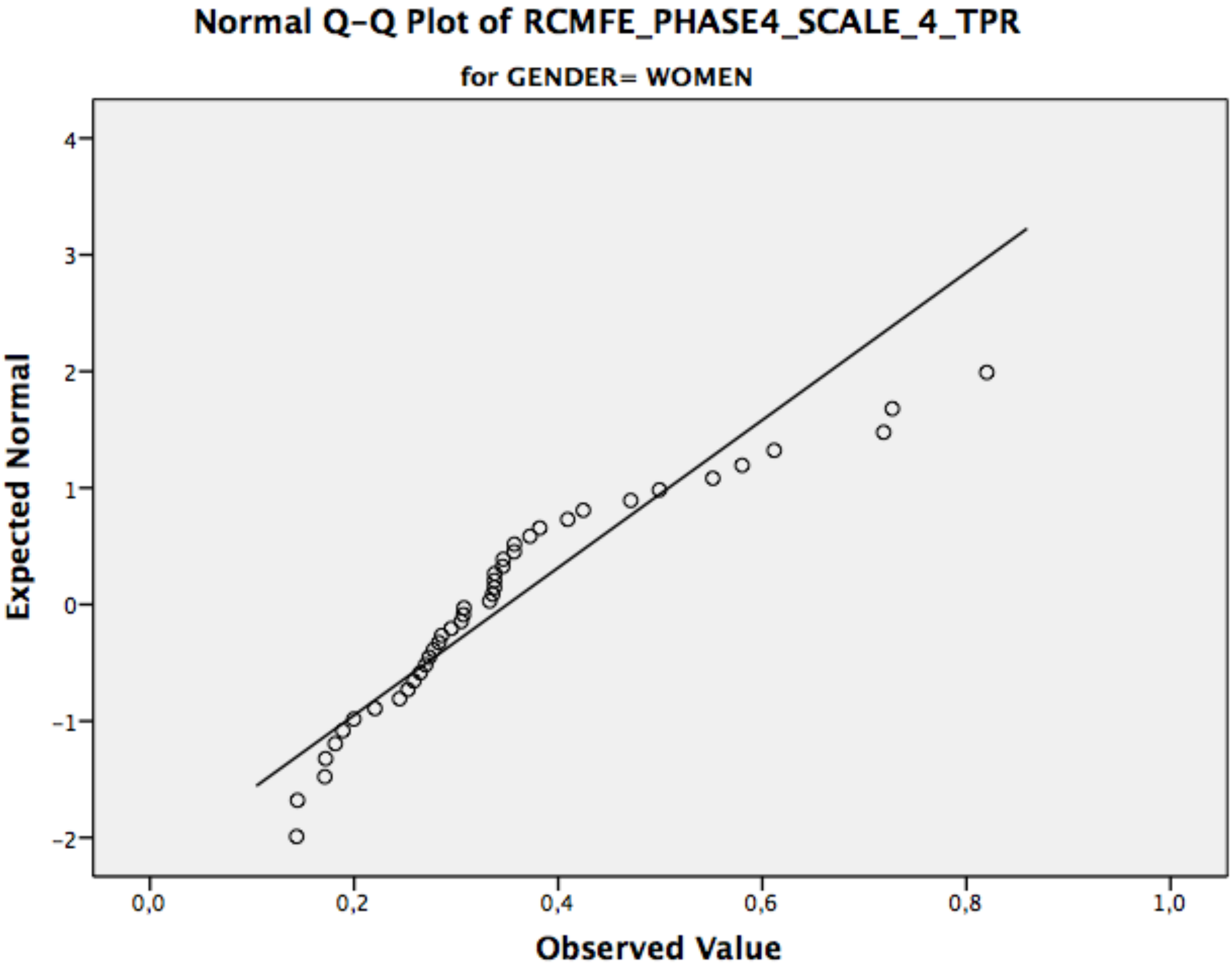

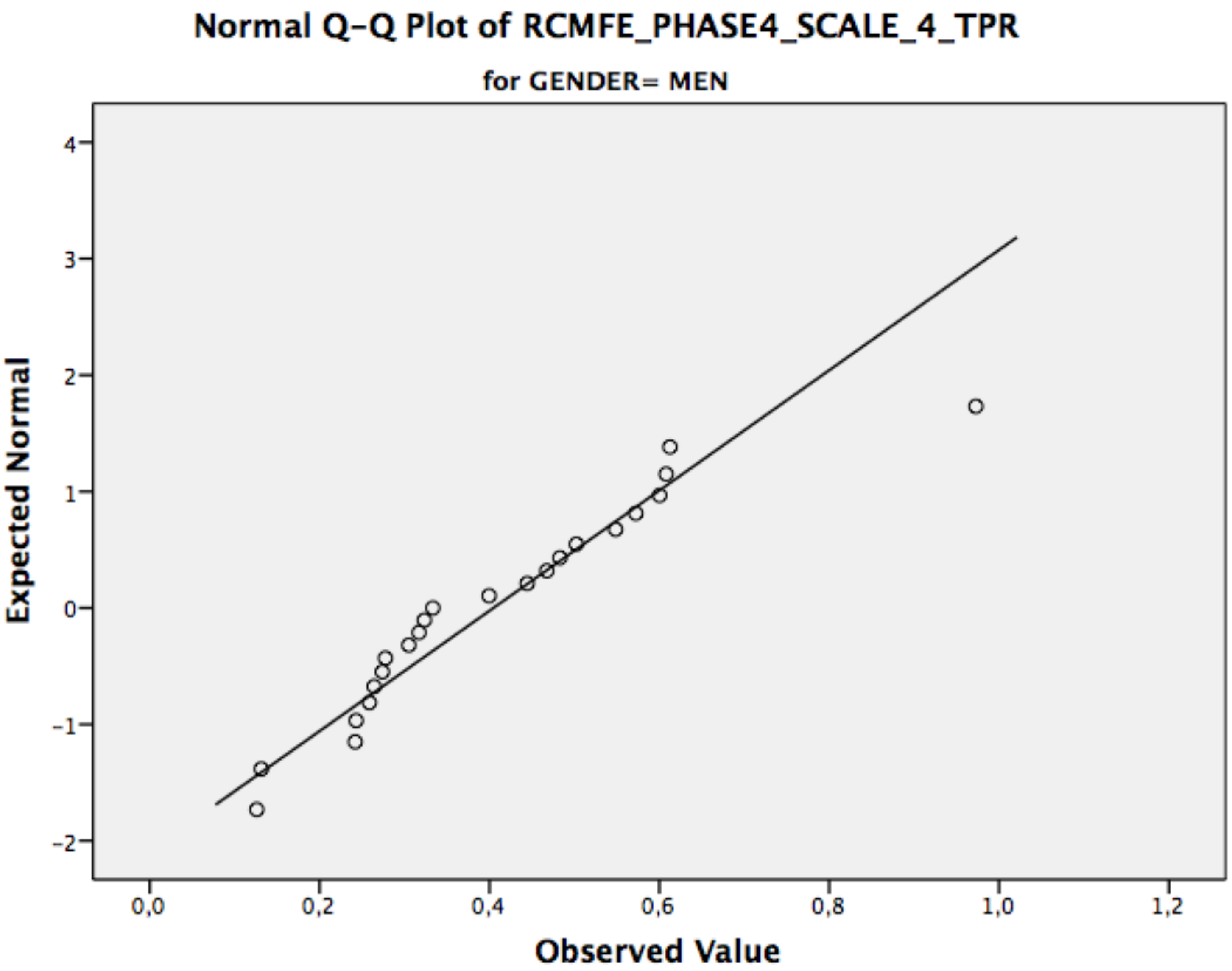
Normal Q-Q Plot of RCMFE_PHASE4_SCALE_4_TPR
for GENDER= MEN
Expected Normal
Observed Value
4
3
2
1
0
-1
-2
0,0
0,2
0,4
0,6
0,8
1,0
1,2

RCMFE_PHASE4_SCALE_5_TPR

Normal Q-Q Plots

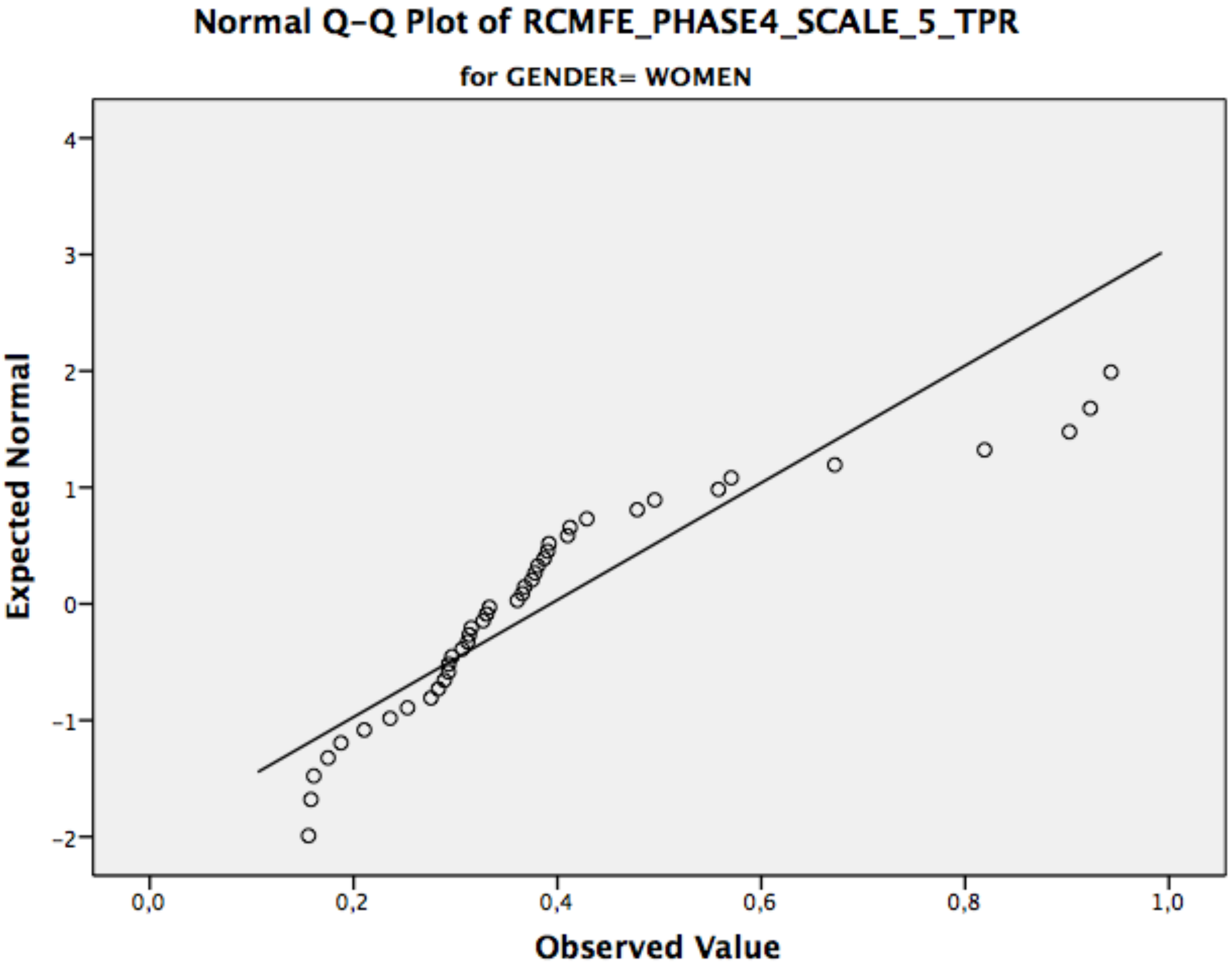

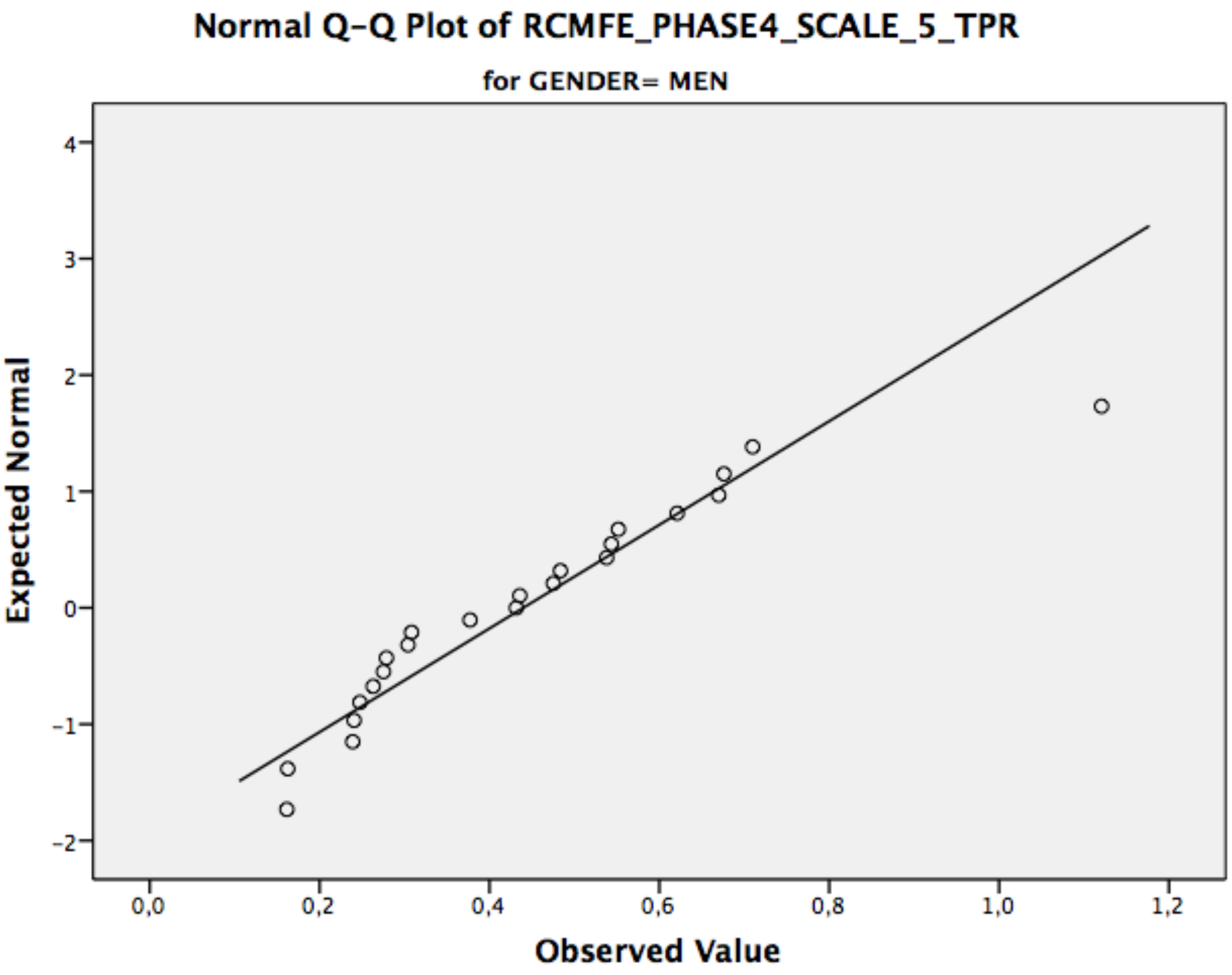
Normal Q-Q Plot of RCMFE_PHASE4_SCALE_5_TPR
for GENDER= MEN
Expected Normal
Observed Value
4
3
2
1
0
-1
-2
0,0
0,2
0,4
0,6
0,8
1,0
1,2

RCMFE_PHASE5_SCALE_1_TPR

Normal Q-Q Plots

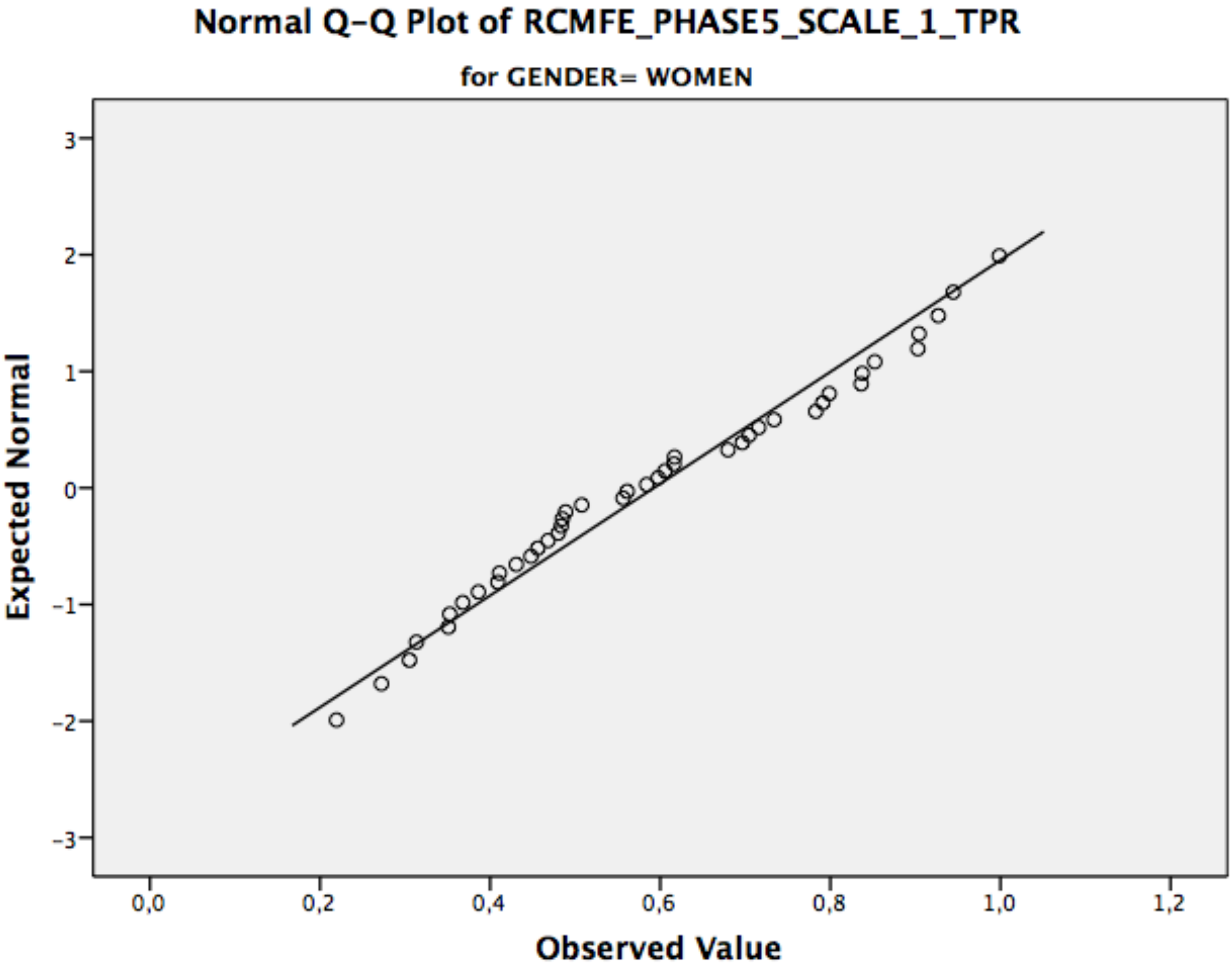

Normal Q-Q Plot of RCMFE_PHASE5_SCALE_1_TPR

for GENDER= MEN

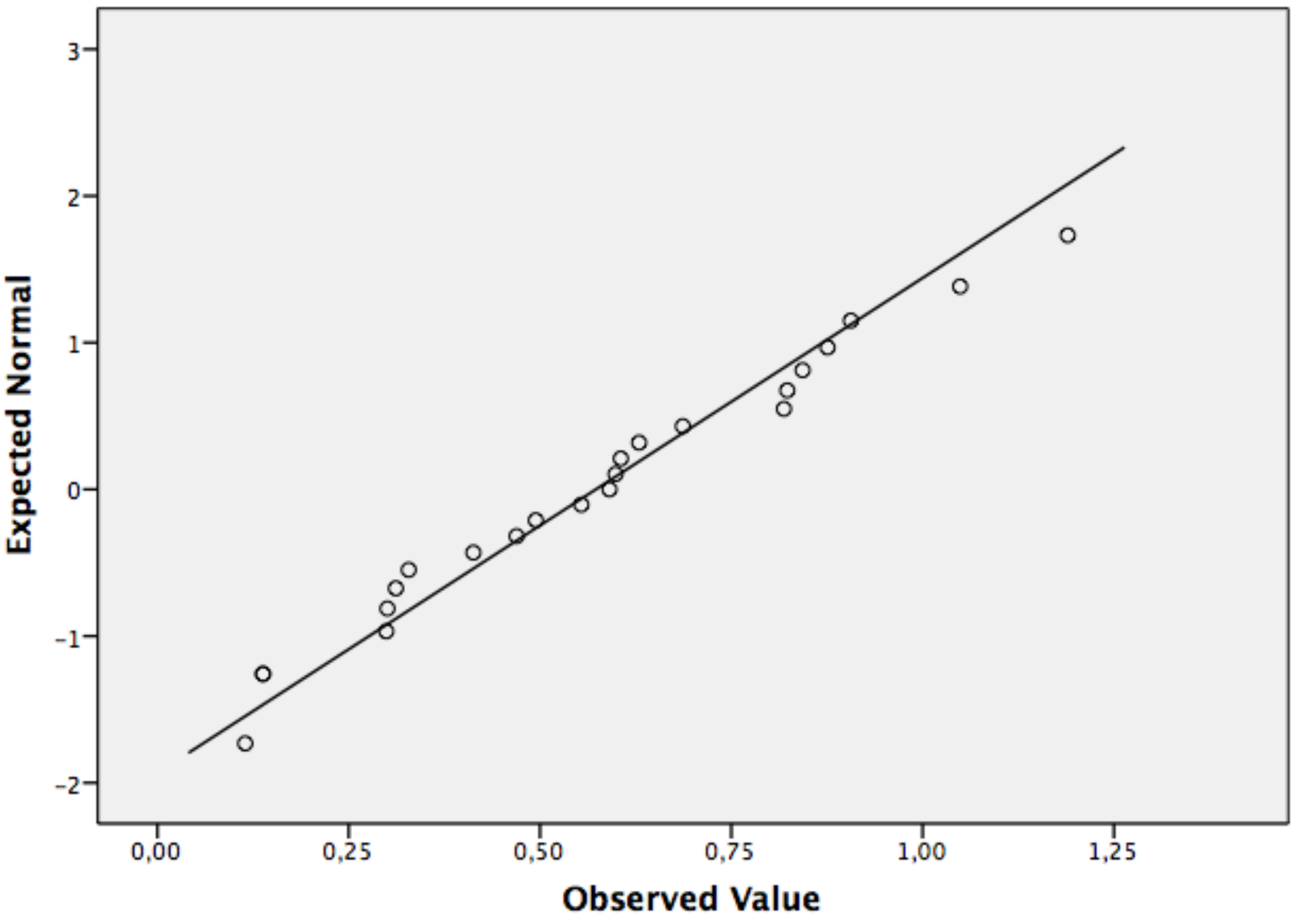

RCMFE_PHASE5_SCALE_2_TPR

Normal Q-Q Plots

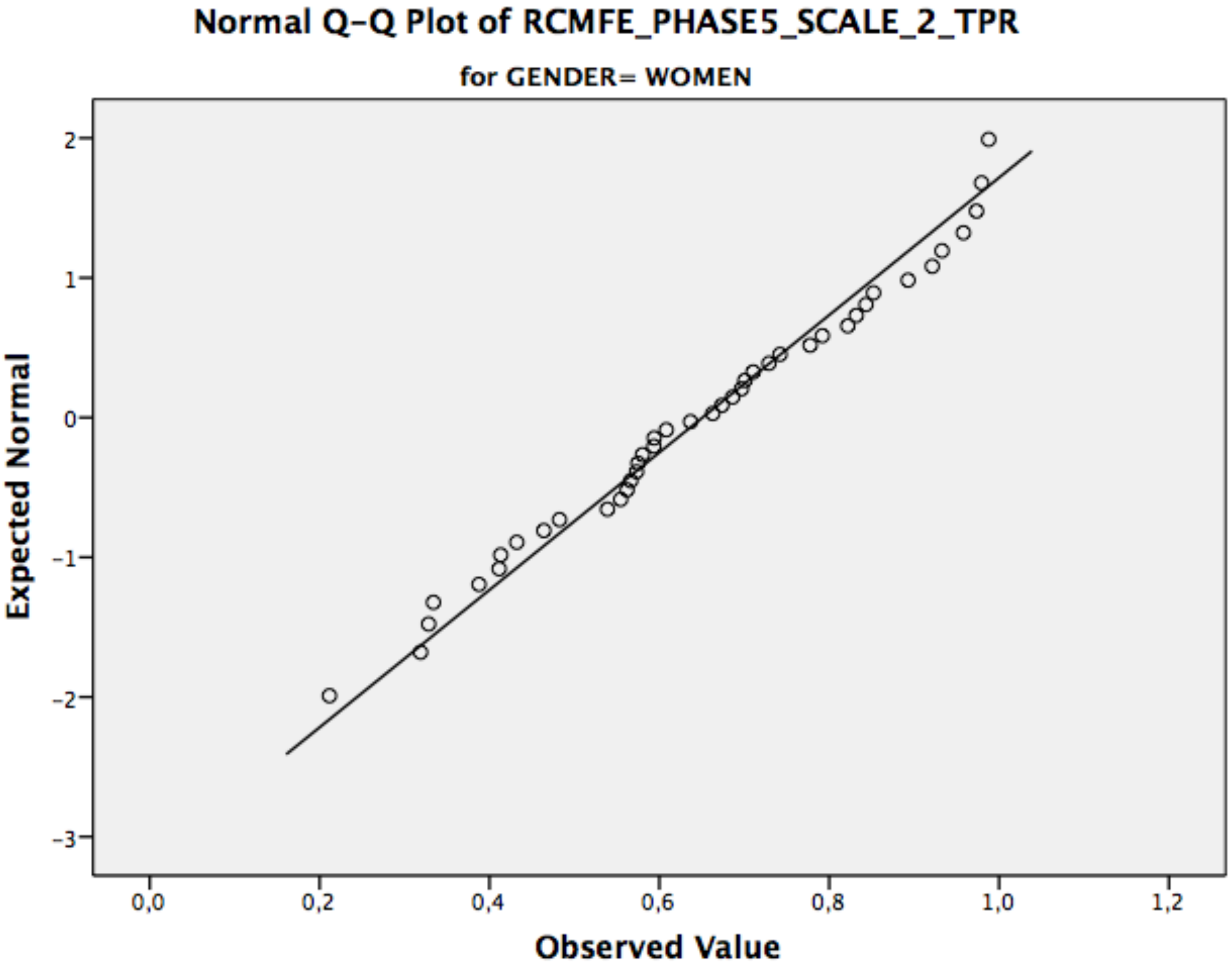

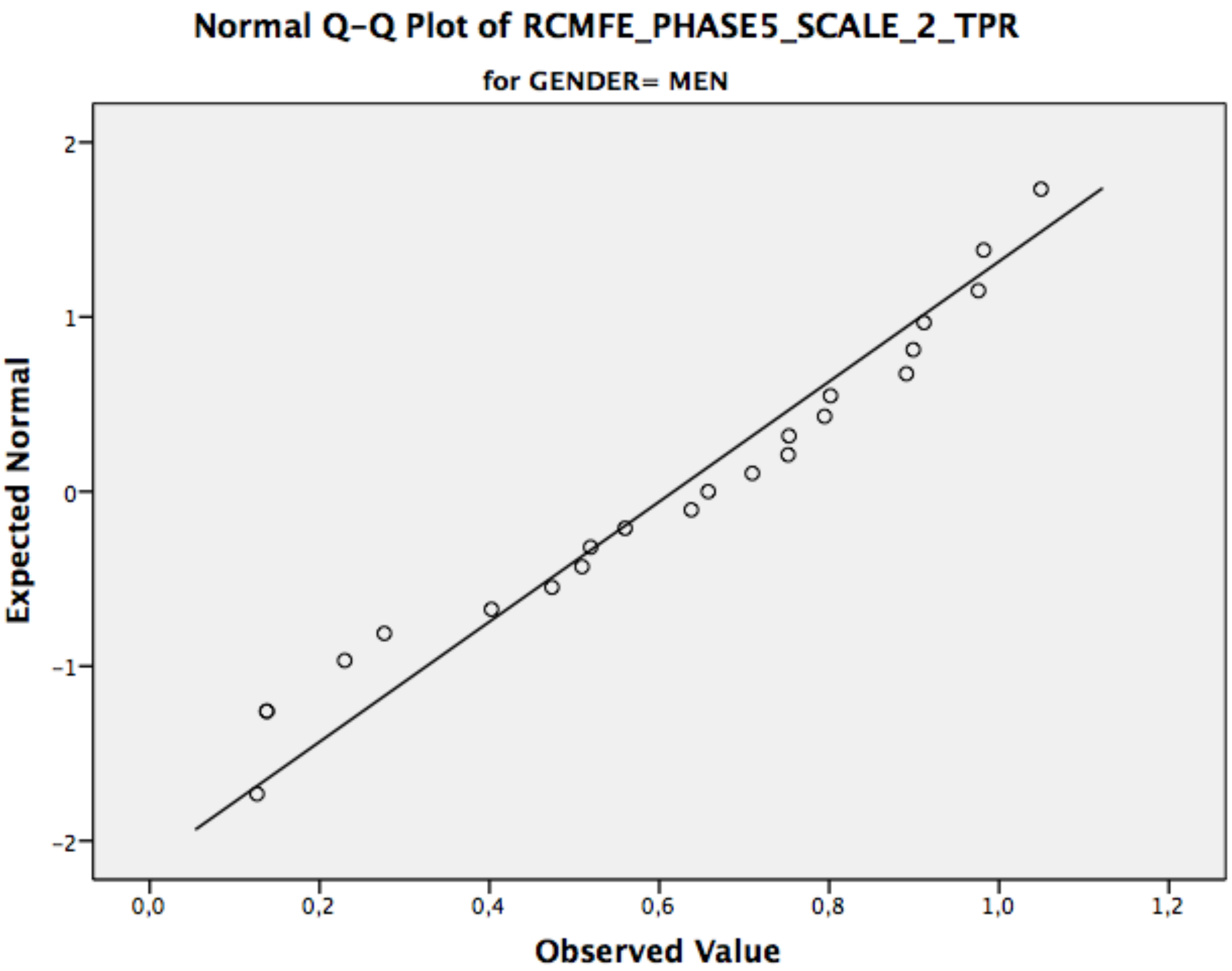
Normal Q-Q Plot of RCMFE_PHASE5_SCALE_2_TPR
for GENDER= MEN
Expected Normal
2
1
0
-1
-2
0,0
0,2
0,4
0,6
0,8
1,0
1,2
Observed Value

RCMFE_PHASE5_SCALE_3_TPR

Normal Q-Q Plots

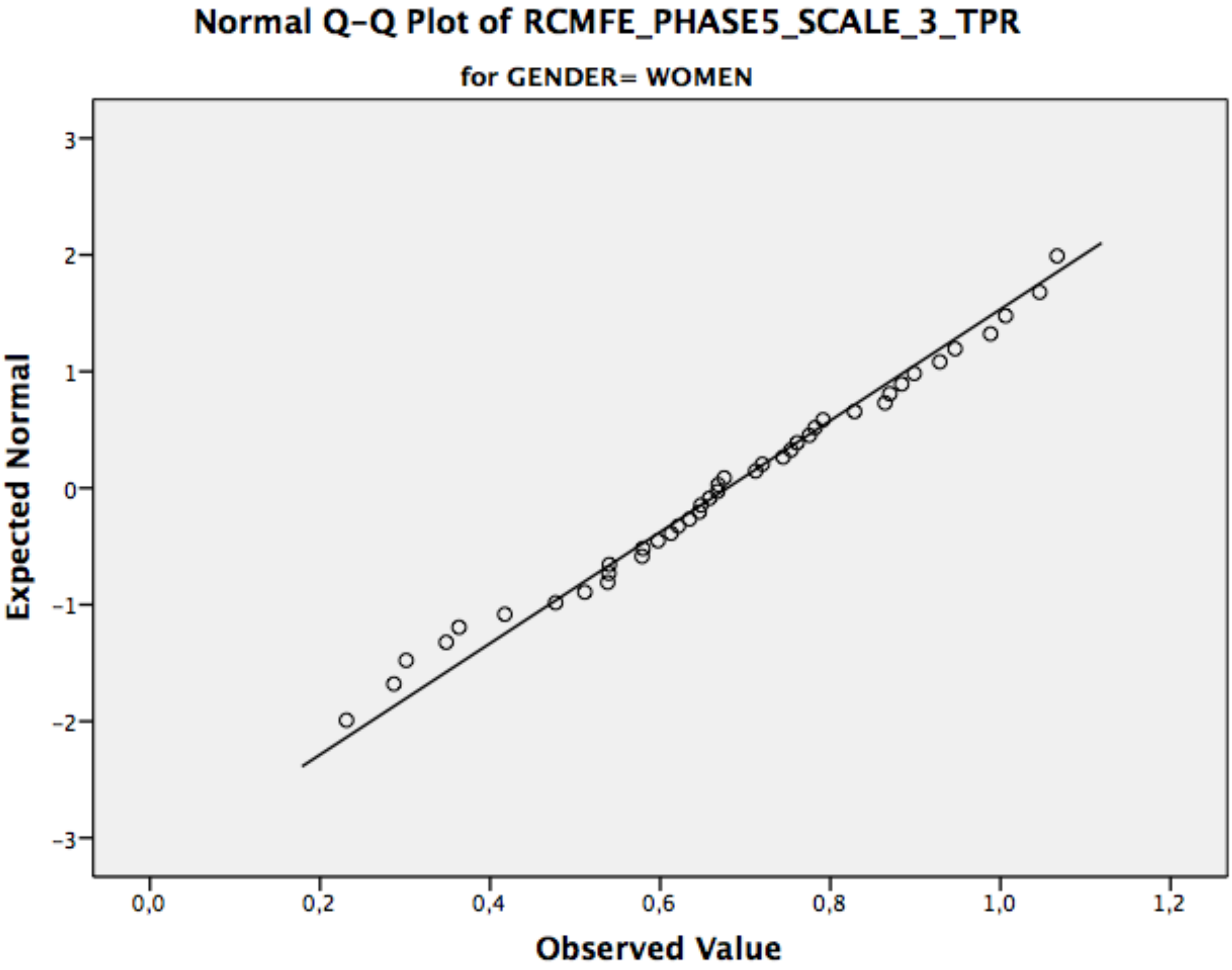

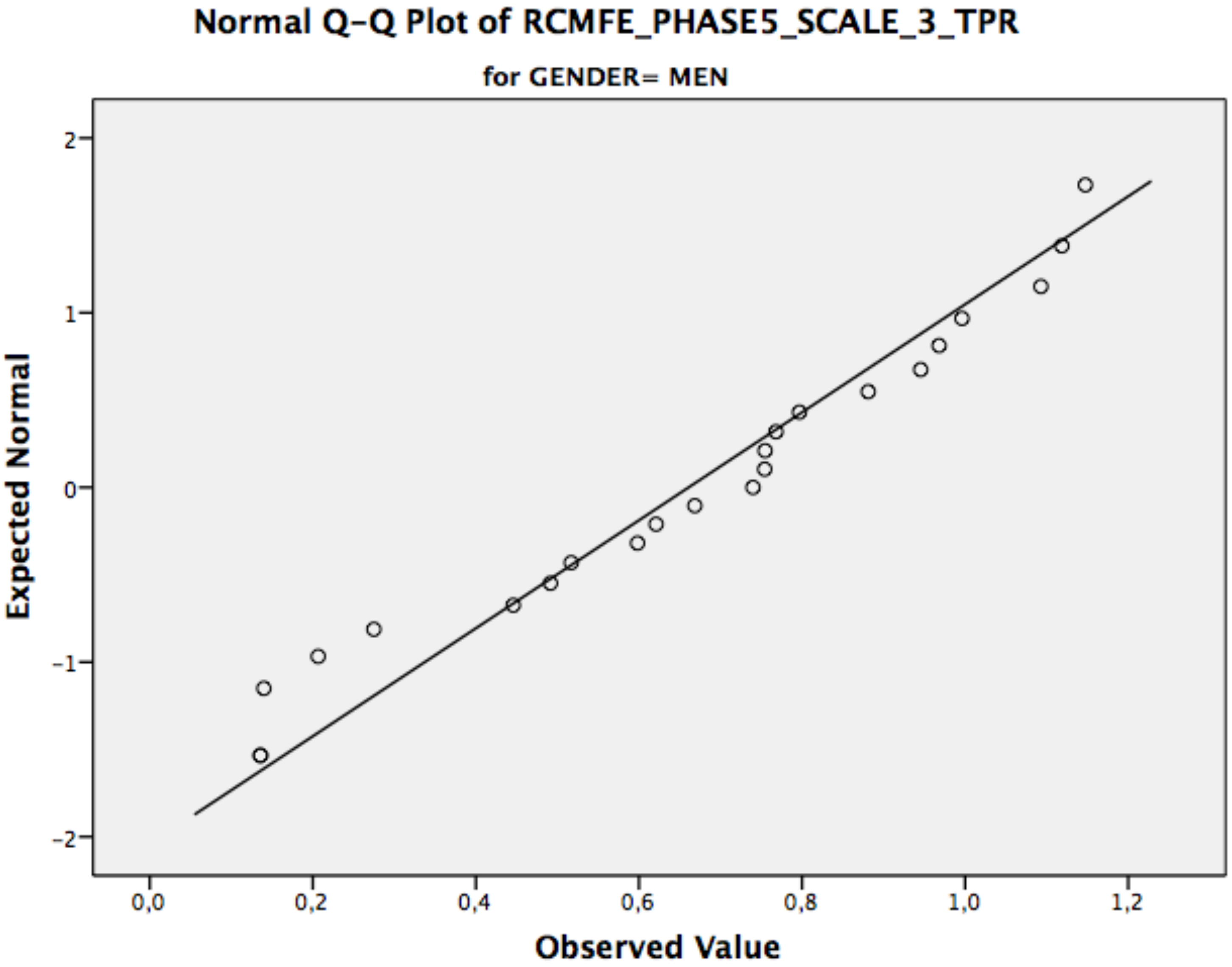

Normal Q-Q Plot of RCMFE_PHASE5_SCALE_3_TPR
for GENDER= MEN
Expected Normal
Observed Value
2
1
0
-1
-2
0,0
0,2
0,4
0,6
0,8
1,0
1,2

RCMFE_PHASE5_SCALE_4_TPR

Normal Q-Q Plots

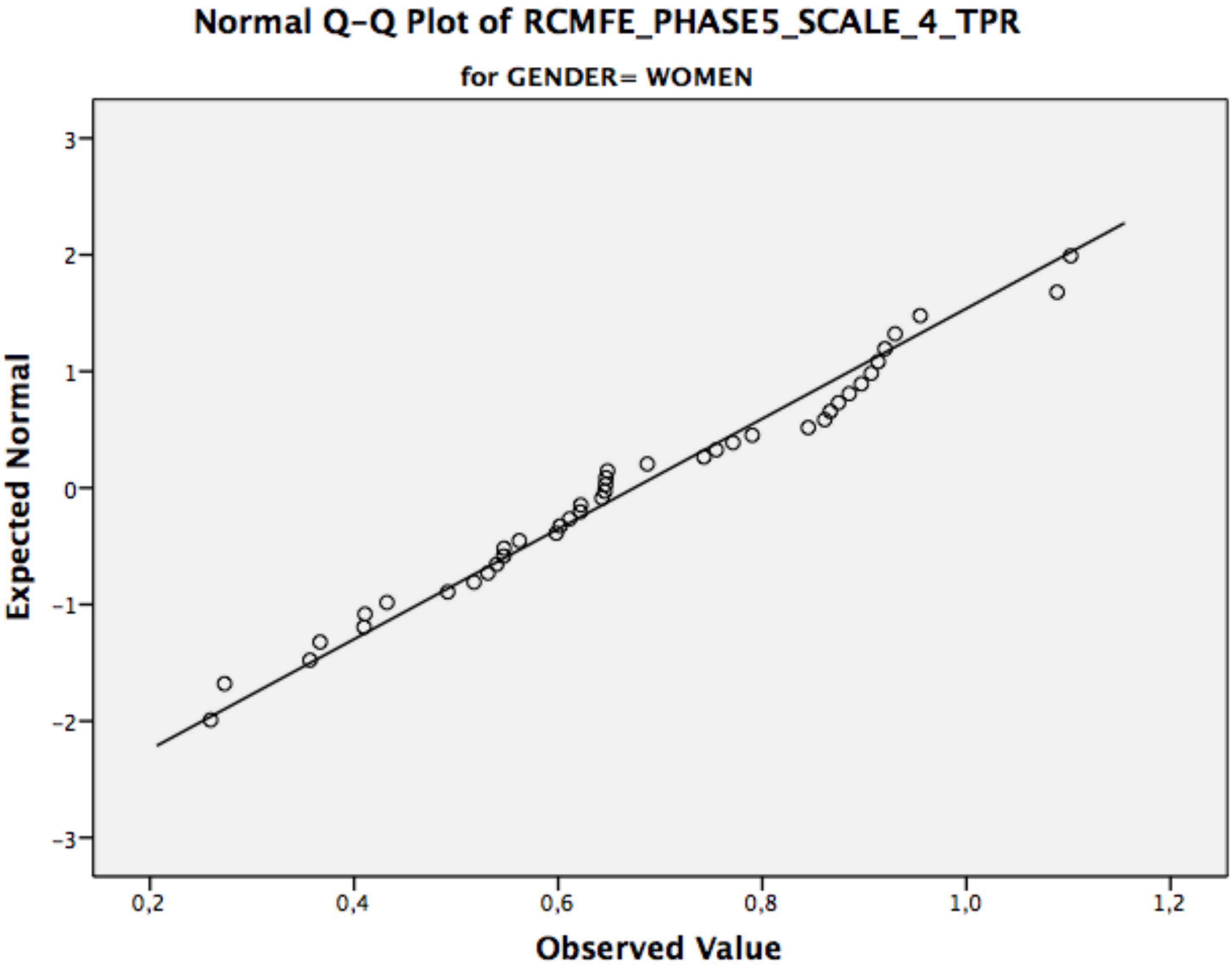

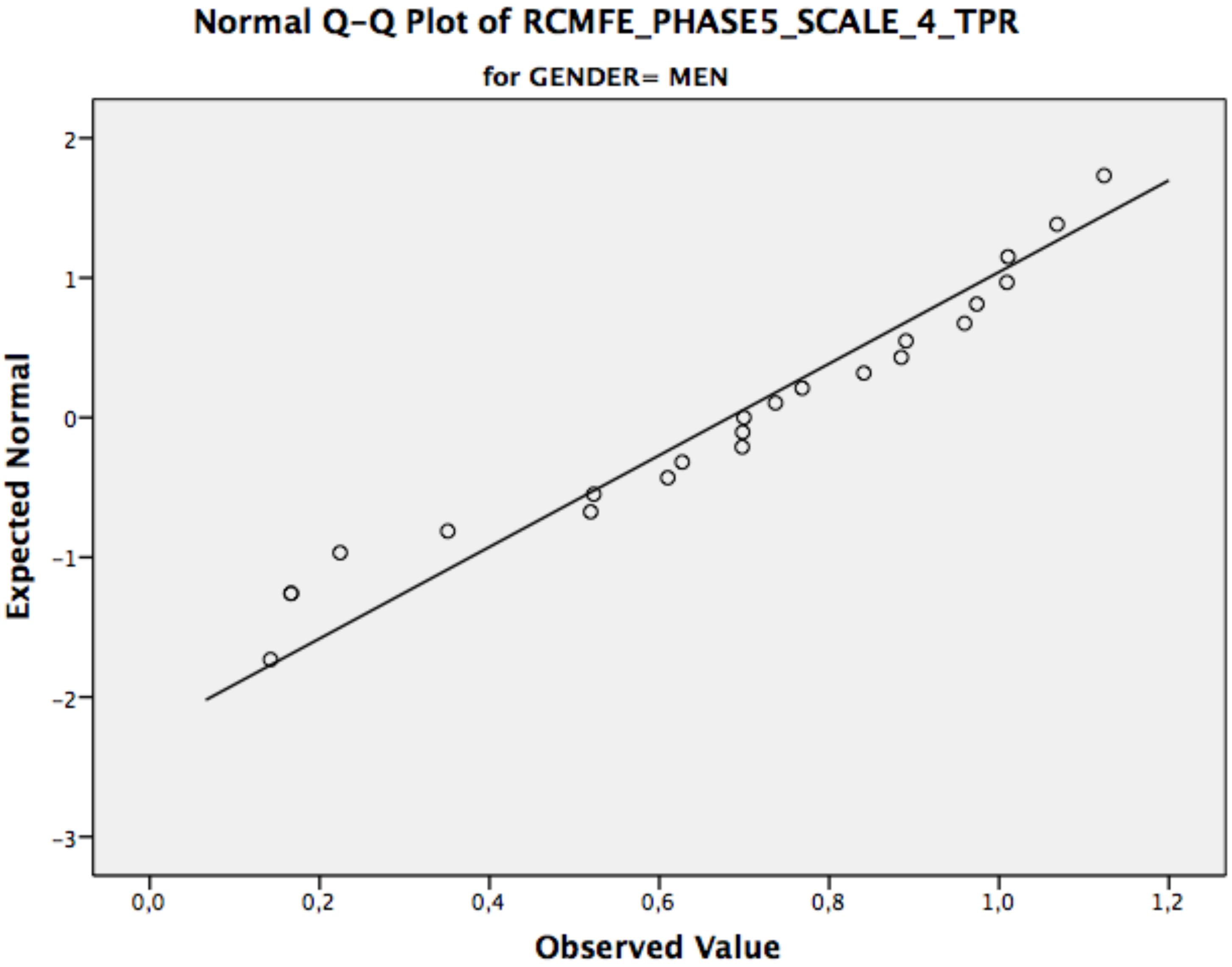
Normal Q-Q Plot of RCMFE_PHASE5_SCALE_4_TPR
for GENDER= MEN
Expected Normal
Observed Value
2
1
0
-1
-2
-3
0,0
0,2
0,4
0,6
0,8
1,0
1,2

RCMFE_PHASE5_SCALE_5_TPR

Normal Q-Q Plots

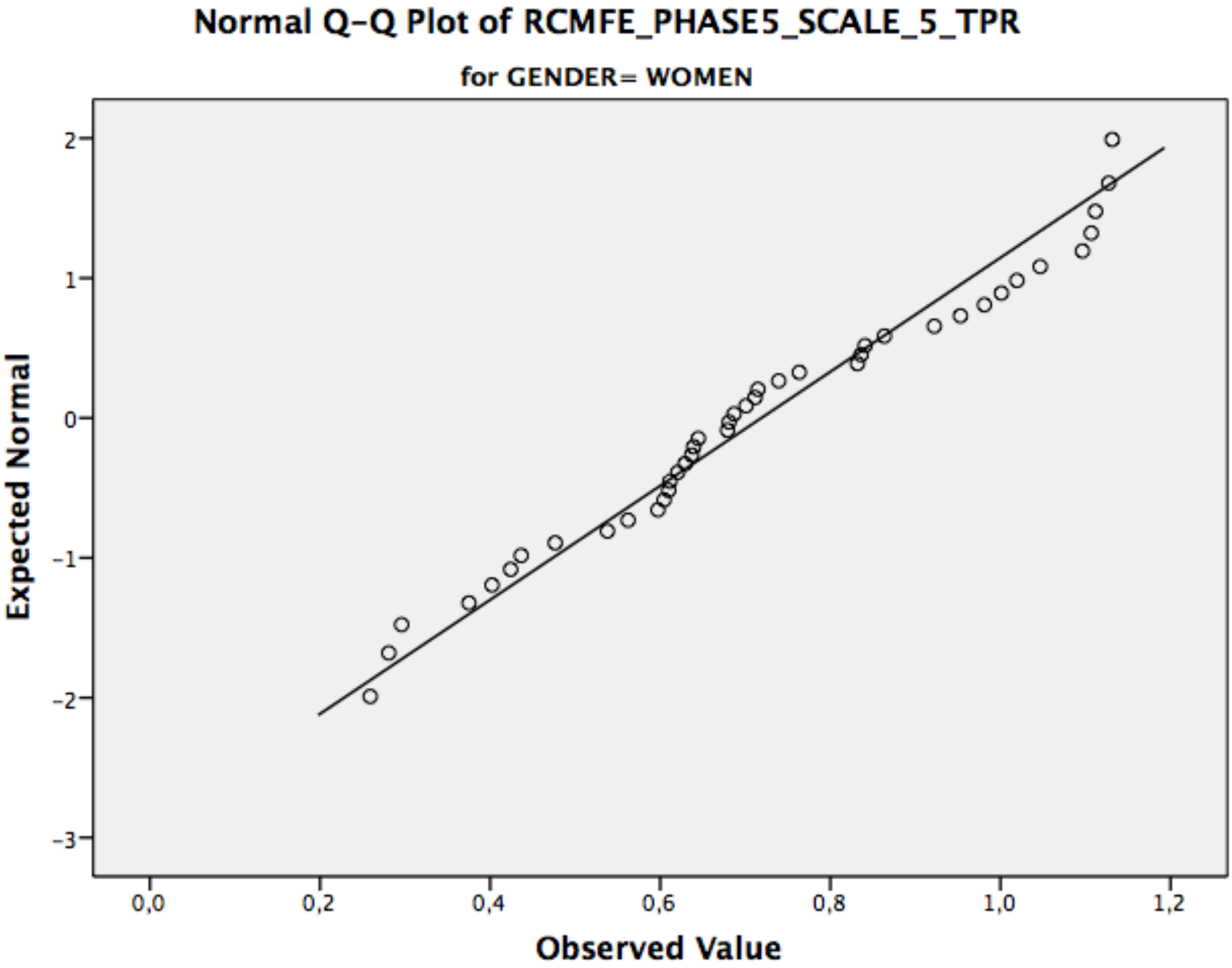

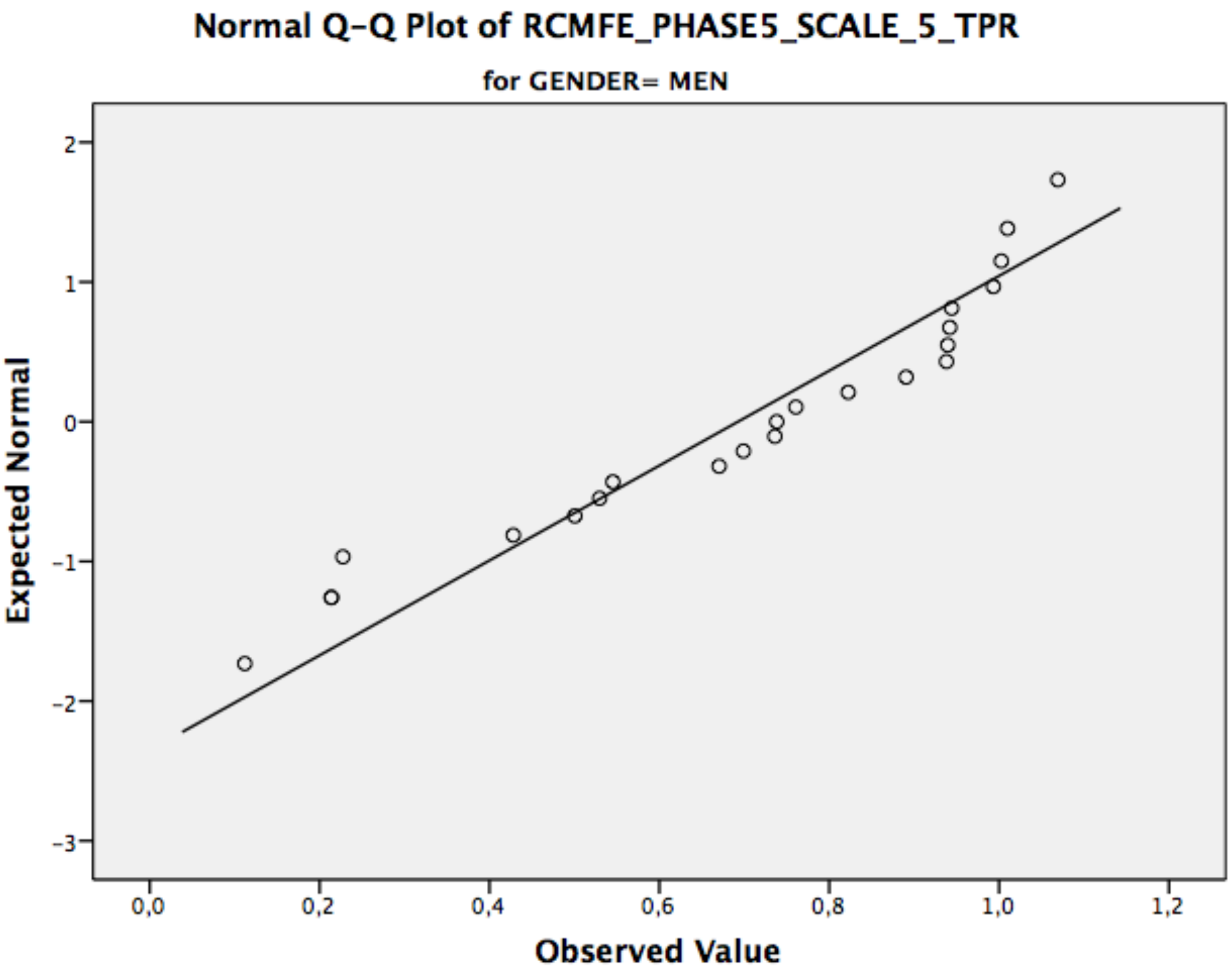

Normal Q-Q Plot of RCMFE_PHASE5_SCALE_5_TPR
for GENDER= MEN
Expected Normal
2
1
0
−1
−2
−3
0,0
0,2
0,4
0,6
0,8
1,0
1,2
Observed Value

Explore TPR

PATHOLOGY

Case Processing Summary

	PATHOLOGY	Cases					
		Valid		Missing		Total	
		N	Percent	N	Percent	N	Percent
RCMFE_PHASE1_SCALE_1_TPR	NO-OI	29	100,0%	0	0,0%	29	100,0%
	OI	36	100,0%	0	0,0%	36	100,0%
RCMFE_PHASE1_SCALE_2_TPR	NO-OI	29	100,0%	0	0,0%	29	100,0%
	OI	36	100,0%	0	0,0%	36	100,0%
RCMFE_PHASE1_SCALE_3_TPR	NO-OI	29	100,0%	0	0,0%	29	100,0%
	OI	36	100,0%	0	0,0%	36	100,0%
RCMFE_PHASE1_SCALE_4_TPR	NO-OI	29	100,0%	0	0,0%	29	100,0%
	OI	36	100,0%	0	0,0%	36	100,0%
RCMFE_PHASE1_SCALE_5_TPR	NO-OI	29	100,0%	0	0,0%	29	100,0%
	OI	36	100,0%	0	0,0%	36	100,0%
RCMFE_PHASE2_SCALE_1_TPR	NO-OI	29	100,0%	0	0,0%	29	100,0%
	OI	36	100,0%	0	0,0%	36	100,0%
RCMFE_PHASE2_SCALE_2_TPR	NO-OI	29	100,0%	0	0,0%	29	100,0%
	OI	36	100,0%	0	0,0%	36	100,0%
RCMFE_PHASE2_SCALE_3_TPR	NO-OI	29	100,0%	0	0,0%	29	100,0%
	OI	36	100,0%	0	0,0%	36	100,0%
RCMFE_PHASE2_SCALE_4_TPR	NO-OI	29	100,0%	0	0,0%	29	100,0%
	OI	36	100,0%	0	0,0%	36	100,0%
RCMFE_PHASE2_SCALE_5_TPR	NO-OI	29	100,0%	0	0,0%	29	100,0%
	OI	36	100,0%	0	0,0%	36	100,0%
RCMFE_PHASE3_SCALE_1_TPR	NO-OI	29	100,0%	0	0,0%	29	100,0%
	OI	36	100,0%	0	0,0%	36	100,0%
RCMFE_PHASE3_SCALE_2_TPR	NO-OI	29	100,0%	0	0,0%	29	100,0%
	OI	36	100,0%	0	0,0%	36	100,0%
RCMFE_PHASE3_SCALE_3_TPR	NO-OI	29	100,0%	0	0,0%	29	100,0%
	OI	36	100,0%	0	0,0%	36	100,0%
RCMFE_PHASE3_SCALE_4_TPR	NO-OI	29	100,0%	0	0,0%	29	100,0%
	OI	36	100,0%	0	0,0%	36	100,0%
RCMFE_PHASE3_SCALE_5_TPR	NO-OI	29	100,0%	0	0,0%	29	100,0%
	OI	36	100,0%	0	0,0%	36	100,0%
RCMFE_PHASE4_SCALE_1_TPR	NO-OI	29	100,0%	0	0,0%	29	100,0%
	OI	36	100,0%	0	0,0%	36	100,0%
RCMFE_PHASE4_SCALE_2_TPR	NO-OI	29	100,0%	0	0,0%	29	100,0%
	OI	36	100,0%	0	0,0%	36	100,0%
RCMFE_PHASE4_SCALE_3	NO-OI	29	100,0%	0	0,0%	29	100,0%
	OI	36	100,0%	0	0,0%	36	100,0%
RCMFE_PHASE4_SCALE_4_TPR	NO-OI	29	100,0%	0	0,0%	29	100,0%
	OI	36	100,0%	0	0,0%	36	100,0%
RCMFE_PHASE4_SCALE_5_TPR	NO-OI	29	100,0%	0	0,0%	29	100,0%
	OI	36	100,0%	0	0,0%	36	100,0%
RCMFE_PHASE5_SCALE_1_TPR	NO-OI	29	100,0%	0	0,0%	29	100,0%

Case Processing Summary

	PATHOLOGY	Cases					
		Valid		Missing		Total	
		N	Percent	N	Percent	N	Percent
RCMFE_PHASE5_SCALE_1_TPR	OI	36	100,0%	0	0,0%	36	100,0%
RCMFE_PHASE5_SCALE_2_TPR	NO-OI	29	100,0%	0	0,0%	29	100,0%
	OI	36	100,0%	0	0,0%	36	100,0%
RCMFE_PHASE5_SCALE_3_TPR	NO-OI	29	100,0%	0	0,0%	29	100,0%
	OI	36	100,0%	0	0,0%	36	100,0%
RCMFE_PHASE5_SCALE_4_TPR	NO-OI	29	100,0%	0	0,0%	29	100,0%
	OI	36	100,0%	0	0,0%	36	100,0%
RCMFE_PHASE5_SCALE_5_TPR	NO-OI	29	100,0%	0	0,0%	29	100,0%
	OI	36	100,0%	0	0,0%	36	100,0%

Tests of Normality

	PATHOLOGY	Kolmogorov-Smirnov[a]			Shapiro-Wilk		
		Statistic	df	Sig.	Statistic	df	Sig.
RCMFE_PHASE1_SCALE_1_TPR	NO-OI	,127	29	,200*	,975	29	,713
	OI	,138	36	,079	,912	36	,007
RCMFE_PHASE1_SCALE_2_TPR	NO-OI	,090	29	,200*	,967	29	,474
	OI	,126	36	,159	,956	36	,163
RCMFE_PHASE1_SCALE_3_TPR	NO-OI	,097	29	,200*	,959	29	,309
	OI	,110	36	,200*	,972	36	,491
RCMFE_PHASE1_SCALE_4_TPR	NO-OI	,115	29	,200*	,956	29	,257
	OI	,114	36	,200*	,971	36	,450
RCMFE_PHASE1_SCALE_5_TPR	NO-OI	,070	29	,200*	,980	29	,836
	OI	,092	36	,200*	,982	36	,807
RCMFE_PHASE2_SCALE_1_TPR	NO-OI	,152	29	,084	,945	29	,136
	OI	,073	36	,200*	,971	36	,455
RCMFE_PHASE2_SCALE_2_TPR	NO-OI	,105	29	,200*	,951	29	,196
	OI	,079	36	,200*	,976	36	,615
RCMFE_PHASE2_SCALE_3_TPR	NO-OI	,105	29	,200*	,964	29	,413
	OI	,104	36	,200*	,955	36	,147
RCMFE_PHASE2_SCALE_4_TPR	NO-OI	,118	29	,200*	,959	29	,303
	OI	,114	36	,200*	,944	36	,069
RCMFE_PHASE2_SCALE_5_TPR	NO-OI	,118	29	,200*	,956	29	,266
	OI	,088	36	,200*	,928	36	,021
RCMFE_PHASE3_SCALE_1_TPR	NO-OI	,098	29	,200*	,976	29	,715
	OI	,114	36	,200*	,967	36	,343
RCMFE_PHASE3_SCALE_2_TPR	NO-OI	,090	29	,200*	,984	29	,928
	OI	,084	36	,200*	,981	36	,782
RCMFE_PHASE3_SCALE_3_TPR	NO-OI	,068	29	,200*	,988	29	,981
	OI	,160	36	,020	,928	36	,022
RCMFE_PHASE3_SCALE_4_TPR	NO-OI	,072	29	,200*	,986	29	,961
	OI	,153	36	,033	,953	36	,126
RCMFE_PHASE3_SCALE_5	NO-OI	,116	29	,200*	,974	29	,660

_TPR	OI	,090	36	,200*	,977	36	,654
RCMFE_PHASE4_SCALE_1	NO-OI	,225	29	,001	,797	29	,050
_TPR	OI	,196	36	,001	,814	36	,053
RCMFE_PHASE4_SCALE_2	NO-OI	,170	29	,031	,835	29	,055
_TPR	OI	,145	36	,055	,868	36	,001
RCMFE_PHASE4_SCALE_3	NO-OI	,172	29	,027	,865	29	,072
	OI	,180	36	,005	,846	36	,052
RCMFE_PHASE4_SCALE_4	NO-OI	,147	29	,108	,947	29	,157
_TPR	OI	,215	36	,000	,854	36	,055
RCMFE_PHASE4_SCALE_5	NO-OI	,189	29	,010	,893	29	,067
_TPR	OI	,170	36	,010	,831	36	,065
RCMFE_PHASE5_SCALE_1	NO-OI	,108	29	,200*	,971	29	,594
_TPR	OI	,093	36	,200*	,963	36	,273

Tests of Normality

	PATHOLOGY	Kolmogorov-Smirnov[a]			Shapiro-Wilk		
		Statistic	df	Sig.	Statistic	df	Sig.
RCMFE_PHASE5_SCALE_2	NO-OI	,112	29	,200*	,953	29	,224
_TPR	OI	,087	36	,200	,963	36	,262
RCMFE_PHASE5_SCALE_3	NO-OI	,128	29	,200*	,942	29	,114
_TPR	OI	,082	36	,200	,979	36	,708
RCMFE_PHASE5_SCALE_4	NO-OI	,125	29	,200*	,949	29	,170
_TPR	OI	,125	36	,166*	,965	36	,297
RCMFE_PHASE5_SCALE_5	NO-OI	,100	29	,200*	,953	29	,213
_TPR	OI	,089	36	,200*	,975	36	,580

*. This is a lower bound of the true significance.

a. Lilliefors Significance Correction

RCMFE_PHASE1_SCALE_1_TPR

Normal Q-Q Plots

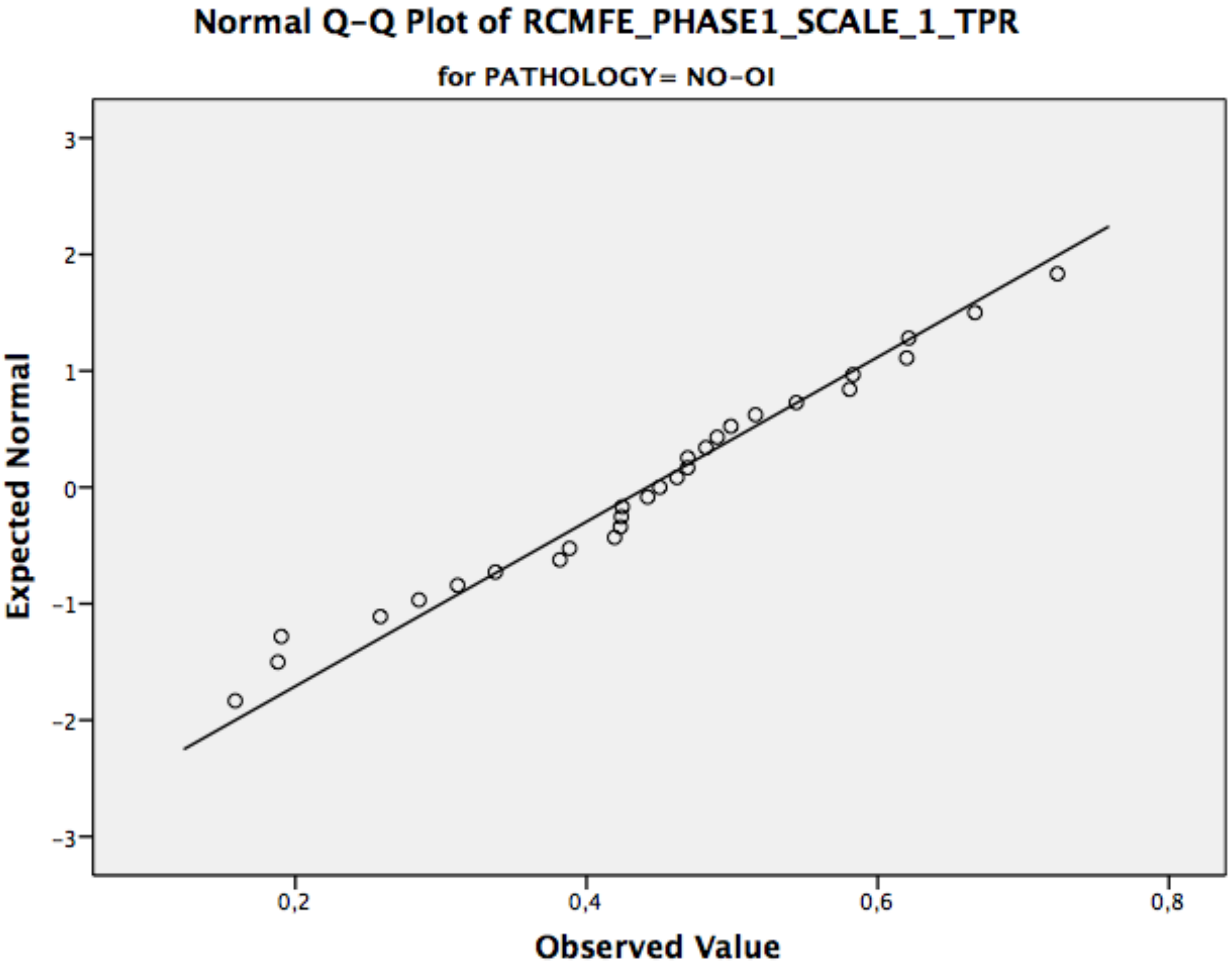

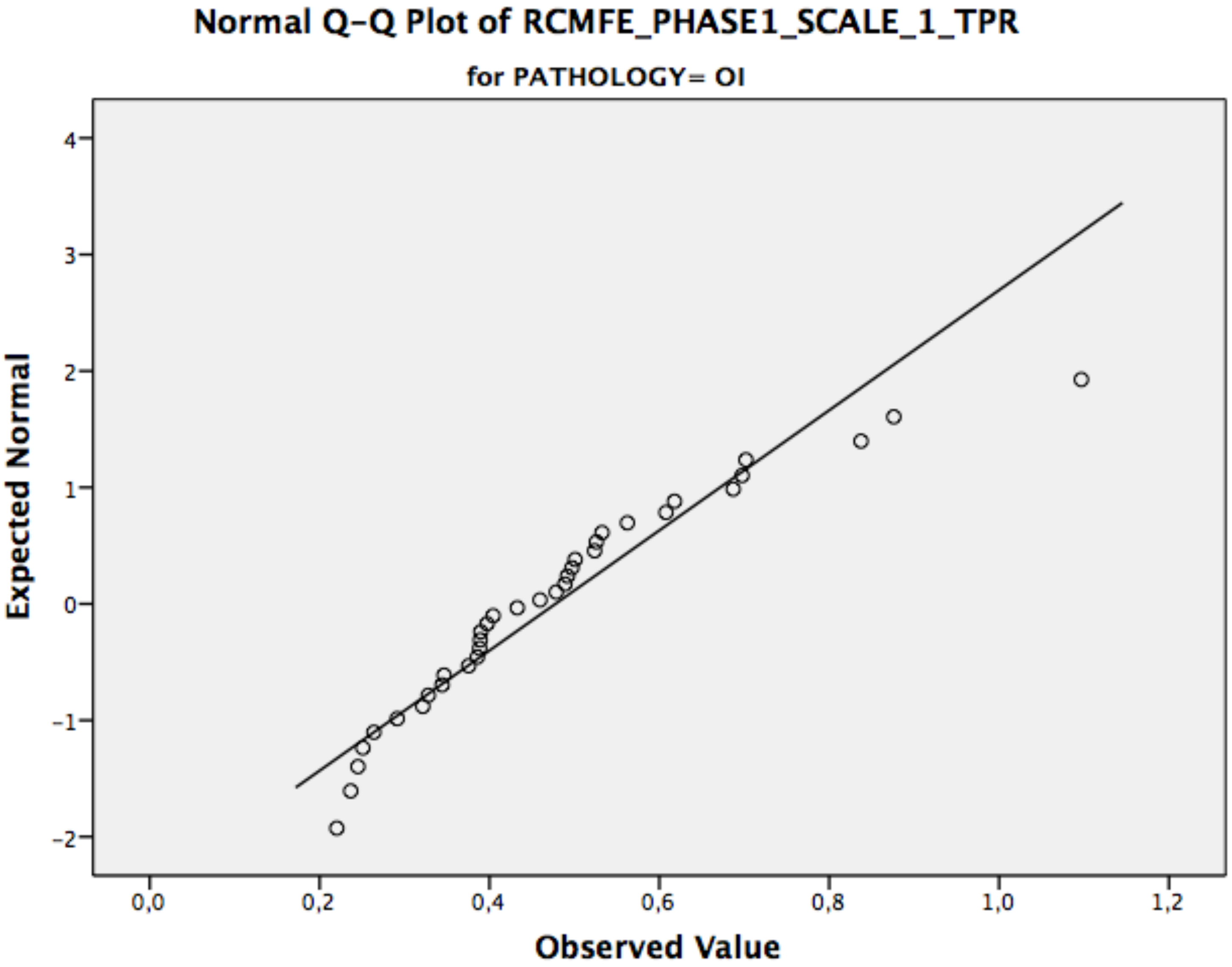
Normal Q-Q Plot of RCMFE_PHASE1_SCALE_1_TPR
for PATHOLOGY= OI
Expected Normal
Observed Value
4
3
2
1
0
-1
-2
0,0
0,2
0,4
0,6
0,8
1,0
1,2

RCMFE_PHASE1_SCALE_2_TPR

Normal Q-Q Plots

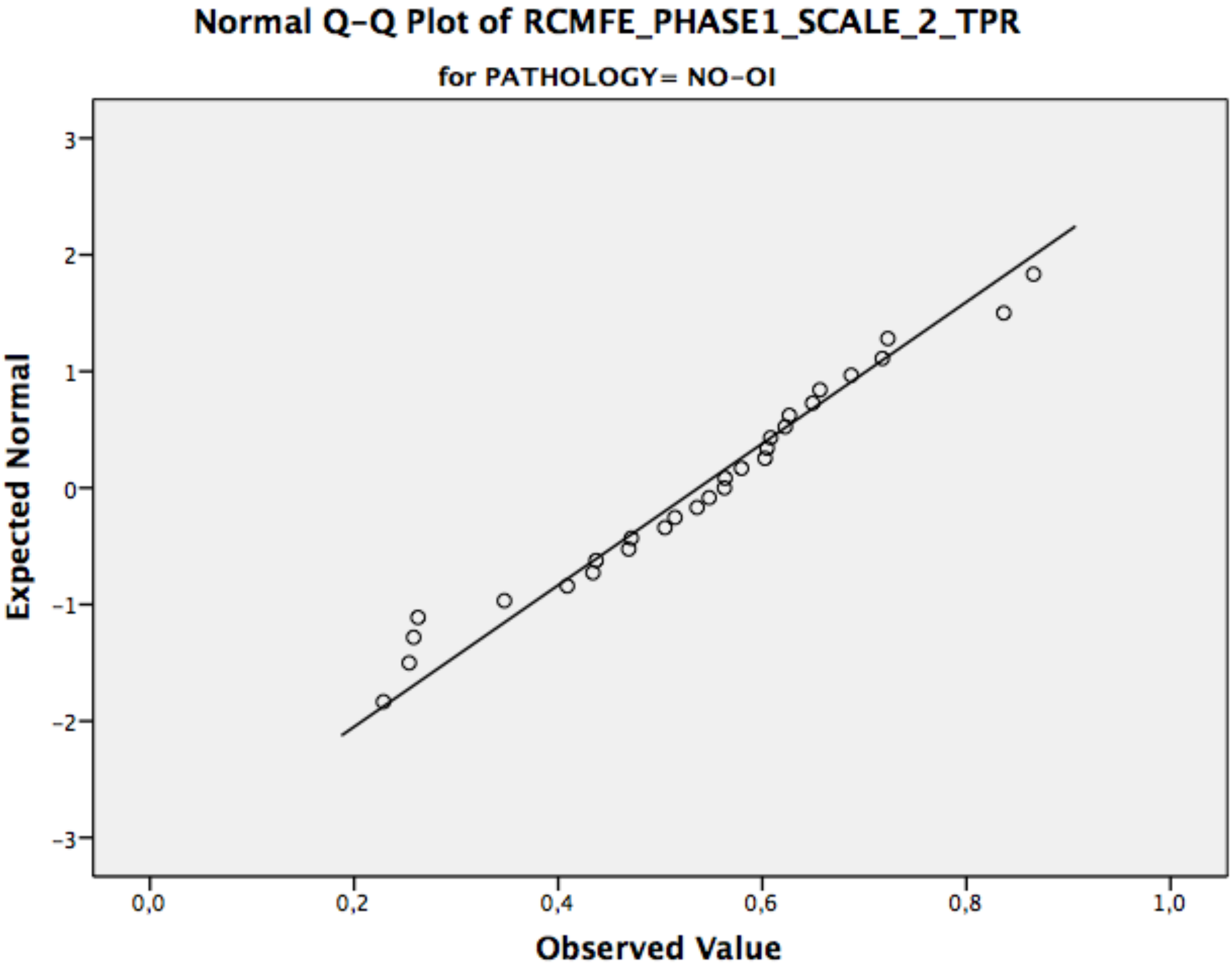

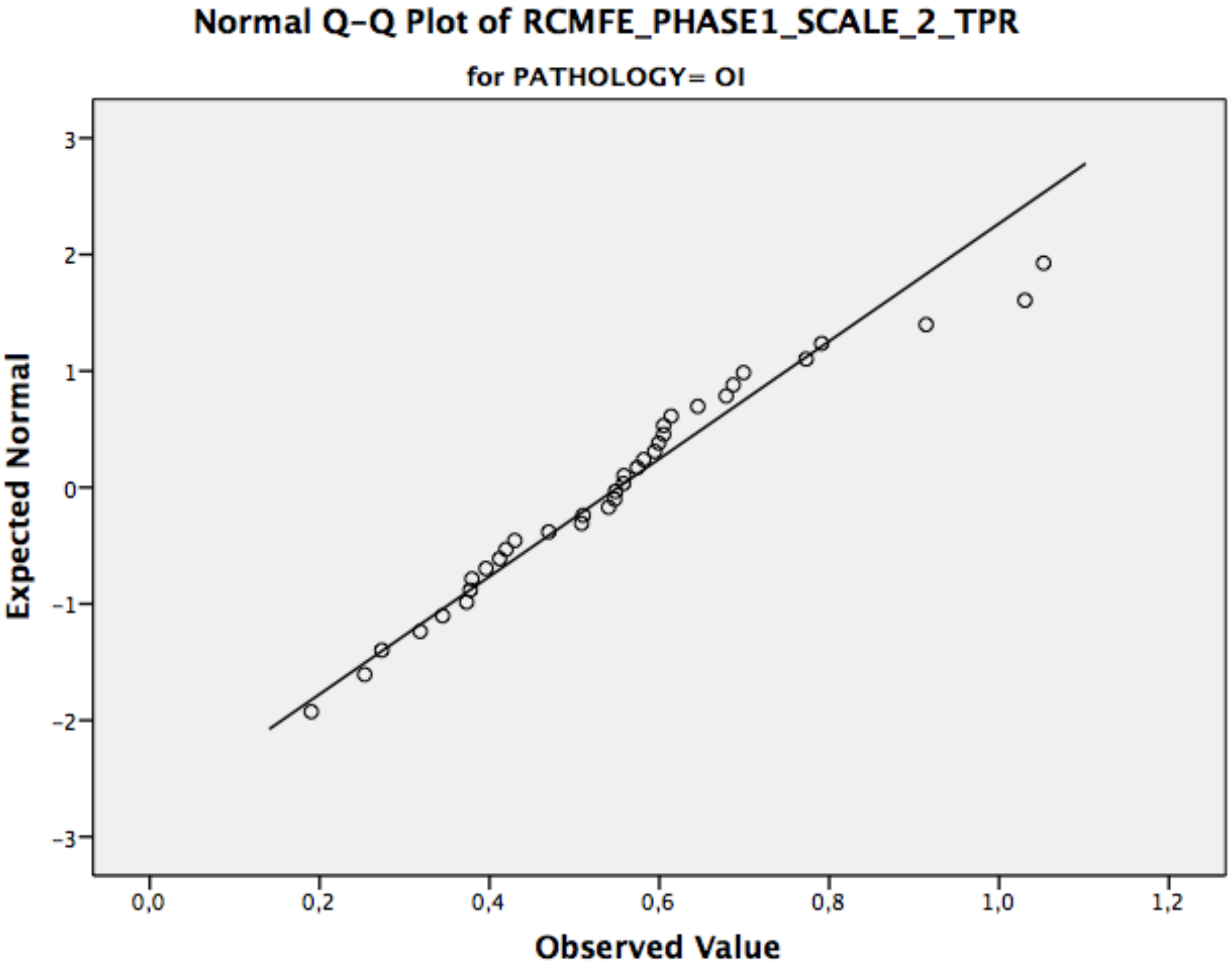

Normal Q-Q Plot of RCMFE_PHASE1_SCALE_2_TPR
for PATHOLOGY= OI
Expected Normal
3
2
1
0
−1
−2
−3
0,0
0,2
0,4
0,6
0,8
1,0
1,2
Observed Value

RCMFE_PHASE1_SCALE_3_TPR

Normal Q-Q Plots

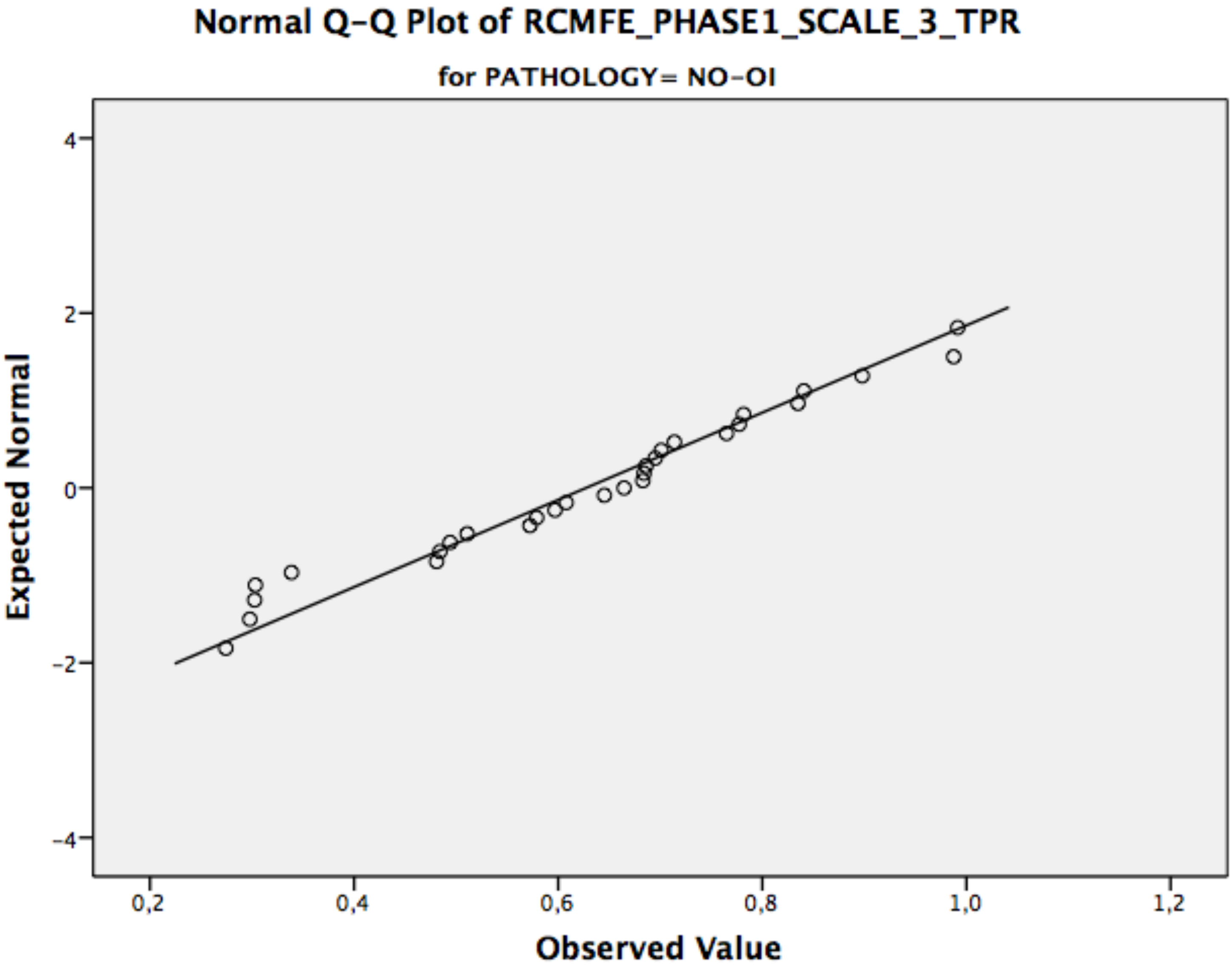

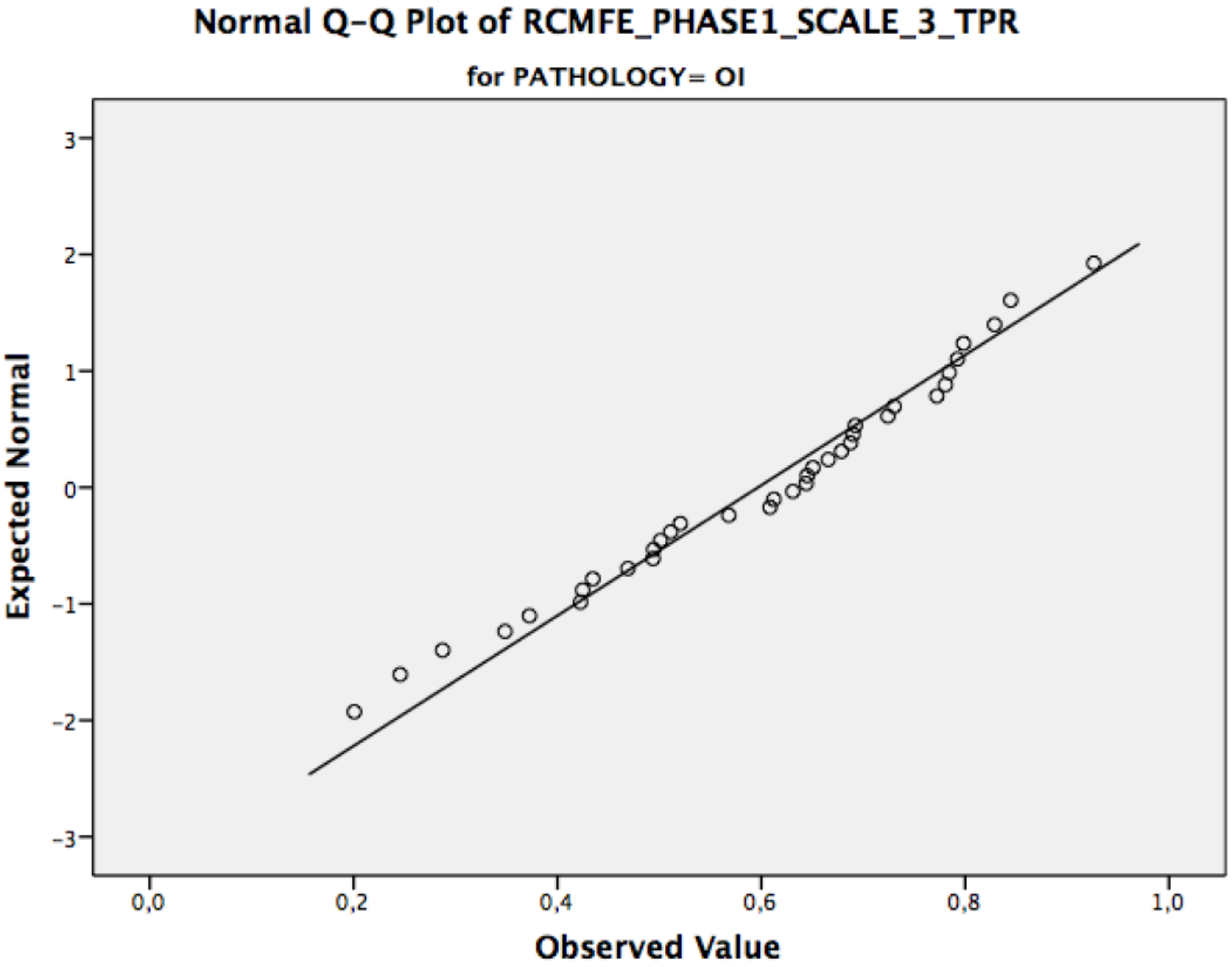
Normal Q-Q Plot of RCMFE_PHASE1_SCALE_3_TPR
for PATHOLOGY= OI
Expected Normal
3
2
1
0
-1
-2
-3
0,0
0,2
0,4
0,6
0,8
1,0
Observed Value

RCMFE_PHASE1_SCALE_4_TPR

Normal Q-Q Plots

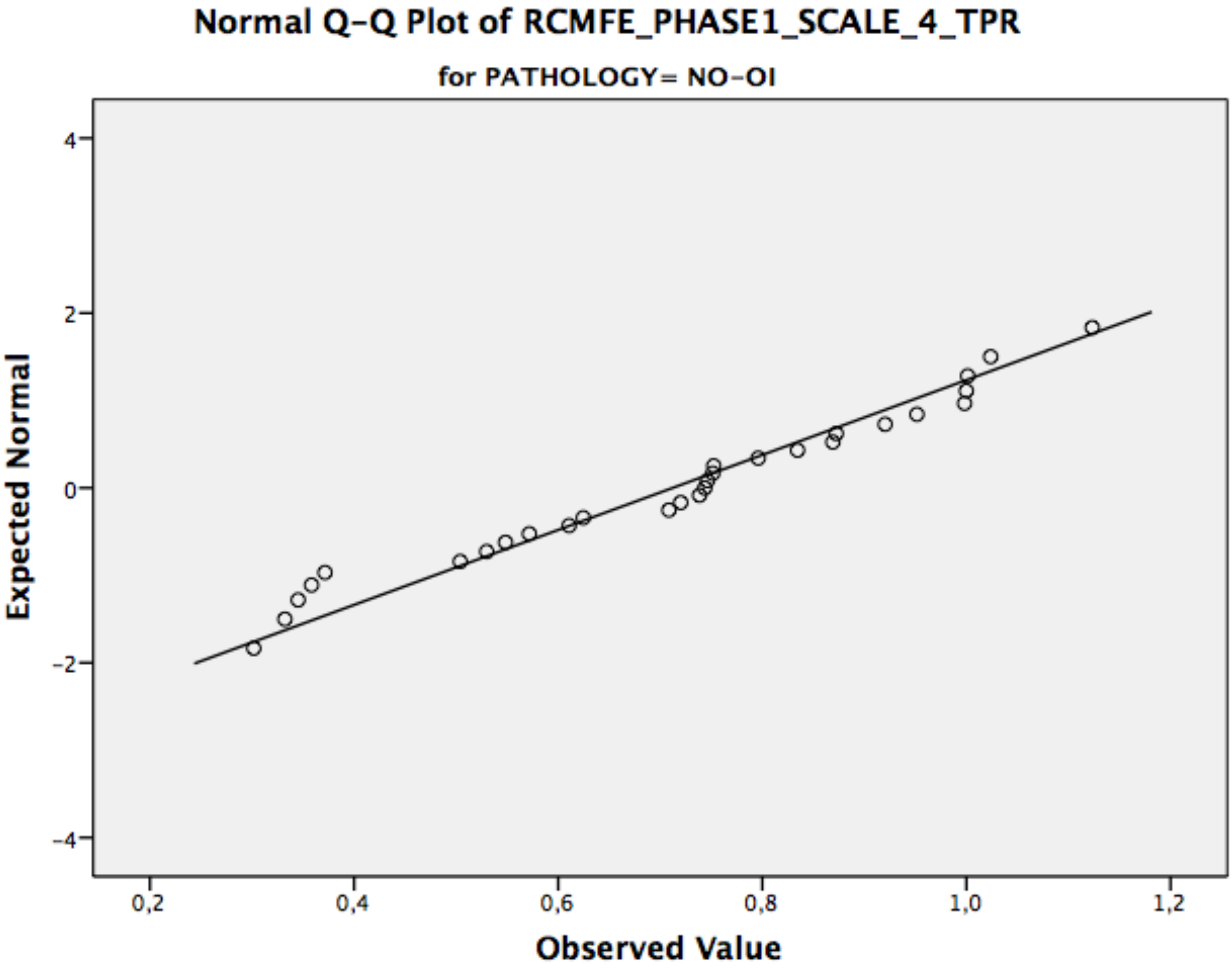

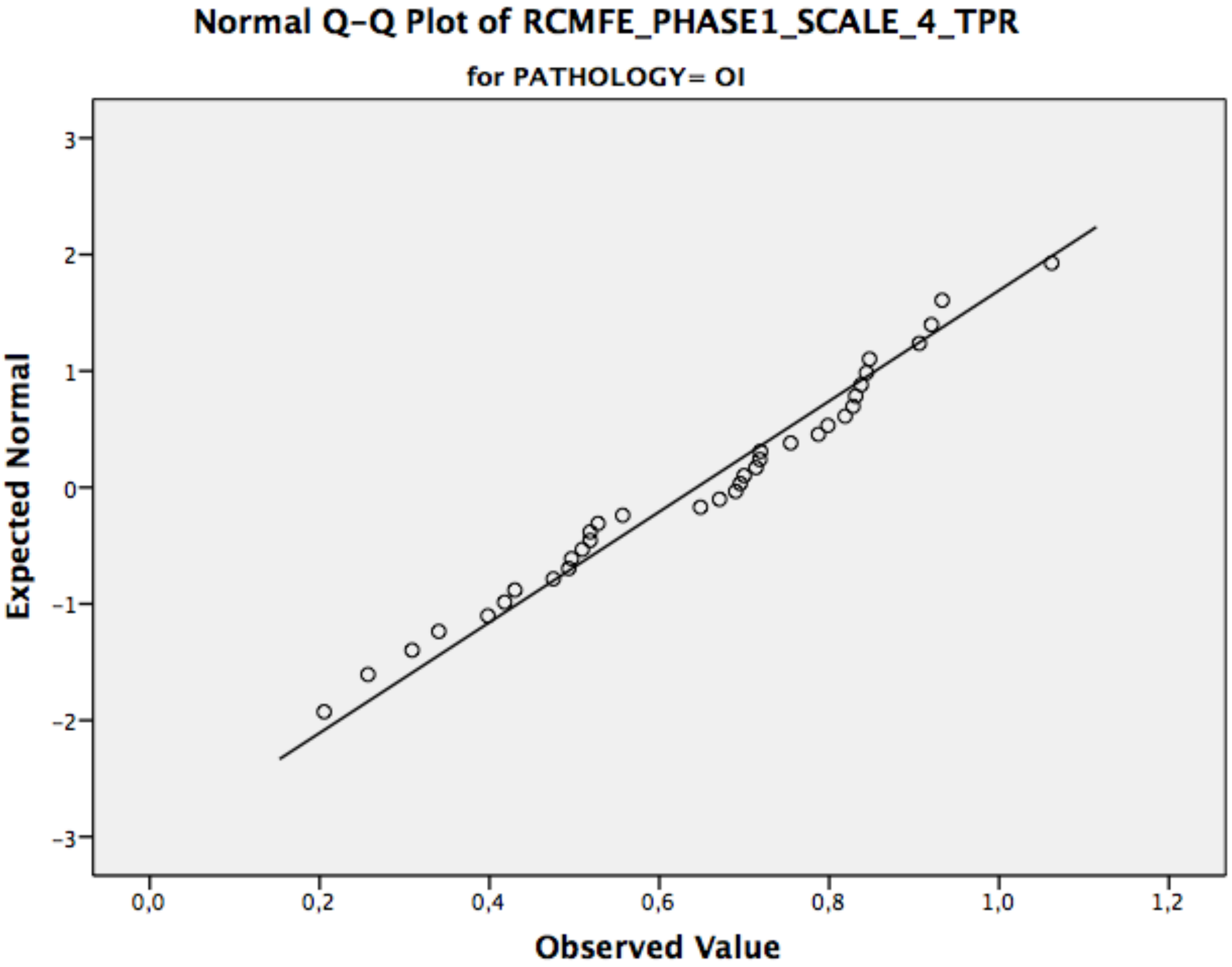

Normal Q-Q Plot of RCMFE_PHASE1_SCALE_4_TPR
for PATHOLOGY= OI
Expected Normal
3
2
1
0
-1
-2
-3
0,0
0,2
0,4
0,6
0,8
1,0
1,2
Observed Value

RCMFE_PHASE1_SCALE_5_TPR

Normal Q-Q Plots

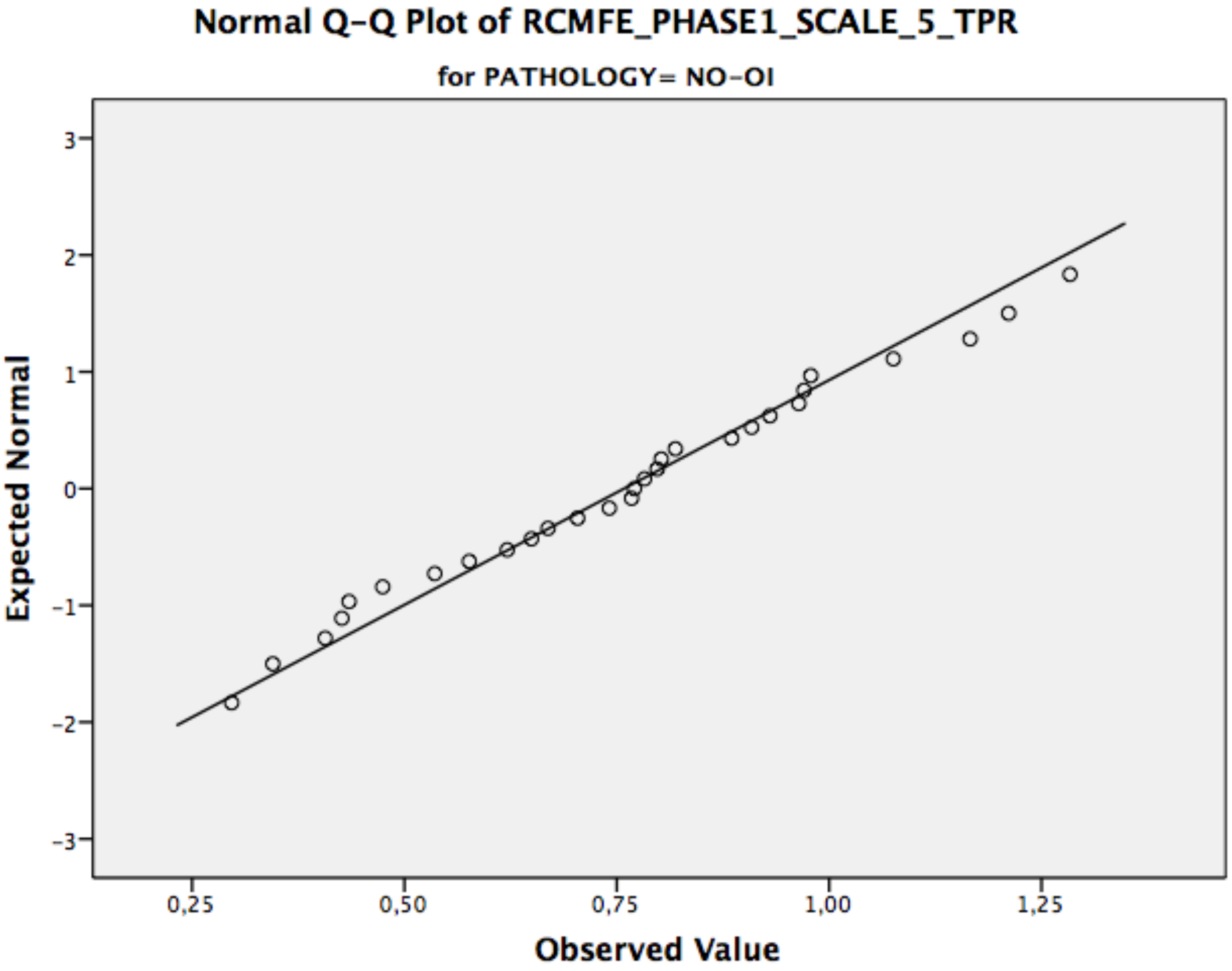

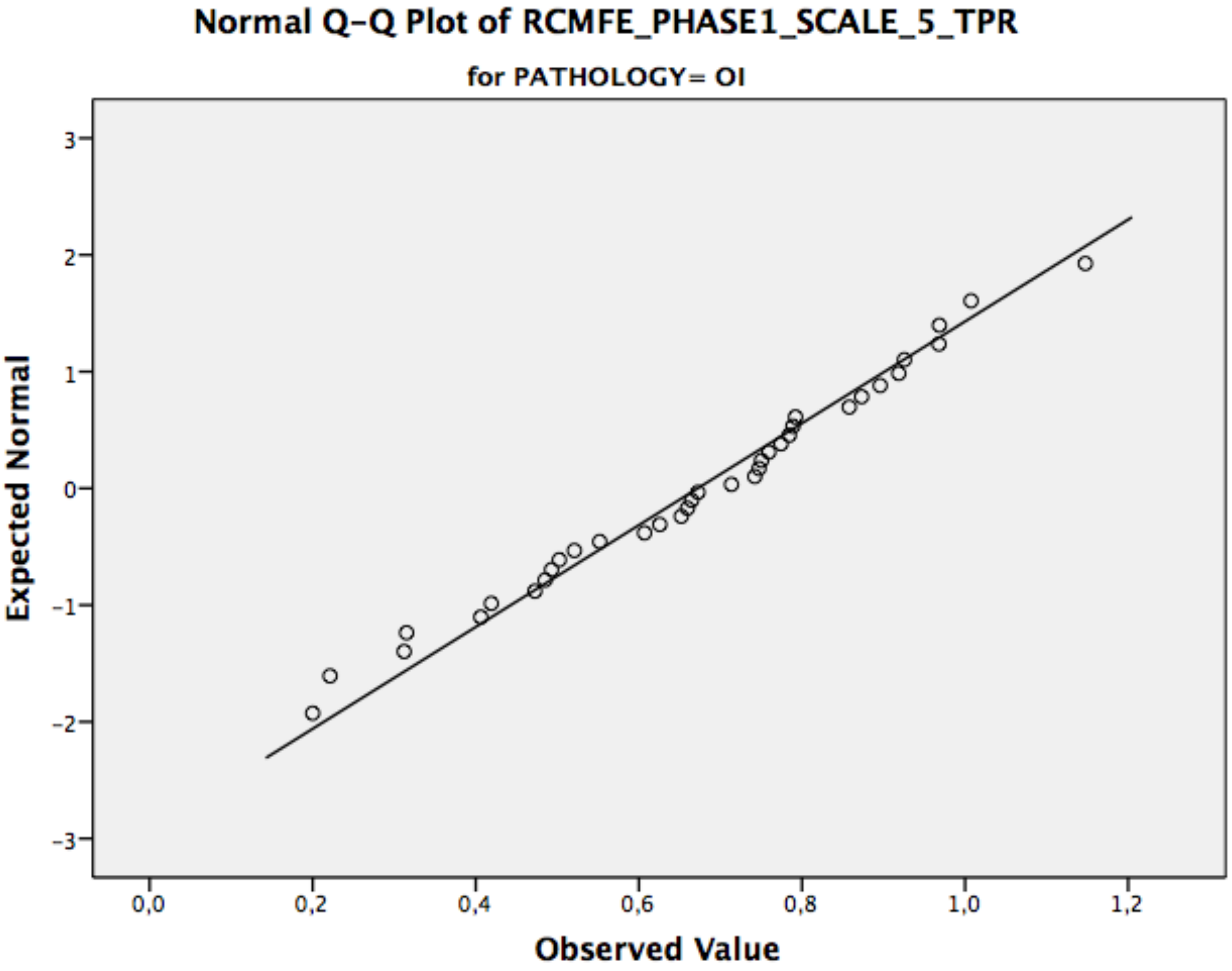
Normal Q-Q Plot of RCMFE_PHASE1_SCALE_5_TPR
for PATHOLOGY= OI
Expected Normal
Observed Value
3
2
1
0
−1
−2
−3
0,0
0,2
0,4
0,6
0,8
1,0
1,2

RCMFE_PHASE2_SCALE_1_TPR

Normal Q-Q Plots

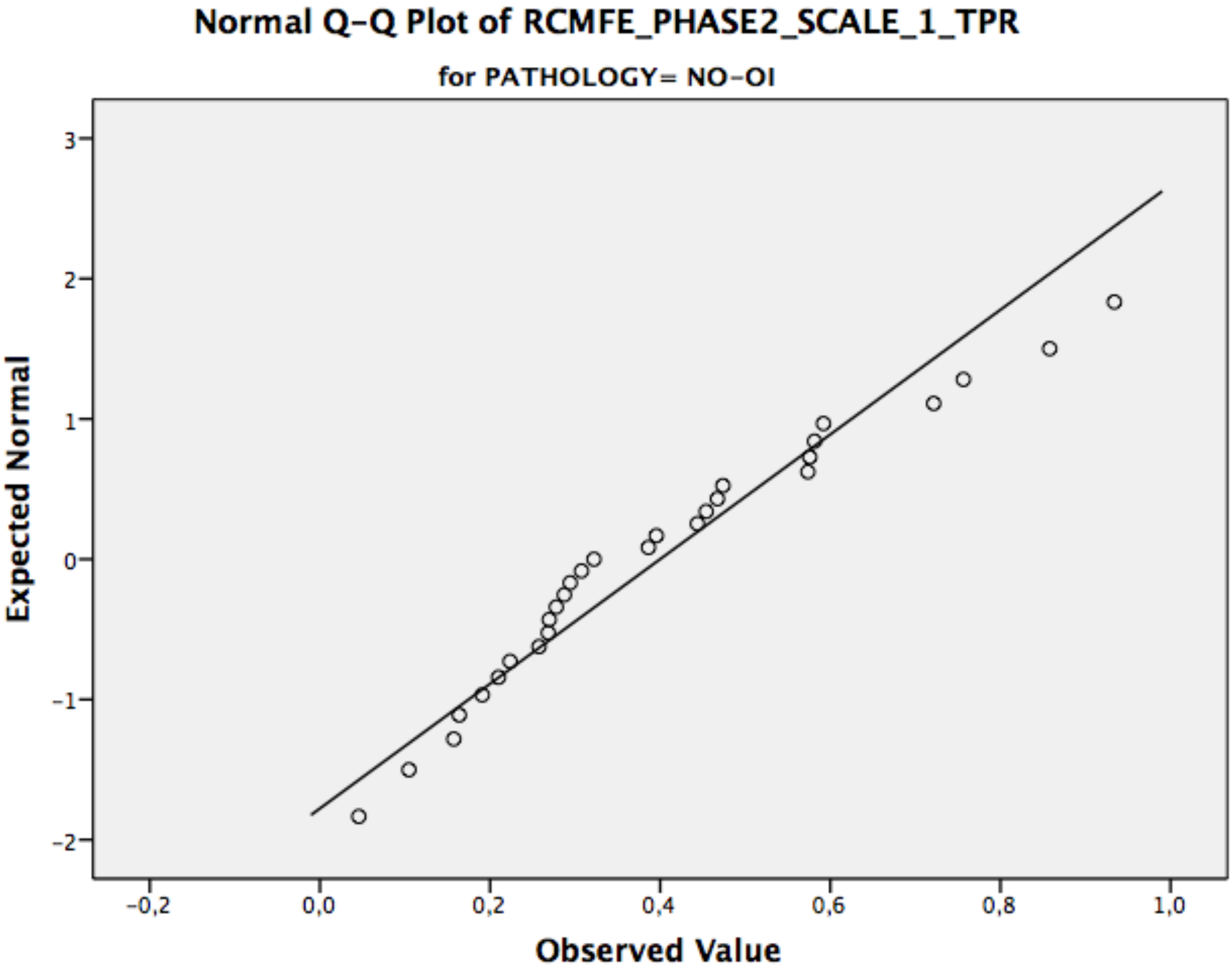

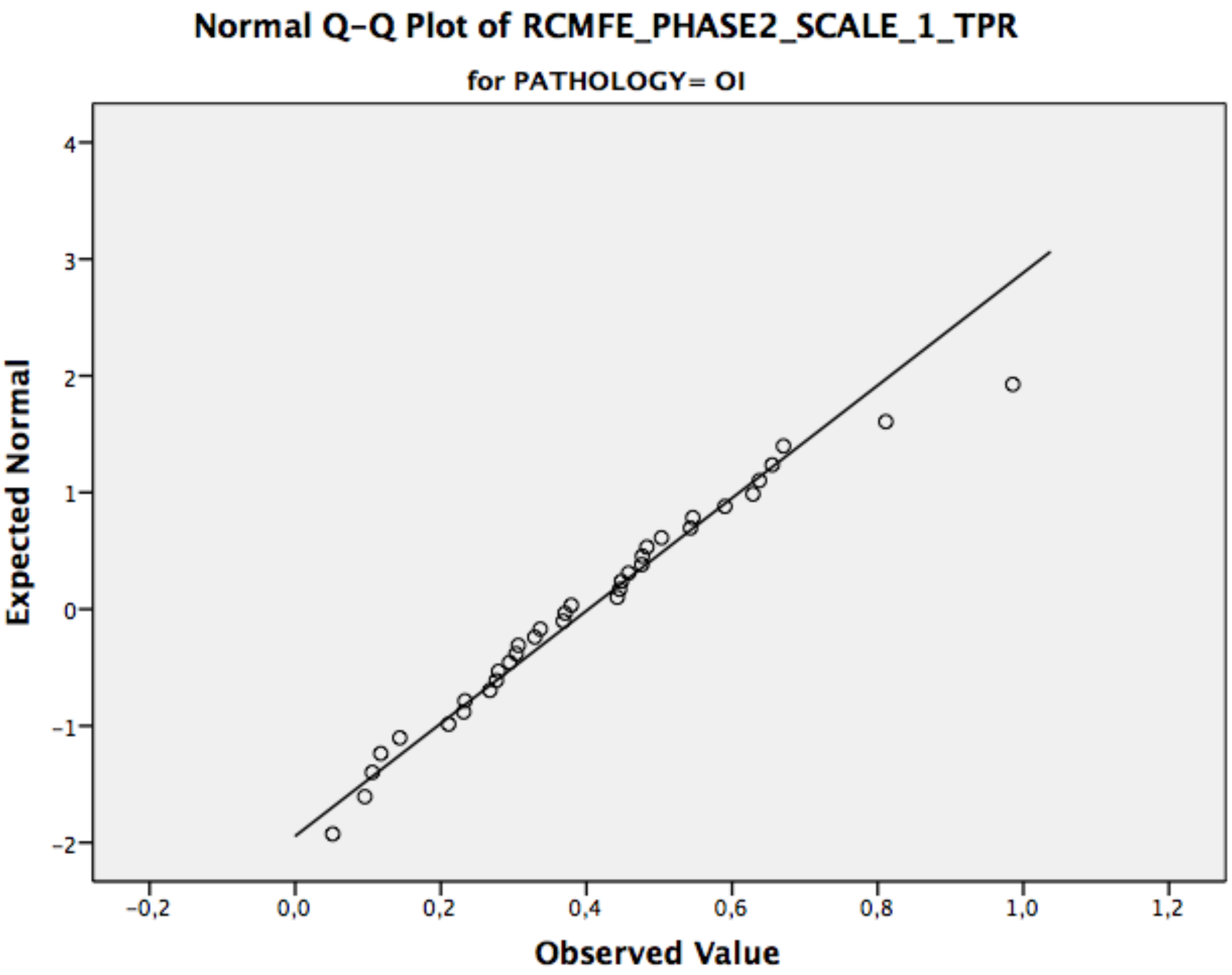

Normal Q-Q Plot of RCMFE_PHASE2_SCALE_1_TPR
for PATHOLOGY= OI
Expected Normal
4
3
2
1
0
-1
-2
-0,2
0,0
0,2
0,4
0,6
0,8
1,0
1,2
Observed Value

RCMFE_PHASE2_SCALE_2_TPR

Normal Q-Q Plots

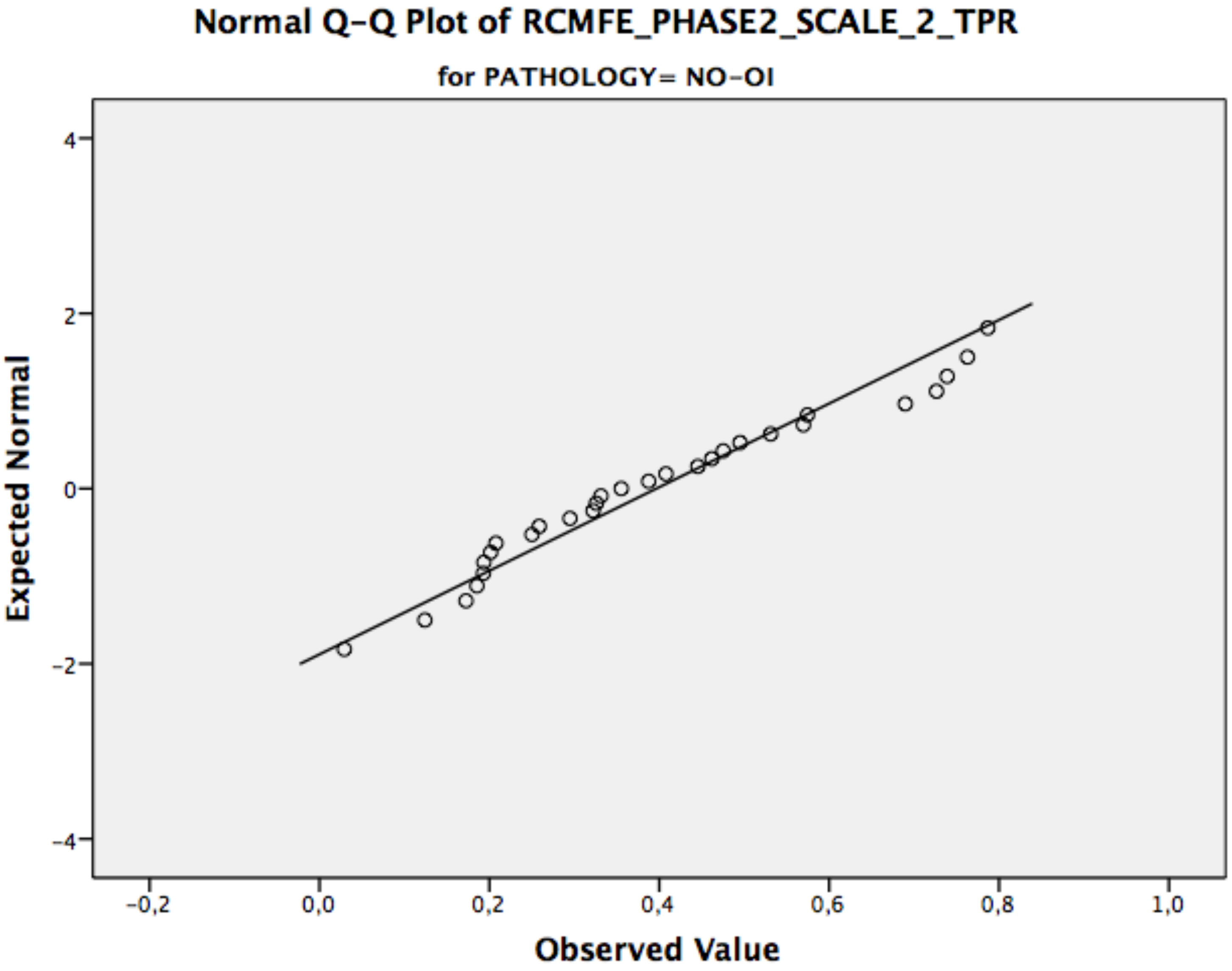

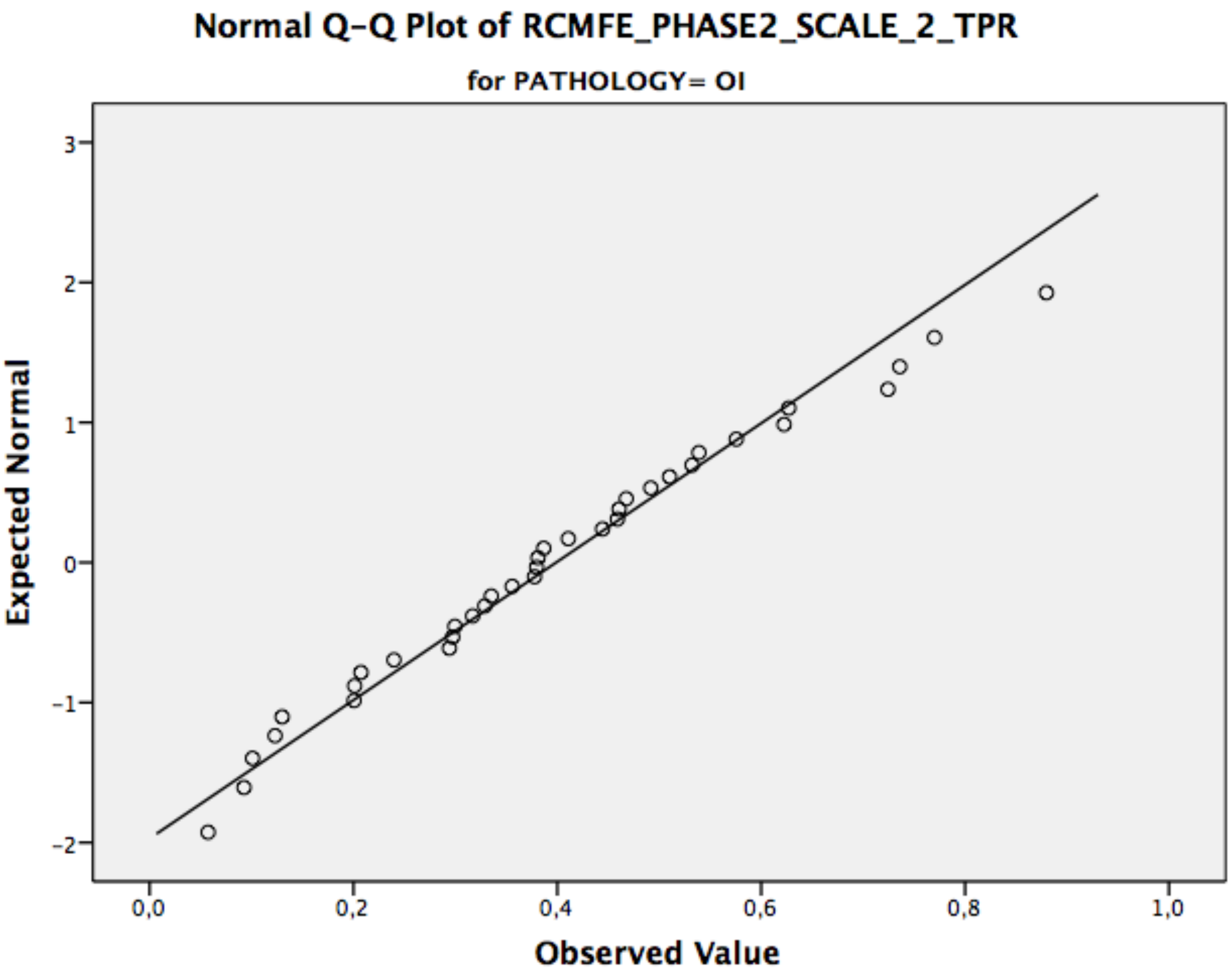
Normal Q-Q Plot of RCMFE_PHASE2_SCALE_2_TPR
for PATHOLOGY= OI
Expected Normal
Observed Value
3
2
1
0
-1
-2
0,0
0,2
0,4
0,6
0,8
1,0

RCMFE_PHASE2_SCALE_3_TPR

Normal Q-Q Plots

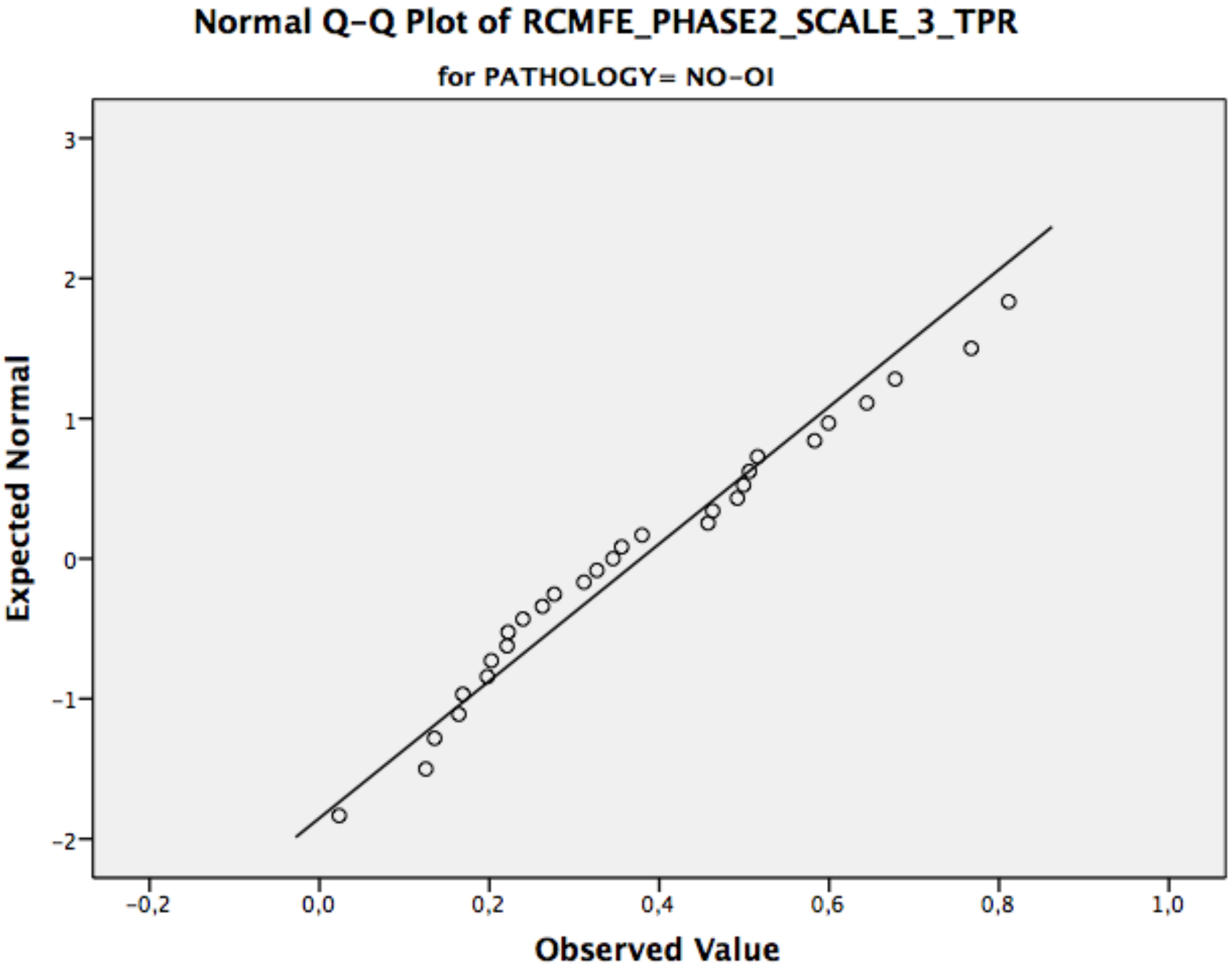

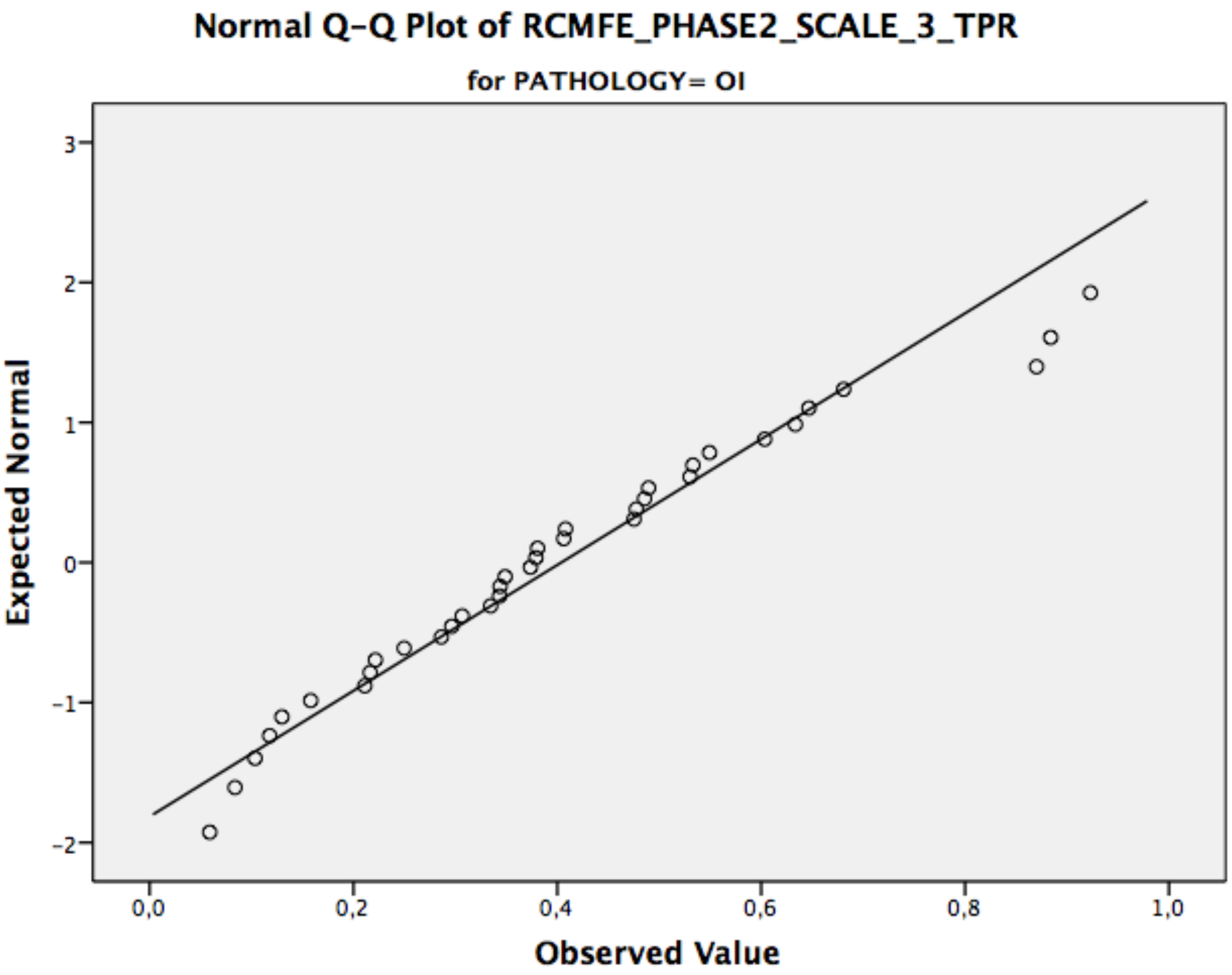

Normal Q-Q Plot of RCMFE_PHASE2_SCALE_3_TPR
for PATHOLOGY= OI
Expected Normal
Observed Value
3
2
1
0
-1
-2
0,0
0,2
0,4
0,6
0,8
1,0

RCMFE_PHASE2_SCALE_4_TPR

Normal Q-Q Plots

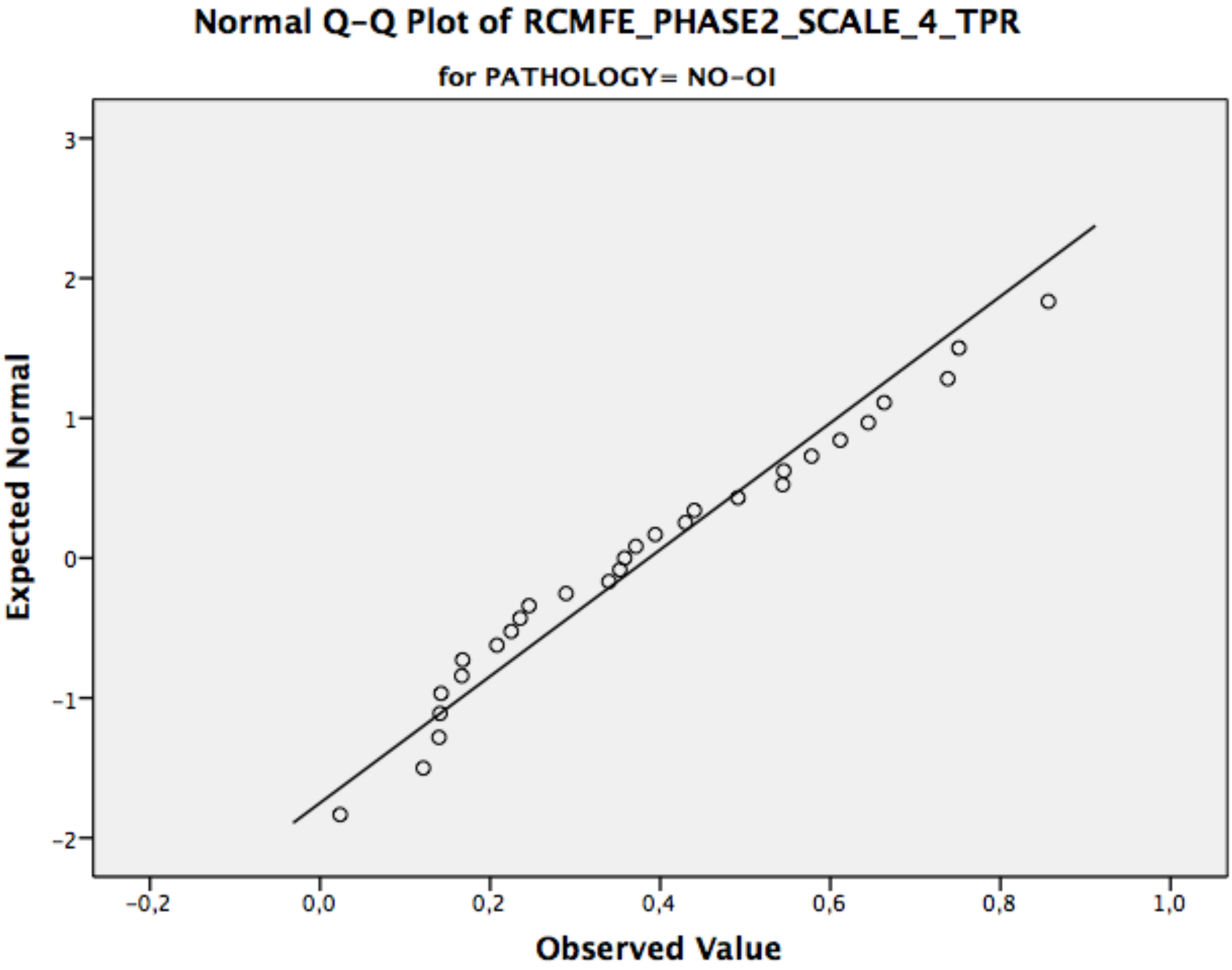

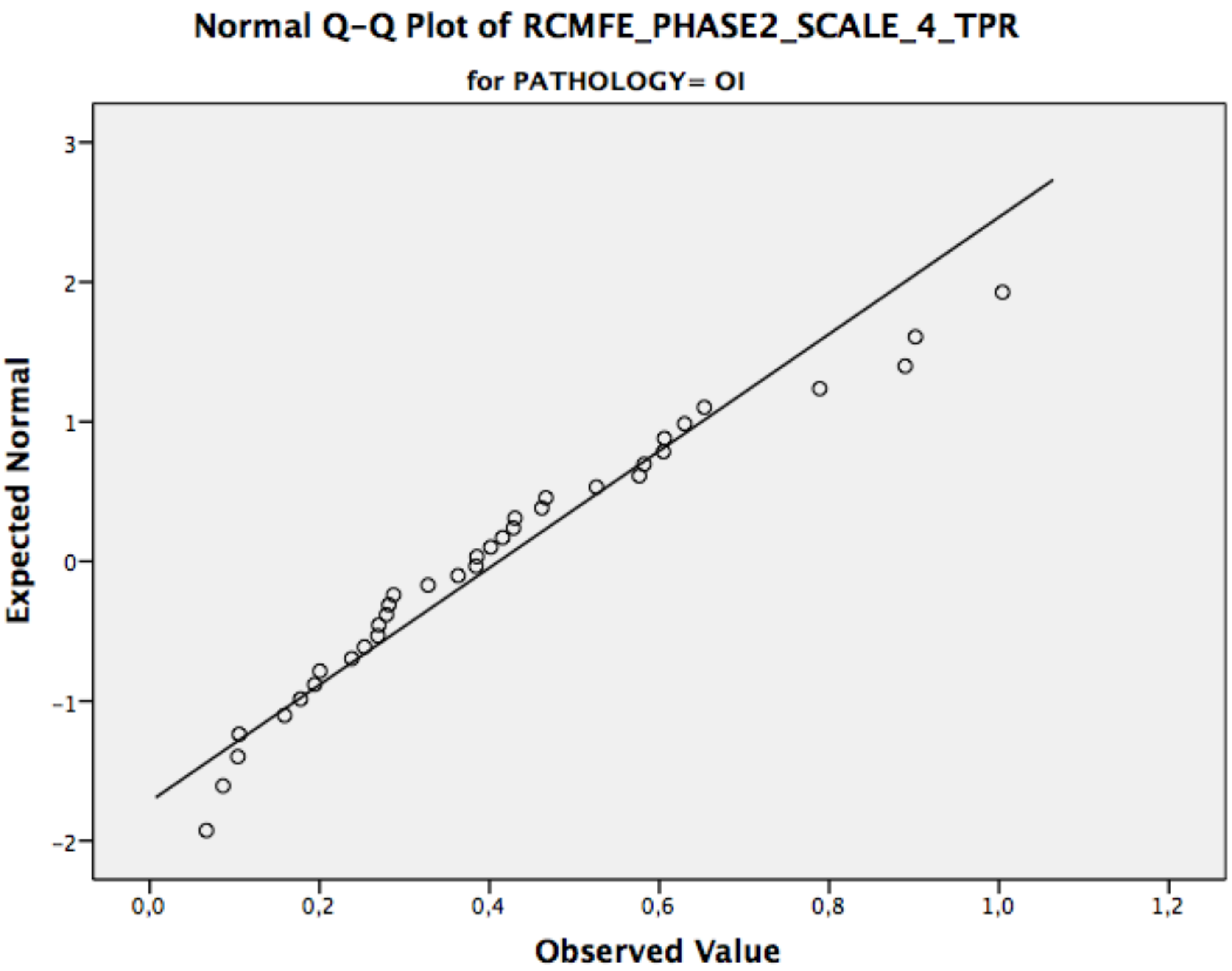
Normal Q-Q Plot of RCMFE_PHASE2_SCALE_4_TPR
for PATHOLOGY= OI
Expected Normal
Observed Value
3
2
1
0
-1
-2
0,0
0,2
0,4
0,6
0,8
1,0
1,2

RCMFE_PHASE2_SCALE_5_TPR

Normal Q-Q Plots

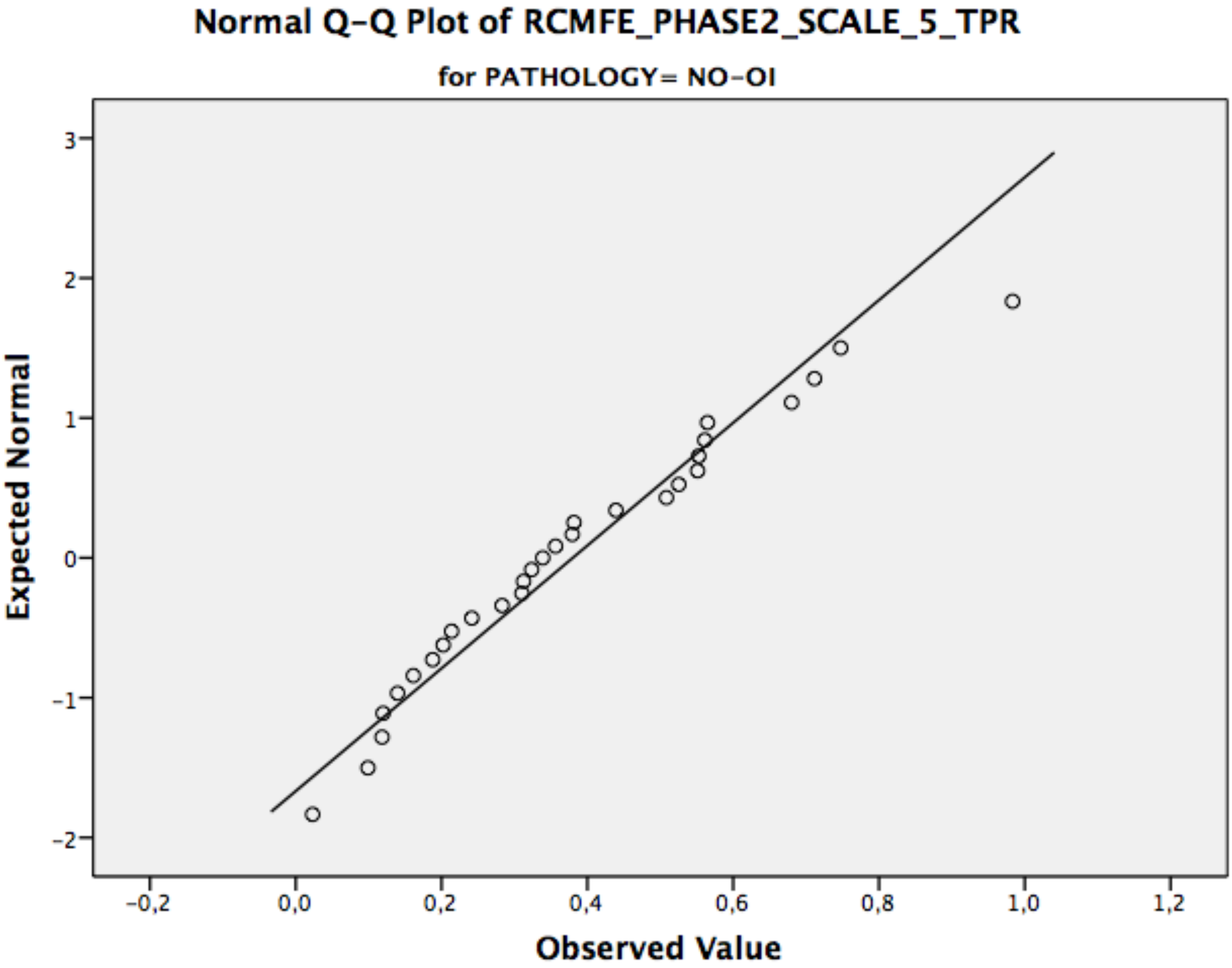

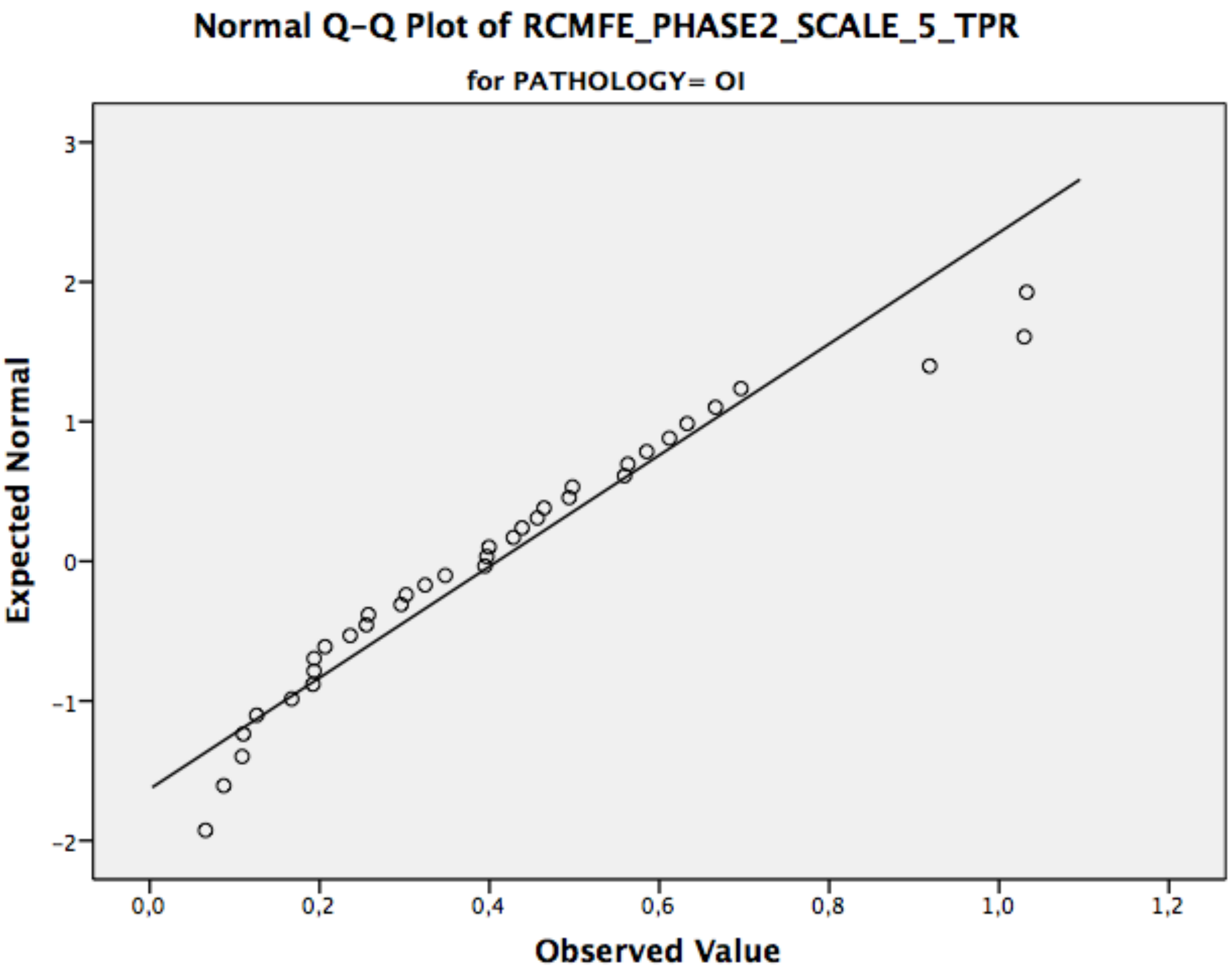

Normal Q-Q Plot of RCMFE_PHASE2_SCALE_5_TPR
for PATHOLOGY= OI
Expected Normal
Observed Value
3
2
1
0
-1
-2
0,0
0,2
0,4
0,6
0,8
1,0
1,2

RCMFE_PHASE3_SCALE_1_TPR

Normal Q-Q Plots

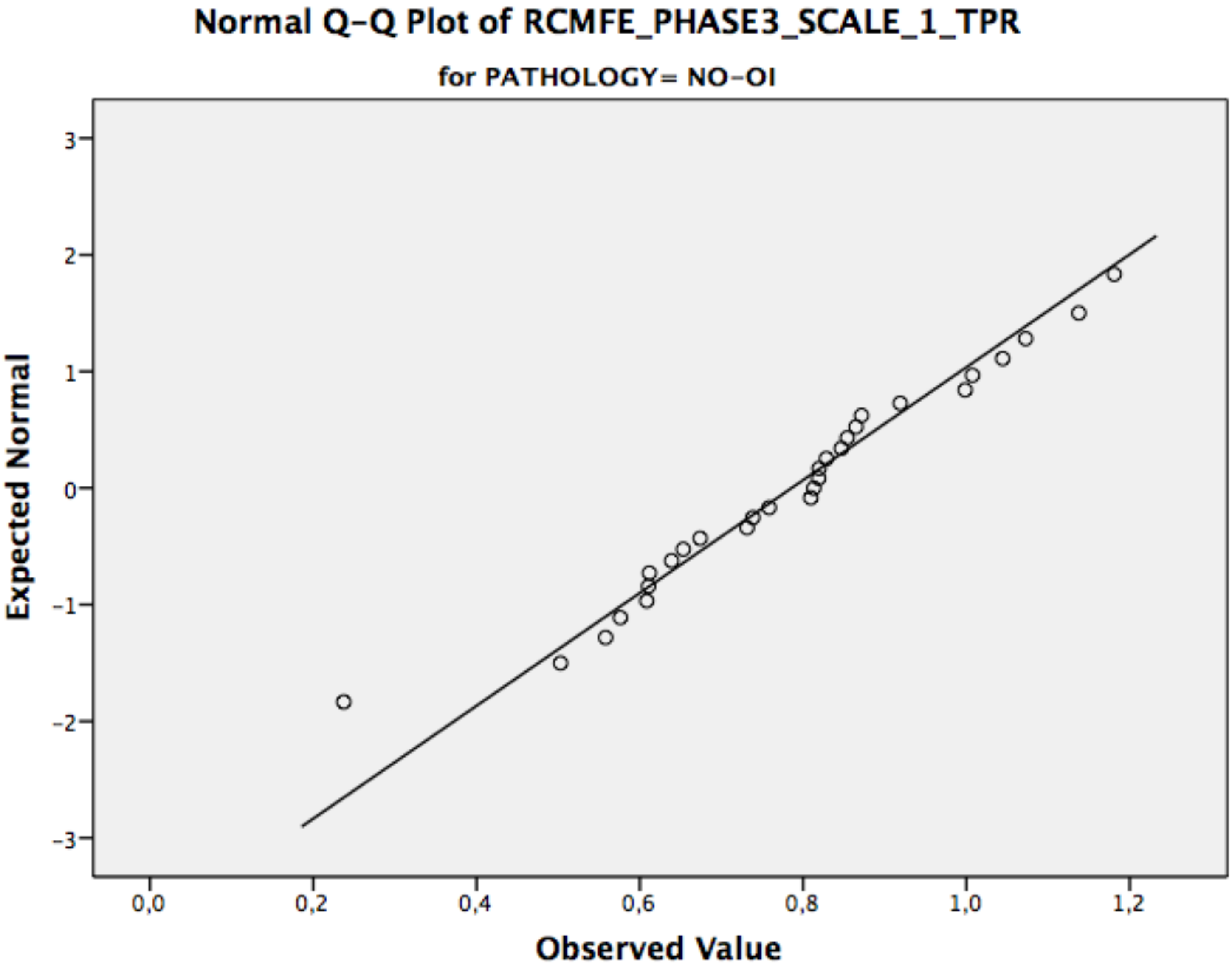

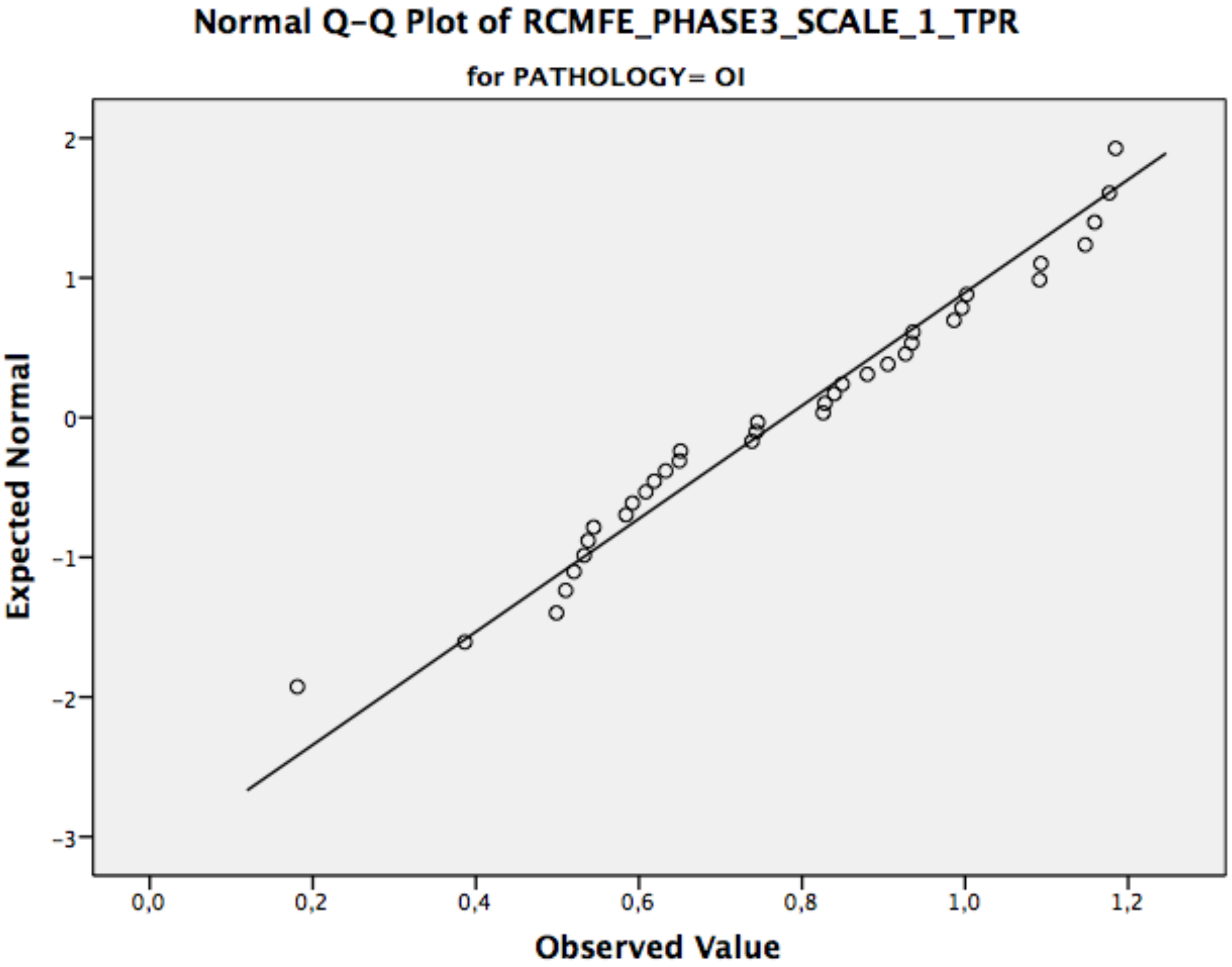

Normal Q-Q Plot of RCMFE_PHASE3_SCALE_1_TPR
for PATHOLOGY= OI
Expected Normal
2
1
0
−1
−2
−3
0,0
0,2
0,4
0,6
0,8
1,0
1,2
Observed Value

RCMFE_PHASE3_SCALE_2_TPR

Normal Q-Q Plots

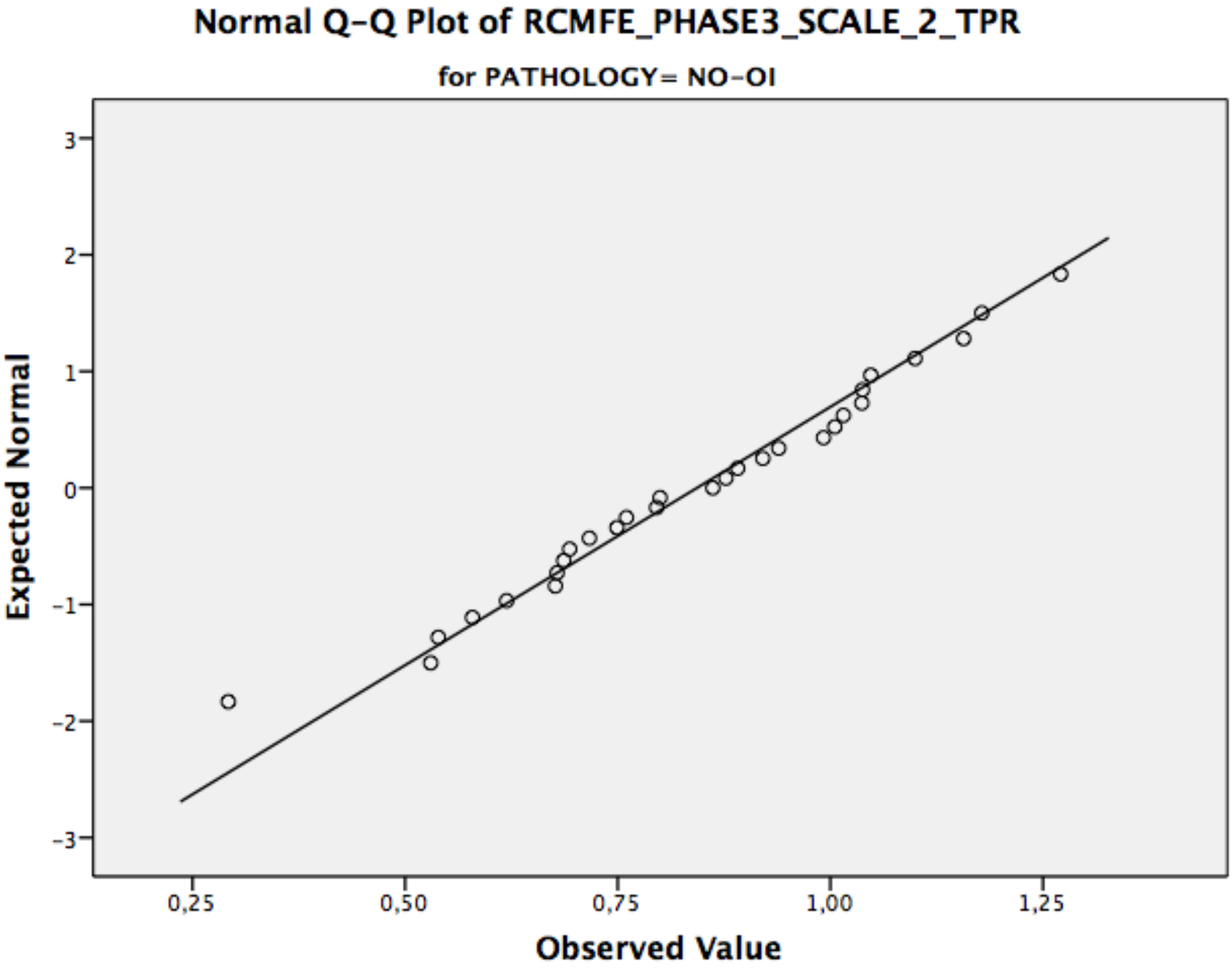

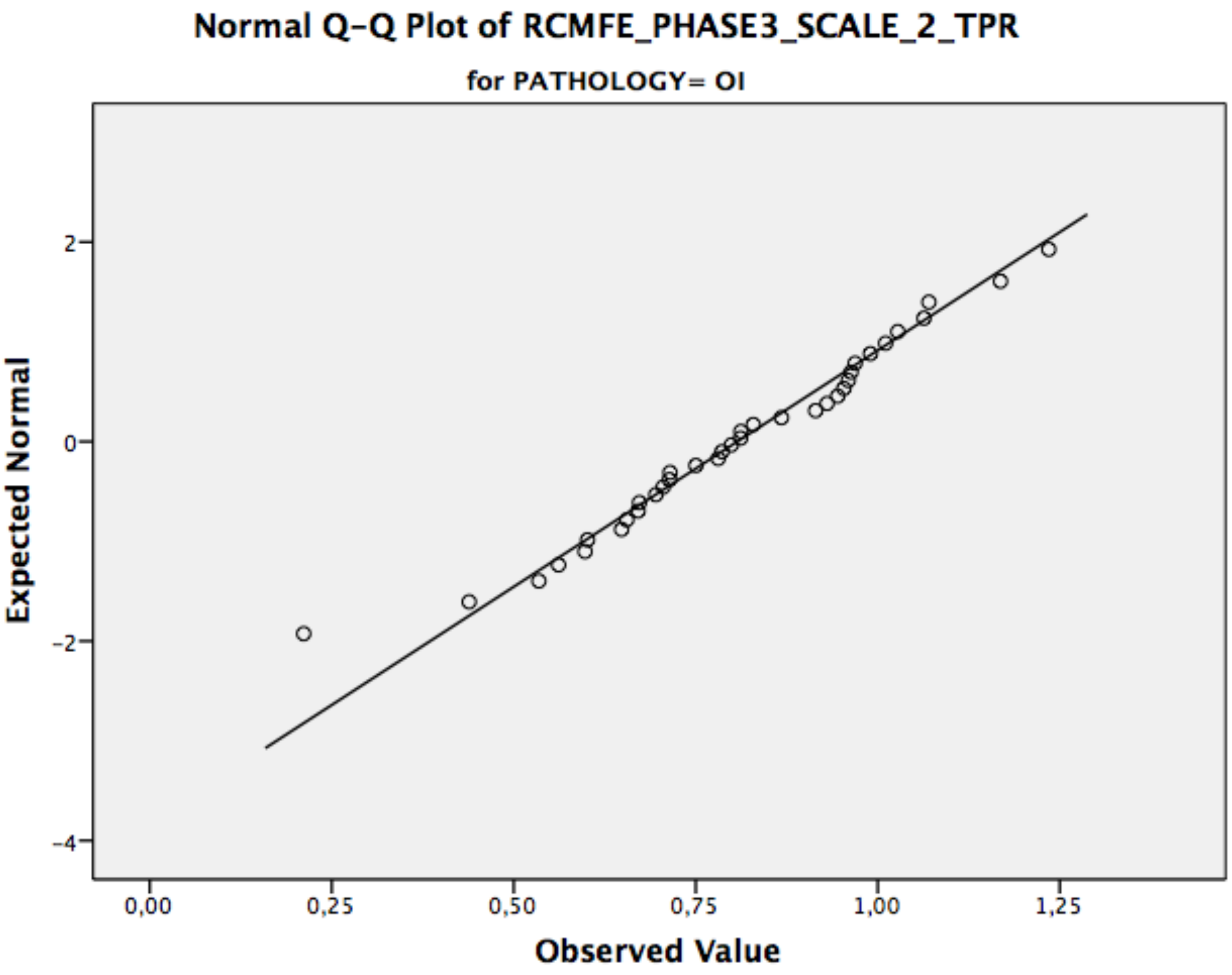
Normal Q-Q Plot of RCMFE_PHASE3_SCALE_2_TPR
for PATHOLOGY= OI
Expected Normal
2
0
-2
-4
0,00
0,25
0,50
0,75
1,00
1,25
Observed Value

RCMFE_PHASE3_SCALE_3_TPR

Normal Q-Q Plots

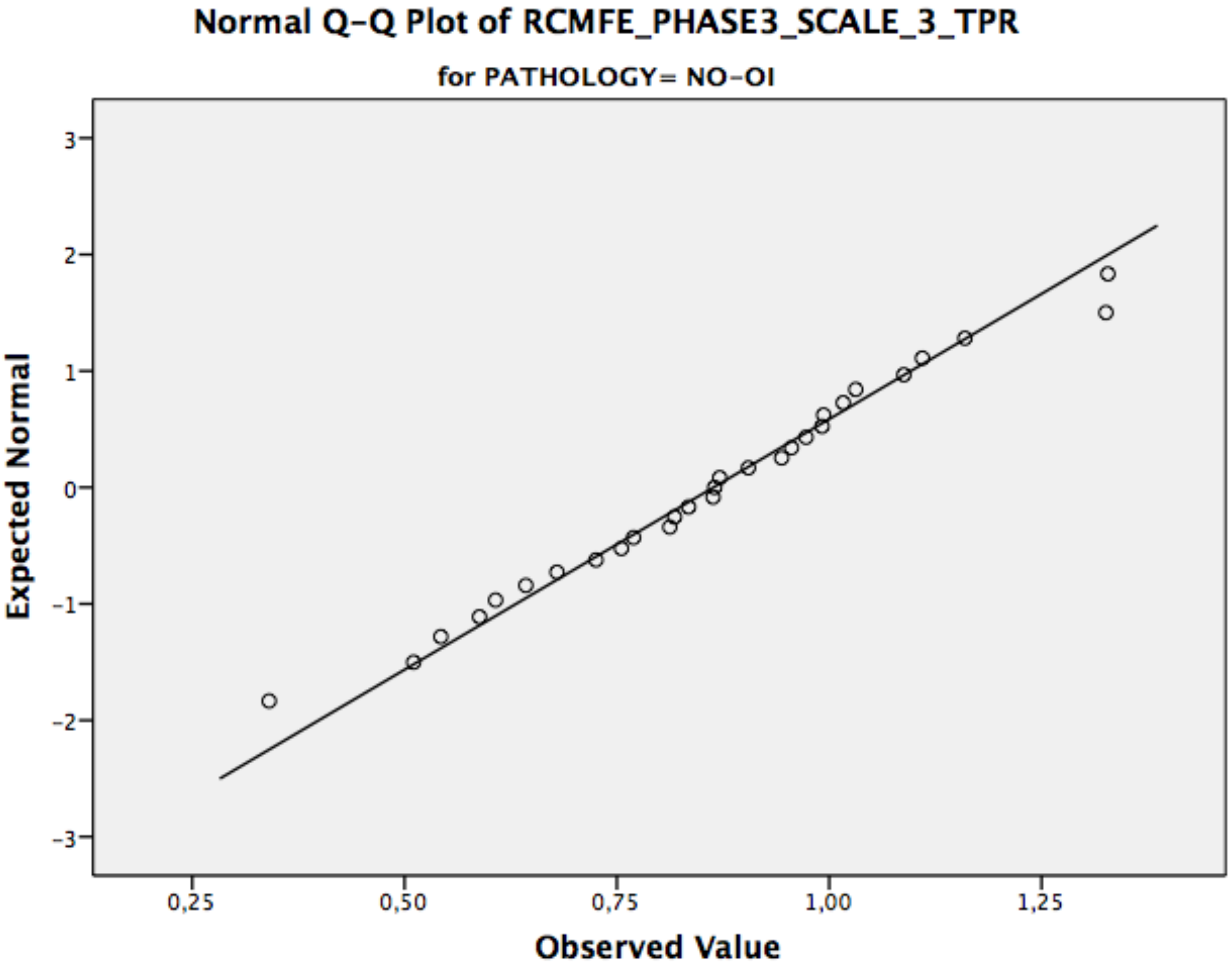

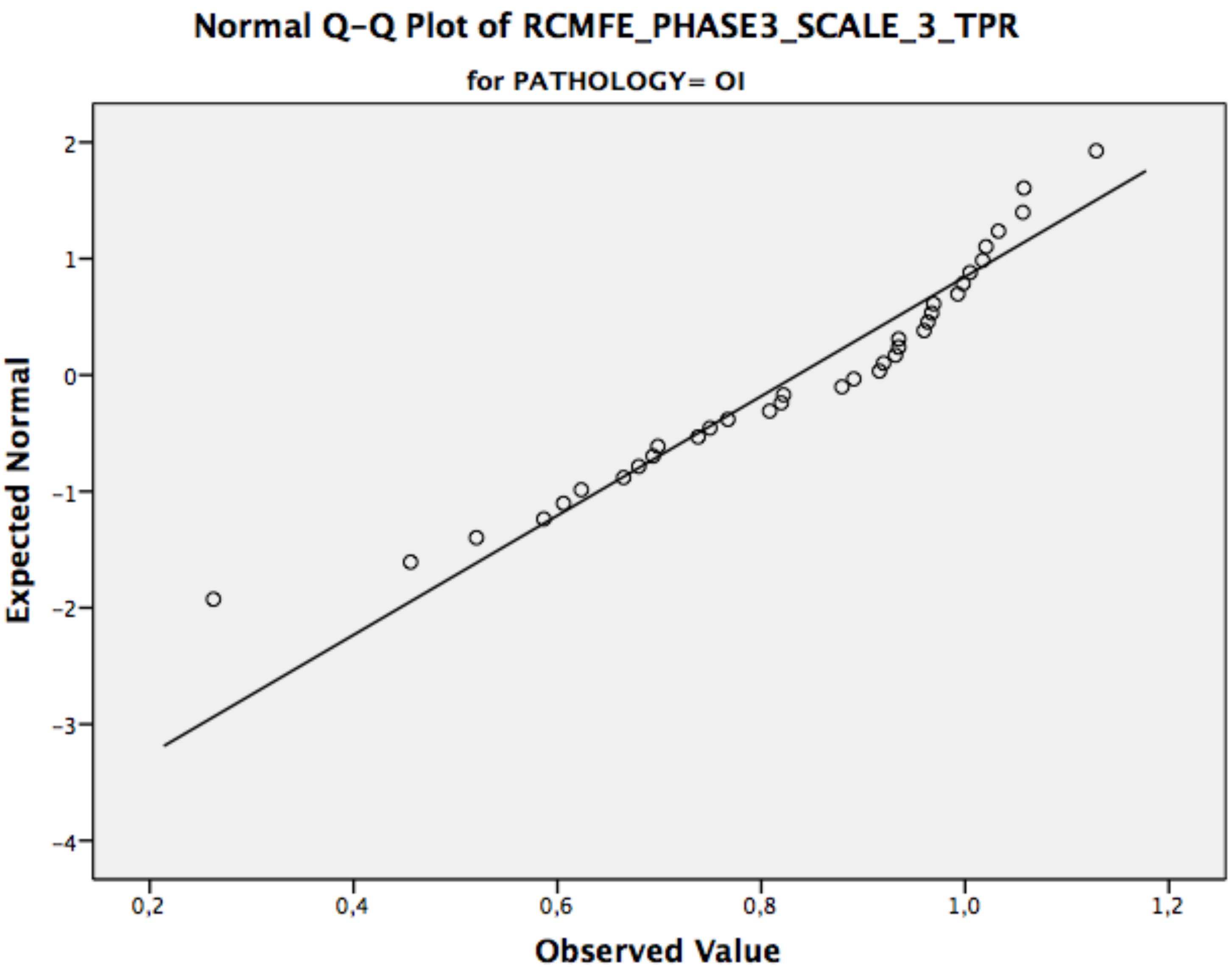
Normal Q-Q Plot of RCMFE_PHASE3_SCALE_3_TPR
for PATHOLOGY= OI
Expected Normal
Observed Value
2
1
0
-1
-2
-3
-4
0,2
0,4
0,6
0,8
1,0
1,2

RCMFE_PHASE3_SCALE_4_TPR

Normal Q-Q Plots

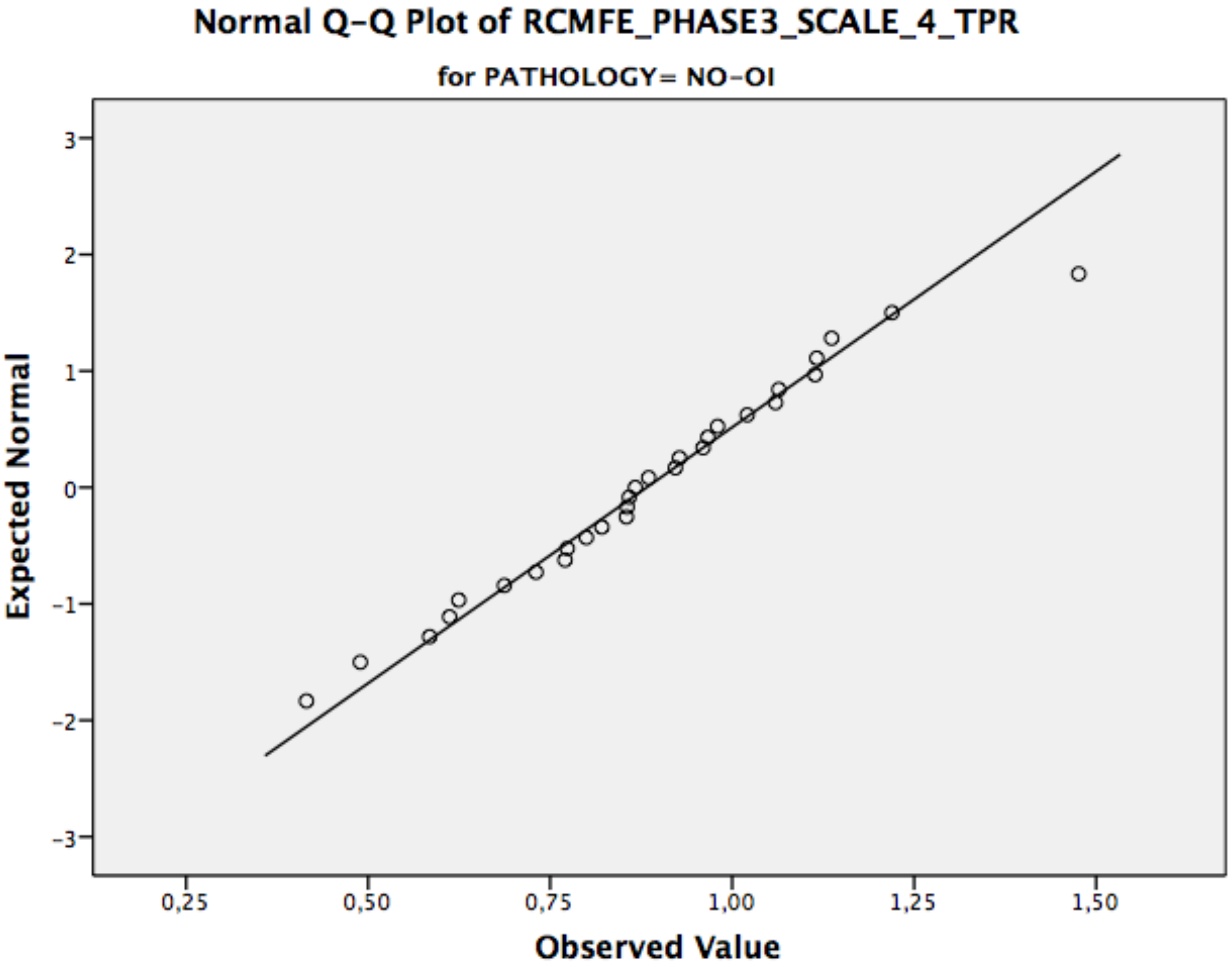

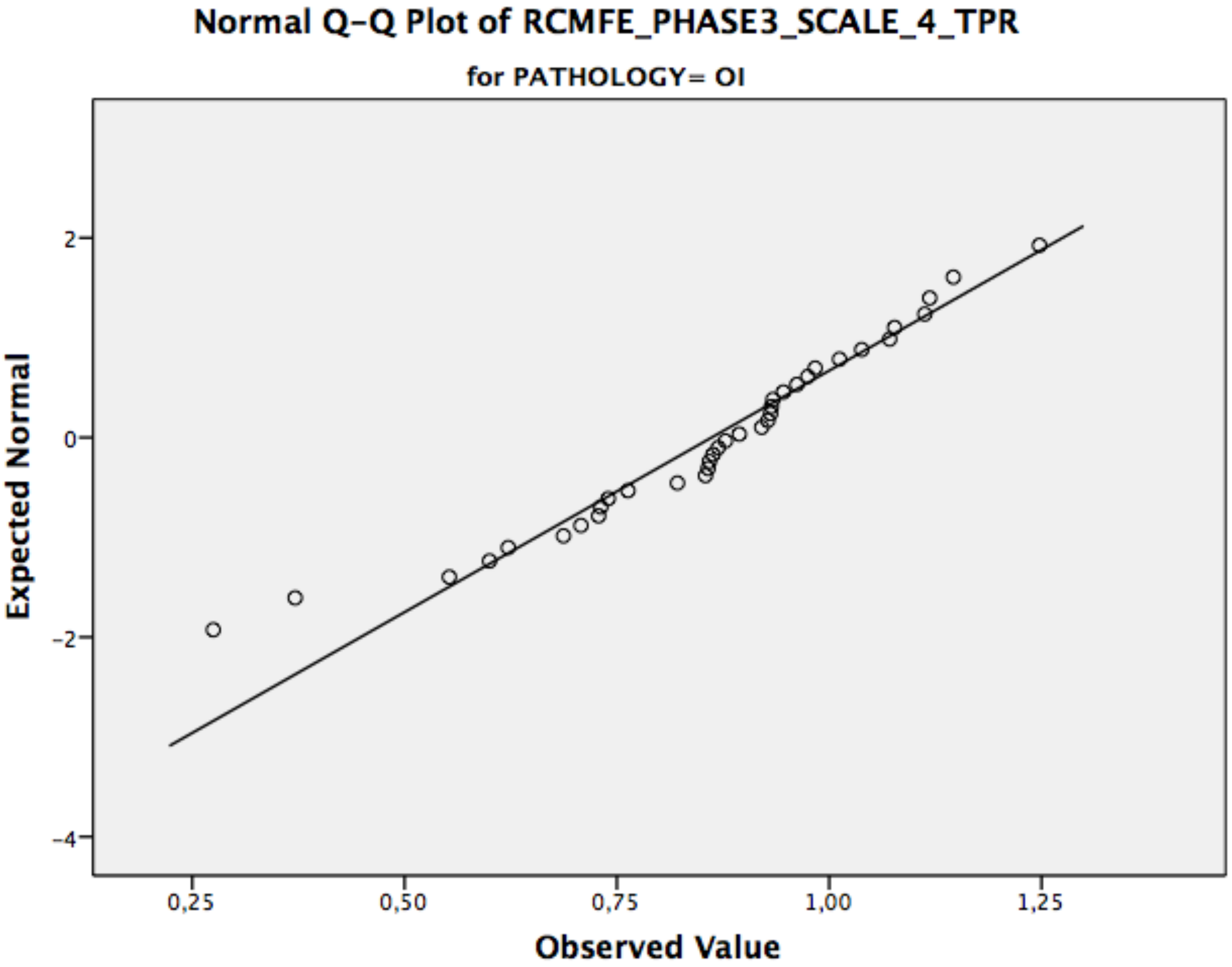
Normal Q-Q Plot of RCMFE_PHASE3_SCALE_4_TPR
for PATHOLOGY= OI
Expected Normal
2
0
-2
-4
0,25
0,50
0,75
1,00
1,25
Observed Value

RCMFE_PHASE3_SCALE_5_TPR

Normal Q-Q Plots

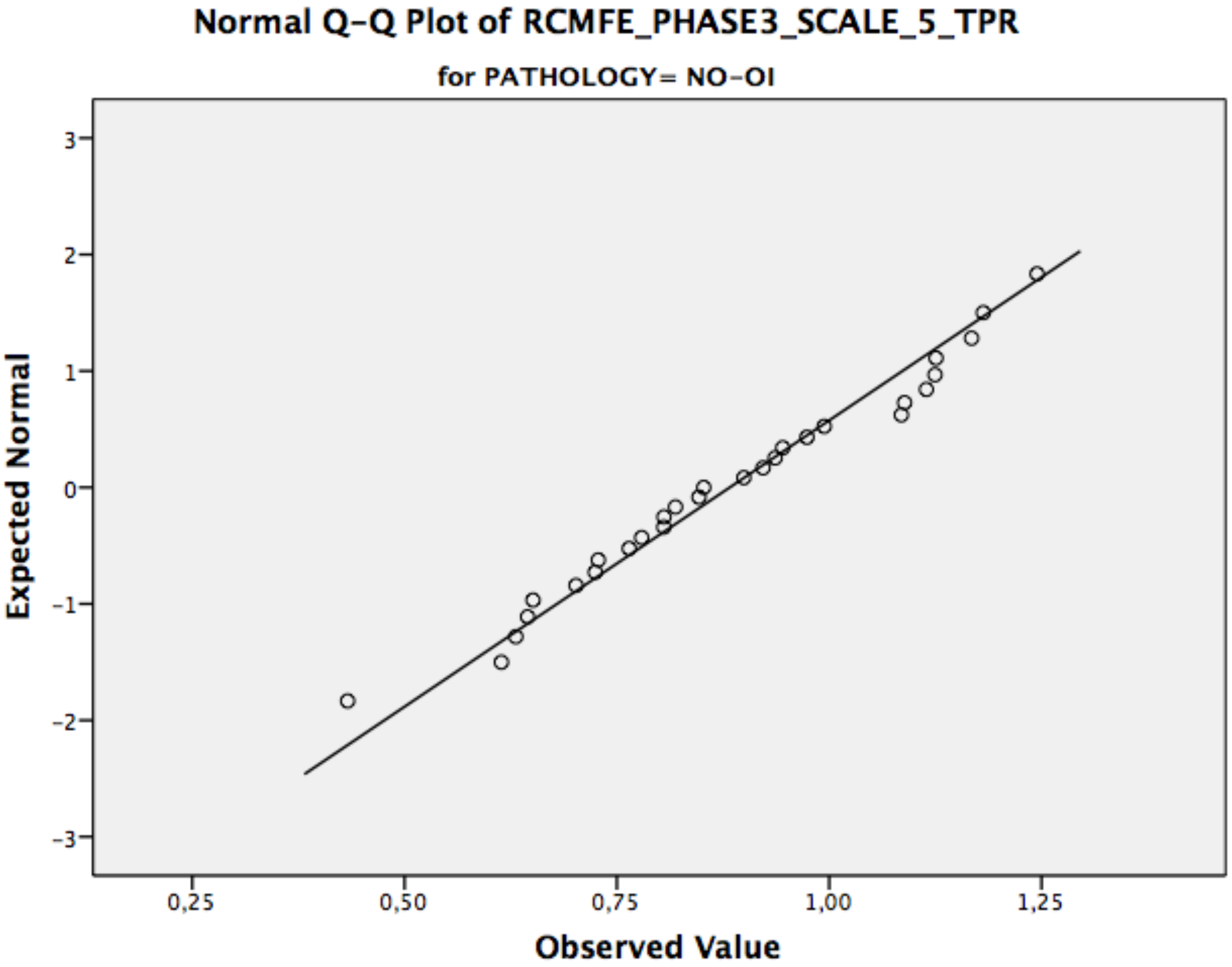

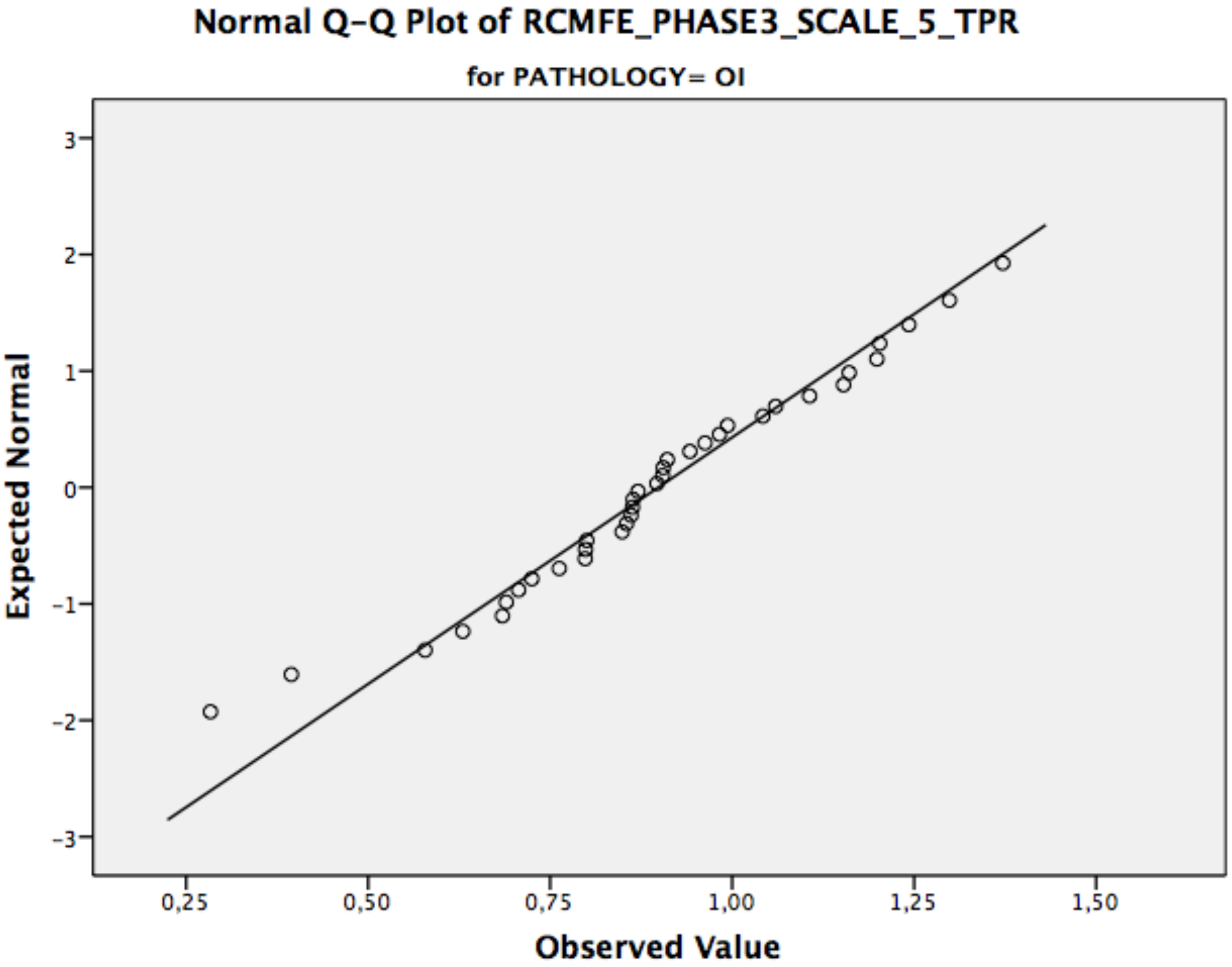

Normal Q-Q Plot of RCMFE_PHASE3_SCALE_5_TPR
for PATHOLOGY= OI
Expected Normal
3
2
1
0
-1
-2
-3
0,25
0,50
0,75
1,00
1,25
1,50
Observed Value

RCMFE_PHASE4_SCALE_1_TPR

Normal Q-Q Plots

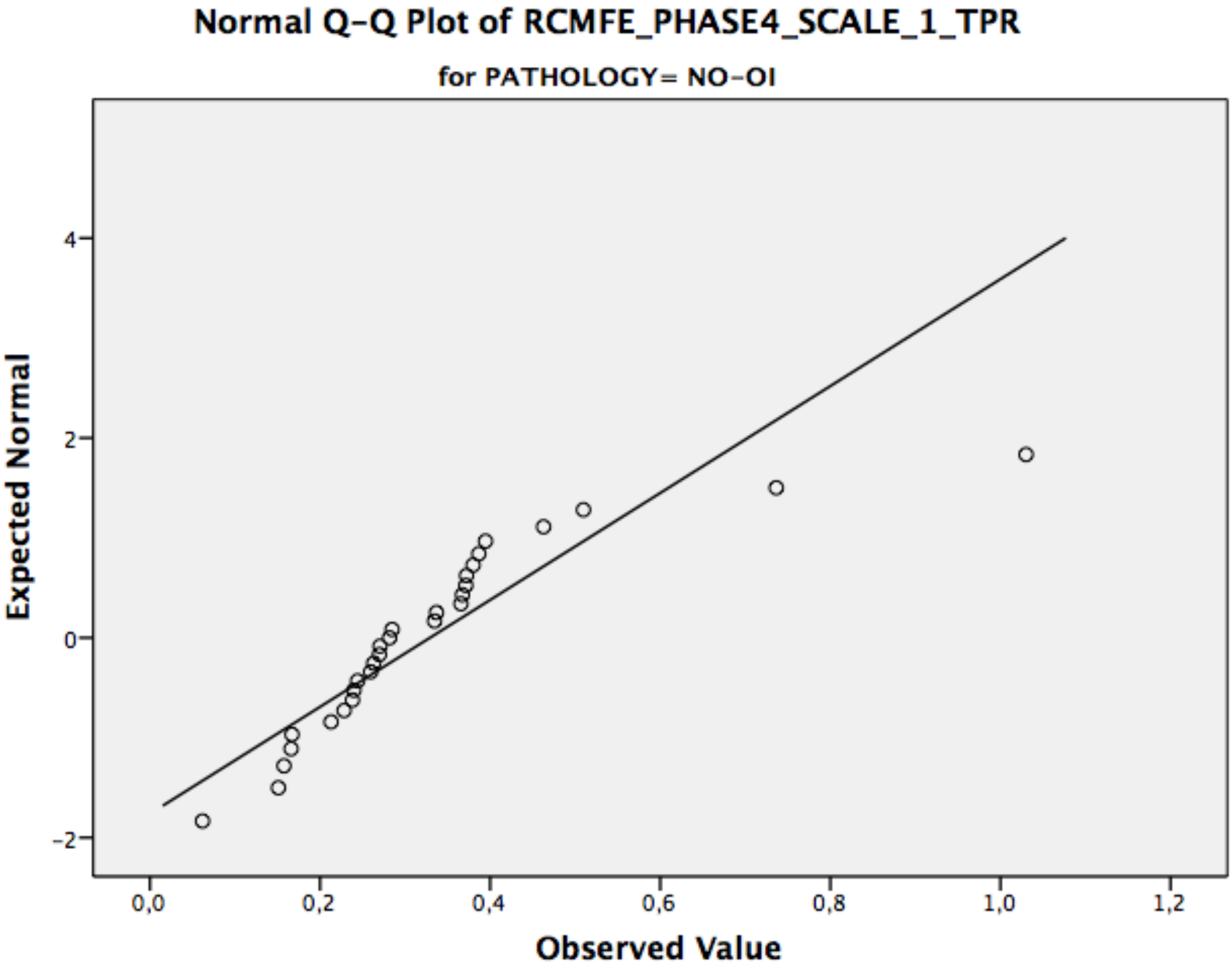

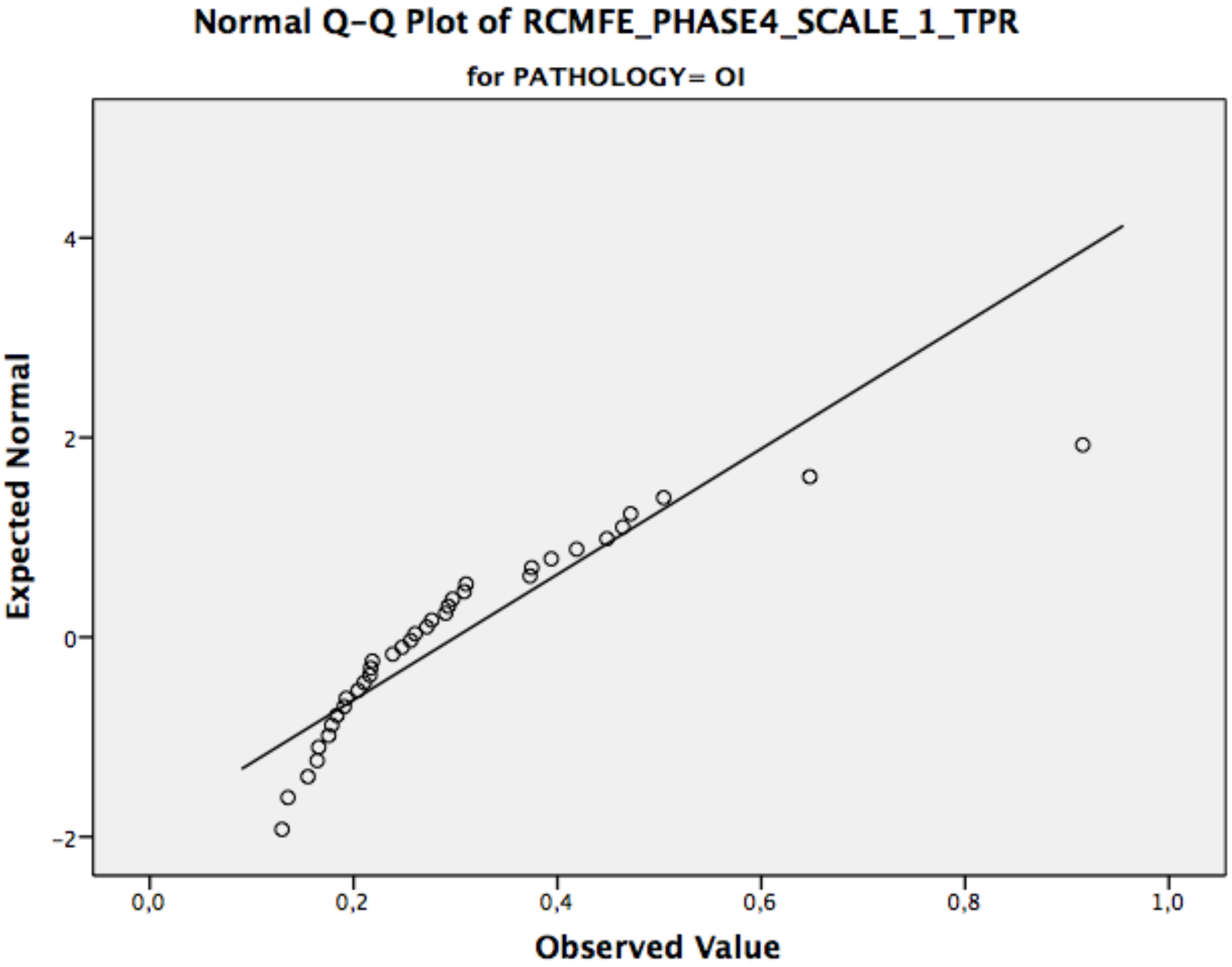

Normal Q-Q Plot of RCMFE_PHASE4_SCALE_1_TPR
for PATHOLOGY= OI
Expected Normal
4
2
0
-2
0,0
0,2
0,4
0,6
0,8
1,0
Observed Value

RCMFE_PHASE4_SCALE_2_TPR

Normal Q-Q Plots

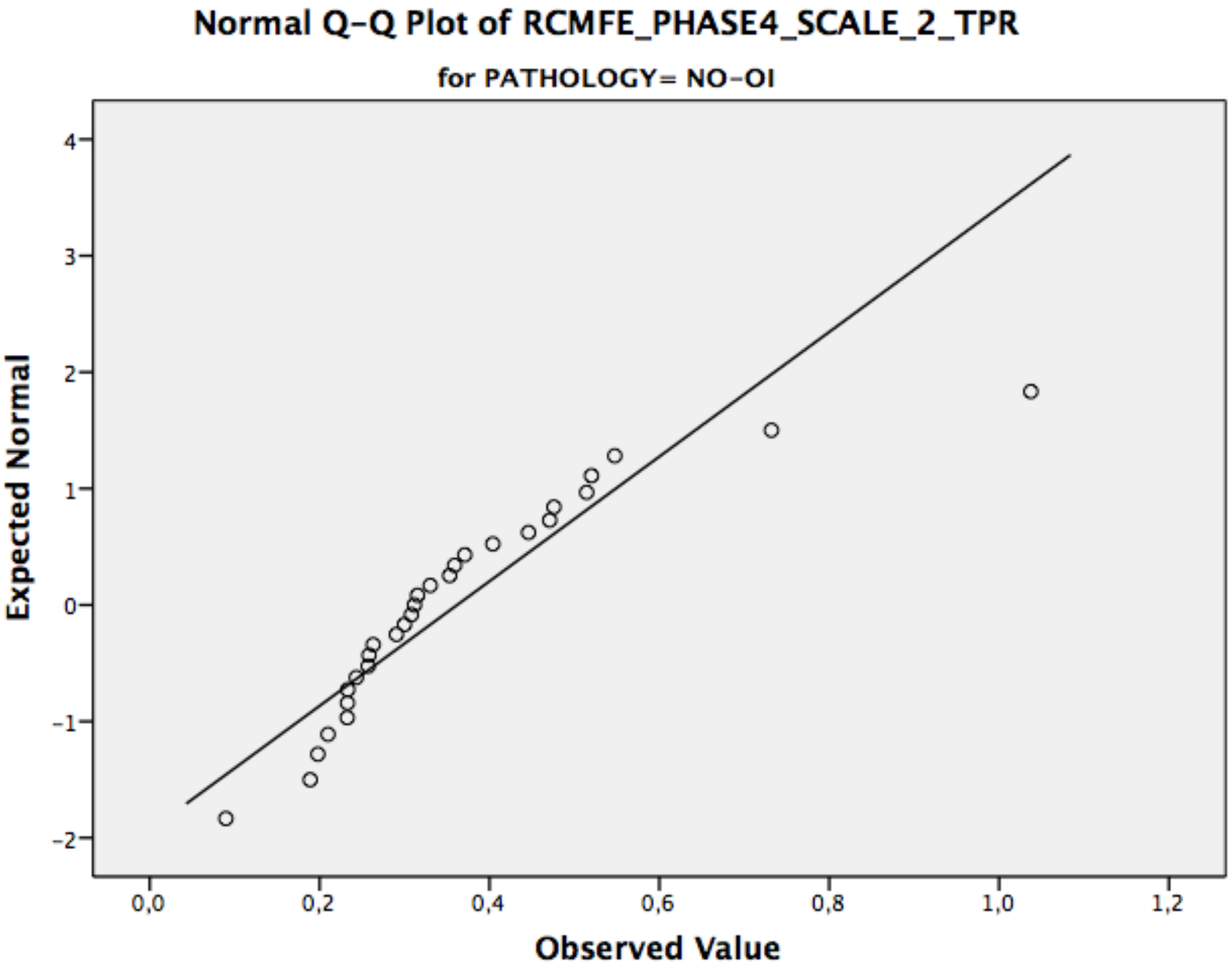

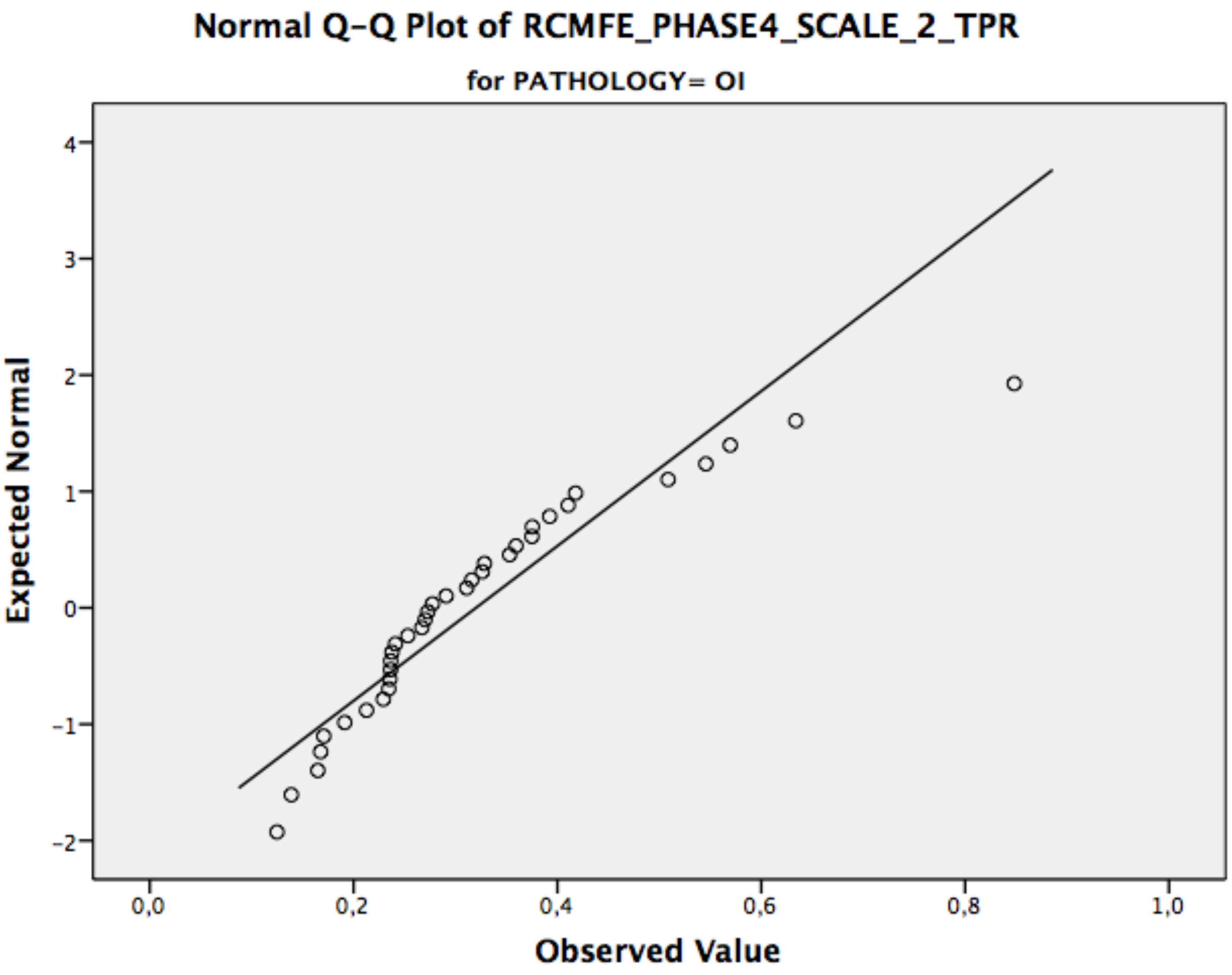

Normal Q-Q Plot of RCMFE_PHASE4_SCALE_2_TPR
for PATHOLOGY= OI
Expected Normal
Observed Value
4
3
2
1
0
-1
-2
0,0
0,2
0,4
0,6
0,8
1,0

RCMFE_PHASE4_SCALE_3_TPR

Normal Q-Q Plots

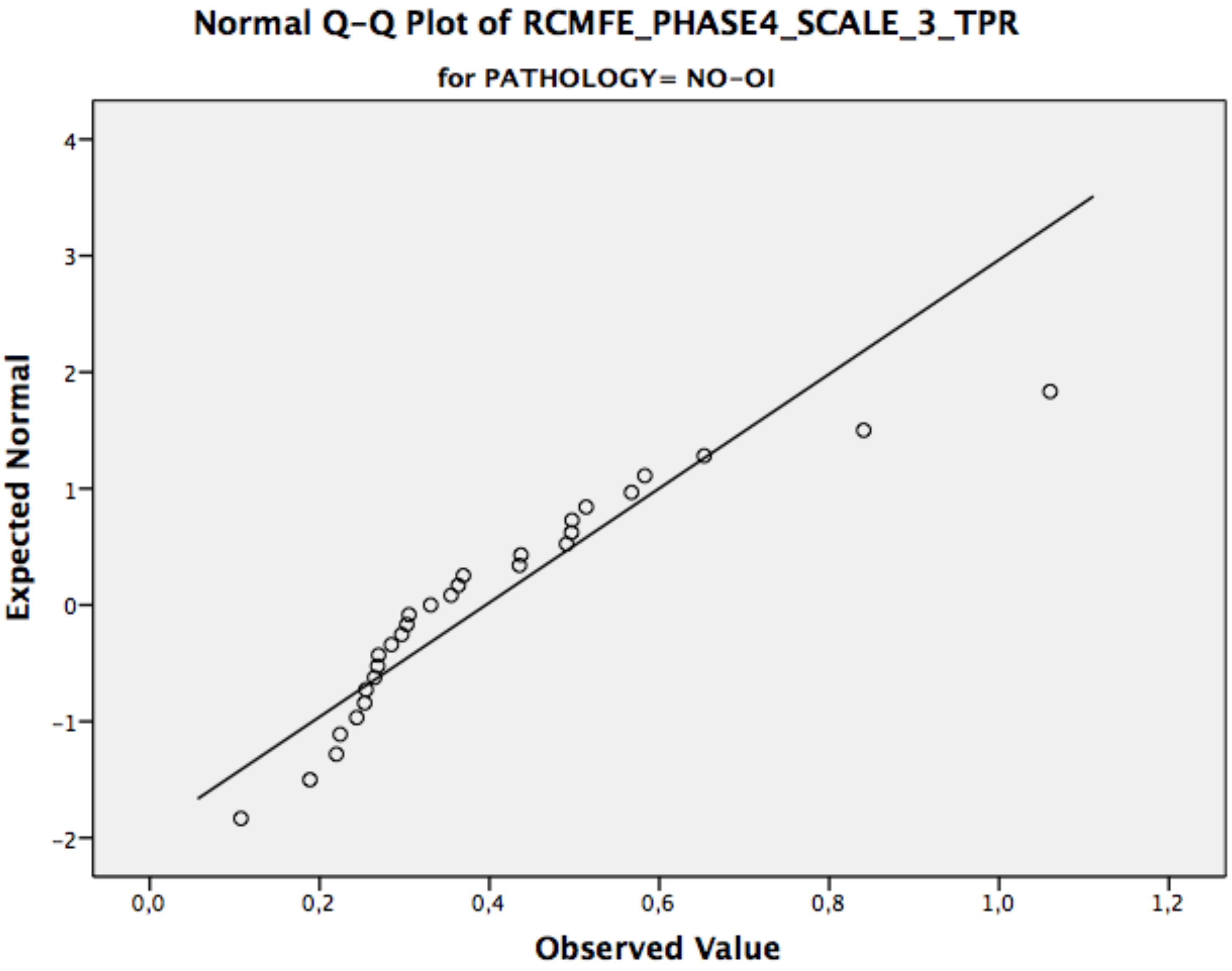

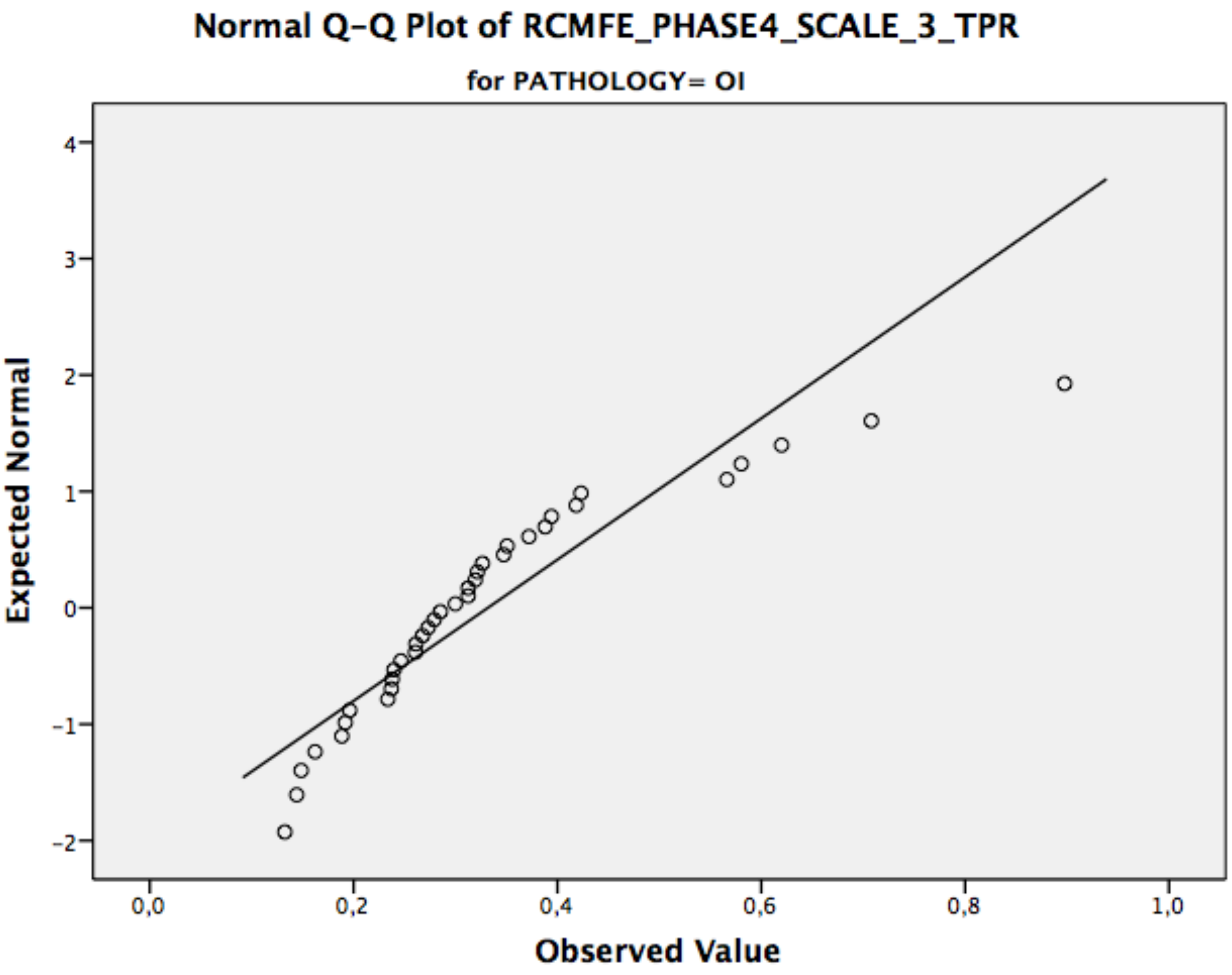
Normal Q-Q Plot of RCMFE_PHASE4_SCALE_3_TPR
for PATHOLOGY= OI
Expected Normal
4
3
2
1
0
-1
-2
0,0
0,2
0,4
0,6
0,8
1,0
Observed Value

RCMFE_PHASE4_SCALE_4_TPR

Normal Q-Q Plots

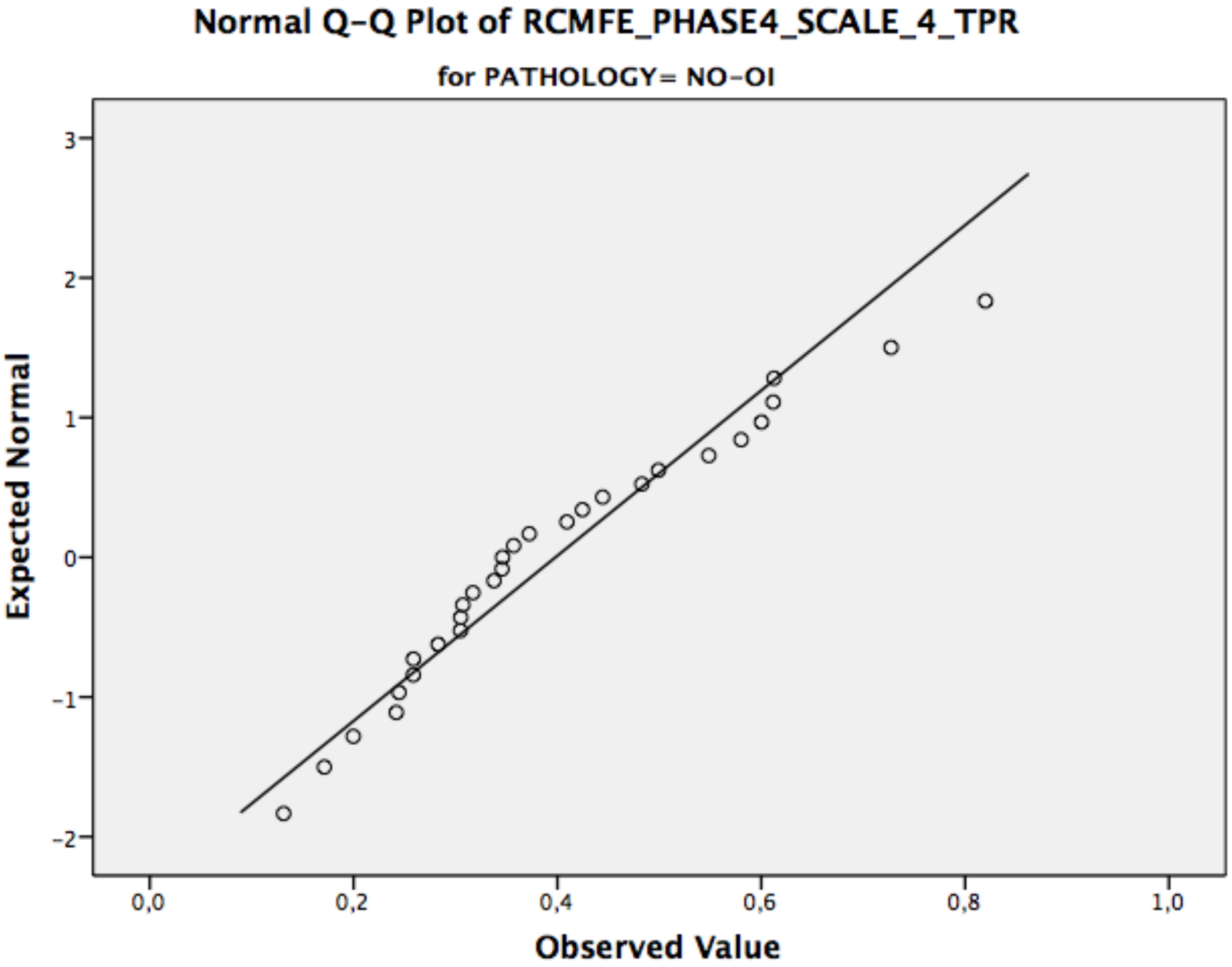

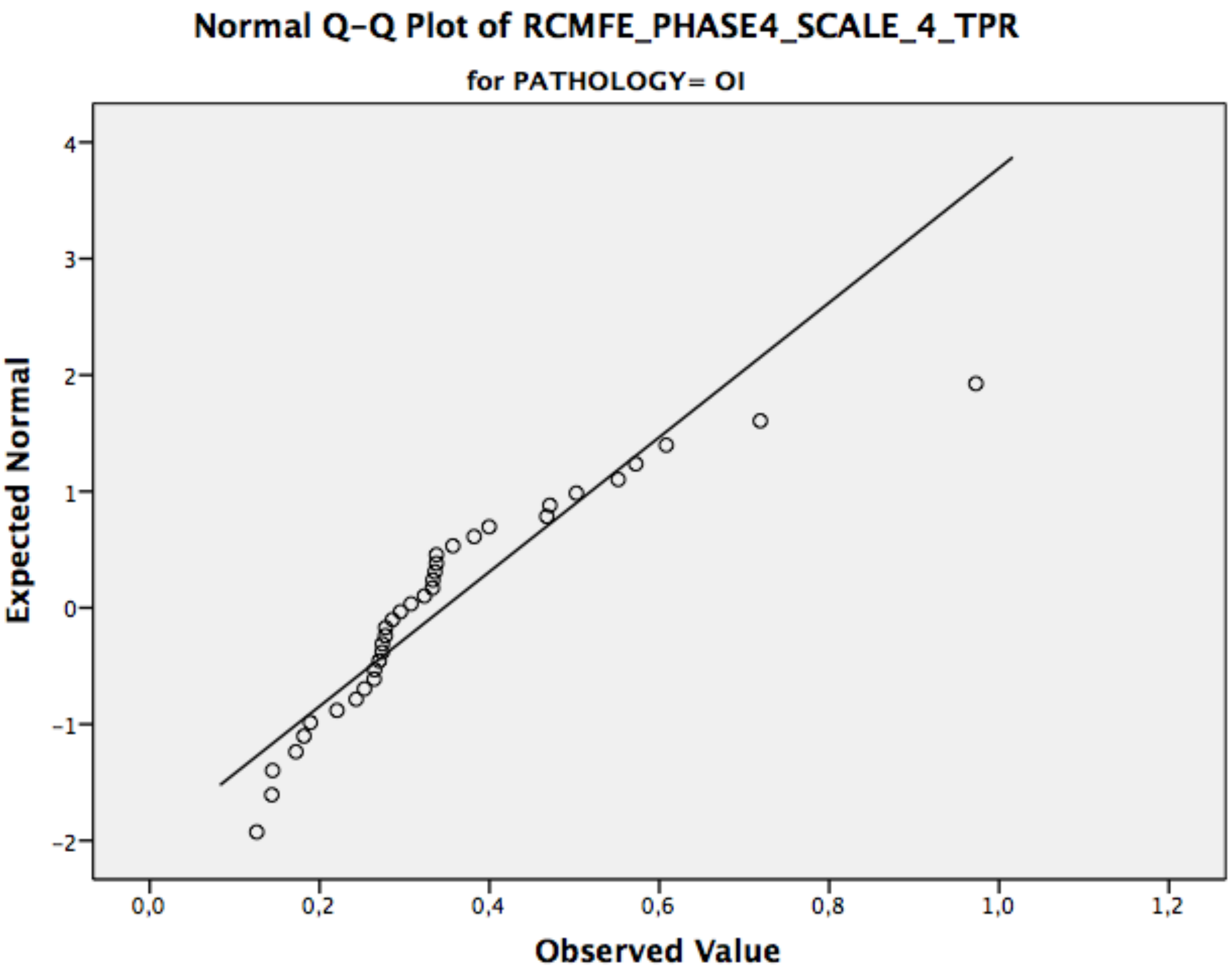
Normal Q-Q Plot of RCMFE_PHASE4_SCALE_4_TPR
for PATHOLOGY= OI
Expected Normal
Observed Value
4
3
2
1
0
-1
-2
0,0
0,2
0,4
0,6
0,8
1,0
1,2

RCMFE_PHASE4_SCALE_5_TPR

Normal Q-Q Plots

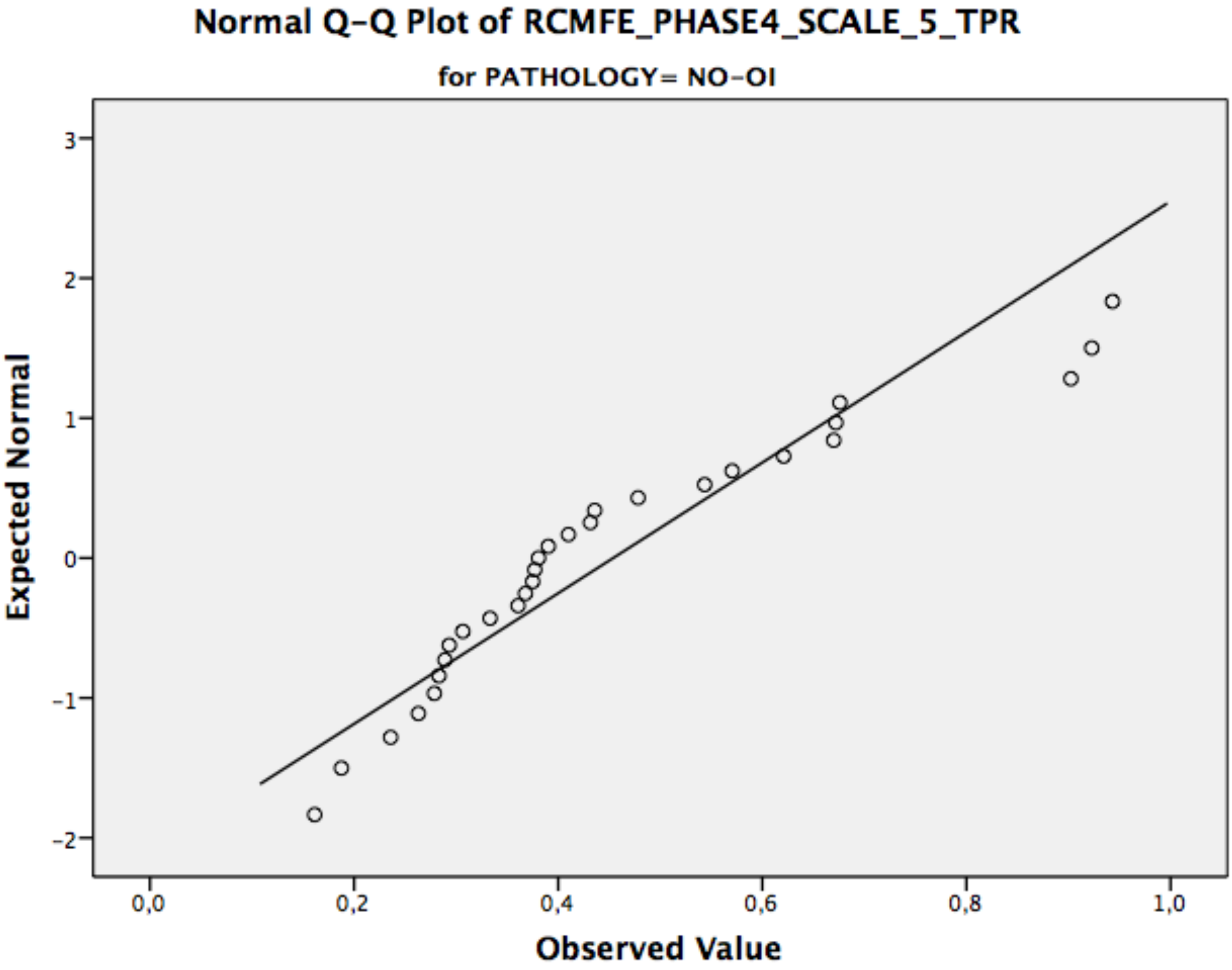

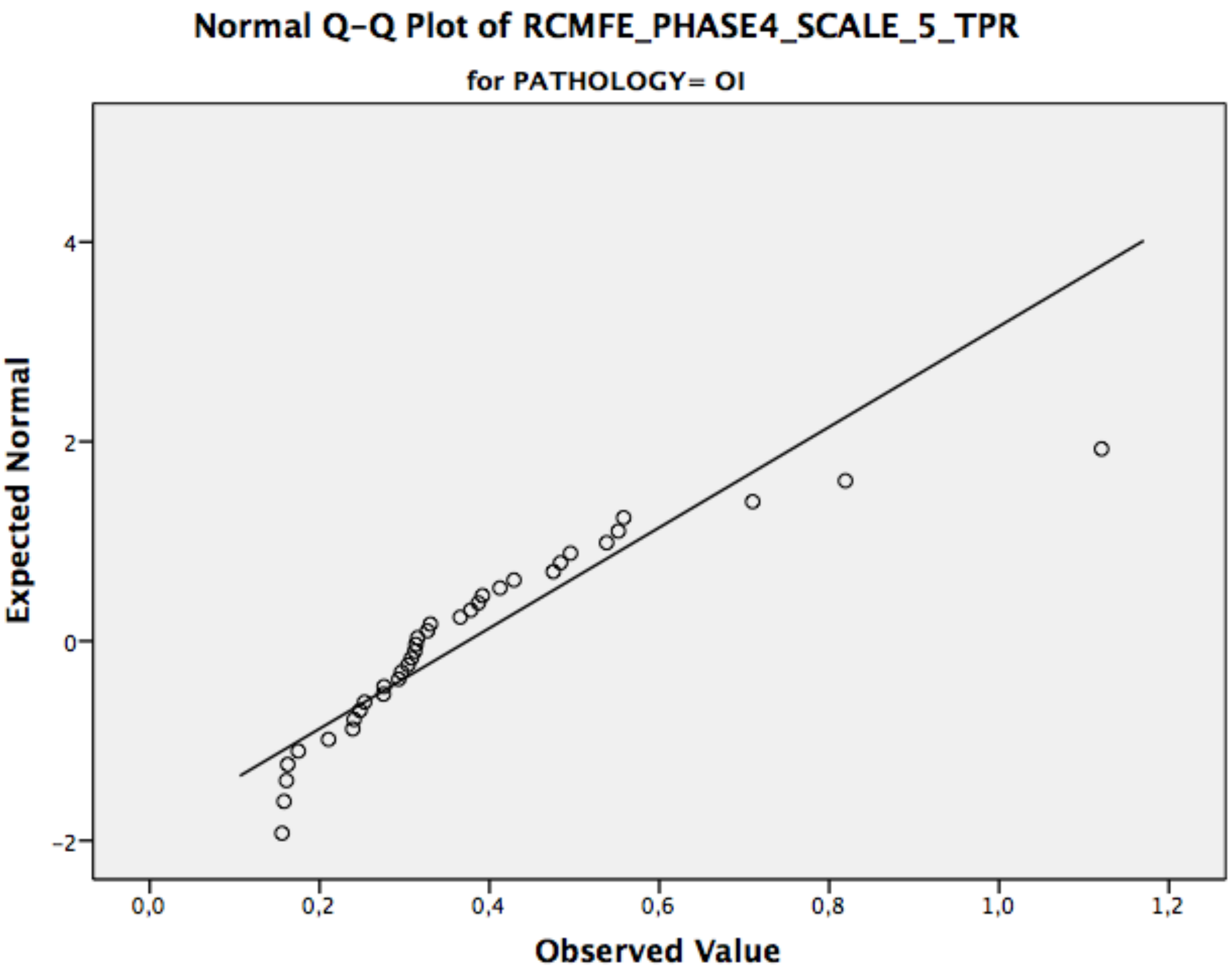

Normal Q-Q Plot of RCMFE_PHASE4_SCALE_5_TPR
for PATHOLOGY= OI
Expected Normal
Observed Value
4
2
0
-2
0,0
0,2
0,4
0,6
0,8
1,0
1,2

RCMFE_PHASE5_SCALE_1_TPR

Normal Q-Q Plots

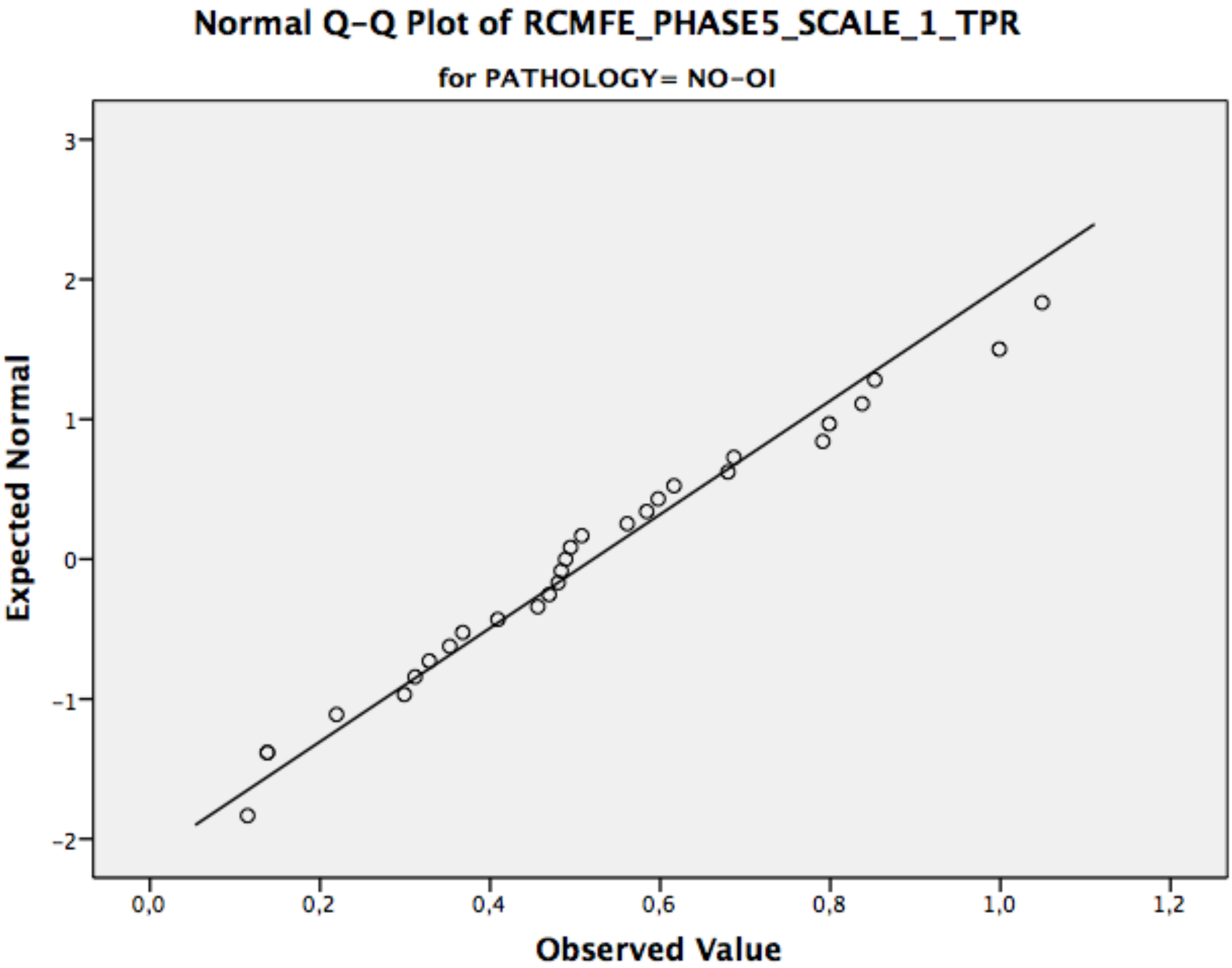

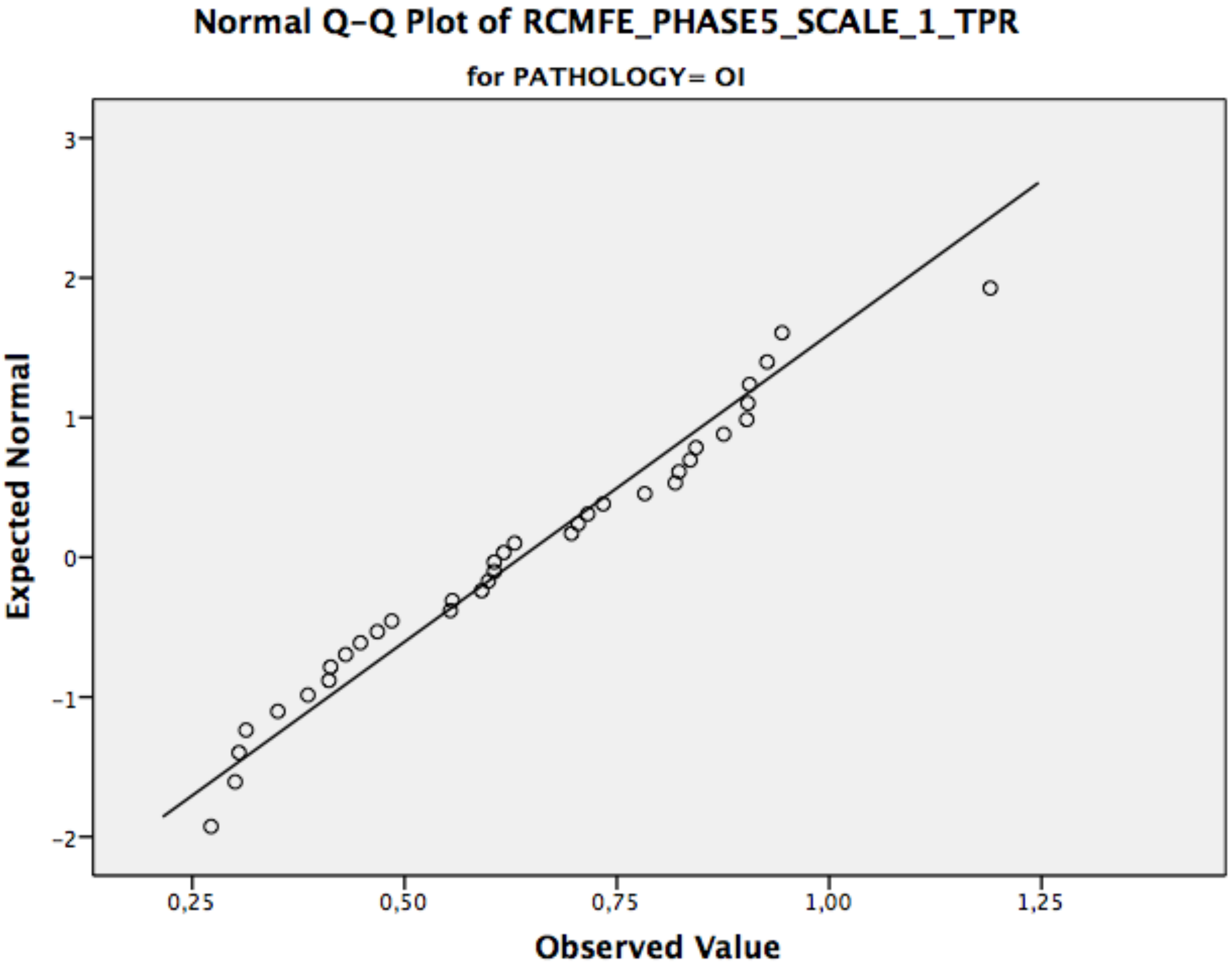
Normal Q-Q Plot of RCMFE_PHASE5_SCALE_1_TPR
for PATHOLOGY= OI
Expected Normal
Observed Value
3
2
1
0
−1
−2
0,25
0,50
0,75
1,00
1,25

RCMFE_PHASE5_SCALE_2_TPR

Normal Q-Q Plots

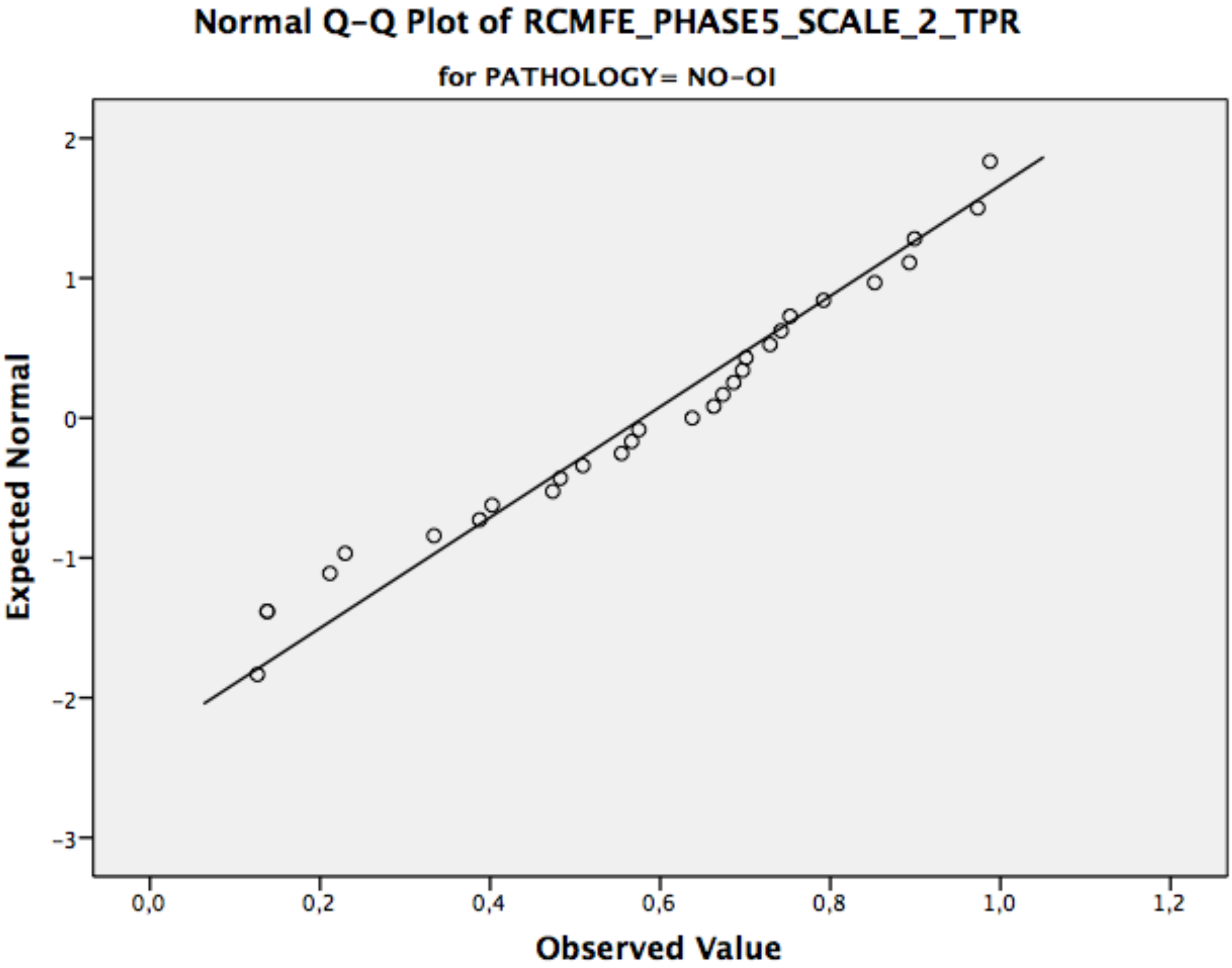

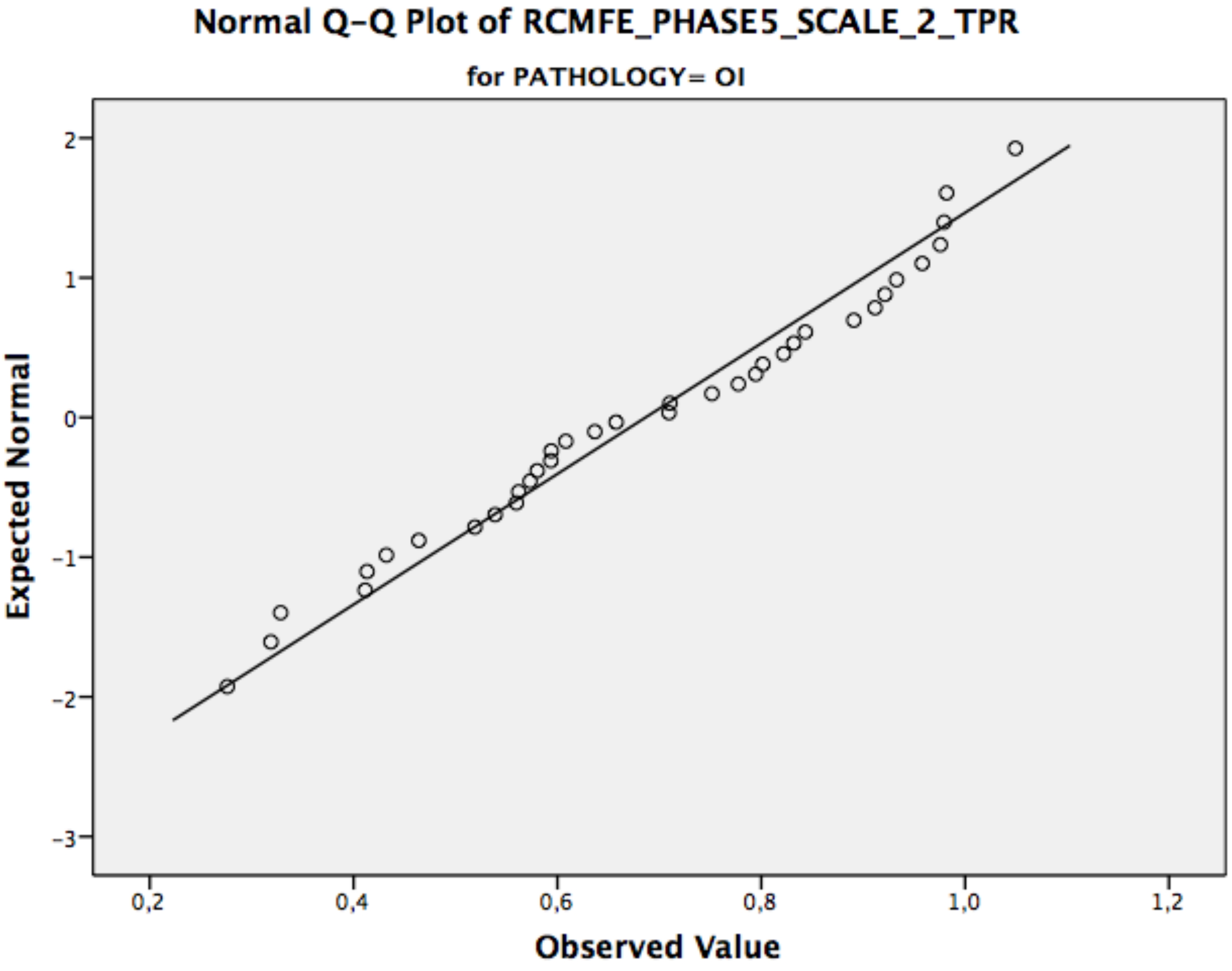

Normal Q-Q Plot of RCMFE_PHASE5_SCALE_2_TPR
for PATHOLOGY= OI
Expected Normal
Observed Value
2
1
0
−1
−2
−3
0,2
0,4
0,6
0,8
1,0
1,2

RCMFE_PHASE5_SCALE_3_TPR

Normal Q-Q Plots

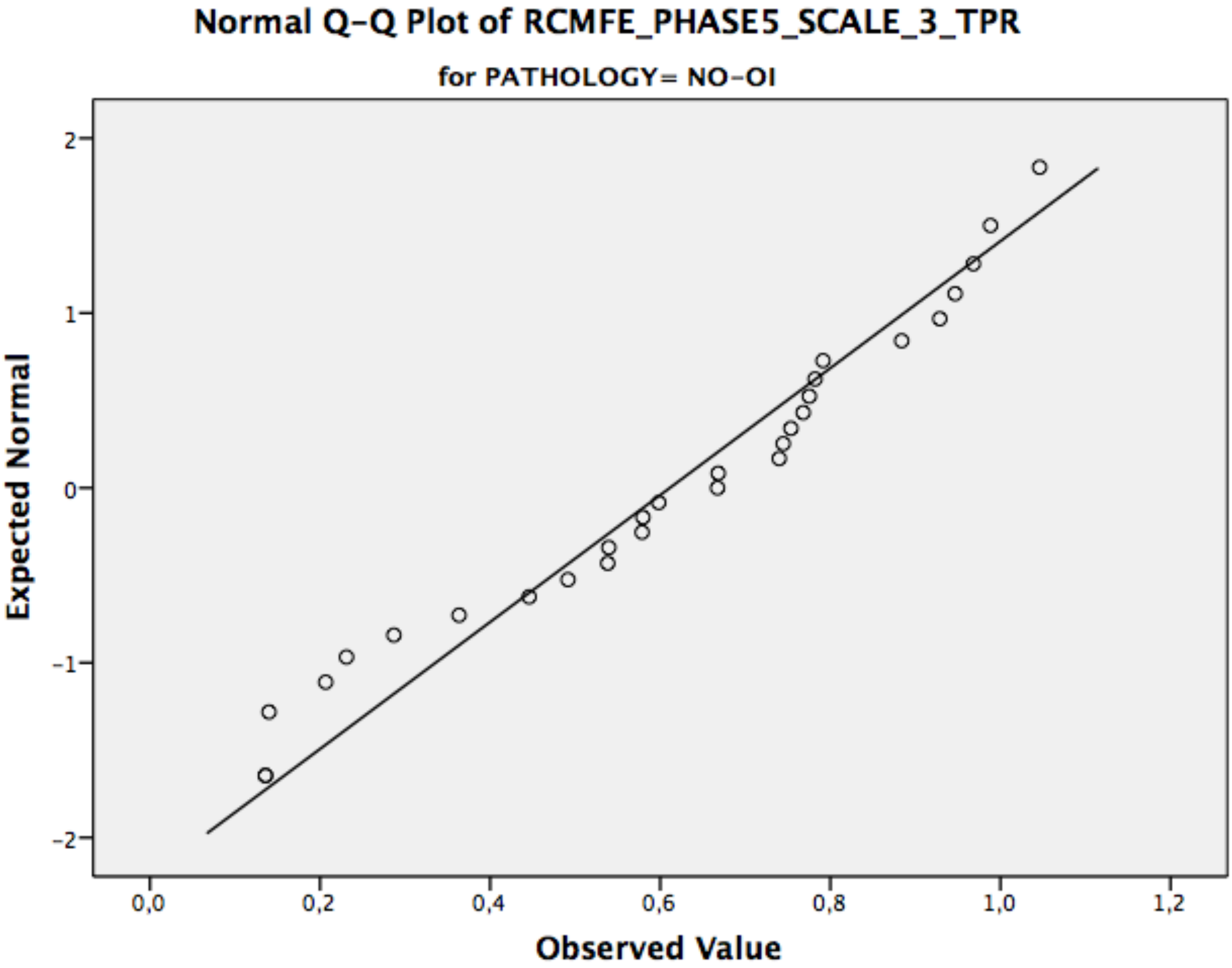

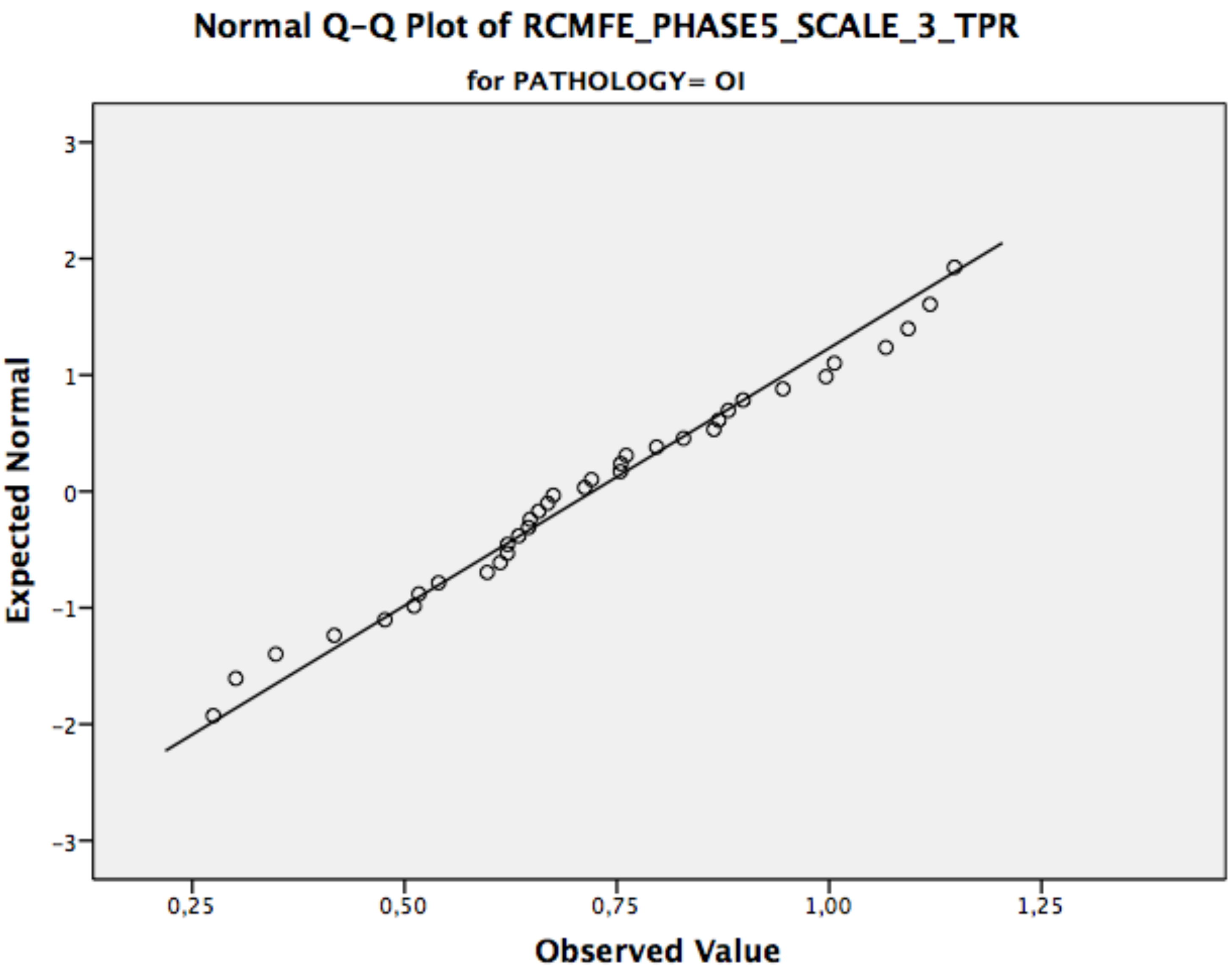
Normal Q-Q Plot of RCMFE_PHASE5_SCALE_3_TPR
for PATHOLOGY= OI
Expected Normal
3
2
1
0
-1
-2
-3
0,25
0,50
0,75
1,00
1,25
Observed Value

RCMFE_PHASE5_SCALE_4_TPR

Normal Q-Q Plots

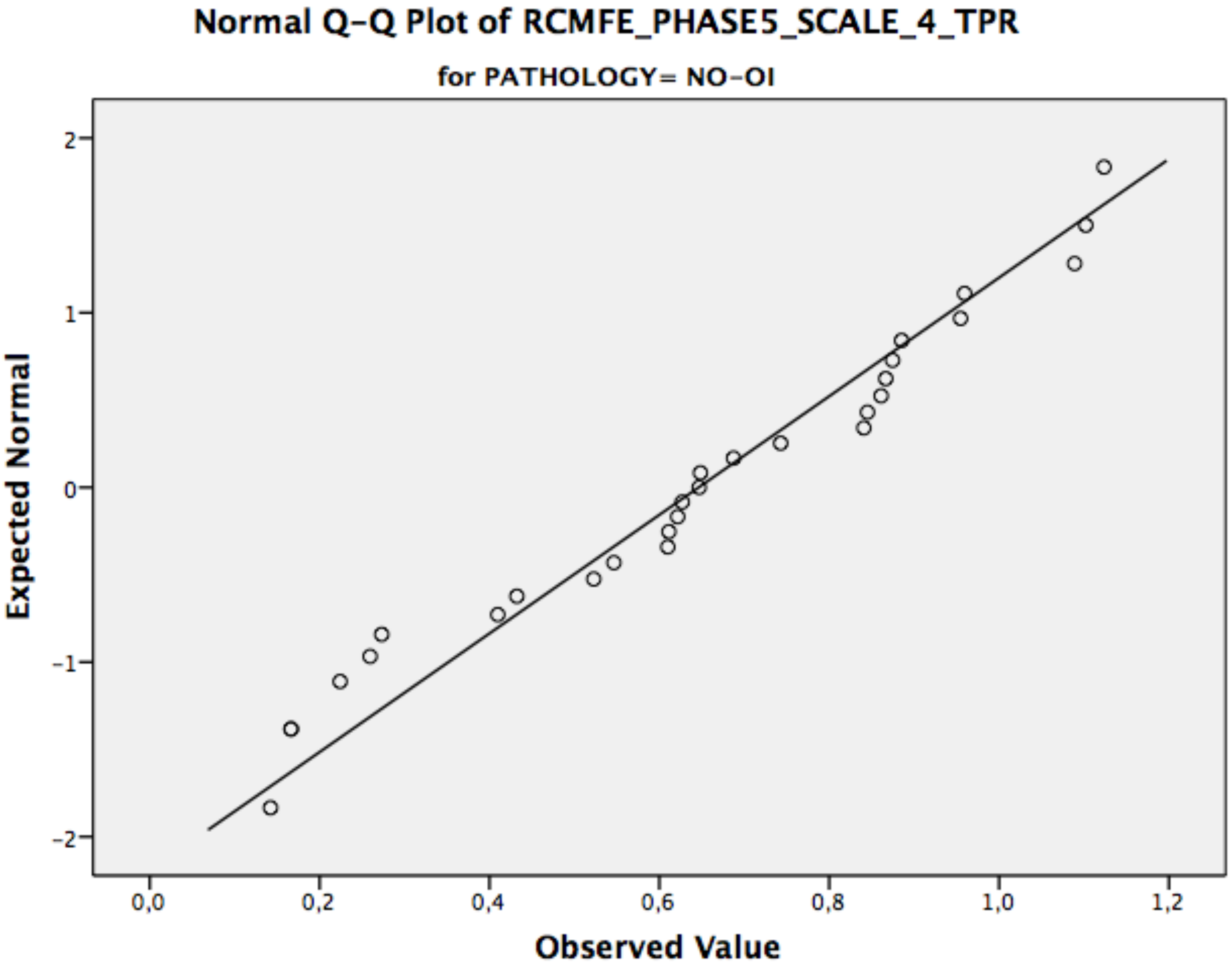

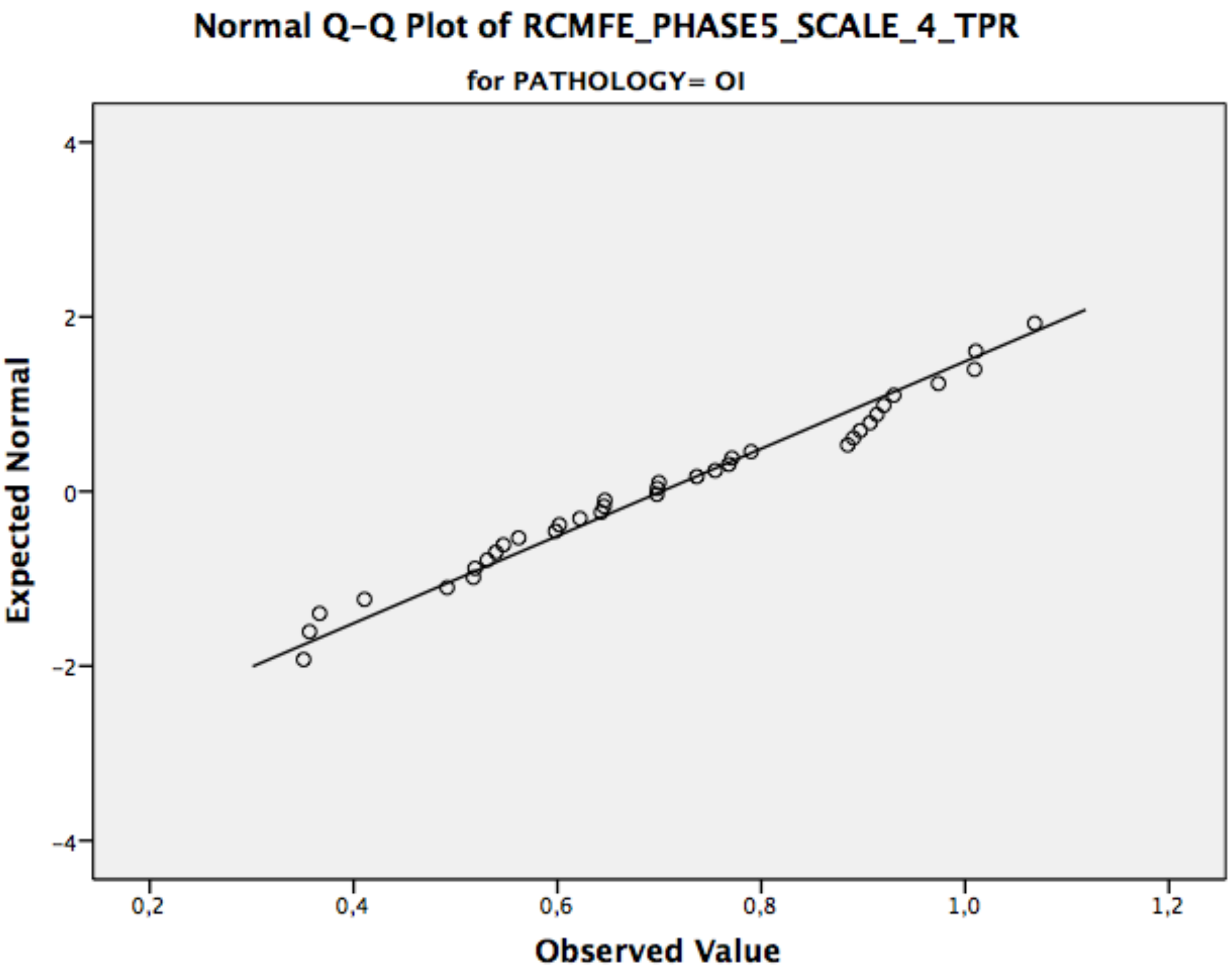
Normal Q-Q Plot of RCMFE_PHASE5_SCALE_4_TPR
for PATHOLOGY= OI
Expected Normal
4
2
0
-2
-4
0,2
0,4
0,6
0,8
1,0
1,2
Observed Value

RCMFE_PHASE5_SCALE_5_TPR

Normal Q-Q Plots

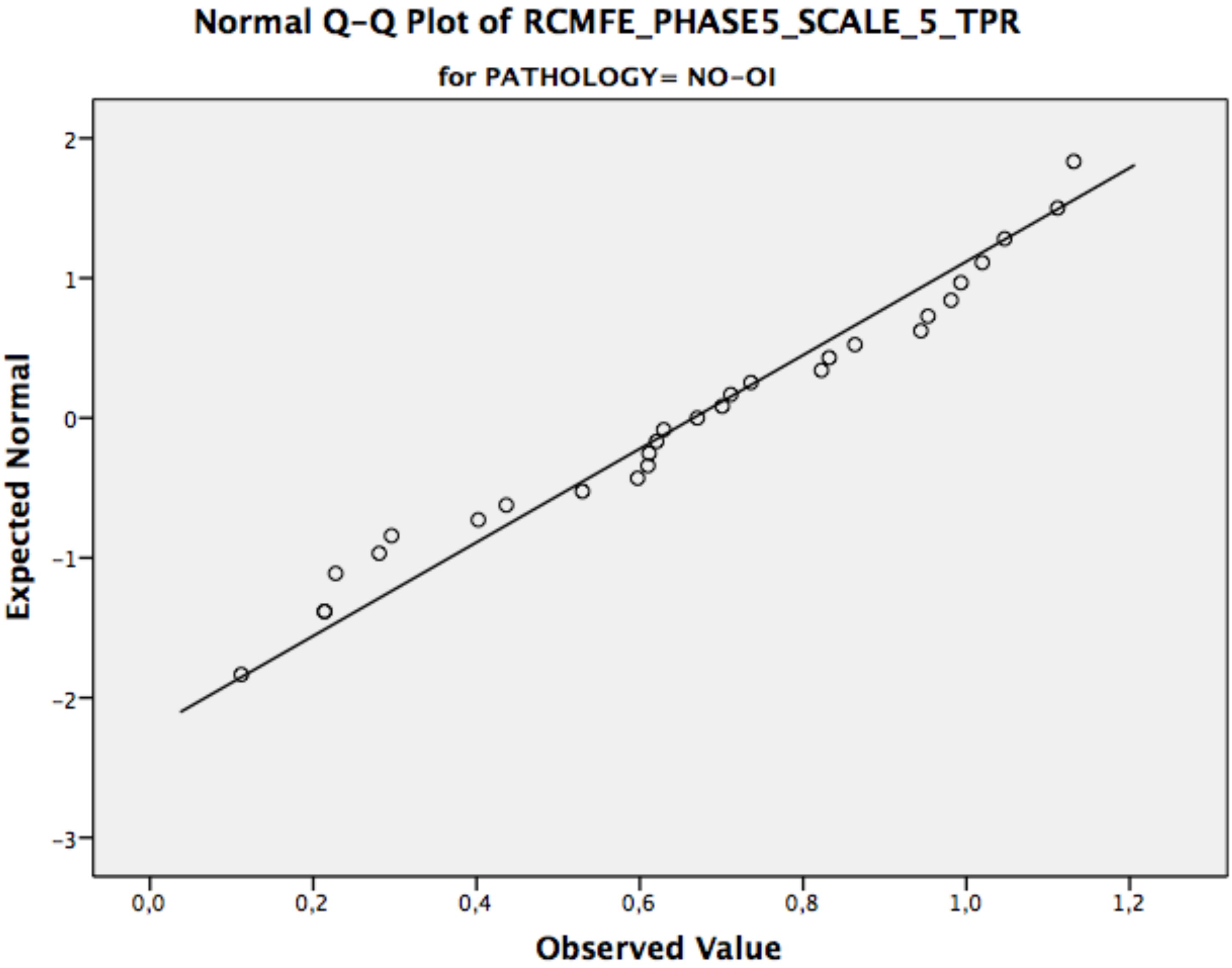

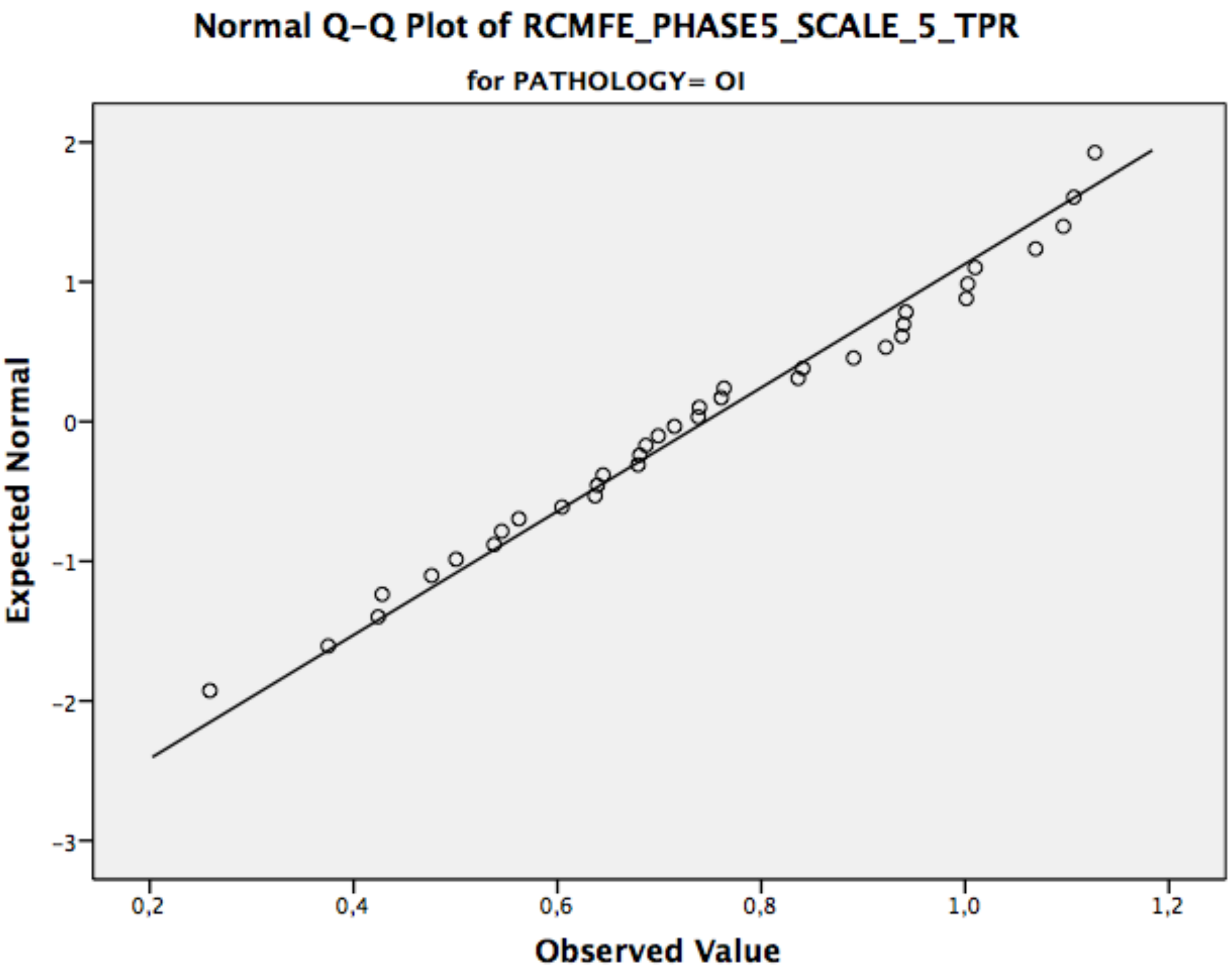

Normal Q-Q Plot of RCMFE_PHASE5_SCALE_5_TPR
for PATHOLOGY= OI
Expected Normal
Observed Value
2
1
0
-1
-2
-3
0,2
0,4
0,6
0,8
1,0
1,2

Explore HR

GENDER

Case Processing Summary

	GENDER	Cases					
		Valid		Missing		Total	
		N	Percent	N	Percent	N	Percent
RCMFE_PHASE1_SCALE_1_HR	WOMEN	42	100,0%	0	0,0%	42	100,0%
	MEN	23	100,0%	0	0,0%	23	100,0%
RCMFE_PHASE1_SCALE_2_HR	WOMEN	42	100,0%	0	0,0%	42	100,0%
	MEN	23	100,0%	0	0,0%	23	100,0%
RCMFE_PHASE1_SCALE_3_HR	WOMEN	42	100,0%	0	0,0%	42	100,0%
	MEN	23	100,0%	0	0,0%	23	100,0%
RCMFE_PHASE1_SCALE_4_HR	WOMEN	42	100,0%	0	0,0%	42	100,0%
	MEN	23	100,0%	0	0,0%	23	100,0%
RCMFE_PHASE1_SCALE_5_HR	WOMEN	42	100,0%	0	0,0%	42	100,0%
	MEN	23	100,0%	0	0,0%	23	100,0%
RCMFE_PHASE2_SCALE_1_HR	WOMEN	42	100,0%	0	0,0%	42	100,0%
	MEN	23	100,0%	0	0,0%	23	100,0%
RCMFE_PHASE2_SCALE_2_HR	WOMEN	42	100,0%	0	0,0%	42	100,0%
	MEN	23	100,0%	0	0,0%	23	100,0%
RCMFE_PHASE2_SCALE_3_HR	WOMEN	42	100,0%	0	0,0%	42	100,0%
	MEN	23	100,0%	0	0,0%	23	100,0%
RCMFE_PHASE2_SCALE_4_HR	WOMEN	42	100,0%	0	0,0%	42	100,0%
	MEN	23	100,0%	0	0,0%	23	100,0%
RCMFE_PHASE2_SCALE_5_HR	WOMEN	42	100,0%	0	0,0%	42	100,0%
	MEN	23	100,0%	0	0,0%	23	100,0%
RCMFE_PHASE3_SCALE_1_HR	WOMEN	42	100,0%	0	0,0%	42	100,0%
	MEN	23	100,0%	0	0,0%	23	100,0%
RCMFE_PHASE3_SCALE_2_HR	WOMEN	42	100,0%	0	0,0%	42	100,0%
	MEN	23	100,0%	0	0,0%	23	100,0%
RCMFE_PHASE3_SCALE_3_HR	WOMEN	42	100,0%	0	0,0%	42	100,0%
	MEN	23	100,0%	0	0,0%	23	100,0%
RCMFE_PHASE3_SCALE_4_HR	WOMEN	42	100,0%	0	0,0%	42	100,0%
	MEN	23	100,0%	0	0,0%	23	100,0%
RCMFE_PHASE3_SCALE_5_HR	WOMEN	42	100,0%	0	0,0%	42	100,0%
	MEN	23	100,0%	0	0,0%	23	100,0%
RCMFE_PHASE4_SCALE_1_HR	WOMEN	42	100,0%	0	0,0%	42	100,0%
	MEN	23	100,0%	0	0,0%	23	100,0%
RCMFE_PHASE4_SCALE_2_HR	WOMEN	42	100,0%	0	0,0%	42	100,0%
	MEN	23	100,0%	0	0,0%	23	100,0%
RCMFE_PHASE4_SCALE_3	WOMEN	42	100,0%	0	0,0%	42	100,0%
	MEN	23	100,0%	0	0,0%	23	100,0%
RCMFE_PHASE4_SCALE_4_HR	WOMEN	42	100,0%	0	0,0%	42	100,0%
	MEN	23	100,0%	0	0,0%	23	100,0%
RCMFE_PHASE4_SCALE_5_HR	WOMEN	42	100,0%	0	0,0%	42	100,0%
	MEN	23	100,0%	0	0,0%	23	100,0%
RCMFE_PHASE5_SCALE_1_HR	WOMEN	42	100,0%	0	0,0%	42	100,0%

Case Processing Summary

	GENDER	Cases					
		Valid		Missing		Total	
		N	Percent	N	Percent	N	Percent
RCMFE_PHASE5_SCALE_1_HR	MEN	23	100,0%	0	0,0%	23	100,0%
RCMFE_PHASE5_SCALE_2_HR	WOMEN	42	100,0%	0	0,0%	42	100,0%
	MEN	23	100,0%	0	0,0%	23	100,0%
RCMFE_PHASE5_SCALE_3_HR	WOMEN	42	100,0%	0	0,0%	42	100,0%
	MEN	23	100,0%	0	0,0%	23	100,0%
RCMFE_PHASE5_SCALE_4_HR	WOMEN	42	100,0%	0	0,0%	42	100,0%
	MEN	23	100,0%	0	0,0%	23	100,0%
RCMFE_PHASE5_SCALE_5_HR	WOMEN	42	100,0%	0	0,0%	42	100,0%
	MEN	23	100,0%	0	0,0%	23	100,0%

Tests of Normality

	GENDER	Kolmogorov-Smirnov[a]			Shapiro-Wilk		
		Statistic	df	Sig.	Statistic	df	Sig.
RCMFE_PHASE1_SCALE_1_HR	WOMEN	,153	42	,015	,975	42	,482
	MEN	,159	23	,135	,950	23	,299
RCMFE_PHASE1_SCALE_2_HR	WOMEN	,067	42	,200*	,975	42	,471
	MEN	,181	23	,049	,921	23	,071
RCMFE_PHASE1_SCALE_3_HR	WOMEN	,070	42	,200*	,989	42	,956
	MEN	,163	23	,118	,955	23	,369
RCMFE_PHASE1_SCALE_4_HR	WOMEN	,068	42	,200*	,978	42	,592
	MEN	,096	23	,200*	,975	23	,810
RCMFE_PHASE1_SCALE_5_HR	WOMEN	,114	42	,199	,973	42	,407
	MEN	,129	23	,200*	,942	23	,194
RCMFE_PHASE2_SCALE_1_HR	WOMEN	,119	42	,150	,931	42	,054
	MEN	,168	23	,092	,897	23	,062
RCMFE_PHASE2_SCALE_2_HR	WOMEN	,144	42	,029	,872	42	,065
	MEN	,206	23	,012	,871	23	,077
RCMFE_PHASE2_SCALE_3_HR	WOMEN	,158	42	,010	,841	42	,079
	MEN	,208	23	,071	,884	23	,012
RCMFE_PHASE2_SCALE_4_HR	WOMEN	,173	42	,003	,830	42	,066
	MEN	,190	23	,031	,901	23	,057
RCMFE_PHASE2_SCALE_5_HR	WOMEN	,166	42	,005	,788	42	,050
	MEN	,150	23	,195	,916	23	,054
RCMFE_PHASE3_SCALE_1_HR	WOMEN	,121	42	,132	,956	42	,102
	MEN	,155	23	,161	,924	23	,083
RCMFE_PHASE3_SCALE_2_HR	WOMEN	,094	42	,200*	,960	42	,148
	MEN	,081	23	,200*	,979	23	,880
RCMFE_PHASE3_SCALE_3_HR	WOMEN	,062	42	,200*	,987	42	,899
	MEN	,084	23	,200*	,985	23	,968
RCMFE_PHASE3_SCALE_4_HR	WOMEN	,087	42	,200*	,986	42	,871
	MEN	,122	23	,200*	,939	23	,171
RCMFE_PHASE3_SCALE_5_	WOMEN	,091	42	,200*	,967	42	,254

HR	MEN	,123	23	,200*	,954	23	,347
RCMFE_PHASE4_SCALE_1_	WOMEN	,154	42	,013	,900	42	,061
HR	MEN	,090	23	,200*	,945	23	,233
RCMFE_PHASE4_SCALE_2_	WOMEN	,164	42	,006	,889	42	,067
HR	MEN	,109	23	,200*	,940	23	,179
RCMFE_PHASE4_SCALE_3	WOMEN	,157	42	,011	,848	42	,055
	MEN	,160	23	,131	,936	23	,145
RCMFE_PHASE4_SCALE_4_	WOMEN	,182	42	,001	,833	42	,054
HR	MEN	,154	23	,167	,913	23	,047
RCMFE_PHASE4_SCALE_5_	WOMEN	,183	42	,001	,788	42	,053
HR	MEN	,179	23	,054	,917	23	,059
RCMFE_PHASE5_SCALE_1_	WOMEN	,101	42	,200*	,974	42	,439
HR	MEN	,088	23	,200*	,988	23	,990

Tests of Normality

	GENDER	Kolmogorov-Smirnov[a]			Shapiro-Wilk		
		Statistic	df	Sig.	Statistic	df	Sig.
RCMFE_PHASE5_SCALE_2_	WOMEN	,079	42	,200	,976	42	,500
HR	MEN	,092	23	,200	,979	23	,884
RCMFE_PHASE5_SCALE_3_	WOMEN	,104	42	,200*	,973	42	,412
HR	MEN	,146	23	,200	,974	23	,784
RCMFE_PHASE5_SCALE_4_	WOMEN	,126	42	,090*	,953	42	,084
HR	MEN	,102	23	,200	,973	23	,771
RCMFE_PHASE5_SCALE_5_	WOMEN	,087	42	,200*	,971	42	,354
HR	MEN	,108	23	,200*	,967	23	,608

*. This is a lower bound of the true significance.

a. Lilliefors Significance Correction

RCMFE_PHASE1_SCALE_1_HR

Normal Q-Q Plots

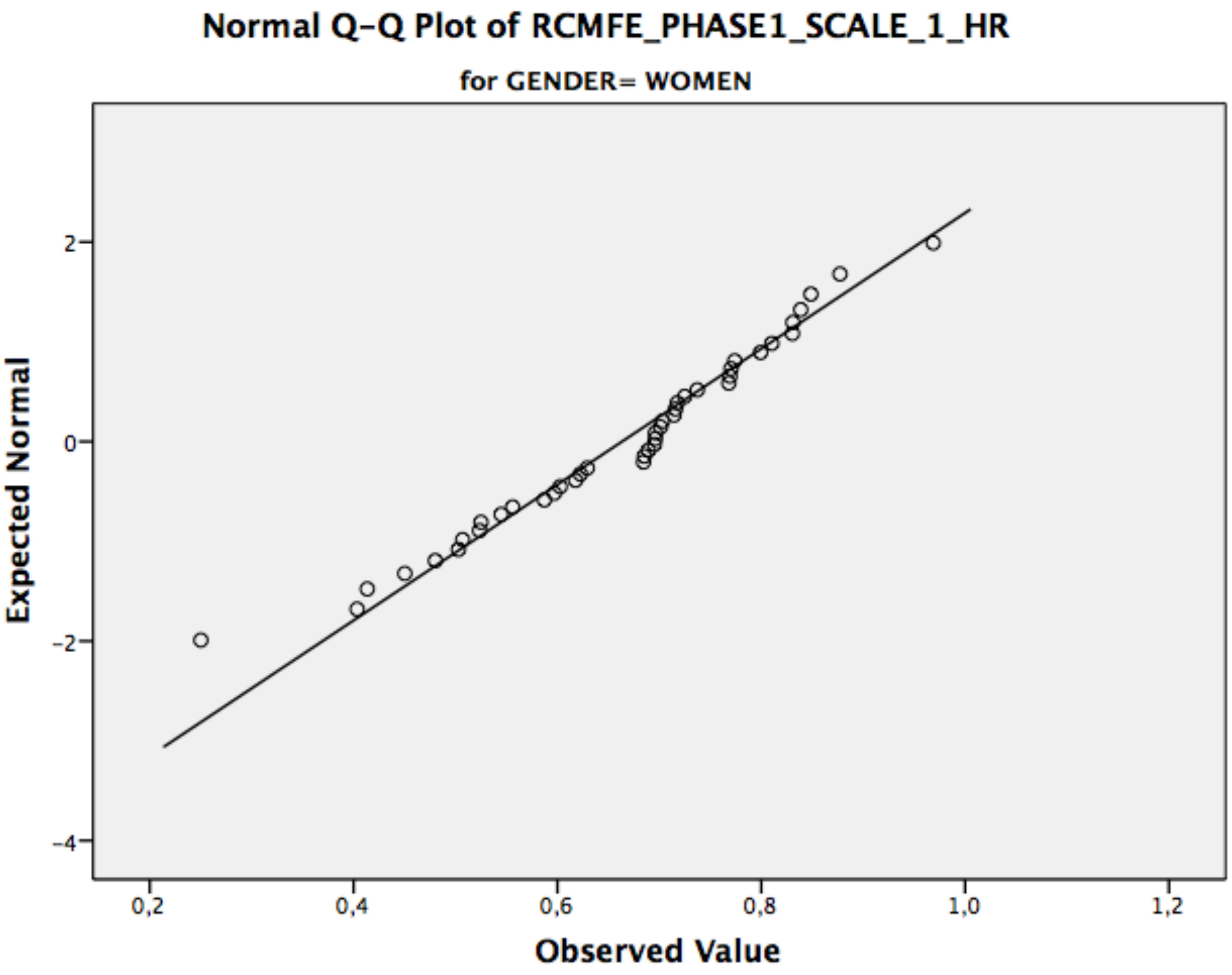

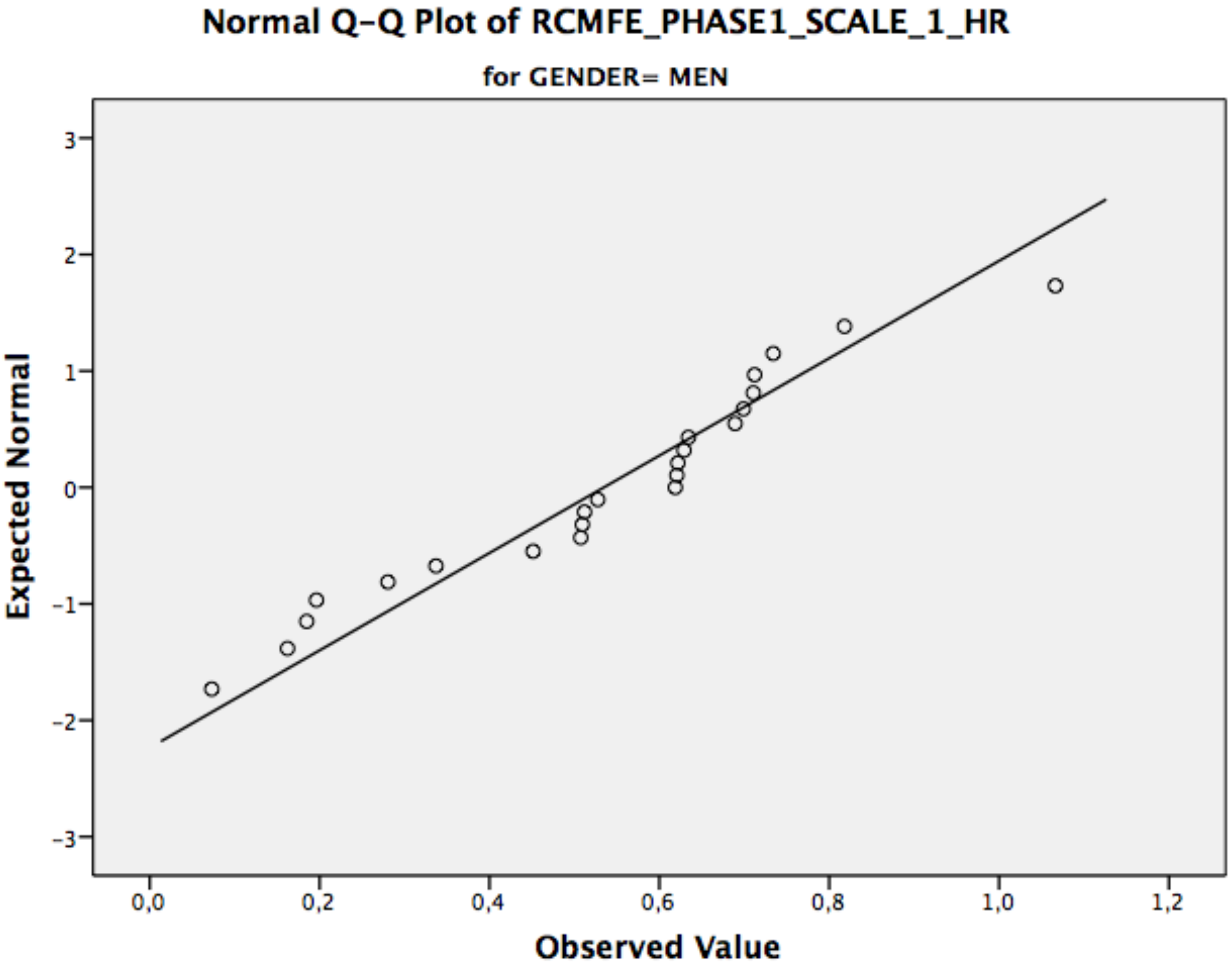
Normal Q-Q Plot of RCMFE_PHASE1_SCALE_1_HR
for GENDER= MEN
Expected Normal
Observed Value
3
2
1
0
-1
-2
-3
0,0
0,2
0,4
0,6
0,8
1,0
1,2

RCMFE_PHASE1_SCALE_2_HR

Normal Q-Q Plots

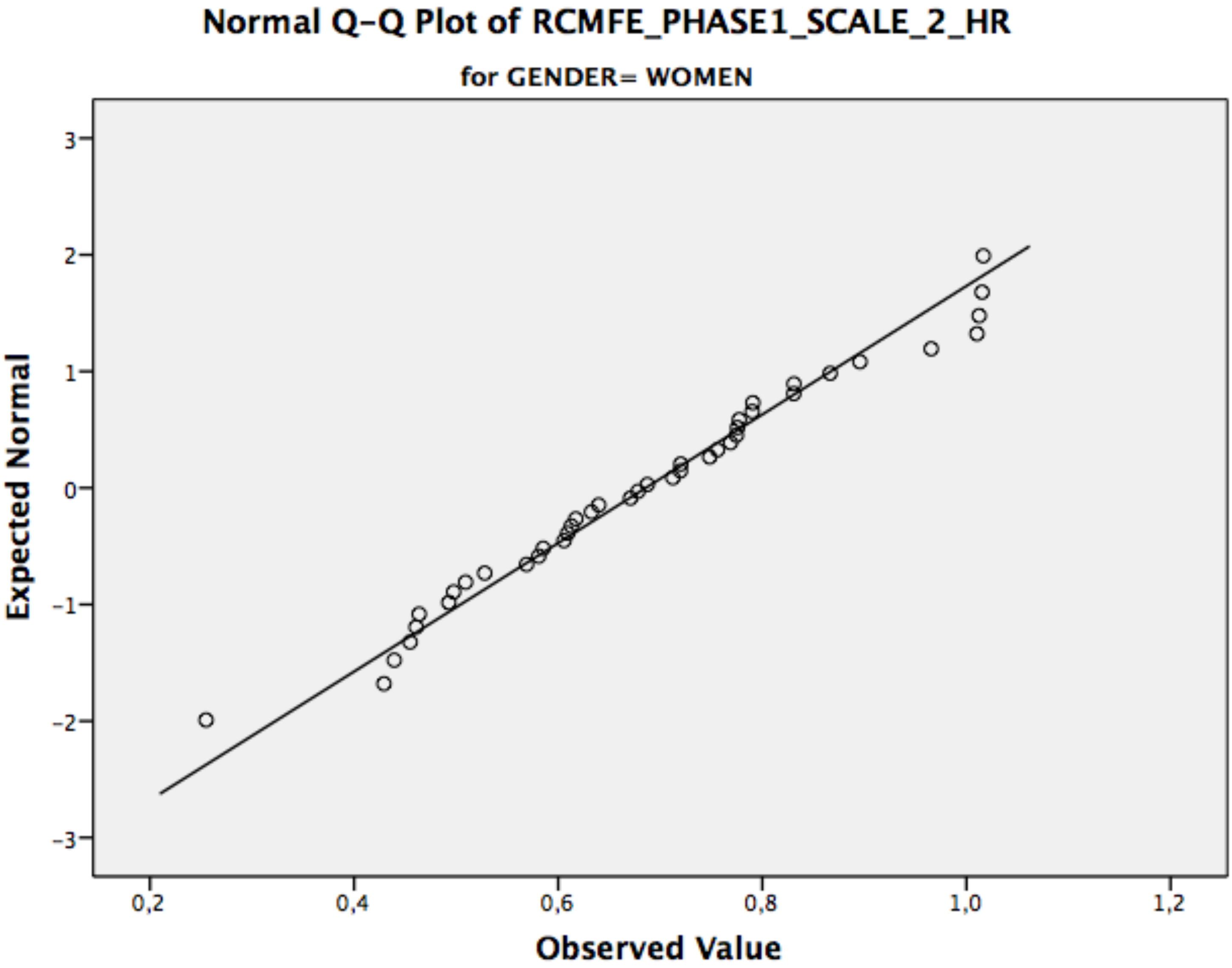

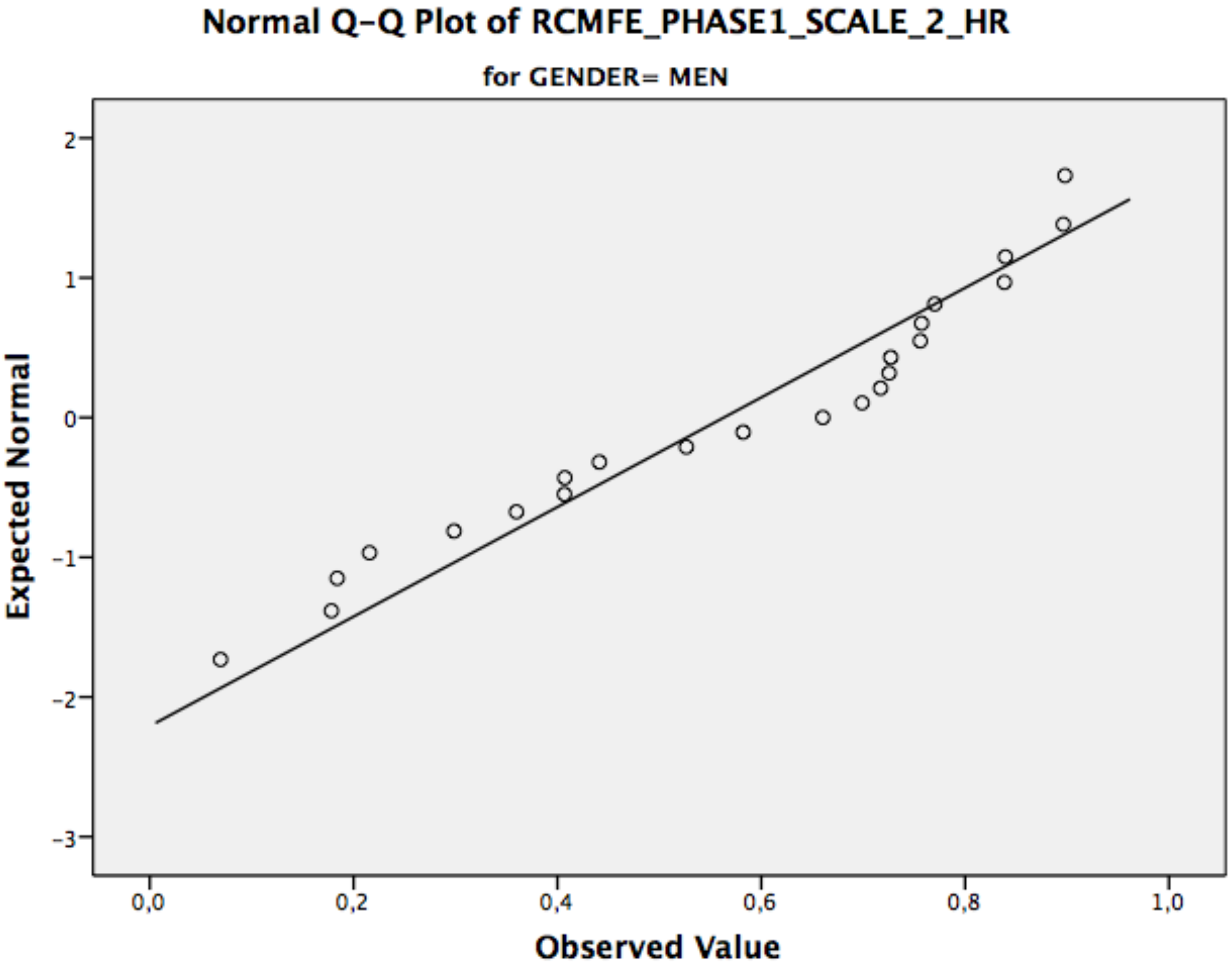

Normal Q-Q Plot of RCMFE_PHASE1_SCALE_2_HR
for GENDER= MEN
Expected Normal
Observed Value
2
1
0
-1
-2
-3
0,0
0,2
0,4
0,6
0,8
1,0

RCMFE_PHASE1_SCALE_3_HR

Normal Q-Q Plots

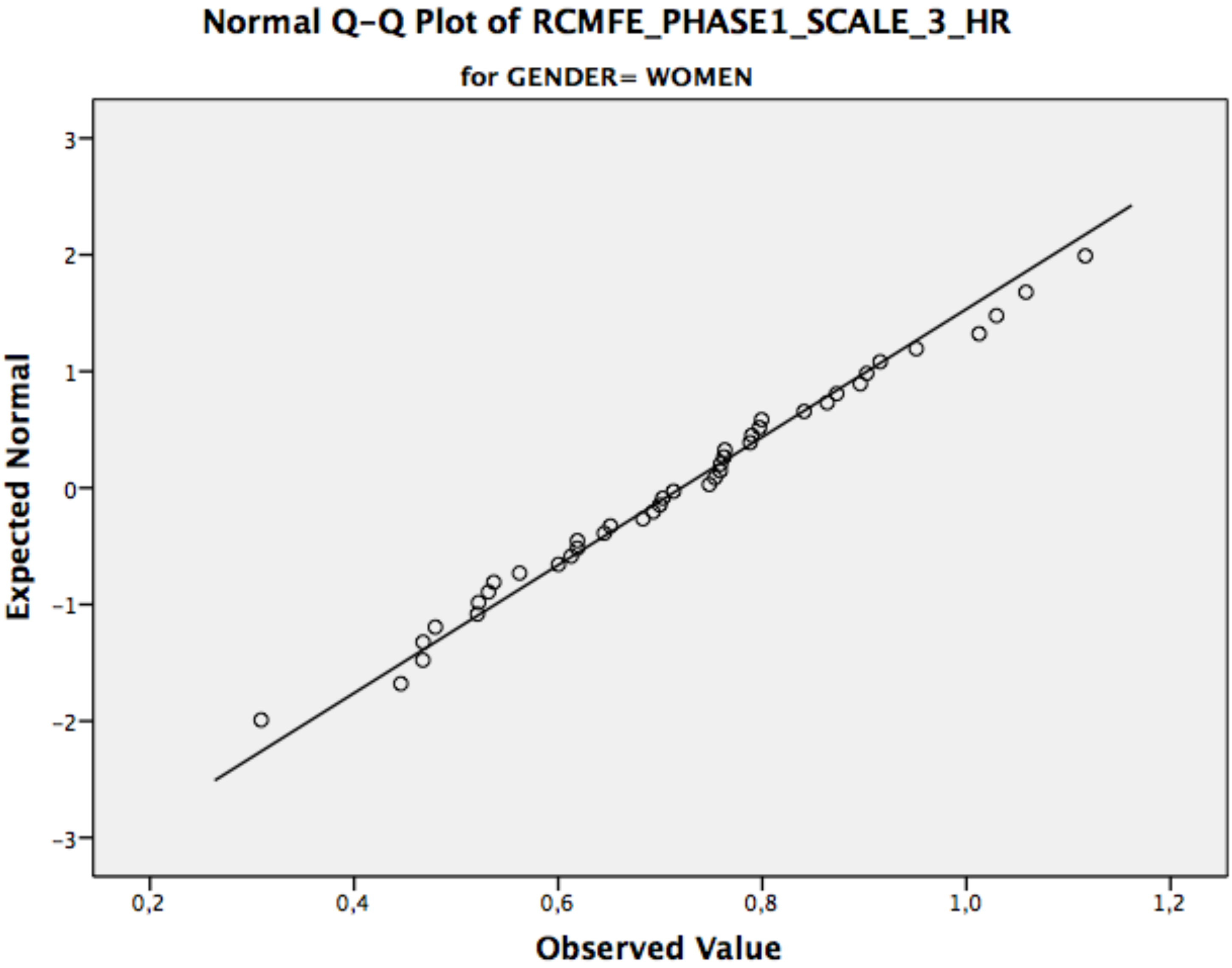

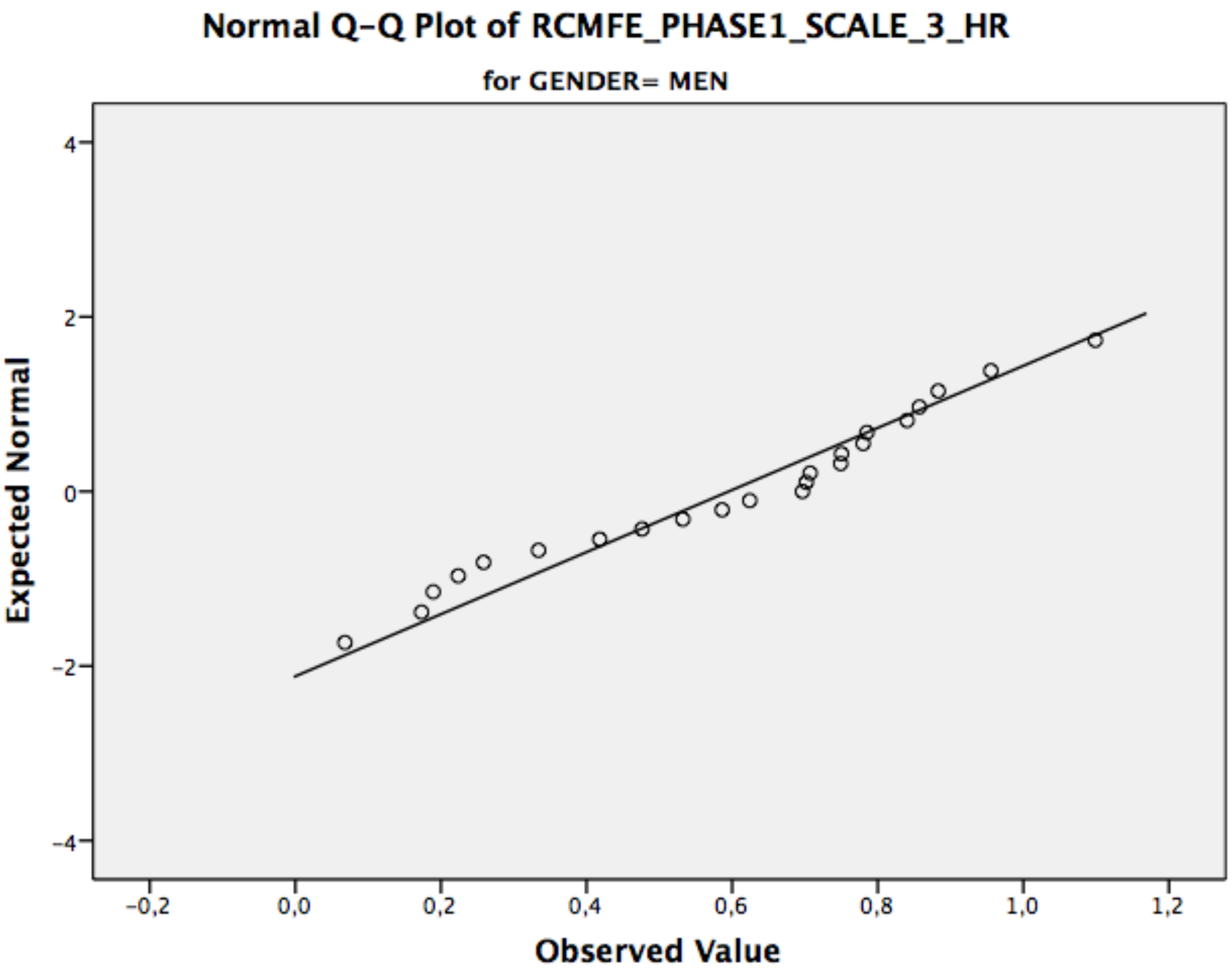

Normal Q-Q Plot of RCMFE_PHASE1_SCALE_3_HR
for GENDER= MEN
Expected Normal
Observed Value
4
2
0
-2
-4
-0,2
0,0
0,2
0,4
0,6
0,8
1,0
1,2

RCMFE_PHASE1_SCALE_4_HR

Normal Q-Q Plots

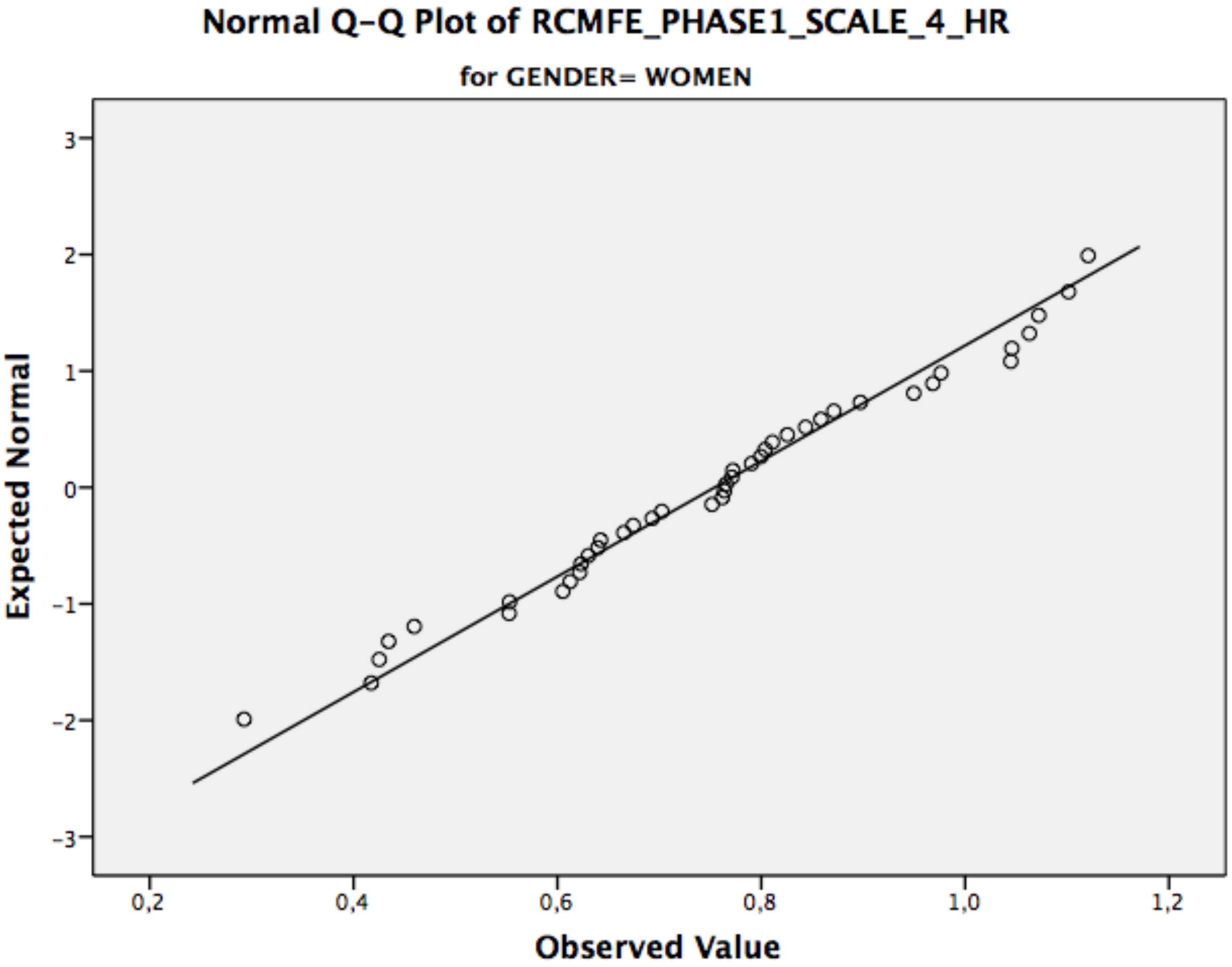

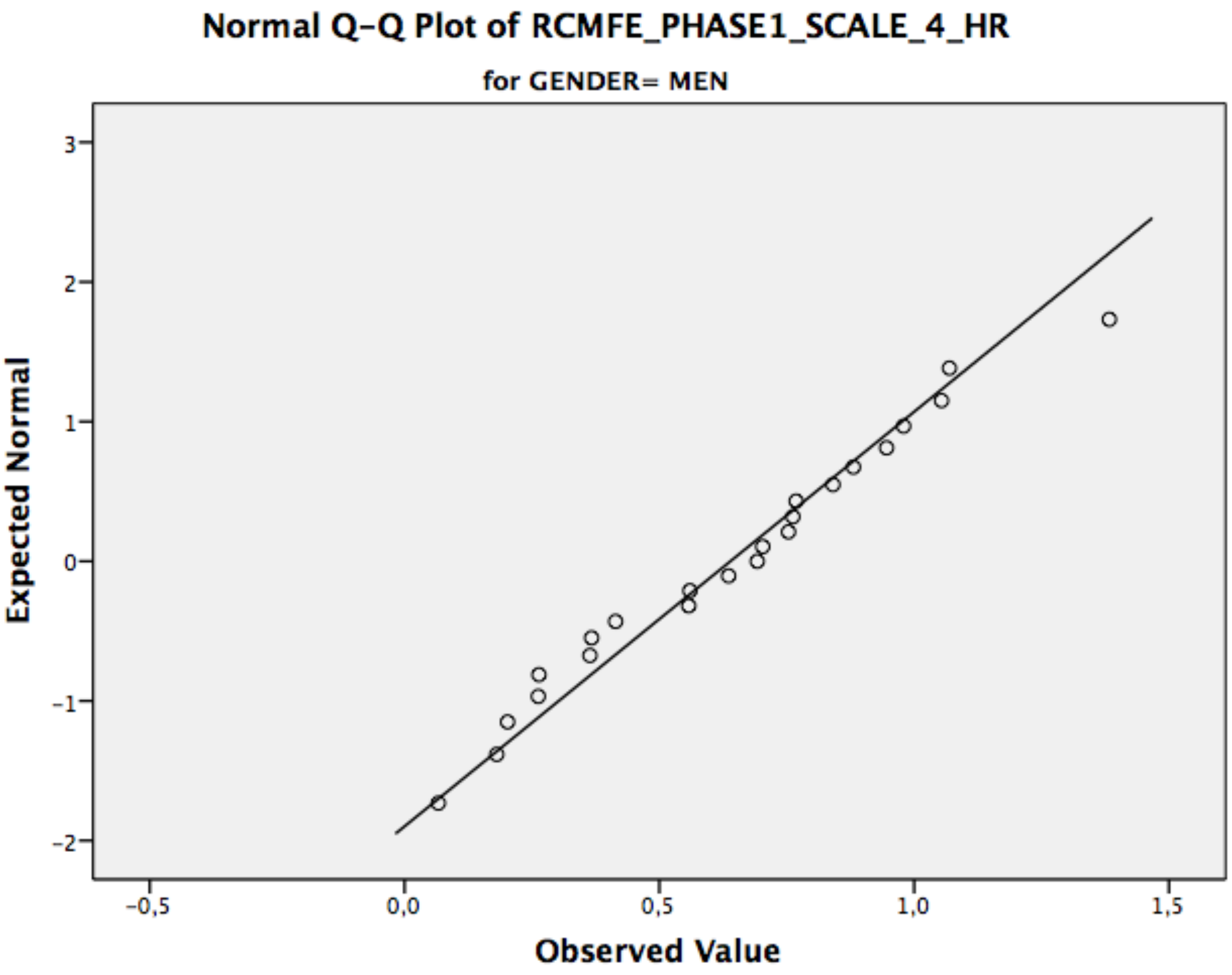
Normal Q-Q Plot of RCMFE_PHASE1_SCALE_4_HR
for GENDER= MEN
Expected Normal
Observed Value
3
2
1
0
-1
-2
-0,5
0,0
0,5
1,0
1,5

RCMFE_PHASE1_SCALE_5_HR

Normal Q-Q Plots

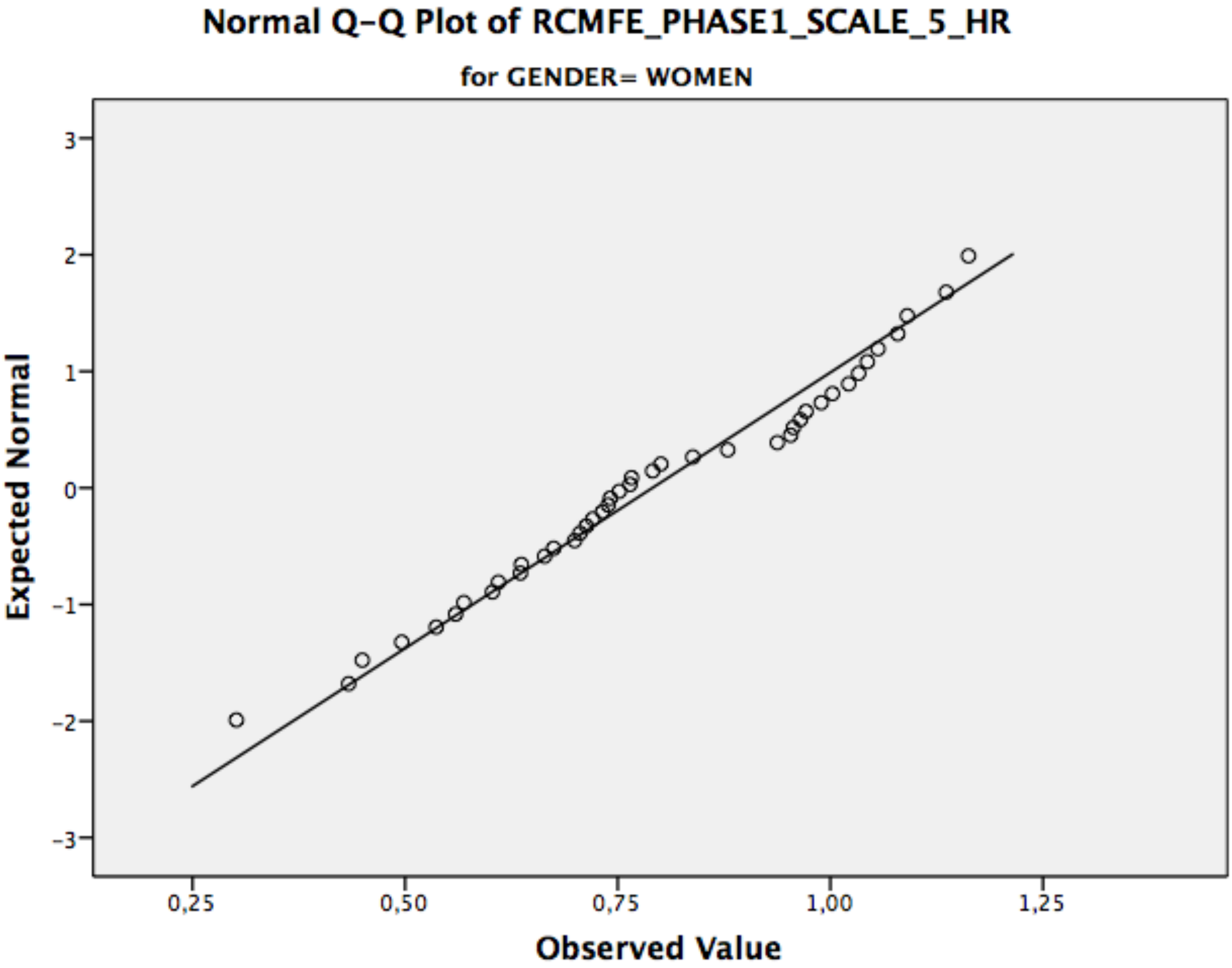

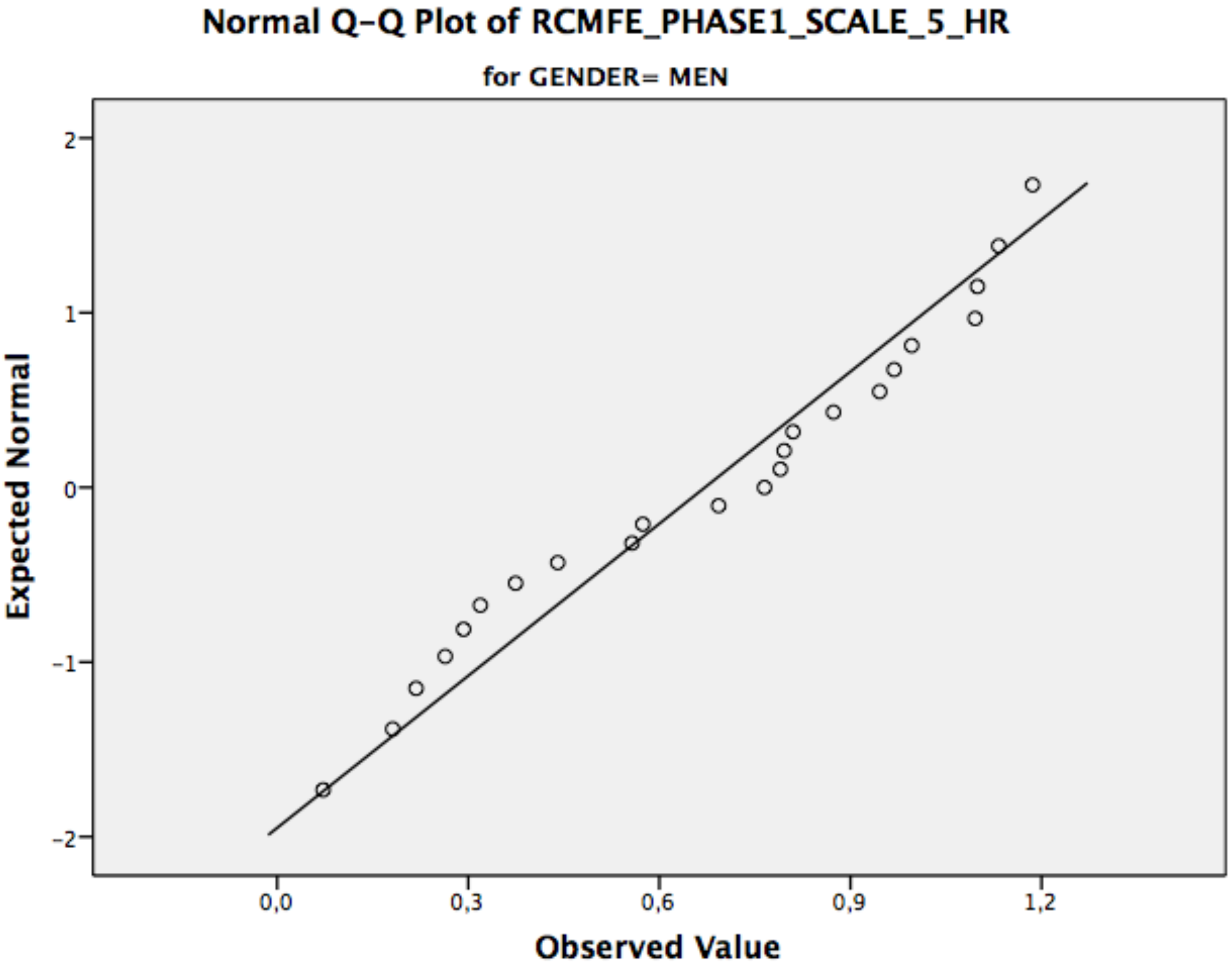
Normal Q-Q Plot of RCMFE_PHASE1_SCALE_5_HR
for GENDER= MEN
Expected Normal
2
1
0
-1
-2
0,0
0,3
0,6
0,9
1,2
Observed Value

RCMFE_PHASE2_SCALE_1_HR

Normal Q-Q Plots

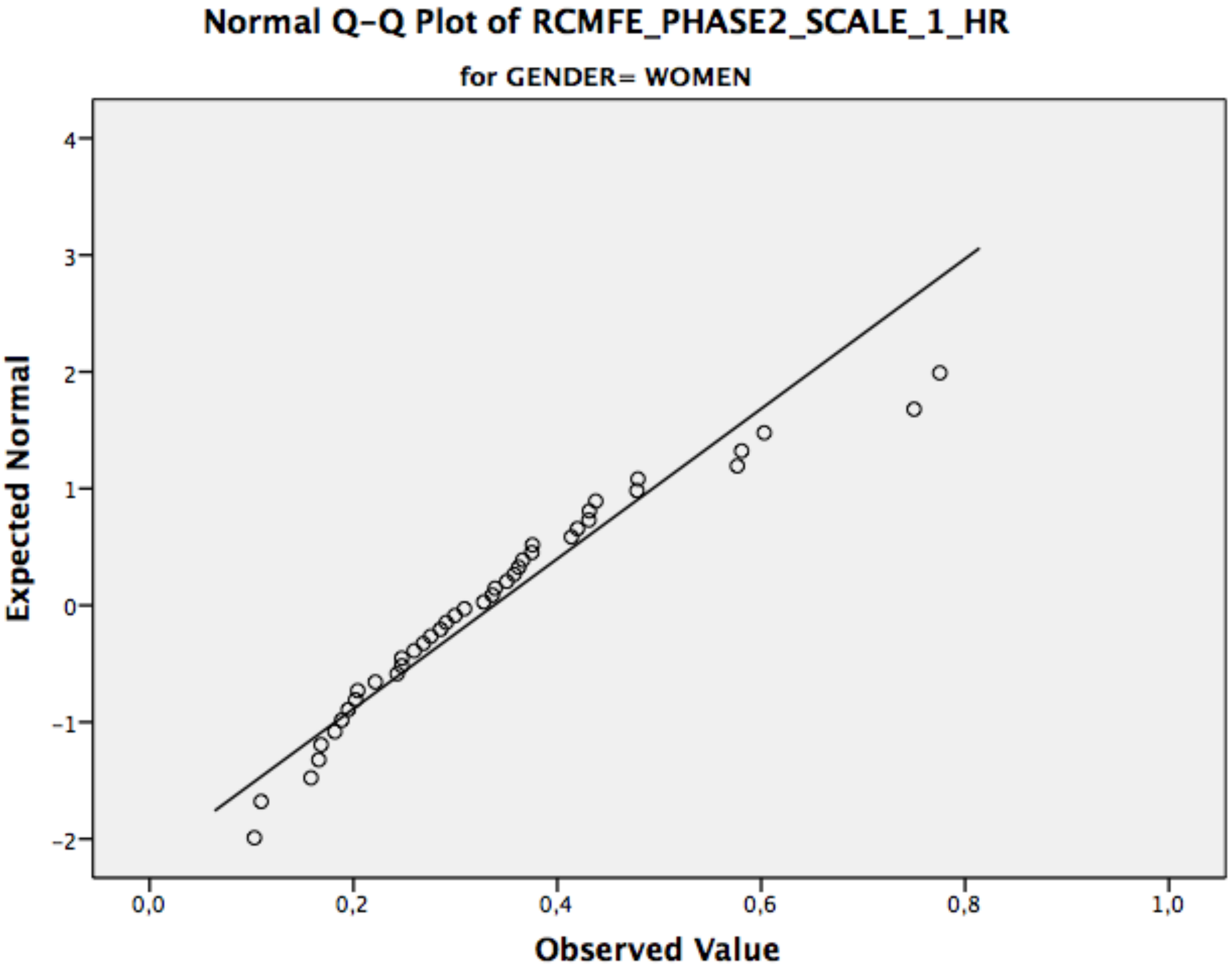

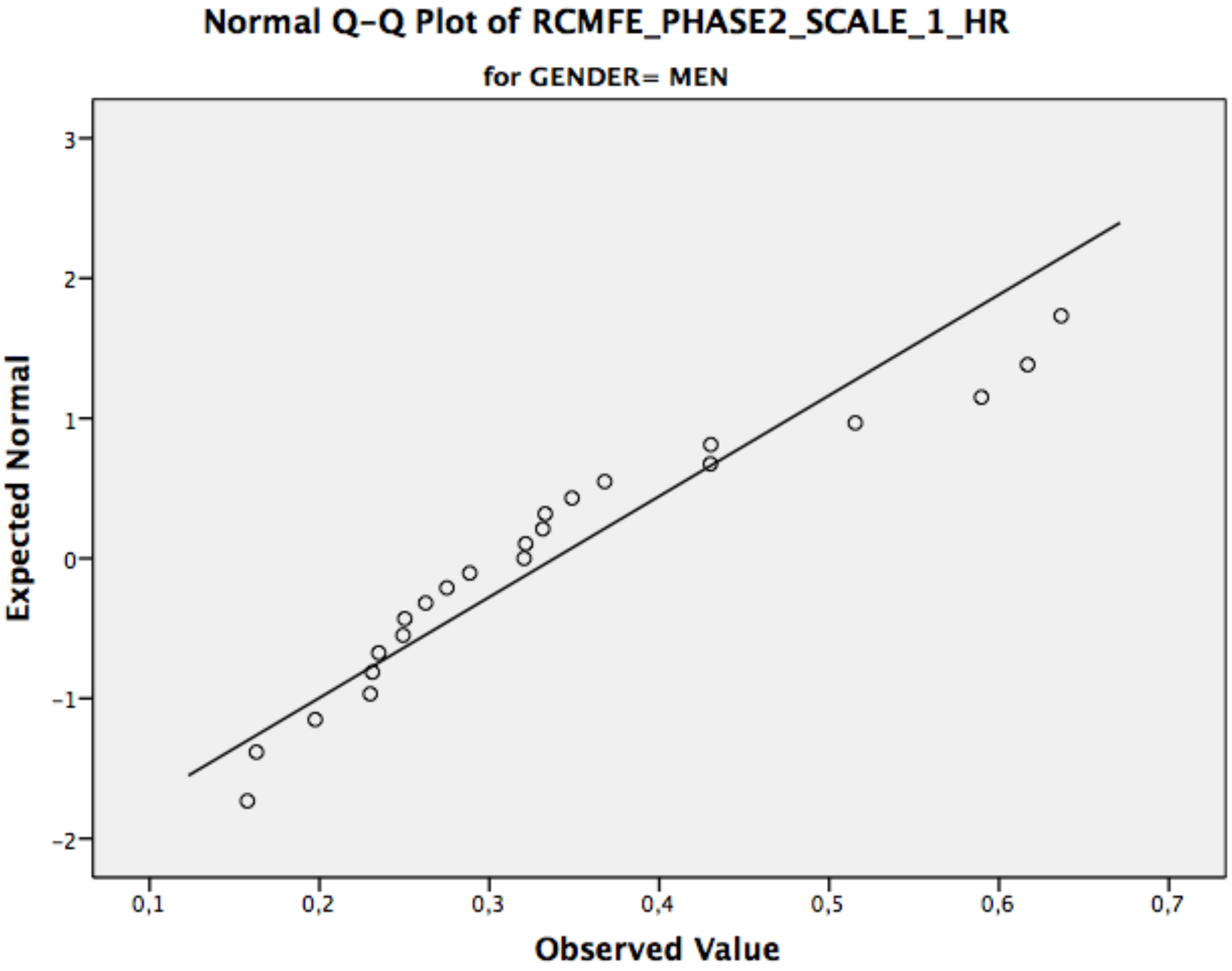
Normal Q-Q Plot of RCMFE_PHASE2_SCALE_1_HR
for GENDER= MEN
Expected Normal
Observed Value
3
2
1
0
-1
-2
0,1
0,2
0,3
0,4
0,5
0,6
0,7

RCMFE_PHASE2_SCALE_2_HR

Normal Q-Q Plots

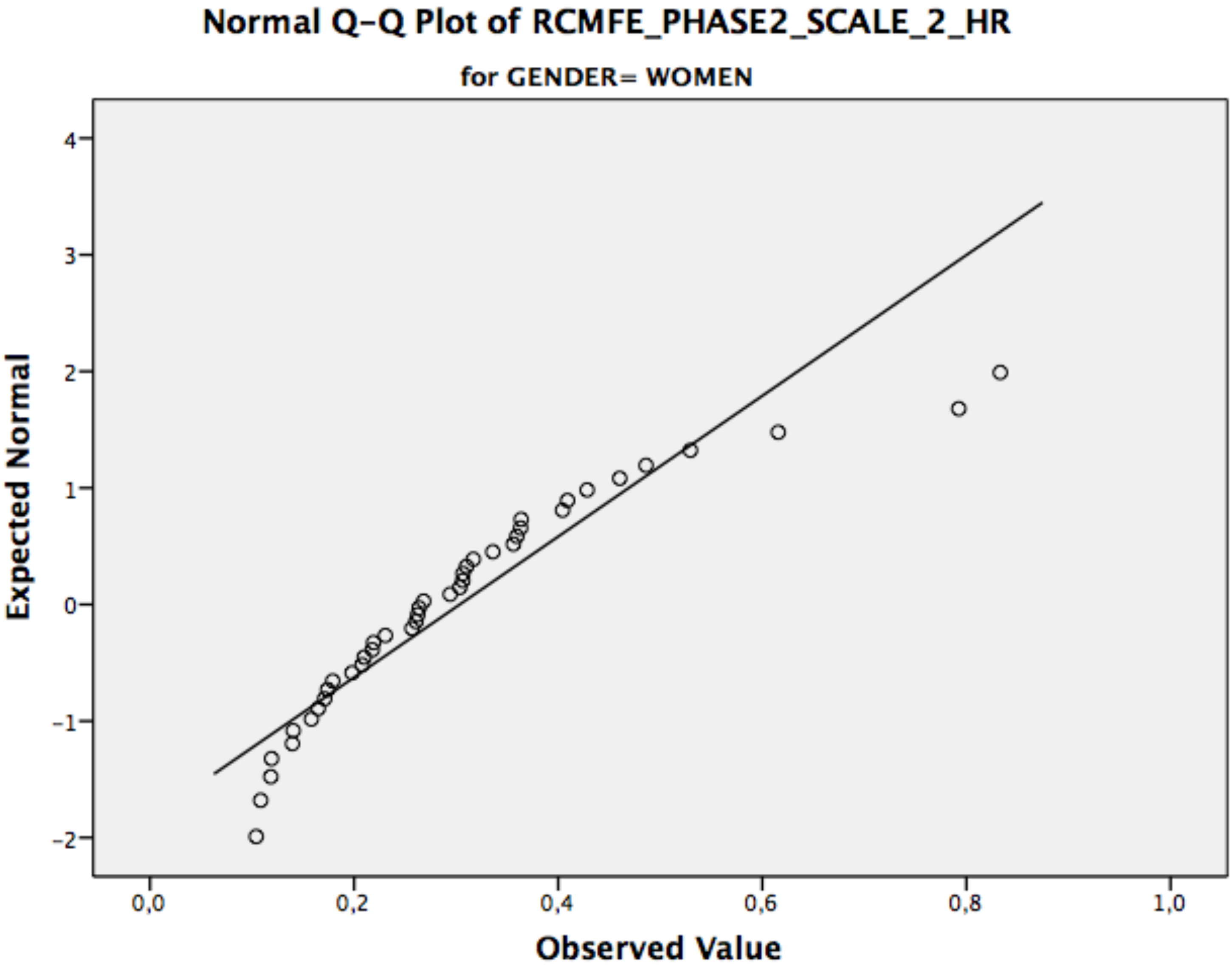

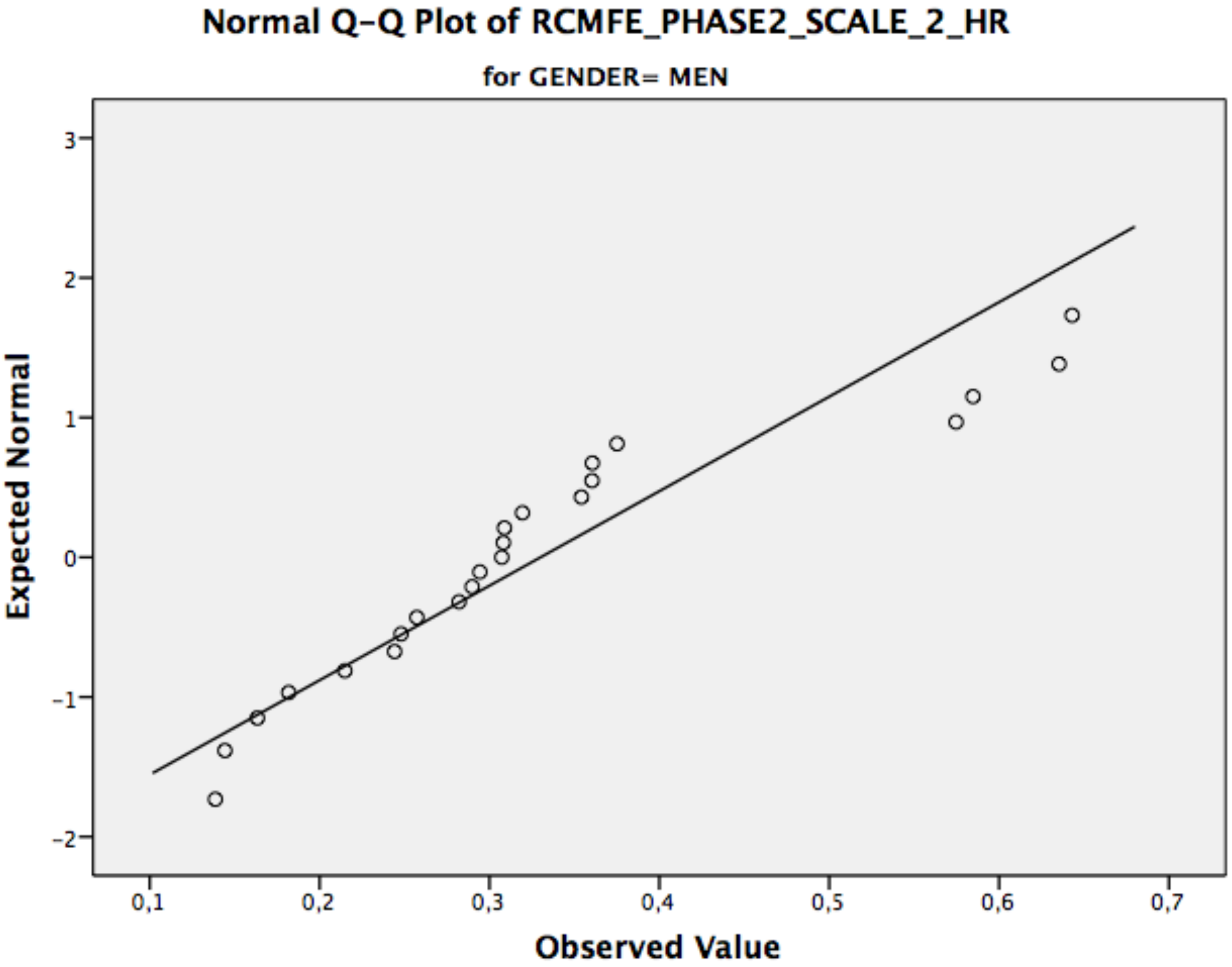
Normal Q-Q Plot of RCMFE_PHASE2_SCALE_2_HR
for GENDER= MEN
Expected Normal
3
2
1
0
-1
-2
0,1
0,2
0,3
0,4
0,5
0,6
0,7
Observed Value

RCMFE_PHASE2_SCALE_3_HR

Normal Q-Q Plots

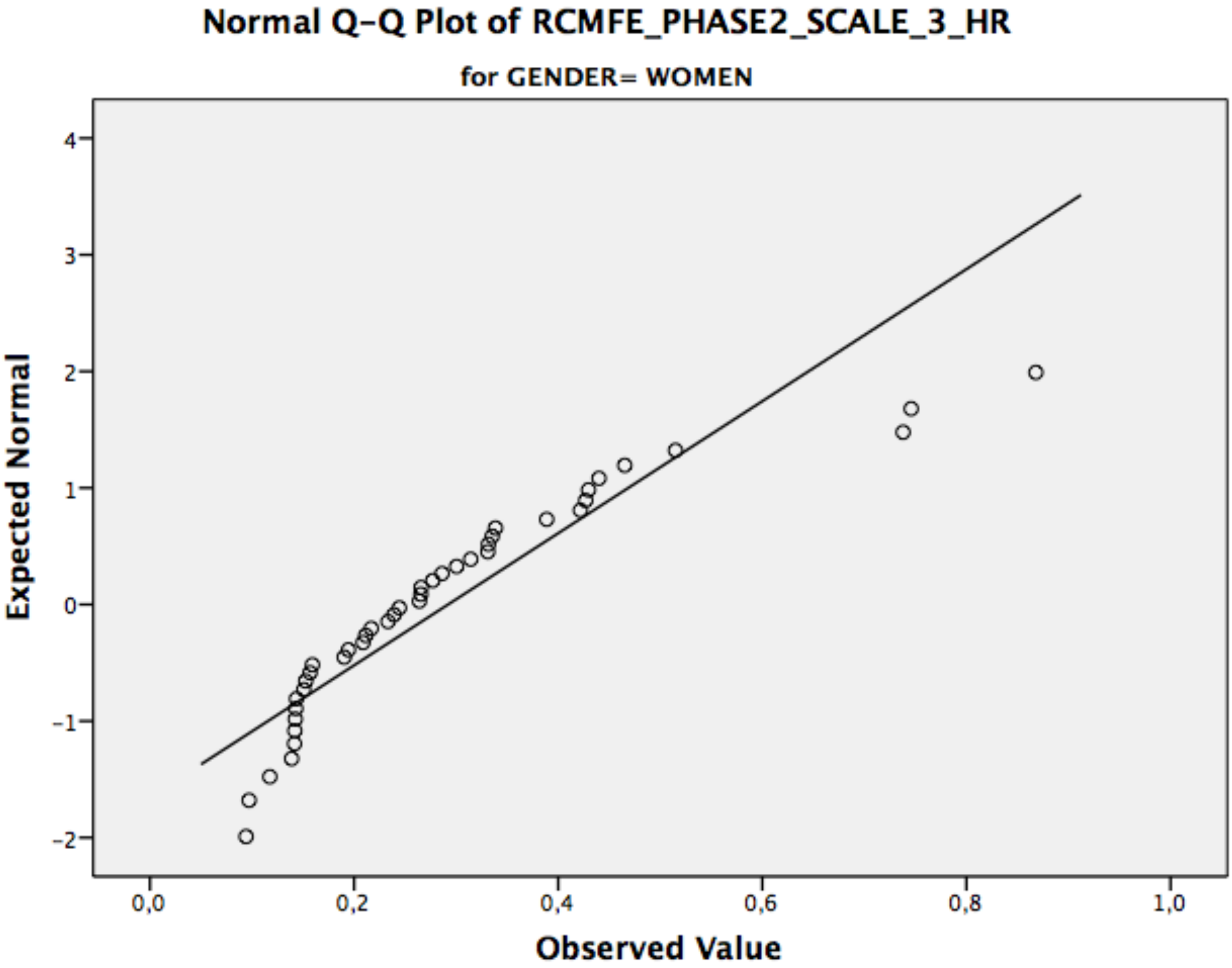

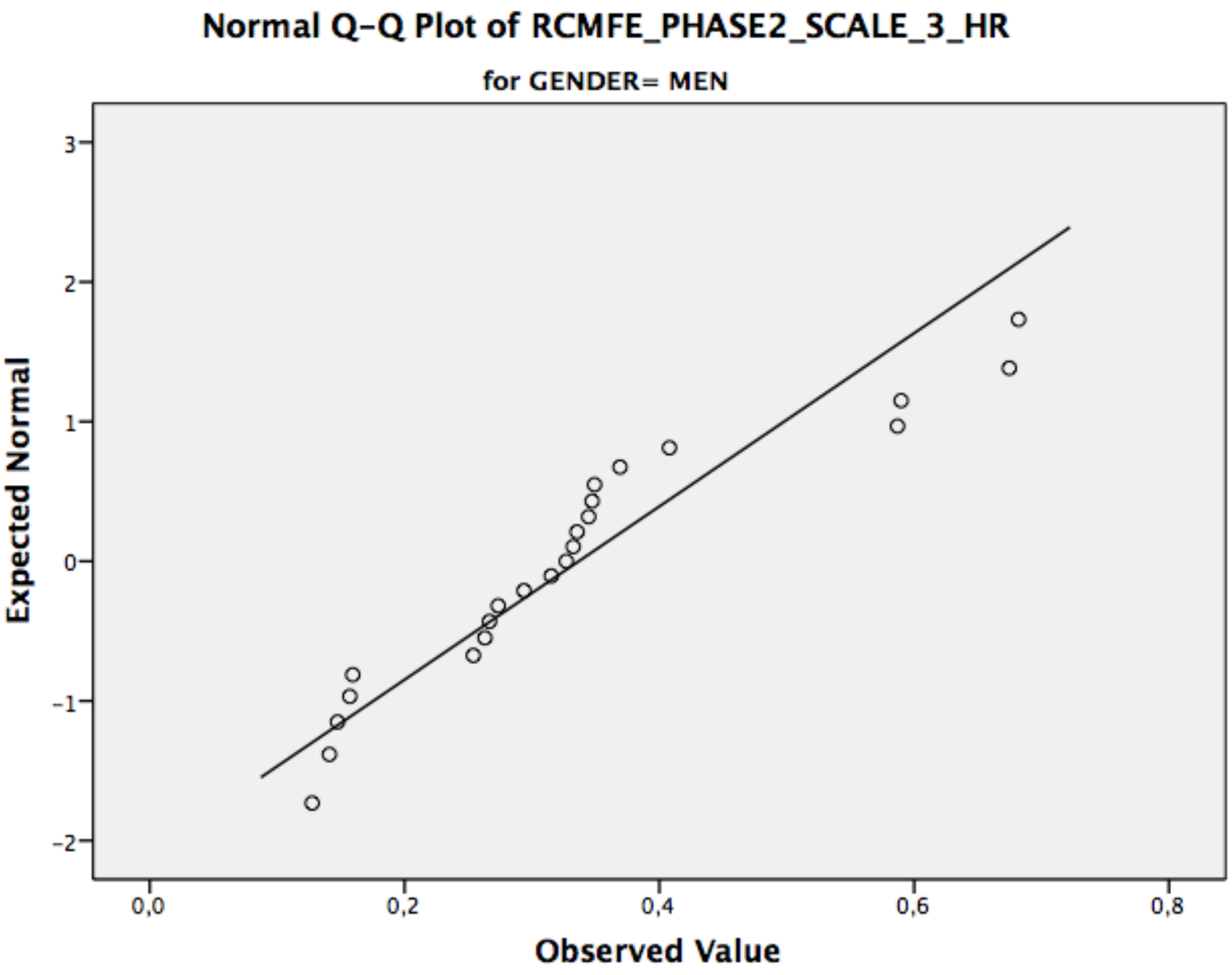
Normal Q-Q Plot of RCMFE_PHASE2_SCALE_3_HR
for GENDER= MEN
Expected Normal
Observed Value
3
2
1
0
-1
-2
0,0
0,2
0,4
0,6
0,8

RCMFE_PHASE2_SCALE_4_HR

Normal Q-Q Plots

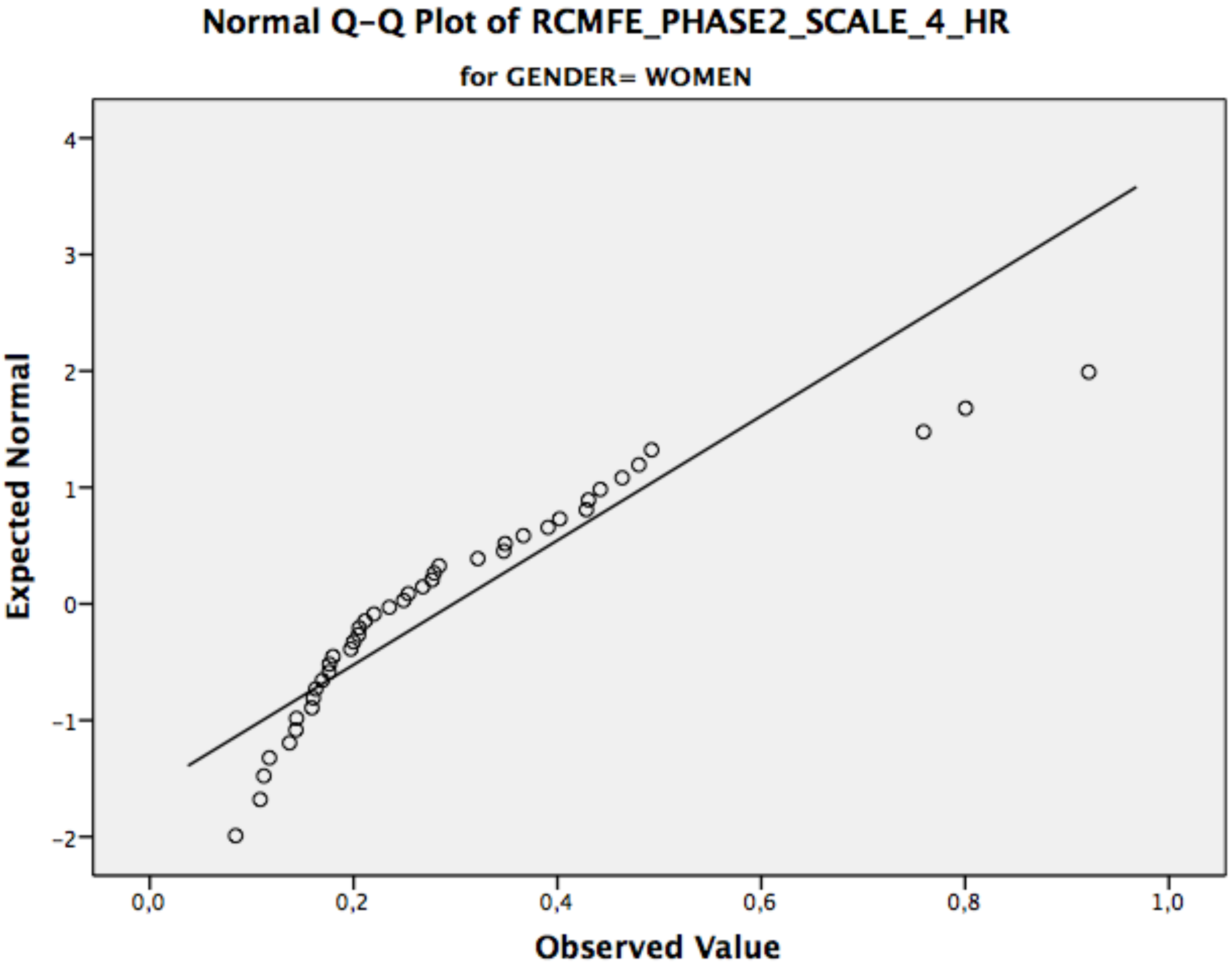

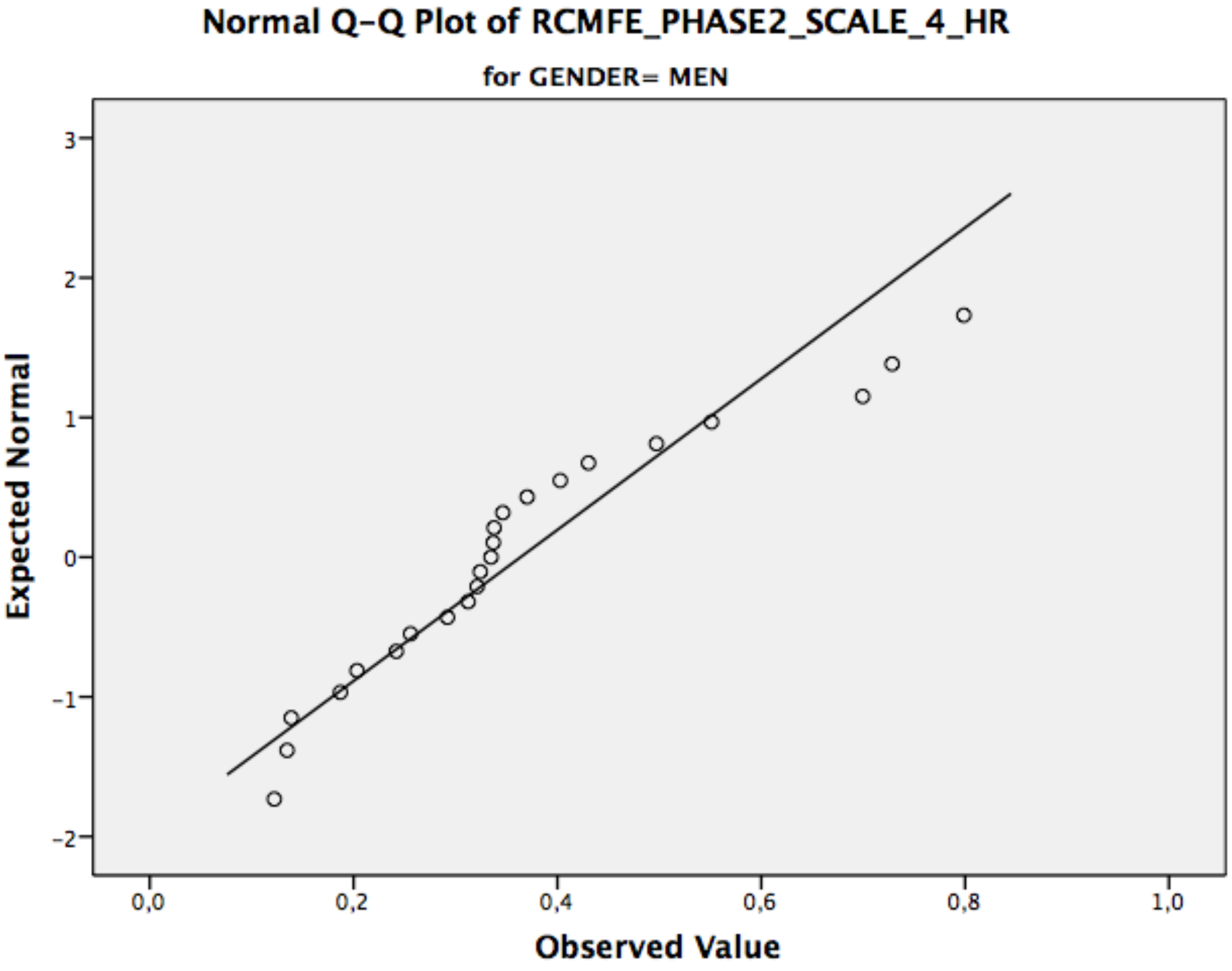

Normal Q-Q Plot of RCMFE_PHASE2_SCALE_4_HR
for GENDER= MEN
Expected Normal
Observed Value
3
2
1
0
-1
-2
0,0
0,2
0,4
0,6
0,8
1,0

RCMFE_PHASE2_SCALE_5_HR

Normal Q-Q Plots

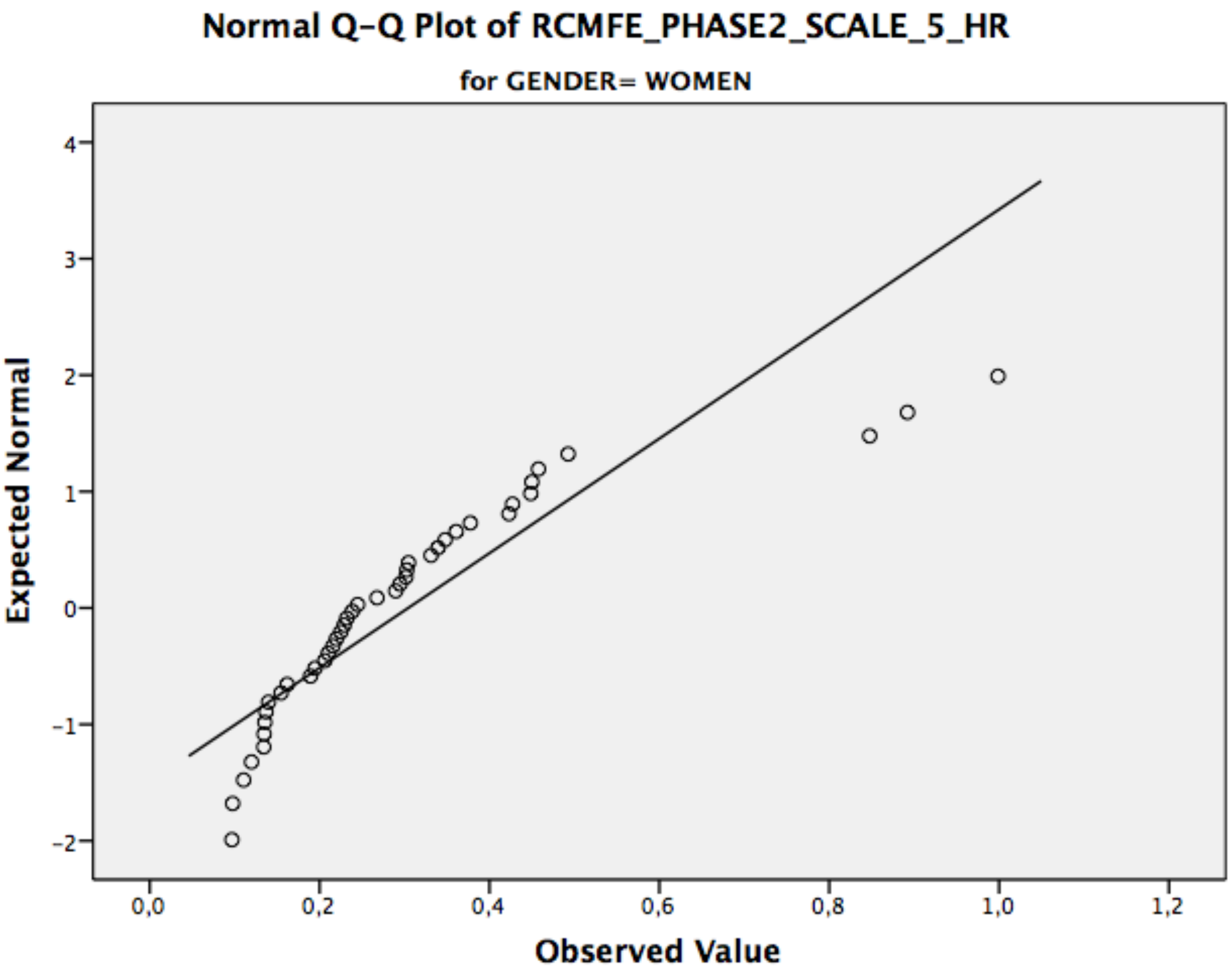

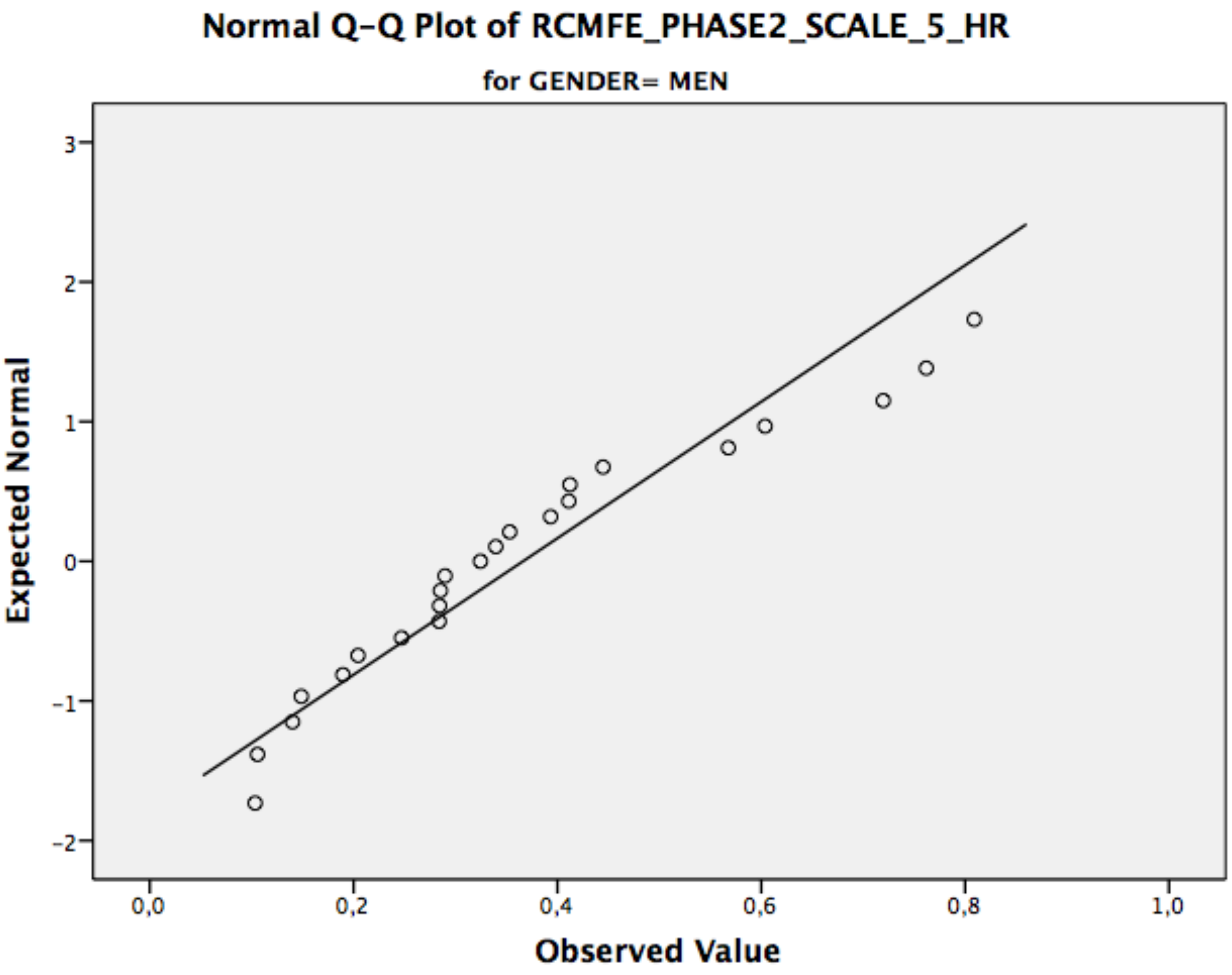

Normal Q-Q Plot of RCMFE_PHASE2_SCALE_5_HR
for GENDER= MEN
Expected Normal
Observed Value
3
2
1
0
-1
-2
0,0
0,2
0,4
0,6
0,8
1,0

RCMFE_PHASE3_SCALE_1_HR

Normal Q-Q Plots

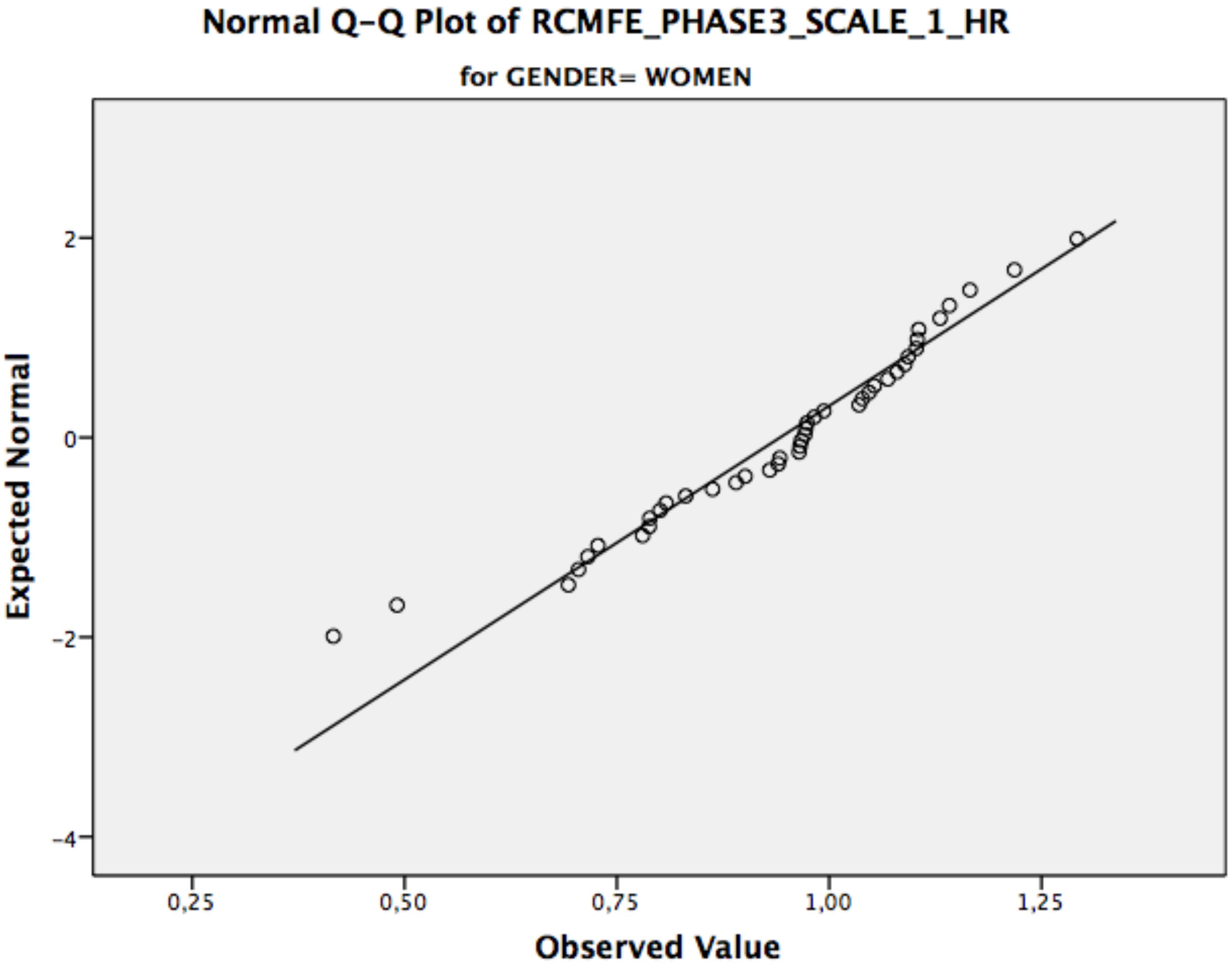

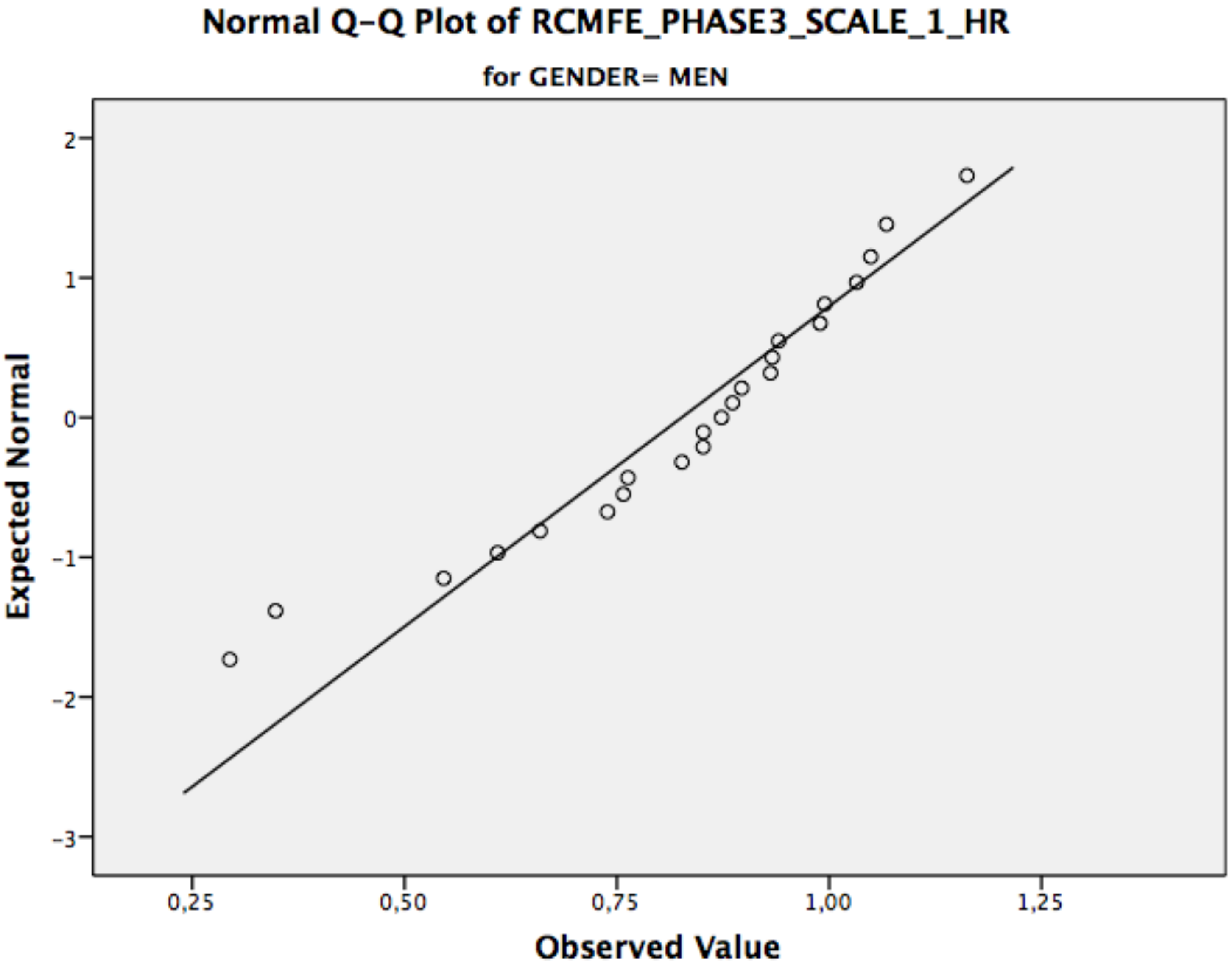

Normal Q-Q Plot of RCMFE_PHASE3_SCALE_1_HR
for GENDER= MEN
Expected Normal
2
1
0
-1
-2
-3
0,25
0,50
0,75
1,00
1,25
Observed Value

RCMFE_PHASE3_SCALE_2_HR

Normal Q-Q Plots

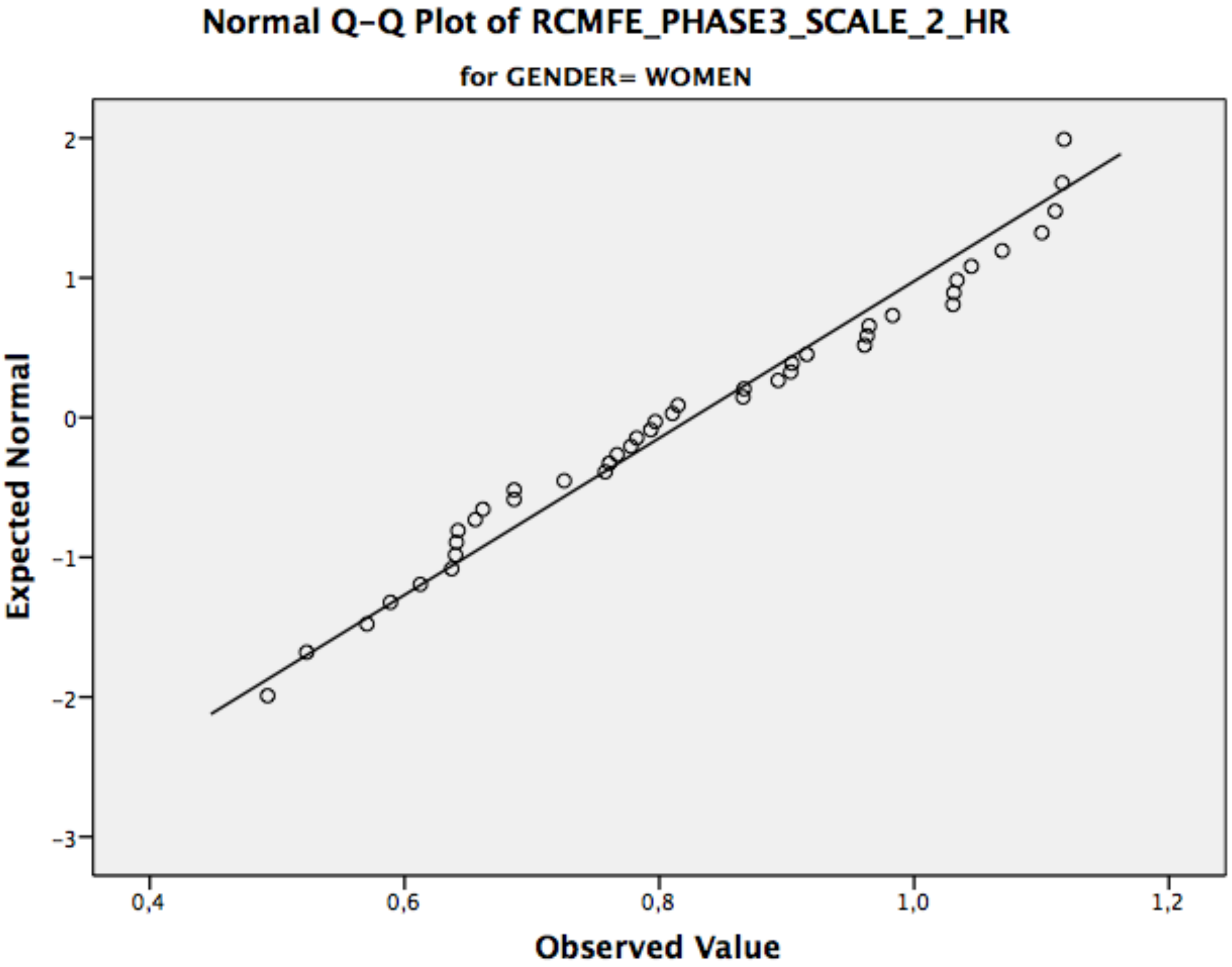

Normal Q-Q Plot of RCMFE_PHASE3_SCALE_2_HR

for GENDER= MEN

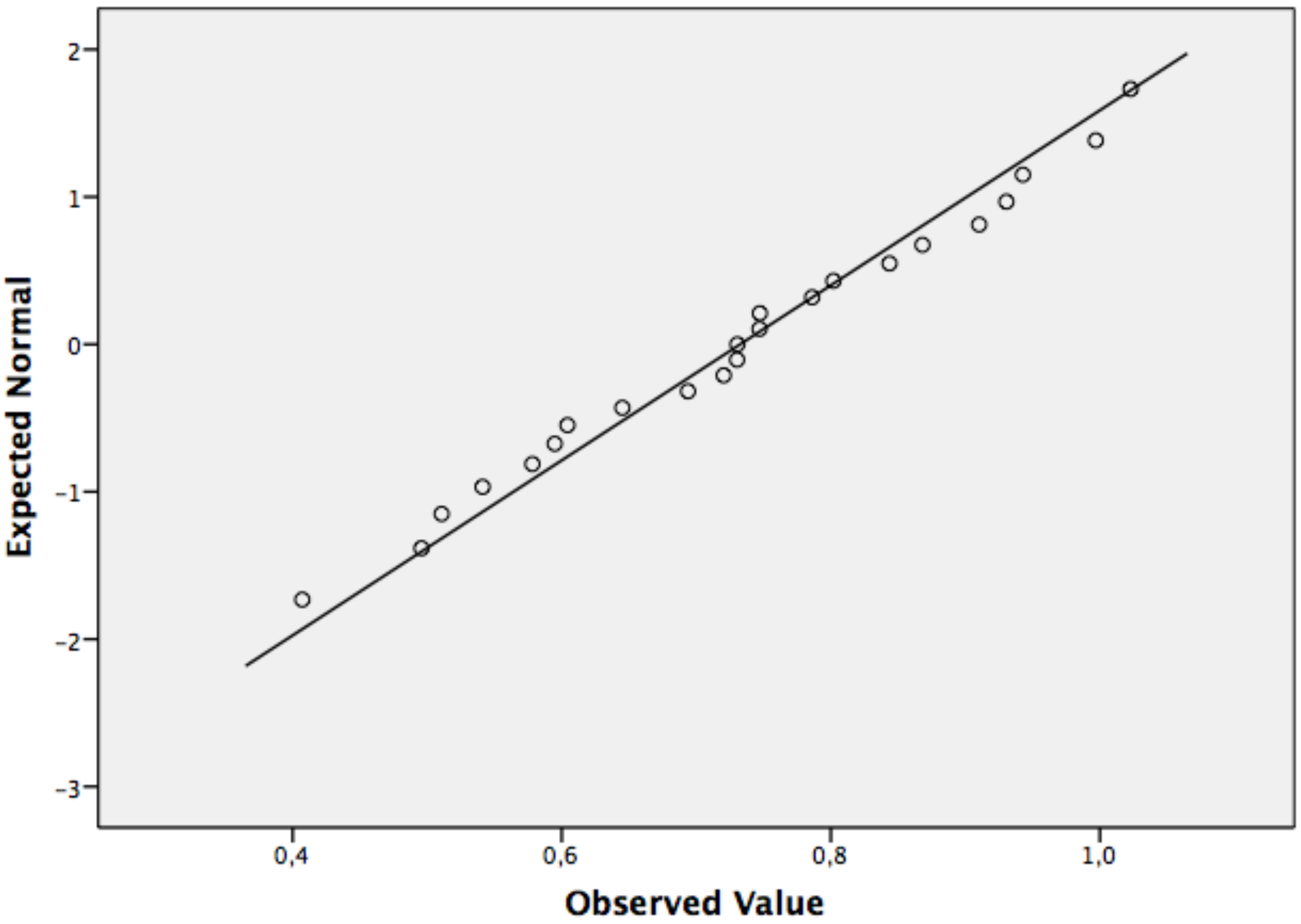

RCMFE_PHASE3_SCALE_3_HR

Normal Q-Q Plots

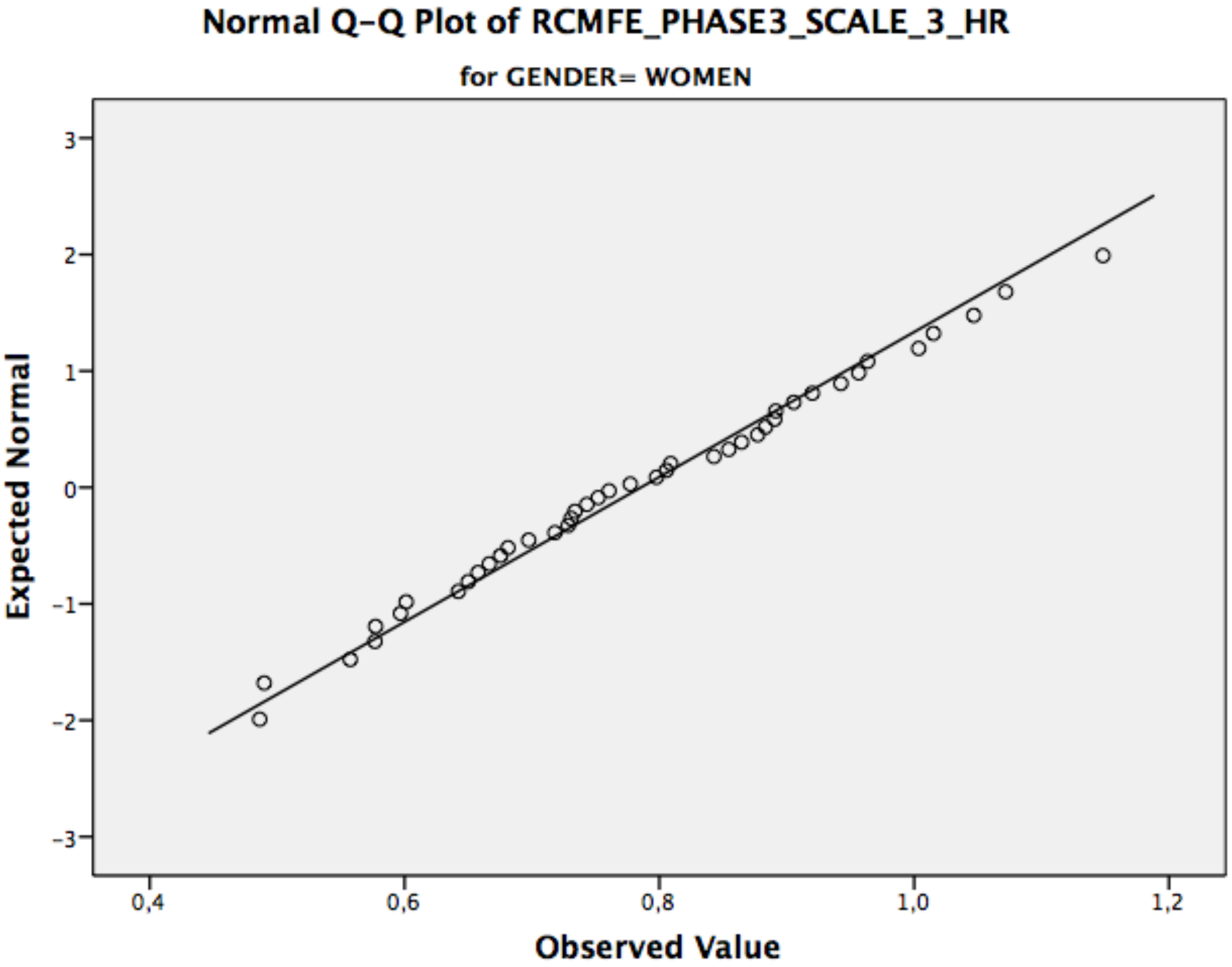

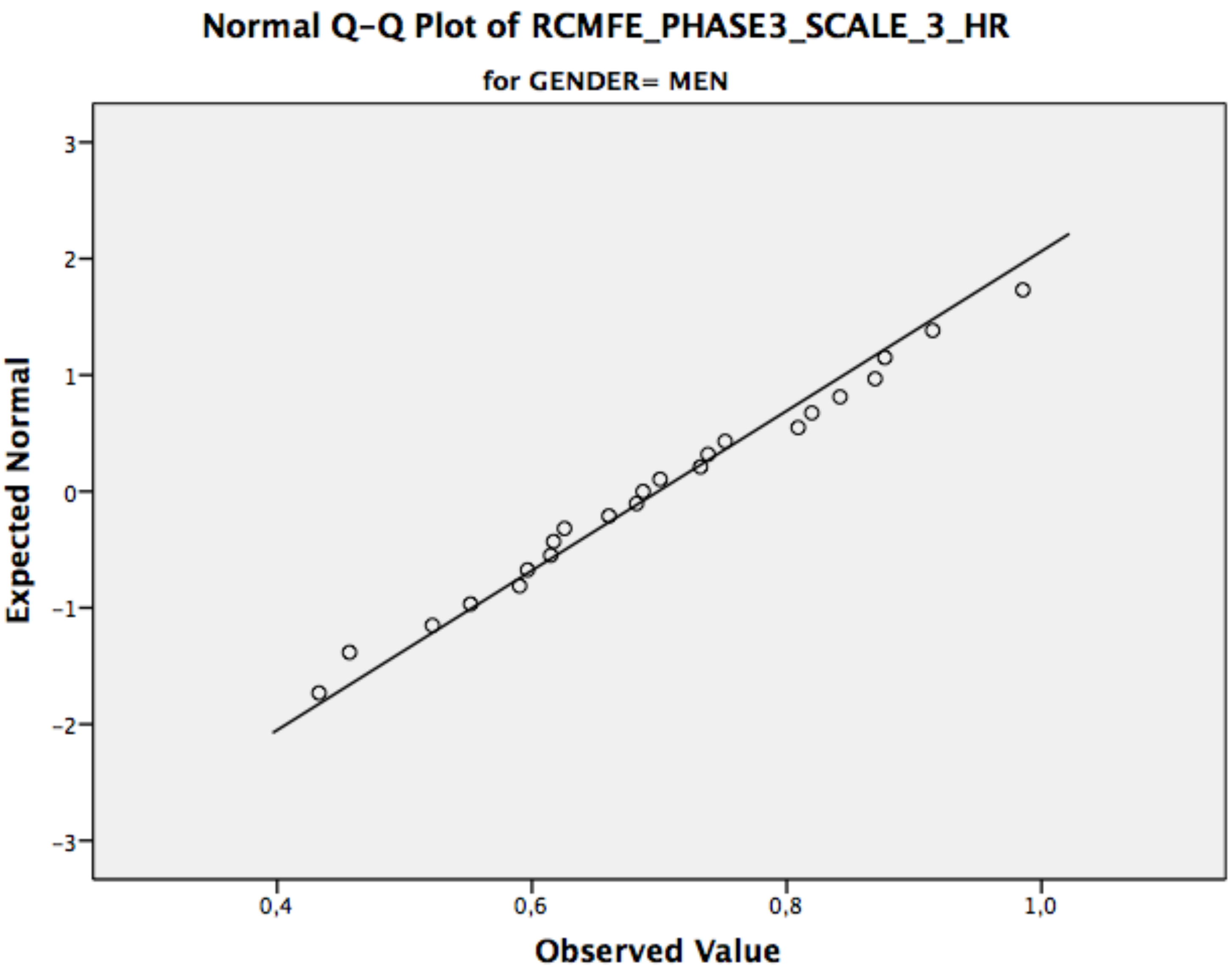

Normal Q-Q Plot of RCMFE_PHASE3_SCALE_3_HR
for GENDER= MEN
Expected Normal
Observed Value
3
2
1
0
-1
-2
-3
0,4
0,6
0,8
1,0

RCMFE_PHASE3_SCALE_4_HR

Normal Q-Q Plots

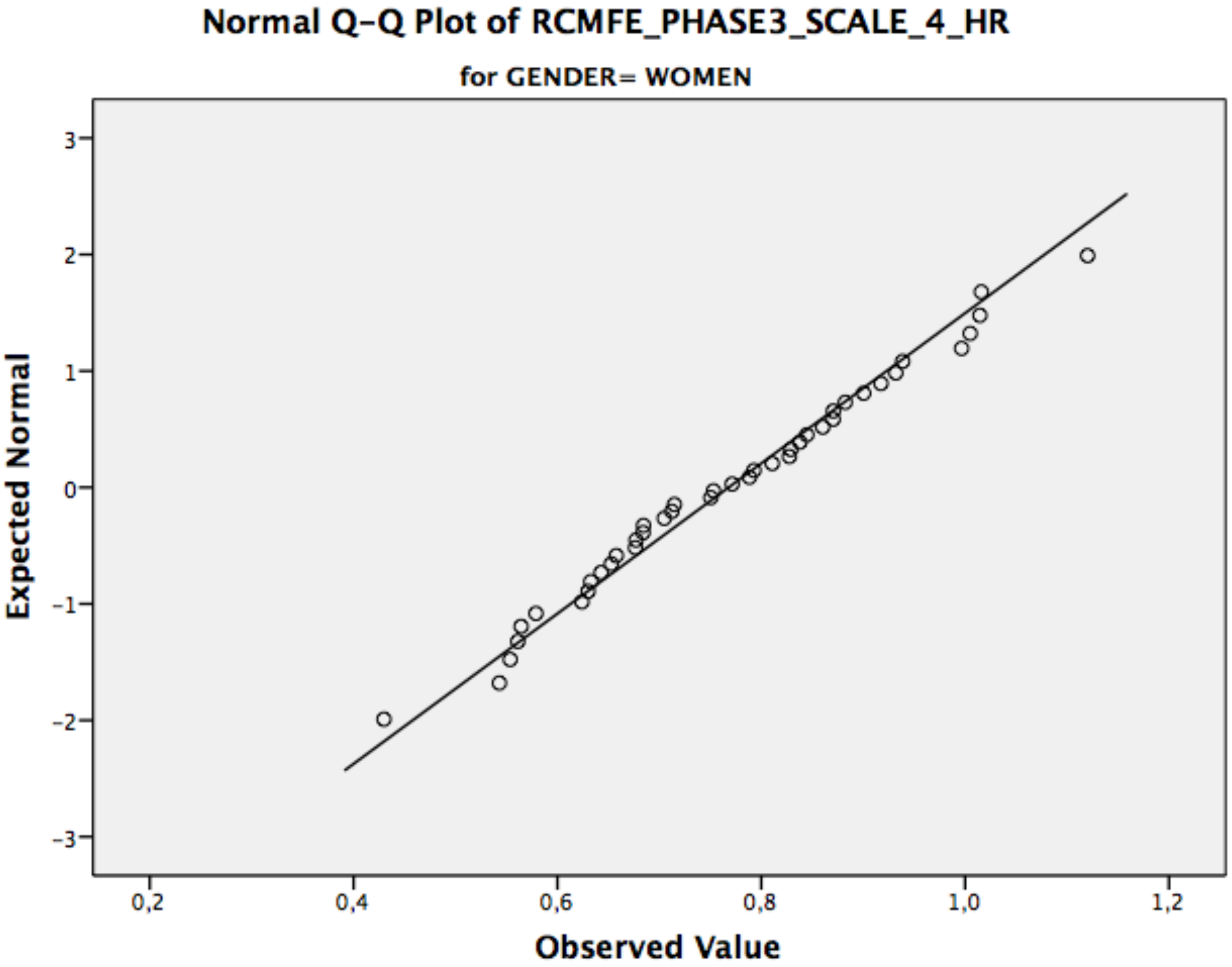

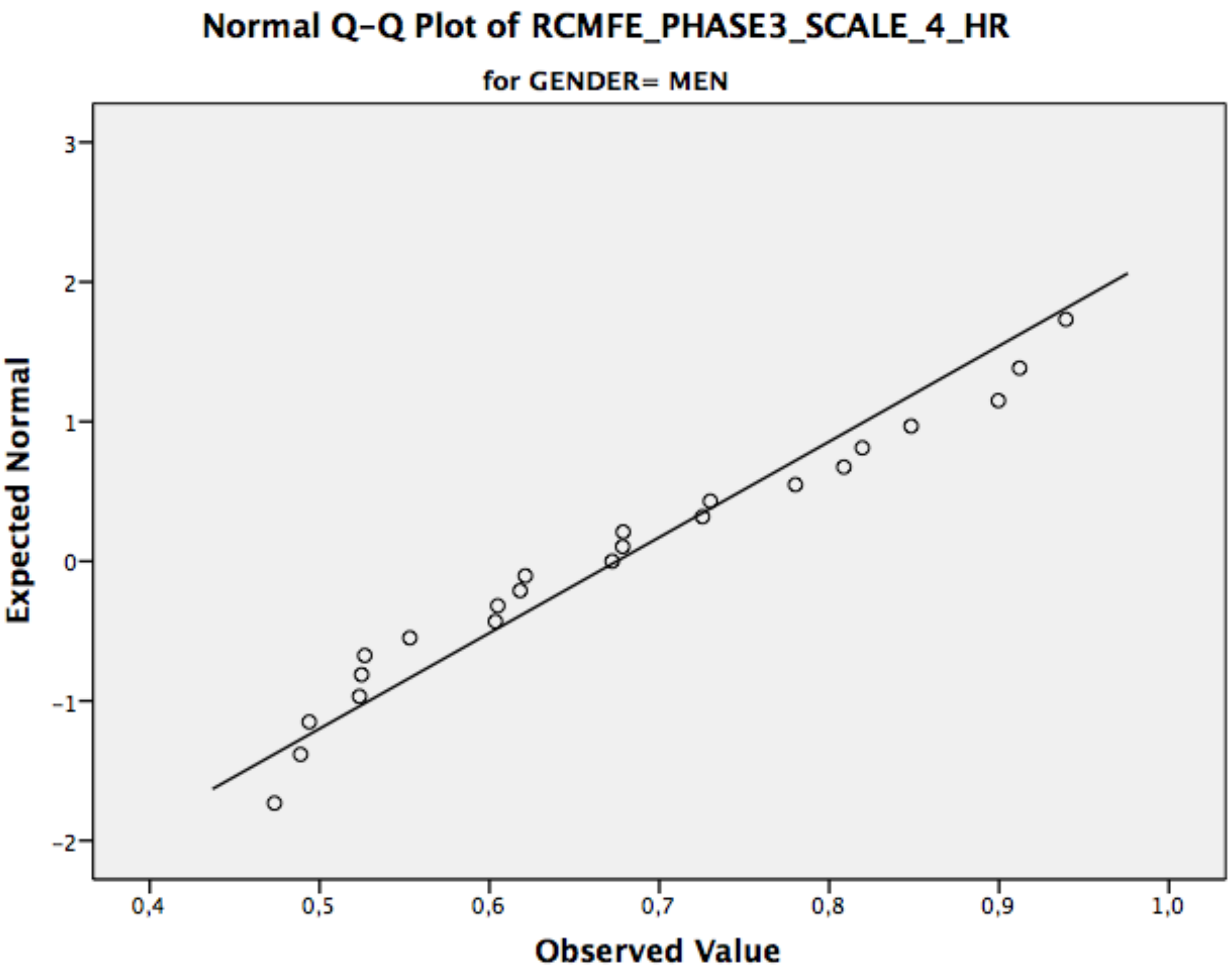

Normal Q-Q Plot of RCMFE_PHASE3_SCALE_4_HR
for GENDER= MEN
Expected Normal
Observed Value
3
2
1
0
-1
-2
0,4
0,5
0,6
0,7
0,8
0,9
1,0

RCMFE_PHASE3_SCALE_5_HR

Normal Q-Q Plots

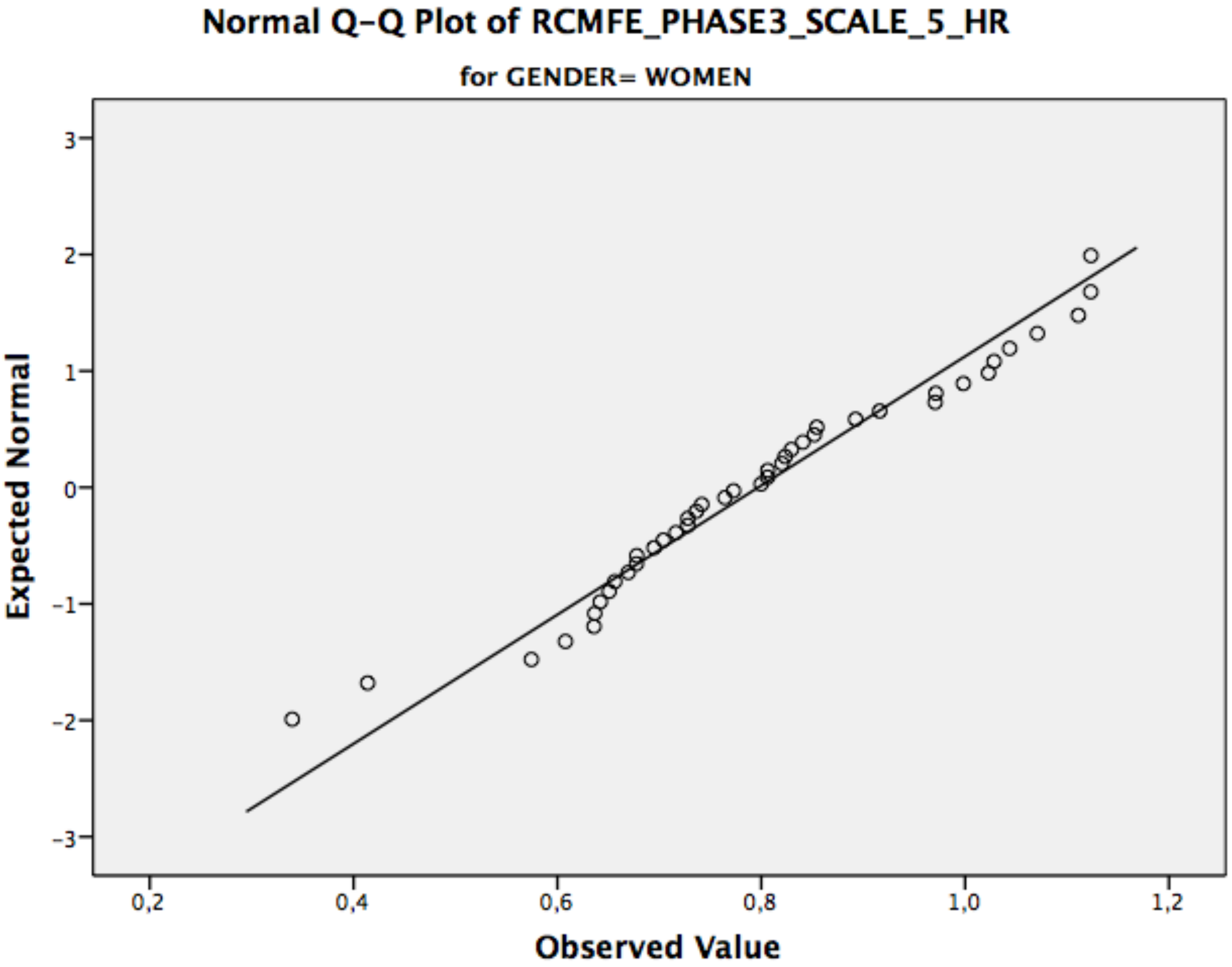

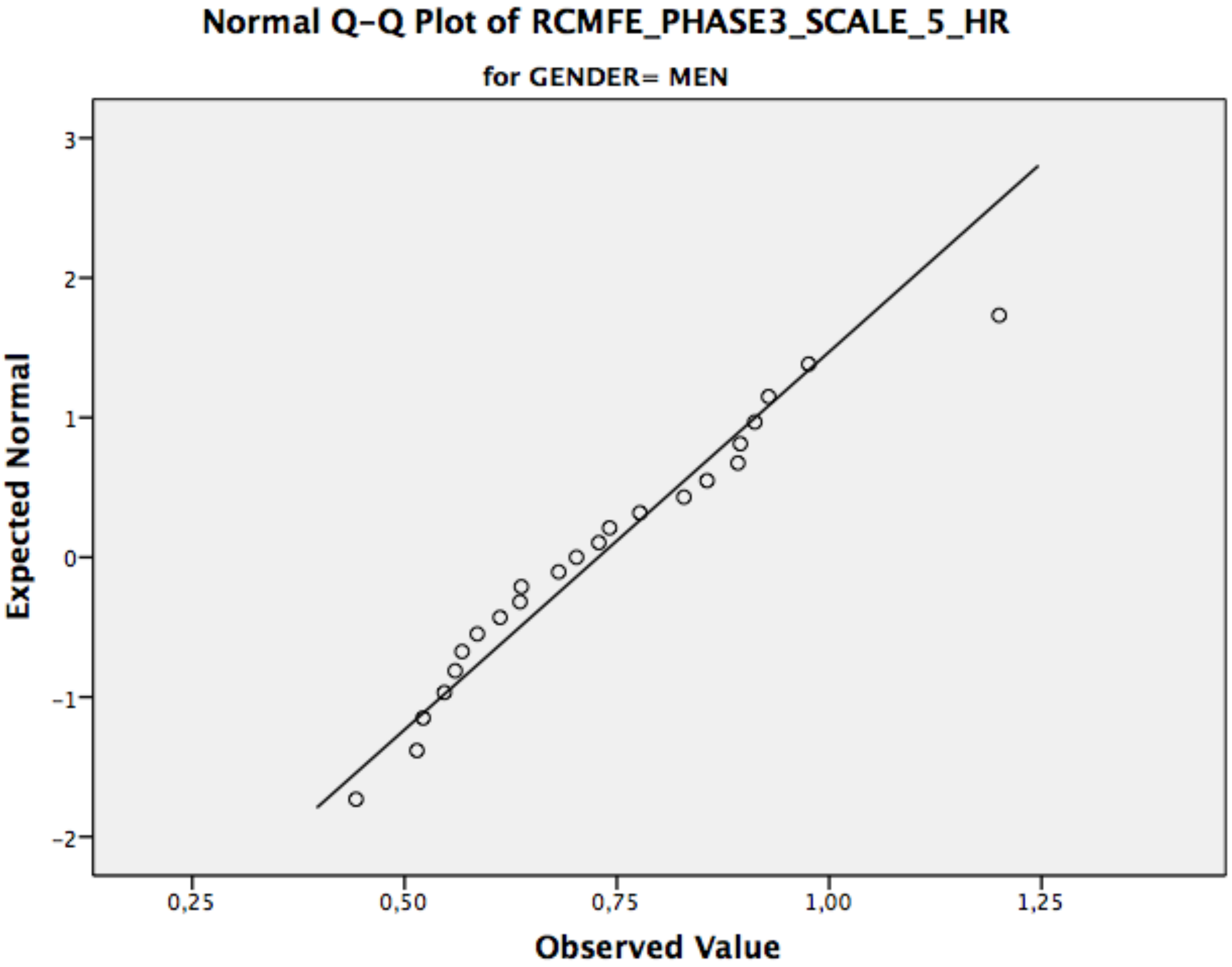
Normal Q-Q Plot of RCMFE_PHASE3_SCALE_5_HR
for GENDER= MEN
Expected Normal
Observed Value
3
2
1
0
-1
-2
0,25
0,50
0,75
1,00
1,25

RCMFE_PHASE4_SCALE_1_HR

Normal Q-Q Plots

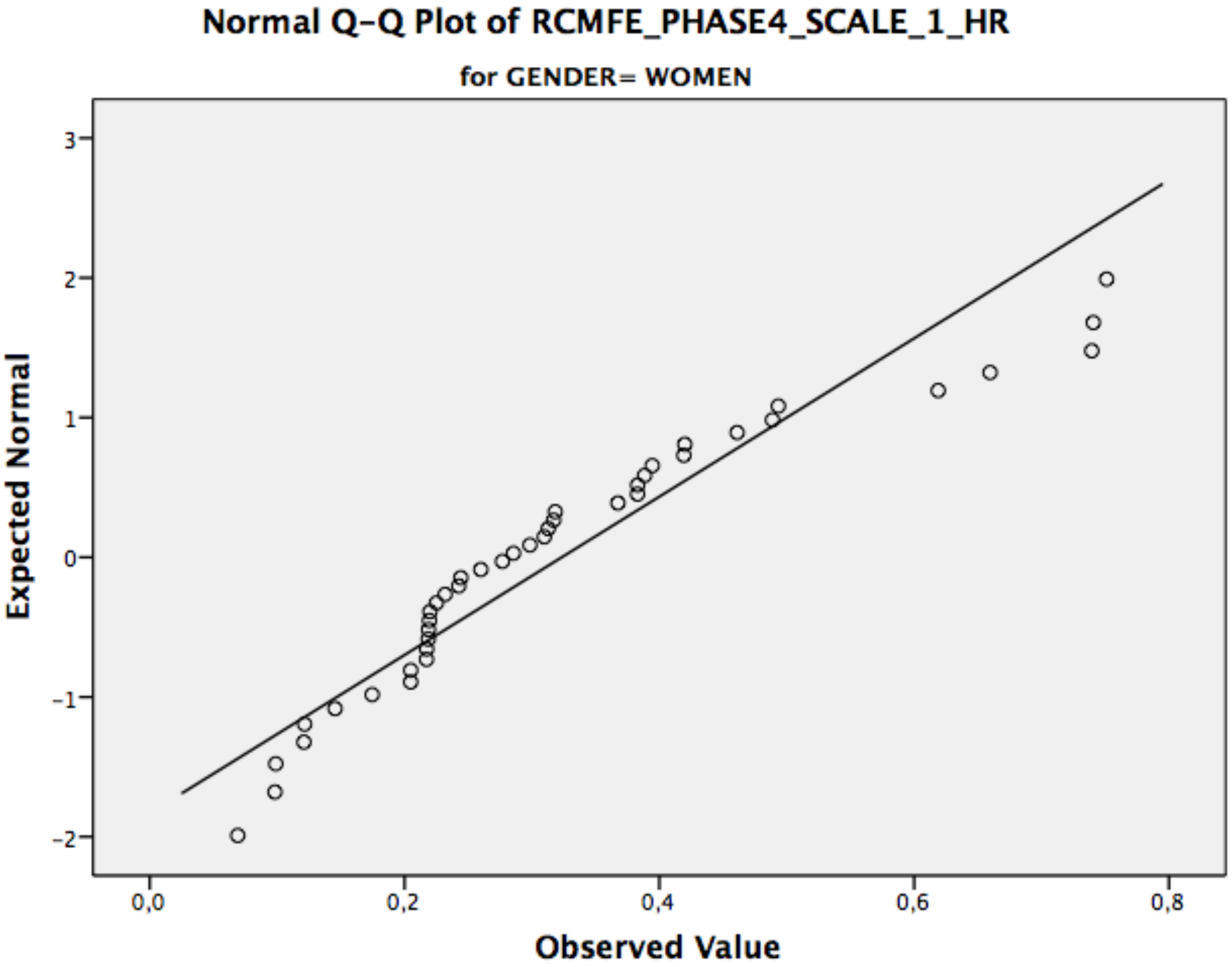

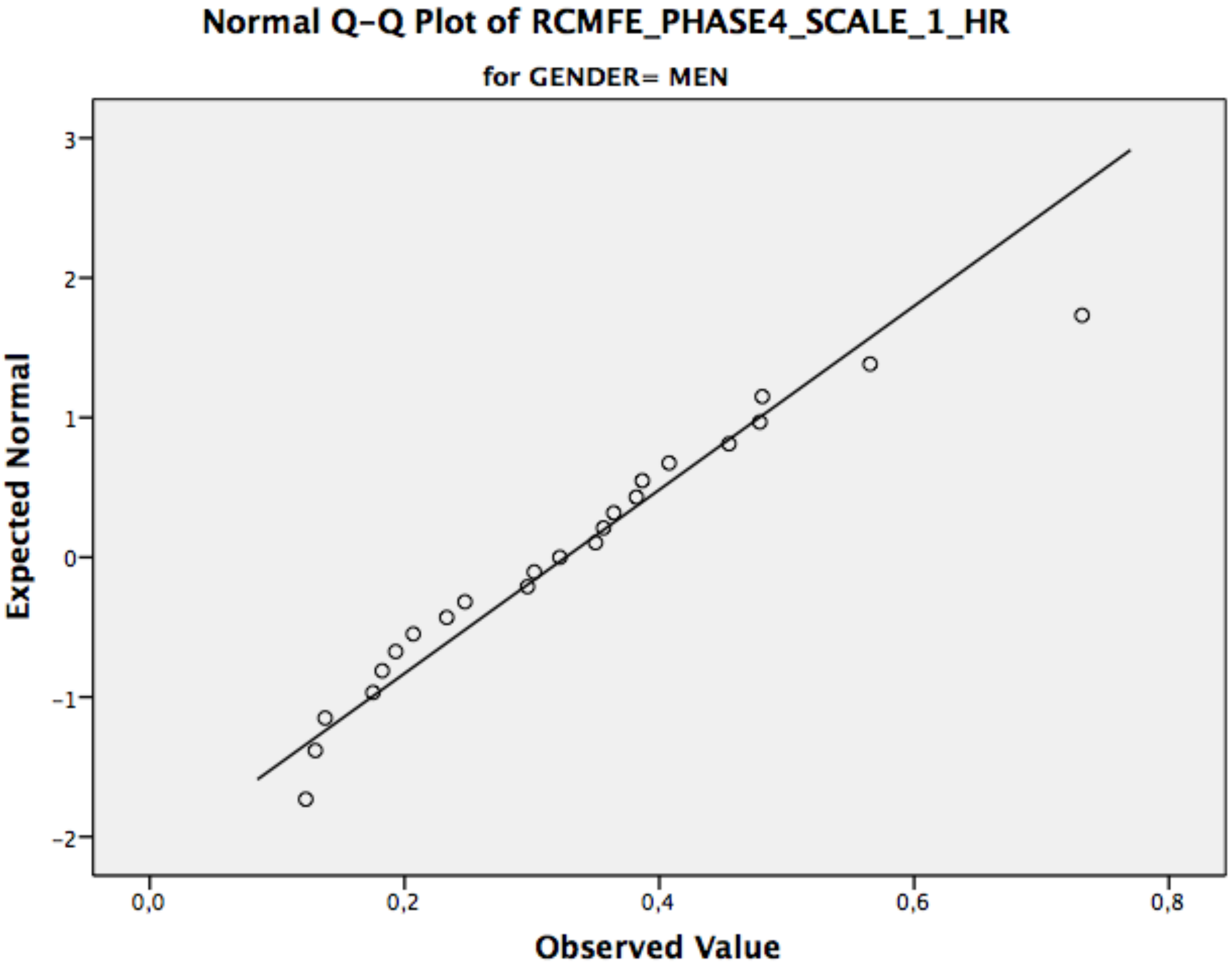
Normal Q-Q Plot of RCMFE_PHASE4_SCALE_1_HR
for GENDER= MEN
Expected Normal
Observed Value
3
2
1
0
-1
-2
0,0
0,2
0,4
0,6
0,8

RCMFE_PHASE4_SCALE_2_HR

Normal Q-Q Plots

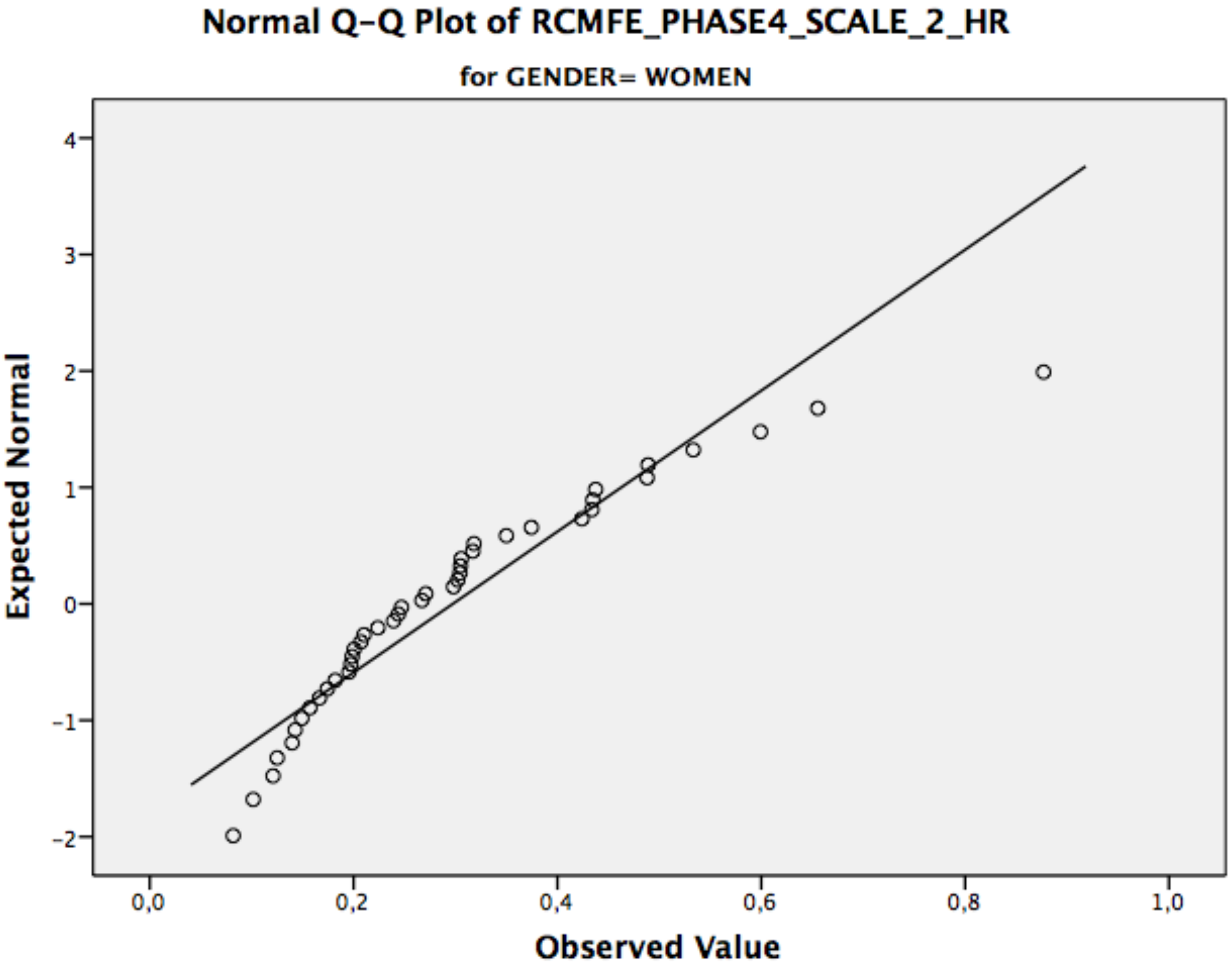

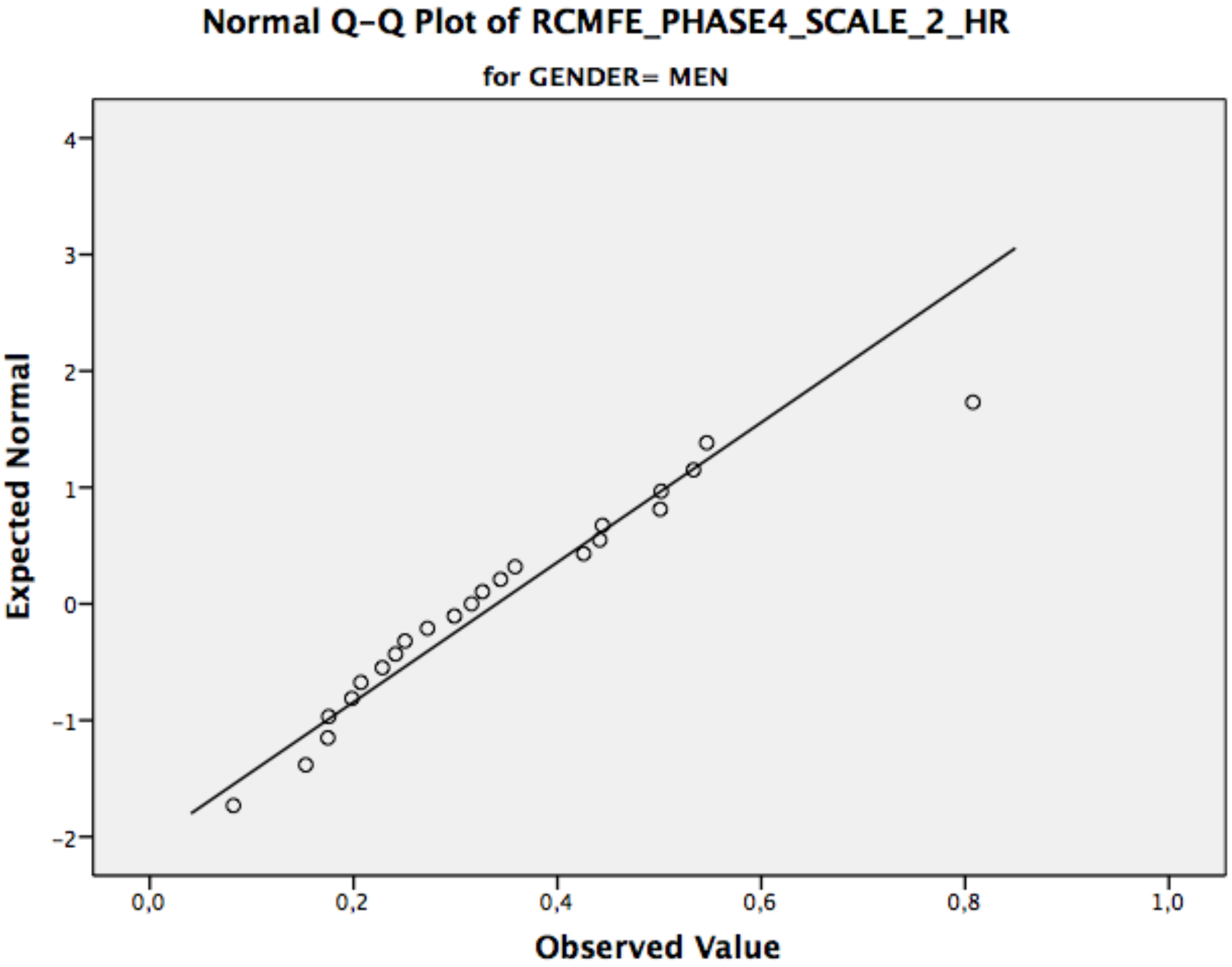

Normal Q-Q Plot of RCMFE_PHASE4_SCALE_2_HR
for GENDER= MEN
Expected Normal
Observed Value
4
3
2
1
0
-1
-2
0,0
0,2
0,4
0,6
0,8
1,0

RCMFE_PHASE4_SCALE_3_HR

Normal Q-Q Plots

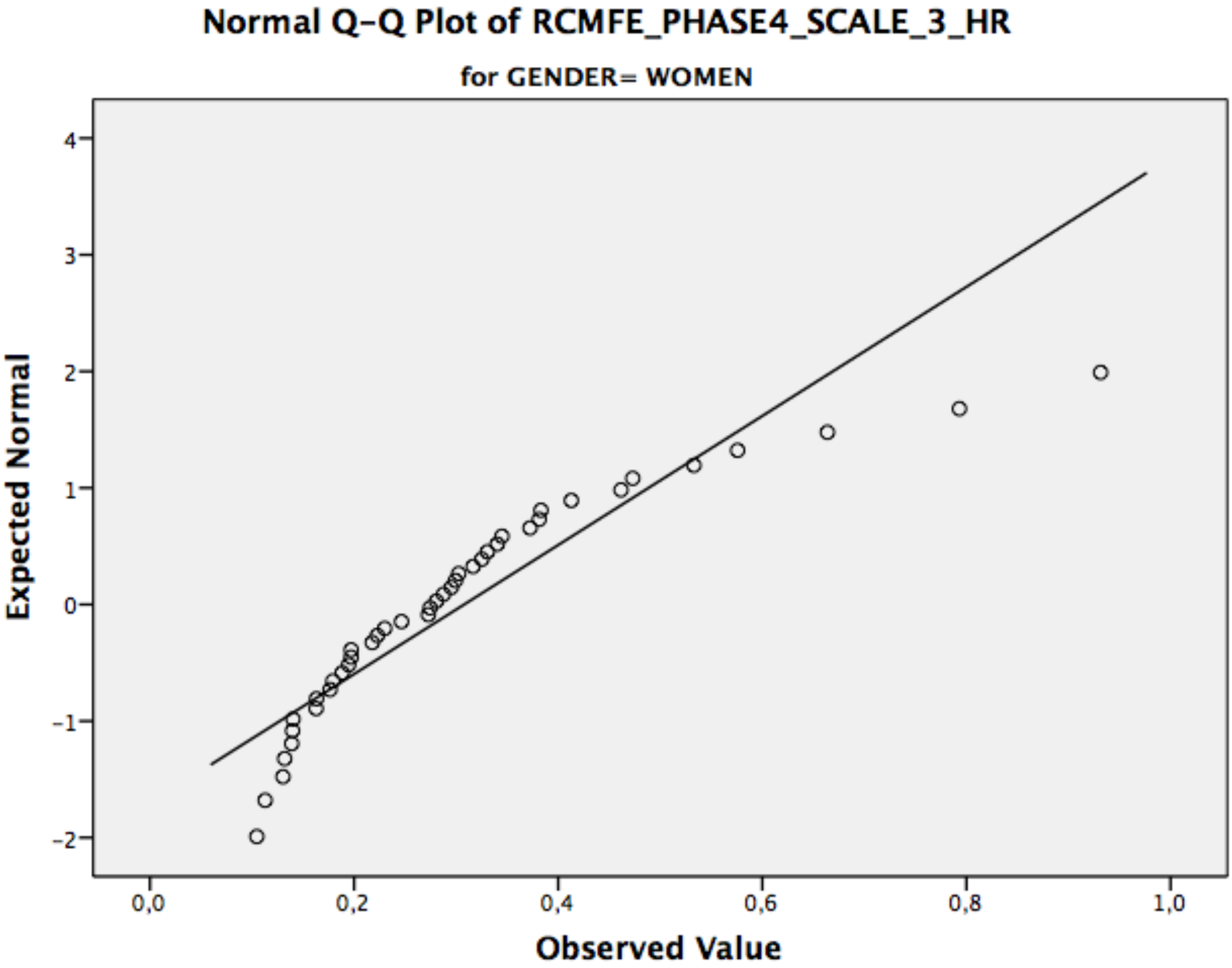

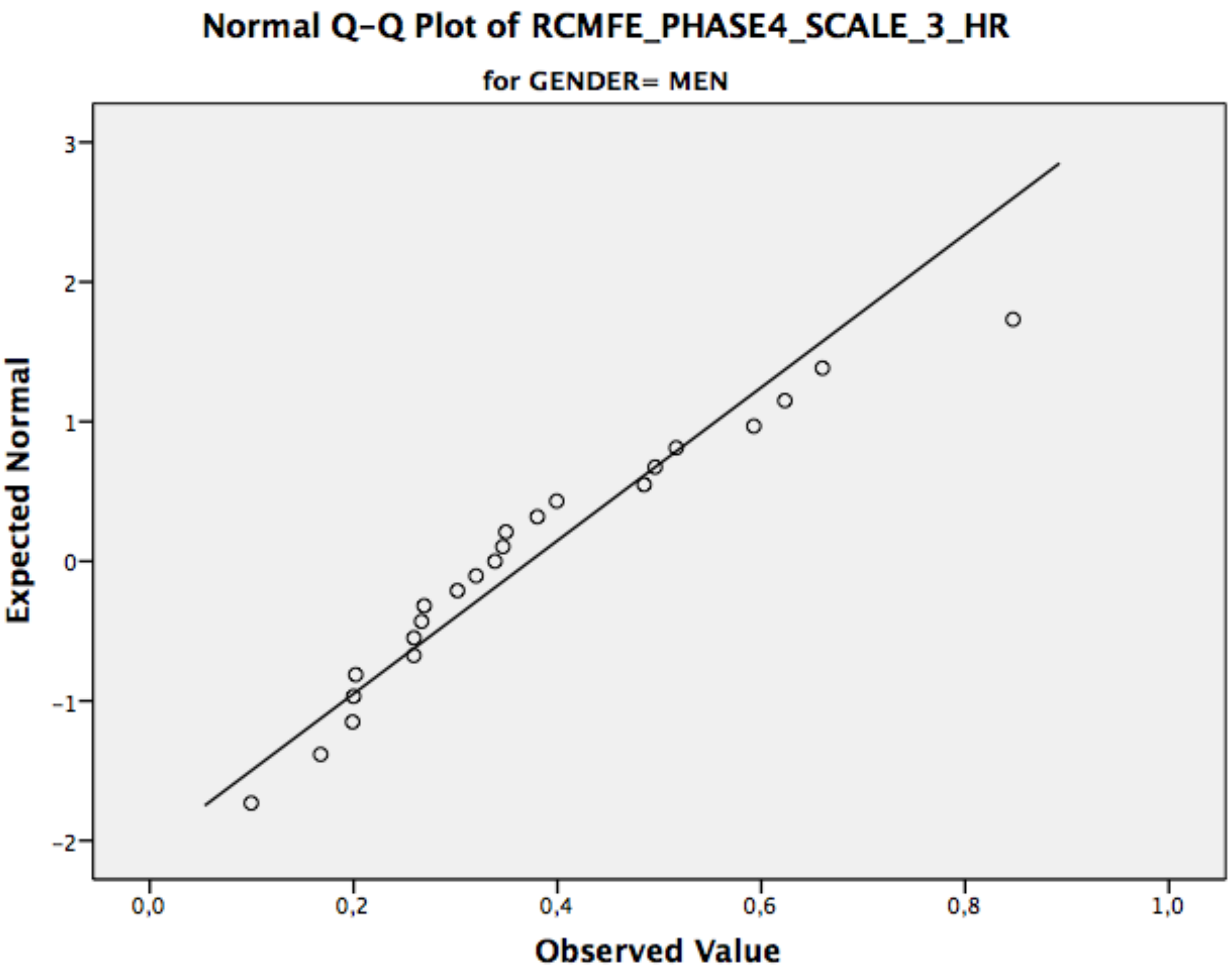
Normal Q-Q Plot of RCMFE_PHASE4_SCALE_3_HR
for GENDER= MEN
Expected Normal
3
2
1
0
-1
-2
0,0
0,2
0,4
0,6
0,8
1,0
Observed Value

RCMFE_PHASE4_SCALE_4_HR

Normal Q-Q Plots

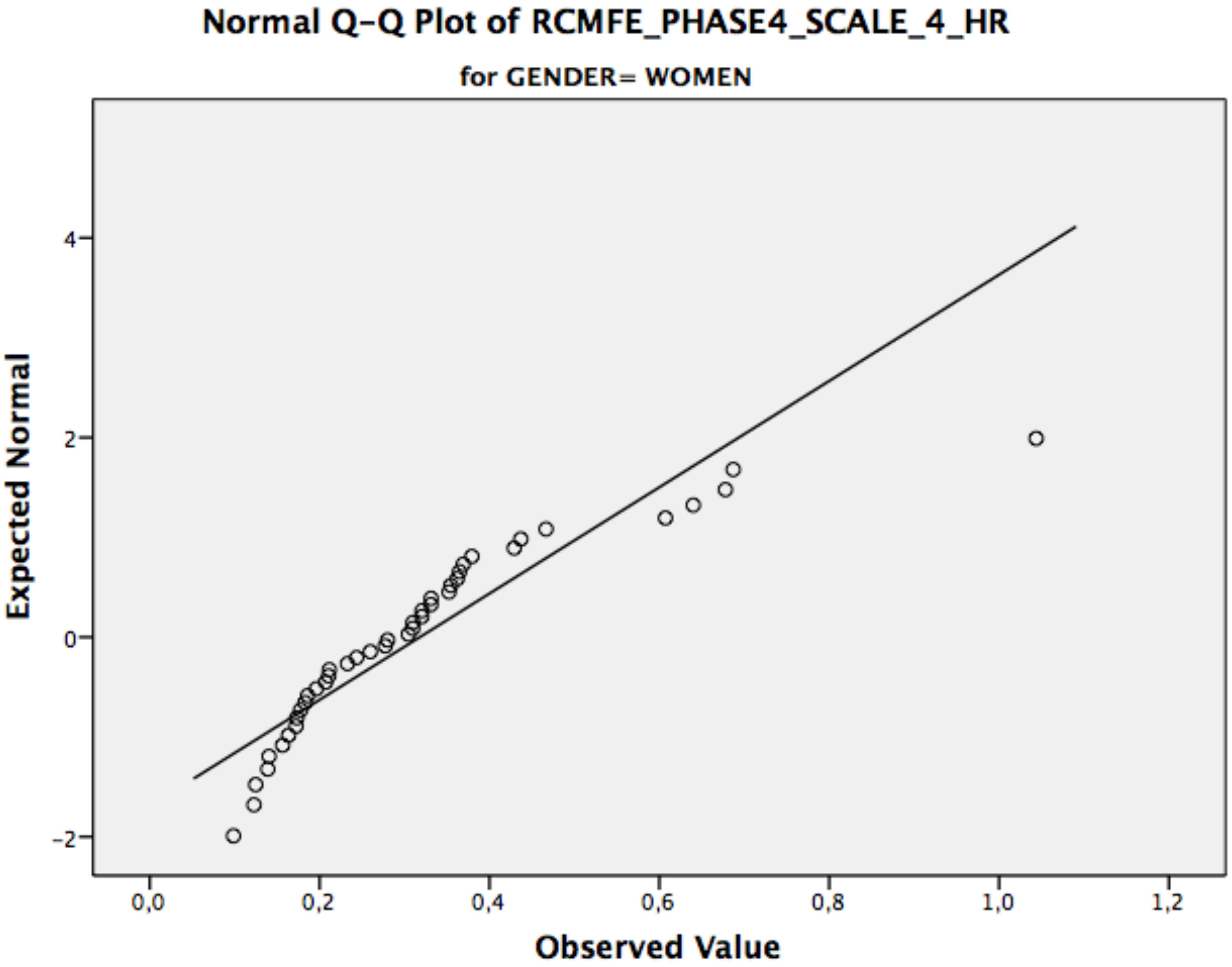

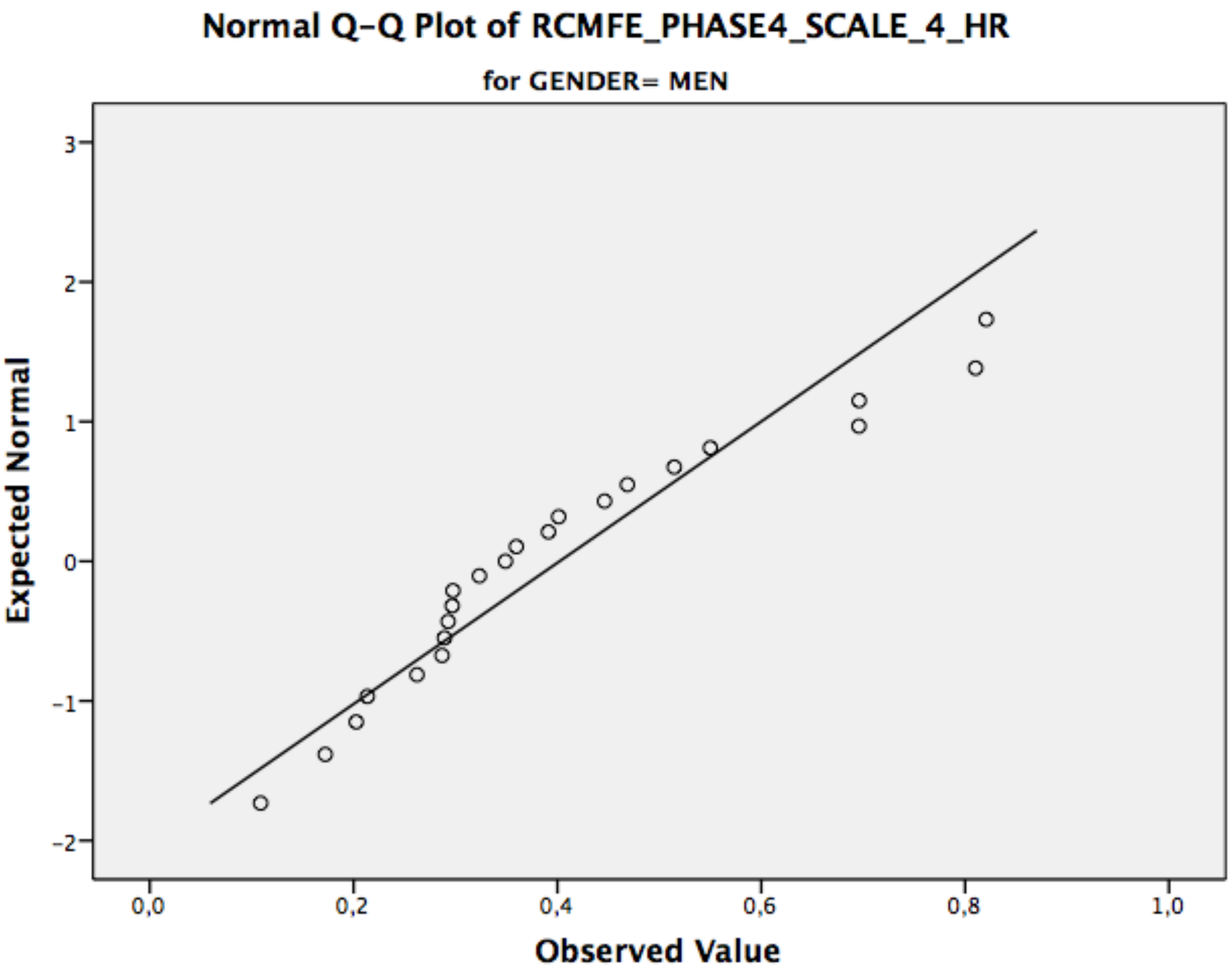

Normal Q-Q Plot of RCMFE_PHASE4_SCALE_4_HR
for GENDER= MEN
Expected Normal
Observed Value
3
2
1
0
-1
-2
0,0
0,2
0,4
0,6
0,8
1,0

RCMFE_PHASE4_SCALE_5_HR

Normal Q-Q Plots

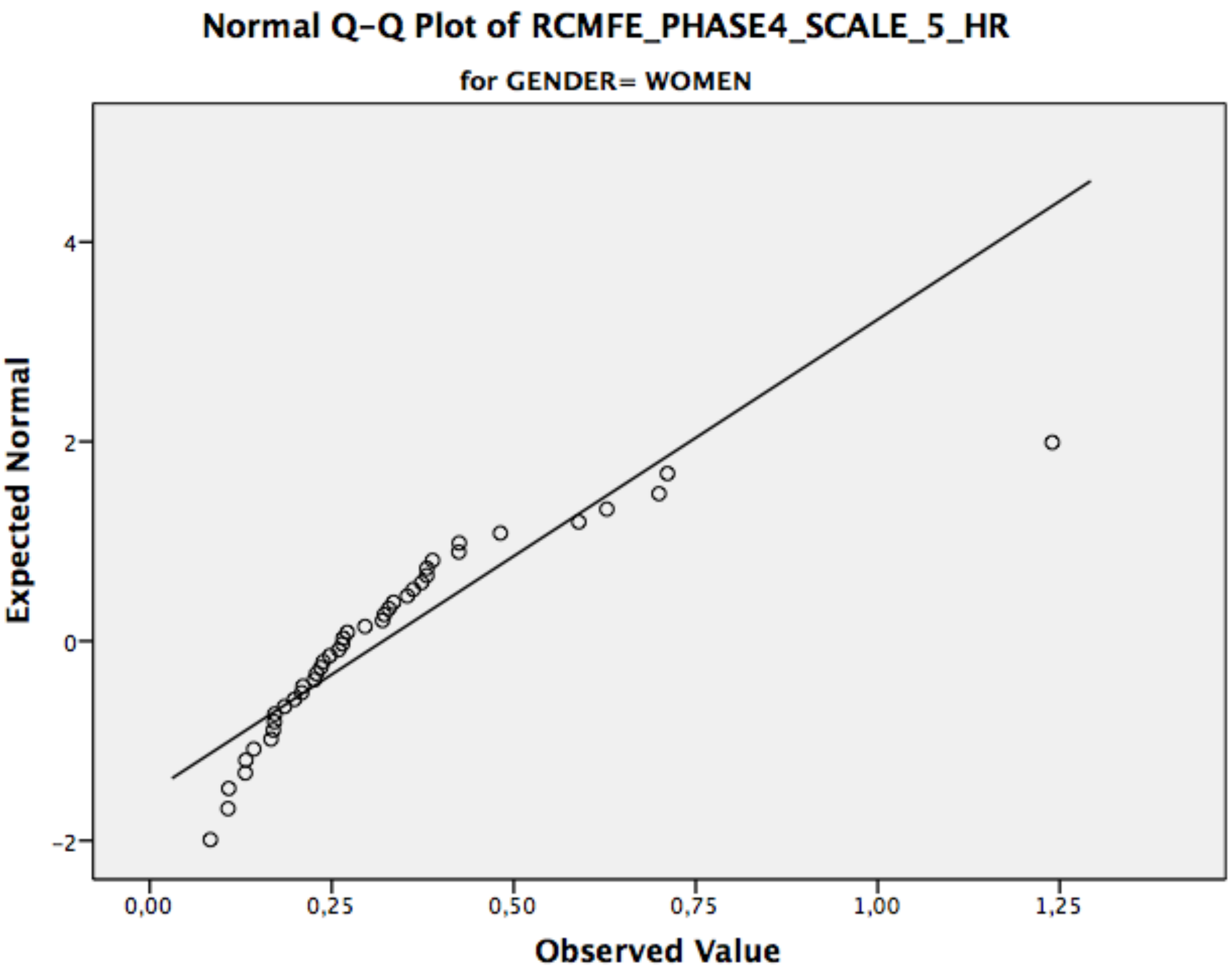

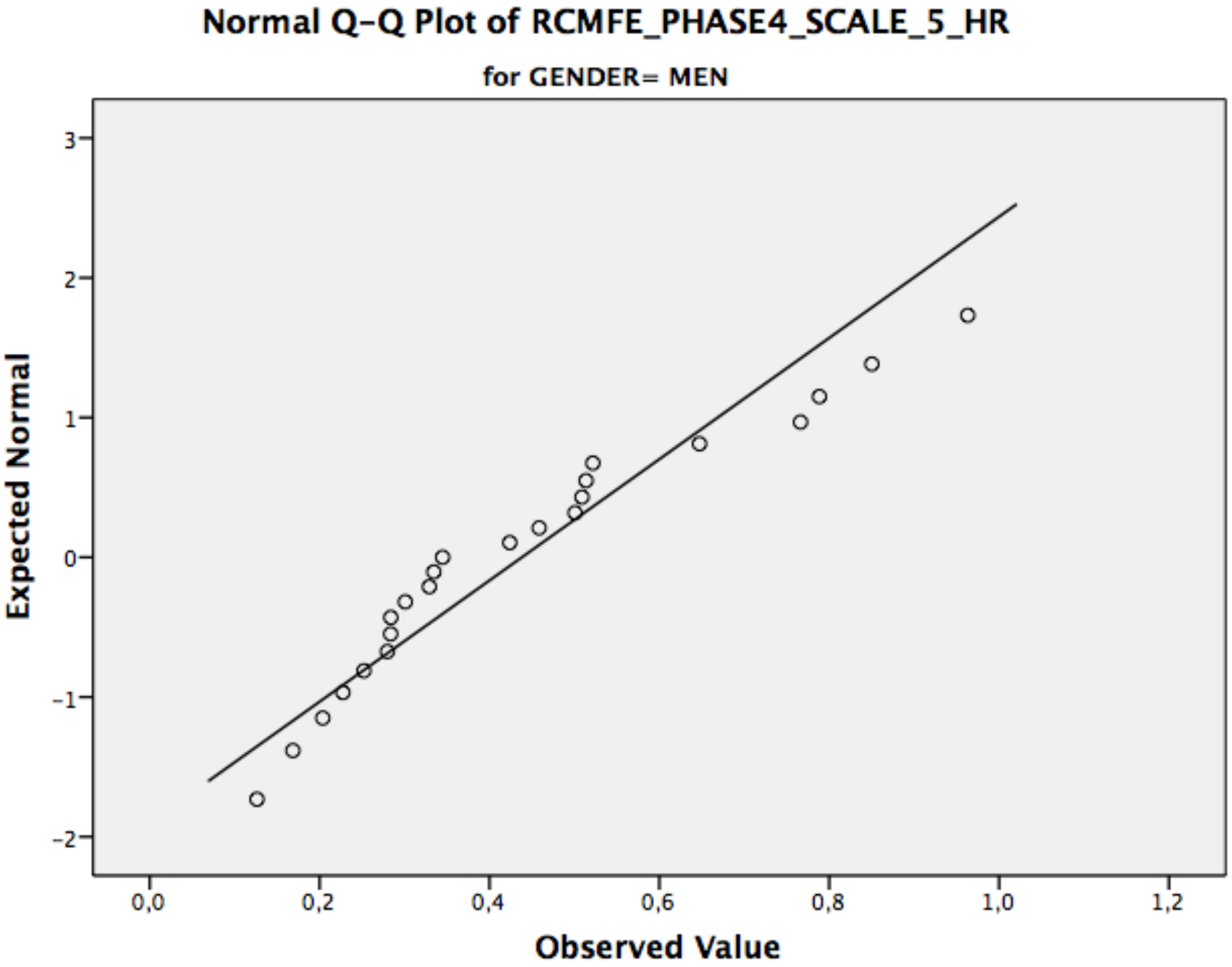
Normal Q-Q Plot of RCMFE_PHASE4_SCALE_5_HR
for GENDER= MEN
Expected Normal
Observed Value
3
2
1
0
-1
-2
0,0
0,2
0,4
0,6
0,8
1,0
1,2

RCMFE_PHASE5_SCALE_1_HR

Normal Q-Q Plots

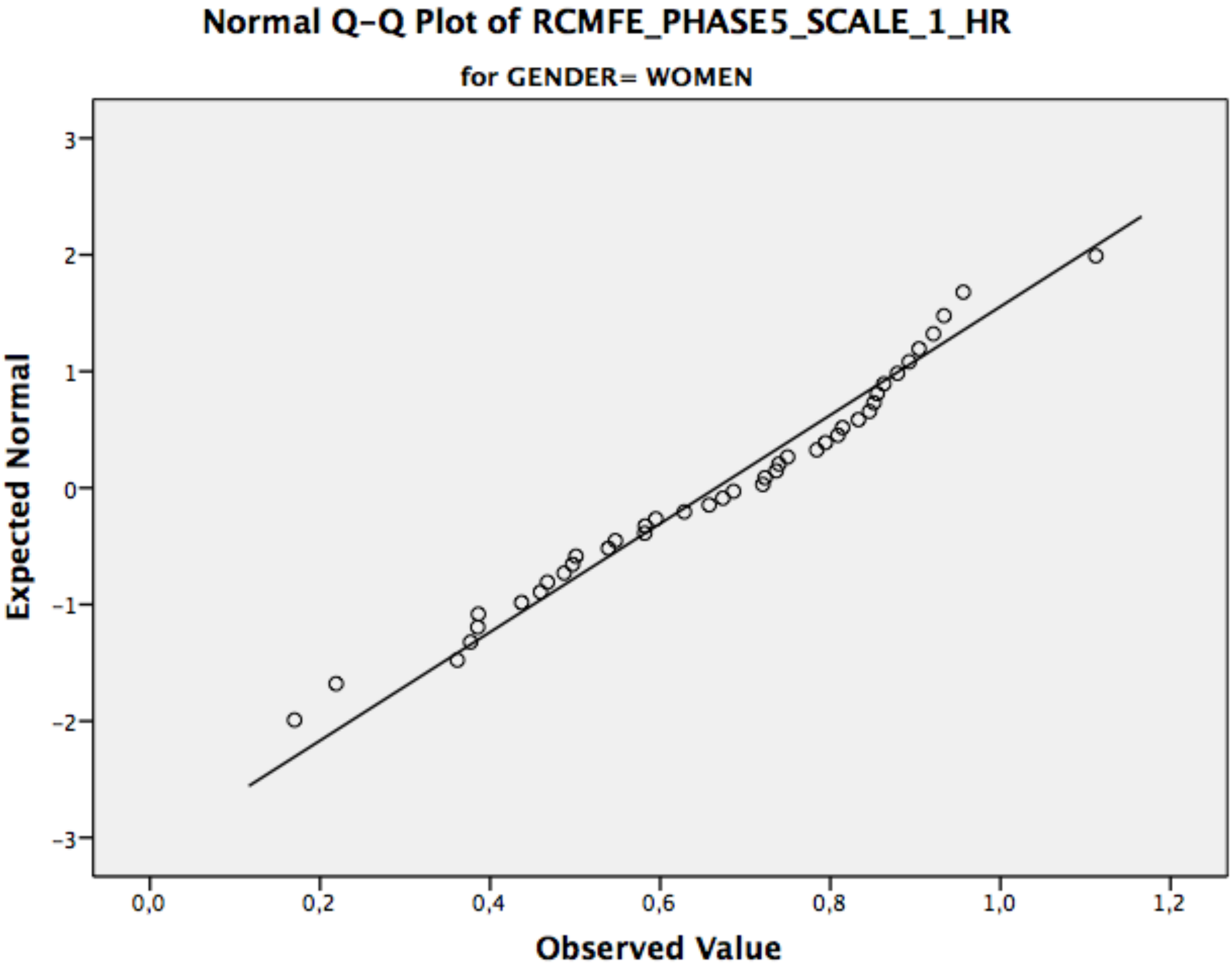

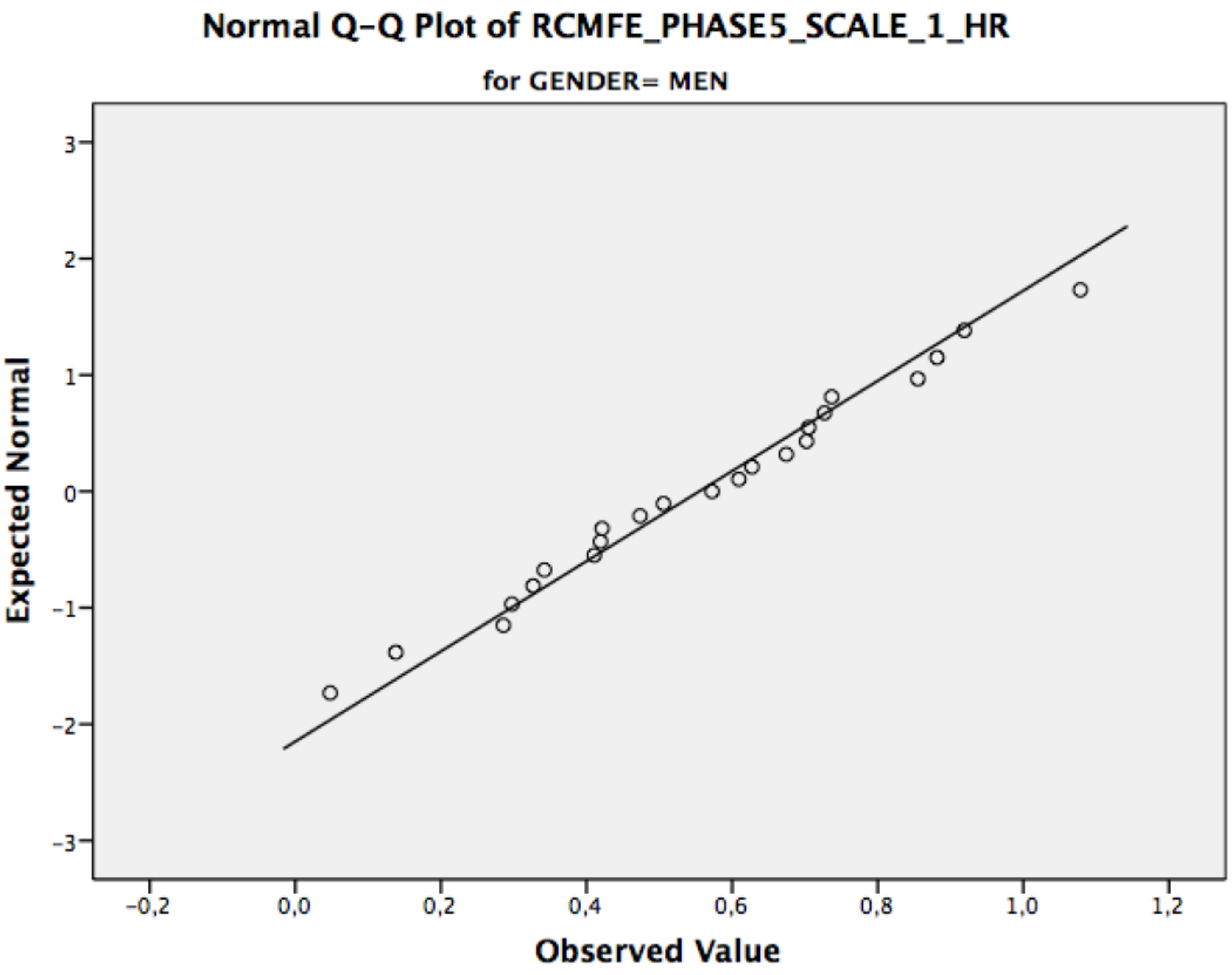
Normal Q-Q Plot of RCMFE_PHASE5_SCALE_1_HR
for GENDER= MEN
Expected Normal
Observed Value
3
2
1
0
−1
−2
−3
−0,2
0,0
0,2
0,4
0,6
0,8
1,0
1,2

RCMFE_PHASE5_SCALE_2_HR

Normal Q-Q Plots

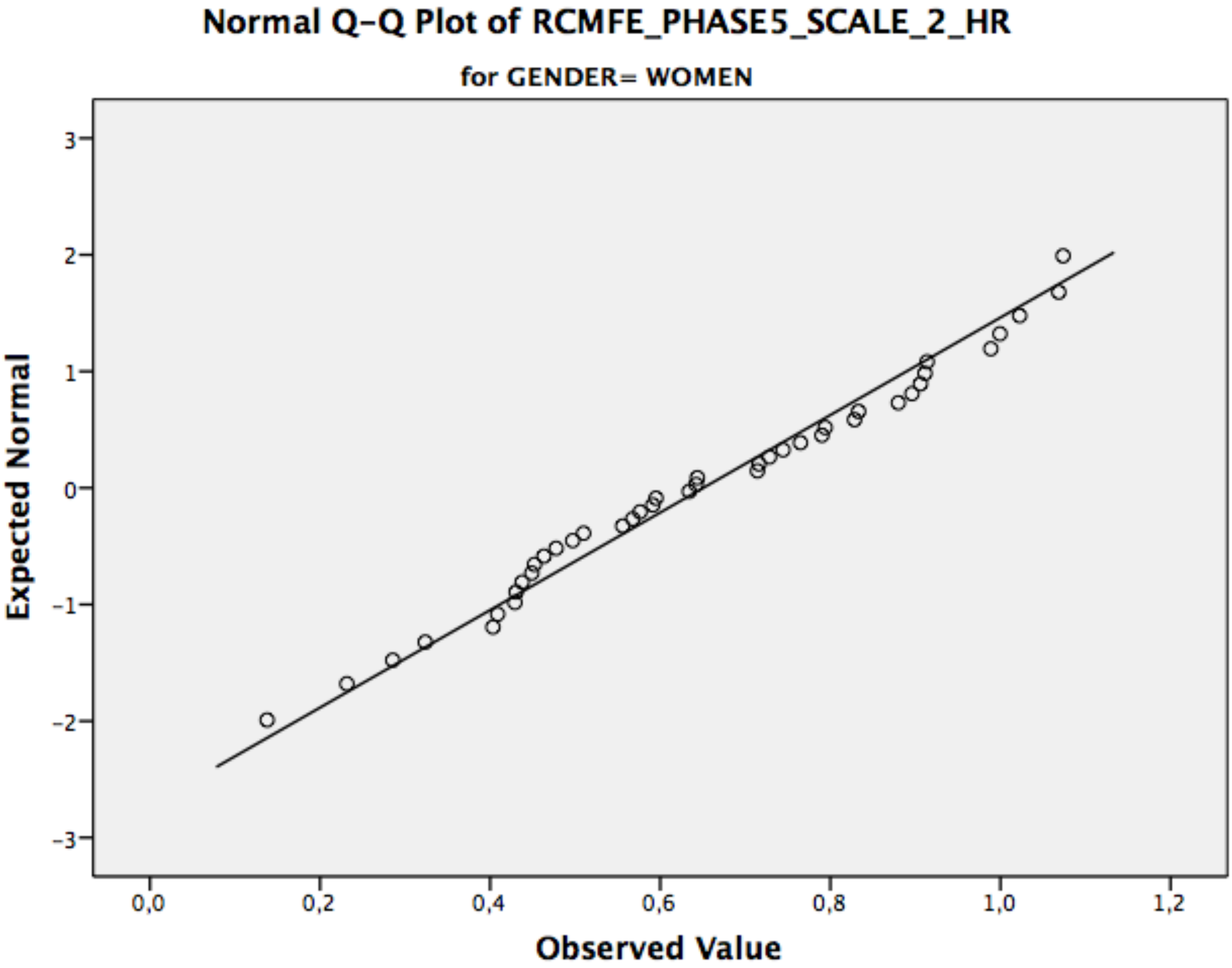

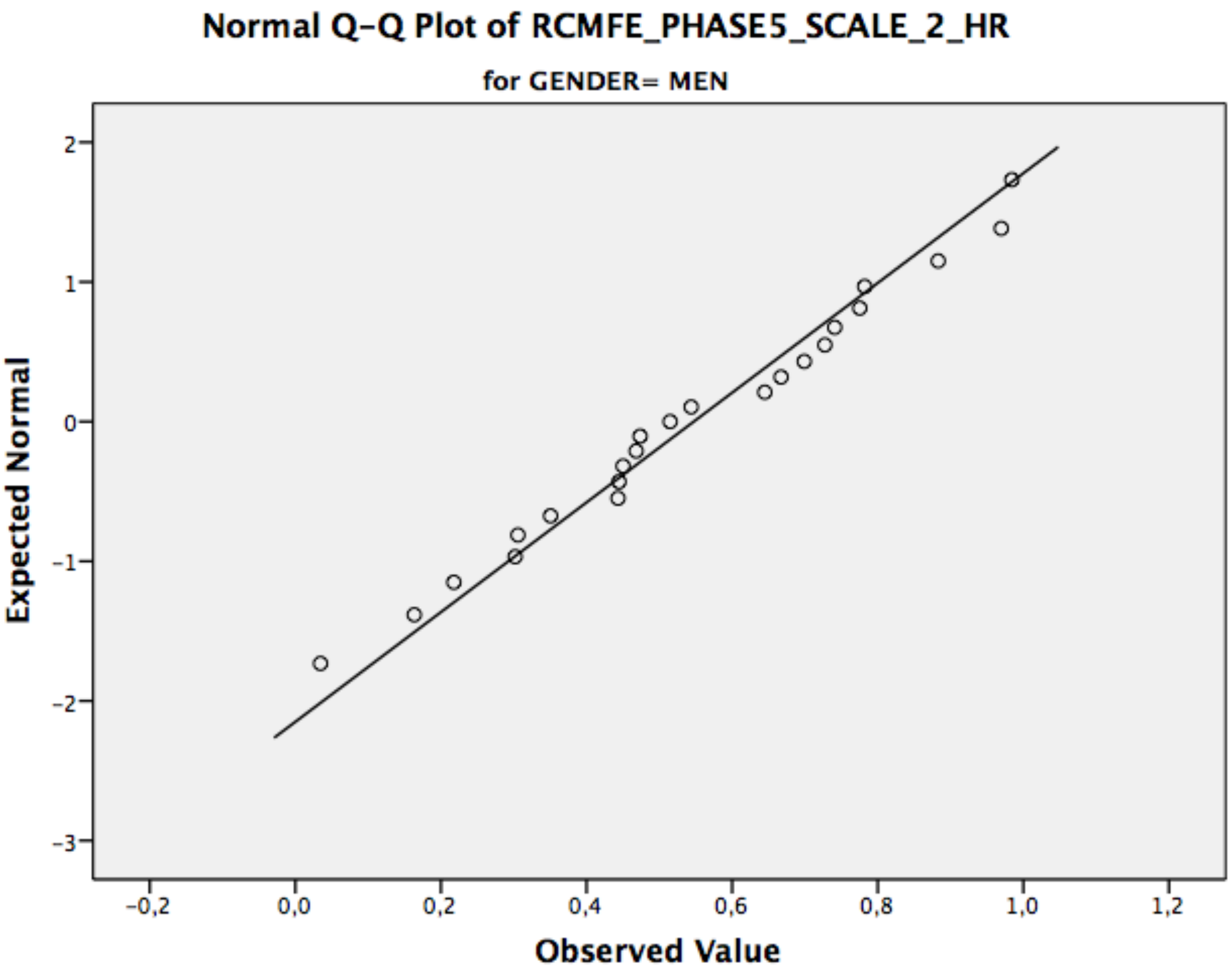
Normal Q-Q Plot of RCMFE_PHASE5_SCALE_2_HR
for GENDER= MEN
Expected Normal
Observed Value
2
1
0
-1
-2
-3
-0,2
0,0
0,2
0,4
0,6
0,8
1,0
1,2

RCMFE_PHASE5_SCALE_3_HR

Normal Q-Q Plots

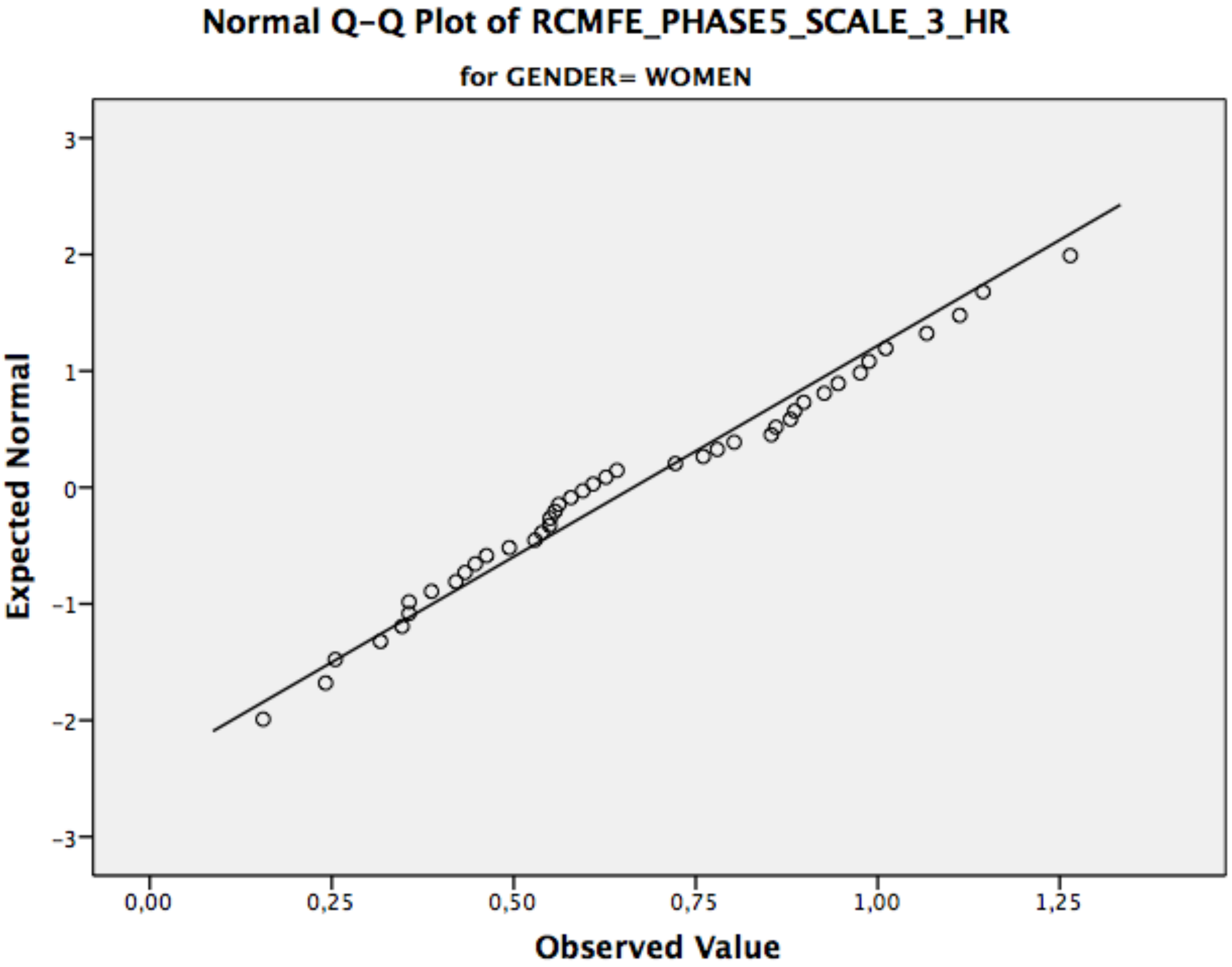

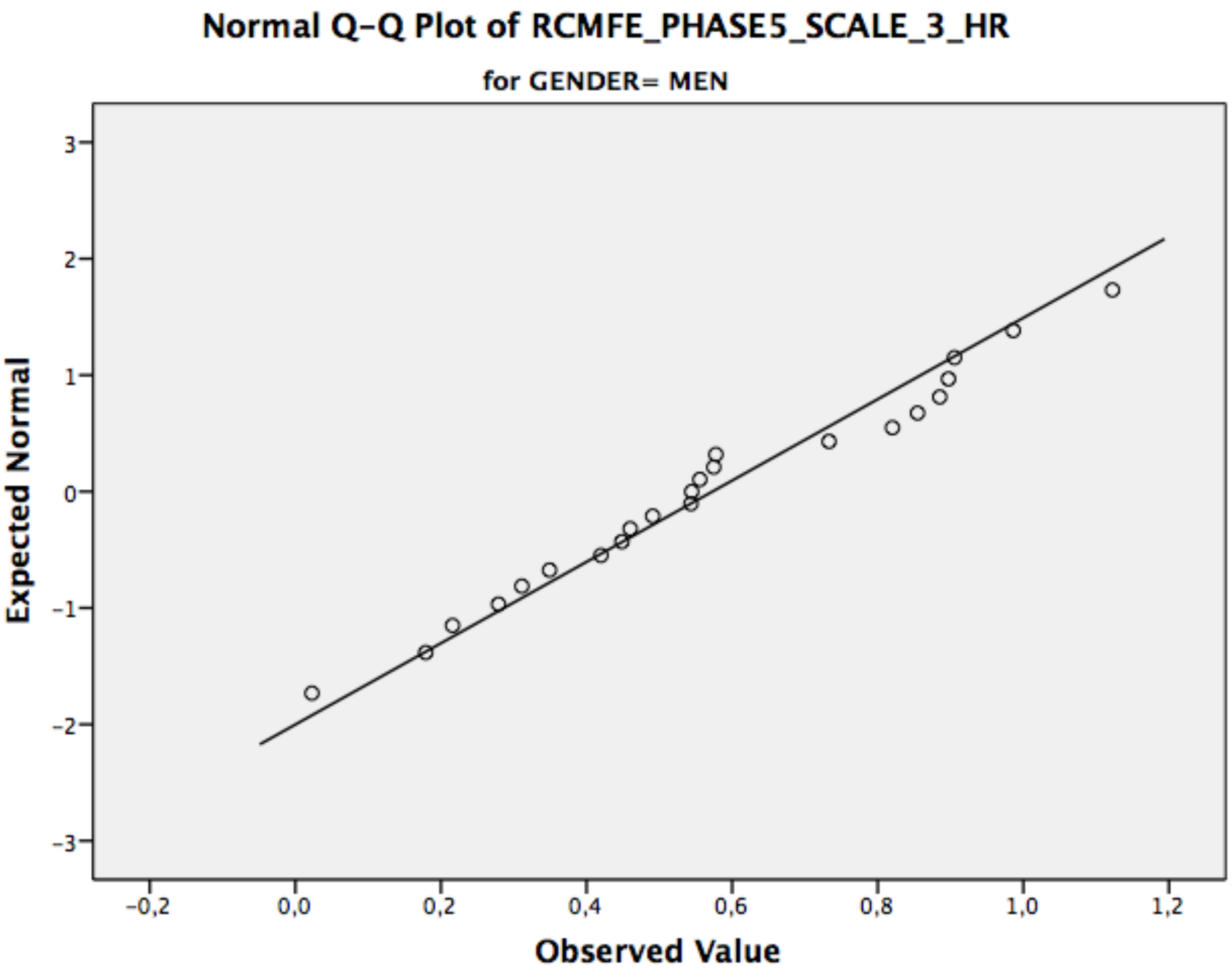

Normal Q-Q Plot of RCMFE_PHASE5_SCALE_3_HR
for GENDER= MEN
Expected Normal
3
2
1
0
-1
-2
-3
-0,2
0,0
0,2
0,4
0,6
0,8
1,0
1,2
Observed Value

RCMFE_PHASE5_SCALE_4_HR

Normal Q-Q Plots

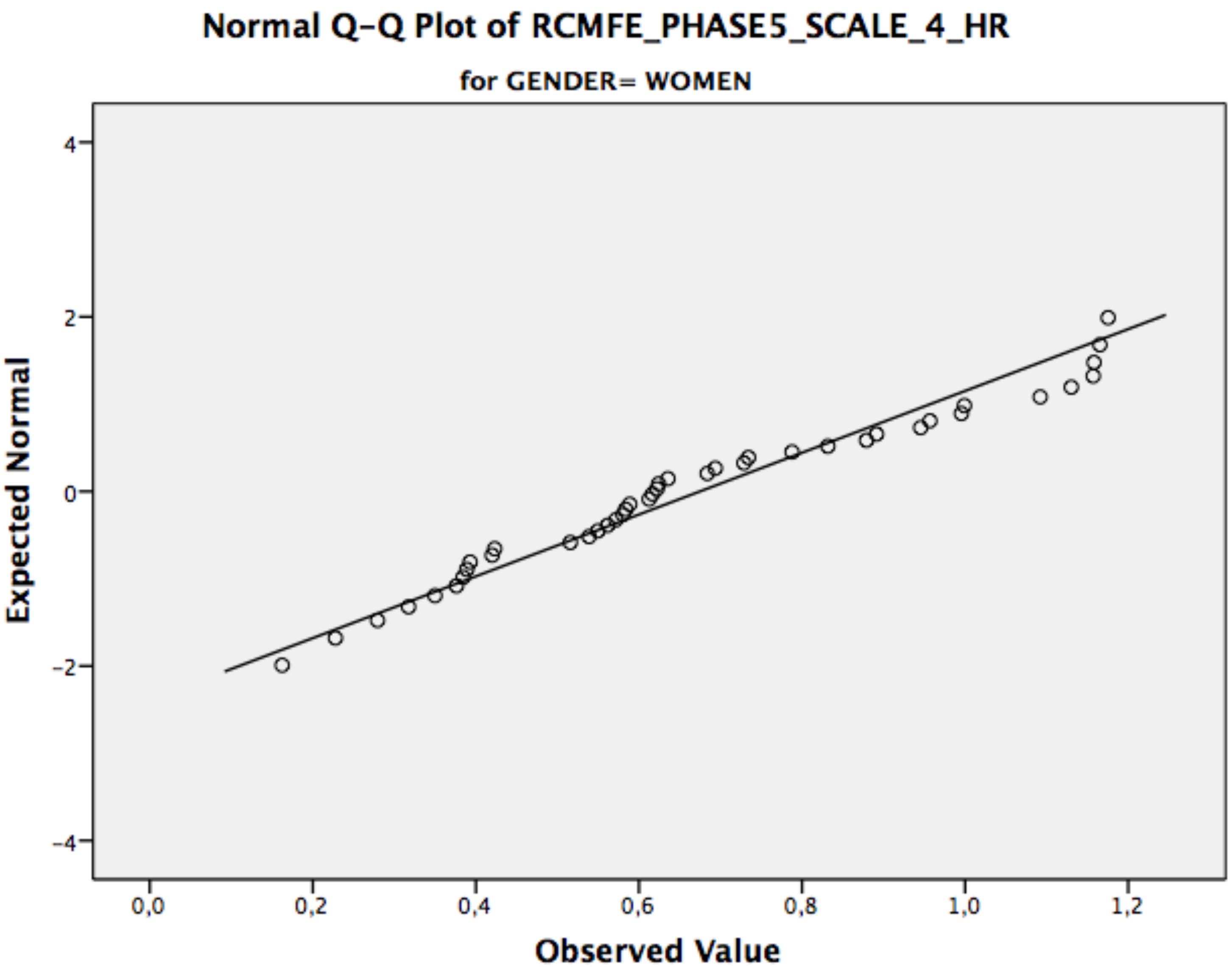

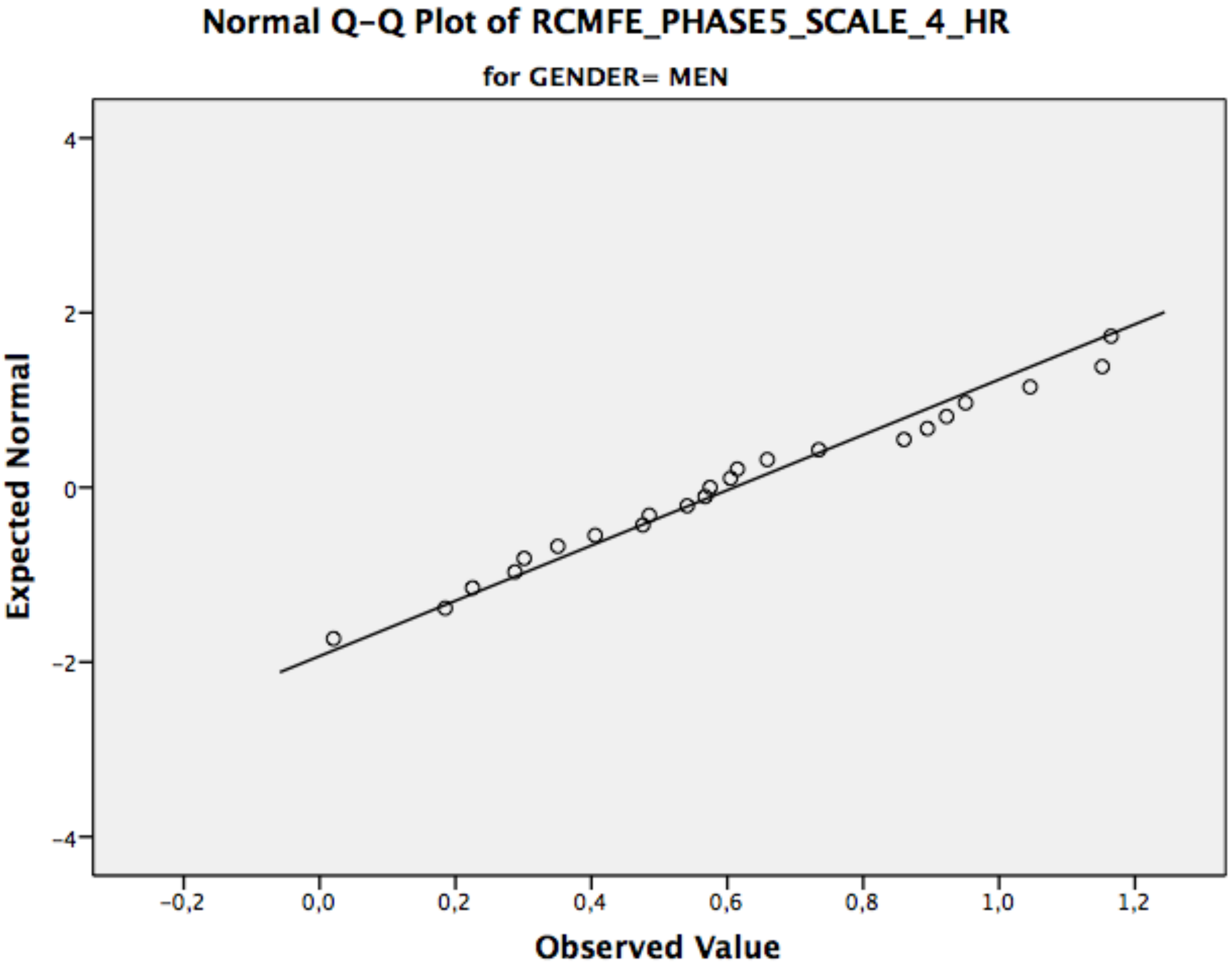

Normal Q-Q Plot of RCMFE_PHASE5_SCALE_4_HR
for GENDER= MEN
Expected Normal
Observed Value
4
2
0
−2
−4
−0,2
0,0
0,2
0,4
0,6
0,8
1,0
1,2

RCMFE_PHASE5_SCALE_5_HR

Normal Q-Q Plots

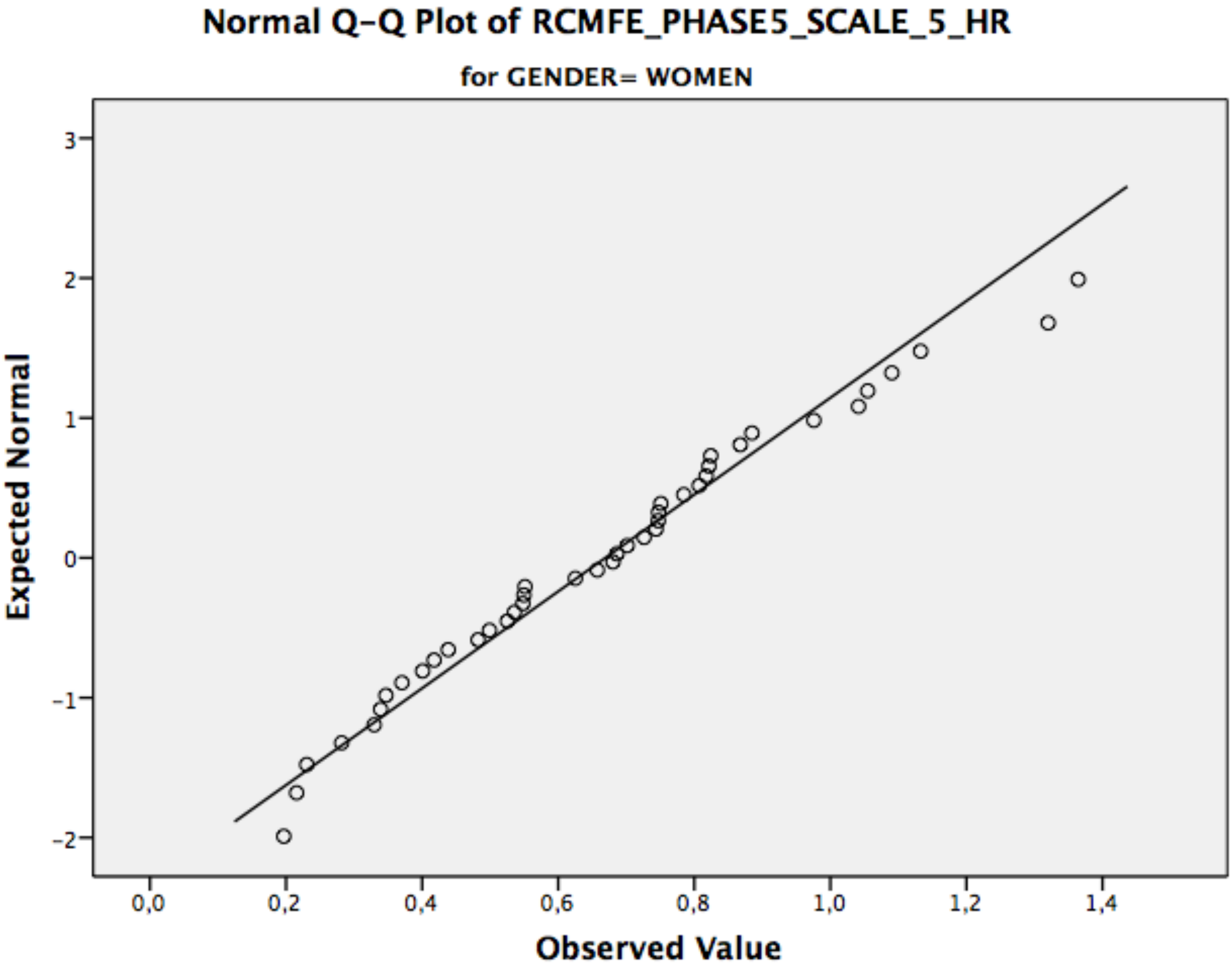

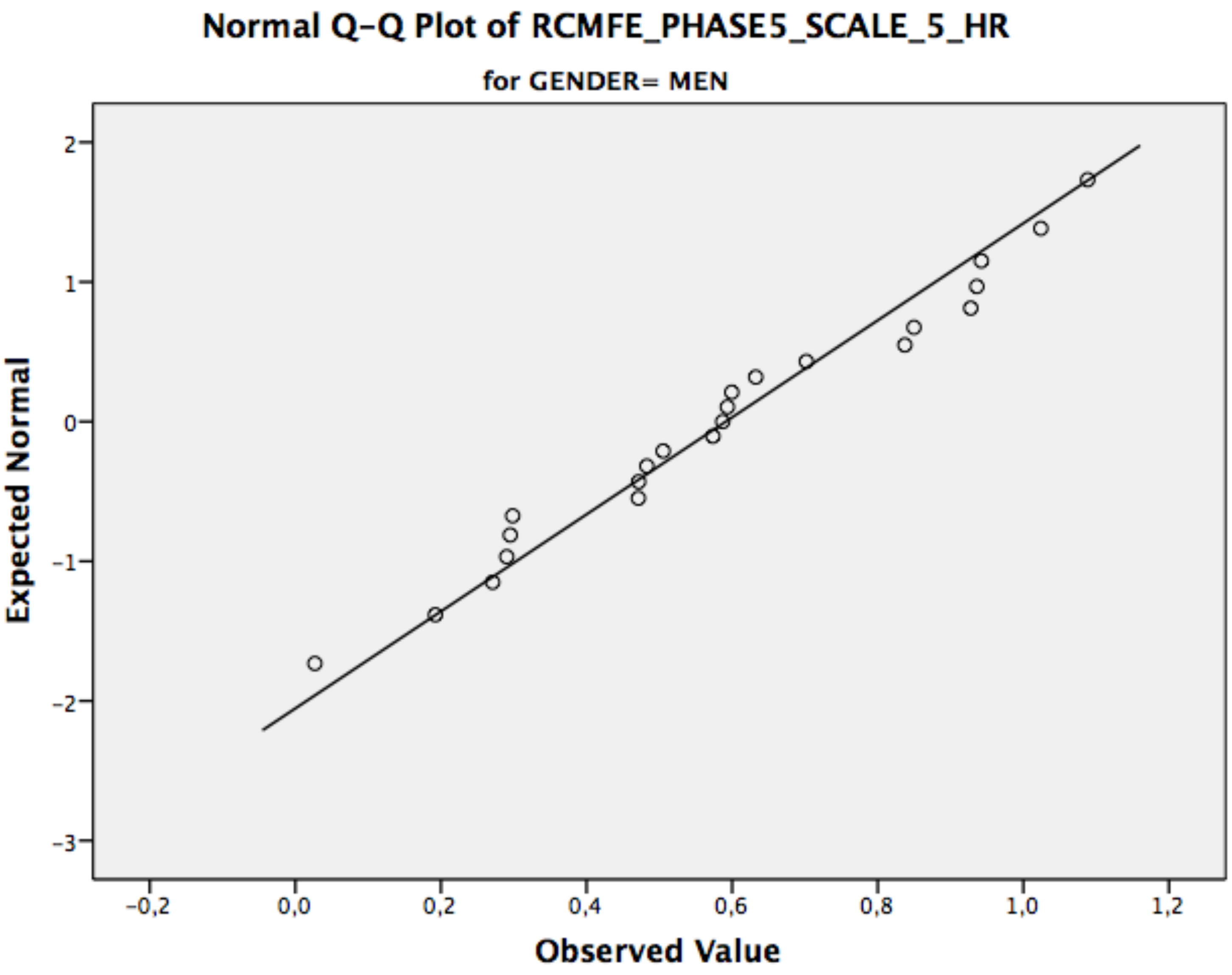
Normal Q-Q Plot of RCMFE_PHASE5_SCALE_5_HR
for GENDER= MEN
Expected Normal
Observed Value
2
1
0
-1
-2
-3
-0,2
0,0
0,2
0,4
0,6
0,8
1,0
1,2

Explore HR
PATHOLOGY

Case Processing Summary

	PATHOLOGY	Cases					
		Valid		Missing		Total	
		N	Percent	N	Percent	N	Percent
RCMFE_PHASE1_SCALE_1_HR	NO-OI	29	100,0%	0	0,0%	29	100,0%
	OI	36	100,0%	0	0,0%	36	100,0%
RCMFE_PHASE1_SCALE_2_HR	NO-OI	29	100,0%	0	0,0%	29	100,0%
	OI	36	100,0%	0	0,0%	36	100,0%
RCMFE_PHASE1_SCALE_3_HR	NO-OI	29	100,0%	0	0,0%	29	100,0%
	OI	36	100,0%	0	0,0%	36	100,0%
RCMFE_PHASE1_SCALE_4_HR	NO-OI	29	100,0%	0	0,0%	29	100,0%
	OI	36	100,0%	0	0,0%	36	100,0%
RCMFE_PHASE1_SCALE_5_HR	NO-OI	29	100,0%	0	0,0%	29	100,0%
	OI	36	100,0%	0	0,0%	36	100,0%
RCMFE_PHASE2_SCALE_1_HR	NO-OI	29	100,0%	0	0,0%	29	100,0%
	OI	36	100,0%	0	0,0%	36	100,0%
RCMFE_PHASE2_SCALE_2_HR	NO-OI	29	100,0%	0	0,0%	29	100,0%
	OI	36	100,0%	0	0,0%	36	100,0%
RCMFE_PHASE2_SCALE_3_HR	NO-OI	29	100,0%	0	0,0%	29	100,0%
	OI	36	100,0%	0	0,0%	36	100,0%
RCMFE_PHASE2_SCALE_4_HR	NO-OI	29	100,0%	0	0,0%	29	100,0%
	OI	36	100,0%	0	0,0%	36	100,0%
RCMFE_PHASE2_SCALE_5_HR	NO-OI	29	100,0%	0	0,0%	29	100,0%
	OI	36	100,0%	0	0,0%	36	100,0%
RCMFE_PHASE3_SCALE_1_HR	NO-OI	29	100,0%	0	0,0%	29	100,0%
	OI	36	100,0%	0	0,0%	36	100,0%
RCMFE_PHASE3_SCALE_2_HR	NO-OI	29	100,0%	0	0,0%	29	100,0%
	OI	36	100,0%	0	0,0%	36	100,0%
RCMFE_PHASE3_SCALE_3_HR	NO-OI	29	100,0%	0	0,0%	29	100,0%
	OI	36	100,0%	0	0,0%	36	100,0%
RCMFE_PHASE3_SCALE_4_HR	NO-OI	29	100,0%	0	0,0%	29	100,0%
	OI	36	100,0%	0	0,0%	36	100,0%
RCMFE_PHASE3_SCALE_5_HR	NO-OI	29	100,0%	0	0,0%	29	100,0%
	OI	36	100,0%	0	0,0%	36	100,0%
RCMFE_PHASE4_SCALE_1_HR	NO-OI	29	100,0%	0	0,0%	29	100,0%
	OI	36	100,0%	0	0,0%	36	100,0%
RCMFE_PHASE4_SCALE_2_HR	NO-OI	29	100,0%	0	0,0%	29	100,0%
	OI	36	100,0%	0	0,0%	36	100,0%
RCMFE_PHASE4_SCALE_3	NO-OI	29	100,0%	0	0,0%	29	100,0%
	OI	36	100,0%	0	0,0%	36	100,0%
RCMFE_PHASE4_SCALE_4_HR	NO-OI	29	100,0%	0	0,0%	29	100,0%
	OI	36	100,0%	0	0,0%	36	100,0%
RCMFE_PHASE4_SCALE_5_HR	NO-OI	29	100,0%	0	0,0%	29	100,0%
	OI	36	100,0%	0	0,0%	36	100,0%
RCMFE_PHASE5_SCALE_1_HR	NO-OI	29	100,0%	0	0,0%	29	100,0%

Case Processing Summary

	PATHOLOGY	Cases					
		Valid		Missing		Total	
		N	Percent	N	Percent	N	Percent
RCMFE_PHASE5_SCALE_1_HR	OI	36	100,0%	0	0,0%	36	100,0%
RCMFE_PHASE5_SCALE_2_HR	NO-OI	29	100,0%	0	0,0%	29	100,0%
	OI	36	100,0%	0	0,0%	36	100,0%
RCMFE_PHASE5_SCALE_3_HR	NO-OI	29	100,0%	0	0,0%	29	100,0%
	OI	36	100,0%	0	0,0%	36	100,0%
RCMFE_PHASE5_SCALE_4_HR	NO-OI	29	100,0%	0	0,0%	29	100,0%
	OI	36	100,0%	0	0,0%	36	100,0%
RCMFE_PHASE5_SCALE_5_HR	NO-OI	29	100,0%	0	0,0%	29	100,0%
	OI	36	100,0%	0	0,0%	36	100,0%

Tests of Normality

	PATHOLOGY	Kolmogorov-Smirnov[a]			Shapiro-Wilk		
		Statistic	df	Sig.	Statistic	df	Sig.
RCMFE_PHASE1_SCALE_1_HR	NO-OI	,111	29	,200*	,977	29	,752
	OI	,195	36	,001	,897	36	,003
RCMFE_PHASE1_SCALE_2_HR	NO-OI	,097	29	,200*	,971	29	,580
	OI	,122	36	,193	,961	36	,233
RCMFE_PHASE1_SCALE_3_HR	NO-OI	,099	29	,200*	,974	29	,669
	OI	,128	36	,144	,970	36	,438
RCMFE_PHASE1_SCALE_4_HR	NO-OI	,071	29	,200*	,990	29	,990
	OI	,115	36	,200*	,968	36	,374
RCMFE_PHASE1_SCALE_5_HR	NO-OI	,172	29	,028	,947	29	,155
	OI	,098	36	,200*	,962	36	,252
RCMFE_PHASE2_SCALE_1_HR	NO-OI	,129	29	,200*	,908	29	,015
	OI	,161	36	,019	,873	36	,051
RCMFE_PHASE2_SCALE_2_HR	NO-OI	,121	29	,200*	,935	29	,075
	OI	,231	36	,050	,856	36	,020
RCMFE_PHASE2_SCALE_3_HR	NO-OI	,182	29	,015	,925	29	,042
	OI	,213	36	,050	,863	36	,080
RCMFE_PHASE2_SCALE_4_HR	NO-OI	,173	29	,027	,914	29	,021
	OI	,207	36	,060	,857	36	,000
RCMFE_PHASE2_SCALE_5_HR	NO-OI	,138	29	,170	,938	29	,090
	OI	,206	36	,070	,840	36	,080
RCMFE_PHASE3_SCALE_1_HR	NO-OI	,093	29	,200*	,941	29	,107
	OI	,137	36	,085	,936	36	,038
RCMFE_PHASE3_SCALE_2_HR	NO-OI	,106	29	,200*	,974	29	,666
	OI	,100	36	,200*	,966	36	,318
RCMFE_PHASE3_SCALE_3_HR	NO-OI	,080	29	,200*	,987	29	,972
	OI	,102	36	,200*	,974	36	,550
RCMFE_PHASE3_SCALE_4_HR	NO-OI	,079	29	,200*	,987	29	,964
	OI	,174	36	,007	,941	36	,056

RCMFE_PHASE3_SCALE_5_HR	NO-OI	,077	29	,200*	,987	29	,969
	OI	,114	36	,200*	,980	36	,735
RCMFE_PHASE4_SCALE_1_HR	NO-OI	,157	29	,067	,904	29	,012
	OI	,134	36	,103	,933	36	,030
RCMFE_PHASE4_SCALE_2_HR	NO-OI	,134	29	,193	,922	29	,034
	OI	,137	36	,085	,914	36	,008
RCMFE_PHASE4_SCALE_3	NO-OI	,138	29	,164	,884	29	,004
	OI	,155	36	,029	,900	36	,053
RCMFE_PHASE4_SCALE_4_HR	NO-OI	,202	29	,004	,836	29	,060
	OI	,154	36	,031	,901	36	,074
RCMFE_PHASE4_SCALE_5_HR	NO-OI	,163	29	,048	,838	29	,000
	OI	,139	36	,075	,897	36	,003
RCMFE_PHASE5_SCALE_1_HR	NO-OI	,107	29	,200*	,972	29	,606
	OI	,065	36	,200*	,981	36	,790

Tests of Normality

	PATHOLOGY	Kolmogorov-Smirnov[a]			Shapiro-Wilk		
		Statistic	df	Sig.	Statistic	df	Sig.
RCMFE_PHASE5_SCALE_2_HR	NO-OI	,097	29	,200*	,963	29	,385
	OI	,142	36	,063	,931	36	,026
RCMFE_PHASE5_SCALE_3_HR	NO-OI	,123	29	,200*	,963	29	,381
	OI	,204	36	,061	,921	36	,074
RCMFE_PHASE5_SCALE_4_HR	NO-OI	,129	29	,200*	,939	29	,094
	OI	,176	36	,057	,905	36	,055
RCMFE_PHASE5_SCALE_5_HR	NO-OI	,141	29	,145*	,953	29	,222
	OI	,102	36	,200*	,950	36	,107

*. This is a lower bound of the true significance.

a. Lilliefors Significance Correction

RCMFE_PHASE1_SCALE_1_HR

Normal Q-Q Plots

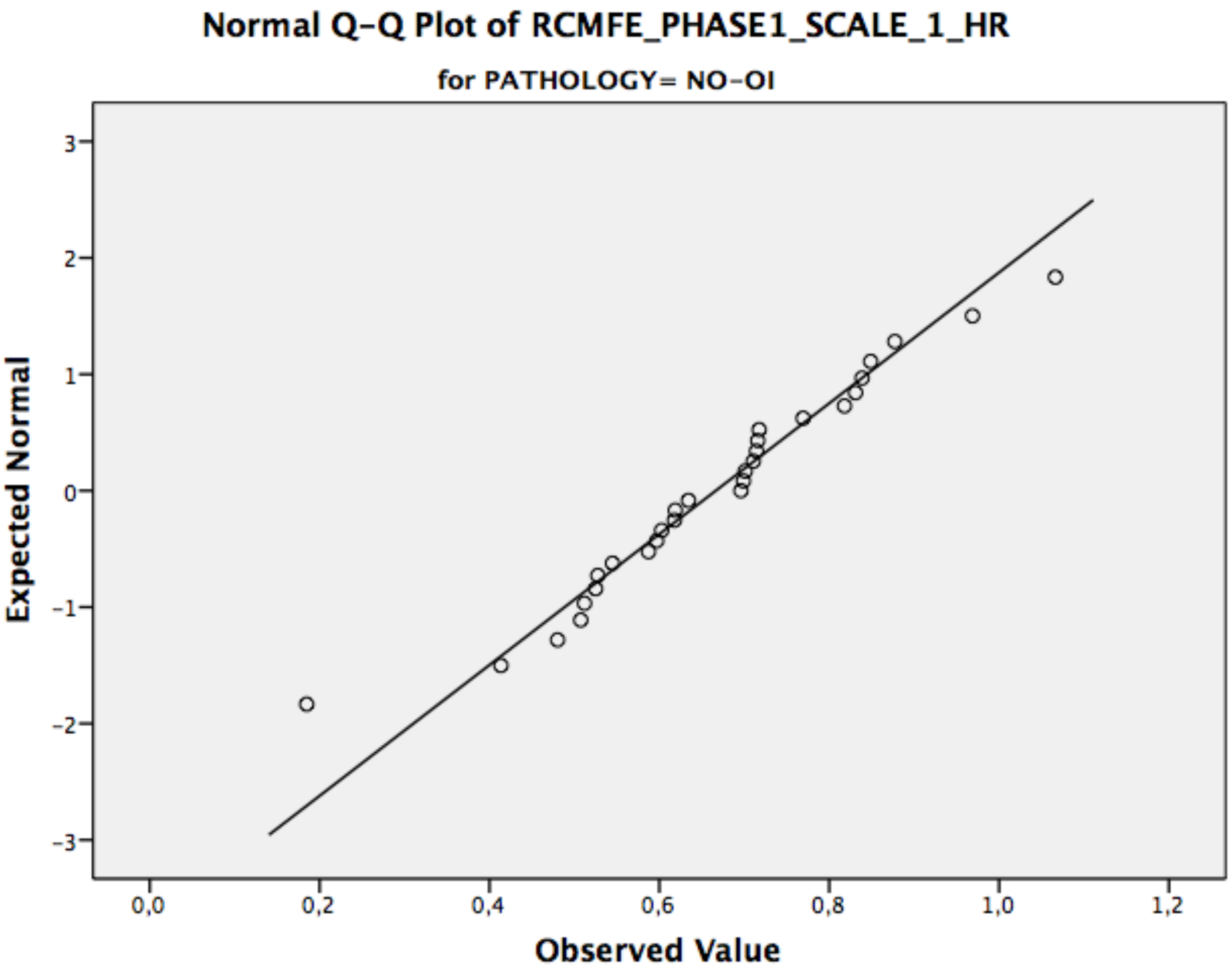

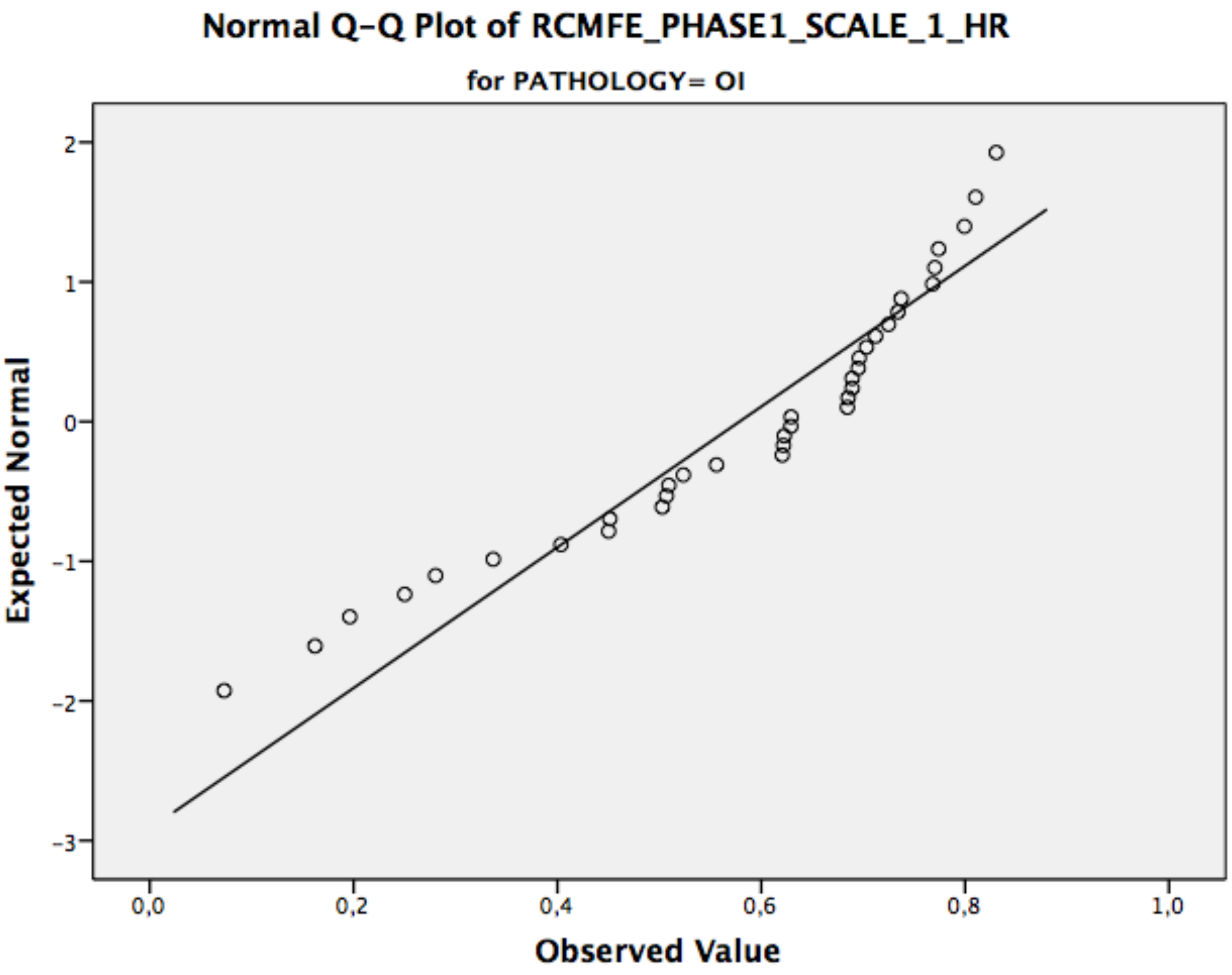
Normal Q-Q Plot of RCMFE_PHASE1_SCALE_1_HR
for PATHOLOGY= OI
Expected Normal
Observed Value
2
1
0
-1
-2
-3
0,0
0,2
0,4
0,6
0,8
1,0

RCMFE_PHASE1_SCALE_2_HR

Normal Q-Q Plots

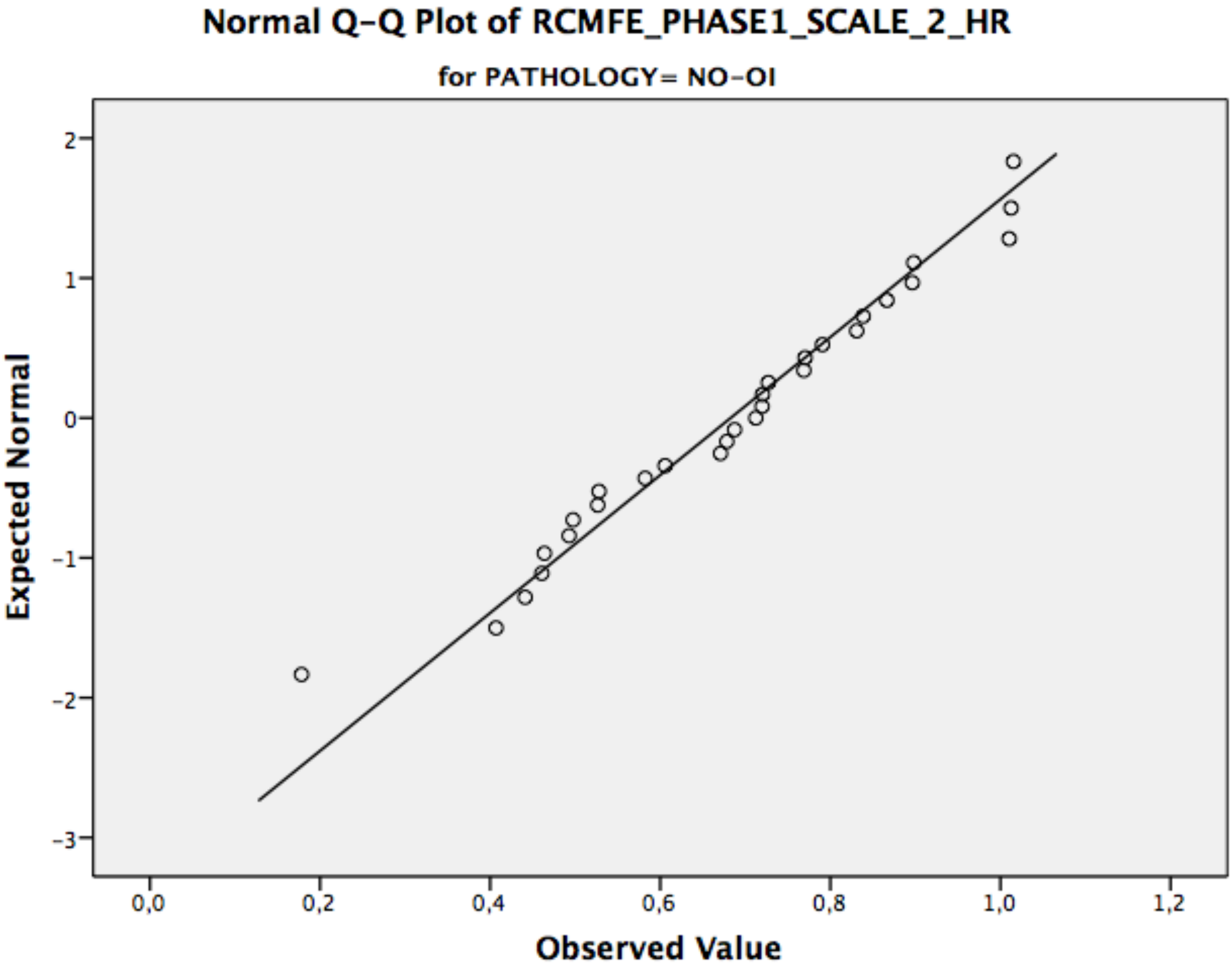

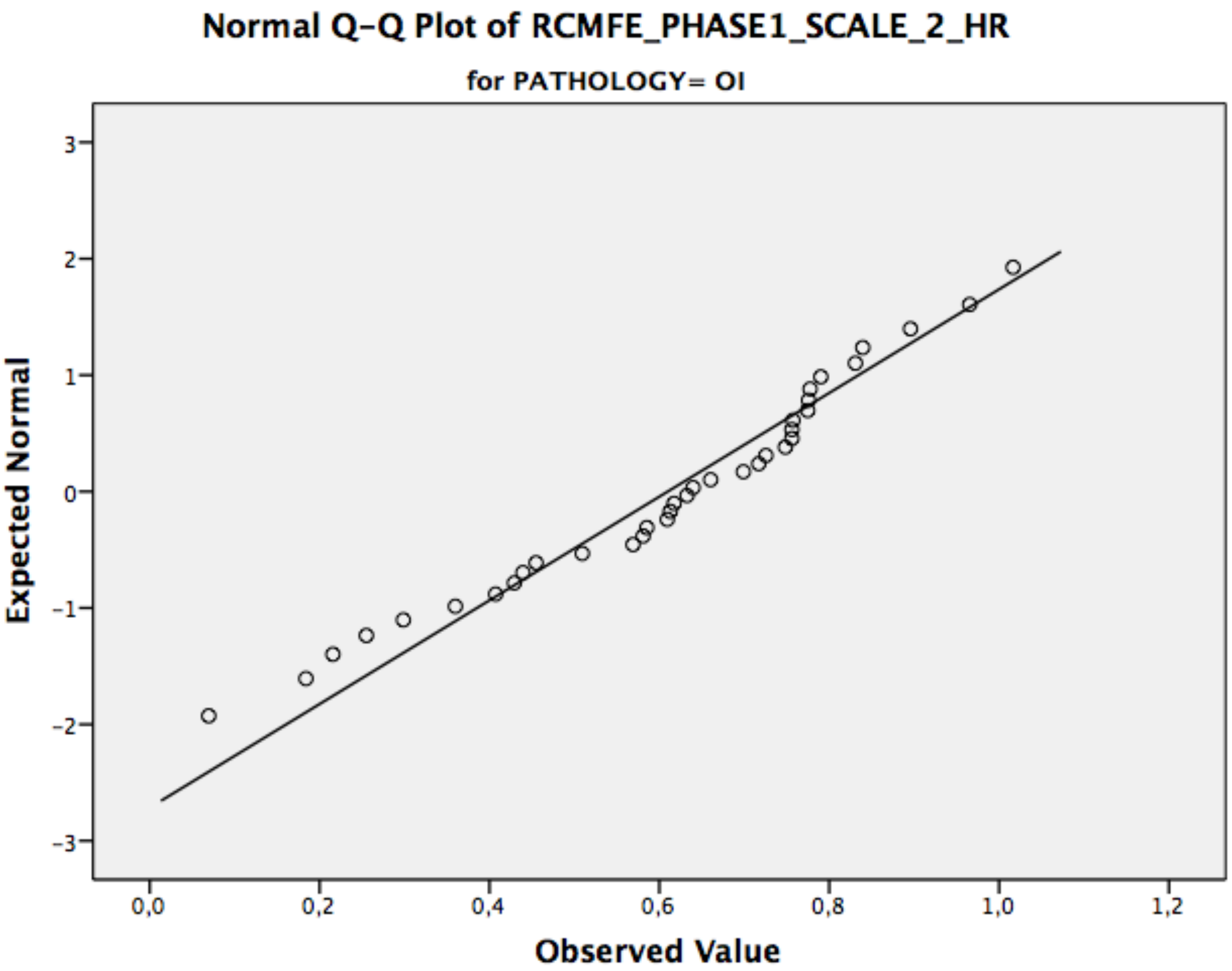
Normal Q-Q Plot of RCMFE_PHASE1_SCALE_2_HR
for PATHOLOGY= OI
Expected Normal
Observed Value
3
2
1
0
−1
−2
−3
0,0
0,2
0,4
0,6
0,8
1,0
1,2

RCMFE_PHASE1_SCALE_3_HR

Normal Q-Q Plots

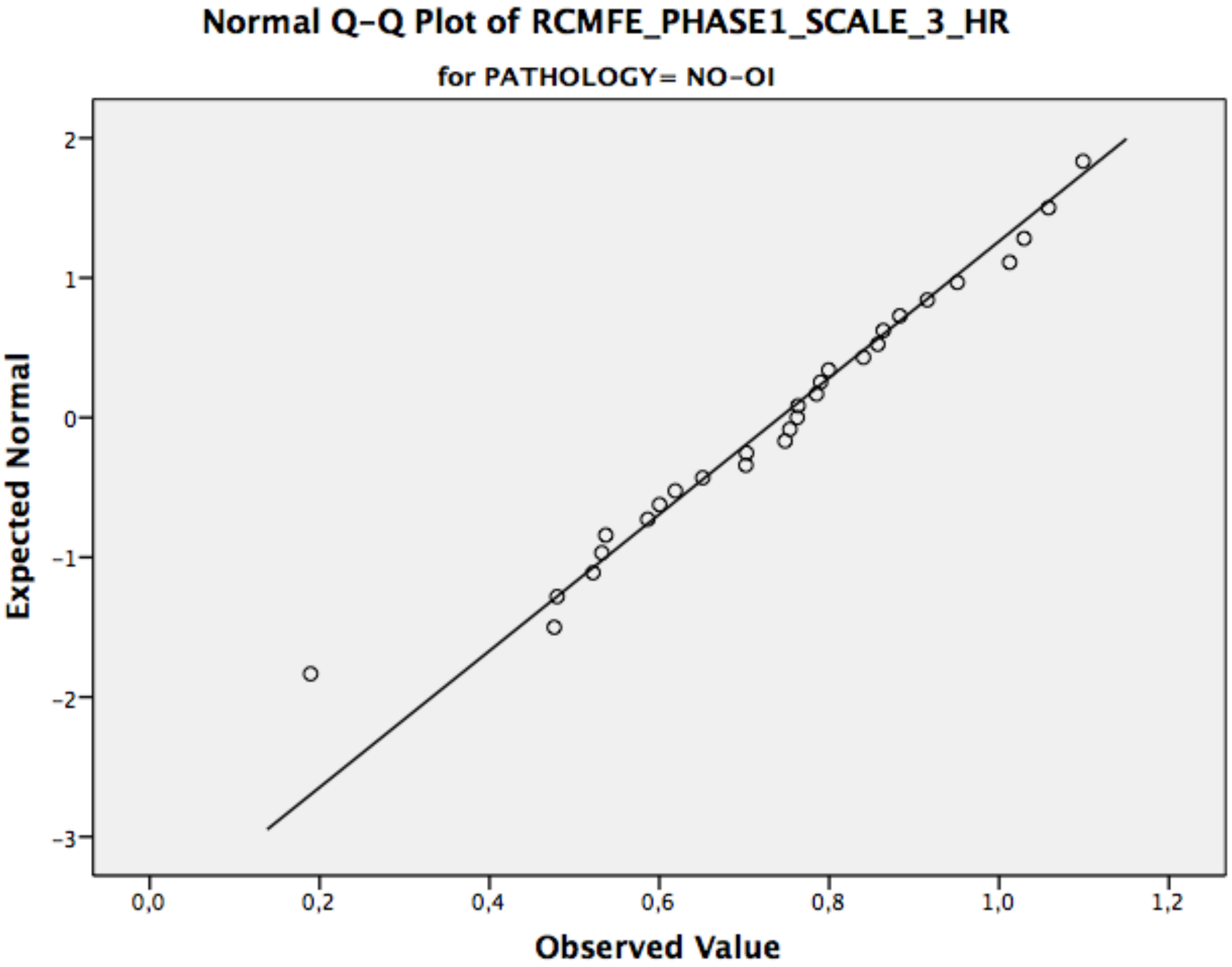

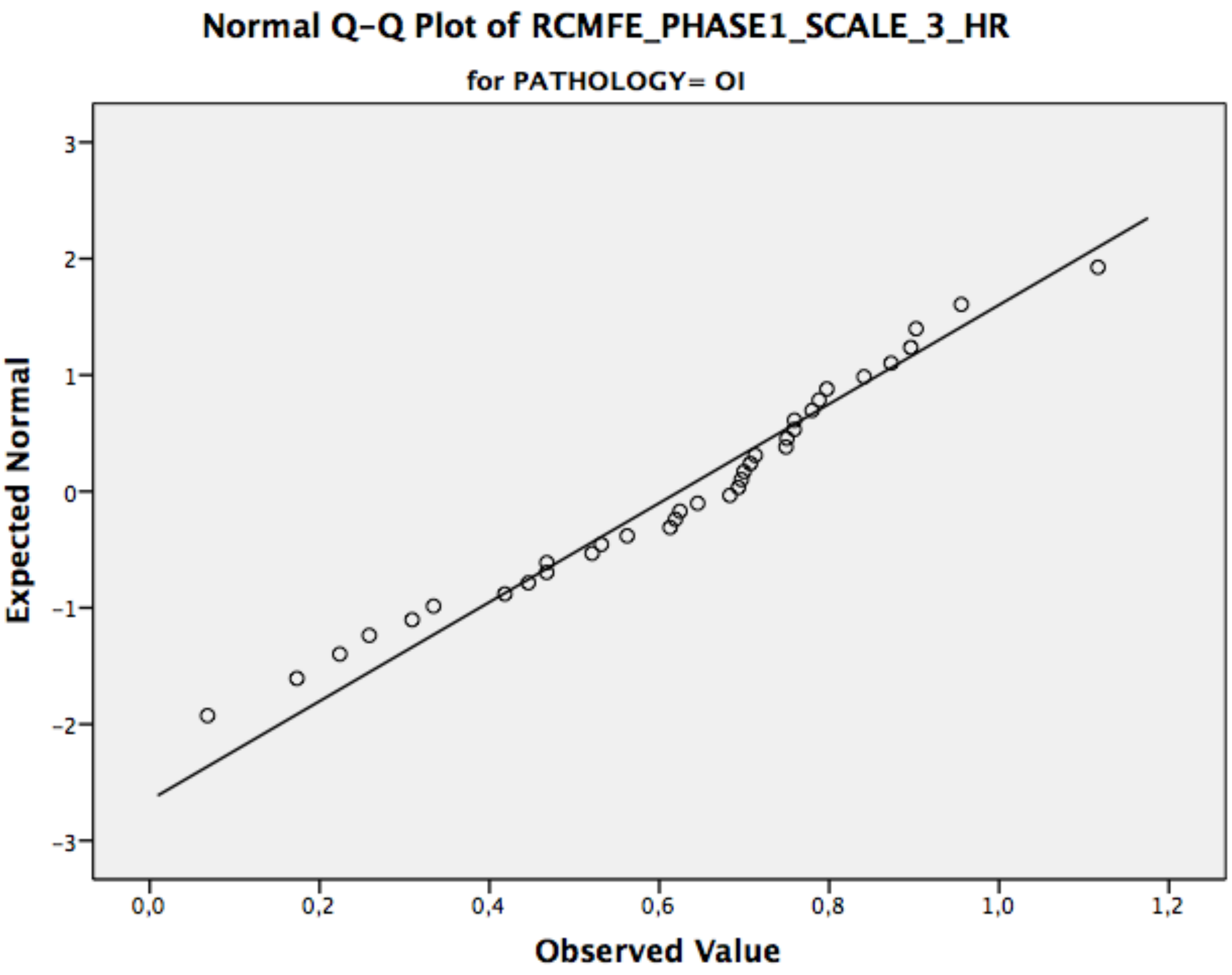
Normal Q-Q Plot of RCMFE_PHASE1_SCALE_3_HR
for PATHOLOGY= OI
Expected Normal
Observed Value
3
2
1
0
-1
-2
-3
0,0
0,2
0,4
0,6
0,8
1,0
1,2

RCMFE_PHASE1_SCALE_4_HR

Normal Q-Q Plots

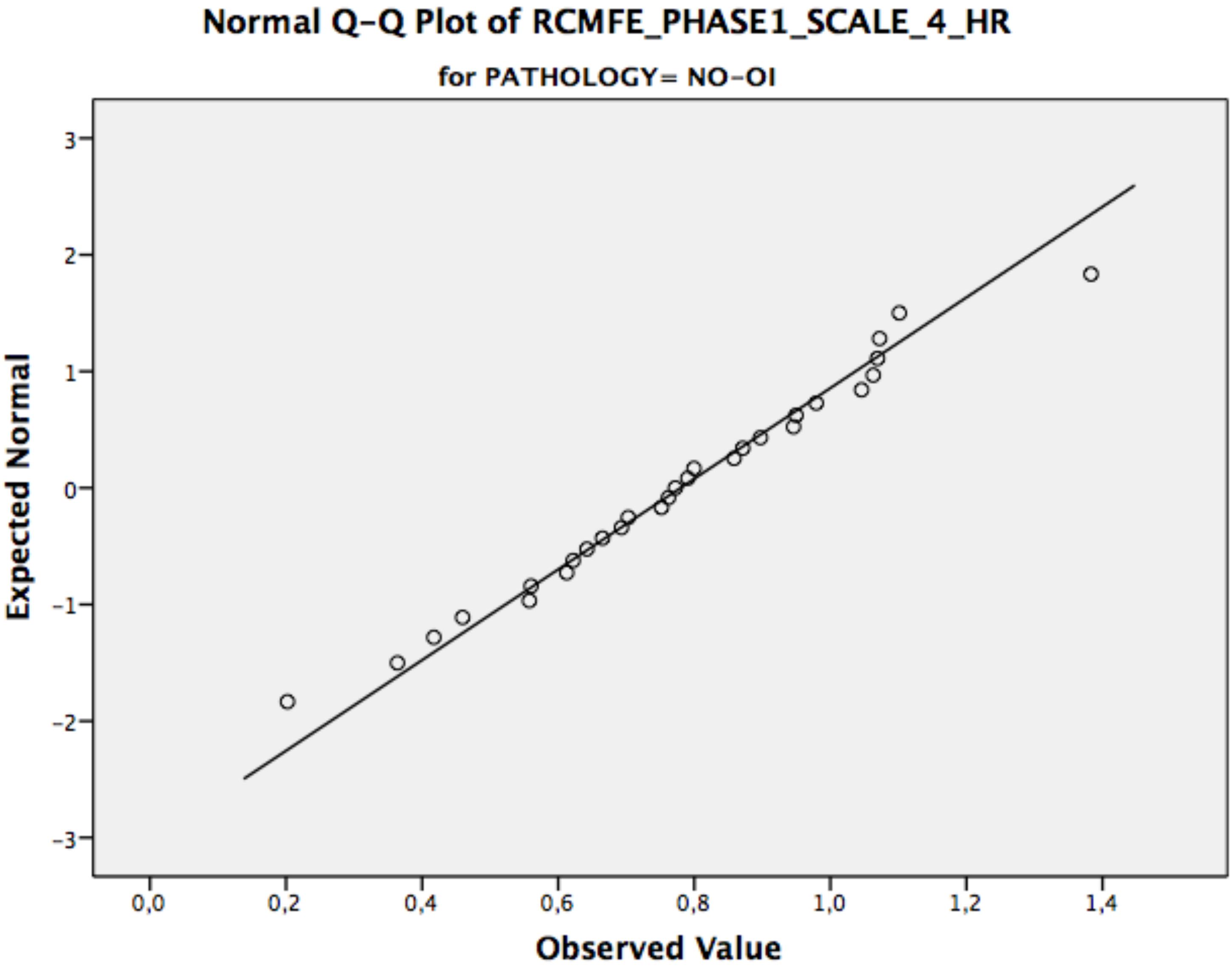

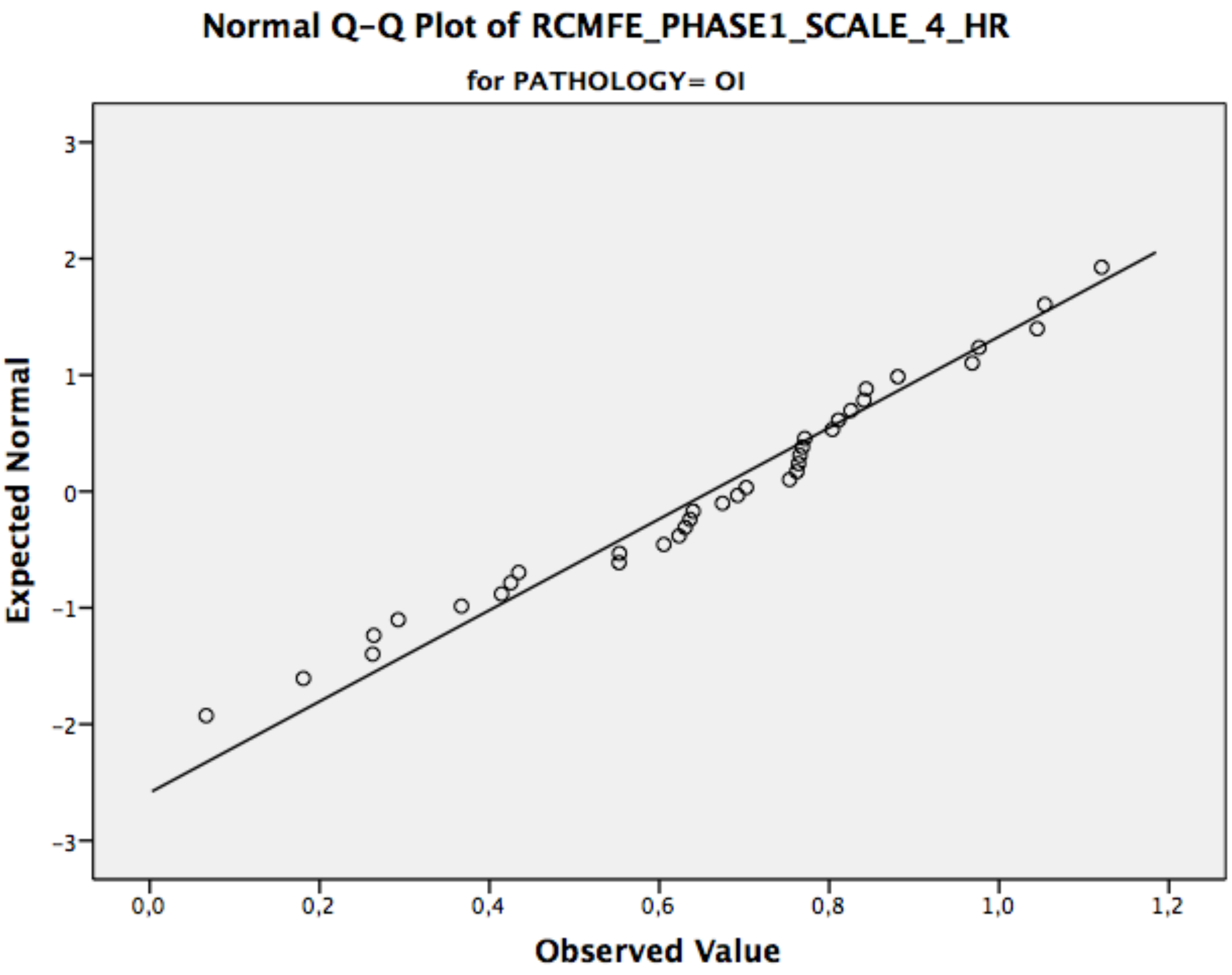
Normal Q-Q Plot of RCMFE_PHASE1_SCALE_4_HR
for PATHOLOGY= OI
Expected Normal
Observed Value
3
2
1
0
−1
−2
−3
0,0
0,2
0,4
0,6
0,8
1,0
1,2

RCMFE_PHASE1_SCALE_5_HR

Normal Q-Q Plots

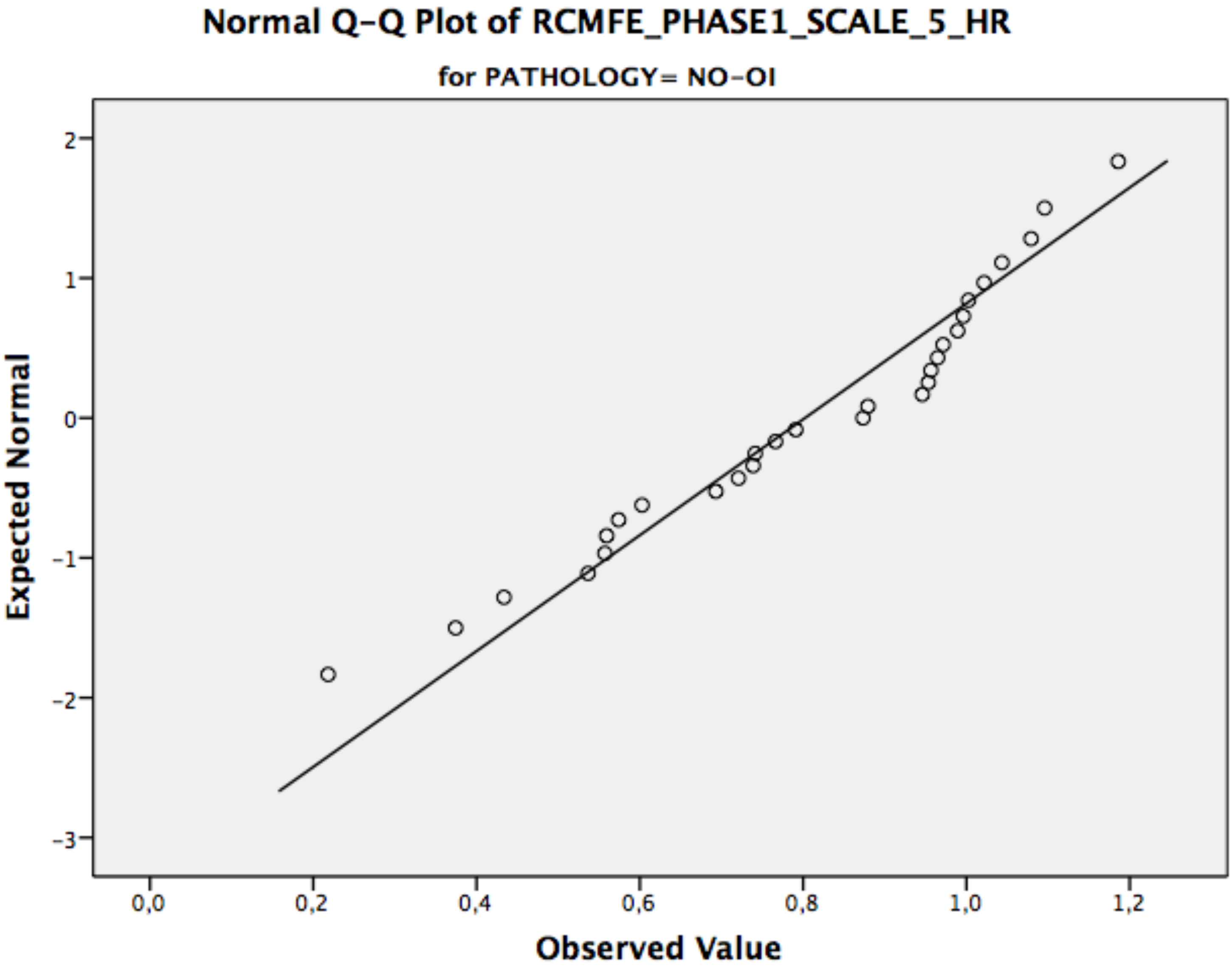

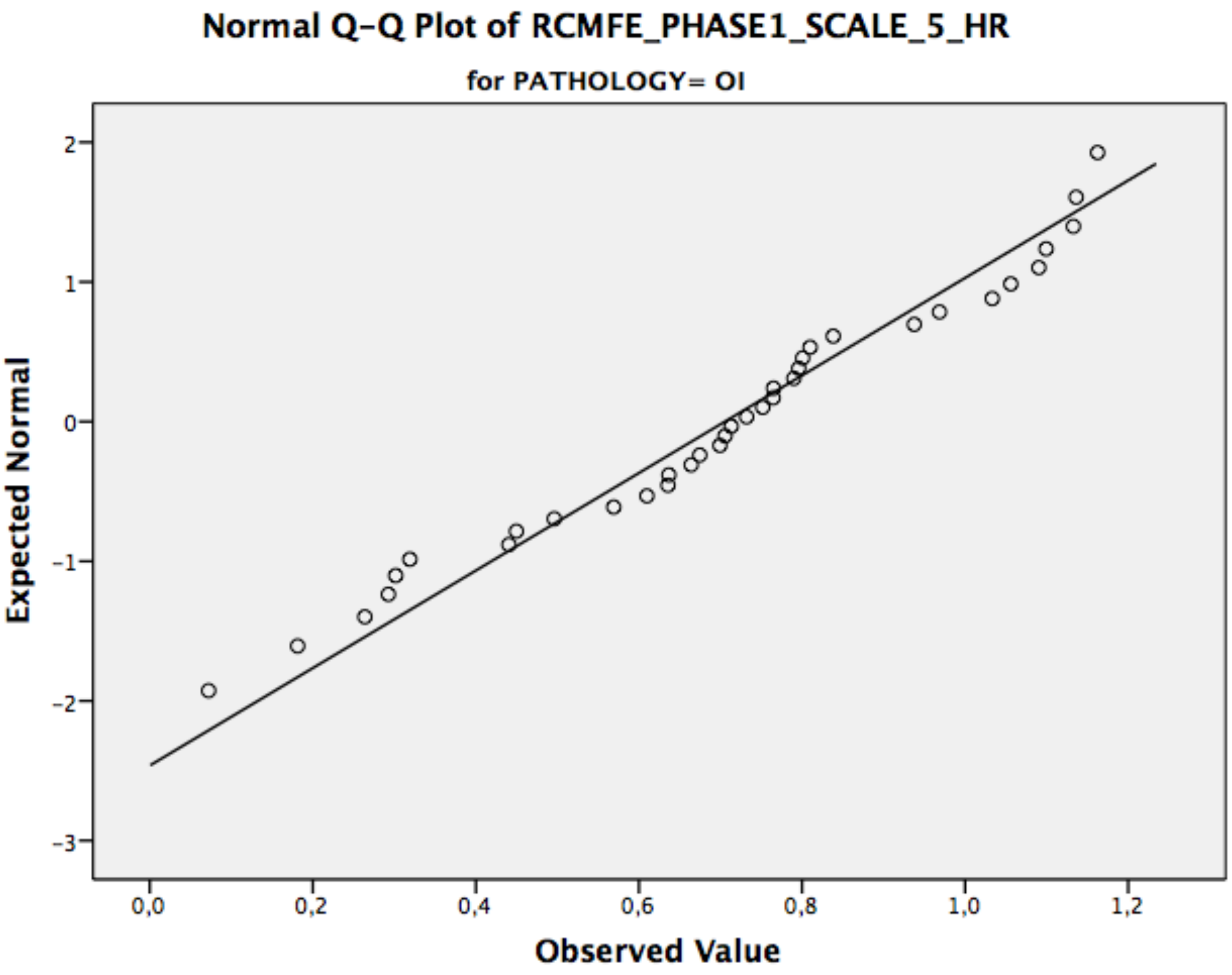

Normal Q-Q Plot of RCMFE_PHASE1_SCALE_5_HR
for PATHOLOGY= OI
Expected Normal
Observed Value
2
1
0
-1
-2
-3
0,0
0,2
0,4
0,6
0,8
1,0
1,2

RCMFE_PHASE2_SCALE_1_HR

Normal Q-Q Plots

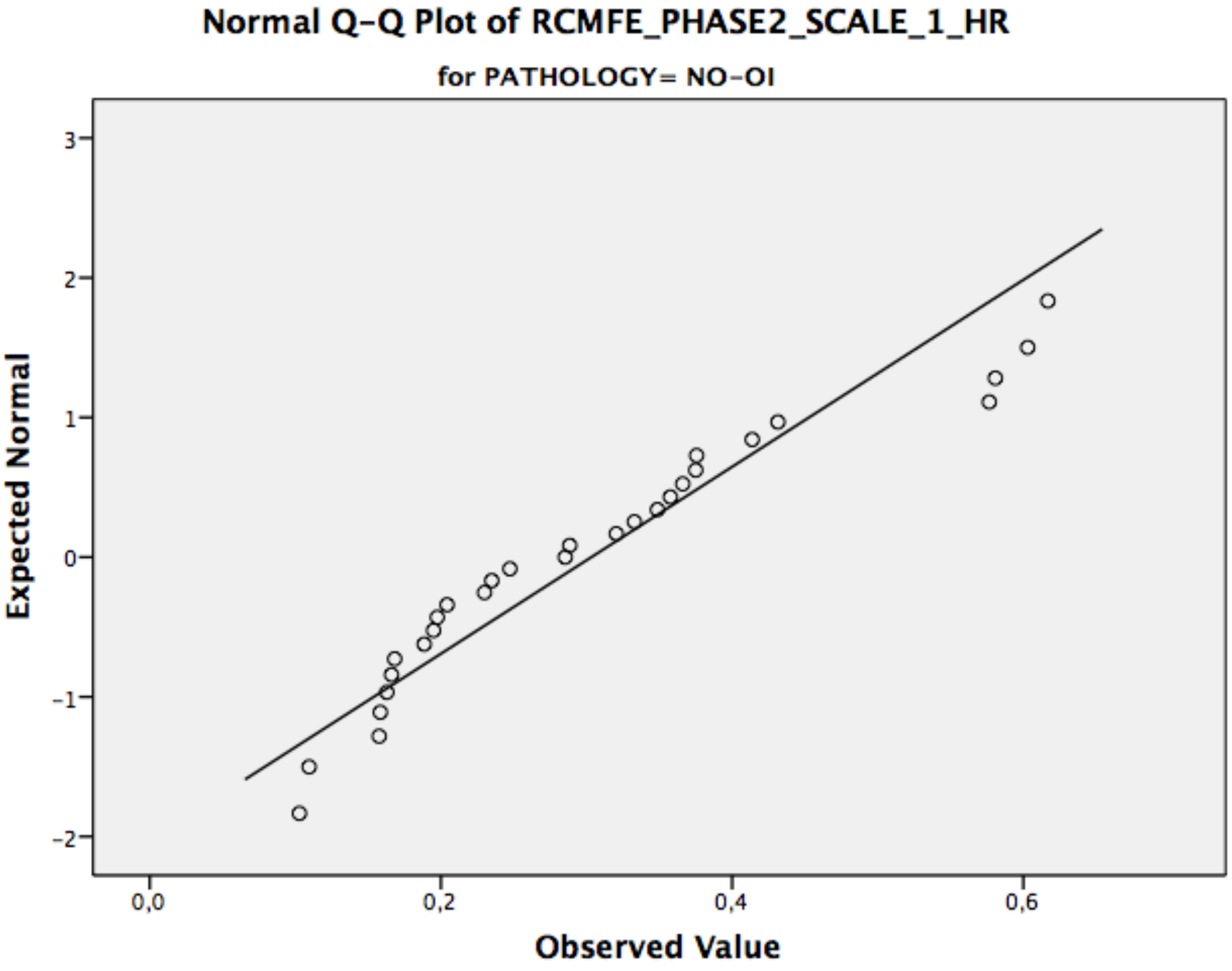

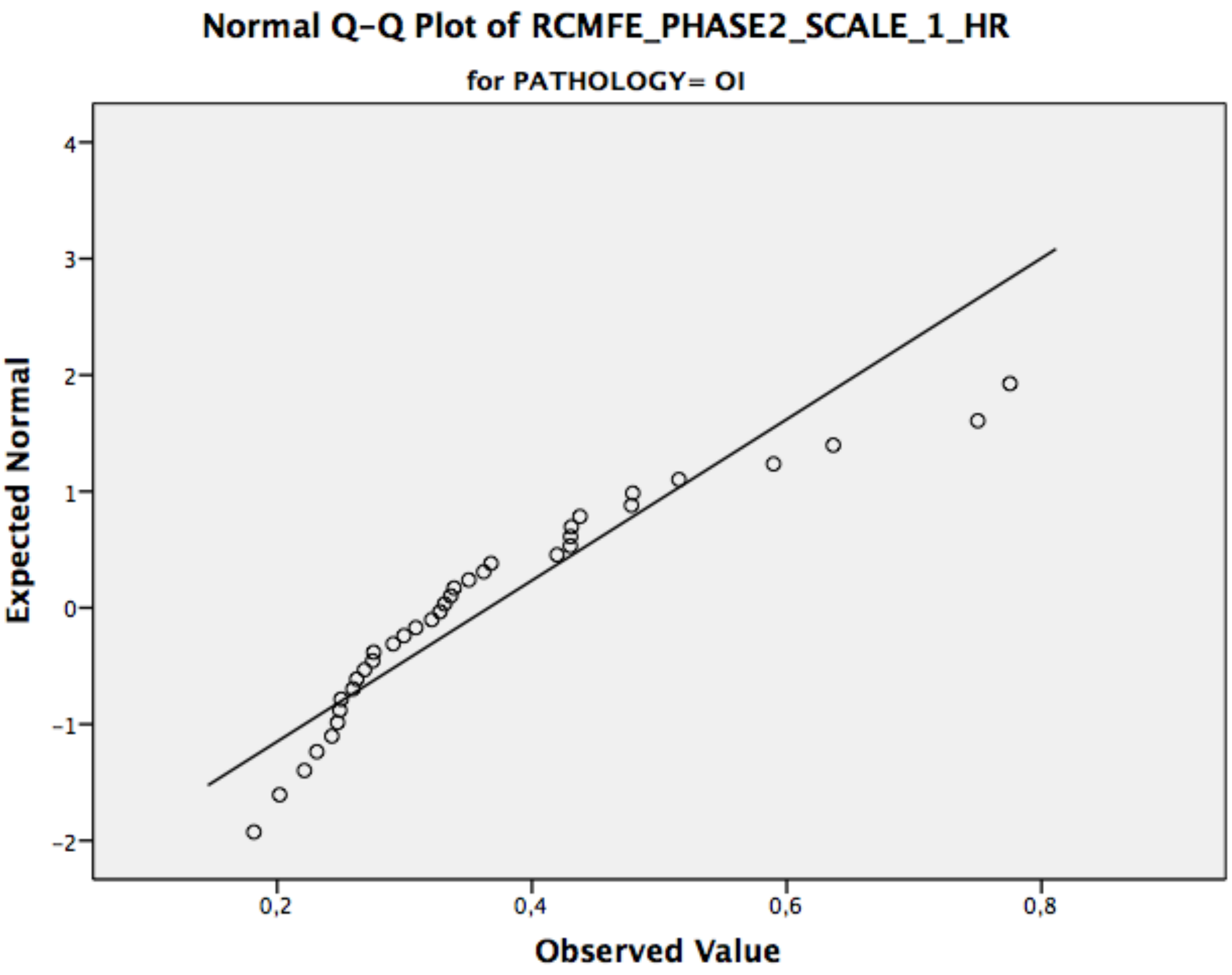
Normal Q-Q Plot of RCMFE_PHASE2_SCALE_1_HR
for PATHOLOGY= OI
Expected Normal
Observed Value
4
3
2
1
0
-1
-2
0,2
0,4
0,6
0,8

RCMFE_PHASE2_SCALE_2_HR

Normal Q-Q Plots

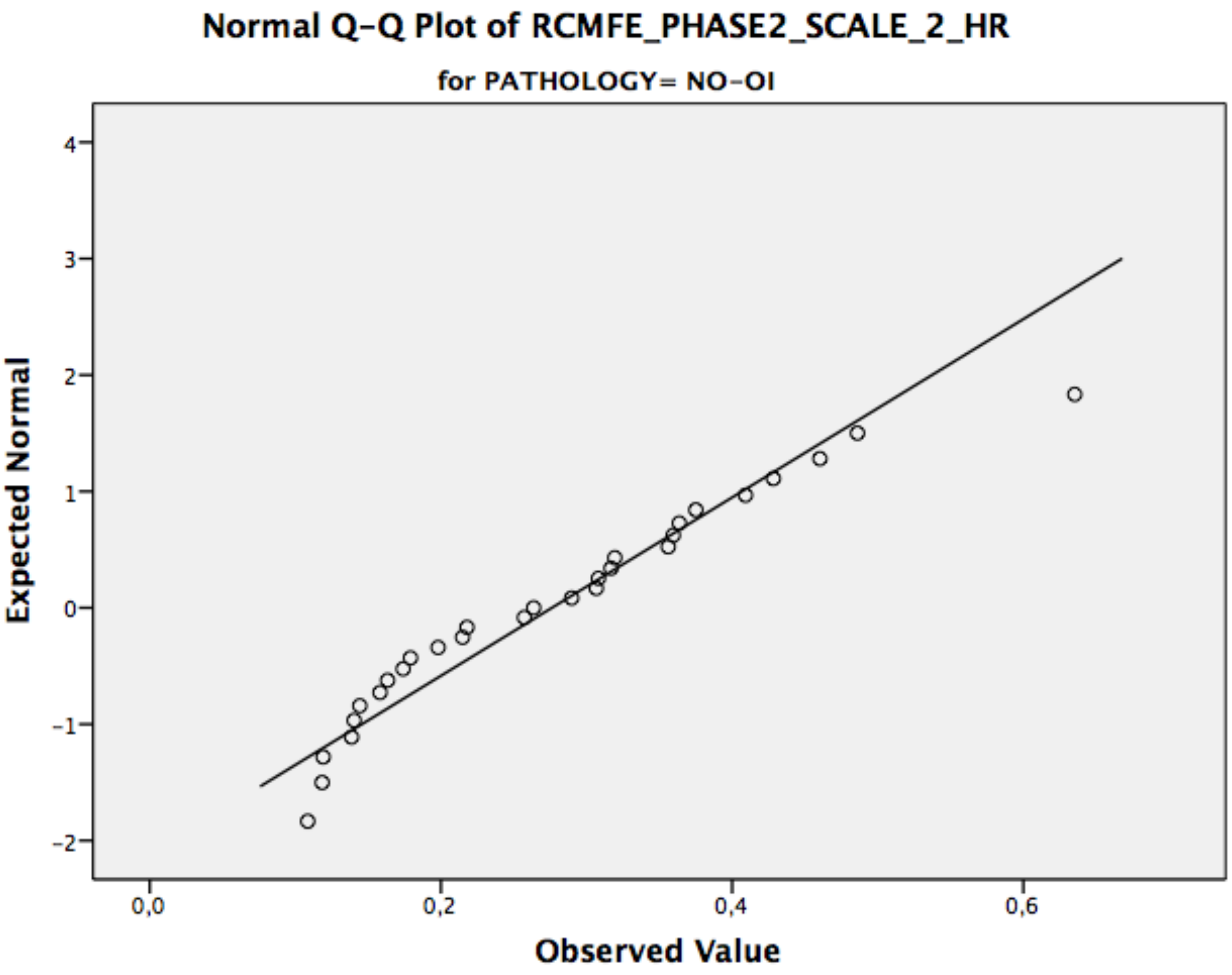

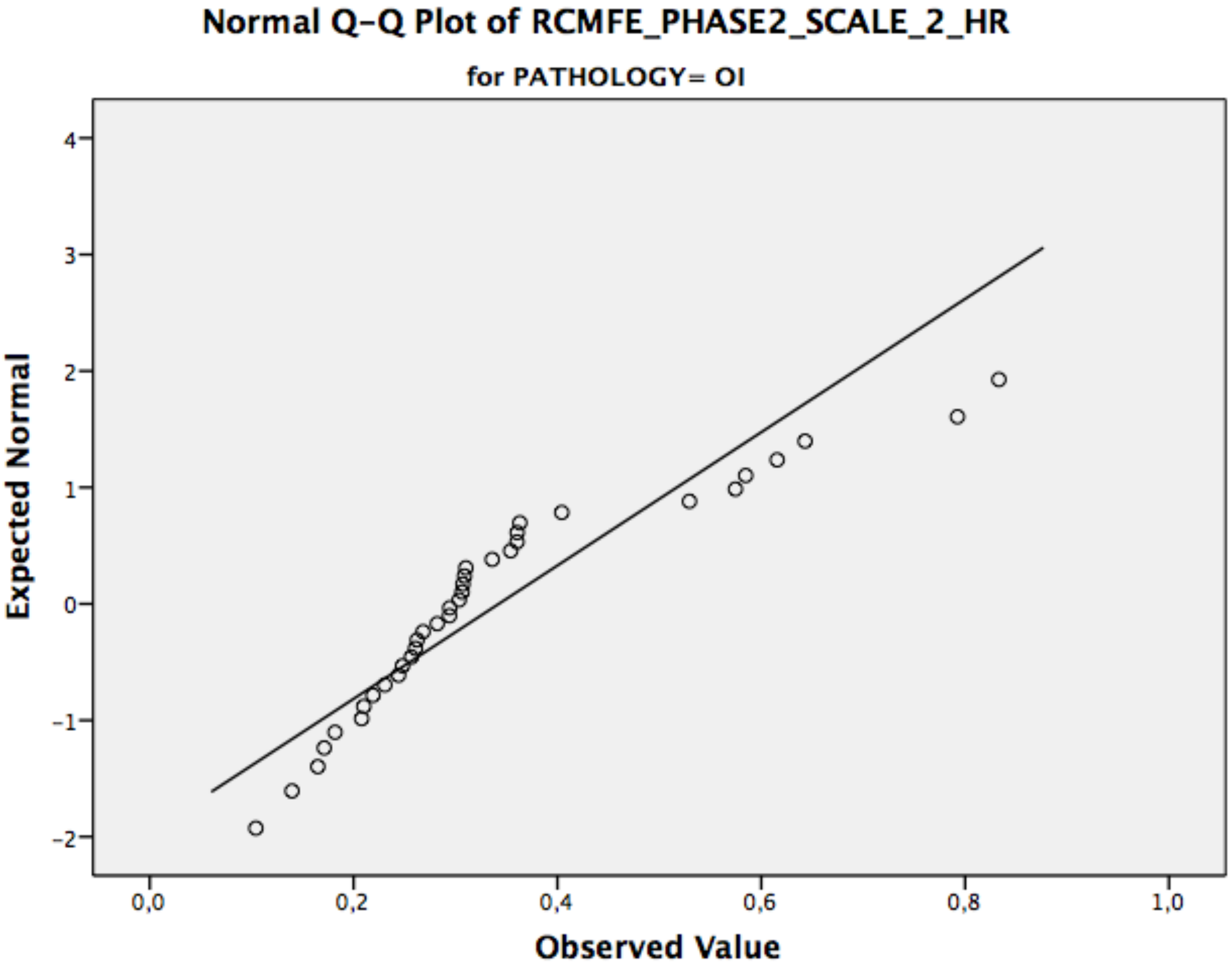

Normal Q-Q Plot of RCMFE_PHASE2_SCALE_2_HR
for PATHOLOGY= OI
Expected Normal
Observed Value
4
3
2
1
0
-1
-2
0,0
0,2
0,4
0,6
0,8
1,0

RCMFE_PHASE2_SCALE_3_HR

Normal Q-Q Plots

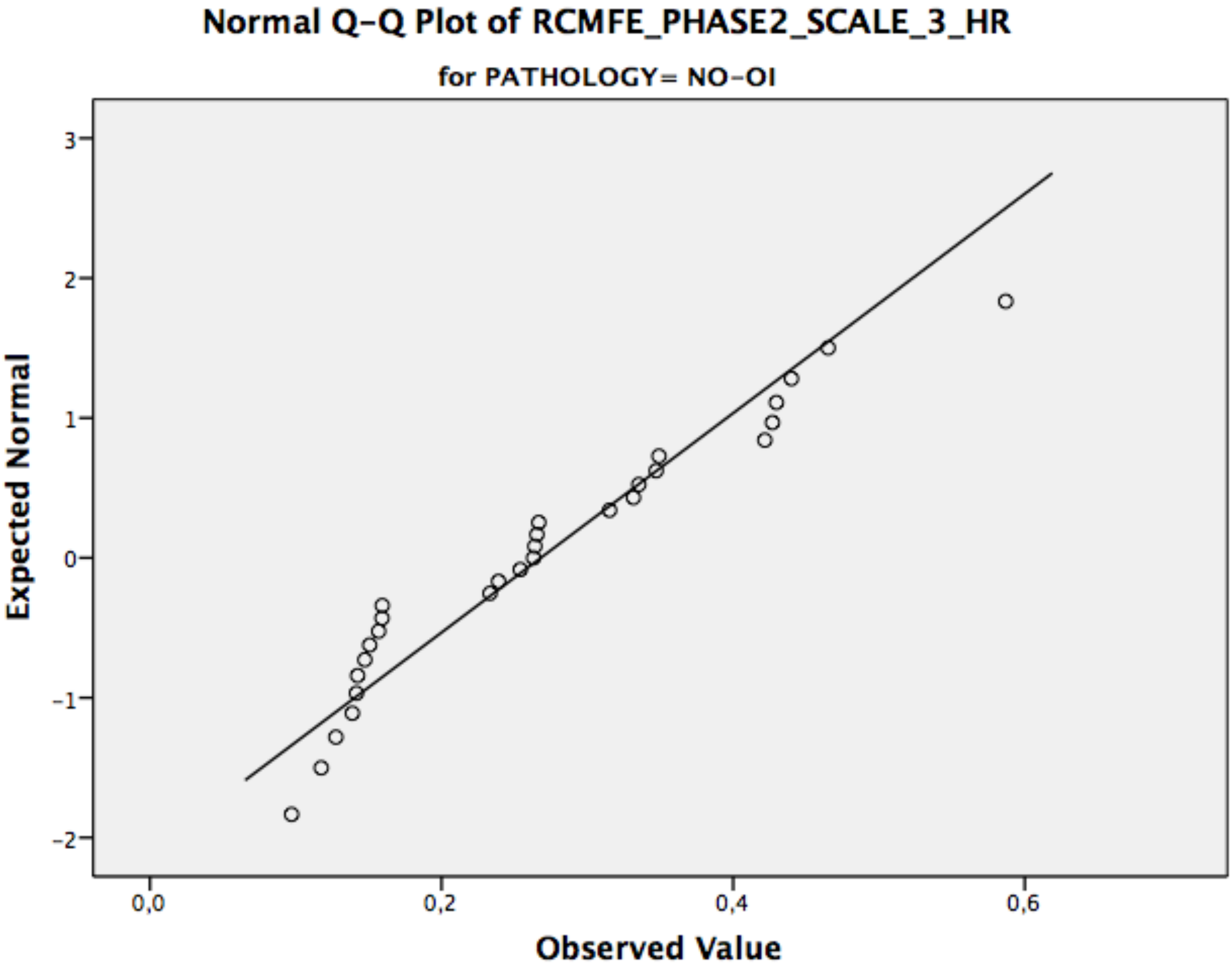

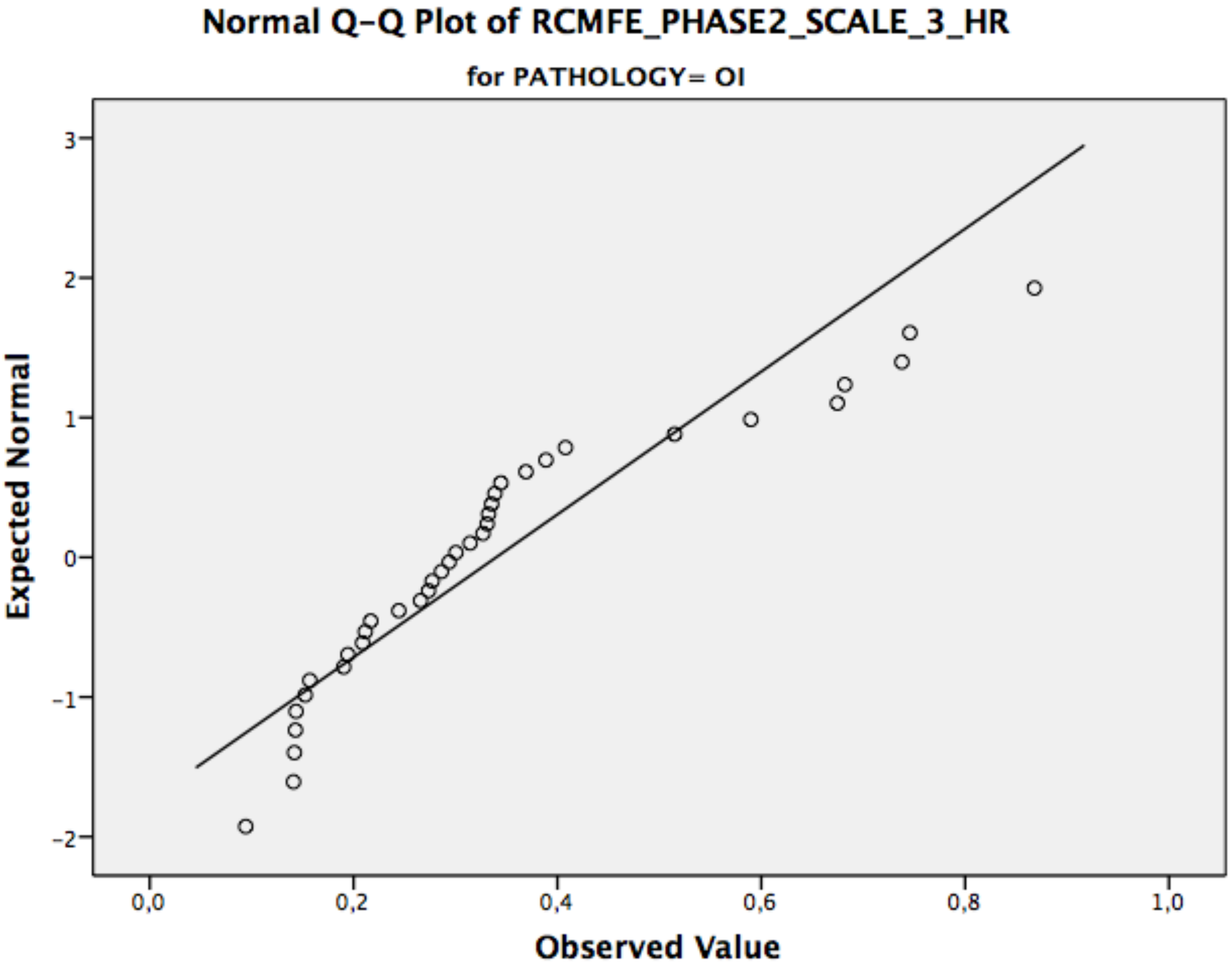

Normal Q-Q Plot of RCMFE_PHASE2_SCALE_3_HR
for PATHOLOGY= OI
Expected Normal
Observed Value
3
2
1
0
-1
-2
0,0
0,2
0,4
0,6
0,8
1,0

RCMFE_PHASE2_SCALE_4_HR

Normal Q-Q Plots

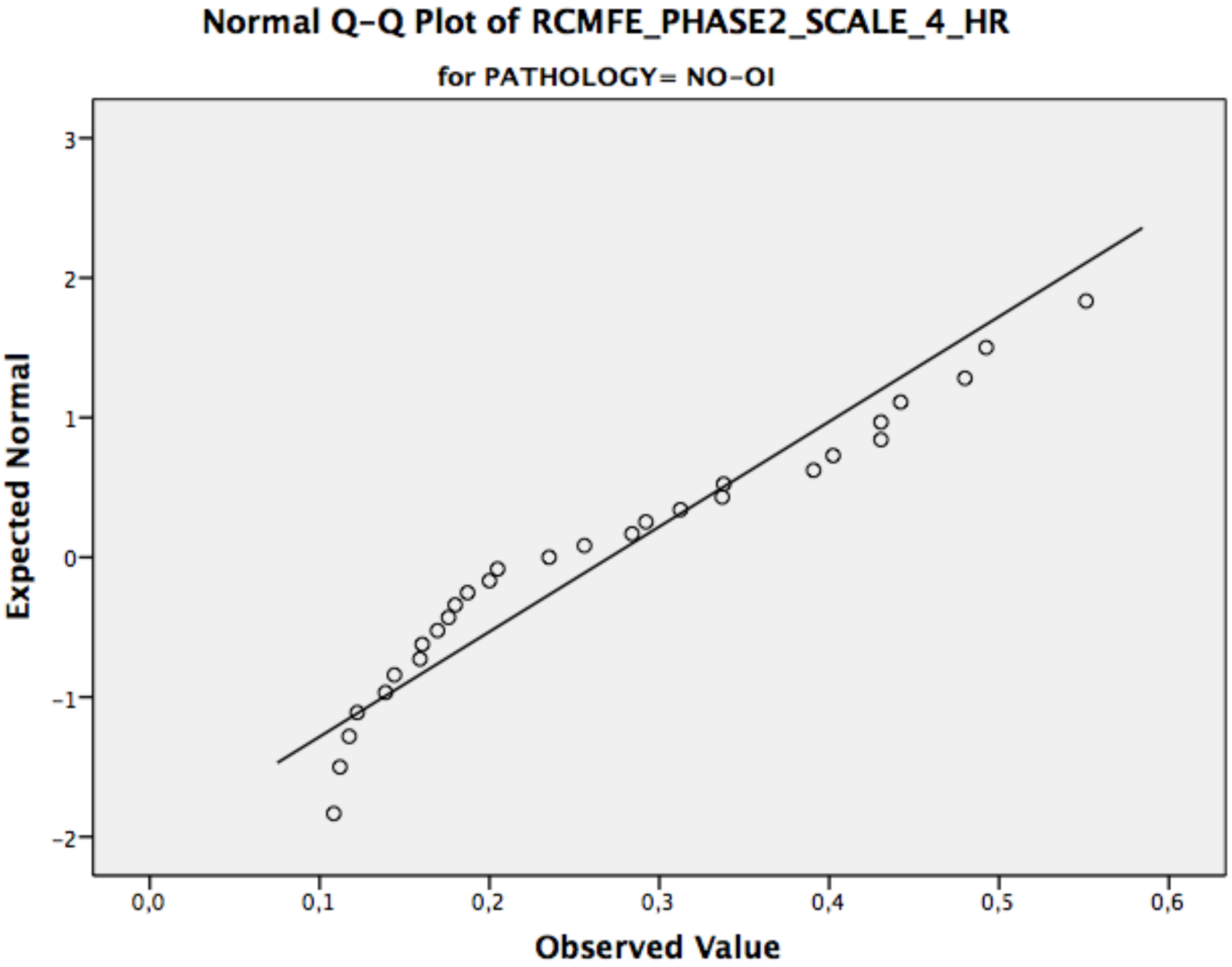

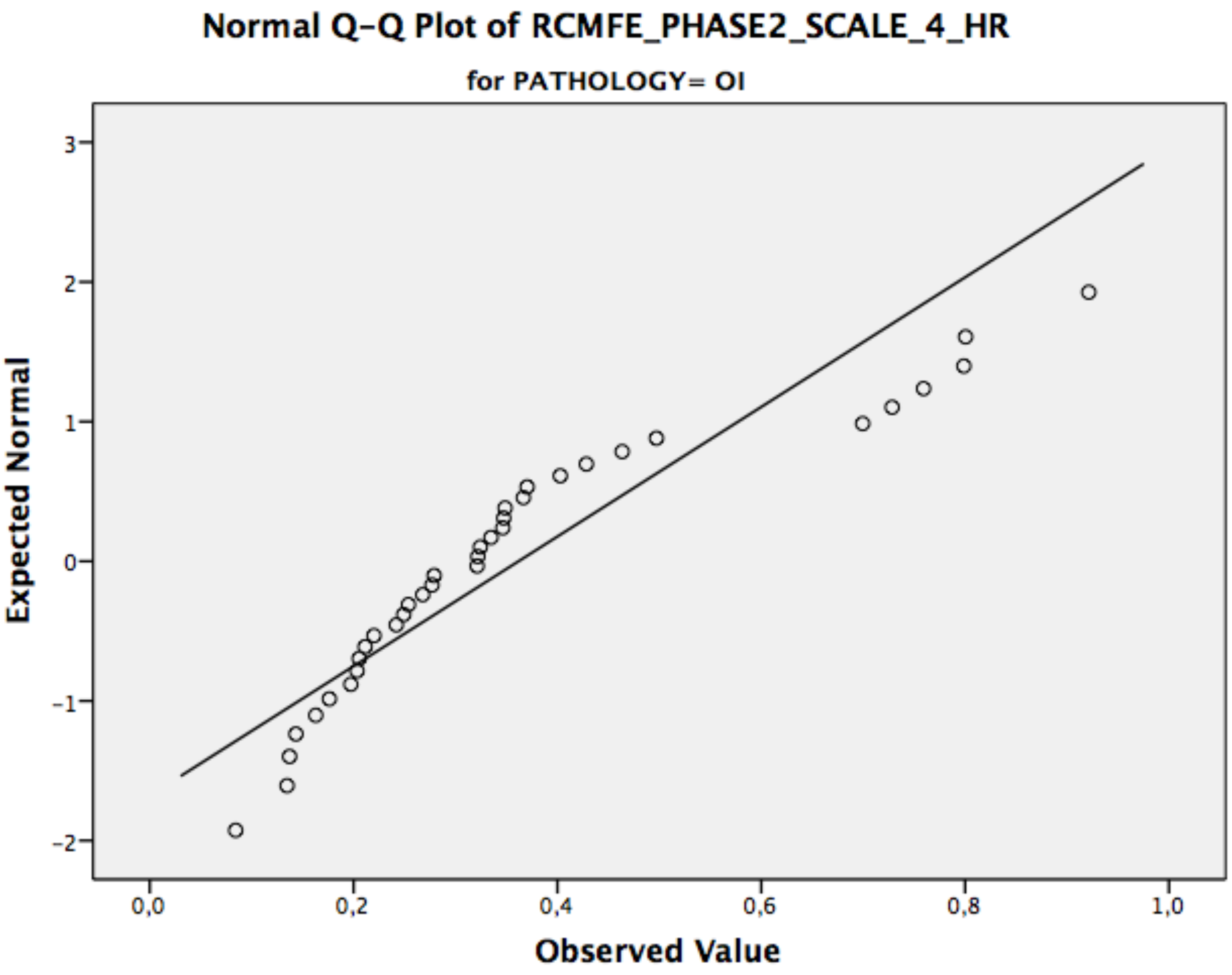
Normal Q-Q Plot of RCMFE_PHASE2_SCALE_4_HR
for PATHOLOGY= OI
Expected Normal
Observed Value
3
2
1
0
-1
-2
0,0
0,2
0,4
0,6
0,8
1,0

RCMFE_PHASE2_SCALE_5_HR

Normal Q-Q Plots

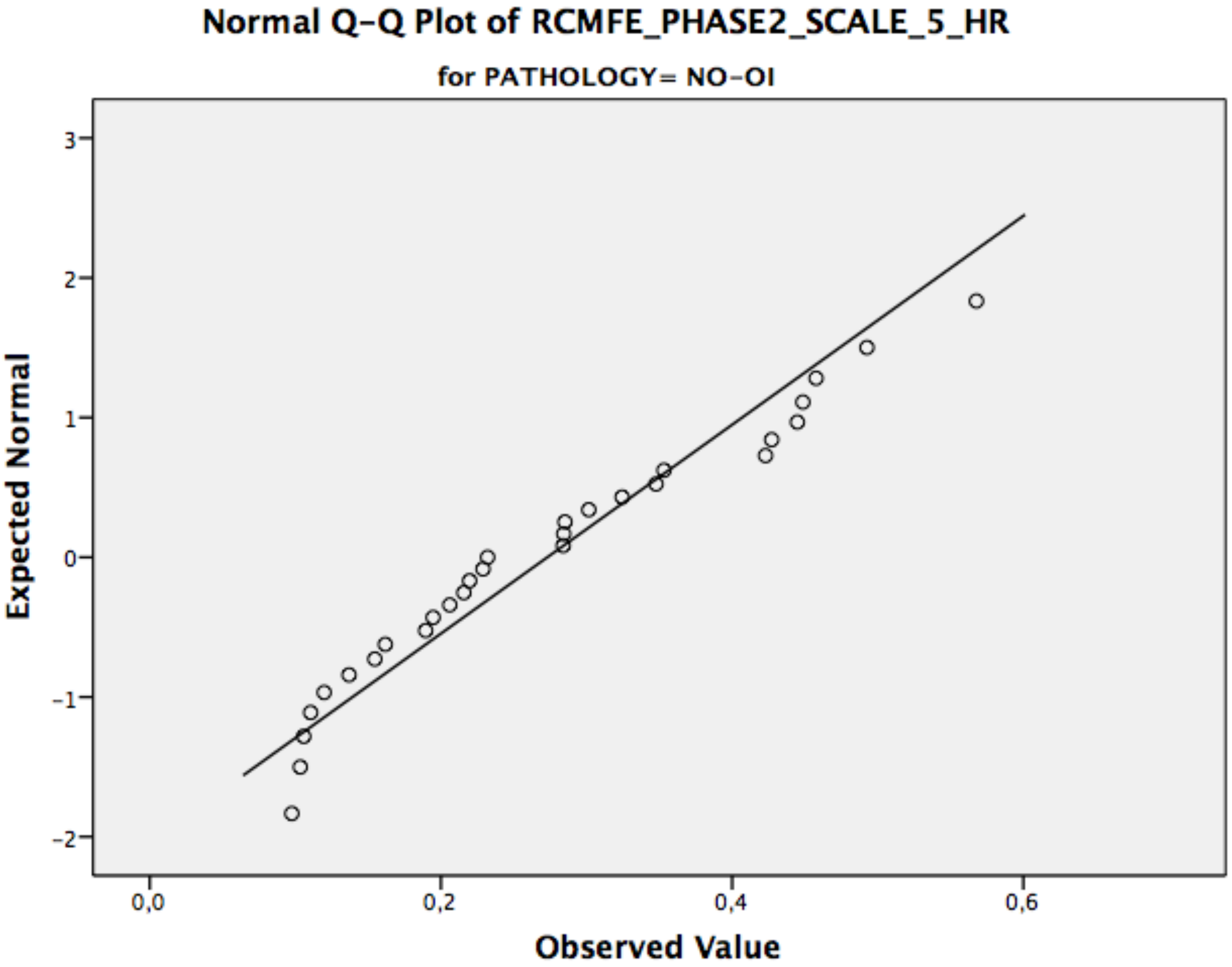

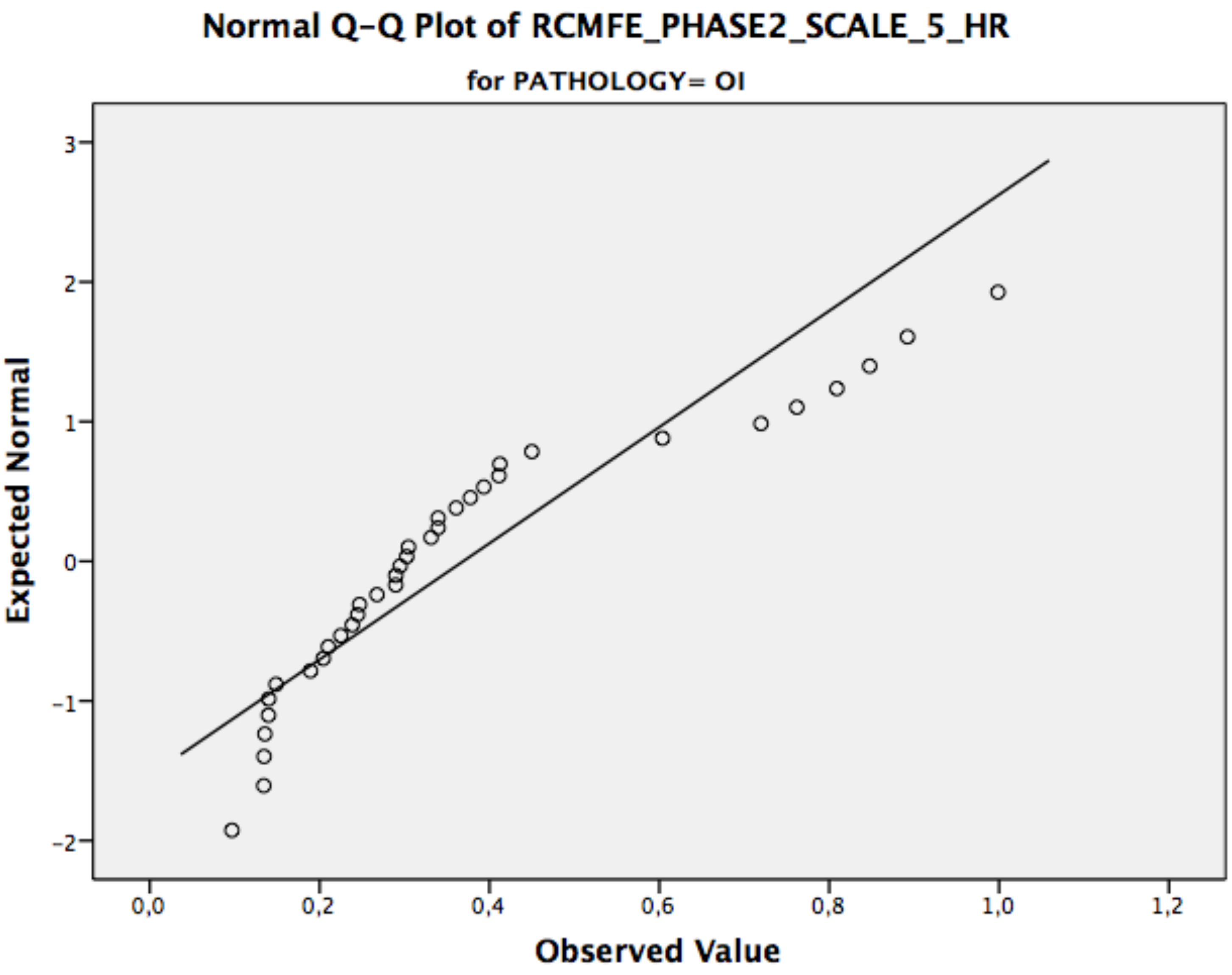

Normal Q-Q Plot of RCMFE_PHASE2_SCALE_5_HR
for PATHOLOGY= OI
Expected Normal
Observed Value
3
2
1
0
−1
−2
0,0
0,2
0,4
0,6
0,8
1,0
1,2

RCMFE_PHASE3_SCALE_1_HR

Normal Q-Q Plots

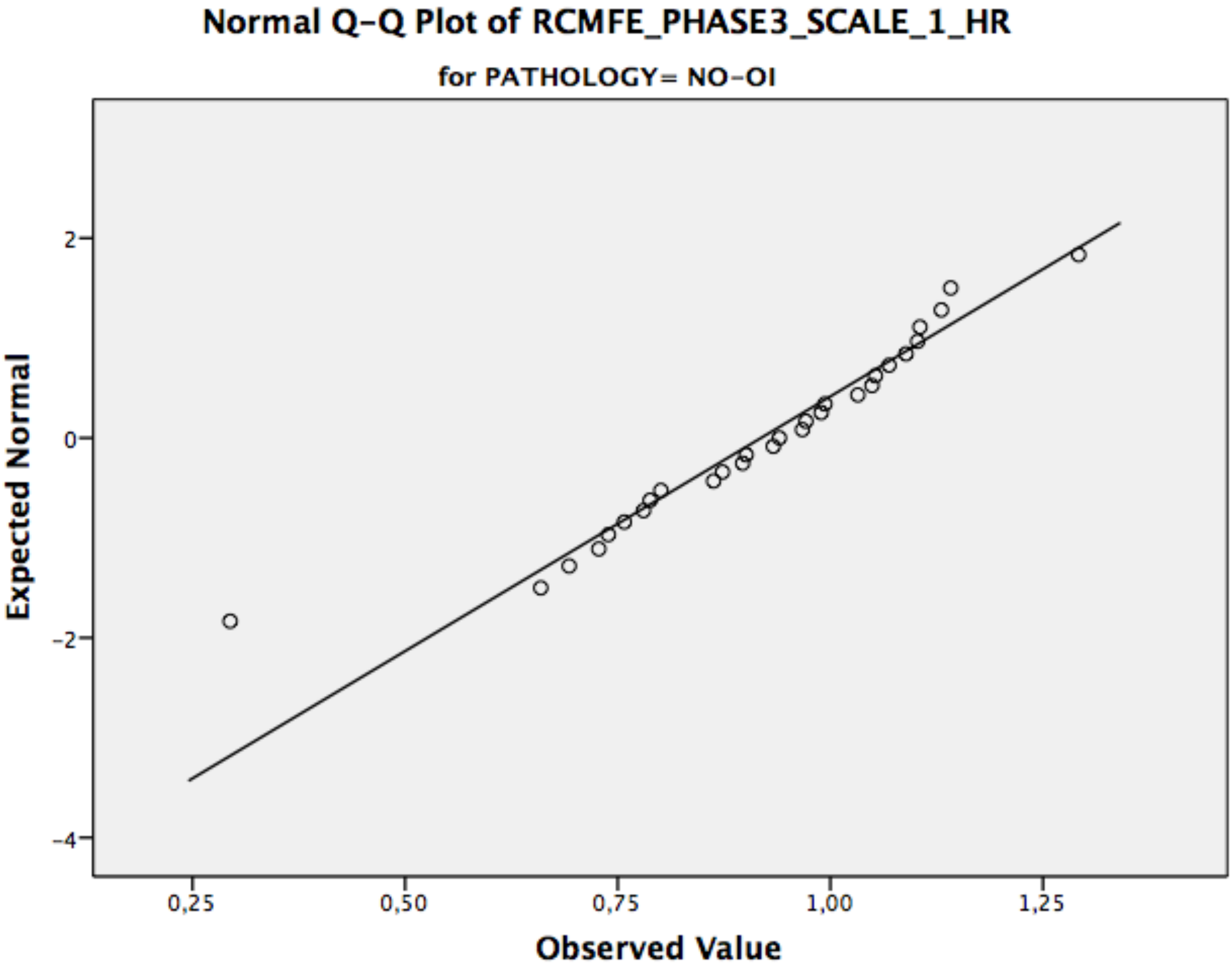

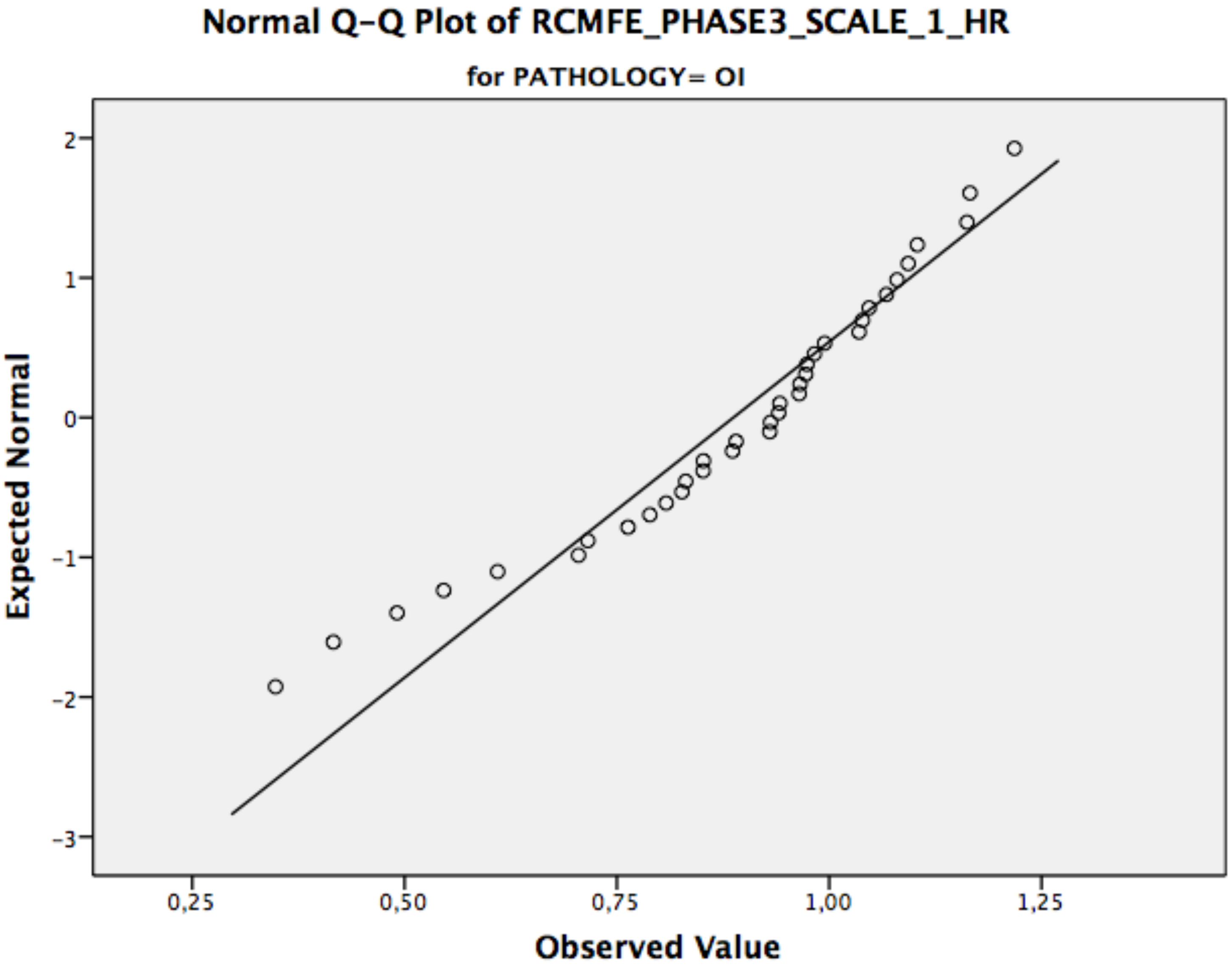
Normal Q-Q Plot of RCMFE_PHASE3_SCALE_1_HR
for PATHOLOGY= OI
Expected Normal
2
1
0
-1
-2
-3
0,25
0,50
0,75
1,00
1,25
Observed Value

RCMFE_PHASE3_SCALE_2_HR

Normal Q-Q Plots

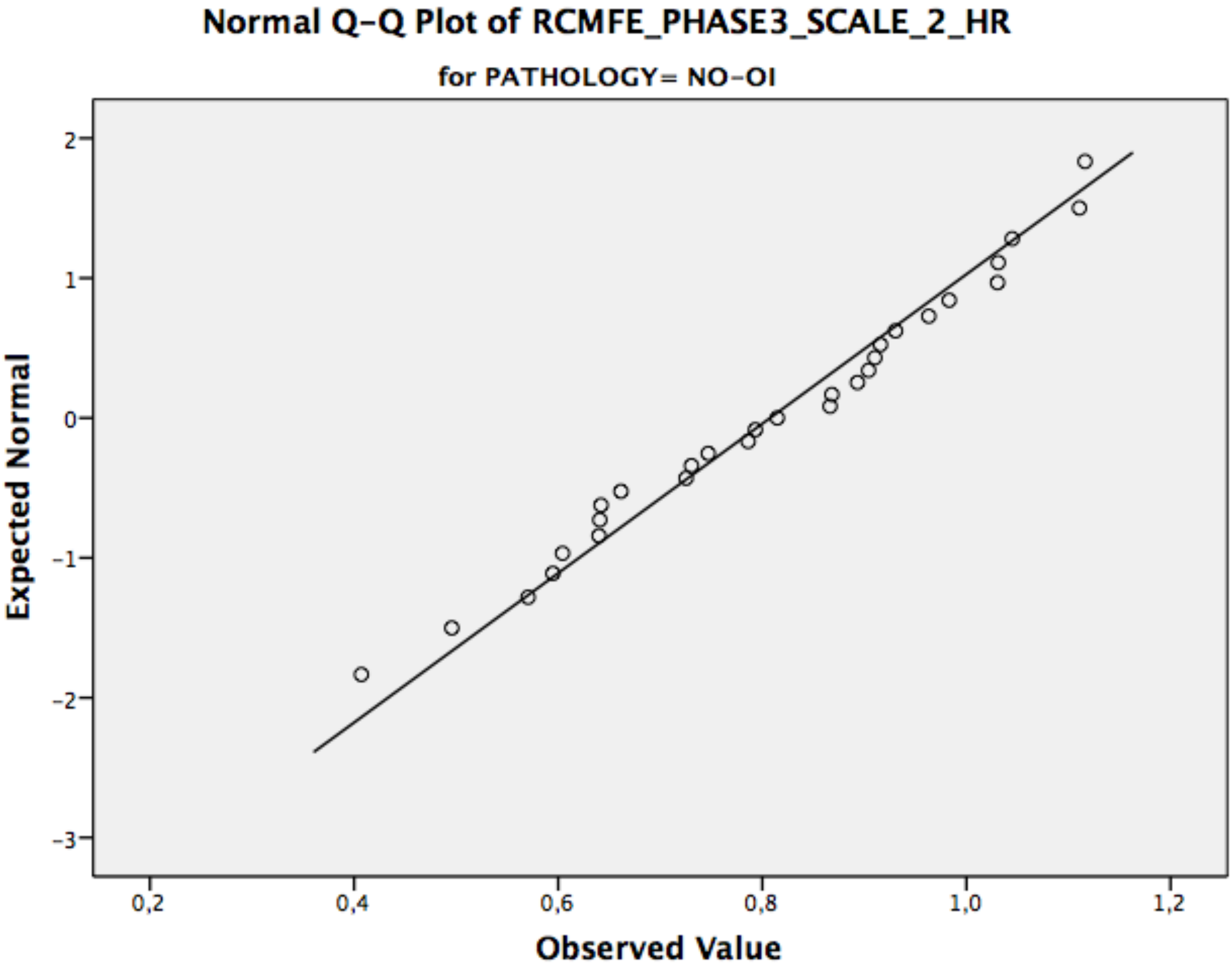

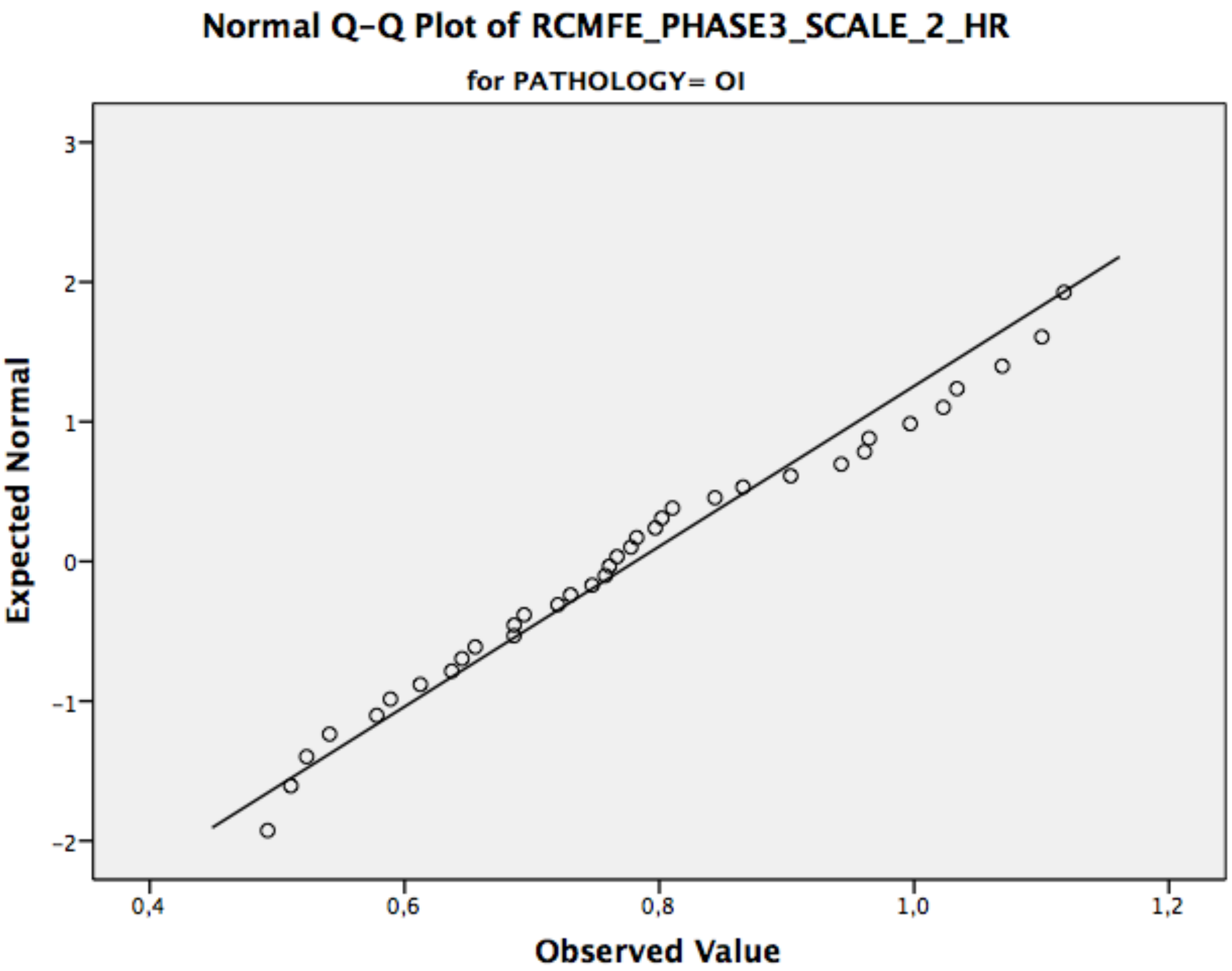
Normal Q-Q Plot of RCMFE_PHASE3_SCALE_2_HR
for PATHOLOGY= OI
Expected Normal
Observed Value
3
2
1
0
−1
−2
0,4
0,6
0,8
1,0
1,2

RCMFE_PHASE3_SCALE_3_HR

Normal Q-Q Plots

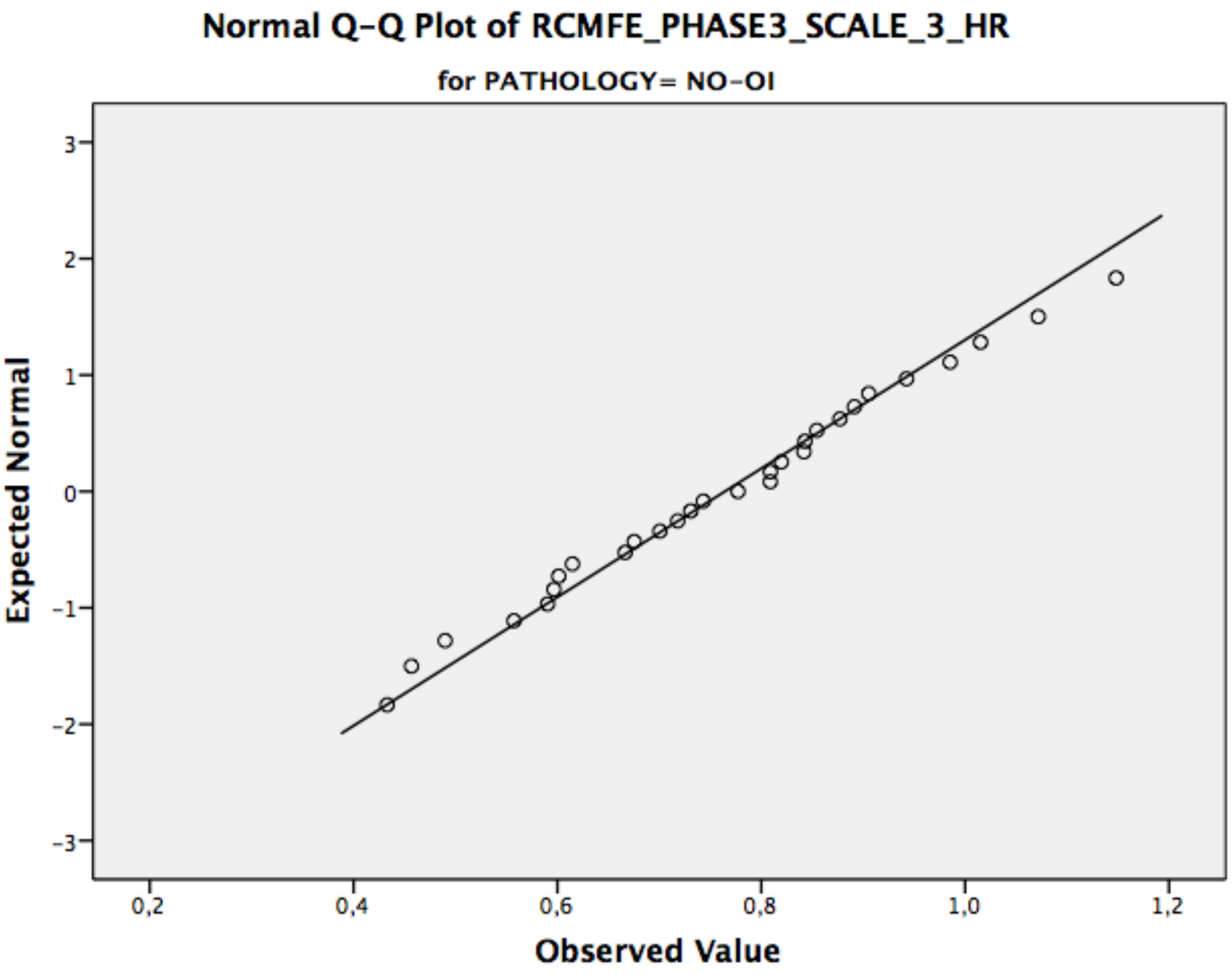

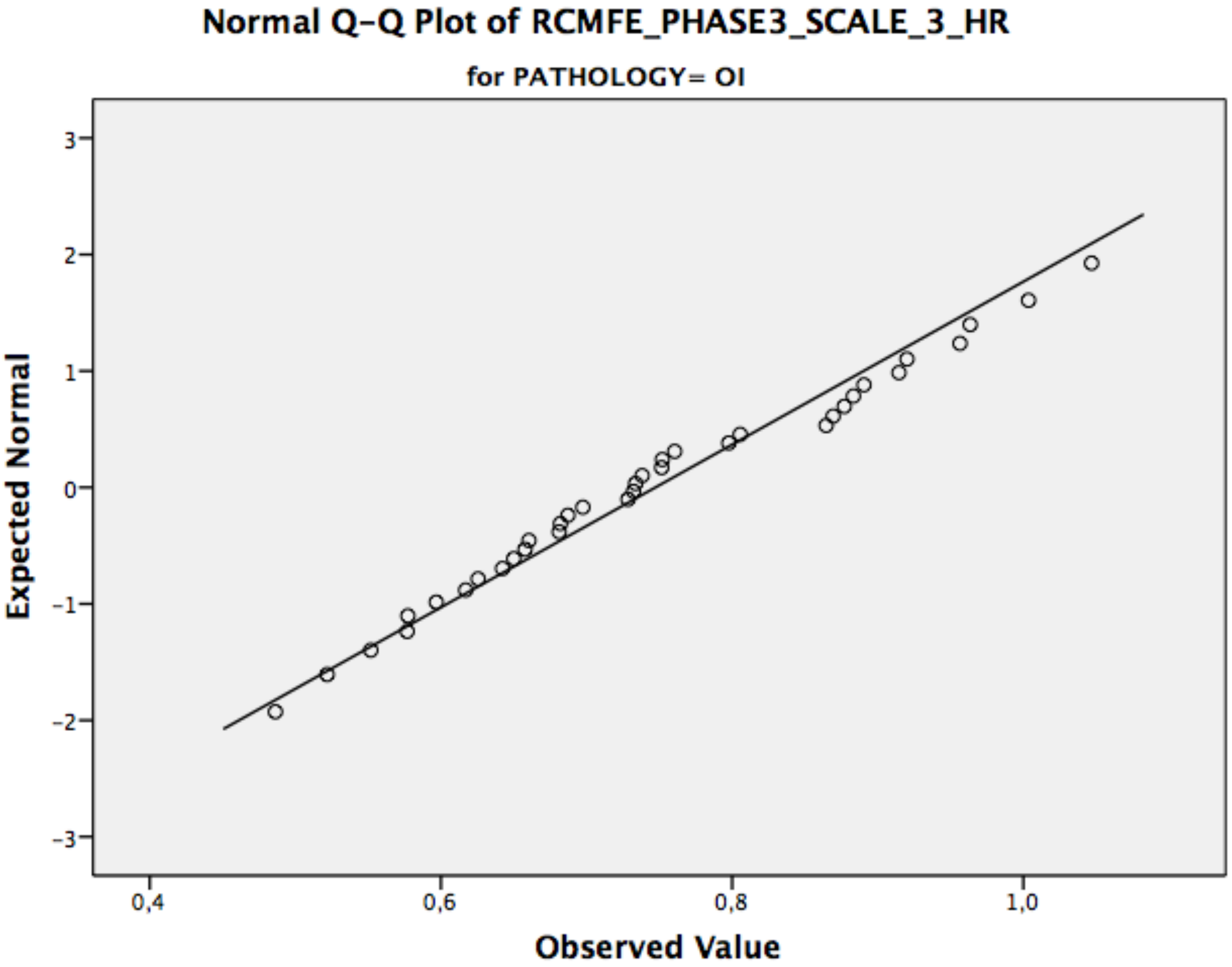
Normal Q-Q Plot of RCMFE_PHASE3_SCALE_3_HR
for PATHOLOGY= OI
Expected Normal
3
2
1
0
-1
-2
-3
0,4
0,6
0,8
1,0
Observed Value

RCMFE_PHASE3_SCALE_4_HR

Normal Q-Q Plots

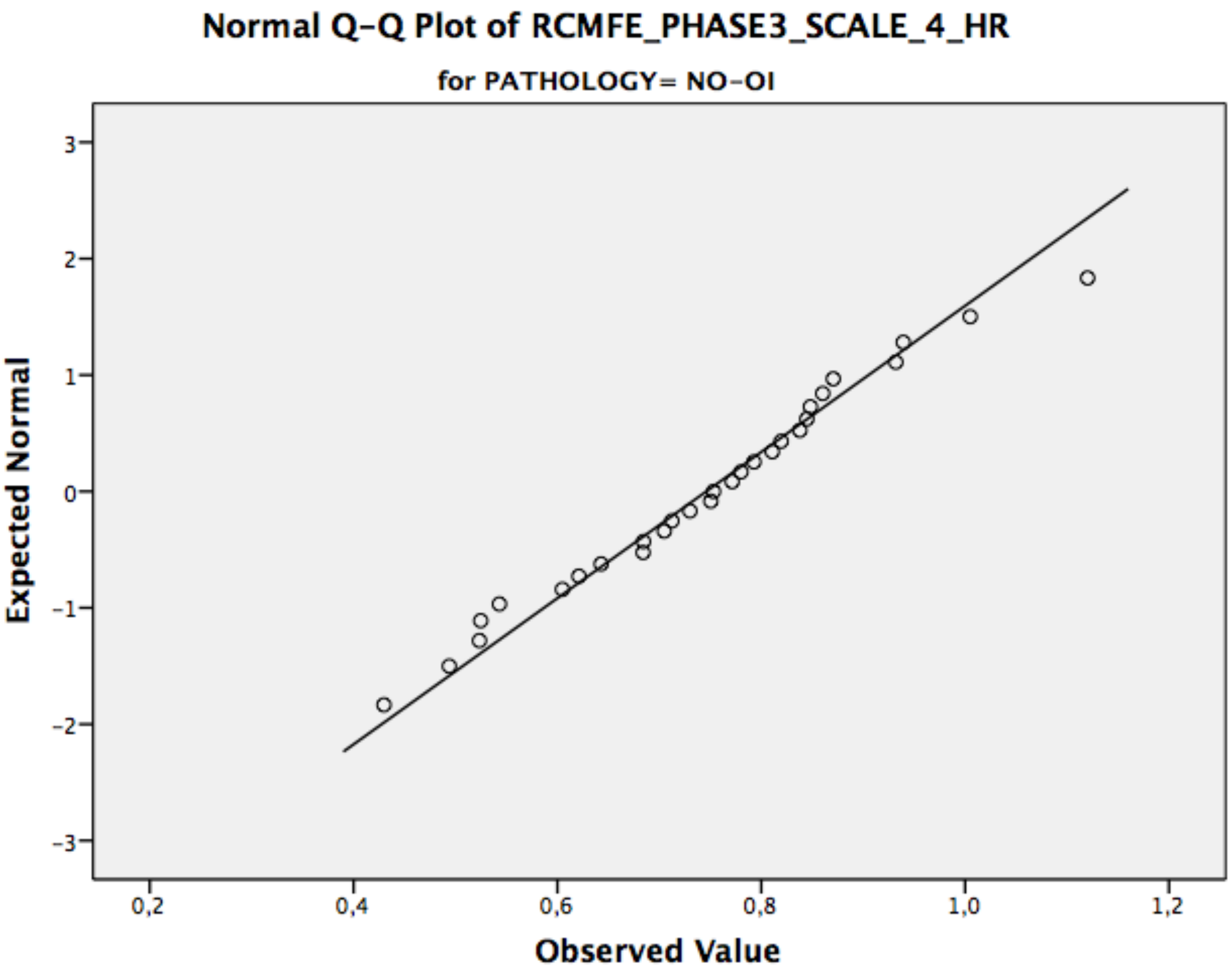

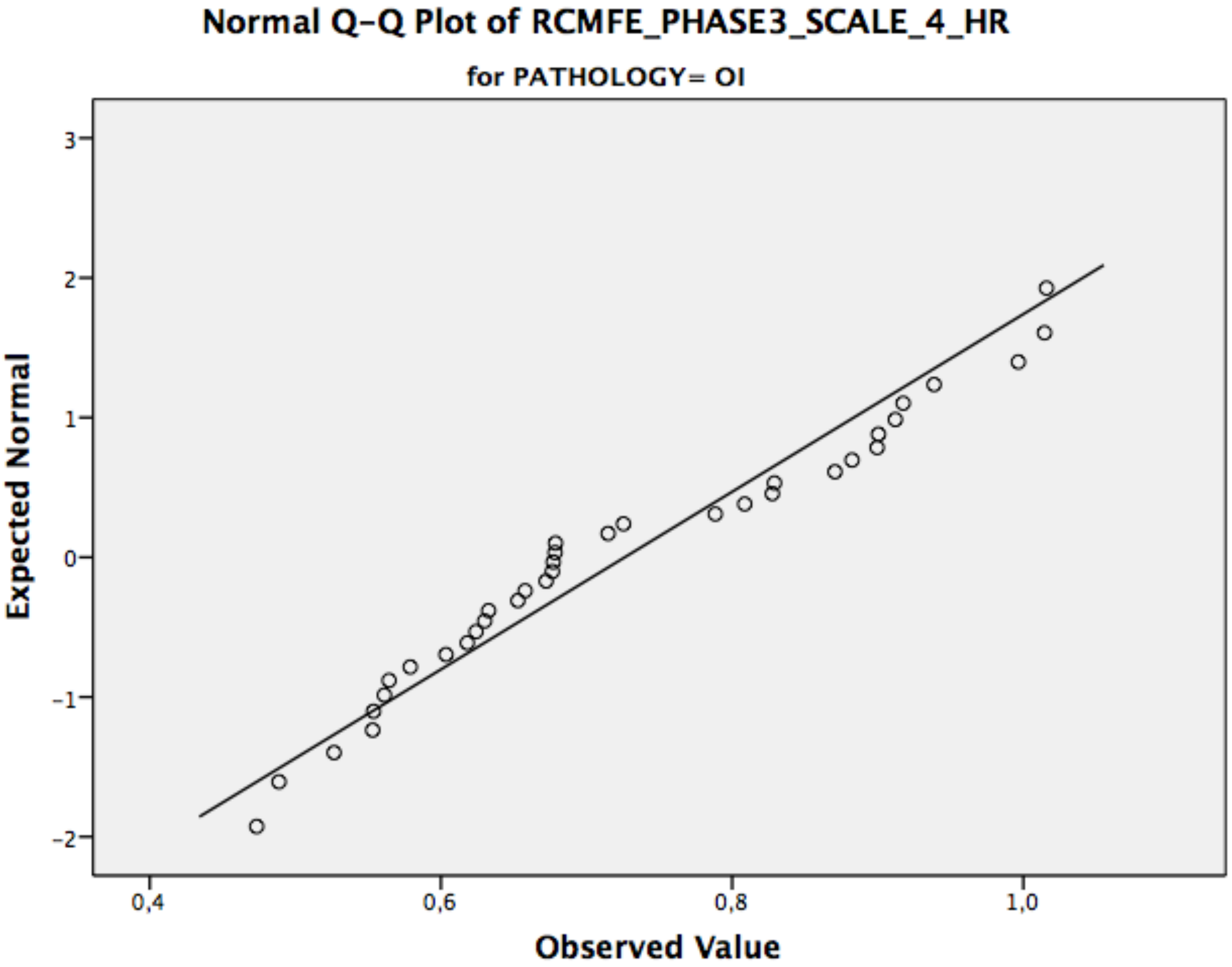
Normal Q-Q Plot of RCMFE_PHASE3_SCALE_4_HR
for PATHOLOGY= OI
Expected Normal
Observed Value
3
2
1
0
-1
-2
0,4
0,6
0,8
1,0

RCMFE_PHASE3_SCALE_5_HR

Normal Q-Q Plots

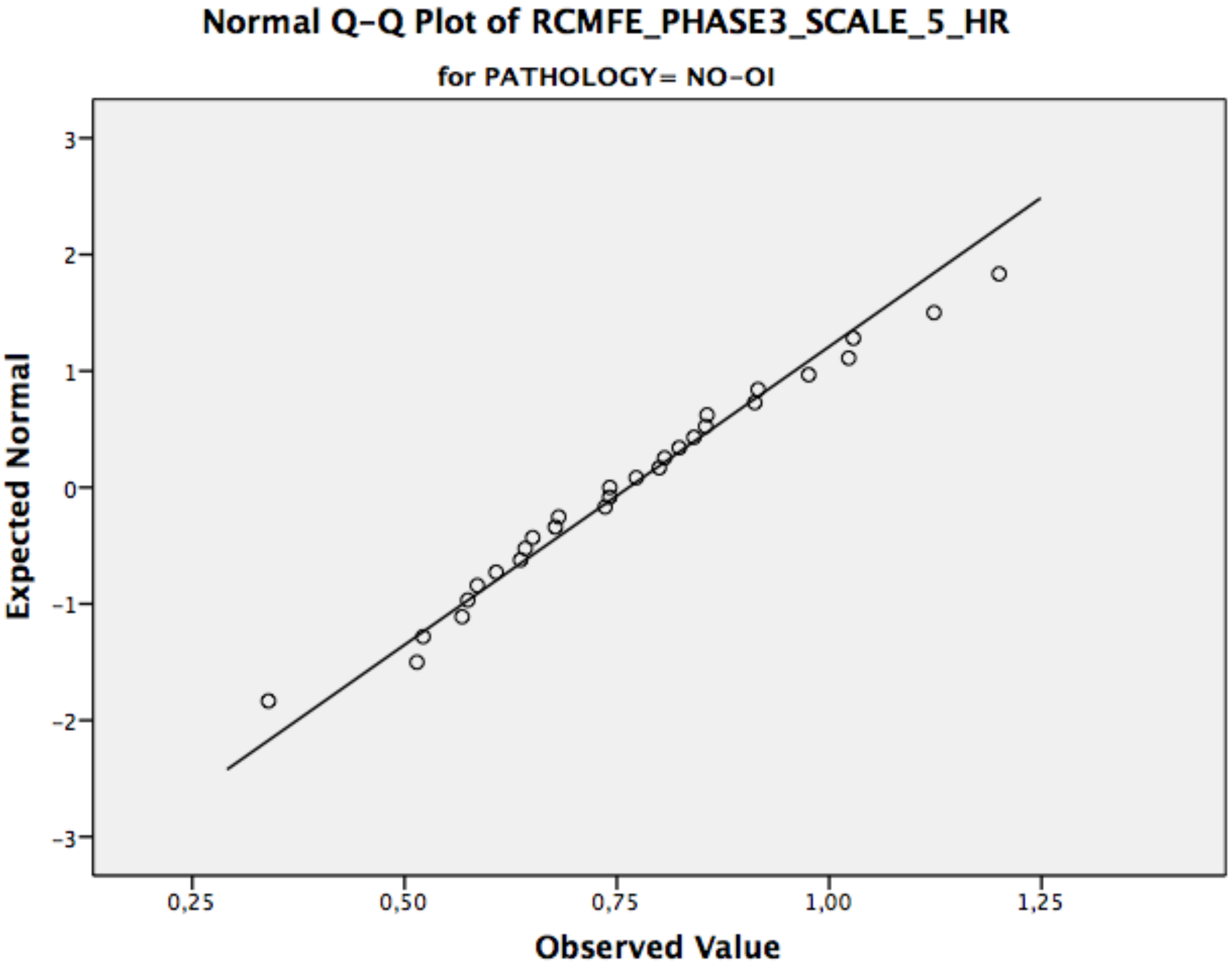

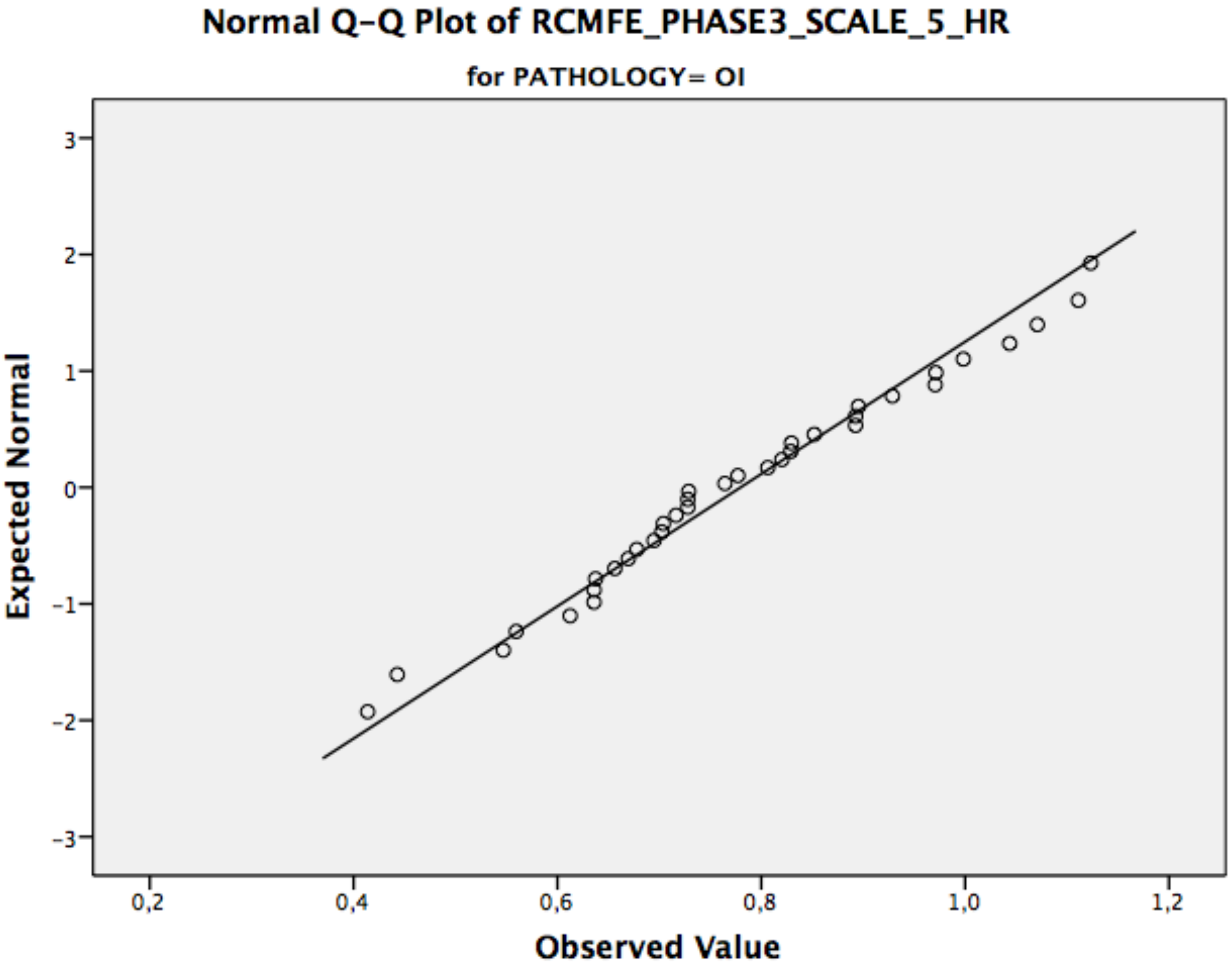
Normal Q-Q Plot of RCMFE_PHASE3_SCALE_5_HR
for PATHOLOGY= OI
Expected Normal
Observed Value
3
2
1
0
-1
-2
-3
0,2
0,4
0,6
0,8
1,0
1,2

RCMFE_PHASE4_SCALE_1_HR

Normal Q-Q Plots

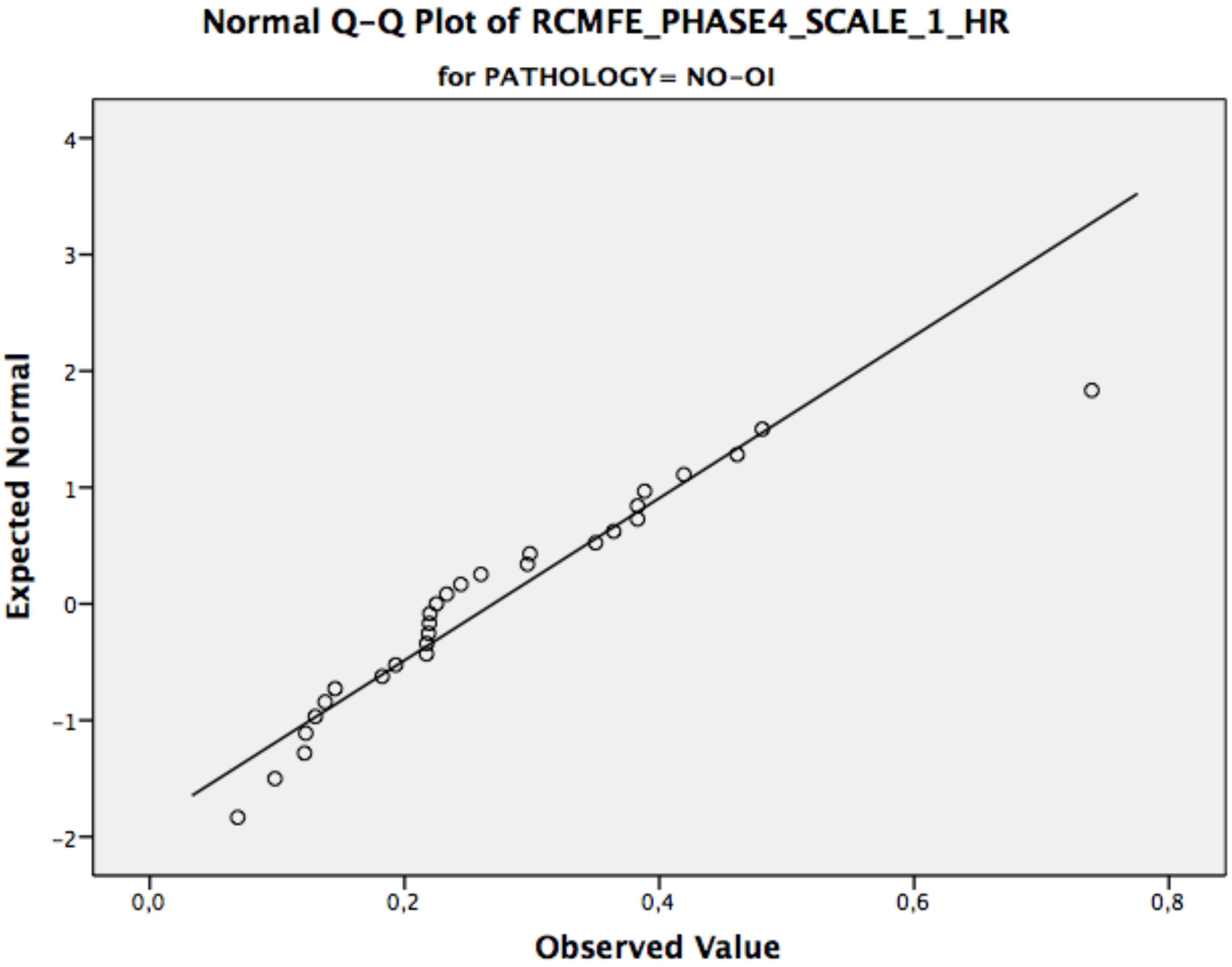

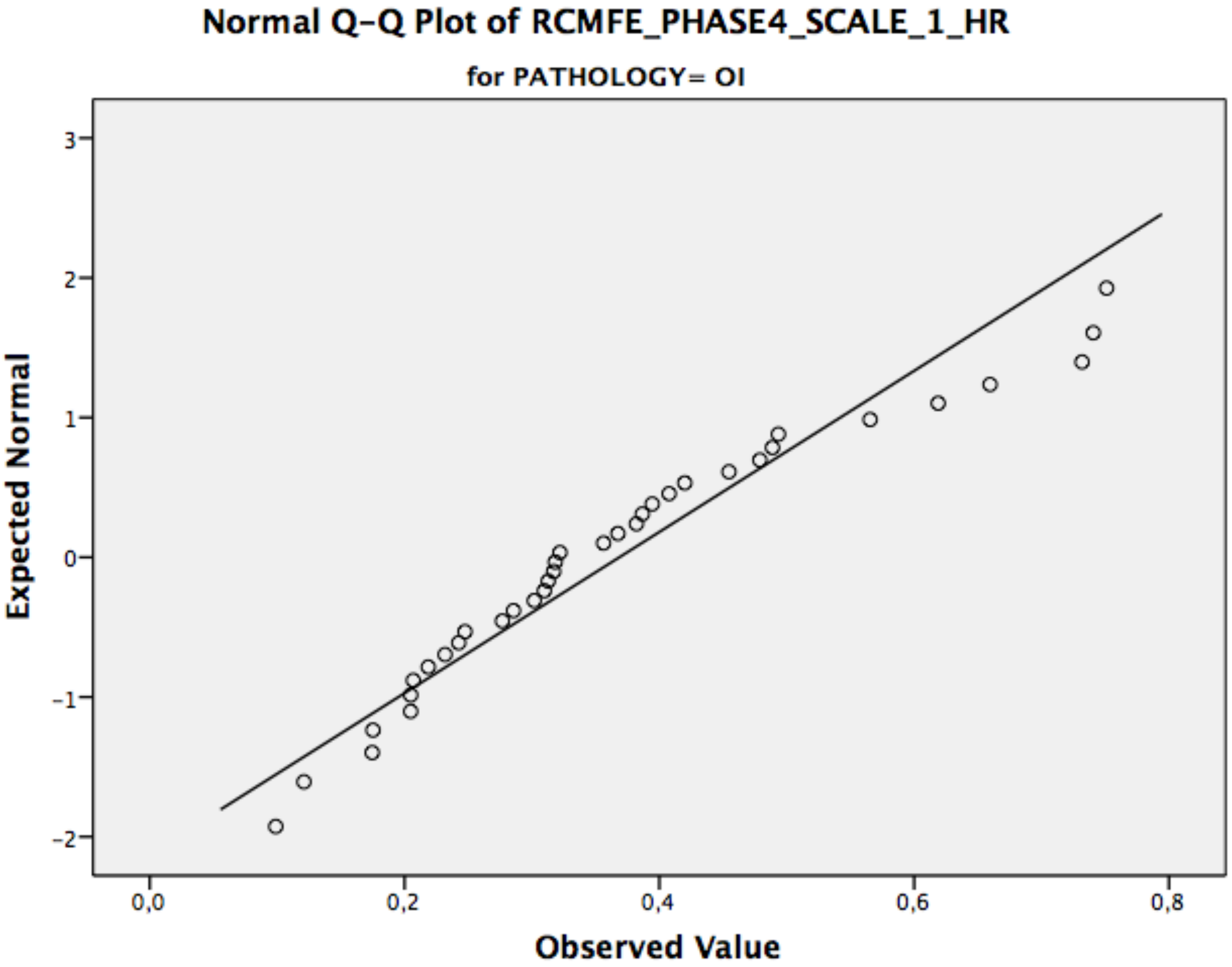

Normal Q-Q Plot of RCMFE_PHASE4_SCALE_1_HR
for PATHOLOGY= OI
Expected Normal
Observed Value
3
2
1
0
-1
-2
0,0
0,2
0,4
0,6
0,8

RCMFE_PHASE4_SCALE_2_HR

Normal Q-Q Plots

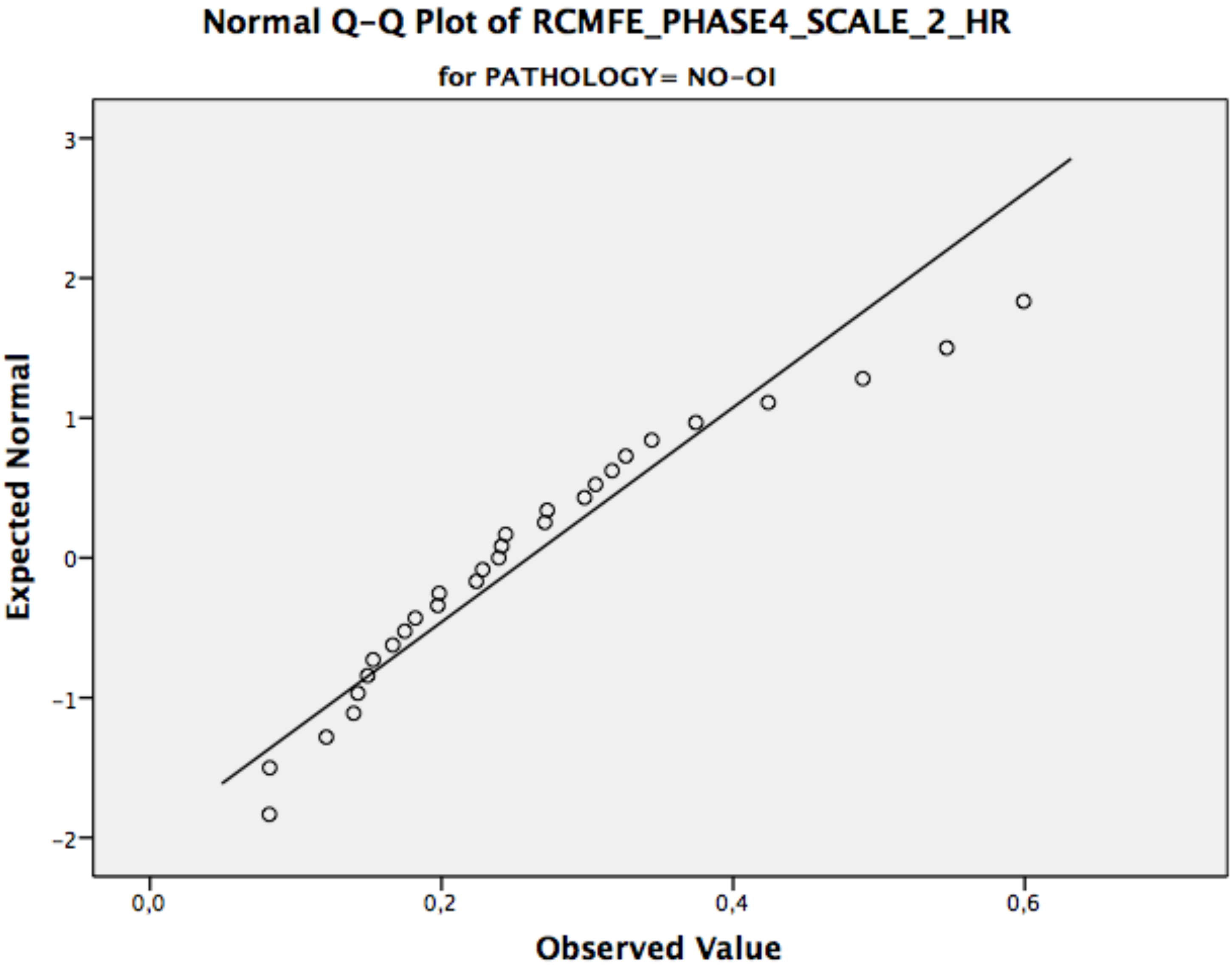

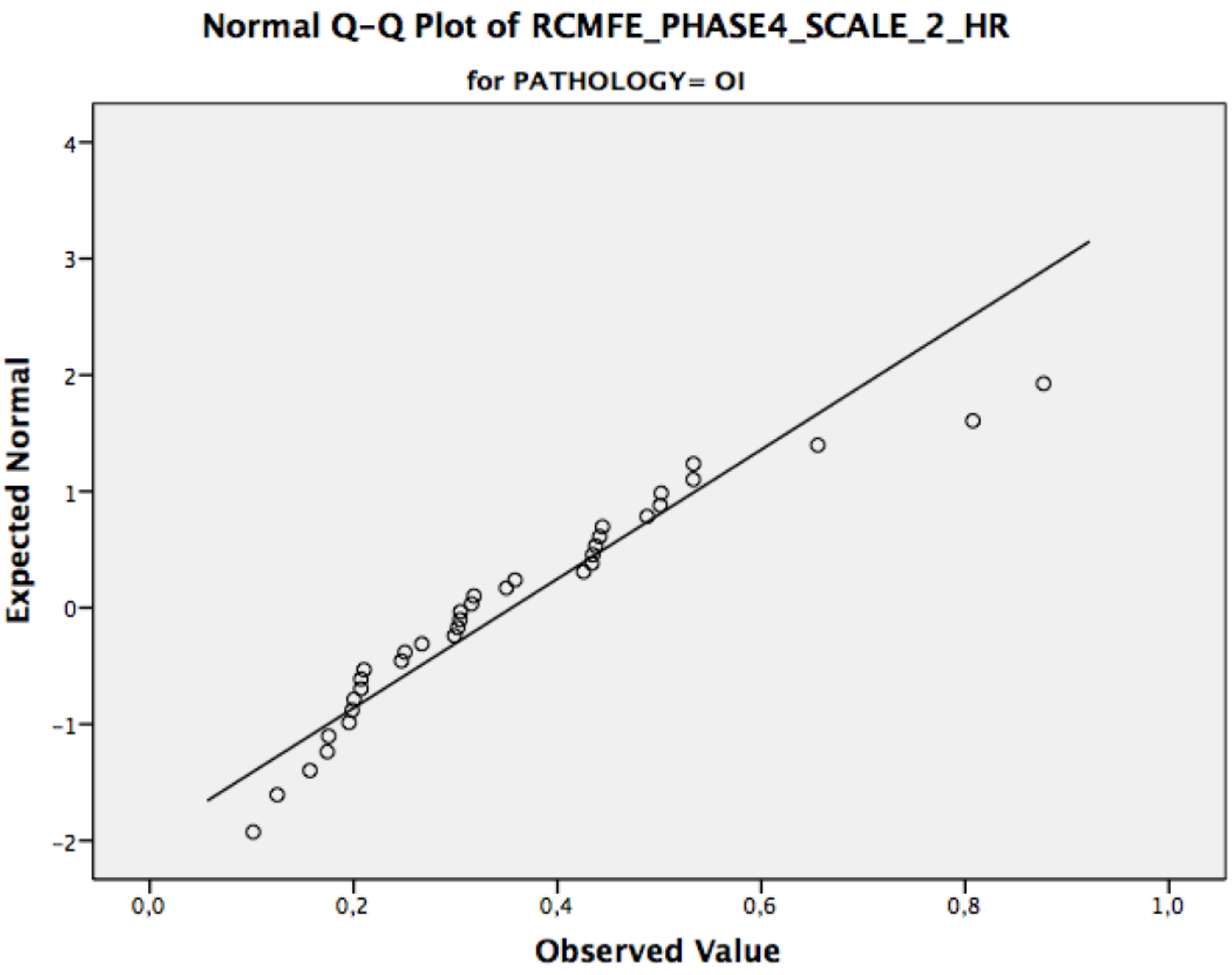

Normal Q-Q Plot of RCMFE_PHASE4_SCALE_2_HR
for PATHOLOGY= OI
Expected Normal
Observed Value
4
3
2
1
0
-1
-2
0,0
0,2
0,4
0,6
0,8
1,0

RCMFE_PHASE4_SCALE_3_HR

Normal Q-Q Plots

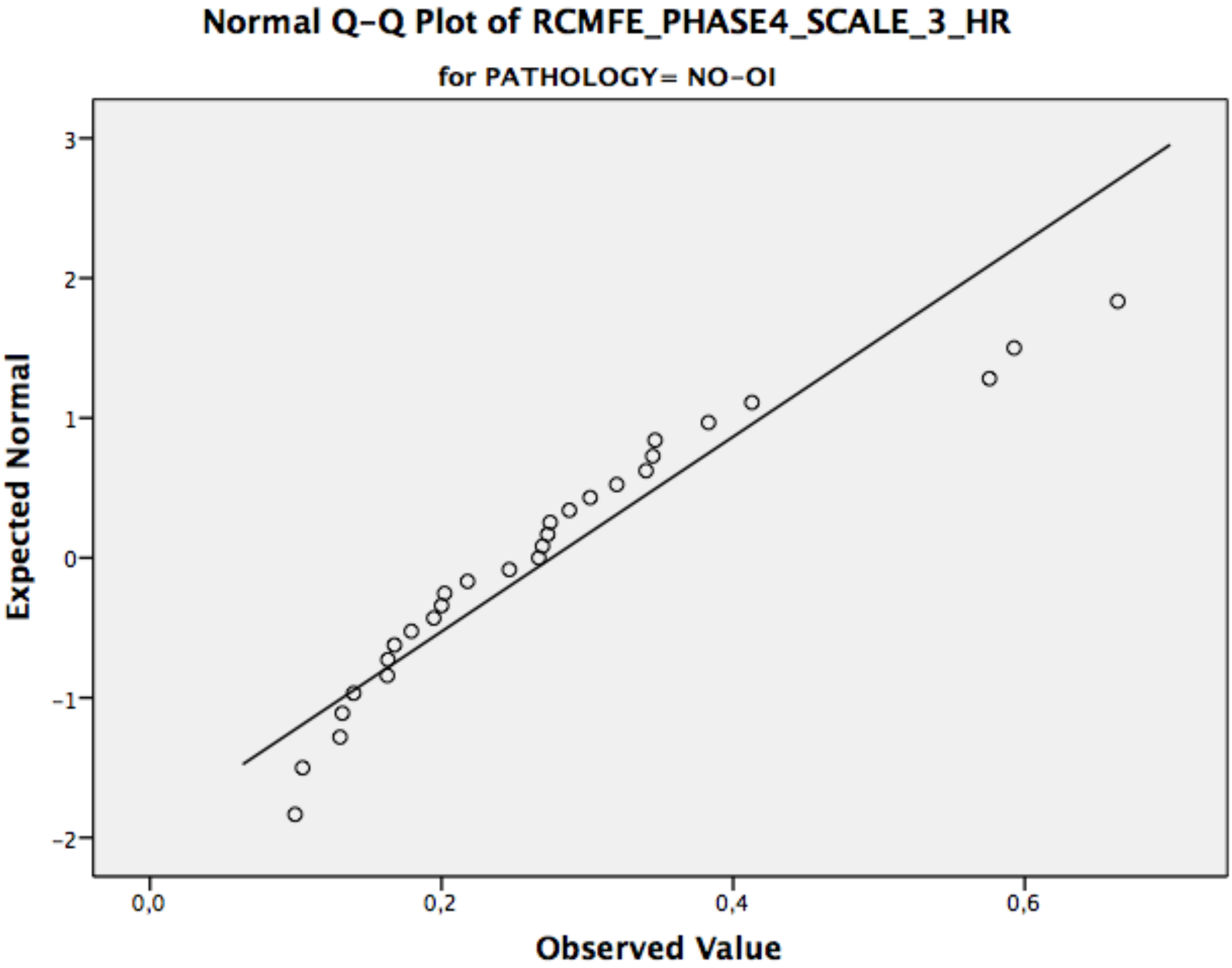

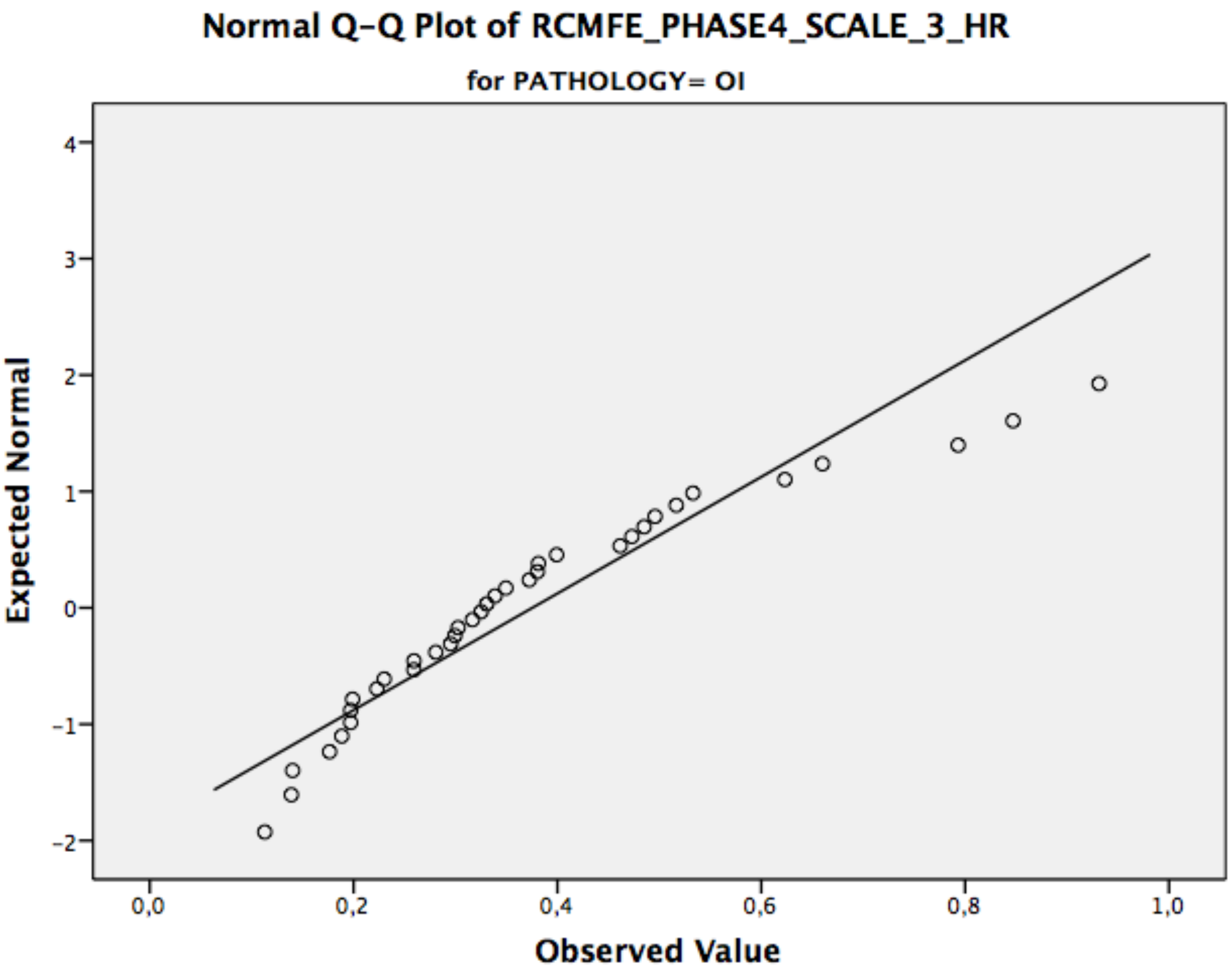
Normal Q-Q Plot of RCMFE_PHASE4_SCALE_3_HR
for PATHOLOGY= OI
Expected Normal
4
3
2
1
0
-1
-2
0,0
0,2
0,4
0,6
0,8
1,0
Observed Value

RCMFE_PHASE4_SCALE_4_HR

Normal Q-Q Plots

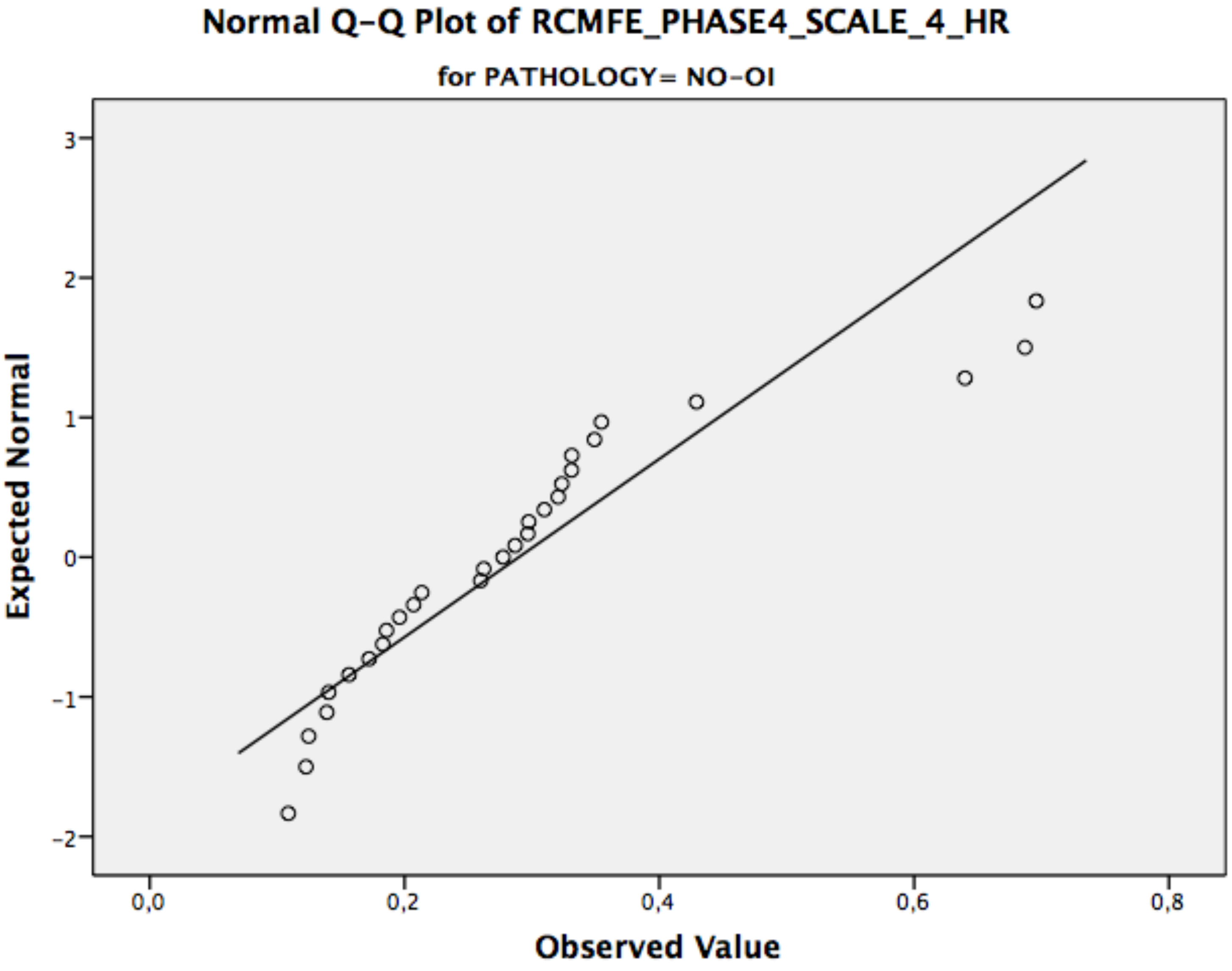

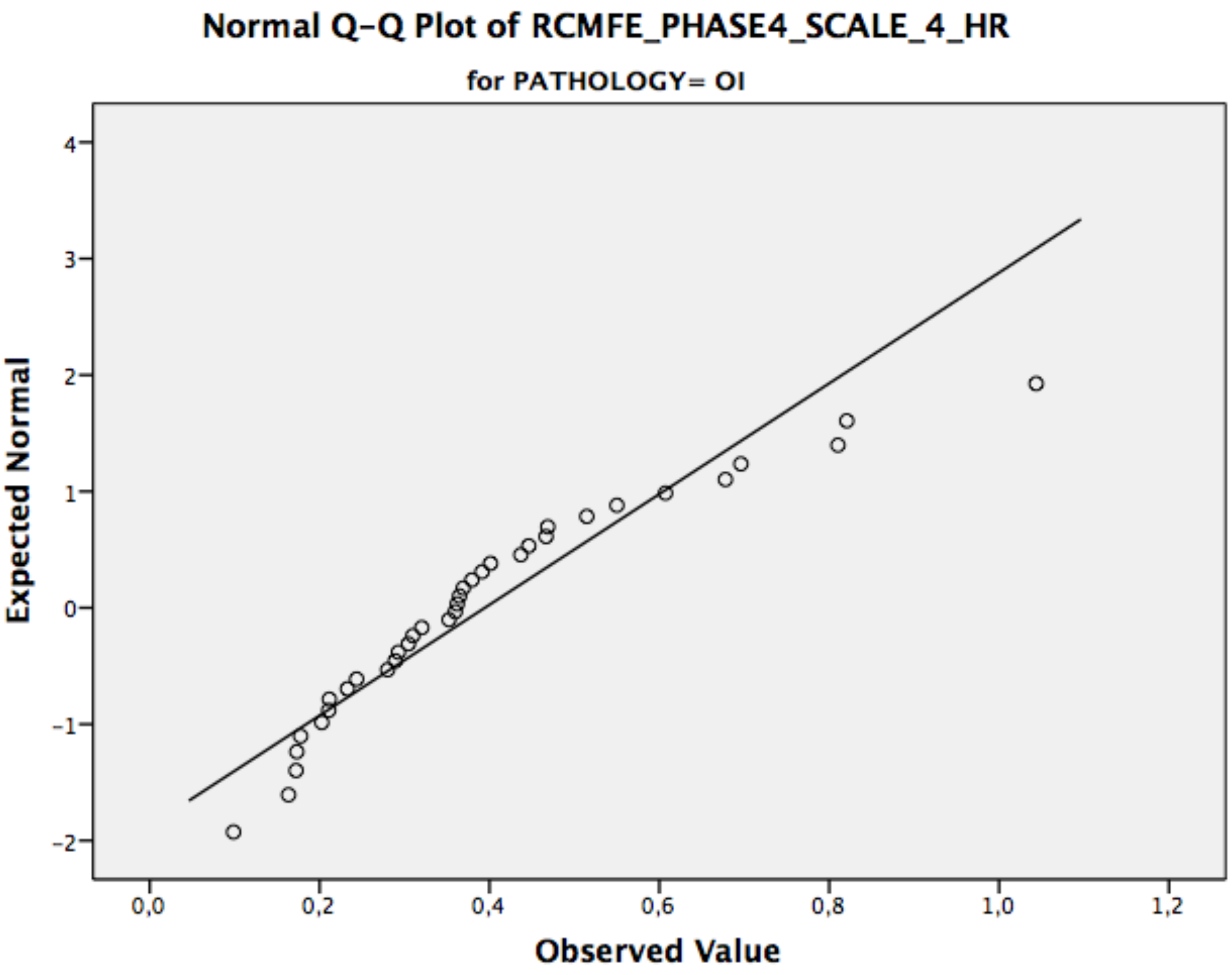
Normal Q-Q Plot of RCMFE_PHASE4_SCALE_4_HR
for PATHOLOGY= OI
Expected Normal
Observed Value
4
3
2
1
0
−1
−2
0,0
0,2
0,4
0,6
0,8
1,0
1,2

RCMFE_PHASE4_SCALE_5_HR

Normal Q-Q Plots

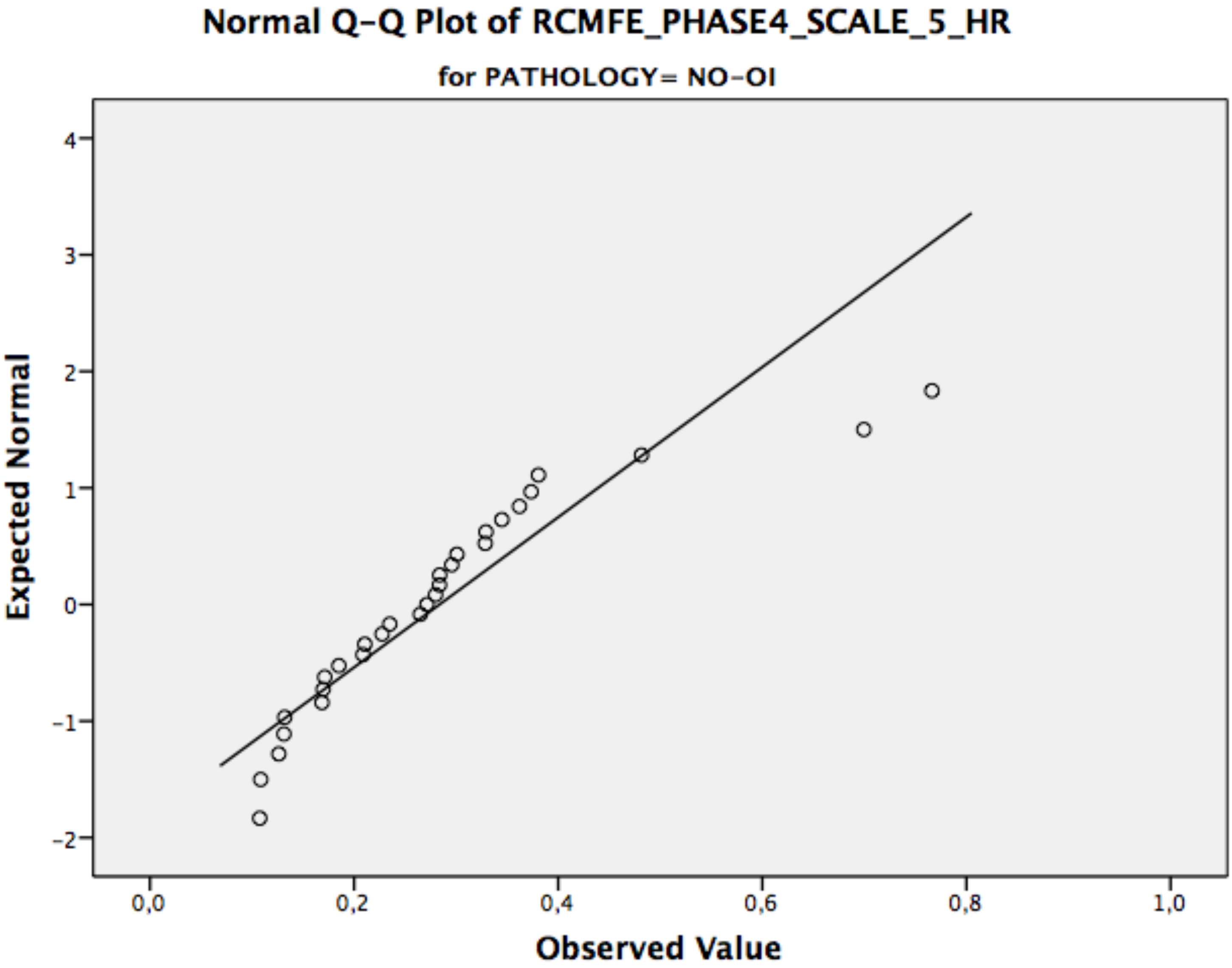

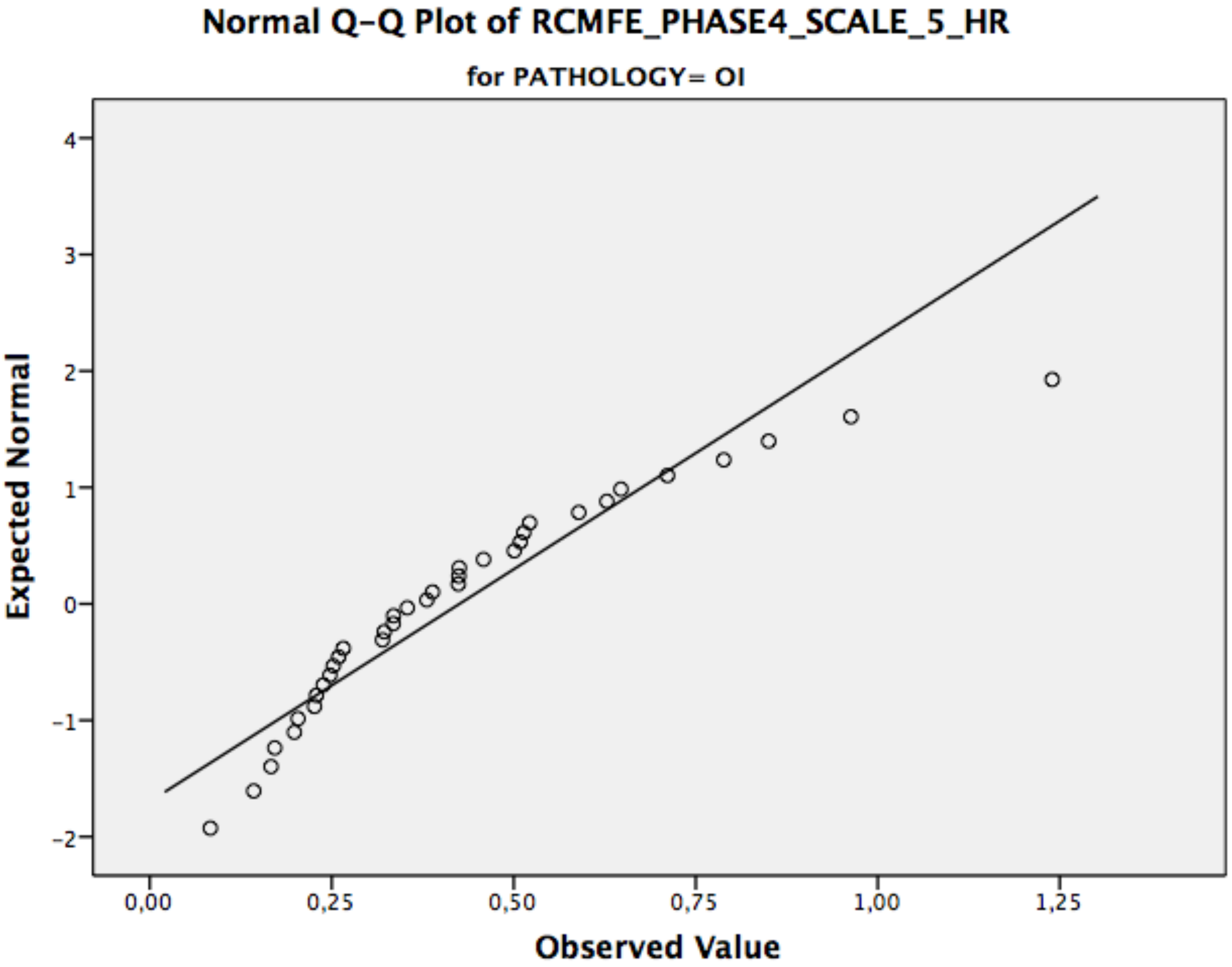

Normal Q-Q Plot of RCMFE_PHASE4_SCALE_5_HR
for PATHOLOGY= OI
Expected Normal
4
3
2
1
0
-1
-2
0,00
0,25
0,50
0,75
1,00
1,25
Observed Value

RCMFE_PHASE5_SCALE_1_HR

Normal Q-Q Plots

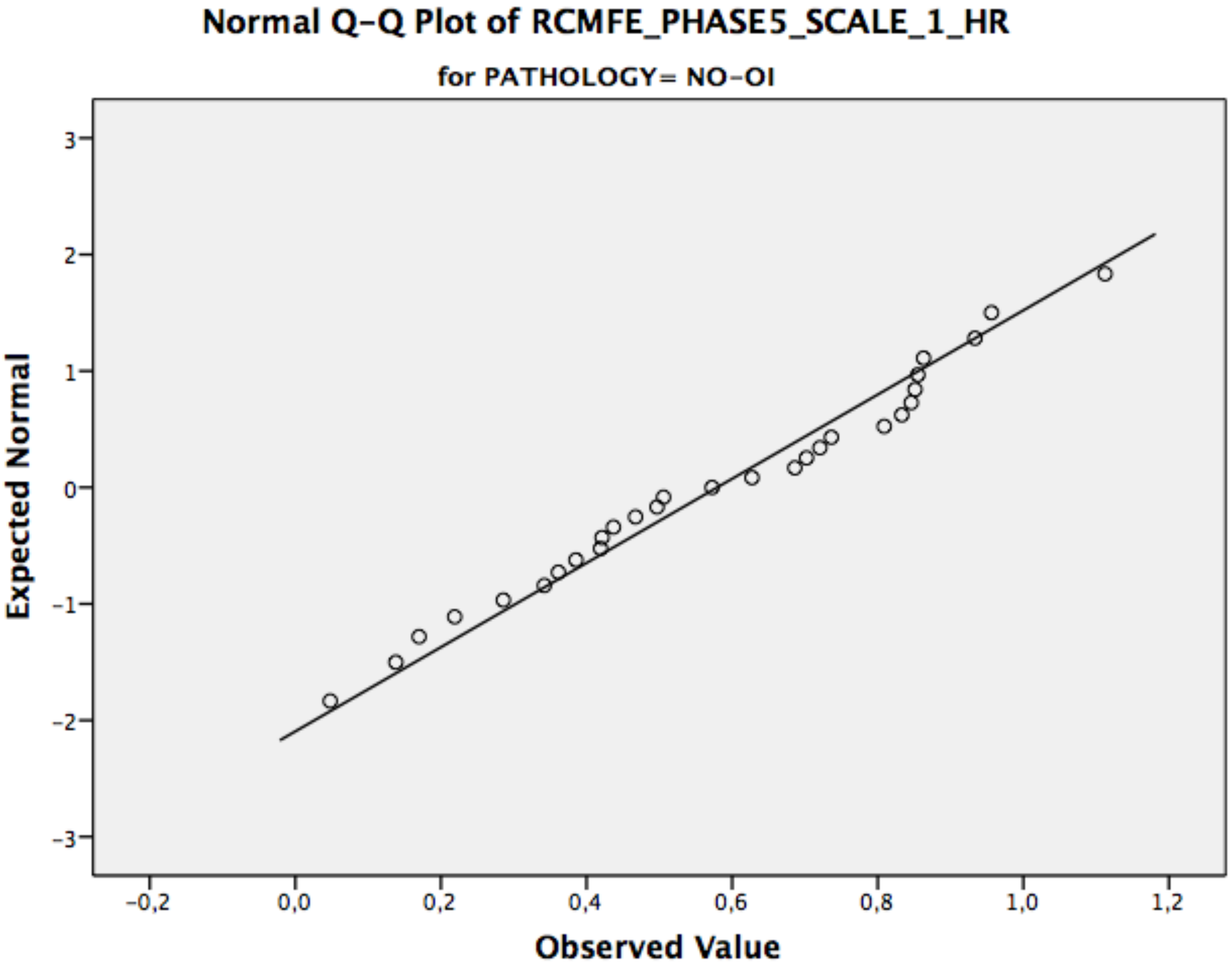

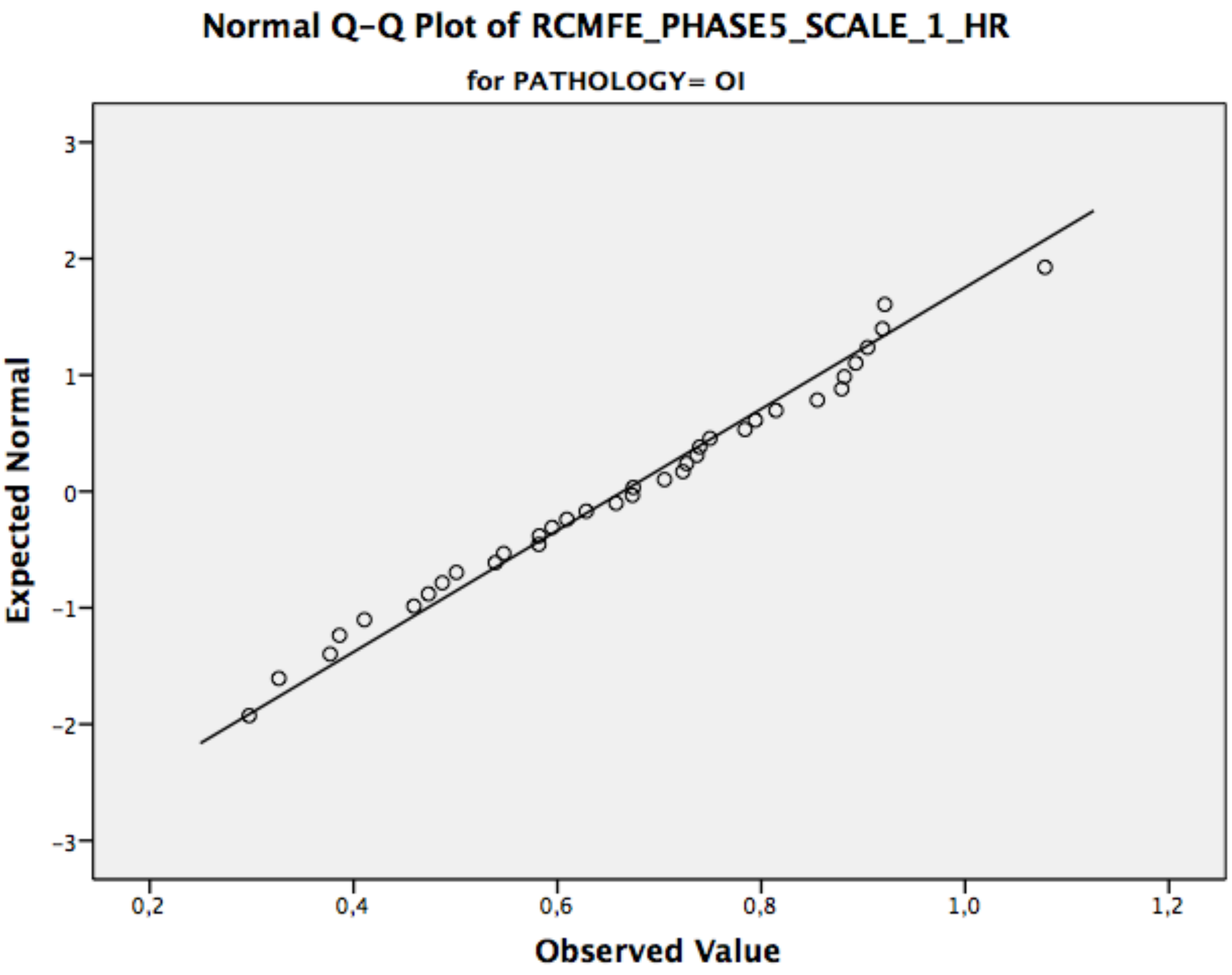
Normal Q-Q Plot of RCMFE_PHASE5_SCALE_1_HR
for PATHOLOGY= OI
Expected Normal
3
2
1
0
-1
-2
-3
0,2
0,4
0,6
0,8
1,0
1,2
Observed Value

RCMFE_PHASE5_SCALE_2_HR

Normal Q-Q Plots

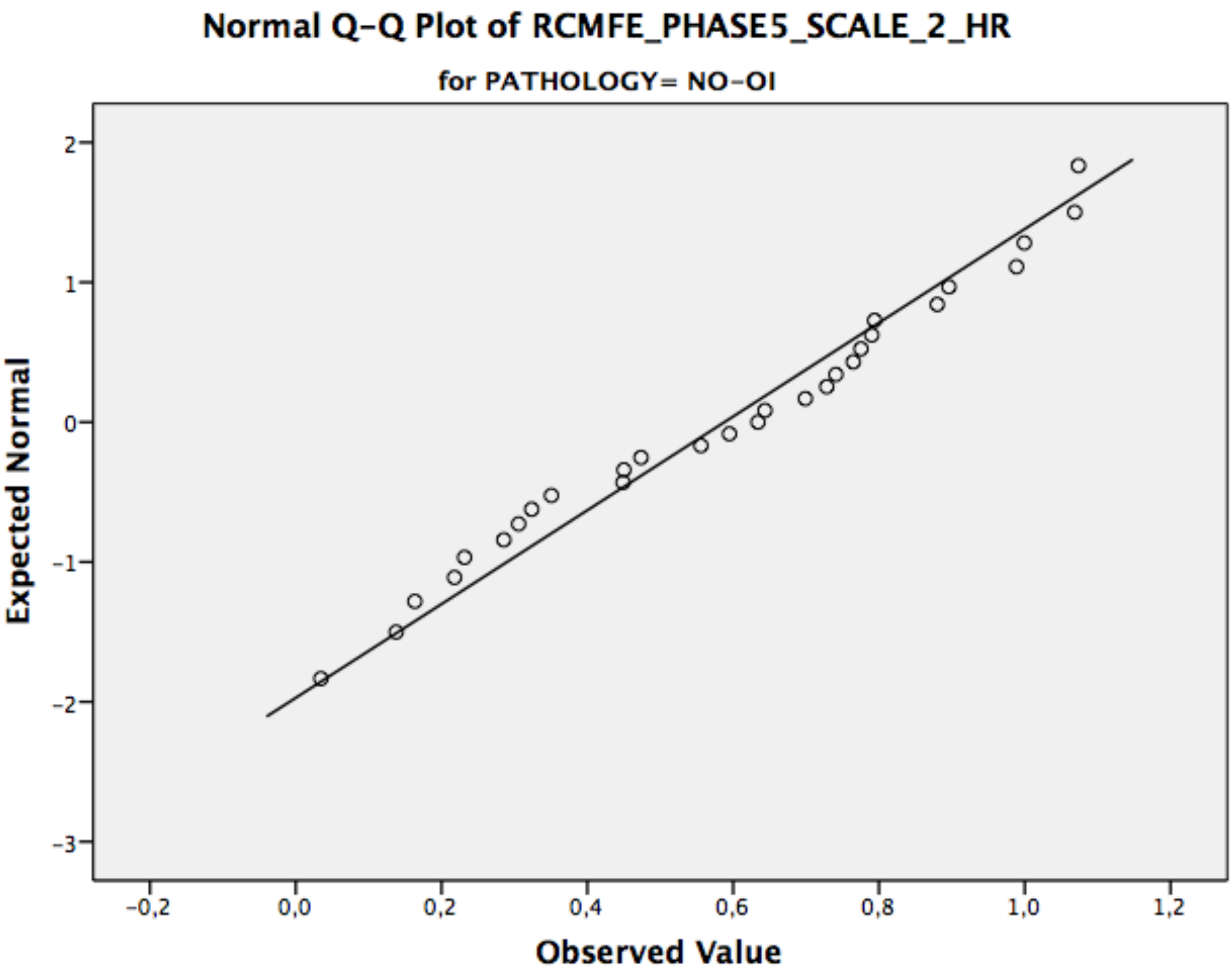

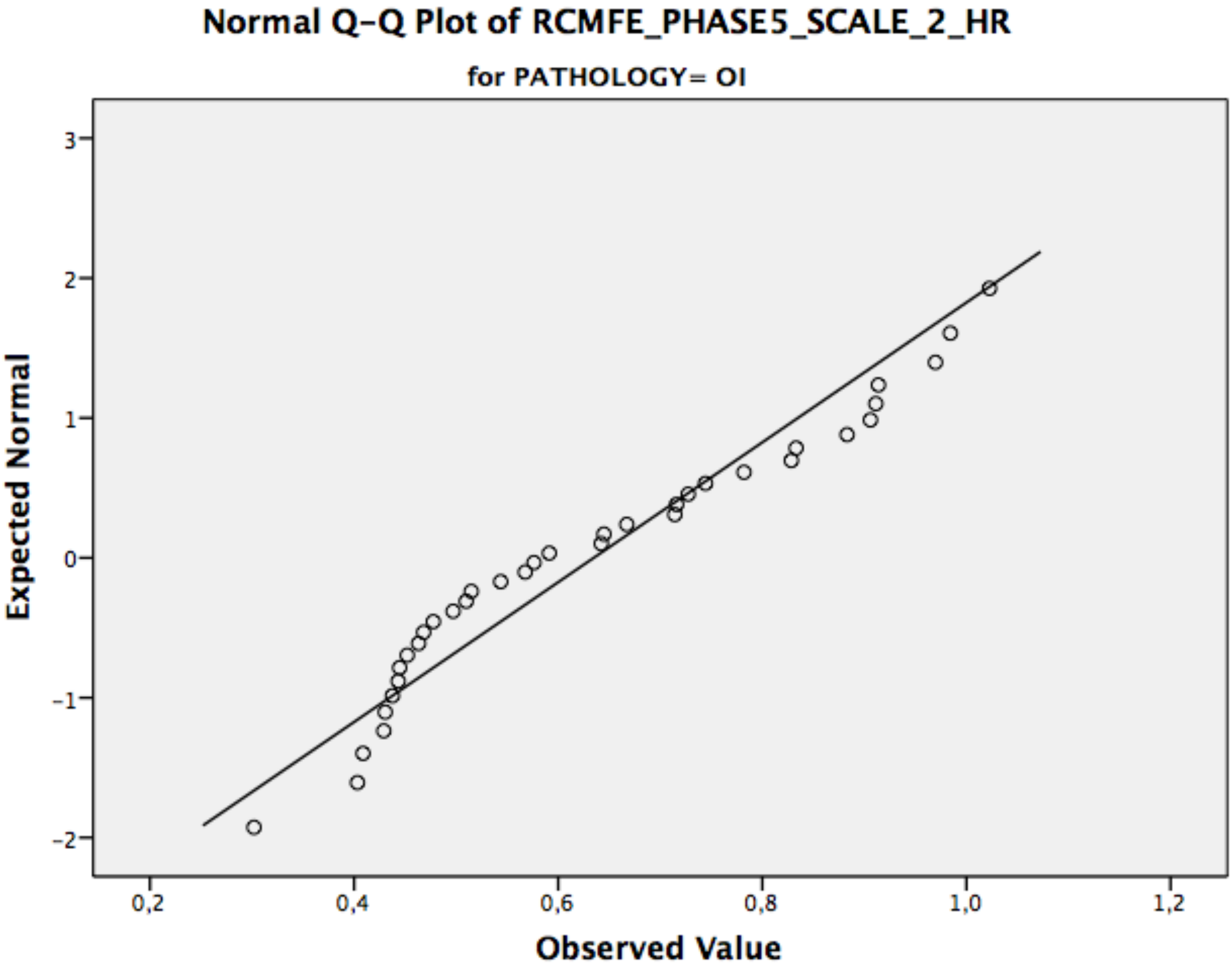
Normal Q-Q Plot of RCMFE_PHASE5_SCALE_2_HR
for PATHOLOGY= OI
Expected Normal
Observed Value
3
2
1
0
-1
-2
0,2
0,4
0,6
0,8
1,0
1,2

RCMFE_PHASE5_SCALE_3_HR

Normal Q-Q Plots

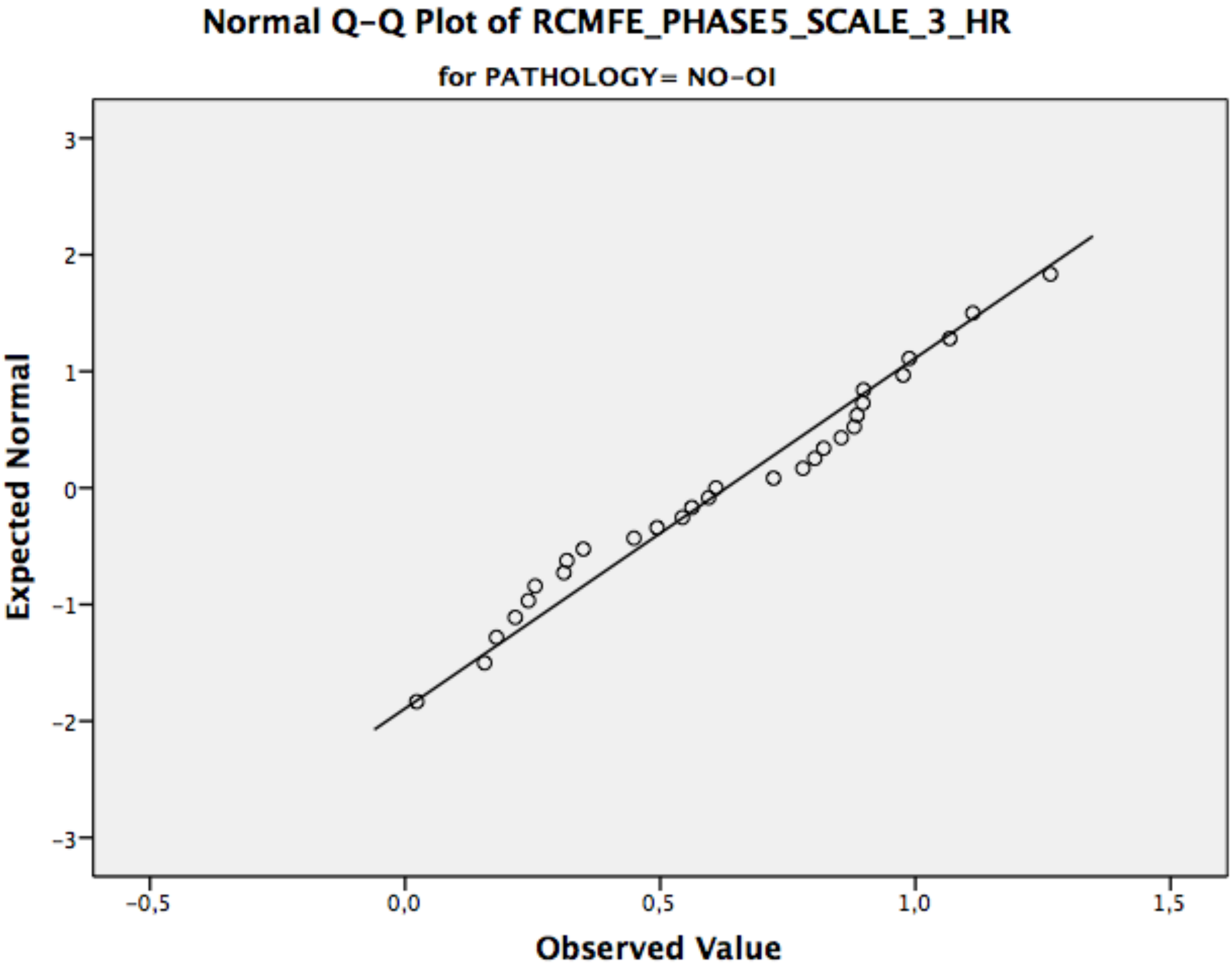

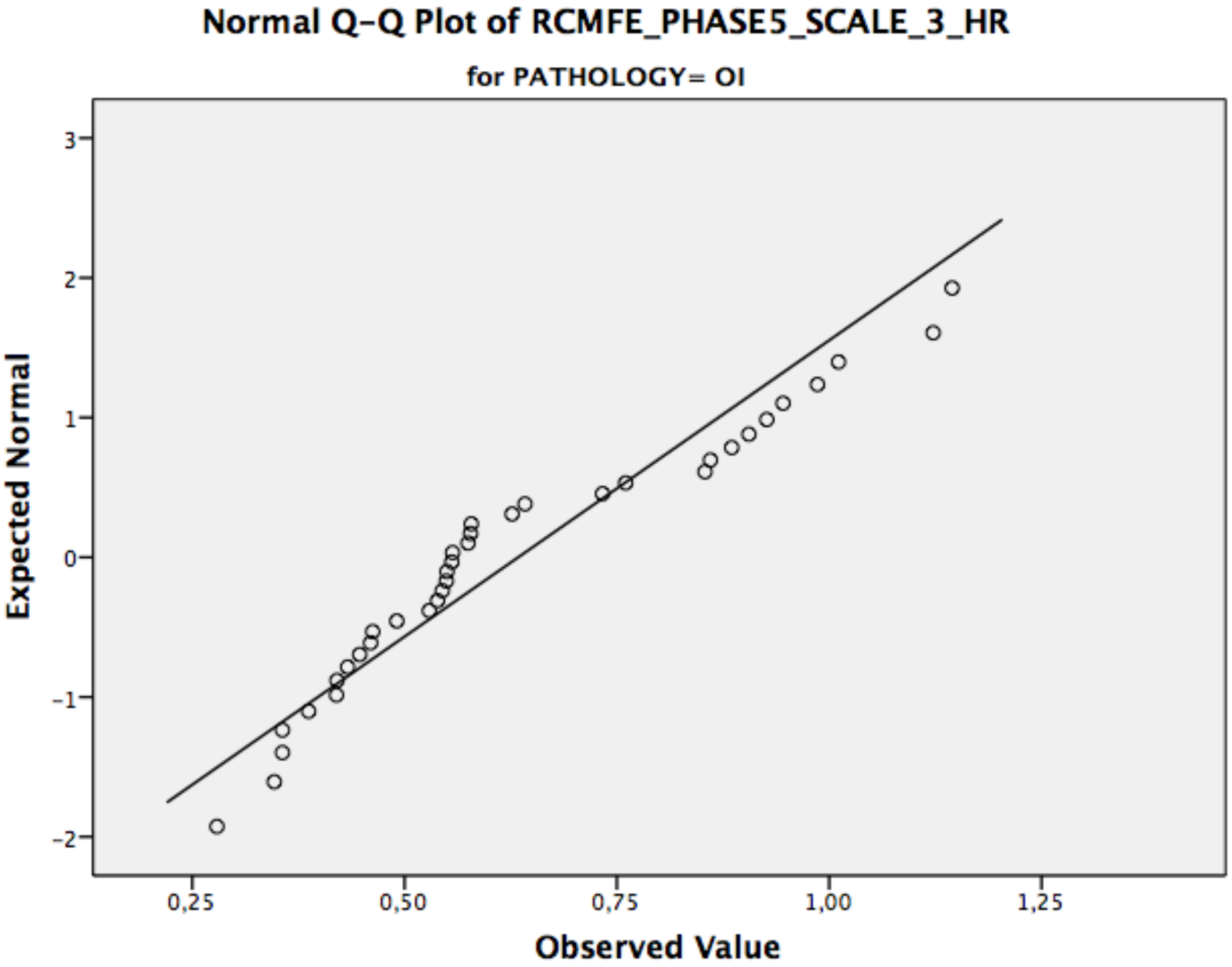

Normal Q-Q Plot of RCMFE_PHASE5_SCALE_3_HR
for PATHOLOGY= OI
Expected Normal
Observed Value
3
2
1
0
-1
-2
0,25
0,50
0,75
1,00
1,25

RCMFE_PHASE5_SCALE_4_HR

Normal Q-Q Plots

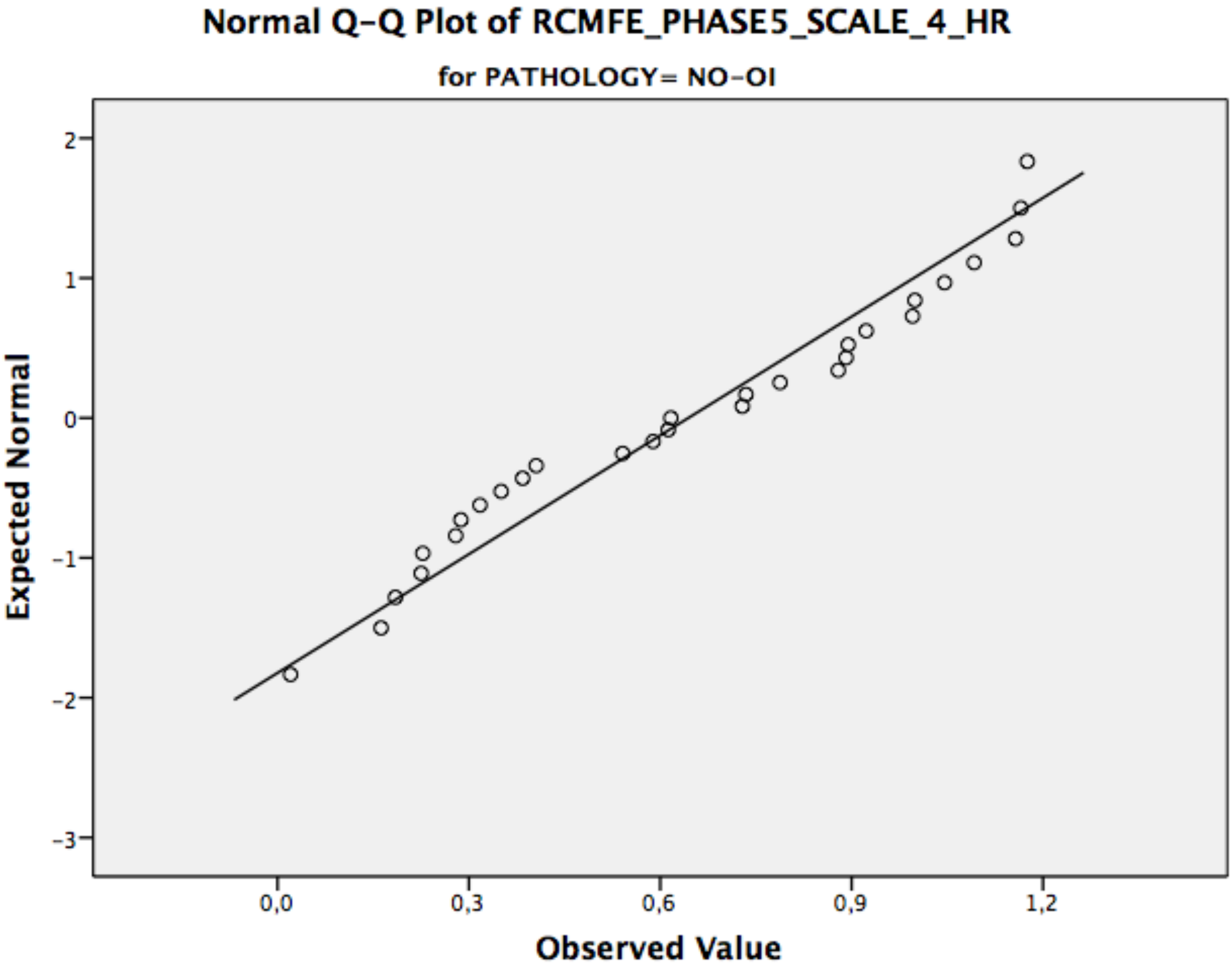

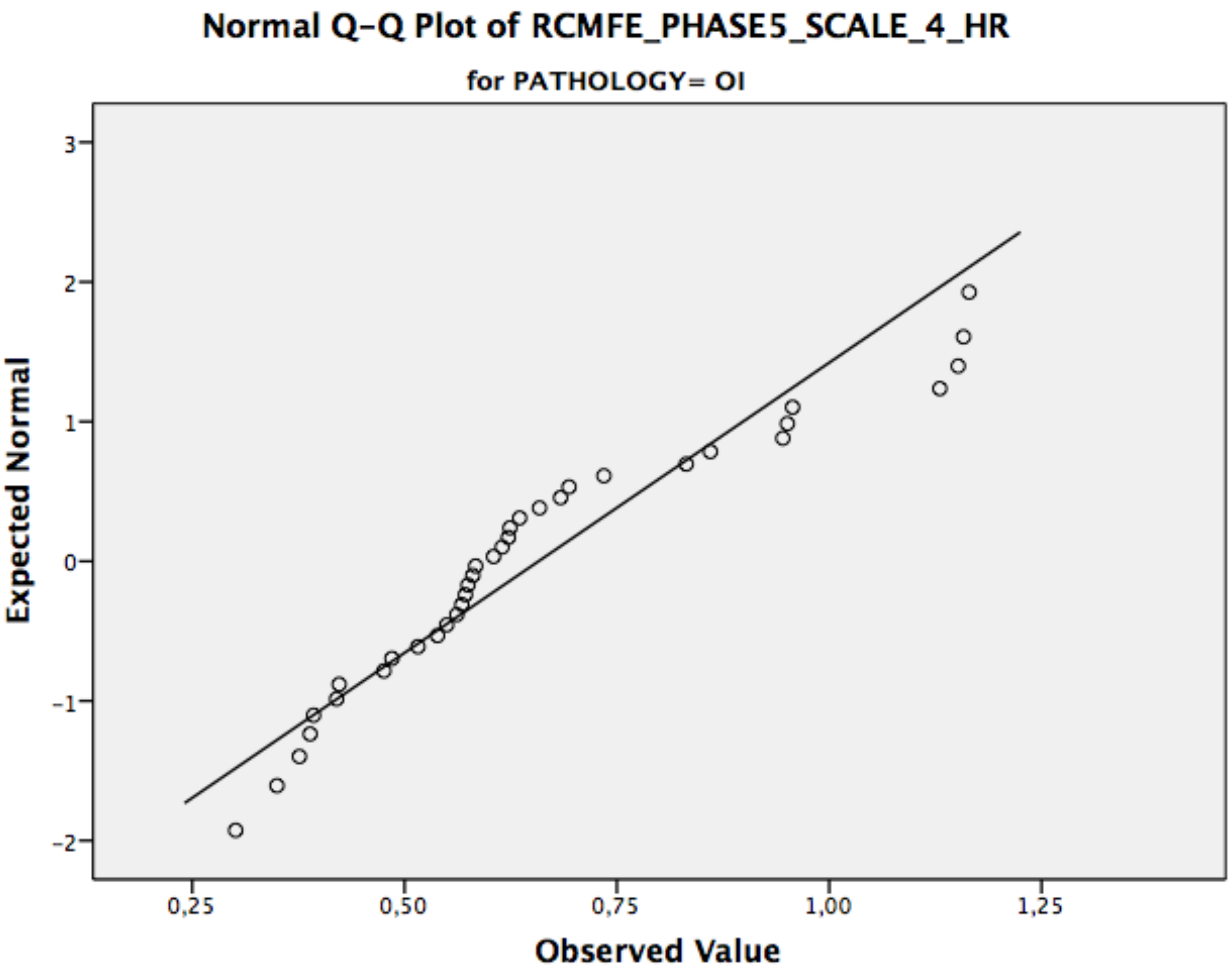

Normal Q-Q Plot of RCMFE_PHASE5_SCALE_4_HR
for PATHOLOGY= OI
Expected Normal
3
2
1
0
-1
-2
0,25
0,50
0,75
1,00
1,25
Observed Value

RCMFE_PHASE5_SCALE_5_HR

Normal Q-Q Plots

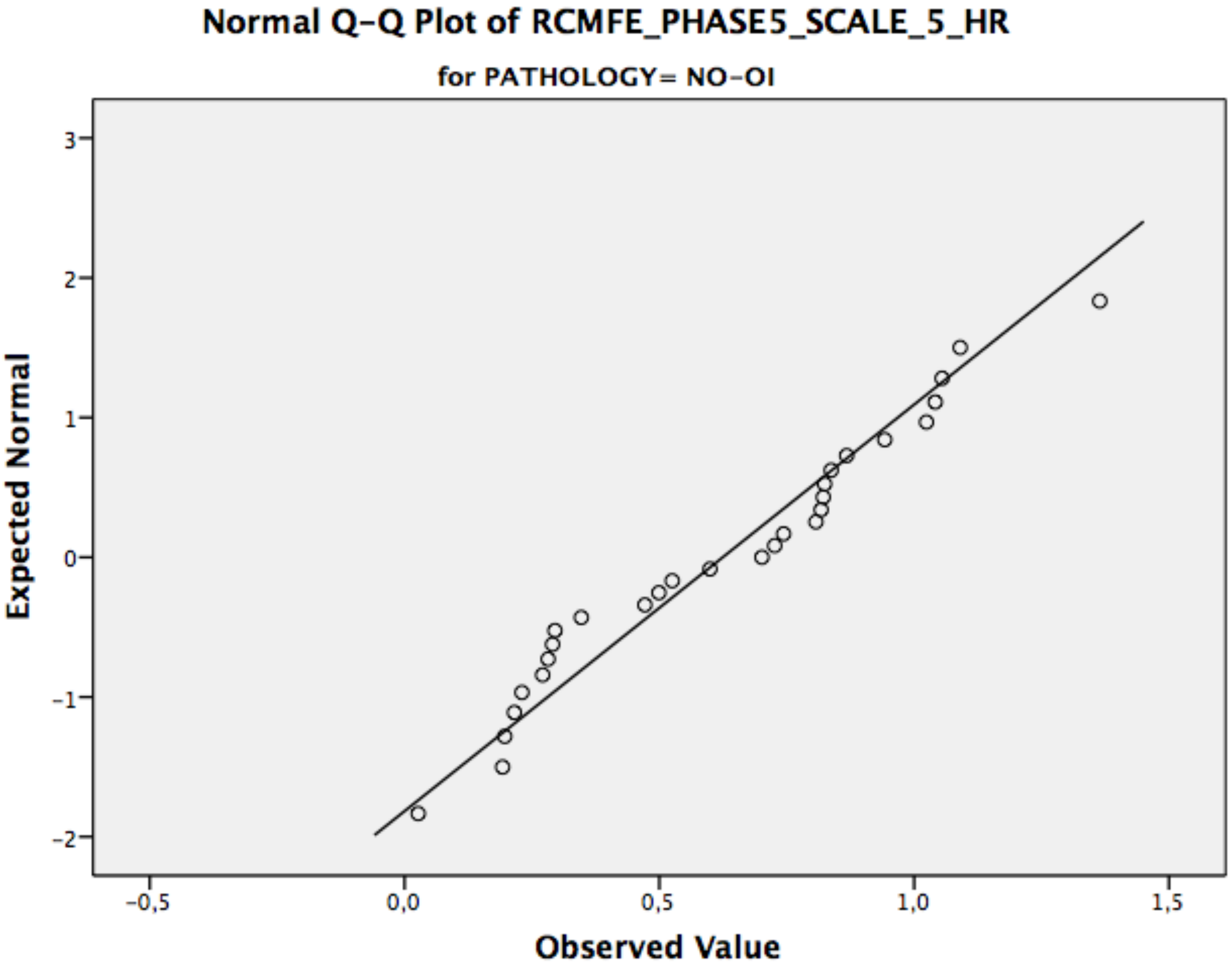

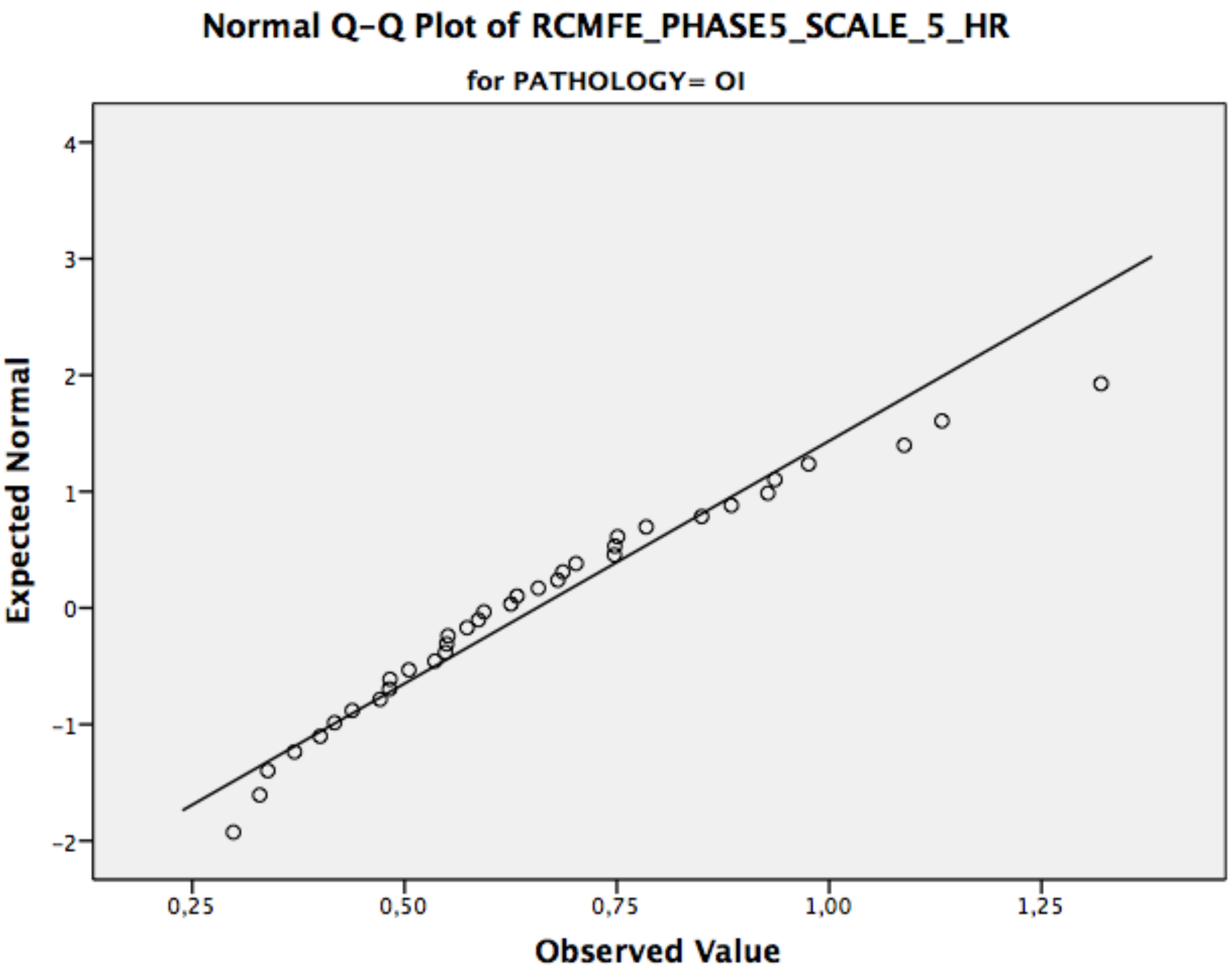
Normal Q-Q Plot of RCMFE_PHASE5_SCALE_5_HR
for PATHOLOGY= OI
Expected Normal
4
3
2
1
0
-1
-2
0,25
0,50
0,75
1,00
1,25
Observed Value

Explore CO

GENDER

Case Processing Summary

	GENDER	Cases					
		Valid		Missing		Total	
		N	Percent	N	Percent	N	Percent
RCMFE_PHASE1_SCALE_1_CO	WOMEN	42	100,0%	0	0,0%	42	100,0%
	MEN	23	100,0%	0	0,0%	23	100,0%
RCMFE_PHASE1_SCALE_2_CO	WOMEN	42	100,0%	0	0,0%	42	100,0%
	MEN	23	100,0%	0	0,0%	23	100,0%
RCMFE_PHASE1_SCALE_3_CO	WOMEN	42	100,0%	0	0,0%	42	100,0%
	MEN	23	100,0%	0	0,0%	23	100,0%
RCMFE_PHASE1_SCALE_4_CO	WOMEN	42	100,0%	0	0,0%	42	100,0%
	MEN	23	100,0%	0	0,0%	23	100,0%
RCMFE_PHASE1_SCALE_5_CO	WOMEN	42	100,0%	0	0,0%	42	100,0%
	MEN	23	100,0%	0	0,0%	23	100,0%
RCMFE_PHASE2_SCALE_1_CO	WOMEN	42	100,0%	0	0,0%	42	100,0%
	MEN	23	100,0%	0	0,0%	23	100,0%
RCMFE_PHASE2_SCALE_2_CO	WOMEN	42	100,0%	0	0,0%	42	100,0%
	MEN	23	100,0%	0	0,0%	23	100,0%
RCMFE_PHASE2_SCALE_3_CO	WOMEN	42	100,0%	0	0,0%	42	100,0%
	MEN	23	100,0%	0	0,0%	23	100,0%
RCMFE_PHASE2_SCALE_4_CO	WOMEN	42	100,0%	0	0,0%	42	100,0%
	MEN	23	100,0%	0	0,0%	23	100,0%
RCMFE_PHASE2_SCALE_5_CO	WOMEN	42	100,0%	0	0,0%	42	100,0%
	MEN	23	100,0%	0	0,0%	23	100,0%
RCMFE_PHASE3_SCALE_1_CO	WOMEN	42	100,0%	0	0,0%	42	100,0%
	MEN	23	100,0%	0	0,0%	23	100,0%
RCMFE_PHASE3_SCALE_2_CO	WOMEN	42	100,0%	0	0,0%	42	100,0%
	MEN	23	100,0%	0	0,0%	23	100,0%
RCMFE_PHASE3_SCALE_3_CO	WOMEN	42	100,0%	0	0,0%	42	100,0%
	MEN	23	100,0%	0	0,0%	23	100,0%
RCMFE_PHASE3_SCALE_4_CO	WOMEN	42	100,0%	0	0,0%	42	100,0%
	MEN	23	100,0%	0	0,0%	23	100,0%
RCMFE_PHASE3_SCALE_5_CO	WOMEN	42	100,0%	0	0,0%	42	100,0%
	MEN	23	100,0%	0	0,0%	23	100,0%
RCMFE_PHASE4_SCALE_1_CO	WOMEN	42	100,0%	0	0,0%	42	100,0%
	MEN	23	100,0%	0	0,0%	23	100,0%
RCMFE_PHASE4_SCALE_2_CO	WOMEN	42	100,0%	0	0,0%	42	100,0%
	MEN	23	100,0%	0	0,0%	23	100,0%
RCMFE_PHASE4_SCALE_3	WOMEN	42	100,0%	0	0,0%	42	100,0%
	MEN	23	100,0%	0	0,0%	23	100,0%
RCMFE_PHASE4_SCALE_4_CO	WOMEN	42	100,0%	0	0,0%	42	100,0%
	MEN	23	100,0%	0	0,0%	23	100,0%
RCMFE_PHASE4_SCALE_5_CO	WOMEN	42	100,0%	0	0,0%	42	100,0%
	MEN	23	100,0%	0	0,0%	23	100,0%
RCMFE_PHASE5_SCALE_1_CO	WOMEN	42	100,0%	0	0,0%	42	100,0%

Case Processing Summary

	GENDER	Cases					
		Valid		Missing		Total	
		N	Percent	N	Percent	N	Percent
RCMFE_PHASE5_SCALE_1_CO	MEN	23	100,0%	0	0,0%	23	100,0%
RCMFE_PHASE5_SCALE_2_CO	WOMEN	42	100,0%	0	0,0%	42	100,0%
	MEN	23	100,0%	0	0,0%	23	100,0%
RCMFE_PHASE5_SCALE_3_CO	WOMEN	42	100,0%	0	0,0%	42	100,0%
	MEN	23	100,0%	0	0,0%	23	100,0%
RCMFE_PHASE5_SCALE_4_CO	WOMEN	42	100,0%	0	0,0%	42	100,0%
	MEN	23	100,0%	0	0,0%	23	100,0%
RCMFE_PHASE5_SCALE_5_CO	WOMEN	42	100,0%	0	0,0%	42	100,0%
	MEN	23	100,0%	0	0,0%	23	100,0%

Tests of Normality

	GENDER	Kolmogorov-Smirnov[a]			Shapiro-Wilk		
		Statistic	df	Sig.	Statistic	df	Sig.
RCMFE_PHASE1_SCALE_1_CO	WOMEN	,078	42	,200*	,982	42	,729
	MEN	,125	23	,200*	,951	23	,301
RCMFE_PHASE1_SCALE_2_CO	WOMEN	,075	42	,200*	,985	42	,838
	MEN	,123	23	,200*	,947	23	,257
RCMFE_PHASE1_SCALE_3_CO	WOMEN	,109	42	,200*	,962	42	,170
	MEN	,134	23	,200*	,945	23	,235
RCMFE_PHASE1_SCALE_4_CO	WOMEN	,120	42	,141	,970	42	,328
	MEN	,123	23	,200*	,955	23	,374
RCMFE_PHASE1_SCALE_5_CO	WOMEN	,068	42	,200*	,979	42	,642
	MEN	,088	23	,200*	,969	23	,654
RCMFE_PHASE2_SCALE_1_CO	WOMEN	,065	42	,200*	,976	42	,501
	MEN	,122	23	,200*	,971	23	,710
RCMFE_PHASE2_SCALE_2_CO	WOMEN	,081	42	,200*	,989	42	,957
	MEN	,093	23	,200*	,981	23	,922
RCMFE_PHASE2_SCALE_3_CO	WOMEN	,085	42	,200*	,973	42	,417
	MEN	,120	23	,200*	,974	23	,792
RCMFE_PHASE2_SCALE_4_CO	WOMEN	,105	42	,200*	,960	42	,144
	MEN	,087	23	,200*	,976	23	,829
RCMFE_PHASE2_SCALE_5_CO	WOMEN	,150	42	,069	,895	42	,001
	MEN	,086	23	,200*	,970	23	,686
RCMFE_PHASE3_SCALE_1_CO	WOMEN	,070	42	,200*	,974	42	,455
	MEN	,224	23	,054	,853	23	,003
RCMFE_PHASE3_SCALE_2_CO	WOMEN	,123	42	,114	,969	42	,297
	MEN	,081	23	,200*	,965	23	,582
RCMFE_PHASE3_SCALE_3_CO	WOMEN	,042	42	,200*	,989	42	,960
	MEN	,164	23	,110	,964	23	,547
RCMFE_PHASE3_SCALE_4_CO	WOMEN	,100	42	,200*	,974	42	,448
	MEN	,082	23	,200*	,987	23	,985
RCMFE_PHASE3_SCALE_5_	WOMEN	,114	42	,192	,973	42	,423

CO	MEN	,104	23	,200*	,973	23	,769
RCMFE_PHASE4_SCALE_1_	WOMEN	,201	42	,040	,775	42	,070
CO	MEN	,143	23	,200*	,952	23	,318
RCMFE_PHASE4_SCALE_2_	WOMEN	,196	42	,050	,778	42	,060
CO	MEN	,131	23	,200*	,950	23	,295
RCMFE_PHASE4_SCALE_3	WOMEN	,135	42	,051	,848	42	,020
	MEN	,153	23	,175	,940	23	,178
RCMFE_PHASE4_SCALE_4_	WOMEN	,169	42	,004	,849	42	,050
CO	MEN	,161	23	,124	,949	23	,282
RCMFE_PHASE4_SCALE_5_	WOMEN	,144	42	,028	,857	42	,060
CO	MEN	,186	23	,038	,922	23	,074
RCMFE_PHASE5_SCALE_1_	WOMEN	,074	42	,200*	,977	42	,545
CO	MEN	,087	23	,200*	,968	23	,633

Tests of Normality

	GENDER	Kolmogorov-Smirnov[a]			Shapiro-Wilk		
		Statistic	df	Sig.	Statistic	df	Sig.
RCMFE_PHASE5_SCALE_2_	WOMEN	,061	42	,200*	,976	42	,521
CO	MEN	,157	23	,149*	,937	23	,157
RCMFE_PHASE5_SCALE_3_	WOMEN	,095	42	,200*	,972	42	,373
CO	MEN	,077	23	,200*	,949	23	,283
RCMFE_PHASE5_SCALE_4_	WOMEN	,101	42	,200*	,958	42	,125
CO	MEN	,117	23	,200*	,952	23	,317
RCMFE_PHASE5_SCALE_5_	WOMEN	,142	42	,033	,942	42	,533
CO	MEN	,176	23	,063*	,952	23	,322

*. This is a lower bound of the true significance.

a. Lilliefors Significance Correction

RCMFE_PHASE1_SCALE_1_CO

Normal Q-Q Plots

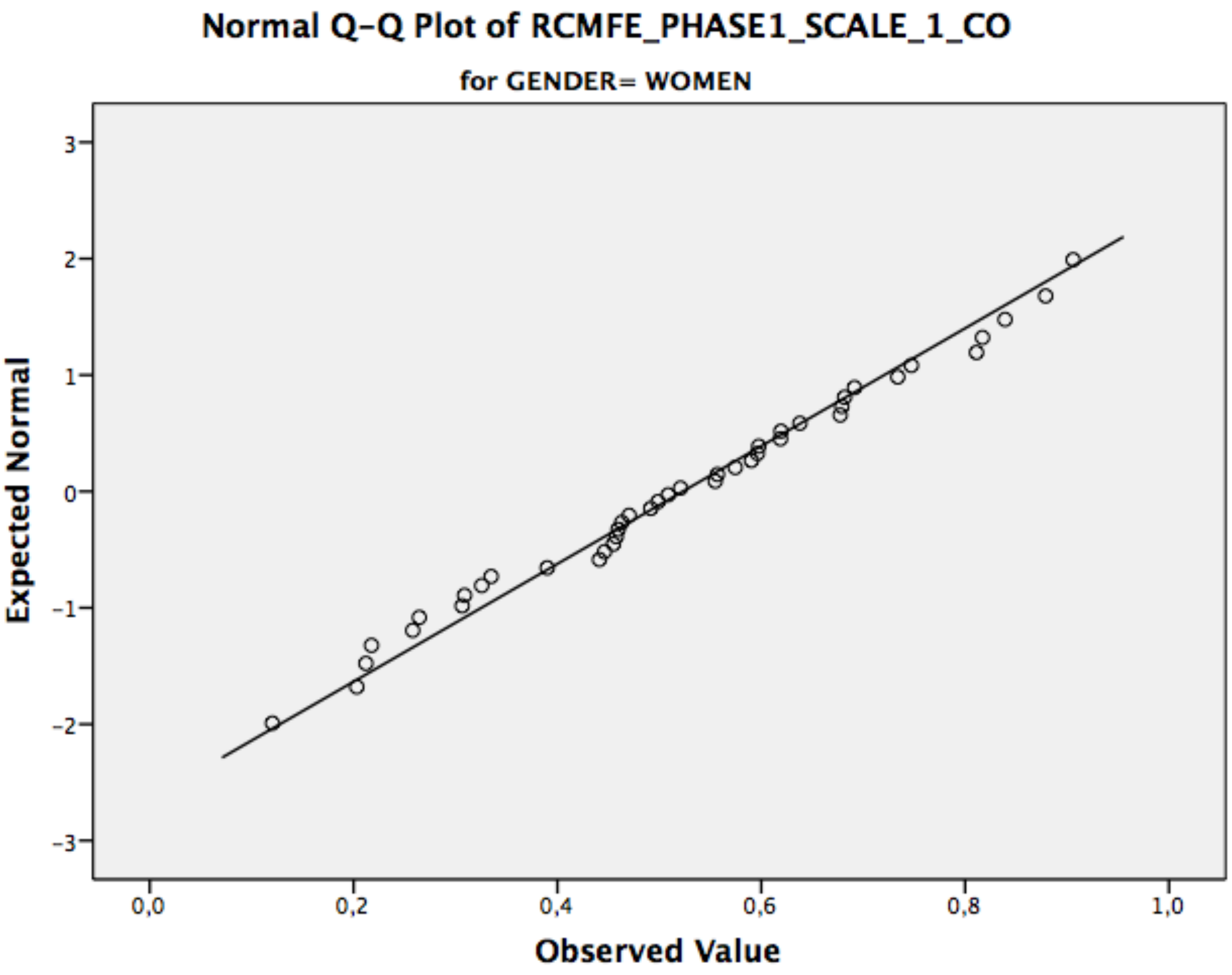

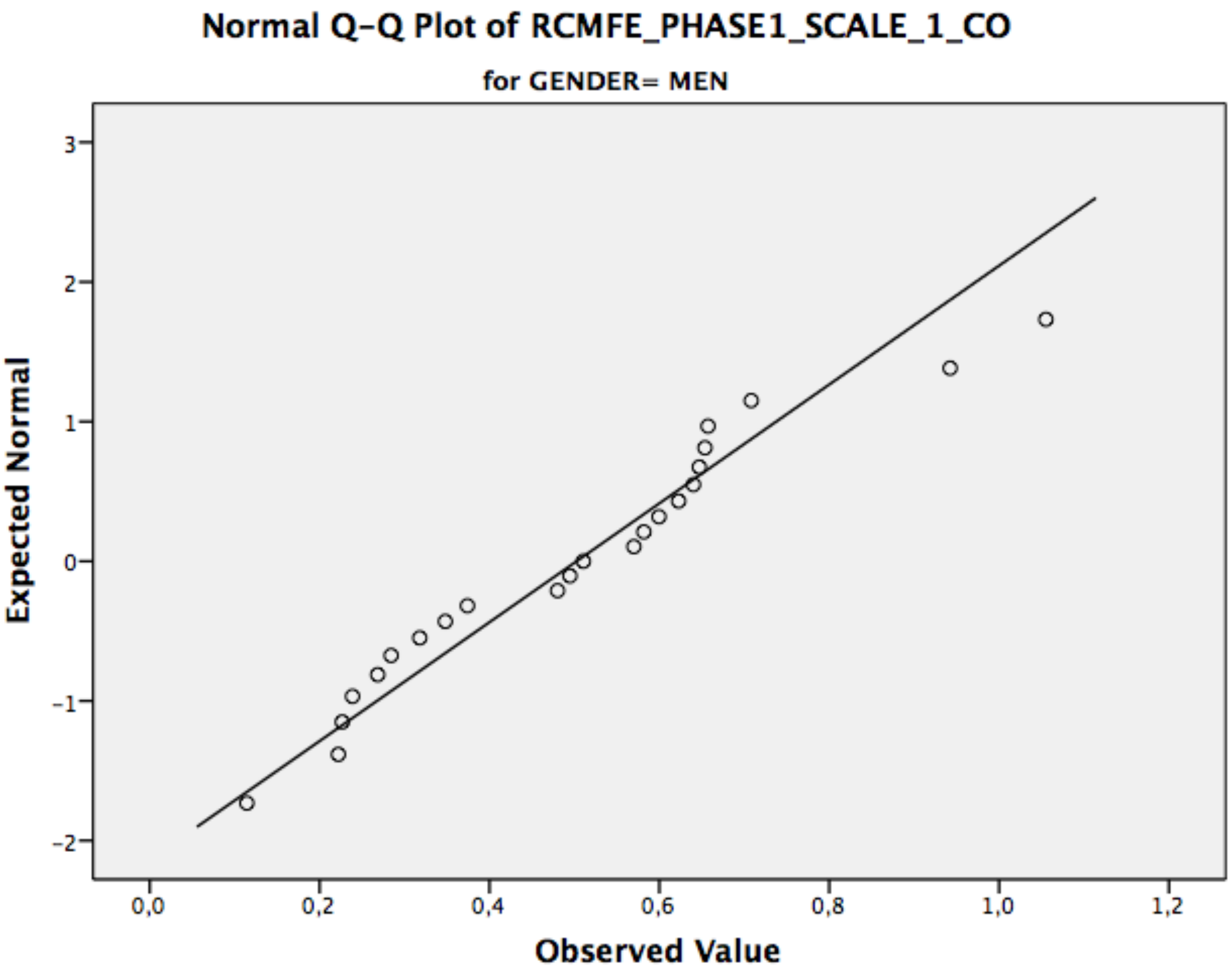
Normal Q-Q Plot of RCMFE_PHASE1_SCALE_1_CO
for GENDER= MEN
Expected Normal
Observed Value
3
2
1
0
−1
−2
0,0
0,2
0,4
0,6
0,8
1,0
1,2

RCMFE_PHASE1_SCALE_2_CO

Normal Q-Q Plots

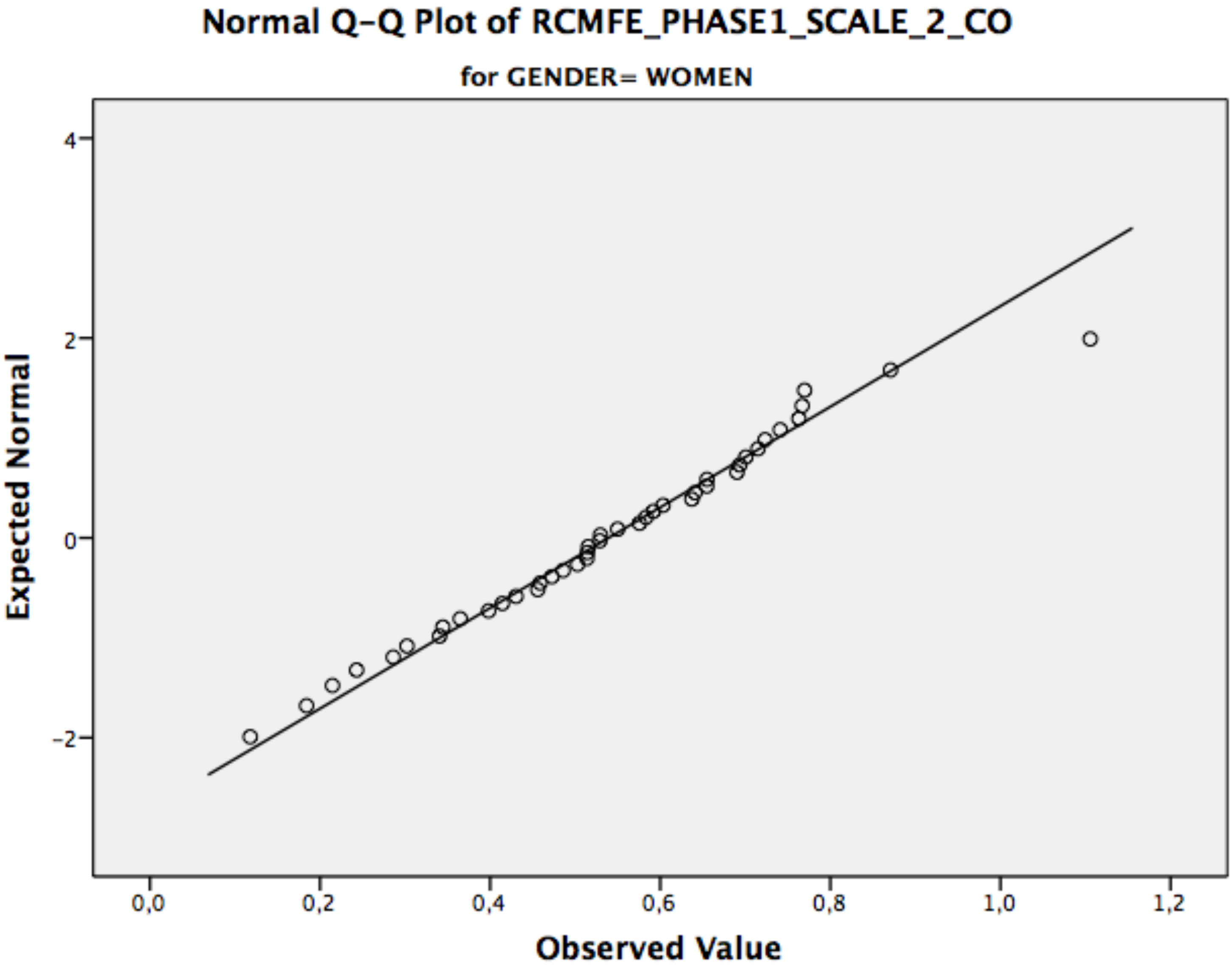

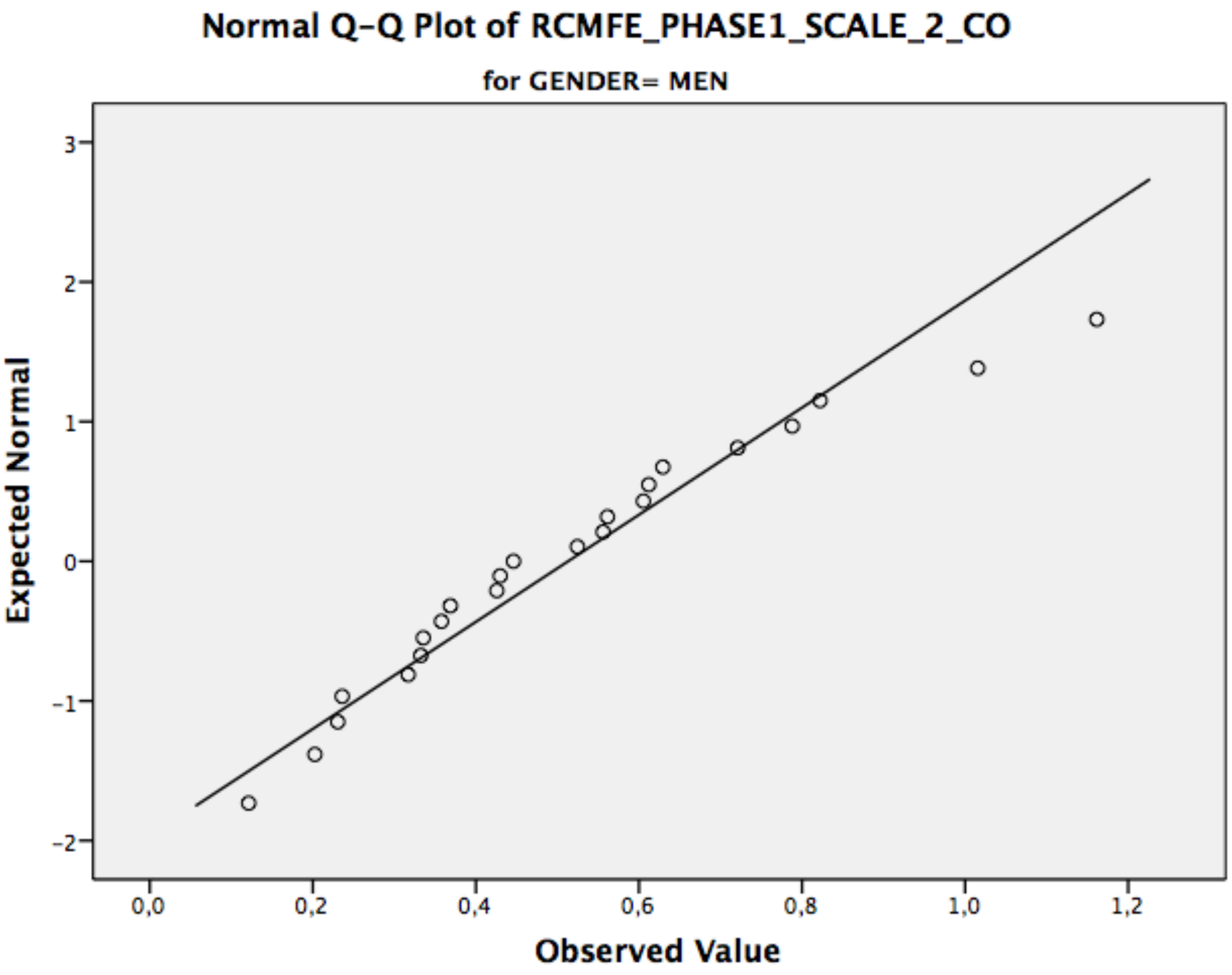
Normal Q-Q Plot of RCMFE_PHASE1_SCALE_2_CO
for GENDER= MEN
Expected Normal
Observed Value
3
2
1
0
-1
-2
0,0
0,2
0,4
0,6
0,8
1,0
1,2

RCMFE_PHASE1_SCALE_3_CO

Normal Q-Q Plots

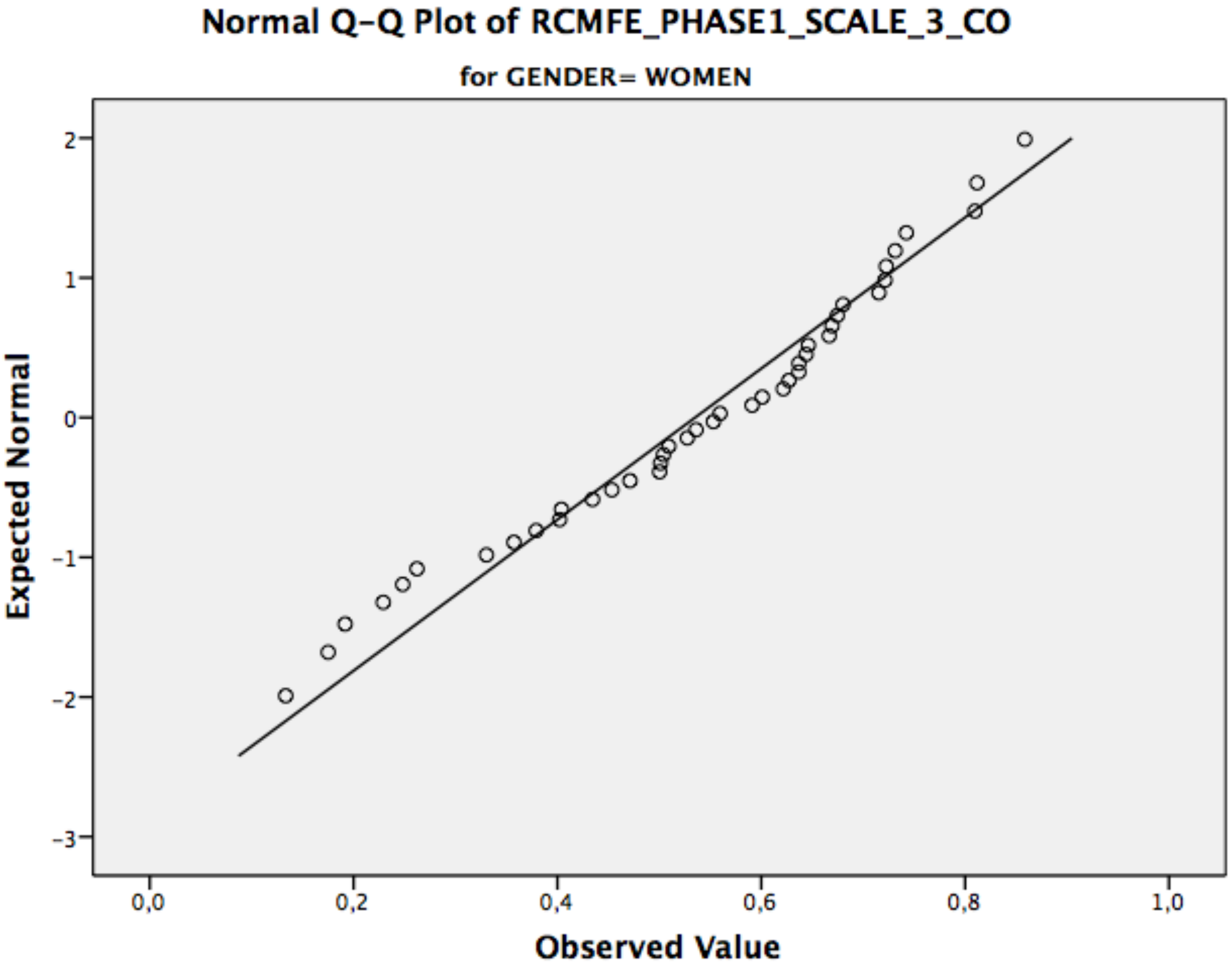

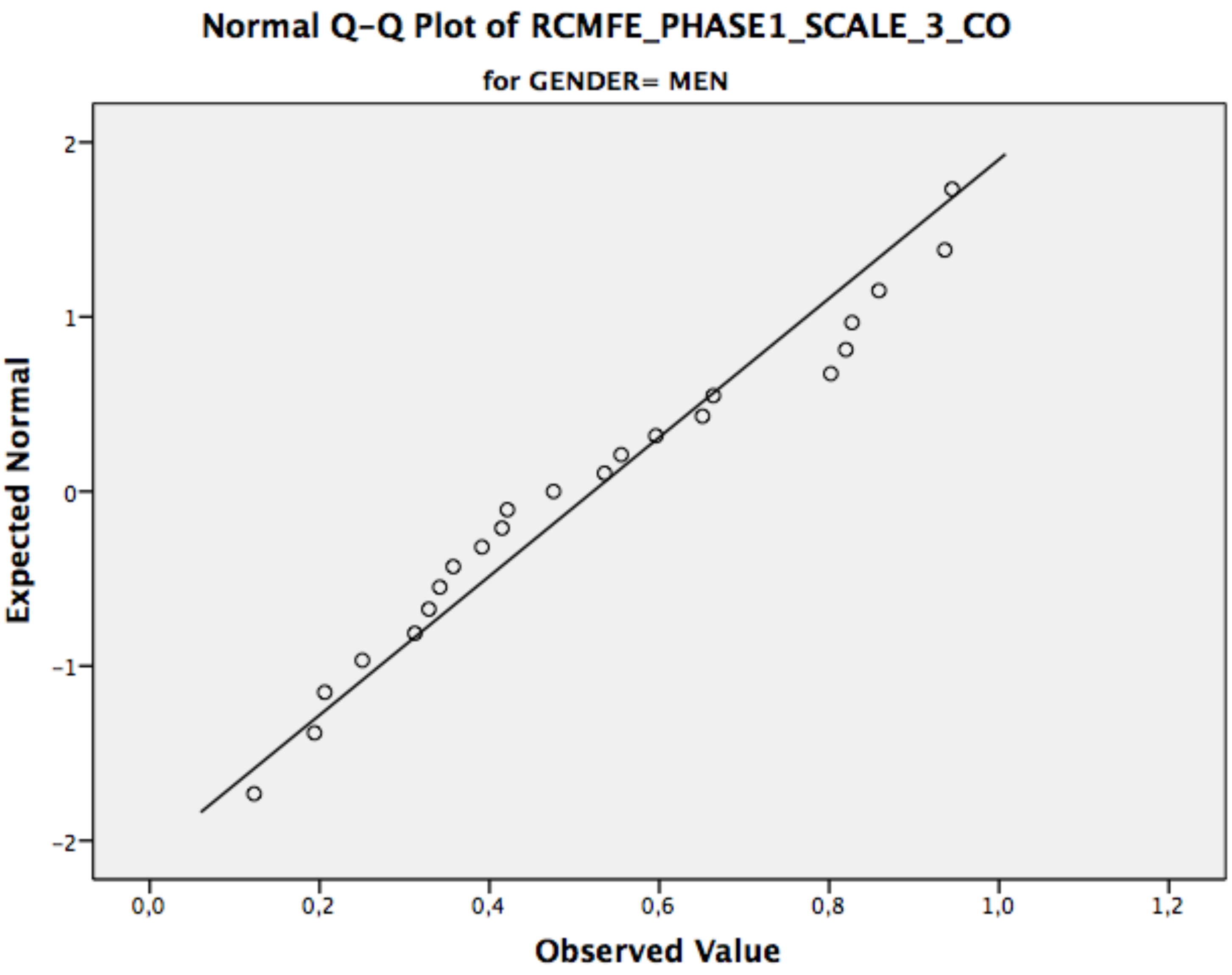

Normal Q-Q Plot of RCMFE_PHASE1_SCALE_3_CO
for GENDER= MEN
Expected Normal
Observed Value
2
1
0
−1
−2
0,0
0,2
0,4
0,6
0,8
1,0
1,2

RCMFE_PHASE1_SCALE_4_CO

Normal Q-Q Plots

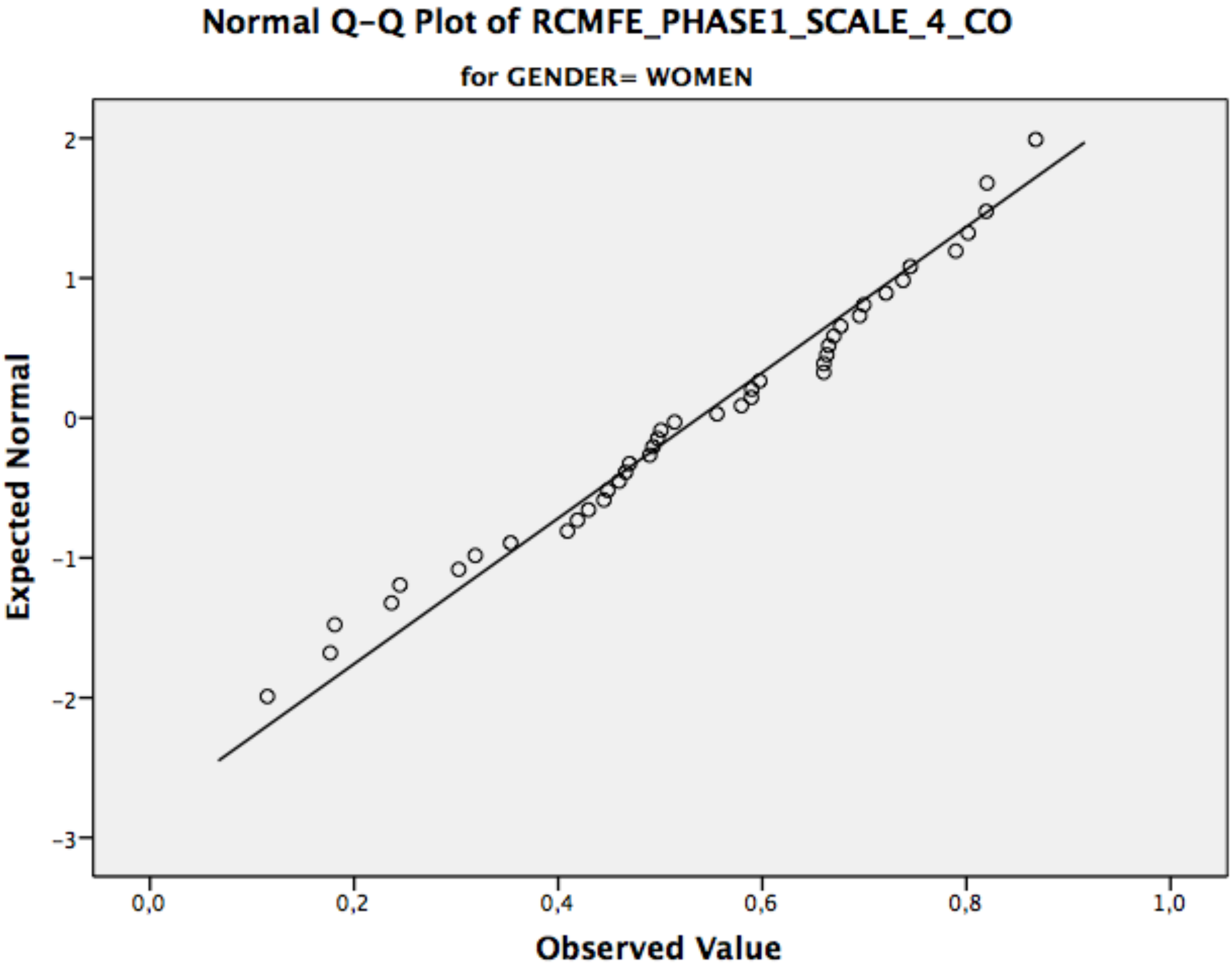

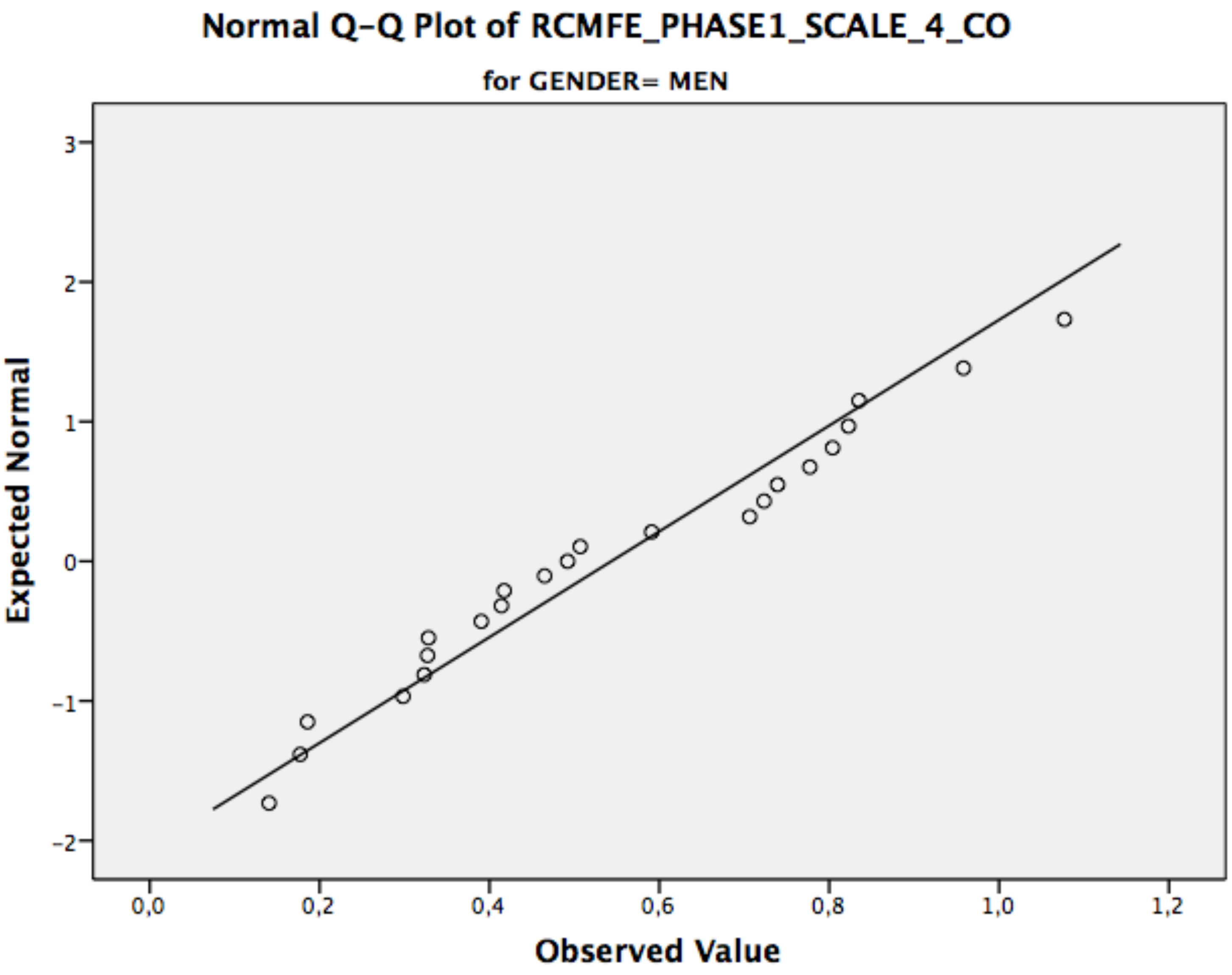
Normal Q-Q Plot of RCMFE_PHASE1_SCALE_4_CO
for GENDER= MEN
Expected Normal
Observed Value
3
2
1
0
-1
-2
0,0
0,2
0,4
0,6
0,8
1,0
1,2

RCMFE_PHASE1_SCALE_5_CO

Normal Q-Q Plots

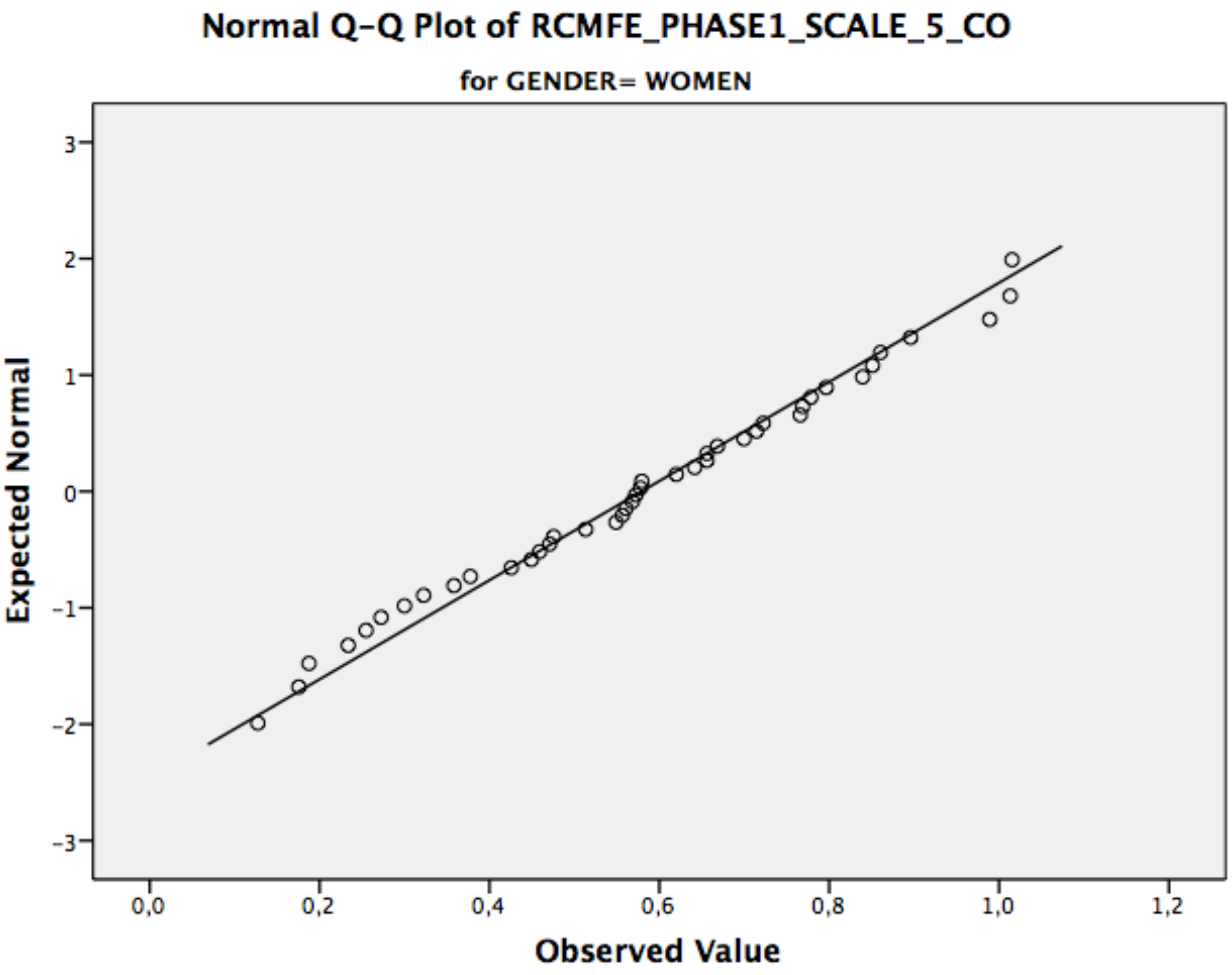

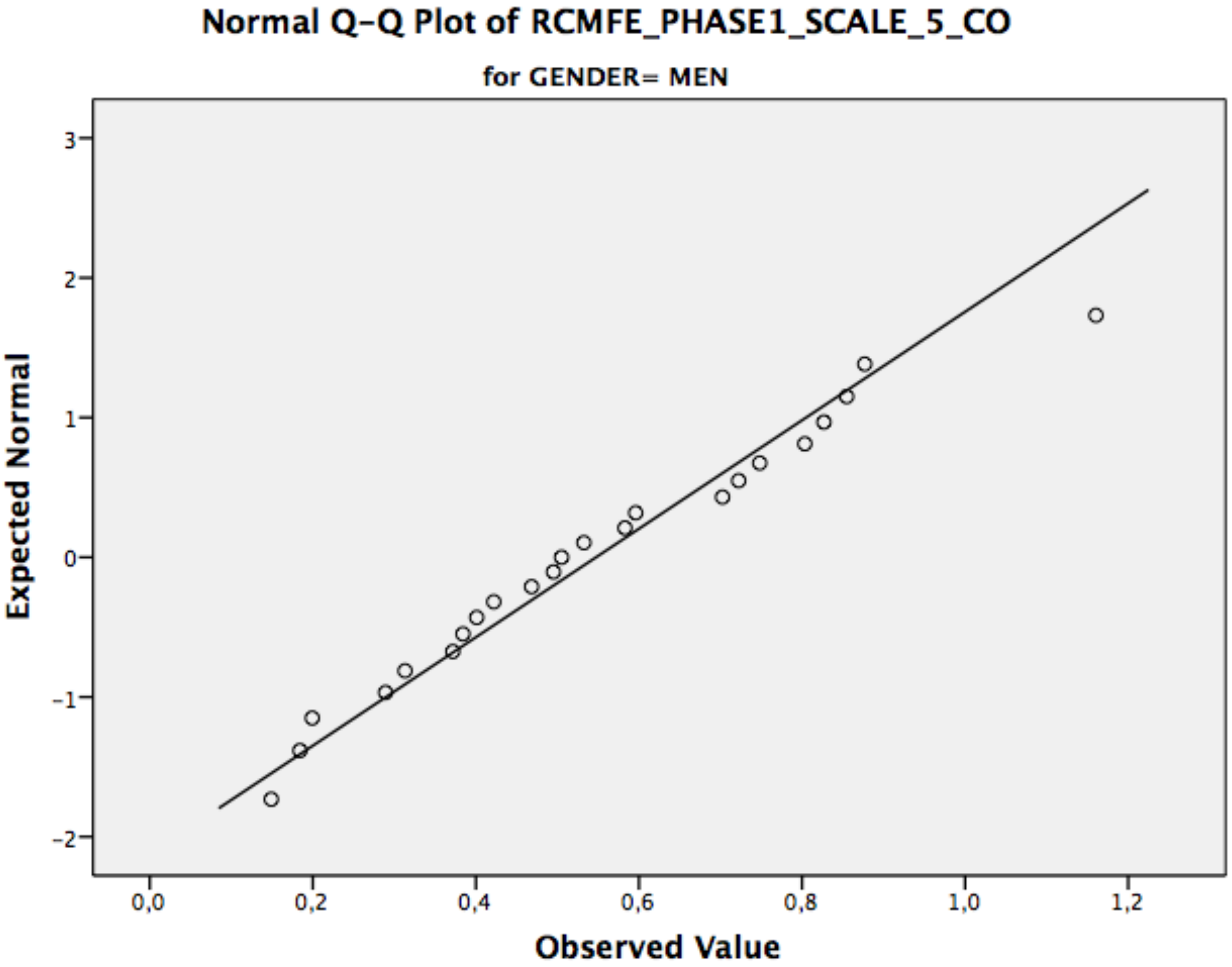
Normal Q-Q Plot of RCMFE_PHASE1_SCALE_5_CO
for GENDER= MEN
Expected Normal
Observed Value
3
2
1
0
-1
-2
0,0
0,2
0,4
0,6
0,8
1,0
1,2

RCMFE_PHASE2_SCALE_1_CO

Normal Q-Q Plots

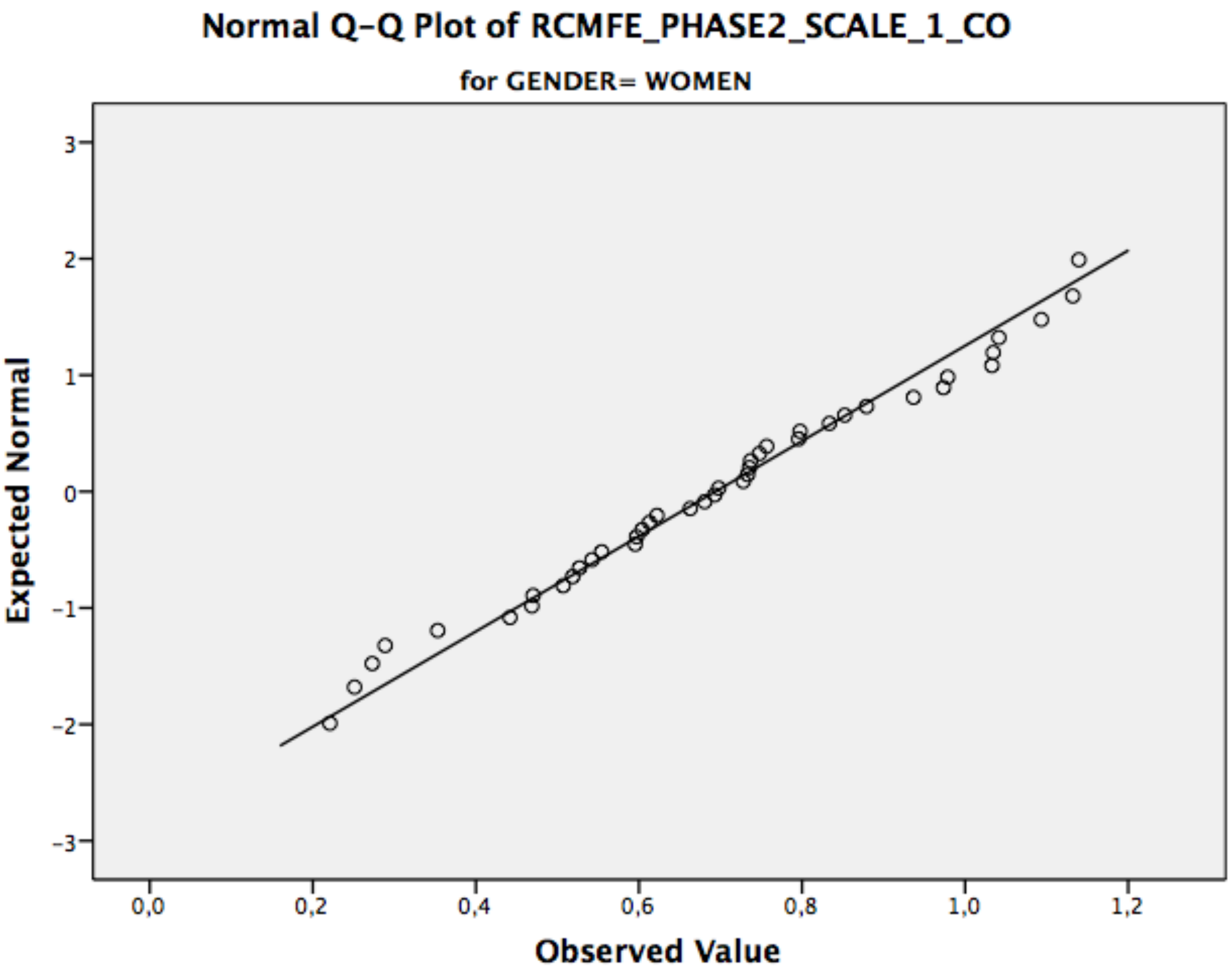

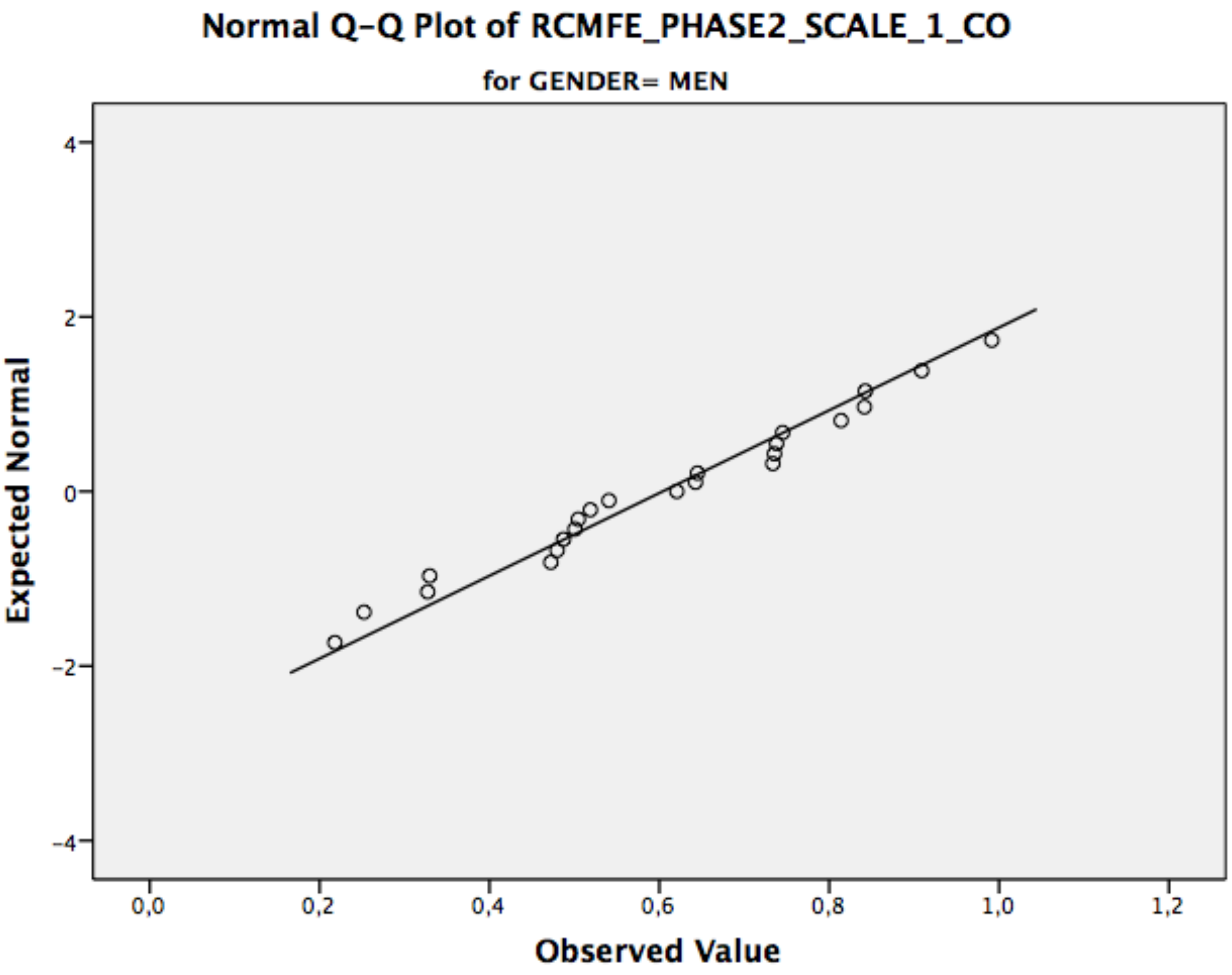
Normal Q-Q Plot of RCMFE_PHASE2_SCALE_1_CO
for GENDER= MEN
Expected Normal
Observed Value
4
2
0
-2
-4
0,0
0,2
0,4
0,6
0,8
1,0
1,2

RCMFE_PHASE2_SCALE_2_CO

Normal Q-Q Plots

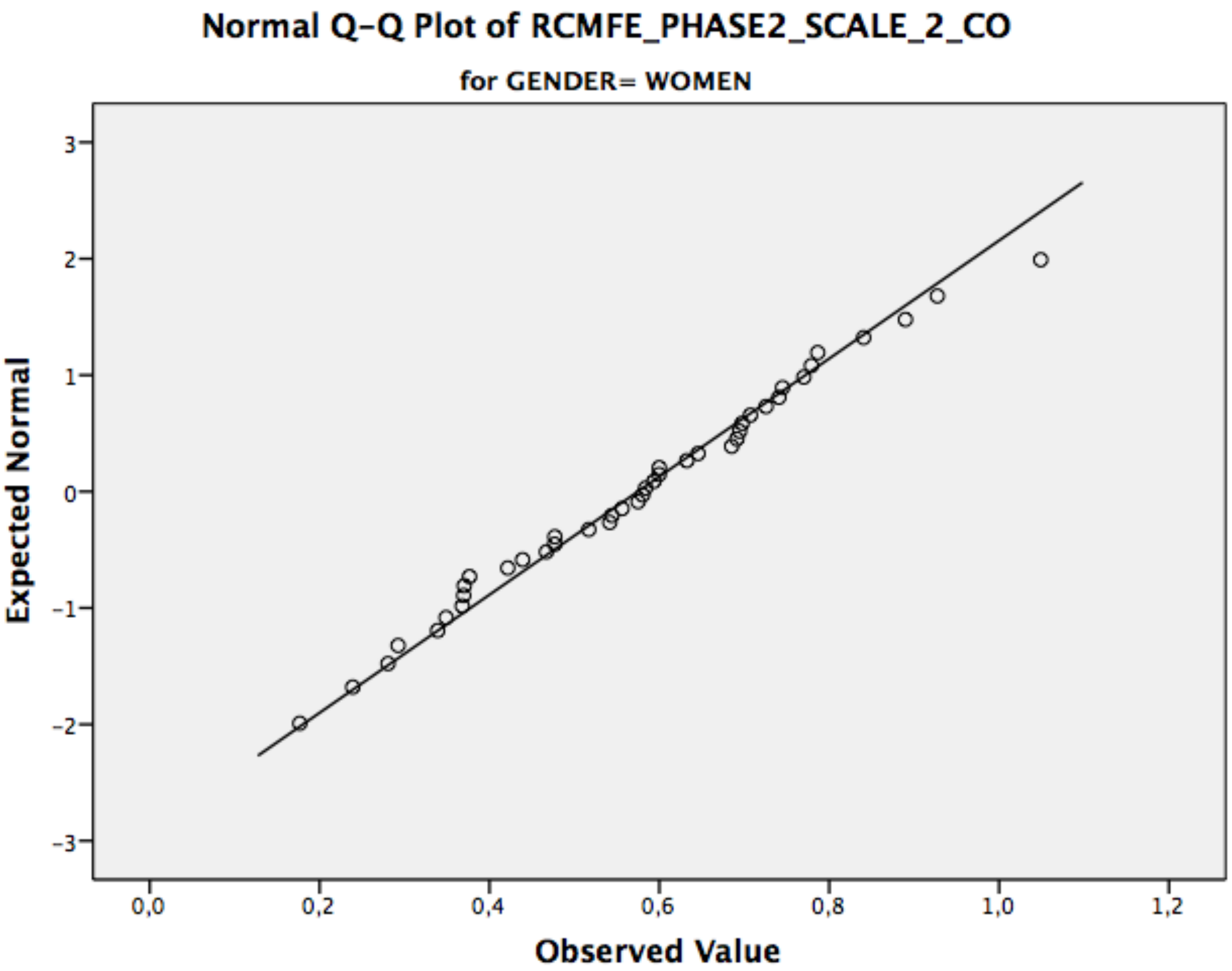

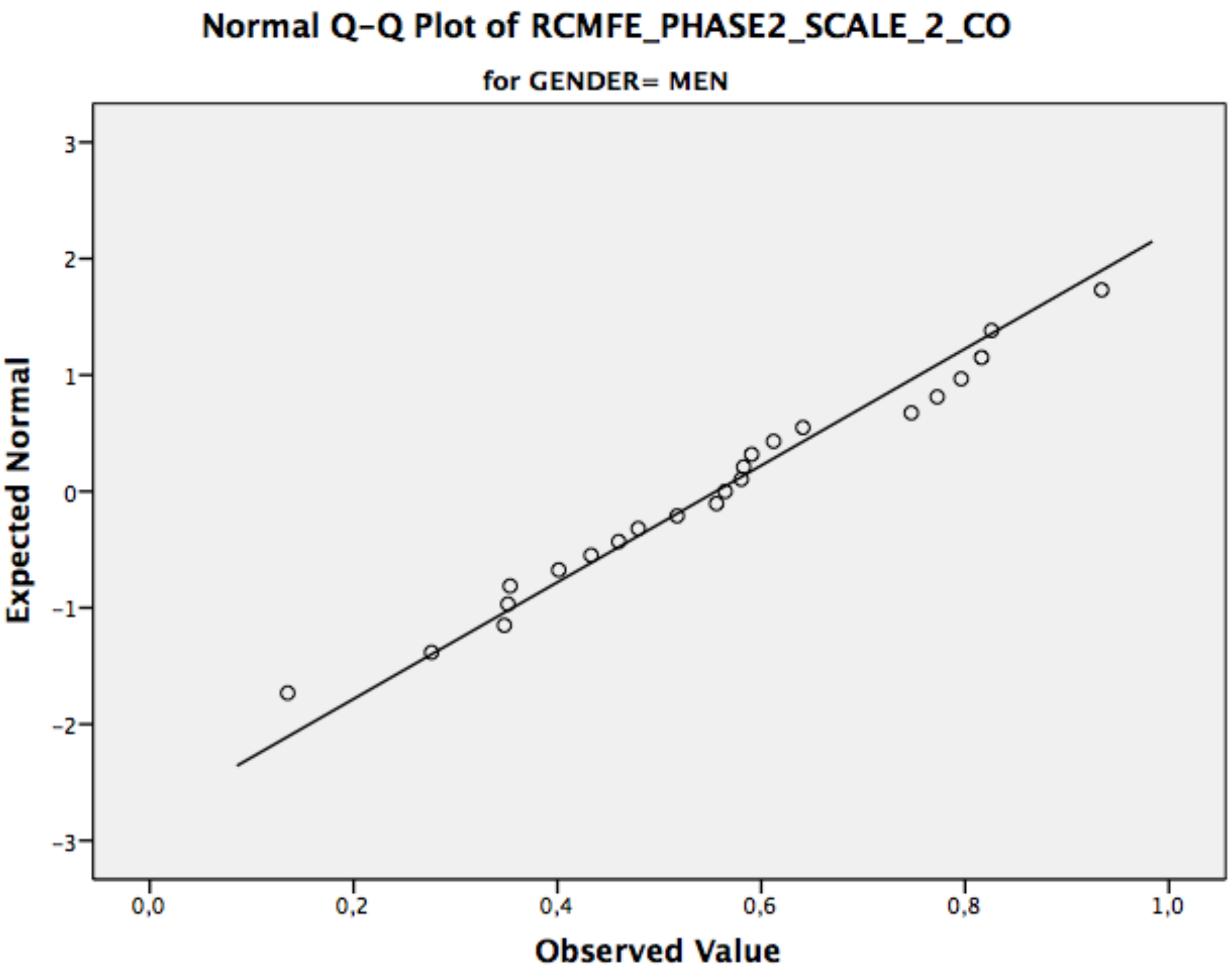
Normal Q-Q Plot of RCMFE_PHASE2_SCALE_2_CO
for GENDER= MEN
Expected Normal
3
2
1
0
-1
-2
-3
0,0
0,2
0,4
0,6
0,8
1,0
Observed Value

RCMFE_PHASE2_SCALE_3_CO

Normal Q-Q Plots

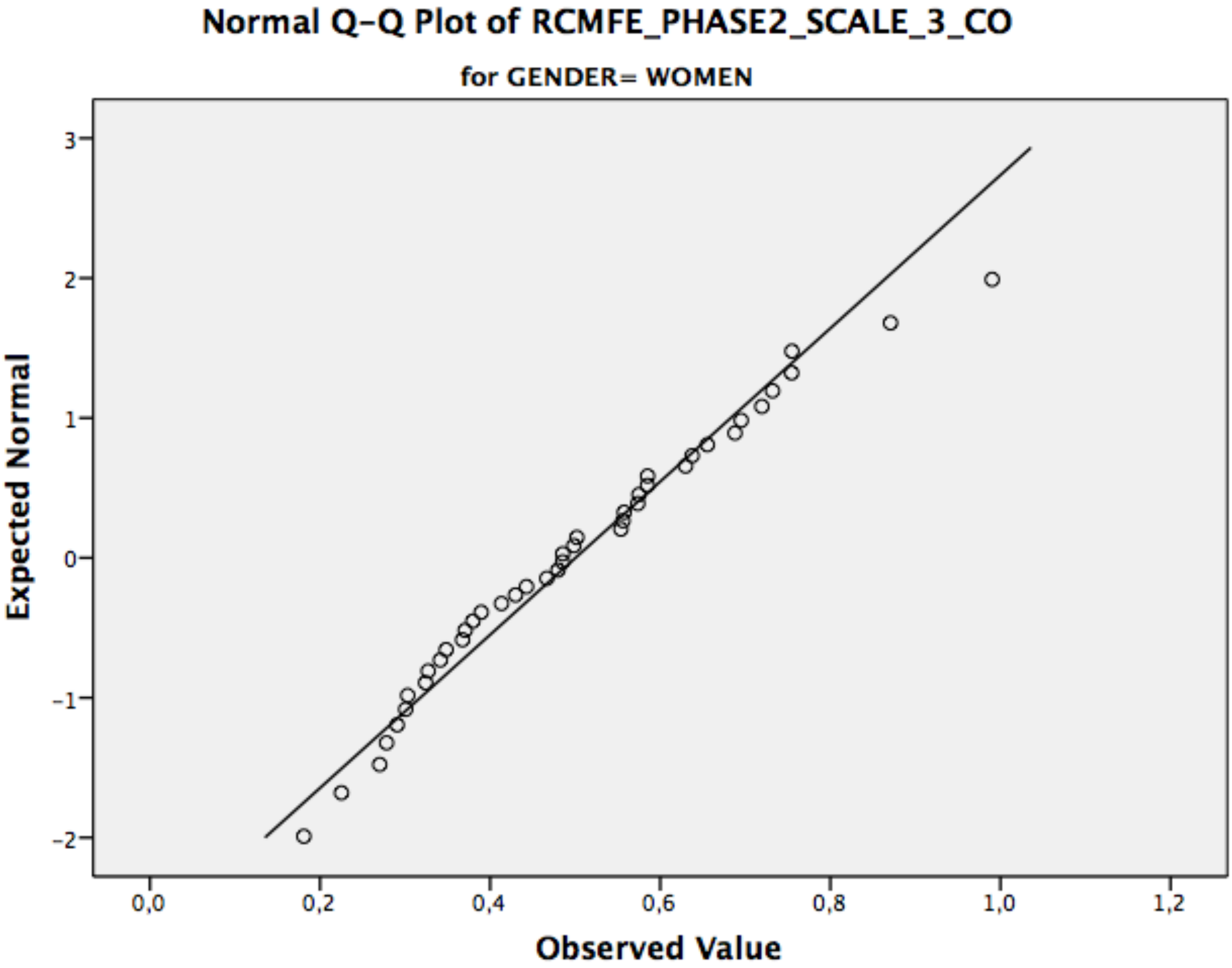

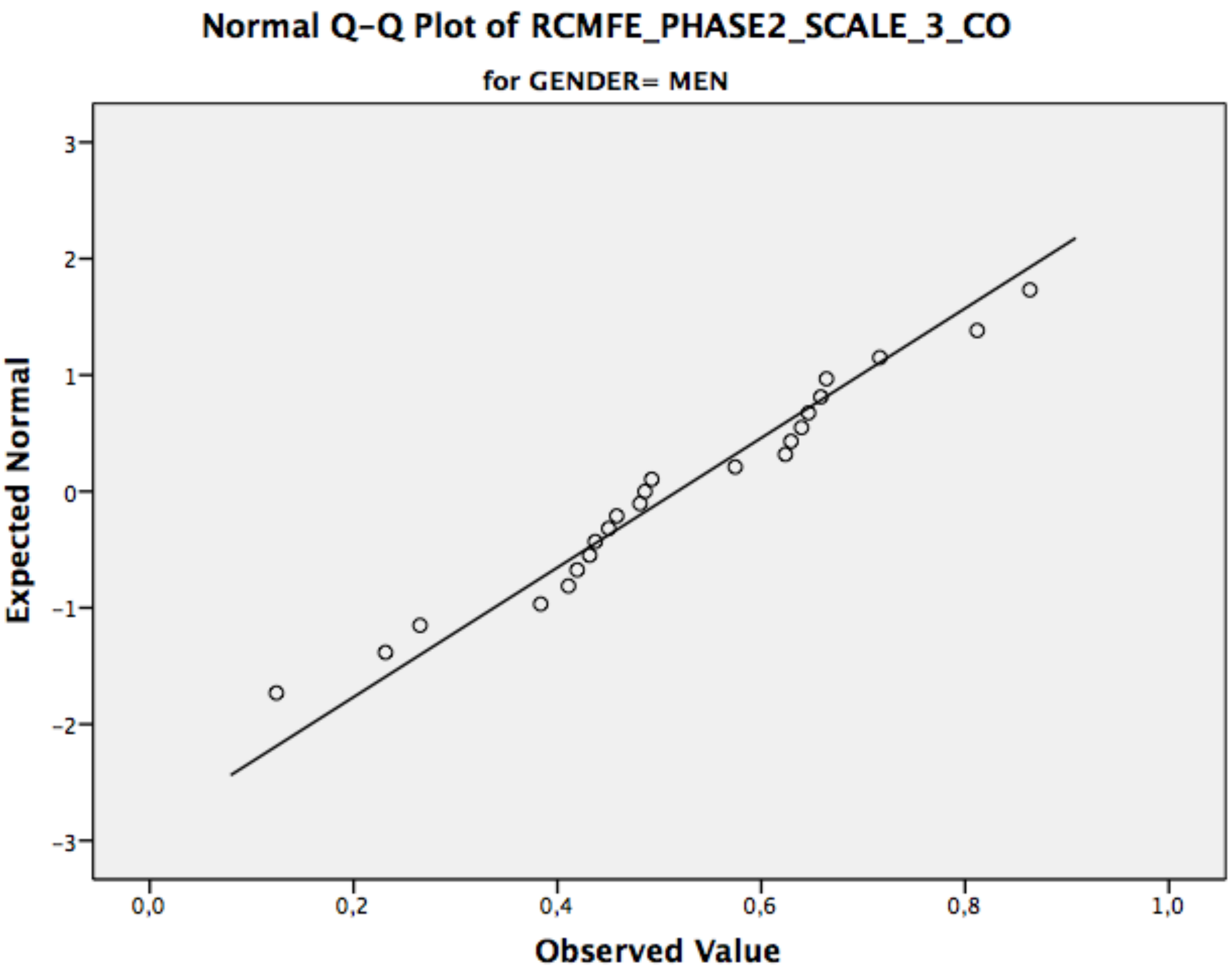
Normal Q-Q Plot of RCMFE_PHASE2_SCALE_3_CO
for GENDER= MEN
Expected Normal
3
2
1
0
-1
-2
-3
0,0
0,2
0,4
0,6
0,8
1,0
Observed Value

RCMFE_PHASE2_SCALE_4_CO

Normal Q-Q Plots

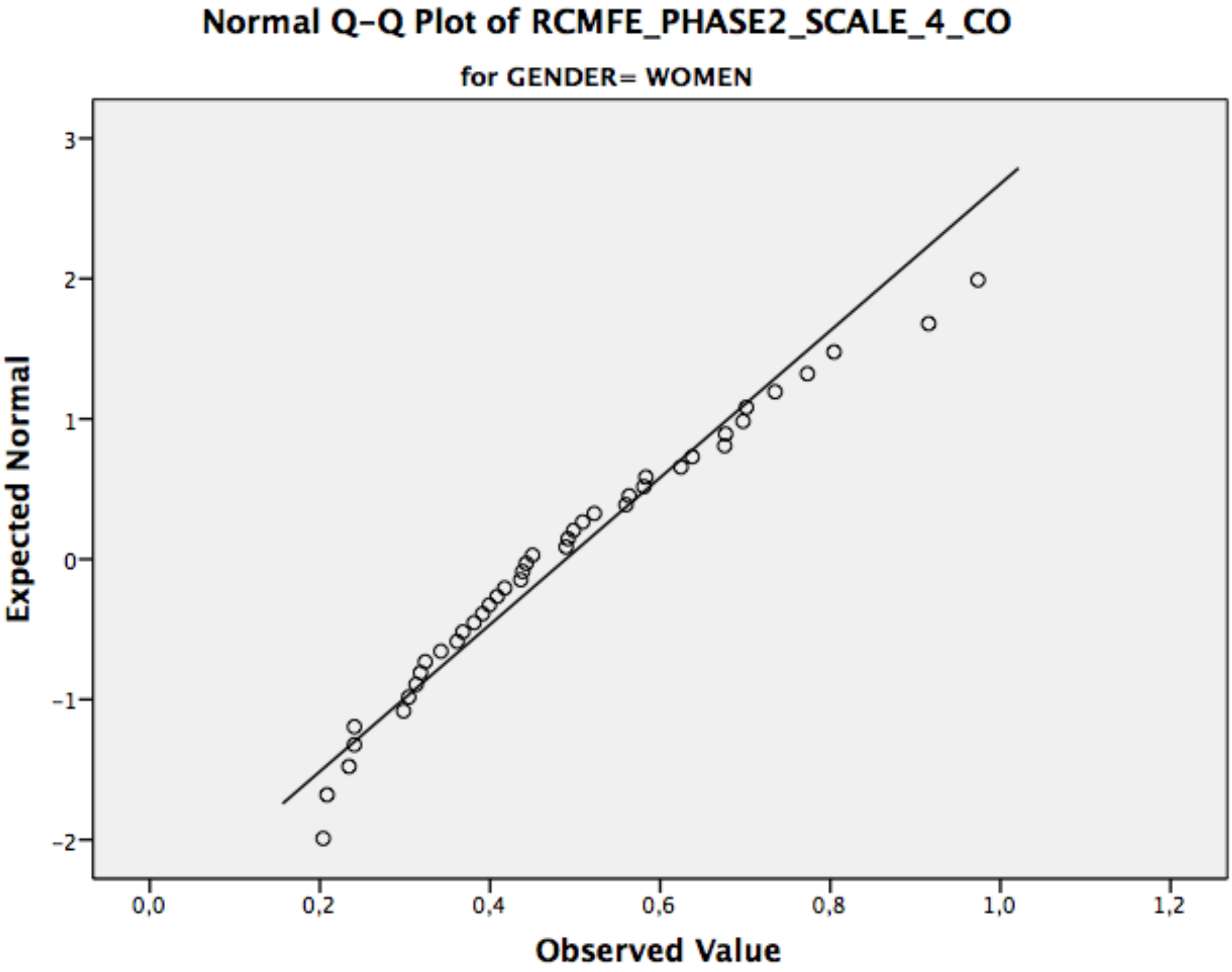

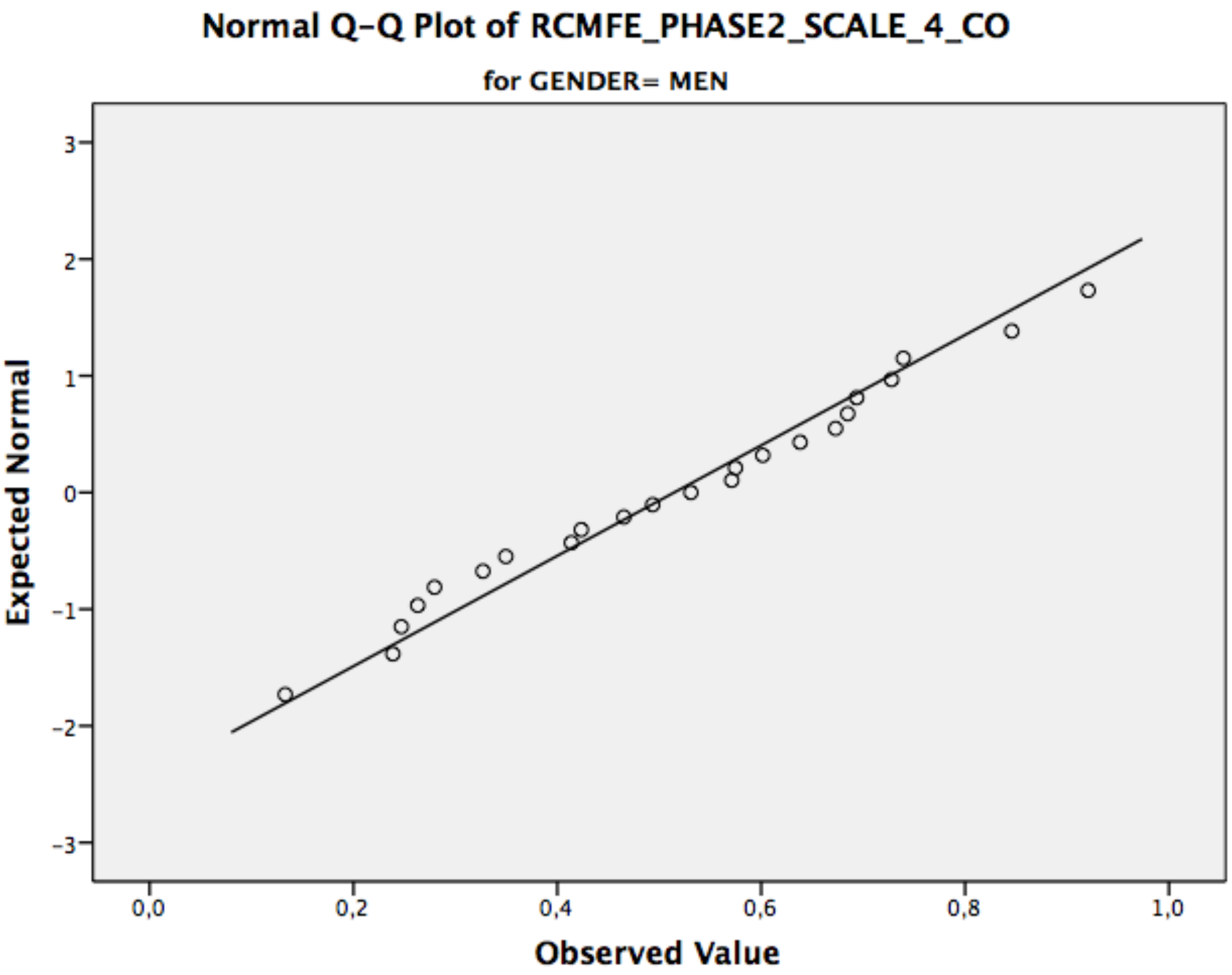
Normal Q-Q Plot of RCMFE_PHASE2_SCALE_4_CO
for GENDER= MEN
Expected Normal
Observed Value
3
2
1
0
-1
-2
-3
0,0
0,2
0,4
0,6
0,8
1,0

RCMFE_PHASE2_SCALE_5_CO

Normal Q-Q Plots

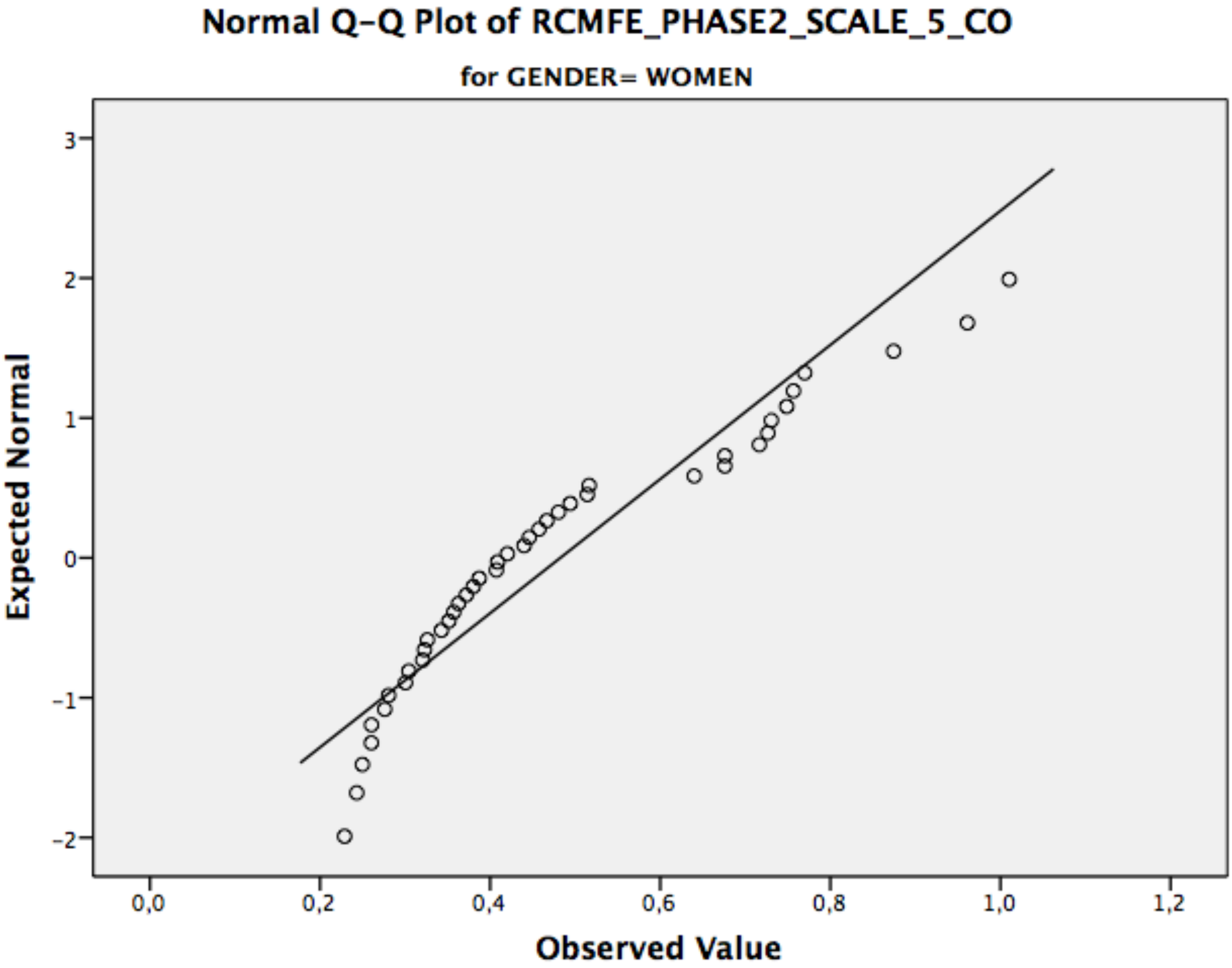

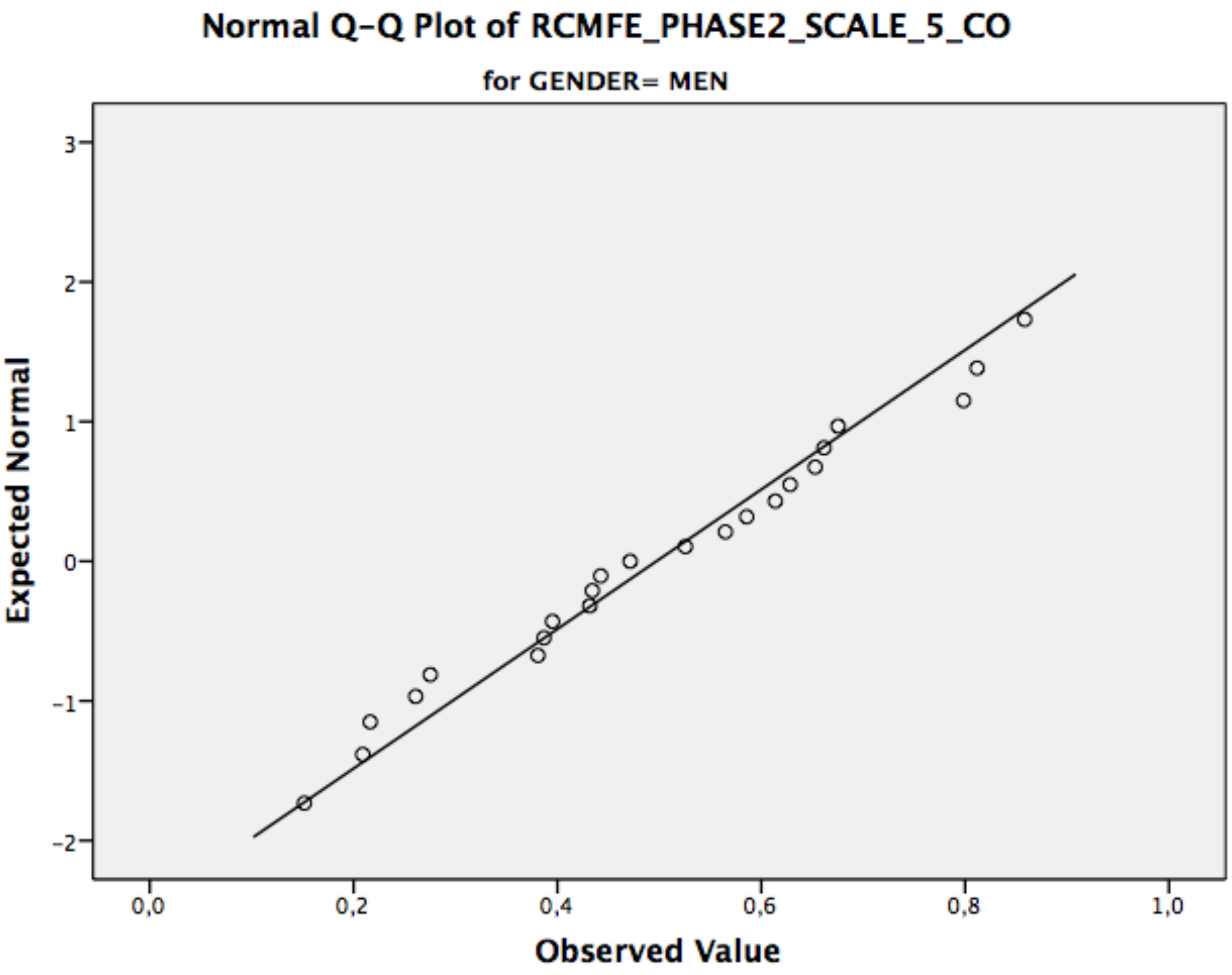
Normal Q-Q Plot of RCMFE_PHASE2_SCALE_5_CO
for GENDER= MEN
Expected Normal
Observed Value
3
2
1
0
-1
-2
0,0
0,2
0,4
0,6
0,8
1,0

RCMFE_PHASE3_SCALE_1_CO

Normal Q-Q Plots

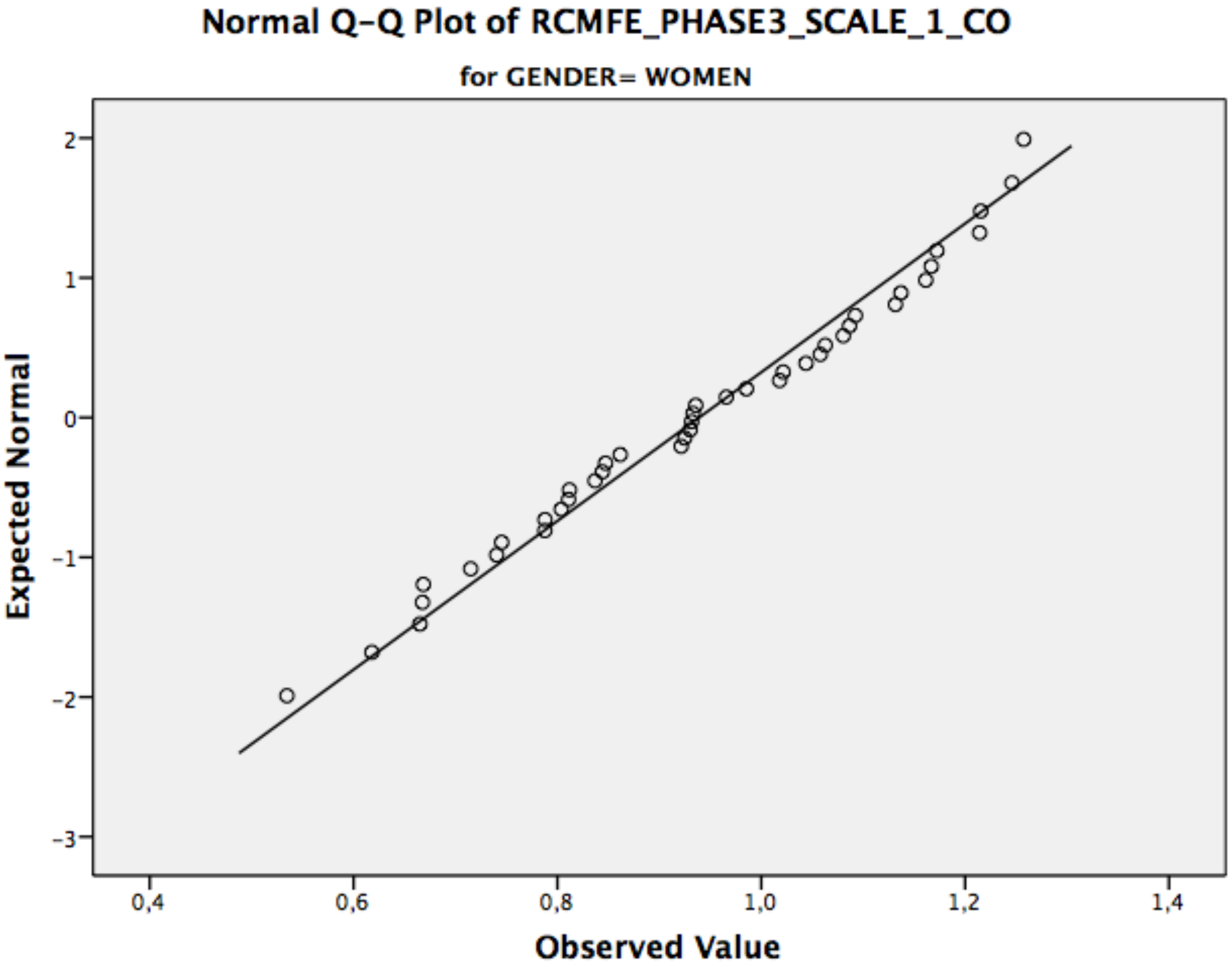

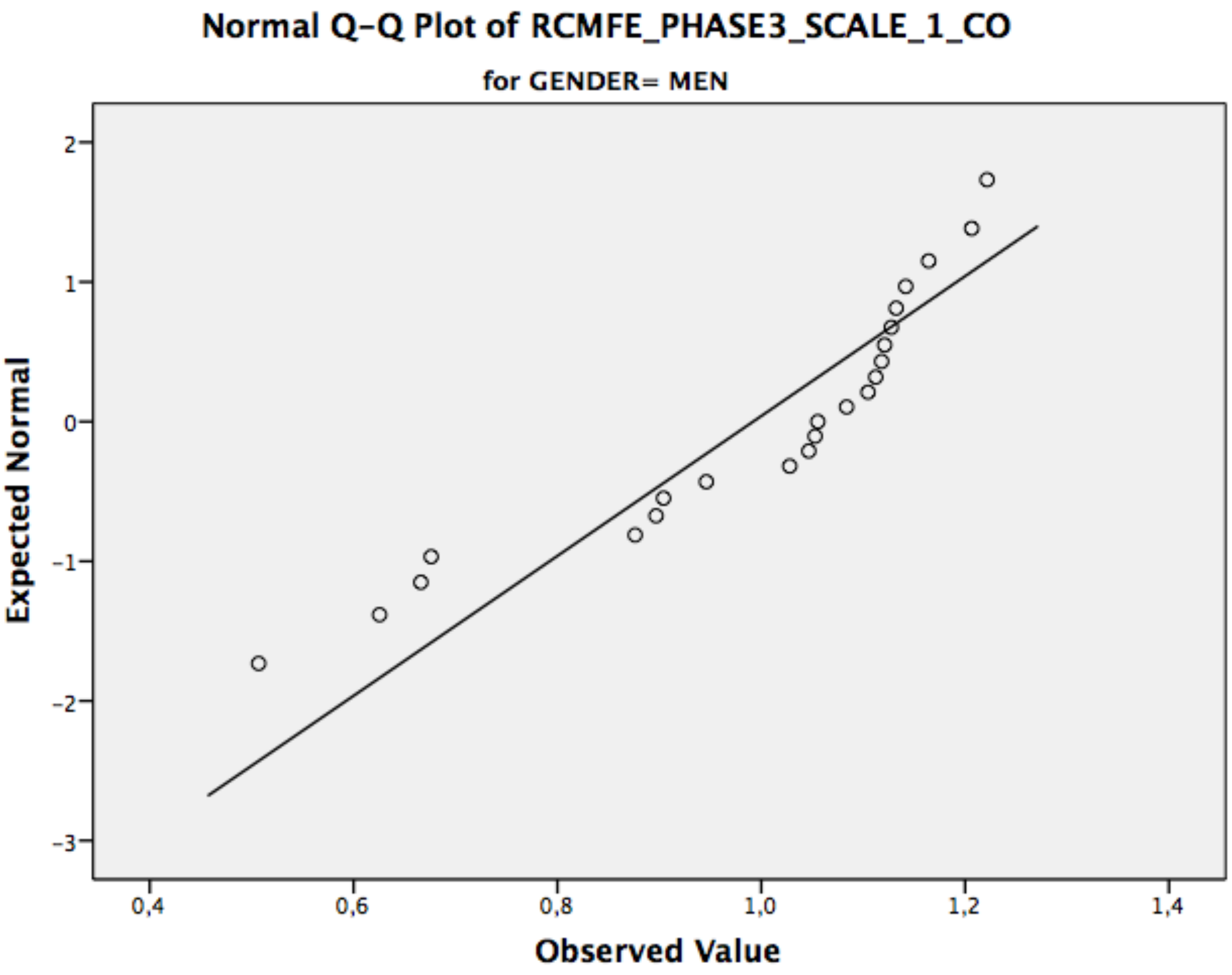

Normal Q-Q Plot of RCMFE_PHASE3_SCALE_1_CO
for GENDER= MEN
Expected Normal
Observed Value
2
1
0
-1
-2
-3
0,4
0,6
0,8
1,0
1,2
1,4

RCMFE_PHASE3_SCALE_2_CO

Normal Q-Q Plots

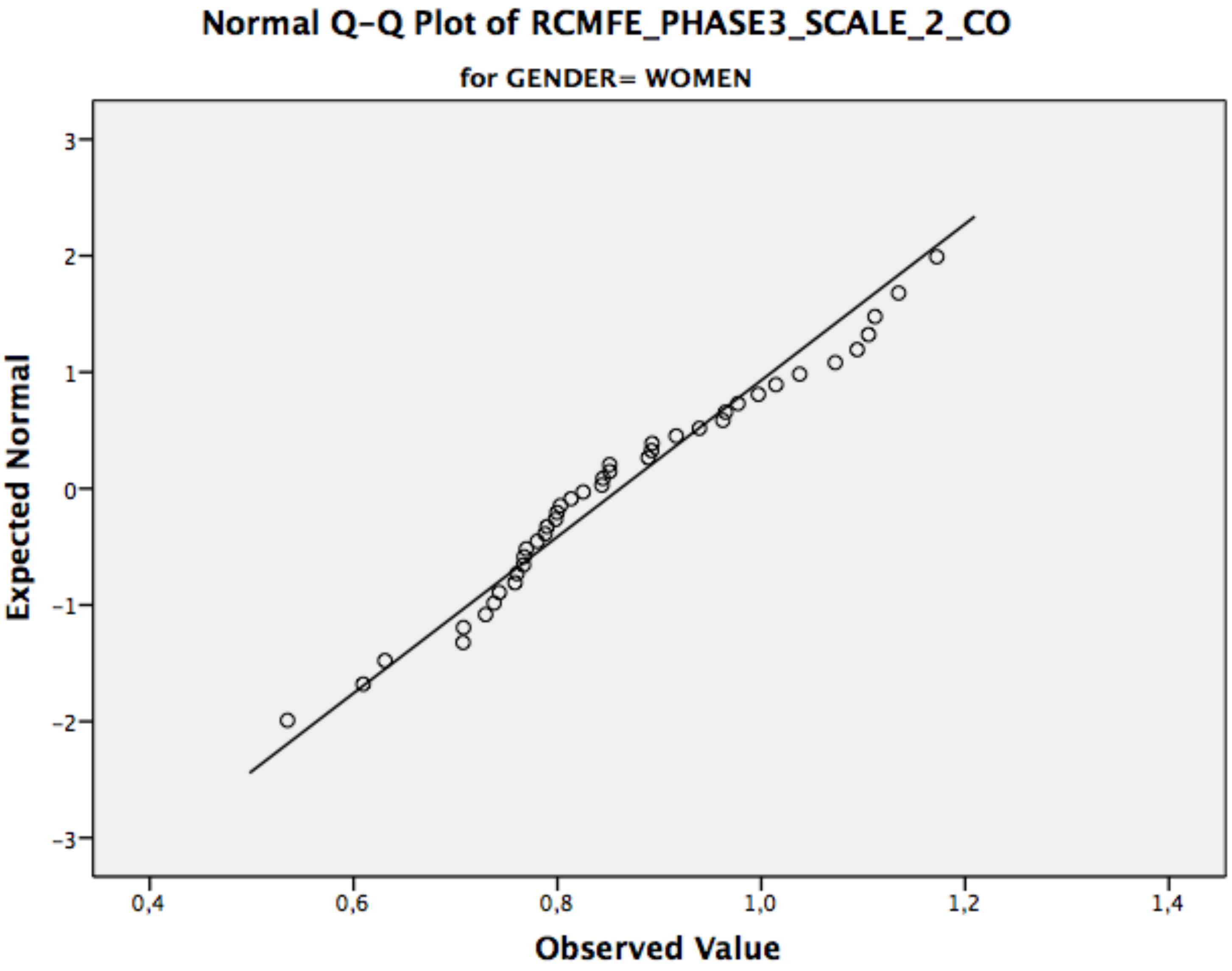

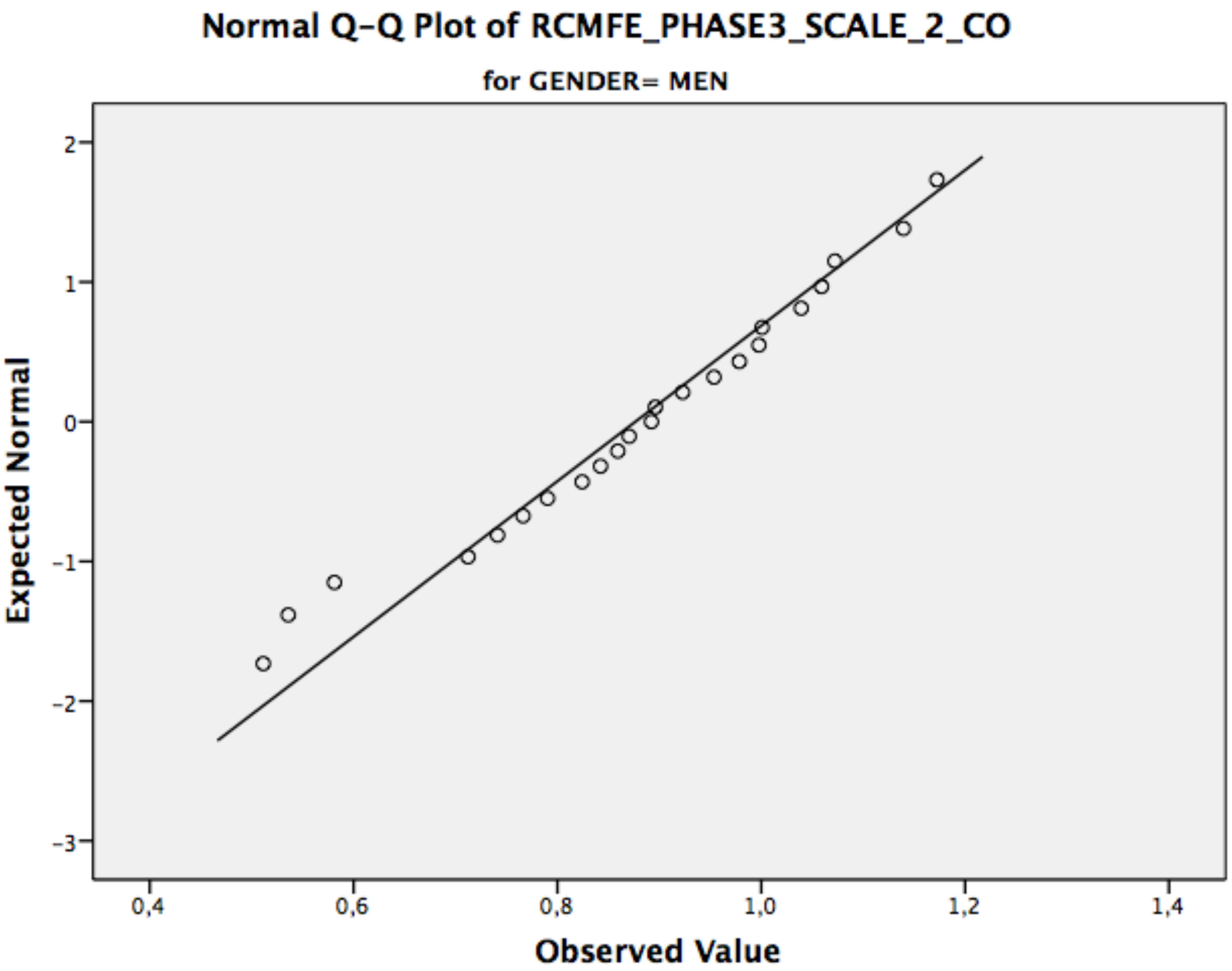

Normal Q-Q Plot of RCMFE_PHASE3_SCALE_2_CO
for GENDER= MEN
Expected Normal
2
1
0
-1
-2
-3
0,4
0,6
0,8
1,0
1,2
1,4
Observed Value

RCMFE_PHASE3_SCALE_3_CO

Normal Q-Q Plots

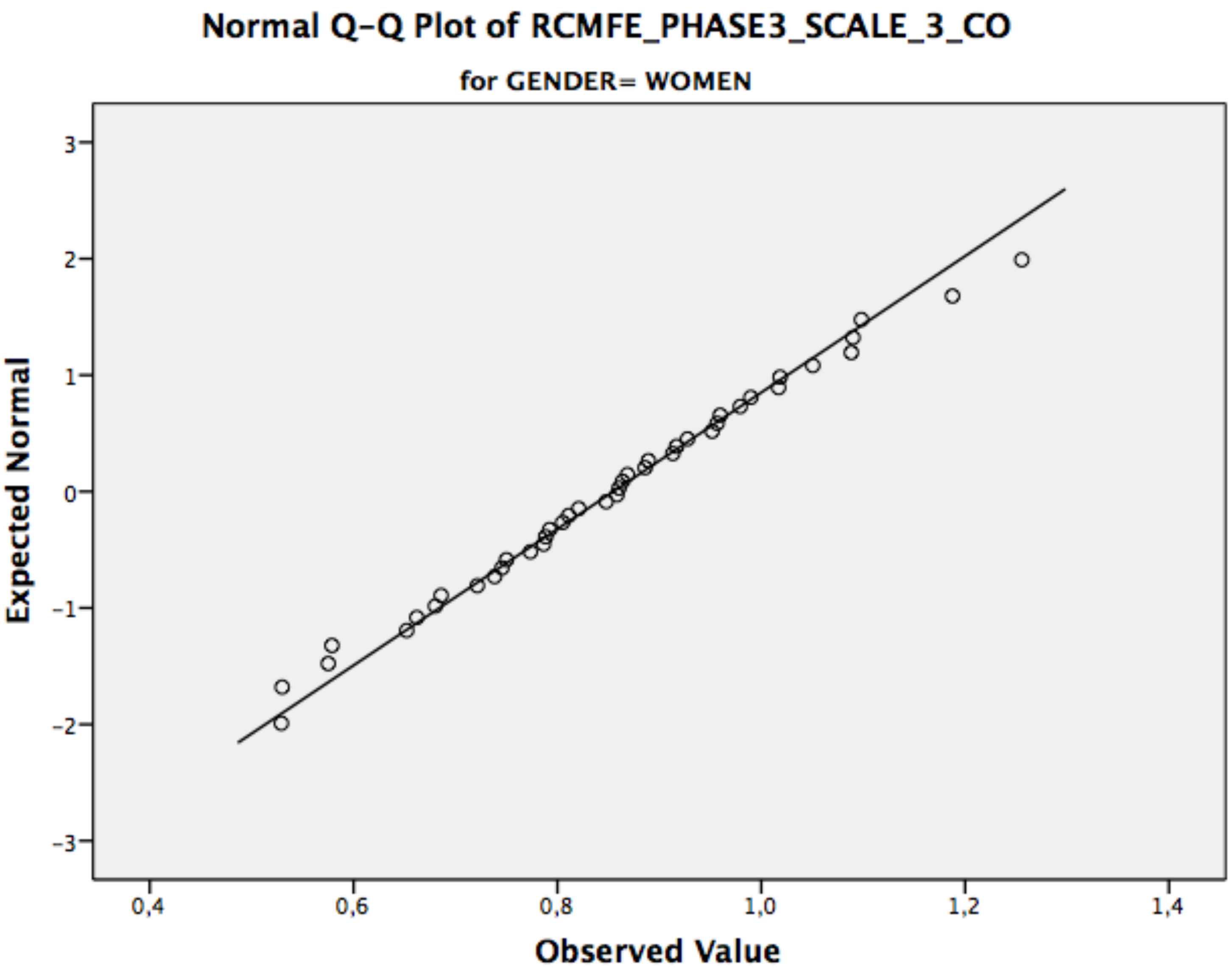

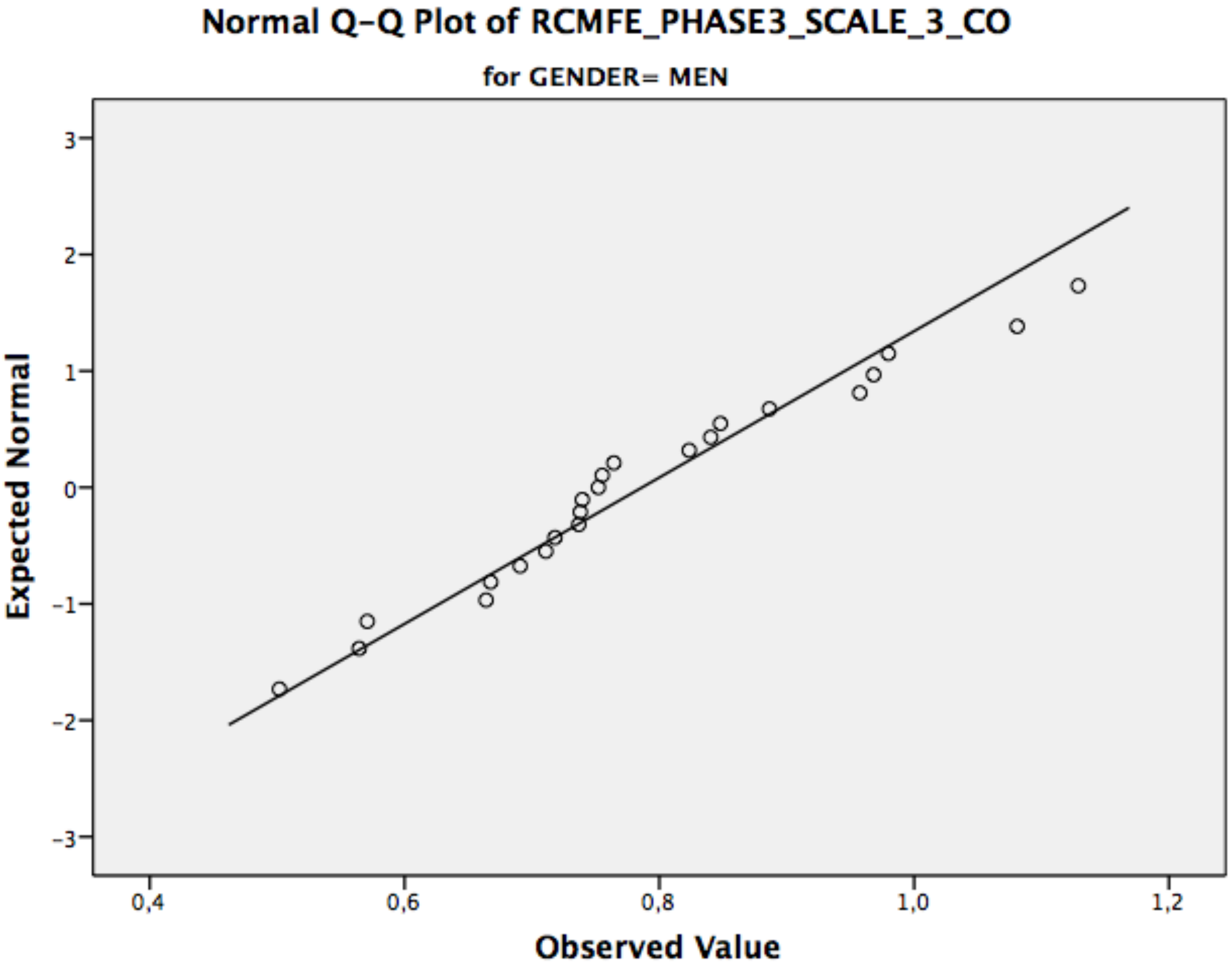
Normal Q-Q Plot of RCMFE_PHASE3_SCALE_3_CO
for GENDER= MEN
Expected Normal
3
2
1
0
-1
-2
-3
0,4
0,6
0,8
1,0
1,2
Observed Value

RCMFE_PHASE3_SCALE_4_CO

Normal Q-Q Plots

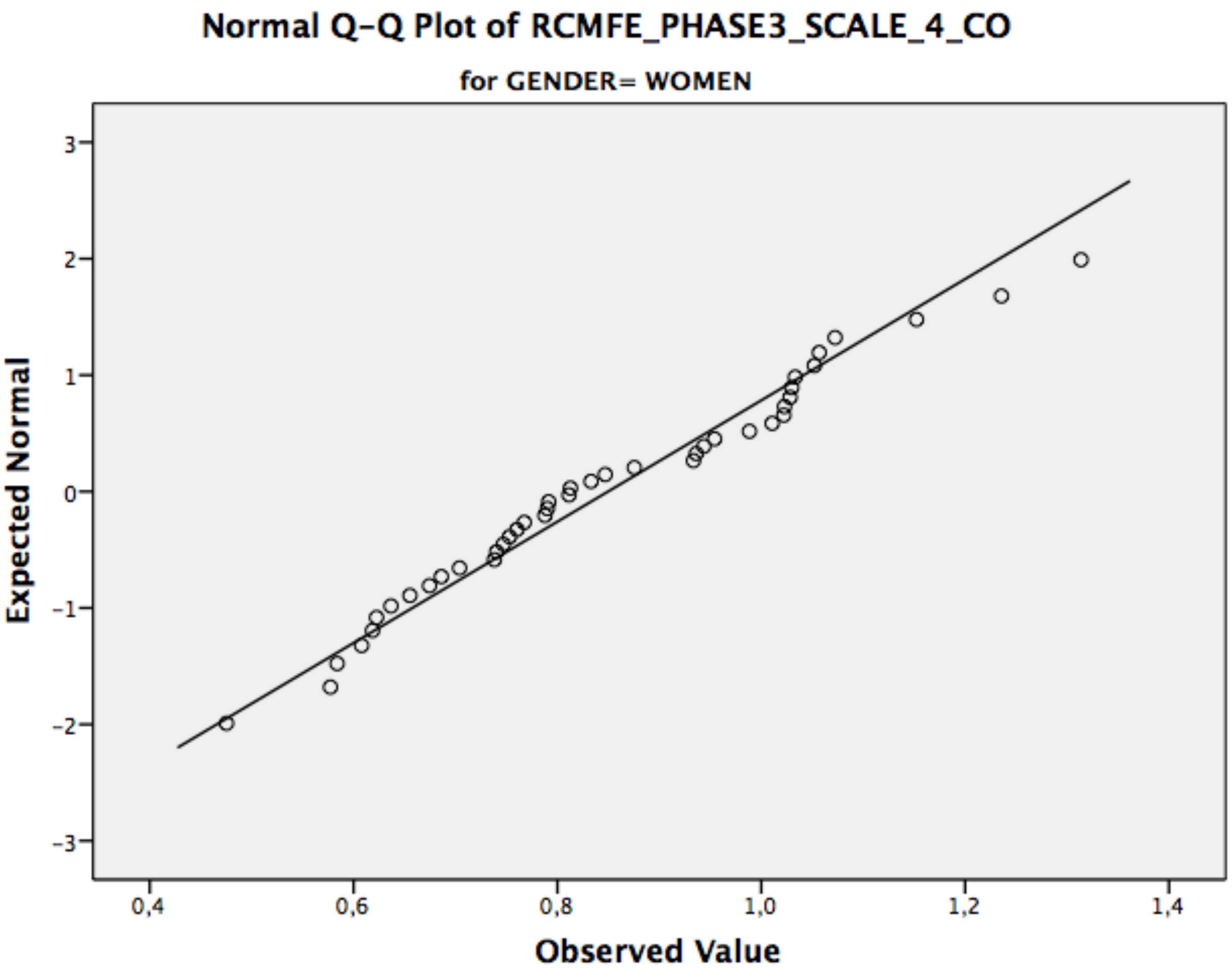

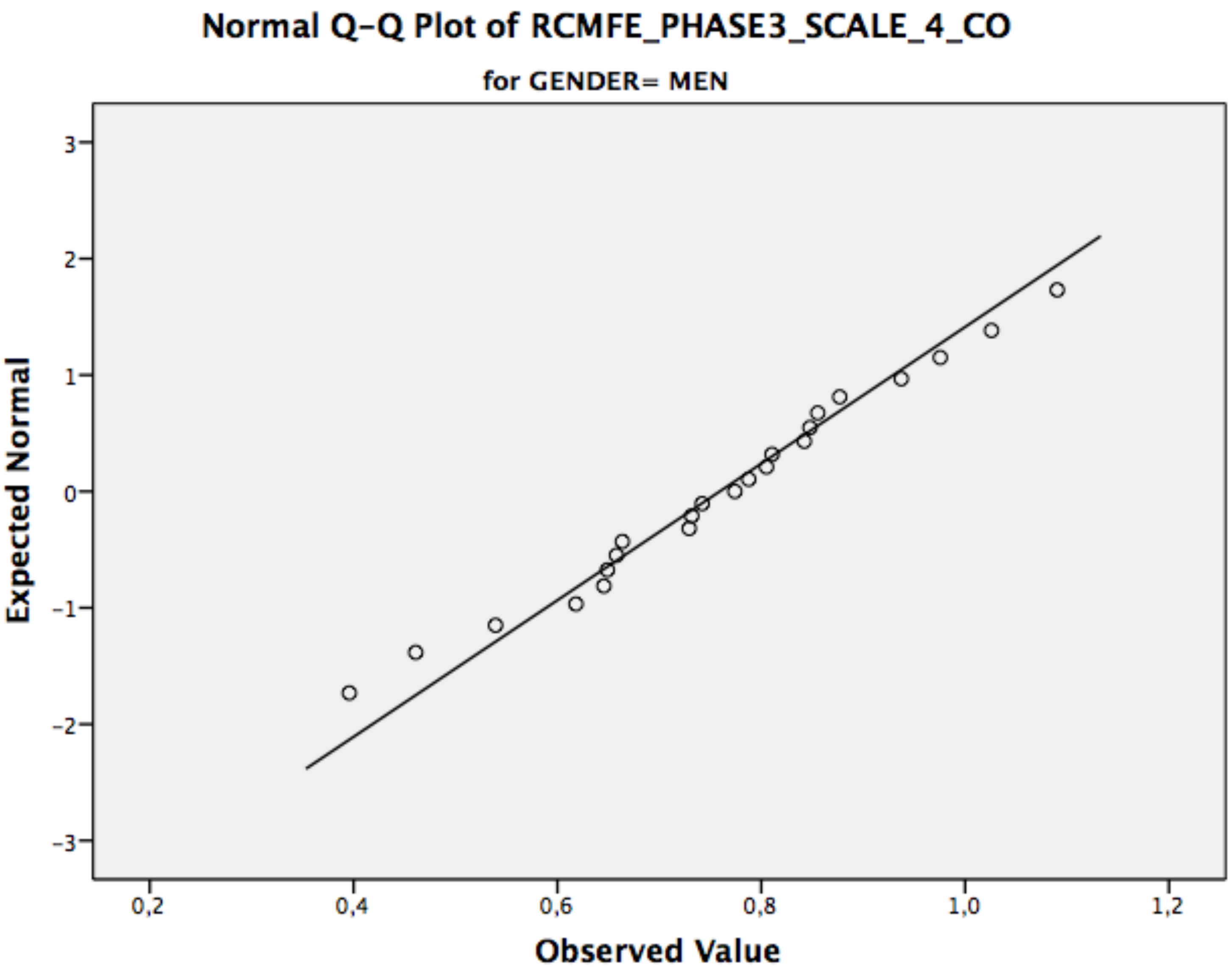
Normal Q-Q Plot of RCMFE_PHASE3_SCALE_4_CO
for GENDER= MEN
Expected Normal
3
2
1
0
-1
-2
-3
0,2
0,4
0,6
0,8
1,0
1,2
Observed Value

RCMFE_PHASE3_SCALE_5_CO

Normal Q-Q Plots

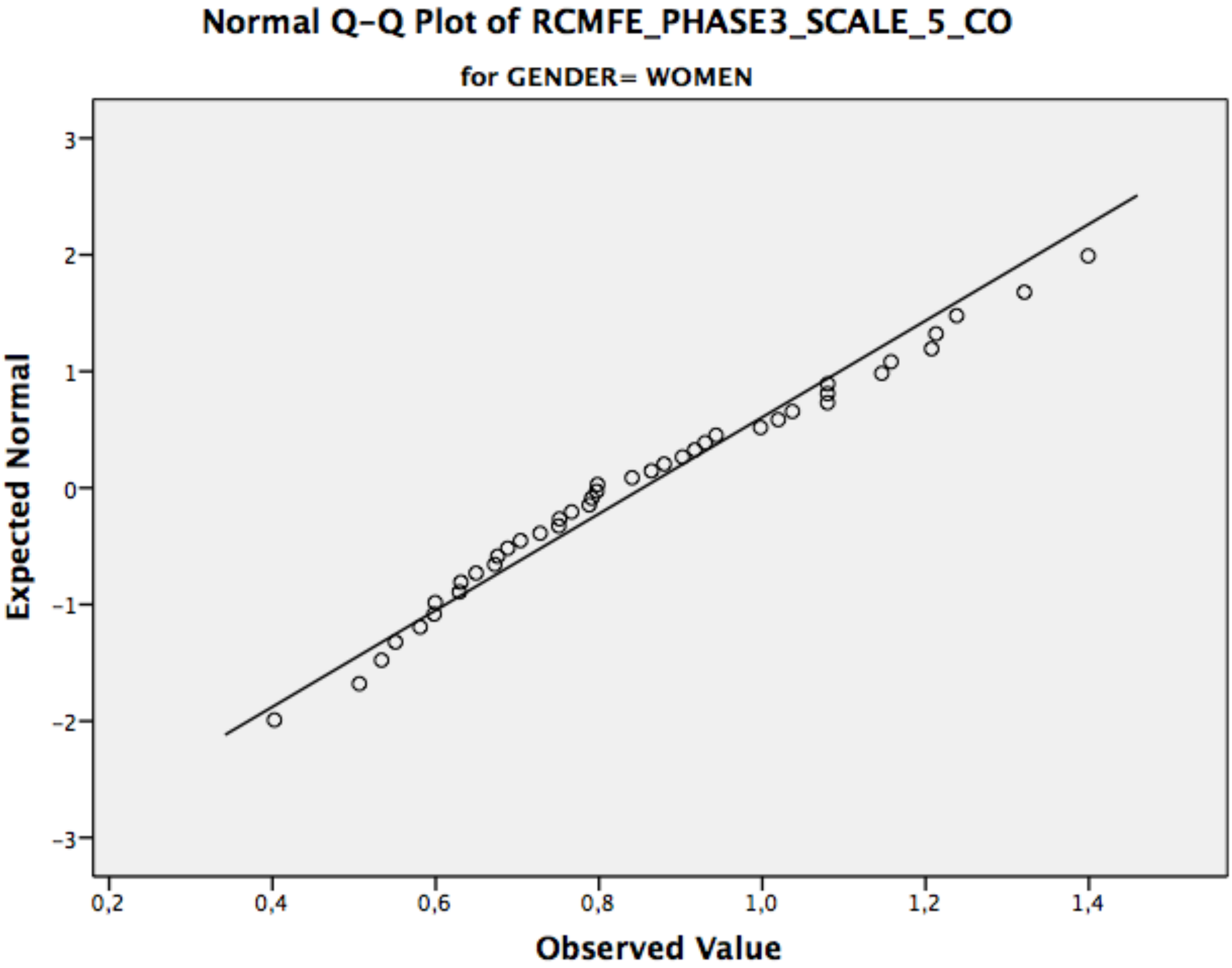

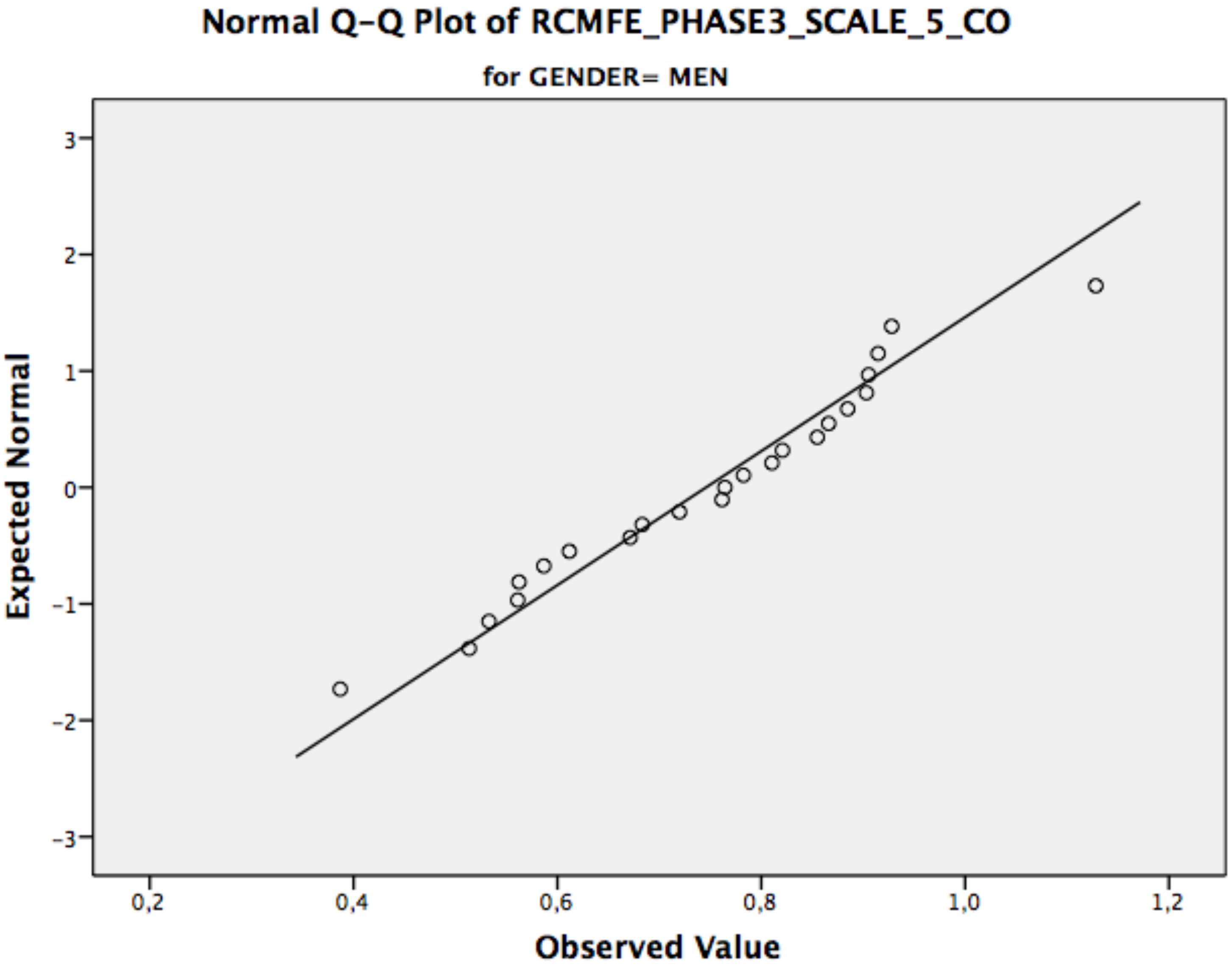
Normal Q-Q Plot of RCMFE_PHASE3_SCALE_5_CO
for GENDER= MEN
Expected Normal
Observed Value
3
2
1
0
-1
-2
-3
0,2
0,4
0,6
0,8
1,0
1,2

RCMFE_PHASE4_SCALE_1_CO

Normal Q-Q Plots

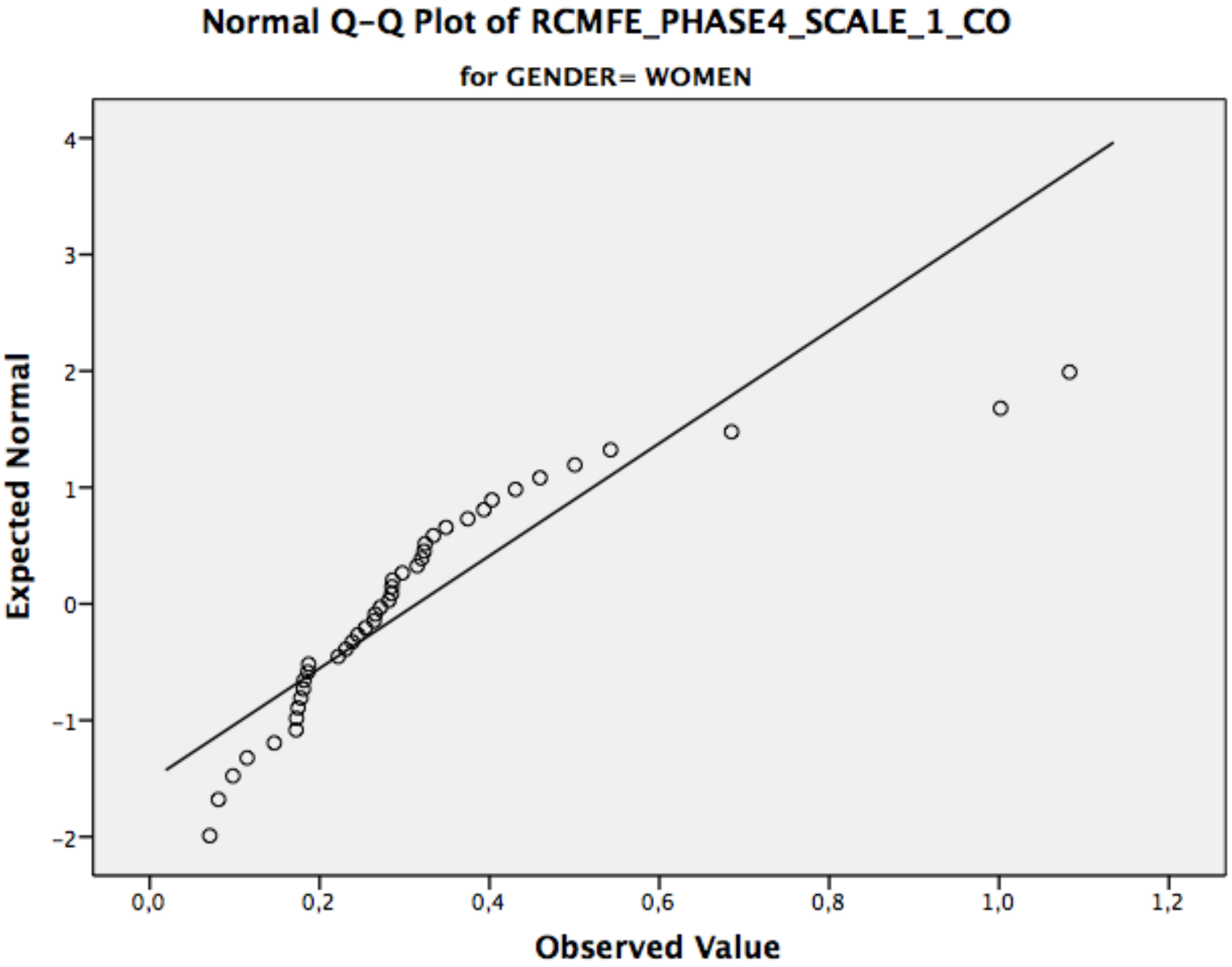

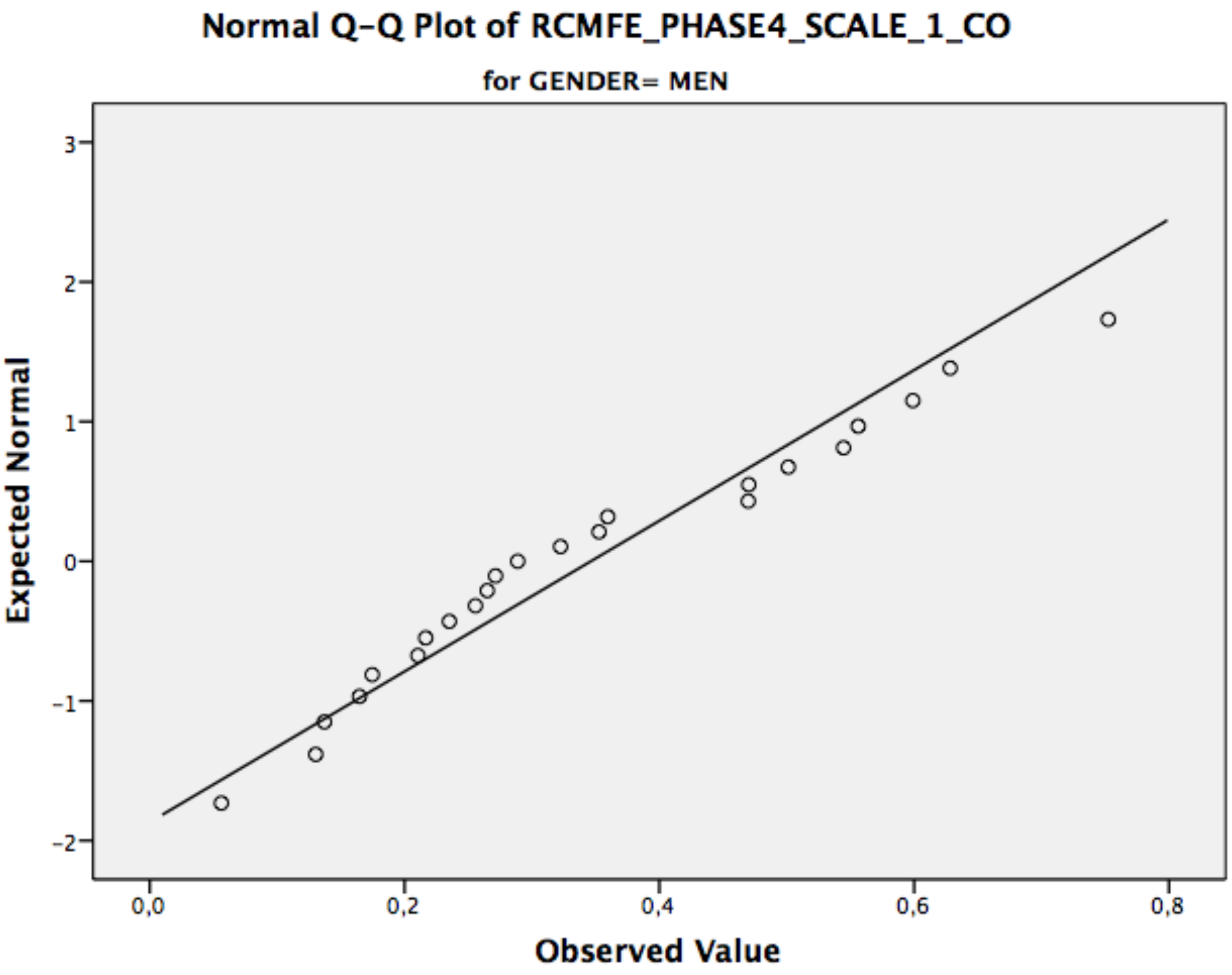

Normal Q-Q Plot of RCMFE_PHASE4_SCALE_1_CO
for GENDER= MEN
Expected Normal
Observed Value
3
2
1
0
-1
-2
0,0
0,2
0,4
0,6
0,8

RCMFE_PHASE4_SCALE_2_CO

Normal Q-Q Plots

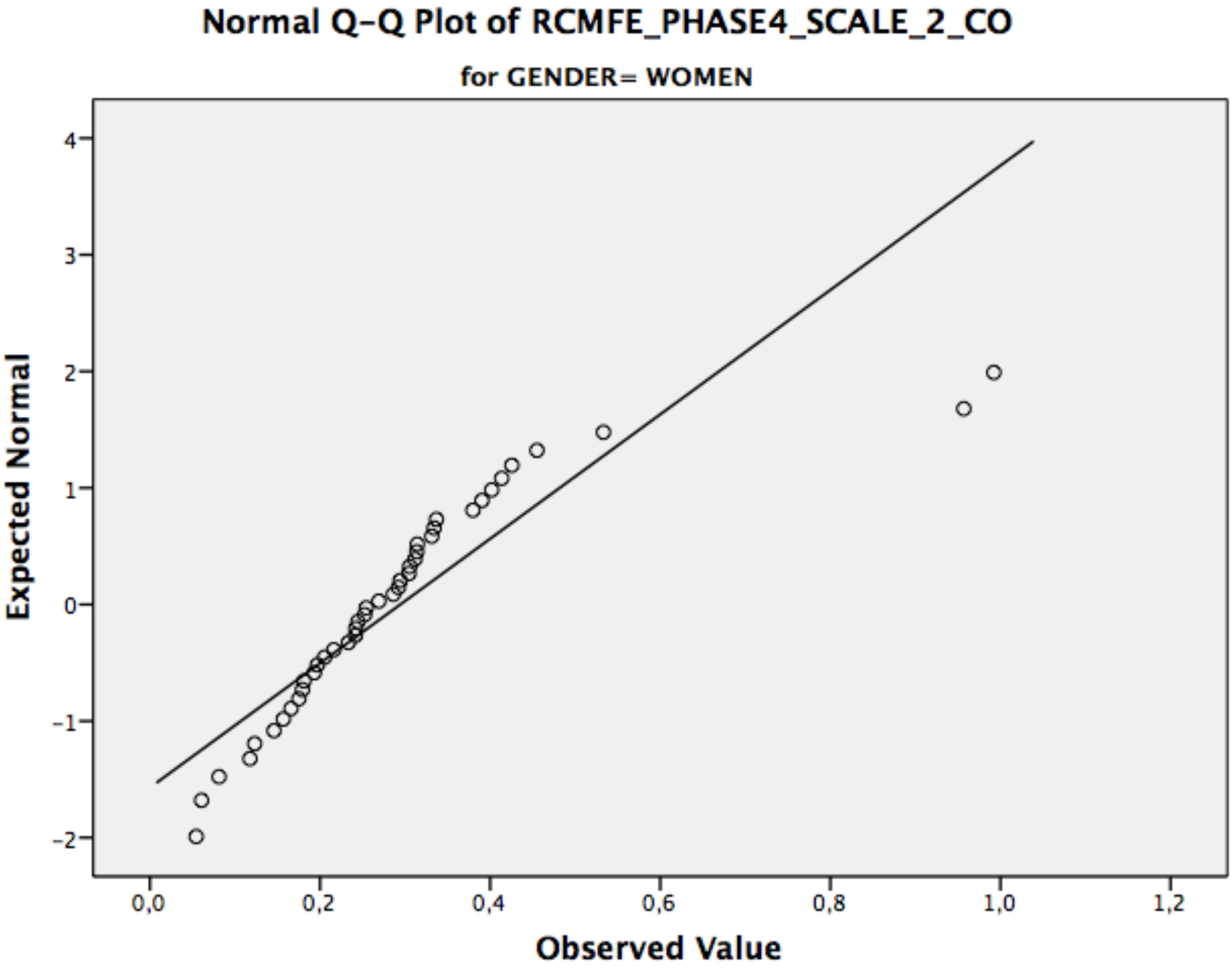

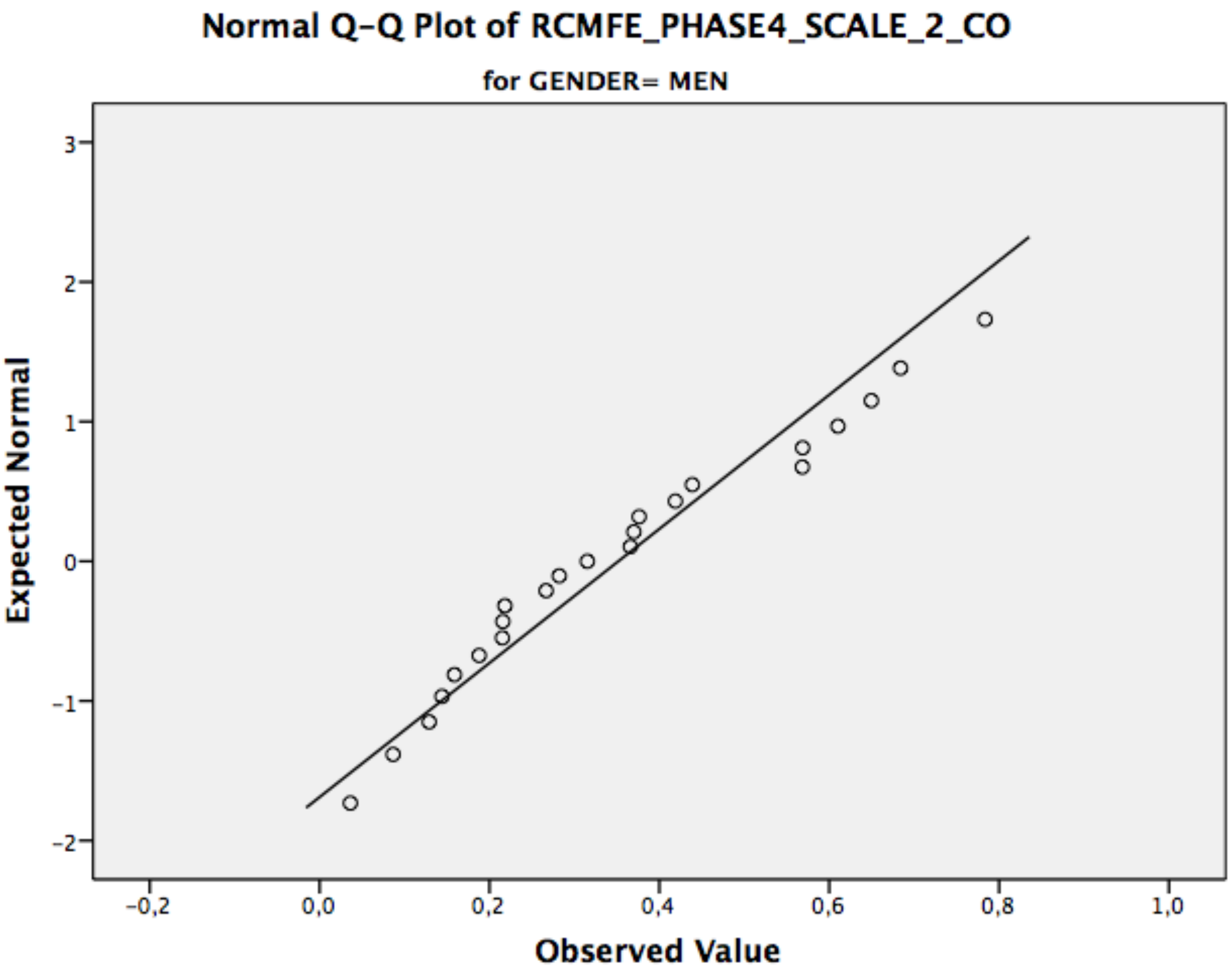
Normal Q-Q Plot of RCMFE_PHASE4_SCALE_2_CO
for GENDER= MEN
Expected Normal
Observed Value
3
2
1
0
−1
−2
−0,2
0,0
0,2
0,4
0,6
0,8
1,0

RCMFE_PHASE4_SCALE_3_CO

Normal Q-Q Plots

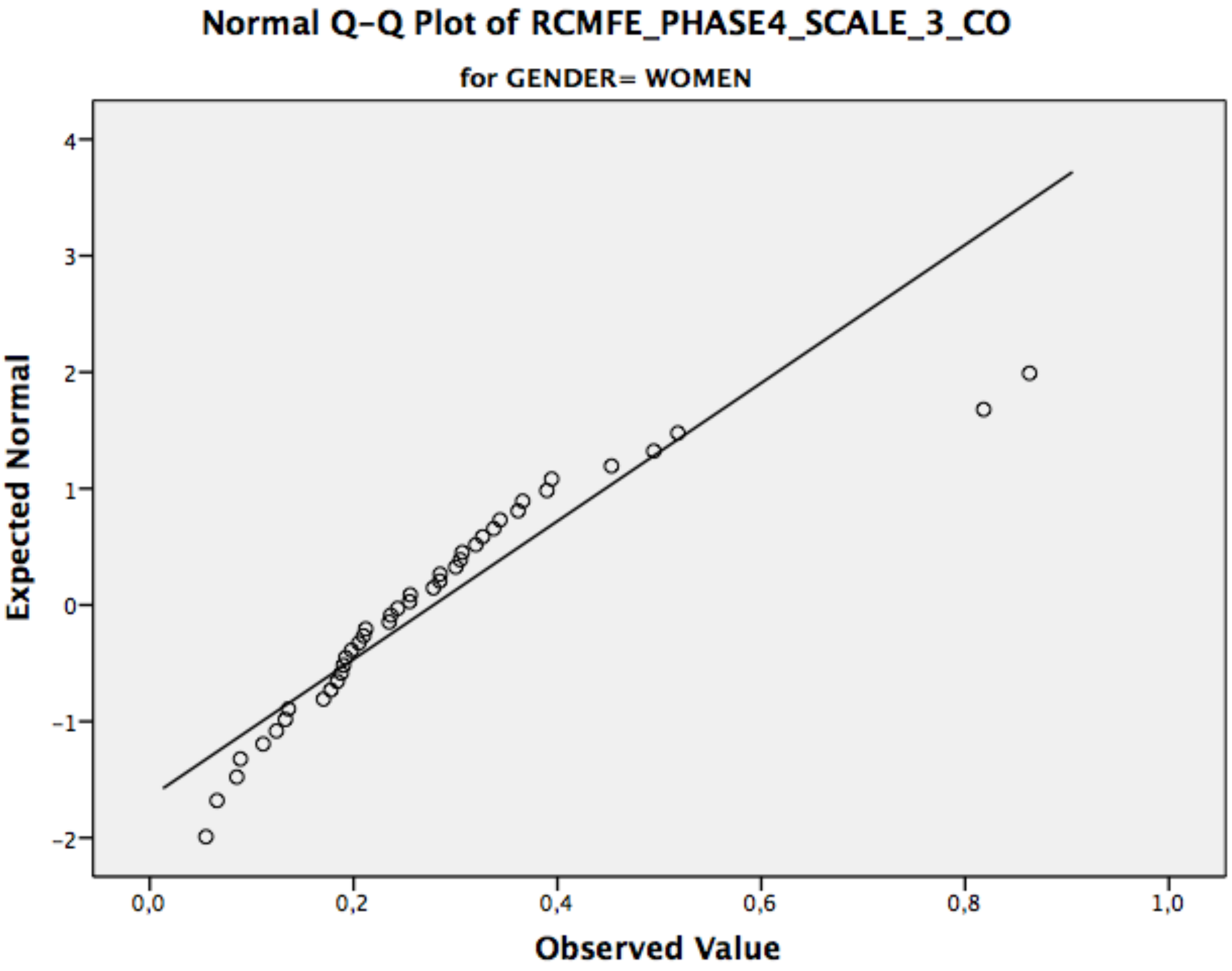

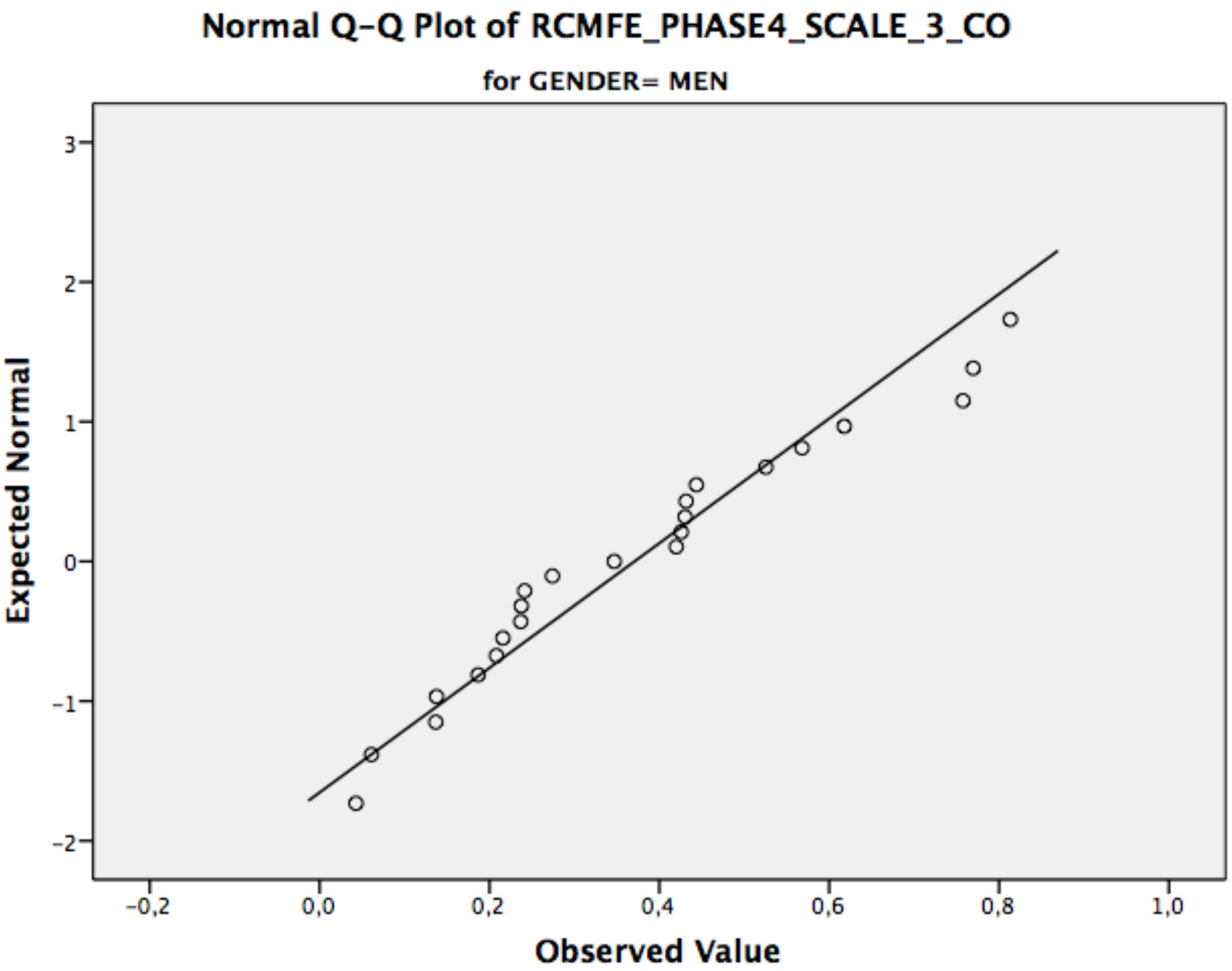
Normal Q-Q Plot of RCMFE_PHASE4_SCALE_3_CO
for GENDER= MEN
Expected Normal
Observed Value
3
2
1
0
-1
-2
-0,2
0,0
0,2
0,4
0,6
0,8
1,0

RCMFE_PHASE4_SCALE_4_CO

Normal Q-Q Plots

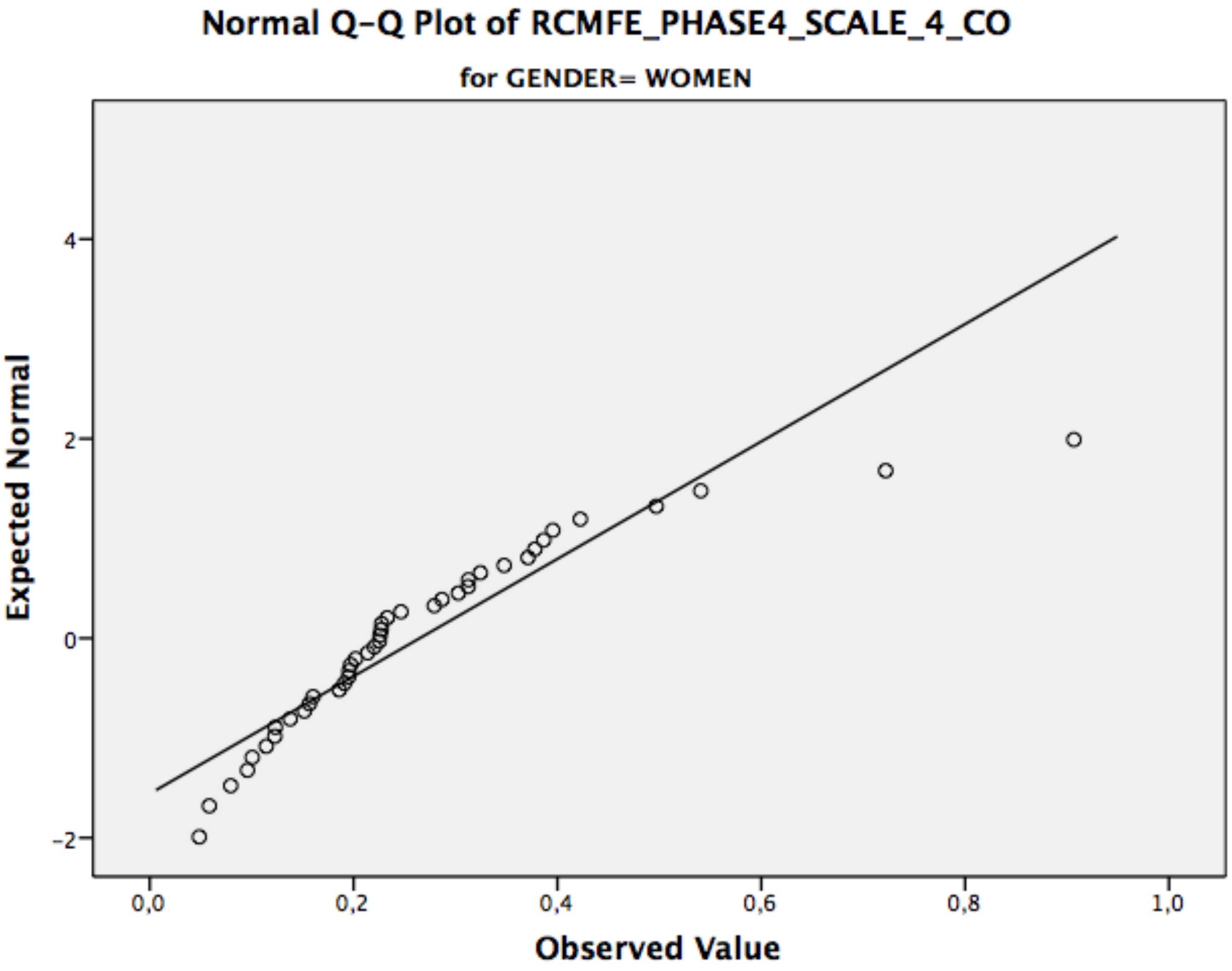

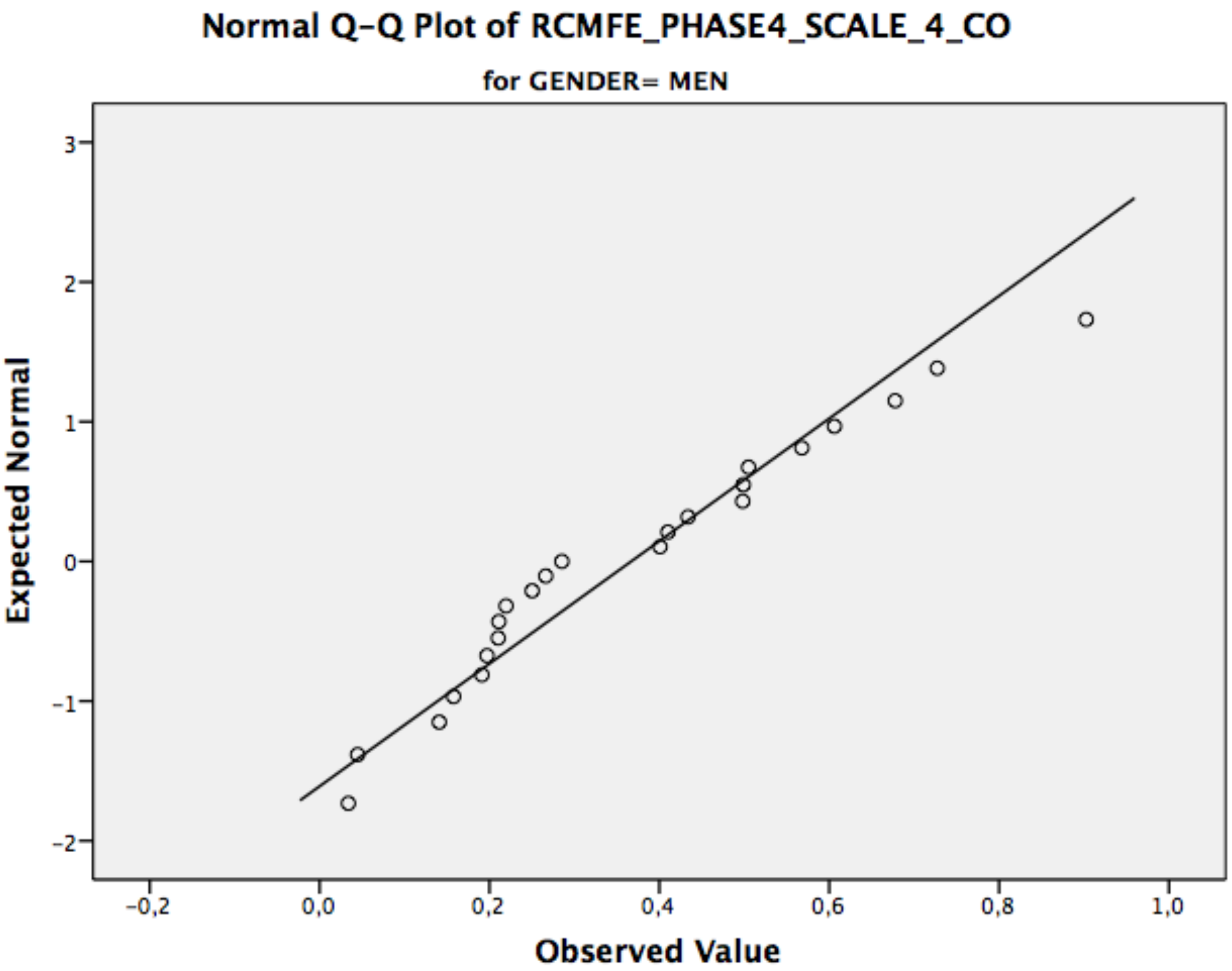

Normal Q-Q Plot of RCMFE_PHASE4_SCALE_4_CO
for GENDER= MEN
Expected Normal
Observed Value
3
2
1
0
-1
-2
-0,2
0,0
0,2
0,4
0,6
0,8
1,0

RCMFE_PHASE4_SCALE_5_CO

Normal Q-Q Plots

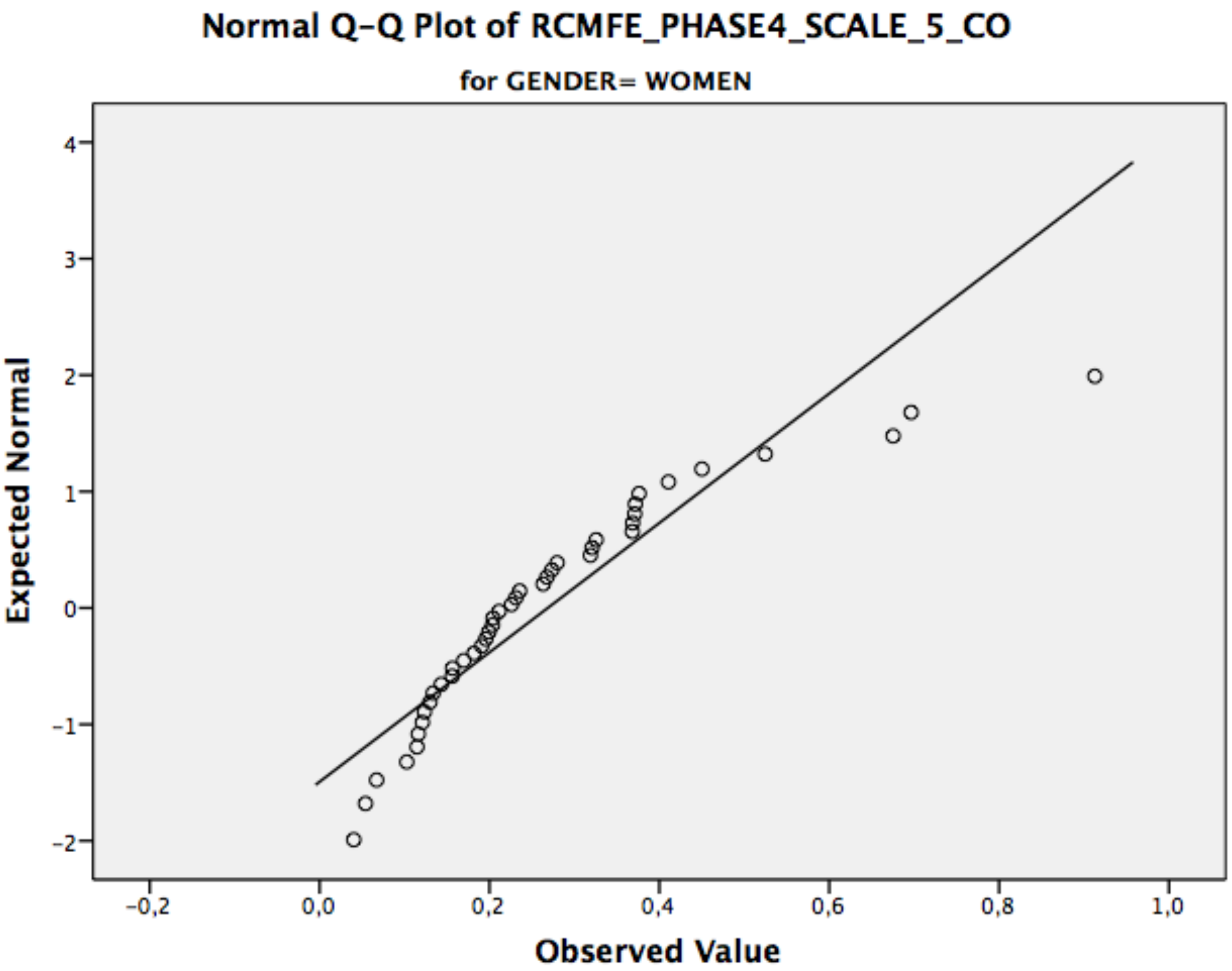

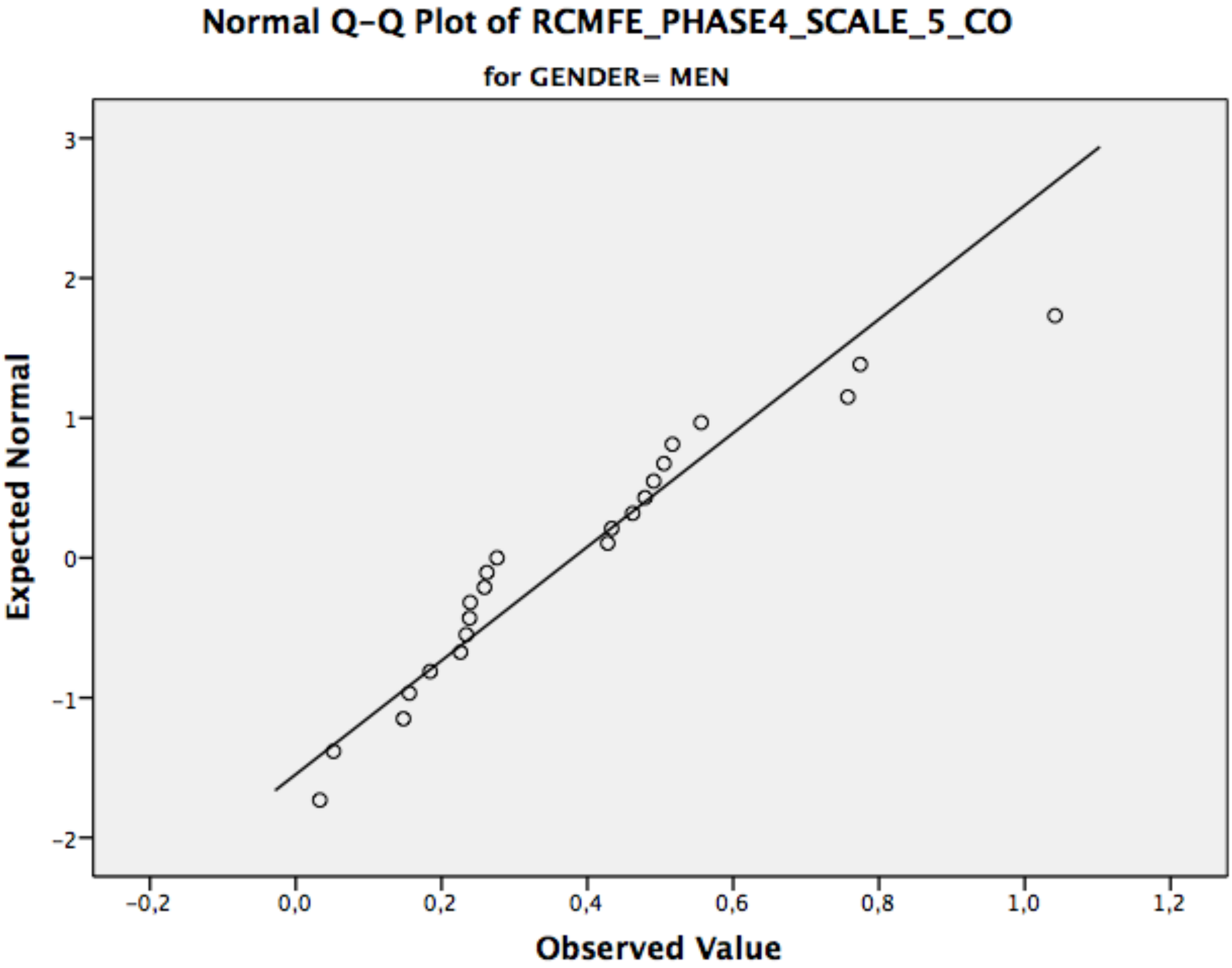

RCMFE_PHASE5_SCALE_1_CO

Normal Q-Q Plots

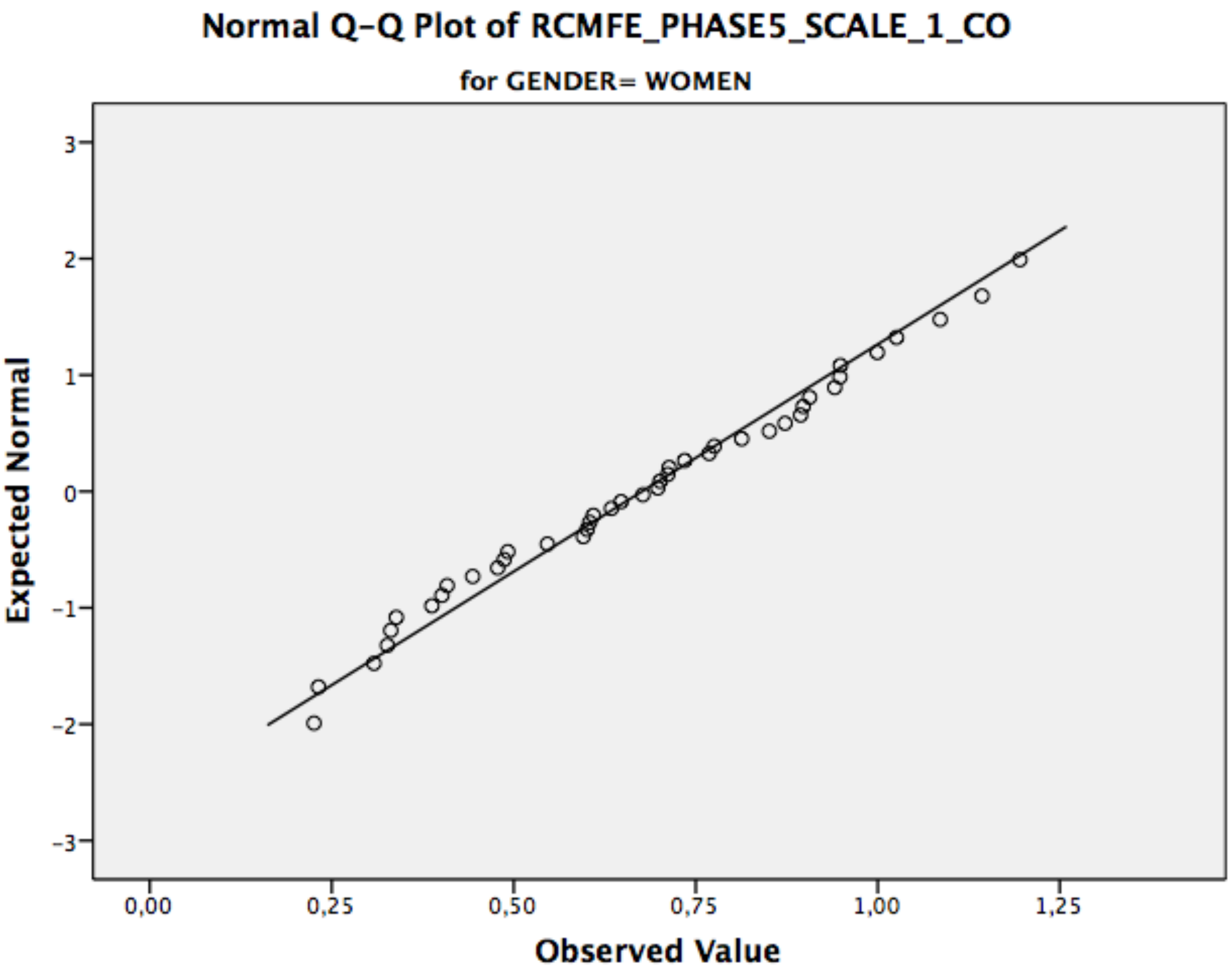

Normal Q-Q Plot of RCMFE_PHASE5_SCALE_1_CO
for GENDER= WOMEN
Expected Normal
3
2
1
0
-1
-2
-3
0,00
0,25
0,50
0,75
1,00
1,25
Observed Value

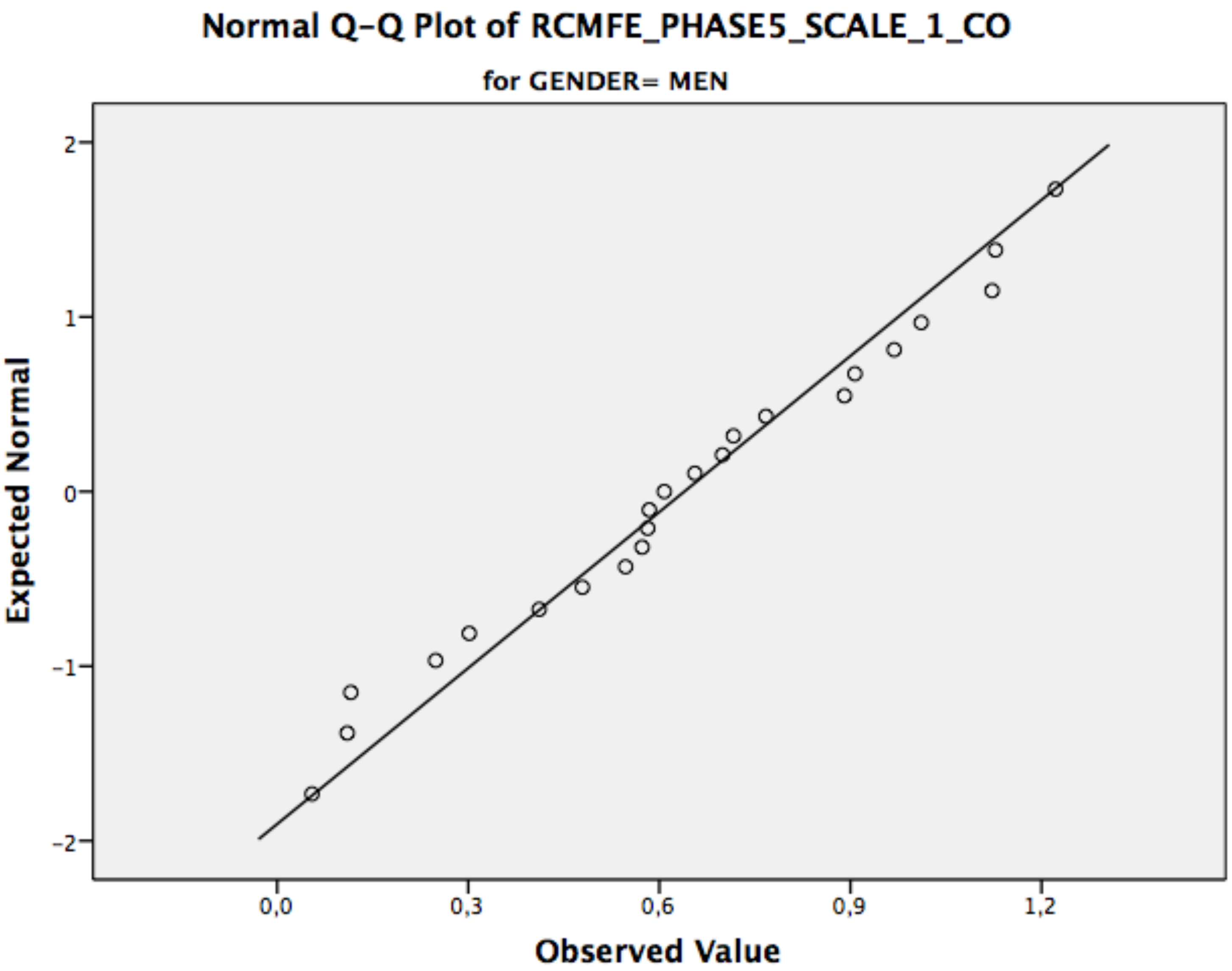
Normal Q-Q Plot of RCMFE_PHASE5_SCALE_1_CO
for GENDER= MEN
Expected Normal
2
1
0
-1
-2
0,0
0,3
0,6
0,9
1,2
Observed Value

RCMFE_PHASE5_SCALE_2_CO

Normal Q-Q Plots

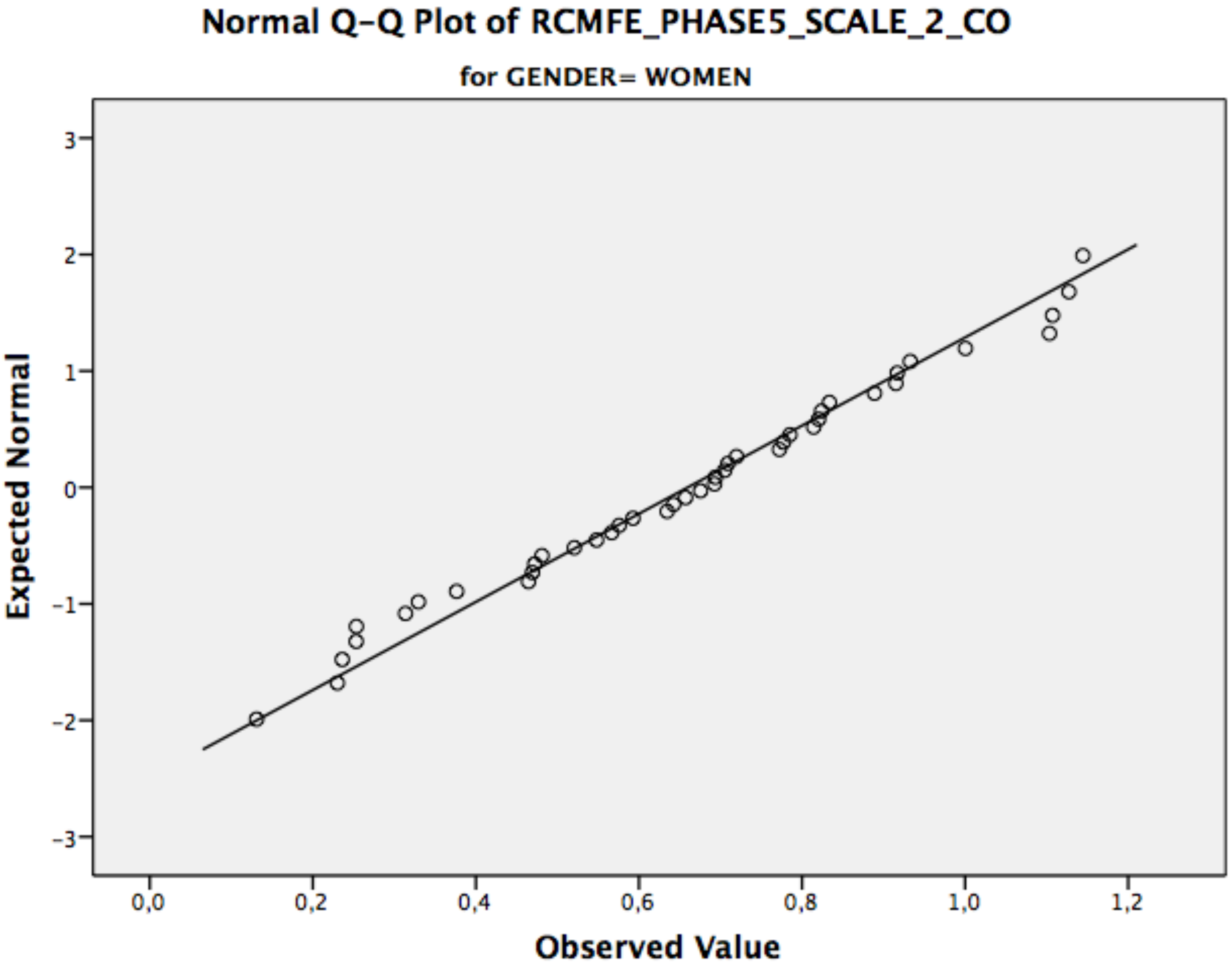

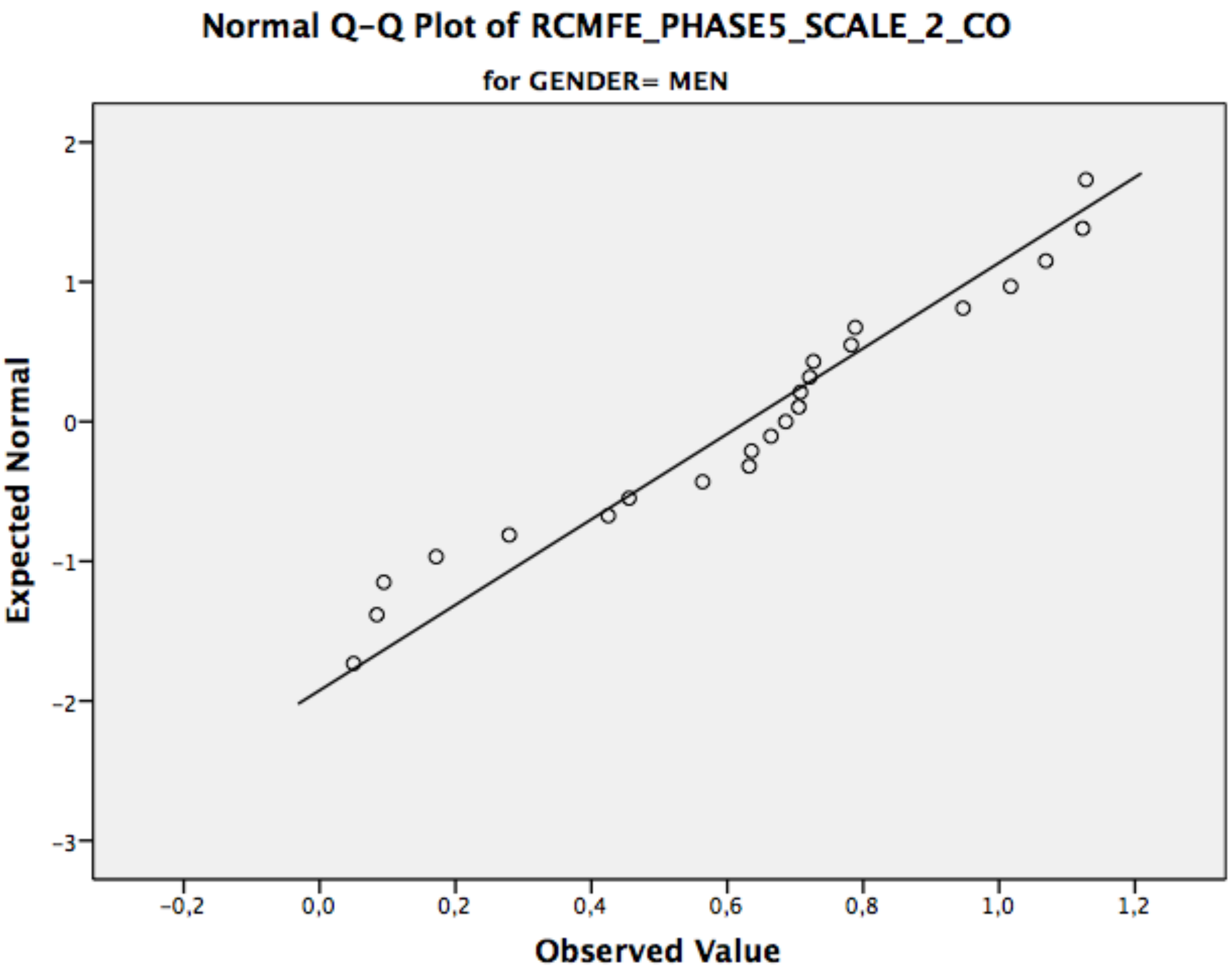

Normal Q-Q Plot of RCMFE_PHASE5_SCALE_2_CO
for GENDER= MEN
Expected Normal
2
1
0
−1
−2
−3
−0,2
0,0
0,2
0,4
0,6
0,8
1,0
1,2
Observed Value

RCMFE_PHASE5_SCALE_3_CO

Normal Q-Q Plots

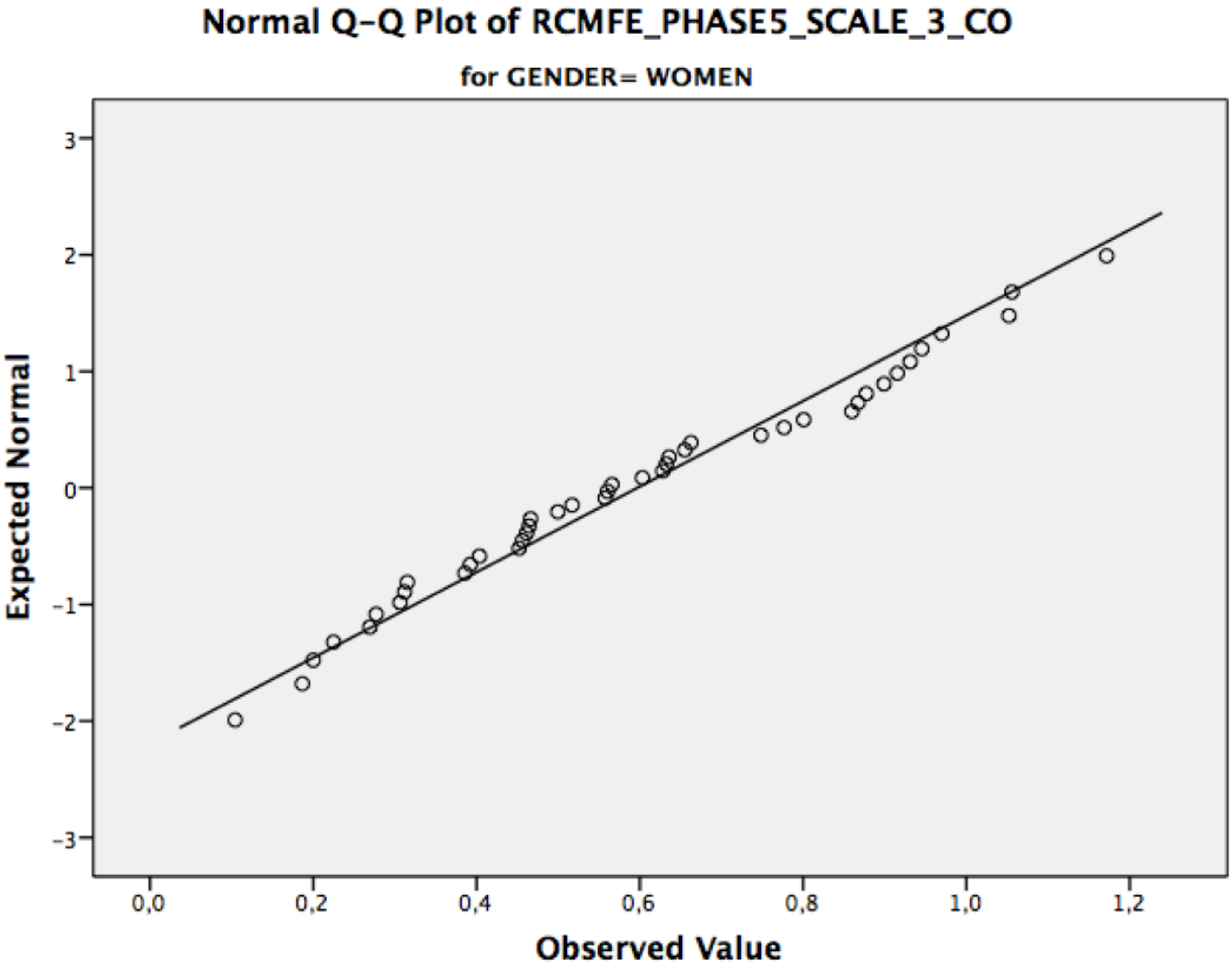

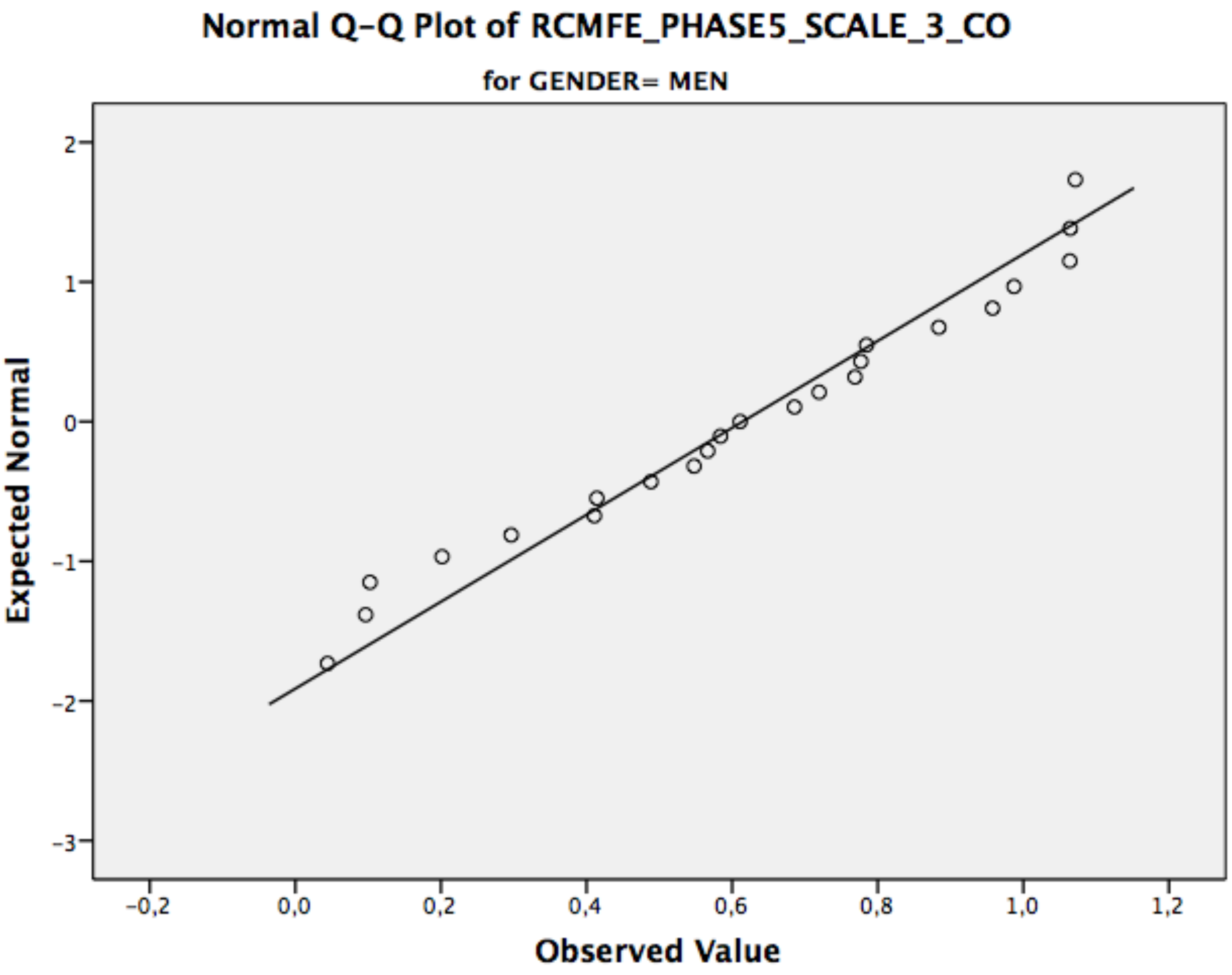

Normal Q-Q Plot of RCMFE_PHASE5_SCALE_3_CO
for GENDER= MEN
Expected Normal
Observed Value
2
1
0
−1
−2
−3
−0,2
0,0
0,2
0,4
0,6
0,8
1,0
1,2

RCMFE_PHASE5_SCALE_4_CO

Normal Q-Q Plots

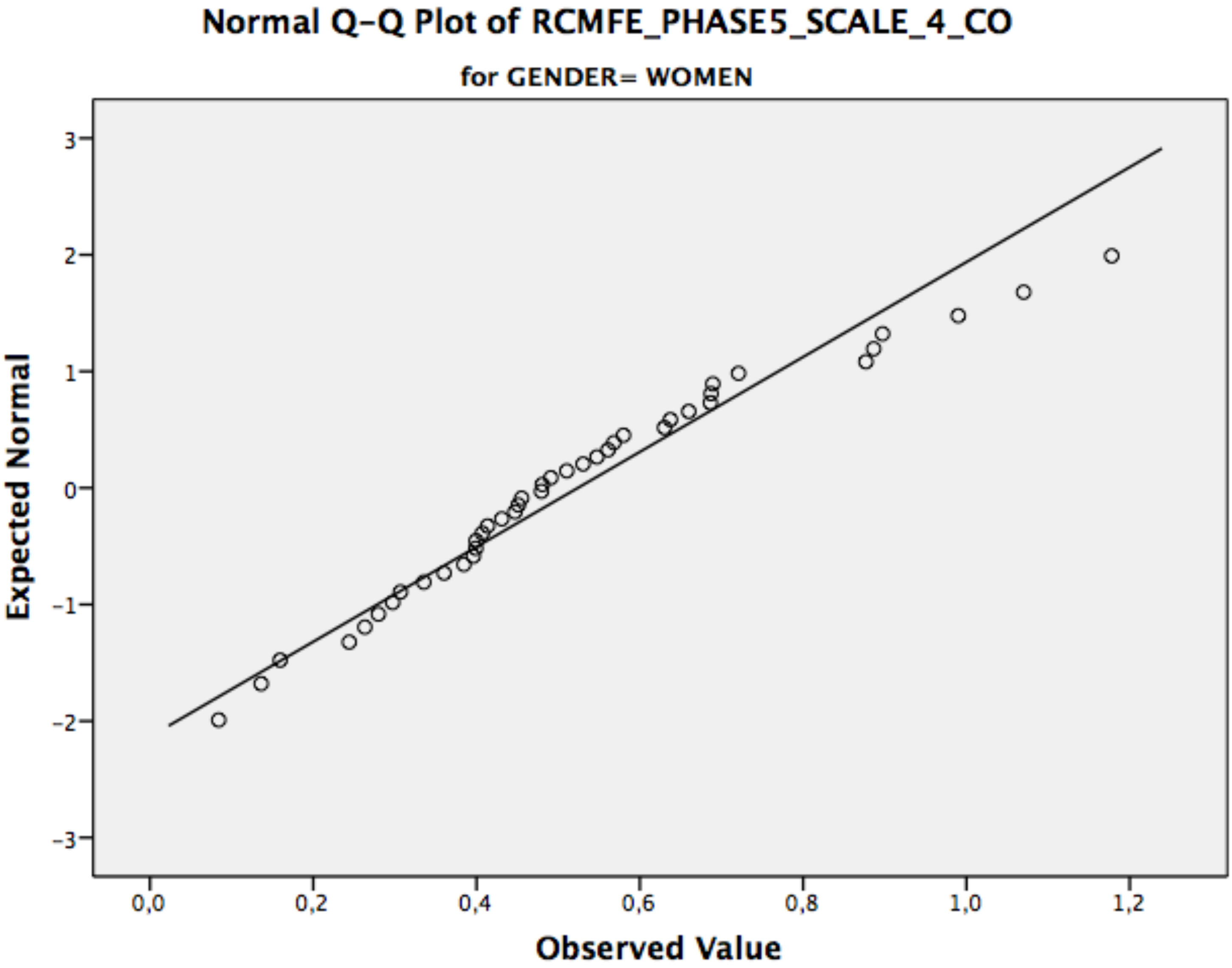

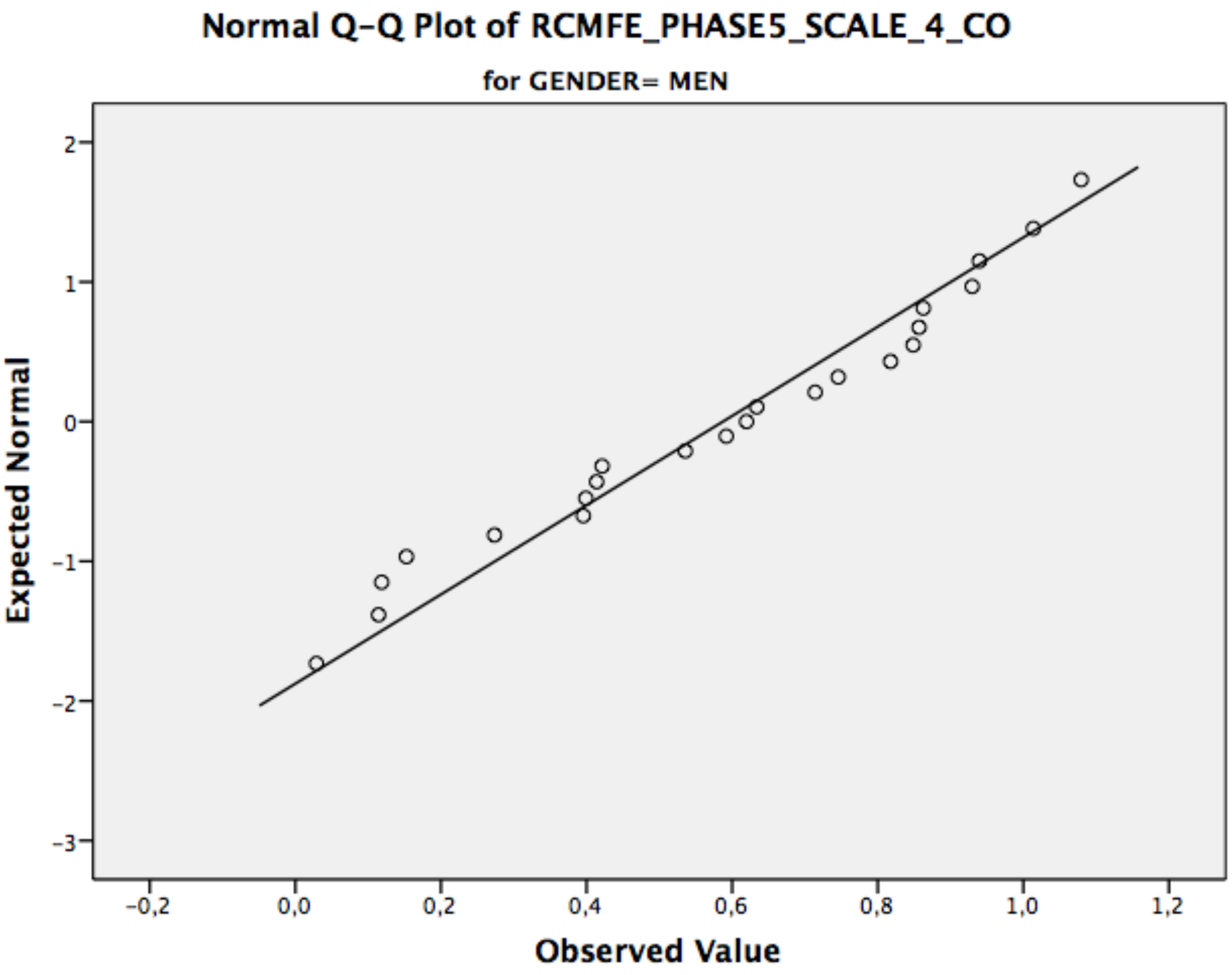
Normal Q-Q Plot of RCMFE_PHASE5_SCALE_4_CO
for GENDER= MEN
Expected Normal
2
1
0
−1
−2
−3
−0,2
0,0
0,2
0,4
0,6
0,8
1,0
1,2
Observed Value

RCMFE_PHASE5_SCALE_5_CO

Normal Q-Q Plots

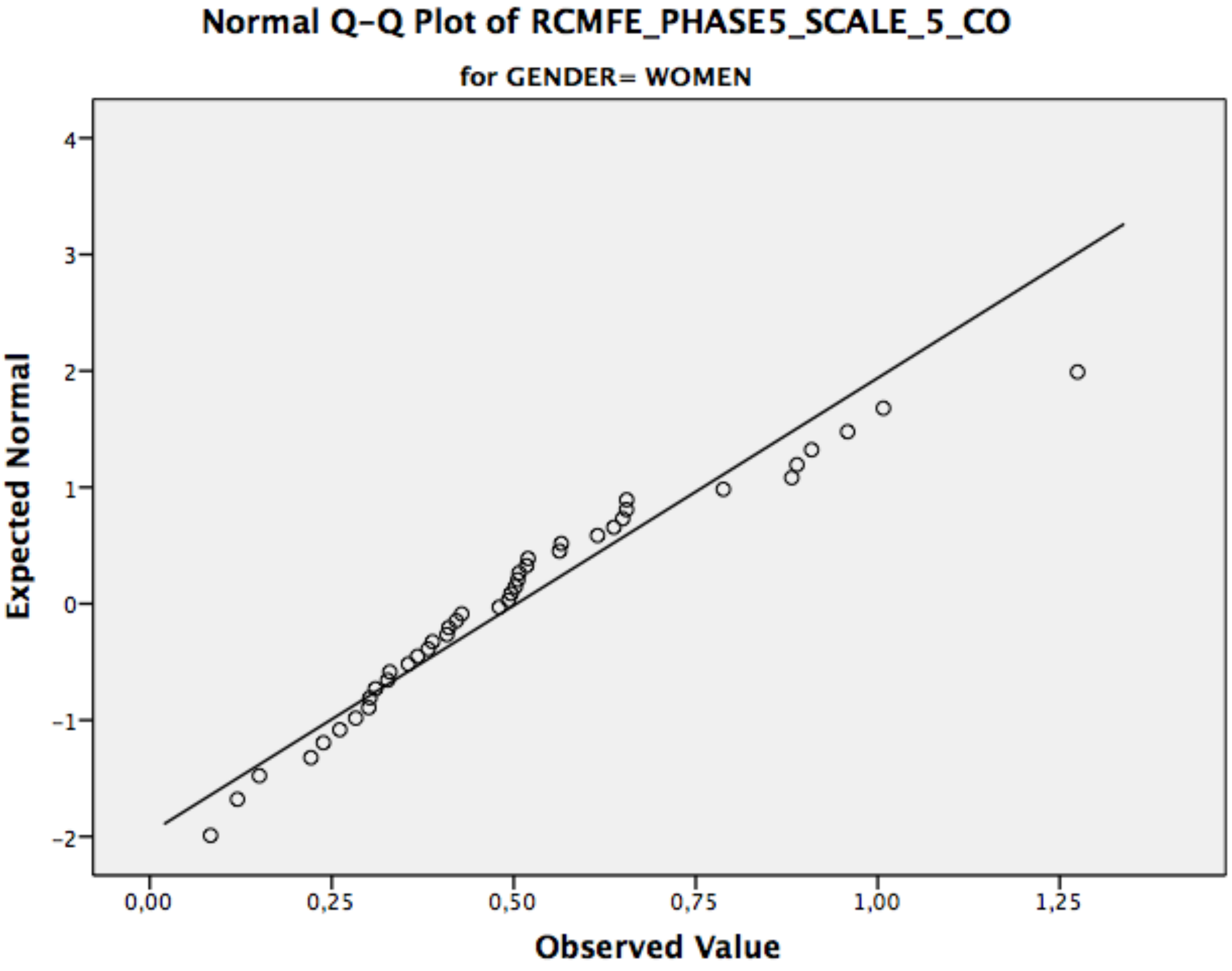

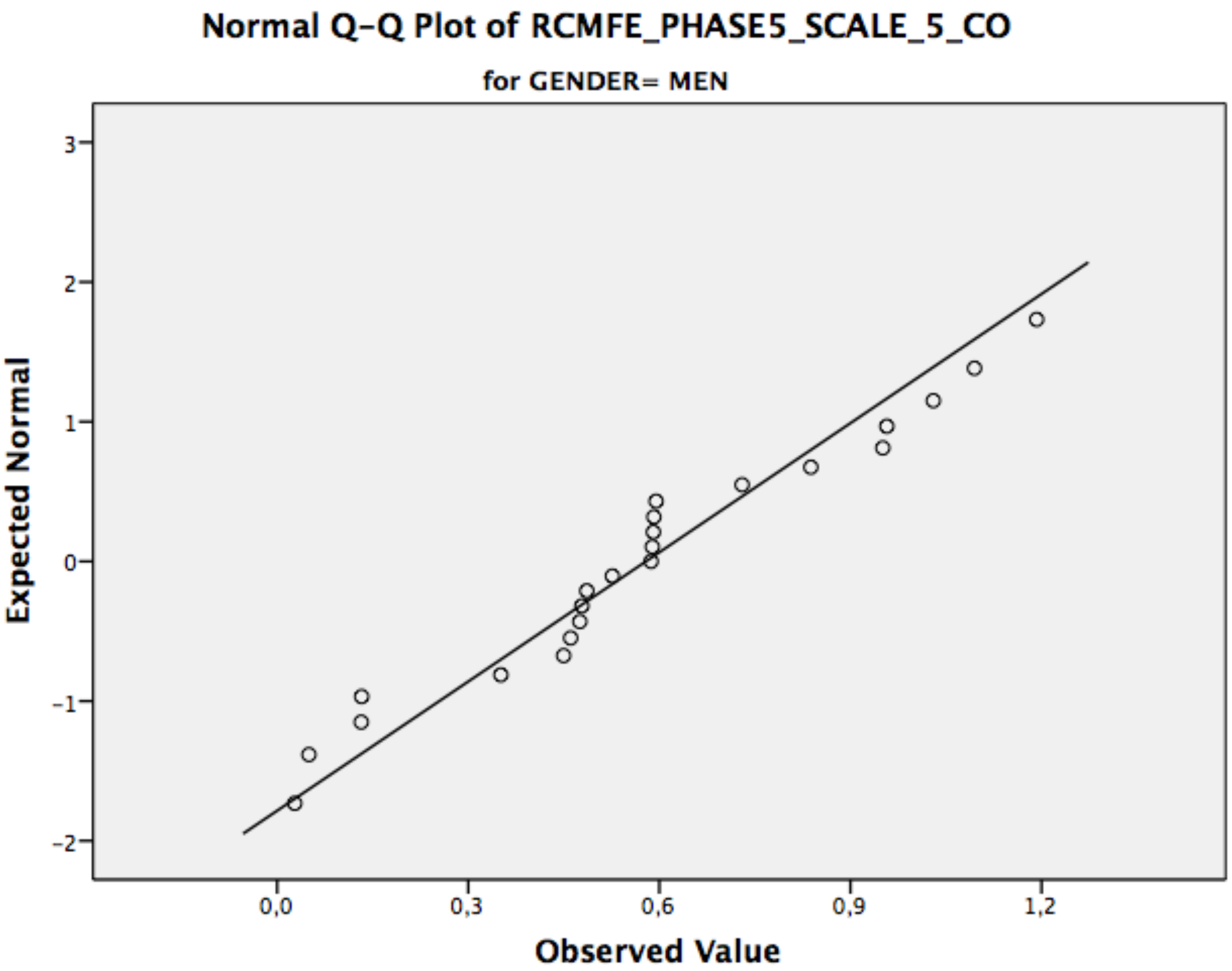

Normal Q-Q Plot of RCMFE_PHASE5_SCALE_5_CO
for GENDER= MEN
Expected Normal
Observed Value
3
2
1
0
-1
-2
0,0
0,3
0,6
0,9
1,2

Explore CO

PATHOLOGY

Case Processing Summary

	PATHOLOGY	Cases					
		Valid		Missing		Total	
		N	Percent	N	Percent	N	Percent
RCMFE_PHASE1_SCALE_1_CO	NO-OI	29	100,0%	0	0,0%	29	100,0%
	OI	36	100,0%	0	0,0%	36	100,0%
RCMFE_PHASE1_SCALE_2_CO	NO-OI	29	100,0%	0	0,0%	29	100,0%
	OI	36	100,0%	0	0,0%	36	100,0%
RCMFE_PHASE1_SCALE_3_CO	NO-OI	29	100,0%	0	0,0%	29	100,0%
	OI	36	100,0%	0	0,0%	36	100,0%
RCMFE_PHASE1_SCALE_4_CO	NO-OI	29	100,0%	0	0,0%	29	100,0%
	OI	36	100,0%	0	0,0%	36	100,0%
RCMFE_PHASE1_SCALE_5_CO	NO-OI	29	100,0%	0	0,0%	29	100,0%
	OI	36	100,0%	0	0,0%	36	100,0%
RCMFE_PHASE2_SCALE_1_CO	NO-OI	29	100,0%	0	0,0%	29	100,0%
	OI	36	100,0%	0	0,0%	36	100,0%
RCMFE_PHASE2_SCALE_2_CO	NO-OI	29	100,0%	0	0,0%	29	100,0%
	OI	36	100,0%	0	0,0%	36	100,0%
RCMFE_PHASE2_SCALE_3_CO	NO-OI	29	100,0%	0	0,0%	29	100,0%
	OI	36	100,0%	0	0,0%	36	100,0%
RCMFE_PHASE2_SCALE_4_CO	NO-OI	29	100,0%	0	0,0%	29	100,0%
	OI	36	100,0%	0	0,0%	36	100,0%
RCMFE_PHASE2_SCALE_5_CO	NO-OI	29	100,0%	0	0,0%	29	100,0%
	OI	36	100,0%	0	0,0%	36	100,0%
RCMFE_PHASE3_SCALE_1_CO	NO-OI	29	100,0%	0	0,0%	29	100,0%
	OI	36	100,0%	0	0,0%	36	100,0%
RCMFE_PHASE3_SCALE_2_CO	NO-OI	29	100,0%	0	0,0%	29	100,0%
	OI	36	100,0%	0	0,0%	36	100,0%
RCMFE_PHASE3_SCALE_3_CO	NO-OI	29	100,0%	0	0,0%	29	100,0%
	OI	36	100,0%	0	0,0%	36	100,0%
RCMFE_PHASE3_SCALE_4_CO	NO-OI	29	100,0%	0	0,0%	29	100,0%
	OI	36	100,0%	0	0,0%	36	100,0%
RCMFE_PHASE3_SCALE_5_CO	NO-OI	29	100,0%	0	0,0%	29	100,0%
	OI	36	100,0%	0	0,0%	36	100,0%
RCMFE_PHASE4_SCALE_1_CO	NO-OI	29	100,0%	0	0,0%	29	100,0%
	OI	36	100,0%	0	0,0%	36	100,0%
RCMFE_PHASE4_SCALE_2_CO	NO-OI	29	100,0%	0	0,0%	29	100,0%
	OI	36	100,0%	0	0,0%	36	100,0%
RCMFE_PHASE4_SCALE_3	NO-OI	29	100,0%	0	0,0%	29	100,0%
	OI	36	100,0%	0	0,0%	36	100,0%
RCMFE_PHASE4_SCALE_4_CO	NO-OI	29	100,0%	0	0,0%	29	100,0%
	OI	36	100,0%	0	0,0%	36	100,0%
RCMFE_PHASE4_SCALE_5_CO	NO-OI	29	100,0%	0	0,0%	29	100,0%
	OI	36	100,0%	0	0,0%	36	100,0%
RCMFE_PHASE5_SCALE_1_CO	NO-OI	29	100,0%	0	0,0%	29	100,0%

Case Processing Summary

	PATHOLOGY	Cases					
		Valid		Missing		Total	
		N	Percent	N	Percent	N	Percent
RCMFE_PHASE5_SCALE_1_CO	OI	36	100,0%	0	0,0%	36	100,0%
RCMFE_PHASE5_SCALE_2_CO	NO-OI	29	100,0%	0	0,0%	29	100,0%
	OI	36	100,0%	0	0,0%	36	100,0%
RCMFE_PHASE5_SCALE_3_CO	NO-OI	29	100,0%	0	0,0%	29	100,0%
	OI	36	100,0%	0	0,0%	36	100,0%
RCMFE_PHASE5_SCALE_4_CO	NO-OI	29	100,0%	0	0,0%	29	100,0%
	OI	36	100,0%	0	0,0%	36	100,0%
RCMFE_PHASE5_SCALE_5_CO	NO-OI	29	100,0%	0	0,0%	29	100,0%
	OI	36	100,0%	0	0,0%	36	100,0%

Tests of Normality

	PATHOLOGY	Kolmogorov-Smirnov[a]			Shapiro-Wilk		
		Statistic	df	Sig.	Statistic	df	Sig.
RCMFE_PHASE1_SCALE_1_CO	NO-OI	,109	29	,200*	,976	29	,719
	OI	,101	36	,200*	,976	36	,605
RCMFE_PHASE1_SCALE_2_CO	NO-OI	,130	29	,200*	,971	29	,582
	OI	,094	36	,200*	,968	36	,380
RCMFE_PHASE1_SCALE_3_CO	NO-OI	,095	29	,200*	,975	29	,690
	OI	,101	36	,200*	,960	36	,222
RCMFE_PHASE1_SCALE_4_CO	NO-OI	,076	29	,200*	,972	29	,626
	OI	,115	36	,200*	,970	36	,415
RCMFE_PHASE1_SCALE_5_CO	NO-OI	,087	29	,200*	,982	29	,896
	OI	,081	36	,200*	,973	36	,528
RCMFE_PHASE2_SCALE_1_CO	NO-OI	,107	29	,200*	,977	29	,750
	OI	,127	36	,154	,969	36	,391
RCMFE_PHASE2_SCALE_2_CO	NO-OI	,098	29	,200*	,978	29	,773
	OI	,091	36	,200*	,981	36	,771
RCMFE_PHASE2_SCALE_3_CO	NO-OI	,112	29	,200*	,949	29	,174
	OI	,067	36	,200*	,982	36	,810
RCMFE_PHASE2_SCALE_4_CO	NO-OI	,103	29	,200*	,953	29	,216
	OI	,078	36	,200*	,976	36	,596
RCMFE_PHASE2_SCALE_5_CO	NO-OI	,155	29	,075	,918	29	,027
	OI	,135	36	,095	,960	36	,209
RCMFE_PHASE3_SCALE_1_CO	NO-OI	,117	29	,200*	,961	29	,346
	OI	,131	36	,125	,939	36	,048
RCMFE_PHASE3_SCALE_2_CO	NO-OI	,075	29	,200*	,978	29	,779
	OI	,087	36	,200*	,977	36	,645
RCMFE_PHASE3_SCALE_3_CO	NO-OI	,110	29	,200*	,984	29	,929
	OI	,065	36	,200*	,983	36	,845
RCMFE_PHASE3_SCALE_4_CO	NO-OI	,131	29	,200*	,964	29	,422
	OI	,110	36	,200*	,960	36	,211
RCMFE_PHASE3_SCALE_5	NO-OI	,086	29	,200*	,975	29	,700

_CO	OI	,108	36	,200*	,966	36	,325
RCMFE_PHASE4_SCALE_1	NO-OI	,213	29	,002	,769	29	,050
_CO	OI	,177	36	,006	,905	36	,055
RCMFE_PHASE4_SCALE_2	NO-OI	,167	29	,038	,833	29	,060
_CO	OI	,152	36	,036	,907	36	,075
RCMFE_PHASE4_SCALE_3	NO-OI	,178	29	,020	,842	29	,051
	OI	,127	36	,155	,931	36	,027
RCMFE_PHASE4_SCALE_4	NO-OI	,189	29	,009	,896	29	,058
_CO	OI	,165	36	,015	,860	36	,070
RCMFE_PHASE4_SCALE_5	NO-OI	,178	29	,019	,890	29	,066
_CO	OI	,174	36	,007	,838	36	,080
RCMFE_PHASE5_SCALE_1	NO-OI	,074	29	,200*	,978	29	,795
_CO	OI	,080	36	,200*	,977	36	,643

Tests of Normality

	PATHOLOGY	Kolmogorov-Smirnov[a]			Shapiro-Wilk		
		Statistic	df	Sig.	Statistic	df	Sig.
RCMFE_PHASE5_SCALE_2	NO-OI	,149	29	,101*	,949	29	,167
_CO	OI	,065	36	,200*	,976	36	,620
RCMFE_PHASE5_SCALE_3	NO-OI	,104	29	,200*	,960	29	,334
_CO	OI	,120	36	,200*	,967	36	,345
RCMFE_PHASE5_SCALE_4	NO-OI	,068	29	,200*	,978	29	,785
_CO	OI	,114	36	,200*	,959	36	,195
RCMFE_PHASE5_SCALE_5	NO-OI	,128	29	,200*	,964	29	,411
_CO	OI	,161	36	,019*	,939	36	,057

*. This is a lower bound of the true significance.

a. Lilliefors Significance Correction

RCMFE_PHASE1_SCALE_1_CO

Normal Q-Q Plots

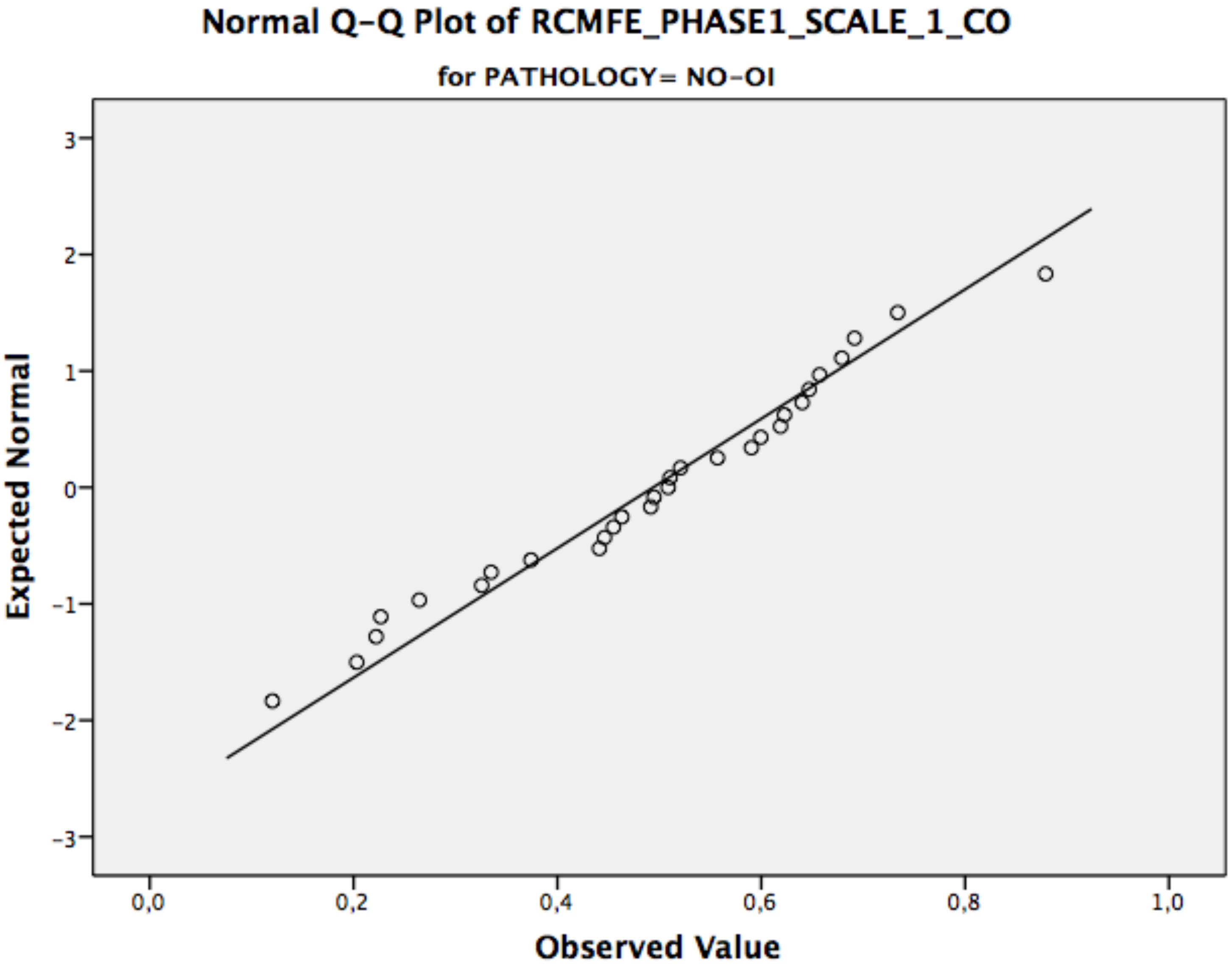

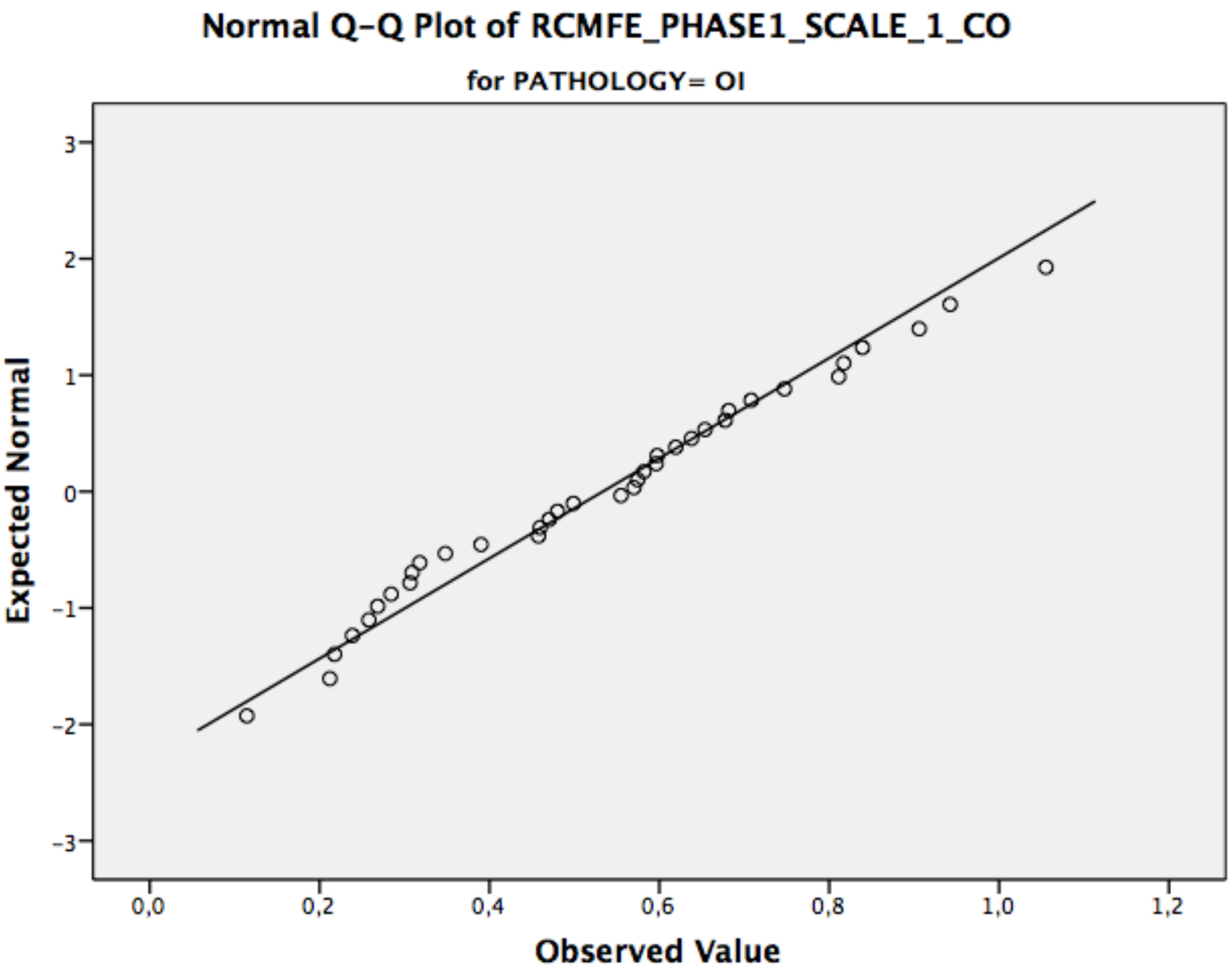

Normal Q-Q Plot of RCMFE_PHASE1_SCALE_1_CO
for PATHOLOGY= OI
Expected Normal
3
2
1
0
−1
−2
−3
0,0
0,2
0,4
0,6
0,8
1,0
1,2
Observed Value

RCMFE_PHASE1_SCALE_2_CO

Normal Q-Q Plots

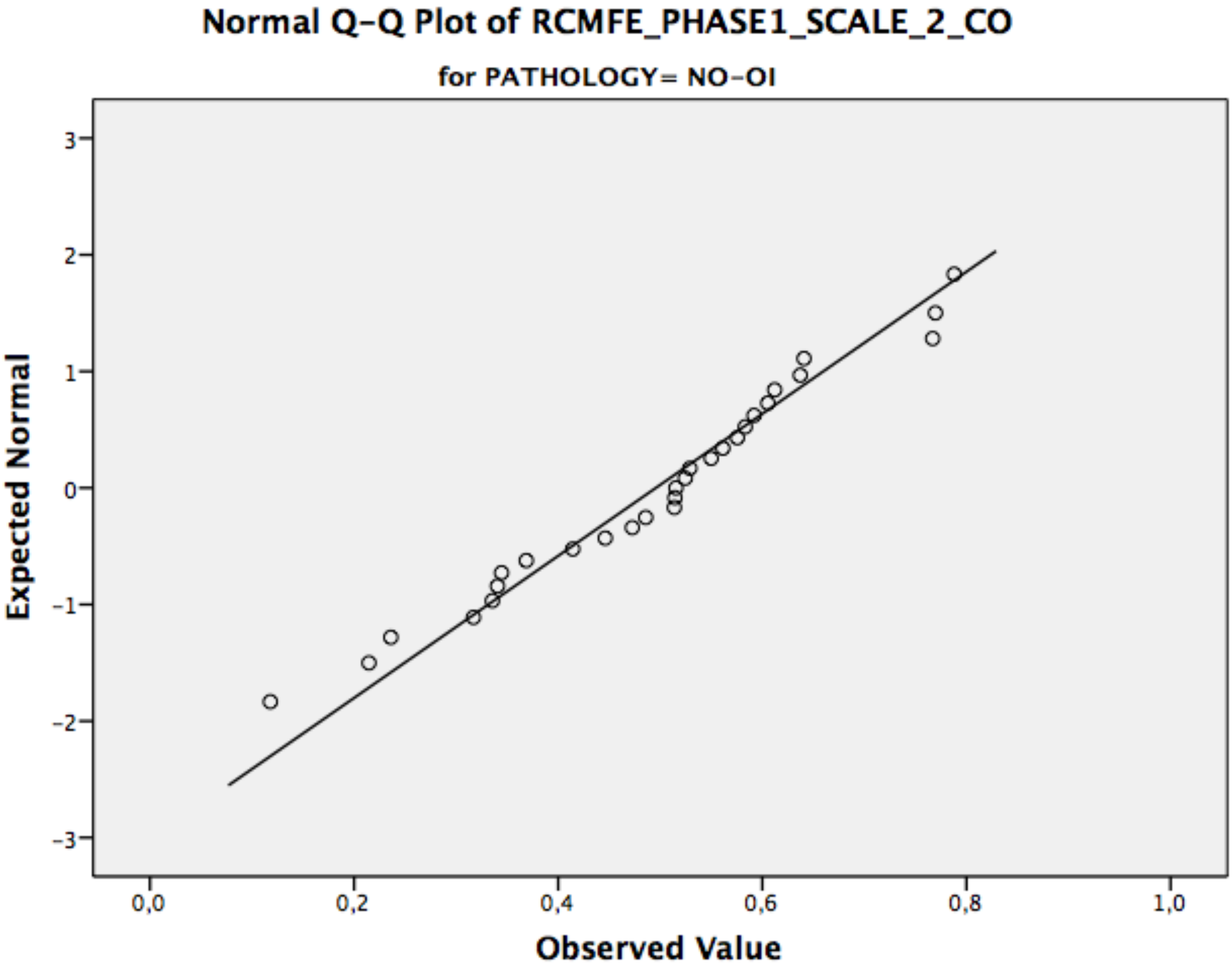

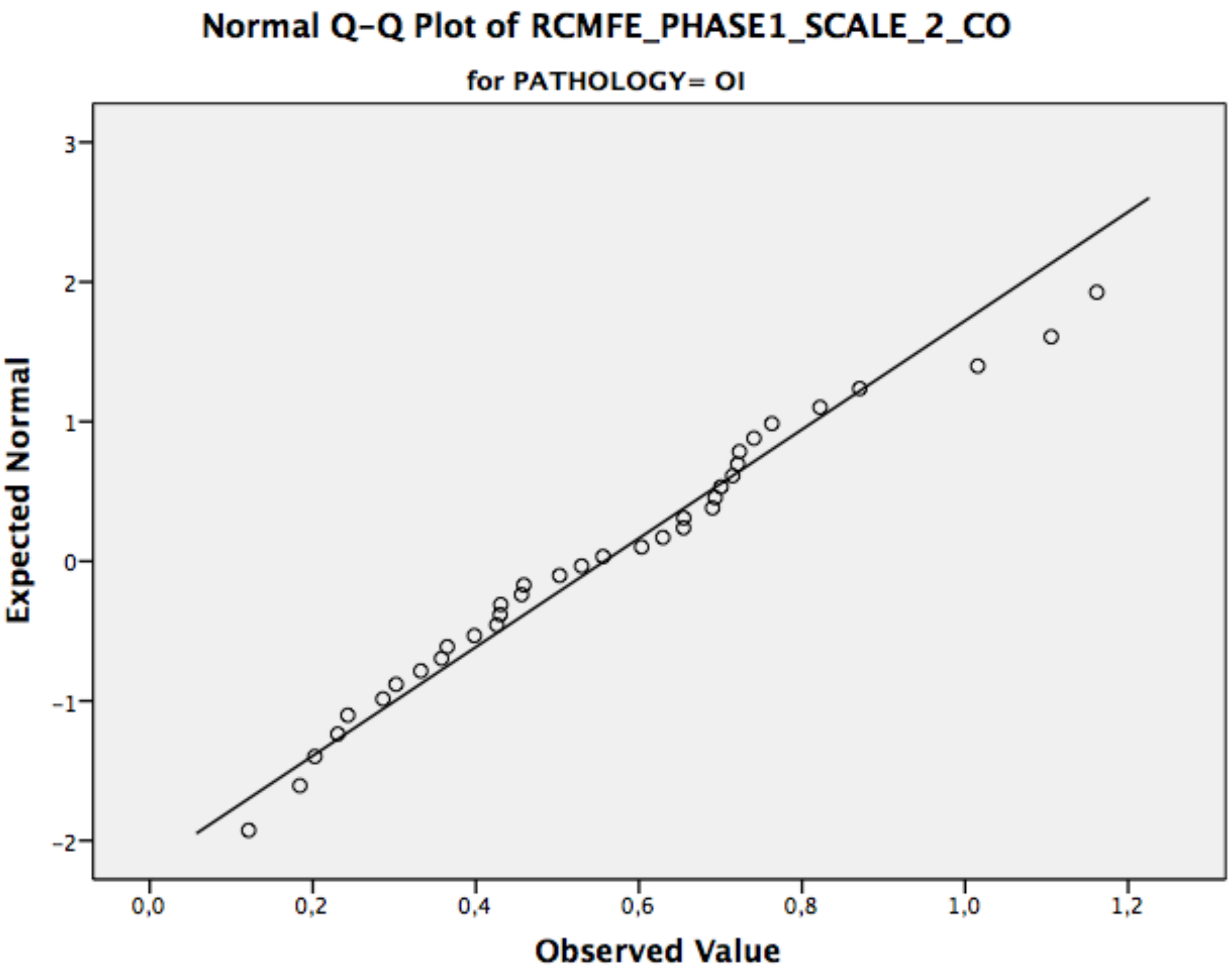

Normal Q-Q Plot of RCMFE_PHASE1_SCALE_2_CO
for PATHOLOGY= OI
Expected Normal
3
2
1
0
−1
−2
0,0
0,2
0,4
0,6
0,8
1,0
1,2
Observed Value

RCMFE_PHASE1_SCALE_3_CO

Normal Q-Q Plots

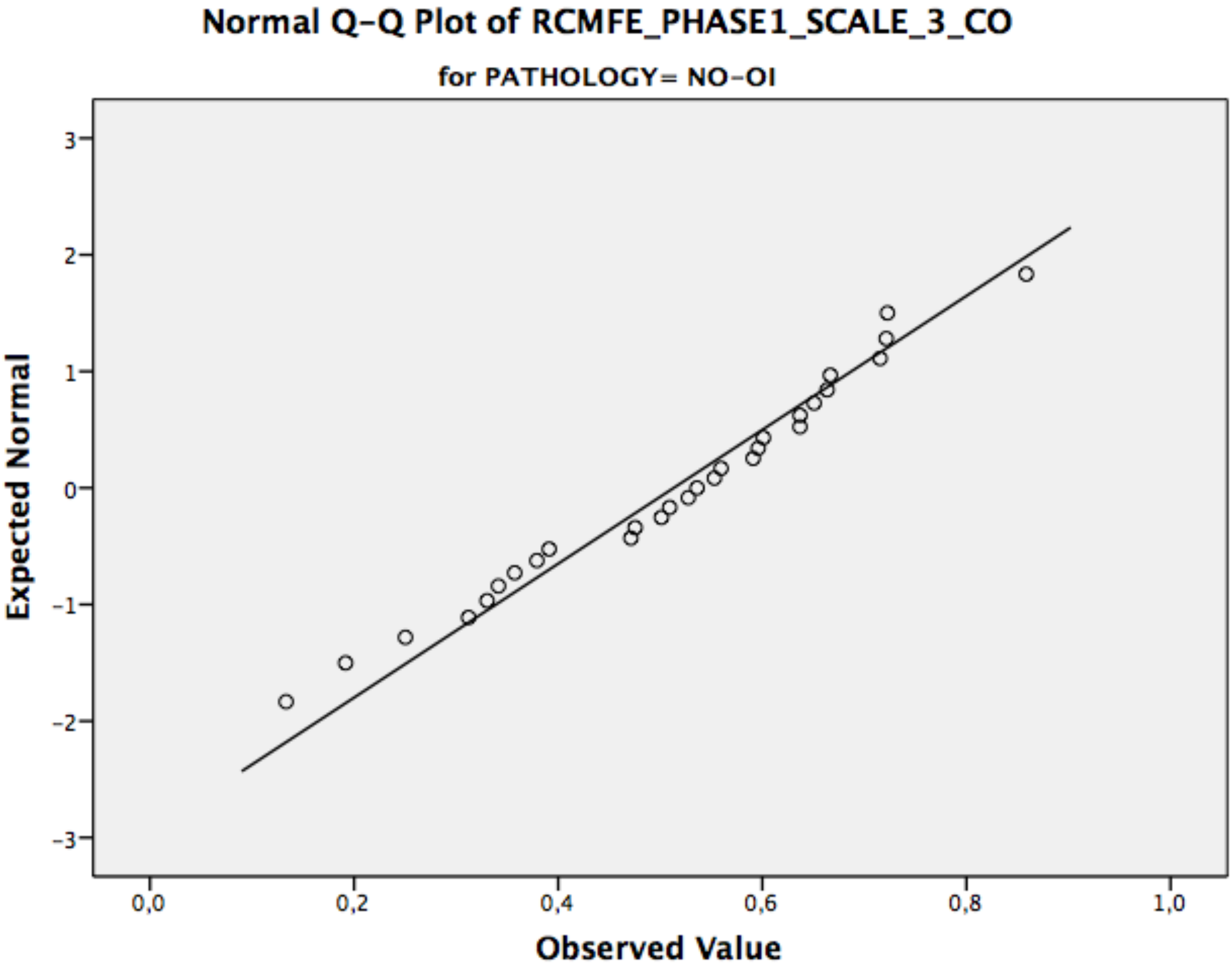

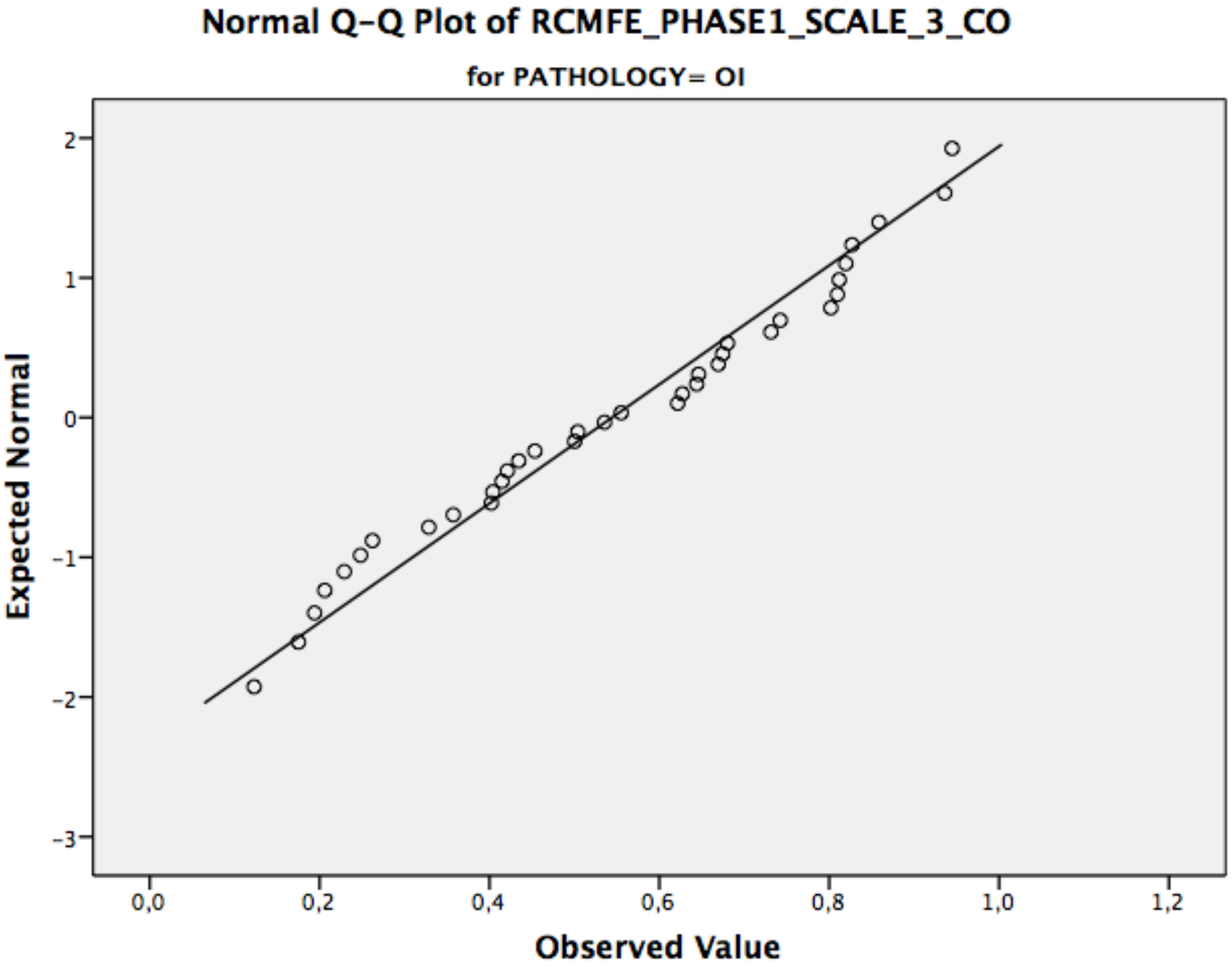
Normal Q-Q Plot of RCMFE_PHASE1_SCALE_3_CO
for PATHOLOGY= OI
Expected Normal
2
1
0
-1
-2
-3
0,0
0,2
0,4
0,6
0,8
1,0
1,2
Observed Value

RCMFE_PHASE1_SCALE_4_CO

Normal Q-Q Plots

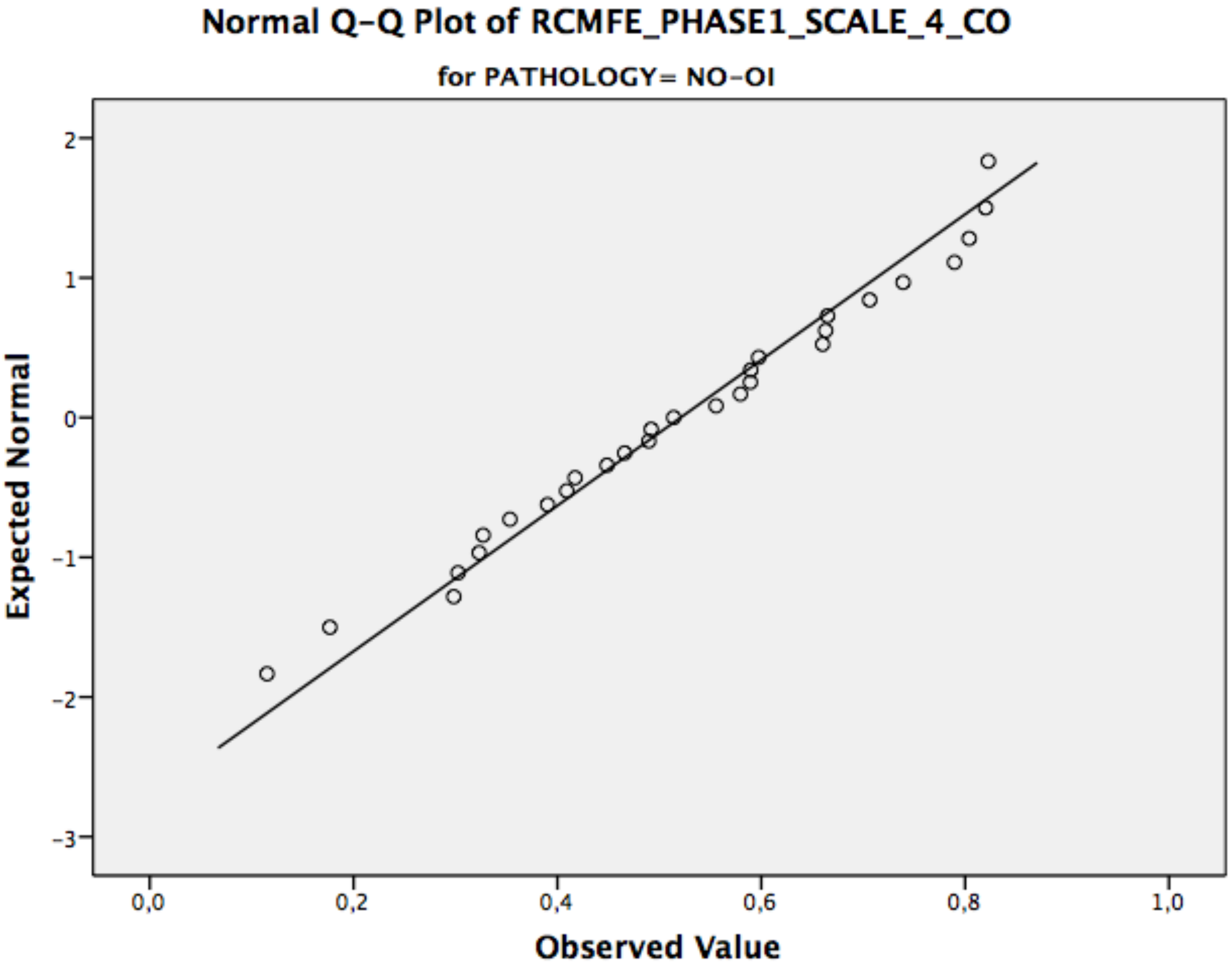

Normal Q-Q Plot of RCMFE_PHASE1_SCALE_4_CO

for PATHOLOGY= OI

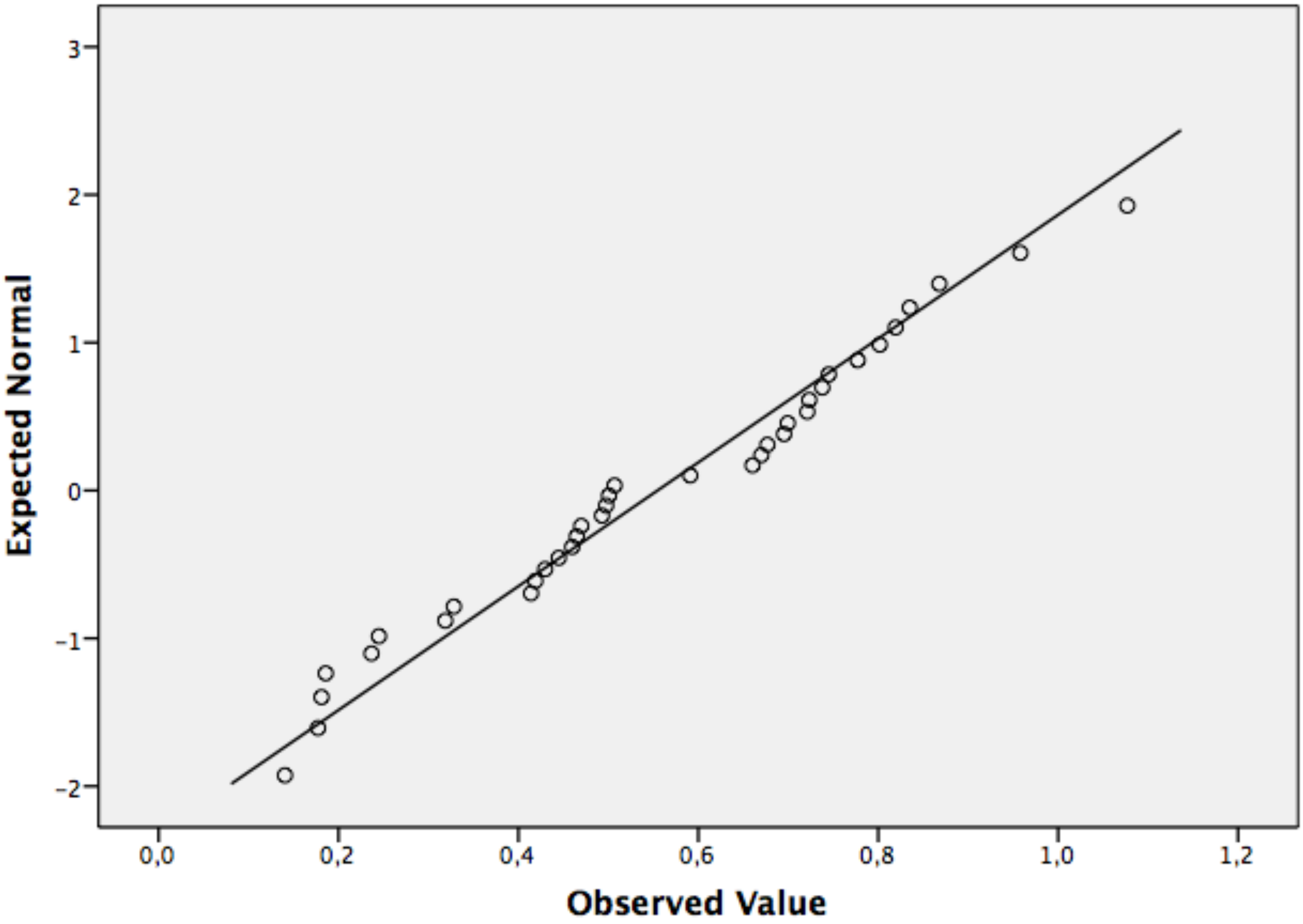

RCMFE_PHASE1_SCALE_5_CO

Normal Q-Q Plots

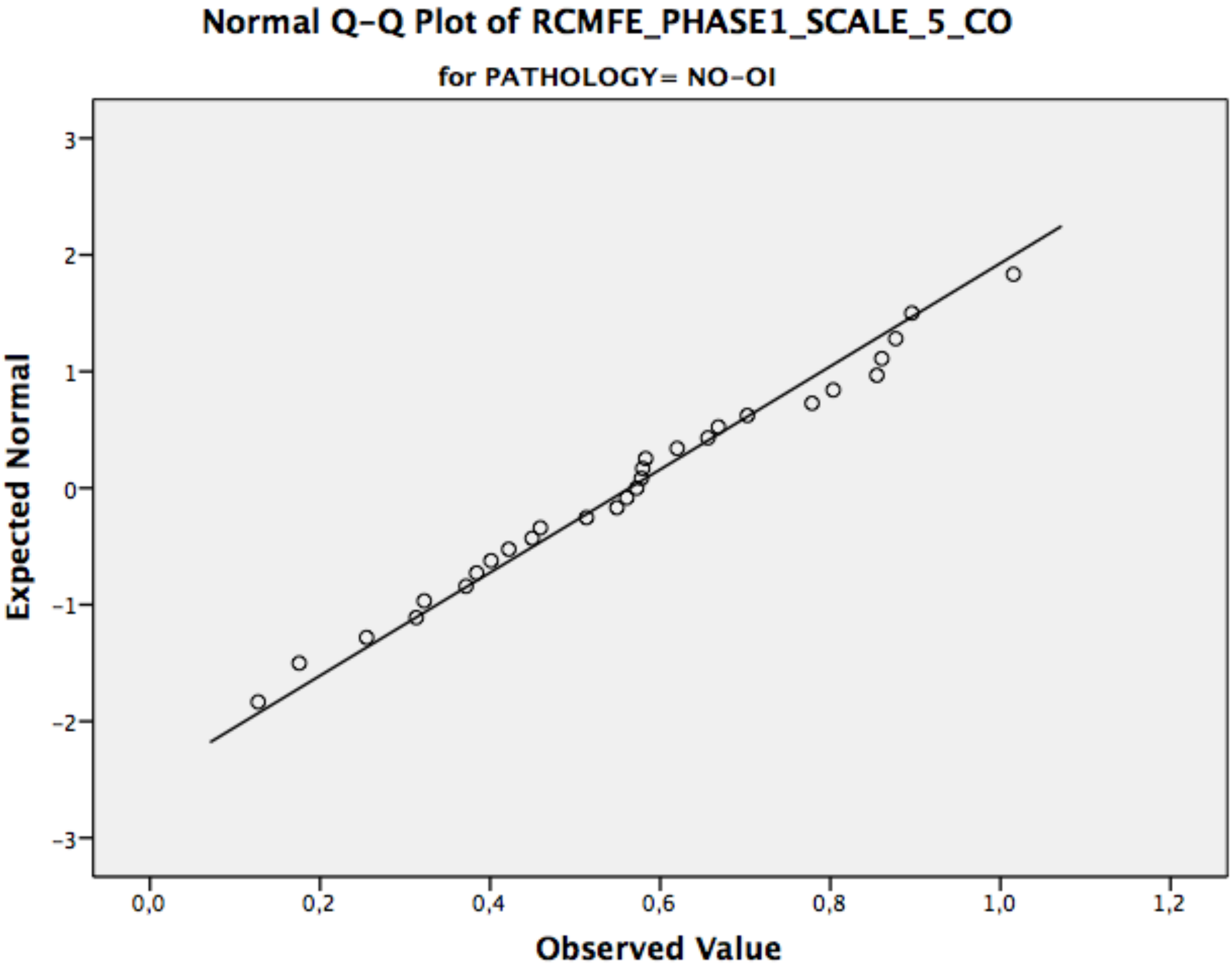

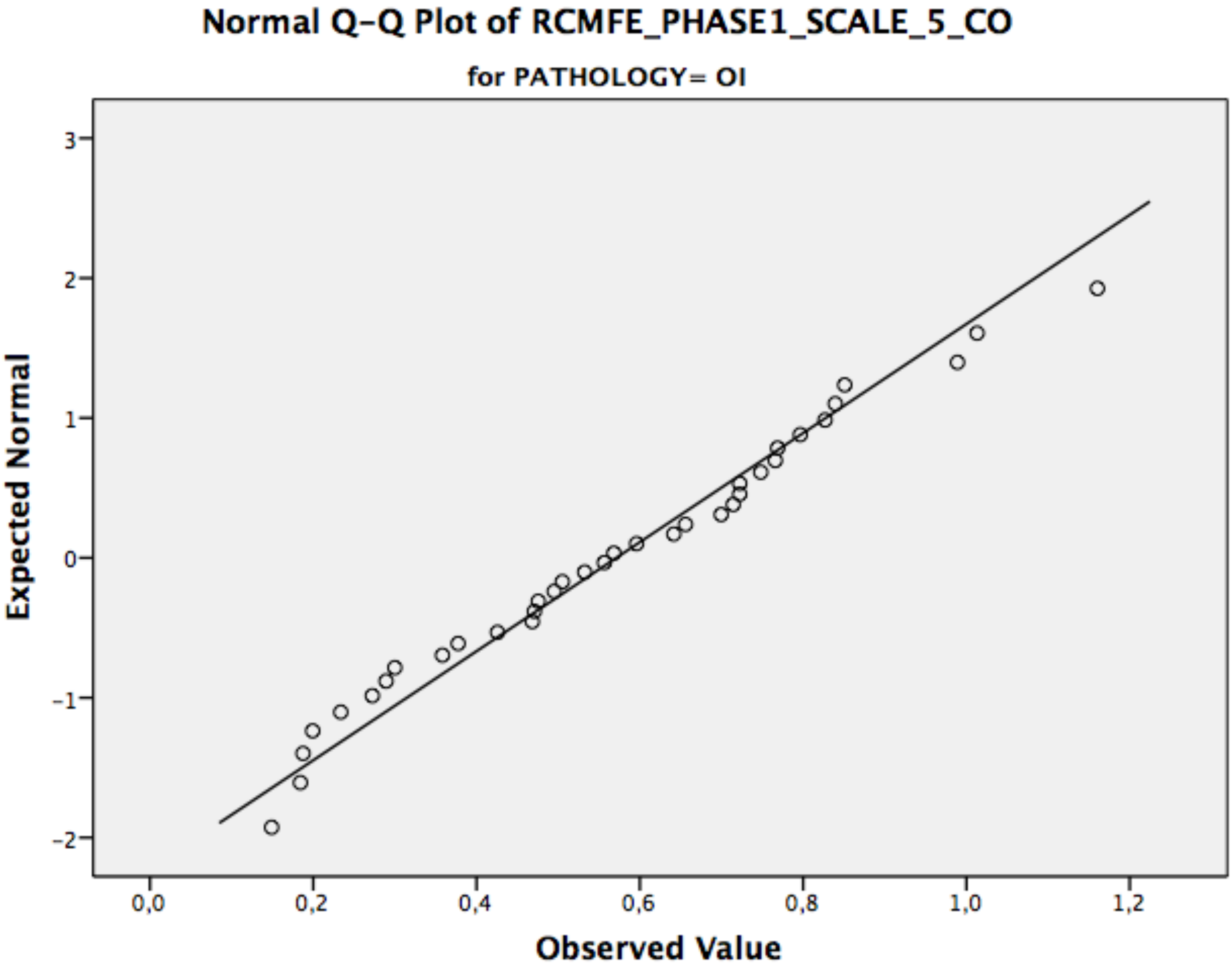
Normal Q-Q Plot of RCMFE_PHASE1_SCALE_5_CO
for PATHOLOGY= OI
Expected Normal
Observed Value
3
2
1
0
−1
−2
0,0
0,2
0,4
0,6
0,8
1,0
1,2

Normal Q-Q Plots

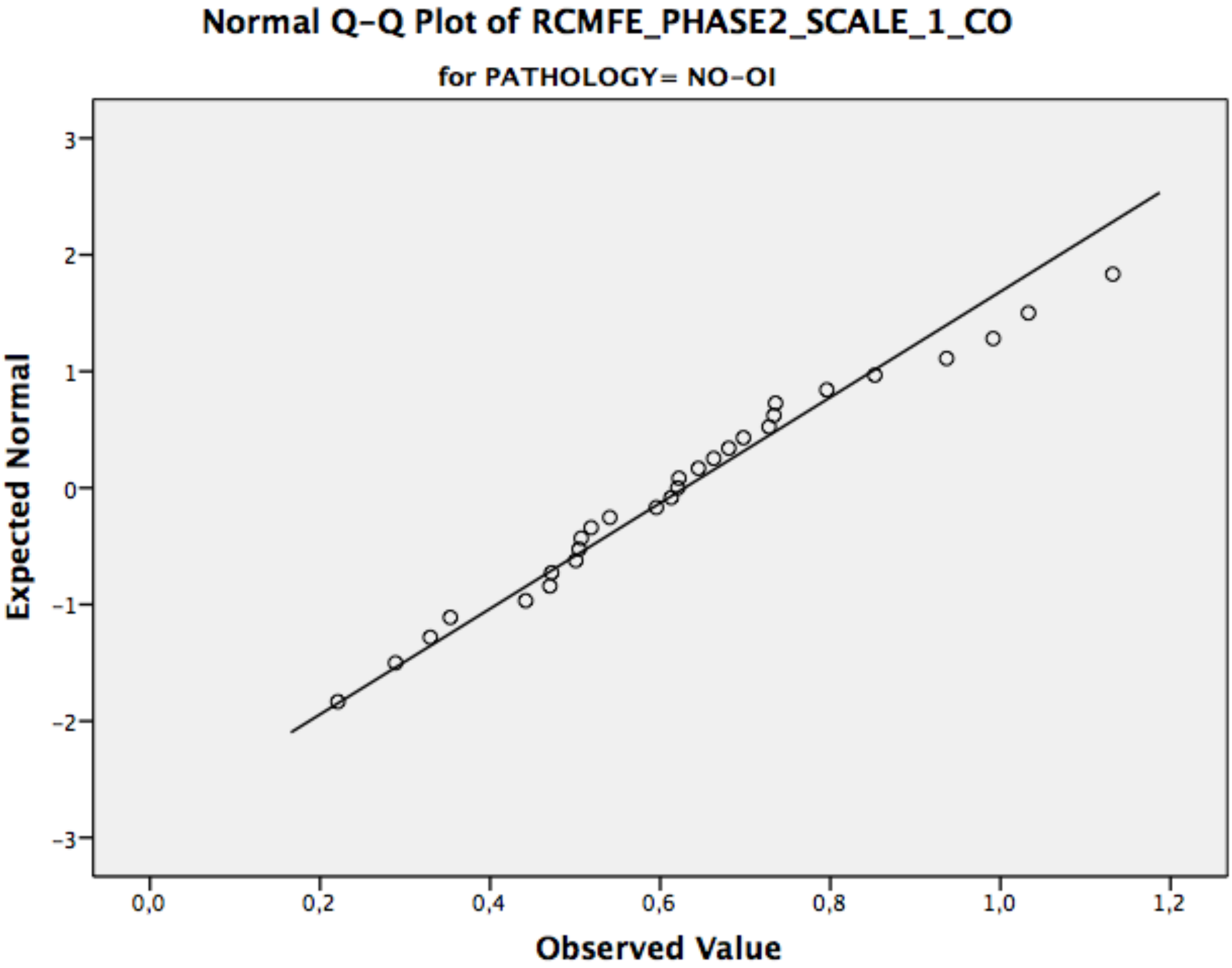

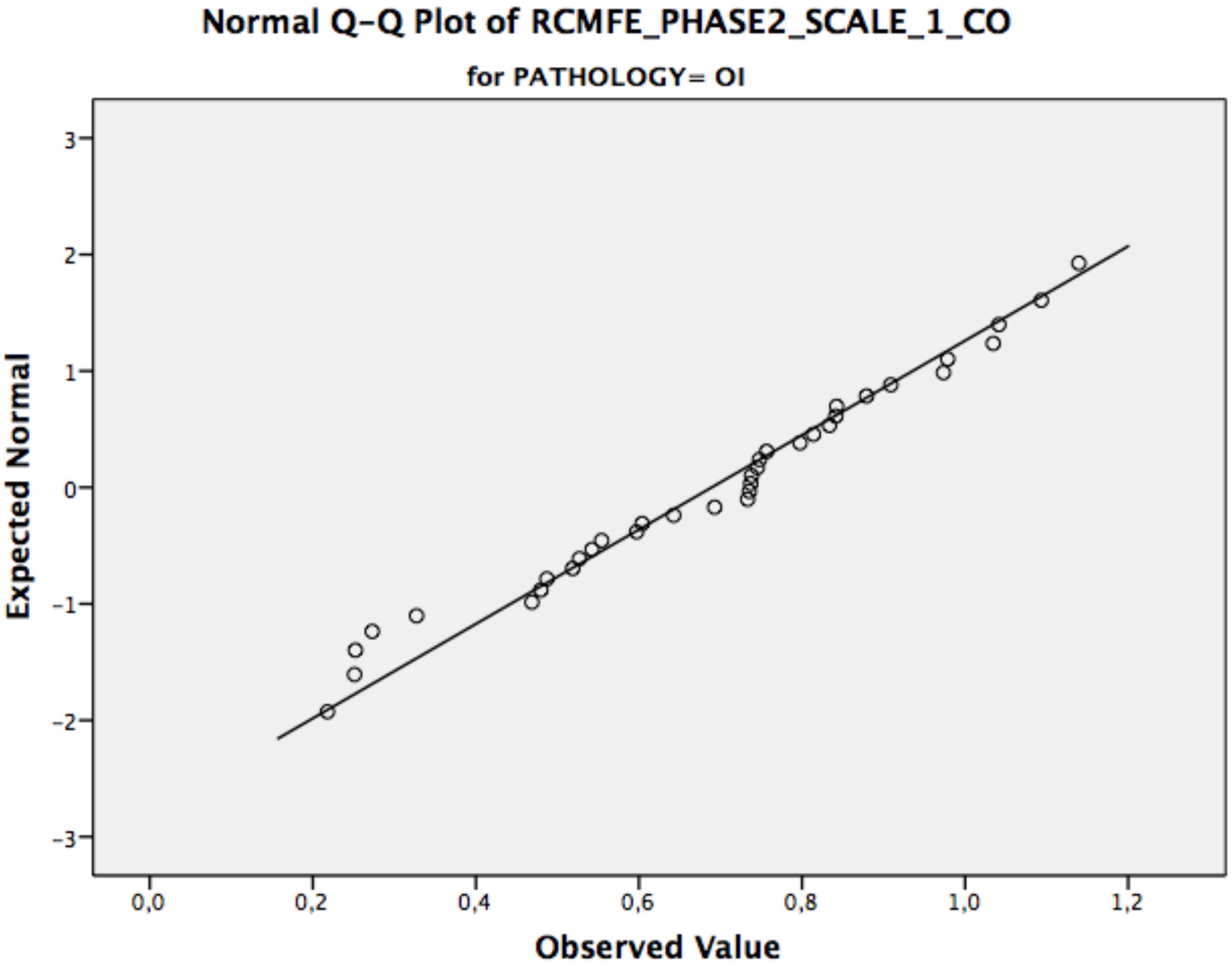
Normal Q-Q Plot of RCMFE_PHASE2_SCALE_1_CO
for PATHOLOGY= OI
Expected Normal
3
2
1
0
-1
-2
-3
0,0
0,2
0,4
0,6
0,8
1,0
1,2
Observed Value

RCMFE_PHASE2_SCALE_2_CO

Normal Q-Q Plots

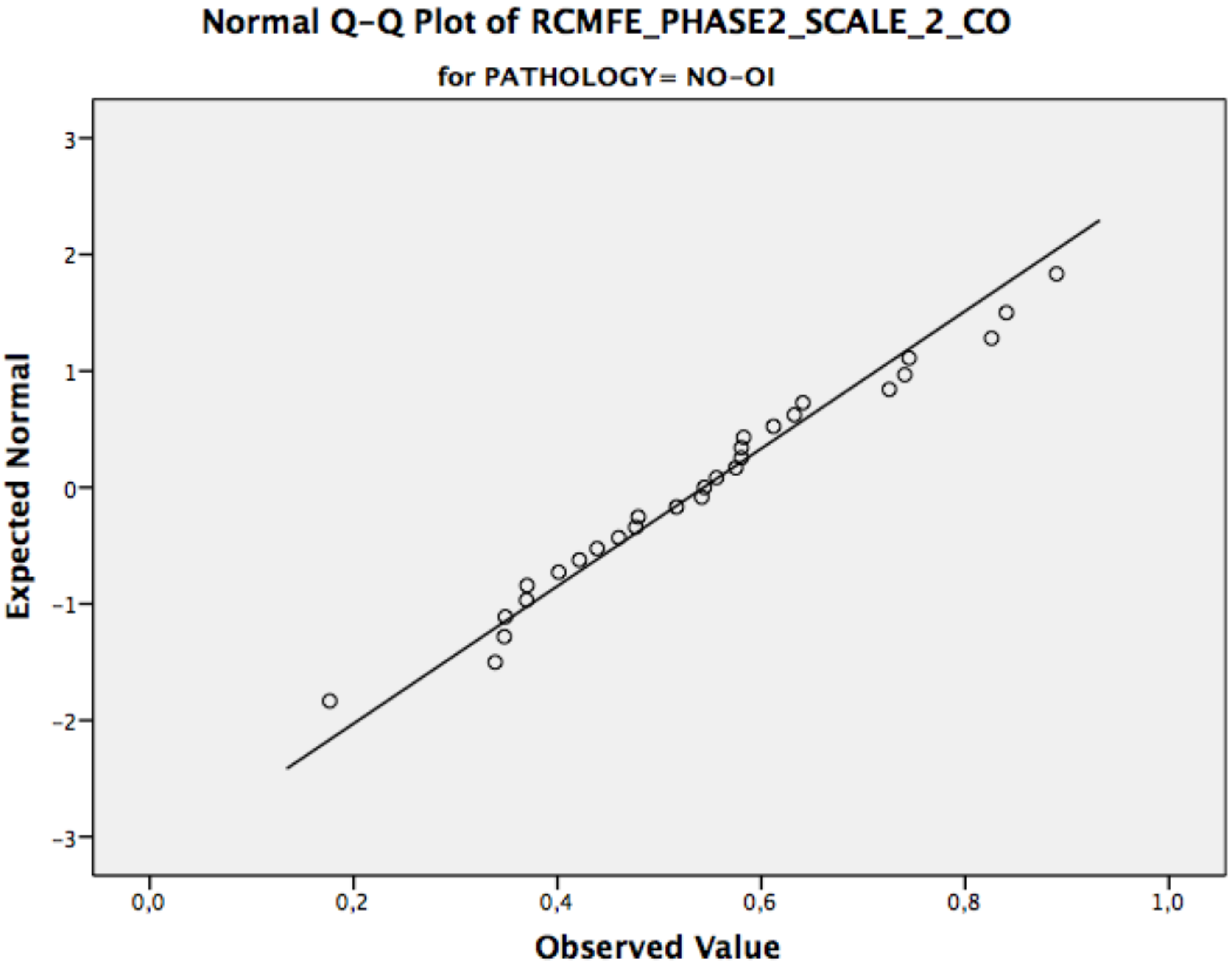

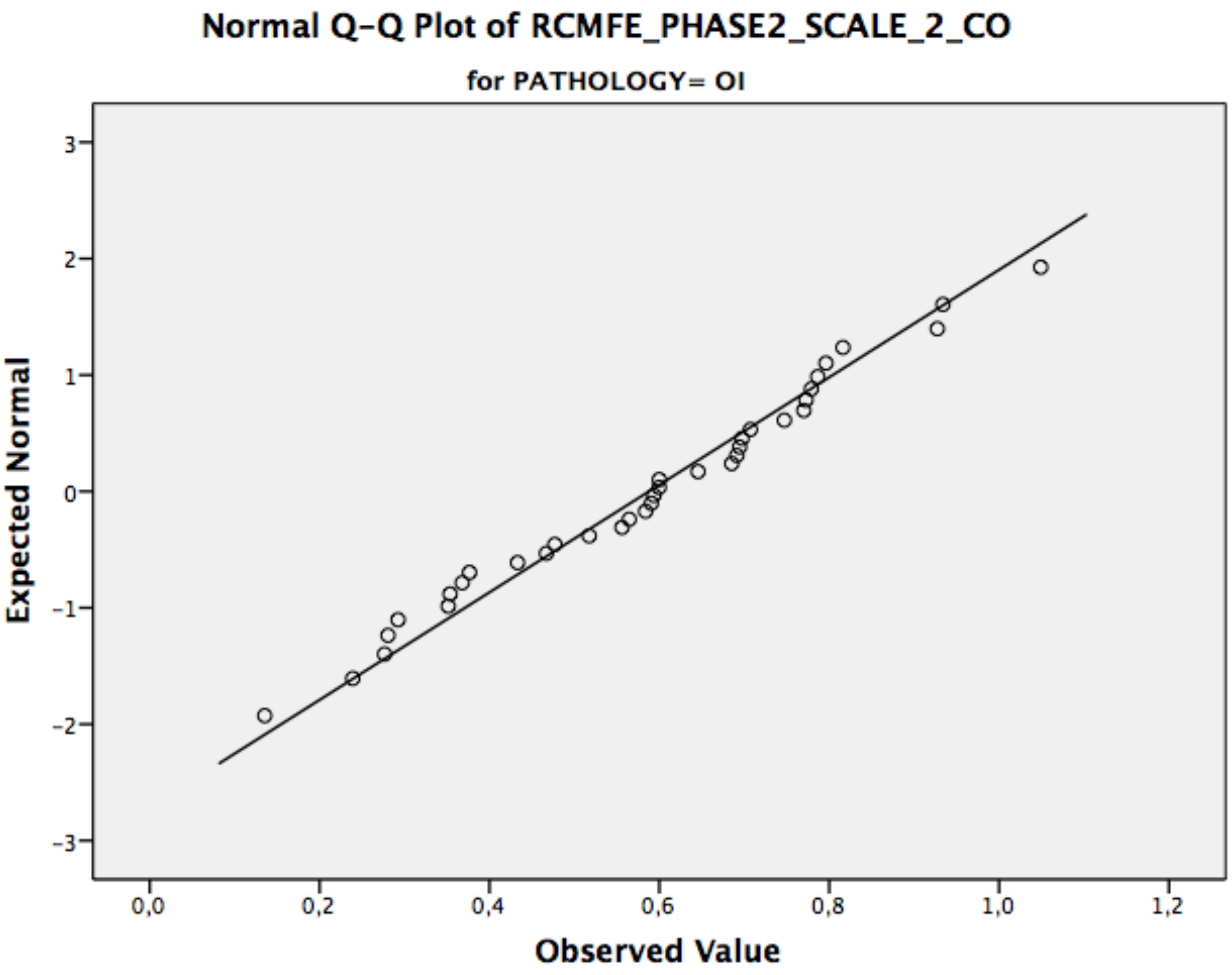
Normal Q-Q Plot of RCMFE_PHASE2_SCALE_2_CO
for PATHOLOGY= OI
Expected Normal
Observed Value
3
2
1
0
-1
-2
-3
0,0
0,2
0,4
0,6
0,8
1,0
1,2

RCMFE_PHASE2_SCALE_3_CO

Normal Q-Q Plots

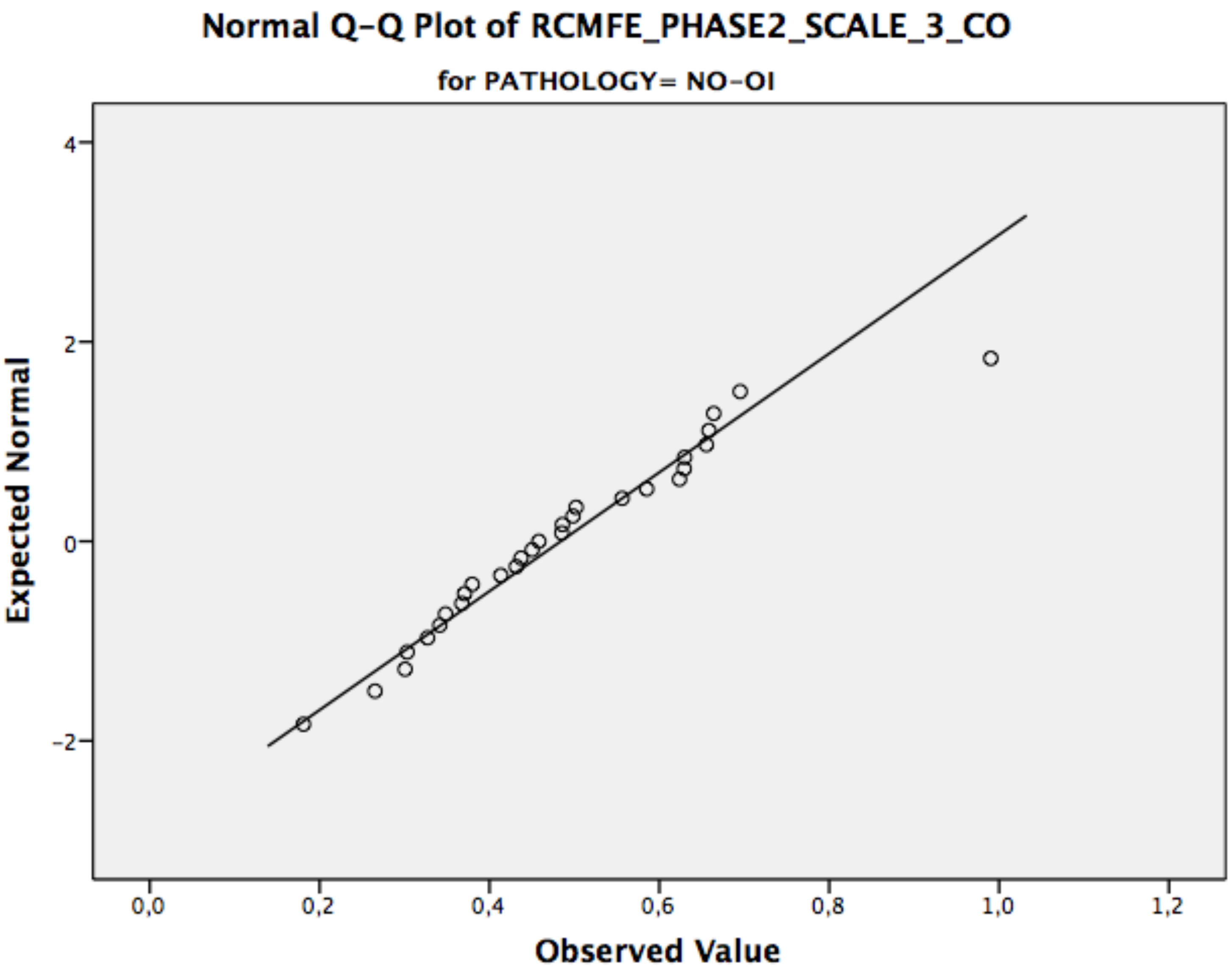

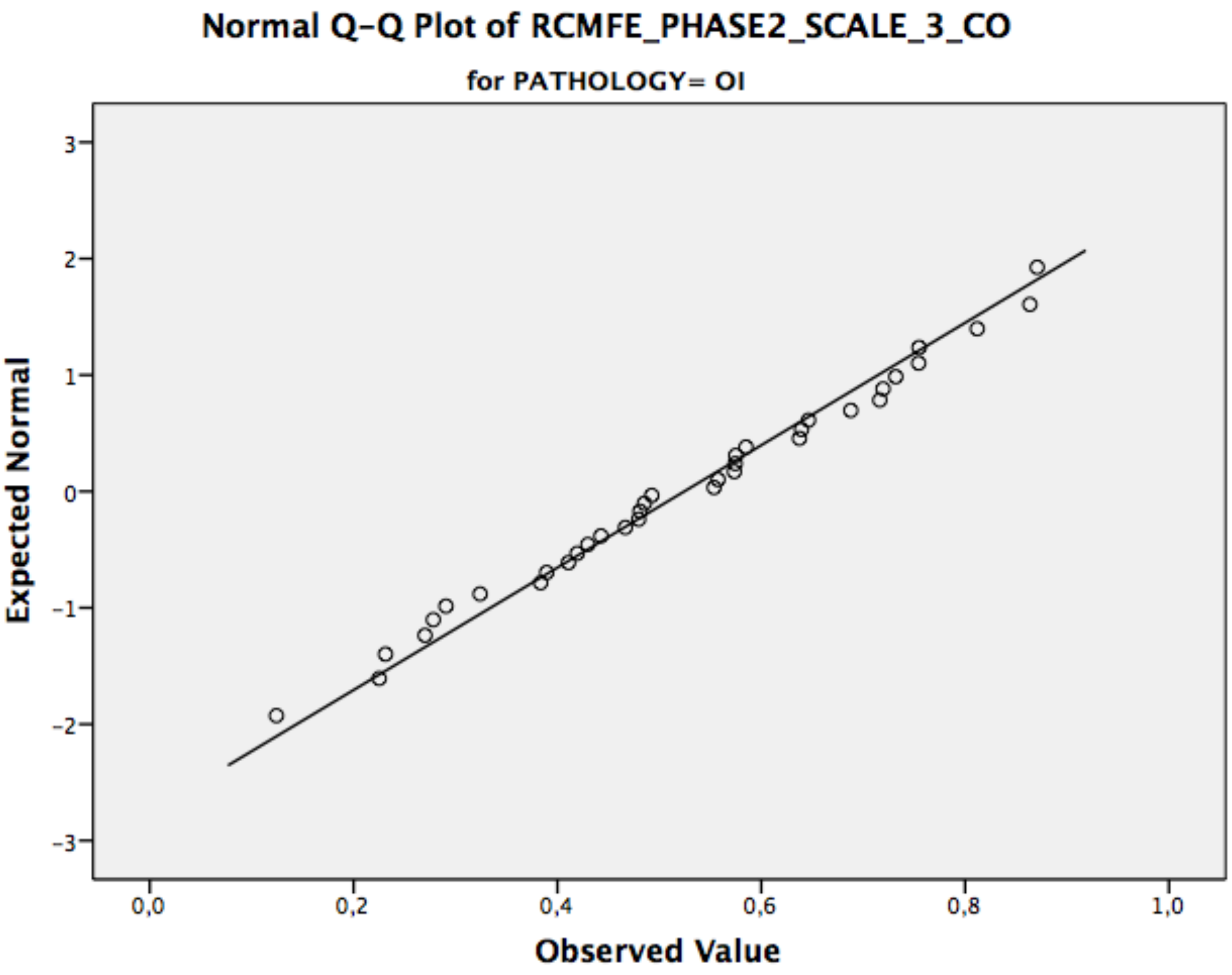

Normal Q-Q Plot of RCMFE_PHASE2_SCALE_3_CO
for PATHOLOGY= OI
Expected Normal
Observed Value
3
2
1
0
-1
-2
-3
0,0
0,2
0,4
0,6
0,8
1,0

RCMFE_PHASE2_SCALE_4_CO

Normal Q-Q Plots

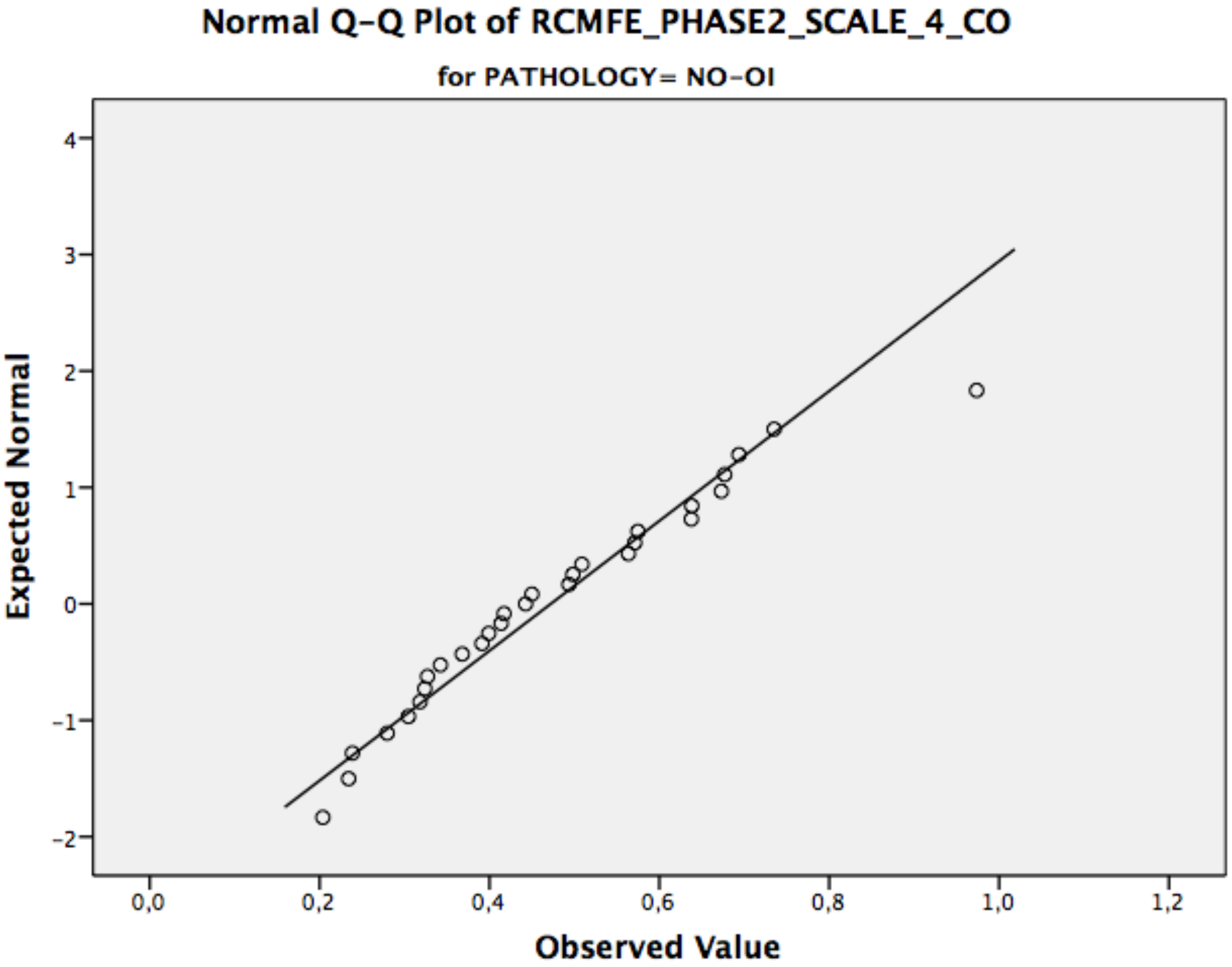

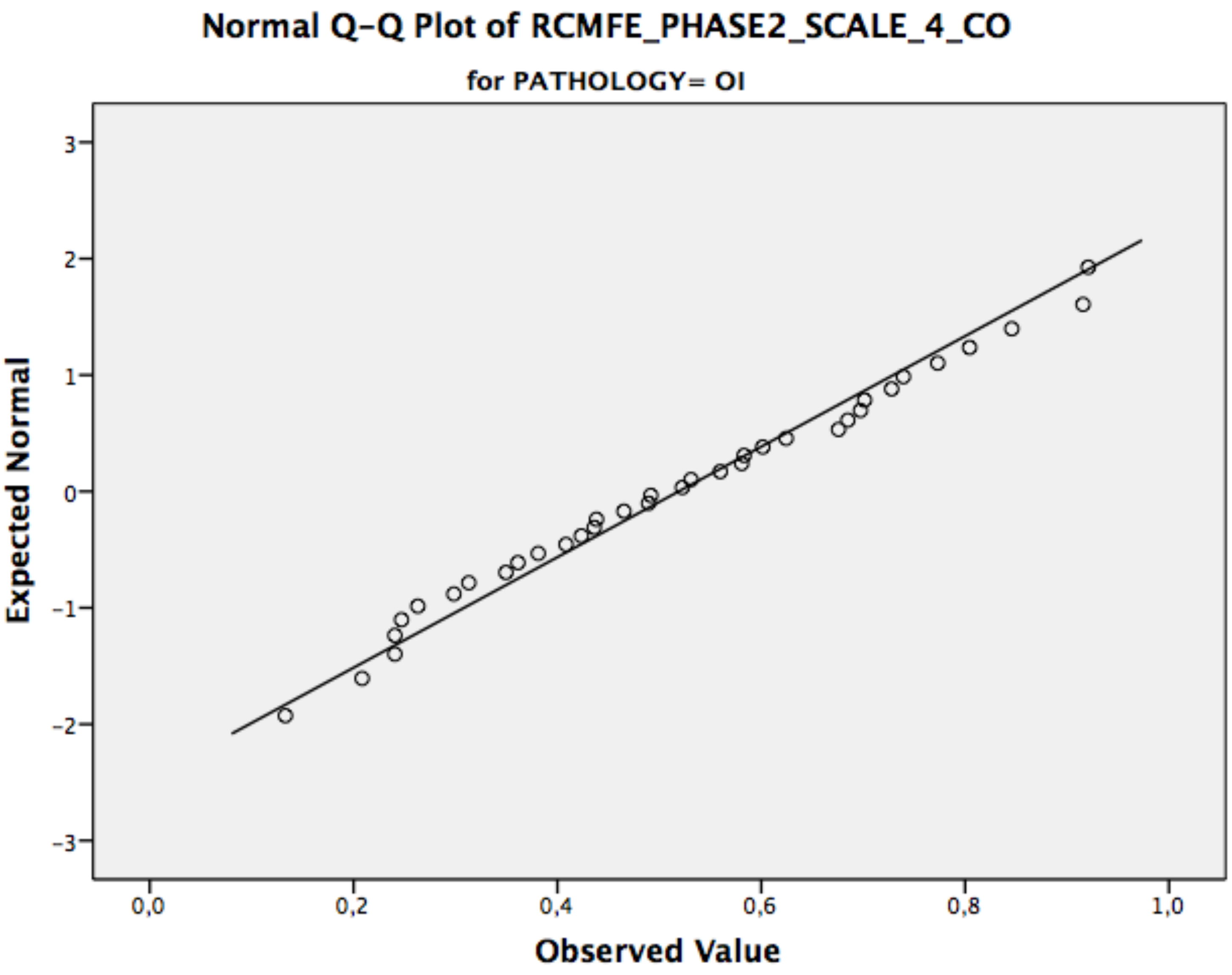
Normal Q-Q Plot of RCMFE_PHASE2_SCALE_4_CO
for PATHOLOGY= OI
Expected Normal
3
2
1
0
-1
-2
-3
0,0
0,2
0,4
0,6
0,8
1,0
Observed Value

RCMFE_PHASE2_SCALE_5_CO

Normal Q-Q Plots

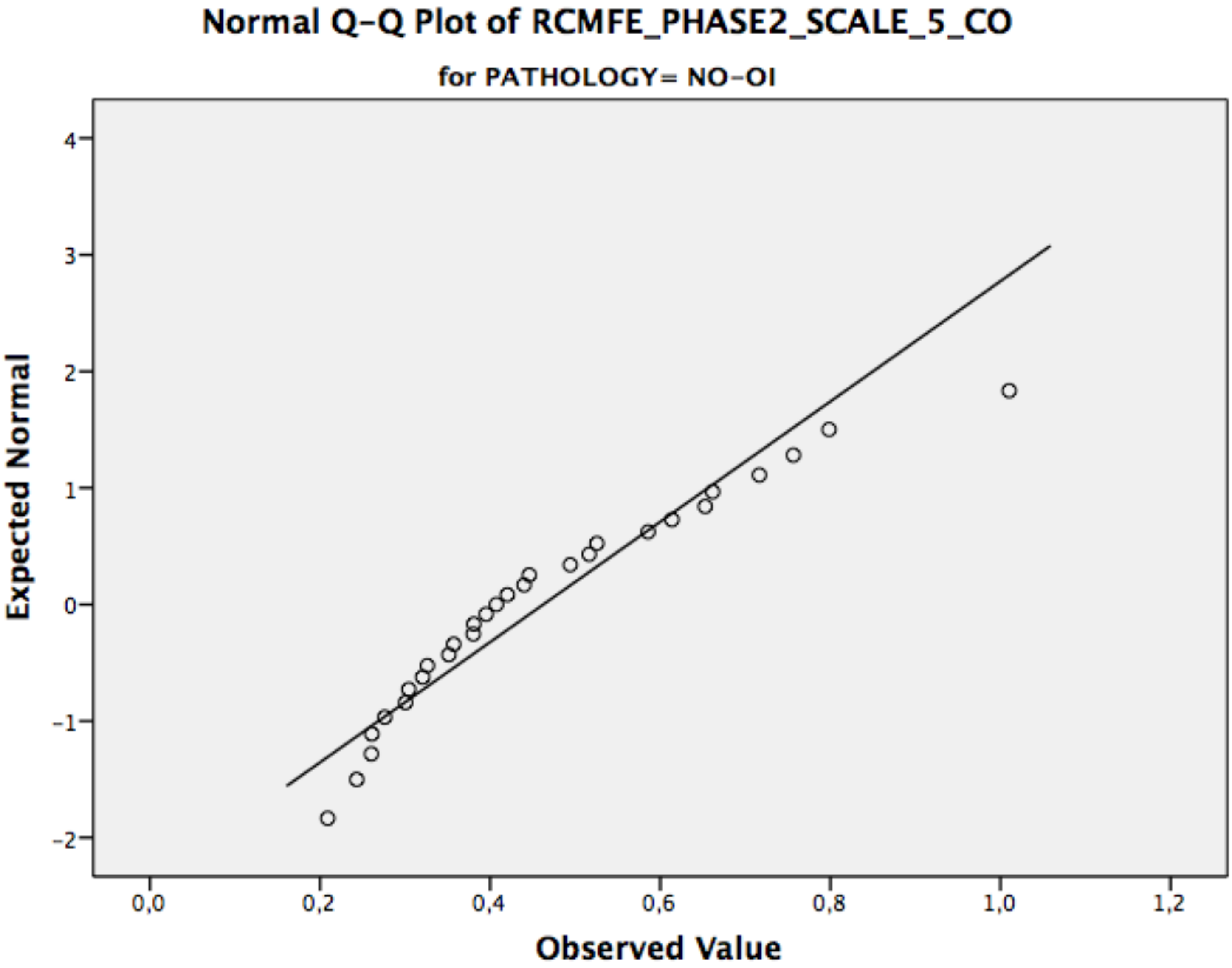

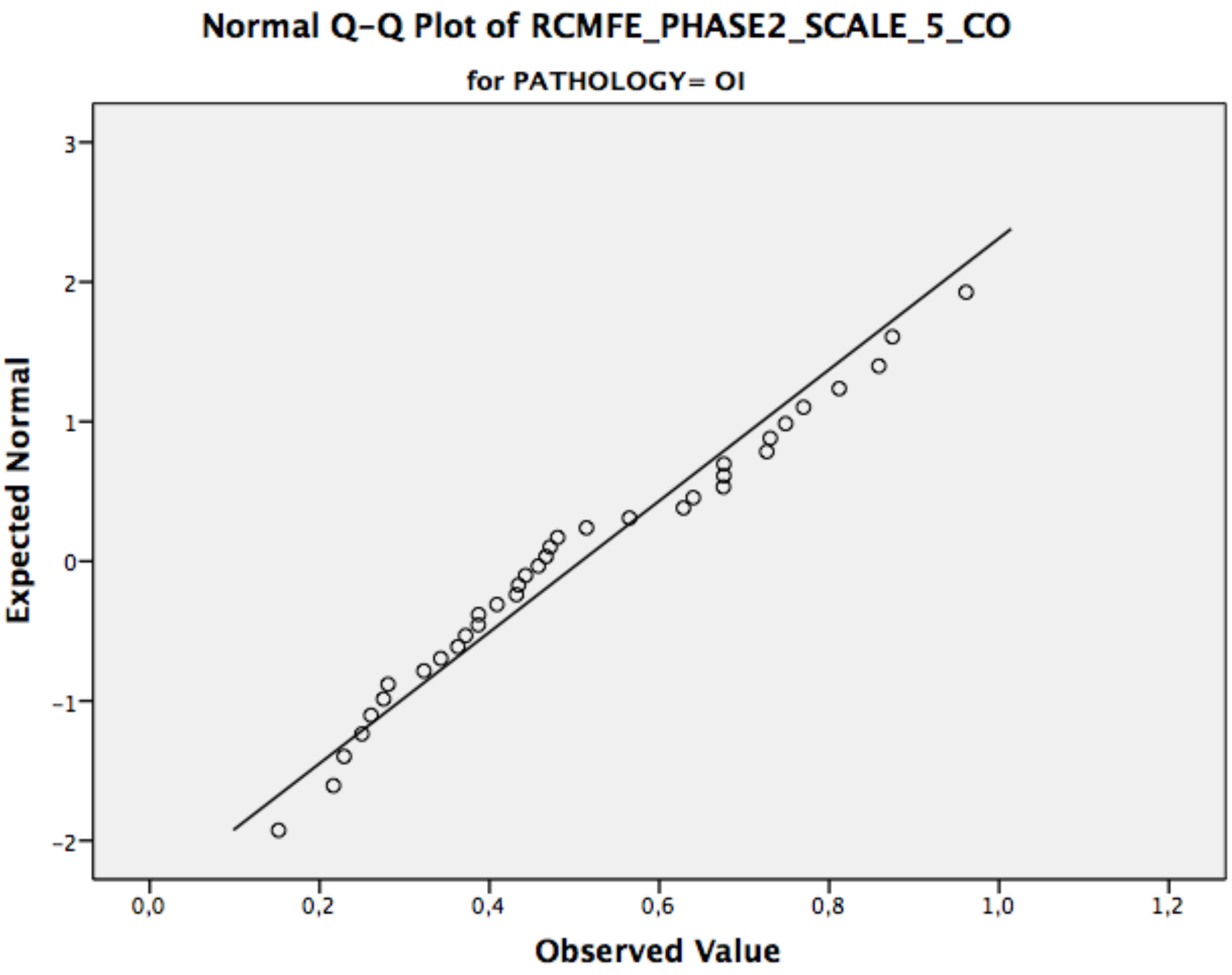
Normal Q-Q Plot of RCMFE_PHASE2_SCALE_5_CO
for PATHOLOGY= OI
Expected Normal
3
2
1
0
-1
-2
0,0
0,2
0,4
0,6
0,8
1,0
1,2
Observed Value

RCMFE_PHASE3_SCALE_1_CO

Normal Q-Q Plots

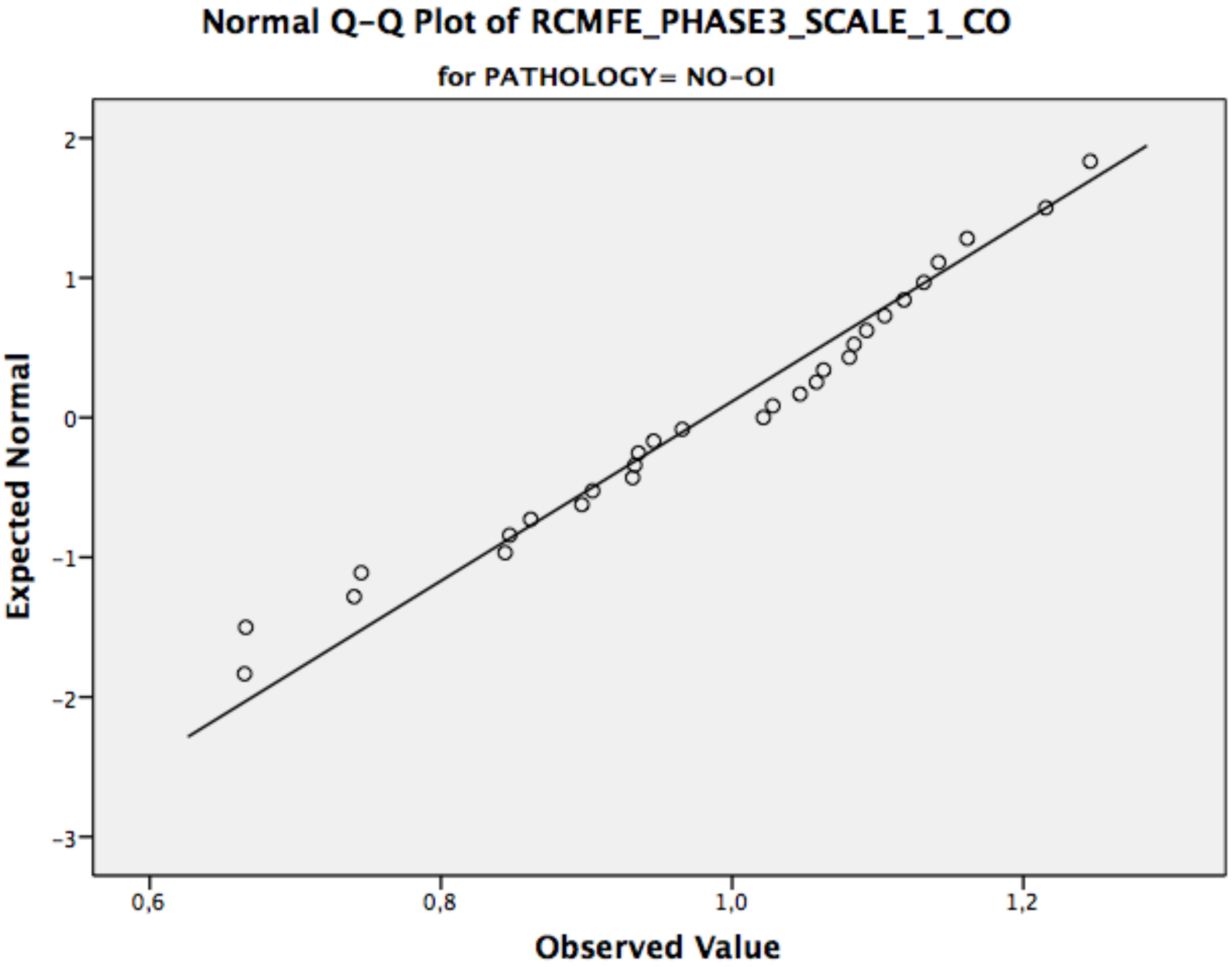

Normal Q-Q Plot of RCMFE_PHASE3_SCALE_1_CO

for PATHOLOGY= OI

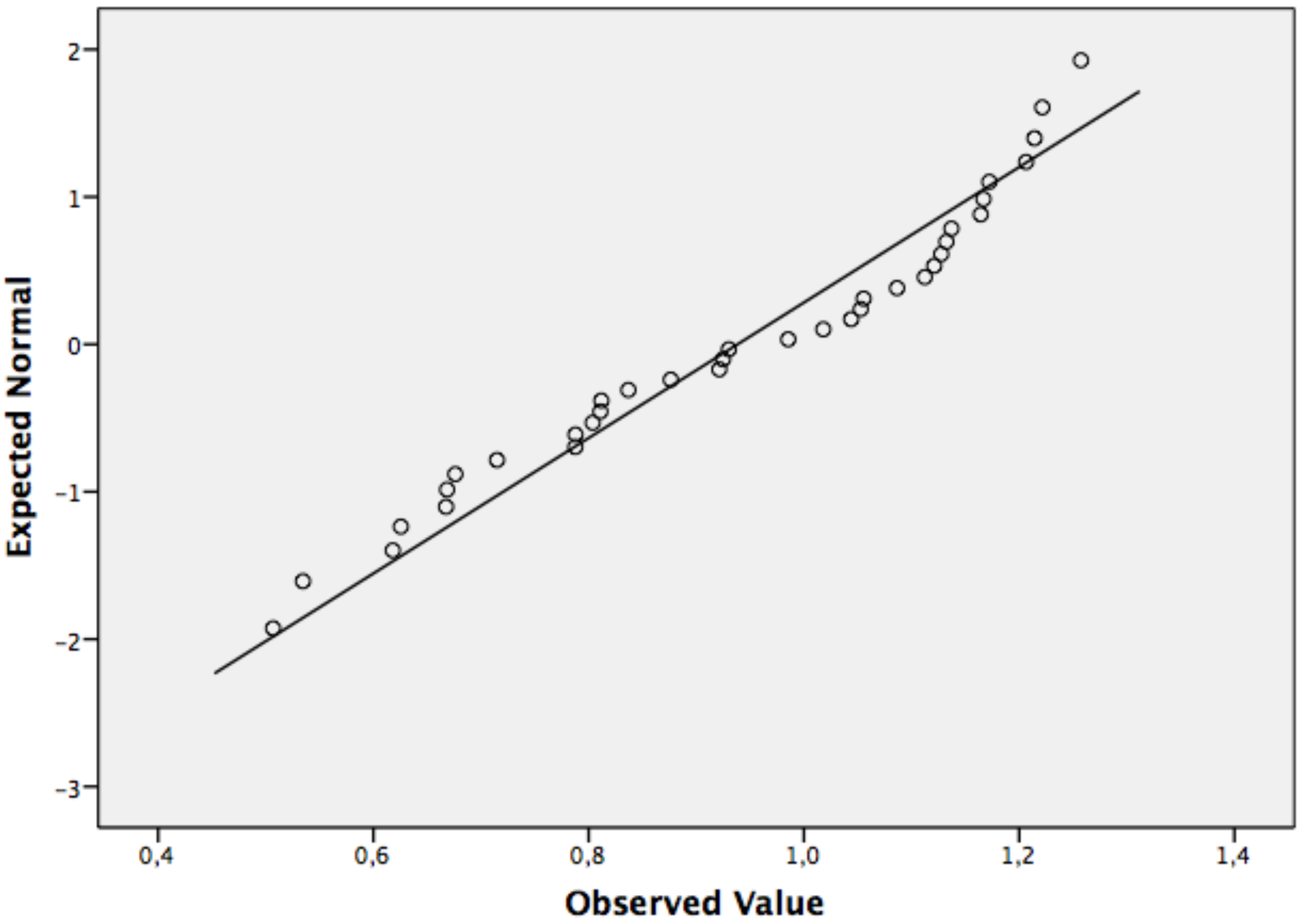

RCMFE_PHASE3_SCALE_2_CO

Normal Q-Q Plots

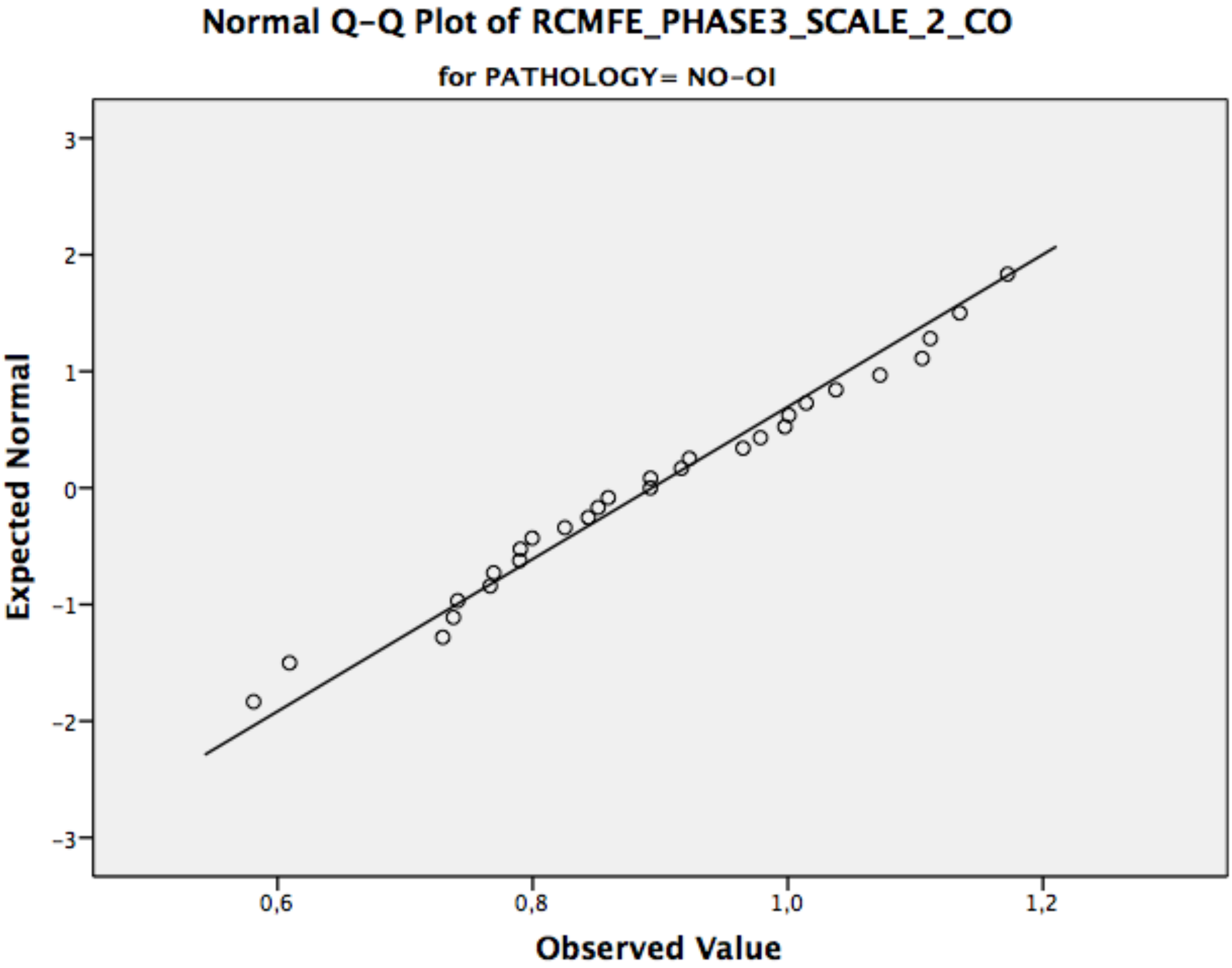

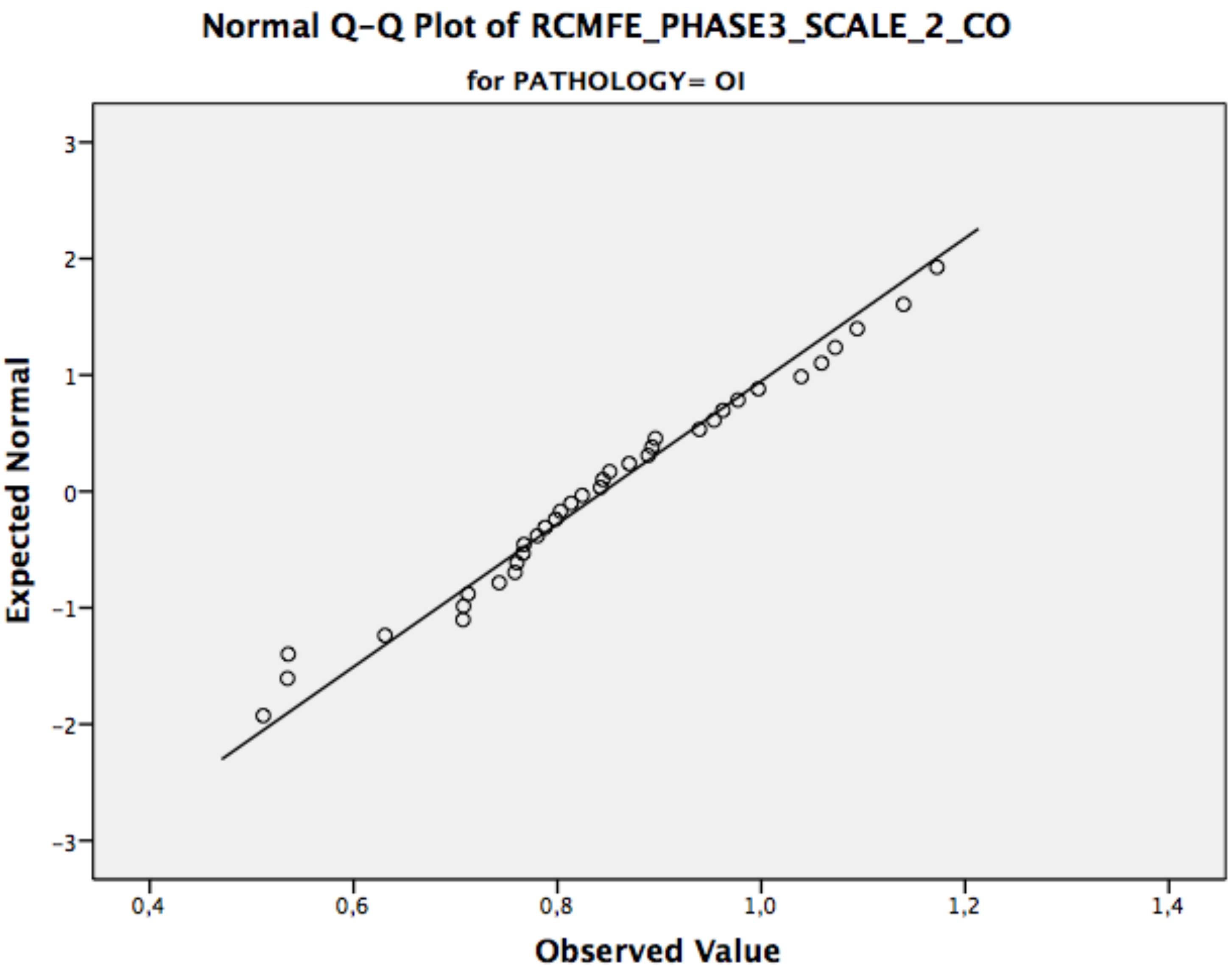
Normal Q-Q Plot of RCMFE_PHASE3_SCALE_2_CO
for PATHOLOGY= OI
Expected Normal
3
2
1
0
-1
-2
-3
0,4
0,6
0,8
1,0
1,2
1,4
Observed Value

RCMFE_PHASE3_SCALE_3_CO

Normal Q-Q Plots

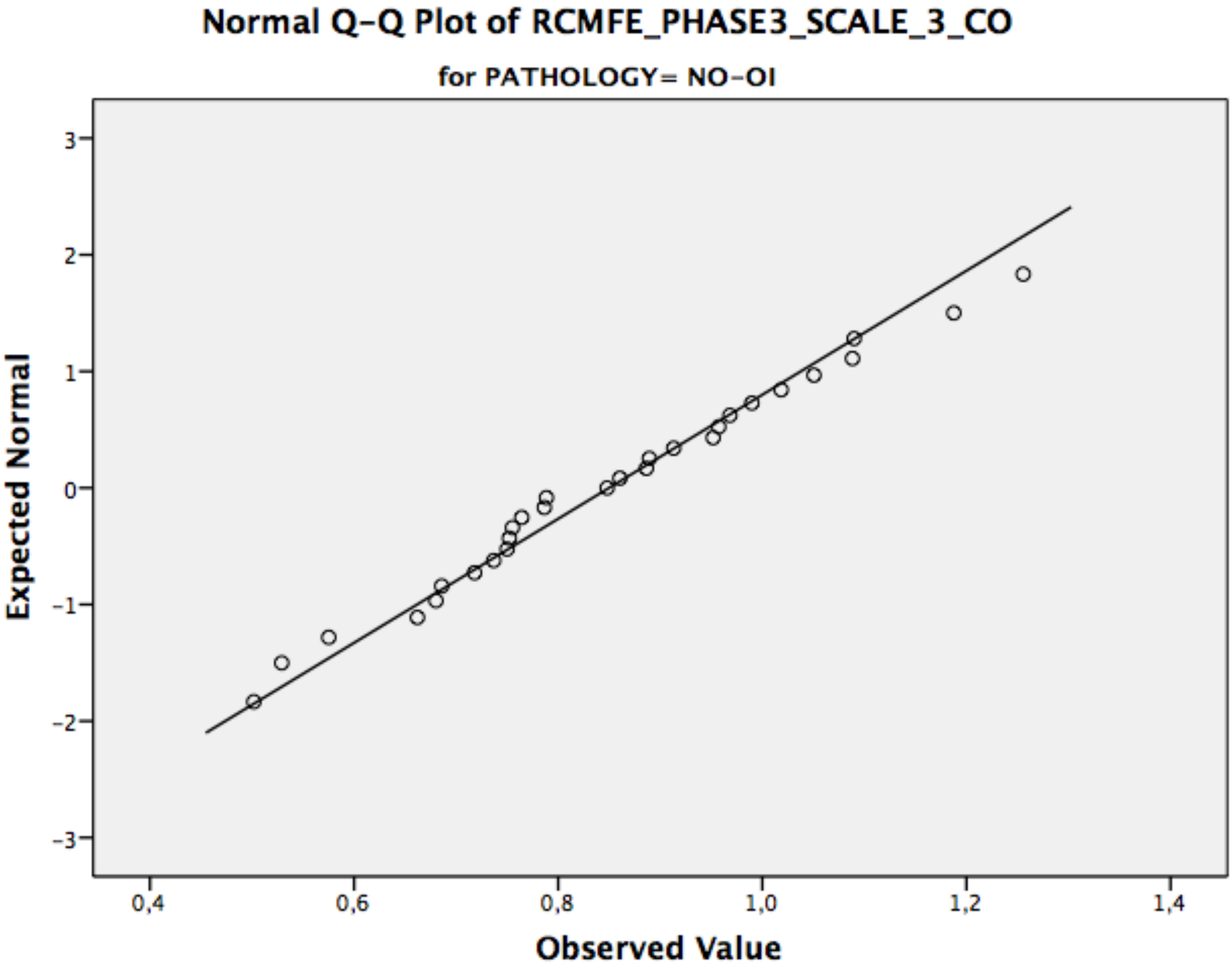

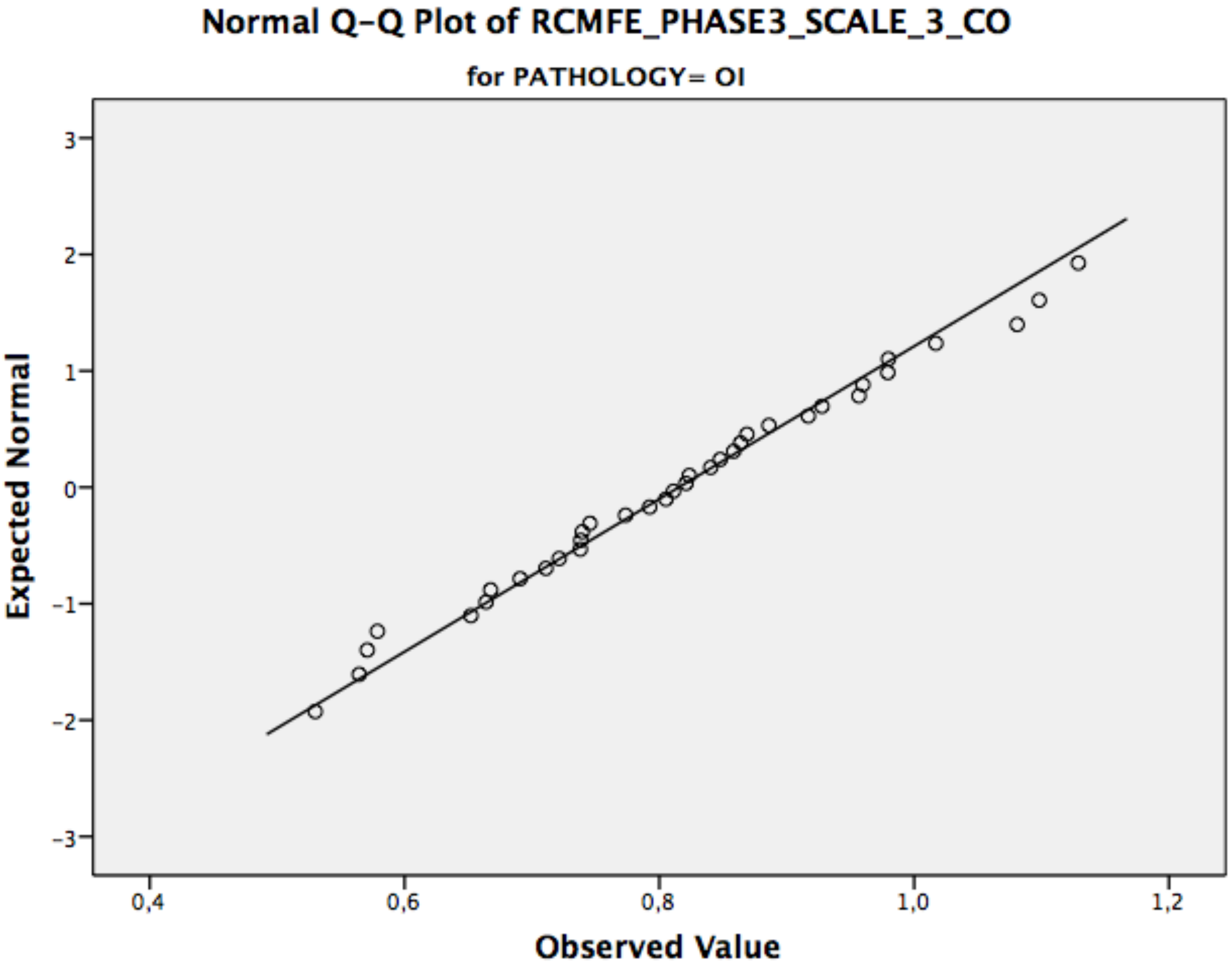
Normal Q-Q Plot of RCMFE_PHASE3_SCALE_3_CO
for PATHOLOGY= OI
Expected Normal
3
2
1
0
-1
-2
-3
0,4
0,6
0,8
1,0
1,2
Observed Value

Normal Q-Q Plots

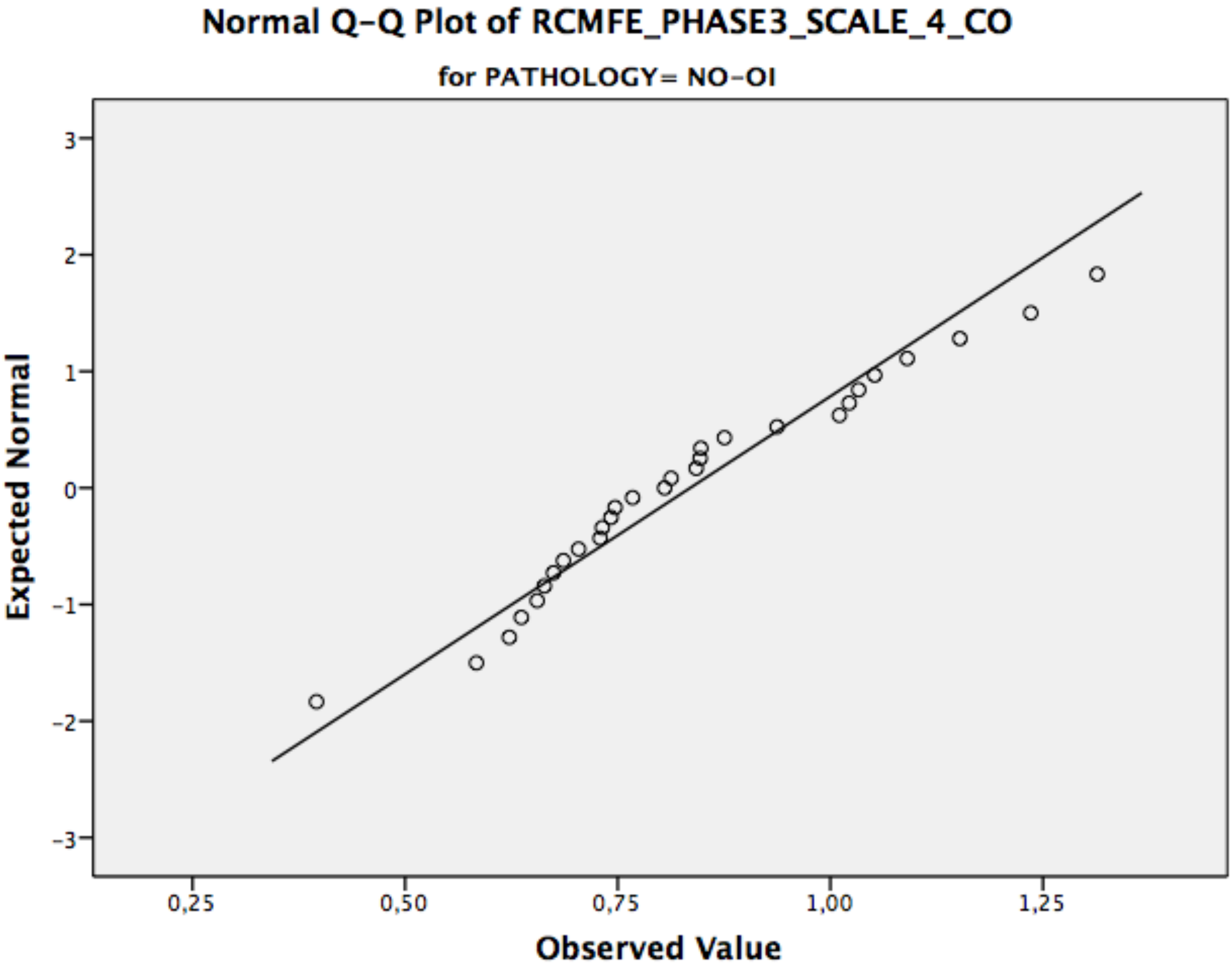

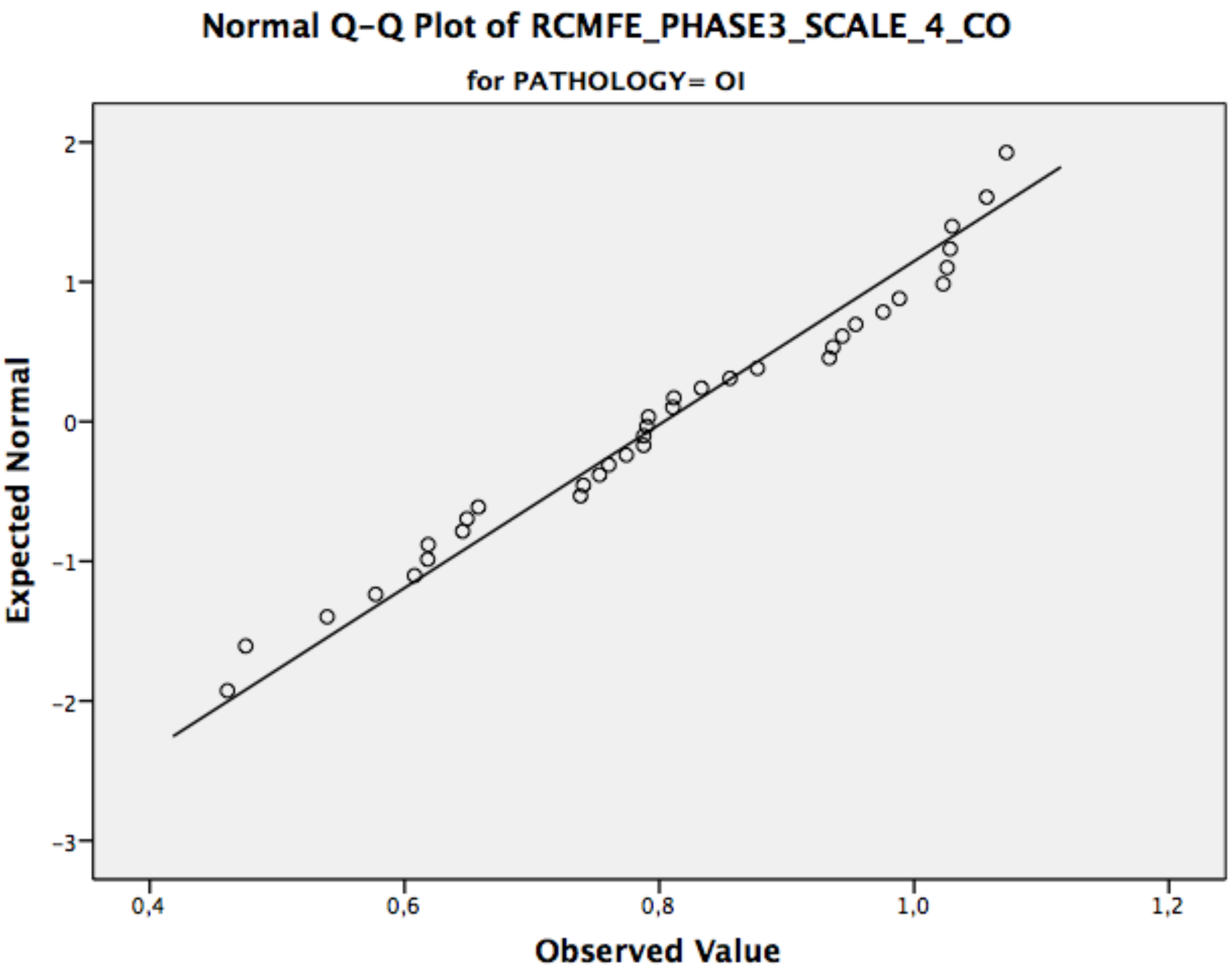
Normal Q-Q Plot of RCMFE_PHASE3_SCALE_4_CO
for PATHOLOGY= OI
Expected Normal
Observed Value
2
1
0
-1
-2
-3
0,4
0,6
0,8
1,0
1,2

RCMFE_PHASE3_SCALE_5_CO

Normal Q-Q Plots

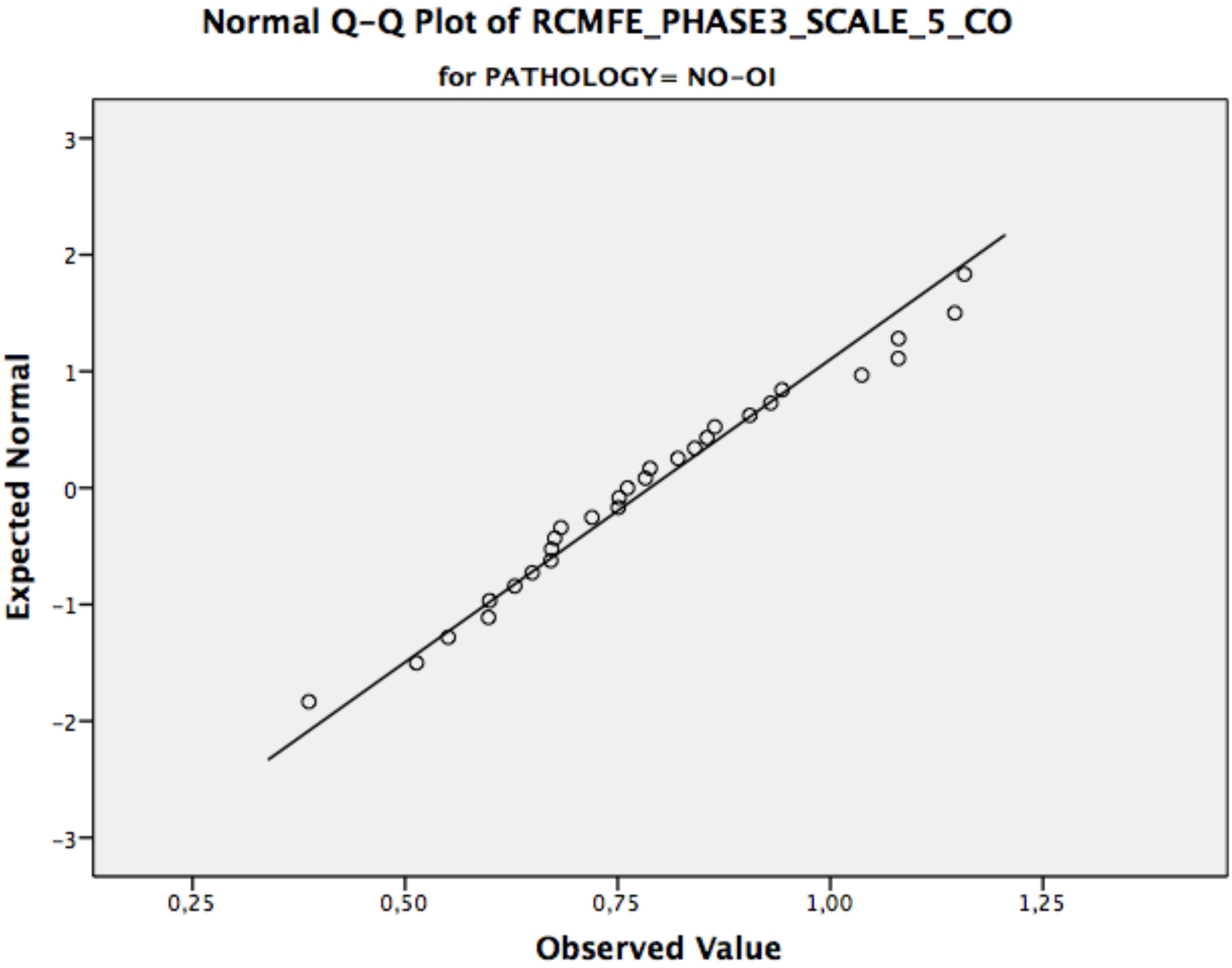

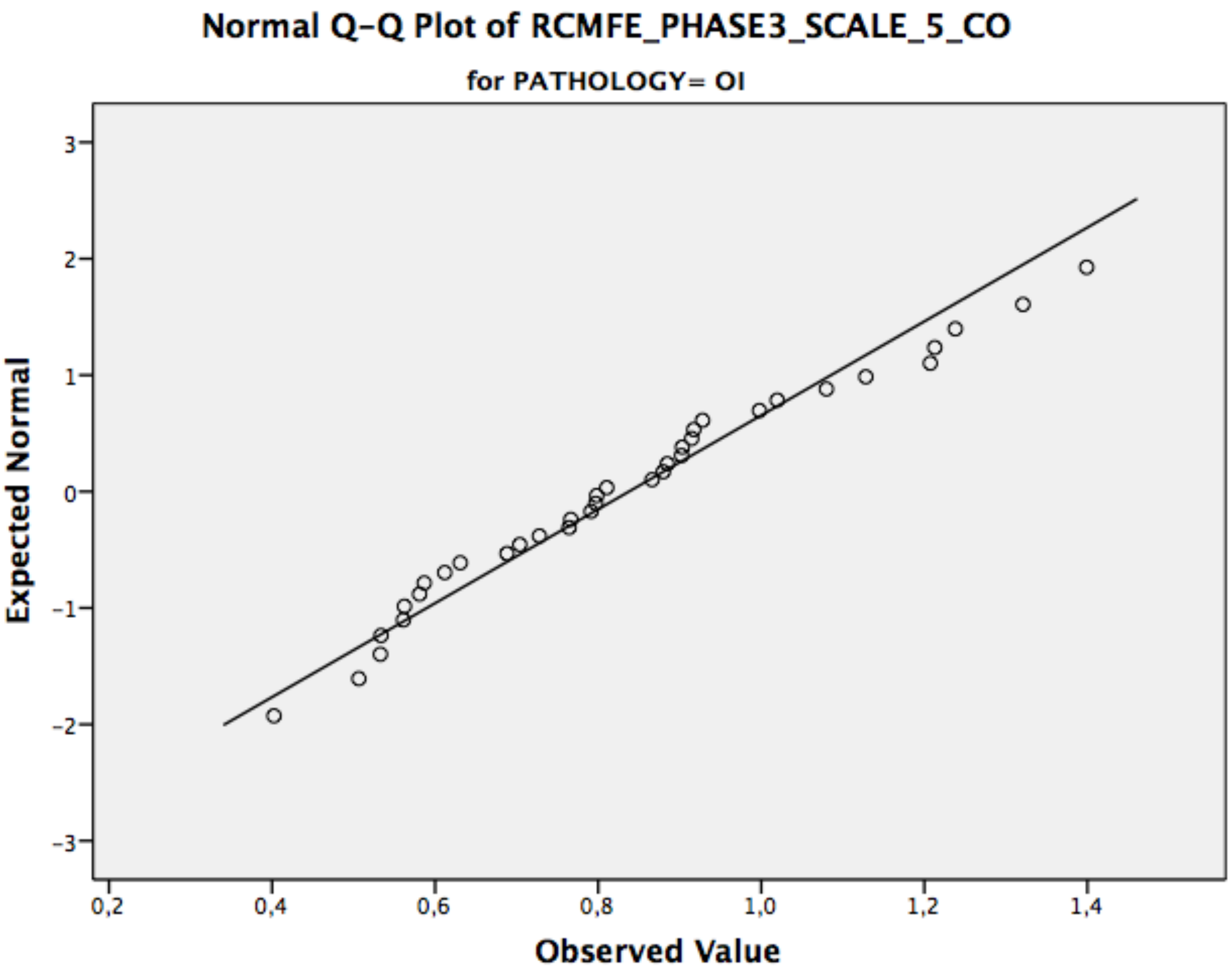

Normal Q-Q Plot of RCMFE_PHASE3_SCALE_5_CO
for PATHOLOGY= OI
Expected Normal
Observed Value
3
2
1
0
−1
−2
−3
0,2
0,4
0,6
0,8
1,0
1,2
1,4

RCMFE_PHASE4_SCALE_1_CO

Normal Q-Q Plots

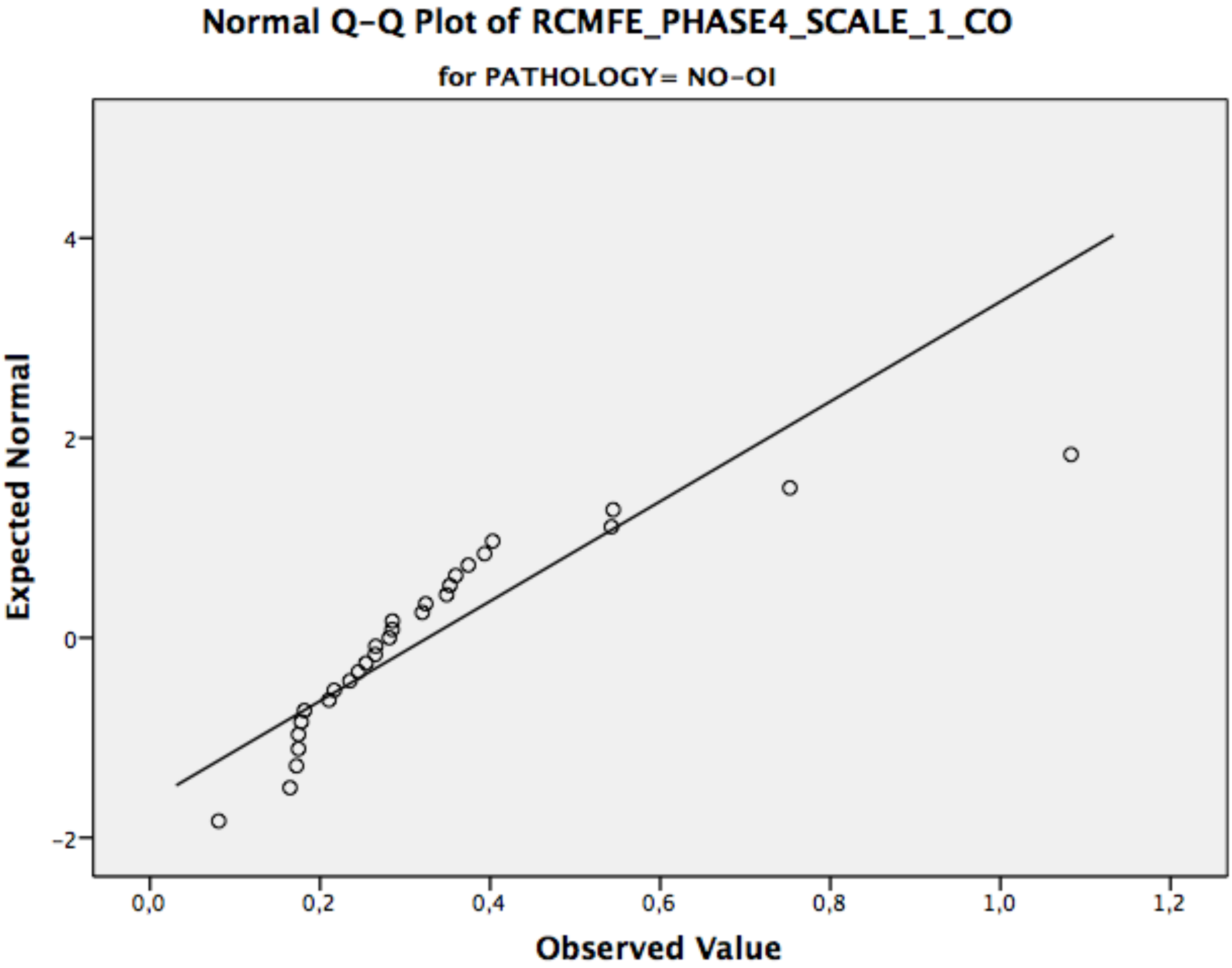

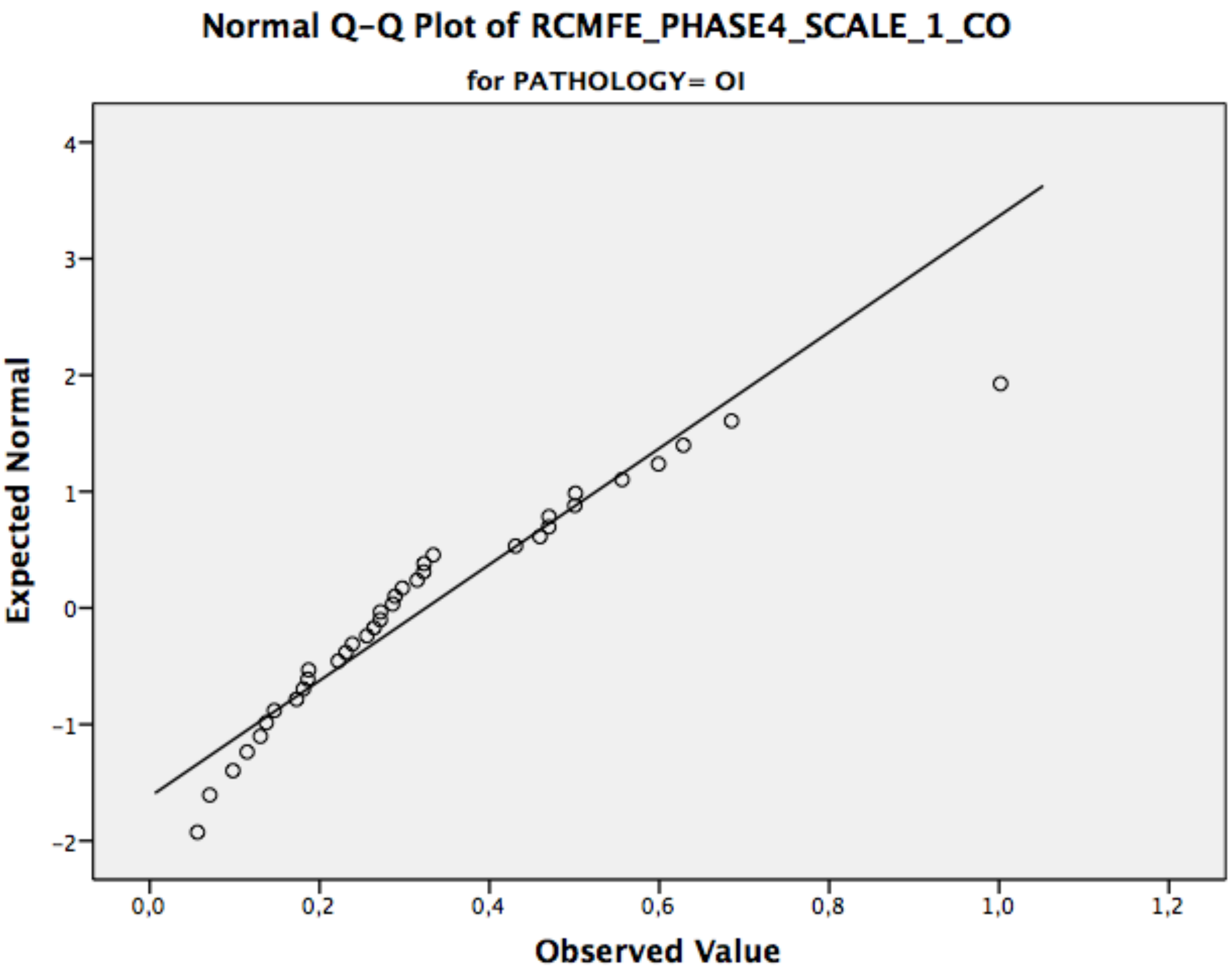

Normal Q-Q Plot of RCMFE_PHASE4_SCALE_1_CO
for PATHOLOGY= OI
Expected Normal
Observed Value
4
3
2
1
0
-1
-2
0,0
0,2
0,4
0,6
0,8
1,0
1,2

RCMFE_PHASE4_SCALE_2_CO

Normal Q-Q Plots

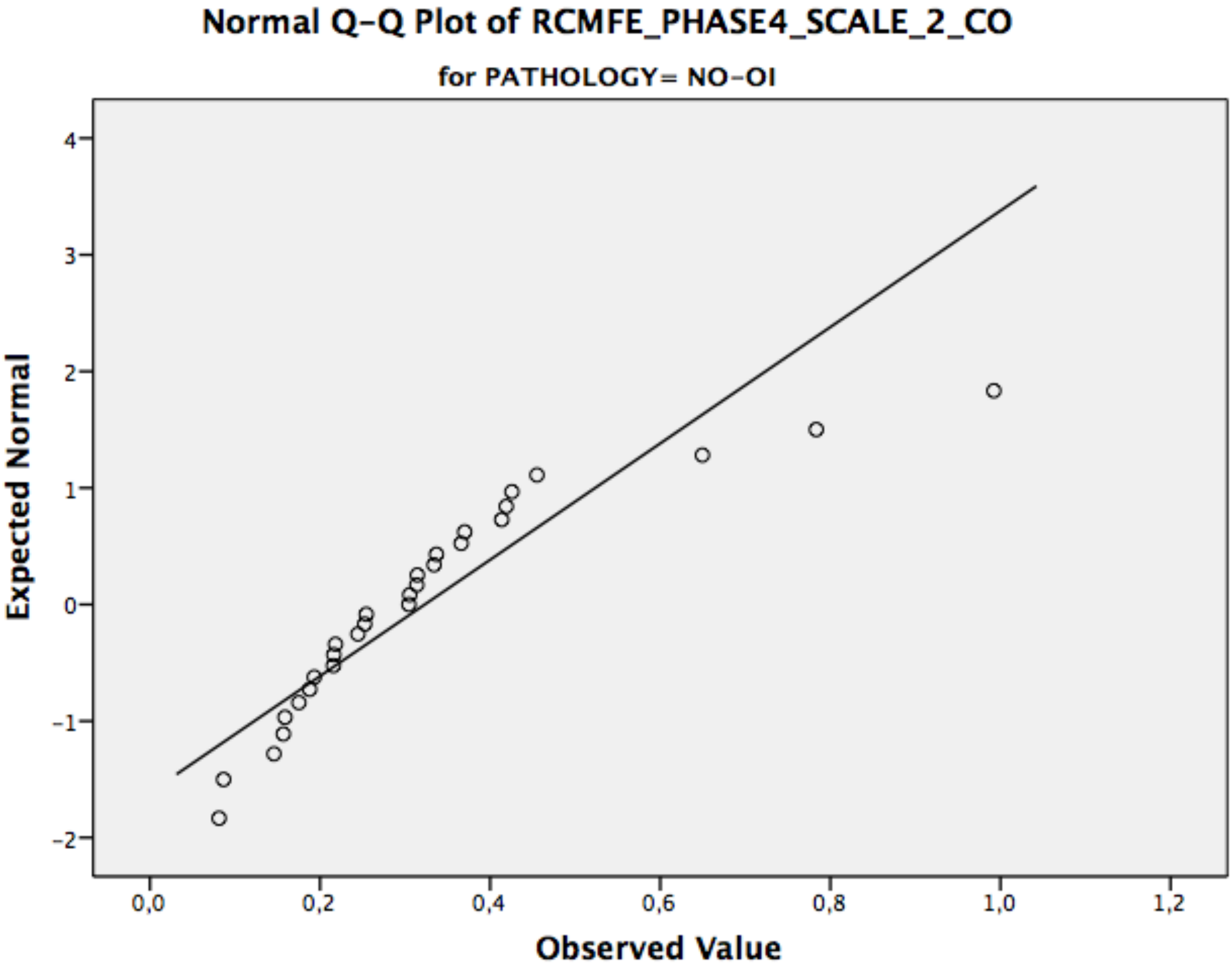

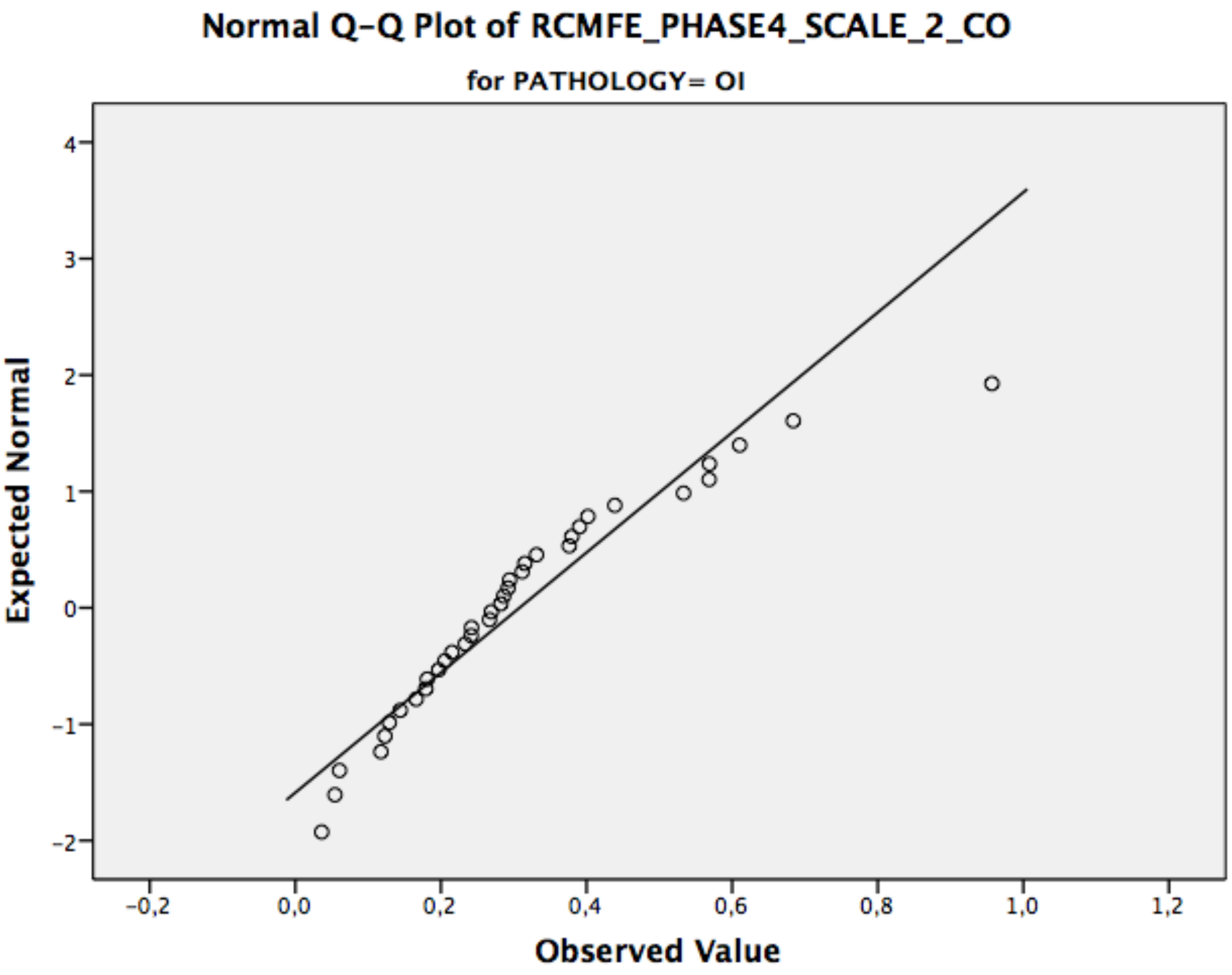
Normal Q-Q Plot of RCMFE_PHASE4_SCALE_2_CO
for PATHOLOGY= OI
Expected Normal
4
3
2
1
0
−1
−2
−0,2
0,0
0,2
0,4
0,6
0,8
1,0
1,2
Observed Value

RCMFE_PHASE4_SCALE_3_CO

Normal Q-Q Plots

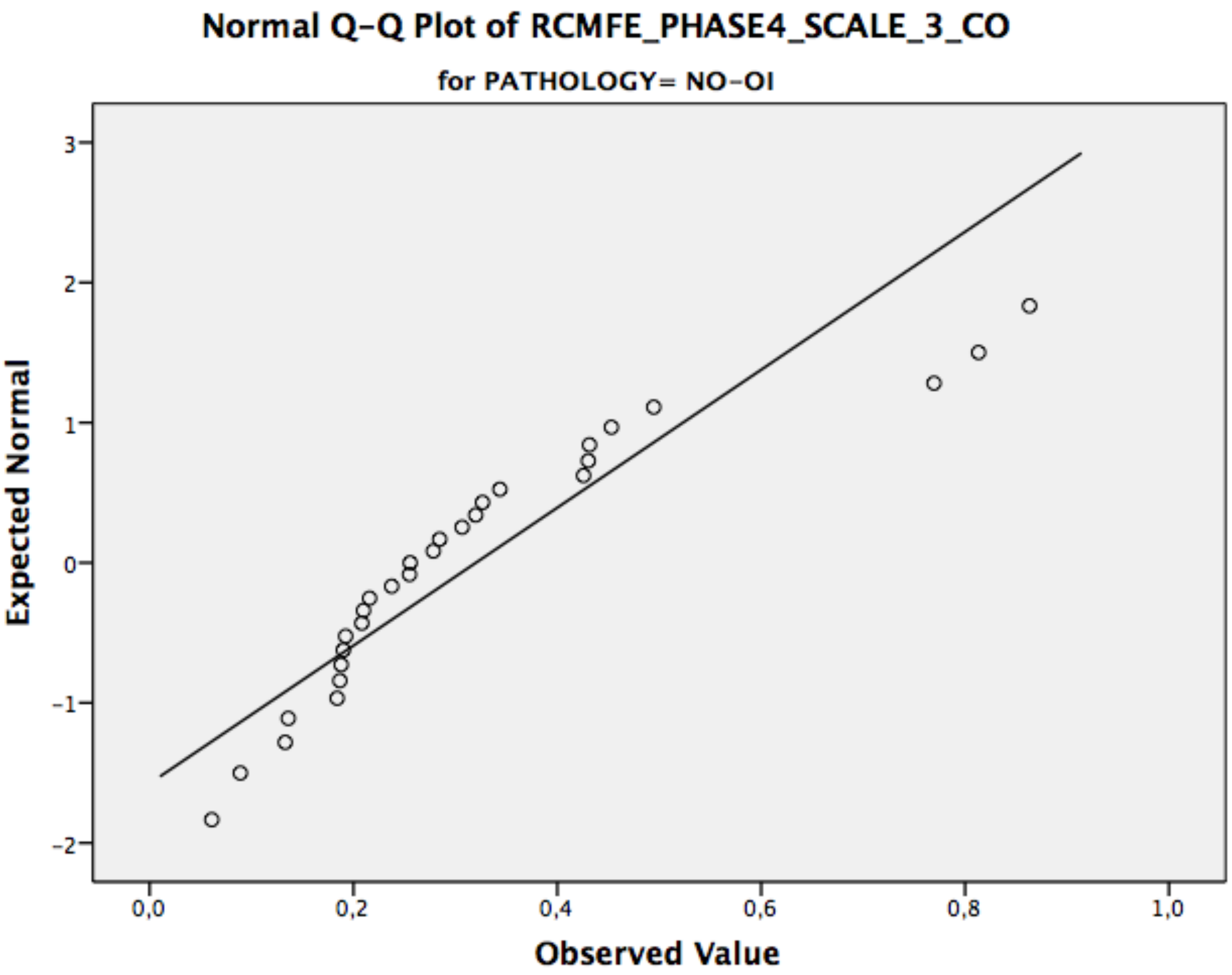

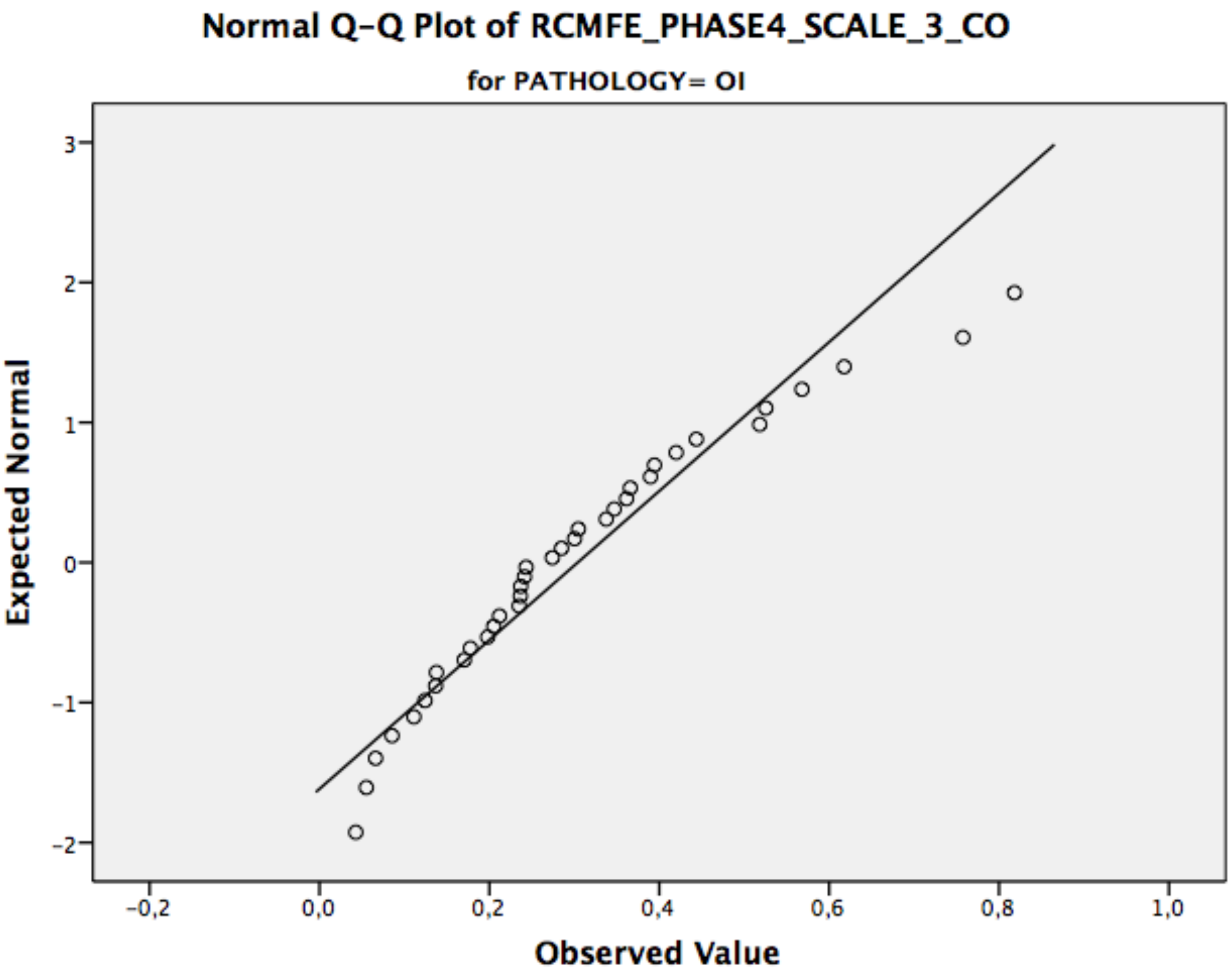

Normal Q-Q Plot of RCMFE_PHASE4_SCALE_3_CO
for PATHOLOGY= OI
Expected Normal
Observed Value
3
2
1
0
-1
-2
-0,2
0,0
0,2
0,4
0,6
0,8
1,0

RCMFE_PHASE4_SCALE_4_CO

Normal Q-Q Plots

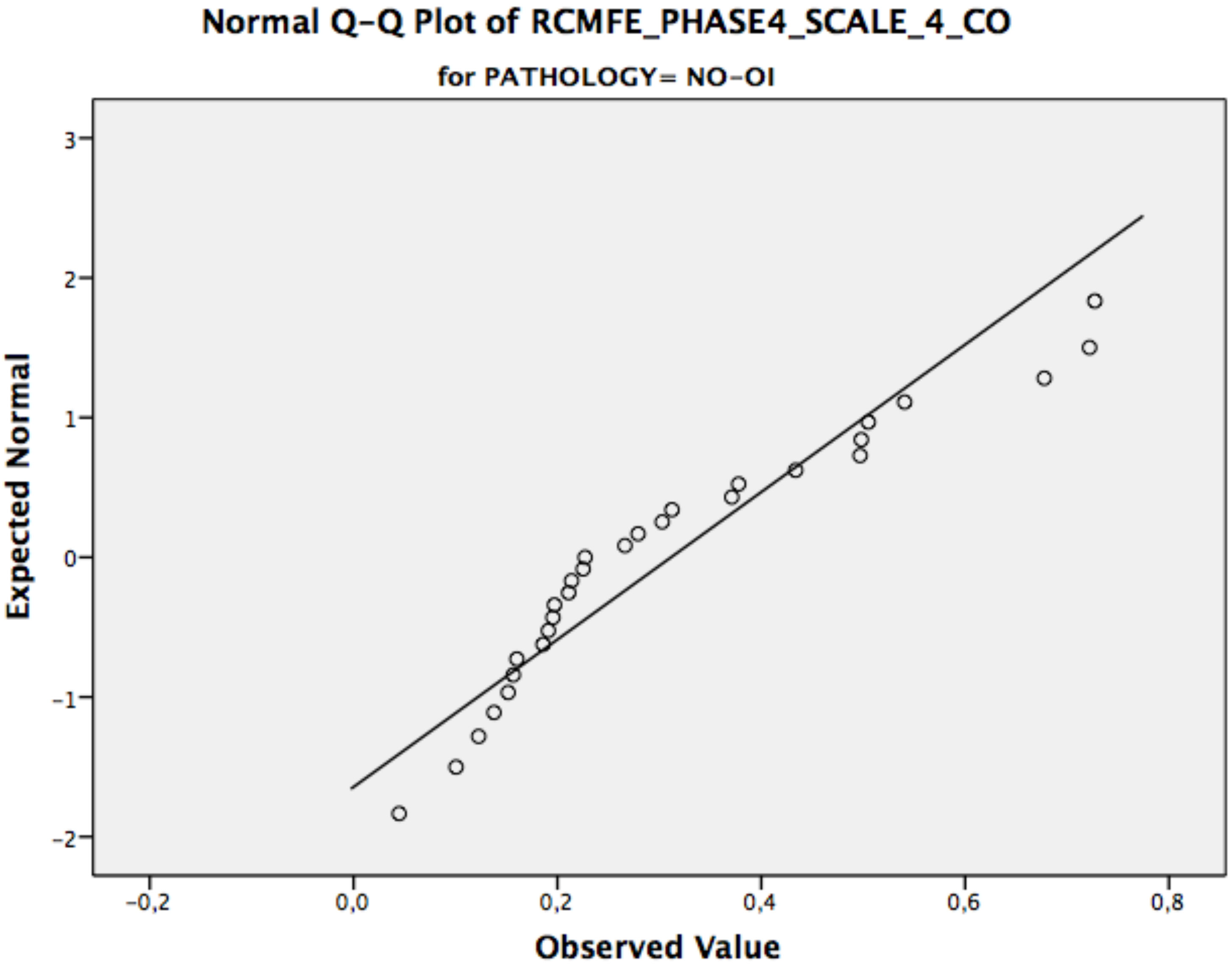

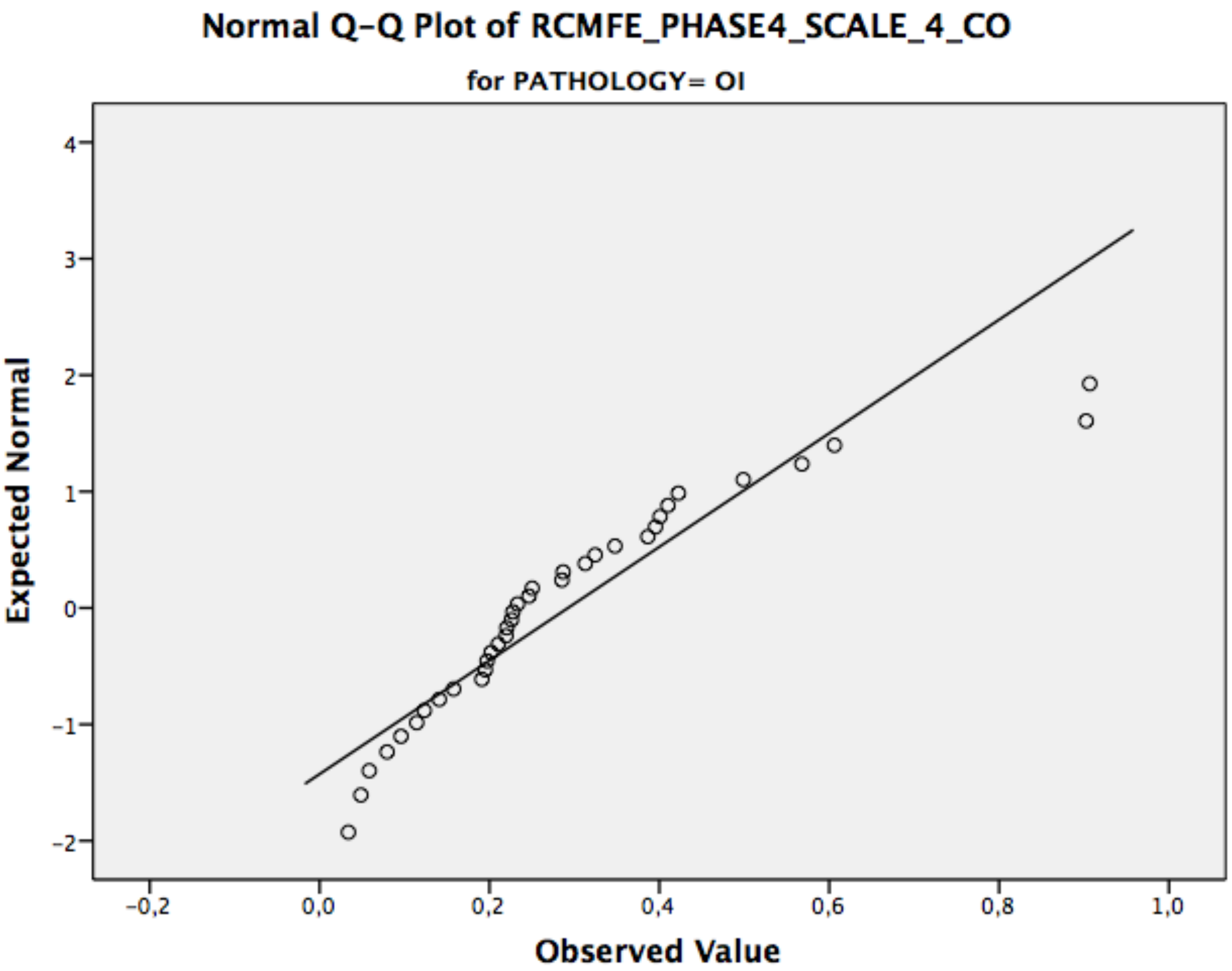
Normal Q-Q Plot of RCMFE_PHASE4_SCALE_4_CO
for PATHOLOGY= OI
Expected Normal
Observed Value
4
3
2
1
0
-1
-2
-0,2
0,0
0,2
0,4
0,6
0,8
1,0

RCMFE_PHASE4_SCALE_5_CO

Normal Q-Q Plots

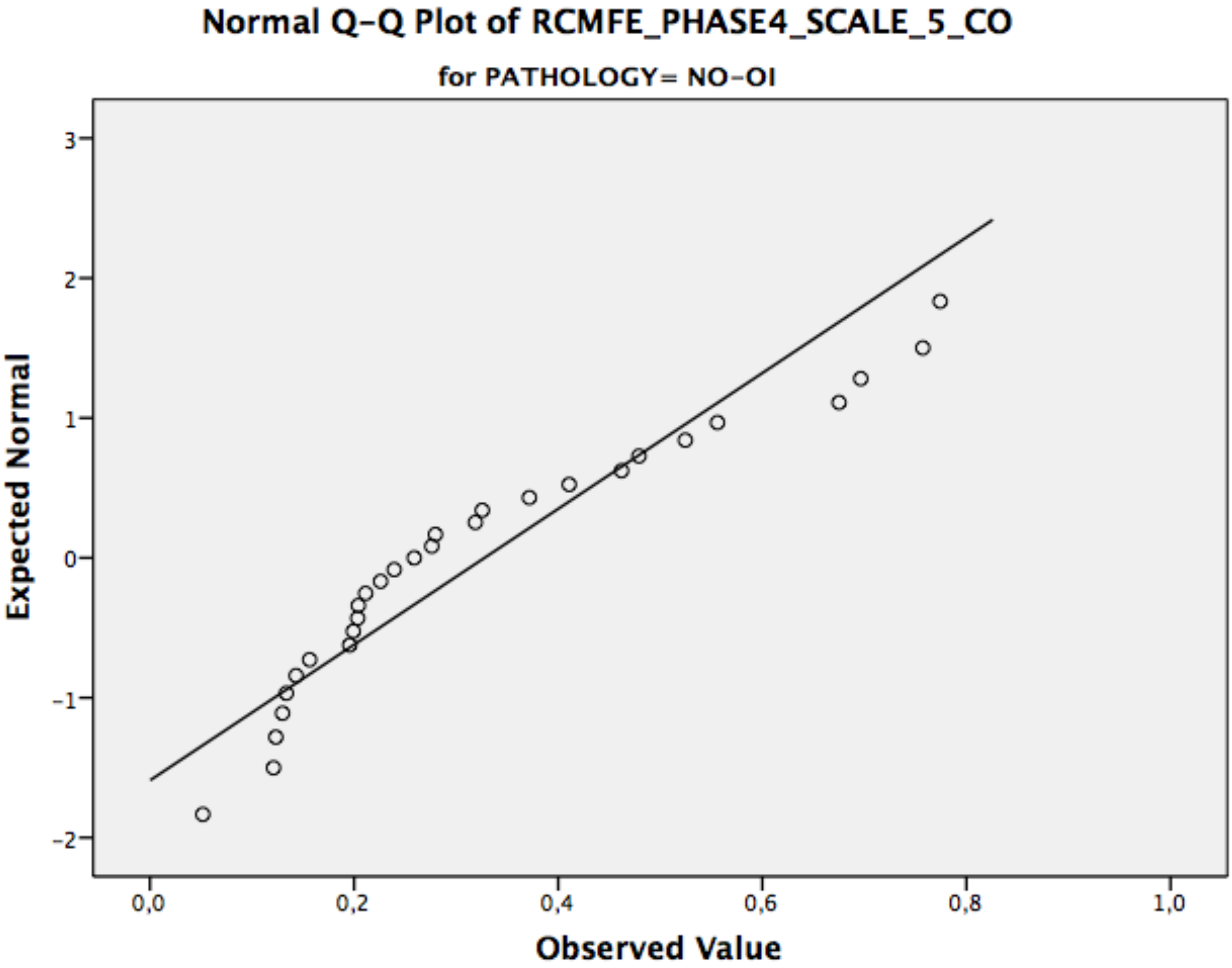

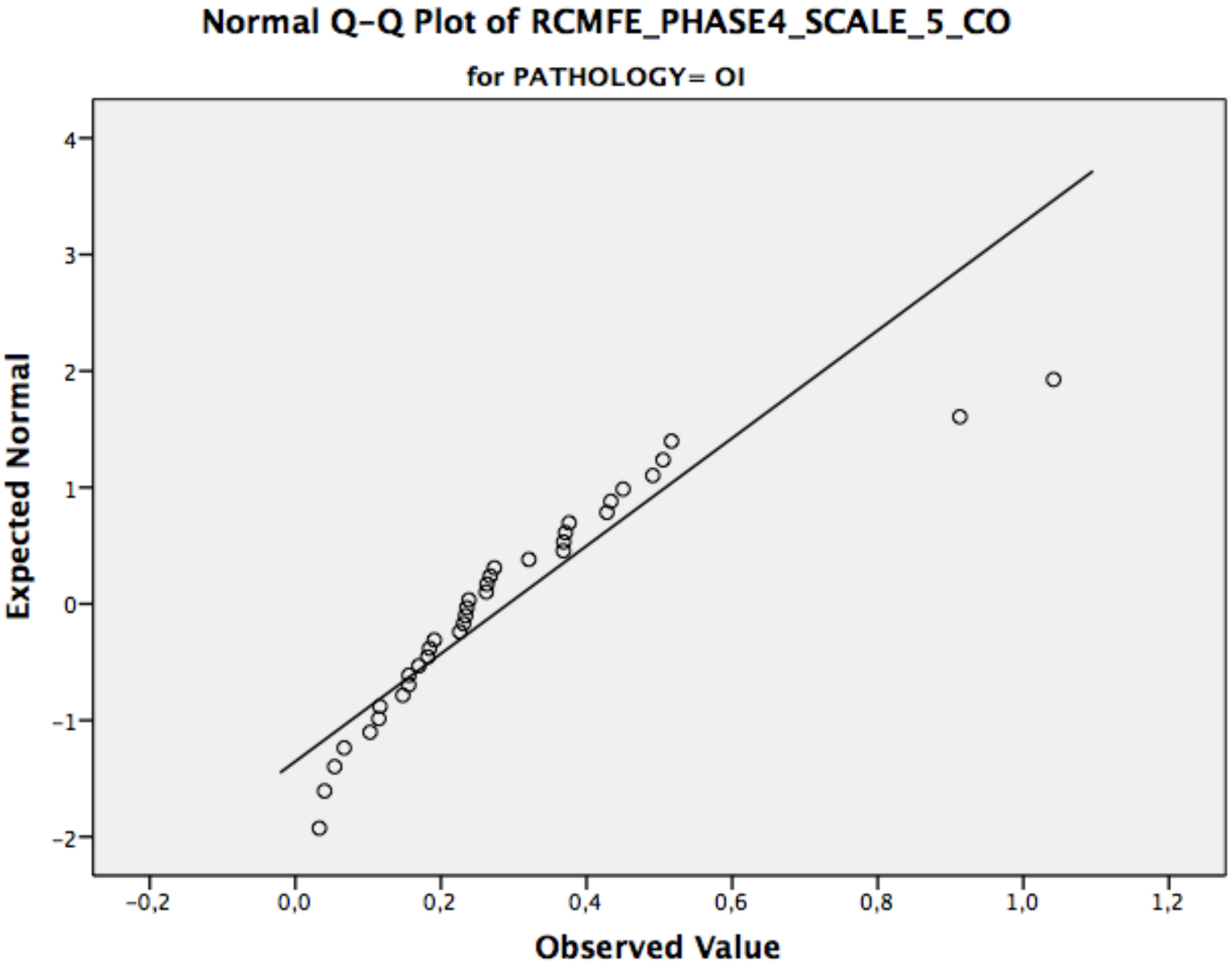

Normal Q-Q Plot of RCMFE_PHASE4_SCALE_5_CO
for PATHOLOGY= OI
Expected Normal
Observed Value
4
3
2
1
0
-1
-2
-0,2
0,0
0,2
0,4
0,6
0,8
1,0
1,2

RCMFE_PHASE5_SCALE_1_CO

Normal Q-Q Plots

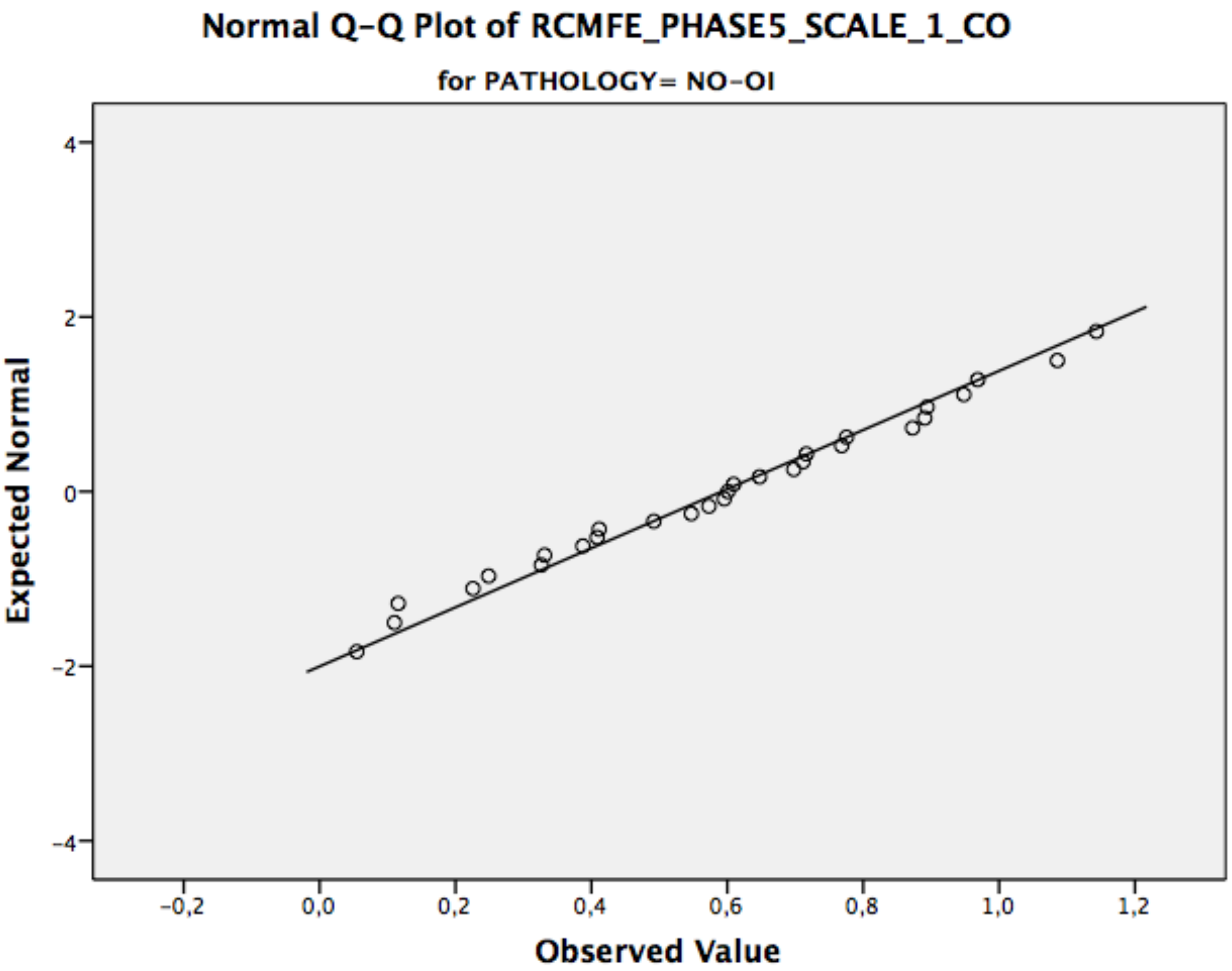

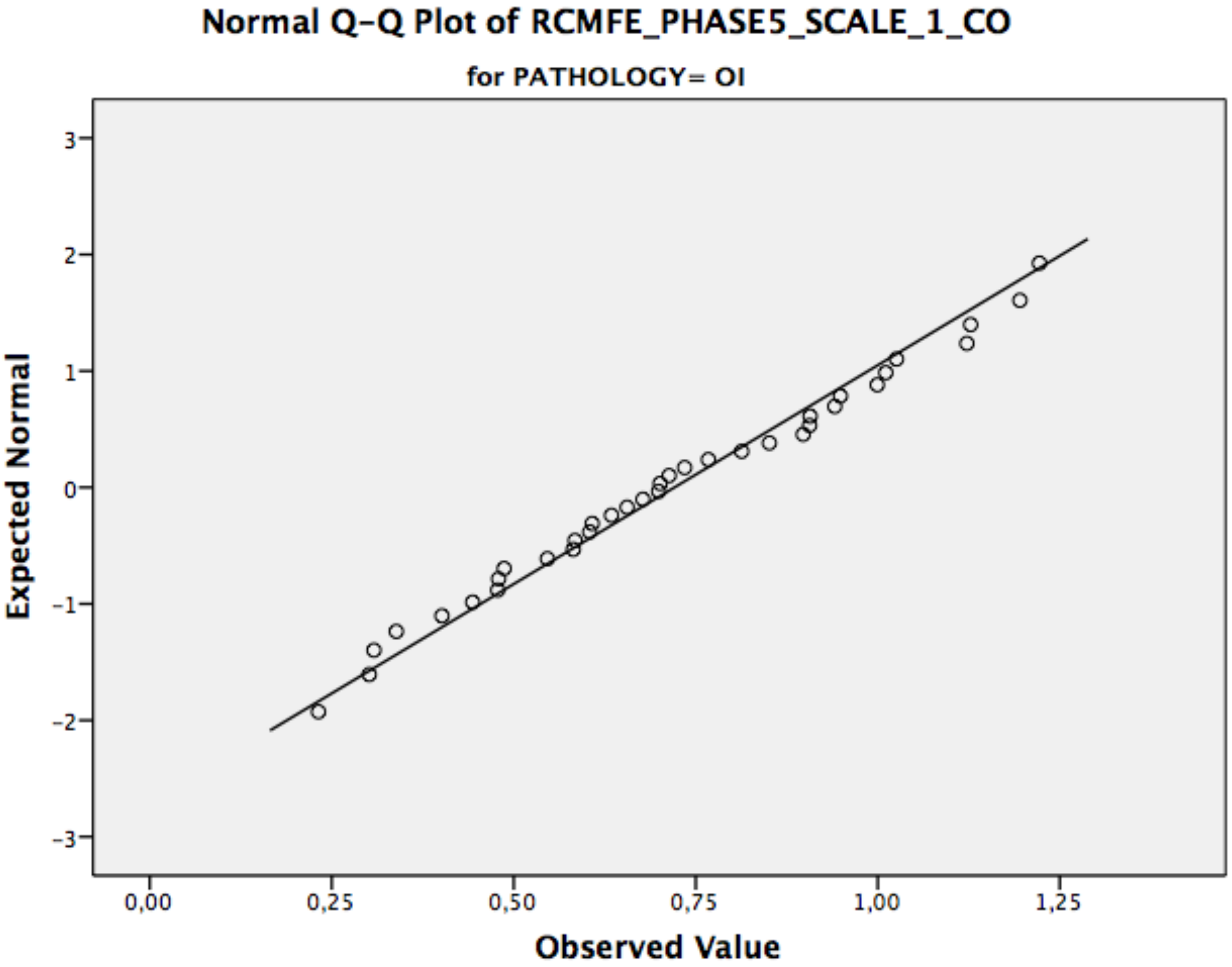

Normal Q-Q Plot of RCMFE_PHASE5_SCALE_1_CO
for PATHOLOGY= OI
Expected Normal
Observed Value
3
2
1
0
-1
-2
-3
0,00
0,25
0,50
0,75
1,00
1,25

RCMFE_PHASE5_SCALE_2_CO

Normal Q-Q Plots

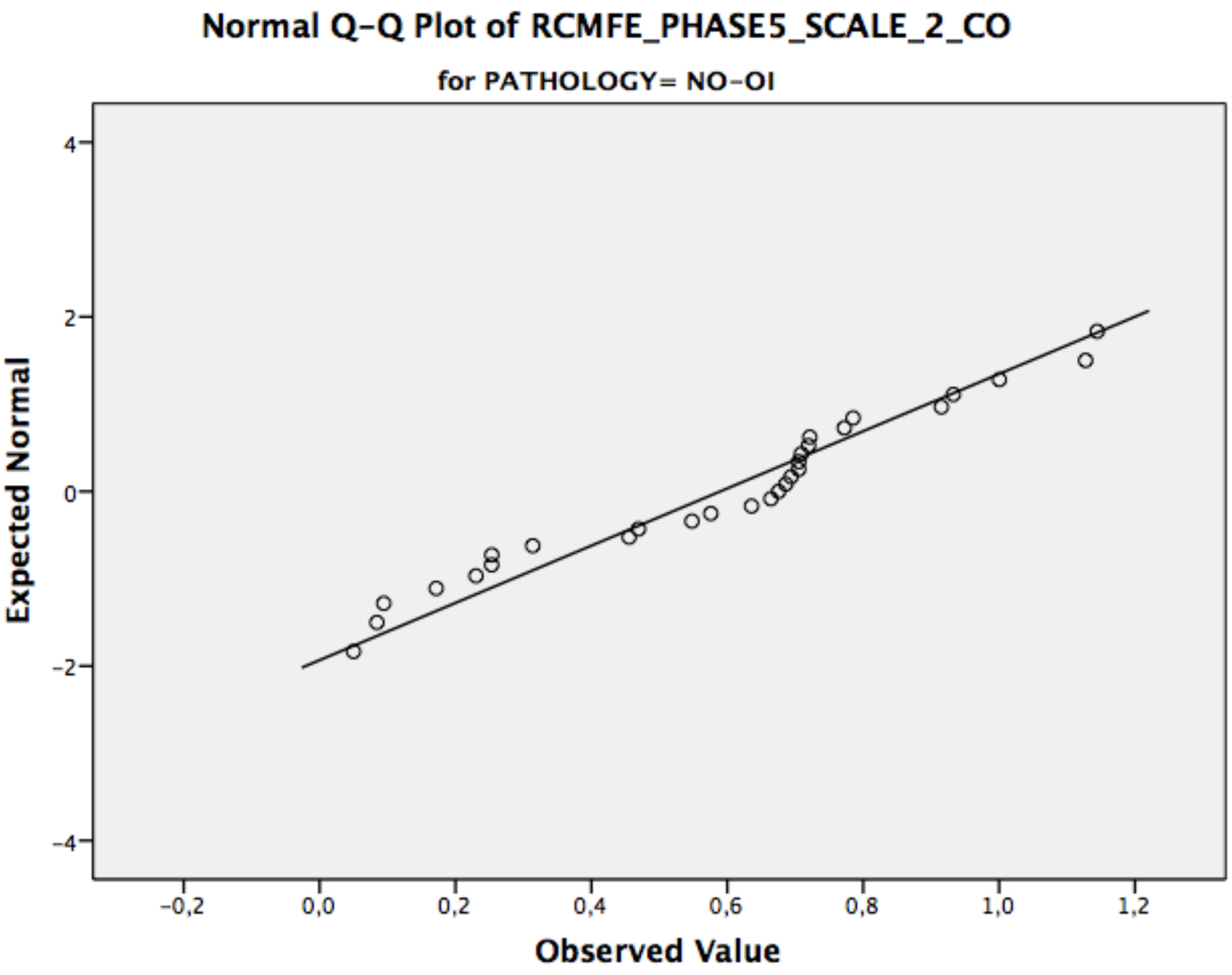

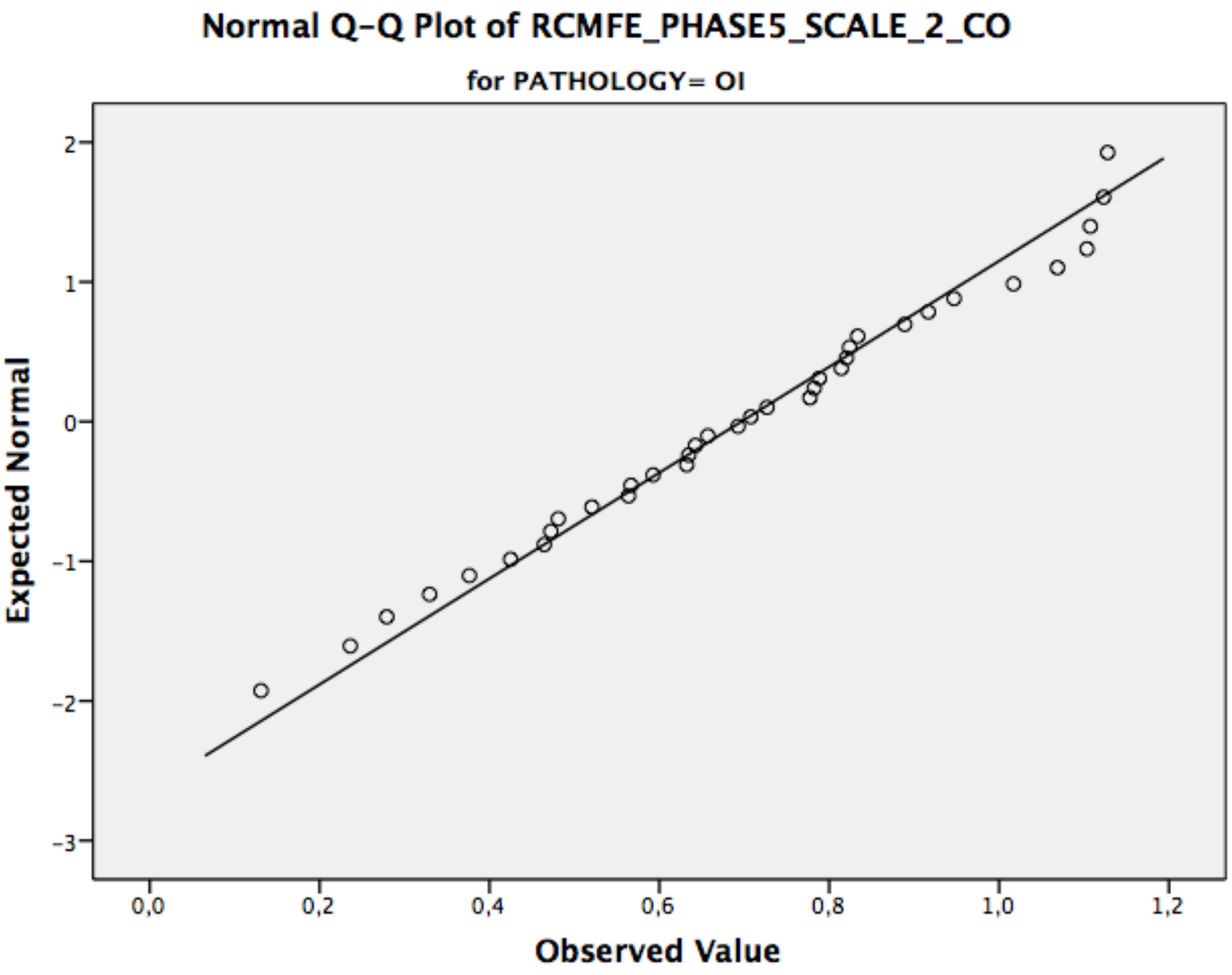

Normal Q-Q Plot of RCMFE_PHASE5_SCALE_2_CO
for PATHOLOGY= OI
Expected Normal
Observed Value
2
1
0
-1
-2
-3
0,0
0,2
0,4
0,6
0,8
1,0
1,2

Normal Q-Q Plots

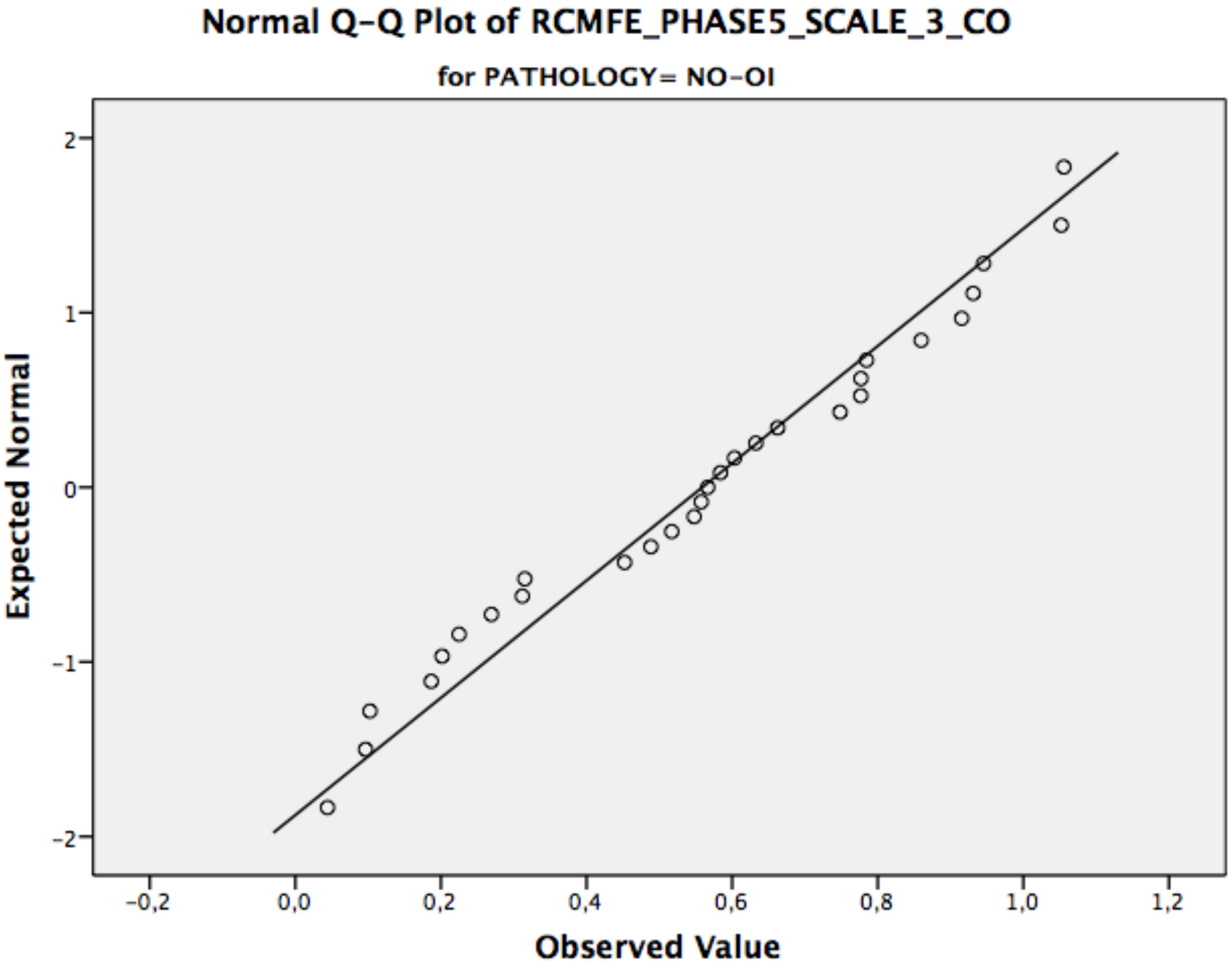

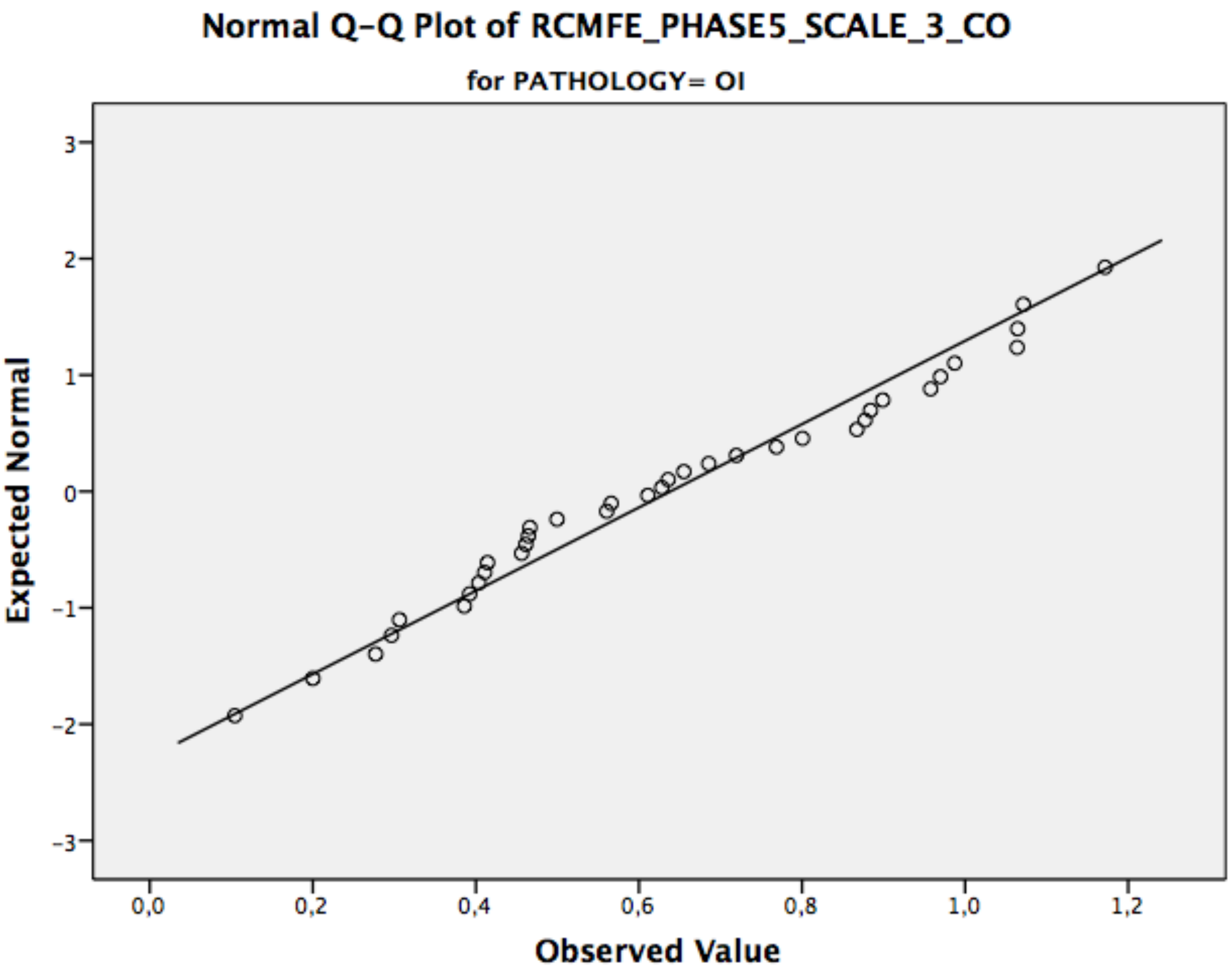
Normal Q-Q Plot of RCMFE_PHASE5_SCALE_3_CO
for PATHOLOGY= OI
Expected Normal
Observed Value
3
2
1
0
-1
-2
-3
0,0
0,2
0,4
0,6
0,8
1,0
1,2

RCMFE_PHASE5_SCALE_4_CO

Normal Q-Q Plots

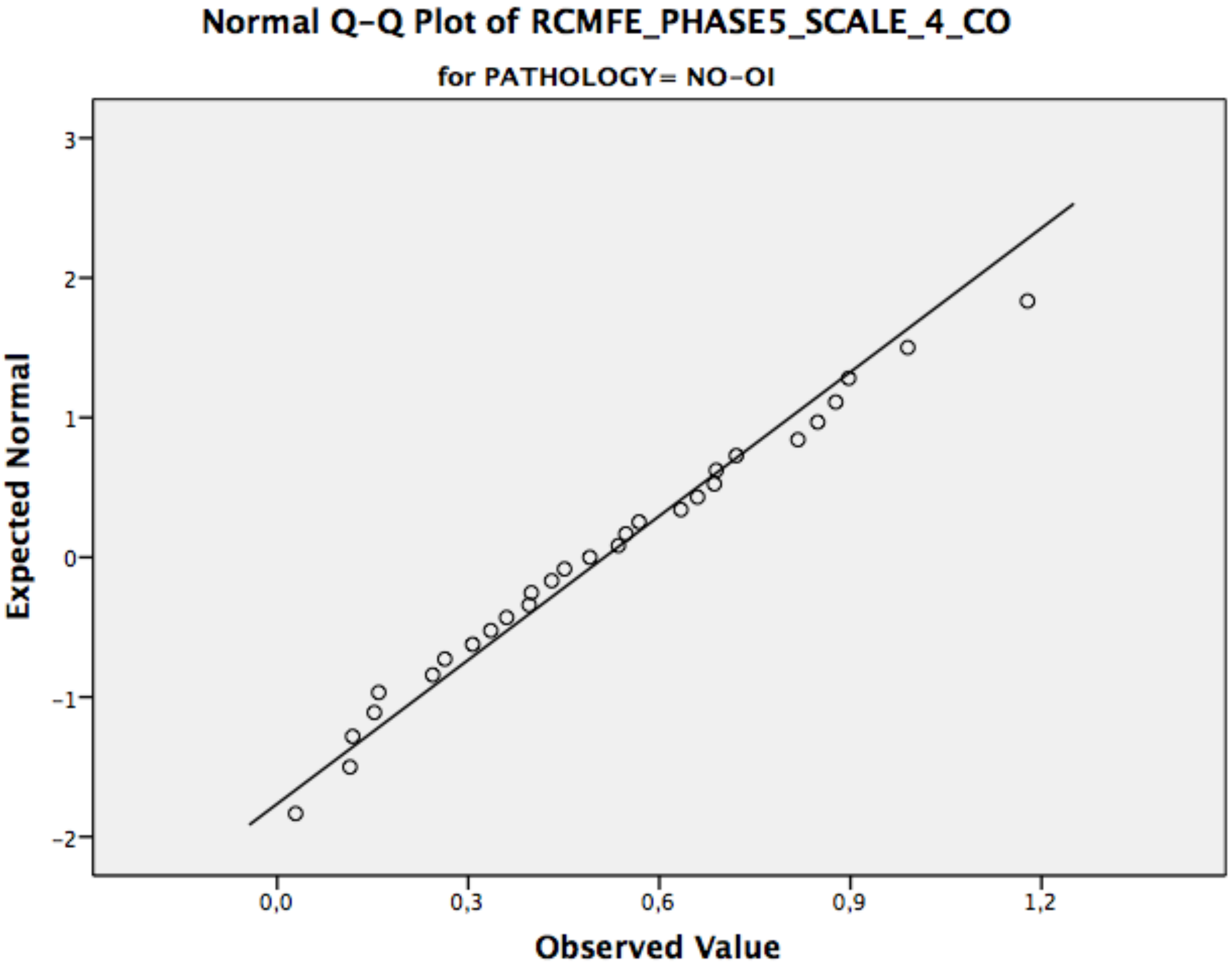

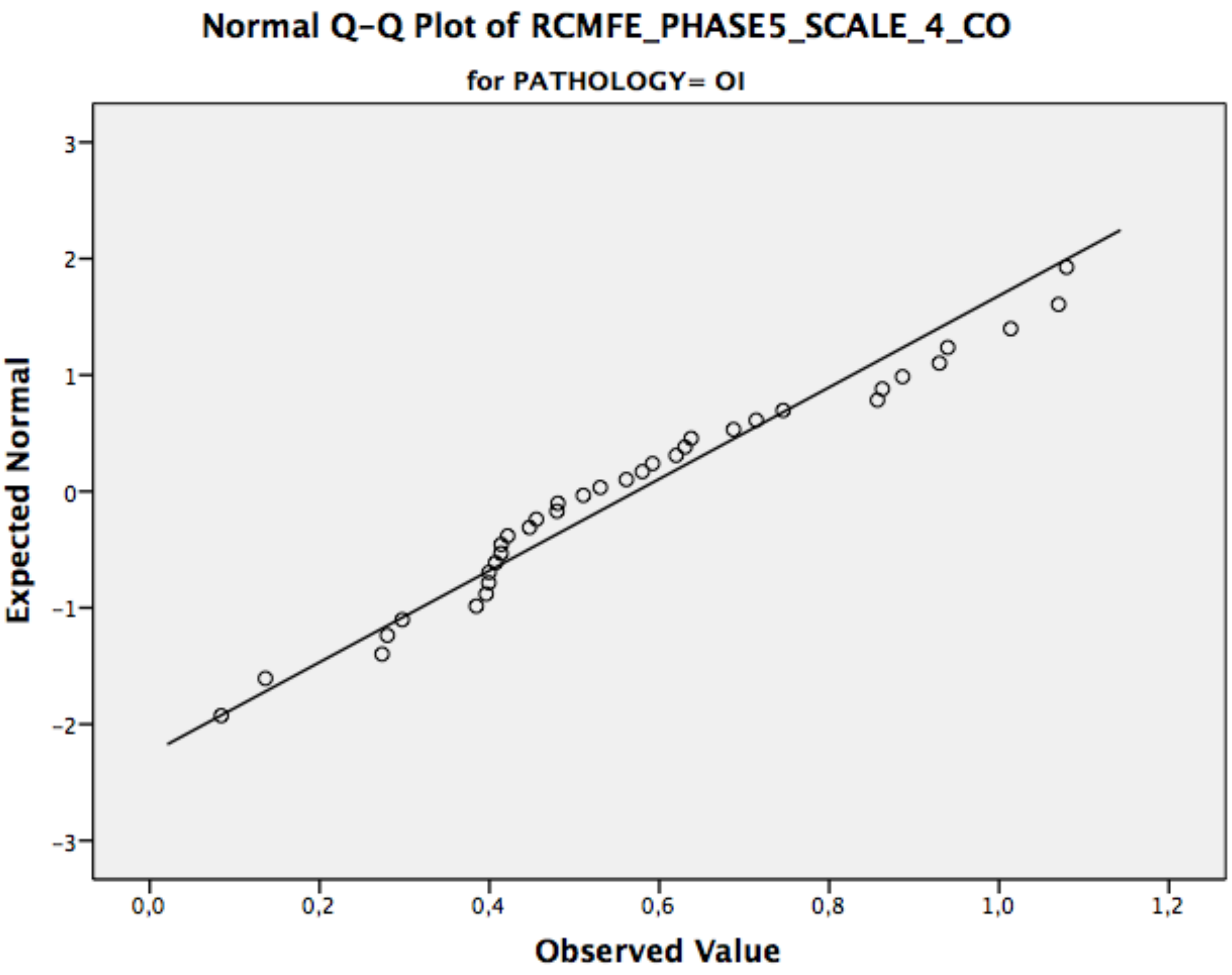

Normal Q-Q Plot of RCMFE_PHASE5_SCALE_4_CO
for PATHOLOGY= OI
Expected Normal
3
2
1
0
-1
-2
-3
0,0
0,2
0,4
0,6
0,8
1,0
1,2
Observed Value

RCMFE_PHASE5_SCALE_5_CO

Normal Q-Q Plots

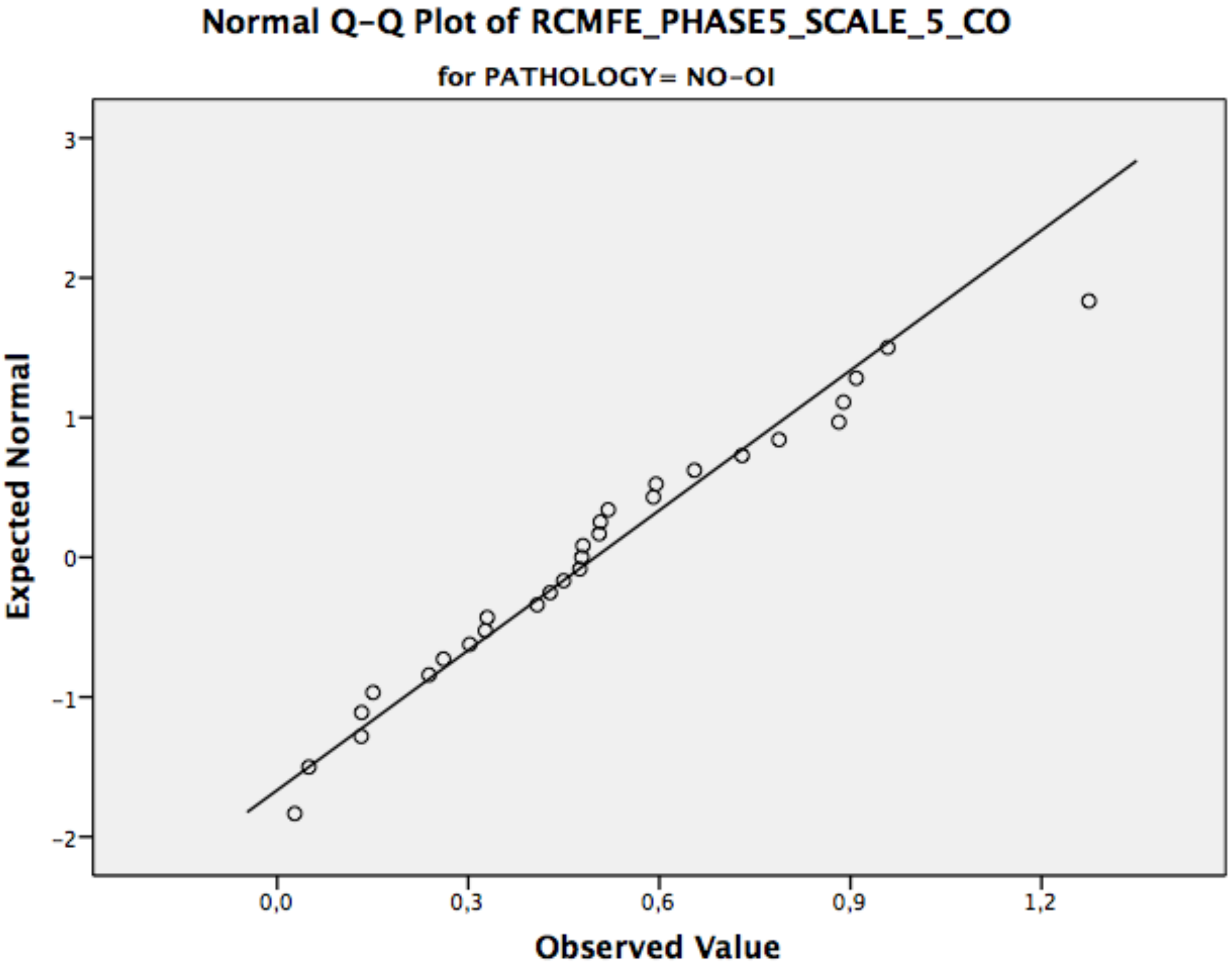

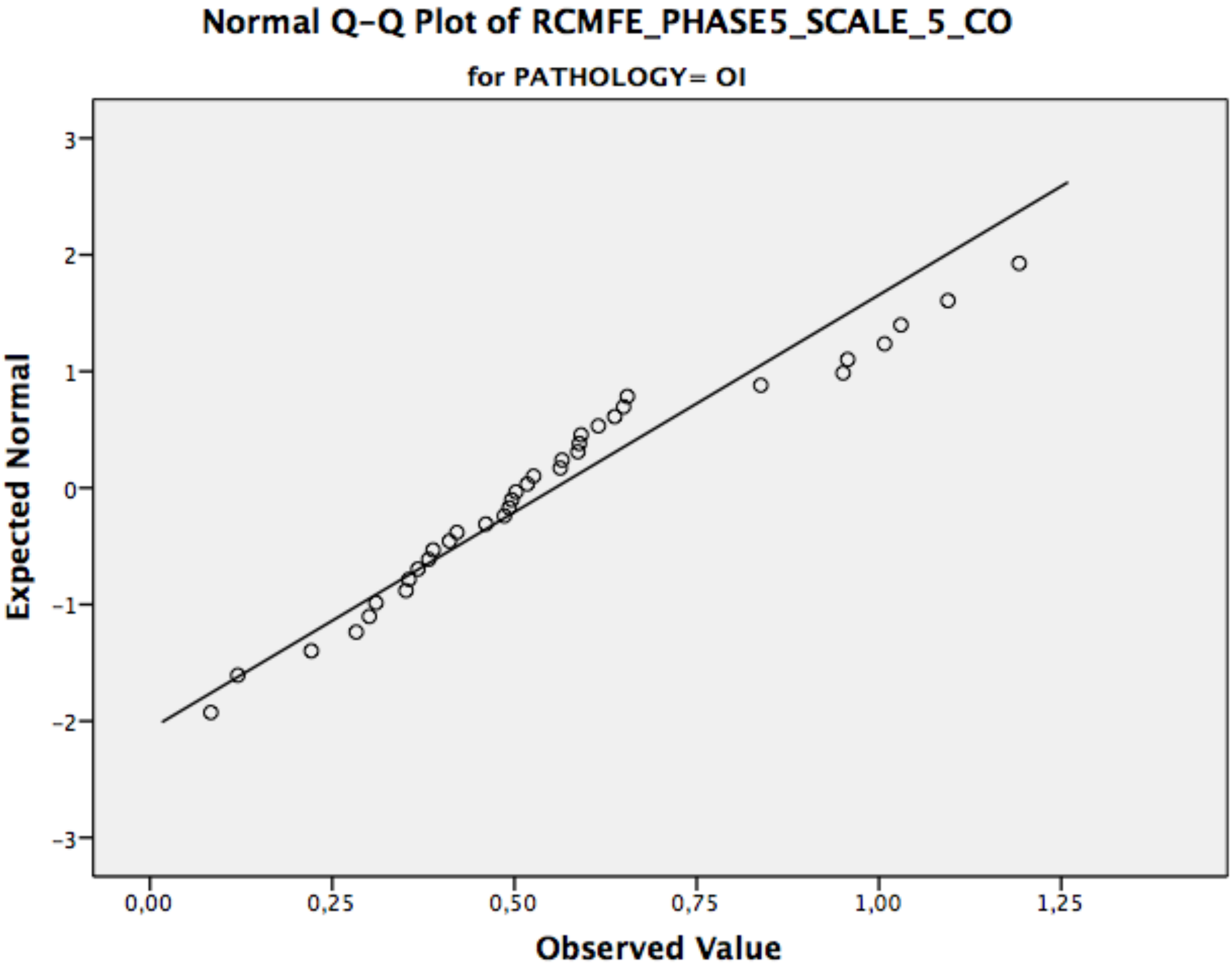
Normal Q-Q Plot of RCMFE_PHASE5_SCALE_5_CO
for PATHOLOGY= OI
Expected Normal
3
2
1
0
-1
-2
-3
0,00
0,25
0,50
0,75
1,00
1,25
Observed Value
